# High resolution tissue and cell type identification via single cell transcriptomic profiling

**DOI:** 10.1371/journal.pone.0318151

**Published:** 2025-03-26

**Authors:** Muyi Liu, Suilan Zheng, Hongmin Li, Bruce Budowle, Le Wang, Zhaohuan Lou, Jianye Ge

**Affiliations:** 1 Center for Human Identification, University of North Texas Health Science Center, Fort Worth, Texas, United States of America; 2 Department of Cell Biology, Harvard Medical School, Boston, Massachusetts, United States of America; 3 Department of Chemistry, Purdue University, West Lafayette, Indiana, United States of America; 4 Department of Computer Science, California State University, East Bay, Hayward, California, United States of America; 5 Department of Forensic Medicine, University of Helsinki, Finland; 6 Department of Electronic and Information Engineering, North China University of Technology, Beijing, China; 7 School of Pharmaceutical Sciences, Zhejiang Chinese Medical University, Hangzhou, China; University of Education, PAKISTAN

## Abstract

Tissue identification can be instrumental in reconstructing a crime scene but remains a challenging task in forensic investigations. Conventionally, identifying the presence of certain tissue from tissue mixture by predefined cell type markers in bulk fashion is challenging due to limitations in sensitivity and accuracy. In contrast, single-cell RNA sequencing (scRNA-Seq) is a promising technology that has the potential to enhance or even revolutionize tissue and cell type identification. In this study, we developed a high sensitive general purpose single cell annotation pipeline, scTissueID, to accurately evaluate the single cell profile quality and precisely determine the cell and tissue types based on scRNA profiles. By incorporating a crucial and unique reference cell quality differentiation phase of targeting only high confident cells as reference, scTissueID achieved better and consistent performance in determining cell and tissue types compared to 8 state-of-art single cell annotation pipelines and 6 widely adopted machine learning algorithms, as demonstrated through a large-scale and comprehensive comparison study using both forensic-relevant and Human Cell Atlas (HCA) data. We highlighted the significance of cell quality differentiation, a previously undervalued factor. Thus, this study offers a tool capable of accurately and efficiently identifying cell and tissue types, with broad applicability to forensic investigations and other biomedical research endeavors.

## Introduction

Tissue or body fluid identification, which determines the origin(s) of organ tissue type(s) of recovered samples from crime scenes, is a challenging task in forensic investigations [1–3]. Confirming the tissue source of a sample could assist in linking a perpetrator, victim, and crime scene, reconstructing a crime scene, and deciding the manner and significance of a crime or even if a crime occurred [4–9]. For example, detecting a large amount of skin tissue on the floor may suggest the dragging of a body to or from the crime scene, and the presence of semen in the vagina mucosa of a sexual assault victim is an exceedingly strong indication of sexualntercourse [10].

Tissue identification traditionally uses varied methodologies including chemical [4] and immunological [11] approaches as presumptive tests, and histological or serological methods with microscopes as confirmatory tests [2,12]. However, these tests may have limited specificity because of cross-reactivity with other substances or tissues, require large amounts of samples, may not be able to process old samples, may not be able to differentiate similar tissues (e.g., blood and menstrual blood), are consumptive and may be destructive to samples, which limit the potential for additional tests, and require different formats making them more difficult to accommodate for an efficient laboratory workflow [2,13,14].

The molecular genetic-based approaches, instead, can alleviate these issues with much higher sensitivity and specificity by characterizing organ tissues with tissue-specific markers, such as proteins, mRNAs, microRNAs, DNA methylation sites, etc [13,15–29] and may be analyzed on a common platform. However, samples can range from mixtures of different tissues (e.g., virginal mucosa, sperm or seminal fluid, and saliva in sexual assault samples) from multiple donors to single-source samples with multiple types of tissues (e.g., epithelial and sperm cells from the same male donor in a penile swab) [13,24]. It is still challenging for the current molecular genetic based approaches to process tissue mixture samples, because these tests often are conducted in bulk fashion [2,9,19,24,30,31]. Thus, these technologies may confirm if a specific tissue is present in a sample, but may not determine how many types of tissues are present, nor their proportions, in a single test. It is also difficult to associate the identified tissues with the donors (namely, not being able to assign which tissue(s) belongs to which donor in a mixture) [9]. In addition, selecting and deciding the tissue-specific markers, or differentially expressed or presented markers among different tissues, from a large suite of markers available in the public database or commercial kits is a major task in developing these molecular genetic-based tests [19,32–34]. Due to limited resources, markers are usually selected from a relatively small number of samples. Also, various studies have led to different marker selections [25], and many factors (e.g., age, gender, health condition, etc.) could affect the presence or expression level of these markers and thus the selection process [35–39].

Proteomic and panel-based testing are the primary biological and forensic methods for tissue type identification, offering high sensitivity and specificity. These approaches benefit from well-established protocols, validated panel designs, cost efficiency, and high-throughput capabilities [40,41]. However, genetic variations in certain tissue-specific proteins can limit the sensitivity of proteomics, potentially leading to false negatives [42–45]. Additionally, the bulk nature of proteomic methods restricts their ability to deconvolute complex mixtures.

Panel-based testing, while effective, also has limitations. It requires prior knowledge for assay design and often consumes valuable evidentiary material [46]. Furthermore, processes such as cell digestion and dissociation can impact forensic outcomes.

Recently, through the utilization of advanced microfluidic flow technology and combinatorial cell/molecule indexing engineering, single-cell RNA Sequencing (scRNA-Seq) [47] and single-cell Assay for Transposase Accessible Chromatin (scATAC-Seq) [48] are capable of capturing the entire transcriptomics and genomics profiles from thousands or even millions of individual cells [49–51]. The unprecedented resolution brought by these single-cell technologies could in theory alleviate the above-mentioned challenges [52].

Enabled by transcriptome-wide gene expression evaluation at single cell resolution, scRNA-Seq has reshaped studies and applications in many fields, such as cell type heterogeneity [53–57], signature gene association [58–60], cell phylogenetics [59,61,62], drug discovery [63,64], immune response [65,66], gene network regulation [67,68], cell interaction [69,70], etc. As examples, multiple levels of cell heterogeneity can be found in cellular composition, chromosomal structures, gene regulation, developmental programming, and inter-cellular signaling [71–73].Hidden cell types and genotype subpopulations have been frequently reported [74–76]. New blood T cells [77], skin fibroblasts [78], and undifferentiated spermatogonia cell types [79], are discovered with single-cell techniques. Marker genes, once considered as cell type unique, have been detected across different cell types [72]. VIM, a well-known marker of messenchymal cells, can also be identified in endothelial cells. CD34, a well-known gene of endothelial cells, is expressed in messenchymal cells. Thus, tissue types may be underrepresented by previous domain knowledge, and designated marker genes may not be rigorous for tissue identification, especially if they are to serve as confirmatory tests. In contrast to proteomic and panel-based testing, scRNA-Seq overcomes the limitations of predefined panel designs and offers high sensitivity in detecting mRNA, the precursor to protein products. Additionally, scRNA-Seq preserves the integrity of cellular mixtures and retains genetic variations, particularly when long-read sequencing is employed. Despite its inherent challenges, approaches such as formalin-fixed paraffin-embedded (FFPE) protocols and single-nuclei sequencing have proven effective in maintaining sample integrity and enabling robust analysis [80,81].

Despite the great potential of current molecular biology technologies, investigating DNA and RNA at the single-cell level is unprecedentedly challenging due to unique problems, such as transcript count sparsity, cell diversity, and high dimensionality [82–84]. For example, the Human Cell Atlas Consortium [85] includes more than 34.3 million cells, dozens of organs, and 1,000 cell types, where at least 10,000 genes are absent per cell. Therefore, direct application from bulk sequencing pipelines is infeasible. Motivated by the great potential of single-cell techniques, many advanced computational pipelines have been developed for specific single-cell tasks. To differentiate cell types, the most cited pipelines, Seurat [54], Scanpy [86], and Monocle [87], transform expression profiles to low-dimensional representatives and cluster cell type groups by cell similarity. However, these pipelines still may be inefficient, due to, for example, performance inconsistency, complicated parameter interpretation, and scale efficiency [49,88,89]. Additionally, these pipelines predominately focus on unsupervised clustering tissue identification but do not perform classification, thus only partially fulfilling the needed task.

More state-of-the-art single cell RNA-seq cell type annotation pipelines have been developed, such as SingleR, scMapCell, SingleCellNet, scID, scVI, CelliD, scReClassify, and scNym. SingleR [90] considers cells with high Spearman coefficient as the confident reference, and labels a cell’s type by Spearman coefficient with reference’s variable genes. scMapCell [91] forms approximated centroids by K means clustering, and labels each query cell by comparing it with the reference centroids. SingleCellNet [92] transforms gene expression matrix to paired-gene binary matrix. Noise is reduced by keeping only highly correlated gene pairs. The random forest classification in this latent space distinguishes the query cell types. scID [93] weights each variable gene from reference clusters by their discriminatory power on query cells. The weighted average expression scores of each query cell fit the Gaussian mixture model to assign cell types. scVI [94], as part of scArches [95], assumes gene count follows zero-inflated negative binomial distributions. A neural network based generative model is used to train the parameters of distribution and impute missing count values in scRNA-seq data. CelliD [96] projects both cells and genes into a common orthogonal space by dimension reduction. The gene markers are defined by the ranking distance specificity to a cell and a cell type. scNym [97] combines semi-supervised and adversarial training to train a mix model on both training and validation sets. The cell type annotations of validation are derived by this mix model. scReClassify [98], from an initial cell type annotation, reclassifies cell by PCA and semi-supervised learning to correct potentially mislabelled cells.

**Fig 1:**
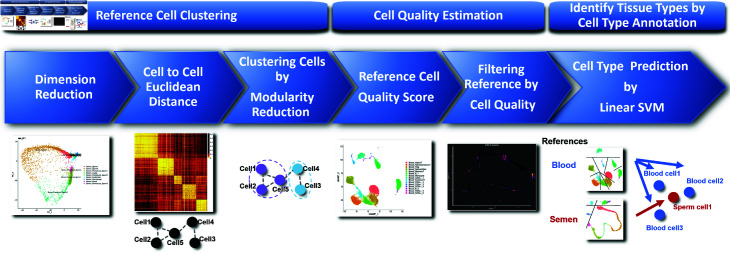
Tissue type identification pipeline.

In this study, scRNA-Seq is applied to identify cell types and subtypes, which in turn facilitates tissue type identification by accurately annotating cell types within tissue mixtures. This method captures mRNA using a non-specific polyA primer, avoiding biases or preferences. In contrast, protein biomarker panels rely on a predefined subset of proteins, with their accuracy constrained by the sensitivity and specificity of antibodies. Furthermore, protein products are not always present even when their corresponding mRNA is available, making the capture of upstream mRNA a viable and often superior alternative. However, scRNA-Seq is not without its challenges, such as transcripts drop out, doublet formation, and ambient mRNA contamination.

To address these issues, we present a tissue classification method, scTissueID, a highly accurate general-purpose cell type annotation tool capable of determining tissue mixture types using scRNA-Seq data. As our unique approach, we demonstrate that quality differentiation rather than relying solely on dimensionality reduction can widely improve scRNA-Seq cell type classification. This proof-of-concept study emphasizes tissue identification through accurate cell type classification, a crucial forensic application where scRNA-Seq holds significant potential. We highlight the importance of cell quality differentiation, which may have been previously overlooked.

## 1. Methods

The scTissueID pipeline, outlined in Fig 1, comprises three primary phases: 1) cell type clustering per reference tissue, 2) the unique cell quality estimation and cell type annotation, and 3) tissue mixture prediction via linear SVM algorithm. In the first phase, the raw data are mapped to the human genome reference and transcript counts are extracted. The cell type clusters are determined by the distance between cell transcription profiles and a modularity reduction algorithm. During the second phase, cell types are annotated based on transcription activity, and noisy cells are filtered out in cases where potential ambient transcripts contaminate cells and/or when intermediate cells exist between different cell types. The final phase predicts the cell and tissue types with the quality controlled reference (i.e., training data). scTissueID first decides the cell type of each cell, and tissue types are determined by consolidating the cell types of all cells. The classification performance was compared with six widely-used classification algorithms, marker-based cell type annotation, and seven state-of-the-art cell type classification pipelines.

### 1.1. Datasets

In this study, we rely on the following datasets collected from Human Cell Atlas (HCA), Human Lung Cell Atlas (HLCA), and other publicly available sources (Table 1). Samples were subjected to scRNA-Seq sequencing, and cDNA molecules were generated on the 10x Chromium and Smartseq2 platforms. Specifically, transcription profiles from 33 major tissues samples, 618 cell types, over 50,000 genes per sample were captured, and more than half million cells were collected as the reference, which includes 25 samples with curated HCA cell type labels, 4 samples with fluorescence-activated cell sorting (FACS) labels, and 4 samples self-labeled in this study. Each cell accumulates at least 100 captured genes and 1,000 total transcripts as an individual profile. All collected samples are from healthy donors. Noticeably, all single-cell samples in this study were sourced from major scRNA-Seq portals and are recognized as high-quality. While existing pipelines and dimensionality reduction methods typically assume uniform reference quality, our pipeline uniquely differentiates cell quality even within these high-quality datasets. By retaining only the most reliable cells, it enhances reference reliability and enables higher-resolution classification without relying on dimensionality reduction.

**Table 1:** Single cell transcription datasets used in the study.

Dataset	*#S* ^1^	*#C* ^2^	*#CT* ^3^	*#G* ^4^	*#AG* ^5^	Labeled	Repository	Reference
Blood	3	20,000^6^	15	21,952	578	FACS^7^	SRP073767^8^	[99]
Skin	5	16,062	9	32,738	614	*✓* ^9^	GSE130973^10^	[100]
Semen	3	6,490	9	27,477	728	*✓*	GSE120508	[60]
Urine	17	23,082	7	21,394	1326	*✓*	GSE157640	[101]
Saliva	2	25,809	14	33,694	882	*✓*	GSE180544	[102]
10Xv2(Blood)	1	3,362	6	22,280	170	FACS	BSP^11^	[103]
10Xv3(Blood)	1	3,184	5	20,041	236	FACS	BSP	[103]
Semen1	2	20,559	1	33,694	2,642	FACS	GSE109037	[104]
HCA_Bladder	15	24,583	15	58,870	1,160	*✓* ^12^	HCA^13^	[105]
HCA_Blood	15	50,115	27	58,870	1,602	*✓*	HCA	[105]
HCA_Bone	15	12,297	17	58,870	514	*✓*	HCA	[105]
HCA_Eye	15	10,650	32	58,870	608	*✓*	HCA	[105]
HCA_Fat	15	20,263	13	58,870	1,132	*✓*	HCA	[105]
HCA_Heart	15	11,505	6	58,870	747	*✓*	HCA	[105]
HCA_Kidney	15	9,641	7	58,870	447	*✓*	HCA	[105]
HCA_LargeIntestine	15	13,680	20	58,870	457	*✓*	HCA	[105]
HCA_Liver	15	5,007	13	58,870	205	*✓*	HCA	[105]
HCA_Lung	15	35,682	40	58,870	2,025	*✓*	HCA	[105]
HCA_LymphNode	15	53,275	29	58,870	1,862	*✓*	HCA	[105]
HCA_Mammary	15	11,375	14	58,870	498	*✓*	HCA	[105]
HCA_Muscle	15	30,746	19	58,870	1,284	*✓*	HCA	[105]
HCA_Pancreas	15	13,497	15	58,870	725	*✓*	HCA	[105]
HCA_Prostate	15	16,375	21	58,870	728	*✓*	HCA	[105]
HCA_Skin	15	9,424	25	58,870	355	*✓*	HCA	[105]
HCA_SmallIntestine	15	12,467	21	58,870	440	*✓*	HCA	[105]
HCA_Spleen	15	34,004	24	58,870	1,386	*✓*	HCA	[105]
HCA_Thymus	15	33,664	32	58,870	1,398	*✓*	HCA	[105]
HCA_Tongue	15	15,020	12	58,870	897	*✓*	HCA	[105]
HCA_Trachea	15	9,522	21	58,870	471	*✓*	HCA	[105]
HCA_Uterus	15	7,124	14	58,870	382	*✓*	HCA	[105]
HCA_Vasculature	15	16,037	14	58,870	1,018	*✓*	HCA	[105]
Lung_Atlas_10x	36	65,662	57	26,485	4,777	*✓*	HLCA^14^	[106]
Lung_Atlas_Smartseq2	36	9,409	44	58,683	409	*✓*	HLCA	[106]

^1^The number of healthy donors.

^2^The number of cells.

^3^The number of cell types.

^4^The total number of captured genes, and genes without any captured transcript are ignored.

^5^The average number of captured genes per cell.

^6^Original publication only deposited the high quality cells.

^7^FACS: fluorescence-activated cell sorting.

^8^The Sequence Read Archive Database.

^9^The cell type annotation was provided by clustering and curated cell type markers.

^10^The Gene Expression Omnibus Database.

^11^The Broad Institute Single Cell Portal.

^12^The cell type annotation was provided by the database.

^13^https://data.humancellatlas.org/.

^14^https://github.com/krasnowlab/HLCA.

### 1.2. Construct reference: clustering and modularity reduction

Most scRNA-Seq experiments provide no cell type labels, due to the deficient knowledge of conventional markers. The samples subjected to the FACS labeling (Table 1), were considered as ground truth. We adopted, Seurat [107], the most cited cell type clustering pipeline to verify its accuracy with the Blood sample.

First, in Seurat, all gene features were reduced to 30 principal components with the Principal Component Analysis (PCA) [108]. Thereafter, the cell similarity network was constructed with the Euclidean distance between pairs of cells. Then this cell network was reduced to optimal cell type communities by the Louvain algorithm [109], in which a cell type was designated to each cell. The pipeline parameters were tuned to maximize the accuracy of the Blood sample labels. The same pipeline was applied to the other samples’ cell type curation if needed.

### 1.3. Cell quality differentiation by reclassification

Despite that unsupervised clustering (e.g., Seurat and Scanpy) provides the cell types, the assumption of a uniform cell quality may not hold true. A reclassification and cell quality estimation differentiates the cells by their qualities.

SVM was employed in reclassification and cell quality estimation. The training algorithm of SVM can be formulated as Eq 1, where *ω* denotes the normal vector and *b* denotes the bias factor, which were subject to the optimization. The optimized solution is recorded during training. $\phi (x_{i})$ is the natural logarithm of one plus the gene expressions in the *ith* cell, $x_{i}$. $y_{i}$ is the cell type obtained from both FACS and the Seurat pipeline. The validation classifies cell *i* to the closest cell type $y_{i}$ in .


min ⁡ω,b12ωTω+ ∑i=1nmax(0,∣yi−(ωTϕ(xi)+b)∣) (1)

$$\begin{array}{c} y_{i}=\omega^{T}\phi (x_{i})+b \end{array}$$ (2)

The cell quality probability estimations (i.e., quality scores), $P(y=y_{i}|x_{i})$, , are provided by the logistic transformation of the SVM scores $\omega^{T}\phi (x_{i})+b$. Cells with high confidence (i.e., $P(y=y_{i}|x_{i})\geq 0.8$), were preserved as reference cells, while noisy cells (i.e., the maximum quality score  < 0 . 8) are generally considered as lacking confidence.


$$\begin{array}{c} P(y=y_{i}|x_{i})=\frac{1}{1+exp(\omega^{T}\phi (x_{i})+b)} \end{array}$$ (3)

### 1.4. Evaluation

*Evaluation scores.* To evaluate the tissue and cell type classification performance, we employed the Accuracy, Adjusted Rand Index (ARI) [110], Normalized Mutual Information (NMI) [111], Median F1 (MF1), Precision, and Recall. These metrics are widely utilized for classification evaluation. Note that the overall accuracy could be dominated by the accuracy of major cell clusters, ARI, NMI, and MF1 scores are better measures for evaluating classifications. In particular, the median value of F1 scores per cell type is to avoid biased classification (i.e., unfairly assigning all cells to several majority groups). Additionally, we recorded the computation time and memory usage of each algorithm.

**Fig 2:**
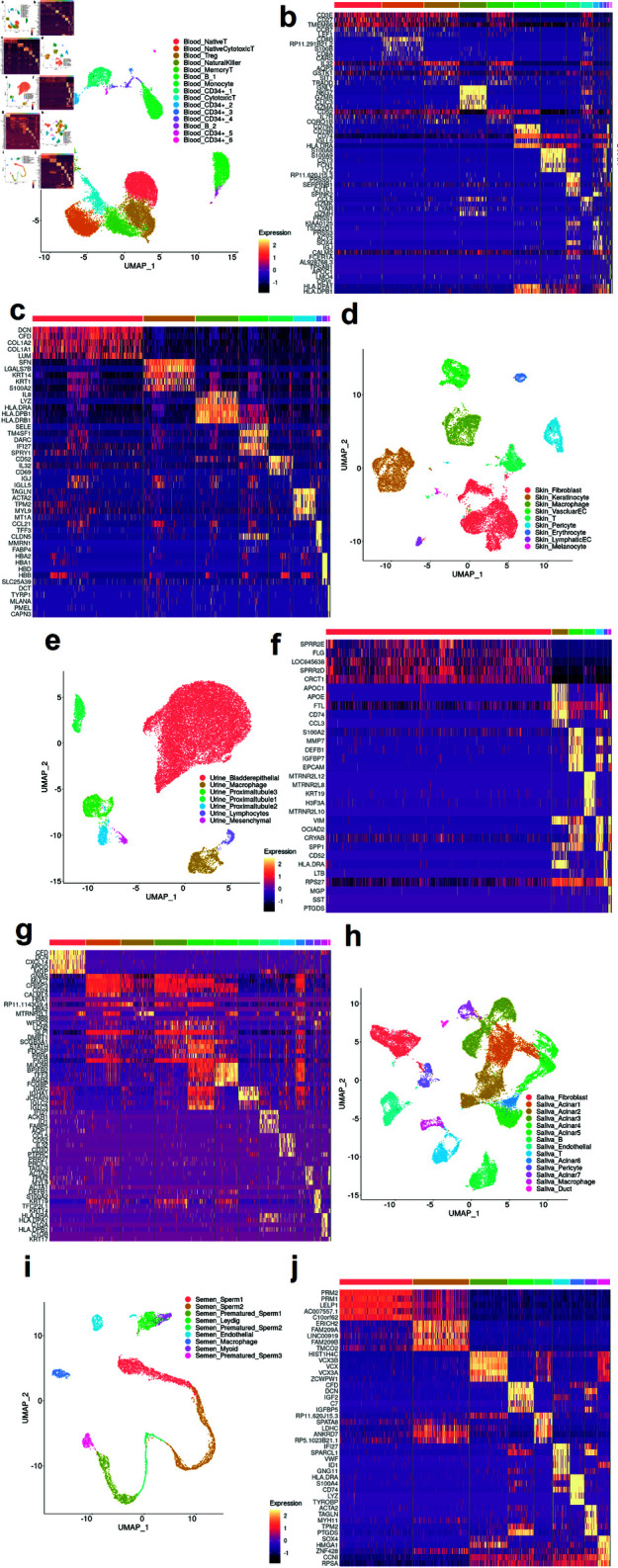
The cell clustering and marker gene expressions. The UMAPs plot the cell type clusters of Blood (a), Skin (d), Urine (e), Saliva (h), and Semen (i). Each dot represents a cell, and cell types are distinguished by colors. The heatmaps display the marker genes (*p*-value  <  $10^{-100}$) of Blood (b), Skin (c), Urine (f), Saliva (g), and Semen (j). The Viridis color map denotes the normalized transcription activities of marker genes. Cell types are annotated by the top color bars.

*Baselines.* We compared scTissueID with both the biological and computational baselines. Because scRNA experiments typically identify the most significant markers to annotate cell types, for the biological baseline, the most differentially expressed genes are collected as biological markers (details in Fig 2b, 2d, 2e, 2g, and 2i, and Supporting information Table S1) to differentiate cells across different tissue types. This biological baseline is akin to conventional antibody tests and cell type curation approaches within the single-cell context. Although more sophisticated marker identification methods are available, scRNA experiments usually adopts the FACS sorting only by several protein markers.

For the computational baselines, six widely-used classification algorithms, including k-Nearest Neighbor (KNN) [112], Nearest Mean Classifier (NMC) [113], Linear Discriminant Analysis (LDA) [114], Random Forest (RF) [115], XGBoost [116], and Elasticnet [117], were chosen as computational baselines. These algorithms annotate cell types using fundamentally different strategies.

To establish robust comparative study for scRNA-Seq cell type annotation, we selected pipelines representing diverse and well-regarded approaches from multiple comprehensive review studies [118–122]. The chosen methods, SingleR, scMapCell, SingleCellNet, scID, scVI, CelliD, scReClassify, and scNym were identified based on their demonstrated performance across various datasets, unique methodological approaches (e.g., marker-based, neural network-based, or probabilistic frameworks), and widespread adoption or citation in the field. These methods offer a range of strategies for cell type annotation, enabling a balanced comparison of traditional and state-of-the-art techniques.

Because scRNA studies typically identify the most significant differentially expressed genes as markers to annotate cell types, the biological baseline utilizes protein and mRNA markers to predict tissue type. The marker genes majority vote (MGMV) ranks the differentially expressed genes in each cell type by their statistical significance (i.e., p-value). The likelihood-ratio test [123] in MGMV quantifies this differential expression by the Log fold-change of the Average Expression (AElogFC) within each cell type. Thereby, the top-ranked genes of cell types are determined as marker genes, and each cell is assigned to a specific cell type by the majority vote of markers’ expression. Although more sophisticated marker identification methods are available, scRNA experiments usually employ FACS sorting based only on several protein markers. This is to understand the limitation of applying only several marker genes in a single-cell context.

In addition, challenging samples, such as imbalanced mixtures with multiple donors or substantial gene dropouts, are common in crime scene samples. We investigated the impacts of random gene dropouts and various mixture ratios on cell type annotation. We simulated low-quality tissue samples by randomly dropping gene counts, ranging from 0 . 60*%* to 0 . 99*%* of total counts. Mixed tissues with imbalanced compositions were simulated by merging cells from five major forensic tissues.

## 2. Results

This study focused on addressing the following major questions: 1) How to construct reliable gene expression profiles as the reference for subsequent classification? 2) How to efficiently classify cells at a large scale? 3) To what extent can the scTissueID pipeline improve performance compared with baseline algorithms? 4) Will the performance be reduced when encountering challenging situations, such as imbalanced tissue mixtures and gene dropout? The remainder of this section presents scTissueID as the solution to these questions.

### 2.1. Quality control over noisy cells

*The heterogeneity in tissue cell types and marker genes’ activity.* The performance of cell-type clustering was assessed using the Blood sample with known FACS cell-type labels. The ARI and NMI scores were 0 . 9813 and 0 . 9431, respectively. The highly accurate clustering of the Blood sample provides high confidence in other samples and downstream steps as the foundation.

A total of 15 blood cell populations and subpopulations were identified with the five forensic tissues (Fig 2a). Major gene markers such as CD3E, CD34, and LYZ were differentially expressed (*p*-value  <  $10^{-100}$) in T cells, peripheral haematopoietic stem cells, and monocytes, respectively (Fig 2b). Although CD3E is a well-known T cell marker, many T cells had low or no CD3E transcription activity. Therefore, the heterogeneity of marker gene activity resulted in uncertainty in tissue type identification. Additionally, besides the diversity in gene activity, many cell types were observed within multiple tissue types, such as T cells in Blood, Skin, and Saliva samples (Fig 2a, 2d, and 2h). Hence, classifying blood tissue solely by T cells was unreliable. In contrast, in Fig 2e and 2f, three distinct kidney proximal tubular cell types expressed APOCE, S100A2, and MTRNR2L12, independently in the urine sample, suggesting low reliability in determining tissue type solely by cell-specific genes. Thus, a high-quality reference is necessary for cell type annotation.

**Fig 3:**
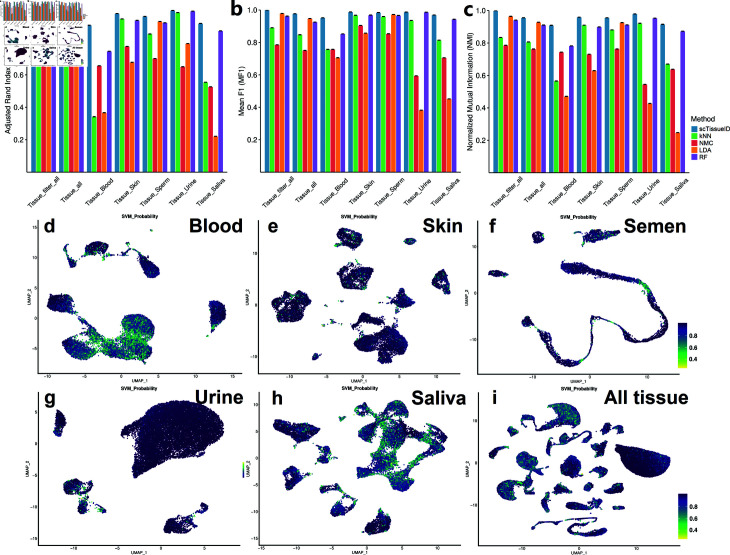
Cell type classification performance improvement by quality control of eliminating noisy cells. The ARI (a), Mean F1 (b), and NMI (c) scores of 10-fold cross-validation by the scTissueID and baseline methods. The UMAP plots of Blood (d), Skin (e), Sperm (f), Urine (g), Saliva (h) samples, and All tissue types (i) are color-coded by the maximum probabilities.

*Post-clustering quality control.* Due to potential doublets, ambient mRNA contamination, and incorrect cell type labels, all tissue samples underwent post-clustering quality control estimation. Cells with a maximum quality score, as defined in Eq 3, of less than 0 . 80 were considered noisy and eliminated from the reference (Fig 3d, 3e, 3f, 3g, and 3h). In principle, doublets were located between two cell clusters, such as cells between urine lymphocytes and mesenchymal cells (Fig 2e). The maximum probabilities of these potential doublets were less than 0.8 (Fig 3e). Only 10 . 1*%* of cells were filtered out. The subsequent 10-fold cross-validation classification accuracy increased from 0 . 9723 to 0 . 9998, suggesting that most of the low-quality cells were eliminated (Fig 3a, 3b, and 3c, and Table 2). Removing these noisy cells could increase classification performance.

Despite the improvement in classification quality achieved by using the maximum score cut-off threshold, lower quality scores were also observed among maturing sperm cells. For example, the scores of cells adjacent to both Semen’s Sperm1 and Sperm2 clusters are all less than 0 . 6 (Figs 2i and 3f). Due to the transitional stage from premature to matured cells, this contradictory example highlights the need for caution when applying the maximum quality score.

### 2.2. Tissue classification by scTissueID and baselines

Even though single-cell samples were annotated, clustered, and quality controlled, the choice of classification algorithm remained undecided. Next, the overall accuracy, computation time complexity, and performance robustness among the common forensic tissues and HCA tissues were used as criteria for deciding the algorithm.

**Table 2:** The comprehensive performance study of major forensic tissue and cell type classification.

	Performance								
Datasets	Method	Accuracy	ARI	MF1	NMI	Train^1^	Test^2^	Total^3^	Memory^4^
Cells Passed QC	scTissueID	0.9999	0.9999	1.0000	0.9997	4,398	79	4,477	32
	KNN	0.8267	0.8535	0.8906	0.8342	188	9,149	9,337	41
	LDA	0.9780	0.9803	0.9789	0.9644	76,530	407	76,937	32
	NMC	0.7817	0.7153	0.7856	0.7858	270	599	869	32
	scReClassify	0.8866	0.9242	0.9281	0.8881	31,185	183,038	214,223	42
	RF	0.9601	0.9691	0.9611	0.9409	2,138	20	2,158	32
	XGBoost	0.9876	0.9904	0.9852	0.9769	1,056,808	25	1,056,833	32
	Elasticnet	0.9687	0.9747	0.9749	0.9547	534,869	153	535,022	32
All Cells	scTissueID	0.9730	0.9763	0.9776	0.9566	1,802	41	1,843	33
	KNN	0.8038	0.8183	0.8561	0.8132	161	6,234	6,395	42
	LDA	0.9532	0.9579	0.9506	0.9317	47,237	356	47,593	33
	NMC	0.7577	0.6963	0.7542	0.7647	254	366	620	33
	scReClassify	0.8652	0.9019	0.907	0.8671	32,050	197,832	229,882	44
	RF	0.9368	0.9476	0.9279	0.9115	1,667	11	1,678	33
	XGBoost	-	-	-	-	-	-	-	-
	Elasticnet	0.9441	0.9514	0.9466	0.9217	485,802	136	485,938	33
Blood	scTissueID	0.9567	0.9062	0.9520	0.9091	123	7	130	11
	KNN	0.6009	0.3420	0.7572	0.5657	11	390	401	14
	LDA	0.6246	0.3669	0.7059	0.4710	36,295	18	36,313	11
	NMC	0.8317	0.6562	0.7572	0.7439	25	22	47	11
	scReClassify	0.9513	0.9010	0.9229	0.8940	7,609	5,700	13,309	12
	RF	0.8718	0.7459	0.8534	0.7831	221	2	223	11
	XGBoost	0.9532	0.8970	0.9462	0.9030	117,858	12	117,870	11
	Elasticnet	0.9254	0.8417	0.9304	0.8577	85,207	33	85,240	11
Skin	scTissueID	0.9899	0.9782	0.9879	0.9592	120	9	129	14
	KNN	0.9727	0.9450	0.9676	0.9099	14	379	393	18
	LDA	0.8500	0.6774	0.8573	0.6296	29,447	17	29,464	14
	NMC	0.8652	0.7754	0.9050	0.7306	31	16	47	14
	scReClassify	0.9923	0.9844	0.99	0.9683	5,915	2,692	8,607	14
	RF	0.9714	0.9381	0.9714	0.9025	241	3	244	14
	XGBoost	0.9874	0.9730	0.9841	0.9499	76,357	17	76,374	14
	Elasticnet	0.9812	0.9582	0.9792	0.9325	66,595	33	66,628	14
Semen	scTissueID	0.9848	0.9605	0.9845	0.9558	111	4	115	5
	KNN	0.9391	0.8522	0.9597	0.8809	5	54	59	6
	LDA	0.9723	0.9294	0.9726	0.9259	2,781	5	2,786	5
	NMC	0.8550	0.7005	0.8537	0.7632	16	6	22	5
	scReClassify	0.9791	0.9419	0.9871	0.9431	1,681	429	2,110	5
	RF	0.9655	0.9213	0.9647	0.9119	60	2	62	5
	XGBoost	0.9782	0.9501	0.9813	0.9379	21,248	8	21,256	5
	Elasticnet	0.9738	0.9315	0.9736	0.9294	13,761	14	13,775	5
Urine	scTissueID	0.9974	0.9972	0.9872	0.9793	90	5	95	12
	KNN	0.9873	0.9842	0.9356	0.9218	13	509	522	15
	LDA	0.8565	0.7929	0.3823	0.4273	44,412	12	44,424	12
	NMC	0.8344	0.6502	0.5943	0.5448	25	13	38	12
	scReClassify	0.9908	0.9883	0.9598	0.9294	6,389	6,640	13,029	13
	RF	0.9925	0.9932	0.9872	0.9538	192	2	194	12
	XGBoost	0.9971	0.9967	0.9856	0.977	53,311	13	53,324	12
	Elasticnet	0.9952	0.9907	0.9839	0.9604	23,540	13	23,553	12
Saliva	scTissueID	0.9624	0.9145	0.9591	0.9077	240	10	250	21
	KNN	0.7885	0.5519	0.8245	0.6524	23	752	775	26
	LDA	0.5495	0.3034	0.4716	0.3187	70,610	30	70,640	20
	NMC	0.7061	0.5199	0.6691	0.6143	71	32	103	20
	scReClassify	0.9726	0.9394	0.9724	0.9265	7,494	5,560	13,054	20
	RF	0.9385	0.8677	0.9319	0.8613	272	3	275	20
	XGBoost	0.9727	0.9393	0.9681	0.9268	189,219	17	189,236	20
	Elasticnet	0.9213	0.8264	0.9109	0.8286	58,102	35	58,137	20

^1^The training time cost in second.

^2^The testing time cost in second.

^3^The total computation time in second.

^4^The memory cost in gigabyte.

*scTissueID’s performance on major forensic tissue types.* Table 2 summarized the performance comparisons among scTissueID (with SVM), KNN, NMC, LDA, scReClassify, RF, XGBoost, and Elastinet. All measures were the average of three replicates. Except for the first dataset “Cells Passed QC”, all other datasets in this table did not apply quality control. These algorithms have been previously applied in different aspects of single-cell analysis. For example, KNN guides Seurat to determine the distance among cells, which is a critical step of cell clustering. Due to the noise inherent in single-cell experiments, such as ambient mRNAs and dropouts [124,125], the accuracy varied among different tissues and experiments. In general, SVM outperformed other algorithms on all collected forensic tissue types (except saliva) with accuracies of at least 0 . 95. XGBoost achieved a higher accuracy than scTissueID only for saliva, but XGBoost was two or three magnitudes slower than SVM. It took more than 12 days for XGBoost to obtain results with "Cells Passed QC," while it was less than one hour for scTissueID. The performance measures of XGBoost with all cells were not obtained due to extremely long running time and limited computational resources. Thus, SVM was chosen as the core classification algorithm in scTissueID.

**Table 3:** The performance of cell type prediction by MGMV.

	Performance				
Datasets	Top n marker genes	Accuracy	ARI	MF1	NMI
Cells Passed QC	n = 1	0.5696	0.7189	0.3489	0.6644
	n = 3	0.5276	0.6055	0.2693	0.6286
	n = 5	0.4234	0.5526	0.0909	0.5259
	n = 10	0.3658	0.4953	0.0426	0.5211
	n = 30	0.3681	0.4241	0.0205	0.4767
All Cells	n = 1	0.5515	0.6894	0.3384	0.6397
	n = 3	0.5157	0.5694	0.2554	0.6116
	n = 5	0.4314	0.5173	0.0924	0.5244
	n = 10	0.3701	0.5018	0.0461	0.5210
	n = 30	0.3483	0.3809	0.0227	0.4621
Blood	n = 1	0.5645	0.3426	0.5275	0.5329
	n = 3	0.5709	0.3410	0.4706	0.5003
	n = 5	0.5569	0.3434	0.4253	0.5087
	n = 10	0.5033	0.3036	0.3618	0.4816
	n = 30	0.3645	0.1903	0.0584	0.3623
Skin	n = 1	0.8132	0.7158	0.8588	0.6182
	n = 3	0.9059	0.8389	0.9038	0.7704
	n = 5	0.9259	0.8650	0.9182	0.8040
	n = 10	0.9113	0.8506	0.8803	0.7902
	n = 30	0.8493	0.7431	0.8280	0.7200
Semen	n = 1	0.6955	0.4459	0.8073	0.5801
	n = 3	0.6405	0.5074	0.7044	0.6106
	n = 5	0.6354	0.5137	0.7304	0.6146
	n = 10	0.6258	0.5035	0.6491	0.5770
	n = 30	0.6117	0.5029	0.5989	0.5796
Urine	n = 1	0.6077	0.2754	0.4544	0.3542
	n = 3	0.6924	0.3523	0.7379	0.3712
	n = 5	0.8442	0.6438	0.6598	0.4824
	n = 10	0.8693	0.7013	0.6889	0.5078
	n = 30	0.8901	0.8233	0.4300	0.5983
Saliva	n = 1	0.3468	0.1678	0.4046	0.3701
	n = 3	0.5017	0.2135	0.4742	0.4372
	n = 5	0.5226	0.2343	0.4959	0.4497
	n = 10	0.3649	0.1587	0.1433	0.3112
	n = 30	0.3071	0.0559	0.0995	0.2168

*Baselines’ performance on major forensic tissue types.* To further evaluate the robustness of scTissueID, we compared the performance with seven baseline algorithms (Table 2). scTissueID substantially outperforms baselines. Distance-based algorithms, such as KNN and NMC, could only classify around 80*%* of cells correctly. In addition, the performance of KNN varied largely in different tissues. Specifically, KNN predicted 60*%* and 78*%* of cells correctly in Blood and Saliva, respectively. This performance was probably due to the existence of many related T cell and Acinar sub-types (Fig 2a and 2h), in Blood and Saliva samples, respectively. Therefore, neighboring cells from different cell types reduced the accuracy of distance-based algorithms. Distribution-based algorithm, LDA, assumes Guassian distributed gene counts, while most gene counts follow a Boltzmann like distribution with zeros as the minimum value (Fig 2b, 2c, 2f, 2g, and 2j). This assumption limits the performance of LDA, i.e., 46*%* accuracy in the Saliva sample. In addition, KNN computed the pair-wised distance among cells, and therefore, required longer computation time (i.e., 5,115 seconds). LDA computed the distance between each cell to the centroid of each cell type, and required 54,973 seconds of computation. XGBoost and Elasticnet generally provided accurate prediction and the performances were consistent across different samples, but required much longer computation time. scReclassify demonstrates high accuracy in segregated tissue types, achieving an accuracy of 0 . 9923 for the Skin sample. Its computational costs are comparable to LDA. However, accuracy diminishes when applied to mixed tissue types, potentially due to increased noise levels and batch effects inherent in tissue mixtures. While reclassification improves accuracy, it cannot fully resolve issues like dropout or ambient mRNA contamination in some reference cells with corrected labels.. Overall, distance-based algorithms require intensive computation and are heavily dependent on the cell type labels of neighboring cells, and distribution-based algorithms place strong assumptions on the single-cell data distribution. In contrast, scTissueID considers the global gaps among cell types and satisfies the classification task of cell types in tissue mixtures.

**Table 4:** Cell type annotation performance study on HCA datasets.

	scTissueID				XGBoost^1^				Elasticnet^2^			
Datasets	Accuracy	Train^3^	Test^4^	Memory^5^	Accuracy	Train	Test	Memory	Accuracy	Train	Test	Memory
HCA_Bladder	0.9998	378	22	38	0.9712	346,326	18	40	0.9606	65,888	57	40
HCA_Blood	0.9985	469	29	76	0.9778	124,014	23	38	0.9428	294,432	146	38
HCA_Bone	0.9984	177	9	18	0.9323	213,519	13	20	0.8755	38,991	53	20
HCA_Eye	0.9992	138	9	16	0.9409	225,550	14	18	0.9272	81,441	102	18
HCA_Fat	0.9999	165	12	33	0.9865	212,584	18	34	0.9754	66,783	50	34
HCA_Heart	0.9998	70	5	18	0.9829	61,578	15	19	0.9507	11,652	19	19
HCA_Kidney	1.0000	58	5	16	0.9899	48,181	12	16	0.9883	9,992	21	16
HCA_LargeIntestine	0.9986	133	10	21	0.9480	228,705	15	23	0.9157	5,323	65	23
HCA_Liver	0.9995	38	4	8	0.9690	39,848	11	8	0.9509	12,603	36	8
HCA_Lung	0.9998	490	33	53	0.9477	127,430	20	27	0.9370	276,684	180	27
HCA_LymphNode	0.9958	415	28	57	0.9747	150,847	23	41	0.8257	310,228	160	40
HCA_Mammary	0.9999	76	7	18	0.9840	113,286	15	19	0.9720	30,610	44	19
HCA_Muscle	0.9999	228	18	48	0.9759	58,196	20	23	0.9558	131,927	83	50
HCA_Pancreas	0.9999	105	8	21	0.9793	128,709	15	22	0.9542	39,560	48	22
HCA_Prostate	0.9997	130	10	23	0.9066	32,861	20	13	0.8393	62,062	71	27
HCA_Skin	0.9993	45	5	11	0.9721	168,173	15	16	0.7590	47,986	73	16
HCA_SmallIntestine	0.9997	114	8	19	0.9571	197,900	15	21	0.9191	49,745	66	21
HCA_Spleen	0.9986	293	20	48	0.9258	62,149	21	46	0.8968	187,204	108	54
HCA_Thymus	0.9997	350	27	51	0.9478	101,719	19	26	0.9204	232,225	143	55
HCA_Tongue	0.9999	117	8	24	0.9854	135,273	15	25	0.9649	40,640	40	25
HCA_Trachea	0.9991	85	7	15	0.9780	15,218	16	7	0.9577	33,154	63	16
HCA_Uterus	0.9986	54	5	10	0.9869	58,841	11	12	0.9209	16,227	40	12
HCA_Vasculature	1.0000	174	11	26	0.9860	150,240	14	26	0.9784	59,896	54	26

^1^No quality control was implemented for XGBoost.

^2^No quality control was implemented for Elasticnet.

^3^The training time cost in second.

^4^The testing time cost in second.

^5^The space cost in gigabyte.

*Lower cell type classification accuracy by marker genes.* Having been accepted as cell type indicators, the marker genes are widely applied to identify cell types. To compare with scTissueID, cells were classified by the method of majority vote of marker genes (MGMV). MGMV showed much lower and inconsistent accuracies among different tissue types (e.g., 0 . 8132 and 0 . 5226 among Skin and Saliva, respectively) (Table 3). The shared marker genes among different tissues, such as DCN in both Fibroblast and Leydig cells (Fig 2g and 2j, respectively), reduced the accuracy. Interestingly, a recent study claimed that activating three transcriptional factors can reprogram Fibroblast to Leydig cells, which are functionally different cell types [126]. This again suggested that cell types from different tissues can share the same marker genes and classification only based on marker genes can be ambiguous. In addition, the increasing number of marker genes reduced classification accuracy. With the top 30 marker genes, the accuracy was the lowest in each tissue type. In contrast, using the complete expression profile of cells is a more reliable method to identify tissue types.

**Fig 4:**
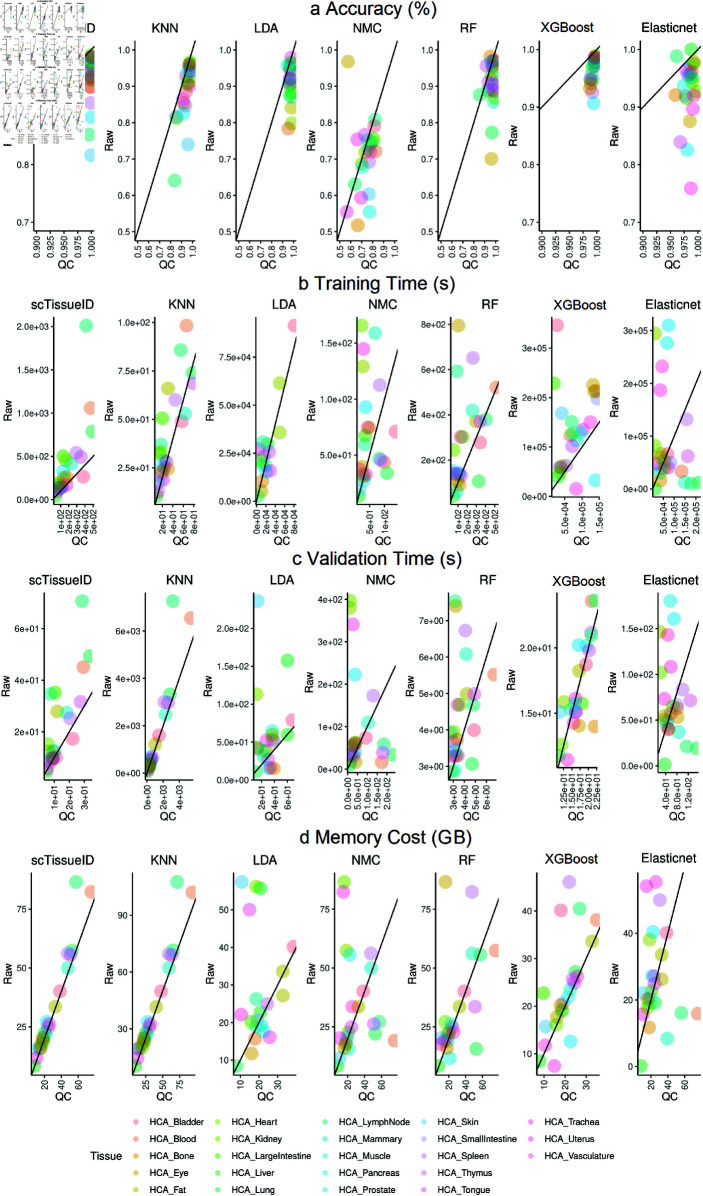
Human Cell Atlas tissue type classification performance improvement by quality control of eliminating noisy cells. Each dot represents one tissue. The accuracy (a), training time (b), validation time (c), and memory cost (d) of 10-fold cross-validation by the scTissueID and baseline methods. The dot plots compare the performance between raw cells (y-axis) as reference and high quality cells (x-axis) as reference. The dots below the diagonal lines are shown as the improved performance. The scTissueID classifies all tissue types, while the baselines with training time over 5⋅10^5^ are not shown.

**Fig 5:**
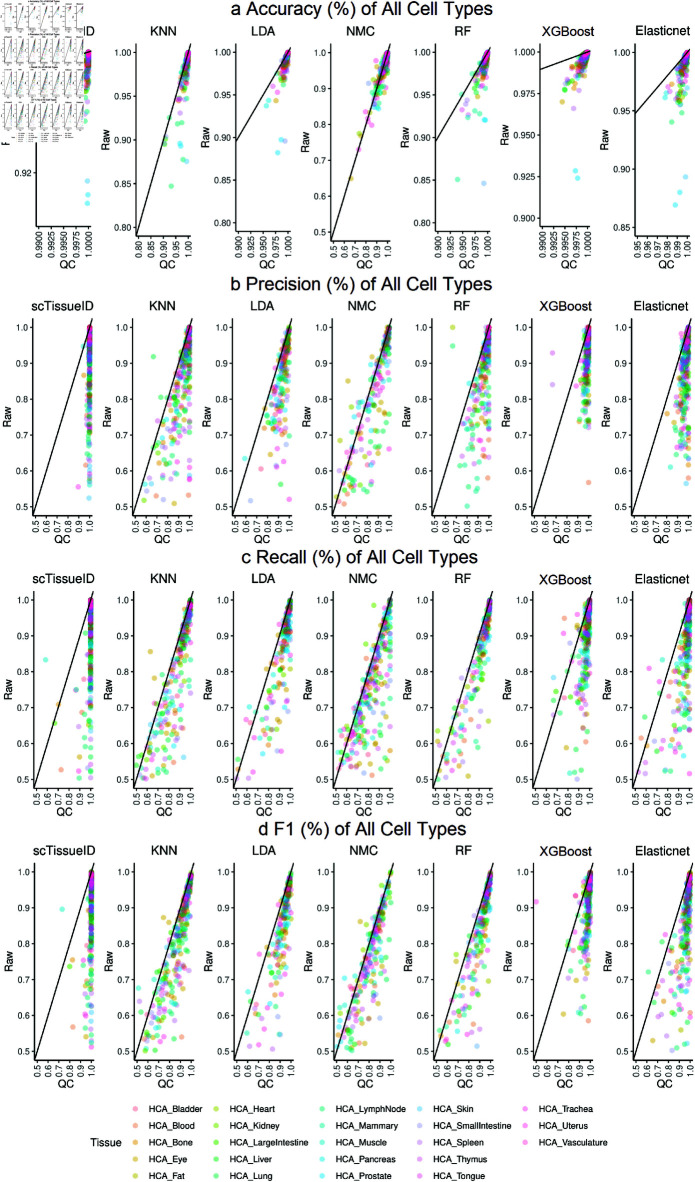
Human Cell Atlas cell type classification performance with all cell types. Each dot represents one cell type. All methods were evaluated with or without quality control on cells, namely, Raw and QC (Quality Control), respectively. The accuracy (a), precision (b), recall (c), and f1 (d) of 10-fold cross-validation by scTissueID and baseline methods. Each dot denotes the performance of one cell type, while all dots with the same color denote cell types of one tissue. The dot plots compare the performance between raw cells (y-axis) as reference and high quality cells (x-axis) as reference. The dots below the diagonal lines are shown as the improved performance. scTissueID classifies all tissue types, while the baselines with training time over 5⋅10^5^ are not shown.

*scTissueID’s performed best on Human Cell Atlas datasets.* We further evaluated scTissueID’s performance on Human Cell Atlas database, including 23 major human tissue types (Table 4). Table 4 shows cell type annotation performance, and no quality control was implemented for XGBoost and Elasticnet. In general, scTissueID achieved higher performance on HCA datasets with less training computation resources (Table 4, Figs 4, and 5). Fig 4 evaluated the tissue type classification, and Fig 5 evaluated the cell type classification (i.e., annotation).

Similar to the results in Table 2, scTissueID outperformed other baseline algorithms. Over 99 . 8*%* of the cells in each tissue type can be accurately classified (Table 4). Most dots of scTissueID in Figs 4a and 5 were close to 1.0 (i.e., 100*%*) after quality control (i.e., X-axis), for all the metrics. However, the dots of other algorithms spread much wider (i.e., away from 1 . 0), indicating lower and less robust performance.

The accuracy was heavily dependent on the quality of the cells. scTissueID’s quality control can increase the accuracy from 0 . 7 to over 0 . 9 on all baselines (Fig 5). However, without the quality control, XGBoost and Elastinet’s performance among different HCA tissues fluctuated more, ranging from 0 . 7590 to 0 . 9883 (Table 4).

The cell quality estimation improved the classification accuracy by up to 20*%* (Fig 4a), suggesting that the cell type classification can be precisely depicted within the linear gene expression space, and the noisy cells can be precisely determined by the quality scores. Considering that scTissueID required less than one hour’s training in all HCA tissues (Fig 4b), it can be efficiently adapted to curate reference datasets. To the best of the authors’ knowledge, our curated HCA training reference is the first with the consideration of scRNA-Seq cell quality control, and is shown as important to improve cell type annotation accuracy.

Considering the impact of imbalanced cell quantity in each HCA tissue, we evaluated the precision, recall, F1 scores in each of the cell types among tissues (Fig 5). With scTissueID, the precision of each cell type was over 0 . 90 and the recall of most cell types was over 0 . 90, which confirmed that scTissueID’s high accuracy was not biased towards imbalanced cell populations. XGBoost also had good performance second to scTissueID, but took much longer computational time. The substantially longer training resulted in inflexibility in customizing training models.

**Fig 6:**
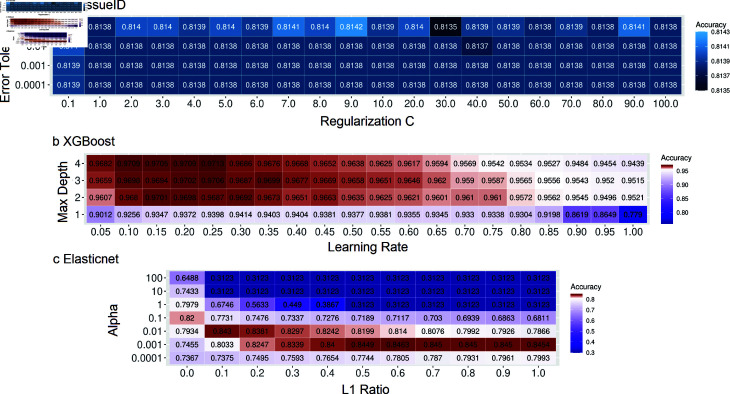
Hyper-parametric sensitivity study by Human Cell Atlas skin sample. The 10-fold cross-validation accuracy of grid search on scTissueID (the Error Tolerance and Regularization C) (a), XGBoost (the Tree Depth and Learning Rate) (b), and Elasticnet (the Alpha and L1 Ratio) (c) methods.

*scTissueID’s performance is parametric insensitive.* As many scRNA analysis pipelines are dependent on hyperparameter optimization, we investigated the impact of hyperparameters for scTissueID, XGBoost, and Elasticnet. As shown in Fig 4a, we identified the HCA_Skin tissue type as having low and varied accuracy predicted by most algorithms and the least accurate by scTissueID, without considering the cell quality. A parameter robustness study was performed based on HCA_Skin tissue (Fig 6a), including all available skin cells. Due to limited computational resources, only the two most important hyperparameters with reasonable value ranges were included in each of the methods. For scTissueID, the error tolerance, as the termination criteria, and the regularization C, to determine the separation hyperplane of linear SVM, were chosen as the major parameters. XGBoost’s learning rate and max tree depth, and Elasticnet’s L1 ratio and alpha (as the learning rate) were chosen.

The accuracy of scTissueID ranged between 0 . 8135 to 0 . 8143, suggesting that a grid search among common parameter combinations resulted in a limited improvement. In contrast, much higher variance in the accuracies was observed with XGBoost (Fig 6b) (ranging from 0 . 7790 to 0 . 9709) and Elasticnet (Fig 6c) (ranging from 0 . 3123 to 0 . 8463). This study also suggested that the classification margin between two different cell types in the linear SVM space was sufficiently large to differentiate those cell types. Considering the marginal accuracy improvement by the parameter space search and the substantial improvement achieved by the cell quality control estimation, the cell quality was again deemed a very important determinant and the limiting factor in cell type annotation.

Moreover, the computation time required by XGBoost and Elasticnet was orders of magnitude higher than that of scTissueID (Table 4), rendering the grid search for optimal parameters impractical, particularly with the increased sample size. Conversely, scTissueID showed resilience to hyperparameters, allowing for more flexible and tailored training approaches.

**Table 5:** Independent validations on cell type classification.

	Performance			
Training set	Validation set	*#* Validation cells	Method	Accuracy
Blood	10Xv2^1^	3,362	scTissueID	0.9671
Skin			scVI	0.8149
Semen			SingleR	0.7371
Urine			scMapCell	0.6462
Saliva			SingleCellNet	0.6237
			scID	0.4530
			CelliD	0.8359
			scNym	0.1865
Blood	10Xv3^2^	3,184	scTissueID	0.8301
Skin			scVI	0.7666
Semen			SingleR	0.6687
Urine			scMapCell	0.7394
Saliva			SingleCellNet	0.7136
			scID	0.5957
			CelliD	0.7106
			scNym	0.2462
Blood	Semen1	20,559	scTissueID	0.9667
Skin			scVI	0.9823
Semen			SingleR	0.9671
Urine			scMapCell	0.9672
Saliva			SingleCellNet	0.9658
			scID	0.0891
			CelliD	0.7368
			scNym	0.0457
HCA_Blood	Blood	20,000	scTissueID	0.9505
			scVI	0.9585
			SingleR	0.8803
			scMapCell	0.9958
			SingleCellNet	0.9474
			scID	0.4154
			CelliD	0.9857
			scNym	0.0000
Lung_Atlas_10x^3^	Lung_Atlas_Smartseq2^4^	9,409	scTissueID	0.8549
			scVI	0.7373
			SingleR	0.7305
			scMapCell	0.8373
			SingleCellNet	0.7495
			scID	0.2976
			CelliD	0.7320
			scNym	0.8347
Lung_Atlas_Smartseq2	Lung_Atlas_10x	65,662	scTissueID	0.8787
			scVI	0.7697
			SingleR	0.5757
			scMapCell	0.8318
			SingleCellNet	0.7864
			scID	0.3461
			CelliD	0.6542
			scNym	0.5110

^1^Blood tissue by 10X platform.

^2^Blood tissue by 10X platform.

^3^Lung tissue by 10X platform.

^4^Lung tissue by Smartseq2 platform.

*Comparison with other baseline pipelines.* scTissueID was also compared with seven state of art pipelines using six pairs of training and validation samples (Table 5). scTissueID performed the best except one blood and the sperm samples. In particular, for the 10Xv3 dataset, scTissueID was the only pipeline that can correctly classify over 95*%* cells. In addition, scTissueID only misclassified 2 cells as irrelevant cell types, and 23 T cells as Natural Killer cells in 10Xv2 and 10Xv3 samples. In contrast, scVI misclassified 402 out of 486 monocytes and 56 T cells as Natural Killer cells. As for the Semen1 dataset, the majority of sperm cells were correctly classified by most pipelines except scID and scNym. The Gaussian mixture model assumption in scID may limit its classification performance. In general, scTissueID performed consistently well among validation datasets, but other pipelines only performed well with some specific tissues.

The accuracies of the baseline methods varied substantially across the six pairs of training and validation sets. For instance, scNym, employing an adversarial neural network, achieved accuracies of 0 . 8347 and 0 . 5110 in lung tissue samples but very few (e.g., 0 . 0457) in Semen1 tissue (Table 5). In contrast, scTissueID showed much less variability in accuracies across tissues, ranging from 0 . 8301 to 0 . 9671.

### 2.3. Performance on challenging situations

*scTissueID accurately classified imbalanced tissue mixture.* A sample can be mixed from multiple tissues with imbalanced proportions from multiple donors. Thus, the classification accuracy was further evaluated with five imbalanced mixtures (Table 6). By downsampling each tissue from 0 . 1*%* (or 3 cells) to 50*%* (or 1 , 500 cells) with 5 tissue repeats and random 10 trials, scTissueID maintained the high average cell type accuracy as 0 . 9994. When two or more minor tissues are mixed with other types (4 , 833 cells), the accuracy increased to 0 . 9998. Even if only capturing three cells in a sample, scTissueID can accurately identify them without substantial performance reduction, and thereby provide strong evidence of the tissue types. This high accuracy was mostly because the machine learning model was based on individual cells, not in a bulk fashion. Namely, the machine learning model does not see a mixture sample, and the mixture is treated as a batch of input for the prediction with the machine learning. Therefore, the classification accuracy of a mixture is simply the overall accuracy of the batch input. Since the classification accuracy for individual cells is close to 100%, the overall accuracy should also be close to 100%. Again, this high accuracy further supports that the heterogeneity among cell and tissue types could be accurately depicted with scRNA-Seq profiles.

**Table 6:** The scTissuID classification performance of imbalanced mixture tissues.

	Classify imbalance mixture tissues by scTissueID				
Tissue mixture ratio	Total Cells	Accuracy	ARI	MF1	NMI
0.001:1:1:1:1^1^	12,003	0.9994	0.9994	1	0.9988
0.01:1:1:1:1	12,030	0.9994	0.9994	1	0.9988
0.1:1:1:1:1	12,300	0.9993	0.9994	1	0.9988
0.5:1:1:1:1	13,500	0.9993	0.9994	1	0.9988
0.001:0.01:0.1:0.5:1	4,833	0.9998	0.9998	1	0.9996

^1^Each of the five tissue types is downsampled to 0 . 1*%* of other tissues.

**Fig 7:**
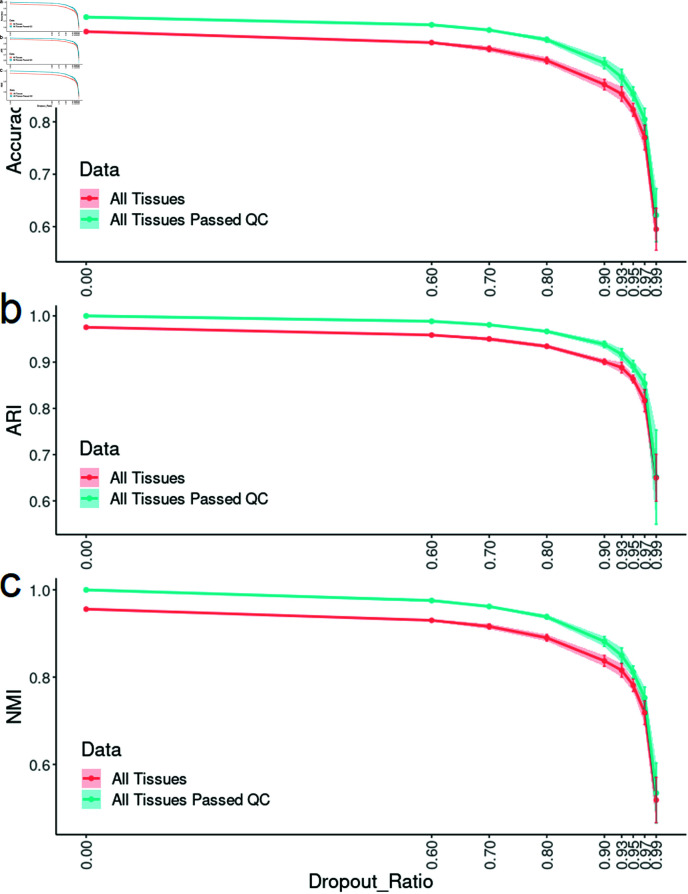
Impact of gene dropout on cell type classification performance. The Accuracy (a), ARI (b), and NMI (c) scores of 10-fold cross-validation by the scTissueID. The standard deviation of 10 folds is denoted by the error bar. The dropout ratio is the percent of gene dropped out (e.g., 99*%* dropout ratio means only 1*%* of genes was sampled).

*Impact of gene dropout on scTissueID accuracy.* Genetic marker and allele dropouts were commonly observed in DNA samples from criminal scenes. We evaluated the classification accuracy when only partial scRNA profiles were available (Fig 7). By randomly dropping out 0*%* to 99*%* of genes from each tissue with 10 trials, scTissueID’s accuracy was reduced from 0 . 9998 to 0 . 5950 and the standard deviation increased from less than 0 . 0001 to 0 . 1011. scTissueID maintained a high accuracy of 0 . 9014 even with 90*%* of genes dropped out. In addition, even if the gene drop out ratio was high, the quality control can still increase the accuracy about 1 ∼ 3*%* (e.g., 0 . 9119 after QC with 90*%* gene dropped out). Note that improving accuracy is very challenging because capturing thousands and tens of thousands of genes result in an accuracy of 0 . 9014 and 0 . 9998, respectively. In general, the number of captured genes was a dominant factor of tissue identification, and scTissueID was able to classify tissue types accurately with only a few thousand genes. However, randomly capturing a few hundred genes was unlikely to precisely determine the tissue types.

## 3. Discussion

Single-cell technologies have found successful applications in numerous biomedical and clinical contexts, including mixture interpretation in forensics [127,128]. In this study, we leveraged the latest advancements in single-cell transcriptomics profiling to identify cell and tissue types, and developed a pipeline called scTissueID. Through a large-scale and comprehensive comparison study using both forensic-relevant and Human Cell Atlas (HCA) data, scTissueID demonstrated better and consistent performance in determining cell and tissue types compared to a vast array of algorithms and state-of-the-art pipelines. scTissueID initially clusters cells using the Louvain algorithm with PCA dimension-reduced data. Subsequently, it reclassifies the cells with all genes based on the clustering results using an SVM to generate quality scores for each cell. Only cells that surpass the quality score threshold (i.e.,  ≥ 0 . 8) are retained in the reference (i.e., training set). The subsequent SVM classification utilizes this reference to develop a model for classifying other samples.

This study was the first to demonstrate the crucial role of quality differentiation in subsequent scRNA-Seq data analysis. Filtering out noisy data based on quality scores substantially improved the accuracies of cell and tissue type identifications. Unlike many state-of-the-art pipelines that assume the high quality of reference cells, our findings suggest that this assumption could compromise annotation accuracy, particularly for customized references. Our study identified a large number of noisy cells across various databases, underscoring their effect on cell annotation accuracy. This quality control step can be adopted in many other single-cell pipelines and applications to enhance their performance. For instance, integrating cell quality control can improve unsupervised cell clustering and subsequent gene differential expression analysis, ensuring that only clean cells’ expression profiles are considered when determining differentially expressed genes.

We typically selected 0 . 8 as the default cell quality score cutoff threshold in scTissueID. However, this parameter may be adjusted for different datasets from various tissues or with different sequencing qualities. Applying a more stringent cutoff threshold could result in a cleaner reference set, but it also carries the risk of reducing the sample sizes of the training set, potentially compromising the model’s generalization capability.

scTissueID adopted SVM as the core algorithm for classification. This linear classifier not only achieves better classification performance but also runs multiple magnitudes faster than other high-performance classifiers (e.g., XGBoost). scTissueID also performed well with challenging samples, such as imbalanced mixtures and substantial gene dropouts. Even with a 90*%* gene dropout, scTissueID maintained  > 90*%* accuracy for the five forensic samples. The comparison with MGMV also confirmed that selecting a small number of markers may not work well for cell identification, especially when dropouts are common. Thus, marker selection is no longer necessary, which reduces the labor intensive efforts to investigate the detailed effects of individual markers.

Despite the versatility of the learning model, we opted not to utilize neural network-based deep learning algorithms in this study. This decision was influenced by the fact that optimizing the hyperparameters of a neural network relies heavily on the training data, and adding additional training data or tissue types would necessitate a complete re-optimization of the network. Additionally, deep learning methods may not always yield optimal results for structured data with a limited number of samples. In contrast, SVM offers a much lighter-weight and parametrically less sensitive solution in this case, and re-optimizing its hyperparameters may not substantially improve the performance, as shown in Fig 6.

The SVM classification offers the probability that each cell belongs to each tissue. In scenarios where a Likelihood Ratio (LR) presentation is preferred, LRs can be calculated from these probabilities by comparing the probabilities for different tissues or by assessing the probabilities with or without the presence of a particular tissue. Such analysis can be crucial in determining the nature of a crime case.

Tissues can often contain similar or identical types of cells. For instance, both urine and saliva may contain many different, yet similar, epithelial cells. Despite this similarity, scTissueID was capable of accurately distinguishing between urine and saliva, even when they contain similar epithelial cells. It is therefore reasonable to expect that scTissueID could also successfully differentiate tissue or cell types for other organs or tissues not included in this study. Furthermore, when dealing with mixtures comprising multiple donors and tissue types, scRNA profiles alone may not provide precise information regarding the number of donors contributing to the mixture and which tissue originates from which donor. However, with multimodal single-cell profiling, which incorporates both DNA and RNA (e.g., Chromium Single Cell Multiome ATAC + Gene Expression [129] and TEA-Seq [130]), it becomes possible to directly associate donors with tissues, even specifying the precise proportions of the donors and tissues. This capability is extremely challenging to achieve with current bulk sequencing-based solutions [9].

Although scTissueID exhibits improved performance, there are scenarios where caution should be exercised when using it. Firstly, developmental transitional stage cells may be erroneously filtered out as noisy by scTissueID. If these transitional stage cells are deemed important in a particular study, it is advisable to annotate them as an independent cell type rather than as transitions between two cell types. Secondly, when cell types are clearly separable in a dataset (i.e., when there is not much noisy data), the performance of other methods may be close to or comparable with that of scTissueID. In addition, in scenarios where datasets include cell types with a very limited number of cells, the accuracy of cell quality scores may be compromised, as machine learning algorithms may prioritize larger clusters.

As depicted in Fig 3f, scTissueID identifies cells in transitional stages as noisy. However, in developmental studies, cell quality control may not always be reliable, particularly during cell differentiation. The observed improvement in the performance of other algorithms with quality control suggests that when cell quality is already high, other algorithms are comparable with scTissueID in terms of accuracy. Nevertheless, it is important to note that achieving very high cell quality from experiments is often rare due to the challenges associated with sample collection and the complexity of single-cell protocols. Additionally, scTissueID relies on a large quantity of cells within a group to estimate quality. Consequently, cell types with few cells may result in inconsistent quality estimation. We regard addressing these challenging scenarios as a potential future direction for this study.

Currently, our study does not focus on donor-specific or human identification classification. However, single-cell multiomics could be a promising approach for integrating tissue identification with human identification in future research. The reference datasets used in our study include multiple donors, indicating that tissue identification via scRNA-Seq can be performed independently of human identification. This proof-of-concept study focuses on tissue identification through accurate scRNA-Seq cell type reference-based classification, a critical forensic application where scRNA-Seq holds significant potential. At the same time, this pipeline also serves as a highly accurate general-purpose cell type annotation tool. We conclude that reference cell quality differentiation is a more critical factor for accurate classification than dimension reduction techniques or the specific algorithm used, which may have been previously undervalued.

## Conclusion

In conclusion, this study emphasizes the importance of reference cell quality differentiation in scRNA-Seq-based tissue classification. Our unique quality differentiation enhances accuracy by excluding potentially precarious reference cells, addressing noise and variability, and enabling higher resolution classification with applications in forensic and biomedical research.

## Supporting information

S1 Text (PDF)
