# Supplementary material for: High resolution tissue and cell type identification via single cell transcriptomic profiling
Source: PLoS One. 2025 Mar 26;20(3):e0318151. doi: 10.1371/journal.pone.0318151 (PMC11940611; doi:10.1371/journal.pone.0318151)
Supplement: S1 Text [file pone.0318151.s001.pdf]

Table S1. Marker genes in 5 tissue types (p-value<0.05)

Marker	p_val	avg_log2F(	pct.1	pct.2	p_val_adj	cluster	gene
MTRNR2L1	0	4.117179	0.444	0.116	0	Saliva_Acir	MTRNR2L1
RP11.1143	0	4.018675	0.739	0.214	0	Saliva_Acir	RP11.1143G9.4
MTRNR2L1	0	3.841112	0.673	0.257	0	Saliva_Acir	MTRNR2L12
MT.CYB	0	3.190218	0.97	0.933	0	Saliva_Acir	MT.CYB
MT.ND3	0	3.184309	0.956	0.79	0	Saliva_Acir	MT.ND3
MT.ATP6	0	3.151279	0.973	0.896	0	Saliva_Acir	MT.ATP6
MT.CO3	0	3.020361	0.977	0.977	0	Saliva_Acir	MT.CO3
MTRNR2L1	0	3.004714	0.384	0.089	0	Saliva_Acir	MTRNR2L10
MT.CO2	0	2.935702	0.968	0.959	0	Saliva_Acir	MT.CO2
MTRNR2L8	0	2.83314	0.511	0.28	0	Saliva_Acir	MTRNR2L8
C6orf58	0	2.826695	0.856	0.197	0	Saliva_Acir	C6orf58
ZG16B	0	2.795275	1	0.397	0	Saliva_Acir	ZG16B
LYZ	0	2.756716	0.994	0.318	0	Saliva_Acir	LYZ
MT.ND1	0	2.535864	0.935	0.927	0	Saliva_Acir	MT.ND1
MT.ND5	0	2.45134	0.772	0.701	0	Saliva_Acir	MT.ND5
MT.ND4	0	2.400417	0.941	0.954	0	Saliva_Acir	MT.ND4
MT.ND2	0	2.33572	0.893	0.908	0	Saliva_Acir	MT.ND2
AZGP1	0	2.038824	0.682	0.192	0	Saliva_Acir	AZGP1
MT.CO1	0	1.988935	0.952	0.986	0	Saliva_Acir	MT.CO1
TCN1	0	1.972697	0.532	0.145	0	Saliva_Acir	TCN1
GNAS	0	1.87764	0.81	0.576	0	Saliva_Acir	GNAS
SLPI	0	1.710299	0.904	0.482	0	Saliva_Acir	SLPI
UBB	1.5E-262	0.352143	0.326	0.743	2.4E-258	Saliva_Acir	UBB
MT.ND4L	8.7E-257	2.582832	0.568	0.412	1.4E-252	Saliva_Acir	MT.ND4L
MT.ATP8	5.2E-185	1.987132	0.295	0.131	8.3E-181	Saliva_Acir	MT.ATP8
FAM3D	1.2E-175	1.315961	0.432	0.234	1.9E-171	Saliva_Acir	FAM3D
FKBP8	4.5E-138	0.341547	0.089	0.348	7.1E-134	Saliva_Acir	FKBP8
MTRNR2L6	1.5E-128	0.903899	0.089	0.02	2.3E-124	Saliva_Acir	MTRNR2L6
MKRN1	5.9E-119	0.291677	0.029	0.225	9.4E-115	Saliva_Acir	MKRN1
GYPC	4.7E-108	0.36111	0.027	0.209	7.4E-104	Saliva_Acir	GYPC
CCL28	1.01E-97	1.100181	0.228	0.108	1.62E-93	Saliva_Acir	CCL28
HBA1	1.26E-88	1.908182	0.073	0.018	2.01E-84	Saliva_Acir	HBA1
FBXO7	6.29E-84	0.26775	0.02	0.163	1E-79	Saliva_Acir	FBXO7
KIAA1324	5.12E-76	0.966898	0.21	0.107	8.17E-72	Saliva_Acir	KIAA1324
CLDN10	4.59E-73	1.132064	0.189	0.093	7.33E-69	Saliva_Acir	CLDN10
S100A1	7.31E-62	0.879146	0.195	0.102	1.17E-57	Saliva_Acir	S100A1
SLC25A39	4.48E-61	1.044353	0.049	0.182	7.15E-57	Saliva_Acir	SLC25A39
DCAF12	4.72E-58	0.359425	0.013	0.113	7.54E-54	Saliva_Acir	DCAF12
HAGH	1.83E-56	0.264927	0.031	0.142	2.92E-52	Saliva_Acir	HAGH
BCL2L1	2.29E-51	0.302634	0.012	0.101	3.65E-47	Saliva_Acir	BCL2L1
SSR4	2.68E-41	0.536768	0.538	0.549	4.27E-37	Saliva_Acir	SSR4
MT.ND6	2.97E-40	1.238925	0.177	0.109	4.74E-36	Saliva_Acir	MT.ND6
HBA2	3.6E-40	2.1247	0.077	0.032	5.74E-36	Saliva_Acir	HBA2
LPO	2.98E-38	0.702362	0.1	0.048	4.75E-34	Saliva_Acir	LPO
FXYD3	2.22E-35	0.863769	0.308	0.231	3.54E-31	Saliva_Acir	FXYD3

SLC25A37	3.64E-28	1.662517	0.085	0.179	5.8E-24	Saliva_Acir	SLC25A37
XBP1	5.2E-27	0.80339	0.385	0.342	8.29E-23	Saliva_Acir	XBP1
STRADB	6.85E-26	0.295261	0.011	0.06	1.09E-21	Saliva_Acir	STRADB
FAM210B	3.09E-25	0.606857	0.033	0.096	4.93E-21	Saliva_Acir	FAM210B
C8orf4	3.69E-24	1.257801	0.171	0.119	5.89E-20	Saliva_Acir	C8orf4
CLDN3	1.26E-21	0.828675	0.145	0.098	2.01E-17	Saliva_Acir	CLDN3
GMDS	2.46E-21	0.25641	0.092	0.173	3.93E-17	Saliva_Acir	GMDS
LAMP2	1.51E-20	0.303015	0.139	0.239	2.41E-16	Saliva_Acir	LAMP2
LRRC26	3.93E-18	0.637354	0.118	0.077	6.28E-14	Saliva_Acir	LRRC26
MPP1	7.03E-18	0.295524	0.009	0.044	1.12E-13	Saliva_Acir	MPP1
EPCAM	5.77E-17	0.72389	0.173	0.13	9.2E-13	Saliva_Acir	EPCAM
TPT1.AS1	4.23E-16	0.364572	0.045	0.095	6.75E-12	Saliva_Acir	TPT1.AS1
TMED3	2.83E-15	0.987405	0.3	0.278	4.52E-11	Saliva_Acir	TMED3
RBM38	1.51E-14	0.374051	0.009	0.038	2.41E-10	Saliva_Acir	RBM38
REEP5	1.88E-14	0.316577	0.207	0.312	2.99E-10	Saliva_Acir	REEP5
GMPR	2.59E-14	0.410828	0.014	0.046	4.13E-10	Saliva_Acir	GMPR
SMIM1	2.87E-14	0.43444	0.031	0.071	4.58E-10	Saliva_Acir	SMIM1
XIST	9.85E-13	0.287424	0.073	0.125	1.57E-08	Saliva_Acir	XIST
RP11.532F	2.87E-12	0.250719	0.041	0.081	4.58E-08	Saliva_Acir	RP11.532F12.5
PPP1R16A	1.3E-11	0.268106	0.06	0.106	2.07E-07	Saliva_Acir	PPP1R16A
SNCA	1.76E-11	1.156585	0.018	0.047	2.81E-07	Saliva_Acir	SNCA
CHPT1	2.47E-11	0.497437	0.137	0.209	3.94E-07	Saliva_Acir	CHPT1
MGST1	2.06E-10	0.286271	0.133	0.197	3.28E-06	Saliva_Acir	MGST1
RP11.20D1	2.3E-10	0.588553	0.006	0.001	3.67E-06	Saliva_Acir	RP11.20D14.6
GK5	3.41E-10	0.308254	0.04	0.075	5.43E-06	Saliva_Acir	GK5
LGALS3BP	4.13E-10	0.823535	0.196	0.17	6.58E-06	Saliva_Acir	LGALS3BP
PTPRF	4.44E-10	0.274318	0.065	0.108	7.08E-06	Saliva_Acir	PTPRF
SEC11C	1.23E-09	0.374882	0.267	0.241	1.96E-05	Saliva_Acir	SEC11C
SELENBP1	2.02E-09	0.497924	0.043	0.077	3.21E-05	Saliva_Acir	SELENBP1
KRT19	2.02E-09	0.368449	0.156	0.122	3.22E-05	Saliva_Acir	KRT19
ANKRD36C	2.43E-09	1.516819	0.162	0.137	3.88E-05	Saliva_Acir	ANKRD36C
PHB	2.72E-09	0.847423	0.257	0.241	4.35E-05	Saliva_Acir	PHB
HBD	7.01E-09	1.154194	0.017	0.007	0.000112	Saliva_Acir	HBD
AC159540.	8.82E-09	0.279992	0.034	0.063	0.000141	Saliva_Acir	AC159540.1
TMED10	1.32E-08	0.368089	0.23	0.324	0.000211	Saliva_Acir	TMED10
TESC	2.55E-08	0.868017	0.137	0.113	0.000407	Saliva_Acir	TESC
DMTN	3.08E-07	0.291034	0.007	0.021	0.004911	Saliva_Acir	DMTN
NUCB2	7.09E-07	0.732851	0.275	0.27	0.011313	Saliva_Acir	NUCB2
OGFRL1	8.41E-07	0.340928	0.079	0.116	0.013418	Saliva_Acir	OGFRL1
FAM46A	9.96E-07	0.770965	0.184	0.164	0.01588	Saliva_Acir	FAM46A
MGLL	3.24E-06	0.471369	0.129	0.179	0.051661	Saliva_Acir	MGLL
DBI	3.36E-06	0.460225	0.314	0.418	0.053547	Saliva_Acir	DBI
SPINT2	5.68E-06	0.736498	0.302	0.314	0.090612	Saliva_Acir	SPINT2
TSPAN13	7.85E-06	0.338083	0.116	0.159	0.125273	Saliva_Acir	TSPAN13
PROM1	8.13E-06	0.256987	0.056	0.084	0.129693	Saliva_Acir	PROM1
TPD52L1	8.95E-06	0.470885	0.097	0.138	0.14272	Saliva_Acir	TPD52L1
TMEM63A	1.66E-05	0.338501	0.055	0.081	0.264409	Saliva_Acir	TMEM63A

NPDC1	1.97E-05	0.63669	0.189	0.173	0.314063	Saliva_Acir	NPDC1
CEP41	5.94E-05	0.370372	0.074	0.103	0.946891	Saliva_Acir	CEP41
TMEM125	8.35E-05	0.293024	0.032	0.05	1	Saliva_Acir	TMEM125
PYCR1	0.000161	0.372783	0.058	0.082	1	Saliva_Acir	PYCR1
LRPAP1	0.000211	0.490661	0.205	0.272	1	Saliva_Acir	LRPAP1
ACTR3B	0.000696	0.391321	0.049	0.069	1	Saliva_Acir	ACTR3B
ELF5	0.000823	0.5956	0.083	0.07	1	Saliva_Acir	ELF5
YIPF2	0.001598	0.496042	0.083	0.11	1	Saliva_Acir	YIPF2
CLDN4	0.003991	0.700262	0.1	0.09	1	Saliva_Acir	CLDN4
MYL4	0.005303	0.30416	0.004	0.009	1	Saliva_Acir	MYL4
CCDC15	0.00633	0.370887	0.046	0.061	1	Saliva_Acir	CCDC15
CXCL5	0.007503	0.364656	0.038	0.029	1	Saliva_Acir	CXCL5
ID1	0	3.510456	0.705	0.097	0	Saliva_End	ID1
A2M	0	3.454412	0.758	0.071	0	Saliva_End	A2M
VWF	0	3.222767	0.657	0.027	0	Saliva_End	VWF
AQP1	0	3.203174	0.704	0.084	0	Saliva_End	AQP1
IFI27	0	3.164566	0.875	0.185	0	Saliva_End	IFI27
CAV1	0	3.10677	0.864	0.185	0	Saliva_End	CAV1
FABP5	0	3.055116	0.617	0.126	0	Saliva_End	FABP5
RAMP2	0	2.962015	0.682	0.063	0	Saliva_End	RAMP2
GNG11	0	2.947395	0.735	0.102	0	Saliva_End	GNG11
EGFL7	0	2.936072	0.66	0.067	0	Saliva_End	EGFL7
KLF2	0	2.733649	0.698	0.182	0	Saliva_End	KLF2
RAMP3	0	2.709923	0.505	0.024	0	Saliva_End	RAMP3
CLDN5	0	2.561464	0.441	0.032	0	Saliva_End	CLDN5
CLU	0	2.498909	0.604	0.192	0	Saliva_End	CLU
EPAS1	0	2.487824	0.537	0.081	0	Saliva_End	EPAS1
PLVAP	0	2.479931	0.476	0.023	0	Saliva_End	PLVAP
ENG	0	2.456919	0.528	0.048	0	Saliva_End	ENG
IGFBP4	0	2.432952	0.656	0.12	0	Saliva_End	IGFBP4
VIM	0	2.357992	0.961	0.485	0	Saliva_End	VIM
IFITM3	0	2.32785	0.86	0.3	0	Saliva_End	IFITM3
NPDC11	0	2.32459	0.651	0.163	0	Saliva_End	NPDC1
ID3	0	2.276089	0.655	0.163	0	Saliva_End	ID3
TGFBR2	0	2.238656	0.54	0.107	0	Saliva_End	TGFBR2
HLA.E	0	2.151703	0.937	0.603	0	Saliva_End	HLA.E
EMCN	0	2.14325	0.429	0.031	0	Saliva_End	EMCN
SYNE2	0	2.136513	0.521	0.13	0	Saliva_End	SYNE2
HYAL2	0	2.115758	0.411	0.049	0	Saliva_End	HYAL2
RNASE1	0	2.109817	0.556	0.084	0	Saliva_End	RNASE1
CRIP2	0	2.09807	0.617	0.196	0	Saliva_End	CRIP2
TSPAN7	0	2.059635	0.405	0.022	0	Saliva_End	TSPAN7
CD34	0	2.056449	0.465	0.06	0	Saliva_End	CD34
IFI44L	0	2.047364	0.319	0.027	0	Saliva_End	IFI44L
ARHGAP29	0	2.04063	0.452	0.108	0	Saliva_End	ARHGAP29
HSPG2	0	2.034897	0.477	0.106	0	Saliva_End	HSPG2
TXNIP	0	2.032298	0.791	0.369	0	Saliva_End	TXNIP

SDPR	0	2.029839	0.328	0.021	0	Saliva_End	SDPR
CD93	0	2.016298	0.398	0.025	0	Saliva_End	CD93
PTPRB	0	1.977931	0.372	0.011	0	Saliva_End	PTPRB
JAM2	0	1.938921	0.388	0.03	0	Saliva_End	JAM2
FLT1	0	1.927361	0.323	0.033	0	Saliva_End	FLT1
NRN1	0	1.902395	0.388	0.03	0	Saliva_End	NRN1
PTRF	0	1.896981	0.575	0.156	0	Saliva_End	PTRF
NOSTRIN	0	1.89473	0.375	0.054	0	Saliva_End	NOSTRIN
TNFSF10	0	1.890674	0.643	0.265	0	Saliva_End	TNFSF10
LMNA	0	1.879283	0.749	0.359	0	Saliva_End	LMNA
MMRN2	0	1.869861	0.348	0.015	0	Saliva_End	MMRN2
LMO2	0	1.844476	0.388	0.085	0	Saliva_End	LMO2
GNAI2	0	1.843882	0.573	0.219	0	Saliva_End	GNAI2
ITM2A	0	1.834409	0.513	0.128	0	Saliva_End	ITM2A
PDLIM1	0	1.823165	0.543	0.218	0	Saliva_End	PDLIM1
LMCD1	0	1.822533	0.403	0.078	0	Saliva_End	LMCD1
SEPW1	0	1.819694	0.649	0.353	0	Saliva_End	SEPW1
GIMAP4	0	1.796459	0.454	0.068	0	Saliva_End	GIMAP4
LIFR	0	1.780239	0.34	0.058	0	Saliva_End	LIFR
CA4	0	1.753316	0.121	0.004	0	Saliva_End	CA4
SPTBN1	0	1.742572	0.497	0.193	0	Saliva_End	SPTBN1
ADAM15	0	1.729285	0.36	0.069	0	Saliva_End	ADAM15
IGFBP2	0	1.721686	0.34	0.048	0	Saliva_End	IGFBP2
THBD	0	1.707492	0.29	0.038	0	Saliva_End	THBD
GIMAP7	0	1.704631	0.55	0.113	0	Saliva_End	GIMAP7
TCF4	0	1.682118	0.524	0.167	0	Saliva_End	TCF4
CALCRL	0	1.65944	0.31	0.028	0	Saliva_End	CALCRL
ESAM	0	1.635451	0.341	0.066	0	Saliva_End	ESAM
KCTD12	0	1.631592	0.36	0.087	0	Saliva_End	KCTD12
NR2F2	0	1.624527	0.365	0.082	0	Saliva_End	NR2F2
BCAM	0	1.601173	0.366	0.07	0	Saliva_End	BCAM
FAM110D	0	1.592511	0.262	0.007	0	Saliva_End	FAM110D
CDH5	0	1.587004	0.277	0.021	0	Saliva_End	CDH5
HEG1	0	1.562861	0.344	0.077	0	Saliva_End	HEG1
TIMP3	0	1.557078	0.541	0.159	0	Saliva_End	TIMP3
NRP1	0	1.515384	0.284	0.052	0	Saliva_End	NRP1
MKL2	0	1.510938	0.296	0.043	0	Saliva_End	MKL2
IFIT3	0	1.481351	0.236	0.034	0	Saliva_End	IFIT3
GIMAP1	0	1.466186	0.361	0.054	0	Saliva_End	GIMAP1
LDB2	0	1.46431	0.291	0.038	0	Saliva_End	LDB2
LIMS2	0	1.462415	0.266	0.027	0	Saliva_End	LIMS2
CD81	0	1.461596	0.716	0.316	0	Saliva_End	CD81
NUAK1	0	1.458645	0.266	0.032	0	Saliva_End	NUAK1
C2CD4B	0	1.452456	0.177	0.017	0	Saliva_End	C2CD4B
MEF2C	0	1.450222	0.314	0.076	0	Saliva_End	MEF2C
ELK3	0	1.425062	0.302	0.056	0	Saliva_End	ELK3
MTRNR2L1	0	1.417008	0.647	0.262	0	Saliva_End	MTRNR2L12

GIMAP6	0	1.410134	0.282	0.026	0	Saliva_End	GIMAP6
GRASP	0	1.40865	0.248	0.017	0	Saliva_End	GRASP
TMEM88	0	1.39446	0.198	0.011	0	Saliva_End	TMEM88
IGFBP7	0	1.384794	0.743	0.256	0	Saliva_End	IGFBP7
ITM2B	0	1.377023	0.909	0.703	0	Saliva_End	ITM2B
COL15A1	0	1.351516	0.272	0.052	0	Saliva_End	COL15A1
CYYR1	0	1.325772	0.24	0.018	0	Saliva_End	CYYR1
XAF1	0	1.301013	0.232	0.039	0	Saliva_End	XAF1
H3F3A	0	1.300327	0.831	0.472	0	Saliva_End	H3F3A
RASIP1	0	1.292412	0.237	0.016	0	Saliva_End	RASIP1
TM4SF18	0	1.285766	0.17	0.015	0	Saliva_End	TM4SF18
SLCO2A1	0	1.271924	0.181	0.008	0	Saliva_End	SLCO2A1
IL3RA	0	1.271282	0.237	0.017	0	Saliva_End	IL3RA
KANK3	0	1.258101	0.208	0.014	0	Saliva_End	KANK3
LRRC32	0	1.243586	0.226	0.03	0	Saliva_End	LRRC32
IFIT1	0	1.221173	0.204	0.034	0	Saliva_End	IFIT1
ADCY4	0	1.215142	0.212	0.01	0	Saliva_End	ADCY4
HLA.DRB1	0	1.185847	0.721	0.209	0	Saliva_End	HLA.DRB1
MYCT1	0	1.171314	0.212	0.021	0	Saliva_End	MYCT1
RASA4	0	1.160197	0.2	0.012	0	Saliva_End	RASA4
MT.ND11	0	1.109731	0.995	0.926	0	Saliva_End	MT.ND1
SHE	0	1.078494	0.184	0.019	0	Saliva_End	SHE
MGP	0	1.059896	0.605	0.205	0	Saliva_End	MGP
CNTNAP3B	0	1.051007	0.168	0.012	0	Saliva_End	CNTNAP3B
IGF2	0	1.049901	0.199	0.03	0	Saliva_End	IGF2
SULF2	0	1.04465	0.187	0.029	0	Saliva_End	SULF2
PLXND1	0	1.032223	0.192	0.027	0	Saliva_End	PLXND1
MT.ATP61	0	1.029125	0.999	0.896	0	Saliva_End	MT.ATP6
GBP4	0	1.025665	0.168	0.022	0	Saliva_End	GBP4
OLFM1	0	1.013631	0.181	0.021	0	Saliva_End	OLFM1
MT.ND51	0	1.006717	0.907	0.699	0	Saliva_End	MT.ND5
FAM69B	0	0.990707	0.162	0.011	0	Saliva_End	FAM69B
PREX2	0	0.986351	0.173	0.019	0	Saliva_End	PREX2
C10orf128	0	0.981695	0.179	0.018	0	Saliva_End	C10orf128
F8	0	0.946784	0.119	0.012	0	Saliva_End	F8
ACVRL1	0	0.943319	0.177	0.025	0	Saliva_End	ACVRL1
KDR	0	0.942371	0.119	0.009	0	Saliva_End	KDR
GIMAP8	0	0.935908	0.168	0.011	0	Saliva_End	GIMAP8
MT.ND31	0	0.932108	0.997	0.791	0	Saliva_End	MT.ND3
HLA.DPA1	0	0.925707	0.595	0.184	0	Saliva_End	HLA.DPA1
RASSF9	0	0.925249	0.153	0.008	0	Saliva_End	RASSF9
GSN	0	0.924525	0.776	0.369	0	Saliva_End	GSN
MT.CYB1	0	0.915398	1	0.933	0	Saliva_End	MT.CYB
TMTC1	0	0.913703	0.155	0.017	0	Saliva_End	TMTC1
AOC3	0	0.909469	0.138	0.01	0	Saliva_End	AOC3
CCL23	0	0.903264	0.105	0.006	0	Saliva_End	CCL23
AVPR2	0	0.885313	0.125	0.004	0	Saliva_End	AVPR2

TEK	0	0.8773	0.133	0.006	0	Saliva_End	TEK
TIE1	0	0.867636	0.125	0.008	0	Saliva_End	TIE1
PDE2A	0	0.858842	0.137	0.009	0	Saliva_End	PDE2A
KCNN3	0	0.849301	0.122	0.007	0	Saliva_End	KCNN3
FGD5	0	0.826172	0.144	0.015	0	Saliva_End	FGD5
S1PR1	0	0.803324	0.146	0.017	0	Saliva_End	S1PR1
KIAA1462	0	0.751697	0.112	0.009	0	Saliva_End	KIAA1462
PGM5	0	0.75071	0.121	0.01	0	Saliva_End	PGM5
ARHGAP31	0	0.747994	0.129	0.013	0	Saliva_End	ARHGAP31
MT.CO31	0	0.740988	1	0.977	0	Saliva_End	MT.CO3
NPR1	0	0.721787	0.115	0.01	0	Saliva_End	NPR1
SLC14A1	0	0.680288	0.054	0.002	0	Saliva_End	SLC14A1
SHANK3	0	0.679161	0.102	0.007	0	Saliva_End	SHANK3
MT.ND41	0	0.623504	0.999	0.953	0	Saliva_End	MT.ND4
MT.CO21	0	0.547156	0.999	0.959	0	Saliva_End	MT.CO2
HLA.DRA	0	0.533188	0.724	0.243	0	Saliva_End	HLA.DRA
CD74	0	0.473854	0.814	0.354	0	Saliva_End	CD74
B2M	1E-307	0.754984	0.998	0.928	1.6E-303	Saliva_End	B2M
ADM5	1.3E-298	0.823957	0.128	0.014	2.1E-294	Saliva_End	ADM5
SNCG	6.3E-291	1.314261	0.38	0.113	1E-286	Saliva_End	SNCG
PODXL	6.2E-288	1.592948	0.23	0.047	9.9E-284	Saliva_End	PODXL
CAV2	1.3E-287	1.525634	0.373	0.115	2E-283	Saliva_End	CAV2
SLFN5	8.4E-287	1.313092	0.282	0.067	1.3E-282	Saliva_End	SLFN5
TAGLN2	1.1E-285	1.447298	0.699	0.403	1.7E-281	Saliva_End	TAGLN2
HLA.C	8.3E-285	1.008416	0.915	0.682	1.3E-280	Saliva_End	HLA.C
EPHA4	3.1E-284	0.793668	0.119	0.013	4.9E-280	Saliva_End	EPHA4
PTPRM	1.9E-280	0.96957	0.165	0.025	3E-276	Saliva_End	PTPRM
STOM	4E-277	1.626245	0.42	0.148	6.3E-273	Saliva_End	STOM
RAPGEF3	2.4E-272	1.072303	0.18	0.031	3.8E-268	Saliva_End	RAPGEF3
VAMP5	9E-268	1.515382	0.443	0.164	1.4E-263	Saliva_End	VAMP5
MX1	4.7E-265	1.348164	0.247	0.056	7.6E-261	Saliva_End	MX1
THSD7A	2.6E-264	0.778058	0.123	0.015	4.2E-260	Saliva_End	THSD7A
MCAM	1.9E-263	0.84027	0.169	0.027	3E-259	Saliva_End	MCAM
ITGA6	3.4E-263	1.59687	0.382	0.131	5.5E-259	Saliva_End	ITGA6
TCF15	8.8E-263	0.67731	0.053	0.003	1.4E-258	Saliva_End	TCF15
CASKIN2	6.4E-262	0.816514	0.125	0.016	1E-257	Saliva_End	CASKIN2
ETS2	9.5E-260	1.523407	0.419	0.152	1.5E-255	Saliva_End	ETS2
ADIRF	3.5E-259	0.946265	0.79	0.429	5.5E-255	Saliva_End	ADIRF
CARD10	1.1E-257	0.690442	0.109	0.012	1.8E-253	Saliva_End	CARD10
EXOC3L2	1.7E-253	0.517178	0.055	0.003	2.7E-249	Saliva_End	EXOC3L2
ANKRD29	4.9E-251	0.950956	0.162	0.027	7.8E-247	Saliva_End	ANKRD29
HHEX	2.2E-249	0.910512	0.168	0.028	3.5E-245	Saliva_End	HHEX
SLC9A3R2	6.7E-247	1.906391	0.416	0.162	1.1E-242	Saliva_End	SLC9A3R2
CD320	2.5E-246	1.538654	0.312	0.092	3.9E-242	Saliva_End	CD320
HLA.A	1.2E-244	0.804393	0.913	0.664	2E-240	Saliva_End	HLA.A
MT.ND21	4.6E-244	0.554682	0.98	0.906	7.3E-240	Saliva_End	MT.ND2
HLA.B	9.9E-244	0.732391	0.932	0.662	1.6E-239	Saliva_End	HLA.B

SCARF1	3.1E-240	0.673415	0.098	0.01	4.9E-236	Saliva_End	SCARF1
SOX7	1E-238	0.825028	0.133	0.019	1.6E-234	Saliva_End	SOX7
MYH9	1.2E-234	1.525725	0.437	0.182	2E-230	Saliva_End	MYH9
ARAP3	9.3E-233	0.473354	0.064	0.005	1.5E-228	Saliva_End	ARAP3
PLAT	9.5E-233	0.464408	0.351	0.102	1.5E-228	Saliva_End	PLAT
TSPAN18	7.5E-232	0.769727	0.115	0.015	1.2E-227	Saliva_End	TSPAN18
ECE1	8.3E-231	1.174575	0.24	0.059	1.3E-226	Saliva_End	ECE1
SH3BP5	1.9E-229	1.3194	0.34	0.111	3E-225	Saliva_End	SH3BP5
SYNPO	5.3E-223	0.748285	0.11	0.014	8.5E-219	Saliva_End	SYNPO
EHD2	1.6E-220	0.946103	0.198	0.043	2.5E-216	Saliva_End	EHD2
TPT1	7.8E-220	0.604613	0.983	0.957	1.2E-215	Saliva_End	TPT1
SHROOM4	2.4E-219	0.609933	0.083	0.008	3.8E-215	Saliva_End	SHROOM4
IFITM2	4.2E-219	1.045949	0.613	0.299	6.7E-215	Saliva_End	IFITM2
SCN1B	3.8E-218	0.556452	0.098	0.012	6.1E-214	Saliva_End	SCN1B
BCL6B	5.8E-216	0.357947	0.059	0.004	9.2E-212	Saliva_End	BCL6B
ITGA5	8.5E-216	0.96926	0.182	0.037	1.4E-211	Saliva_End	ITGA5
DLL4	1.5E-214	0.541863	0.067	0.006	2.4E-210	Saliva_End	DLL4
RPS17	2.3E-213	0.919896	0.508	0.217	3.7E-209	Saliva_End	RPS17
FAM167B	4.2E-213	0.55869	0.071	0.006	6.6E-209	Saliva_End	FAM167B
LHX6	4.6E-211	0.693905	0.124	0.019	7.3E-207	Saliva_End	LHX6
CYSLTR1	1.3E-209	0.674798	0.112	0.015	2.2E-205	Saliva_End	CYSLTR1
TMEM204	5.9E-208	1.088665	0.182	0.038	9.4E-204	Saliva_End	TMEM204
DPYSL3	6.7E-208	1.025357	0.217	0.052	1.1E-203	Saliva_End	DPYSL3
FRY	1.3E-205	0.784629	0.122	0.019	2.1E-201	Saliva_End	FRY
JAG2	4.1E-205	0.741458	0.093	0.011	6.6E-201	Saliva_End	JAG2
USHBP1	1.1E-198	0.38959	0.054	0.004	1.7E-194	Saliva_End	USHBP1
RPL30	4.4E-198	0.704338	0.947	0.843	7.1E-194	Saliva_End	RPL30
LFNG	2.4E-195	0.818337	0.143	0.026	3.9E-191	Saliva_End	LFNG
MCTP1	3.5E-194	0.853913	0.162	0.032	5.6E-190	Saliva_End	MCTP1
SRP14	3.5E-193	1.190153	0.803	0.7	5.5E-189	Saliva_End	SRP14
TACC1	1.8E-191	1.407926	0.39	0.163	2.9E-187	Saliva_End	TACC1
TINAGL1	6.4E-190	1.218623	0.263	0.079	1E-185	Saliva_End	TINAGL1
APOL3	6.2E-188	0.822801	0.15	0.029	9.8E-184	Saliva_End	APOL3
ENDOD1	1.5E-186	0.834474	0.155	0.031	2.4E-182	Saliva_End	ENDOD1
ZNF385D	4.3E-186	1.003039	0.2	0.049	6.9E-182	Saliva_End	ZNF385D
BTNL9	1.6E-185	0.857193	0.073	0.007	2.6E-181	Saliva_End	BTNL9
NHSL2	4.5E-185	0.753185	0.132	0.023	7.2E-181	Saliva_End	NHSL2
PCDH12	2.4E-181	0.338303	0.038	0.002	3.8E-177	Saliva_End	PCDH12
S100A10	9.9E-180	0.791928	0.87	0.672	1.6E-175	Saliva_End	S100A10
CALM1	7.4E-179	1.049673	0.775	0.649	1.2E-174	Saliva_End	CALM1
ENPP2	1.5E-178	0.997451	0.148	0.03	2.4E-174	Saliva_End	ENPP2
ITGB4	3.2E-178	1.265802	0.261	0.084	5E-174	Saliva_End	ITGB4
NFIB	3.9E-178	1.320126	0.417	0.187	6.2E-174	Saliva_End	NFIB
RUNX1T1	1.6E-177	0.883503	0.17	0.038	2.5E-173	Saliva_End	RUNX1T1
CCDC68	1.4E-175	0.742724	0.112	0.018	2.2E-171	Saliva_End	CCDC68
TSPAN2	1.6E-175	0.879425	0.131	0.024	2.6E-171	Saliva_End	TSPAN2
IFI16	2E-175	1.365652	0.421	0.195	3.1E-171	Saliva_End	IFI16

ENTPD1	9.2E-175	0.91642	0.184	0.044	1.5E-170	Saliva_End	ENTPD1
FAM198B	1.2E-174	0.832149	0.155	0.033	1.9E-170	Saliva_End	FAM198B
ADAMTS1	2.1E-173	1.261632	0.215	0.058	3.4E-169	Saliva_End	ADAMTS1
TMEM255I	5.5E-173	0.688857	0.114	0.019	8.7E-169	Saliva_End	TMEM255B
NDRG1	7.1E-173	1.26817	0.351	0.14	1.1E-168	Saliva_End	NDRG1
MGLL1	1.3E-171	1.491928	0.386	0.173	2.1E-167	Saliva_End	MGLL
EPHB4	2.8E-169	0.808486	0.144	0.029	4.4E-165	Saliva_End	EPHB4
RAB3C	1.3E-168	0.399266	0.061	0.006	2E-164	Saliva_End	RAB3C
MT.CO11	9.4E-161	0.60319	0.995	0.984	1.5E-156	Saliva_End	MT.CO1
PTMS	3.6E-160	1.286599	0.464	0.24	5.7E-156	Saliva_End	PTMS
PRCP	1.4E-156	1.349845	0.307	0.122	2.3E-152	Saliva_End	PRCP
ICAM2	3.7E-156	1.057179	0.224	0.065	5.9E-152	Saliva_End	ICAM2
SOCS3	2.3E-154	1.461304	0.372	0.161	3.7E-150	Saliva_End	SOCS3
NRP2	1.1E-153	0.921866	0.136	0.029	1.8E-149	Saliva_End	NRP2
FLI1	7.9E-149	0.755846	0.16	0.038	1.3E-144	Saliva_End	FLI1
SNTB2	4.7E-147	1.04177	0.236	0.077	7.6E-143	Saliva_End	SNTB2
THSD1	9.1E-147	0.516851	0.081	0.011	1.5E-142	Saliva_End	THSD1
BST2	9.3E-146	1.298748	0.389	0.178	1.5E-141	Saliva_End	BST2
CEP68	6.2E-144	0.803033	0.161	0.04	9.9E-140	Saliva_End	CEP68
SEMA3G	9.2E-143	0.495586	0.045	0.004	1.5E-138	Saliva_End	SEMA3G
BRI3	1.5E-141	1.164387	0.532	0.343	2.3E-137	Saliva_End	BRI3
EMP2	1.7E-141	1.235958	0.388	0.187	2.7E-137	Saliva_End	EMP2
SEMA6A	4.2E-141	0.716399	0.127	0.027	6.7E-137	Saliva_End	SEMA6A
NOTCH4	5.4E-140	0.896136	0.102	0.018	8.6E-136	Saliva_End	NOTCH4
FILIP1L	1E-139	1.090717	0.241	0.082	1.6E-135	Saliva_End	FILIP1L
PCDH17	1.3E-139	0.686486	0.095	0.016	2.1E-135	Saliva_End	PCDH17
PIK3R3	7.3E-139	1.024796	0.155	0.039	1.2E-134	Saliva_End	PIK3R3
GUK1	2.8E-138	1.019854	0.665	0.556	4.4E-134	Saliva_End	GUK1
TM4SF1	9.6E-138	0.632016	0.566	0.31	1.5E-133	Saliva_End	TM4SF1
GALNT18	8.4E-136	0.534451	0.083	0.013	1.3E-131	Saliva_End	GALNT18
ITGA1	5.8E-135	0.964447	0.111	0.022	9.2E-131	Saliva_End	ITGA1
AHNAK	2.5E-134	1.081714	0.57	0.382	4.1E-130	Saliva_End	AHNAK
ITGA9	5.3E-134	0.534154	0.074	0.011	8.5E-130	Saliva_End	ITGA9
CXCL12	6.1E-134	1.369927	0.284	0.107	9.7E-130	Saliva_End	CXCL12
DAAM1	3.4E-133	1.3829	0.348	0.166	5.4E-129	Saliva_End	DAAM1
DPYSL2	3.6E-132	0.997342	0.262	0.096	5.7E-128	Saliva_End	DPYSL2
EFNB2	4.4E-132	1.084753	0.173	0.049	7E-128	Saliva_End	EFNB2
GPR146	1.8E-130	0.673855	0.1	0.019	2.9E-126	Saliva_End	GPR146
RHOC	3E-130	1.267722	0.416	0.221	4.7E-126	Saliva_End	RHOC
RPS24	3.5E-130	0.583652	0.925	0.838	5.6E-126	Saliva_End	RPS24
PARP14	6.7E-130	1.098346	0.226	0.077	1.1E-125	Saliva_End	PARP14
STAT3	1.9E-125	1.20669	0.373	0.187	3E-121	Saliva_End	STAT3
ABI3	2.5E-125	0.59523	0.132	0.03	4E-121	Saliva_End	ABI3
GFOD1	4.4E-125	0.661712	0.114	0.024	7E-121	Saliva_End	GFOD1
IFIT2	1.5E-124	0.753617	0.108	0.022	2.4E-120	Saliva_End	IFIT2
CCDC85B	5.3E-123	1.112555	0.468	0.269	8.4E-119	Saliva_End	CCDC85B
ZNF521	8.3E-123	0.611414	0.106	0.021	1.3E-118	Saliva_End	ZNF521

HLA.DMA	1.6E-122	0.865359	0.326	0.136	2.6E-118	Saliva_End	HLA.DMA
CTSC	5.9E-122	1.06311	0.289	0.118	9.4E-118	Saliva_End	CTSC
PTMA	1.5E-121	0.507991	0.942	0.817	2.3E-117	Saliva_End	PTMA
EEF1A1	2.9E-121	0.493803	0.977	0.914	4.6E-117	Saliva_End	EEF1A1
CTSH	4E-121	1.37596	0.412	0.235	6.3E-117	Saliva_End	CTSH
TSPAN4	1.2E-120	1.045475	0.28	0.115	1.9E-116	Saliva_End	TSPAN4
PSMB9	1.3E-119	1.041948	0.357	0.166	2.1E-115	Saliva_End	PSMB9
ZFP36L2	3E-119	0.833028	0.603	0.389	4.8E-115	Saliva_End	ZFP36L2
MTUS1	1.7E-118	1.25283	0.324	0.155	2.7E-114	Saliva_End	MTUS1
AKR1C3	1.7E-118	0.81624	0.185	0.058	2.7E-114	Saliva_End	AKR1C3
HIF3A	3.1E-118	0.59639	0.086	0.015	4.9E-114	Saliva_End	HIF3A
IRF9	1.2E-117	0.672163	0.107	0.023	1.9E-113	Saliva_End	IRF9
CYTL1	1.8E-117	0.628103	0.097	0.019	2.8E-113	Saliva_End	CYTL1
TGFBR3	2.8E-117	0.990229	0.238	0.089	4.5E-113	Saliva_End	TGFBR3
SH2D3C	3.9E-117	0.531996	0.082	0.014	6.2E-113	Saliva_End	SH2D3C
PLK2	8.4E-117	1.109334	0.226	0.083	1.3E-112	Saliva_End	PLK2
HLA.F	8.9E-117	1.001411	0.286	0.117	1.4E-112	Saliva_End	HLA.F
SPARC	4.6E-116	0.698819	0.356	0.159	7.3E-112	Saliva_End	SPARC
RASL10A	7.2E-115	0.324259	0.043	0.004	1.2E-110	Saliva_End	RASL10A
NEURL1B	2.6E-114	0.658899	0.105	0.022	4.1E-110	Saliva_End	NEURL1B
BMPR2	8.5E-113	1.130589	0.249	0.101	1.4E-108	Saliva_End	BMPR2
PIK3C2B	2.3E-112	0.629134	0.094	0.019	3.6E-108	Saliva_End	PIK3C2B
SPHK1	1.9E-111	0.829054	0.188	0.062	3E-107	Saliva_End	SPHK1
RALGAPA2	2.4E-111	0.877768	0.172	0.054	3.8E-107	Saliva_End	RALGAPA2
CRIM1	1.7E-110	0.991491	0.212	0.076	2.8E-106	Saliva_End	CRIM1
RBP7	2.5E-110	1.292992	0.222	0.083	3.9E-106	Saliva_End	RBP7
SYT15	7.1E-109	0.356996	0.057	0.008	1.1E-104	Saliva_End	SYT15
TSPAN9	1.2E-108	0.699096	0.121	0.03	1.9E-104	Saliva_End	TSPAN9
LAP3	1.8E-108	1.262108	0.276	0.123	2.9E-104	Saliva_End	LAP3
IL1R1	2.3E-105	0.908289	0.203	0.073	3.7E-101	Saliva_End	IL1R1
MAPK11	3.9E-104	0.41816	0.061	0.009	6.3E-100	Saliva_End	MAPK11
CPLX1	5.5E-103	0.507279	0.079	0.015	8.7E-99	Saliva_End	CPLX1
RDX	2.3E-102	1.381345	0.342	0.184	3.66E-98	Saliva_End	RDX
EDNRB	2.6E-102	1.096455	0.103	0.024	4.14E-98	Saliva_End	EDNRB
DOCK4	3.6E-102	0.538331	0.095	0.02	5.82E-98	Saliva_End	DOCK4
CPXM2	1.2E-101	0.594062	0.123	0.032	1.85E-97	Saliva_End	CPXM2
VWA1	2.1E-101	1.129494	0.206	0.078	3.37E-97	Saliva_End	VWA1
CTNNAL1	2.1E-101	1.155661	0.22	0.087	3.43E-97	Saliva_End	CTNNAL1
CPNE2	2.5E-101	0.582208	0.106	0.025	4.06E-97	Saliva_End	CPNE2
TFPI	3.3E-101	1.028292	0.21	0.078	5.27E-97	Saliva_End	TFPI
FOXP1	4.1E-101	1.158424	0.381	0.216	6.58E-97	Saliva_End	FOXP1
CSRP2	7.4E-101	1.021429	0.209	0.079	1.19E-96	Saliva_End	CSRP2
ARPC1B	8.3E-101	0.763876	0.491	0.289	1.32E-96	Saliva_End	ARPC1B
MSN	9.6E-101	1.044432	0.317	0.154	1.53E-96	Saliva_End	MSN
LEPROT	1.3E-100	1.204564	0.413	0.254	2.14E-96	Saliva_End	LEPROT
ACTN1	1.9E-100	1.052334	0.301	0.145	2.99E-96	Saliva_End	ACTN1
SNHG7	2.2E-100	0.943443	0.401	0.22	3.45E-96	Saliva_End	SNHG7

APOLD1	2.4E-99	0.788861	0.117	0.029	3.78E-95	Saliva_End	APOLD1
FCGRT	9.45E-96	1.017935	0.443	0.275	1.51E-91	Saliva_End	FCGRT
TMEM173	3.01E-95	0.913095	0.253	0.108	4.79E-91	Saliva_End	TMEM173
IL6ST	4.24E-95	1.241411	0.373	0.22	6.77E-91	Saliva_End	IL6ST
PLCB1	5.47E-95	0.827423	0.132	0.038	8.72E-91	Saliva_End	PLCB1
NOS3	1.11E-94	0.456424	0.074	0.014	1.77E-90	Saliva_End	NOS3
XIST1	2.41E-94	1.391502	0.264	0.12	3.84E-90	Saliva_End	XIST
SEMA6B	2.76E-94	0.358461	0.052	0.007	4.4E-90	Saliva_End	SEMA6B
PLXDC2	1.02E-93	0.742068	0.141	0.042	1.62E-89	Saliva_End	PLXDC2
RAPGEF5	1.96E-93	0.76503	0.129	0.037	3.13E-89	Saliva_End	RAPGEF5
IRF1	4.85E-93	1.305829	0.373	0.213	7.74E-89	Saliva_End	IRF1
SLC8A1	4.17E-91	0.389166	0.073	0.014	6.65E-87	Saliva_End	SLC8A1
CD151	4.88E-91	1.014389	0.377	0.218	7.78E-87	Saliva_End	CD151
FOS	5.23E-91	1.108825	0.577	0.423	8.35E-87	Saliva_End	FOS
RPL7A	6.5E-91	0.445378	0.92	0.812	1.04E-86	Saliva_End	RPL7A
PDIA5	8.73E-91	0.570156	0.108	0.028	1.39E-86	Saliva_End	PDIA5
ATP2B1	1.08E-90	1.04063	0.298	0.148	1.72E-86	Saliva_End	ATP2B1
TSHZ2	1.67E-90	0.983494	0.246	0.108	2.67E-86	Saliva_End	TSHZ2
RGS16	4.43E-90	0.946817	0.218	0.086	7.07E-86	Saliva_End	RGS16
CRHBP	6.71E-90	0.391618	0.063	0.011	1.07E-85	Saliva_End	CRHBP
RPL22	4.22E-89	0.597929	0.798	0.692	6.73E-85	Saliva_End	RPL22
UTRN	1.56E-88	1.086918	0.251	0.113	2.48E-84	Saliva_End	UTRN
TSC22D1	3.05E-88	1.182383	0.424	0.265	4.87E-84	Saliva_End	TSC22D1
CD9	3.86E-88	0.989151	0.603	0.45	6.16E-84	Saliva_End	CD9
GATA2	8.91E-88	0.660537	0.097	0.023	1.42E-83	Saliva_End	GATA2
EHD4	1.16E-87	1.101665	0.214	0.089	1.86E-83	Saliva_End	EHD4
HLA.DQB1	3.66E-87	0.369562	0.292	0.128	5.84E-83	Saliva_End	HLA.DQB1
PSMB8	5.32E-87	0.991091	0.348	0.188	8.49E-83	Saliva_End	PSMB8
FAM13C	4.52E-86	0.579257	0.092	0.022	7.21E-82	Saliva_End	FAM13C
MGST3	9.1E-86	0.80523	0.503	0.332	1.45E-81	Saliva_End	MGST3
RPL24	1.74E-85	0.476522	0.872	0.801	2.77E-81	Saliva_End	RPL24
CPNE8	4.77E-85	0.671657	0.13	0.039	7.61E-81	Saliva_End	CPNE8
C10orf10	1.09E-84	1.044314	0.181	0.068	1.74E-80	Saliva_End	C10orf10
NACA	2.38E-84	0.502065	0.84	0.831	3.79E-80	Saliva_End	NACA
EDN1	2.6E-83	0.818598	0.125	0.037	4.15E-79	Saliva_End	EDN1
MTRNR2L1	2.97E-83	0.790331	0.229	0.096	4.73E-79	Saliva_End	MTRNR2L10
FKBP1A	4.04E-83	1.061531	0.472	0.335	6.45E-79	Saliva_End	FKBP1A
DDX5	7.87E-83	0.721214	0.694	0.6	1.25E-78	Saliva_End	DDX5
TUBA1B	9.35E-83	0.83991	0.517	0.351	1.49E-78	Saliva_End	TUBA1B
ARHGAP23	1.12E-81	0.557179	0.094	0.024	1.79E-77	Saliva_End	ARHGAP23
ATP2B4	5.73E-81	0.851953	0.195	0.078	9.15E-77	Saliva_End	ATP2B4
CLEC2B	8.47E-81	0.911	0.302	0.153	1.35E-76	Saliva_End	CLEC2B
C1orf115	3.23E-80	0.782829	0.137	0.045	5.15E-76	Saliva_End	C1orf115
FBLN2	5.09E-80	0.723798	0.219	0.089	8.13E-76	Saliva_End	FBLN2
MMP28	5.42E-80	0.374859	0.069	0.014	8.64E-76	Saliva_End	MMP28
N4BP2L2	1.67E-79	1.128403	0.425	0.291	2.66E-75	Saliva_End	N4BP2L2
RASGRF2	2.67E-79	0.380015	0.047	0.007	4.26E-75	Saliva_End	RASGRF2

CLIC2	8.59E-79	0.691772	0.138	0.045	1.37E-74	Saliva_End	CLIC2
FLNC	1.05E-78	0.436328	0.063	0.012	1.67E-74	Saliva_End	FLNC
RPS23	4.7E-78	0.386641	0.949	0.811	7.5E-74	Saliva_End	RPS23
GJA4	9.39E-78	0.823005	0.074	0.016	1.5E-73	Saliva_End	GJA4
NEDD4	1.17E-77	0.46363	0.071	0.015	1.86E-73	Saliva_End	NEDD4
FLNB	1.35E-77	0.925723	0.188	0.077	2.16E-73	Saliva_End	FLNB
RPL37	1.77E-77	0.439097	0.885	0.847	2.83E-73	Saliva_End	RPL37
POLR2J3	2.12E-77	0.920612	0.216	0.095	3.38E-73	Saliva_End	POLR2J3
RPL9	3.21E-77	0.329378	0.923	0.747	5.12E-73	Saliva_End	RPL9
RP11.473M	3.45E-77	0.318935	0.043	0.006	5.5E-73	Saliva_End	RP11.473M20.16
RSAD2	3.56E-76	0.632742	0.079	0.018	5.68E-72	Saliva_End	RSAD2
EBF1	4.6E-76	0.65956	0.165	0.06	7.34E-72	Saliva_End	EBF1
PDGFB	1.14E-75	0.627434	0.079	0.019	1.82E-71	Saliva_End	PDGFB
IFI6	3.08E-75	1.09359	0.293	0.152	4.9E-71	Saliva_End	IFI6
ST6GALNA	3.71E-75	0.282992	0.032	0.004	5.91E-71	Saliva_End	ST6GALNAC3
MGST2	4.18E-74	1.195223	0.336	0.207	6.67E-70	Saliva_End	MGST2
RN7SL832I	5.44E-74	0.28131	0.041	0.006	8.67E-70	Saliva_End	RN7SL832P
SAMHD1	2.28E-73	0.698703	0.174	0.067	3.64E-69	Saliva_End	SAMHD1
GAS6	2.48E-73	0.971597	0.211	0.093	3.96E-69	Saliva_End	GAS6
TMSB10	1.94E-72	0.30801	0.966	0.877	3.09E-68	Saliva_End	TMSB10
LRRFIP1	3.97E-72	0.941514	0.489	0.371	6.33E-68	Saliva_End	LRRFIP1
PCGF2	7.1E-72	0.665117	0.117	0.037	1.13E-67	Saliva_End	PCGF2
PPFIBP1	9.87E-72	1.215162	0.221	0.104	1.57E-67	Saliva_End	PPFIBP1
ASAP1	4E-71	0.818517	0.209	0.093	6.38E-67	Saliva_End	ASAP1
RPS6KA2	4.68E-71	0.728824	0.12	0.039	7.46E-67	Saliva_End	RPS6KA2
LAMA5	7.03E-71	0.617187	0.097	0.027	1.12E-66	Saliva_End	LAMA5
STARD9	7.97E-71	0.475189	0.087	0.022	1.27E-66	Saliva_End	STARD9
CCDC3	8.34E-71	0.532362	0.092	0.025	1.33E-66	Saliva_End	CCDC3
GRB10	1.35E-70	0.806288	0.126	0.042	2.15E-66	Saliva_End	GRB10
CLEC1A	5.26E-70	0.32119	0.049	0.008	8.39E-66	Saliva_End	CLEC1A
NASP	2.29E-69	0.857691	0.28	0.147	3.65E-65	Saliva_End	NASP
PLSCR4	2.29E-69	0.624839	0.133	0.046	3.65E-65	Saliva_End	PLSCR4
BTN3A2	6.76E-69	0.667041	0.156	0.058	1.08E-64	Saliva_End	BTN3A2
DNASE1L3	7.41E-69	0.456101	0.061	0.013	1.18E-64	Saliva_End	DNASE1L3
COL18A1	2.86E-68	0.781298	0.176	0.071	4.55E-64	Saliva_End	COL18A1
ITPR1	5.18E-68	0.510578	0.099	0.029	8.27E-64	Saliva_End	ITPR1
HLX	8.06E-68	0.503871	0.063	0.014	1.29E-63	Saliva_End	HLX
MT.ND4L1	2.26E-67	0.714739	0.543	0.415	3.6E-63	Saliva_End	MT.ND4L
IRAK3	2.73E-67	0.52655	0.09	0.025	4.36E-63	Saliva_End	IRAK3
RAB13	5.25E-67	1.108021	0.336	0.217	8.38E-63	Saliva_End	RAB13
DNM3	9.99E-67	0.322227	0.053	0.01	1.59E-62	Saliva_End	DNM3
WNK1	1.93E-66	1.036698	0.282	0.158	3.08E-62	Saliva_End	WNK1
DCHS1	2.93E-66	0.388208	0.057	0.012	4.68E-62	Saliva_End	DCHS1
PARVB	3.02E-66	0.606116	0.124	0.042	4.82E-62	Saliva_End	PARVB
TPM3	3.31E-66	0.910125	0.45	0.33	5.28E-62	Saliva_End	TPM3
ZEB1	1.22E-65	0.713589	0.151	0.058	1.95E-61	Saliva_End	ZEB1
KLHL3	2.14E-65	0.394085	0.066	0.015	3.41E-61	Saliva_End	KLHL3

NKTR	3.2E-65	1.04344	0.301	0.174	5.11E-61	Saliva_End	NKTR
YWHAB	3.81E-65	0.887243	0.507	0.411	6.07E-61	Saliva_End	YWHAB
MSRB3	4.78E-65	0.513862	0.107	0.034	7.62E-61	Saliva_End	MSRB3
AFAP1L1	2.24E-64	0.456405	0.074	0.018	3.57E-60	Saliva_End	AFAP1L1
IFI44	3.44E-64	0.765928	0.13	0.046	5.48E-60	Saliva_End	IFI44
MTRNR2L8	6.76E-64	0.681023	0.436	0.284	1.08E-59	Saliva_End	MTRNR2L8
NOTCH1	1.12E-63	0.591419	0.11	0.036	1.79E-59	Saliva_End	NOTCH1
IER2	1.35E-63	0.871682	0.631	0.545	2.16E-59	Saliva_End	IER2
PLA2G16	1.69E-63	0.844781	0.241	0.123	2.69E-59	Saliva_End	PLA2G16
LIMS1	4.2E-63	1.031648	0.288	0.166	6.7E-59	Saliva_End	LIMS1
MAP3K11	5.63E-63	0.827314	0.195	0.089	8.99E-59	Saliva_End	MAP3K11
FAM213A	8.6E-63	0.992013	0.238	0.126	1.37E-58	Saliva_End	FAM213A
TMOD3	9.21E-63	1.005081	0.364	0.24	1.47E-58	Saliva_End	TMOD3
HSP90AB1	1.11E-62	0.663245	0.654	0.569	1.77E-58	Saliva_End	HSP90AB1
TNFRSF1A	1.55E-62	0.917624	0.281	0.158	2.48E-58	Saliva_End	TNFRSF1A
MECOM	1.71E-62	0.684824	0.097	0.03	2.73E-58	Saliva_End	MECOM
YBX3	4.33E-62	0.723967	0.585	0.494	6.91E-58	Saliva_End	YBX3
AKT3	4.83E-62	0.609416	0.113	0.038	7.7E-58	Saliva_End	AKT3
TLR4	4.99E-62	0.339653	0.062	0.014	7.96E-58	Saliva_End	TLR4
CLIC5	5.5E-62	0.553318	0.062	0.014	8.77E-58	Saliva_End	CLIC5
ETS1	5.65E-62	0.72833	0.177	0.075	9E-58	Saliva_End	ETS1
ABHD17A	8.8E-62	0.883982	0.228	0.114	1.4E-57	Saliva_End	ABHD17A
RPL5	1.26E-61	0.295412	0.882	0.725	2.02E-57	Saliva_End	RPL5
GNB4	1E-60	0.572118	0.113	0.038	1.6E-56	Saliva_End	GNB4
SYNE1	2.12E-60	0.742387	0.144	0.055	3.39E-56	Saliva_End	SYNE1
MPDZ	4.05E-60	0.563924	0.095	0.029	6.46E-56	Saliva_End	MPDZ
ST6GAL1	4.17E-60	0.548871	0.112	0.038	6.65E-56	Saliva_End	ST6GAL1
CA8	1.31E-59	0.273162	0.044	0.008	2.08E-55	Saliva_End	CA8
CD109	2.84E-59	0.617072	0.123	0.044	4.53E-55	Saliva_End	CD109
DLL1	5.45E-59	0.387094	0.062	0.015	8.7E-55	Saliva_End	DLL1
HMGB1	6.35E-59	0.676354	0.64	0.575	1.01E-54	Saliva_End	HMGB1
ATP8A1	1.77E-58	0.365236	0.063	0.015	2.82E-54	Saliva_End	ATP8A1
MACF1	6.41E-58	1.015149	0.28	0.166	1.02E-53	Saliva_End	MACF1
DOCK6	6.79E-58	0.530494	0.082	0.023	1.08E-53	Saliva_End	DOCK6
MTRNR2L1	7.21E-58	0.906565	0.242	0.124	1.15E-53	Saliva_End	MTRNR2L1
ADARB1	2.52E-57	0.451527	0.078	0.022	4.01E-53	Saliva_End	ADARB1
CSF2RB	3.6E-57	0.548275	0.109	0.037	5.74E-53	Saliva_End	CSF2RB
EPS8	5.5E-57	0.754519	0.173	0.078	8.76E-53	Saliva_End	EPS8
ATP11C	6.16E-57	0.608709	0.115	0.041	9.82E-53	Saliva_End	ATP11C
PMP22	2.32E-56	0.614186	0.263	0.136	3.7E-52	Saliva_End	PMP22
RAPGEF4	2.99E-56	0.310801	0.044	0.008	4.77E-52	Saliva_End	RAPGEF4
UACA	3.38E-56	0.971607	0.214	0.11	5.39E-52	Saliva_End	UACA
PKN3	2.89E-55	0.390732	0.055	0.013	4.62E-51	Saliva_End	PKN3
TGM2	8.72E-55	0.614095	0.087	0.027	1.39E-50	Saliva_End	TGM2
TRIM47	1.14E-54	0.626532	0.142	0.057	1.83E-50	Saliva_End	TRIM47
ARGLU1	1.38E-54	0.992472	0.41	0.307	2.21E-50	Saliva_End	ARGLU1
NSUN6	2.55E-54	0.816284	0.129	0.051	4.06E-50	Saliva_End	NSUN6

LAMB2	3.36E-54	0.769012	0.178	0.082	5.36E-50	Saliva_End	LAMB2
PIK3C2A	5.84E-54	0.840492	0.171	0.079	9.31E-50	Saliva_End	PIK3C2A
ORAI1	4.47E-53	0.748674	0.184	0.087	7.14E-49	Saliva_End	ORAI1
RPS27A	5.28E-53	0.307022	0.943	0.848	8.42E-49	Saliva_End	RPS27A
PROS1	8.07E-53	0.539795	0.116	0.042	1.29E-48	Saliva_End	PROS1
DUSP23	2.27E-52	0.963627	0.258	0.151	3.62E-48	Saliva_End	DUSP23
FAM43A	2.37E-52	0.534319	0.102	0.035	3.79E-48	Saliva_End	FAM43A
PLXNA2	2.96E-52	0.541645	0.073	0.02	4.72E-48	Saliva_End	PLXNA2
SRSF7	3.65E-52	0.898352	0.445	0.341	5.82E-48	Saliva_End	SRSF7
ZNF467	4.18E-52	0.419951	0.081	0.024	6.66E-48	Saliva_End	ZNF467
PTPN14	1.09E-51	0.746461	0.136	0.056	1.75E-47	Saliva_End	PTPN14
IPO11	1.15E-51	0.489553	0.093	0.03	1.84E-47	Saliva_End	IPO11
FMNL3	3.58E-51	0.334399	0.05	0.011	5.71E-47	Saliva_End	FMNL3
DDX60	7.49E-51	0.486503	0.077	0.023	1.19E-46	Saliva_End	DDX60
MYRIP	8.08E-51	0.323367	0.056	0.014	1.29E-46	Saliva_End	MYRIP
TAPBP	1.13E-50	0.835846	0.274	0.162	1.8E-46	Saliva_End	TAPBP
SMIM10	1.18E-50	0.409899	0.07	0.02	1.88E-46	Saliva_End	SMIM10
PPP1R16B	1.44E-50	0.268934	0.037	0.006	2.29E-46	Saliva_End	PPP1R16B
TNFRSF1B	1.83E-50	0.441593	0.123	0.047	2.91E-46	Saliva_End	TNFRSF1B
CRYBG3	5.92E-50	0.740371	0.142	0.061	9.45E-46	Saliva_End	CRYBG3
APOL1	2.01E-49	0.470278	0.093	0.031	3.21E-45	Saliva_End	APOL1
ITPR2	2.95E-49	0.812482	0.167	0.079	4.7E-45	Saliva_End	ITPR2
RPGR	3.75E-49	0.496157	0.096	0.033	5.98E-45	Saliva_End	RPGR
DDX58	4.53E-49	0.482772	0.074	0.022	7.23E-45	Saliva_End	DDX58
PDLIM3	1.39E-48	0.675566	0.135	0.056	2.22E-44	Saliva_End	PDLIM3
LY6E	1.48E-48	0.741968	0.453	0.329	2.36E-44	Saliva_End	LY6E
TNFRSF10I	1.53E-48	0.40988	0.062	0.017	2.45E-44	Saliva_End	TNFRSF10D
RPS8	8.37E-48	0.269549	0.937	0.848	1.34E-43	Saliva_End	RPS8
MBNL1	1.23E-47	0.866892	0.278	0.169	1.96E-43	Saliva_End	MBNL1
ABCG2	1.28E-47	0.250873	0.035	0.006	2.04E-43	Saliva_End	ABCG2
LRRC8C	3.45E-47	0.544162	0.093	0.032	5.51E-43	Saliva_End	LRRC8C
HERC1	4.43E-47	0.737621	0.145	0.065	7.06E-43	Saliva_End	HERC1
PEAR1	4.64E-47	0.303207	0.045	0.01	7.39E-43	Saliva_End	PEAR1
EIF2AK2	7.29E-47	0.8774	0.185	0.094	1.16E-42	Saliva_End	EIF2AK2
RRAS	1.1E-46	0.892186	0.19	0.097	1.75E-42	Saliva_End	RRAS
CHD9	1.31E-46	0.910838	0.268	0.165	2.09E-42	Saliva_End	CHD9
SP100	1.74E-46	0.818781	0.241	0.138	2.78E-42	Saliva_End	SP100
ARL2	2.27E-46	0.794469	0.318	0.209	3.63E-42	Saliva_End	ARL2
MCF2L	2.44E-46	0.5529	0.083	0.028	3.89E-42	Saliva_End	MCF2L
AMIGO2	3.18E-46	0.27599	0.049	0.011	5.08E-42	Saliva_End	AMIGO2
RPS13	5.28E-46	0.26928	0.909	0.816	8.42E-42	Saliva_End	RPS13
STAB1	5.42E-46	0.297862	0.058	0.015	8.64E-42	Saliva_End	STAB1
FKBP9	5.54E-46	0.714027	0.162	0.077	8.84E-42	Saliva_End	FKBP9
FILIP1	7.9E-46	0.530186	0.071	0.021	1.26E-41	Saliva_End	FILIP1
DDX17	1.88E-45	0.915233	0.432	0.353	3E-41	Saliva_End	DDX17
ARHGAP26	2.02E-45	0.64036	0.129	0.055	3.22E-41	Saliva_End	ARHGAP26
GBP1	4.1E-45	0.686585	0.139	0.061	6.54E-41	Saliva_End	GBP1

ITGA10	5.86E-45	0.343076	0.052	0.013	9.35E-41	Saliva_End	ITGA10
EPB41L3	1.26E-44	0.580929	0.132	0.057	2.01E-40	Saliva_End	EPB41L3
PLCG2	1.89E-44	0.479267	0.083	0.028	3.01E-40	Saliva_End	PLCG2
PLEC	1.99E-43	0.878236	0.253	0.154	3.17E-39	Saliva_End	PLEC
NTS	3.04E-43	0.515279	0.027	0.004	4.85E-39	Saliva_End	NTS
DAB2	4.07E-43	0.65408	0.184	0.093	6.5E-39	Saliva_End	DAB2
ARL4A	5.09E-43	0.598496	0.342	0.23	8.11E-39	Saliva_End	ARL4A
INTS6	1.02E-42	0.9733	0.23	0.135	1.63E-38	Saliva_End	INTS6
ATOH8	1.35E-42	0.481332	0.056	0.015	2.16E-38	Saliva_End	ATOH8
LPAR6	1.42E-42	0.835878	0.237	0.14	2.26E-38	Saliva_End	LPAR6
TMEM140	1.63E-42	0.396383	0.067	0.02	2.6E-38	Saliva_End	TMEM140
RP11.109G	1.94E-42	0.272699	0.041	0.009	3.1E-38	Saliva_End	RP11.109G23.3
GIPC2	2.11E-42	0.445759	0.077	0.025	3.36E-38	Saliva_End	GIPC2
HIP1	2.54E-42	0.422725	0.132	0.056	4.05E-38	Saliva_End	HIP1
RPL29	2.96E-42	0.253993	0.909	0.828	4.72E-38	Saliva_End	RPL29
SOCS2	3.04E-42	0.672468	0.131	0.058	4.85E-38	Saliva_End	SOCS2
OLFML2A	4.93E-42	0.364691	0.072	0.023	7.86E-38	Saliva_End	OLFML2A
NFE2L1	8.94E-42	0.765632	0.173	0.09	1.43E-37	Saliva_End	NFE2L1
FOXO1	2.38E-41	0.565266	0.113	0.046	3.8E-37	Saliva_End	FOXO1
NCOA7	2.78E-41	0.755459	0.196	0.106	4.43E-37	Saliva_End	NCOA7
ACACB	2.97E-41	0.431814	0.077	0.026	4.74E-37	Saliva_End	ACACB
KLF13	1.04E-40	0.697209	0.163	0.081	1.66E-36	Saliva_End	KLF13
CD36	1.23E-40	0.485252	0.058	0.017	1.96E-36	Saliva_End	CD36
PPM1F	1.94E-40	0.464502	0.084	0.03	3.09E-36	Saliva_End	PPM1F
SYNE3	2.67E-40	0.326052	0.061	0.018	4.25E-36	Saliva_End	SYNE3
FAM65A	2.68E-40	0.621998	0.113	0.047	4.28E-36	Saliva_End	FAM65A
VCAM1	3.81E-40	0.495092	0.088	0.032	6.08E-36	Saliva_End	VCAM1
ASB9	5.61E-40	0.352176	0.061	0.018	8.94E-36	Saliva_End	ASB9
DYNLL1	5.96E-40	0.471888	0.63	0.56	9.51E-36	Saliva_End	DYNLL1
MXRA7	6.07E-40	0.683245	0.164	0.082	9.68E-36	Saliva_End	MXRA7
MATN2	7.24E-40	0.544296	0.103	0.041	1.15E-35	Saliva_End	MATN2
TNFRSF10/	8.14E-40	0.30689	0.049	0.013	1.3E-35	Saliva_End	TNFRSF10A
FZD6	9.94E-40	0.512751	0.095	0.037	1.59E-35	Saliva_End	FZD6
WASF2	1.13E-39	0.879672	0.396	0.322	1.79E-35	Saliva_End	WASF2
TAP2	1.18E-39	0.415079	0.066	0.021	1.88E-35	Saliva_End	TAP2
RPS28	1.3E-39	0.256358	0.934	0.88	2.08E-35	Saliva_End	RPS28
CLIC4	1.62E-39	0.836964	0.213	0.124	2.58E-35	Saliva_End	CLIC4
KIAA1671	1.83E-39	0.629779	0.109	0.046	2.92E-35	Saliva_End	KIAA1671
CALHM2	3.72E-39	0.409174	0.09	0.033	5.93E-35	Saliva_End	CALHM2
SCARB1	4.16E-39	0.580649	0.101	0.041	6.63E-35	Saliva_End	SCARB1
BOD1L1	4.28E-39	0.860318	0.215	0.126	6.82E-35	Saliva_End	BOD1L1
RBM43	6.77E-39	0.405547	0.075	0.026	1.08E-34	Saliva_End	RBM43
TNXB	7.89E-39	0.320927	0.154	0.072	1.26E-34	Saliva_End	TNXB
MYOF	8.15E-39	0.561474	0.127	0.057	1.3E-34	Saliva_End	MYOF
PRDX1	1.27E-38	0.584469	0.529	0.451	2.02E-34	Saliva_End	PRDX1
MYO1C	1.28E-38	0.920791	0.233	0.144	2.05E-34	Saliva_End	MYO1C
PLCB4	1.65E-38	0.647435	0.122	0.055	2.63E-34	Saliva_End	PLCB4

ODF3B	2.05E-38	0.679095	0.169	0.087	3.28E-34	Saliva_End	ODF3B
JAG1	3.25E-38	0.706084	0.115	0.049	5.18E-34	Saliva_End	JAG1
HECW2	8.38E-38	0.367686	0.037	0.008	1.34E-33	Saliva_End	HECW2
NUCKS1	9.17E-38	0.761256	0.374	0.282	1.46E-33	Saliva_End	NUCKS1
CSGALNAC	1.13E-37	0.508481	0.103	0.043	1.81E-33	Saliva_End	CSGALNACT1
RP11.326I1	1.19E-37	0.312023	0.049	0.013	1.89E-33	Saliva_End	RP11.326I11.3
OAS3	1.82E-37	0.560642	0.086	0.033	2.9E-33	Saliva_End	OAS3
GPRC5B	2.64E-37	0.656287	0.095	0.038	4.21E-33	Saliva_End	GPRC5B
MMP15	8.42E-37	0.363542	0.053	0.015	1.34E-32	Saliva_End	MMP15
TPST2	2.27E-36	0.526629	0.143	0.069	3.61E-32	Saliva_End	TPST2
RNF125	2.38E-36	0.418036	0.091	0.036	3.8E-32	Saliva_End	RNF125
ELMO1	3.42E-36	0.411018	0.085	0.032	5.45E-32	Saliva_End	ELMO1
FLOT1	3.72E-36	0.801354	0.228	0.141	5.93E-32	Saliva_End	FLOT1
PIM3	4.65E-36	0.824474	0.371	0.29	7.42E-32	Saliva_End	PIM3
JUN	7.26E-36	0.676169	0.56	0.487	1.16E-31	Saliva_End	JUN
PDGFD	1.15E-35	0.491627	0.066	0.022	1.83E-31	Saliva_End	PDGFD
PDE7B	1.36E-35	0.308512	0.039	0.009	2.16E-31	Saliva_End	PDE7B
HYAL1	3.09E-35	0.304191	0.037	0.008	4.93E-31	Saliva_End	HYAL1
CD63	3.55E-35	0.254534	0.747	0.623	5.66E-31	Saliva_End	CD63
F2R	5.91E-35	0.515046	0.114	0.051	9.42E-31	Saliva_End	F2R
MLKL	5.97E-35	0.367732	0.064	0.021	9.52E-31	Saliva_End	MLKL
TMEM150	6.1E-35	0.418439	0.059	0.019	9.73E-31	Saliva_End	TMEM150C
JUND	7.49E-35	0.638041	0.521	0.463	1.2E-30	Saliva_End	JUND
ELOVL7	1.12E-34	0.391285	0.063	0.021	1.79E-30	Saliva_End	ELOVL7
PRMT1	1.27E-34	0.837388	0.28	0.191	2.03E-30	Saliva_End	PRMT1
TRIM56	1.44E-34	0.667241	0.149	0.077	2.3E-30	Saliva_End	TRIM56
CYB5R3	1.54E-34	0.818478	0.307	0.22	2.45E-30	Saliva_End	CYB5R3
AHR	1.94E-34	0.669502	0.19	0.107	3.1E-30	Saliva_End	AHR
CSF1	2.94E-34	0.389741	0.084	0.032	4.69E-30	Saliva_End	CSF1
ZDHHC17	3.16E-34	0.4927	0.087	0.034	5.04E-30	Saliva_End	ZDHHC17
KTN1	6.66E-34	0.763864	0.452	0.397	1.06E-29	Saliva_End	KTN1
GAS2L1	8.49E-34	0.536576	0.111	0.051	1.35E-29	Saliva_End	GAS2L1
EFNA1	1.03E-33	0.890311	0.162	0.089	1.63E-29	Saliva_End	EFNA1
SMAD1	1.08E-33	0.61127	0.118	0.055	1.72E-29	Saliva_End	SMAD1
KLF9	1.14E-33	0.733359	0.185	0.105	1.82E-29	Saliva_End	KLF9
ABHD6	1.19E-33	0.276597	0.045	0.012	1.89E-29	Saliva_End	ABHD6
MT.ND61	1.28E-33	0.731009	0.19	0.11	2.04E-29	Saliva_End	MT.ND6
CX3CL1	1.44E-33	0.566893	0.066	0.023	2.29E-29	Saliva_End	CX3CL1
SLC2A3	5.01E-33	0.500782	0.224	0.128	7.98E-29	Saliva_End	SLC2A3
MYL12A	5.8E-33	0.338746	0.704	0.658	9.26E-29	Saliva_End	MYL12A
SLC29A1	6.39E-33	0.501235	0.105	0.046	1.02E-28	Saliva_End	SLC29A1
ALDH1A2	7.5E-33	0.395106	0.067	0.024	1.2E-28	Saliva_End	ALDH1A2
SMAD5	7.66E-33	0.732033	0.167	0.093	1.22E-28	Saliva_End	SMAD5
ABCB1	7.84E-33	0.36037	0.054	0.017	1.25E-28	Saliva_End	ABCB1
FGF2	1.65E-32	0.346006	0.058	0.019	2.63E-28	Saliva_End	FGF2
EVA1B	2.26E-32	0.623904	0.138	0.07	3.6E-28	Saliva_End	EVA1B
FUS	2.9E-32	0.824789	0.355	0.276	4.62E-28	Saliva_End	FUS

SYT1	3.52E-32	0.296898	0.034	0.008	5.61E-28	Saliva_End	SYT1
PPP3CA	3.71E-32	0.727512	0.238	0.154	5.91E-28	Saliva_End	PPP3CA
FLNA	4.43E-32	0.549248	0.24	0.148	7.07E-28	Saliva_End	FLNA
C14orf37	5.25E-32	0.313697	0.045	0.013	8.37E-28	Saliva_End	C14orf37
TMEM44	6.56E-32	0.325038	0.054	0.017	1.05E-27	Saliva_End	TMEM44
PLEKHG1	7.57E-32	0.313399	0.062	0.021	1.21E-27	Saliva_End	PLEKHG1
DGKH	9.9E-32	0.475316	0.086	0.035	1.58E-27	Saliva_End	DGKH
ROCK2	1.27E-31	0.710752	0.167	0.094	2.02E-27	Saliva_End	ROCK2
NFATC2	1.64E-31	0.469252	0.101	0.044	2.62E-27	Saliva_End	NFATC2
TTC28	1.82E-31	0.454117	0.082	0.033	2.9E-27	Saliva_End	TTC28
RBMS3	2.4E-31	0.536744	0.111	0.051	3.83E-27	Saliva_End	RBMS3
PIR	2.95E-31	0.387922	0.064	0.023	4.71E-27	Saliva_End	PIR
LHFP	3.52E-31	0.527598	0.161	0.086	5.61E-27	Saliva_End	LHFP
EXOC6	3.6E-31	0.524131	0.089	0.038	5.74E-27	Saliva_End	EXOC6
TMCC3	5.98E-31	0.364215	0.069	0.026	9.54E-27	Saliva_End	TMCC3
ARHGEF3	6.08E-31	0.518337	0.102	0.046	9.7E-27	Saliva_End	ARHGEF3
ATXN3	8.62E-31	0.675125	0.15	0.081	1.37E-26	Saliva_End	ATXN3
MAP3K1	1.33E-30	0.612228	0.13	0.067	2.11E-26	Saliva_End	MAP3K1
BAALC	1.51E-30	0.367353	0.05	0.015	2.41E-26	Saliva_End	BAALC
PRKCDBP	3.53E-30	0.584955	0.212	0.128	5.63E-26	Saliva_End	PRKCDBP
DGKE	4.76E-30	0.443772	0.071	0.027	7.6E-26	Saliva_End	DGKE
TMEM109	6.34E-30	0.692864	0.206	0.127	1.01E-25	Saliva_End	TMEM109
CDC42EP3	1.31E-29	0.617447	0.166	0.094	2.08E-25	Saliva_End	CDC42EP3
SEC11A	1.33E-29	0.725146	0.381	0.314	2.12E-25	Saliva_End	SEC11A
TCN2	1.65E-29	0.424761	0.083	0.035	2.63E-25	Saliva_End	TCN2
MAP3K3	1.92E-29	0.437134	0.083	0.035	3.06E-25	Saliva_End	MAP3K3
PKP4	2.23E-29	0.697886	0.141	0.077	3.56E-25	Saliva_End	PKP4
APP	2.44E-29	0.813813	0.319	0.243	3.89E-25	Saliva_End	APP
FIS1	4.22E-29	0.691109	0.401	0.337	6.73E-25	Saliva_End	FIS1
RILPL2	4.55E-29	0.69675	0.167	0.096	7.26E-25	Saliva_End	RILPL2
HINT1	6.42E-29	0.411573	0.624	0.57	1.02E-24	Saliva_End	HINT1
CRBN	6.45E-29	0.720417	0.219	0.142	1.03E-24	Saliva_End	CRBN
GRK5	7.2E-29	0.275012	0.058	0.02	1.15E-24	Saliva_End	GRK5
PITPNC1	1.04E-28	0.566	0.11	0.053	1.66E-24	Saliva_End	PITPNC1
PPP1R10	1.96E-28	0.794712	0.228	0.151	3.13E-24	Saliva_End	PPP1R10
ADAMTS6	1.99E-28	0.286463	0.035	0.009	3.18E-24	Saliva_End	ADAMTS6
EGR1	2.79E-28	0.820836	0.253	0.172	4.45E-24	Saliva_End	EGR1
ABCD4	2.86E-28	0.60629	0.125	0.064	4.56E-24	Saliva_End	ABCD4
EZH1	7.87E-28	0.484297	0.092	0.041	1.26E-23	Saliva_End	EZH1
HLA.DOA	8.2E-28	0.285051	0.05	0.016	1.31E-23	Saliva_End	HLA.DOA
C2orf40	9.13E-28	0.382182	0.09	0.039	1.46E-23	Saliva_End	C2orf40
BTF3	1.26E-27	0.311836	0.72	0.712	2.01E-23	Saliva_End	BTF3
ZNF347	1.31E-27	0.273247	0.043	0.013	2.09E-23	Saliva_End	ZNF347
CCDC50	1.38E-27	0.767955	0.209	0.137	2.2E-23	Saliva_End	CCDC50
FRMD4A	2.79E-27	0.643908	0.104	0.05	4.45E-23	Saliva_End	FRMD4A
NUDT3	2.89E-27	0.636426	0.171	0.102	4.61E-23	Saliva_End	NUDT3
CD59	4.31E-27	0.420316	0.592	0.514	6.88E-23	Saliva_End	CD59

RPS4Y1	5.08E-27	0.710672	0.35	0.272	8.11E-23	Saliva_End	RPS4Y1
EID1	5.94E-27	0.581849	0.427	0.357	9.48E-23	Saliva_End	EID1
S100A16	6.18E-27	0.584046	0.361	0.281	9.85E-23	Saliva_End	S100A16
PPP1R14B	1.07E-26	0.74456	0.249	0.176	1.71E-22	Saliva_End	PPP1R14B
ERAP2	1.08E-26	0.382886	0.07	0.028	1.72E-22	Saliva_End	ERAP2
ATRX	1.36E-26	0.759884	0.277	0.206	2.17E-22	Saliva_End	ATRX
CCND1	1.63E-26	0.738385	0.188	0.118	2.59E-22	Saliva_End	CCND1
NRGN	1.9E-26	0.262539	0.046	0.015	3.03E-22	Saliva_End	NRGN
SERPINI1	2.29E-26	0.483672	0.06	0.022	3.65E-22	Saliva_End	SERPINI1
DOCK9	2.41E-26	0.695971	0.164	0.098	3.85E-22	Saliva_End	DOCK9
RAC1	2.45E-26	0.501648	0.575	0.548	3.9E-22	Saliva_End	RAC1
SOGA1	2.69E-26	0.30092	0.06	0.022	4.3E-22	Saliva_End	SOGA1
ELN	2.7E-26	0.507149	0.113	0.056	4.31E-22	Saliva_End	ELN
QKI	4.44E-26	0.809918	0.242	0.171	7.08E-22	Saliva_End	QKI
BTN3A3	4.55E-26	0.281171	0.049	0.016	7.26E-22	Saliva_End	BTN3A3
C1QTNF2	5.95E-26	0.37651	0.061	0.023	9.49E-22	Saliva_End	C1QTNF2
BTG2	6.7E-26	0.599589	0.316	0.231	1.07E-21	Saliva_End	BTG2
IKBIP	7.57E-26	0.563909	0.116	0.06	1.21E-21	Saliva_End	IKBIP
ATP5J	1.77E-25	0.645207	0.47	0.436	2.83E-21	Saliva_End	ATP5J
DNAJA1	2.24E-25	0.777005	0.41	0.361	3.58E-21	Saliva_End	DNAJA1
SAMD9L	2.61E-25	0.434663	0.095	0.045	4.17E-21	Saliva_End	SAMD9L
ODF2L	3.13E-25	0.685505	0.18	0.112	4.99E-21	Saliva_End	ODF2L
PEF1	3.55E-25	0.625436	0.16	0.095	5.66E-21	Saliva_End	PEF1
GATAD1	3.77E-25	0.623635	0.135	0.075	6.02E-21	Saliva_End	GATAD1
TSPAN12	7.77E-25	0.555123	0.102	0.051	1.24E-20	Saliva_End	TSPAN12
FAR2	9.03E-25	0.261659	0.044	0.014	1.44E-20	Saliva_End	FAR2
POLE4	1.04E-24	0.616643	0.238	0.165	1.66E-20	Saliva_End	POLE4
GSDMD	1.09E-24	0.55647	0.166	0.098	1.74E-20	Saliva_End	GSDMD
MAOB	1.17E-24	0.264372	0.057	0.021	1.86E-20	Saliva_End	MAOB
DLC1	1.28E-24	0.465279	0.1	0.048	2.04E-20	Saliva_End	DLC1
TRIM25	1.3E-24	0.519106	0.117	0.061	2.07E-20	Saliva_End	TRIM25
STOX2	2.01E-24	0.373181	0.062	0.024	3.2E-20	Saliva_End	STOX2
HMGN3	2.1E-24	0.712041	0.342	0.277	3.35E-20	Saliva_End	HMGN3
SLC39A1	2.68E-24	0.667636	0.173	0.108	4.28E-20	Saliva_End	SLC39A1
SEPT7	2.89E-24	0.578228	0.404	0.339	4.61E-20	Saliva_End	SEPT7
MFNG	4.42E-24	0.272572	0.094	0.043	7.05E-20	Saliva_End	MFNG
CASP10	4.47E-24	0.434285	0.081	0.036	7.12E-20	Saliva_End	CASP10
NCL	4.93E-24	0.659919	0.338	0.27	7.86E-20	Saliva_End	NCL
NXN	4.93E-24	0.553874	0.13	0.073	7.86E-20	Saliva_End	NXN
KIAA0355	7.12E-24	0.635965	0.141	0.081	1.14E-19	Saliva_End	KIAA0355
C4orf32	7.69E-24	0.600128	0.14	0.081	1.23E-19	Saliva_End	C4orf32
INMT	8.86E-24	0.391851	0.053	0.019	1.41E-19	Saliva_End	INMT
CNRIP1	1.09E-23	0.369656	0.083	0.038	1.74E-19	Saliva_End	CNRIP1
HRH1	1.31E-23	0.352058	0.078	0.035	2.09E-19	Saliva_End	HRH1
BICD1	1.64E-23	0.255076	0.041	0.013	2.62E-19	Saliva_End	BICD1
SPTAN1	2.56E-23	0.8045	0.236	0.17	4.09E-19	Saliva_End	SPTAN1
ENY2	3.04E-23	0.744953	0.365	0.321	4.85E-19	Saliva_End	ENY2

MAPK3	4.9E-23	0.69718	0.176	0.112	7.82E-19	Saliva_End	MAPK3
CCDC69	5.28E-23	0.526564	0.188	0.119	8.42E-19	Saliva_End	CCDC69
HAPLN3	5.89E-23	0.353702	0.077	0.034	9.39E-19	Saliva_End	HAPLN3
SEC14L1	6.1E-23	0.837288	0.221	0.156	9.73E-19	Saliva_End	SEC14L1
SORBS2	9.03E-23	0.737685	0.175	0.112	1.44E-18	Saliva_End	SORBS2
FOXC1	1.11E-22	0.6454	0.173	0.111	1.77E-18	Saliva_End	FOXC1
TUBB	1.27E-22	0.552228	0.383	0.31	2.02E-18	Saliva_End	TUBB
CARD8	1.42E-22	0.463803	0.104	0.053	2.26E-18	Saliva_End	CARD8
MT.ATP81	2.58E-22	0.619189	0.201	0.135	4.11E-18	Saliva_End	MT.ATP8
WARS	3.39E-22	0.642259	0.136	0.079	5.41E-18	Saliva_End	WARS
SNTG2	4.82E-22	0.261558	0.041	0.014	7.68E-18	Saliva_End	SNTG2
FXYD6	6.1E-22	0.446064	0.067	0.029	9.72E-18	Saliva_End	FXYD6
OAS2	1.04E-21	0.426767	0.071	0.032	1.67E-17	Saliva_End	OAS2
MARCKSL1	1.28E-21	0.688295	0.27	0.204	2.04E-17	Saliva_End	MARCKSL1
CHURC1	1.86E-21	0.745084	0.276	0.216	2.97E-17	Saliva_End	CHURC1
PSIP1	2.04E-21	0.506837	0.191	0.123	3.25E-17	Saliva_End	PSIP1
DYNC1H1	2.11E-21	0.711551	0.2	0.139	3.36E-17	Saliva_End	DYNC1H1
FAM101B	2.33E-21	0.448787	0.051	0.019	3.72E-17	Saliva_End	FAM101B
SSB	2.78E-21	0.646741	0.264	0.197	4.44E-17	Saliva_End	SSB
TNFAIP1	2.96E-21	0.575342	0.105	0.056	4.71E-17	Saliva_End	TNFAIP1
PNISR	3.36E-21	0.65719	0.393	0.347	5.35E-17	Saliva_End	PNISR
NFIA	3.96E-21	0.648207	0.238	0.172	6.31E-17	Saliva_End	NFIA
TSPAN14	5.49E-21	0.696096	0.184	0.122	8.76E-17	Saliva_End	TSPAN14
LTBR	5.5E-21	0.604287	0.144	0.088	8.77E-17	Saliva_End	LTBR
TNRC6B	9.05E-21	0.680833	0.234	0.171	1.44E-16	Saliva_End	TNRC6B
LTC4S	1.03E-20	0.37247	0.07	0.032	1.64E-16	Saliva_End	LTC4S
STIM2	1.04E-20	0.465732	0.102	0.054	1.66E-16	Saliva_End	STIM2
RECQL	1.2E-20	0.405742	0.105	0.056	1.92E-16	Saliva_End	RECQL
CDC42BPB	1.45E-20	0.545595	0.116	0.065	2.32E-16	Saliva_End	CDC42BPB
SHC1	1.62E-20	0.522045	0.118	0.066	2.59E-16	Saliva_End	SHC1
CLDN15	1.66E-20	0.31885	0.055	0.022	2.65E-16	Saliva_End	CLDN15
NQO1	1.72E-20	0.455588	0.078	0.037	2.74E-16	Saliva_End	NQO1
EPB41L4A	2.17E-20	0.377825	0.064	0.028	3.46E-16	Saliva_End	EPB41L4A
WSB1	2.2E-20	0.761606	0.306	0.249	3.51E-16	Saliva_End	WSB1
GON4L	3.16E-20	0.583083	0.134	0.08	5.04E-16	Saliva_End	GON4L
SDCBP	3.99E-20	0.479476	0.46	0.411	6.36E-16	Saliva_End	SDCBP
NRARP	4.3E-20	0.445723	0.082	0.04	6.86E-16	Saliva_End	NRARP
CD200	8.27E-20	0.402843	0.05	0.02	1.32E-15	Saliva_End	CD200
SERTAD4.A	8.88E-20	0.469978	0.091	0.047	1.42E-15	Saliva_End	SERTAD4.AS1
ADAR	1.11E-19	0.717599	0.194	0.135	1.77E-15	Saliva_End	ADAR
HDAC7	1.3E-19	0.608652	0.166	0.107	2.07E-15	Saliva_End	HDAC7
PARP9	2.15E-19	0.538447	0.1	0.054	3.42E-15	Saliva_End	PARP9
FRYL	2.47E-19	0.639907	0.142	0.089	3.94E-15	Saliva_End	FRYL
SWAP70	2.69E-19	0.734058	0.197	0.139	4.29E-15	Saliva_End	SWAP70
SERBP1	2.92E-19	0.613632	0.399	0.354	4.66E-15	Saliva_End	SERBP1
CALCOCO2	3.35E-19	0.599592	0.198	0.137	5.34E-15	Saliva_End	CALCOCO2
ZNF462	3.82E-19	0.506901	0.098	0.053	6.1E-15	Saliva_End	ZNF462

RCAN1	4.15E-19	0.580589	0.124	0.072	6.61E-15	Saliva_End	RCAN1
SERPING1	4.19E-19	0.341678	0.248	0.172	6.68E-15	Saliva_End	SERPING1
U2SURP	5.43E-19	0.719437	0.23	0.171	8.67E-15	Saliva_End	U2SURP
TRIOBP	5.66E-19	0.652462	0.162	0.107	9.03E-15	Saliva_End	TRIOBP
RALGDS	6.39E-19	0.547754	0.125	0.074	1.02E-14	Saliva_End	RALGDS
PGM2L1	6.53E-19	0.424131	0.086	0.044	1.04E-14	Saliva_End	PGM2L1
NOC3L	1.09E-18	0.406681	0.091	0.048	1.74E-14	Saliva_End	NOC3L
ATP1A1	1.3E-18	0.679188	0.308	0.255	2.07E-14	Saliva_End	ATP1A1
TRIM22	1.54E-18	0.43971	0.097	0.052	2.45E-14	Saliva_End	TRIM22
HMCN1	1.56E-18	0.280487	0.039	0.014	2.48E-14	Saliva_End	HMCN1
PRRC2C	2.34E-18	0.68618	0.325	0.278	3.73E-14	Saliva_End	PRRC2C
AKAP13	2.7E-18	0.707064	0.27	0.216	4.31E-14	Saliva_End	AKAP13
GGT5	2.81E-18	0.273893	0.057	0.024	4.48E-14	Saliva_End	GGT5
MEF2A	2.84E-18	0.642407	0.16	0.106	4.54E-14	Saliva_End	MEF2A
PML	2.88E-18	0.526172	0.107	0.061	4.6E-14	Saliva_End	PML
TAOK2	2.89E-18	0.470127	0.075	0.037	4.61E-14	Saliva_End	TAOK2
SLC12A7	3.69E-18	0.358459	0.055	0.024	5.89E-14	Saliva_End	SLC12A7
NLRC5	4.04E-18	0.266722	0.045	0.018	6.44E-14	Saliva_End	NLRC5
CDC37	4.15E-18	0.708681	0.32	0.274	6.61E-14	Saliva_End	CDC37
CRTAP	4.26E-18	0.547803	0.171	0.114	6.8E-14	Saliva_End	CRTAP
SNAI1	4.43E-18	0.30257	0.049	0.02	7.07E-14	Saliva_End	SNAI1
DEK	5.13E-18	0.669301	0.309	0.258	8.18E-14	Saliva_End	DEK
MYCBP2	6.38E-18	0.614566	0.202	0.144	1.02E-13	Saliva_End	MYCBP2
S100A13	6.59E-18	0.514967	0.319	0.256	1.05E-13	Saliva_End	S100A13
SAP30	6.83E-18	0.467698	0.097	0.054	1.09E-13	Saliva_End	SAP30
COL4A2	7.51E-18	0.527947	0.099	0.054	1.2E-13	Saliva_End	COL4A2
GUCY1A3	7.75E-18	0.487371	0.069	0.033	1.24E-13	Saliva_End	GUCY1A3
LYL1	8.77E-18	0.359698	0.122	0.071	1.4E-13	Saliva_End	LYL1
POLK	1.22E-17	0.560095	0.118	0.07	1.95E-13	Saliva_End	POLK
RCN1	1.69E-17	0.736333	0.2	0.147	2.7E-13	Saliva_End	RCN1
ICAM1	1.72E-17	0.428249	0.198	0.135	2.74E-13	Saliva_End	ICAM1
LYST	1.82E-17	0.530958	0.145	0.093	2.9E-13	Saliva_End	LYST
GNAQ	1.93E-17	0.749551	0.216	0.163	3.08E-13	Saliva_End	GNAQ
APBB2	2.29E-17	0.433846	0.066	0.032	3.65E-13	Saliva_End	APBB2
DDX60L	3.42E-17	0.42239	0.075	0.038	5.45E-13	Saliva_End	DDX60L
RP11.798N	4.94E-17	0.25133	0.049	0.021	7.88E-13	Saliva_End	RP11.798M19.6
PSMB1	5.21E-17	0.575956	0.422	0.4	8.3E-13	Saliva_End	PSMB1
CMPK2	5.44E-17	0.321351	0.042	0.016	8.67E-13	Saliva_End	CMPK2
TAF1C	6.11E-17	0.3087	0.053	0.023	9.74E-13	Saliva_End	TAF1C
EBPL	6.77E-17	0.57226	0.189	0.134	1.08E-12	Saliva_End	EBPL
PXDN	7.01E-17	0.402865	0.076	0.039	1.12E-12	Saliva_End	PXDN
APPL1	7.2E-17	0.679284	0.176	0.123	1.15E-12	Saliva_End	APPL1
BCAP29	8.89E-17	0.742334	0.205	0.153	1.42E-12	Saliva_End	BCAP29
TMEM2	1.13E-16	0.503695	0.102	0.058	1.8E-12	Saliva_End	TMEM2
SERTAD1	1.21E-16	0.742182	0.281	0.23	1.93E-12	Saliva_End	SERTAD1
EVA1C	1.66E-16	0.511678	0.118	0.071	2.64E-12	Saliva_End	EVA1C
TMEM43	1.69E-16	0.455917	0.115	0.069	2.7E-12	Saliva_End	TMEM43

SLC43A3	1.72E-16	0.359826	0.067	0.033	2.74E-12	Saliva_End	SLC43A3
TMEM184I	1.88E-16	0.384907	0.065	0.031	3.01E-12	Saliva_End	TMEM184B
EPHX1	1.99E-16	0.648745	0.174	0.121	3.18E-12	Saliva_End	EPHX1
TRIM44	2.19E-16	0.579956	0.138	0.089	3.49E-12	Saliva_End	TRIM44
SRFBP1	2.26E-16	0.516725	0.105	0.061	3.6E-12	Saliva_End	SRFBP1
PLOD3	2.37E-16	0.366043	0.075	0.039	3.78E-12	Saliva_End	PLOD3
DAB2IP	2.62E-16	0.356729	0.058	0.027	4.18E-12	Saliva_End	DAB2IP
SRRM2	2.72E-16	0.689438	0.374	0.348	4.33E-12	Saliva_End	SRRM2
CDC25B	3.37E-16	0.252979	0.074	0.037	5.38E-12	Saliva_End	CDC25B
VPS13C	3.54E-16	0.567968	0.142	0.092	5.65E-12	Saliva_End	VPS13C
STXBP1	4.05E-16	0.370679	0.069	0.034	6.45E-12	Saliva_End	STXBP1
SAMD1	4.53E-16	0.333591	0.067	0.033	7.23E-12	Saliva_End	SAMD1
HELZ2	4.79E-16	0.441867	0.085	0.046	7.65E-12	Saliva_End	HELZ2
TMEM245	6.01E-16	0.556434	0.114	0.069	9.59E-12	Saliva_End	TMEM245
TSPO	9.81E-16	0.319966	0.491	0.466	1.56E-11	Saliva_End	TSPO
VGLL4	9.87E-16	0.664914	0.185	0.135	1.57E-11	Saliva_End	VGLL4
TAP1	1.19E-15	0.548644	0.146	0.095	1.89E-11	Saliva_End	TAP1
CNN3	1.23E-15	0.560456	0.277	0.224	1.95E-11	Saliva_End	CNN3
STAT1	1.28E-15	0.537845	0.134	0.086	2.05E-11	Saliva_End	STAT1
PTP4A3	1.3E-15	0.436328	0.065	0.032	2.07E-11	Saliva_End	PTP4A3
GIT1	1.42E-15	0.433379	0.085	0.046	2.27E-11	Saliva_End	GIT1
BTN3A1	1.45E-15	0.25472	0.051	0.022	2.31E-11	Saliva_End	BTN3A1
CNTLN	2.54E-15	0.381843	0.071	0.036	4.05E-11	Saliva_End	CNTLN
FAM89A	2.95E-15	0.306671	0.045	0.019	4.71E-11	Saliva_End	FAM89A
HNRNPA2E	3.16E-15	0.483404	0.505	0.492	5.04E-11	Saliva_End	HNRNPA2B1
HLA.DMB	3.31E-15	0.272114	0.114	0.067	5.28E-11	Saliva_End	HLA.DMB
IQGAP1	3.47E-15	0.695724	0.297	0.258	5.53E-11	Saliva_End	IQGAP1
FES	3.94E-15	0.374132	0.07	0.036	6.28E-11	Saliva_End	FES
BNIP2	3.95E-15	0.538547	0.175	0.124	6.3E-11	Saliva_End	BNIP2
SOX13	5.14E-15	0.393289	0.057	0.027	8.2E-11	Saliva_End	SOX13
PRKDC	5.56E-15	0.532779	0.145	0.098	8.86E-11	Saliva_End	PRKDC
ACE	6.74E-15	0.43169	0.087	0.048	1.07E-10	Saliva_End	ACE
ABLIM3	7.46E-15	0.368018	0.069	0.035	1.19E-10	Saliva_End	ABLIM3
RUNDC3B	9.35E-15	0.303411	0.044	0.019	1.49E-10	Saliva_End	RUNDC3B
CMIP	1.02E-14	0.544513	0.163	0.114	1.62E-10	Saliva_End	CMIP
C9orf3	1.07E-14	0.588239	0.145	0.098	1.71E-10	Saliva_End	C9orf3
NAV1	1.08E-14	0.309774	0.06	0.029	1.73E-10	Saliva_End	NAV1
ZNF37A	1.09E-14	0.440962	0.095	0.056	1.73E-10	Saliva_End	ZNF37A
KCNK5	1.3E-14	0.250682	0.029	0.01	2.08E-10	Saliva_End	KCNK5
HSPA1A	1.32E-14	0.417983	0.381	0.326	2.1E-10	Saliva_End	HSPA1A
C9orf40	1.33E-14	0.269929	0.042	0.018	2.13E-10	Saliva_End	C9orf40
PLLP	1.48E-14	0.421232	0.05	0.023	2.36E-10	Saliva_End	PLLP
MYO5C	1.53E-14	0.479894	0.086	0.049	2.45E-10	Saliva_End	MYO5C
C10orf54	1.65E-14	0.607044	0.225	0.171	2.63E-10	Saliva_End	C10orf54
CDK17	1.67E-14	0.393081	0.074	0.04	2.66E-10	Saliva_End	CDK17
KLF7	1.78E-14	0.422232	0.085	0.048	2.84E-10	Saliva_End	KLF7
GNS	1.89E-14	0.59804	0.129	0.085	3.02E-10	Saliva_End	GNS

MYBBP1A	2.21E-14	0.320449	0.054	0.026	3.53E-10	Saliva_End	MYBBP1A
ANGPT2	2.31E-14	0.447273	0.058	0.028	3.69E-10	Saliva_End	ANGPT2
SELM	2.31E-14	0.388225	0.404	0.346	3.69E-10	Saliva_End	SELM
HPCAL1	3.26E-14	0.456123	0.122	0.078	5.2E-10	Saliva_End	HPCAL1
SRSF10	3.52E-14	0.654593	0.203	0.156	5.61E-10	Saliva_End	SRSF10
NUDCD1	3.8E-14	0.384311	0.079	0.044	6.06E-10	Saliva_End	NUDCD1
CADPS2	3.96E-14	0.413548	0.079	0.044	6.31E-10	Saliva_End	CADPS2
SLC48A1	4.58E-14	0.520374	0.085	0.049	7.3E-10	Saliva_End	SLC48A1
FSCN1	5.31E-14	0.522194	0.108	0.067	8.46E-10	Saliva_End	FSCN1
SPTLC2	7.12E-14	0.567215	0.123	0.081	1.14E-09	Saliva_End	SPTLC2
MTPN	7.27E-14	0.552229	0.198	0.149	1.16E-09	Saliva_End	MTPN
FRMD4B	8.23E-14	0.664689	0.172	0.127	1.31E-09	Saliva_End	FRMD4B
TPR	8.86E-14	0.581886	0.25	0.202	1.41E-09	Saliva_End	TPR
SEMA3F	9.02E-14	0.300052	0.057	0.028	1.44E-09	Saliva_End	SEMA3F
XRCC5	9.83E-14	0.654389	0.285	0.247	1.57E-09	Saliva_End	XRCC5
SLC25A3	1.33E-13	0.478724	0.46	0.456	2.12E-09	Saliva_End	SLC25A3
LAMC1	1.35E-13	0.43909	0.12	0.077	2.15E-09	Saliva_End	LAMC1
TM6SF1	1.58E-13	0.25543	0.042	0.018	2.51E-09	Saliva_End	TM6SF1
ITGA3	1.76E-13	0.343645	0.062	0.032	2.8E-09	Saliva_End	ITGA3
TGFB1I1	1.91E-13	0.367731	0.089	0.052	3.05E-09	Saliva_End	TGFB1I1
DDX18	2.16E-13	0.60924	0.25	0.204	3.45E-09	Saliva_End	DDX18
DOCK1	2.37E-13	0.358999	0.069	0.037	3.78E-09	Saliva_End	DOCK1
SLC44A2	3.55E-13	0.552831	0.122	0.08	5.66E-09	Saliva_End	SLC44A2
RPL7L1	4.15E-13	0.541212	0.164	0.118	6.61E-09	Saliva_End	RPL7L1
CCNI	4.36E-13	0.386134	0.51	0.519	6.95E-09	Saliva_End	CCNI
RASAL2	5.51E-13	0.59493	0.134	0.092	8.78E-09	Saliva_End	RASAL2
TBX1	5.76E-13	0.334877	0.048	0.023	9.19E-09	Saliva_End	TBX1
KIAA0141	5.84E-13	0.368869	0.072	0.04	9.31E-09	Saliva_End	KIAA0141
ARL15	5.84E-13	0.373777	0.049	0.023	9.32E-09	Saliva_End	ARL15
FYN	7.05E-13	0.408045	0.107	0.066	1.12E-08	Saliva_End	FYN
DNASE1L1	7.25E-13	0.265566	0.053	0.026	1.16E-08	Saliva_End	DNASE1L1
MCM3	1.02E-12	0.331934	0.069	0.038	1.63E-08	Saliva_End	MCM3
TRIO	1.02E-12	0.319437	0.062	0.032	1.63E-08	Saliva_End	TRIO
FNIP2	1.22E-12	0.521425	0.132	0.09	1.94E-08	Saliva_End	FNIP2
LUZP1	1.23E-12	0.579897	0.168	0.123	1.97E-08	Saliva_End	LUZP1
LIMA1	1.29E-12	0.627047	0.226	0.182	2.05E-08	Saliva_End	LIMA1
SLFN11	1.37E-12	0.291154	0.061	0.031	2.19E-08	Saliva_End	SLFN11
CENPC	1.55E-12	0.456161	0.099	0.062	2.48E-08	Saliva_End	CENPC
HGSNAT	1.56E-12	0.454234	0.109	0.07	2.48E-08	Saliva_End	HGSNAT
KMT2A	1.95E-12	0.598418	0.174	0.13	3.11E-08	Saliva_End	KMT2A
INPP1	2.81E-12	0.331579	0.126	0.083	4.48E-08	Saliva_End	INPP1
SUMO2	3.07E-12	0.36367	0.575	0.602	4.89E-08	Saliva_End	SUMO2
COX7A1	3.37E-12	0.510477	0.185	0.139	5.38E-08	Saliva_End	COX7A1
STARD13	4.14E-12	0.261003	0.042	0.019	6.6E-08	Saliva_End	STARD13
COBLL1	4.62E-12	0.596984	0.14	0.099	7.37E-08	Saliva_End	COBLL1
IFIH1	4.82E-12	0.421991	0.082	0.048	7.69E-08	Saliva_End	IFIH1
PHKB	5.01E-12	0.687204	0.146	0.106	7.98E-08	Saliva_End	PHKB

PPP1R14A	5.25E-12	0.495411	0.079	0.046	8.37E-08	Saliva_End	PPP1R14A
TRIM38	5.29E-12	0.502513	0.107	0.069	8.44E-08	Saliva_End	TRIM38
ZNF22	5.4E-12	0.553683	0.15	0.108	8.61E-08	Saliva_End	ZNF22
FAM171A1	5.45E-12	0.335547	0.057	0.03	8.69E-08	Saliva_End	FAM171A1
PTPRE	5.49E-12	0.415117	0.089	0.053	8.75E-08	Saliva_End	PTPRE
IL15	6.02E-12	0.28977	0.044	0.021	9.61E-08	Saliva_End	IL15
SPRY1	6.38E-12	0.268216	0.137	0.091	1.02E-07	Saliva_End	SPRY1
ARHGAP17	6.5E-12	0.346072	0.066	0.036	1.04E-07	Saliva_End	ARHGAP17
EHBP1L1	7.32E-12	0.316472	0.089	0.054	1.17E-07	Saliva_End	EHBP1L1
CTNND1	7.38E-12	0.588771	0.139	0.099	1.18E-07	Saliva_End	CTNND1
DICER1	7.82E-12	0.614801	0.144	0.103	1.25E-07	Saliva_End	DICER1
ZCCHC24	1.11E-11	0.254733	0.063	0.034	1.77E-07	Saliva_End	ZCCHC24
VAMP3	1.23E-11	0.627734	0.174	0.135	1.96E-07	Saliva_End	VAMP3
PURA	1.23E-11	0.608154	0.166	0.125	1.96E-07	Saliva_End	PURA
ELK4	1.44E-11	0.520569	0.105	0.068	2.29E-07	Saliva_End	ELK4
MARC2	1.5E-11	0.290837	0.05	0.025	2.39E-07	Saliva_End	MARC2
FERMT2	1.59E-11	0.564663	0.12	0.081	2.54E-07	Saliva_End	FERMT2
UVRAG	1.95E-11	0.371616	0.081	0.048	3.11E-07	Saliva_End	UVRAG
APLP2	2.08E-11	0.636055	0.328	0.304	3.31E-07	Saliva_End	APLP2
PARP4	2.38E-11	0.376204	0.071	0.041	3.79E-07	Saliva_End	PARP4
HSD17B11	2.73E-11	0.482312	0.184	0.138	4.35E-07	Saliva_End	HSD17B11
SENP7	2.74E-11	0.353443	0.065	0.036	4.37E-07	Saliva_End	SENP7
PPP1R13B	2.76E-11	0.467511	0.091	0.057	4.4E-07	Saliva_End	PPP1R13B
NBL1	2.91E-11	0.295124	0.158	0.11	4.65E-07	Saliva_End	NBL1
ADD1	2.99E-11	0.61021	0.178	0.137	4.77E-07	Saliva_End	ADD1
LUC7L3	3.29E-11	0.682984	0.294	0.266	5.25E-07	Saliva_End	LUC7L3
PHF14	3.46E-11	0.584922	0.165	0.125	5.52E-07	Saliva_End	PHF14
PALM	3.55E-11	0.295418	0.047	0.023	5.66E-07	Saliva_End	PALM
CTSL	3.71E-11	0.60275	0.145	0.104	5.92E-07	Saliva_End	CTSL
DYNC1LI2	4.02E-11	0.714564	0.198	0.162	6.41E-07	Saliva_End	DYNC1LI2
ZSCAN18	4.36E-11	0.515597	0.117	0.08	6.95E-07	Saliva_End	ZSCAN18
MAGI1	4.37E-11	0.537558	0.103	0.068	6.97E-07	Saliva_End	MAGI1
ZNF787	4.71E-11	0.287413	0.059	0.032	7.52E-07	Saliva_End	ZNF787
ZNF280D	4.84E-11	0.587545	0.121	0.084	7.72E-07	Saliva_End	ZNF280D
PHC2	5.32E-11	0.524424	0.102	0.067	8.49E-07	Saliva_End	PHC2
ATP11A	7.11E-11	0.35166	0.073	0.043	1.13E-06	Saliva_End	ATP11A
EEA1	7.64E-11	0.499392	0.138	0.099	1.22E-06	Saliva_End	EEA1
HERC2	8.49E-11	0.442339	0.105	0.069	1.35E-06	Saliva_End	HERC2
SLC27A3	1.01E-10	0.278962	0.053	0.028	1.6E-06	Saliva_End	SLC27A3
RNF115	1.29E-10	0.579282	0.145	0.106	2.05E-06	Saliva_End	RNF115
PSMA7	1.35E-10	0.374818	0.464	0.465	2.15E-06	Saliva_End	PSMA7
PSMA3	1.42E-10	0.669824	0.228	0.193	2.26E-06	Saliva_End	PSMA3
INHBB	1.45E-10	0.251617	0.042	0.021	2.31E-06	Saliva_End	INHBB
FDPS	1.61E-10	0.745226	0.225	0.192	2.57E-06	Saliva_End	FDPS
SH2B3	1.65E-10	0.306326	0.055	0.03	2.63E-06	Saliva_End	SH2B3
CLTA	1.67E-10	0.526422	0.389	0.386	2.66E-06	Saliva_End	CLTA
DPP8	1.77E-10	0.531578	0.108	0.074	2.82E-06	Saliva_End	DPP8

IKBKB	1.85E-10	0.37828	0.082	0.051	2.95E-06	Saliva_End	IKBKB
YPEL2	2E-10	0.555213	0.142	0.104	3.18E-06	Saliva_End	YPEL2
SHISA5	2.18E-10	0.386322	0.145	0.103	3.48E-06	Saliva_End	SHISA5
TPD52L11	2.46E-10	0.488002	0.173	0.136	3.93E-06	Saliva_End	TPD52L1
NFAT5	2.82E-10	0.583332	0.166	0.127	4.49E-06	Saliva_End	NFAT5
TLN1	3.15E-10	0.466422	0.17	0.13	5.02E-06	Saliva_End	TLN1
EEF2K	3.61E-10	0.414911	0.074	0.045	5.75E-06	Saliva_End	EEF2K
PROX1	4.01E-10	0.397194	0.034	0.015	6.4E-06	Saliva_End	PROX1
RAPGEF2	4.08E-10	0.55542	0.088	0.057	6.51E-06	Saliva_End	RAPGEF2
KLHL5	4.09E-10	0.326805	0.055	0.03	6.52E-06	Saliva_End	KLHL5
ELOVL1	4.13E-10	0.479163	0.134	0.098	6.58E-06	Saliva_End	ELOVL1
BFAR	4.53E-10	0.433094	0.111	0.075	7.23E-06	Saliva_End	BFAR
BCL7C	4.63E-10	0.452737	0.157	0.118	7.39E-06	Saliva_End	BCL7C
MRPS18C	5.7E-10	0.671948	0.22	0.188	9.09E-06	Saliva_End	MRPS18C
DTX3L	6.05E-10	0.36393	0.073	0.044	9.66E-06	Saliva_End	DTX3L
ITPKB	6.55E-10	0.305513	0.066	0.039	1.04E-05	Saliva_End	ITPKB
LXN	6.72E-10	0.502417	0.087	0.056	1.07E-05	Saliva_End	LXN
ZMIZ1	7.39E-10	0.424222	0.114	0.079	1.18E-05	Saliva_End	ZMIZ1
CFLAR	7.48E-10	0.63078	0.297	0.271	1.19E-05	Saliva_End	CFLAR
SEMA4C	1.07E-09	0.27261	0.048	0.025	1.7E-05	Saliva_End	SEMA4C
PEAK1	1.08E-09	0.294102	0.067	0.04	1.72E-05	Saliva_End	PEAK1
SYNJ2BP	1.18E-09	0.441676	0.103	0.07	1.88E-05	Saliva_End	SYNJ2BP
NUMA1	1.18E-09	0.512707	0.157	0.12	1.89E-05	Saliva_End	NUMA1
ZFP14	1.45E-09	0.332441	0.056	0.031	2.32E-05	Saliva_End	ZFP14
SOS1	1.72E-09	0.590629	0.09	0.06	2.74E-05	Saliva_End	SOS1
DDX24	1.8E-09	0.491281	0.273	0.237	2.88E-05	Saliva_End	DDX24
PTPRG	1.91E-09	0.386659	0.064	0.038	3.04E-05	Saliva_End	PTPRG
BPTF	1.97E-09	0.583308	0.198	0.164	3.14E-05	Saliva_End	BPTF
SET	2.02E-09	0.528386	0.33	0.311	3.23E-05	Saliva_End	SET
ZNF621	2.45E-09	0.265783	0.043	0.022	3.9E-05	Saliva_End	ZNF621
NMI	2.58E-09	0.327201	0.099	0.066	4.12E-05	Saliva_End	NMI
MAP4	2.69E-09	0.559888	0.164	0.129	4.29E-05	Saliva_End	MAP4
HSD17B12	2.71E-09	0.625491	0.147	0.113	4.32E-05	Saliva_End	HSD17B12
FAM126A	2.87E-09	0.410396	0.093	0.061	4.58E-05	Saliva_End	FAM126A
CYB5A	2.88E-09	0.56411	0.217	0.182	4.59E-05	Saliva_End	CYB5A
CA5B	3.15E-09	0.300472	0.058	0.033	5.02E-05	Saliva_End	CA5B
C8orf59	3.69E-09	0.600952	0.259	0.235	5.88E-05	Saliva_End	C8orf59
SSBP4	3.76E-09	0.44036	0.139	0.102	5.99E-05	Saliva_End	SSBP4
CMTM8	3.96E-09	0.558581	0.1	0.069	6.32E-05	Saliva_End	CMTM8
CHN1	4.15E-09	0.265713	0.043	0.022	6.62E-05	Saliva_End	CHN1
PIK3CA	4.17E-09	0.317852	0.066	0.04	6.66E-05	Saliva_End	PIK3CA
HEY1	4.24E-09	0.45487	0.05	0.027	6.76E-05	Saliva_End	HEY1
PNN	4.39E-09	0.627141	0.241	0.212	7E-05	Saliva_End	PNN
NDUFB8	4.9E-09	0.566322	0.209	0.178	7.82E-05	Saliva_End	NDUFB8
EIF2AK4	5.65E-09	0.425055	0.1	0.069	9E-05	Saliva_End	EIF2AK4
XPC	5.76E-09	0.343507	0.091	0.061	9.19E-05	Saliva_End	XPC
GBP3	5.77E-09	0.314641	0.07	0.043	9.21E-05	Saliva_End	GBP3

SERPINH1	5.83E-09	0.414607	0.153	0.115	9.3E-05	Saliva_End	SERPINH1
IFI35	5.91E-09	0.394613	0.108	0.075	9.43E-05	Saliva_End	IFI35
CD40	7.15E-09	0.37089	0.107	0.074	0.000114	Saliva_End	CD40
SEPT10	8.29E-09	0.610038	0.188	0.157	0.000132	Saliva_End	SEPT10
DIAPH2	8.43E-09	0.318084	0.083	0.054	0.000134	Saliva_End	DIAPH2
HSPA2	8.85E-09	0.339439	0.101	0.069	0.000141	Saliva_End	HSPA2
EFNB1	1.04E-08	0.358451	0.059	0.035	0.000165	Saliva_End	EFNB1
TECPR1	1.07E-08	0.333855	0.058	0.034	0.000171	Saliva_End	TECPR1
GINM1	1.15E-08	0.555848	0.181	0.148	0.000183	Saliva_End	GINM1
NFIC	1.18E-08	0.559209	0.205	0.171	0.000188	Saliva_End	NFIC
ESF1	1.23E-08	0.535666	0.149	0.115	0.000196	Saliva_End	ESF1
RHOU	1.26E-08	0.32397	0.071	0.045	0.0002	Saliva_End	RHOU
XRN2	1.46E-08	0.583209	0.182	0.15	0.000232	Saliva_End	XRN2
AIDA	1.48E-08	0.49188	0.136	0.103	0.000236	Saliva_End	AIDA
ARRB1	1.56E-08	0.267961	0.06	0.036	0.000249	Saliva_End	ARRB1
FEZ2	2.03E-08	0.497295	0.142	0.109	0.000324	Saliva_End	FEZ2
EIF5	2.22E-08	0.596392	0.361	0.36	0.000354	Saliva_End	EIF5
TGOLN2	2.43E-08	0.619492	0.201	0.172	0.000388	Saliva_End	TGOLN2
SNRK	2.44E-08	0.465223	0.146	0.112	0.000388	Saliva_End	SNRK
SEPN1	2.84E-08	0.318555	0.068	0.043	0.000452	Saliva_End	SEPN1
PLSCR1	2.91E-08	0.576207	0.24	0.212	0.000464	Saliva_End	PLSCR1
MPHOSPH	3.37E-08	0.494467	0.121	0.089	0.000538	Saliva_End	MPHOSPH10
LMAN1	3.91E-08	0.508849	0.224	0.193	0.000624	Saliva_End	LMAN1
ERICH1	4.36E-08	0.481156	0.138	0.105	0.000695	Saliva_End	ERICH1
LAYN	4.55E-08	0.336208	0.049	0.028	0.000726	Saliva_End	LAYN
NDST1	4.72E-08	0.368265	0.064	0.04	0.000753	Saliva_End	NDST1
PRKCH	4.97E-08	0.418857	0.118	0.086	0.000792	Saliva_End	PRKCH
PARP12	4.97E-08	0.322212	0.057	0.034	0.000793	Saliva_End	PARP12
UBN1	5.74E-08	0.422886	0.117	0.087	0.000916	Saliva_End	UBN1
MLEC	6.16E-08	0.396185	0.123	0.19	0.000982	Saliva_End	MLEC
TOMM20	7.03E-08	0.528031	0.333	0.326	0.001122	Saliva_End	TOMM20
AKR1C1	7.19E-08	0.258089	0.071	0.045	0.001147	Saliva_End	AKR1C1
TSHZ1	7.28E-08	0.314318	0.062	0.039	0.001161	Saliva_End	TSHZ1
PCNXL4	7.88E-08	0.427001	0.086	0.059	0.001256	Saliva_End	PCNXL4
RAPGEF1	7.95E-08	0.402173	0.094	0.065	0.001268	Saliva_End	RAPGEF1
TRNT1	8.87E-08	0.316922	0.065	0.041	0.001415	Saliva_End	TRNT1
EGLN1	1.17E-07	0.535691	0.125	0.095	0.001863	Saliva_End	EGLN1
MKNK2	1.17E-07	0.4897	0.148	0.117	0.001867	Saliva_End	MKNK2
CAMK1	1.18E-07	0.27083	0.057	0.035	0.00188	Saliva_End	CAMK1
GBP2	1.2E-07	0.474297	0.142	0.11	0.00192	Saliva_End	GBP2
FOSB	1.28E-07	0.503377	0.281	0.251	0.002039	Saliva_End	FOSB
ZNF302	1.35E-07	0.443737	0.107	0.078	0.002155	Saliva_End	ZNF302
RPS6KA3	1.38E-07	0.439804	0.126	0.095	0.002193	Saliva_End	RPS6KA3
ADRB2	1.39E-07	0.316846	0.07	0.045	0.00221	Saliva_End	ADRB2
EIF3J.AS1	1.53E-07	0.381833	0.085	0.058	0.002434	Saliva_End	EIF3J.AS1
TBC1D1	1.66E-07	0.406811	0.085	0.058	0.002648	Saliva_End	TBC1D1
RIF1	1.67E-07	0.475686	0.122	0.092	0.002663	Saliva_End	RIF1

CAST	1.88E-07	0.493679	0.39	0.398	0.002993	Saliva_End	CAST
UHRF2	2.19E-07	0.353571	0.07	0.046	0.003501	Saliva_End	UHRF2
HSPA8	2.73E-07	0.31128	0.495	0.511	0.004346	Saliva_End	HSPA8
TUBA1A	3.16E-07	0.34398	0.271	0.235	0.005034	Saliva_End	TUBA1A
MTDH	3.42E-07	0.511907	0.322	0.309	0.005453	Saliva_End	MTDH
ZBED6	3.47E-07	0.277197	0.058	0.036	0.005542	Saliva_End	ZBED6
NAA10	3.64E-07	0.539377	0.188	0.159	0.005808	Saliva_End	NAA10
ZRANB2	3.68E-07	0.57008	0.212	0.186	0.005873	Saliva_End	ZRANB2
HIPK3	3.83E-07	0.535268	0.148	0.119	0.006114	Saliva_End	HIPK3
WWC3	4.36E-07	0.295483	0.065	0.042	0.00696	Saliva_End	WWC3
TCF7L1	4.45E-07	0.257981	0.05	0.03	0.007103	Saliva_End	TCF7L1
PHACTR2	4.89E-07	0.457584	0.211	0.181	0.007797	Saliva_End	PHACTR2
KBTBD2	5.84E-07	0.321377	0.077	0.053	0.009322	Saliva_End	KBTBD2
GPATCH2	5.91E-07	0.390554	0.086	0.061	0.009426	Saliva_End	GPATCH2
CP	6.2E-07	0.382335	0.045	0.026	0.009882	Saliva_End	CP
RGS3	6.27E-07	0.320802	0.066	0.043	0.01	Saliva_End	RGS3
ZC3H13	6.29E-07	0.431735	0.152	0.122	0.010037	Saliva_End	ZC3H13
TAF1	6.47E-07	0.283127	0.056	0.035	0.010324	Saliva_End	TAF1
LIPA	7.38E-07	0.272876	0.102	0.073	0.011765	Saliva_End	LIPA
SRSF3	7.71E-07	0.421171	0.423	0.434	0.012296	Saliva_End	SRSF3
LSM2	9.38E-07	0.399635	0.199	0.169	0.014965	Saliva_End	LSM2
LARS	1.13E-06	0.519907	0.141	0.114	0.018067	Saliva_End	LARS
FAM111A	1.23E-06	0.400649	0.073	0.05	0.019592	Saliva_End	FAM111A
BCLAF1	1.31E-06	0.593834	0.221	0.199	0.020888	Saliva_End	BCLAF1
EXOSC6	1.39E-06	0.327515	0.07	0.047	0.022121	Saliva_End	EXOSC6
NOV	1.39E-06	0.446719	0.044	0.026	0.022138	Saliva_End	NOV
LTBP4	1.4E-06	0.310326	0.164	0.129	0.022311	Saliva_End	LTBP4
ZBTB20	1.46E-06	0.487184	0.162	0.134	0.023223	Saliva_End	ZBTB20
PARK7	1.54E-06	0.333351	0.372	0.356	0.024609	Saliva_End	PARK7
CDC42BPA	1.72E-06	0.486119	0.095	0.07	0.027355	Saliva_End	CDC42BPA
TNFRSF10E	1.77E-06	0.324601	0.065	0.043	0.028182	Saliva_End	TNFRSF10B
IMP3	1.8E-06	0.464066	0.214	0.187	0.028733	Saliva_End	IMP3
ROCK1	1.84E-06	0.484851	0.183	0.156	0.029352	Saliva_End	ROCK1
SF3B1	1.86E-06	0.588205	0.266	0.253	0.02966	Saliva_End	SF3B1
NR3C1	1.93E-06	0.481034	0.177	0.149	0.030714	Saliva_End	NR3C1
AMOTL1	1.95E-06	0.360655	0.079	0.056	0.031075	Saliva_End	AMOTL1
LY96	2.16E-06	0.283419	0.095	0.068	0.034497	Saliva_End	LY96
SSBP1	2.31E-06	0.5398	0.292	0.283	0.036906	Saliva_End	SSBP1
THUMPD1	2.32E-06	0.438537	0.129	0.102	0.037061	Saliva_End	THUMPD1
ZNF791	2.49E-06	0.361972	0.095	0.07	0.039743	Saliva_End	ZNF791
MLLT6	2.62E-06	0.447353	0.13	0.103	0.041721	Saliva_End	MLLT6
PPIG	2.79E-06	0.569669	0.265	0.25	0.044505	Saliva_End	PPIG
CDC42EP4	2.84E-06	0.310491	0.079	0.056	0.045301	Saliva_End	CDC42EP4
MIER2	2.95E-06	0.314634	0.059	0.039	0.047119	Saliva_End	MIER2
C8orf41	3E-06	0.570774	0.148	0.12	0.047872	Saliva_End	C8orf4
PXN	3.09E-06	0.41197	0.101	0.075	0.049319	Saliva_End	PXN
CENPB	3.35E-06	0.304317	0.07	0.048	0.053463	Saliva_End	CENPB

TMEM168	3.42E-06	0.306476	0.057	0.037	0.054537	Saliva_End	TMEM168
C1orf43	3.42E-06	0.266834	0.159	0.23	0.054603	Saliva_End	C1orf43
SLC3A2	3.99E-06	0.526042	0.221	0.197	0.063591	Saliva_End	SLC3A2
MSX1	4.07E-06	0.275137	0.057	0.037	0.06491	Saliva_End	MSX1
ZNHIT6	4.7E-06	0.274845	0.054	0.035	0.074964	Saliva_End	ZNHIT6
CCT6A	4.83E-06	0.503456	0.202	0.179	0.076961	Saliva_End	CCT6A
TTC19	4.94E-06	0.513118	0.147	0.122	0.078806	Saliva_End	TTC19
RBM25	5E-06	0.536511	0.257	0.24	0.079819	Saliva_End	RBM25
CYSTM1	5.57E-06	0.408452	0.249	0.226	0.088854	Saliva_End	CYSTM1
SCAF11	5.68E-06	0.571482	0.246	0.232	0.090615	Saliva_End	SCAF11
GTF2I	6.19E-06	0.519105	0.177	0.154	0.098669	Saliva_End	GTF2I
ATL3	6.37E-06	0.471056	0.145	0.119	0.101666	Saliva_End	ATL3
SETX	6.88E-06	0.468378	0.127	0.102	0.109755	Saliva_End	SETX
SFXN3	7.04E-06	0.286535	0.064	0.043	0.112355	Saliva_End	SFXN3
CNOT6L	7.09E-06	0.504895	0.124	0.099	0.113022	Saliva_End	CNOT6L
RAB11A	7.16E-06	0.412422	0.382	0.384	0.114224	Saliva_End	RAB11A
ZNFX1	7.2E-06	0.391189	0.091	0.067	0.114805	Saliva_End	ZNFX1
MON1B	8.13E-06	0.294342	0.067	0.046	0.129625	Saliva_End	MON1B
LBR	8.2E-06	0.346769	0.089	0.065	0.130768	Saliva_End	LBR
KLF4	8.33E-06	0.475715	0.248	0.226	0.132858	Saliva_End	KLF4
ZMAT1	8.67E-06	0.279384	0.051	0.033	0.138308	Saliva_End	ZMAT1
MYO9A	8.68E-06	0.389098	0.098	0.074	0.138423	Saliva_End	MYO9A
RGS12	8.7E-06	0.307316	0.059	0.04	0.138814	Saliva_End	RGS12
BAZ1B	8.81E-06	0.395026	0.107	0.083	0.140474	Saliva_End	BAZ1B
C1orf54	8.99E-06	0.331882	0.069	0.048	0.143432	Saliva_End	C1orf54
KRR1	9.24E-06	0.54362	0.156	0.132	0.147316	Saliva_End	KRR1
CEP290	9.24E-06	0.293903	0.07	0.049	0.147421	Saliva_End	CEP290
CRK	1.03E-05	0.53834	0.143	0.119	0.163754	Saliva_End	CRK
TDRD3	1.07E-05	0.303708	0.061	0.042	0.170055	Saliva_End	TDRD3
KIAA1551	1.09E-05	0.438557	0.144	0.118	0.173396	Saliva_End	KIAA1551
LUC7L2	1.1E-05	0.392312	0.072	0.051	0.175934	Saliva_End	LUC7L2
PAK2	1.18E-05	0.578717	0.2	0.181	0.188935	Saliva_End	PAK2
MICAL3	1.2E-05	0.285706	0.046	0.029	0.191449	Saliva_End	MICAL3
PTPN12	1.29E-05	0.508606	0.132	0.107	0.206472	Saliva_End	PTPN12
ITGB1BP1	1.38E-05	0.442491	0.177	0.151	0.220113	Saliva_End	ITGB1BP1
PJA2	1.4E-05	0.495305	0.174	0.151	0.222657	Saliva_End	PJA2
APC	1.4E-05	0.322848	0.082	0.06	0.223059	Saliva_End	APC
NCK1	1.67E-05	0.476214	0.133	0.108	0.266327	Saliva_End	NCK1
EIF3E	1.87E-05	0.266375	0.449	0.45	0.297833	Saliva_End	EIF3E
HBEGF	1.9E-05	0.346387	0.07	0.049	0.302563	Saliva_End	HBEGF
AAED1	1.97E-05	0.362043	0.099	0.076	0.314422	Saliva_End	AAED1
IQCK	2.02E-05	0.468837	0.067	0.048	0.322493	Saliva_End	IQCK
TRIP11	2.05E-05	0.519326	0.143	0.121	0.326197	Saliva_End	TRIP11
MAGEH1	2.08E-05	0.269378	0.066	0.046	0.332428	Saliva_End	MAGEH1
RNF213	2.14E-05	0.452757	0.171	0.147	0.341388	Saliva_End	RNF213
ATF7IP	2.15E-05	0.402207	0.086	0.065	0.342438	Saliva_End	ATF7IP
THRAP3	2.16E-05	0.506454	0.196	0.175	0.344011	Saliva_End	THRAP3

AP1S2	2.28E-05	0.268571	0.154	0.125	0.364256	Saliva_End	AP1S2
TNS1	2.35E-05	0.303392	0.081	0.059	0.375003	Saliva_End	TNS1
LRRC57	2.39E-05	0.257876	0.05	0.033	0.380872	Saliva_End	LRRC57
PPP1R12A	2.49E-05	0.538149	0.198	0.178	0.397704	Saliva_End	PPP1R12A
LRCH1	2.51E-05	0.277302	0.058	0.04	0.400518	Saliva_End	LRCH1
PNP	2.62E-05	0.436508	0.098	0.075	0.418443	Saliva_End	PNP
METTL16	2.7E-05	0.271818	0.055	0.037	0.431362	Saliva_End	METTL16
WWTR1	2.91E-05	0.461807	0.152	0.129	0.464741	Saliva_End	WWTR1
PDLIM7	2.98E-05	0.304245	0.086	0.064	0.475935	Saliva_End	PDLIM7
CYP20A1	3E-05	0.483952	0.118	0.096	0.478233	Saliva_End	CYP20A1
SYPL1	3.22E-05	0.605553	0.269	0.264	0.513539	Saliva_End	SYPL1
SLC6A6	3.3E-05	0.443195	0.058	0.04	0.527046	Saliva_End	SLC6A6
SLC25A29	3.45E-05	0.380531	0.075	0.056	0.54974	Saliva_End	SLC25A29
ACAP2	3.54E-05	0.517031	0.164	0.143	0.564693	Saliva_End	ACAP2
DNAJB14	4.02E-05	0.449898	0.126	0.103	0.640629	Saliva_End	DNAJB14
TTC14	4.06E-05	0.488044	0.125	0.102	0.647277	Saliva_End	TTC14
RAI14	4.08E-05	0.436188	0.105	0.083	0.650243	Saliva_End	RAI14
HNRNPA3	4.21E-05	0.497506	0.352	0.366	0.671087	Saliva_End	HNRNPA3
TMEM14C	4.34E-05	0.54595	0.261	0.256	0.692184	Saliva_End	TMEM14C
IER5L	4.38E-05	0.354027	0.118	0.093	0.698955	Saliva_End	IER5L
SBF2	4.63E-05	0.408188	0.086	0.065	0.738545	Saliva_End	SBF2
SHPRH	4.89E-05	0.372984	0.091	0.07	0.779163	Saliva_End	SHPRH
PON2	4.89E-05	0.428773	0.092	0.072	0.779553	Saliva_End	PON2
CHSY1	4.99E-05	0.351581	0.074	0.054	0.796023	Saliva_End	CHSY1
SLCO3A1	5E-05	0.321971	0.074	0.054	0.797626	Saliva_End	SLCO3A1
LNPEP	5.22E-05	0.350245	0.089	0.067	0.833234	Saliva_End	LNPEP
ZNF638	5.24E-05	0.51927	0.151	0.13	0.835553	Saliva_End	ZNF638
C19orf66	5.28E-05	0.33266	0.106	0.083	0.842833	Saliva_End	C19orf66
IER5	5.37E-05	0.311409	0.236	0.21	0.856821	Saliva_End	IER5
DKK3	5.62E-05	0.423494	0.062	0.044	0.897017	Saliva_End	DKK3
CTTNBP2N	5.73E-05	0.472661	0.13	0.109	0.913109	Saliva_End	CTTNBP2NL
HNRNPR	5.74E-05	0.459156	0.21	0.193	0.915497	Saliva_End	HNRNPR
SMC3	5.82E-05	0.297499	0.166	0.142	0.927838	Saliva_End	SMC3
ADAM10	5.87E-05	0.389764	0.097	0.076	0.936986	Saliva_End	ADAM10
RABAC1	5.91E-05	0.263258	0.407	0.405	0.94229	Saliva_End	RABAC1
ZNF326	6.17E-05	0.412988	0.107	0.086	0.984839	Saliva_End	ZNF326
JAK1	6.58E-05	0.498463	0.258	0.249	1	Saliva_End	JAK1
CAPNS1	6.59E-05	0.578072	0.254	0.25	1	Saliva_End	CAPNS1
OSBPL1A	6.71E-05	0.643594	0.176	0.16	1	Saliva_End	OSBPL1A
TPM1	6.85E-05	0.277936	0.21	0.187	1	Saliva_End	TPM1
KIAA1109	7.05E-05	0.472007	0.106	0.086	1	Saliva_End	KIAA1109
DAD1	7.47E-05	0.418328	0.38	0.4	1	Saliva_End	DAD1
AGO2	7.83E-05	0.312694	0.071	0.052	1	Saliva_End	AGO2
TTC37	8.36E-05	0.461267	0.105	0.085	1	Saliva_End	TTC37
C10orf11	9.11E-05	0.331206	0.07	0.051	1	Saliva_End	C10orf11
OCIAD1	0.000102	0.585751	0.256	0.253	1	Saliva_End	OCIAD1
LIX1L	0.000105	0.270169	0.065	0.046	1	Saliva_End	LIX1L

CCNL1	0.000105	0.542059	0.331	0.341	1	Saliva_End	CCNL1
RAB3GAP2	0.000108	0.362895	0.077	0.058	1	Saliva_End	RAB3GAP2
ZFYVE21	0.000109	0.540128	0.166	0.149	1	Saliva_End	ZFYVE21
IFIT5	0.000122	0.282227	0.052	0.036	1	Saliva_End	IFIT5
MIER1	0.000128	0.528953	0.143	0.124	1	Saliva_End	MIER1
C4orf27	0.000129	0.299175	0.099	0.078	1	Saliva_End	C4orf27
BDP1	0.000133	0.419741	0.125	0.104	1	Saliva_End	BDP1
MRE11A	0.000135	0.27243	0.051	0.035	1	Saliva_End	MRE11A
MVP	0.000136	0.323797	0.113	0.091	1	Saliva_End	MVP
PRRC2B	0.000139	0.398773	0.108	0.088	1	Saliva_End	PRRC2B
VEZT	0.000146	0.394906	0.101	0.081	1	Saliva_End	VEZT
GNB1	0.000151	0.614288	0.221	0.214	1	Saliva_End	GNB1
SON	0.000162	0.457393	0.387	0.411	1	Saliva_End	SON
RFC1	0.000164	0.445279	0.134	0.115	1	Saliva_End	RFC1
TESC1	0.000165	0.320154	0.135	0.113	1	Saliva_End	TESC
SEC62	0.000175	0.36379	0.388	0.402	1	Saliva_End	SEC62
LYSMD2	0.000176	0.339113	0.094	0.074	1	Saliva_End	LYSMD2
TRIB1	0.000176	0.264064	0.064	0.047	1	Saliva_End	TRIB1
RHOA	0.000177	0.39988	0.437	0.475	1	Saliva_End	RHOA
ESYT1	0.000181	0.271356	0.08	0.061	1	Saliva_End	ESYT1
CSNK1A1	0.000192	0.450068	0.277	0.272	1	Saliva_End	CSNK1A1
ACOT9	0.000198	0.312686	0.079	0.06	1	Saliva_End	ACOT9
GFOD2	0.0002	0.342265	0.078	0.06	1	Saliva_End	GFOD2
AGTRAP	0.000208	0.396246	0.128	0.108	1	Saliva_End	AGTRAP
CD2AP	0.000214	0.260037	0.099	0.139	1	Saliva_End	CD2AP
CAMKK2	0.000217	0.305142	0.063	0.046	1	Saliva_End	CAMKK2
MLLT1	0.000231	0.294524	0.054	0.039	1	Saliva_End	MLLT1
CTDSP1	0.000234	0.38099	0.121	0.1	1	Saliva_End	CTDSP1
C16orf52	0.000237	0.308374	0.064	0.047	1	Saliva_End	C16orf52
UPF3A	0.000246	0.451657	0.164	0.145	1	Saliva_End	UPF3A
DENND4A	0.000254	0.258438	0.057	0.041	1	Saliva_End	DENND4A
SERINC1	0.000257	0.516289	0.209	0.197	1	Saliva_End	SERINC1
FAM84B	0.000263	0.414205	0.1	0.082	1	Saliva_End	FAM84B
RTN4	0.000271	0.37825	0.387	0.395	1	Saliva_End	RTN4
EMC2	0.000273	0.280663	0.09	0.127	1	Saliva_End	EMC2
PEPD	0.000276	0.427713	0.121	0.102	1	Saliva_End	PEPD
A4GALT	0.000282	0.481962	0.105	0.087	1	Saliva_End	A4GALT
NME3	0.000301	0.496192	0.222	0.209	1	Saliva_End	NME3
TCEAL4	0.000301	0.360117	0.178	0.16	1	Saliva_End	TCEAL4
PDLIM4	0.000301	0.362328	0.125	0.105	1	Saliva_End	PDLIM4
AQR	0.000307	0.340297	0.066	0.05	1	Saliva_End	AQR
CCPG1	0.000324	0.59852	0.182	0.168	1	Saliva_End	CCPG1
PHACTR4	0.000332	0.466521	0.142	0.124	1	Saliva_End	PHACTR4
PDCD10	0.000338	0.319874	0.151	0.205	1	Saliva_End	PDCD10
TNFSF12	0.000339	0.301694	0.078	0.06	1	Saliva_End	TNFSF12
SKI	0.000345	0.309566	0.078	0.06	1	Saliva_End	SKI
EGR2	0.00035	0.278143	0.049	0.034	1	Saliva_End	EGR2

TM2D1	0.000351	0.267807	0.115	0.158	1	Saliva_End	TM2D1
SMARCA5	0.000363	0.494983	0.147	0.13	1	Saliva_End	SMARCA5
ITIH5	0.000363	0.297654	0.055	0.039	1	Saliva_End	ITIH5
ESD	0.000369	0.483873	0.228	0.218	1	Saliva_End	ESD
SERINC3	0.000386	0.532309	0.217	0.208	1	Saliva_End	SERINC3
IRF2	0.000404	0.388562	0.107	0.089	1	Saliva_End	IRF2
CHM	0.000418	0.26443	0.058	0.042	1	Saliva_End	CHM
MYL12B	0.000439	0.267904	0.555	0.608	1	Saliva_End	MYL12B
DDX23	0.000474	0.290882	0.078	0.06	1	Saliva_End	DDX23
ABCE1	0.000479	0.399492	0.115	0.097	1	Saliva_End	ABCE1
RBM26	0.000483	0.418127	0.115	0.098	1	Saliva_End	RBM26
LSM3	0.000495	0.254006	0.229	0.306	1	Saliva_End	LSM3
PFDN1	0.000504	0.409337	0.134	0.116	1	Saliva_End	PFDN1
NOL11	0.000508	0.302195	0.067	0.051	1	Saliva_End	NOL11
GNA11	0.000538	0.488228	0.085	0.068	1	Saliva_End	GNA11
PTPRN2	0.000557	0.27275	0.061	0.045	1	Saliva_End	PTPRN2
SNHG8	0.000586	0.368049	0.302	0.297	1	Saliva_End	SNHG8
MIS18BP1	0.000615	0.450323	0.12	0.103	1	Saliva_End	MIS18BP1
GLS	0.000697	0.355113	0.136	0.117	1	Saliva_End	GLS
FLOT2	0.000699	0.261832	0.075	0.058	1	Saliva_End	FLOT2
FLYWCH2	0.000776	0.433673	0.131	0.114	1	Saliva_End	FLYWCH2
SFPQ	0.000798	0.51675	0.283	0.286	1	Saliva_End	SFPQ
MLH3	0.000811	0.297063	0.062	0.047	1	Saliva_End	MLH3
TMEM179I	0.000929	0.522233	0.206	0.198	1	Saliva_End	TMEM179B
PKIG	0.000961	0.448702	0.117	0.1	1	Saliva_End	PKIG
ZNF148	0.001049	0.488507	0.141	0.127	1	Saliva_End	ZNF148
DDX3X	0.001069	0.535131	0.293	0.302	1	Saliva_End	DDX3X
LIMCH1	0.001075	0.302143	0.094	0.129	1	Saliva_End	LIMCH1
SMARCE1	0.001102	0.417997	0.15	0.135	1	Saliva_End	SMARCE1
USP31	0.001119	0.269551	0.051	0.037	1	Saliva_End	USP31
NTAN1	0.001123	0.437206	0.14	0.124	1	Saliva_End	NTAN1
MGAT1	0.001148	0.28429	0.163	0.215	1	Saliva_End	MGAT1
GPALPP1	0.001227	0.261838	0.051	0.038	1	Saliva_End	GPALPP1
C2orf74	0.001287	0.332334	0.075	0.06	1	Saliva_End	C2orf74
PSMD5.AS	0.001298	0.323146	0.079	0.063	1	Saliva_End	PSMD5.AS1
MYADM	0.001341	0.299961	0.169	0.15	1	Saliva_End	MYADM
CEP164	0.001372	0.256567	0.071	0.055	1	Saliva_End	CEP164
MPHOSPH	0.001391	0.415141	0.258	0.254	1	Saliva_End	MPHOSPH8
MESDC1	0.001443	0.34644	0.095	0.079	1	Saliva_End	MESDC1
NEDD9	0.001445	0.403389	0.09	0.074	1	Saliva_End	NEDD9
RRAGA	0.001448	0.42518	0.144	0.129	1	Saliva_End	RRAGA
ACTN4	0.001553	0.450111	0.301	0.308	1	Saliva_End	ACTN4
SLC41A3	0.001582	0.365745	0.079	0.064	1	Saliva_End	SLC41A3
TANC1	0.00161	0.341952	0.073	0.058	1	Saliva_End	TANC1
IGFBP3	0.001612	1.075599	0.082	0.067	1	Saliva_End	IGFBP3
TBC1D5	0.001691	0.322566	0.074	0.059	1	Saliva_End	TBC1D5
EIF4B	0.001785	0.429235	0.291	0.301	1	Saliva_End	EIF4B

COMMD7	0.001962	0.397277	0.144	0.129	1	Saliva_End	COMMD7
LRCH3	0.00197	0.25438	0.055	0.041	1	Saliva_End	LRCH3
CDC27	0.001974	0.351209	0.092	0.076	1	Saliva_End	CDC27
STK25	0.00198	0.383059	0.106	0.091	1	Saliva_End	STK25
ASPH	0.002021	0.265377	0.146	0.192	1	Saliva_End	ASPH
DNTTIP2	0.002165	0.477636	0.122	0.107	1	Saliva_End	DNTTIP2
CTSZ	0.002208	0.272053	0.205	0.192	1	Saliva_End	CTSZ
H6PD	0.002304	0.319261	0.081	0.066	1	Saliva_End	H6PD
PSMG2	0.002332	0.288401	0.177	0.231	1	Saliva_End	PSMG2
OAS1	0.002345	0.366612	0.095	0.08	1	Saliva_End	OAS1
SH3BP4	0.002375	0.423634	0.095	0.081	1	Saliva_End	SH3BP4
NUCB1	0.002377	0.491143	0.212	0.207	1	Saliva_End	NUCB1
TTF1	0.002465	0.385799	0.102	0.086	1	Saliva_End	TTF1
TOX4	0.002533	0.285821	0.127	0.167	1	Saliva_End	TOX4
KAT6A	0.002695	0.340087	0.091	0.076	1	Saliva_End	KAT6A
RPAP2	0.002875	0.293077	0.065	0.051	1	Saliva_End	RPAP2
LRP5	0.002896	0.316566	0.056	0.043	1	Saliva_End	LRP5
RYK	0.002925	0.418347	0.089	0.075	1	Saliva_End	RYK
YTHDF2	0.003043	0.288603	0.135	0.177	1	Saliva_End	YTHDF2
EAPP	0.003052	0.257506	0.142	0.185	1	Saliva_End	EAPP
NPHP3	0.003103	0.264882	0.059	0.046	1	Saliva_End	NPHP3
TUG1	0.003135	0.28521	0.101	0.134	1	Saliva_End	TUG1
RAB14	0.003264	0.300528	0.145	0.189	1	Saliva_End	RAB14
RBMS2	0.00331	0.367793	0.101	0.086	1	Saliva_End	RBMS2
PRPSAP1	0.003319	0.470046	0.132	0.119	1	Saliva_End	PRPSAP1
ARID4A	0.003366	0.421742	0.123	0.109	1	Saliva_End	ARID4A
CDC42EP1	0.003409	0.270072	0.07	0.095	1	Saliva_End	CDC42EP1
PRDX3	0.003684	0.255604	0.121	0.158	1	Saliva_End	PRDX3
KLHDC8B	0.003688	0.33794	0.06	0.047	1	Saliva_End	KLHDC8B
PTBP1	0.003764	0.265296	0.114	0.15	1	Saliva_End	PTBP1
MESDC2	0.003783	0.281845	0.133	0.174	1	Saliva_End	MESDC2
HADHA	0.003805	0.463289	0.206	0.201	1	Saliva_End	HADHA
EPRS	0.003943	0.444354	0.145	0.133	1	Saliva_End	EPRS
CANX	0.003954	0.256865	0.245	0.314	1	Saliva_End	CANX
UBTF	0.003986	0.341455	0.101	0.086	1	Saliva_End	UBTF
VPS35	0.004235	0.286421	0.142	0.185	1	Saliva_End	VPS35
RBM28	0.004278	0.260427	0.057	0.044	1	Saliva_End	RBM28
LPCAT2	0.00431	0.279311	0.051	0.039	1	Saliva_End	LPCAT2
DIS3	0.004468	0.447506	0.097	0.084	1	Saliva_End	DIS3
SNRNP48	0.004496	0.260548	0.062	0.049	1	Saliva_End	SNRNP48
SMIM8	0.004518	0.275616	0.058	0.046	1	Saliva_End	SMIM8
ASL	0.004797	0.264237	0.066	0.053	1	Saliva_End	ASL
SLC39A10	0.004821	0.314439	0.071	0.058	1	Saliva_End	SLC39A10
SRSF2	0.004824	0.452958	0.319	0.333	1	Saliva_End	SRSF2
RGCC	0.004843	1.372542	0.216	0.215	1	Saliva_End	RGCC
SLC35D2	0.005139	0.261514	0.046	0.035	1	Saliva_End	SLC35D2
MRTO4	0.00519	0.289361	0.08	0.066	1	Saliva_End	MRTO4

BTBD7	0.005394	0.405946	0.111	0.099	1	Saliva_End	BTBD7
LONP2	0.005714	0.459917	0.101	0.088	1	Saliva_End	LONP2
NSMCE4A	0.005751	0.366351	0.095	0.082	1	Saliva_End	NSMCE4A
STX2	0.005784	0.283152	0.057	0.045	1	Saliva_End	STX2
DNAJC21	0.005805	0.467129	0.132	0.121	1	Saliva_End	DNAJC21
ALYREF	0.005886	0.299075	0.062	0.05	1	Saliva_End	ALYREF
USP14	0.006034	0.373537	0.113	0.1	1	Saliva_End	USP14
STARD3	0.006049	0.304167	0.075	0.062	1	Saliva_End	STARD3
CIR1	0.006271	0.271766	0.132	0.17	1	Saliva_End	CIR1
AFF1	0.006297	0.302094	0.083	0.07	1	Saliva_End	AFF1
FAM120A	0.006302	0.271047	0.115	0.149	1	Saliva_End	FAM120A
ARHGAP18	0.006537	0.292585	0.072	0.097	1	Saliva_End	ARHGAP18
IARS2	0.00668	0.431526	0.103	0.091	1	Saliva_End	IARS2
GFM1	0.006812	0.290861	0.065	0.053	1	Saliva_End	GFM1
RRP36	0.007053	0.316783	0.07	0.058	1	Saliva_End	RRP36
NIFK	0.007085	0.312609	0.11	0.097	1	Saliva_End	NIFK
BACE2	0.007128	0.510866	0.168	0.163	1	Saliva_End	BACE2
PHF20L1	0.007276	0.266856	0.1	0.131	1	Saliva_End	PHF20L1
MRPL17	0.00734	0.457009	0.11	0.098	1	Saliva_End	MRPL17
MAP4K4	0.007536	0.334954	0.124	0.111	1	Saliva_End	MAP4K4
LINC00657	0.007649	0.268108	0.16	0.205	1	Saliva_End	LINC00657
RNF13	0.007696	0.516524	0.173	0.167	1	Saliva_End	RNF13
INF2	0.007901	0.266942	0.06	0.048	1	Saliva_End	INF2
TNFRSF14	0.008117	0.309698	0.131	0.117	1	Saliva_End	TNFRSF14
PCSK5	0.008449	0.394348	0.044	0.034	1	Saliva_End	PCSK5
SUGT1	0.008631	0.413354	0.146	0.135	1	Saliva_End	SUGT1
TMEM14B	0.008743	0.276775	0.198	0.254	1	Saliva_End	TMEM14B
MKKS	0.008747	0.269089	0.125	0.161	1	Saliva_End	MKKS
CWF19L2	0.008859	0.319677	0.083	0.07	1	Saliva_End	CWF19L2
UQCRC2	0.0089	0.257324	0.165	0.21	1	Saliva_End	UQCRC2
SCGB3A1	0	3.822604	0.845	0.118	0	Saliva_Acir	SCGB3A1
SLPI1	0	3.402403	1	0.479	0	Saliva_Acir	SLPI
ZG16B1	0	3.253742	1	0.397	0	Saliva_Acir	ZG16B
LYZ1	0	3.050659	1	0.317	0	Saliva_Acir	LYZ
AZGP11	0	2.984439	1	0.181	0	Saliva_Acir	AZGP1
GNAS1	0	2.859819	1	0.57	0	Saliva_Acir	GNAS
TCN11	0	2.859737	0.995	0.13	0	Saliva_Acir	TCN1
C6orf581	0	2.640629	0.964	0.193	0	Saliva_Acir	C6orf58
CCL281	0	2.411585	0.981	0.084	0	Saliva_Acir	CCL28
PRR4	0	2.20984	0.65	0.094	0	Saliva_Acir	PRR4
RP11.1143	0	2.189563	0.971	0.206	0	Saliva_Acir	RP11.1143G9.4
FAM3D1	0	1.967034	0.985	0.216	0	Saliva_Acir	FAM3D
FAM46A1	0	1.87359	0.922	0.141	0	Saliva_Acir	FAM46A
S100A11	0	1.859788	0.917	0.078	0	Saliva_Acir	S100A1
KRT191	0	1.837156	0.919	0.098	0	Saliva_Acir	KRT19
WFDC2	0	1.778066	0.872	0.217	0	Saliva_Acir	WFDC2
ANKRD36C	0	1.749108	0.833	0.116	0	Saliva_Acir	ANKRD36C

KIAA13241	0	1.740656	0.94	0.083	0	Saliva_Acir	KIAA1324
LRRC261	0	1.72805	0.888	0.052	0	Saliva_Acir	LRRC26
XBP11	0	1.707846	0.987	0.322	0	Saliva_Acir	XBP1
PHB1	0	1.696885	0.95	0.219	0	Saliva_Acir	PHB
SSR41	0	1.616693	0.999	0.534	0	Saliva_Acir	SSR4
CLDN31	0	1.611814	0.877	0.074	0	Saliva_Acir	CLDN3
LGALS3BP1	0	1.610254	0.945	0.146	0	Saliva_Acir	LGALS3BP
FXYD31	0	1.522617	0.965	0.21	0	Saliva_Acir	FXYD3
RGCC1	0	1.503725	0.902	0.193	0	Saliva_Acir	RGCC
TMED31	0	1.47591	0.941	0.258	0	Saliva_Acir	TMED3
NUCB21	0	1.467474	0.95	0.248	0	Saliva_Acir	NUCB2
IFI61	0	1.410648	0.615	0.14	0	Saliva_Acir	IFI6
CLDN101	0	1.403707	0.745	0.075	0	Saliva_Acir	CLDN10
ELF51	0	1.363862	0.818	0.046	0	Saliva_Acir	ELF5
SPINT21	0	1.342826	0.964	0.293	0	Saliva_Acir	SPINT2
C8orf42	0	1.324454	0.786	0.099	0	Saliva_Acir	C8orf4
CLDN41	0	1.309059	0.647	0.072	0	Saliva_Acir	CLDN4
MCFD2	0	1.263991	0.856	0.172	0	Saliva_Acir	MCFD2
EPCAM1	0	1.261088	0.882	0.107	0	Saliva_Acir	EPCAM
P4HB	0	1.231443	0.975	0.401	0	Saliva_Acir	P4HB
MGLL2	0	1.185081	0.822	0.157	0	Saliva_Acir	MGLL
DBI1	0	1.15829	0.975	0.397	0	Saliva_Acir	DBI
NUPR1	0	1.144806	0.908	0.239	0	Saliva_Acir	NUPR1
SFRP1	0	1.140544	0.737	0.075	0	Saliva_Acir	SFRP1
TPD52L12	0	1.123515	0.82	0.114	0	Saliva_Acir	TPD52L1
OGFRL11	0	1.095006	0.795	0.093	0	Saliva_Acir	OGFRL1
KRT7	0	1.093126	0.885	0.253	0	Saliva_Acir	KRT7
PROM11	0	1.071354	0.715	0.062	0	Saliva_Acir	PROM1
NPDC12	0	1.071341	0.871	0.151	0	Saliva_Acir	NPDC1
PEBP1	0	1.054945	0.971	0.44	0	Saliva_Acir	PEBP1
CHPT11	0	1.034035	0.856	0.186	0	Saliva_Acir	CHPT1
SEC11C1	0	1.02756	0.947	0.22	0	Saliva_Acir	SEC11C
RARRES1	0	0.997993	0.261	0.034	0	Saliva_Acir	RARRES1
TMED101	0	0.99267	0.932	0.302	0	Saliva_Acir	TMED10
PHGDH	0	0.971859	0.746	0.095	0	Saliva_Acir	PHGDH
LPO1	0	0.958165	0.543	0.034	0	Saliva_Acir	LPO
GMDS1	0	0.953465	0.801	0.151	0	Saliva_Acir	GMDS
PRDX4	0	0.95239	0.896	0.236	0	Saliva_Acir	PRDX4
FAM107B	0	0.945545	0.852	0.281	0	Saliva_Acir	FAM107B
DCXR	0	0.943562	0.863	0.2	0	Saliva_Acir	DCXR
ITGA61	0	0.93842	0.779	0.115	0	Saliva_Acir	ITGA6
NDUFC2	0	0.91553	0.941	0.369	0	Saliva_Acir	NDUFC2
TM7SF2	0	0.912563	0.716	0.084	0	Saliva_Acir	TM7SF2
CD14	0	0.894124	0.691	0.088	0	Saliva_Acir	CD14
CA2	0	0.890157	0.659	0.08	0	Saliva_Acir	CA2
PART1	0	0.880721	0.694	0.052	0	Saliva_Acir	PART1
FOXC11	0	0.865167	0.69	0.094	0	Saliva_Acir	FOXC1

TPT11	0	0.862397	1	0.957	0	Saliva_Acir	TPT1
MT.ND32	0	0.858905	1	0.788	0	Saliva_Acir	MT.ND3
CHI3L2	0	0.858247	0.542	0.043	0	Saliva_Acir	CHI3L2
TMEM123	0	0.856752	0.893	0.263	0	Saliva_Acir	TMEM123
NDUFA4	0	0.852414	0.98	0.524	0	Saliva_Acir	NDUFA4
SAA2	0	0.85102	0.294	0.046	0	Saliva_Acir	SAA2
FKBP2	0	0.847411	0.932	0.333	0	Saliva_Acir	FKBP2
CEP411	0	0.845495	0.666	0.084	0	Saliva_Acir	CEP41
GOLGA4	0	0.841372	0.818	0.217	0	Saliva_Acir	GOLGA4
GJC3	0	0.83986	0.632	0.043	0	Saliva_Acir	GJC3
HDLBP	0	0.835045	0.859	0.242	0	Saliva_Acir	HDLBP
LRPAP11	0	0.824352	0.852	0.251	0	Saliva_Acir	LRPAP1
KRT8	0	0.794769	0.829	0.18	0	Saliva_Acir	KRT8
TESC2	0	0.792511	0.635	0.097	0	Saliva_Acir	TESC
RP11.532F	0	0.792076	0.673	0.061	0	Saliva_Acir	RP11.532F12.5
MANSC1	0	0.784358	0.651	0.063	0	Saliva_Acir	MANSC1
PPP1R16A:	0	0.78018	0.682	0.085	0	Saliva_Acir	PPP1R16A
METRN	0	0.77941	0.679	0.075	0	Saliva_Acir	METRN
RHOBTB3	0	0.776885	0.695	0.1	0	Saliva_Acir	RHOBTB3
SH3BP41	0	0.771474	0.63	0.064	0	Saliva_Acir	SH3BP4
PTPRF1	0	0.771091	0.659	0.089	0	Saliva_Acir	PTPRF
REEP51	0	0.767987	0.903	0.29	0	Saliva_Acir	REEP5
RPS171	0	0.767212	0.938	0.2	0	Saliva_Acir	RPS17
KRT18	0	0.767019	0.948	0.283	0	Saliva_Acir	KRT18
FGGY	0	0.761841	0.621	0.068	0	Saliva_Acir	FGGY
KRTCAP3	0	0.758748	0.635	0.063	0	Saliva_Acir	KRTCAP3
YIPF21	0	0.756503	0.682	0.09	0	Saliva_Acir	YIPF2
EIF4EBP1	0	0.750822	0.737	0.132	0	Saliva_Acir	EIF4EBP1
SEC61B	0	0.744285	0.952	0.444	0	Saliva_Acir	SEC61B
NFIB1	0	0.732156	0.8	0.172	0	Saliva_Acir	NFIB
MT.ATP62	0	0.73083	1	0.895	0	Saliva_Acir	MT.ATP6
ACTR3B1	0	0.72807	0.597	0.051	0	Saliva_Acir	ACTR3B
SLITRK6	0	0.723914	0.548	0.044	0	Saliva_Acir	SLITRK6
SLC31A2	0	0.718195	0.657	0.093	0	Saliva_Acir	SLC31A2
TSPAN131	0	0.715605	0.743	0.139	0	Saliva_Acir	TSPAN13
LAMP21	0	0.710114	0.825	0.217	0	Saliva_Acir	LAMP2
MGST31	0	0.709377	0.945	0.316	0	Saliva_Acir	MGST3
MT.CYB2	0	0.705525	1	0.932	0	Saliva_Acir	MT.CYB
EHF	0	0.692241	0.844	0.225	0	Saliva_Acir	EHF
TTC3	0	0.689768	0.822	0.215	0	Saliva_Acir	TTC3
NCALD	0	0.68806	0.586	0.053	0	Saliva_Acir	NCALD
CD811	0	0.677202	0.957	0.303	0	Saliva_Acir	CD81
CD631	0	0.675801	0.992	0.613	0	Saliva_Acir	CD63
FAM114A1	0	0.674631	0.681	0.105	0	Saliva_Acir	FAM114A1
TSTD1	0	0.671994	0.876	0.253	0	Saliva_Acir	TSTD1
SPCS3	0	0.665062	0.836	0.232	0	Saliva_Acir	SPCS3
LINC00342	0	0.658612	0.501	0.04	0	Saliva_Acir	LINC00342

SLC39A8	0	0.657554	0.637	0.077	0	Saliva_Acir	SLC39A8
ALDH9A1	0	0.655768	0.708	0.131	0	Saliva_Acir	ALDH9A1
SERP1	0	0.648227	0.96	0.534	0	Saliva_Acir	SERP1
EPRS1	0	0.647665	0.687	0.115	0	Saliva_Acir	EPRS
SELM1	0	0.644093	0.945	0.328	0	Saliva_Acir	SELM
ATP1A11	0	0.641857	0.84	0.237	0	Saliva_Acir	ATP1A1
TMBIM6	0	0.640511	0.988	0.566	0	Saliva_Acir	TMBIM6
ANG	0	0.638825	0.647	0.083	0	Saliva_Acir	ANG
PDIA6	0	0.633669	0.877	0.26	0	Saliva_Acir	PDIA6
MEIS2	0	0.633587	0.6	0.088	0	Saliva_Acir	MEIS2
GOLGB1	0	0.630359	0.724	0.183	0	Saliva_Acir	GOLGB1
PYCR11	0	0.625088	0.601	0.064	0	Saliva_Acir	PYCR1
RAB4A	0	0.623685	0.741	0.191	0	Saliva_Acir	RAB4A
NDUFB81	0	0.618365	0.747	0.16	0	Saliva_Acir	NDUFB8
CCDC151	0	0.614004	0.511	0.046	0	Saliva_Acir	CCDC15
MDK	0	0.613502	0.69	0.132	0	Saliva_Acir	MDK
PLIN5	0	0.61014	0.52	0.039	0	Saliva_Acir	PLIN5
PPIB	0	0.60726	0.93	0.377	0	Saliva_Acir	PPIB
BARX2	0	0.605795	0.509	0.049	0	Saliva_Acir	BARX2
MPZL1	0	0.604865	0.664	0.124	0	Saliva_Acir	MPZL1
ARG2	0	0.599846	0.536	0.053	0	Saliva_Acir	ARG2
CADM1	0	0.593858	0.606	0.088	0	Saliva_Acir	CADM1
KCNN4	0	0.593382	0.605	0.065	0	Saliva_Acir	KCNN4
MYBPC1	0	0.591591	0.33	0.009	0	Saliva_Acir	MYBPC1
TCEA3	0	0.59045	0.623	0.084	0	Saliva_Acir	TCEA3
GRN	0	0.585398	0.892	0.294	0	Saliva_Acir	GRN
BACE21	0	0.579417	0.681	0.146	0	Saliva_Acir	BACE2
TPT1.AS11	0	0.575702	0.364	0.084	0	Saliva_Acir	TPT1.AS1
CHID1	0	0.573463	0.716	0.153	0	Saliva_Acir	CHID1
CNPY2	0	0.572029	0.773	0.188	0	Saliva_Acir	CNPY2
GK51	0	0.571315	0.535	0.059	0	Saliva_Acir	GK5
TFCP2L1	0	0.569965	0.383	0.037	0	Saliva_Acir	TFCP2L1
PLA2R1	0	0.569858	0.479	0.042	0	Saliva_Acir	PLA2R1
TOMM201	0	0.561486	0.864	0.309	0	Saliva_Acir	TOMM20
OAT	0	0.555949	0.712	0.154	0	Saliva_Acir	OAT
PHPT1	0	0.550129	0.881	0.307	0	Saliva_Acir	PHPT1
JTB	0	0.549697	0.908	0.354	0	Saliva_Acir	JTB
KDELR1	0	0.548596	0.803	0.234	0	Saliva_Acir	KDELR1
TMEM63A	0	0.548512	0.53	0.066	0	Saliva_Acir	TMEM63A
SLC12A2	0	0.547535	0.543	0.099	0	Saliva_Acir	SLC12A2
APIP	0	0.547459	0.538	0.083	0	Saliva_Acir	APIP
NANS	0	0.54657	0.753	0.214	0	Saliva_Acir	NANS
INSR	0	0.542654	0.567	0.116	0	Saliva_Acir	INSR
TMEM125	0	0.539847	0.486	0.035	0	Saliva_Acir	TMEM125
DHRS7	0	0.539839	0.771	0.191	0	Saliva_Acir	DHRS7
ITPR21	0	0.539306	0.541	0.066	0	Saliva_Acir	ITPR2
CCPG11	0	0.53564	0.714	0.151	0	Saliva_Acir	CCPG1

DSC2	0	0.533078	0.494	0.055	0	Saliva_Acir	DSC2
CPD	0	0.53222	0.622	0.105	0	Saliva_Acir	CPD
SEC61A1	0	0.524856	0.667	0.136	0	Saliva_Acir	SEC61A1
CHCHD10	0	0.521198	0.826	0.227	0	Saliva_Acir	CHCHD10
IRX3	0	0.518897	0.505	0.059	0	Saliva_Acir	IRX3
BNIP3	0	0.518791	0.659	0.134	0	Saliva_Acir	BNIP3
LIMCH11	0	0.518195	0.588	0.113	0	Saliva_Acir	LIMCH1
OS9	0	0.517509	0.769	0.209	0	Saliva_Acir	OS9
AKR1A1	0	0.513442	0.751	0.187	0	Saliva_Acir	AKR1A1
CD591	0	0.510862	0.984	0.501	0	Saliva_Acir	CD59
SOX2	0	0.506916	0.439	0.033	0	Saliva_Acir	SOX2
BSPRY	0	0.503942	0.449	0.038	0	Saliva_Acir	BSPRY
TMEM87A	0	0.502478	0.664	0.156	0	Saliva_Acir	TMEM87A
ZNF652	0	0.501959	0.546	0.081	0	Saliva_Acir	ZNF652
PGM3	0	0.497217	0.537	0.078	0	Saliva_Acir	PGM3
KRT23	0	0.49351	0.736	0.173	0	Saliva_Acir	KRT23
MLEC1	0	0.492393	0.708	0.172	0	Saliva_Acir	MLEC
KDELR2	0	0.491522	0.858	0.303	0	Saliva_Acir	KDELR2
LMO4	0	0.490161	0.661	0.149	0	Saliva_Acir	LMO4
ARL1	0	0.483496	0.717	0.19	0	Saliva_Acir	ARL1
LMAN11	0	0.481215	0.732	0.177	0	Saliva_Acir	LMAN1
FARP1	0	0.480051	0.524	0.086	0	Saliva_Acir	FARP1
ATRAID	0	0.479069	0.833	0.274	0	Saliva_Acir	ATRAID
PCYOX1	0	0.477626	0.616	0.116	0	Saliva_Acir	PCYOX1
TSHZ21	0	0.476711	0.603	0.094	0	Saliva_Acir	TSHZ2
MT.ND42	0	0.475875	1	0.952	0	Saliva_Acir	MT.ND4
ANXA3	0	0.475782	0.631	0.14	0	Saliva_Acir	ANXA3
CALR	0	0.474258	0.927	0.408	0	Saliva_Acir	CALR
CLSTN1	0	0.473704	0.573	0.108	0	Saliva_Acir	CLSTN1
TMEM205	0	0.473028	0.753	0.187	0	Saliva_Acir	TMEM205
DHCR24	0	0.472719	0.511	0.086	0	Saliva_Acir	DHCR24
RDH11	0	0.472399	0.583	0.096	0	Saliva_Acir	RDH11
C1orf122	0	0.471913	0.693	0.168	0	Saliva_Acir	C1orf122
FAHD2A	0	0.471076	0.475	0.075	0	Saliva_Acir	FAHD2A
HPSE	0	0.469554	0.468	0.051	0	Saliva_Acir	HPSE
DPP7	0	0.466736	0.841	0.241	0	Saliva_Acir	DPP7
DAP	0	0.465933	0.621	0.129	0	Saliva_Acir	DAP
SELENBP11	0	0.461591	0.526	0.061	0	Saliva_Acir	SELENBP1
PKDCC	0	0.458965	0.451	0.04	0	Saliva_Acir	PKDCC
TMED4	0	0.458816	0.697	0.157	0	Saliva_Acir	TMED4
CYB561	0	0.458736	0.53	0.074	0	Saliva_Acir	CYB561
SSR3	0	0.458686	0.837	0.284	0	Saliva_Acir	SSR3
INADL	0	0.457333	0.471	0.075	0	Saliva_Acir	INADL
CANT1	0	0.450481	0.557	0.095	0	Saliva_Acir	CANT1
ANK3	0	0.449189	0.474	0.059	0	Saliva_Acir	ANK3
VKORC1	0	0.445043	0.749	0.208	0	Saliva_Acir	VKORC1
ARHGAP18	0	0.444433	0.496	0.083	0	Saliva_Acir	ARHGAP18

MZT2B	0	0.443981	0.919	0.447	0	Saliva_Acir	MZT2B
DNAJC3	0	0.443653	0.713	0.192	0	Saliva_Acir	DNAJC3
MT.CO22	0	0.441283	1	0.958	0	Saliva_Acir	MT.CO2
SCARB2	0	0.439822	0.639	0.142	0	Saliva_Acir	SCARB2
CKAP4	0	0.438632	0.664	0.15	0	Saliva_Acir	CKAP4
TNFRSF19	0	0.4373	0.447	0.054	0	Saliva_Acir	TNFRSF19
TIMP1	0	0.435868	0.921	0.365	0	Saliva_Acir	TIMP1
ETFB	0	0.433297	0.79	0.256	0	Saliva_Acir	ETFB
SMIM11	0	0.432857	0.486	0.057	0	Saliva_Acir	SMIM1
RPS27L	0	0.432444	0.928	0.43	0	Saliva_Acir	RPS27L
RRAGD	0	0.430193	0.494	0.08	0	Saliva_Acir	RRAGD
WIPI1	0	0.429058	0.525	0.085	0	Saliva_Acir	WIPI1
HOOK2	0	0.42845	0.517	0.09	0	Saliva_Acir	HOOK2
CTSZ1	0	0.428149	0.761	0.174	0	Saliva_Acir	CTSZ
ARFGAP3	0	0.427347	0.625	0.144	0	Saliva_Acir	ARFGAP3
FNDC3A	0	0.427254	0.553	0.103	0	Saliva_Acir	FNDC3A
MESP1	0	0.424435	0.361	0.045	0	Saliva_Acir	MESP1
KIF21A	0	0.421406	0.476	0.069	0	Saliva_Acir	KIF21A
PKP2	0	0.420308	0.384	0.04	0	Saliva_Acir	PKP2
CANX1	0	0.418878	0.82	0.296	0	Saliva_Acir	CANX
IVNS1ABP	0	0.418563	0.585	0.136	0	Saliva_Acir	IVNS1ABP
SLC31A1	0	0.416063	0.489	0.067	0	Saliva_Acir	SLC31A1
PRKACB	0	0.414661	0.515	0.088	0	Saliva_Acir	PRKACB
SMCO4	0	0.414541	0.553	0.105	0	Saliva_Acir	SMCO4
HIPK2	0	0.414188	0.448	0.068	0	Saliva_Acir	HIPK2
PAPSS1	0	0.413893	0.533	0.096	0	Saliva_Acir	PAPSS1
SPCS1	0	0.412053	0.935	0.447	0	Saliva_Acir	SPCS1
MT.ND52	0	0.409647	0.997	0.693	0	Saliva_Acir	MT.ND5
CXCL51	0	0.409439	0.289	0.021	0	Saliva_Acir	CXCL5
GMPPB	0	0.40854	0.455	0.053	0	Saliva_Acir	GMPPB
KDELR3	0	0.405741	0.52	0.072	0	Saliva_Acir	KDELR3
PDCD4	0	0.404981	0.68	0.169	0	Saliva_Acir	PDCD4
CTDSPL	0	0.404809	0.487	0.072	0	Saliva_Acir	CTDSPL
PAIP2B	0	0.402042	0.446	0.05	0	Saliva_Acir	PAIP2B
SEC63	0	0.401598	0.637	0.157	0	Saliva_Acir	SEC63
ATOX1	0	0.401252	0.787	0.258	0	Saliva_Acir	ATOX1
COPB2	0	0.401221	0.612	0.141	0	Saliva_Acir	COPB2
YIPF3	0	0.401096	0.72	0.218	0	Saliva_Acir	YIPF3
TPD52	0	0.397317	0.583	0.113	0	Saliva_Acir	TPD52
MGST11	0	0.397207	0.737	0.177	0	Saliva_Acir	MGST1
NR2F6	0	0.39146	0.494	0.07	0	Saliva_Acir	NR2F6
HID1	0	0.389969	0.408	0.035	0	Saliva_Acir	HID1
SYNGR1	0	0.389492	0.562	0.129	0	Saliva_Acir	SYNGR1
VAMP8	0	0.387188	0.929	0.393	0	Saliva_Acir	VAMP8
TCEAL2	0	0.385052	0.359	0.03	0	Saliva_Acir	TCEAL2
VIMP	0	0.383878	0.817	0.275	0	Saliva_Acir	VIMP
PPP1R9A	0	0.383823	0.357	0.041	0	Saliva_Acir	PPP1R9A

PAM	0	0.382258	0.623	0.127	0	Saliva_Acir	PAM
NENF	0	0.382184	0.832	0.313	0	Saliva_Acir	NENF
PPP1R14B1	0	0.382072	0.652	0.163	0	Saliva_Acir	PPP1R14B
DPM3	0	0.381421	0.649	0.166	0	Saliva_Acir	DPM3
ACSL3	0	0.379851	0.578	0.146	0	Saliva_Acir	ACSL3
FMO5	0	0.379522	0.375	0.029	0	Saliva_Acir	FMO5
TNFSF15	0	0.377184	0.204	0.01	0	Saliva_Acir	TNFSF15
DEPTOR	0	0.376625	0.381	0.047	0	Saliva_Acir	DEPTOR
ASCL2	0	0.376504	0.421	0.04	0	Saliva_Acir	ASCL2
GFPT1	0	0.375426	0.562	0.114	0	Saliva_Acir	GFPT1
GNPTAB	0	0.375381	0.467	0.077	0	Saliva_Acir	GNPTAB
ELL2	0	0.375317	0.555	0.112	0	Saliva_Acir	ELL2
RABAC11	0	0.372871	0.912	0.389	0	Saliva_Acir	RABAC1
GPT2	0	0.372008	0.466	0.072	0	Saliva_Acir	GPT2
NME4	0	0.371639	0.605	0.128	0	Saliva_Acir	NME4
CREB3L1	0	0.370822	0.473	0.062	0	Saliva_Acir	CREB3L1
DUSP6	0	0.369937	0.54	0.115	0	Saliva_Acir	DUSP6
SLC37A2	0	0.368941	0.342	0.028	0	Saliva_Acir	SLC37A2
RAD51B	0	0.368196	0.352	0.032	0	Saliva_Acir	RAD51B
SEZ6L2	0	0.367794	0.359	0.036	0	Saliva_Acir	SEZ6L2
GTF2I1	0	0.367347	0.596	0.14	0	Saliva_Acir	GTF2I
MT.ND4L2	0	0.365893	0.947	0.4	0	Saliva_Acir	MT.ND4L
HOMER2	0	0.364229	0.374	0.04	0	Saliva_Acir	HOMER2
MXD4	0	0.363549	0.569	0.106	0	Saliva_Acir	MXD4
PARK71	0	0.363067	0.89	0.339	0	Saliva_Acir	PARK7
VEGFB	0	0.362064	0.541	0.086	0	Saliva_Acir	VEGFB
TECR	0	0.362064	0.844	0.314	0	Saliva_Acir	TECR
CTSF	0	0.360739	0.62	0.143	0	Saliva_Acir	CTSF
CLPTM1L	0	0.360406	0.52	0.079	0	Saliva_Acir	CLPTM1L
TRIP111	0	0.358997	0.526	0.108	0	Saliva_Acir	TRIP11
NAALADL2	0	0.358878	0.32	0.041	0	Saliva_Acir	NAALADL2
TSPAN33	0	0.358271	0.389	0.034	0	Saliva_Acir	TSPAN33
SMIM14	0	0.356129	0.727	0.235	0	Saliva_Acir	SMIM14
FKBP11	0	0.356002	0.716	0.136	0	Saliva_Acir	FKBP11
SLC39A7	0	0.355267	0.525	0.101	0	Saliva_Acir	SLC39A7
RAB25	0	0.355	0.539	0.121	0	Saliva_Acir	RAB25
ADI1	0	0.351603	0.705	0.206	0	Saliva_Acir	ADI1
RPN2	0	0.349504	0.768	0.23	0	Saliva_Acir	RPN2
SURF4	0	0.349213	0.632	0.163	0	Saliva_Acir	SURF4
ZKSCAN1	0	0.34895	0.497	0.105	0	Saliva_Acir	ZKSCAN1
RAB3D	0	0.34873	0.397	0.046	0	Saliva_Acir	RAB3D
GGT6	0	0.347059	0.348	0.03	0	Saliva_Acir	GGT6
SORBS21	0	0.346687	0.517	0.1	0	Saliva_Acir	SORBS2
ANAPC5	0	0.346297	0.638	0.146	0	Saliva_Acir	ANAPC5
CHPF	0	0.342766	0.479	0.071	0	Saliva_Acir	CHPF
TC2N	0	0.34259	0.597	0.14	0	Saliva_Acir	TC2N
PKP41	0	0.341558	0.418	0.067	0	Saliva_Acir	PKP4

PBX1	0	0.341104	0.497	0.103	0	Saliva_Acir	PBX1
TM9SF2	0	0.33989	0.635	0.181	0	Saliva_Acir	TM9SF2
AGFG2	0	0.339121	0.563	0.124	0	Saliva_Acir	AGFG2
ALG5	0	0.336088	0.614	0.14	0	Saliva_Acir	ALG5
INPP5D	0	0.335969	0.437	0.048	0	Saliva_Acir	INPP5D
CD1511	0	0.333398	0.751	0.204	0	Saliva_Acir	CD151
NBEA	0	0.332538	0.32	0.039	0	Saliva_Acir	NBEA
DUS1L	0	0.328012	0.493	0.085	0	Saliva_Acir	DUS1L
RRBP1	0	0.327832	0.802	0.296	0	Saliva_Acir	RRBP1
CGREF1	0	0.325748	0.377	0.038	0	Saliva_Acir	CGREF1
AC159540.	0	0.324795	0.297	0.055	0	Saliva_Acir	AC159540.1
FOLH1	0	0.322355	0.335	0.025	0	Saliva_Acir	FOLH1
PITPNA	0	0.322072	0.47	0.09	0	Saliva_Acir	PITPNA
SSFA2	0	0.321403	0.576	0.155	0	Saliva_Acir	SSFA2
MAGED1	0	0.320979	0.457	0.074	0	Saliva_Acir	MAGED1
TMEM56	0	0.31976	0.333	0.033	0	Saliva_Acir	TMEM56
FGFR2	0	0.319355	0.308	0.029	0	Saliva_Acir	FGFR2
TNFRSF11/	0	0.319333	0.397	0.046	0	Saliva_Acir	TNFRSF11A
HM13	0	0.318251	0.68	0.189	0	Saliva_Acir	HM13
WWC1	0	0.318089	0.433	0.078	0	Saliva_Acir	WWC1
NAGLU	0	0.317955	0.431	0.058	0	Saliva_Acir	NAGLU
CTBS	0	0.317207	0.489	0.091	0	Saliva_Acir	CTBS
SPTLC21	0	0.316614	0.427	0.07	0	Saliva_Acir	SPTLC2
SLC26A2	0	0.315263	0.445	0.066	0	Saliva_Acir	SLC26A2
MTURN	0	0.315255	0.453	0.068	0	Saliva_Acir	MTURN
XAB2	0	0.314998	0.472	0.084	0	Saliva_Acir	XAB2
CDC42EP5	0	0.31482	0.743	0.18	0	Saliva_Acir	CDC42EP5
SNRPN	0	0.313499	0.489	0.094	0	Saliva_Acir	SNRPN
KCNQ1OT1	0	0.31337	0.509	0.13	0	Saliva_Acir	KCNQ1OT1
BEX4	0	0.312985	0.624	0.156	0	Saliva_Acir	BEX4
SLC39A6	0	0.31267	0.46	0.087	0	Saliva_Acir	SLC39A6
GATM	0	0.312286	0.401	0.067	0	Saliva_Acir	GATM
SPDEF	0	0.311435	0.467	0.051	0	Saliva_Acir	SPDEF
AKT1	0	0.311107	0.455	0.077	0	Saliva_Acir	AKT1
NFIC1	0	0.310577	0.632	0.157	0	Saliva_Acir	NFIC
CCDC71L	0	0.307513	0.346	0.057	0	Saliva_Acir	CCDC71L
FUCA1	0	0.306891	0.538	0.127	0	Saliva_Acir	FUCA1
SREBF1	0	0.304678	0.444	0.081	0	Saliva_Acir	SREBF1
TMEM45A	0	0.303509	0.503	0.088	0	Saliva_Acir	TMEM45A
GBAS	0	0.302794	0.523	0.113	0	Saliva_Acir	GBAS
MAPK13	0	0.302466	0.492	0.098	0	Saliva_Acir	MAPK13
NDUFB10	0	0.301151	0.879	0.369	0	Saliva_Acir	NDUFB10
SLC35E1	0	0.300426	0.395	0.065	0	Saliva_Acir	SLC35E1
CMTM81	0	0.298684	0.404	0.059	0	Saliva_Acir	CMTM8
KCNK6	0	0.29753	0.465	0.09	0	Saliva_Acir	KCNK6
SSR1	0	0.29599	0.638	0.177	0	Saliva_Acir	SSR1
SNHG18	0	0.295794	0.395	0.052	0	Saliva_Acir	SNHG18

NFIX	0	0.295744	0.485	0.098	0	Saliva_Acir	NFIX
ATP6V0B	0	0.29444	0.874	0.372	0	Saliva_Acir	ATP6V0B
IBTK	0	0.29373	0.443	0.082	0	Saliva_Acir	IBTK
LHFPL2	0	0.292951	0.353	0.044	0	Saliva_Acir	LHFPL2
RNF130	0	0.292902	0.572	0.128	0	Saliva_Acir	RNF130
PODXL1	0	0.292775	0.372	0.04	0	Saliva_Acir	PODXL
PXMP4	0	0.292726	0.348	0.04	0	Saliva_Acir	PXMP4
RHCE	0	0.292507	0.304	0.017	0	Saliva_Acir	RHCE
HEXB	0	0.291829	0.615	0.147	0	Saliva_Acir	HEXB
CES3	0	0.290731	0.299	0.021	0	Saliva_Acir	CES3
FBP1	0	0.288617	0.575	0.116	0	Saliva_Acir	FBP1
ATP6AP1	0	0.288475	0.578	0.148	0	Saliva_Acir	ATP6AP1
COPG1	0	0.286755	0.446	0.084	0	Saliva_Acir	COPG1
ERBB2	0	0.285535	0.325	0.051	0	Saliva_Acir	ERBB2
CERS4	0	0.285382	0.389	0.058	0	Saliva_Acir	CERS4
CHKA	0	0.285335	0.42	0.083	0	Saliva_Acir	CHKA
ARHGEF28	0	0.284707	0.331	0.046	0	Saliva_Acir	ARHGEF28
SGSM3	0	0.283949	0.5	0.1	0	Saliva_Acir	SGSM3
BRI31	0	0.283161	0.843	0.331	0	Saliva_Acir	BRI3
AACS	0	0.283018	0.345	0.055	0	Saliva_Acir	AACS
CADPS21	0	0.281214	0.327	0.036	0	Saliva_Acir	CADPS2
RP11.379B	0	0.279988	0.294	0.022	0	Saliva_Acir	RP11.379B8.1
TBC1D24	0	0.279754	0.324	0.031	0	Saliva_Acir	TBC1D24
PDHA1	0	0.278697	0.455	0.094	0	Saliva_Acir	PDHA1
SHROOM3	0	0.277566	0.293	0.043	0	Saliva_Acir	SHROOM3
PDIA3	0	0.277446	0.831	0.307	0	Saliva_Acir	PDIA3
ECI1	0	0.277279	0.534	0.126	0	Saliva_Acir	ECI1
NDUFB7	0	0.277174	0.875	0.357	0	Saliva_Acir	NDUFB7
PRKAR2B	0	0.27593	0.322	0.04	0	Saliva_Acir	PRKAR2B
DAD11	0	0.274632	0.879	0.384	0	Saliva_Acir	DAD1
MRPL20	0	0.273472	0.777	0.255	0	Saliva_Acir	MRPL20
FAM171B	0	0.273185	0.304	0.028	0	Saliva_Acir	FAM171B
ZNF704	0	0.272873	0.372	0.06	0	Saliva_Acir	ZNF704
TRAK1	0	0.272696	0.356	0.057	0	Saliva_Acir	TRAK1
ECHDC1	0	0.27254	0.474	0.106	0	Saliva_Acir	ECHDC1
C8orf591	0	0.271837	0.69	0.221	0	Saliva_Acir	C8orf59
CRNDE	0	0.271347	0.625	0.141	0	Saliva_Acir	CRNDE
TMC4	0	0.271293	0.392	0.071	0	Saliva_Acir	TMC4
ALDH18A1	0	0.27078	0.355	0.048	0	Saliva_Acir	ALDH18A1
ANKRD9	0	0.270543	0.398	0.085	0	Saliva_Acir	ANKRD9
SLC38A1	0	0.268291	0.469	0.088	0	Saliva_Acir	SLC38A1
CREG1	0	0.268192	0.532	0.133	0	Saliva_Acir	CREG1
CMYA5	0	0.266135	0.285	0.026	0	Saliva_Acir	CMYA5
TUBA1C	0	0.265164	0.496	0.132	0	Saliva_Acir	TUBA1C
PLTP	0	0.264764	0.432	0.084	0	Saliva_Acir	PLTP
QDPR	0	0.264441	0.453	0.083	0	Saliva_Acir	QDPR
ERLEC1	0	0.263827	0.608	0.169	0	Saliva_Acir	ERLEC1

RANBP17	0	0.263288	0.298	0.038	0	Saliva_Acir	RANBP17
PHKB1	0	0.263231	0.457	0.095	0	Saliva_Acir	PHKB
LRRC59	0	0.263058	0.539	0.132	0	Saliva_Acir	LRRC59
SUCLG1	0	0.262883	0.604	0.164	0	Saliva_Acir	SUCLG1
GPAA1	0	0.261751	0.565	0.13	0	Saliva_Acir	GPAA1
LXN1	0	0.261655	0.266	0.05	0	Saliva_Acir	LXN
ARFIP2	0	0.261643	0.465	0.095	0	Saliva_Acir	ARFIP2
SDF2L1	0	0.26094	0.741	0.214	0	Saliva_Acir	SDF2L1
SLC1A5	0	0.260162	0.402	0.067	0	Saliva_Acir	SLC1A5
COBL	0	0.260102	0.321	0.042	0	Saliva_Acir	COBL
CREB3L4	0	0.259263	0.367	0.048	0	Saliva_Acir	CREB3L4
FRMD4B1	0	0.259071	0.48	0.116	0	Saliva_Acir	FRMD4B
PRDX2	0	0.258768	0.836	0.295	0	Saliva_Acir	PRDX2
CLCN3	0	0.257598	0.477	0.119	0	Saliva_Acir	CLCN3
CDH1	0	0.257566	0.36	0.066	0	Saliva_Acir	CDH1
DMD	0	0.257526	0.323	0.043	0	Saliva_Acir	DMD
TPTEP1	0	0.257415	0.36	0.065	0	Saliva_Acir	TPTEP1
FAM84B1	0	0.254763	0.405	0.072	0	Saliva_Acir	FAM84B
MRPS26	0	0.253806	0.586	0.136	0	Saliva_Acir	MRPS26
FAM210B1	0	0.252307	0.457	0.082	0	Saliva_Acir	FAM210B
MACROD1	0	0.251991	0.379	0.061	0	Saliva_Acir	MACROD1
SLC17A5	0	0.251952	0.379	0.074	0	Saliva_Acir	SLC17A5
ERGIC1	0	0.251601	0.509	0.122	0	Saliva_Acir	ERGIC1
PURA1	0	0.251302	0.481	0.114	0	Saliva_Acir	PURA
PDE3B	0	0.251135	0.31	0.034	0	Saliva_Acir	PDE3B
FAM129A	0	0.250575	0.417	0.094	0	Saliva_Acir	FAM129A
TMEM214	0	0.250441	0.388	0.063	0	Saliva_Acir	TMEM214
TMEM258	5E-298	0.392898	0.905	0.439	8E-294	Saliva_Acir	TMEM258
H3F3A1	4.2E-286	0.451803	0.988	0.463	6.8E-282	Saliva_Acir	H3F3A
MT.ND12	9.5E-285	0.372988	0.999	0.925	1.5E-280	Saliva_Acir	MT.ND1
MT.ND22	9.8E-285	0.280582	0.999	0.904	1.6E-280	Saliva_Acir	MT.ND2
MT.CO12	2.7E-280	0.360481	1	0.984	4.3E-276	Saliva_Acir	MT.CO1
UQCRQ	3E-239	0.414953	0.909	0.487	4.7E-235	Saliva_Acir	UQCRQ
ITM2B1	1.8E-234	0.411593	0.993	0.697	2.9E-230	Saliva_Acir	ITM2B
BTG21	2E-202	0.561573	0.491	0.225	3.1E-198	Saliva_Acir	BTG2
CALM11	1.8E-181	0.404639	0.977	0.641	2.9E-177	Saliva_Acir	CALM1
RPL39	2.9E-179	0.33125	0.997	0.667	4.6E-175	Saliva_Acir	RPL39
TMEM59	7.5E-179	0.300177	0.974	0.598	1.2E-174	Saliva_Acir	TMEM59
FOSB1	8.2E-130	0.388339	0.461	0.245	1.3E-125	Saliva_Acir	FOSB
RPL7A1	4.1E-121	0.294365	0.999	0.808	6.6E-117	Saliva_Acir	RPL7A
ATF3	8.42E-49	0.324707	0.372	0.241	1.34E-44	Saliva_Acir	ATF3
MZB1	0	5.526792	0.969	0.025	0	Saliva_B	MZB1
DERL3	0	4.55768	0.89	0.047	0	Saliva_B	DERL3
SEC11C2	0	4.284387	0.964	0.227	0	Saliva_B	SEC11C
SSR42	0	4.030365	0.994	0.539	0	Saliva_B	SSR4
HSP90B1	0	3.733097	0.959	0.385	0	Saliva_B	HSP90B1
HERPUD1	0	3.663997	0.92	0.321	0	Saliva_B	HERPUD1

FKBP111	0	3.589215	0.875	0.139	0	Saliva_B	FKBP11
TNFRSF17	0	3.536537	0.784	0.005	0	Saliva_B	TNFRSF17
ITM2C	0	3.226426	0.787	0.107	0	Saliva_B	ITM2C
PRDX41	0	2.997009	0.882	0.243	0	Saliva_B	PRDX4
SPCS11	0	2.981198	0.945	0.452	0	Saliva_B	SPCS1
FKBP21	0	2.926242	0.908	0.34	0	Saliva_B	FKBP2
CYBA	0	2.893786	0.971	0.43	0	Saliva_B	CYBA
SPCS2	0	2.799578	0.901	0.397	0	Saliva_B	SPCS2
SDF2L11	0	2.798534	0.834	0.218	0	Saliva_B	SDF2L1
MANF	0	2.759846	0.81	0.222	0	Saliva_B	MANF
SSR31	0	2.718076	0.86	0.29	0	Saliva_B	SSR3
CD79A	0	2.654729	0.686	0.035	0	Saliva_B	CD79A
SPCS31	0	2.5449	0.793	0.24	0	Saliva_B	SPCS3
SUB1	0	2.483617	0.896	0.472	0	Saliva_B	SUB1
PDIA61	0	2.443532	0.816	0.268	0	Saliva_B	PDIA6
SEC61B1	0	2.41976	0.914	0.45	0	Saliva_B	SEC61B
VIMP1	0	2.412407	0.826	0.281	0	Saliva_B	VIMP
VIM1	0	2.385008	0.965	0.485	0	Saliva_B	VIM
XBP12	0	2.35402	0.876	0.332	0	Saliva_B	XBP1
CD27	0	2.351509	0.686	0.074	0	Saliva_B	CD27
PPIB1	0	2.34907	0.89	0.384	0	Saliva_B	PPIB
UBE2J1	0	2.261489	0.715	0.193	0	Saliva_B	UBE2J1
RABAC12	0	2.249435	0.878	0.395	0	Saliva_B	RABAC1
TXNDC15	0	2.155257	0.6	0.113	0	Saliva_B	TXNDC15
CRELD2	0	2.128633	0.634	0.14	0	Saliva_B	CRELD2
BTG22	0	2.09991	0.616	0.225	0	Saliva_B	BTG2
DDOST	0	2.06813	0.645	0.186	0	Saliva_B	DDOST
LMAN12	0	2.060932	0.643	0.185	0	Saliva_B	LMAN1
JSRP1	0	2.028461	0.379	0.007	0	Saliva_B	JSRP1
CLPTM1L1	0	2.003043	0.52	0.083	0	Saliva_B	CLPTM1L
EAF2	0	1.93846	0.508	0.04	0	Saliva_B	EAF2
FAM46C	0	1.93311	0.566	0.077	0	Saliva_B	FAM46C
ERLEC11	0	1.915181	0.569	0.174	0	Saliva_B	ERLEC1
TMEM208	0	1.907469	0.616	0.188	0	Saliva_B	TMEM208
CALR1	0	1.89973	0.817	0.416	0	Saliva_B	CALR
TMEM258	0	1.884339	0.829	0.445	0	Saliva_B	TMEM258
TMED102	0	1.855527	0.737	0.313	0	Saliva_B	TMED10
SEL1L	0	1.838471	0.483	0.085	0	Saliva_B	SEL1L
REEP52	0	1.832279	0.759	0.3	0	Saliva_B	REEP5
RPN21	0	1.810206	0.652	0.238	0	Saliva_B	RPN2
SLAMF7	0	1.794378	0.429	0.01	0	Saliva_B	SLAMF7
ALG51	0	1.790469	0.55	0.146	0	Saliva_B	ALG5
MEI1	0	1.78557	0.462	0.033	0	Saliva_B	MEI1
CCPG12	0	1.761603	0.562	0.16	0	Saliva_B	CCPG1
NPC2	0	1.747416	0.757	0.434	0	Saliva_B	NPC2
FCRL5	0	1.739161	0.399	0.009	0	Saliva_B	FCRL5
AHNAK1	0	1.724799	0.699	0.38	0	Saliva_B	AHNAK

DNAJB9	0	1.708194	0.596	0.2	0	Saliva_B	DNAJB9
RRBP11	0	1.670201	0.629	0.305	0	Saliva_B	RRBP1
LMAN2	0	1.668308	0.628	0.248	0	Saliva_B	LMAN2
OSTC	0	1.66379	0.66	0.27	0	Saliva_B	OSTC
ISG20	0	1.662931	0.658	0.193	0	Saliva_B	ISG20
HM131	0	1.637286	0.565	0.197	0	Saliva_B	HM13
DNAJC1	0	1.610078	0.503	0.157	0	Saliva_B	DNAJC1
SELK	0	1.591491	0.803	0.463	0	Saliva_B	SELK
DNAJC31	0	1.588858	0.556	0.201	0	Saliva_B	DNAJC3
DAD12	0	1.588002	0.762	0.392	0	Saliva_B	DAD1
KDELR21	0	1.586715	0.701	0.312	0	Saliva_B	KDELR2
GNG7	0	1.575122	0.423	0.054	0	Saliva_B	GNG7
LY961	0	1.574044	0.447	0.061	0	Saliva_B	LY96
KRTCAP2	0	1.556584	0.639	0.307	0	Saliva_B	KRTCAP2
TMEM591	0	1.548583	0.882	0.604	0	Saliva_B	TMEM59
TMED9	0	1.535847	0.625	0.285	0	Saliva_B	TMED9
SRM	0	1.534078	0.513	0.157	0	Saliva_B	SRM
PDK1	0	1.529569	0.386	0.055	0	Saliva_B	PDK1
TRAM1	0	1.52855	0.644	0.293	0	Saliva_B	TRAM1
SDC1	0	1.524168	0.48	0.13	0	Saliva_B	SDC1
PABPC4	0	1.518945	0.471	0.122	0	Saliva_B	PABPC4
KDELR11	0	1.51409	0.596	0.244	0	Saliva_B	KDELR1
CDV3	0	1.511099	0.505	0.168	0	Saliva_B	CDV3
HSPA5	0	1.504785	0.738	0.365	0	Saliva_B	HSPA5
SSR2	0	1.497391	0.789	0.421	0	Saliva_B	SSR2
POU2AF1	0	1.476082	0.35	0.009	0	Saliva_B	POU2AF1
PDIA4	0	1.472723	0.475	0.167	0	Saliva_B	PDIA4
CCDC167	0	1.459345	0.472	0.133	0	Saliva_B	CCDC167
RGS1	0	1.440698	0.356	0.064	0	Saliva_B	RGS1
NUCB22	0	1.420175	0.657	0.262	0	Saliva_B	NUCB2
ANKRD28	0	1.416065	0.4	0.104	0	Saliva_B	ANKRD28
TXNDC11	0	1.41308	0.368	0.068	0	Saliva_B	TXNDC11
SRGN	0	1.394852	0.787	0.212	0	Saliva_B	SRGN
ATRAID1	0	1.392758	0.615	0.284	0	Saliva_B	ATRAID
LGALS1	0	1.391761	0.889	0.401	0	Saliva_B	LGALS1
ZBTB38	0	1.385142	0.468	0.164	0	Saliva_B	ZBTB38
SLC35B1	0	1.363593	0.405	0.116	0	Saliva_B	SLC35B1
TPST21	0	1.360152	0.359	0.064	0	Saliva_B	TPST2
CHPF1	0	1.358609	0.369	0.077	0	Saliva_B	CHPF
SEC621	0	1.356356	0.733	0.395	0	Saliva_B	SEC62
CD632	0	1.355707	0.917	0.619	0	Saliva_B	CD63
SEC61G	0	1.355058	0.725	0.409	0	Saliva_B	SEC61G
B2M1	0	1.351362	1	0.928	0	Saliva_B	B2M
SRPRB	0	1.346593	0.383	0.107	0	Saliva_B	SRPRB
DERL1	0	1.338747	0.406	0.13	0	Saliva_B	DERL1
SERP11	0	1.322127	0.809	0.541	0	Saliva_B	SERP1
RPLP1	0	1.320387	0.99	0.914	0	Saliva_B	RPLP1

SPAG4	0	1.318802	0.306	0.028	0	Saliva_B	SPAG4
PLP2	0	1.286252	0.583	0.225	0	Saliva_B	PLP2
MT.ND53	0	1.286061	0.961	0.697	0	Saliva_B	MT.ND5
MEF2C1	0	1.268013	0.394	0.074	0	Saliva_B	MEF2C
CUTA	0	1.266154	0.708	0.37	0	Saliva_B	CUTA
TMBIM61	0	1.260097	0.828	0.574	0	Saliva_B	TMBIM6
CDK2AP2	0	1.2542	0.466	0.159	0	Saliva_B	CDK2AP2
SEL1L3	0	1.252933	0.312	0.042	0	Saliva_B	SEL1L3
MANEA	0	1.243384	0.289	0.022	0	Saliva_B	MANEA
EMP3	0	1.220359	0.788	0.343	0	Saliva_B	EMP3
GLCCI1	0	1.188733	0.282	0.038	0	Saliva_B	GLCCI1
TNFRSF18	0	1.184224	0.283	0.043	0	Saliva_B	TNFRSF18
CCR10	0	1.175558	0.244	0.009	0	Saliva_B	CCR10
IFNAR2	0	1.172183	0.316	0.066	0	Saliva_B	IFNAR2
C16orf74	0	1.171928	0.279	0.016	0	Saliva_B	C16orf74
RASGRP3	0	1.159463	0.279	0.043	0	Saliva_B	RASGRP3
EMB	0	1.149893	0.311	0.036	0	Saliva_B	EMB
ICAM3	0	1.149347	0.468	0.138	0	Saliva_B	ICAM3
MT.ND33	0	1.149307	0.996	0.791	0	Saliva_B	MT.ND3
MT.ATP63	0	1.140482	0.998	0.896	0	Saliva_B	MT.ATP6
EIF2AK41	0	1.138925	0.295	0.065	0	Saliva_B	EIF2AK4
MT.CYB3	0	1.125457	0.998	0.933	0	Saliva_B	MT.CYB
PIM2	0	1.114266	0.294	0.037	0	Saliva_B	PIM2
EEF1B2	0	1.078825	0.879	0.591	0	Saliva_B	EEF1B2
ST6GALNA	0	1.076116	0.264	0.023	0	Saliva_B	ST6GALNAC4
EVI2B	0	1.059481	0.314	0.059	0	Saliva_B	EVI2B
TPT12	0	1.047765	0.985	0.957	0	Saliva_B	TPT1
CD38	0	1.04436	0.194	0.007	0	Saliva_B	CD38
HSPA13	0	1.033031	0.258	0.042	0	Saliva_B	HSPA13
IKBIP1	0	1.031353	0.281	0.056	0	Saliva_B	IKBIP
ICAM21	0	1.019169	0.334	0.063	0	Saliva_B	ICAM2
RPL36AL	0	1.014616	0.892	0.727	0	Saliva_B	RPL36AL
RPS172	0	1.010611	0.638	0.214	0	Saliva_B	RPS17
MT.ND13	0	0.982374	0.994	0.926	0	Saliva_B	MT.ND1
EDEM2	0	0.968897	0.254	0.052	0	Saliva_B	EDEM2
RPL7A2	0	0.932634	0.96	0.811	0	Saliva_B	RPL7A
MT.CO23	0	0.928488	0.999	0.959	0	Saliva_B	MT.CO2
CECR1	0	0.926063	0.231	0.036	0	Saliva_B	CECR1
A1BG	0	0.916754	0.288	0.058	0	Saliva_B	A1BG
ZBP1	0	0.898521	0.209	0.009	0	Saliva_B	ZBP1
HSH2D	0	0.887328	0.216	0.028	0	Saliva_B	HSH2D
ORAI2	0	0.870282	0.217	0.038	0	Saliva_B	ORAI2
IRF4	0	0.86625	0.176	0.009	0	Saliva_B	IRF4
SLC1A4	0	0.862372	0.191	0.026	0	Saliva_B	SLC1A4
TNFRSF4	0	0.837177	0.206	0.025	0	Saliva_B	TNFRSF4
MT.CO32	0	0.827156	0.997	0.977	0	Saliva_B	MT.CO3
WNT10A	0	0.81803	0.181	0.009	0	Saliva_B	WNT10A

CPNE5	0	0.791925	0.166	0.004	0	Saliva_B	CPNE5
MT.ND43	0	0.788045	0.996	0.953	0	Saliva_B	MT.ND4
CXorf21	0	0.785586	0.145	0.008	0	Saliva_B	CXorf21
SLAMF1	0	0.75081	0.173	0.01	0	Saliva_B	SLAMF1
RAB30	0	0.731201	0.156	0.019	0	Saliva_B	RAB30
PNOC	0	0.685526	0.159	0.007	0	Saliva_B	PNOC
ITGA4	0	0.68542	0.182	0.025	0	Saliva_B	ITGA4
C16orf54	0	0.664711	0.128	0.008	0	Saliva_B	C16orf54
LAMP5	0	0.613232	0.083	0.003	0	Saliva_B	LAMP5
PKHD1L1	0	0.610307	0.127	0.002	0	Saliva_B	PKHD1L1
TMEM156	0	0.608207	0.121	0.01	0	Saliva_B	TMEM156
SLC17A9	0	0.606209	0.122	0.008	0	Saliva_B	SLC17A9
AC093818.	0	0.550033	0.118	0.011	0	Saliva_B	AC093818.1
KLHL6	0	0.549193	0.122	0.011	0	Saliva_B	KLHL6
LAX1	0	0.538174	0.106	0.003	0	Saliva_B	LAX1
HLA.DOB	0	0.529133	0.121	0.01	0	Saliva_B	HLA.DOB
IKZF3	0	0.528844	0.125	0.01	0	Saliva_B	IKZF3
BMP6	0	0.506688	0.097	0.003	0	Saliva_B	BMP6
KCNN31	0	0.493148	0.103	0.008	0	Saliva_B	KCNN3
ZNF215	0	0.471422	0.092	0.004	0	Saliva_B	ZNF215
P2RX1	0	0.462086	0.088	0.005	0	Saliva_B	P2RX1
ENAM	0	0.434549	0.081	0.002	0	Saliva_B	ENAM
AC026202.	0	0.412414	0.077	0.003	0	Saliva_B	AC026202.3
FTL	0	0.401272	0.977	0.894	0	Saliva_B	FTL
NUGGC	0	0.384599	0.073	0.001	0	Saliva_B	NUGGC
PGLYRP2	0	0.382443	0.073	0.003	0	Saliva_B	PGLYRP2
IL5RA	0	0.294699	0.049	0.001	0	Saliva_B	IL5RA
MIXL1	0	0.251751	0.048	0.001	0	Saliva_B	MIXL1
MTDH1	4.2E-306	1.476425	0.621	0.303	6.7E-302	Saliva_B	MTDH
CRIP1	3.1E-305	1.815232	0.602	0.271	5E-301	Saliva_B	CRIP1
ERP29	2.5E-301	1.278297	0.682	0.348	4.1E-297	Saliva_B	ERP29
GMPPB1	7E-296	1.027403	0.265	0.061	1.1E-291	Saliva_B	GMPPB
PRDX11	6.2E-294	1.276289	0.756	0.446	9.9E-290	Saliva_B	PRDX1
LMNA1	3.4E-291	1.976982	0.68	0.36	5.3E-287	Saliva_B	LMNA
OST4	6.6E-287	1.191502	0.783	0.495	1.1E-282	Saliva_B	OST4
TMED41	1.6E-284	1.248043	0.452	0.168	2.6E-280	Saliva_B	TMED4
APOBEC3G	3.3E-284	0.785683	0.222	0.042	5.3E-280	Saliva_B	APOBEC3G
DNAJB11	4.3E-284	1.339967	0.438	0.161	6.8E-280	Saliva_B	DNAJB11
EIF1	7.2E-282	0.775501	0.964	0.908	1.2E-277	Saliva_B	EIF1
HLA.C1	3.2E-277	0.96228	0.912	0.682	5.1E-273	Saliva_B	HLA.C
HDLBP1	4.1E-276	1.307149	0.557	0.255	6.6E-272	Saliva_B	HDLBP
MGST32	2.1E-274	1.129825	0.676	0.328	3.4E-270	Saliva_B	MGST3
ITGB7	9.1E-273	0.684304	0.197	0.035	1.5E-268	Saliva_B	ITGB7
HAX1	3.1E-271	1.259925	0.489	0.197	4.9E-267	Saliva_B	HAX1
IL2RG	1.7E-270	0.882026	0.42	0.127	2.7E-266	Saliva_B	IL2RG
CD79B	6.9E-268	0.402063	0.245	0.05	1.1E-263	Saliva_B	CD79B
KCNA3	2E-266	0.310078	0.071	0.005	3.2E-262	Saliva_B	KCNA3

TMEM205:	1.2E-262	1.27934	0.484	0.199	1.8E-258	Saliva_B	TMEM205
COMMD3	4.6E-261	1.238619	0.423	0.156	7.3E-257	Saliva_B	COMMD3
COL4A4	2.7E-260	0.40515	0.09	0.008	4.3E-256	Saliva_B	COL4A4
ERGIC2	3.4E-255	1.223343	0.411	0.151	5.4E-251	Saliva_B	ERGIC2
SELM2	3.4E-254	1.080307	0.684	0.34	5.4E-250	Saliva_B	SELM
TAPBP1	1.3E-253	1.143017	0.432	0.159	2.1E-249	Saliva_B	TAPBP
SH2B2	3.8E-251	0.469729	0.105	0.012	6.1E-247	Saliva_B	SH2B2
TPD521	1.3E-249	1.150575	0.364	0.122	2.1E-245	Saliva_B	TPD52
C12orf45	5.1E-249	1.018704	0.277	0.074	8.1E-245	Saliva_B	C12orf45
CTA.384D8	1.2E-248	0.26028	0.053	0.003	1.8E-244	Saliva_B	CTA.384D8.34
PRELID1	2.9E-248	1.173602	0.658	0.373	4.7E-244	Saliva_B	PRELID1
SLC3A21	1.3E-245	1.497773	0.463	0.192	2E-241	Saliva_B	SLC3A2
MLEC2	2.2E-242	1.21835	0.448	0.184	3.4E-238	Saliva_B	MLEC
HINT11	3.6E-242	1.012833	0.83	0.565	5.8E-238	Saliva_B	HINT1
PLCG21	1.4E-241	0.632776	0.158	0.026	2.2E-237	Saliva_B	PLCG2
VOPP1	3.5E-241	1.117274	0.342	0.11	5.5E-237	Saliva_B	VOPP1
NT5DC2	5.2E-241	0.854999	0.204	0.043	8.2E-237	Saliva_B	NT5DC2
AC078883.	3.9E-238	0.279013	0.064	0.004	6.2E-234	Saliva_B	AC078883.3
GOLGB11	1.1E-234	1.32517	0.457	0.194	1.7E-230	Saliva_B	GOLGB1
BTN3A21	1.5E-234	0.787145	0.24	0.056	2.4E-230	Saliva_B	BTN3A2
FAU	7.5E-232	0.713114	0.949	0.854	1.2E-227	Saliva_B	FAU
SIL1	5.2E-230	1.191285	0.333	0.11	8.3E-226	Saliva_B	SIL1
MT.ND62	1.8E-229	1.145365	0.333	0.106	2.8E-225	Saliva_B	MT.ND6
WBSCR22	2.3E-229	1.135533	0.446	0.18	3.6E-225	Saliva_B	WBSCR22
RPL18	5.9E-227	0.653522	0.961	0.877	9.4E-223	Saliva_B	RPL18
CTSS	8E-225	0.949939	0.502	0.21	1.3E-220	Saliva_B	CTSS
CYTIP	7.6E-221	0.773631	0.292	0.08	1.2E-216	Saliva_B	CYTIP
SYVN1	1.9E-220	0.983716	0.259	0.071	3E-216	Saliva_B	SYVN1
BMP8B	9.4E-220	0.470436	0.099	0.012	1.5E-215	Saliva_B	BMP8B
TIMP11	2.1E-219	0.853353	0.71	0.376	3.3E-215	Saliva_B	TIMP1
BST21	4.1E-219	0.845627	0.469	0.176	6.5E-215	Saliva_B	BST2
RPL8	4.5E-214	0.64981	0.966	0.847	7.1E-210	Saliva_B	RPL8
TRIB11	6.6E-213	0.854859	0.197	0.044	1E-208	Saliva_B	TRIB1
MT.ND23	8.9E-213	0.435869	0.99	0.905	1.4E-208	Saliva_B	MT.ND2
FNDC3A1	3.3E-211	1.082725	0.326	0.112	5.3E-207	Saliva_B	FNDC3A
CADM11	7.7E-210	1.275428	0.302	0.1	1.2E-205	Saliva_B	CADM1
RPN1	7E-209	1.220576	0.413	0.174	1.1E-204	Saliva_B	RPN1
MT.CO13	5.5E-204	0.542673	0.996	0.984	8.7E-200	Saliva_B	MT.CO1
JUND1	6.5E-204	1.527513	0.669	0.46	1E-199	Saliva_B	JUND
EPRS2	1.9E-202	1.077522	0.348	0.129	3.1E-198	Saliva_B	EPRS
FICD	4.2E-202	0.714536	0.169	0.035	6.7E-198	Saliva_B	FICD
LY9	1.6E-199	0.554223	0.122	0.018	2.5E-195	Saliva_B	LY9
BRSK1	2.2E-197	0.29687	0.054	0.004	3.5E-193	Saliva_B	BRSK1
ATP6V0B1	7.9E-197	1.033035	0.64	0.383	1.3E-192	Saliva_B	ATP6V0B
C8orf592	1.8E-194	1.117704	0.475	0.23	2.9E-190	Saliva_B	C8orf59
TMCO1	5.7E-194	1.185317	0.471	0.227	9.1E-190	Saliva_B	TMCO1
NUDT22	3.6E-193	0.976413	0.305	0.103	5.8E-189	Saliva_B	NUDT22

RPL22L1	1.6E-192	1.123633	0.461	0.214	2.5E-188	Saliva_B	RPL22L1
SLC38A5	6.3E-192	0.401993	0.095	0.012	1E-187	Saliva_B	SLC38A5
LMF1	7.6E-192	0.861967	0.179	0.04	1.2E-187	Saliva_B	LMF1
TYMP	1.7E-189	0.887347	0.432	0.181	2.7E-185	Saliva_B	TYMP
CHCHD101	2.6E-188	0.978201	0.504	0.24	4.1E-184	Saliva_B	CHCHD10
COPE	2.6E-186	1.05442	0.658	0.436	4.2E-182	Saliva_B	COPE
TGFB1	2.1E-185	0.910993	0.298	0.095	3.3E-181	Saliva_B	TGFB1
PSMB11	6.2E-185	1.028245	0.638	0.396	9.9E-181	Saliva_B	PSMB1
ZNF275	1.3E-182	0.538443	0.121	0.02	2E-178	Saliva_B	ZNF275
SPATS2	1.6E-182	1.00201	0.296	0.103	2.6E-178	Saliva_B	SPATS2
ARSG	2.2E-181	0.538309	0.125	0.022	3.5E-177	Saliva_B	ARSG
ZBTB8OS	6.3E-180	1.063281	0.332	0.127	1E-175	Saliva_B	ZBTB8OS
HOMER3	2.5E-179	0.813852	0.207	0.054	4.1E-175	Saliva_B	HOMER3
PHPT11	6.6E-179	1.018206	0.573	0.32	1.1E-174	Saliva_B	PHPT1
IRAK1	7.1E-179	0.731929	0.195	0.049	1.1E-174	Saliva_B	IRAK1
UBXN4	5E-178	1.064893	0.536	0.296	7.9E-174	Saliva_B	UBXN4
RPS11	2.1E-177	0.6494	0.93	0.774	3.3E-173	Saliva_B	RPS11
LINC00309	7.7E-177	0.267723	0.04	0.002	1.2E-172	Saliva_B	LINC00309
ZNF706	1.4E-174	1.088318	0.58	0.34	2.2E-170	Saliva_B	ZNF706
BTN2A2	1E-173	0.568637	0.118	0.02	1.6E-169	Saliva_B	BTN2A2
ATP5G1	1.5E-173	1.003953	0.501	0.256	2.3E-169	Saliva_B	ATP5G1
RPS27L1	3.2E-173	0.843044	0.709	0.44	5.1E-169	Saliva_B	RPS27L
AIG1	1.1E-171	0.953585	0.296	0.106	1.8E-167	Saliva_B	AIG1
MBD2	9.4E-171	1.048493	0.338	0.135	1.5E-166	Saliva_B	MBD2
SELT	1E-170	1.052475	0.454	0.223	1.7E-166	Saliva_B	SELT
RPL10	4.3E-170	0.583221	0.985	0.876	6.8E-166	Saliva_B	RPL10
CISD2	1.1E-169	1.138397	0.377	0.167	1.8E-165	Saliva_B	CISD2
POU2F2	2.9E-169	0.646573	0.185	0.045	4.7E-165	Saliva_B	POU2F2
STK4	9.3E-169	0.769302	0.314	0.11	1.5E-164	Saliva_B	STK4
HSD17B8	2E-168	0.785411	0.212	0.059	3.2E-164	Saliva_B	HSD17B8
GLRX	5.9E-166	0.910121	0.454	0.209	9.5E-162	Saliva_B	GLRX
ADAM28	5.6E-165	0.520664	0.126	0.024	9E-161	Saliva_B	ADAM28
EDF1	8E-165	0.825126	0.789	0.621	1.3E-160	Saliva_B	EDF1
RALGPS2	4.7E-163	0.891937	0.258	0.085	7.4E-159	Saliva_B	RALGPS2
ST6GAL11	5.3E-163	0.658095	0.16	0.037	8.5E-159	Saliva_B	ST6GAL1
DERL2	7E-163	1.043674	0.379	0.171	1.1E-158	Saliva_B	DERL2
UFM1	2E-162	1.087891	0.445	0.226	3.2E-158	Saliva_B	UFM1
RPS5	3.6E-162	0.560379	0.934	0.783	5.8E-158	Saliva_B	RPS5
GTF3A	4.8E-161	0.92491	0.488	0.246	7.7E-157	Saliva_B	GTF3A
NCF1	8.6E-161	0.410745	0.17	0.039	1.4E-156	Saliva_B	NCF1
RPL51	1.2E-160	0.530109	0.942	0.724	1.9E-156	Saliva_B	RPL5
GPRC5D	1E-159	0.250016	0.043	0.003	1.7E-155	Saliva_B	GPRC5D
ISCU	1.8E-159	0.996713	0.583	0.349	2.9E-155	Saliva_B	ISCU
KLF131	1.1E-154	0.737598	0.247	0.079	1.8E-150	Saliva_B	KLF13
ARFGAP31	7E-154	1.048015	0.353	0.155	1.1E-149	Saliva_B	ARFGAP3
PARP1	7.5E-153	0.847464	0.313	0.12	1.2E-148	Saliva_B	PARP1
FOSB2	3.2E-152	1.531531	0.466	0.248	5E-148	Saliva_B	FOSB

NDUFB71	5.3E-152	0.910908	0.595	0.369	8.4E-148	Saliva_B	NDUFB7
RPL301	8.7E-152	0.555908	0.955	0.842	1.4E-147	Saliva_B	RPL30
RPL391	1.4E-151	0.586776	0.923	0.672	2.3E-147	Saliva_B	RPL39
PDIA31	2.5E-149	0.984169	0.545	0.318	4E-145	Saliva_B	PDIA3
LRPAP12	3E-148	0.993143	0.491	0.265	4.7E-144	Saliva_B	LRPAP1
TMEM147	2.4E-146	0.938783	0.459	0.24	3.8E-142	Saliva_B	TMEM147
RPLP0	7.8E-145	0.550533	0.915	0.796	1.2E-140	Saliva_B	RPLP0
LMO41	1.7E-144	0.999209	0.359	0.161	2.7E-140	Saliva_B	LMO4
BCL2	1.7E-144	0.582349	0.161	0.039	2.8E-140	Saliva_B	BCL2
TCF3	2.4E-143	0.575748	0.148	0.035	3.9E-139	Saliva_B	TCF3
ARMCX3	3.1E-143	0.966731	0.331	0.142	5E-139	Saliva_B	ARMCX3
SEMA4A	3.3E-143	0.631892	0.148	0.035	5.3E-139	Saliva_B	SEMA4A
ATF4	2.3E-142	0.969567	0.552	0.333	3.7E-138	Saliva_B	ATF4
CLIC1	1.4E-141	0.825375	0.71	0.471	2.3E-137	Saliva_B	CLIC1
TAGLN21	2.8E-140	0.876908	0.644	0.404	4.4E-136	Saliva_B	TAGLN2
RGCC2	1.1E-138	0.819437	0.451	0.21	1.7E-134	Saliva_B	RGCC
FAM69A	1.3E-136	0.370822	0.082	0.013	2E-132	Saliva_B	FAM69A
E2F5	3.2E-136	0.360398	0.076	0.011	5.1E-132	Saliva_B	E2F5
DNAJC15	3.3E-136	0.913986	0.386	0.186	5.2E-132	Saliva_B	DNAJC15
DPP71	1.1E-134	0.849544	0.476	0.255	1.7E-130	Saliva_B	DPP7
AKR1A11	1.8E-134	0.960494	0.4	0.201	2.9E-130	Saliva_B	AKR1A1
MTRNR2L1	6.4E-134	0.328514	0.532	0.264	1E-129	Saliva_B	MTRNR2L12
CCM2	1.4E-133	0.733009	0.242	0.083	2.2E-129	Saliva_B	CCM2
TRABD	1.6E-132	0.824649	0.289	0.114	2.5E-128	Saliva_B	TRABD
GLT8D1	2.9E-132	0.794311	0.241	0.086	4.6E-128	Saliva_B	GLT8D1
UQCRH	2.5E-131	0.802058	0.676	0.475	4E-127	Saliva_B	UQCRH
ANKRD12	3E-131	0.93218	0.526	0.319	4.9E-127	Saliva_B	ANKRD12
SLC44A1	3.4E-131	0.778017	0.228	0.077	5.4E-127	Saliva_B	SLC44A1
NFE2L3	3.9E-131	0.394248	0.088	0.015	6.2E-127	Saliva_B	NFE2L3
NACA1	7.3E-131	0.519387	0.917	0.829	1.2E-126	Saliva_B	NACA
NOL7	1.3E-130	0.93783	0.404	0.202	2E-126	Saliva_B	NOL7
SLC52A2	5.5E-130	0.774875	0.223	0.076	8.8E-126	Saliva_B	SLC52A2
WIPI11	3.3E-129	0.898836	0.251	0.095	5.3E-125	Saliva_B	WIPI1
SEC61A11	4.9E-129	0.971465	0.329	0.149	7.8E-125	Saliva_B	SEC61A1
JTB1	2E-128	0.859464	0.58	0.367	3.2E-124	Saliva_B	JTB
CD48	3.4E-128	0.492718	0.325	0.119	5.4E-124	Saliva_B	CD48
NME1	4.2E-128	0.891818	0.347	0.161	6.7E-124	Saliva_B	NME1
TRAM2	9.4E-128	0.575236	0.141	0.035	1.5E-123	Saliva_B	TRAM2
ERP44	5.5E-127	1.014366	0.358	0.174	8.8E-123	Saliva_B	ERP44
NANS1	1.8E-125	0.95639	0.42	0.227	2.9E-121	Saliva_B	NANS
TAPBPL	1.7E-124	0.676041	0.198	0.063	2.7E-120	Saliva_B	TAPBPL
MESDC21	1.2E-122	0.936746	0.351	0.169	1.8E-118	Saliva_B	MESDC2
ODC1	3.8E-122	0.81664	0.314	0.134	6.1E-118	Saliva_B	ODC1
CDK14	5.7E-122	0.524165	0.113	0.025	9E-118	Saliva_B	CDK14
MIAT	2E-121	0.288517	0.054	0.006	3.2E-117	Saliva_B	MIAT
P2RX5	4E-121	0.30898	0.076	0.012	6.4E-117	Saliva_B	P2RX5
PSMD5.AS	2E-120	0.663702	0.191	0.06	3.2E-116	Saliva_B	PSMD5.AS1

CYSLTR11	2.5E-120	0.364492	0.088	0.016	4E-116	Saliva_B	CYSLTR1
IGF1	1.7E-119	0.412571	0.141	0.035	2.7E-115	Saliva_B	IGF1
OS91	3.8E-119	1.000968	0.409	0.223	6.1E-115	Saliva_B	OS9
CREB3L2	8.6E-119	0.825478	0.249	0.097	1.4E-114	Saliva_B	CREB3L2
MT.ND4L3	3.4E-118	0.593069	0.639	0.413	5.4E-114	Saliva_B	MT.ND4L
FOS1	4.1E-118	1.400837	0.595	0.422	6.6E-114	Saliva_B	FOS
TMEM19	5.8E-118	0.582812	0.153	0.042	9.2E-114	Saliva_B	TMEM19
CHST12	6.3E-117	0.667195	0.224	0.079	1E-112	Saliva_B	CHST12
SYNE31	2.3E-116	0.394199	0.091	0.017	3.6E-112	Saliva_B	SYNE3
WNT5B	4.6E-116	0.415356	0.083	0.015	7.3E-112	Saliva_B	WNT5B
DOK3	7.6E-116	0.291691	0.055	0.007	1.2E-111	Saliva_B	DOK3
SMDT1	1.7E-115	0.846081	0.562	0.369	2.8E-111	Saliva_B	SMDT1
FBXW7	2.7E-115	0.770746	0.244	0.094	4.3E-111	Saliva_B	FBXW7
GLDC	5.8E-115	0.330739	0.066	0.01	9.2E-111	Saliva_B	GLDC
BOD1L11	1.2E-114	0.822502	0.289	0.125	2E-110	Saliva_B	BOD1L1
APOBEC3C	6.6E-114	0.668223	0.172	0.052	1.1E-109	Saliva_B	APOBEC3C
FUT8	7.2E-114	0.584397	0.145	0.04	1.1E-109	Saliva_B	FUT8
DCPS	5E-112	0.660594	0.176	0.055	8E-108	Saliva_B	DCPS
NDUFB82	5.5E-112	0.894994	0.349	0.175	8.8E-108	Saliva_B	NDUFB8
PPP1R14B1	1.7E-111	0.913238	0.35	0.174	2.8E-107	Saliva_B	PPP1R14B
SRP72	1.9E-111	0.91205	0.354	0.18	3E-107	Saliva_B	SRP72
ORMDL1	3.8E-110	0.846895	0.347	0.171	6.1E-106	Saliva_B	ORMDL1
NDUFB11	5.4E-110	0.750897	0.651	0.471	8.6E-106	Saliva_B	NDUFB11
PSMA31	1.3E-109	0.903907	0.364	0.191	2.1E-105	Saliva_B	PSMA3
TMED5	2.5E-109	0.802529	0.282	0.125	3.9E-105	Saliva_B	TMED5
SEC11A1	3.1E-109	0.852135	0.502	0.312	4.9E-105	Saliva_B	SEC11A
TAGAP	4.1E-109	0.447387	0.118	0.028	6.6E-105	Saliva_B	TAGAP
CNPY21	7.2E-109	0.920752	0.382	0.202	1.2E-104	Saliva_B	CNPY2
NDUFB101	7.3E-109	0.755347	0.573	0.381	1.2E-104	Saliva_B	NDUFB10
RNASET2	1.7E-108	0.68558	0.437	0.233	2.8E-104	Saliva_B	RNASET2
PDE4B	2.4E-108	0.680925	0.162	0.049	3.8E-104	Saliva_B	PDE4B
GSTP1	6.4E-108	0.679297	0.754	0.553	1E-103	Saliva_B	GSTP1
PSMD8	1.4E-107	0.830714	0.511	0.329	2.2E-103	Saliva_B	PSMD8
QPCT	2.8E-106	1.002703	0.189	0.065	4.4E-102	Saliva_B	QPCT
CASP101	1.2E-105	0.547355	0.132	0.035	1.8E-101	Saliva_B	CASP10
SEPT6	1.2E-105	0.615556	0.242	0.092	1.9E-101	Saliva_B	SEPT6
ALOX5	4.8E-105	0.419144	0.091	0.019	7.7E-101	Saliva_B	ALOX5
TMEM134	1.6E-104	0.853804	0.339	0.172	2.6E-100	Saliva_B	TMEM134
TECR1	1.9E-104	0.81481	0.512	0.327	3E-100	Saliva_B	TECR
ATP5J1	3.1E-104	0.787043	0.605	0.433	5E-100	Saliva_B	ATP5J
NCF4	8.8E-104	0.483927	0.144	0.04	1.4E-99	Saliva_B	NCF4
DENND5B	1.3E-103	0.409134	0.094	0.02	2.1E-99	Saliva_B	DENND5B
VKORC11	2E-103	0.797926	0.401	0.221	3.2E-99	Saliva_B	VKORC1
TRMT112	3.3E-103	0.808684	0.56	0.371	5.3E-99	Saliva_B	TRMT112
SSR11	3.6E-103	0.896702	0.358	0.188	5.7E-99	Saliva_B	SSR1
ATP5G3	1.3E-102	0.763437	0.549	0.358	2.15E-98	Saliva_B	ATP5G3
MAN1A2	5.5E-102	0.834575	0.256	0.111	8.83E-98	Saliva_B	MAN1A2

UBE2M	7.3E-102	0.821354	0.328	0.163	1.16E-97	Saliva_B	UBE2M
ARSA	8.2E-102	0.77385	0.216	0.085	1.31E-97	Saliva_B	ARSA
DHRS71	1.8E-101	0.744077	0.387	0.205	2.87E-97	Saliva_B	DHRS7
KLF6	3.3E-101	1.50868	0.546	0.399	5.25E-97	Saliva_B	KLF6
DENND6B	9.3E-101	0.480983	0.118	0.03	1.49E-96	Saliva_B	DENND6B
C11orf24	2.2E-100	0.619186	0.173	0.058	3.56E-96	Saliva_B	C11orf24
CD53	1.7E-99	0.454922	0.297	0.119	2.75E-95	Saliva_B	CD53
RAB8A	3E-99	0.831795	0.288	0.134	4.77E-95	Saliva_B	RAB8A
GOLGA41	3.9E-99	0.868058	0.406	0.232	6.14E-95	Saliva_B	GOLGA4
MARCH9	4.2E-99	0.456463	0.11	0.027	6.68E-95	Saliva_B	MARCH9
TRADD	4.9E-99	0.66315	0.263	0.111	7.79E-95	Saliva_B	TRADD
CSF2RB1	1.37E-98	0.482463	0.132	0.036	2.19E-94	Saliva_B	CSF2RB
EMC7	7.83E-98	0.862624	0.362	0.196	1.25E-93	Saliva_B	EMC7
PRMT11	1.1E-97	0.785738	0.364	0.189	1.76E-93	Saliva_B	PRMT1
JUN1	1.26E-97	2.179139	0.57	0.486	2.01E-93	Saliva_B	JUN
RHOQ	6.46E-97	0.742756	0.215	0.084	1.03E-92	Saliva_B	RHOQ
NDUFAB1	8.02E-97	0.866135	0.414	0.243	1.28E-92	Saliva_B	NDUFAB1
PARK72	2.07E-96	0.665674	0.558	0.352	3.3E-92	Saliva_B	PARK7
QPRT	2.54E-96	0.43592	0.099	0.023	4.05E-92	Saliva_B	QPRT
IFI27L1	3.43E-96	0.702549	0.199	0.075	5.46E-92	Saliva_B	IFI27L1
RPS27A1	3.6E-96	0.415348	0.97	0.847	5.74E-92	Saliva_B	RPS27A
SPINK2	3.81E-96	0.452653	0.212	0.075	6.07E-92	Saliva_B	SPINK2
MXD41	4.26E-95	0.741371	0.263	0.118	6.8E-91	Saliva_B	MXD4
COX5A	2.09E-94	0.775521	0.578	0.409	3.34E-90	Saliva_B	COX5A
RRAD	4.44E-94	0.886353	0.122	0.033	7.08E-90	Saliva_B	RRAD
EDEM3	5.82E-93	0.628643	0.185	0.067	9.29E-89	Saliva_B	EDEM3
TOR3A	1.16E-92	0.541329	0.158	0.051	1.84E-88	Saliva_B	TOR3A
ARL11	2.25E-92	0.904092	0.36	0.204	3.59E-88	Saliva_B	ARL1
TTLL7	2.38E-91	0.64212	0.176	0.063	3.8E-87	Saliva_B	TTLL7
MBD4	2.98E-91	0.674216	0.225	0.093	4.75E-87	Saliva_B	MBD4
SLC38A10	3E-91	0.795195	0.23	0.097	4.78E-87	Saliva_B	SLC38A10
SRRM21	4.33E-91	0.810556	0.513	0.345	6.9E-87	Saliva_B	SRRM2
TRPV3	1.15E-89	0.283443	0.051	0.007	1.83E-85	Saliva_B	TRPV3
UFC1	1.57E-89	0.787894	0.504	0.344	2.5E-85	Saliva_B	UFC1
RPS14	3.7E-89	0.377755	0.971	0.881	5.9E-85	Saliva_B	RPS14
EEF1A11	5E-89	0.414058	0.971	0.914	7.98E-85	Saliva_B	EEF1A1
GYPC1	6.74E-89	0.319282	0.413	0.199	1.07E-84	Saliva_B	GYPC
TP53INP1	7.69E-89	0.655944	0.169	0.06	1.23E-84	Saliva_B	TP53INP1
RP11.356I2	3.08E-88	0.362549	0.082	0.018	4.92E-84	Saliva_B	RP11.356I2.4
HYOU1	5.69E-88	0.711601	0.197	0.078	9.08E-84	Saliva_B	HYOU1
SCFD1	6.23E-88	0.769464	0.247	0.112	9.93E-84	Saliva_B	SCFD1
LSM10	6.25E-88	0.639342	0.278	0.129	9.96E-84	Saliva_B	LSM10
ATP5D	7.56E-88	0.596731	0.626	0.443	1.21E-83	Saliva_B	ATP5D
PTPN6	1.62E-87	0.48981	0.199	0.074	2.58E-83	Saliva_B	PTPN6
SVIP	1.89E-87	0.819693	0.335	0.178	3.01E-83	Saliva_B	SVIP
NCKAP1L	9.9E-87	0.34284	0.091	0.021	1.58E-82	Saliva_B	NCKAP1L
MRPL201	8.91E-86	0.76906	0.431	0.268	1.42E-81	Saliva_B	MRPL20

ITGA62	5.45E-85	0.812868	0.274	0.133	8.69E-81	Saliva_B	ITGA6
CCDC691	1.56E-84	0.649524	0.263	0.117	2.48E-80	Saliva_B	CCDC69
INPP4A	3.5E-84	0.436512	0.107	0.029	5.58E-80	Saliva_B	INPP4A
PDCD5	2.26E-83	0.686341	0.408	0.238	3.6E-79	Saliva_B	PDCD5
GUSB	4.21E-83	0.824136	0.273	0.135	6.72E-79	Saliva_B	GUSB
CHID11	1.19E-82	0.793131	0.314	0.168	1.91E-78	Saliva_B	CHID1
ACBD3	1.43E-82	0.77229	0.254	0.12	2.29E-78	Saliva_B	ACBD3
RPS19BP1	2.74E-82	0.740761	0.437	0.277	4.38E-78	Saliva_B	RPS19BP1
FAM3C	1.43E-81	0.764661	0.349	0.194	2.28E-77	Saliva_B	FAM3C
YIF1A	1.47E-81	0.752617	0.317	0.17	2.34E-77	Saliva_B	YIF1A
C7orf50	1.88E-81	0.710873	0.271	0.132	3E-77	Saliva_B	C7orf50
MPG	2.36E-81	0.738527	0.32	0.17	3.77E-77	Saliva_B	MPG
AUP1	2.45E-81	0.794043	0.351	0.201	3.92E-77	Saliva_B	AUP1
CD47	2.65E-81	0.74764	0.389	0.227	4.22E-77	Saliva_B	CD47
RPL3	5.21E-81	0.25375	0.971	0.841	8.31E-77	Saliva_B	RPL3
ALDH1L2	1.01E-80	0.45955	0.106	0.029	1.61E-76	Saliva_B	ALDH1L2
ATP5L	1.64E-80	0.532244	0.776	0.658	2.62E-76	Saliva_B	ATP5L
IFNAR1	2.96E-80	0.812914	0.317	0.172	4.72E-76	Saliva_B	IFNAR1
EEF1D	4.17E-80	0.302008	0.863	0.744	6.64E-76	Saliva_B	EEF1D
SAR1B	6.01E-80	0.841292	0.322	0.18	9.58E-76	Saliva_B	SAR1B
GRK6	7.36E-80	0.49985	0.197	0.076	1.17E-75	Saliva_B	GRK6
P4HB1	9E-80	0.603131	0.602	0.416	1.43E-75	Saliva_B	P4HB
PBXIP1	1.13E-79	0.765384	0.247	0.112	1.81E-75	Saliva_B	PBXIP1
SP140	1.14E-79	0.311149	0.079	0.018	1.81E-75	Saliva_B	SP140
EIF5A	1.5E-79	0.785902	0.432	0.276	2.39E-75	Saliva_B	EIF5A
ERN1	2.95E-79	0.561499	0.157	0.056	4.71E-75	Saliva_B	ERN1
ATF5	7.88E-79	0.85447	0.189	0.077	1.26E-74	Saliva_B	ATF5
NGLY1	1.09E-78	0.561043	0.23	0.1	1.73E-74	Saliva_B	NGLY1
NDUFA41	7.89E-78	0.53219	0.718	0.535	1.26E-73	Saliva_B	NDUFA4
VEGFB1	9.44E-78	0.700433	0.221	0.097	1.51E-73	Saliva_B	VEGFB
UNC93B1	1.25E-77	0.583856	0.17	0.064	1.99E-73	Saliva_B	UNC93B1
BTN3A11	2.31E-77	0.363356	0.088	0.022	3.68E-73	Saliva_B	BTN3A1
PPP1R101	4.51E-77	0.744177	0.29	0.15	7.2E-73	Saliva_B	PPP1R10
POLR2J31	4.54E-77	0.637981	0.217	0.095	7.24E-73	Saliva_B	POLR2J3
SEC14L11	6.39E-77	0.730979	0.296	0.154	1.02E-72	Saliva_B	SEC14L1
LARP1B	1.84E-76	0.665323	0.203	0.086	2.93E-72	Saliva_B	LARP1B
ANAPC51	2.8E-76	0.708746	0.299	0.159	4.47E-72	Saliva_B	ANAPC5
USO1	4.16E-76	0.788283	0.255	0.126	6.64E-72	Saliva_B	USO1
RPL241	6.12E-76	0.400089	0.906	0.8	9.77E-72	Saliva_B	RPL24
SMPDL3B	6.25E-76	0.350874	0.079	0.019	9.97E-72	Saliva_B	SMPDL3B
TCF25	5.54E-75	0.752679	0.44	0.291	8.84E-71	Saliva_B	TCF25
C19orf60	7.77E-75	0.68086	0.385	0.226	1.24E-70	Saliva_B	C19orf60
YIPF22	1.88E-74	0.627136	0.228	0.106	3E-70	Saliva_B	YIPF2
CCDC88C	4.15E-74	0.441247	0.127	0.04	6.62E-70	Saliva_B	CCDC88C
SLC50A1	4.99E-74	0.652567	0.188	0.078	7.96E-70	Saliva_B	SLC50A1
NDUFC21	5.46E-74	0.659101	0.534	0.384	8.7E-70	Saliva_B	NDUFC2
RNASEH2C	1.18E-73	0.701709	0.329	0.184	1.88E-69	Saliva_B	RNASEH2C

FCGR2B	2.28E-73	0.34687	0.102	0.028	3.64E-69	Saliva_B	FCGR2B
CLCC1	2.84E-72	0.482734	0.124	0.041	4.52E-68	Saliva_B	CLCC1
CLECL1	7.42E-72	0.282433	0.061	0.012	1.18E-67	Saliva_B	CLECL1
PLXNC1	2.51E-71	0.30669	0.067	0.015	4E-67	Saliva_B	PLXNC1
MGAT2	3.21E-71	0.618692	0.174	0.07	5.13E-67	Saliva_B	MGAT2
IQGAP2	3.66E-71	0.587731	0.241	0.113	5.84E-67	Saliva_B	IQGAP2
DOCK2	1.5E-70	0.346716	0.079	0.019	2.39E-66	Saliva_B	DOCK2
SURF1	1.74E-70	0.755844	0.31	0.175	2.78E-66	Saliva_B	SURF1
AVEN	2.34E-70	0.393327	0.092	0.026	3.73E-66	Saliva_B	AVEN
GLS1	1.06E-69	0.676259	0.241	0.115	1.68E-65	Saliva_B	GLS
LSM5	1.26E-69	0.69775	0.339	0.199	2.02E-65	Saliva_B	LSM5
ATP2B11	1.4E-69	0.703096	0.285	0.148	2.24E-65	Saliva_B	ATP2B1
SERPINI11	3.64E-69	0.28744	0.085	0.022	5.81E-65	Saliva_B	SERPINI1
DNAJC4	3.79E-69	0.407432	0.323	0.177	6.04E-65	Saliva_B	DNAJC4
SLC25A4	4.13E-69	0.732614	0.231	0.112	6.59E-65	Saliva_B	SLC25A4
RPL291	8.01E-69	0.307942	0.945	0.827	1.28E-64	Saliva_B	RPL29
MRPL40	1.75E-68	0.714356	0.296	0.164	2.79E-64	Saliva_B	MRPL40
FAM89B	2.61E-68	0.73966	0.31	0.172	4.17E-64	Saliva_B	FAM89B
PMM2	5.47E-68	0.505722	0.118	0.039	8.72E-64	Saliva_B	PMM2
LAMP22	6.31E-68	0.830987	0.366	0.234	1.01E-63	Saliva_B	LAMP2
MAP2K2	8.67E-68	0.671485	0.335	0.196	1.38E-63	Saliva_B	MAP2K2
BTK	1.35E-67	0.305518	0.07	0.016	2.15E-63	Saliva_B	BTK
TMEM179I	1.38E-67	0.721197	0.332	0.196	2.2E-63	Saliva_B	TMEM179B
DDX171	4.86E-67	0.707352	0.491	0.352	7.74E-63	Saliva_B	DDX17
HAGH1	7.31E-67	0.655917	0.261	0.136	1.17E-62	Saliva_B	HAGH
STK17B	8.98E-67	0.55057	0.223	0.102	1.43E-62	Saliva_B	STK17B
TTC31	1.29E-66	0.668121	0.381	0.231	2.06E-62	Saliva_B	TTC3
KLF21	1.33E-66	0.892438	0.34	0.189	2.13E-62	Saliva_B	KLF2
ANKRD37	1.43E-66	0.574076	0.409	0.25	2.29E-62	Saliva_B	ANKRD37
ST8SIA4	1.63E-65	0.350023	0.094	0.027	2.6E-61	Saliva_B	ST8SIA4
ECI11	2.57E-65	0.671858	0.258	0.136	4.1E-61	Saliva_B	ECI1
PRDX21	4.13E-65	0.598608	0.462	0.309	6.58E-61	Saliva_B	PRDX2
EIF51	9.83E-65	0.715869	0.492	0.358	1.57E-60	Saliva_B	EIF5
MRPS2	3.55E-64	0.60796	0.174	0.074	5.67E-60	Saliva_B	MRPS2
SERF2	4.28E-64	0.375884	0.891	0.822	6.83E-60	Saliva_B	SERF2
PSMD7	4.9E-64	0.720769	0.342	0.21	7.82E-60	Saliva_B	PSMD7
TSC22D3	5.03E-64	0.800559	0.434	0.279	8.02E-60	Saliva_B	TSC22D3
H3F3A2	5.1E-64	0.359646	0.744	0.473	8.13E-60	Saliva_B	H3F3A
UBA5	2.13E-63	0.625759	0.202	0.094	3.4E-59	Saliva_B	UBA5
ABHD14A	2.82E-63	0.551601	0.214	0.101	4.49E-59	Saliva_B	ABHD14A
CANX2	2.85E-63	0.754538	0.441	0.31	4.55E-59	Saliva_B	CANX
RPS81	1E-62	0.314341	0.927	0.848	1.6E-58	Saliva_B	RPS8
ARL6IP4	1.35E-62	0.537289	0.56	0.419	2.16E-58	Saliva_B	ARL6IP4
SIVA1	1.36E-62	0.651074	0.415	0.271	2.17E-58	Saliva_B	SIVA1
BIRC3	2.46E-62	0.523793	0.291	0.152	3.93E-58	Saliva_B	BIRC3
IQCB1	4.19E-62	0.421851	0.125	0.044	6.69E-58	Saliva_B	IQCB1
EIF1AY	4.85E-62	0.635727	0.188	0.085	7.74E-58	Saliva_B	EIF1AY

TGFBR3L	1.8E-61	0.256075	0.044	0.008	2.87E-57	Saliva_B	TGFBR3L
C21orf2	2.03E-61	0.605942	0.199	0.092	3.24E-57	Saliva_B	C21orf2
FKBP14	3.45E-61	0.476831	0.12	0.042	5.51E-57	Saliva_B	FKBP14
CTSH1	4.44E-61	0.912966	0.361	0.236	7.07E-57	Saliva_B	CTSH
TIFA	8.27E-61	0.50161	0.148	0.059	1.32E-56	Saliva_B	TIFA
ATP8B2	8.89E-61	0.255374	0.057	0.012	1.42E-56	Saliva_B	ATP8B2
RFC11	1.59E-60	0.649357	0.225	0.113	2.54E-56	Saliva_B	RFC1
MRPS34	3.63E-60	0.6232	0.326	0.195	5.8E-56	Saliva_B	MRPS34
PSME2	3.79E-60	0.516674	0.437	0.285	6.04E-56	Saliva_B	PSME2
PCMTD1	4.28E-60	0.764577	0.3	0.178	6.83E-56	Saliva_B	PCMTD1
CHST15	1.59E-59	0.332571	0.081	0.023	2.54E-55	Saliva_B	CHST15
PSMB6	1.18E-58	0.668538	0.446	0.319	1.87E-54	Saliva_B	PSMB6
RELB	1.72E-58	0.503341	0.156	0.064	2.74E-54	Saliva_B	RELB
MRPL51	4.08E-58	0.572394	0.419	0.279	6.51E-54	Saliva_B	MRPL51
FUCA2	4.42E-58	0.595678	0.184	0.085	7.05E-54	Saliva_B	FUCA2
GRB2	4.65E-58	0.670407	0.293	0.17	7.42E-54	Saliva_B	GRB2
GUK11	6.7E-58	0.509304	0.661	0.556	1.07E-53	Saliva_B	GUK1
ATOX11	1.25E-57	0.655368	0.403	0.271	1.99E-53	Saliva_B	ATOX1
RPS231	1.66E-57	0.303004	0.956	0.811	2.65E-53	Saliva_B	RPS23
SSSCA1	2.81E-57	0.704171	0.246	0.133	4.49E-53	Saliva_B	SSSCA1
PPM1K	2.95E-57	0.569264	0.203	0.097	4.7E-53	Saliva_B	PPM1K
NCOA3	4.91E-57	0.656656	0.202	0.098	7.83E-53	Saliva_B	NCOA3
MAN2A1	9.9E-57	0.508525	0.135	0.053	1.58E-52	Saliva_B	MAN2A1
FIS11	1.47E-56	0.55439	0.471	0.336	2.35E-52	Saliva_B	FIS1
NCL1	2.45E-56	0.656516	0.409	0.268	3.91E-52	Saliva_B	NCL
B4GALT7	3.64E-56	0.466673	0.132	0.051	5.8E-52	Saliva_B	B4GALT7
IFI351	4.09E-56	0.501205	0.169	0.074	6.52E-52	Saliva_B	IFI35
SLC39A71	5.82E-56	0.618576	0.218	0.112	9.29E-52	Saliva_B	SLC39A7
MZT2B1	8.85E-56	0.555359	0.597	0.459	1.41E-51	Saliva_B	MZT2B
TOMM202	1.1E-55	0.620974	0.454	0.323	1.75E-51	Saliva_B	TOMM20
NAGLU1	1.34E-55	0.526113	0.157	0.068	2.13E-51	Saliva_B	NAGLU
FMNL1	2.81E-55	0.376617	0.155	0.062	4.47E-51	Saliva_B	FMNL1
MSI2	2.86E-55	0.570738	0.158	0.069	4.57E-51	Saliva_B	MSI2
GADD45GI	5.69E-55	0.576989	0.413	0.277	9.07E-51	Saliva_B	GADD45GIP1
AKAP9	9.11E-55	0.709662	0.39	0.268	1.45E-50	Saliva_B	AKAP9
SLC16A1.A	1.06E-54	0.319985	0.075	0.021	1.69E-50	Saliva_B	SLC16A1.AS1
FAM96B	1.51E-54	0.668534	0.384	0.256	2.4E-50	Saliva_B	FAM96B
ERGIC3	2.51E-54	0.635959	0.434	0.309	4.01E-50	Saliva_B	ERGIC3
CALU	5.1E-54	0.649622	0.293	0.173	8.14E-50	Saliva_B	CALU
MAN1A1	1.04E-53	0.421188	0.186	0.085	1.66E-49	Saliva_B	MAN1A1
SEC24D	1.12E-53	0.570584	0.184	0.088	1.79E-49	Saliva_B	SEC24D
AURKAIP1	3.65E-53	0.387769	0.507	0.374	5.82E-49	Saliva_B	AURKAIP1
MRPS31	4.05E-53	0.492273	0.165	0.074	6.46E-49	Saliva_B	MRPS31
FBXO18	4.44E-53	0.452646	0.143	0.06	7.08E-49	Saliva_B	FBXO18
HLA.E1	4.62E-53	0.328698	0.743	0.607	7.37E-49	Saliva_B	HLA.E
RPS15A	7.44E-53	0.272316	0.947	0.848	1.19E-48	Saliva_B	RPS15A
DNPH1	2.46E-52	0.632188	0.258	0.145	3.93E-48	Saliva_B	DNPH1

IRF91	3.48E-52	0.277578	0.079	0.023	5.55E-48	Saliva_B	IRF9
ZNF7911	4.25E-52	0.485603	0.156	0.068	6.77E-48	Saliva_B	ZNF791
FAIM	5.64E-52	0.448332	0.114	0.042	9E-48	Saliva_B	FAIM
ARHGDIA	7.33E-52	0.55966	0.324	0.195	1.17E-47	Saliva_B	ARHGDIA
ELL21	1.62E-51	0.676945	0.227	0.124	2.58E-47	Saliva_B	ELL2
NDUFS6	1.67E-51	0.627185	0.437	0.322	2.67E-47	Saliva_B	NDUFS6
STT3B	1.78E-51	0.583039	0.24	0.131	2.84E-47	Saliva_B	STT3B
GORASP2	2.97E-51	0.704662	0.249	0.142	4.74E-47	Saliva_B	GORASP2
UFSP2	3.37E-51	0.578485	0.186	0.091	5.38E-47	Saliva_B	UFSP2
RP11.841C	5.92E-51	0.345658	0.073	0.021	9.44E-47	Saliva_B	RP11.841O20.2
PGM31	6.37E-51	0.671694	0.183	0.09	1.02E-46	Saliva_B	PGM3
GPR160	6.89E-51	0.531026	0.137	0.057	1.1E-46	Saliva_B	GPR160
KIF21A1	1.26E-50	0.604659	0.168	0.08	2.01E-46	Saliva_B	KIF21A
C15orf57	1.27E-50	0.475385	0.15	0.065	2.02E-46	Saliva_B	C15orf57
SRSF9	1.42E-50	0.643591	0.464	0.352	2.26E-46	Saliva_B	SRSF9
IL10RA	2.32E-50	0.281294	0.119	0.044	3.71E-46	Saliva_B	IL10RA
WDR45	2.59E-50	0.610879	0.243	0.137	4.13E-46	Saliva_B	WDR45
CAPN12	3.7E-50	0.293023	0.072	0.021	5.89E-46	Saliva_B	CAPN12
ALG14	4.27E-50	0.575697	0.144	0.063	6.81E-46	Saliva_B	ALG14
TSR3	5.63E-50	0.53788	0.202	0.103	8.98E-46	Saliva_B	TSR3
EIF3K	9.43E-50	0.308979	0.65	0.516	1.5E-45	Saliva_B	EIF3K
CTA.292E1	1.53E-49	0.434194	0.115	0.045	2.44E-45	Saliva_B	CTA.292E10.6
CLN6	1.95E-49	0.425688	0.108	0.04	3.11E-45	Saliva_B	CLN6
NDUFA1	2.39E-49	0.577623	0.524	0.41	3.81E-45	Saliva_B	NDUFA1
MYCBP21	2.62E-49	0.563824	0.254	0.143	4.18E-45	Saliva_B	MYCBP2
PRSS16	3.05E-49	0.293617	0.065	0.018	4.86E-45	Saliva_B	PRSS16
TXNL4A	4.42E-49	0.678502	0.343	0.231	7.04E-45	Saliva_B	TXNL4A
SRP54	4.83E-49	0.650017	0.3	0.188	7.71E-45	Saliva_B	SRP54
IKZF1	6.14E-49	0.279659	0.108	0.038	9.79E-45	Saliva_B	IKZF1
CYP20A11	9.98E-49	0.597515	0.189	0.094	1.59E-44	Saliva_B	CYP20A1
MARS	1.5E-48	0.455328	0.132	0.055	2.39E-44	Saliva_B	MARS
DUS1L1	2.18E-48	0.549343	0.188	0.095	3.48E-44	Saliva_B	DUS1L
ARID3A	2.48E-48	0.467411	0.11	0.041	3.96E-44	Saliva_B	ARID3A
PMEPA1	4.05E-48	0.639917	0.219	0.118	6.47E-44	Saliva_B	PMEPA1
RPS6KB2	7.72E-48	0.542667	0.208	0.11	1.23E-43	Saliva_B	RPS6KB2
COX7A2	8.4E-48	0.305854	0.686	0.569	1.34E-43	Saliva_B	COX7A2
DDX181	1.31E-47	0.615382	0.32	0.202	2.09E-43	Saliva_B	DDX18
PREB	1.49E-47	0.500995	0.157	0.072	2.38E-43	Saliva_B	PREB
ARF4	1.82E-47	0.556523	0.525	0.416	2.91E-43	Saliva_B	ARF4
RP11.727F	2.5E-47	0.429714	0.106	0.039	3.99E-43	Saliva_B	RP11.727F15.9
LRRC591	5.23E-47	0.644772	0.245	0.143	8.34E-43	Saliva_B	LRRC59
SHMT2	9.42E-47	0.416628	0.147	0.065	1.5E-42	Saliva_B	SHMT2
ZRANB21	5.41E-46	0.626948	0.294	0.185	8.63E-42	Saliva_B	ZRANB2
SMAP2	7.18E-46	0.380009	0.151	0.067	1.15E-41	Saliva_B	SMAP2
PSMB91	7.36E-46	0.338027	0.296	0.167	1.17E-41	Saliva_B	PSMB9
EZR	7.87E-46	0.694999	0.399	0.28	1.26E-41	Saliva_B	EZR
TMEM243	9.36E-46	0.507641	0.231	0.127	1.49E-41	Saliva_B	TMEM243

CYCS	2.62E-45	0.286968	0.471	0.339	4.17E-41	Saliva_B	CYCS
PRADC1	4.2E-45	0.556758	0.17	0.084	6.7E-41	Saliva_B	PRADC1
PLA2G161	8.01E-45	0.547844	0.225	0.124	1.28E-40	Saliva_B	PLA2G16
TSPYL2	1.75E-44	0.527365	0.177	0.087	2.79E-40	Saliva_B	TSPYL2
PDIA51	2.08E-44	0.291462	0.083	0.028	3.32E-40	Saliva_B	PDIA5
CCDC112	3.18E-44	0.418404	0.134	0.059	5.08E-40	Saliva_B	CCDC112
RNH1	4.81E-44	0.557946	0.427	0.313	7.67E-40	Saliva_B	RNH1
NKTR1	5.05E-44	0.571753	0.283	0.174	8.06E-40	Saliva_B	NKTR
ATP5A1	8.36E-44	0.515392	0.435	0.311	1.33E-39	Saliva_B	ATP5A1
PSAT1	1E-43	0.361742	0.082	0.028	1.6E-39	Saliva_B	PSAT1
ENTPD11	1.01E-43	0.316977	0.114	0.045	1.61E-39	Saliva_B	ENTPD1
STT3A	1.26E-43	0.492182	0.141	0.065	2.01E-39	Saliva_B	STT3A
PSMA71	1.27E-43	0.433346	0.574	0.462	2.03E-39	Saliva_B	PSMA7
UFL1	1.28E-43	0.60924	0.206	0.114	2.05E-39	Saliva_B	UFL1
GNAI21	1.68E-43	0.488541	0.345	0.224	2.68E-39	Saliva_B	GNAI2
MCUR1	4.33E-43	0.614306	0.255	0.156	6.91E-39	Saliva_B	MCUR1
ACTA1	5.76E-43	0.496405	0.031	0.005	9.19E-39	Saliva_B	ACTA1
RHBDD1	2.32E-42	0.34724	0.074	0.024	3.7E-38	Saliva_B	RHBDD1
PIGK	2.78E-42	0.458956	0.133	0.059	4.44E-38	Saliva_B	PIGK
NAGK	3.68E-42	0.525948	0.218	0.124	5.86E-38	Saliva_B	NAGK
SLC33A1	3.7E-42	0.449917	0.123	0.053	5.89E-38	Saliva_B	SLC33A1
CREB3	4.23E-42	0.563638	0.225	0.13	6.74E-38	Saliva_B	CREB3
DYNLL11	4.64E-42	0.320723	0.695	0.559	7.4E-38	Saliva_B	DYNLL1
PPOX	5.19E-42	0.335635	0.086	0.031	8.27E-38	Saliva_B	PPOX
C5orf56	7.72E-42	0.342264	0.147	0.066	1.23E-37	Saliva_B	C5orf56
NEU1	8.39E-42	0.628421	0.244	0.147	1.34E-37	Saliva_B	NEU1
BASP1	1.18E-41	0.32515	0.171	0.082	1.88E-37	Saliva_B	BASP1
CHAC2	1.38E-41	0.259593	0.054	0.015	2.2E-37	Saliva_B	CHAC2
ATP2A3	1.71E-41	0.43085	0.124	0.054	2.72E-37	Saliva_B	ATP2A3
SURF41	1.87E-41	0.656857	0.273	0.176	2.99E-37	Saliva_B	SURF4
SLC35F2	2.54E-41	0.335019	0.082	0.029	4.05E-37	Saliva_B	SLC35F2
UBC	2.8E-41	0.313919	0.875	0.841	4.46E-37	Saliva_B	UBC
PARVB1	1.5E-40	0.334281	0.106	0.042	2.4E-36	Saliva_B	PARVB
ME2	1.73E-40	0.415323	0.11	0.045	2.76E-36	Saliva_B	ME2
TSTD11	4.4E-40	0.518008	0.389	0.27	7.02E-36	Saliva_B	TSTD1
CTA.293F1	4.92E-40	0.445347	0.099	0.04	7.84E-36	Saliva_B	CTA.293F17.1
LINC00152	7.39E-40	0.459919	0.289	0.176	1.18E-35	Saliva_B	LINC00152
NENF1	1.19E-39	0.540324	0.432	0.327	1.89E-35	Saliva_B	NENF
RRP361	1.55E-39	0.521599	0.126	0.057	2.47E-35	Saliva_B	RRP36
NHLRC3	3.23E-39	0.459427	0.147	0.071	5.15E-35	Saliva_B	NHLRC3
APOA1BP	4E-39	0.551208	0.223	0.131	6.38E-35	Saliva_B	APOA1BP
NXPE3	8.11E-39	0.262072	0.066	0.021	1.29E-34	Saliva_B	NXPE3
MRPL52	9.82E-39	0.623838	0.321	0.223	1.57E-34	Saliva_B	MRPL52
SF3B5	1.09E-38	0.506151	0.462	0.361	1.74E-34	Saliva_B	SF3B5
STOML2	1.23E-38	0.603522	0.272	0.175	1.97E-34	Saliva_B	STOML2
SMARCB1	2.6E-38	0.536369	0.219	0.127	4.15E-34	Saliva_B	SMARCB1
FCHSD2	3.13E-38	0.315748	0.08	0.028	5E-34	Saliva_B	FCHSD2

OGFOD3	5.83E-38	0.517583	0.159	0.082	9.31E-34	Saliva_B	OGFOD3
AGA	9.17E-38	0.521903	0.158	0.081	1.46E-33	Saliva_B	AGA
SYTL1	1.22E-37	0.45814	0.2	0.111	1.94E-33	Saliva_B	SYTL1
THEMIS2	1.33E-37	0.257731	0.1	0.04	2.13E-33	Saliva_B	THEMIS2
IFI62	1.43E-37	0.462874	0.259	0.153	2.27E-33	Saliva_B	IFI6
TEX9	1.64E-37	0.42461	0.094	0.037	2.61E-33	Saliva_B	TEX9
EVI2A	2.02E-37	0.292287	0.095	0.036	3.23E-33	Saliva_B	EVI2A
BRCA2	3.15E-37	0.287645	0.065	0.021	5.03E-33	Saliva_B	BRCA2
SAT2	4.16E-37	0.518297	0.302	0.203	6.64E-33	Saliva_B	SAT2
NDUFAF3	7.96E-37	0.449182	0.367	0.259	1.27E-32	Saliva_B	NDUFAF3
CCT8	1.02E-36	0.587308	0.303	0.203	1.62E-32	Saliva_B	CCT8
PARM1	1.13E-36	0.415193	0.116	0.052	1.81E-32	Saliva_B	PARM1
CALM12	1.71E-36	0.382827	0.73	0.65	2.74E-32	Saliva_B	CALM1
ESR2	2.25E-36	0.270999	0.051	0.015	3.59E-32	Saliva_B	ESR2
SEC631	2.37E-36	0.633705	0.26	0.17	3.78E-32	Saliva_B	SEC63
BHLHE41	3.41E-36	0.377647	0.104	0.044	5.44E-32	Saliva_B	BHLHE41
BLNK	5.48E-36	0.351982	0.109	0.047	8.75E-32	Saliva_B	BLNK
ITGAE	5.74E-36	0.496428	0.22	0.131	9.16E-32	Saliva_B	ITGAE
CCDC107	8.38E-36	0.468193	0.275	0.175	1.34E-31	Saliva_B	CCDC107
APRT	9.88E-36	0.358808	0.495	0.367	1.58E-31	Saliva_B	APRT
N4BP2L21	1.07E-35	0.55291	0.389	0.292	1.7E-31	Saliva_B	N4BP2L2
DNAJC211	2.19E-35	0.563883	0.203	0.119	3.5E-31	Saliva_B	DNAJC21
ERC1	4.45E-35	0.385585	0.114	0.052	7.09E-31	Saliva_B	ERC1
RAD9A	5.62E-35	0.388677	0.119	0.055	8.96E-31	Saliva_B	RAD9A
DOCK8	5.66E-35	0.324618	0.115	0.051	9.03E-31	Saliva_B	DOCK8
AKAP17A	6.98E-35	0.452974	0.171	0.093	1.11E-30	Saliva_B	AKAP17A
CHCHD2	9.06E-35	0.396563	0.709	0.619	1.45E-30	Saliva_B	CHCHD2
FDPS1	1.14E-34	0.535126	0.283	0.19	1.82E-30	Saliva_B	FDPS
N4BP2	1.46E-34	0.475343	0.113	0.051	2.33E-30	Saliva_B	N4BP2
DDHD1	1.49E-34	0.408647	0.119	0.054	2.38E-30	Saliva_B	DDHD1
SLC25A31	1.57E-34	0.410074	0.55	0.454	2.5E-30	Saliva_B	SLC25A3
SNRPN1	1.7E-34	0.539858	0.184	0.105	2.72E-30	Saliva_B	SNRPN
NFKBID	2.17E-34	0.322673	0.104	0.044	3.46E-30	Saliva_B	NFKBID
NAA101	2.51E-34	0.531022	0.25	0.158	4.01E-30	Saliva_B	NAA10
GMPPA	2.93E-34	0.390796	0.111	0.05	4.68E-30	Saliva_B	GMPPA
C19orf70	2.98E-34	0.26931	0.387	0.277	4.76E-30	Saliva_B	C19orf70
ZNF37A1	3.61E-34	0.432844	0.119	0.055	5.75E-30	Saliva_B	ZNF37A
C19orf24	3.76E-34	0.589444	0.278	0.187	6E-30	Saliva_B	C19orf24
UXT	4.54E-34	0.424743	0.47	0.368	7.24E-30	Saliva_B	UXT
PUS7L	6.73E-34	0.491096	0.137	0.069	1.07E-29	Saliva_B	PUS7L
TMEM107	1.24E-33	0.450889	0.139	0.07	1.98E-29	Saliva_B	TMEM107
MCTS1	1.27E-33	0.63857	0.232	0.149	2.02E-29	Saliva_B	MCTS1
CKLF	1.52E-33	0.41338	0.245	0.149	2.42E-29	Saliva_B	CKLF
RPS4Y11	1.85E-33	0.357709	0.401	0.271	2.96E-29	Saliva_B	RPS4Y1
SFT2D1	2.16E-33	0.579872	0.289	0.202	3.45E-29	Saliva_B	SFT2D1
TUFM	4.09E-33	0.511987	0.385	0.29	6.52E-29	Saliva_B	TUFM
LIMD1	7.2E-33	0.302429	0.071	0.026	1.15E-28	Saliva_B	LIMD1

PMVK	7.22E-33	0.488568	0.215	0.13	1.15E-28	Saliva_B	PMVK
YIPF5	1.39E-32	0.553123	0.195	0.116	2.21E-28	Saliva_B	YIPF5
CCDC53	1.39E-32	0.403822	0.228	0.136	2.22E-28	Saliva_B	CCDC53
TMED2	1.55E-32	0.49909	0.438	0.344	2.48E-28	Saliva_B	TMED2
STUB1	1.88E-32	0.504317	0.343	0.251	3E-28	Saliva_B	STUB1
IFI27L2	3.08E-32	0.397706	0.232	0.139	4.9E-28	Saliva_B	IFI27L2
RPL221	3.4E-32	0.261238	0.831	0.691	5.42E-28	Saliva_B	RPL22
PNKP	4.57E-32	0.464681	0.148	0.078	7.29E-28	Saliva_B	PNKP
TARSL2	4.7E-32	0.378523	0.108	0.049	7.5E-28	Saliva_B	TARSL2
USP16	5.74E-32	0.568363	0.263	0.177	9.15E-28	Saliva_B	USP16
PNISR1	6.2E-32	0.453144	0.443	0.346	9.88E-28	Saliva_B	PNISR
RHBDF2	8.07E-32	0.266323	0.069	0.025	1.29E-27	Saliva_B	RHBDF2
PEBP11	1.46E-31	0.338105	0.583	0.454	2.32E-27	Saliva_B	PEBP1
ANTXR2	1.94E-31	0.297785	0.082	0.033	3.1E-27	Saliva_B	ANTXR2
MDFIC	2.26E-31	0.425165	0.144	0.075	3.61E-27	Saliva_B	MDFIC
C12orf57	2.97E-31	0.452422	0.457	0.356	4.73E-27	Saliva_B	C12orf57
KLHL51	3.6E-31	0.317444	0.077	0.03	5.74E-27	Saliva_B	KLHL5
MYO5A	4.3E-31	0.33618	0.081	0.033	6.86E-27	Saliva_B	MYO5A
CKAP41	6.11E-31	0.495313	0.25	0.164	9.75E-27	Saliva_B	CKAP4
H1FX	8.17E-31	0.577646	0.354	0.259	1.3E-26	Saliva_B	H1FX
FAM129A1	1.4E-30	0.46375	0.18	0.103	2.24E-26	Saliva_B	FAM129A
NME31	1.44E-30	0.451679	0.301	0.208	2.3E-26	Saliva_B	NME3
CASP3	1.61E-30	0.470039	0.142	0.075	2.57E-26	Saliva_B	CASP3
TTC141	1.66E-30	0.442612	0.177	0.101	2.64E-26	Saliva_B	TTC14
TMEM214	1.71E-30	0.499711	0.137	0.072	2.73E-26	Saliva_B	TMEM214
UAP1	1.94E-30	0.529859	0.267	0.182	3.09E-26	Saliva_B	UAP1
IER21	2.22E-30	1.121119	0.564	0.546	3.53E-26	Saliva_B	IER2
RER1	2.37E-30	0.55042	0.421	0.34	3.77E-26	Saliva_B	RER1
COPZ1	3.66E-30	0.625586	0.297	0.216	5.84E-26	Saliva_B	COPZ1
MRPL54	4.09E-30	0.475893	0.296	0.208	6.53E-26	Saliva_B	MRPL54
PCMT1	4.25E-30	0.480062	0.307	0.216	6.78E-26	Saliva_B	PCMT1
PHACTR1	4.77E-30	0.488673	0.082	0.034	7.61E-26	Saliva_B	PHACTR1
TAF10	5.47E-30	0.57354	0.26	0.175	8.72E-26	Saliva_B	TAF10
FNBP1	7.68E-30	0.423389	0.21	0.125	1.23E-25	Saliva_B	FNBP1
SND1	1.29E-29	0.462231	0.173	0.101	2.06E-25	Saliva_B	SND1
NDUFS8	1.9E-29	0.49051	0.352	0.263	3.02E-25	Saliva_B	NDUFS8
SEC31A	1.96E-29	0.554058	0.296	0.215	3.12E-25	Saliva_B	SEC31A
FAM117A	2.11E-29	0.279422	0.082	0.034	3.37E-25	Saliva_B	FAM117A
DUSP22	3.22E-29	0.506096	0.158	0.089	5.13E-25	Saliva_B	DUSP22
C1GALT1C	3.42E-29	0.516884	0.149	0.083	5.45E-25	Saliva_B	C1GALT1C1
CADPS22	3.91E-29	0.430004	0.096	0.044	6.24E-25	Saliva_B	CADPS2
PAIP2B1	4.63E-29	0.502891	0.12	0.061	7.39E-25	Saliva_B	PAIP2B
PHF19	6.37E-29	0.278319	0.071	0.027	1.02E-24	Saliva_B	PHF19
MGAT11	8.75E-29	0.544366	0.296	0.212	1.4E-24	Saliva_B	MGAT1
PSMB2	1.12E-28	0.510268	0.288	0.205	1.79E-24	Saliva_B	PSMB2
ATPIF1	1.28E-28	0.369678	0.479	0.392	2.04E-24	Saliva_B	ATPIF1
MPC1	1.47E-28	0.529463	0.287	0.209	2.35E-24	Saliva_B	MPC1

FAM58A	1.61E-28	0.354941	0.125	0.063	2.57E-24	Saliva_B	FAM58A
GNL3	1.75E-28	0.517416	0.285	0.203	2.79E-24	Saliva_B	GNL3
WARS1	2.11E-28	0.435271	0.145	0.079	3.36E-24	Saliva_B	WARS
ALYREF1	2.91E-28	0.345239	0.104	0.049	4.64E-24	Saliva_B	ALYREF
RGS2	3.56E-28	0.378629	0.171	0.096	5.67E-24	Saliva_B	RGS2
HSPB11	4.68E-28	0.523756	0.209	0.133	7.47E-24	Saliva_B	HSPB11
TMEM160	5.35E-28	0.431937	0.291	0.203	8.54E-24	Saliva_B	TMEM160
SLC25A42	5.74E-28	0.267896	0.063	0.023	9.16E-24	Saliva_B	SLC25A42
COMTD1	8.23E-28	0.526955	0.219	0.144	1.31E-23	Saliva_B	COMTD1
TMEM230	8.57E-28	0.500336	0.351	0.266	1.37E-23	Saliva_B	TMEM230
MPHOSPH	8.76E-28	0.43736	0.34	0.253	1.4E-23	Saliva_B	MPHOSPH8
PGRMC2	9.04E-28	0.581799	0.216	0.141	1.44E-23	Saliva_B	PGRMC2
SCARB21	1.72E-27	0.552139	0.231	0.156	2.74E-23	Saliva_B	SCARB2
USP48	1.77E-27	0.468307	0.163	0.095	2.82E-23	Saliva_B	USP48
EIF3A	3.47E-27	0.584675	0.365	0.292	5.53E-23	Saliva_B	EIF3A
EPSTI1	3.87E-27	0.32084	0.098	0.046	6.17E-23	Saliva_B	EPSTI1
UBALD2	4.04E-27	0.572279	0.249	0.17	6.44E-23	Saliva_B	UBALD2
CHD91	5.26E-27	0.486968	0.244	0.166	8.38E-23	Saliva_B	CHD9
ANKRD36	7.74E-27	0.447817	0.125	0.066	1.23E-22	Saliva_B	ANKRD36
ERGIC11	1.02E-26	0.491663	0.205	0.132	1.63E-22	Saliva_B	ERGIC1
GALNT2	1.16E-26	0.375239	0.111	0.055	1.85E-22	Saliva_B	GALNT2
GCSH	1.36E-26	0.539114	0.196	0.125	2.17E-22	Saliva_B	GCSH
EIF5B	1.67E-26	0.45097	0.361	0.276	2.67E-22	Saliva_B	EIF5B
IGSF8	1.91E-26	0.326795	0.113	0.056	3.04E-22	Saliva_B	IGSF8
ALG2	2.25E-26	0.453426	0.135	0.075	3.59E-22	Saliva_B	ALG2
CORO7	2.3E-26	0.271198	0.081	0.035	3.66E-22	Saliva_B	CORO7
PTGES3	2.45E-26	0.452935	0.478	0.392	3.91E-22	Saliva_B	PTGES3
UCHL5	4.23E-26	0.400328	0.122	0.064	6.75E-22	Saliva_B	UCHL5
MT.ATP82	5.48E-26	0.399957	0.21	0.134	8.74E-22	Saliva_B	MT.ATP8
RBX1	1.15E-25	0.490411	0.424	0.348	1.84E-21	Saliva_B	RBX1
HYI	2.05E-25	0.471187	0.148	0.086	3.27E-21	Saliva_B	HYI
P4HTM	2.43E-25	0.421914	0.136	0.075	3.87E-21	Saliva_B	P4HTM
DGKD	3E-25	0.276001	0.076	0.032	4.79E-21	Saliva_B	DGKD
IDS	3.21E-25	0.523747	0.262	0.184	5.12E-21	Saliva_B	IDS
MOGS	4.57E-25	0.35854	0.118	0.061	7.29E-21	Saliva_B	MOGS
PJA21	6.1E-25	0.497391	0.223	0.15	9.73E-21	Saliva_B	PJA2
SMCHD1	8.69E-25	0.428055	0.195	0.123	1.39E-20	Saliva_B	SMCHD1
TXNDC12	9.17E-25	0.446464	0.211	0.139	1.46E-20	Saliva_B	TXNDC12
AARS	1.34E-24	0.419433	0.102	0.051	2.14E-20	Saliva_B	AARS
TMEM167	1.38E-24	0.545257	0.305	0.235	2.2E-20	Saliva_B	TMEM167A
PSMD13	1.46E-24	0.438785	0.232	0.158	2.33E-20	Saliva_B	PSMD13
SRSF71	1.58E-24	0.483378	0.422	0.342	2.52E-20	Saliva_B	SRSF7
RBM251	1.81E-24	0.486557	0.317	0.238	2.89E-20	Saliva_B	RBM25
STARD5	2.27E-24	0.25157	0.053	0.02	3.62E-20	Saliva_B	STARD5
KDELR31	2.58E-24	0.456207	0.146	0.085	4.11E-20	Saliva_B	KDELR3
EBAG9	3.07E-24	0.470939	0.175	0.109	4.89E-20	Saliva_B	EBAG9
PLEKHH3	3.96E-24	0.315727	0.116	0.061	6.31E-20	Saliva_B	PLEKHH3

ORMDL2	4.35E-24	0.585973	0.25	0.183	6.93E-20	Saliva_B	ORMDL2
GALK1	4.83E-24	0.346488	0.12	0.064	7.7E-20	Saliva_B	GALK1
COPG11	5.05E-24	0.460669	0.156	0.094	8.06E-20	Saliva_B	COPG1
PPP1R35	5.09E-24	0.422228	0.144	0.083	8.12E-20	Saliva_B	PPP1R35
MAGED2	5.69E-24	0.483695	0.23	0.154	9.07E-20	Saliva_B	MAGED2
KCNQ1OT1	6.87E-24	0.542418	0.211	0.141	1.1E-19	Saliva_B	KCNQ1OT1
RBM6	6.88E-24	0.468426	0.177	0.11	1.1E-19	Saliva_B	RBM6
ARPC2	8.6E-24	0.282696	0.647	0.573	1.37E-19	Saliva_B	ARPC2
SH3GLB1	9.1E-24	0.503945	0.348	0.28	1.45E-19	Saliva_B	SH3GLB1
HINT2	2.27E-23	0.518685	0.241	0.171	3.62E-19	Saliva_B	HINT2
COPB21	2.31E-23	0.530032	0.222	0.155	3.69E-19	Saliva_B	COPB2
MIA3	2.84E-23	0.420625	0.179	0.111	4.52E-19	Saliva_B	MIA3
GNPTG	3.78E-23	0.422947	0.152	0.09	6.03E-19	Saliva_B	GNPTG
MAGEH11	3.92E-23	0.278565	0.094	0.045	6.26E-19	Saliva_B	MAGEH1
DUSP2	4.43E-23	0.684054	0.198	0.124	7.07E-19	Saliva_B	DUSP2
PSMB3	6.22E-23	0.437404	0.413	0.343	9.92E-19	Saliva_B	PSMB3
LSM31	1.26E-22	0.475691	0.367	0.303	2E-18	Saliva_B	LSM3
MARCKS	1.36E-22	0.406478	0.363	0.282	2.18E-18	Saliva_B	MARCKS
SETDB2	1.39E-22	0.265932	0.071	0.031	2.22E-18	Saliva_B	SETDB2
BET1	1.43E-22	0.477927	0.171	0.109	2.28E-18	Saliva_B	BET1
XRN1	1.51E-22	0.499698	0.158	0.097	2.41E-18	Saliva_B	XRN1
EDEM1	1.61E-22	0.416723	0.146	0.086	2.56E-18	Saliva_B	EDEM1
HNRNPUL1	1.86E-22	0.537225	0.231	0.162	2.96E-18	Saliva_B	HNRNPUL1
VCP	2.01E-22	0.495014	0.311	0.244	3.21E-18	Saliva_B	VCP
COPA	2.17E-22	0.52719	0.211	0.145	3.46E-18	Saliva_B	COPA
SP3	3.55E-22	0.434901	0.153	0.092	5.67E-18	Saliva_B	SP3
ATP13A1	3.77E-22	0.344901	0.085	0.041	6E-18	Saliva_B	ATP13A1
ATM	6.22E-22	0.373807	0.151	0.09	9.92E-18	Saliva_B	ATM
SS18L2	7.35E-22	0.431098	0.195	0.129	1.17E-17	Saliva_B	SS18L2
CDC42SE2	7.83E-22	0.392839	0.186	0.119	1.25E-17	Saliva_B	CDC42SE2
PIGP	9.28E-22	0.458012	0.208	0.143	1.48E-17	Saliva_B	PIGP
MX11	1.45E-21	0.339236	0.111	0.059	2.31E-17	Saliva_B	MX1
WFS1	1.73E-21	0.290612	0.068	0.03	2.76E-17	Saliva_B	WFS1
HID11	2.36E-21	0.340695	0.091	0.046	3.77E-17	Saliva_B	HID1
CASP8	3.83E-21	0.274136	0.083	0.04	6.11E-17	Saliva_B	CASP8
FNDC3B	3.99E-21	0.501116	0.214	0.149	6.36E-17	Saliva_B	FNDC3B
C17orf62	4.09E-21	0.307178	0.151	0.09	6.53E-17	Saliva_B	C17orf62
MBTPS1	4.36E-21	0.40143	0.142	0.085	6.95E-17	Saliva_B	MBTPS1
GAB1	4.6E-21	0.266776	0.077	0.036	7.34E-17	Saliva_B	GAB1
CERCAM	5.57E-21	0.351589	0.106	0.057	8.89E-17	Saliva_B	CERCAM
EIF2AK3	5.92E-21	0.309822	0.088	0.044	9.44E-17	Saliva_B	EIF2AK3
YIPF31	6.65E-21	0.469986	0.295	0.233	1.06E-16	Saliva_B	YIPF3
METTL23	6.73E-21	0.416636	0.188	0.124	1.07E-16	Saliva_B	METTL23
TSPAN31	8E-21	0.363389	0.124	0.071	1.28E-16	Saliva_B	TSPAN31
GGH	8.92E-21	0.414799	0.137	0.081	1.42E-16	Saliva_B	GGH
CHMP4A	9.77E-21	0.335549	0.238	0.164	1.56E-16	Saliva_B	CHMP4A
COPB1	1.19E-20	0.474467	0.2	0.139	1.89E-16	Saliva_B	COPB1

FAM173A	1.46E-20	0.375247	0.204	0.137	2.32E-16	Saliva_B	FAM173A
CXXC5	1.47E-20	0.397345	0.222	0.152	2.35E-16	Saliva_B	CXXC5
NDUFB4	1.67E-20	0.377139	0.51	0.445	2.67E-16	Saliva_B	NDUFB4
PIGT	1.99E-20	0.458179	0.229	0.163	3.18E-16	Saliva_B	PIGT
MDM4	2.56E-20	0.518395	0.199	0.136	4.08E-16	Saliva_B	MDM4
ANXA5	2.68E-20	0.260569	0.414	0.316	4.27E-16	Saliva_B	ANXA5
TNFAIP8	2.82E-20	0.251918	0.17	0.104	4.49E-16	Saliva_B	TNFAIP8
MRPS16	3.15E-20	0.470085	0.216	0.154	5.02E-16	Saliva_B	MRPS16
PRDM1	3.67E-20	0.277747	0.251	0.169	5.85E-16	Saliva_B	PRDM1
POMP	4.08E-20	0.347588	0.471	0.402	6.51E-16	Saliva_B	POMP
CLINT1	5.3E-20	0.533311	0.236	0.174	8.46E-16	Saliva_B	CLINT1
RNF181	6.4E-20	0.486729	0.36	0.302	1.02E-15	Saliva_B	RNF181
B4GALT3	7.06E-20	0.407943	0.143	0.088	1.13E-15	Saliva_B	B4GALT3
PRDX5	7.26E-20	0.328746	0.553	0.49	1.16E-15	Saliva_B	PRDX5
ADI11	9.87E-20	0.491726	0.284	0.221	1.57E-15	Saliva_B	ADI1
CTBS1	1.07E-19	0.471485	0.158	0.102	1.7E-15	Saliva_B	CTBS
P4HA1	1.16E-19	0.334609	0.138	0.082	1.85E-15	Saliva_B	P4HA1
MCL1	1.7E-19	0.497781	0.434	0.38	2.71E-15	Saliva_B	MCL1
MAP3K12	1.82E-19	0.39406	0.116	0.067	2.9E-15	Saliva_B	MAP3K1
BCLAF11	1.9E-19	0.490223	0.262	0.198	3.02E-15	Saliva_B	BCLAF1
C1orf1221	1.96E-19	0.559076	0.244	0.183	3.13E-15	Saliva_B	C1orf122
WDR54	2.12E-19	0.26278	0.114	0.063	3.38E-15	Saliva_B	WDR54
UBE2D2	3.54E-19	0.380726	0.409	0.343	5.65E-15	Saliva_B	UBE2D2
TMEM50B	6.47E-19	0.480608	0.214	0.153	1.03E-14	Saliva_B	TMEM50B
SRSF21	6.49E-19	0.362473	0.401	0.331	1.03E-14	Saliva_B	SRSF2
OCIAD11	7.2E-19	0.479168	0.312	0.252	1.15E-14	Saliva_B	OCIAD1
CSDE1	7.37E-19	0.504963	0.41	0.358	1.18E-14	Saliva_B	CSDE1
DDT	7.74E-19	0.414927	0.397	0.34	1.23E-14	Saliva_B	DDT
STRBP	8.43E-19	0.499893	0.131	0.08	1.34E-14	Saliva_B	STRBP
BLOC1S6	1.02E-18	0.399286	0.15	0.095	1.63E-14	Saliva_B	BLOC1S6
ARL14EP	1.24E-18	0.316412	0.138	0.084	1.98E-14	Saliva_B	ARL14EP
KMT2A1	1.67E-18	0.468483	0.189	0.13	2.66E-14	Saliva_B	KMT2A
ADAR1	1.95E-18	0.375997	0.196	0.135	3.1E-14	Saliva_B	ADAR
PLOD31	2.37E-18	0.25654	0.078	0.039	3.78E-14	Saliva_B	PLOD3
CHPF2	3.05E-18	0.284648	0.077	0.038	4.86E-14	Saliva_B	CHPF2
ATRX1	3.63E-18	0.432308	0.269	0.206	5.79E-14	Saliva_B	ATRX
SSBP11	3.67E-18	0.448978	0.344	0.282	5.85E-14	Saliva_B	SSBP1
HSPE1	5.53E-18	0.295045	0.444	0.371	8.83E-14	Saliva_B	HSPE1
TMEM218	6.81E-18	0.266464	0.094	0.051	1.09E-13	Saliva_B	TMEM218
CNIH4	8.14E-18	0.43521	0.195	0.136	1.3E-13	Saliva_B	CNIH4
HEXB1	8.6E-18	0.457729	0.222	0.161	1.37E-13	Saliva_B	HEXB
C14orf166	8.99E-18	0.368115	0.398	0.337	1.43E-13	Saliva_B	C14orf166
UFD1L	1.25E-17	0.437045	0.239	0.178	2E-13	Saliva_B	UFD1L
USE1	1.3E-17	0.36294	0.168	0.112	2.07E-13	Saliva_B	USE1
EGR11	1.33E-17	0.382388	0.24	0.172	2.12E-13	Saliva_B	EGR1
AAED11	1.56E-17	0.298318	0.127	0.075	2.49E-13	Saliva_B	AAED1
INSIG2	1.59E-17	0.327781	0.115	0.068	2.53E-13	Saliva_B	INSIG2

RUNX2	2.05E-17	0.281427	0.078	0.039	3.27E-13	Saliva_B	RUNX2
HIPK21	2.42E-17	0.450058	0.127	0.079	3.86E-13	Saliva_B	HIPK2
PSMB5	2.77E-17	0.444194	0.295	0.24	4.43E-13	Saliva_B	PSMB5
RAN	2.8E-17	0.294713	0.44	0.366	4.46E-13	Saliva_B	RAN
MPHOSPH	3.08E-17	0.330532	0.141	0.089	4.91E-13	Saliva_B	MPHOSPH10
NCLN	3.25E-17	0.307271	0.098	0.055	5.18E-13	Saliva_B	NCLN
GOLT1B	4.06E-17	0.426035	0.147	0.096	6.48E-13	Saliva_B	GOLT1B
EIF2A	7.78E-17	0.4963	0.209	0.153	1.24E-12	Saliva_B	EIF2A
RNPEPL1	8.84E-17	0.347406	0.164	0.109	1.41E-12	Saliva_B	RNPEPL1
USP141	2.33E-16	0.376547	0.151	0.1	3.71E-12	Saliva_B	USP14
NDUFV2	2.48E-16	0.314125	0.438	0.385	3.96E-12	Saliva_B	NDUFV2
TRIP112	2.61E-16	0.445623	0.172	0.12	4.17E-12	Saliva_B	TRIP11
TCN21	2.96E-16	0.270671	0.07	0.035	4.73E-12	Saliva_B	TCN2
PHC3	3.67E-16	0.317504	0.097	0.055	5.85E-12	Saliva_B	PHC3
GALK2	4.1E-16	0.292611	0.076	0.039	6.54E-12	Saliva_B	GALK2
SNX29	5.19E-16	0.318192	0.106	0.062	8.27E-12	Saliva_B	SNX29
METTL5	6.35E-16	0.352727	0.175	0.12	1.01E-11	Saliva_B	METTL5
PHYKPL	7.79E-16	0.267388	0.115	0.069	1.24E-11	Saliva_B	PHYKPL
PHB2	7.79E-16	0.367415	0.255	0.195	1.24E-11	Saliva_B	PHB2
PSMB4	8.14E-16	0.446497	0.235	0.179	1.3E-11	Saliva_B	PSMB4
KCNK61	9.07E-16	0.414024	0.149	0.1	1.45E-11	Saliva_B	KCNK6
IMPDH1	1.26E-15	0.25197	0.09	0.05	2E-11	Saliva_B	IMPDH1
NSMCE4A1	2.07E-15	0.374287	0.128	0.081	3.3E-11	Saliva_B	NSMCE4A
HSCB	2.95E-15	0.275567	0.096	0.055	4.71E-11	Saliva_B	HSCB
SLC38A11	4.11E-15	0.357248	0.148	0.099	6.55E-11	Saliva_B	SLC38A1
SUCO	4.5E-15	0.379169	0.138	0.092	7.18E-11	Saliva_B	SUCO
B9D1	5E-15	0.261892	0.085	0.047	7.97E-11	Saliva_B	B9D1
CEP350	7.26E-15	0.332669	0.181	0.127	1.16E-10	Saliva_B	CEP350
SEC13	7.99E-15	0.496303	0.183	0.134	1.27E-10	Saliva_B	SEC13
ZC2HC1A	1.2E-14	0.288357	0.083	0.046	1.91E-10	Saliva_B	ZC2HC1A
EXOSC1	1.28E-14	0.373859	0.139	0.091	2.04E-10	Saliva_B	EXOSC1
PSMA5	1.29E-14	0.412073	0.235	0.182	2.06E-10	Saliva_B	PSMA5
IFT27	1.29E-14	0.357111	0.125	0.08	2.06E-10	Saliva_B	IFT27
B3GAT3	1.41E-14	0.29442	0.128	0.081	2.26E-10	Saliva_B	B3GAT3
DNAJC7	1.47E-14	0.3973	0.212	0.159	2.34E-10	Saliva_B	DNAJC7
ANAPC16	1.74E-14	0.293339	0.424	0.37	2.77E-10	Saliva_B	ANAPC16
C11orf80	1.79E-14	0.26142	0.067	0.035	2.86E-10	Saliva_B	C11orf80
SMIM7	1.8E-14	0.435607	0.231	0.181	2.87E-10	Saliva_B	SMIM7
GOLGA3	2.07E-14	0.393913	0.133	0.088	3.3E-10	Saliva_B	GOLGA3
DESI1	2.07E-14	0.322862	0.143	0.095	3.31E-10	Saliva_B	DESI1
LAP31	2.29E-14	0.430961	0.174	0.125	3.65E-10	Saliva_B	LAP3
UTP3	2.35E-14	0.310047	0.086	0.049	3.74E-10	Saliva_B	UTP3
SIAH2	2.39E-14	0.381169	0.156	0.105	3.81E-10	Saliva_B	SIAH2
DPAGT1	2.81E-14	0.278951	0.077	0.043	4.49E-10	Saliva_B	DPAGT1
SP1001	3.07E-14	0.321718	0.194	0.139	4.89E-10	Saliva_B	SP100
ATG4A	3.08E-14	0.256876	0.072	0.038	4.91E-10	Saliva_B	ATG4A
HMGN31	3.37E-14	0.314776	0.335	0.278	5.37E-10	Saliva_B	HMGN3

IRF3	3.72E-14	0.300283	0.148	0.099	5.94E-10	Saliva_B	IRF3
PPP1CA	3.87E-14	0.311329	0.366	0.313	6.17E-10	Saliva_B	PPP1CA
ASCC3	4.34E-14	0.33494	0.099	0.06	6.92E-10	Saliva_B	ASCC3
TXNL1	4.72E-14	0.468992	0.292	0.25	7.53E-10	Saliva_B	TXNL1
DNAJC10	5.32E-14	0.423996	0.167	0.117	8.49E-10	Saliva_B	DNAJC10
PQBP1	5.61E-14	0.301636	0.209	0.153	8.95E-10	Saliva_B	PQBP1
MED10	5.93E-14	0.365073	0.238	0.184	9.46E-10	Saliva_B	MED10
DTWD1	6.46E-14	0.348896	0.124	0.08	1.03E-09	Saliva_B	DTWD1
MBNL2	7.01E-14	0.414907	0.166	0.118	1.12E-09	Saliva_B	MBNL2
GPATCH11	8.52E-14	0.252506	0.073	0.039	1.36E-09	Saliva_B	GPATCH11
USMG5	8.77E-14	0.273469	0.488	0.436	1.4E-09	Saliva_B	USMG5
IARS	8.99E-14	0.368583	0.113	0.071	1.43E-09	Saliva_B	IARS
WDR83OS	9.82E-14	0.32071	0.414	0.368	1.57E-09	Saliva_B	WDR83OS
POP5	1.23E-13	0.323614	0.144	0.097	1.96E-09	Saliva_B	POP5
FUOM	1.44E-13	0.334584	0.12	0.078	2.3E-09	Saliva_B	FUOM
AIP	1.65E-13	0.27594	0.183	0.128	2.63E-09	Saliva_B	AIP
SARS	1.74E-13	0.380173	0.205	0.155	2.77E-09	Saliva_B	SARS
DDX241	1.81E-13	0.316387	0.296	0.237	2.88E-09	Saliva_B	DDX24
IMP4	2.07E-13	0.414163	0.182	0.134	3.29E-09	Saliva_B	IMP4
RAD50	3.07E-13	0.395818	0.13	0.087	4.9E-09	Saliva_B	RAD50
CYLD	3.39E-13	0.365642	0.173	0.124	5.41E-09	Saliva_B	CYLD
SDF4	3.4E-13	0.434276	0.258	0.208	5.42E-09	Saliva_B	SDF4
CPNE1	3.42E-13	0.339364	0.179	0.127	5.46E-09	Saliva_B	CPNE1
ARCN1	4.07E-13	0.404738	0.16	0.115	6.49E-09	Saliva_B	ARCN1
TMEM39A	5.93E-13	0.280443	0.082	0.048	9.46E-09	Saliva_B	TMEM39A
U2SURP1	6.12E-13	0.378024	0.222	0.171	9.75E-09	Saliva_B	U2SURP
HCG18	6.13E-13	0.2696	0.102	0.063	9.78E-09	Saliva_B	HCG18
LTN1	6.78E-13	0.37706	0.122	0.081	1.08E-08	Saliva_B	LTN1
ZNF593	7.83E-13	0.365078	0.175	0.129	1.25E-08	Saliva_B	ZNF593
RBBP4	1.07E-12	0.381906	0.197	0.149	1.7E-08	Saliva_B	RBBP4
CFLAR1	1.22E-12	0.419053	0.319	0.271	1.95E-08	Saliva_B	CFLAR
MAGT1	1.26E-12	0.452826	0.174	0.13	2.01E-08	Saliva_B	MAGT1
CLPP	1.38E-12	0.339145	0.198	0.15	2.21E-08	Saliva_B	CLPP
DCTN3	1.53E-12	0.330045	0.282	0.234	2.44E-08	Saliva_B	DCTN3
SH3GLB2	1.56E-12	0.391118	0.152	0.108	2.49E-08	Saliva_B	SH3GLB2
TFG	1.6E-12	0.49381	0.23	0.188	2.56E-08	Saliva_B	TFG
ASAH1	1.87E-12	0.392883	0.32	0.278	2.99E-08	Saliva_B	ASAH1
C17orf89	2.09E-12	0.405646	0.237	0.19	3.33E-08	Saliva_B	C17orf89
THAP7	2.68E-12	0.352735	0.134	0.091	4.28E-08	Saliva_B	THAP7
RUFY3	2.8E-12	0.316749	0.114	0.074	4.47E-08	Saliva_B	RUFY3
ALG3	3.56E-12	0.392646	0.142	0.1	5.68E-08	Saliva_B	ALG3
TSPAN3	3.59E-12	0.373569	0.218	0.171	5.73E-08	Saliva_B	TSPAN3
GANAB	4.83E-12	0.402066	0.146	0.103	7.7E-08	Saliva_B	GANAB
IDH2	4.97E-12	0.426396	0.239	0.194	7.93E-08	Saliva_B	IDH2
VPS36	5.62E-12	0.371762	0.156	0.113	8.96E-08	Saliva_B	VPS36
CBWD5	6.99E-12	0.261743	0.071	0.04	1.12E-07	Saliva_B	CBWD5
DDRGK1	7.99E-12	0.36682	0.196	0.151	1.27E-07	Saliva_B	DDRGK1

OGT	1.15E-11	0.38978	0.148	0.106	1.83E-07	Saliva_B	OGT
COX6A1	1.34E-11	0.271014	0.569	0.531	2.14E-07	Saliva_B	COX6A1
TTC371	1.35E-11	0.353296	0.124	0.085	2.15E-07	Saliva_B	TTC37
ECI2	1.52E-11	0.335043	0.137	0.096	2.43E-07	Saliva_B	ECI2
TIMM17A	1.6E-11	0.4611	0.177	0.135	2.55E-07	Saliva_B	TIMM17A
MAP41	2.03E-11	0.366235	0.172	0.129	3.23E-07	Saliva_B	MAP4
GTF2I2	2.07E-11	0.367034	0.197	0.153	3.3E-07	Saliva_B	GTF2I
FDX1	2.14E-11	0.415268	0.208	0.166	3.41E-07	Saliva_B	FDX1
TMEM220	2.17E-11	0.330942	0.085	0.052	3.47E-07	Saliva_B	TMEM220
CLIC41	2.45E-11	0.307915	0.17	0.125	3.91E-07	Saliva_B	CLIC4
NFKB2	2.5E-11	0.282758	0.117	0.078	3.99E-07	Saliva_B	NFKB2
EPM2AIP1	2.53E-11	0.302924	0.11	0.072	4.04E-07	Saliva_B	EPM2AIP1
CCS	2.95E-11	0.321453	0.153	0.11	4.7E-07	Saliva_B	CCS
EBP	3.39E-11	0.363479	0.161	0.118	5.41E-07	Saliva_B	EBP
NFX1	3.53E-11	0.259117	0.08	0.048	5.64E-07	Saliva_B	NFX1
PTP4A2	3.56E-11	0.326446	0.334	0.292	5.67E-07	Saliva_B	PTP4A2
BMI1	3.85E-11	0.287268	0.094	0.059	6.14E-07	Saliva_B	BMI1
CASC4	4.35E-11	0.410771	0.163	0.122	6.94E-07	Saliva_B	CASC4
SLC22A17	5.33E-11	0.259222	0.071	0.041	8.5E-07	Saliva_B	SLC22A17
AC159540.	5.7E-11	0.271515	0.096	0.062	9.08E-07	Saliva_B	AC159540.1
CEP2901	7.51E-11	0.254143	0.08	0.048	1.2E-06	Saliva_B	CEP290
DAP1	7.71E-11	0.431255	0.183	0.144	1.23E-06	Saliva_B	DAP
HDDC3	9.4E-11	0.286611	0.099	0.064	1.5E-06	Saliva_B	HDDC3
HNRNPAB	1.1E-10	0.371106	0.21	0.166	1.76E-06	Saliva_B	HNRNPAB
THAP5	1.15E-10	0.284506	0.115	0.077	1.83E-06	Saliva_B	THAP5
TMEM256	1.17E-10	0.371386	0.26	0.219	1.86E-06	Saliva_B	TMEM256
MRPS33	1.28E-10	0.384432	0.189	0.148	2.05E-06	Saliva_B	MRPS33
EVI5	1.28E-10	0.359592	0.114	0.078	2.05E-06	Saliva_B	EVI5
MFSD12	1.32E-10	0.35621	0.13	0.092	2.1E-06	Saliva_B	MFSD12
MCEE	1.62E-10	0.348166	0.088	0.056	2.58E-06	Saliva_B	MCEE
TIMM17B	1.74E-10	0.356425	0.159	0.118	2.78E-06	Saliva_B	TIMM17B
HSD17B10	1.85E-10	0.262192	0.215	0.169	2.95E-06	Saliva_B	HSD17B10
TRIM27	2.47E-10	0.288629	0.099	0.065	3.94E-06	Saliva_B	TRIM27
KMT2B	2.55E-10	0.266936	0.077	0.047	4.06E-06	Saliva_B	KMT2B
SETX1	2.94E-10	0.365757	0.14	0.101	4.68E-06	Saliva_B	SETX
COA1	3.46E-10	0.300782	0.14	0.101	5.51E-06	Saliva_B	COA1
POP7	3.55E-10	0.39877	0.178	0.139	5.66E-06	Saliva_B	POP7
THRAP31	3.61E-10	0.339916	0.218	0.175	5.76E-06	Saliva_B	THRAP3
NAPG	3.92E-10	0.34161	0.114	0.079	6.26E-06	Saliva_B	NAPG
CYB561D2	5.97E-10	0.277641	0.105	0.07	9.52E-06	Saliva_B	CYB561D2
ENTPD4	6.69E-10	0.285019	0.094	0.061	1.07E-05	Saliva_B	ENTPD4
PQLC3	7.14E-10	0.256669	0.13	0.091	1.14E-05	Saliva_B	PQLC3
ANXA7	8.09E-10	0.347155	0.236	0.195	1.29E-05	Saliva_B	ANXA7
LINC00969	8.37E-10	0.304756	0.15	0.111	1.33E-05	Saliva_B	LINC00969
SLC2A4RG	1.08E-09	0.28208	0.137	0.098	1.72E-05	Saliva_B	SLC2A4RG
SPOP	1.7E-09	0.287016	0.152	0.114	2.71E-05	Saliva_B	SPOP
ENY21	1.77E-09	0.284705	0.353	0.321	2.82E-05	Saliva_B	ENY2

SEC23B	1.89E-09	0.386052	0.146	0.111	3.01E-05	Saliva_B	SEC23B
PGPEP1	2.07E-09	0.268597	0.08	0.051	3.3E-05	Saliva_B	PGPEP1
HBS1L	2.2E-09	0.320447	0.102	0.07	3.51E-05	Saliva_B	HBS1L
ZNHIT1	2.2E-09	0.347569	0.326	0.295	3.51E-05	Saliva_B	ZNHIT1
METRN1	2.45E-09	0.331337	0.128	0.093	3.91E-05	Saliva_B	METRN
UBE2I	2.61E-09	0.339936	0.298	0.263	4.17E-05	Saliva_B	UBE2I
QTRT1	3.07E-09	0.327296	0.108	0.074	4.89E-05	Saliva_B	QTRT1
UQCRQ1	3.22E-09	0.279501	0.527	0.499	5.14E-05	Saliva_B	UQCRQ
SLC35E3	3.24E-09	0.262621	0.074	0.047	5.16E-05	Saliva_B	SLC35E3
TM9SF21	3.3E-09	0.490874	0.226	0.194	5.26E-05	Saliva_B	TM9SF2
ZCCHC7	3.39E-09	0.308455	0.11	0.076	5.4E-05	Saliva_B	ZCCHC7
MYEOV2	3.4E-09	0.303205	0.39	0.361	5.42E-05	Saliva_B	MYEOV2
ARF1	3.5E-09	0.288779	0.479	0.466	5.59E-05	Saliva_B	ARF1
PRPF40A	3.79E-09	0.343195	0.225	0.187	6.05E-05	Saliva_B	PRPF40A
WDR33	3.9E-09	0.394009	0.188	0.151	6.22E-05	Saliva_B	WDR33
JOSD2	4.45E-09	0.295067	0.155	0.117	7.1E-05	Saliva_B	JOSD2
AKAP11	4.51E-09	0.274779	0.085	0.055	7.2E-05	Saliva_B	AKAP11
COX17	4.73E-09	0.314403	0.308	0.276	7.55E-05	Saliva_B	COX17
PTRH2	5.17E-09	0.287932	0.071	0.044	8.24E-05	Saliva_B	PTRH2
NSA2	5.32E-09	0.338504	0.285	0.249	8.48E-05	Saliva_B	NSA2
PSENEN	5.69E-09	0.415166	0.252	0.217	9.08E-05	Saliva_B	PSENEN
C11orf1	5.96E-09	0.263489	0.135	0.099	9.5E-05	Saliva_B	C11orf1
KIF13B	5.99E-09	0.318922	0.081	0.053	9.56E-05	Saliva_B	KIF13B
RBM33	6.89E-09	0.345673	0.119	0.086	0.00011	Saliva_B	RBM33
SLC7A1	7.27E-09	0.252175	0.077	0.049	0.000116	Saliva_B	SLC7A1
TXLNA	7.88E-09	0.28781	0.082	0.054	0.000126	Saliva_B	TXLNA
IER3IP1	7.99E-09	0.424716	0.176	0.143	0.000127	Saliva_B	IER3IP1
EIF2AK1	8.18E-09	0.294789	0.137	0.102	0.00013	Saliva_B	EIF2AK1
IQGAP11	8.3E-09	0.342137	0.29	0.258	0.000132	Saliva_B	IQGAP1
GLA	9.04E-09	0.31811	0.123	0.088	0.000144	Saliva_B	GLA
LCORL	9.29E-09	0.266453	0.073	0.046	0.000148	Saliva_B	LCORL
NOP56	9.72E-09	0.337814	0.163	0.124	0.000155	Saliva_B	NOP56
C11orf73	1.24E-08	0.295155	0.168	0.13	0.000197	Saliva_B	C11orf73
TMEM70	1.29E-08	0.342624	0.148	0.113	0.000206	Saliva_B	TMEM70
MRPL3	1.33E-08	0.349103	0.166	0.132	0.000212	Saliva_B	MRPL3
SF1	1.59E-08	0.292611	0.271	0.234	0.000254	Saliva_B	SF1
EIF2S2	1.73E-08	0.35022	0.365	0.342	0.000277	Saliva_B	EIF2S2
PTPN2	1.96E-08	0.301754	0.152	0.117	0.000312	Saliva_B	PTPN2
RAD17	2.4E-08	0.295714	0.084	0.056	0.000383	Saliva_B	RAD17
SNHG12	2.63E-08	0.26314	0.114	0.082	0.00042	Saliva_B	SNHG12
BRD2	2.9E-08	0.35216	0.302	0.271	0.000462	Saliva_B	BRD2
NUDT8	3.41E-08	0.250567	0.085	0.057	0.000544	Saliva_B	NUDT8
GTF3C6	3.56E-08	0.3772	0.232	0.202	0.000568	Saliva_B	GTF3C6
PDCD41	3.7E-08	0.324792	0.219	0.184	0.000591	Saliva_B	PDCD4
ELMOD2	4.11E-08	0.264484	0.072	0.047	0.000655	Saliva_B	ELMOD2
NDUFA12	4.96E-08	0.342137	0.303	0.275	0.000791	Saliva_B	NDUFA12
FAM98A	4.96E-08	0.281062	0.088	0.061	0.000792	Saliva_B	FAM98A

MGMT	5.57E-08	0.332569	0.196	0.162	0.000889	Saliva_B	MGMT
TCEA1	5.58E-08	0.357105	0.271	0.24	0.000891	Saliva_B	TCEA1
ACADVL	5.63E-08	0.369206	0.26	0.232	0.000898	Saliva_B	ACADVL
OSBP	5.93E-08	0.328973	0.101	0.072	0.000946	Saliva_B	OSBP
CRYBG31	6E-08	0.284266	0.091	0.062	0.000957	Saliva_B	CRYBG3
MRPL35	6.27E-08	0.270349	0.082	0.055	0.000999	Saliva_B	MRPL35
B4GALT2	6.42E-08	0.273708	0.066	0.042	0.001024	Saliva_B	B4GALT2
GBF1	6.78E-08	0.25074	0.076	0.05	0.001081	Saliva_B	GBF1
USF2	6.98E-08	0.342745	0.151	0.117	0.001113	Saliva_B	USF2
TCTN3	8.15E-08	0.316081	0.101	0.072	0.0013	Saliva_B	TCTN3
GFPT11	9.61E-08	0.351994	0.159	0.127	0.001533	Saliva_B	GFPT1
DPM2	1.09E-07	0.355214	0.144	0.113	0.001745	Saliva_B	DPM2
ATG5	1.15E-07	0.337712	0.121	0.09	0.001826	Saliva_B	ATG5
RBM261	1.19E-07	0.314538	0.128	0.097	0.001891	Saliva_B	RBM26
NDUFV1	1.2E-07	0.290611	0.206	0.172	0.001921	Saliva_B	NDUFV1
ESCO1	1.41E-07	0.320755	0.113	0.084	0.002257	Saliva_B	ESCO1
BCAS2	1.42E-07	0.358769	0.185	0.152	0.002259	Saliva_B	BCAS2
GPAA11	1.62E-07	0.270155	0.176	0.142	0.002586	Saliva_B	GPAA1
CTDNEP1	1.68E-07	0.312929	0.167	0.135	0.002686	Saliva_B	CTDNEP1
TPGS1	1.78E-07	0.262118	0.133	0.1	0.002838	Saliva_B	TPGS1
APPL11	1.93E-07	0.338476	0.155	0.123	0.003073	Saliva_B	APPL1
C2orf741	1.97E-07	0.251529	0.087	0.06	0.00314	Saliva_B	C2orf74
SLC30A7	1.97E-07	0.333552	0.111	0.082	0.003148	Saliva_B	SLC30A7
IFT20	2.03E-07	0.375169	0.19	0.159	0.003245	Saliva_B	IFT20
MRPS12	2.06E-07	0.318887	0.172	0.14	0.003287	Saliva_B	MRPS12
SRP19	2.24E-07	0.350907	0.207	0.178	0.003569	Saliva_B	SRP19
PSMD14	2.6E-07	0.33783	0.149	0.118	0.00415	Saliva_B	PSMD14
HSPD1	2.77E-07	0.299954	0.291	0.257	0.004416	Saliva_B	HSPD1
PPIG1	3.02E-07	0.288144	0.28	0.25	0.00481	Saliva_B	PPIG
PNN1	3.15E-07	0.357494	0.242	0.211	0.005024	Saliva_B	PNN
UNC50	3.39E-07	0.324954	0.154	0.123	0.0054	Saliva_B	UNC50
EIF2AK21	4.05E-07	0.298682	0.125	0.095	0.006458	Saliva_B	EIF2AK2
RNASEH2B	4.2E-07	0.274689	0.136	0.104	0.006691	Saliva_B	RNASEH2B
ZP3	4.36E-07	0.311606	0.057	0.036	0.006946	Saliva_B	ZP3
SCAMP2	5.13E-07	0.369236	0.217	0.188	0.008186	Saliva_B	SCAMP2
PHF1	5.38E-07	0.3215	0.146	0.115	0.008587	Saliva_B	PHF1
MRPL43	5.88E-07	0.317481	0.221	0.192	0.00938	Saliva_B	MRPL43
ACO2	7.26E-07	0.290936	0.127	0.099	0.011587	Saliva_B	ACO2
SS18	7.46E-07	0.338196	0.096	0.07	0.011902	Saliva_B	SS18
HNRNPD	8.55E-07	0.340371	0.265	0.24	0.013638	Saliva_B	HNRNPD
SEP15	1.15E-06	0.366094	0.326	0.306	0.018384	Saliva_B	SEP15
MRPL15	1.16E-06	0.335721	0.119	0.092	0.018496	Saliva_B	MRPL15
CCNC	1.25E-06	0.426081	0.226	0.204	0.019938	Saliva_B	CCNC
FAM96A	1.37E-06	0.303411	0.127	0.099	0.021774	Saliva_B	FAM96A
LACTB	1.44E-06	0.251264	0.113	0.085	0.022891	Saliva_B	LACTB
RTCA	1.46E-06	0.287329	0.114	0.087	0.023238	Saliva_B	RTCA
VKORC1L1	1.5E-06	0.283112	0.085	0.061	0.023991	Saliva_B	VKORC1L1

VPS29	1.58E-06	0.369575	0.301	0.282	0.025129	Saliva_B	VPS29
ILF2	1.67E-06	0.288791	0.235	0.205	0.026574	Saliva_B	ILF2
VARS	1.86E-06	0.273802	0.08	0.056	0.029642	Saliva_B	VARS
PCBD1	1.91E-06	0.304131	0.186	0.158	0.030428	Saliva_B	PCBD1
DDX6	2.03E-06	0.318151	0.167	0.138	0.032439	Saliva_B	DDX6
FAM177A1	2.18E-06	0.339475	0.242	0.215	0.034775	Saliva_B	FAM177A1
PSMC2	2.23E-06	0.288558	0.155	0.126	0.035565	Saliva_B	PSMC2
SECISBP2	2.33E-06	0.278586	0.165	0.135	0.037169	Saliva_B	SECISBP2
ZNF292	2.37E-06	0.363766	0.198	0.172	0.037749	Saliva_B	ZNF292
NMRK1	2.67E-06	0.321066	0.156	0.128	0.042512	Saliva_B	NMRK1
CDK4	3.01E-06	0.258596	0.142	0.113	0.048043	Saliva_B	CDK4
THOC2	3.03E-06	0.288698	0.154	0.126	0.04828	Saliva_B	THOC2
SCYL1	3.03E-06	0.274391	0.125	0.098	0.048368	Saliva_B	SCYL1
MRPL36	3.09E-06	0.322522	0.217	0.19	0.049223	Saliva_B	MRPL36
MRPS18C1	3.33E-06	0.393371	0.212	0.189	0.053093	Saliva_B	MRPS18C
POLR3D	3.6E-06	0.261538	0.073	0.051	0.057491	Saliva_B	POLR3D
SRSF101	3.89E-06	0.349922	0.183	0.157	0.06209	Saliva_B	SRSF10
SPAG7	4.15E-06	0.317767	0.203	0.176	0.066186	Saliva_B	SPAG7
GARS	4.34E-06	0.32036	0.126	0.1	0.069159	Saliva_B	GARS
XPC1	4.63E-06	0.251415	0.085	0.061	0.073802	Saliva_B	XPC
TBCA	4.64E-06	0.250573	0.371	0.351	0.074076	Saliva_B	TBCA
EMC4	4.92E-06	0.320682	0.274	0.256	0.078522	Saliva_B	EMC4
KAT6B	4.99E-06	0.256197	0.102	0.077	0.079584	Saliva_B	KAT6B
CDK5RAP3	5E-06	0.293797	0.156	0.128	0.079748	Saliva_B	CDK5RAP3
FAM174A	5.1E-06	0.301443	0.108	0.083	0.081279	Saliva_B	FAM174A
NFKBIA	5.57E-06	0.304674	0.524	0.517	0.088786	Saliva_B	NFKBIA
RHEB	6.36E-06	0.285557	0.357	0.338	0.101438	Saliva_B	RHEB
NKAP	6.38E-06	0.25881	0.124	0.097	0.101676	Saliva_B	NKAP
UBE2S	6.9E-06	0.278415	0.16	0.131	0.110007	Saliva_B	UBE2S
DEF8	7.3E-06	0.278808	0.113	0.088	0.116377	Saliva_B	DEF8
SLC30A5	7.83E-06	0.261288	0.094	0.071	0.124951	Saliva_B	SLC30A5
USP3	9.43E-06	0.355649	0.137	0.111	0.150446	Saliva_B	USP3
PRRC2C1	1.03E-05	0.254753	0.299	0.279	0.164628	Saliva_B	PRRC2C
GFER	1.21E-05	0.311094	0.143	0.118	0.193272	Saliva_B	GFER
FAF2	1.51E-05	0.298902	0.087	0.065	0.24128	Saliva_B	FAF2
PYCR12	1.65E-05	0.257818	0.104	0.081	0.26373	Saliva_B	PYCR1
POLR2K	1.84E-05	0.285276	0.306	0.293	0.294169	Saliva_B	POLR2K
PRRC1	2.09E-05	0.278812	0.098	0.076	0.334011	Saliva_B	PRRC1
NIPA2	2.22E-05	0.276468	0.12	0.097	0.354705	Saliva_B	NIPA2
NME41	2.38E-05	0.27447	0.167	0.143	0.38	Saliva_B	NME4
NUDT5	2.72E-05	0.321151	0.136	0.112	0.434101	Saliva_B	NUDT5
BCKDK	3.43E-05	0.334306	0.125	0.103	0.547703	Saliva_B	BCKDK
MRPL22	3.44E-05	0.290563	0.177	0.154	0.548487	Saliva_B	MRPL22
EMC21	4.38E-05	0.33556	0.147	0.126	0.698654	Saliva_B	EMC2
LSM1	4.99E-05	0.322174	0.173	0.15	0.79651	Saliva_B	LSM1
MED8	5.24E-05	0.317099	0.109	0.087	0.836499	Saliva_B	MED8
UBE2Q1	5.85E-05	0.279011	0.1	0.079	0.932286	Saliva_B	UBE2Q1

GOLGA2	6.59E-05	0.321283	0.167	0.146	1	Saliva_B	GOLGA2
TRIM441	6.8E-05	0.262977	0.112	0.09	1	Saliva_B	TRIM44
TMX2	8.12E-05	0.260207	0.123	0.101	1	Saliva_B	TMX2
UROS	9.43E-05	0.302785	0.121	0.1	1	Saliva_B	UROS
TMEM14C	0.000118	0.285996	0.271	0.256	1	Saliva_B	TMEM14C
C1orf27	0.000126	0.309655	0.094	0.075	1	Saliva_B	C1orf27
COBLL11	0.000174	0.327526	0.119	0.099	1	Saliva_B	COBLL1
CCL3	0.000194	0.36728	0.067	0.048	1	Saliva_B	CCL3
KIAA1033	0.000209	0.289788	0.101	0.082	1	Saliva_B	KIAA1033
WHSC1L1	0.000233	0.257714	0.214	0.195	1	Saliva_B	WHSC1L1
NSD1	0.000256	0.290298	0.121	0.101	1	Saliva_B	NSD1
CYHR1	0.000278	0.282793	0.137	0.117	1	Saliva_B	CYHR1
ZNF6521	0.000285	0.309627	0.114	0.095	1	Saliva_B	ZNF652
ERCC1	0.000302	0.371472	0.172	0.155	1	Saliva_B	ERCC1
MSRA	0.000336	0.277974	0.103	0.084	1	Saliva_B	MSRA
EMC6	0.000344	0.277173	0.225	0.212	1	Saliva_B	EMC6
FNBP4	0.000358	0.30797	0.158	0.138	1	Saliva_B	FNBP4
SLTM	0.000463	0.338494	0.211	0.195	1	Saliva_B	SLTM
PSMC4	0.000464	0.266955	0.18	0.162	1	Saliva_B	PSMC4
TGOLN21	0.000492	0.259406	0.189	0.172	1	Saliva_B	TGOLN2
LUC7L31	0.000509	0.254698	0.279	0.266	1	Saliva_B	LUC7L3
NDUFS3	0.000531	0.318986	0.204	0.188	1	Saliva_B	NDUFS3
ISOC2	0.00054	0.284151	0.15	0.132	1	Saliva_B	ISOC2
EIF3J	0.00054	0.30216	0.209	0.195	1	Saliva_B	EIF3J
IMPAD1	0.000655	0.262262	0.11	0.092	1	Saliva_B	IMPAD1
AP3B1	0.000703	0.252338	0.106	0.088	1	Saliva_B	AP3B1
COMMD1	0.000707	0.313039	0.181	0.166	1	Saliva_B	COMMD1
SDF2	0.000809	0.290283	0.157	0.14	1	Saliva_B	SDF2
EIF4B1	0.000812	0.268615	0.309	0.3	1	Saliva_B	EIF4B
SDAD1	0.000833	0.269566	0.104	0.086	1	Saliva_B	SDAD1
CHCHD1	0.000987	0.326863	0.186	0.173	1	Saliva_B	CHCHD1
PRDM2	0.001088	0.275706	0.124	0.107	1	Saliva_B	PRDM2
R3HCC1	0.001102	0.254544	0.097	0.08	1	Saliva_B	R3HCC1
GLG1	0.001107	0.308621	0.144	0.127	1	Saliva_B	GLG1
PHF3	0.001111	0.308912	0.148	0.133	1	Saliva_B	PHF3
YME1L1	0.001367	0.268351	0.216	0.202	1	Saliva_B	YME1L1
TMEM131	0.001401	0.261909	0.078	0.063	1	Saliva_B	TMEM131
STAG1	0.001564	0.25147	0.087	0.071	1	Saliva_B	STAG1
PGAM1	0.002117	0.282089	0.239	0.229	1	Saliva_B	PGAM1
MCFD21	0.002165	0.255437	0.207	0.193	1	Saliva_B	MCFD2
CLN5	0.00257	0.256285	0.094	0.079	1	Saliva_B	CLN5
MTIF3	0.002938	0.296152	0.218	0.209	1	Saliva_B	MTIF3
SLC25A36	0.003197	0.270757	0.138	0.123	1	Saliva_B	SLC25A36
SRA1	0.003282	0.315958	0.19	0.182	1	Saliva_B	SRA1
SNRPA1	0.003473	0.263085	0.146	0.131	1	Saliva_B	SNRPA1
PCGF5	0.00363	0.26183	0.139	0.125	1	Saliva_B	PCGF5
CDC26	0.004861	0.289735	0.139	0.127	1	Saliva_B	CDC26

PSMC1	0.005088	0.288986	0.201	0.191	1	Saliva_B	PSMC1
CCDC47	0.005874	0.279308	0.152	0.14	1	Saliva_B	CCDC47
KIAA2013	0.006289	0.252541	0.098	0.085	1	Saliva_B	KIAA2013
MRPS23	0.007353	0.250623	0.111	0.098	1	Saliva_B	MRPS23
PRKDC1	0.007772	0.250028	0.112	0.098	1	Saliva_B	PRKDC
FOXO3	0.008849	0.2643	0.183	0.173	1	Saliva_B	FOXO3
RPA3	0.009361	0.256695	0.157	0.147	1	Saliva_B	RPA3
XIST2	0.009512	0.439826	0.135	0.123	1	Saliva_B	XIST
MGP1	0	4.84203	0.973	0.183	0	Saliva_Fibr	MGP
APOD	0	4.390619	0.843	0.113	0	Saliva_Fibr	APOD
CFD	0	4.25252	0.94	0.314	0	Saliva_Fibr	CFD
GSN1	0	3.967051	0.979	0.353	0	Saliva_Fibr	GSN
IGFBP6	0	3.948238	0.856	0.11	0	Saliva_Fibr	IGFBP6
CFH	0	3.833708	0.832	0.097	0	Saliva_Fibr	CFH
IGFBP5	0	3.406412	0.759	0.112	0	Saliva_Fibr	IGFBP5
LTBP41	0	3.09933	0.777	0.104	0	Saliva_Fibr	LTBP4
DCN	0	3.094804	0.972	0.168	0	Saliva_Fibr	DCN
TIMP31	0	3.043411	0.824	0.141	0	Saliva_Fibr	TIMP3
ANGPTL1	0	2.971951	0.617	0.025	0	Saliva_Fibr	ANGPTL1
MFAP4	0	2.792127	0.661	0.077	0	Saliva_Fibr	MFAP4
TNXB1	0	2.729366	0.587	0.053	0	Saliva_Fibr	TNXB
C1R	0	2.684963	0.708	0.088	0	Saliva_Fibr	C1R
LUM	0	2.650648	0.767	0.093	0	Saliva_Fibr	LUM
CCDC80	0	2.645357	0.726	0.128	0	Saliva_Fibr	CCDC80
MFAP5	0	2.640592	0.423	0.038	0	Saliva_Fibr	MFAP5
CD812	0	2.602377	0.909	0.3	0	Saliva_Fibr	CD81
FBLN1	0	2.569714	0.768	0.115	0	Saliva_Fibr	FBLN1
SERPING11	0	2.535755	0.742	0.15	0	Saliva_Fibr	SERPING1
C3	0	2.517088	0.529	0.067	0	Saliva_Fibr	C3
FBN1	0	2.478604	0.43	0.059	0	Saliva_Fibr	FBN1
COL6A2	0	2.382447	0.811	0.135	0	Saliva_Fibr	COL6A2
C1S	0	2.37692	0.683	0.097	0	Saliva_Fibr	C1S
LRP1	0	2.370835	0.582	0.063	0	Saliva_Fibr	LRP1
VIM2	0	2.347229	0.99	0.475	0	Saliva_Fibr	VIM
FN1	0	2.322341	0.54	0.081	0	Saliva_Fibr	FN1
FGL2	0	2.314666	0.576	0.08	0	Saliva_Fibr	FGL2
CXCL121	0	2.313864	0.617	0.09	0	Saliva_Fibr	CXCL12
COL6A1	0	2.287363	0.694	0.12	0	Saliva_Fibr	COL6A1
SCARA5	0	2.211799	0.462	0.025	0	Saliva_Fibr	SCARA5
COL1A2	0	2.195066	0.855	0.13	0	Saliva_Fibr	COL1A2
SSPN	0	2.166978	0.491	0.051	0	Saliva_Fibr	SSPN
PDGFRL	0	2.166024	0.457	0.035	0	Saliva_Fibr	PDGFRL
ABI3BP	0	2.152116	0.473	0.038	0	Saliva_Fibr	ABI3BP
RARRES2	0	2.077609	0.484	0.073	0	Saliva_Fibr	RARRES2
CALD1	0	2.054897	0.748	0.173	0	Saliva_Fibr	CALD1
COL6A3	0	2.02002	0.507	0.076	0	Saliva_Fibr	COL6A3
PMP221	0	2.014385	0.588	0.121	0	Saliva_Fibr	PMP22

FBLN21	0	2.009248	0.505	0.075	0	Saliva_Fibr	FBLN2
SPON2	0	1.97872	0.384	0.05	0	Saliva_Fibr	SPON2
VCAN	0	1.975688	0.466	0.078	0	Saliva_Fibr	VCAN
IGFBP41	0	1.972574	0.508	0.116	0	Saliva_Fibr	IGFBP4
COL14A1	0	1.972386	0.41	0.027	0	Saliva_Fibr	COL14A1
S100A6	0	1.924785	0.994	0.702	0	Saliva_Fibr	S100A6
PCOLCE	0	1.909639	0.487	0.076	0	Saliva_Fibr	PCOLCE
WISP2	0	1.89864	0.368	0.045	0	Saliva_Fibr	WISP2
MSX11	0	1.891799	0.365	0.024	0	Saliva_Fibr	MSX1
TUBA1A1	0	1.890358	0.622	0.22	0	Saliva_Fibr	TUBA1A
NFIB2	0	1.860582	0.578	0.176	0	Saliva_Fibr	NFIB
MXRA8	0	1.85188	0.426	0.044	0	Saliva_Fibr	MXRA8
PCOLCE2	0	1.844184	0.252	0.026	0	Saliva_Fibr	PCOLCE2
SERPINF1	0	1.835607	0.623	0.12	0	Saliva_Fibr	SERPINF1
S100A131	0	1.817463	0.638	0.242	0	Saliva_Fibr	S100A13
NTRK2	0	1.800099	0.387	0.04	0	Saliva_Fibr	NTRK2
CD248	0	1.797729	0.353	0.024	0	Saliva_Fibr	CD248
PIK3R1	0	1.752429	0.408	0.099	0	Saliva_Fibr	PIK3R1
NFIC2	0	1.748569	0.48	0.16	0	Saliva_Fibr	NFIC
FSTL1	0	1.738998	0.436	0.143	0	Saliva_Fibr	FSTL1
FGFR1	0	1.717312	0.413	0.075	0	Saliva_Fibr	FGFR1
PLAGL1	0	1.71095	0.356	0.084	0	Saliva_Fibr	PLAGL1
AHNAK3	0	1.701731	0.724	0.373	0	Saliva_Fibr	AHNAK
TCF41	0	1.699894	0.531	0.16	0	Saliva_Fibr	TCF4
SFRP11	0	1.690363	0.3	0.088	0	Saliva_Fibr	SFRP1
PTGIS	0	1.682769	0.304	0.014	0	Saliva_Fibr	PTGIS
MMP2	0	1.682597	0.554	0.106	0	Saliva_Fibr	MMP2
PDGFRB	0	1.678165	0.381	0.055	0	Saliva_Fibr	PDGFRB
LMNA2	0	1.673173	0.753	0.351	0	Saliva_Fibr	LMNA
SDC2	0	1.671769	0.411	0.097	0	Saliva_Fibr	SDC2
MXRA71	0	1.660589	0.381	0.072	0	Saliva_Fibr	MXRA7
ABCA6	0	1.656879	0.277	0.017	0	Saliva_Fibr	ABCA6
HTRA1	0	1.652082	0.437	0.099	0	Saliva_Fibr	HTRA1
HSPG21	0	1.648419	0.392	0.103	0	Saliva_Fibr	HSPG2
EFEMP1	0	1.642183	0.357	0.059	0	Saliva_Fibr	EFEMP1
LGALS11	0	1.638006	0.941	0.39	0	Saliva_Fibr	LGALS1
ITM2A1	0	1.631085	0.48	0.122	0	Saliva_Fibr	ITM2A
FXYD1	0	1.603268	0.41	0.073	0	Saliva_Fibr	FXYD1
S100A101	0	1.579737	0.926	0.666	0	Saliva_Fibr	S100A10
GPNMB	0	1.575845	0.507	0.112	0	Saliva_Fibr	GPNMB
ANXA51	0	1.567728	0.65	0.305	0	Saliva_Fibr	ANXA5
HTRA3	0	1.554286	0.275	0.017	0	Saliva_Fibr	HTRA3
DDAH2	0	1.552646	0.491	0.2	0	Saliva_Fibr	DDAH2
CYBRD1	0	1.53845	0.429	0.102	0	Saliva_Fibr	CYBRD1
TMEM176I	0	1.53585	0.414	0.078	0	Saliva_Fibr	TMEM176B
IFITM31	0	1.528101	0.765	0.293	0	Saliva_Fibr	IFITM3
THY1	0	1.520288	0.31	0.041	0	Saliva_Fibr	THY1

SPRY11	0	1.50654	0.364	0.081	0	Saliva_Fibr	SPRY1
TPPP3	0	1.504181	0.403	0.098	0	Saliva_Fibr	TPPP3
S100A4	0	1.502041	0.919	0.447	0	Saliva_Fibr	S100A4
SPTBN11	0	1.499974	0.462	0.188	0	Saliva_Fibr	SPTBN1
DDR2	0	1.495267	0.304	0.034	0	Saliva_Fibr	DDR2
MEG3	0	1.485955	0.326	0.084	0	Saliva_Fibr	MEG3
IGF11	0	1.484417	0.244	0.029	0	Saliva_Fibr	IGF1
CHRDL1	0	1.47879	0.274	0.017	0	Saliva_Fibr	CHRDL1
EPHX11	0	1.47458	0.346	0.113	0	Saliva_Fibr	EPHX1
GAS1	0	1.472553	0.286	0.026	0	Saliva_Fibr	GAS1
AEBP1	0	1.467963	0.359	0.069	0	Saliva_Fibr	AEBP1
COL15A11	0	1.459077	0.252	0.049	0	Saliva_Fibr	COL15A1
ARL6IP5	0	1.457674	0.605	0.299	0	Saliva_Fibr	ARL6IP5
FGF7	0	1.456046	0.29	0.037	0	Saliva_Fibr	FGF7
SFRP4	0	1.452766	0.176	0.008	0	Saliva_Fibr	SFRP4
SELM3	0	1.449823	0.719	0.333	0	Saliva_Fibr	SELM
AKAP12	0	1.430818	0.306	0.066	0	Saliva_Fibr	AKAP12
TIMP12	0	1.430237	0.711	0.369	0	Saliva_Fibr	TIMP1
CD633	0	1.428495	0.938	0.613	0	Saliva_Fibr	CD63
EID11	0	1.419538	0.656	0.346	0	Saliva_Fibr	EID1
MGST12	0	1.419515	0.43	0.185	0	Saliva_Fibr	MGST1
TMEM176/	0	1.418362	0.325	0.054	0	Saliva_Fibr	TMEM176A
COL1A1	0	1.409639	0.637	0.117	0	Saliva_Fibr	COL1A1
CPQ	0	1.405049	0.32	0.066	0	Saliva_Fibr	CPQ
MRC2	0	1.403687	0.276	0.035	0	Saliva_Fibr	MRC2
PI16	0	1.400116	0.267	0.042	0	Saliva_Fibr	PI16
IGFBP71	0	1.384097	0.755	0.246	0	Saliva_Fibr	IGFBP7
JUND2	0	1.383863	0.711	0.454	0	Saliva_Fibr	JUND
LEPR	0	1.381792	0.282	0.059	0	Saliva_Fibr	LEPR
RRAS1	0	1.376614	0.331	0.09	0	Saliva_Fibr	RRAS
PDGFRA	0	1.36508	0.302	0.046	0	Saliva_Fibr	PDGFRA
NFIA1	0	1.355074	0.397	0.165	0	Saliva_Fibr	NFIA
SEPP1	0	1.352751	0.593	0.253	0	Saliva_Fibr	SEPP1
LAPTM4A	0	1.336182	0.789	0.509	0	Saliva_Fibr	LAPTM4A
CAV11	0	1.334336	0.595	0.183	0	Saliva_Fibr	CAV1
PRKCDBP1	0	1.333919	0.386	0.119	0	Saliva_Fibr	PRKCDBP
PRELP	0	1.322045	0.27	0.032	0	Saliva_Fibr	PRELP
CST3	0	1.314387	0.943	0.58	0	Saliva_Fibr	CST3
LAMA2	0	1.311097	0.223	0.018	0	Saliva_Fibr	LAMA2
MAP1B	0	1.305582	0.327	0.08	0	Saliva_Fibr	MAP1B
MT.ATP64	0	1.305335	1	0.894	0	Saliva_Fibr	MT.ATP6
GPC3	0	1.303126	0.205	0.021	0	Saliva_Fibr	GPC3
ACKR3	0	1.286348	0.221	0.02	0	Saliva_Fibr	ACKR3
CD341	0	1.284264	0.302	0.059	0	Saliva_Fibr	CD34
RAB34	0	1.283139	0.321	0.094	0	Saliva_Fibr	RAB34
CTSK	0	1.281849	0.412	0.086	0	Saliva_Fibr	CTSK
MYADM1	0	1.280396	0.368	0.142	0	Saliva_Fibr	MYADM

LHFP1	0	1.276825	0.32	0.078	0	Saliva_Fibr LHFP
HIC1	0	1.270827	0.228	0.015	0	Saliva_Fibr HIC1
PTRF1	0	1.266003	0.438	0.153	0	Saliva_Fibr PTRF
MGST33	0	1.264645	0.626	0.324	0	Saliva_Fibr MGST3
NOVA1	0	1.259804	0.251	0.048	0	Saliva_Fibr NOVA1
CILP	0	1.258297	0.212	0.021	0	Saliva_Fibr CILP
NDN	0	1.240083	0.273	0.055	0	Saliva_Fibr NDN
ABCA10	0	1.234252	0.183	0.025	0	Saliva_Fibr ABCA10
MTRNR2L1	0	1.22915	0.626	0.255	0	Saliva_Fibr MTRNR2L12
TSPAN41	0	1.225826	0.33	0.109	0	Saliva_Fibr TSPAN4
COLEC12	0	1.225484	0.228	0.021	0	Saliva_Fibr COLEC12
FHL1	0	1.203414	0.346	0.121	0	Saliva_Fibr FHL1
LAMB21	0	1.197386	0.271	0.076	0	Saliva_Fibr LAMB2
MT.CYB4	0	1.194459	1	0.931	0	Saliva_Fibr MT.CYB
EBF11	0	1.193208	0.265	0.054	0	Saliva_Fibr EBF1
EPB41L2	0	1.183686	0.273	0.085	0	Saliva_Fibr EPB41L2
MT.ND54	0	1.181663	0.963	0.693	0	Saliva_Fibr MT.ND5
FBLN5	0	1.181485	0.277	0.059	0	Saliva_Fibr FBLN5
ITM2B2	0	1.179504	0.915	0.698	0	Saliva_Fibr ITM2B
MT.ND14	0	1.177207	0.998	0.925	0	Saliva_Fibr MT.ND1
SPARC1	0	1.175045	0.491	0.15	0	Saliva_Fibr SPARC
DPYSL31	0	1.161969	0.243	0.048	0	Saliva_Fibr DPYSL3
LTBP2	0	1.156616	0.174	0.011	0	Saliva_Fibr LTBP2
TGFBR31	0	1.156077	0.267	0.085	0	Saliva_Fibr TGFBR3
GAS7	0	1.154598	0.226	0.041	0	Saliva_Fibr GAS7
LRRN4CL	0	1.137199	0.21	0.016	0	Saliva_Fibr LRRN4CL
OLFML3	0	1.13646	0.244	0.039	0	Saliva_Fibr OLFML3
TWIST1	0	1.103629	0.245	0.039	0	Saliva_Fibr TWIST1
MEDAG	0	1.101304	0.218	0.025	0	Saliva_Fibr MEDAG
GOLIM4	0	1.101184	0.252	0.077	0	Saliva_Fibr GOLIM4
MT.ND34	0	1.101051	1	0.787	0	Saliva_Fibr MT.ND3
ANGPTL2	0	1.10013	0.197	0.026	0	Saliva_Fibr ANGPTL2
NBL11	0	1.097896	0.321	0.103	0	Saliva_Fibr NBL1
SYNE11	0	1.097475	0.238	0.05	0	Saliva_Fibr SYNE1
DBN1	0	1.091386	0.207	0.046	0	Saliva_Fibr DBN1
ID31	0	1.07512	0.402	0.164	0	Saliva_Fibr ID3
DLC11	0	1.069241	0.218	0.043	0	Saliva_Fibr DLC1
PCDH18	0	1.067075	0.181	0.015	0	Saliva_Fibr PCDH18
GGT51	0	1.056677	0.157	0.02	0	Saliva_Fibr GGT5
OAF	0	1.053513	0.204	0.047	0	Saliva_Fibr OAF
NAV11	0	1.049319	0.189	0.023	0	Saliva_Fibr NAV1
ELN1	0	1.047864	0.204	0.051	0	Saliva_Fibr ELN
LTBP1	0	1.045208	0.217	0.046	0	Saliva_Fibr LTBP1
IL11RA	0	1.032946	0.2	0.047	0	Saliva_Fibr IL11RA
NRP11	0	1.01904	0.199	0.051	0	Saliva_Fibr NRP1
PDGFD1	0	1.018806	0.18	0.017	0	Saliva_Fibr PDGFD
MT.ND44	0	1.017256	0.999	0.952	0	Saliva_Fibr MT.ND4

MAP1A	0	1.010184	0.187	0.03	0	Saliva_Fibr	MAP1A
NUPR11	0	1.000698	0.51	0.25	0	Saliva_Fibr	NUPR1
TGFB1I11	0	0.994192	0.209	0.046	0	Saliva_Fibr	TGFB1I1
MME	0	0.983353	0.133	0.007	0	Saliva_Fibr	MME
AKR1C11	0	0.981092	0.188	0.04	0	Saliva_Fibr	AKR1C1
GSTM5	0	0.977895	0.177	0.023	0	Saliva_Fibr	GSTM5
ADAMTS5	0	0.97341	0.147	0.015	0	Saliva_Fibr	ADAMTS5
PHLDB1	0	0.970293	0.189	0.043	0	Saliva_Fibr	PHLDB1
RGMA	0	0.964226	0.147	0.022	0	Saliva_Fibr	RGMA
EMP31	0	0.958636	0.63	0.341	0	Saliva_Fibr	EMP3
LOXL1	0	0.954164	0.165	0.027	0	Saliva_Fibr	LOXL1
OLFML2B	0	0.942624	0.162	0.023	0	Saliva_Fibr	OLFML2B
FXYD61	0	0.942196	0.155	0.024	0	Saliva_Fibr	FXYD6
COL5A2	0	0.94191	0.203	0.036	0	Saliva_Fibr	COL5A2
PRG4	0	0.933276	0.075	0.003	0	Saliva_Fibr	PRG4
ABCA9	0	0.931518	0.146	0.01	0	Saliva_Fibr	ABCA9
ITIH51	0	0.931246	0.181	0.034	0	Saliva_Fibr	ITIH5
OLFML2A1	0	0.929782	0.154	0.018	0	Saliva_Fibr	OLFML2A
KANK2	0	0.92812	0.186	0.038	0	Saliva_Fibr	KANK2
RAMP21	0	0.925496	0.248	0.068	0	Saliva_Fibr	RAMP2
PGF	0	0.914246	0.143	0.025	0	Saliva_Fibr	PGF
RUNX1T11	0	0.912374	0.171	0.035	0	Saliva_Fibr	RUNX1T1
ZFP36L21	0	0.908628	0.658	0.382	0	Saliva_Fibr	ZFP36L2
SPOCK1	0	0.905894	0.149	0.016	0	Saliva_Fibr	SPOCK1
PCDH9	0	0.891901	0.138	0.013	0	Saliva_Fibr	PCDH9
GLT8D2	0	0.880065	0.155	0.02	0	Saliva_Fibr	GLT8D2
LPAR1	0	0.872388	0.168	0.034	0	Saliva_Fibr	LPAR1
VCAM11	0	0.86922	0.146	0.028	0	Saliva_Fibr	VCAM1
SYNE32	0	0.861005	0.151	0.013	0	Saliva_Fibr	SYNE3
PALM1	0	0.848915	0.154	0.018	0	Saliva_Fibr	PALM
MT.CO33	0	0.848408	0.999	0.977	0	Saliva_Fibr	MT.CO3
RP11.14N7	0	0.847931	0.171	0.033	0	Saliva_Fibr	RP11.14N7.2
SSC5D	0	0.843692	0.147	0.011	0	Saliva_Fibr	SSC5D
ANXA2	0	0.842585	0.809	0.59	0	Saliva_Fibr	ANXA2
PLXDC1	0	0.836084	0.141	0.013	0	Saliva_Fibr	PLXDC1
H3F3A3	0	0.830504	0.789	0.467	0	Saliva_Fibr	H3F3A
CLMP	0	0.815702	0.134	0.009	0	Saliva_Fibr	CLMP
OLFML1	0	0.814818	0.146	0.015	0	Saliva_Fibr	OLFML1
AR	0	0.807169	0.133	0.017	0	Saliva_Fibr	AR
CYP26B1	0	0.805012	0.134	0.023	0	Saliva_Fibr	CYP26B1
ZCCHC241	0	0.803282	0.158	0.029	0	Saliva_Fibr	ZCCHC24
NTM	0	0.802294	0.132	0.008	0	Saliva_Fibr	NTM
MT.ND24	0	0.779427	0.996	0.904	0	Saliva_Fibr	MT.ND2
NT5E	0	0.768639	0.141	0.023	0	Saliva_Fibr	NT5E
ACVRL11	0	0.764148	0.132	0.024	0	Saliva_Fibr	ACVRL1
DNM1	0	0.728527	0.127	0.014	0	Saliva_Fibr	DNM1
LRRC17	0	0.715643	0.123	0.012	0	Saliva_Fibr	LRRC17

COL6A6	0	0.71238	0.104	0.003	0	Saliva_Fibr	COL6A6
C14orf132	0	0.694467	0.124	0.014	0	Saliva_Fibr	C14orf132
LGALS3	0	0.677992	0.869	0.559	0	Saliva_Fibr	LGALS3
MAN1C1	0	0.673032	0.113	0.012	0	Saliva_Fibr	MAN1C1
CAPN6	0	0.661581	0.081	0.004	0	Saliva_Fibr	CAPN6
MT.CO24	0	0.614809	1	0.958	0	Saliva_Fibr	MT.CO2
CRLF1	0	0.595368	0.074	0.005	0	Saliva_Fibr	CRLF1
TNNT3	0	0.58787	0.089	0.009	0	Saliva_Fibr	TNNT3
GAP43	0	0.564961	0.091	0.008	0	Saliva_Fibr	GAP43
HHIP.AS1	0	0.540912	0.084	0.006	0	Saliva_Fibr	HHIP.AS1
COL8A2	0	0.472571	0.074	0.007	0	Saliva_Fibr	COL8A2
EPHA3	0	0.472369	0.063	0.003	0	Saliva_Fibr	EPHA3
FMO1	0	0.462298	0.058	0.003	0	Saliva_Fibr	FMO1
ENPP6	0	0.447947	0.067	0.002	0	Saliva_Fibr	ENPP6
ADIRF1	0	0.412222	0.793	0.422	0	Saliva_Fibr	ADIRF
FGF18	0	0.38686	0.055	0.004	0	Saliva_Fibr	FGF18
FAM43B	0	0.30127	0.038	0.001	0	Saliva_Fibr	FAM43B
VEGFB2	1.3E-303	1.185763	0.272	0.093	2.1E-299	Saliva_Fibr	VEGFB
NNMT	1.5E-303	0.951026	0.306	0.103	2.4E-299	Saliva_Fibr	NNMT
MT.ND4L4	1.6E-303	0.889831	0.637	0.408	2.5E-299	Saliva_Fibr	MT.ND4L
EVA1B1	3.7E-302	1.051149	0.224	0.065	5.8E-298	Saliva_Fibr	EVA1B
MT.CO14	1.4E-301	0.584204	0.998	0.984	2.2E-297	Saliva_Fibr	MT.CO1
BOC	1.5E-301	0.754681	0.123	0.022	2.5E-297	Saliva_Fibr	BOC
ADD3	8.6E-298	1.196239	0.362	0.153	1.4E-293	Saliva_Fibr	ADD3
NUCKS11	1.6E-291	1.221192	0.493	0.276	2.6E-287	Saliva_Fibr	NUCKS1
CLU1	1.6E-284	1.183881	0.413	0.192	2.6E-280	Saliva_Fibr	CLU
MMP16	1.4E-282	0.474709	0.067	0.007	2.2E-278	Saliva_Fibr	MMP16
DPYSL21	7.2E-279	1.010507	0.269	0.093	1.2E-274	Saliva_Fibr	DPYSL2
SGCE	1.1E-275	0.80379	0.154	0.035	1.8E-271	Saliva_Fibr	SGCE
EFEMP2	2E-275	0.982492	0.211	0.062	3.2E-271	Saliva_Fibr	EFEMP2
NID1	2.1E-275	0.682425	0.126	0.024	3.3E-271	Saliva_Fibr	NID1
WNT5B1	5.8E-273	0.642655	0.091	0.013	9.3E-269	Saliva_Fibr	WNT5B
AOX1	1.4E-270	0.554125	0.092	0.014	2.2E-266	Saliva_Fibr	AOX1
COL5A1	4E-269	0.694906	0.135	0.028	6.4E-265	Saliva_Fibr	COL5A1
ARHGAP6	7.3E-269	0.552845	0.086	0.012	1.2E-264	Saliva_Fibr	ARHGAP6
CRABP2	1E-265	1.000548	0.24	0.077	1.6E-261	Saliva_Fibr	CRABP2
FGFBP2	4E-265	0.703063	0.153	0.034	6.4E-261	Saliva_Fibr	FGFBP2
CDON	1.1E-264	0.610421	0.096	0.015	1.7E-260	Saliva_Fibr	CDON
NR2F21	4.5E-264	1.071045	0.245	0.081	7.2E-260	Saliva_Fibr	NR2F2
PTMS1	1.8E-263	1.132509	0.449	0.237	2.8E-259	Saliva_Fibr	PTMS
CADM3	8.7E-263	0.613691	0.11	0.019	1.4E-258	Saliva_Fibr	CADM3
HMCN11	5.7E-262	0.514211	0.084	0.012	9.1E-258	Saliva_Fibr	HMCN1
SHOX2	3.8E-259	0.544411	0.086	0.012	6.1E-255	Saliva_Fibr	SHOX2
RBMS31	4.2E-259	0.895501	0.177	0.047	6.8E-255	Saliva_Fibr	RBMS3
ANTXR21	4.3E-259	0.766273	0.137	0.029	6.9E-255	Saliva_Fibr	ANTXR2
CELF2	3.2E-258	1.061198	0.314	0.126	5.1E-254	Saliva_Fibr	CELF2
CREB5	1.7E-256	1.025125	0.185	0.052	2.7E-252	Saliva_Fibr	CREB5

NEGR1	9.6E-256	0.711448	0.123	0.024	1.5E-251	Saliva_Fibr	NEGR1
LAMC11	5.2E-253	1.017388	0.222	0.072	8.2E-249	Saliva_Fibr	LAMC1
MN1	3.1E-251	0.537006	0.087	0.013	4.9E-247	Saliva_Fibr	MN1
LIMA11	7.5E-251	1.265259	0.364	0.175	1.2E-246	Saliva_Fibr	LIMA1
ADAM33	1.3E-250	0.561535	0.087	0.013	2.1E-246	Saliva_Fibr	ADAM33
TFPI1	4.6E-250	0.961032	0.228	0.075	7.4E-246	Saliva_Fibr	TFPI
FOSB3	2.7E-248	1.631069	0.441	0.244	4.3E-244	Saliva_Fibr	FOSB
PEBP12	9.2E-248	0.926735	0.643	0.449	1.5E-243	Saliva_Fibr	PEBP1
CHD92	9.3E-247	1.251988	0.343	0.16	1.5E-242	Saliva_Fibr	CHD9
CALHM21	2.4E-244	0.69018	0.136	0.03	3.8E-240	Saliva_Fibr	CALHM2
BDH2	2.9E-243	1.053455	0.219	0.073	4.6E-239	Saliva_Fibr	BDH2
AXL	1.8E-241	0.812926	0.262	0.092	2.8E-237	Saliva_Fibr	AXL
TUBA1B1	1.4E-240	0.928832	0.565	0.346	2.3E-236	Saliva_Fibr	TUBA1B
SCN7A	9.3E-239	0.551835	0.063	0.007	1.5E-234	Saliva_Fibr	SCN7A
EGR12	1.6E-238	1.333983	0.356	0.166	2.5E-234	Saliva_Fibr	EGR1
SLIT3	4.1E-237	0.996752	0.225	0.076	6.5E-233	Saliva_Fibr	SLIT3
TIMP2	1.1E-236	0.987826	0.544	0.343	1.7E-232	Saliva_Fibr	TIMP2
GFPT2	6.3E-236	0.564644	0.096	0.017	1E-231	Saliva_Fibr	GFPT2
FSTL3	6.4E-236	0.70818	0.123	0.026	1E-231	Saliva_Fibr	FSTL3
BICC1	1.3E-235	0.701011	0.15	0.037	2E-231	Saliva_Fibr	BICC1
RTN41	2.2E-235	1.022273	0.568	0.388	3.5E-231	Saliva_Fibr	RTN4
ZEB11	8E-235	0.865856	0.185	0.054	1.3E-230	Saliva_Fibr	ZEB1
EHD21	1.2E-234	0.804602	0.158	0.041	1.9E-230	Saliva_Fibr	EHD2
PTHLH	1.3E-233	0.503696	0.072	0.01	2E-229	Saliva_Fibr	PTHLH
FGF21	1.3E-232	0.558889	0.095	0.016	2.1E-228	Saliva_Fibr	FGF2
PLSCR41	1.8E-232	0.801986	0.161	0.043	2.9E-228	Saliva_Fibr	PLSCR4
IGF21	1.5E-229	0.674276	0.131	0.029	2.3E-225	Saliva_Fibr	IGF2
DAB21	1.7E-229	0.94238	0.245	0.089	2.7E-225	Saliva_Fibr	DAB2
SEPT71	1.8E-227	1.009428	0.524	0.333	2.9E-223	Saliva_Fibr	SEPT7
C12orf571	7.5E-226	1.01433	0.538	0.351	1.2E-221	Saliva_Fibr	C12orf57
CPXM21	1.3E-225	0.714652	0.13	0.03	2.1E-221	Saliva_Fibr	CPXM2
TMEM47	3.1E-225	0.910921	0.173	0.05	5E-221	Saliva_Fibr	TMEM47
TCEAL7	1.5E-222	0.480479	0.077	0.012	2.3E-218	Saliva_Fibr	TCEAL7
RCN3	1.6E-220	0.691395	0.138	0.034	2.6E-216	Saliva_Fibr	RCN3
KCNJ8	2.4E-220	0.348377	0.053	0.005	3.8E-216	Saliva_Fibr	KCNJ8
ANXA4	3.6E-219	1.101194	0.298	0.132	5.7E-215	Saliva_Fibr	ANXA4
CPED1	9E-217	0.529844	0.096	0.018	1.4E-212	Saliva_Fibr	CPED1
SNED1	2.3E-214	0.578532	0.089	0.015	3.7E-210	Saliva_Fibr	SNED1
EEA11	1.1E-213	1.063033	0.242	0.094	1.8E-209	Saliva_Fibr	EEA1
RASL12	1.3E-213	0.457256	0.069	0.01	2E-209	Saliva_Fibr	RASL12
CD99	1.8E-213	0.843609	0.649	0.486	2.9E-209	Saliva_Fibr	CD99
PTGFRN	6.6E-213	0.751904	0.141	0.037	1.1E-208	Saliva_Fibr	PTGFRN
ANPEP	2.2E-211	0.669632	0.128	0.03	3.6E-207	Saliva_Fibr	ANPEP
RNH11	4.2E-210	1.135793	0.476	0.308	6.8E-206	Saliva_Fibr	RNH1
MYL9	1.4E-209	0.472481	0.281	0.112	2.2E-205	Saliva_Fibr	MYL9
ECM2	2.2E-209	0.63281	0.128	0.031	3.5E-205	Saliva_Fibr	ECM2
GXYLT2	8.1E-207	0.698526	0.124	0.03	1.3E-202	Saliva_Fibr	GXYLT2

TRIO1	9.3E-206	0.711077	0.123	0.029	1.5E-201	Saliva_Fibr	TRIO
SRPX	1.9E-205	0.742449	0.12	0.028	3E-201	Saliva_Fibr	SRPX
BGN	3.8E-203	0.916829	0.194	0.063	6.1E-199	Saliva_Fibr	BGN
ZNF503	4.8E-202	0.963514	0.192	0.064	7.7E-198	Saliva_Fibr	ZNF503
GPX8	6.5E-202	0.735927	0.137	0.036	1E-197	Saliva_Fibr	GPX8
CDO1	3.6E-200	0.469831	0.072	0.011	5.7E-196	Saliva_Fibr	CDO1
TCF7L2	4E-200	0.999671	0.221	0.083	6.3E-196	Saliva_Fibr	TCF7L2
FRMD6	2.7E-198	1.031023	0.219	0.081	4.4E-194	Saliva_Fibr	FRMD6
OSR2	3.2E-198	0.656828	0.15	0.041	5.2E-194	Saliva_Fibr	OSR2
NFIX1	4.4E-197	1.066509	0.25	0.104	7.1E-193	Saliva_Fibr	NFIX
RPS173	2E-193	0.479368	0.427	0.214	3.2E-189	Saliva_Fibr	RPS17
FOS2	1.2E-192	1.301907	0.578	0.42	1.9E-188	Saliva_Fibr	FOS
ADAMTSL4	1.5E-190	0.645357	0.121	0.03	2.3E-186	Saliva_Fibr	ADAMTSL4
A4GALT1	2.4E-189	0.935033	0.215	0.082	3.8E-185	Saliva_Fibr	A4GALT
SETBP1	5.9E-189	0.713606	0.127	0.033	9.4E-185	Saliva_Fibr	SETBP1
LTBP3	4.9E-188	1.137039	0.3	0.145	7.8E-184	Saliva_Fibr	LTBP3
MRGPRF	3.6E-187	0.564576	0.104	0.023	5.8E-183	Saliva_Fibr	MRGPRF
EMILIN1	2.3E-186	0.550632	0.09	0.018	3.7E-182	Saliva_Fibr	EMILIN1
RAMP2.AS	1.4E-184	0.285573	0.044	0.004	2.2E-180	Saliva_Fibr	RAMP2.AS1
TXNIP1	1E-181	0.819483	0.559	0.37	1.6E-177	Saliva_Fibr	TXNIP
NOV1	1.8E-181	0.809867	0.103	0.023	2.8E-177	Saliva_Fibr	NOV
TENM1	2.9E-181	0.287593	0.041	0.004	4.7E-177	Saliva_Fibr	TENM1
GLIS2	4.8E-181	0.533824	0.085	0.017	7.6E-177	Saliva_Fibr	GLIS2
LEPROT1	7.4E-181	1.117079	0.407	0.251	1.2E-176	Saliva_Fibr	LEPROT
HHIP	1.3E-180	0.295465	0.045	0.005	2.1E-176	Saliva_Fibr	HHIP
SESTD1	1.8E-180	0.975889	0.213	0.083	3E-176	Saliva_Fibr	SESTD1
FAM43A1	2.4E-180	0.680181	0.125	0.033	3.8E-176	Saliva_Fibr	FAM43A
MAN1A11	3.8E-177	0.82036	0.214	0.082	6E-173	Saliva_Fibr	MAN1A1
C1QTNF21	1.2E-176	0.498062	0.095	0.021	2E-172	Saliva_Fibr	C1QTNF2
GSTM3	2E-176	0.570643	0.323	0.152	3.2E-172	Saliva_Fibr	GSTM3
HSD3B7	3.2E-176	0.685325	0.121	0.032	5.1E-172	Saliva_Fibr	HSD3B7
PRDX12	3.6E-176	0.782819	0.599	0.447	5.7E-172	Saliva_Fibr	PRDX1
GLI3	1.6E-174	0.519629	0.09	0.019	2.6E-170	Saliva_Fibr	GLI3
RECK	3.8E-174	0.687264	0.141	0.041	6.1E-170	Saliva_Fibr	RECK
DST	3.4E-173	0.848733	0.341	0.178	5.4E-169	Saliva_Fibr	DST
IGDCC4	4E-173	0.376979	0.058	0.008	6.5E-169	Saliva_Fibr	IGDCC4
XG	5.1E-171	0.590795	0.11	0.027	8.2E-167	Saliva_Fibr	XG
SLIT2	1.4E-170	0.584866	0.096	0.021	2.2E-166	Saliva_Fibr	SLIT2
MFGE8	1E-168	0.909993	0.231	0.095	1.7E-164	Saliva_Fibr	MFGE8
WSB11	3.9E-168	1.109619	0.399	0.245	6.2E-164	Saliva_Fibr	WSB1
BMP4	5.2E-168	0.528091	0.089	0.019	8.4E-164	Saliva_Fibr	BMP4
CTGF	2.8E-166	0.879589	0.253	0.107	4.5E-162	Saliva_Fibr	CTGF
PROS11	2.6E-165	0.701763	0.136	0.04	4.2E-161	Saliva_Fibr	PROS1
FAT4	1.7E-164	0.344273	0.055	0.008	2.8E-160	Saliva_Fibr	FAT4
PID1	7.4E-164	0.604903	0.124	0.034	1.2E-159	Saliva_Fibr	PID1
SH3BP51	8.9E-164	0.939776	0.249	0.11	1.4E-159	Saliva_Fibr	SH3BP5
ARHGAP21	2.3E-163	0.999071	0.217	0.091	3.6E-159	Saliva_Fibr	ARHGAP21

CYB5R31	3.2E-163	1.007167	0.37	0.216	5E-159	Saliva_Fibr	CYB5R3
SNX21	3.2E-162	0.622125	0.114	0.03	5.2E-158	Saliva_Fibr	SNX21
ANKRD35	1.3E-161	0.717286	0.125	0.036	2.1E-157	Saliva_Fibr	ANKRD35
MAFB	5.3E-160	1.015095	0.209	0.084	8.4E-156	Saliva_Fibr	MAFB
TSHZ22	9.7E-160	0.940103	0.239	0.105	1.5E-155	Saliva_Fibr	TSHZ2
BST1	2E-158	0.438234	0.079	0.016	3.3E-154	Saliva_Fibr	BST1
PLXDC21	2.4E-158	0.730826	0.134	0.04	3.8E-154	Saliva_Fibr	PLXDC2
SGCD	3.7E-158	0.514978	0.085	0.018	5.9E-154	Saliva_Fibr	SGCD
PNISR2	6E-157	0.965922	0.488	0.342	9.5E-153	Saliva_Fibr	PNISR
FBXO17	9.4E-156	0.643705	0.112	0.03	1.5E-151	Saliva_Fibr	FBXO17
COL4A21	1.1E-155	0.822112	0.153	0.051	1.8E-151	Saliva_Fibr	COL4A2
MARVELD1	1E-154	0.588773	0.111	0.03	1.6E-150	Saliva_Fibr	MARVELD1
GRK51	6.1E-153	0.479664	0.083	0.018	9.7E-149	Saliva_Fibr	GRK5
FCGRT1	1.7E-150	0.783585	0.431	0.272	2.7E-146	Saliva_Fibr	FCGRT
SLC9A9	8.3E-150	0.452539	0.071	0.014	1.3E-145	Saliva_Fibr	SLC9A9
TGFBI	2.4E-149	0.762993	0.167	0.059	3.8E-145	Saliva_Fibr	TGFBI
DYNLL12	5.5E-149	0.595138	0.691	0.556	8.8E-145	Saliva_Fibr	DYNLL1
CHRD	1.3E-147	0.318228	0.048	0.007	2.1E-143	Saliva_Fibr	CHRD
GSTP11	4.9E-147	0.501814	0.708	0.551	7.7E-143	Saliva_Fibr	GSTP1
CMKLR1	6.6E-147	0.364613	0.051	0.008	1.1E-142	Saliva_Fibr	CMKLR1
FILIP1L1	2.9E-146	0.808412	0.2	0.08	4.6E-142	Saliva_Fibr	FILIP1L
ZBTB201	4E-146	0.980302	0.262	0.13	6.4E-142	Saliva_Fibr	ZBTB20
ZEB2	4.9E-146	0.765992	0.208	0.084	7.8E-142	Saliva_Fibr	ZEB2
CYGB	7.3E-146	0.545113	0.093	0.023	1.2E-141	Saliva_Fibr	CYGB
AHNAK2	5.6E-145	0.786006	0.165	0.061	8.9E-141	Saliva_Fibr	AHNAK2
GNG111	3.1E-144	0.548817	0.248	0.109	4.9E-140	Saliva_Fibr	GNG11
INTS61	3.5E-144	1.115801	0.263	0.132	5.6E-140	Saliva_Fibr	INTS6
PRDM8	6.7E-144	0.266934	0.039	0.005	1.1E-139	Saliva_Fibr	PRDM8
COPZ2	9E-144	0.852361	0.193	0.079	1.4E-139	Saliva_Fibr	COPZ2
JUN2	4.5E-142	1.11667	0.601	0.483	7.2E-138	Saliva_Fibr	JUN
JDP2	1.2E-141	0.634438	0.127	0.04	1.8E-137	Saliva_Fibr	JDP2
ATP2B41	8.5E-141	0.774905	0.189	0.076	1.4E-136	Saliva_Fibr	ATP2B4
ADCY3	1.3E-140	0.491649	0.084	0.02	2.1E-136	Saliva_Fibr	ADCY3
FAM19A5	2.4E-140	0.393984	0.063	0.012	3.9E-136	Saliva_Fibr	FAM19A5
CCNI1	2.5E-140	0.691622	0.623	0.514	4E-136	Saliva_Fibr	CCNI
CDKN1C	2.7E-140	1.014837	0.292	0.156	4.3E-136	Saliva_Fibr	CDKN1C
ITGB1BP11	1.8E-139	0.905438	0.283	0.146	2.8E-135	Saliva_Fibr	ITGB1BP1
CALM13	2.1E-138	0.64688	0.717	0.649	3.3E-134	Saliva_Fibr	CALM1
CPE	5.8E-138	0.911731	0.225	0.098	9.2E-134	Saliva_Fibr	CPE
SRRM22	9.4E-138	0.967075	0.465	0.344	1.5E-133	Saliva_Fibr	SRRM2
KLF132	1.3E-137	0.78333	0.191	0.078	2.1E-133	Saliva_Fibr	KLF13
DIXDC1	2.6E-137	0.701527	0.132	0.043	4.1E-133	Saliva_Fibr	DIXDC1
MYCBP22	9.9E-137	1.01144	0.271	0.14	1.6E-132	Saliva_Fibr	MYCBP2
CBR3	2.1E-136	0.8216	0.189	0.078	3.4E-132	Saliva_Fibr	CBR3
SLITRK4	1.6E-135	0.504726	0.086	0.021	2.6E-131	Saliva_Fibr	SLITRK4
FMO3	7.2E-135	0.275837	0.027	0.002	1.1E-130	Saliva_Fibr	FMO3
ADAMTS2	7.3E-135	0.340714	0.055	0.009	1.2E-130	Saliva_Fibr	ADAMTS2

FAM114A1	3E-134	0.940634	0.241	0.119	4.8E-130	Saliva_Fibr	FAM114A1
FERMT21	3.1E-134	0.822249	0.188	0.078	5E-130	Saliva_Fibr	FERMT2
IFIT11	3.4E-134	0.57714	0.114	0.034	5.4E-130	Saliva_Fibr	IFIT1
RABAC13	2.5E-133	0.705539	0.538	0.4	4E-129	Saliva_Fibr	RABAC1
KDELC2	4E-133	0.597712	0.112	0.033	6.3E-129	Saliva_Fibr	KDELC2
SRI	5.6E-133	0.96528	0.373	0.237	9E-129	Saliva_Fibr	SRI
LARP6	7.7E-132	0.864922	0.185	0.077	1.2E-127	Saliva_Fibr	LARP6
ENPP21	2.9E-131	0.629138	0.103	0.029	4.6E-127	Saliva_Fibr	ENPP2
CAB39L	2E-130	0.719262	0.136	0.047	3.1E-126	Saliva_Fibr	CAB39L
RHOBTB31	3.4E-130	0.972179	0.233	0.114	5.4E-126	Saliva_Fibr	RHOBTB3
REEP53	3.8E-130	0.841906	0.438	0.304	6.1E-126	Saliva_Fibr	REEP5
ECE11	4.6E-130	0.756736	0.156	0.059	7.4E-126	Saliva_Fibr	ECE1
APOE	5.5E-130	0.856852	0.301	0.161	8.8E-126	Saliva_Fibr	APOE
COL5A3	2.8E-128	0.334774	0.051	0.008	4.5E-124	Saliva_Fibr	COL5A3
TCF7L11	2.9E-128	0.57958	0.099	0.028	4.7E-124	Saliva_Fibr	TCF7L1
SAMHD11	4.3E-128	0.706343	0.169	0.065	6.8E-124	Saliva_Fibr	SAMHD1
DENND2A	4.2E-127	0.266458	0.043	0.006	6.6E-123	Saliva_Fibr	DENND2A
ESD1	7.8E-123	0.979044	0.341	0.213	1.2E-118	Saliva_Fibr	ESD
CTSL1	1.1E-122	0.775385	0.216	0.1	1.7E-118	Saliva_Fibr	CTSL
DDX51	5.6E-122	0.602925	0.683	0.599	8.9E-118	Saliva_Fibr	DDX5
RAB301	1.6E-121	0.463945	0.079	0.019	2.6E-117	Saliva_Fibr	RAB30
ALDH1A1	1.2E-119	0.831997	0.174	0.073	1.9E-115	Saliva_Fibr	ALDH1A1
JAM3	5.9E-119	0.470675	0.083	0.022	9.4E-115	Saliva_Fibr	JAM3
PGRMC1	5E-118	1.000739	0.282	0.162	8E-114	Saliva_Fibr	PGRMC1
SCPEP1	9.3E-118	0.73595	0.187	0.081	1.5E-113	Saliva_Fibr	SCPEP1
MEIS3	1.2E-117	0.384168	0.057	0.011	1.8E-113	Saliva_Fibr	MEIS3
FKBP10	3.5E-117	0.526165	0.105	0.032	5.5E-113	Saliva_Fibr	FKBP10
SLC44A11	6.8E-117	0.774047	0.178	0.076	1.1E-112	Saliva_Fibr	SLC44A1
KCNQ1OT1	8.6E-116	1.181408	0.254	0.138	1.4E-111	Saliva_Fibr	KCNQ1OT1
SNTB21	8.8E-114	0.735722	0.177	0.076	1.4E-109	Saliva_Fibr	SNTB2
EMP21	1.1E-113	0.868371	0.314	0.186	1.8E-109	Saliva_Fibr	EMP2
HSPA1A1	1.7E-113	0.623984	0.468	0.321	2.7E-109	Saliva_Fibr	HSPA1A
SEMA3C	3E-113	0.633443	0.105	0.033	4.7E-109	Saliva_Fibr	SEMA3C
SEPW11	7.6E-113	0.780597	0.471	0.355	1.2E-108	Saliva_Fibr	SEPW1
TMEM431	9.9E-113	0.686852	0.161	0.066	1.6E-108	Saliva_Fibr	TMEM43
ZNF5211	1E-112	0.436972	0.08	0.021	1.7E-108	Saliva_Fibr	ZNF521
CD1091	1.5E-112	0.590514	0.123	0.043	2.3E-108	Saliva_Fibr	CD109
NR3C11	6.9E-111	0.884767	0.263	0.145	1.1E-106	Saliva_Fibr	NR3C1
F2R1	2.9E-110	0.895656	0.132	0.049	4.6E-106	Saliva_Fibr	F2R
ABL1	1.2E-109	0.628185	0.123	0.044	1.9E-105	Saliva_Fibr	ABL1
GLG11	1.3E-108	0.913399	0.233	0.123	2.1E-104	Saliva_Fibr	GLG1
SYNPO2	2.1E-108	0.410825	0.086	0.024	3.4E-104	Saliva_Fibr	SYNPO2
EMX2	3.7E-108	0.538868	0.095	0.029	6E-104	Saliva_Fibr	EMX2
PLEKHA5	4.2E-107	0.856796	0.18	0.082	6.7E-103	Saliva_Fibr	PLEKHA5
PRRT2	5.6E-106	0.350548	0.051	0.01	9E-102	Saliva_Fibr	PRRT2
PTPRG1	2.1E-105	0.561352	0.107	0.035	3.4E-101	Saliva_Fibr	PTPRG
TRIM471	8E-104	0.651134	0.141	0.056	1.3E-99	Saliva_Fibr	TRIM47

RFTN1	3.9E-103	0.543566	0.106	0.035	6.2E-99	Saliva_Fibr	RFTN1
SMAD51	1.4E-102	0.842469	0.189	0.091	2.2E-98	Saliva_Fibr	SMAD5
C1QTNF3	1.6E-102	0.639486	0.112	0.039	2.61E-98	Saliva_Fibr	C1QTNF3
IER5L1	3.3E-102	0.698452	0.192	0.09	5.23E-98	Saliva_Fibr	IER5L
PKD2	1.4E-100	0.482991	0.082	0.024	2.23E-96	Saliva_Fibr	PKD2
NUMBL	7.6E-100	0.477226	0.092	0.029	1.21E-95	Saliva_Fibr	NUMBL
BNC2	1.3E-99	0.439608	0.069	0.018	2.12E-95	Saliva_Fibr	BNC2
N4BP2L22	2E-99	0.914826	0.395	0.29	3.12E-95	Saliva_Fibr	N4BP2L2
FGD51	5.36E-98	0.406004	0.065	0.016	8.54E-94	Saliva_Fibr	FGD5
FUS1	1.37E-97	0.888936	0.383	0.273	2.18E-93	Saliva_Fibr	FUS
ENPP1	1.9E-97	0.394342	0.062	0.015	3.02E-93	Saliva_Fibr	ENPP1
HSP90AB1	2E-97	0.528502	0.659	0.567	3.18E-93	Saliva_Fibr	HSP90AB1
LGALS3BP2	4.56E-96	0.802093	0.28	0.167	7.27E-92	Saliva_Fibr	LGALS3BP
SCARF2	6.58E-96	0.328878	0.049	0.01	1.05E-91	Saliva_Fibr	SCARF2
SOBP	1.53E-95	0.586098	0.103	0.036	2.44E-91	Saliva_Fibr	SOBP
PLTP1	2.3E-95	0.790046	0.187	0.091	3.67E-91	Saliva_Fibr	PLTP
CC2D2A	2.65E-95	0.364811	0.059	0.014	4.22E-91	Saliva_Fibr	CC2D2A
TNS11	5.44E-95	0.601184	0.139	0.056	8.67E-91	Saliva_Fibr	TNS1
IL17D	1.68E-94	0.275753	0.049	0.01	2.69E-90	Saliva_Fibr	IL17D
CADPS	3.35E-94	0.339558	0.05	0.011	5.34E-90	Saliva_Fibr	CADPS
TMEM204	3.89E-94	0.480018	0.109	0.038	6.2E-90	Saliva_Fibr	TMEM204
ADH5	5.35E-94	0.86232	0.236	0.132	8.53E-90	Saliva_Fibr	ADH5
RET	7.8E-94	0.257567	0.026	0.003	1.24E-89	Saliva_Fibr	RET
DCHS11	1.72E-93	0.288644	0.051	0.011	2.75E-89	Saliva_Fibr	DCHS1
DDX172	3.22E-93	0.880628	0.438	0.351	5.13E-89	Saliva_Fibr	DDX17
MFAP2	1.22E-92	0.423315	0.081	0.024	1.94E-88	Saliva_Fibr	MFAP2
CTSF1	2.27E-92	0.92403	0.257	0.153	3.63E-88	Saliva_Fibr	CTSF
MTRNR2L8	3.67E-92	0.427717	0.425	0.281	5.85E-88	Saliva_Fibr	MTRNR2L8
NFATC4	4.13E-92	0.392919	0.062	0.016	6.59E-88	Saliva_Fibr	NFATC4
EMCN1	8.53E-92	0.568672	0.104	0.036	1.36E-87	Saliva_Fibr	EMCN
GAS61	1.08E-91	0.79635	0.187	0.092	1.73E-87	Saliva_Fibr	GAS6
TNRC6C	1.55E-91	0.705854	0.141	0.06	2.47E-87	Saliva_Fibr	TNRC6C
BOD1L12	5E-91	0.871096	0.225	0.124	7.98E-87	Saliva_Fibr	BOD1L1
PLP21	5.6E-91	0.678469	0.35	0.228	8.93E-87	Saliva_Fibr	PLP2
HMGB11	8.18E-91	0.528255	0.642	0.573	1.3E-86	Saliva_Fibr	HMGB1
FAM127A	1.44E-89	0.713072	0.18	0.088	2.3E-85	Saliva_Fibr	FAM127A
MARCKS1	1.45E-89	0.808676	0.388	0.28	2.31E-85	Saliva_Fibr	MARCKS
EIF52	2.03E-89	0.766031	0.448	0.357	3.23E-85	Saliva_Fibr	EIF5
ENHO	1.98E-88	0.386309	0.054	0.013	3.16E-84	Saliva_Fibr	ENHO
METTL7A	3.45E-88	0.798932	0.177	0.088	5.49E-84	Saliva_Fibr	METTL7A
METRNL	1.08E-87	0.728782	0.29	0.177	1.72E-83	Saliva_Fibr	METRNL
AGAP1	1.25E-87	0.535977	0.095	0.033	1.99E-83	Saliva_Fibr	AGAP1
NCAM1	3.97E-87	0.275676	0.044	0.009	6.32E-83	Saliva_Fibr	NCAM1
NTRK3	4.97E-87	0.560571	0.076	0.023	7.93E-83	Saliva_Fibr	NTRK3
EFHD1	8.96E-87	0.46425	0.085	0.027	1.43E-82	Saliva_Fibr	EFHD1
MAGEH12	9.37E-87	0.519765	0.114	0.043	1.49E-82	Saliva_Fibr	MAGEH1
TUBB1	1.69E-86	0.687171	0.424	0.307	2.69E-82	Saliva_Fibr	TUBB

BRI32	3.37E-86	0.696348	0.438	0.343	5.37E-82	Saliva_Fibr	BRI3
ANG1	1.04E-85	0.800202	0.188	0.097	1.65E-81	Saliva_Fibr	ANG
SOD1	2.89E-85	0.616311	0.517	0.412	4.6E-81	Saliva_Fibr	SOD1
PRSS23	6.97E-85	0.672535	0.186	0.091	1.11E-80	Saliva_Fibr	PRSS23
TMEM109[illegible]	9.61E-85	0.757931	0.225	0.125	1.53E-80	Saliva_Fibr	TMEM109
NENF2	1.02E-84	0.733865	0.418	0.325	1.62E-80	Saliva_Fibr	NENF
LRP6	1.4E-84	0.704573	0.129	0.054	2.23E-80	Saliva_Fibr	LRP6
PARK73	3.85E-84	0.62364	0.459	0.352	6.13E-80	Saliva_Fibr	PARK7
MTRNR2L1[illegible]	4.65E-84	0.591179	0.192	0.094	7.41E-80	Saliva_Fibr	MTRNR2L10
JAG11	5.46E-84	0.561871	0.12	0.048	8.72E-80	Saliva_Fibr	JAG1
PPIB2	1.67E-82	0.59533	0.49	0.391	2.67E-78	Saliva_Fibr	PPIB
HCFC1R1	9.51E-82	0.787779	0.231	0.133	1.52E-77	Saliva_Fibr	HCFC1R1
PHLDA3	2.29E-81	0.82969	0.207	0.114	3.65E-77	Saliva_Fibr	PHLDA3
FZD7	2.33E-81	0.542906	0.095	0.034	3.71E-77	Saliva_Fibr	FZD7
BHMT2	1.79E-80	0.28169	0.043	0.009	2.85E-76	Saliva_Fibr	BHMT2
VAMP51	2.81E-80	0.649716	0.275	0.165	4.48E-76	Saliva_Fibr	VAMP5
TMEM119	5.04E-80	0.343341	0.05	0.012	8.04E-76	Saliva_Fibr	TMEM119
CALU1	9.87E-80	0.879348	0.268	0.172	1.57E-75	Saliva_Fibr	CALU
RAI2	3.02E-79	0.405383	0.069	0.021	4.82E-75	Saliva_Fibr	RAI2
LDB21	3.2E-79	0.520454	0.105	0.04	5.1E-75	Saliva_Fibr	LDB2
RBM431	4.14E-79	0.423358	0.077	0.025	6.6E-75	Saliva_Fibr	RBM43
SLC16A7	4.92E-79	0.516494	0.101	0.038	7.85E-75	Saliva_Fibr	SLC16A7
KLF22	6.22E-79	0.584053	0.308	0.187	9.92E-75	Saliva_Fibr	KLF2
CCDC8	1.59E-77	0.31737	0.044	0.01	2.53E-73	Saliva_Fibr	CCDC8
RAB32	2.33E-77	0.671395	0.171	0.086	3.71E-73	Saliva_Fibr	RAB32
ZC2HC1A1	2.7E-77	0.596725	0.111	0.045	4.31E-73	Saliva_Fibr	ZC2HC1A
CYB5A1	1.06E-76	0.844934	0.275	0.179	1.68E-72	Saliva_Fibr	CYB5A
PTPRS	1.73E-76	0.64867	0.134	0.06	2.75E-72	Saliva_Fibr	PTPRS
RAB3IL1	9.6E-76	0.503074	0.088	0.031	1.53E-71	Saliva_Fibr	RAB3IL1
NOTCH2	2.95E-75	0.745619	0.175	0.091	4.71E-71	Saliva_Fibr	NOTCH2
P4HA2	3.11E-75	0.574665	0.114	0.048	4.96E-71	Saliva_Fibr	P4HA2
SRSF72	1.61E-74	0.729785	0.427	0.34	2.57E-70	Saliva_Fibr	SRSF7
COLGALT2	7.24E-74	0.382118	0.059	0.017	1.15E-69	Saliva_Fibr	COLGALT2
SERPINE2	9.5E-74	0.676369	0.13	0.057	1.52E-69	Saliva_Fibr	SERPINE2
KIAA0930	1.02E-73	0.652506	0.143	0.067	1.63E-69	Saliva_Fibr	KIAA0930
CYP4B1	1.03E-73	0.399655	0.068	0.021	1.64E-69	Saliva_Fibr	CYP4B1
MYOF1	1.46E-73	0.587434	0.126	0.056	2.32E-69	Saliva_Fibr	MYOF
DAD13	2.18E-73	0.670901	0.467	0.397	3.47E-69	Saliva_Fibr	DAD1
CSF11	4.77E-73	0.424746	0.087	0.031	7.61E-69	Saliva_Fibr	CSF1
CNPY4	1.27E-72	0.330479	0.062	0.018	2.03E-68	Saliva_Fibr	CNPY4
REV3L	1.34E-72	0.735407	0.172	0.089	2.14E-68	Saliva_Fibr	REV3L
LPP	1.72E-72	0.745841	0.307	0.208	2.74E-68	Saliva_Fibr	LPP
SLC25A32	2.03E-72	0.606312	0.515	0.454	3.23E-68	Saliva_Fibr	SLC25A3
WASF21	2.49E-72	0.818914	0.394	0.32	3.97E-68	Saliva_Fibr	WASF2
MPDZ1	6.31E-72	0.4607	0.081	0.029	1.01E-67	Saliva_Fibr	MPDZ
FLNC1	9.46E-72	0.287647	0.047	0.012	1.51E-67	Saliva_Fibr	FLNC
APPL12	1.19E-71	0.784529	0.207	0.121	1.9E-67	Saliva_Fibr	APPL1

ITGB5	2.28E-71	0.697511	0.152	0.075	3.63E-67	Saliva_Fibr	ITGB5
RRAGA1	5.38E-71	0.770243	0.213	0.126	8.59E-67	Saliva_Fibr	RRAGA
POLR2J32	5.96E-70	0.713838	0.176	0.094	9.51E-66	Saliva_Fibr	POLR2J3
FILIP11	6.23E-70	0.369981	0.066	0.021	9.93E-66	Saliva_Fibr	FILIP1
MTDH2	6.64E-70	0.728371	0.392	0.306	1.06E-65	Saliva_Fibr	MTDH
CD91	6.89E-70	0.426359	0.56	0.449	1.1E-65	Saliva_Fibr	CD9
ZNF703	1.05E-69	0.529372	0.103	0.042	1.68E-65	Saliva_Fibr	ZNF703
IL6ST1	1.74E-69	0.849577	0.307	0.22	2.78E-65	Saliva_Fibr	IL6ST
SUMO21	9.36E-69	0.477828	0.636	0.601	1.49E-64	Saliva_Fibr	SUMO2
TNFAIP6	1.34E-68	0.279576	0.166	0.081	2.14E-64	Saliva_Fibr	TNFAIP6
TACC11	1.44E-68	0.732381	0.256	0.164	2.29E-64	Saliva_Fibr	TACC1
DDX242	1.46E-68	0.706092	0.33	0.234	2.32E-64	Saliva_Fibr	DDX24
FKBP91	3.2E-68	0.599951	0.152	0.075	5.1E-64	Saliva_Fibr	FKBP9
KDELR12	8.09E-68	0.761541	0.332	0.248	1.29E-63	Saliva_Fibr	KDELR1
PRICKLE1	1.04E-67	0.470181	0.077	0.027	1.66E-63	Saliva_Fibr	PRICKLE1
GATAD11	1.46E-67	0.713078	0.147	0.074	2.33E-63	Saliva_Fibr	GATAD1
PAPLN	1.73E-67	0.251666	0.039	0.009	2.76E-63	Saliva_Fibr	PAPLN
ARL21	3.96E-67	0.723018	0.302	0.207	6.32E-63	Saliva_Fibr	ARL2
XIST3	4.32E-67	0.958147	0.207	0.12	6.89E-63	Saliva_Fibr	XIST
AGO21	5.19E-67	0.58572	0.114	0.05	8.28E-63	Saliva_Fibr	AGO2
PDE5A	5.61E-67	0.441594	0.062	0.019	8.95E-63	Saliva_Fibr	PDE5A
FAM20C	6.81E-66	0.43309	0.077	0.027	1.09E-61	Saliva_Fibr	FAM20C
EMILIN2	1.02E-65	0.352854	0.08	0.029	1.62E-61	Saliva_Fibr	EMILIN2
PRKG1	2.17E-65	0.416545	0.063	0.02	3.47E-61	Saliva_Fibr	PRKG1
SCN1B1	3.4E-65	0.277266	0.046	0.012	5.42E-61	Saliva_Fibr	SCN1B
DYNC2H1	3.68E-65	0.497487	0.082	0.031	5.87E-61	Saliva_Fibr	DYNC2H1
SEPT11	4.34E-65	0.616676	0.161	0.083	6.92E-61	Saliva_Fibr	SEPT11
PSMB12	4.35E-65	0.666528	0.458	0.398	6.94E-61	Saliva_Fibr	PSMB1
MICU3	4.59E-65	0.432838	0.074	0.026	7.32E-61	Saliva_Fibr	MICU3
FLNA1	1.26E-64	0.530295	0.243	0.146	2E-60	Saliva_Fibr	FLNA
PSAP	3.24E-64	0.257539	0.514	0.432	5.17E-60	Saliva_Fibr	PSAP
MTRNR2L1	9.22E-64	0.714954	0.213	0.123	1.47E-59	Saliva_Fibr	MTRNR2L1
ALDH2	1.45E-63	0.764394	0.201	0.121	2.31E-59	Saliva_Fibr	ALDH2
RASD1	4.3E-63	0.645465	0.148	0.075	6.85E-59	Saliva_Fibr	RASD1
PCYOX11	4.87E-63	0.809277	0.208	0.129	7.76E-59	Saliva_Fibr	PCYOX1
PPFIBP11	6.16E-63	0.765138	0.181	0.103	9.83E-59	Saliva_Fibr	PPFIBP1
FEZ1	7.01E-63	0.457094	0.088	0.034	1.12E-58	Saliva_Fibr	FEZ1
BHLHE411	7.66E-63	0.575096	0.101	0.043	1.22E-58	Saliva_Fibr	BHLHE41
RP11.983P	8.5E-63	0.409376	0.077	0.028	1.36E-58	Saliva_Fibr	RP11.983P16.4
TAGLN22	1.4E-62	0.465969	0.505	0.405	2.23E-58	Saliva_Fibr	TAGLN2
APP1	2.44E-62	0.744149	0.326	0.241	3.9E-58	Saliva_Fibr	APP
TPM11	3.25E-62	0.554033	0.278	0.184	5.19E-58	Saliva_Fibr	TPM1
YBX31	3.32E-62	0.430519	0.566	0.493	5.29E-58	Saliva_Fibr	YBX3
CLEC3B	7.97E-62	0.306048	0.063	0.021	1.27E-57	Saliva_Fibr	CLEC3B
DOCK41	1.17E-61	0.340462	0.062	0.02	1.86E-57	Saliva_Fibr	DOCK4
SPG20	1.78E-61	0.704722	0.167	0.092	2.83E-57	Saliva_Fibr	SPG20
CTSO	2.33E-61	0.61789	0.126	0.06	3.71E-57	Saliva_Fibr	CTSO

KCTD121	3.35E-61	0.588274	0.168	0.09	5.34E-57	Saliva_Fibr	KCTD12
TRIOBP1	7.96E-61	0.74164	0.182	0.105	1.27E-56	Saliva_Fibr	TRIOBP
SRP141	4.55E-60	0.334627	0.736	0.7	7.25E-56	Saliva_Fibr	SRP14
ZNF37A2	7.45E-60	0.614708	0.116	0.054	1.19E-55	Saliva_Fibr	ZNF37A
MAMDC2	1.04E-59	0.454485	0.093	0.038	1.66E-55	Saliva_Fibr	MAMDC2
RHOQ1	2.01E-59	0.659871	0.156	0.084	3.21E-55	Saliva_Fibr	RHOQ
TNFSF121	2.28E-59	0.544501	0.122	0.058	3.64E-55	Saliva_Fibr	TNFSF12
TNRC6B1	2.41E-59	0.777441	0.251	0.169	3.85E-55	Saliva_Fibr	TNRC6B
TUSC1	3.69E-59	0.629669	0.132	0.066	5.89E-55	Saliva_Fibr	TUSC1
TMEM98	4.56E-59	0.650614	0.142	0.074	7.27E-55	Saliva_Fibr	TMEM98
TMOD1	5.92E-59	0.265059	0.045	0.012	9.44E-55	Saliva_Fibr	TMOD1
NCL2	1.14E-58	0.694042	0.352	0.268	1.82E-54	Saliva_Fibr	NCL
CBX6	1.92E-58	0.568017	0.154	0.082	3.07E-54	Saliva_Fibr	CBX6
IFI27L21	2.12E-58	0.59689	0.223	0.137	3.39E-54	Saliva_Fibr	IFI27L2
PAK3	3.23E-58	0.253982	0.041	0.011	5.15E-54	Saliva_Fibr	PAK3
OPHN1	6.89E-58	0.421096	0.073	0.027	1.1E-53	Saliva_Fibr	OPHN1
CYP27A1	1.22E-57	0.556873	0.105	0.048	1.94E-53	Saliva_Fibr	CYP27A1
LRP3	1.73E-57	0.469007	0.08	0.031	2.76E-53	Saliva_Fibr	LRP3
TGFBR21	2.78E-57	0.559825	0.194	0.113	4.44E-53	Saliva_Fibr	TGFBR2
ECI21	3.65E-57	0.65425	0.166	0.094	5.82E-53	Saliva_Fibr	ECI2
MAGED21	5.8E-57	0.729391	0.233	0.153	9.24E-53	Saliva_Fibr	MAGED2
TMEM55A	8.54E-57	0.4475	0.093	0.039	1.36E-52	Saliva_Fibr	TMEM55A
INMT1	1.26E-56	0.329818	0.057	0.018	2.01E-52	Saliva_Fibr	INMT
SLC29A11	1.47E-56	0.518903	0.102	0.045	2.35E-52	Saliva_Fibr	SLC29A1
IFI161	1.91E-56	0.698341	0.281	0.196	3.05E-52	Saliva_Fibr	IFI16
MORC4	2.78E-56	0.478174	0.09	0.038	4.44E-52	Saliva_Fibr	MORC4
FAXDC2	3.05E-56	0.43357	0.074	0.028	4.86E-52	Saliva_Fibr	FAXDC2
CTDSPL1	3.67E-56	0.691851	0.15	0.083	5.86E-52	Saliva_Fibr	CTDSPL
WLS	8.51E-56	0.484967	0.095	0.041	1.36E-51	Saliva_Fibr	WLS
TBC1D2B	1.38E-55	0.396726	0.079	0.031	2.2E-51	Saliva_Fibr	TBC1D2B
TSHZ3	4.44E-55	0.291229	0.046	0.013	7.08E-51	Saliva_Fibr	TSHZ3
YWHAB1	6.13E-55	0.618194	0.459	0.411	9.78E-51	Saliva_Fibr	YWHAB
RASSF8.AS	9.16E-55	0.449745	0.072	0.027	1.46E-50	Saliva_Fibr	RASSF8.AS1
TNFSF13B	9.65E-55	0.432693	0.094	0.04	1.54E-50	Saliva_Fibr	TNFSF13B
CCPG13	1.53E-54	0.763545	0.242	0.166	2.44E-50	Saliva_Fibr	CCPG1
KDSR	3.12E-54	0.721118	0.202	0.127	4.98E-50	Saliva_Fibr	KDSR
RNF24	3.31E-54	0.639889	0.125	0.063	5.27E-50	Saliva_Fibr	RNF24
TULP3	7.59E-54	0.537146	0.105	0.049	1.21E-49	Saliva_Fibr	TULP3
C1orf21	7.7E-54	0.651866	0.17	0.099	1.23E-49	Saliva_Fibr	C1orf21
SHC3	1.28E-53	0.257187	0.038	0.01	2.04E-49	Saliva_Fibr	SHC3
PSIP11	1.81E-53	0.61034	0.2	0.121	2.89E-49	Saliva_Fibr	PSIP1
S1PR3	2.4E-53	0.257447	0.039	0.01	3.83E-49	Saliva_Fibr	S1PR3
BCL7C1	2.62E-53	0.670315	0.19	0.116	4.17E-49	Saliva_Fibr	BCL7C
IL15RA	3.94E-53	0.304048	0.052	0.016	6.28E-49	Saliva_Fibr	IL15RA
MOB3B	7.81E-53	0.383405	0.067	0.025	1.25E-48	Saliva_Fibr	MOB3B
LUC7L32	1.03E-52	0.796478	0.333	0.264	1.65E-48	Saliva_Fibr	LUC7L3
CAMKK21	1.22E-52	0.490587	0.098	0.044	1.95E-48	Saliva_Fibr	CAMKK2

DYNC1H11	3.42E-52	0.748676	0.21	0.137	5.45E-48	Saliva_Fibr	DYNC1H1
SEC622	3.85E-52	0.577923	0.456	0.4	6.13E-48	Saliva_Fibr	SEC62
DSEL	4.37E-52	0.451026	0.081	0.034	6.97E-48	Saliva_Fibr	DSEL
NDUFB83	4.79E-52	0.759671	0.248	0.176	7.63E-48	Saliva_Fibr	NDUFB8
RASSF8	6.3E-52	0.44963	0.121	0.059	1E-47	Saliva_Fibr	RASSF8
VKORC12	6.41E-52	0.684658	0.296	0.222	1.02E-47	Saliva_Fibr	VKORC1
MBNL11	6.46E-52	0.674237	0.249	0.168	1.03E-47	Saliva_Fibr	MBNL1
SLC12A4	1.28E-51	0.330635	0.057	0.02	2.04E-47	Saliva_Fibr	SLC12A4
CENPC1	1.46E-51	0.579189	0.12	0.06	2.33E-47	Saliva_Fibr	CENPC
RPL222	6.21E-51	0.287538	0.783	0.691	9.9E-47	Saliva_Fibr	RPL22
CYP2U1	6.62E-51	0.270918	0.044	0.013	1.06E-46	Saliva_Fibr	CYP2U1
PTN	8.75E-51	0.831137	0.129	0.067	1.39E-46	Saliva_Fibr	PTN
FAM110B	1.62E-50	0.500259	0.086	0.037	2.59E-46	Saliva_Fibr	FAM110B
CNRIP11	5.47E-50	0.391248	0.086	0.037	8.72E-46	Saliva_Fibr	CNRIP1
PJA22	7.06E-50	0.755586	0.221	0.149	1.13E-45	Saliva_Fibr	PJA2
MRFAP1	7.79E-50	0.742952	0.33	0.264	1.24E-45	Saliva_Fibr	MRFAP1
TSKU	9.8E-50	0.460091	0.08	0.034	1.56E-45	Saliva_Fibr	TSKU
LRRK2	1.01E-49	0.339484	0.058	0.021	1.62E-45	Saliva_Fibr	LRRK2
KLF91	1.07E-49	0.64064	0.174	0.104	1.71E-45	Saliva_Fibr	KLF9
STXBP6	1.23E-49	0.402941	0.076	0.031	1.96E-45	Saliva_Fibr	STXBP6
FAM13A	1.42E-49	0.555104	0.099	0.046	2.26E-45	Saliva_Fibr	FAM13A
PRTFDC1	1.5E-49	0.259046	0.041	0.012	2.39E-45	Saliva_Fibr	PRTFDC1
TGOLN22	1.53E-49	0.736036	0.242	0.169	2.43E-45	Saliva_Fibr	TGOLN2
SOCS1	1.64E-49	0.408362	0.1	0.046	2.62E-45	Saliva_Fibr	SOCS1
GINM11	1.69E-49	0.721913	0.217	0.146	2.7E-45	Saliva_Fibr	GINM1
VSTM4	2.89E-49	0.407789	0.069	0.027	4.61E-45	Saliva_Fibr	VSTM4
PTPRA	3.56E-49	0.673885	0.177	0.109	5.68E-45	Saliva_Fibr	PTPRA
PTGER3	5.9E-49	0.452836	0.072	0.029	9.42E-45	Saliva_Fibr	PTGER3
DIAPH21	6.29E-49	0.50494	0.108	0.053	1E-44	Saliva_Fibr	DIAPH2
KIDINS220	6.93E-49	0.680547	0.167	0.1	1.11E-44	Saliva_Fibr	KIDINS220
PDLIM71	7.59E-49	0.531579	0.121	0.062	1.21E-44	Saliva_Fibr	PDLIM7
CACNB4	9.83E-49	0.318055	0.05	0.016	1.57E-44	Saliva_Fibr	CACNB4
STAT31	1.23E-48	0.696854	0.263	0.188	1.96E-44	Saliva_Fibr	STAT3
RECQL1	1.83E-48	0.478831	0.111	0.055	2.92E-44	Saliva_Fibr	RECQL
CHL1	2.19E-48	0.299557	0.042	0.013	3.5E-44	Saliva_Fibr	CHL1
ZNF6381	2.36E-48	0.739711	0.197	0.128	3.76E-44	Saliva_Fibr	ZNF638
ATRAID2	5.09E-48	0.638853	0.355	0.289	8.12E-44	Saliva_Fibr	ATRAID
SLC16A1.A	9.43E-48	0.311945	0.058	0.021	1.5E-43	Saliva_Fibr	SLC16A1.AS1
SPRY2	1.73E-47	0.430195	0.068	0.027	2.76E-43	Saliva_Fibr	SPRY2
ROCK21	2.46E-47	0.672219	0.158	0.093	3.93E-43	Saliva_Fibr	ROCK2
NME32	9.36E-47	0.687411	0.279	0.207	1.49E-42	Saliva_Fibr	NME3
CDC42EP2	1.03E-46	0.402596	0.081	0.035	1.65E-42	Saliva_Fibr	CDC42EP2
OLFM11	1.22E-46	0.303068	0.062	0.023	1.95E-42	Saliva_Fibr	OLFM1
TNFAIP2	1.5E-46	0.512641	0.215	0.137	2.4E-42	Saliva_Fibr	TNFAIP2
MAP9	1.96E-46	0.468852	0.085	0.038	3.12E-42	Saliva_Fibr	MAP9
COX7A11	2.69E-46	0.577182	0.21	0.137	4.29E-42	Saliva_Fibr	COX7A1
CD276	2.91E-46	0.50492	0.099	0.048	4.65E-42	Saliva_Fibr	CD276

PCNXL2	7.69E-46	0.30835	0.047	0.015	1.23E-41	Saliva_Fibr	PCNXL2
PDLIM2	8.59E-46	0.670436	0.249	0.176	1.37E-41	Saliva_Fibr	PDLIM2
DSTN	1.28E-45	0.317635	0.617	0.561	2.04E-41	Saliva_Fibr	DSTN
LMAN13	1.52E-45	0.708079	0.26	0.191	2.43E-41	Saliva_Fibr	LMAN1
CHCHD21	2.53E-45	0.347051	0.642	0.62	4.04E-41	Saliva_Fibr	CHCHD2
GPX3	3.06E-45	0.605211	0.195	0.124	4.89E-41	Saliva_Fibr	GPX3
CCDC102B	3.51E-45	0.389292	0.062	0.024	5.6E-41	Saliva_Fibr	CCDC102B
MT.ATP83	7.78E-45	0.600024	0.203	0.133	1.24E-40	Saliva_Fibr	MT.ATP8
MPZL11	2.09E-44	0.78113	0.203	0.139	3.33E-40	Saliva_Fibr	MPZL1
IL16	2.31E-44	0.33588	0.107	0.052	3.68E-40	Saliva_Fibr	IL16
HGSNAT1	2.52E-44	0.539593	0.126	0.069	4.02E-40	Saliva_Fibr	HGSNAT
GSTO1	3.02E-44	0.602797	0.283	0.211	4.81E-40	Saliva_Fibr	GSTO1
ZRANB22	4.01E-44	0.78519	0.25	0.184	6.4E-40	Saliva_Fibr	ZRANB2
GALNT16	6.6E-44	0.334198	0.058	0.022	1.05E-39	Saliva_Fibr	GALNT16
FUCA21	9.92E-44	0.640522	0.144	0.085	1.58E-39	Saliva_Fibr	FUCA2
DTWD11	1.84E-43	0.642765	0.137	0.079	2.93E-39	Saliva_Fibr	DTWD1
TLR41	2.09E-43	0.257725	0.043	0.014	3.33E-39	Saliva_Fibr	TLR4
C1QTNF1	5.06E-43	0.401234	0.076	0.033	8.07E-39	Saliva_Fibr	C1QTNF1
SLC16A4	7.07E-43	0.297878	0.048	0.016	1.13E-38	Saliva_Fibr	SLC16A4
TPST1	1.99E-42	0.463916	0.105	0.053	3.17E-38	Saliva_Fibr	TPST1
CERCAM1	2.77E-42	0.536884	0.108	0.056	4.41E-38	Saliva_Fibr	CERCAM
CRISPLD2	2.77E-42	0.45344	0.109	0.055	4.42E-38	Saliva_Fibr	CRISPLD2
NCOA71	2.78E-42	0.549136	0.171	0.106	4.43E-38	Saliva_Fibr	NCOA7
CYR61	3.46E-42	0.438976	0.181	0.111	5.52E-38	Saliva_Fibr	CYR61
SEC31A1	4.54E-42	0.783164	0.274	0.215	7.25E-38	Saliva_Fibr	SEC31A
FAM195B	7.14E-42	0.601096	0.196	0.129	1.14E-37	Saliva_Fibr	FAM195B
ADD11	2.57E-41	0.664899	0.2	0.135	4.1E-37	Saliva_Fibr	ADD1
AKR1C2	2.62E-41	0.376745	0.063	0.026	4.19E-37	Saliva_Fibr	AKR1C2
ANTXR1	3.06E-41	0.403811	0.097	0.048	4.88E-37	Saliva_Fibr	ANTXR1
TLN11	3.17E-41	0.61107	0.195	0.128	5.06E-37	Saliva_Fibr	TLN1
PPP1R102	8.23E-41	0.70606	0.215	0.151	1.31E-36	Saliva_Fibr	PPP1R10
CDC42EP4:	8.31E-41	0.539243	0.105	0.054	1.32E-36	Saliva_Fibr	CDC42EP4
SSB1	8.35E-41	0.599398	0.265	0.196	1.33E-36	Saliva_Fibr	SSB
CAMK2N1	9.94E-41	0.532536	0.185	0.118	1.59E-36	Saliva_Fibr	CAMK2N1
FKBP7	1.07E-40	0.484242	0.106	0.055	1.71E-36	Saliva_Fibr	FKBP7
TPBG	1.66E-40	0.61822	0.132	0.076	2.64E-36	Saliva_Fibr	TPBG
RCAN2	2.8E-40	0.348821	0.068	0.029	4.47E-36	Saliva_Fibr	RCAN2
GALNT10	3.1E-40	0.352676	0.066	0.028	4.95E-36	Saliva_Fibr	GALNT10
LGR4	7.87E-40	0.41147	0.076	0.035	1.26E-35	Saliva_Fibr	LGR4
CD1512	9.38E-40	0.606246	0.283	0.218	1.5E-35	Saliva_Fibr	CD151
RIF11	1.35E-39	0.600694	0.149	0.09	2.15E-35	Saliva_Fibr	RIF1
CMBL	1.5E-39	0.43502	0.077	0.036	2.39E-35	Saliva_Fibr	CMBL
SSBP41	1.51E-39	0.545669	0.163	0.1	2.4E-35	Saliva_Fibr	SSBP4
PIGT1	1.84E-39	0.700743	0.224	0.162	2.93E-35	Saliva_Fibr	PIGT
CBR1	3.86E-39	0.61848	0.178	0.117	6.15E-35	Saliva_Fibr	CBR1
LRPAP13	9.4E-39	0.579479	0.327	0.268	1.5E-34	Saliva_Fibr	LRPAP1
FAM129A2	1.15E-38	0.632231	0.162	0.102	1.83E-34	Saliva_Fibr	FAM129A

CLEC2B1	1.39E-38	0.490188	0.225	0.154	2.22E-34	Saliva_Fibr	CLEC2B
C17orf58	3.78E-38	0.433341	0.08	0.037	6.03E-34	Saliva_Fibr	C17orf58
PAM1	3.86E-38	0.610397	0.203	0.14	6.16E-34	Saliva_Fibr	PAM
CIRBP	3.87E-38	0.382416	0.579	0.545	6.18E-34	Saliva_Fibr	CIRBP
SVIL	9.42E-38	0.64659	0.177	0.118	1.5E-33	Saliva_Fibr	SVIL
AKT31	1.16E-37	0.447761	0.08	0.038	1.85E-33	Saliva_Fibr	AKT3
RILPL1	1.26E-37	0.25468	0.038	0.013	2.01E-33	Saliva_Fibr	RILPL1
ASPH1	1.4E-37	0.697915	0.248	0.189	2.23E-33	Saliva_Fibr	ASPH
PTGIR	1.5E-37	0.295427	0.051	0.019	2.4E-33	Saliva_Fibr	PTGIR
PRRC2C2	1.72E-37	0.708322	0.326	0.277	2.75E-33	Saliva_Fibr	PRRC2C
PHF31	2.13E-37	0.692177	0.189	0.131	3.4E-33	Saliva_Fibr	PHF3
SLC2A4RG1	4.68E-37	0.607689	0.155	0.097	7.47E-33	Saliva_Fibr	SLC2A4RG
FYN1	4.91E-37	0.331164	0.12	0.065	7.83E-33	Saliva_Fibr	FYN
AQP11	8.07E-37	0.31725	0.159	0.094	1.29E-32	Saliva_Fibr	AQP1
ZNF322	2.7E-36	0.420545	0.079	0.038	4.31E-32	Saliva_Fibr	ZNF322
EDNRB1	2.92E-36	0.311914	0.058	0.024	4.65E-32	Saliva_Fibr	EDNRB
PRKDC2	4.91E-36	0.606632	0.152	0.097	7.83E-32	Saliva_Fibr	PRKDC
ZFPM1	5.54E-36	0.354127	0.061	0.026	8.84E-32	Saliva_Fibr	ZFPM1
CRTAP1	9.01E-36	0.569992	0.172	0.113	1.44E-31	Saliva_Fibr	CRTAP
SLC27A31	1.48E-35	0.301128	0.063	0.027	2.36E-31	Saliva_Fibr	SLC27A3
FAIM2	3.33E-35	0.286787	0.046	0.017	5.31E-31	Saliva_Fibr	FAIM2
GNAI22	3.39E-35	0.529952	0.286	0.224	5.41E-31	Saliva_Fibr	GNAI2
LINC00969	3.48E-35	0.65977	0.166	0.109	5.55E-31	Saliva_Fibr	LINC00969
TTC32	4.63E-35	0.610057	0.29	0.232	7.39E-31	Saliva_Fibr	TTC3
SGCB	5.1E-35	0.539413	0.116	0.067	8.14E-31	Saliva_Fibr	SGCB
TMOD2	1.03E-34	0.278594	0.045	0.017	1.64E-30	Saliva_Fibr	TMOD2
PHYH	1.93E-34	0.52682	0.12	0.07	3.07E-30	Saliva_Fibr	PHYH
KMT2A2	3.26E-34	0.614053	0.186	0.129	5.2E-30	Saliva_Fibr	KMT2A
PDE4DIP	3.36E-34	0.536978	0.113	0.065	5.36E-30	Saliva_Fibr	PDE4DIP
GLIPR2	3.93E-34	0.447676	0.138	0.083	6.27E-30	Saliva_Fibr	GLIPR2
MAP42	4.06E-34	0.631935	0.184	0.127	6.47E-30	Saliva_Fibr	MAP4
MT.ND63	4.39E-34	0.558566	0.166	0.109	7E-30	Saliva_Fibr	MT.ND6
GPR153	5.27E-34	0.335047	0.059	0.025	8.41E-30	Saliva_Fibr	GPR153
AFF3	6.58E-34	0.30268	0.053	0.022	1.05E-29	Saliva_Fibr	AFF3
AKAP131	8.39E-34	0.709632	0.268	0.215	1.34E-29	Saliva_Fibr	AKAP13
VAT1	1.09E-33	0.649695	0.192	0.137	1.73E-29	Saliva_Fibr	VAT1
PURA2	1.93E-33	0.693809	0.178	0.124	3.08E-29	Saliva_Fibr	PURA
ANKRD121	2.3E-33	0.686459	0.358	0.322	3.66E-29	Saliva_Fibr	ANKRD12
PROCR	2.64E-33	0.3107	0.065	0.03	4.21E-29	Saliva_Fibr	PROCR
TGFB3	3E-33	0.269822	0.044	0.017	4.79E-29	Saliva_Fibr	TGFB3
PQLC31	4.04E-33	0.538194	0.144	0.09	6.45E-29	Saliva_Fibr	PQLC3
LMO42	4.35E-33	0.66245	0.219	0.163	6.94E-29	Saliva_Fibr	LMO4
FST	4.39E-33	0.301674	0.058	0.025	7.01E-29	Saliva_Fibr	FST
TRAM21	4.65E-33	0.336149	0.074	0.036	7.42E-29	Saliva_Fibr	TRAM2
WDFY2	5.25E-33	0.46458	0.09	0.048	8.38E-29	Saliva_Fibr	WDFY2
ANO6	1.06E-32	0.539521	0.113	0.066	1.69E-28	Saliva_Fibr	ANO6
ZFHX3	1.33E-32	0.601678	0.14	0.088	2.11E-28	Saliva_Fibr	ZFHX3

MTPN1	1.59E-32	0.596916	0.205	0.148	2.54E-28	Saliva_Fibr	MTPN
SDK1	1.62E-32	0.262659	0.052	0.021	2.59E-28	Saliva_Fibr	SDK1
COPRS	1.72E-32	0.655282	0.203	0.149	2.74E-28	Saliva_Fibr	COPRS
CD44	1.87E-32	0.285914	0.422	0.346	2.98E-28	Saliva_Fibr	CD44
CMTM3	2.09E-32	0.39324	0.126	0.074	3.33E-28	Saliva_Fibr	CMTM3
PCDH7	2.2E-32	0.335317	0.05	0.021	3.5E-28	Saliva_Fibr	PCDH7
RGL1	2.28E-32	0.300352	0.052	0.021	3.63E-28	Saliva_Fibr	RGL1
ARHGAP1C	2.53E-32	0.528427	0.13	0.079	4.04E-28	Saliva_Fibr	ARHGAP10
RNF1151	2.88E-32	0.595746	0.158	0.105	4.59E-28	Saliva_Fibr	RNF115
NDRG11	3.12E-32	0.483022	0.203	0.142	4.97E-28	Saliva_Fibr	NDRG1
KREMEN1	5.87E-32	0.436368	0.087	0.046	9.37E-28	Saliva_Fibr	KREMEN1
PLXNA1	6.33E-32	0.318525	0.051	0.021	1.01E-27	Saliva_Fibr	PLXNA1
EXTL2	8.02E-32	0.417309	0.077	0.038	1.28E-27	Saliva_Fibr	EXTL2
VCL	1.07E-31	0.595688	0.165	0.112	1.7E-27	Saliva_Fibr	VCL
CAMK11	1.11E-31	0.31944	0.07	0.034	1.77E-27	Saliva_Fibr	CAMK1
XYLT1	1.31E-31	0.273322	0.047	0.018	2.09E-27	Saliva_Fibr	XYLT1
COL18A11	1.43E-31	0.429412	0.121	0.072	2.28E-27	Saliva_Fibr	COL18A1
GLT8D11	1.52E-31	0.565916	0.138	0.087	2.42E-27	Saliva_Fibr	GLT8D1
CAST1	1.55E-31	0.534469	0.424	0.397	2.47E-27	Saliva_Fibr	CAST
PCNXL41	1.56E-31	0.503053	0.102	0.058	2.49E-27	Saliva_Fibr	PCNXL4
PRR5	1.85E-31	0.363122	0.084	0.043	2.94E-27	Saliva_Fibr	PRR5
CCDC146	1.94E-31	0.425521	0.077	0.039	3.09E-27	Saliva_Fibr	CCDC146
LIMS21	2.46E-31	0.28627	0.066	0.031	3.93E-27	Saliva_Fibr	LIMS2
BPTF1	2.47E-31	0.713999	0.215	0.162	3.93E-27	Saliva_Fibr	BPTF
PREX21	2.57E-31	0.287277	0.05	0.021	4.1E-27	Saliva_Fibr	PREX2
AKR1C31	3.19E-31	0.44721	0.104	0.058	5.09E-27	Saliva_Fibr	AKR1C3
TSPO1	4E-31	0.28819	0.499	0.465	6.38E-27	Saliva_Fibr	TSPO
SFPQ1	4.99E-31	0.623143	0.327	0.285	7.96E-27	Saliva_Fibr	SFPQ
RPL7L11	7.19E-31	0.593332	0.17	0.116	1.15E-26	Saliva_Fibr	RPL7L1
CD302	1.15E-30	0.528718	0.108	0.063	1.83E-26	Saliva_Fibr	CD302
TRMT11	2.27E-30	0.380763	0.075	0.038	3.63E-26	Saliva_Fibr	TRMT11
PKD1	2.73E-30	0.448916	0.087	0.047	4.35E-26	Saliva_Fibr	PKD1
SEC11A2	2.87E-30	0.587026	0.352	0.314	4.58E-26	Saliva_Fibr	SEC11A
BACE1	3.22E-30	0.368555	0.072	0.036	5.14E-26	Saliva_Fibr	BACE1
SMC31	3.95E-30	0.387801	0.198	0.141	6.3E-26	Saliva_Fibr	SMC3
MCOLN3	5.38E-30	0.283887	0.049	0.021	8.59E-26	Saliva_Fibr	MCOLN3
ILK	6.34E-30	0.611503	0.198	0.146	1.01E-25	Saliva_Fibr	ILK
CAPS	6.6E-30	0.610022	0.117	0.071	1.05E-25	Saliva_Fibr	CAPS
HIF3A1	6.73E-30	0.253441	0.041	0.016	1.07E-25	Saliva_Fibr	HIF3A
TCEAL41	7.43E-30	0.544731	0.213	0.158	1.18E-25	Saliva_Fibr	TCEAL4
MAML2	8.94E-30	0.45848	0.09	0.049	1.43E-25	Saliva_Fibr	MAML2
LDHA	9.14E-30	0.276794	0.495	0.438	1.46E-25	Saliva_Fibr	LDHA
KIFAP3	1E-29	0.472706	0.105	0.061	1.6E-25	Saliva_Fibr	KIFAP3
SMIM101	1.17E-29	0.266775	0.047	0.02	1.87E-25	Saliva_Fibr	SMIM10
TTC281	1.25E-29	0.375373	0.067	0.032	2E-25	Saliva_Fibr	TTC28
TMEM237	1.99E-29	0.402682	0.079	0.041	3.17E-25	Saliva_Fibr	TMEM237
H6PD1	2.11E-29	0.529682	0.109	0.065	3.37E-25	Saliva_Fibr	H6PD

CHST14	2.32E-29	0.258475	0.04	0.015	3.71E-25	Saliva_Fibr	CHST14
UBTF1	2.42E-29	0.524719	0.133	0.084	3.86E-25	Saliva_Fibr	UBTF
ITGA51	2.8E-29	0.313931	0.076	0.038	4.47E-25	Saliva_Fibr	ITGA5
MBTPS11	2.94E-29	0.549035	0.132	0.084	4.69E-25	Saliva_Fibr	MBTPS1
PXK	4.19E-29	0.385376	0.08	0.042	6.68E-25	Saliva_Fibr	PXK
SLC25A361	4.59E-29	0.634343	0.172	0.122	7.32E-25	Saliva_Fibr	SLC25A36
AGL	4.78E-29	0.410317	0.078	0.041	7.63E-25	Saliva_Fibr	AGL
LRCH31	5.26E-29	0.386017	0.077	0.04	8.39E-25	Saliva_Fibr	LRCH3
DEXI	5.94E-29	0.495082	0.118	0.072	9.48E-25	Saliva_Fibr	DEXI
SOGA11	8.31E-29	0.284986	0.051	0.022	1.33E-24	Saliva_Fibr	SOGA1
PDLIM41	1.38E-28	0.536064	0.154	0.104	2.19E-24	Saliva_Fibr	PDLIM4
LINC00657	1.98E-28	0.703345	0.247	0.202	3.16E-24	Saliva_Fibr	LINC00657
NTN4	6.47E-28	0.427028	0.105	0.062	1.03E-23	Saliva_Fibr	NTN4
RFNG	6.6E-28	0.408385	0.082	0.044	1.05E-23	Saliva_Fibr	RFNG
LSAMP	2.83E-27	0.272517	0.045	0.019	4.52E-23	Saliva_Fibr	LSAMP
CNTLN1	2.94E-27	0.375797	0.07	0.036	4.68E-23	Saliva_Fibr	CNTLN
ECHDC2	5.14E-27	0.691248	0.183	0.136	8.19E-23	Saliva_Fibr	ECHDC2
C1RL	6.4E-27	0.400757	0.074	0.039	1.02E-22	Saliva_Fibr	C1RL
IFNGR1	7.24E-27	0.605461	0.183	0.133	1.15E-22	Saliva_Fibr	IFNGR1
PPIG2	8.95E-27	0.651771	0.289	0.249	1.43E-22	Saliva_Fibr	PPIG
AIG11	9.08E-27	0.604559	0.155	0.108	1.45E-22	Saliva_Fibr	AIG1
SON1	1.46E-26	0.527157	0.426	0.41	2.34E-22	Saliva_Fibr	SON
PARVA	2.3E-26	0.505385	0.099	0.059	3.68E-22	Saliva_Fibr	PARVA
MATN21	2.72E-26	0.401356	0.077	0.041	4.34E-22	Saliva_Fibr	MATN2
SIVA11	3E-26	0.537052	0.314	0.273	4.78E-22	Saliva_Fibr	SIVA1
PRDX22	4.31E-26	0.476119	0.35	0.311	6.88E-22	Saliva_Fibr	PRDX2
ARHGEF10	5.02E-26	0.447227	0.091	0.052	8.01E-22	Saliva_Fibr	ARHGEF10
ANKH	5.26E-26	0.335532	0.068	0.035	8.39E-22	Saliva_Fibr	ANKH
EIF2AK22	5.7E-26	0.531451	0.14	0.094	9.08E-22	Saliva_Fibr	EIF2AK2
NKTR2	5.98E-26	0.59177	0.223	0.174	9.54E-22	Saliva_Fibr	NKTR
EZH11	6.45E-26	0.363117	0.076	0.041	1.03E-21	Saliva_Fibr	EZH1
NREP	6.84E-26	0.387879	0.074	0.039	1.09E-21	Saliva_Fibr	NREP
NTAN11	7.71E-26	0.574159	0.17	0.122	1.23E-21	Saliva_Fibr	NTAN1
ZNF385A	8.39E-26	0.360199	0.07	0.036	1.34E-21	Saliva_Fibr	ZNF385A
ARL3	1.23E-25	0.587037	0.16	0.114	1.97E-21	Saliva_Fibr	ARL3
IRAK31	1.33E-25	0.279392	0.054	0.025	2.13E-21	Saliva_Fibr	IRAK3
KLHL42	1.4E-25	0.349503	0.069	0.036	2.24E-21	Saliva_Fibr	KLHL42
MLH31	1.56E-25	0.449751	0.082	0.046	2.49E-21	Saliva_Fibr	MLH3
MTR	2.54E-25	0.366168	0.072	0.038	4.05E-21	Saliva_Fibr	MTR
EXT1	2.61E-25	0.357114	0.068	0.035	4.16E-21	Saliva_Fibr	EXT1
ATXN7L3B	3.4E-25	0.501741	0.114	0.072	5.42E-21	Saliva_Fibr	ATXN7L3B
TRIM561	4.09E-25	0.507962	0.119	0.076	6.52E-21	Saliva_Fibr	TRIM56
PDS5B	5.52E-25	0.50972	0.126	0.082	8.8E-21	Saliva_Fibr	PDS5B
SLC39A11	7.58E-25	0.568083	0.153	0.107	1.21E-20	Saliva_Fibr	SLC39A1
SERINC11	9.02E-25	0.65563	0.238	0.196	1.44E-20	Saliva_Fibr	SERINC1
NOTCH3	9.9E-25	0.345896	0.069	0.036	1.58E-20	Saliva_Fibr	NOTCH3
HNRNPR1	1.42E-24	0.600516	0.236	0.191	2.26E-20	Saliva_Fibr	HNRNPR

KAZALD1	1.61E-24	0.376433	0.06	0.03	2.57E-20	Saliva_Fibr	KAZALD1
CA5B1	1.63E-24	0.310157	0.064	0.033	2.6E-20	Saliva_Fibr	CA5B
PDCD51	1.99E-24	0.510105	0.284	0.239	3.18E-20	Saliva_Fibr	PDCD5
COL16A1	2.09E-24	0.34602	0.063	0.032	3.34E-20	Saliva_Fibr	COL16A1
ZNF708	5.22E-24	0.263109	0.044	0.02	8.32E-20	Saliva_Fibr	ZNF708
FAM184A	5.9E-24	0.321044	0.047	0.021	9.41E-20	Saliva_Fibr	FAM184A
PER3	8.05E-24	0.484636	0.105	0.066	1.28E-19	Saliva_Fibr	PER3
TNFRSF1A1	1.08E-23	0.588877	0.205	0.159	1.73E-19	Saliva_Fibr	TNFRSF1A
SLC43A31	1.17E-23	0.279336	0.063	0.032	1.87E-19	Saliva_Fibr	SLC43A3
PLD3	1.37E-23	0.563479	0.21	0.164	2.18E-19	Saliva_Fibr	PLD3
UGDH	3.03E-23	0.422926	0.113	0.072	4.83E-19	Saliva_Fibr	UGDH
DKK31	4.24E-23	0.423952	0.076	0.043	6.76E-19	Saliva_Fibr	DKK3
NDUFB102	4.71E-23	0.438392	0.403	0.385	7.51E-19	Saliva_Fibr	NDUFB10
NCOR2	5.75E-23	0.544996	0.143	0.1	9.18E-19	Saliva_Fibr	NCOR2
RARRES11	1.41E-22	0.42548	0.072	0.04	2.25E-18	Saliva_Fibr	RARRES1
RDH14	1.69E-22	0.373018	0.092	0.056	2.69E-18	Saliva_Fibr	RDH14
DHRS3	2.57E-22	0.656958	0.191	0.148	4.1E-18	Saliva_Fibr	DHRS3
SKI1	2.72E-22	0.421161	0.096	0.059	4.34E-18	Saliva_Fibr	SKI
PBX11	4.65E-22	0.585667	0.155	0.113	7.42E-18	Saliva_Fibr	PBX1
TNKS1BP1	7.48E-22	0.442648	0.101	0.063	1.19E-17	Saliva_Fibr	TNKS1BP1
S1PR2	1.19E-21	0.293866	0.047	0.022	1.89E-17	Saliva_Fibr	S1PR2
DESI2	1.56E-21	0.572652	0.14	0.1	2.49E-17	Saliva_Fibr	DESI2
SMARCA1	1.82E-21	0.442318	0.093	0.057	2.9E-17	Saliva_Fibr	SMARCA1
ARAP1	2.28E-21	0.30978	0.07	0.039	3.63E-17	Saliva_Fibr	ARAP1
MTCH1	2.33E-21	0.633191	0.273	0.242	3.71E-17	Saliva_Fibr	MTCH1
FAM126A1	3.69E-21	0.397665	0.097	0.06	5.89E-17	Saliva_Fibr	FAM126A
CKS1B	4.13E-21	0.557347	0.18	0.138	6.59E-17	Saliva_Fibr	CKS1B
INTU	4.14E-21	0.277121	0.049	0.024	6.6E-17	Saliva_Fibr	INTU
MPG1	5.49E-21	0.570282	0.212	0.172	8.75E-17	Saliva_Fibr	MPG
RUFY31	6.19E-21	0.49755	0.111	0.074	9.87E-17	Saliva_Fibr	RUFY3
C1orf123	8.47E-21	0.481466	0.16	0.117	1.35E-16	Saliva_Fibr	C1orf123
NQO2	1.45E-20	0.589956	0.151	0.111	2.31E-16	Saliva_Fibr	NQO2
QKI1	1.6E-20	0.602075	0.211	0.171	2.56E-16	Saliva_Fibr	QKI
CMYA51	1.83E-20	0.392254	0.062	0.033	2.92E-16	Saliva_Fibr	CMYA5
ZMIZ11	2.08E-20	0.476642	0.116	0.078	3.32E-16	Saliva_Fibr	ZMIZ1
GCSH1	2.11E-20	0.633408	0.164	0.125	3.36E-16	Saliva_Fibr	GCSH
ABCA1	2.98E-20	0.40609	0.102	0.065	4.76E-16	Saliva_Fibr	ABCA1
EGR3	3.16E-20	0.340918	0.061	0.033	5.03E-16	Saliva_Fibr	EGR3
NRXN2	3.36E-20	0.261188	0.036	0.016	5.37E-16	Saliva_Fibr	NRXN2
PCM1	3.68E-20	0.567678	0.198	0.156	5.87E-16	Saliva_Fibr	PCM1
CSGALNAC	3.78E-20	0.333243	0.074	0.043	6.03E-16	Saliva_Fibr	CSGALNACT1
ATXN31	3.82E-20	0.513638	0.119	0.081	6.09E-16	Saliva_Fibr	ATXN3
TRIP113	4.78E-20	0.621137	0.158	0.12	7.63E-16	Saliva_Fibr	TRIP11
SELENBP12	4.85E-20	0.491707	0.111	0.074	7.74E-16	Saliva_Fibr	SELENBP1
SERPINE1	5.67E-20	0.273946	0.04	0.018	9.04E-16	Saliva_Fibr	SERPINE1
ZYG11B	6.41E-20	0.424255	0.082	0.049	1.02E-15	Saliva_Fibr	ZYG11B
CBX5	7.5E-20	0.564602	0.151	0.112	1.2E-15	Saliva_Fibr	CBX5

LIMS11	8.11E-20	0.571734	0.207	0.167	1.29E-15	Saliva_Fibr	LIMS1
SH3D19	8.65E-20	0.480182	0.138	0.099	1.38E-15	Saliva_Fibr	SH3D19
U2SURP2	9.84E-20	0.58661	0.21	0.171	1.57E-15	Saliva_Fibr	U2SURP
CAMK1D	1E-19	0.305445	0.071	0.04	1.59E-15	Saliva_Fibr	CAMK1D
SETD7	1.06E-19	0.42044	0.094	0.06	1.7E-15	Saliva_Fibr	SETD7
KHDRBS3	1.2E-19	0.476976	0.109	0.072	1.92E-15	Saliva_Fibr	KHDRBS3
TRIM221	1.51E-19	0.308902	0.085	0.051	2.41E-15	Saliva_Fibr	TRIM22
PPIL4	1.55E-19	0.520775	0.138	0.099	2.47E-15	Saliva_Fibr	PPIL4
CLEC11A	1.57E-19	0.410637	0.117	0.079	2.51E-15	Saliva_Fibr	CLEC11A
ARHGEF40	1.58E-19	0.357755	0.065	0.037	2.53E-15	Saliva_Fibr	ARHGEF40
ST3GAL5	1.58E-19	0.269845	0.053	0.028	2.53E-15	Saliva_Fibr	ST3GAL5
MYO9A1	2.58E-19	0.489228	0.109	0.073	4.11E-15	Saliva_Fibr	MYO9A
PPP1R14B3	3.01E-19	0.648353	0.212	0.176	4.81E-15	Saliva_Fibr	PPP1R14B
RORA	3.41E-19	0.497766	0.161	0.12	5.44E-15	Saliva_Fibr	RORA
XAF11	4.06E-19	0.280362	0.073	0.042	6.47E-15	Saliva_Fibr	XAF1
TPR1	4.69E-19	0.520775	0.241	0.202	7.48E-15	Saliva_Fibr	TPR
IRX31	5.12E-19	0.522017	0.106	0.072	8.17E-15	Saliva_Fibr	IRX3
ZBTB7A	5.95E-19	0.534172	0.161	0.122	9.49E-15	Saliva_Fibr	ZBTB7A
SERPINH11	5.98E-19	0.421433	0.156	0.114	9.54E-15	Saliva_Fibr	SERPINH1
WIPF1	6.61E-19	0.378621	0.132	0.091	1.05E-14	Saliva_Fibr	WIPF1
APLP21	6.92E-19	0.544148	0.326	0.304	1.1E-14	Saliva_Fibr	APLP2
PVRL3	8.51E-19	0.36114	0.079	0.048	1.36E-14	Saliva_Fibr	PVRL3
MYO1C1	9.96E-19	0.578864	0.182	0.145	1.59E-14	Saliva_Fibr	MYO1C
VGLL41	1.02E-18	0.619141	0.171	0.135	1.62E-14	Saliva_Fibr	VGLL4
H1FX1	1.35E-18	0.478281	0.296	0.26	2.16E-14	Saliva_Fibr	H1FX
ARMCX1	1.76E-18	0.384266	0.088	0.055	2.81E-14	Saliva_Fibr	ARMCX1
BCLAF12	1.8E-18	0.611568	0.231	0.199	2.87E-14	Saliva_Fibr	BCLAF1
CYTH3	1.86E-18	0.297557	0.053	0.028	2.96E-14	Saliva_Fibr	CYTH3
UTRN1	2.86E-18	0.453001	0.155	0.115	4.56E-14	Saliva_Fibr	UTRN
MRPL202	2.9E-18	0.521648	0.294	0.27	4.63E-14	Saliva_Fibr	MRPL20
HP1BP3	2.93E-18	0.625899	0.253	0.224	4.67E-14	Saliva_Fibr	HP1BP3
RBM252	3.88E-18	0.603418	0.268	0.239	6.18E-14	Saliva_Fibr	RBM25
UBXN41	4E-18	0.577252	0.317	0.3	6.38E-14	Saliva_Fibr	UBXN4
THRA	4.62E-18	0.327986	0.068	0.04	7.37E-14	Saliva_Fibr	THRA
HEG11	5.03E-18	0.330502	0.119	0.081	8.02E-14	Saliva_Fibr	HEG1
FOXO11	5.66E-18	0.353107	0.077	0.046	9.03E-14	Saliva_Fibr	FOXO1
GSTA4	5.77E-18	0.327145	0.07	0.041	9.2E-14	Saliva_Fibr	GSTA4
CBLB	5.95E-18	0.381333	0.086	0.054	9.48E-14	Saliva_Fibr	CBLB
VPS13C1	6.73E-18	0.494691	0.129	0.092	1.07E-13	Saliva_Fibr	VPS13C
NPHP31	8.6E-18	0.340922	0.074	0.045	1.37E-13	Saliva_Fibr	NPHP3
MPHOSPH8	9.28E-18	0.519318	0.283	0.253	1.48E-13	Saliva_Fibr	MPHOSPH8
ATRX2	9.48E-18	0.612222	0.238	0.206	1.51E-13	Saliva_Fibr	ATRX
ZHX3	9.96E-18	0.292724	0.052	0.028	1.59E-13	Saliva_Fibr	ZHX3
DEK1	1.1E-17	0.519209	0.286	0.258	1.76E-13	Saliva_Fibr	DEK
SEMA5A	1.22E-17	0.310944	0.049	0.025	1.94E-13	Saliva_Fibr	SEMA5A
RHBDF1	1.22E-17	0.370966	0.075	0.046	1.94E-13	Saliva_Fibr	RHBDF1
ATP6AP2	1.45E-17	0.562197	0.268	0.238	2.32E-13	Saliva_Fibr	ATP6AP2

MSRB2	1.73E-17	0.561986	0.164	0.129	2.77E-13	Saliva_Fibr	MSRB2
SOCS31	1.94E-17	0.303543	0.21	0.163	3.09E-13	Saliva_Fibr	SOCS3
RAB11FIP2	2.01E-17	0.321652	0.071	0.043	3.2E-13	Saliva_Fibr	RAB11FIP2
HIVEP1	2.25E-17	0.292936	0.067	0.039	3.59E-13	Saliva_Fibr	HIVEP1
EPB41L31	2.46E-17	0.358326	0.089	0.057	3.92E-13	Saliva_Fibr	EPB41L3
CUEDC1	2.52E-17	0.309087	0.055	0.03	4.02E-13	Saliva_Fibr	CUEDC1
CCND2	2.6E-17	0.44913	0.138	0.101	4.14E-13	Saliva_Fibr	CCND2
ROBO1	2.82E-17	0.27998	0.053	0.029	4.5E-13	Saliva_Fibr	ROBO1
TLN2	3.36E-17	0.270354	0.044	0.023	5.35E-13	Saliva_Fibr	TLN2
GNB41	3.68E-17	0.341709	0.066	0.039	5.87E-13	Saliva_Fibr	GNB4
STOM1	4.3E-17	0.441647	0.192	0.152	6.85E-13	Saliva_Fibr	STOM
RBM12B	4.3E-17	0.325616	0.056	0.031	6.86E-13	Saliva_Fibr	RBM12B
NHSL21	4.37E-17	0.30097	0.047	0.025	6.97E-13	Saliva_Fibr	NHSL2
ST6GALNA	4.76E-17	0.384475	0.104	0.07	7.59E-13	Saliva_Fibr	ST6GALNAC6
SEC23A	5.26E-17	0.440064	0.102	0.069	8.4E-13	Saliva_Fibr	SEC23A
ZDHHC171	5.33E-17	0.289325	0.06	0.034	8.51E-13	Saliva_Fibr	ZDHHC17
CXXC51	5.61E-17	0.557282	0.189	0.152	8.96E-13	Saliva_Fibr	CXXC5
NDUFS4	8.08E-17	0.597148	0.244	0.217	1.29E-12	Saliva_Fibr	NDUFS4
RBM281	8.23E-17	0.339961	0.071	0.043	1.31E-12	Saliva_Fibr	RBM28
AHI1	9.62E-17	0.53232	0.147	0.111	1.53E-12	Saliva_Fibr	AHI1
CCDC66	1.26E-16	0.498035	0.126	0.091	2E-12	Saliva_Fibr	CCDC66
SPTAN11	1.42E-16	0.584325	0.203	0.17	2.27E-12	Saliva_Fibr	SPTAN1
DNAJB141	1.42E-16	0.506819	0.137	0.102	2.27E-12	Saliva_Fibr	DNAJB14
ZNF7061	1.51E-16	0.486663	0.354	0.345	2.41E-12	Saliva_Fibr	ZNF706
CYP4V2	2.13E-16	0.26964	0.046	0.024	3.4E-12	Saliva_Fibr	CYP4V2
ACVR1	3.1E-16	0.251095	0.055	0.031	4.94E-12	Saliva_Fibr	ACVR1
GUCY1B3	3.19E-16	0.272395	0.041	0.021	5.09E-12	Saliva_Fibr	GUCY1B3
MACF11	3.21E-16	0.595165	0.199	0.167	5.12E-12	Saliva_Fibr	MACF1
VIMP2	3.28E-16	0.45206	0.313	0.292	5.23E-12	Saliva_Fibr	VIMP
SPRYD3	3.94E-16	0.332497	0.072	0.044	6.28E-12	Saliva_Fibr	SPRYD3
ACOT91	4.47E-16	0.377252	0.09	0.059	7.14E-12	Saliva_Fibr	ACOT9
TBC1D12	4.8E-16	0.346786	0.072	0.045	7.66E-12	Saliva_Fibr	TBC1D12
EML1	5.5E-16	0.344445	0.071	0.044	8.78E-12	Saliva_Fibr	EML1
CYP20A12	5.58E-16	0.513136	0.129	0.095	8.91E-12	Saliva_Fibr	CYP20A1
C4orf271	5.9E-16	0.42399	0.11	0.077	9.4E-12	Saliva_Fibr	C4orf27
DHX36	6.39E-16	0.543342	0.213	0.181	1.02E-11	Saliva_Fibr	DHX36
CTDSP11	7.43E-16	0.427993	0.135	0.099	1.18E-11	Saliva_Fibr	CTDSP1
PLBD1	7.91E-16	0.478632	0.116	0.084	1.26E-11	Saliva_Fibr	PLBD1
LRRC58	8.27E-16	0.435034	0.104	0.072	1.32E-11	Saliva_Fibr	LRRC58
ABI2	8.63E-16	0.485082	0.12	0.087	1.38E-11	Saliva_Fibr	ABI2
TET1	1.01E-15	0.284701	0.05	0.027	1.61E-11	Saliva_Fibr	TET1
EIF3J.AS11	1.17E-15	0.398196	0.087	0.057	1.86E-11	Saliva_Fibr	EIF3J.AS1
NCOA1	1.36E-15	0.514903	0.12	0.088	2.18E-11	Saliva_Fibr	NCOA1
FMNL2	1.6E-15	0.327635	0.065	0.039	2.56E-11	Saliva_Fibr	FMNL2
RSRC1	1.67E-15	0.490062	0.124	0.091	2.66E-11	Saliva_Fibr	RSRC1
KRR11	1.71E-15	0.570652	0.164	0.132	2.73E-11	Saliva_Fibr	KRR1
GPATCH2L	1.72E-15	0.507733	0.113	0.081	2.74E-11	Saliva_Fibr	GPATCH2L

RARG	1.73E-15	0.375471	0.092	0.062	2.75E-11	Saliva_Fibr	RARG
GALNT11	2.16E-15	0.384961	0.086	0.057	3.44E-11	Saliva_Fibr	GALNT11
RNF131	2.98E-15	0.596899	0.196	0.166	4.75E-11	Saliva_Fibr	RNF13
ZNF224	2.99E-15	0.254925	0.05	0.028	4.77E-11	Saliva_Fibr	ZNF224
MIA31	3.69E-15	0.466146	0.146	0.111	5.88E-11	Saliva_Fibr	MIA3
RAD501	3.92E-15	0.49549	0.119	0.087	6.26E-11	Saliva_Fibr	RAD50
ERC11	4.11E-15	0.403038	0.08	0.052	6.55E-11	Saliva_Fibr	ERC1
SH3PXD2B	4.87E-15	0.324786	0.066	0.041	7.77E-11	Saliva_Fibr	SH3PXD2B
RBFOX2	5.2E-15	0.516535	0.138	0.106	8.3E-11	Saliva_Fibr	RBFOX2
TCF12	8.76E-15	0.479493	0.102	0.072	1.4E-10	Saliva_Fibr	TCF12
PLEKHH2	8.81E-15	0.302864	0.063	0.038	1.4E-10	Saliva_Fibr	PLEKHH2
IQSEC1	8.86E-15	0.331682	0.079	0.051	1.41E-10	Saliva_Fibr	IQSEC1
MSRB31	9.11E-15	0.250181	0.058	0.034	1.45E-10	Saliva_Fibr	MSRB3
MVP1	1.1E-14	0.395244	0.124	0.09	1.75E-10	Saliva_Fibr	MVP
SACS	1.23E-14	0.29674	0.056	0.032	1.97E-10	Saliva_Fibr	SACS
PHPT12	1.97E-14	0.434941	0.338	0.325	3.14E-10	Saliva_Fibr	PHPT1
SNX6	2.14E-14	0.557034	0.202	0.173	3.41E-10	Saliva_Fibr	SNX6
ABHD14A1	2.47E-14	0.465764	0.134	0.102	3.94E-10	Saliva_Fibr	ABHD14A
ATL31	2.59E-14	0.494111	0.151	0.119	4.13E-10	Saliva_Fibr	ATL3
PRNP	2.63E-14	0.451809	0.194	0.159	4.19E-10	Saliva_Fibr	PRNP
PARVB2	2.68E-14	0.25537	0.068	0.042	4.27E-10	Saliva_Fibr	PARVB
TMEM14C:	2.81E-14	0.579074	0.271	0.255	4.48E-10	Saliva_Fibr	TMEM14C
RUFY2	2.82E-14	0.318439	0.065	0.041	4.49E-10	Saliva_Fibr	RUFY2
NIFK1	2.83E-14	0.426192	0.128	0.096	4.52E-10	Saliva_Fibr	NIFK
AIDA1	2.9E-14	0.508219	0.134	0.103	4.62E-10	Saliva_Fibr	AIDA
ZNF7912	2.94E-14	0.404263	0.099	0.069	4.69E-10	Saliva_Fibr	ZNF791
CDK41	3.1E-14	0.488576	0.144	0.112	4.95E-10	Saliva_Fibr	CDK4
ZNF3021	3.16E-14	0.435956	0.108	0.077	5.04E-10	Saliva_Fibr	ZNF302
VEZF1	3.57E-14	0.473727	0.117	0.087	5.7E-10	Saliva_Fibr	VEZF1
ITGB1	5.47E-14	0.413244	0.366	0.357	8.73E-10	Saliva_Fibr	ITGB1
RBMS1	6.78E-14	0.49429	0.241	0.214	1.08E-09	Saliva_Fibr	RBMS1
SRSF102	7.53E-14	0.560219	0.185	0.156	1.2E-09	Saliva_Fibr	SRSF10
SRFBP11	7.68E-14	0.422059	0.089	0.061	1.22E-09	Saliva_Fibr	SRFBP1
MIER11	8.26E-14	0.518195	0.153	0.123	1.32E-09	Saliva_Fibr	MIER1
IRX5	8.94E-14	0.272469	0.048	0.027	1.43E-09	Saliva_Fibr	IRX5
ERAP21	1.06E-13	0.285245	0.049	0.028	1.69E-09	Saliva_Fibr	ERAP2
SPOP1	1.12E-13	0.507553	0.144	0.113	1.79E-09	Saliva_Fibr	SPOP
CCDC501	1.27E-13	0.539477	0.166	0.137	2.02E-09	Saliva_Fibr	CCDC50
RFC12	1.49E-13	0.550652	0.144	0.114	2.37E-09	Saliva_Fibr	RFC1
BMPR1A	1.55E-13	0.3046	0.055	0.033	2.47E-09	Saliva_Fibr	BMPR1A
HLF	1.61E-13	0.308427	0.052	0.03	2.57E-09	Saliva_Fibr	HLF
EIF1B	1.78E-13	0.461971	0.273	0.25	2.83E-09	Saliva_Fibr	EIF1B
ANXA71	2.11E-13	0.508348	0.221	0.195	3.36E-09	Saliva_Fibr	ANXA7
ZFP141	2.19E-13	0.267248	0.053	0.031	3.5E-09	Saliva_Fibr	ZFP14
YIF1A1	2.5E-13	0.596603	0.198	0.172	3.98E-09	Saliva_Fibr	YIF1A
C16orf521	2.89E-13	0.354805	0.071	0.047	4.62E-09	Saliva_Fibr	C16orf52
ZC3H131	2.9E-13	0.484006	0.152	0.121	4.62E-09	Saliva_Fibr	ZC3H13

APC1	2.92E-13	0.38298	0.087	0.06	4.66E-09	Saliva_Fibr	APC
AKAP111	3.89E-13	0.365911	0.081	0.055	6.21E-09	Saliva_Fibr	AKAP11
TRIM442	5.38E-13	0.480787	0.118	0.089	8.58E-09	Saliva_Fibr	TRIM44
EPC1	5.43E-13	0.431769	0.181	0.148	8.66E-09	Saliva_Fibr	EPC1
RHOC1	6.24E-13	0.417196	0.252	0.224	9.95E-09	Saliva_Fibr	RHOC
SKP1	6.27E-13	0.276315	0.546	0.572	1E-08	Saliva_Fibr	SKP1
ASAP2	6.59E-13	0.404876	0.108	0.079	1.05E-08	Saliva_Fibr	ASAP2
PTPN13	6.97E-13	0.379472	0.083	0.056	1.11E-08	Saliva_Fibr	PTPN13
MPHOSPH	7.24E-13	0.429863	0.118	0.089	1.16E-08	Saliva_Fibr	MPHOSPH10
RP11.553L	7.37E-13	0.331785	0.077	0.051	1.18E-08	Saliva_Fibr	RP11.553L6.5
PSMA72	9.18E-13	0.27434	0.453	0.465	1.46E-08	Saliva_Fibr	PSMA7
MIR22HG	9.54E-13	0.399139	0.114	0.085	1.52E-08	Saliva_Fibr	MIR22HG
SPRED1	1.14E-12	0.337057	0.066	0.042	1.81E-08	Saliva_Fibr	SPRED1
NT5C	1.37E-12	0.4181	0.156	0.124	2.18E-08	Saliva_Fibr	NT5C
ARID5B	1.39E-12	0.253901	0.3	0.268	2.21E-08	Saliva_Fibr	ARID5B
CHM1	1.42E-12	0.315927	0.065	0.042	2.27E-08	Saliva_Fibr	CHM
NDUFB72	1.44E-12	0.392574	0.37	0.374	2.3E-08	Saliva_Fibr	NDUFB7
PSD3	1.87E-12	0.43476	0.113	0.084	2.99E-08	Saliva_Fibr	PSD3
ADI12	2.38E-12	0.572957	0.238	0.221	3.8E-08	Saliva_Fibr	ADI1
C8orf593	2.62E-12	0.58385	0.249	0.235	4.18E-08	Saliva_Fibr	C8orf59
PEAK11	2.83E-12	0.295835	0.062	0.039	4.52E-08	Saliva_Fibr	PEAK1
PLEC1	3.66E-12	0.512751	0.183	0.155	5.83E-08	Saliva_Fibr	PLEC
ATP5G11	3.79E-12	0.46715	0.278	0.26	6.04E-08	Saliva_Fibr	ATP5G1
SHPRH1	4.6E-12	0.429405	0.096	0.069	7.34E-08	Saliva_Fibr	SHPRH
RYK1	5.52E-12	0.433779	0.101	0.074	8.81E-08	Saliva_Fibr	RYK
TMX3	6.18E-12	0.348174	0.074	0.05	9.86E-08	Saliva_Fibr	TMX3
UBL4A	6.31E-12	0.32417	0.073	0.049	1.01E-07	Saliva_Fibr	UBL4A
ATP5G31	7.09E-12	0.346285	0.366	0.362	1.13E-07	Saliva_Fibr	ATP5G3
PBDC1	7.68E-12	0.431993	0.115	0.087	1.22E-07	Saliva_Fibr	PBDC1
ZNF7041	8.4E-12	0.422793	0.094	0.069	1.34E-07	Saliva_Fibr	ZNF704
IGSF81	8.5E-12	0.356145	0.082	0.056	1.35E-07	Saliva_Fibr	IGSF8
GNPDA2	8.72E-12	0.305738	0.062	0.04	1.39E-07	Saliva_Fibr	GNPDA2
TERF1	8.87E-12	0.426593	0.134	0.106	1.42E-07	Saliva_Fibr	TERF1
ZNHIT61	9.62E-12	0.267459	0.056	0.035	1.54E-07	Saliva_Fibr	ZNHIT6
RPAP21	1.1E-11	0.387138	0.074	0.05	1.76E-07	Saliva_Fibr	RPAP2
TSPAN311	1.22E-11	0.425815	0.097	0.071	1.95E-07	Saliva_Fibr	TSPAN31
NOC3L1	1.35E-11	0.355866	0.071	0.048	2.15E-07	Saliva_Fibr	NOC3L
C5orf45	1.82E-11	0.316623	0.064	0.042	2.9E-07	Saliva_Fibr	C5orf45
RRBP12	1.87E-11	0.476913	0.315	0.312	2.98E-07	Saliva_Fibr	RRBP1
PLA2G162	1.88E-11	0.411697	0.153	0.125	2.99E-07	Saliva_Fibr	PLA2G16
TXNDC151	1.88E-11	0.444949	0.15	0.122	3.01E-07	Saliva_Fibr	TXNDC15
SRSF11	2.67E-11	0.470395	0.309	0.303	4.25E-07	Saliva_Fibr	SRSF11
SLC35E2B	3.25E-11	0.347397	0.068	0.046	5.19E-07	Saliva_Fibr	SLC35E2B
AGO3	3.29E-11	0.424522	0.102	0.076	5.25E-07	Saliva_Fibr	AGO3
ZADH2	3.6E-11	0.377137	0.075	0.052	5.74E-07	Saliva_Fibr	ZADH2
TMX4	4.15E-11	0.366962	0.189	0.159	6.62E-07	Saliva_Fibr	TMX4
FKBP141	4.34E-11	0.306353	0.065	0.043	6.92E-07	Saliva_Fibr	FKBP14

SORBS3	4.78E-11	0.437381	0.132	0.105	7.62E-07	Saliva_Fibr	SORBS3
TMTC3	4.92E-11	0.314682	0.057	0.037	7.84E-07	Saliva_Fibr	TMTC3
LPCAT21	6.6E-11	0.308742	0.059	0.038	1.05E-06	Saliva_Fibr	LPCAT2
TUBB6	8.19E-11	0.417897	0.216	0.19	1.31E-06	Saliva_Fibr	TUBB6
DICER11	9.21E-11	0.508046	0.128	0.103	1.47E-06	Saliva_Fibr	DICER1
SLC39A101	9.4E-11	0.348847	0.08	0.057	1.5E-06	Saliva_Fibr	SLC39A10
EXT2	9.64E-11	0.374464	0.076	0.054	1.54E-06	Saliva_Fibr	EXT2
CAT	9.78E-11	0.446376	0.141	0.114	1.56E-06	Saliva_Fibr	CAT
FAIM1	9.87E-11	0.315153	0.064	0.043	1.57E-06	Saliva_Fibr	FAIM
CLIC42	1.04E-10	0.476685	0.151	0.125	1.65E-06	Saliva_Fibr	CLIC4
WHSC1L11	1.07E-10	0.515452	0.215	0.194	1.71E-06	Saliva_Fibr	WHSC1L1
GALK11	1.1E-10	0.41568	0.088	0.064	1.76E-06	Saliva_Fibr	GALK1
PTGR1	1.12E-10	0.515963	0.128	0.103	1.78E-06	Saliva_Fibr	PTGR1
WDR83OS	1.24E-10	0.373923	0.362	0.369	1.98E-06	Saliva_Fibr	WDR83OS
ENDOD11	1.31E-10	0.258778	0.052	0.033	2.09E-06	Saliva_Fibr	ENDOD1
CRYBG32	1.44E-10	0.418936	0.086	0.062	2.3E-06	Saliva_Fibr	CRYBG3
DCTN31	1.5E-10	0.489177	0.25	0.234	2.39E-06	Saliva_Fibr	DCTN3
FNDC3A2	1.55E-10	0.505078	0.14	0.116	2.47E-06	Saliva_Fibr	FNDC3A
C5orf42	1.6E-10	0.353279	0.062	0.042	2.55E-06	Saliva_Fibr	C5orf42
SMURF2	1.85E-10	0.331936	0.08	0.057	2.96E-06	Saliva_Fibr	SMURF2
VPS13B	1.89E-10	0.263332	0.049	0.03	3.02E-06	Saliva_Fibr	VPS13B
ARGLU11	2.16E-10	0.514046	0.311	0.309	3.45E-06	Saliva_Fibr	ARGLU1
CWC22	2.17E-10	0.362818	0.079	0.056	3.46E-06	Saliva_Fibr	CWC22
PARP41	2.33E-10	0.286949	0.062	0.041	3.71E-06	Saliva_Fibr	PARP4
RHOA1	2.45E-10	0.376021	0.443	0.476	3.91E-06	Saliva_Fibr	RHOA
PHYKPL1	2.61E-10	0.338638	0.094	0.069	4.16E-06	Saliva_Fibr	PHYKPL
PDLIM31	2.82E-10	0.278988	0.081	0.057	4.5E-06	Saliva_Fibr	PDLIM3
TTC142	2.9E-10	0.449513	0.127	0.102	4.63E-06	Saliva_Fibr	TTC14
SNX27	3.13E-10	0.362426	0.076	0.054	4.99E-06	Saliva_Fibr	SNX27
IL1R11	3.15E-10	0.341058	0.1	0.075	5.02E-06	Saliva_Fibr	IL1R1
ENDOG	3.51E-10	0.380531	0.087	0.064	5.59E-06	Saliva_Fibr	ENDOG
CDC271	3.67E-10	0.379157	0.1	0.076	5.85E-06	Saliva_Fibr	CDC27
DDHD11	3.92E-10	0.329704	0.077	0.055	6.25E-06	Saliva_Fibr	DDHD1
PLOD32	4.24E-10	0.266992	0.059	0.039	6.76E-06	Saliva_Fibr	PLOD3
NRIP1	4.36E-10	0.368879	0.071	0.05	6.96E-06	Saliva_Fibr	NRIP1
ANAPC52	4.43E-10	0.556144	0.182	0.161	7.07E-06	Saliva_Fibr	ANAPC5
UBXN2A	4.48E-10	0.361769	0.083	0.06	7.14E-06	Saliva_Fibr	UBXN2A
PHF141	4.59E-10	0.481192	0.149	0.125	7.32E-06	Saliva_Fibr	PHF14
HEXDC	4.68E-10	0.30432	0.058	0.038	7.47E-06	Saliva_Fibr	HEXDC
CDC14B	5.07E-10	0.255492	0.047	0.03	8.09E-06	Saliva_Fibr	CDC14B
PCGF21	5.1E-10	0.291103	0.057	0.038	8.13E-06	Saliva_Fibr	PCGF2
ZSCAN181	5.29E-10	0.431825	0.104	0.08	8.43E-06	Saliva_Fibr	ZSCAN18
RRP362	5.43E-10	0.363474	0.08	0.057	8.66E-06	Saliva_Fibr	RRP36
ODF2L1	6.68E-10	0.48023	0.137	0.112	1.06E-05	Saliva_Fibr	ODF2L
RBX11	6.79E-10	0.399834	0.344	0.35	1.08E-05	Saliva_Fibr	RBX1
DPYD	7.57E-10	0.293667	0.062	0.041	1.21E-05	Saliva_Fibr	DPYD
ST5	7.91E-10	0.403964	0.082	0.06	1.26E-05	Saliva_Fibr	ST5

FNDC3B1	8.37E-10	0.486585	0.171	0.15	1.33E-05	Saliva_Fibr	FNDC3B
SSBP2	1.09E-09	0.394559	0.094	0.071	1.74E-05	Saliva_Fibr	SSBP2
ACO1	1.16E-09	0.315071	0.061	0.042	1.86E-05	Saliva_Fibr	ACO1
MESDC22	1.24E-09	0.526294	0.191	0.172	1.98E-05	Saliva_Fibr	MESDC2
THRAP32	1.26E-09	0.508605	0.195	0.175	2.01E-05	Saliva_Fibr	THRAP3
SMG6	1.3E-09	0.333957	0.068	0.047	2.07E-05	Saliva_Fibr	SMG6
STX21	1.33E-09	0.313961	0.065	0.044	2.12E-05	Saliva_Fibr	STX2
LATS1	1.41E-09	0.325436	0.062	0.042	2.25E-05	Saliva_Fibr	LATS1
N6AMT2	1.45E-09	0.298025	0.052	0.034	2.32E-05	Saliva_Fibr	N6AMT2
PNN2	1.51E-09	0.548279	0.226	0.212	2.41E-05	Saliva_Fibr	PNN
CD471	1.69E-09	0.479723	0.245	0.23	2.69E-05	Saliva_Fibr	CD47
SFT2D2	1.87E-09	0.494625	0.132	0.109	2.99E-05	Saliva_Fibr	SFT2D2
HYI1	1.88E-09	0.442071	0.109	0.086	2.99E-05	Saliva_Fibr	HYI
ITGAV	2.06E-09	0.454887	0.118	0.095	3.28E-05	Saliva_Fibr	ITGAV
GNA111	2.13E-09	0.408811	0.09	0.068	3.4E-05	Saliva_Fibr	GNA11
TXLNA1	2.37E-09	0.324916	0.074	0.054	3.79E-05	Saliva_Fibr	TXLNA
OCIAD12	2.4E-09	0.508481	0.26	0.253	3.83E-05	Saliva_Fibr	OCIAD1
SPAG9	2.55E-09	0.50094	0.21	0.193	4.06E-05	Saliva_Fibr	SPAG9
IKBIP2	2.57E-09	0.312636	0.082	0.06	4.11E-05	Saliva_Fibr	IKBIP
ROCK11	2.6E-09	0.417296	0.179	0.155	4.15E-05	Saliva_Fibr	ROCK1
GADD45GI	2.76E-09	0.407543	0.287	0.28	4.4E-05	Saliva_Fibr	GADD45GIP1
TMEM115	3E-09	0.39572	0.089	0.067	4.78E-05	Saliva_Fibr	TMEM115
POLK1	3.09E-09	0.381646	0.092	0.07	4.93E-05	Saliva_Fibr	POLK
TMEM9	3.64E-09	0.500528	0.122	0.1	5.81E-05	Saliva_Fibr	TMEM9
ZMYND11	3.7E-09	0.44085	0.102	0.08	5.91E-05	Saliva_Fibr	ZMYND11
CERS5	5.29E-09	0.266081	0.052	0.034	8.44E-05	Saliva_Fibr	CERS5
SEPT2	6.22E-09	0.558549	0.242	0.236	9.92E-05	Saliva_Fibr	SEPT2
SPCS21	6.65E-09	0.301901	0.387	0.408	0.000106	Saliva_Fibr	SPCS2
CYSTM11	6.68E-09	0.37323	0.245	0.226	0.000107	Saliva_Fibr	CYSTM1
EVI51	6.68E-09	0.425113	0.099	0.078	0.000107	Saliva_Fibr	EVI5
IGFBP21	6.8E-09	0.523157	0.073	0.053	0.000109	Saliva_Fibr	IGFBP2
ZNF280D1	7.29E-09	0.460747	0.105	0.084	0.000116	Saliva_Fibr	ZNF280D
CASC41	7.4E-09	0.495391	0.143	0.122	0.000118	Saliva_Fibr	CASC4
ASH1L	8.41E-09	0.547966	0.186	0.169	0.000134	Saliva_Fibr	ASH1L
CPNE3	9.07E-09	0.517079	0.207	0.192	0.000145	Saliva_Fibr	CPNE3
WDR13	9.38E-09	0.453089	0.132	0.11	0.00015	Saliva_Fibr	WDR13
ERGIC12	1.02E-08	0.500853	0.153	0.133	0.000162	Saliva_Fibr	ERGIC1
ZNF106	1.09E-08	0.557246	0.157	0.138	0.000174	Saliva_Fibr	ZNF106
SCAF111	1.34E-08	0.525152	0.24	0.232	0.000214	Saliva_Fibr	SCAF11
OSBPL1A1	1.51E-08	0.524305	0.177	0.16	0.000241	Saliva_Fibr	OSBPL1A
SLC35F6	1.8E-08	0.335054	0.072	0.052	0.000287	Saliva_Fibr	SLC35F6
VPS361	1.86E-08	0.517748	0.133	0.113	0.000296	Saliva_Fibr	VPS36
SNRPD3	1.97E-08	0.492367	0.24	0.232	0.000314	Saliva_Fibr	SNRPD3
CTSA	2E-08	0.389863	0.154	0.132	0.000319	Saliva_Fibr	CTSA
STAG2	2.01E-08	0.450986	0.132	0.111	0.00032	Saliva_Fibr	STAG2
SUPT16H	2.08E-08	0.42631	0.13	0.108	0.000331	Saliva_Fibr	SUPT16H
ATP6V0A1	2.12E-08	0.328265	0.076	0.056	0.000338	Saliva_Fibr	ATP6V0A1

SLC2A10	2.16E-08	0.294385	0.053	0.036	0.000344	Saliva_Fibr	SLC2A10
AKR1B1	2.22E-08	0.260686	0.136	0.11	0.000354	Saliva_Fibr	AKR1B1
SNX9	2.26E-08	0.351536	0.254	0.238	0.000361	Saliva_Fibr	SNX9
PIK3CA1	2.39E-08	0.255475	0.058	0.04	0.000382	Saliva_Fibr	PIK3CA
CDC40	2.41E-08	0.434796	0.123	0.102	0.000384	Saliva_Fibr	CDC40
GTF2I3	2.43E-08	0.507933	0.171	0.154	0.000387	Saliva_Fibr	GTF2I
FAF21	2.43E-08	0.382225	0.084	0.064	0.000388	Saliva_Fibr	FAF2
IFT52	2.57E-08	0.332906	0.081	0.061	0.000409	Saliva_Fibr	IFT52
GOSR1	2.85E-08	0.463486	0.119	0.099	0.000455	Saliva_Fibr	GOSR1
KLF41	3.16E-08	0.391363	0.243	0.226	0.000504	Saliva_Fibr	KLF4
SNX7	3.47E-08	0.351039	0.086	0.066	0.000554	Saliva_Fibr	SNX7
LRRC16A	3.61E-08	0.280651	0.058	0.04	0.000575	Saliva_Fibr	LRRC16A
USP11	4E-08	0.387502	0.094	0.073	0.000637	Saliva_Fibr	USP11
DSE	4.26E-08	0.295491	0.069	0.05	0.000679	Saliva_Fibr	DSE
EPN2	4.82E-08	0.418924	0.089	0.069	0.000768	Saliva_Fibr	EPN2
DSTYK	4.87E-08	0.293743	0.058	0.041	0.000777	Saliva_Fibr	DSTYK
LZTS2	5.65E-08	0.288526	0.059	0.042	0.000902	Saliva_Fibr	LZTS2
AHCYL1	5.9E-08	0.558689	0.179	0.165	0.000942	Saliva_Fibr	AHCYL1
PMEPA11	6.16E-08	0.576321	0.139	0.119	0.000983	Saliva_Fibr	PMEPA1
CCDC1071	6.28E-08	0.403494	0.195	0.176	0.001002	Saliva_Fibr	CCDC107
PMM1	8.25E-08	0.363006	0.091	0.071	0.001316	Saliva_Fibr	PMM1
H2AFX	8.41E-08	0.340352	0.084	0.065	0.001341	Saliva_Fibr	H2AFX
UTP23	8.42E-08	0.326029	0.073	0.054	0.001342	Saliva_Fibr	UTP23
FAM63B	8.47E-08	0.442907	0.123	0.103	0.001351	Saliva_Fibr	FAM63B
SEC632	8.78E-08	0.494667	0.186	0.171	0.0014	Saliva_Fibr	SEC63
IAH1	9.44E-08	0.409463	0.18	0.16	0.001506	Saliva_Fibr	IAH1
TAOK1	1.01E-07	0.450655	0.13	0.11	0.001606	Saliva_Fibr	TAOK1
ARHGDIA1	1.07E-07	0.313643	0.217	0.196	0.001713	Saliva_Fibr	ARHGDIA
ZNF221	1.12E-07	0.444696	0.128	0.108	0.00178	Saliva_Fibr	ZNF22
C12orf76	1.15E-07	0.268959	0.065	0.047	0.001831	Saliva_Fibr	C12orf76
C16orf45	1.27E-07	0.31988	0.083	0.064	0.002028	Saliva_Fibr	C16orf45
SMARCA2	1.27E-07	0.480519	0.132	0.114	0.002033	Saliva_Fibr	SMARCA2
TOMM203	1.32E-07	0.375342	0.318	0.326	0.002103	Saliva_Fibr	TOMM20
TYW3	1.45E-07	0.413685	0.097	0.077	0.002305	Saliva_Fibr	TYW3
MBD3	1.46E-07	0.295621	0.06	0.043	0.002322	Saliva_Fibr	MBD3
TBC1D16	1.52E-07	0.307338	0.058	0.041	0.002419	Saliva_Fibr	TBC1D16
NUMA11	1.53E-07	0.454187	0.139	0.12	0.002436	Saliva_Fibr	NUMA1
EXOSC61	1.62E-07	0.293843	0.065	0.047	0.002589	Saliva_Fibr	EXOSC6
ZNF32	1.65E-07	0.313007	0.091	0.071	0.002636	Saliva_Fibr	ZNF32
SLFN51	1.65E-07	0.275518	0.092	0.071	0.002636	Saliva_Fibr	SLFN5
IDS1	1.88E-07	0.453524	0.199	0.185	0.002992	Saliva_Fibr	IDS
PDCD7	1.9E-07	0.353117	0.111	0.09	0.003026	Saliva_Fibr	PDCD7
ZC3H6	1.98E-07	0.393753	0.089	0.07	0.003152	Saliva_Fibr	ZC3H6
LMF2	1.98E-07	0.418139	0.109	0.089	0.003155	Saliva_Fibr	LMF2
PEPD1	2.03E-07	0.392618	0.122	0.101	0.00324	Saliva_Fibr	PEPD
RBPJ	2.18E-07	0.451819	0.206	0.192	0.00347	Saliva_Fibr	RBPJ
BAZ1B1	2.29E-07	0.360019	0.102	0.083	0.003655	Saliva_Fibr	BAZ1B

ABLIM1	2.36E-07	0.300991	0.297	0.287	0.003763	Saliva_Fibr	ABLIM1
FER	2.42E-07	0.284158	0.059	0.043	0.003853	Saliva_Fibr	FER
LRIG3	2.6E-07	0.324799	0.066	0.049	0.004153	Saliva_Fibr	LRIG3
AMOTL11	2.68E-07	0.340765	0.074	0.056	0.004281	Saliva_Fibr	AMOTL1
DOCK11	2.73E-07	0.274202	0.053	0.037	0.004354	Saliva_Fibr	DOCK1
METTL161	2.77E-07	0.256823	0.053	0.037	0.004417	Saliva_Fibr	METTL16
SCARB22	2.9E-07	0.522228	0.171	0.157	0.004621	Saliva_Fibr	SCARB2
RHOU1	3E-07	0.3313	0.062	0.045	0.004778	Saliva_Fibr	RHOU
RCN11	3.03E-07	0.49102	0.163	0.147	0.004829	Saliva_Fibr	RCN1
MSN1	3.34E-07	0.286701	0.178	0.156	0.005323	Saliva_Fibr	MSN
FAM102B	3.34E-07	0.261056	0.044	0.029	0.005325	Saliva_Fibr	FAM102B
UAP11	4.3E-07	0.500814	0.195	0.183	0.006861	Saliva_Fibr	UAP1
C19orf601	4.5E-07	0.369036	0.242	0.229	0.007177	Saliva_Fibr	C19orf60
MKLN1	4.71E-07	0.480823	0.127	0.11	0.007508	Saliva_Fibr	MKLN1
BBX	5.07E-07	0.396114	0.202	0.186	0.008093	Saliva_Fibr	BBX
FAM127B	5.13E-07	0.394654	0.098	0.08	0.008174	Saliva_Fibr	FAM127B
NDUFS1	5.52E-07	0.430034	0.107	0.088	0.008799	Saliva_Fibr	NDUFS1
EPM2AIP1	5.68E-07	0.387817	0.091	0.072	0.009057	Saliva_Fibr	EPM2AIP1
TMEM203	5.94E-07	0.325516	0.099	0.079	0.009467	Saliva_Fibr	TMEM203
PKDCC1	6.63E-07	0.340638	0.068	0.052	0.010575	Saliva_Fibr	PKDCC
ZKSCAN11	6.97E-07	0.466394	0.133	0.116	0.01111	Saliva_Fibr	ZKSCAN1
PTGES31	7.17E-07	0.359321	0.374	0.394	0.011442	Saliva_Fibr	PTGES3
CUL5	7.57E-07	0.413793	0.095	0.078	0.012076	Saliva_Fibr	CUL5
CIR11	9.97E-07	0.505294	0.18	0.168	0.015901	Saliva_Fibr	CIR1
ZBTB4	1.01E-06	0.374605	0.092	0.074	0.016111	Saliva_Fibr	ZBTB4
WBSCR16	1.01E-06	0.287871	0.056	0.041	0.016124	Saliva_Fibr	WBSCR16
RTN3	1.02E-06	0.265206	0.193	0.266	0.01625	Saliva_Fibr	RTN3
VPS28	1.06E-06	0.389878	0.332	0.35	0.016851	Saliva_Fibr	VPS28
PLBD2	1.25E-06	0.253472	0.066	0.05	0.019934	Saliva_Fibr	PLBD2
UPF3A1	1.43E-06	0.423527	0.162	0.145	0.022764	Saliva_Fibr	UPF3A
SCFD11	1.44E-06	0.461747	0.13	0.114	0.022965	Saliva_Fibr	SCFD1
WDR73	1.47E-06	0.272751	0.061	0.045	0.023439	Saliva_Fibr	WDR73
SESN3	1.59E-06	0.292581	0.099	0.08	0.025375	Saliva_Fibr	SESN3
USP142	1.66E-06	0.432076	0.117	0.1	0.026507	Saliva_Fibr	USP14
SAT21	1.68E-06	0.419352	0.214	0.204	0.026761	Saliva_Fibr	SAT2
XRCC51	1.9E-06	0.473499	0.248	0.248	0.030351	Saliva_Fibr	XRCC5
SIK2	1.91E-06	0.26171	0.052	0.037	0.030521	Saliva_Fibr	SIK2
PSMD81	1.94E-06	0.399834	0.317	0.333	0.030985	Saliva_Fibr	PSMD8
CHURC11	1.97E-06	0.493986	0.222	0.217	0.031438	Saliva_Fibr	CHURC1
FGD5.AS1	2.05E-06	0.44593	0.126	0.109	0.032686	Saliva_Fibr	FGD5.AS1
EXOSC7	2.14E-06	0.387541	0.106	0.088	0.03418	Saliva_Fibr	EXOSC7
ARHGAP1	2.24E-06	0.322647	0.077	0.06	0.035785	Saliva_Fibr	ARHGAP1
VEGFA	2.25E-06	0.35841	0.102	0.084	0.035891	Saliva_Fibr	VEGFA
RPS6KA5	2.48E-06	0.37032	0.084	0.067	0.039594	Saliva_Fibr	RPS6KA5
SRSF31	2.62E-06	0.308584	0.411	0.435	0.041781	Saliva_Fibr	SRSF3
HNRNPAB1	3.36E-06	0.432133	0.179	0.166	0.053665	Saliva_Fibr	HNRNPAB
PSMB51	3.49E-06	0.478237	0.238	0.241	0.055626	Saliva_Fibr	PSMB5

UVRAG1	3.58E-06	0.258816	0.064	0.048	0.05708	Saliva_Fibr	UVRAG
CAMK2D	3.63E-06	0.313499	0.075	0.059	0.057951	Saliva_Fibr	CAMK2D
ABHD17A1	3.84E-06	0.384357	0.133	0.116	0.061307	Saliva_Fibr	ABHD17A
FAM210B2	4.09E-06	0.411358	0.109	0.093	0.065301	Saliva_Fibr	FAM210B
PKIG1	4.66E-06	0.412379	0.116	0.099	0.074366	Saliva_Fibr	PKIG
PALLD	4.79E-06	0.467294	0.172	0.16	0.076474	Saliva_Fibr	PALLD
SYNCRIP	4.85E-06	0.266818	0.138	0.188	0.077344	Saliva_Fibr	SYNCRIP
SSBP12	4.92E-06	0.430139	0.277	0.284	0.078522	Saliva_Fibr	SSBP1
XPA	5.26E-06	0.353635	0.104	0.087	0.083928	Saliva_Fibr	XPA
COPA1	5.31E-06	0.472725	0.159	0.146	0.08466	Saliva_Fibr	COPA
BLVRB	5.34E-06	0.25487	0.289	0.288	0.085227	Saliva_Fibr	BLVRB
PRDX6	5.44E-06	0.392161	0.349	0.376	0.086767	Saliva_Fibr	PRDX6
AIP1	5.57E-06	0.314634	0.147	0.129	0.088855	Saliva_Fibr	AIP
PRRC2B1	5.64E-06	0.373647	0.104	0.087	0.090017	Saliva_Fibr	PRRC2B
RFX2	5.71E-06	0.255345	0.063	0.048	0.091	Saliva_Fibr	RFX2
C6orf203	5.73E-06	0.280966	0.062	0.047	0.091417	Saliva_Fibr	C6orf203
SF11	6.46E-06	0.429075	0.238	0.235	0.103084	Saliva_Fibr	SF1
ZBED5	7.6E-06	0.314122	0.073	0.058	0.121214	Saliva_Fibr	ZBED5
CLUAP1	7.6E-06	0.303071	0.075	0.059	0.121273	Saliva_Fibr	CLUAP1
CABIN1	7.89E-06	0.286401	0.07	0.054	0.125865	Saliva_Fibr	CABIN1
CREB3L21	8.24E-06	0.456776	0.115	0.1	0.131413	Saliva_Fibr	CREB3L2
PHACTR21	8.87E-06	0.421416	0.194	0.182	0.141481	Saliva_Fibr	PHACTR2
ABCE11	9.11E-06	0.440989	0.112	0.096	0.145218	Saliva_Fibr	ABCE1
ZNF358	9.22E-06	0.319684	0.07	0.055	0.147123	Saliva_Fibr	ZNF358
TGFBR1	9.26E-06	0.330804	0.077	0.061	0.147692	Saliva_Fibr	TGFBR1
GALNT21	1.08E-05	0.322405	0.071	0.056	0.171606	Saliva_Fibr	GALNT2
DNASE2	1.13E-05	0.316363	0.086	0.07	0.180183	Saliva_Fibr	DNASE2
EIF1AX	1.16E-05	0.427629	0.318	0.339	0.185248	Saliva_Fibr	EIF1AX
TMEM179I	1.16E-05	0.43354	0.205	0.198	0.185316	Saliva_Fibr	TMEM179B
TM2D11	1.17E-05	0.509236	0.167	0.157	0.186933	Saliva_Fibr	TM2D1
RALGAPA1	1.21E-05	0.405837	0.09	0.075	0.193638	Saliva_Fibr	RALGAPA1
LNPEP1	1.28E-05	0.337558	0.083	0.067	0.203368	Saliva_Fibr	LNPEP
TFDP2	1.37E-05	0.326389	0.124	0.108	0.218654	Saliva_Fibr	TFDP2
VKORC1L1	1.4E-05	0.357265	0.075	0.06	0.22386	Saliva_Fibr	VKORC1L1
CLN51	1.52E-05	0.389203	0.094	0.078	0.242873	Saliva_Fibr	CLN5
KDELR32	1.53E-05	0.355804	0.101	0.086	0.244152	Saliva_Fibr	KDELR3
NDUFS5	1.66E-05	0.260314	0.424	0.457	0.265396	Saliva_Fibr	NDUFS5
AKT2	1.73E-05	0.346534	0.083	0.068	0.276161	Saliva_Fibr	AKT2
CHCHD11	1.81E-05	0.259138	0.13	0.175	0.287893	Saliva_Fibr	CHCHD1
MRPS261	1.82E-05	0.392679	0.163	0.15	0.290125	Saliva_Fibr	MRPS26
HCFC2	1.83E-05	0.274518	0.059	0.045	0.291875	Saliva_Fibr	HCFC2
AKR7A2	1.86E-05	0.456518	0.159	0.148	0.296222	Saliva_Fibr	AKR7A2
TRIM381	1.97E-05	0.319968	0.085	0.07	0.314206	Saliva_Fibr	TRIM38
HEY11	1.99E-05	0.254465	0.039	0.027	0.31736	Saliva_Fibr	HEY1
TNRC6A	2.02E-05	0.385569	0.111	0.096	0.321746	Saliva_Fibr	TNRC6A
ZNF493	2.05E-05	0.260811	0.056	0.042	0.32634	Saliva_Fibr	ZNF493
MRAS	2.07E-05	0.263068	0.046	0.033	0.330183	Saliva_Fibr	MRAS

SAMD4B	2.11E-05	0.408059	0.107	0.092	0.336728	Saliva_Fibr	SAMD4B
AP2A1	2.13E-05	0.350276	0.096	0.081	0.339877	Saliva_Fibr	AP2A1
AUTS2	2.2E-05	0.326251	0.072	0.058	0.350912	Saliva_Fibr	AUTS2
ARL12	2.21E-05	0.524044	0.207	0.207	0.351987	Saliva_Fibr	ARL1
EAPP1	2.27E-05	0.480867	0.19	0.184	0.361629	Saliva_Fibr	EAPP
DPF2	2.32E-05	0.368924	0.089	0.074	0.369651	Saliva_Fibr	DPF2
CEP681	2.43E-05	0.250583	0.056	0.042	0.3868	Saliva_Fibr	CEP68
TTC372	2.47E-05	0.406456	0.099	0.085	0.394515	Saliva_Fibr	TTC37
TUG11	2.58E-05	0.260683	0.099	0.134	0.411961	Saliva_Fibr	TUG1
MAP7D3	2.79E-05	0.28242	0.078	0.063	0.444465	Saliva_Fibr	MAP7D3
DNAJC32	2.84E-05	0.261194	0.159	0.21	0.453495	Saliva_Fibr	DNAJC3
LHFPL21	3.03E-05	0.322177	0.067	0.053	0.482979	Saliva_Fibr	LHFPL2
PCBD11	3.16E-05	0.463129	0.167	0.158	0.504185	Saliva_Fibr	PCBD1
ARFGAP32	3.44E-05	0.281254	0.12	0.16	0.548841	Saliva_Fibr	ARFGAP3
TGS1	3.81E-05	0.290937	0.073	0.059	0.607153	Saliva_Fibr	TGS1
SF3B11	3.85E-05	0.47119	0.248	0.254	0.614789	Saliva_Fibr	SF3B1
EPS81	4.28E-05	0.326967	0.094	0.079	0.682244	Saliva_Fibr	EPS8
ERH	4.31E-05	0.352037	0.301	0.312	0.687525	Saliva_Fibr	ERH
HERC11	4.31E-05	0.344344	0.08	0.066	0.688124	Saliva_Fibr	HERC1
LTBR1	4.33E-05	0.39225	0.102	0.088	0.69029	Saliva_Fibr	LTBR
LUC7L21	4.38E-05	0.30681	0.065	0.051	0.698988	Saliva_Fibr	LUC7L2
SAV1	4.44E-05	0.404437	0.12	0.106	0.708448	Saliva_Fibr	SAV1
SLTM1	4.74E-05	0.255468	0.15	0.197	0.756586	Saliva_Fibr	SLTM
MAP3K2	5.17E-05	0.483978	0.136	0.124	0.824728	Saliva_Fibr	MAP3K2
MAZ	5.21E-05	0.284929	0.104	0.088	0.831534	Saliva_Fibr	MAZ
ZMPSTE24	5.32E-05	0.369351	0.092	0.078	0.849043	Saliva_Fibr	ZMPSTE24
MIB1	5.45E-05	0.348585	0.079	0.065	0.868675	Saliva_Fibr	MIB1
DAG1	5.5E-05	0.347063	0.075	0.062	0.877469	Saliva_Fibr	DAG1
TDRD31	5.69E-05	0.258139	0.054	0.041	0.908084	Saliva_Fibr	TDRD3
SEPT8	5.73E-05	0.292798	0.069	0.056	0.913518	Saliva_Fibr	SEPT8
BLOC1S61	5.85E-05	0.371616	0.11	0.096	0.932603	Saliva_Fibr	BLOC1S6
ZFYVE211	6.09E-05	0.483374	0.157	0.149	0.971779	Saliva_Fibr	ZFYVE21
MAVS	6.55E-05	0.31023	0.064	0.051	1	Saliva_Fibr	MAVS
CREBZF	6.66E-05	0.337837	0.088	0.074	1	Saliva_Fibr	CREBZF
C11orf58	6.74E-05	0.369643	0.342	0.37	1	Saliva_Fibr	C11orf58
SETD5	6.81E-05	0.434716	0.13	0.118	1	Saliva_Fibr	SETD5
SLC22A171	6.89E-05	0.290328	0.053	0.041	1	Saliva_Fibr	SLC22A17
SERTAD4.A	7.49E-05	0.334964	0.06	0.047	1	Saliva_Fibr	SERTAD4.AS1
PTCD3	7.56E-05	0.340569	0.082	0.068	1	Saliva_Fibr	PTCD3
IL13RA1	7.6E-05	0.464102	0.132	0.12	1	Saliva_Fibr	IL13RA1
MFSD10	7.81E-05	0.409767	0.136	0.123	1	Saliva_Fibr	MFSD10
FOXN3	7.83E-05	0.354622	0.138	0.125	1	Saliva_Fibr	FOXN3
ZFYVE16	8.23E-05	0.369666	0.087	0.073	1	Saliva_Fibr	ZFYVE16
SERINC31	8.68E-05	0.487701	0.208	0.208	1	Saliva_Fibr	SERINC3
ASMTL	9.51E-05	0.263564	0.065	0.052	1	Saliva_Fibr	ASMTL
CLSTN11	9.56E-05	0.484954	0.133	0.122	1	Saliva_Fibr	CLSTN1
CLCC11	0.000106	0.29148	0.054	0.042	1	Saliva_Fibr	CLCC1

TK2	0.00012	0.274612	0.054	0.042	1	Saliva_Fibr	TK2
KIF13A	0.000126	0.269182	0.063	0.051	1	Saliva_Fibr	KIF13A
SLC39A61	0.000135	0.257735	0.074	0.1	1	Saliva_Fibr	SLC39A6
RNF187	0.000139	0.367403	0.138	0.126	1	Saliva_Fibr	RNF187
TCF251	0.000148	0.448821	0.279	0.295	1	Saliva_Fibr	TCF25
RHOB	0.00016	0.312091	0.208	0.201	1	Saliva_Fibr	RHOB
MYLIP	0.000167	0.359894	0.175	0.164	1	Saliva_Fibr	MYLIP
STAG11	0.00017	0.318973	0.084	0.071	1	Saliva_Fibr	STAG1
MPC11	0.000185	0.276669	0.162	0.213	1	Saliva_Fibr	MPC1
PXDN1	0.000188	0.250248	0.05	0.039	1	Saliva_Fibr	PXDN
HDLBP2	0.000189	0.368674	0.255	0.262	1	Saliva_Fibr	HDLBP
IER22	0.0002	0.263332	0.501	0.549	1	Saliva_Fibr	IER2
DNM1L	0.00024	0.368459	0.092	0.08	1	Saliva_Fibr	DNM1L
MAP2K21	0.000261	0.403553	0.202	0.198	1	Saliva_Fibr	MAP2K2
CIZ1	0.000282	0.336912	0.079	0.066	1	Saliva_Fibr	CIZ1
OSBPL8	0.000299	0.4152	0.182	0.177	1	Saliva_Fibr	OSBPL8
SPPL2A	0.000306	0.494194	0.138	0.13	1	Saliva_Fibr	SPPL2A
SENP6	0.000317	0.436608	0.119	0.108	1	Saliva_Fibr	SENP6
C15orf61	0.000319	0.383878	0.124	0.113	1	Saliva_Fibr	C15orf61
UACA1	0.000323	0.458887	0.122	0.111	1	Saliva_Fibr	UACA
FNIP1	0.000329	0.376551	0.096	0.084	1	Saliva_Fibr	FNIP1
ACBD6	0.000332	0.339807	0.099	0.087	1	Saliva_Fibr	ACBD6
PHAX	0.000336	0.381497	0.11	0.099	1	Saliva_Fibr	PHAX
ENAH	0.000396	0.423545	0.128	0.118	1	Saliva_Fibr	ENAH
FAM217B	0.000414	0.270191	0.061	0.05	1	Saliva_Fibr	FAM217B
BTBD6	0.000449	0.352236	0.076	0.064	1	Saliva_Fibr	BTBD6
SMC1A	0.000513	0.337789	0.085	0.074	1	Saliva_Fibr	SMC1A
SMAD4	0.000523	0.313763	0.077	0.066	1	Saliva_Fibr	SMAD4
HOMER31	0.000543	0.267681	0.068	0.057	1	Saliva_Fibr	HOMER3
HOOK3	0.000551	0.425693	0.155	0.149	1	Saliva_Fibr	HOOK3
COPB22	0.000565	0.263454	0.122	0.157	1	Saliva_Fibr	COPB2
APOOL	0.000585	0.250135	0.049	0.039	1	Saliva_Fibr	APOOL
SDF21	0.000632	0.252852	0.11	0.141	1	Saliva_Fibr	SDF2
MRPL431	0.000639	0.429079	0.193	0.192	1	Saliva_Fibr	MRPL43
GPATCH21	0.000663	0.318452	0.072	0.061	1	Saliva_Fibr	GPATCH2
ATL1	0.000667	0.257686	0.049	0.039	1	Saliva_Fibr	ATL1
ATP5J2	0.000684	0.300544	0.393	0.439	1	Saliva_Fibr	ATP5J
CARM1	0.000702	0.256729	0.047	0.037	1	Saliva_Fibr	CARM1
ACYP1	0.000734	0.257943	0.075	0.064	1	Saliva_Fibr	ACYP1
LARP7	0.000741	0.433353	0.141	0.134	1	Saliva_Fibr	LARP7
TP53I13	0.000795	0.303883	0.084	0.073	1	Saliva_Fibr	TP53I13
SH3BGRL	0.000796	0.284462	0.247	0.245	1	Saliva_Fibr	SH3BGRL
ERCC11	0.000804	0.25127	0.122	0.157	1	Saliva_Fibr	ERCC1
DDX182	0.000814	0.379631	0.207	0.205	1	Saliva_Fibr	DDX18
BMPR21	0.000828	0.356217	0.114	0.103	1	Saliva_Fibr	BMPR2
JAGN1	0.00083	0.374896	0.115	0.105	1	Saliva_Fibr	JAGN1
PRKRIR	0.000838	0.256204	0.058	0.047	1	Saliva_Fibr	PRKRIR

CDC42BPB	0.000848	0.322866	0.077	0.066	1	Saliva_Fibr	CDC42BPB
PSMD1	0.000892	0.356348	0.114	0.104	1	Saliva_Fibr	PSMD1
CCS1	0.000931	0.339805	0.121	0.111	1	Saliva_Fibr	CCS
C11orf68	0.000971	0.290778	0.07	0.059	1	Saliva_Fibr	C11orf68
PSMG21	0.000976	0.438264	0.224	0.23	1	Saliva_Fibr	PSMG2
CDIPT	0.000988	0.369767	0.125	0.115	1	Saliva_Fibr	CDIPT
GOPC	0.001019	0.375254	0.111	0.101	1	Saliva_Fibr	GOPC
DHRS72	0.001037	0.338292	0.21	0.209	1	Saliva_Fibr	DHRS7
ZNF397	0.001137	0.258226	0.059	0.049	1	Saliva_Fibr	ZNF397
ZNF1481	0.001154	0.432199	0.134	0.127	1	Saliva_Fibr	ZNF148
WDFY3	0.0012	0.28411	0.061	0.051	1	Saliva_Fibr	WDFY3
CNIH41	0.001221	0.427442	0.143	0.137	1	Saliva_Fibr	CNIH4
YIPF51	0.001237	0.417519	0.126	0.118	1	Saliva_Fibr	YIPF5
YY1	0.001327	0.440994	0.221	0.228	1	Saliva_Fibr	YY1
EIF2AK42	0.001376	0.303075	0.08	0.069	1	Saliva_Fibr	EIF2AK4
SMARCA51	0.001426	0.389308	0.137	0.13	1	Saliva_Fibr	SMARCA5
FAM133B	0.001533	0.292495	0.159	0.201	1	Saliva_Fibr	FAM133B
CRCP	0.001605	0.274946	0.06	0.05	1	Saliva_Fibr	CRCP
XPC2	0.00163	0.257103	0.071	0.061	1	Saliva_Fibr	XPC
TBL1XR1	0.001677	0.443204	0.156	0.151	1	Saliva_Fibr	TBL1XR1
JOSD21	0.001684	0.339208	0.127	0.118	1	Saliva_Fibr	JOSD2
TNPO1	0.001701	0.268034	0.099	0.127	1	Saliva_Fibr	TNPO1
HLTF	0.001758	0.252337	0.059	0.05	1	Saliva_Fibr	HLTF
POGZ	0.00177	0.367282	0.098	0.089	1	Saliva_Fibr	POGZ
AK3	0.001806	0.267088	0.126	0.16	1	Saliva_Fibr	AK3
ARHGAP12	0.001808	0.374296	0.092	0.083	1	Saliva_Fibr	ARHGAP12
VDAC3	0.001857	0.370555	0.188	0.187	1	Saliva_Fibr	VDAC3
MID1IP1	0.001957	0.272725	0.086	0.075	1	Saliva_Fibr	MID1IP1
FUNDC2	0.002099	0.310358	0.191	0.188	1	Saliva_Fibr	FUNDC2
NFAT51	0.00217	0.425565	0.135	0.128	1	Saliva_Fibr	NFAT5
NSD11	0.002173	0.388332	0.11	0.101	1	Saliva_Fibr	NSD1
ALDH3A2	0.002187	0.422229	0.133	0.127	1	Saliva_Fibr	ALDH3A2
MNAT1	0.002198	0.319944	0.077	0.068	1	Saliva_Fibr	MNAT1
PHYHD1	0.002398	0.328538	0.069	0.06	1	Saliva_Fibr	PHYHD1
TM2D2	0.00241	0.287013	0.101	0.129	1	Saliva_Fibr	TM2D2
PSMA32	0.002443	0.432883	0.192	0.194	1	Saliva_Fibr	PSMA3
ANKFY1	0.002454	0.257716	0.052	0.043	1	Saliva_Fibr	ANKFY1
SH3PXD2A	0.002514	0.29298	0.094	0.084	1	Saliva_Fibr	SH3PXD2A
PABPN1	0.002536	0.255219	0.121	0.152	1	Saliva_Fibr	PABPN1
SETX2	0.002904	0.364937	0.11	0.102	1	Saliva_Fibr	SETX
EPRS3	0.002991	0.399935	0.138	0.133	1	Saliva_Fibr	EPRS
NHLRC2	0.003097	0.266596	0.05	0.042	1	Saliva_Fibr	NHLRC2
THOC7	0.003101	0.27731	0.156	0.197	1	Saliva_Fibr	THOC7
NGFRAP1	0.003197	0.346303	0.206	0.207	1	Saliva_Fibr	NGFRAP1
MAP4K5	0.003227	0.365951	0.096	0.087	1	Saliva_Fibr	MAP4K5
UBR3	0.003513	0.275139	0.062	0.052	1	Saliva_Fibr	UBR3
VAMP31	0.003749	0.26723	0.108	0.136	1	Saliva_Fibr	VAMP3

TMCO11	0.003796	0.259281	0.186	0.234	1	Saliva_Fibr	TMCO1
TPRG1L	0.003801	0.303287	0.076	0.067	1	Saliva_Fibr	TPRG1L
NUDC	0.003802	0.398049	0.24	0.251	1	Saliva_Fibr	NUDC
NF1	0.004049	0.406209	0.095	0.087	1	Saliva_Fibr	NF1
NUFIP2	0.004215	0.257684	0.123	0.154	1	Saliva_Fibr	NUFIP2
YME1L11	0.004334	0.426968	0.199	0.202	1	Saliva_Fibr	YME1L1
MRTO41	0.004337	0.292298	0.075	0.066	1	Saliva_Fibr	MRTO4
YIPF6	0.004499	0.333103	0.117	0.148	1	Saliva_Fibr	YIPF6
NR2F61	0.004508	0.373868	0.091	0.083	1	Saliva_Fibr	NR2F6
FAM172A	0.004554	0.29397	0.075	0.066	1	Saliva_Fibr	FAM172A
CDC261	0.004706	0.304885	0.102	0.128	1	Saliva_Fibr	CDC26
ATXN10	0.004779	0.277204	0.114	0.143	1	Saliva_Fibr	ATXN10
RBFA	0.004794	0.253262	0.067	0.058	1	Saliva_Fibr	RBFA
UGGT2	0.00483	0.272048	0.054	0.045	1	Saliva_Fibr	UGGT2
TBC1D51	0.004875	0.286119	0.068	0.059	1	Saliva_Fibr	TBC1D5
PPP1R12A1	0.0049	0.361224	0.18	0.178	1	Saliva_Fibr	PPP1R12A
SAP301	0.005041	0.285304	0.063	0.054	1	Saliva_Fibr	SAP30
CTDSP2	0.00534	0.286438	0.137	0.171	1	Saliva_Fibr	CTDSP2
MUM1	0.005637	0.365165	0.096	0.088	1	Saliva_Fibr	MUM1
NSA21	0.005663	0.250654	0.202	0.252	1	Saliva_Fibr	NSA2
DECR1	0.005816	0.259067	0.132	0.165	1	Saliva_Fibr	DECR1
RSF1	0.005842	0.458273	0.179	0.182	1	Saliva_Fibr	RSF1
NDUFAB11	0.006086	0.381813	0.234	0.247	1	Saliva_Fibr	NDUFAB1
FLYWCH21	0.006089	0.373399	0.12	0.114	1	Saliva_Fibr	FLYWCH2
KIN	0.006148	0.299841	0.072	0.064	1	Saliva_Fibr	KIN
DLGAP4	0.006154	0.386052	0.124	0.119	1	Saliva_Fibr	DLGAP4
KMT2C	0.006357	0.287452	0.125	0.155	1	Saliva_Fibr	KMT2C
DDIT3	0.006376	0.348372	0.159	0.155	1	Saliva_Fibr	DDIT3
PHGDH1	0.006487	0.266682	0.092	0.116	1	Saliva_Fibr	PHGDH
GABARAPL	0.006645	0.259362	0.136	0.169	1	Saliva_Fibr	GABARAPL1
LPIN2	0.006748	0.280355	0.083	0.074	1	Saliva_Fibr	LPIN2
BRIX1	0.007476	0.328048	0.099	0.091	1	Saliva_Fibr	BRIX1
MRPL27	0.007674	0.430525	0.145	0.144	1	Saliva_Fibr	MRPL27
CYHR11	0.008134	0.274183	0.095	0.118	1	Saliva_Fibr	CYHR1
SRM1	0.008283	0.341911	0.167	0.164	1	Saliva_Fibr	SRM
UBE3A	0.008559	0.290308	0.111	0.137	1	Saliva_Fibr	UBE3A
PUS7L1	0.008594	0.325177	0.078	0.07	1	Saliva_Fibr	PUS7L
AKAP91	0.0088	0.452334	0.252	0.272	1	Saliva_Fibr	AKAP9
KIAA11091	0.009196	0.365709	0.093	0.086	1	Saliva_Fibr	KIAA1109
DHX29	0.00925	0.424026	0.134	0.131	1	Saliva_Fibr	DHX29
GON4L1	0.009262	0.32405	0.088	0.081	1	Saliva_Fibr	GON4L
LTV1	0.009703	0.263366	0.065	0.057	1	Saliva_Fibr	LTV1
ASB8	0.009841	0.293356	0.082	0.074	1	Saliva_Fibr	ASB8
TAGLN	0	5.030607	0.858	0.077	0	Saliva_Peri	TAGLN
ACTA2	0	4.621732	0.781	0.059	0	Saliva_Peri	ACTA2
TPM2	0	4.399587	0.802	0.079	0	Saliva_Peri	TPM2
MYL91	0	3.930595	0.784	0.113	0	Saliva_Peri	MYL9

TPM12	0	3.701088	0.762	0.183	0	Saliva_Peri	TPM1
MYH11	0	3.605143	0.498	0.035	0	Saliva_Peri	MYH11
CALD11	0	3.16329	0.844	0.19	0	Saliva_Peri	CALD1
RGS5	0	3.135748	0.407	0.026	0	Saliva_Peri	RGS5
MCAM1	0	3.008213	0.568	0.025	0	Saliva_Peri	MCAM
C11orf96	0	2.976205	0.698	0.099	0	Saliva_Peri	C11orf96
NR2F22	0	2.92307	0.603	0.083	0	Saliva_Peri	NR2F2
IGFBP72	0	2.912549	0.918	0.26	0	Saliva_Peri	IGFBP7
IGFBP51	0	2.808038	0.739	0.132	0	Saliva_Peri	IGFBP5
PPP1R14A1	0	2.642799	0.48	0.043	0	Saliva_Peri	PPP1R14A
TINAGL11	0	2.59141	0.55	0.079	0	Saliva_Peri	TINAGL1
NOTCH31	0	2.580166	0.481	0.034	0	Saliva_Peri	NOTCH3
PDGFRB1	0	2.477578	0.484	0.064	0	Saliva_Peri	PDGFRB
CPE1	0	2.415844	0.537	0.1	0	Saliva_Peri	CPE
CAV12	0	2.3823	0.758	0.194	0	Saliva_Peri	CAV1
MFGE81	0	2.301031	0.49	0.096	0	Saliva_Peri	MFGE8
SYNPO21	0	2.122014	0.385	0.023	0	Saliva_Peri	SYNPO2
GUCY1A31	0	2.046074	0.376	0.031	0	Saliva_Peri	GUCY1A3
A2M1	0	2.038864	0.506	0.081	0	Saliva_Peri	A2M
COX4I2	0	2.026244	0.329	0.012	0	Saliva_Peri	COX4I2
MYLK	0	2.015045	0.407	0.052	0	Saliva_Peri	MYLK
PLN	0	1.903781	0.307	0.017	0	Saliva_Peri	PLN
GJA41	0	1.847645	0.302	0.015	0	Saliva_Peri	GJA4
RCAN21	0	1.814157	0.324	0.028	0	Saliva_Peri	RCAN2
FRZB	0	1.788314	0.286	0.011	0	Saliva_Peri	FRZB
FILIP12	0	1.733546	0.288	0.02	0	Saliva_Peri	FILIP1
ITGA7	0	1.727126	0.245	0.008	0	Saliva_Peri	ITGA7
MT.ATP65	0	1.695734	1	0.897	0	Saliva_Peri	MT.ATP6
KCNJ81	0	1.537709	0.156	0.006	0	Saliva_Peri	KCNJ8
ACTG2	0	1.407933	0.214	0.014	0	Saliva_Peri	ACTG2
ADRA2A	0	1.39213	0.219	0.018	0	Saliva_Peri	ADRA2A
LGI4	0	1.311668	0.16	0.01	0	Saliva_Peri	LGI4
TCF151	0	1.200246	0.119	0.003	0	Saliva_Peri	TCF15
AVPR1A	0	1.152694	0.123	0.003	0	Saliva_Peri	AVPR1A
ATP1A2	0	1.11786	0.151	0.004	0	Saliva_Peri	ATP1A2
HRC	0	0.718112	0.086	0.003	0	Saliva_Peri	HRC
DGKG	0	0.629896	0.083	0.002	0	Saliva_Peri	DGKG
GPR20	0	0.416192	0.061	0.001	0	Saliva_Peri	GPR20
RBPMS2	5.3E-308	0.980089	0.142	0.008	8.4E-304	Saliva_Peri	RBPMS2
MT.CYB5	1.1E-301	1.503839	1	0.933	1.8E-297	Saliva_Peri	MT.CYB
CCDC31	2E-301	1.569699	0.241	0.024	3.1E-297	Saliva_Peri	CCDC3
MRVI1	9.4E-299	1.266649	0.189	0.015	1.5E-294	Saliva_Peri	MRVI1
AOC31	4.5E-298	1.165026	0.165	0.011	7.2E-294	Saliva_Peri	AOC3
PDE5A1	1.7E-294	1.416736	0.213	0.019	2.8E-290	Saliva_Peri	PDE5A
NRIP2	1.2E-288	0.549745	0.073	0.002	2E-284	Saliva_Peri	NRIP2
KCNAB1	1.6E-288	1.19575	0.162	0.011	2.5E-284	Saliva_Peri	KCNAB1
MT.ND15	4.9E-284	1.538536	0.999	0.927	7.8E-280	Saliva_Peri	MT.ND1

COL18A12	7.3E-284	2.05362	0.401	0.071	1.2E-279	Saliva_Peri	COL18A1
MT.CO34	2.7E-281	1.40745	1	0.977	4.4E-277	Saliva_Peri	MT.CO3
COX6A2	2.3E-276	1.703842	0.059	0.001	3.6E-272	Saliva_Peri	COX6A2
TIMP32	3.4E-273	2.177889	0.613	0.163	5.5E-269	Saliva_Peri	TIMP3
NFASC	4E-272	1.04678	0.133	0.008	6.3E-268	Saliva_Peri	NFASC
RRAD1	2.1E-268	1.570873	0.268	0.033	3.3E-264	Saliva_Peri	RRAD
NDUFA4L2	2.3E-264	2.578218	0.393	0.073	3.6E-260	Saliva_Peri	NDUFA4L2
COL14A11	3.6E-261	1.650545	0.293	0.04	5.8E-257	Saliva_Peri	COL14A1
FILIP1L2	4.3E-260	2.204232	0.416	0.082	6.8E-256	Saliva_Peri	FILIP1L
C2orf401	5E-260	2.060638	0.284	0.038	8E-256	Saliva_Peri	C2orf40
MT.ND35	1.8E-258	1.341627	1	0.793	2.9E-254	Saliva_Peri	MT.ND3
WTIP	1.1E-257	1.32031	0.201	0.02	1.8E-253	Saliva_Peri	WTIP
ITIH52	2.6E-244	1.469183	0.274	0.037	4.2E-240	Saliva_Peri	ITIH5
MT.CO25	1.5E-238	1.088292	1	0.959	2.4E-234	Saliva_Peri	MT.CO2
CAP2	3E-238	0.873402	0.133	0.009	4.8E-234	Saliva_Peri	CAP2
FXYD11	4.2E-238	1.842542	0.409	0.083	6.6E-234	Saliva_Peri	FXYD1
LINC00310	8.9E-236	0.314487	0.043	0.001	1.4E-231	Saliva_Peri	LINC00310
EFHD11	1.2E-235	1.468303	0.23	0.028	1.8E-231	Saliva_Peri	EFHD1
FLNA2	7.9E-235	2.417324	0.524	0.147	1.3E-230	Saliva_Peri	FLNA
PPP1R12B	3E-229	1.690245	0.299	0.048	4.7E-225	Saliva_Peri	PPP1R12B
ATP1B2	1.9E-226	0.7999	0.111	0.007	3E-222	Saliva_Peri	ATP1B2
LHFP2	7.4E-224	2.039548	0.398	0.085	1.2E-219	Saliva_Peri	LHFP
MT.ND45	1.1E-222	1.087351	1	0.953	1.7E-218	Saliva_Peri	MT.ND4
MRGPRF1	2E-218	1.326245	0.209	0.025	3.1E-214	Saliva_Peri	MRGPRF
NRGN1	1.6E-216	1.050048	0.158	0.014	2.5E-212	Saliva_Peri	NRGN
MGST34	1.7E-215	2.09738	0.715	0.332	2.6E-211	Saliva_Peri	MGST3
SEPT72	2.2E-211	2.107216	0.718	0.337	3.5E-207	Saliva_Peri	SEPT7
ARHGEF17	2.4E-209	1.260282	0.159	0.015	3.9E-205	Saliva_Peri	ARHGEF17
KLHL23	8.9E-206	1.217558	0.169	0.017	1.4E-201	Saliva_Peri	KLHL23
TGFB1I12	2.3E-205	1.529221	0.295	0.05	3.6E-201	Saliva_Peri	TGFB1I1
CD813	2.2E-203	1.808347	0.732	0.32	3.5E-199	Saliva_Peri	CD81
CSRP21	6.6E-203	1.974844	0.367	0.079	1.1E-198	Saliva_Peri	CSRP2
MYOZ1	6.5E-201	1.116126	0.079	0.004	1E-196	Saliva_Peri	MYOZ1
ADIRF2	3E-200	1.753869	0.835	0.433	4.7E-196	Saliva_Peri	ADIRF
VIM3	1.3E-193	1.478947	0.899	0.491	2.1E-189	Saliva_Peri	VIM
SORBS22	4.7E-193	2.344788	0.422	0.111	7.4E-189	Saliva_Peri	SORBS2
PDE3A	4.8E-191	0.540963	0.076	0.004	7.7E-187	Saliva_Peri	PDE3A
DACT3	2.9E-188	0.869296	0.122	0.01	4.7E-184	Saliva_Peri	DACT3
JAG12	6.1E-181	1.569856	0.274	0.049	9.8E-177	Saliva_Peri	JAG1
MT.ND55	4.9E-179	1.22972	0.916	0.701	7.9E-175	Saliva_Peri	MT.ND5
LDB3	1.1E-178	0.813839	0.102	0.007	1.8E-174	Saliva_Peri	LDB3
MT.CO15	7.8E-178	1.064852	1	0.985	1.3E-173	Saliva_Peri	MT.CO1
GUCY1B31	3.7E-176	1.236073	0.17	0.02	5.9E-172	Saliva_Peri	GUCY1B3
SCN4B	1.1E-173	0.811766	0.072	0.004	1.8E-169	Saliva_Peri	SCN4B
ACTA11	2.2E-173	5.030559	0.086	0.005	3.4E-169	Saliva_Peri	ACTA1
MTRNR2L1	7.1E-172	1.902546	0.661	0.266	1.1E-167	Saliva_Peri	MTRNR2L12
CD361	1.5E-171	1.672249	0.151	0.016	2.4E-167	Saliva_Peri	CD36

NTRK21	4.1E-169	1.403084	0.278	0.052	6.5E-165	Saliva_Peri	NTRK2
SEPT4	1.7E-167	1.2689	0.181	0.024	2.8E-163	Saliva_Peri	SEPT4
MT.ND25	4.1E-166	0.899968	0.99	0.907	6.5E-162	Saliva_Peri	MT.ND2
PRKCDBP2	3E-157	1.726795	0.427	0.127	4.8E-153	Saliva_Peri	PRKCDBP
MAP1B1	2.8E-154	1.628206	0.351	0.087	4.5E-150	Saliva_Peri	MAP1B
PPP1R12A1	5.3E-151	1.982578	0.483	0.176	8.4E-147	Saliva_Peri	PPP1R12A
PTRF2	9E-151	1.707203	0.48	0.161	1.4E-146	Saliva_Peri	PTRF
EPAS11	9.3E-150	1.687274	0.347	0.089	1.5E-145	Saliva_Peri	EPAS1
CSRP1	3E-149	2.096694	0.45	0.153	4.8E-145	Saliva_Peri	CSRP1
EPS82	1.4E-147	1.960192	0.318	0.078	2.2E-143	Saliva_Peri	EPS8
RASL121	1.4E-146	0.78305	0.115	0.011	2.2E-142	Saliva_Peri	RASL12
SUCNR1	2E-145	0.843116	0.055	0.003	3.3E-141	Saliva_Peri	SUCNR1
MSRB32	8.8E-145	1.165256	0.205	0.034	1.4E-140	Saliva_Peri	MSRB3
MGP2	1.5E-141	0.976381	0.58	0.21	2.4E-137	Saliva_Peri	MGP
LBH	2.8E-140	1.658424	0.353	0.094	4.5E-136	Saliva_Peri	LBH
CSDC2	7.9E-138	0.50823	0.061	0.003	1.3E-133	Saliva_Peri	CSDC2
HES4	1E-137	1.637413	0.509	0.193	1.6E-133	Saliva_Peri	HES4
SLC38A111	4.1E-135	0.495122	0.047	0.002	6.5E-131	Saliva_Peri	SLC38A11
KANK21	8.1E-134	1.257071	0.224	0.042	1.3E-129	Saliva_Peri	KANK2
C1QTNF11	2.8E-131	1.266767	0.195	0.033	4.4E-127	Saliva_Peri	C1QTNF1
NEXN	8.2E-129	1.088414	0.152	0.021	1.3E-124	Saliva_Peri	NEXN
SPARC2	1.7E-127	1.629669	0.462	0.16	2.8E-123	Saliva_Peri	SPARC
MEF2C2	3.2E-127	1.636779	0.308	0.079	5.1E-123	Saliva_Peri	MEF2C
CSPG4	4E-127	0.590526	0.072	0.005	6.3E-123	Saliva_Peri	CSPG4
RASL11A	3.2E-126	0.817208	0.104	0.01	5.1E-122	Saliva_Peri	RASL11A
PRKG11	3.8E-119	0.972796	0.145	0.021	6.1E-115	Saliva_Peri	PRKG1
IFITM32	4.9E-119	1.385505	0.647	0.309	7.8E-115	Saliva_Peri	IFITM3
PTP4A31	3.1E-118	1.197889	0.18	0.031	4.9E-114	Saliva_Peri	PTP4A3
SUSD2	1E-117	0.448413	0.054	0.003	1.6E-113	Saliva_Peri	SUSD2
EHD22	1E-117	1.31883	0.217	0.044	1.7E-113	Saliva_Peri	EHD2
SELM4	1.8E-117	1.767285	0.624	0.345	2.8E-113	Saliva_Peri	SELM
RRAS2	1.8E-117	1.584084	0.332	0.097	2.9E-113	Saliva_Peri	RRAS
BCAM1	6.1E-115	1.392417	0.286	0.074	9.7E-111	Saliva_Peri	BCAM
RAMP1	3.3E-112	1.058192	0.152	0.024	5.3E-108	Saliva_Peri	RAMP1
ANGPT1	4.9E-112	0.790323	0.107	0.012	7.8E-108	Saliva_Peri	ANGPT1
SYNM	2.2E-111	0.844653	0.133	0.019	3.5E-107	Saliva_Peri	SYNM
TCAP	1.8E-107	3.187166	0.058	0.004	2.8E-103	Saliva_Peri	TCAP
GGT52	1.3E-103	1.36979	0.147	0.024	2.1E-99	Saliva_Peri	GGT5
SERPINI12	2.5E-103	0.998485	0.141	0.022	4E-99	Saliva_Peri	SERPINI1
TNS12	1.1E-101	1.281055	0.238	0.058	1.72E-97	Saliva_Peri	TNS1
DSTN1	4.8E-99	2.098685	0.686	0.562	7.58E-95	Saliva_Peri	DSTN
NEURL1B1	2.61E-97	1.022346	0.14	0.023	4.16E-93	Saliva_Peri	NEURL1B
ZFHX31	4.8E-96	1.533471	0.29	0.088	7.66E-92	Saliva_Peri	ZFHX3
COL4A22	9.87E-94	1.457492	0.22	0.054	1.57E-89	Saliva_Peri	COL4A2
LGALS12	5.74E-93	0.685185	0.788	0.408	9.15E-89	Saliva_Peri	LGALS1
SNCG1	3.5E-92	1.475481	0.34	0.117	5.58E-88	Saliva_Peri	SNCG
PDLIM32	4.54E-92	1.401275	0.225	0.057	7.24E-88	Saliva_Peri	PDLIM3

GJC1	1.82E-90	0.877122	0.1	0.013	2.9E-86	Saliva_Peri	GJC1
ISYNA1	5.62E-89	1.34858	0.224	0.058	8.97E-85	Saliva_Peri	ISYNA1
ANGPT21	1.01E-87	1.196251	0.148	0.028	1.61E-83	Saliva_Peri	ANGPT2
ARHGAP61	1.39E-87	0.762582	0.102	0.014	2.21E-83	Saliva_Peri	ARHGAP6
IGFBP22	3.05E-85	2.132263	0.207	0.052	4.86E-81	Saliva_Peri	IGFBP2
PRDM16	4.57E-83	0.271488	0.039	0.002	7.29E-79	Saliva_Peri	PRDM16
KCNMB1	8.8E-81	0.658559	0.104	0.015	1.4E-76	Saliva_Peri	KCNMB1
ARHGEF25	2.92E-80	0.51959	0.079	0.009	4.66E-76	Saliva_Peri	ARHGEF25
SEPW12	3.25E-79	1.583778	0.567	0.358	5.19E-75	Saliva_Peri	SEPW1
MTRNR2L1	3.72E-79	1.409642	0.293	0.097	5.94E-75	Saliva_Peri	MTRNR2L10
LINC00924	3.02E-77	0.431629	0.048	0.004	4.82E-73	Saliva_Peri	LINC00924
S100A41	1.04E-76	0.916623	0.744	0.463	1.66E-72	Saliva_Peri	S100A4
ZEB21	2.81E-76	1.258449	0.271	0.087	4.48E-72	Saliva_Peri	ZEB2
AC100830.	5.62E-76	0.501951	0.051	0.004	8.96E-72	Saliva_Peri	AC100830.3
DLC12	1.28E-75	1.205842	0.191	0.048	2.04E-71	Saliva_Peri	DLC1
CABP1	2.56E-75	0.329608	0.041	0.003	4.08E-71	Saliva_Peri	CABP1
WFDC1	4.7E-75	1.002477	0.147	0.031	7.49E-71	Saliva_Peri	WFDC1
CAV21	8.96E-75	1.334258	0.315	0.118	1.43E-70	Saliva_Peri	CAV2
LPP1	2.05E-74	1.572696	0.429	0.21	3.26E-70	Saliva_Peri	LPP
ID32	9.62E-74	1.30135	0.405	0.171	1.53E-69	Saliva_Peri	ID3
GUCY1A2	4.53E-73	0.689031	0.08	0.01	7.23E-69	Saliva_Peri	GUCY1A2
FHL11	3.43E-72	1.593826	0.329	0.128	5.47E-68	Saliva_Peri	FHL1
ANGPTL11	6.69E-72	0.998109	0.188	0.047	1.07E-67	Saliva_Peri	ANGPTL1
PGM51	1.87E-71	0.675489	0.083	0.011	2.98E-67	Saliva_Peri	PGM5
GPRC5C	5.99E-71	1.225295	0.187	0.049	9.55E-67	Saliva_Peri	GPRC5C
MYL6	9.19E-70	0.95032	0.82	0.806	1.47E-65	Saliva_Peri	MYL6
EGR13	2.6E-69	1.869153	0.38	0.172	4.15E-65	Saliva_Peri	EGR1
ADCY31	5.06E-69	0.857459	0.116	0.021	8.07E-65	Saliva_Peri	ADCY3
FOS3	2.71E-68	1.867106	0.607	0.424	4.32E-64	Saliva_Peri	FOS
CALM2	4.49E-68	0.918418	0.736	0.562	7.17E-64	Saliva_Peri	CALM2
PLCE1	2.94E-67	0.899265	0.116	0.022	4.69E-63	Saliva_Peri	PLCE1
CHCHD102	8.23E-67	1.50496	0.45	0.244	1.31E-62	Saliva_Peri	CHCHD10
CKM	1.14E-66	3.393034	0.069	0.009	1.82E-62	Saliva_Peri	CKM
COX7A12	2.12E-66	1.662486	0.329	0.138	3.39E-62	Saliva_Peri	COX7A1
CD634	7.31E-66	0.923385	0.819	0.623	1.17E-61	Saliva_Peri	CD63
COL5A31	7.93E-64	0.701042	0.072	0.01	1.26E-59	Saliva_Peri	COL5A3
AEBP11	5.14E-63	1.099993	0.241	0.079	8.2E-59	Saliva_Peri	AEBP1
ATP2B42	1.48E-62	1.251572	0.235	0.079	2.36E-58	Saliva_Peri	ATP2B4
RGS161	2.36E-62	1.459506	0.253	0.087	3.77E-58	Saliva_Peri	RGS16
ARHGAP29	2.71E-62	1.384967	0.289	0.114	4.32E-58	Saliva_Peri	ARHGAP29
LMNA3	4.91E-62	1.072225	0.585	0.365	7.84E-58	Saliva_Peri	LMNA
RBM24	1.6E-61	0.383902	0.055	0.006	2.55E-57	Saliva_Peri	RBM24
BGN1	2.49E-61	0.940644	0.217	0.067	3.98E-57	Saliva_Peri	BGN
KIRREL	1.41E-60	0.45051	0.055	0.006	2.24E-56	Saliva_Peri	KIRREL
HIC11	7.24E-60	1.064982	0.112	0.022	1.16E-55	Saliva_Peri	HIC1
RARRES21	7.81E-60	1.237849	0.249	0.087	1.25E-55	Saliva_Peri	RARRES2
NDUFA42	1.78E-59	1.35669	0.632	0.538	2.84E-55	Saliva_Peri	NDUFA4

ANKRD291	2.66E-59	0.978707	0.129	0.029	4.24E-55	Saliva_Peri	ANKRD29
PYGM	3.25E-58	0.829867	0.065	0.009	5.18E-54	Saliva_Peri	PYGM
ENO3	1.75E-57	2.551368	0.064	0.008	2.8E-53	Saliva_Peri	ENO3
COL6A21	1.07E-56	0.720435	0.378	0.159	1.7E-52	Saliva_Peri	COL6A2
GRK52	4.81E-56	0.731589	0.102	0.02	7.67E-52	Saliva_Peri	GRK5
NT5DC21	6.72E-56	1.070066	0.163	0.046	1.07E-51	Saliva_Peri	NT5DC2
CACNA1H	1.83E-55	0.368512	0.055	0.007	2.92E-51	Saliva_Peri	CACNA1H
CYR611	1.98E-55	1.4114	0.282	0.112	3.16E-51	Saliva_Peri	CYR61
H3F3A4	2.02E-55	0.88431	0.7	0.477	3.22E-51	Saliva_Peri	H3F3A
SPEG	3.52E-55	0.579563	0.082	0.014	5.61E-51	Saliva_Peri	SPEG
LPL	6.36E-55	1.056167	0.08	0.013	1.01E-50	Saliva_Peri	LPL
ID4	8.15E-55	1.346661	0.221	0.077	1.3E-50	Saliva_Peri	ID4
TLN12	4.76E-54	1.3215	0.297	0.129	7.6E-50	Saliva_Peri	TLN1
PTMS2	5.13E-54	1.400672	0.429	0.243	8.17E-50	Saliva_Peri	PTMS
C1orf95	7.46E-54	0.353701	0.035	0.003	1.19E-49	Saliva_Peri	C1orf95
VCL1	9.51E-54	1.363533	0.271	0.112	1.52E-49	Saliva_Peri	VCL
HRH2	1.81E-53	0.440458	0.059	0.008	2.89E-49	Saliva_Peri	HRH2
PDGFA	2.15E-53	0.850986	0.137	0.034	3.43E-49	Saliva_Peri	PDGFA
EBF12	2.8E-53	0.959536	0.194	0.061	4.46E-49	Saliva_Peri	EBF1
NET1	7.46E-53	1.358727	0.27	0.112	1.19E-48	Saliva_Peri	NET1
PGF1	1.2E-52	0.811735	0.123	0.029	1.92E-48	Saliva_Peri	PGF
NTRK31	1.67E-52	0.743392	0.111	0.024	2.66E-48	Saliva_Peri	NTRK3
ITGA11	6.55E-52	0.851094	0.108	0.023	1.04E-47	Saliva_Peri	ITGA1
HGF	1.64E-51	0.645379	0.062	0.009	2.61E-47	Saliva_Peri	HGF
SYNE12	4.31E-51	0.97307	0.181	0.056	6.88E-47	Saliva_Peri	SYNE1
SMARCD3	1.2E-49	0.757449	0.115	0.027	1.91E-45	Saliva_Peri	SMARCD3
UBA2	1.54E-49	1.312659	0.27	0.115	2.46E-45	Saliva_Peri	UBA2
DUSP26	1.6E-49	0.278524	0.035	0.003	2.55E-45	Saliva_Peri	DUSP26
TNNI2	2.55E-49	3.394746	0.065	0.01	4.06E-45	Saliva_Peri	TNNI2
IER5L2	2.7E-49	1.193625	0.239	0.093	4.3E-45	Saliva_Peri	IER5L
TUBA1A2	2.3E-47	1.250382	0.418	0.234	3.67E-43	Saliva_Peri	TUBA1A
MYL2	7.07E-47	1.339156	0.022	0.001	1.13E-42	Saliva_Peri	MYL2
PPP1R12C	1.04E-46	0.972957	0.162	0.05	1.65E-42	Saliva_Peri	PPP1R12C
MTRNR2L8	2.8E-46	1.206645	0.467	0.285	4.47E-42	Saliva_Peri	MTRNR2L8
RP11.54O7	1.21E-45	0.321784	0.029	0.002	1.93E-41	Saliva_Peri	RP11.54O7.17
FBXL22	3.71E-45	0.363628	0.05	0.007	5.91E-41	Saliva_Peri	FBXL22
PDLIM72	2.61E-44	1.034315	0.183	0.063	4.16E-40	Saliva_Peri	PDLIM7
UTRN2	8.06E-44	1.247364	0.26	0.115	1.29E-39	Saliva_Peri	UTRN
HCFC1R11	2.25E-42	1.286116	0.284	0.135	3.6E-38	Saliva_Peri	HCFC1R1
JUN3	7.19E-42	1.520193	0.593	0.487	1.15E-37	Saliva_Peri	JUN
ANK1	8.81E-42	0.417886	0.039	0.004	1.4E-37	Saliva_Peri	ANK1
DES	1.6E-41	2.863983	0.08	0.016	2.56E-37	Saliva_Peri	DES
ADAMTS11	3.19E-40	1.329431	0.173	0.06	5.08E-36	Saliva_Peri	ADAMTS1
TSC22D11	6.11E-40	1.04246	0.425	0.267	9.74E-36	Saliva_Peri	TSC22D1
ADCY5	5.68E-39	0.37545	0.047	0.007	9.05E-35	Saliva_Peri	ADCY5
ACTN2	8.98E-39	0.628883	0.035	0.004	1.43E-34	Saliva_Peri	ACTN2
MT.ND4L5	1.31E-38	0.958286	0.542	0.416	2.09E-34	Saliva_Peri	MT.ND4L

MTRNR2L1	1.38E-38	1.320838	0.274	0.125	2.2E-34	Saliva_Peri	MTRNR2L1
NCKAP5	2.36E-38	0.648966	0.076	0.016	3.77E-34	Saliva_Peri	NCKAP5
TBC1D11	6.16E-38	0.981319	0.163	0.058	9.82E-34	Saliva_Peri	TBC1D1
TNNT31	6.51E-38	3.115829	0.062	0.011	1.04E-33	Saliva_Peri	TNNT3
NDN1	6.74E-38	0.955676	0.172	0.062	1.07E-33	Saliva_Peri	NDN
ATP2A1	1.01E-37	0.670597	0.032	0.003	1.61E-33	Saliva_Peri	ATP2A1
SMIM102	1.08E-37	0.545718	0.087	0.02	1.72E-33	Saliva_Peri	SMIM10
MICU31	1.64E-37	0.743979	0.104	0.027	2.62E-33	Saliva_Peri	MICU3
CRIM11	3.71E-37	1.048865	0.195	0.078	5.92E-33	Saliva_Peri	CRIM1
HEY2	5.81E-37	0.569354	0.089	0.021	9.27E-33	Saliva_Peri	HEY2
PEAR11	7.53E-37	0.558015	0.058	0.01	1.2E-32	Saliva_Peri	PEAR1
MAP3K7CL	1.94E-36	0.518052	0.071	0.014	3.09E-32	Saliva_Peri	MAP3K7CL
SBSPON	2.9E-35	0.542022	0.082	0.019	4.63E-31	Saliva_Peri	SBSPON
TRPC6	4.48E-35	0.279156	0.028	0.003	7.14E-31	Saliva_Peri	TRPC6
CCL8	4.54E-35	1.137359	0.059	0.011	7.23E-31	Saliva_Peri	CCL8
MYO1B	6.52E-35	1.296191	0.18	0.072	1.04E-30	Saliva_Peri	MYO1B
FOSB4	7.57E-35	1.537571	0.393	0.251	1.21E-30	Saliva_Peri	FOSB
ECM21	1.01E-34	0.762052	0.115	0.034	1.6E-30	Saliva_Peri	ECM2
PTK2	7.47E-34	1.16304	0.234	0.112	1.19E-29	Saliva_Peri	PTK2
TTN	2.1E-33	1.140356	0.055	0.01	3.35E-29	Saliva_Peri	TTN
PHLDB2	1.43E-32	0.748687	0.147	0.052	2.29E-28	Saliva_Peri	PHLDB2
ENPEP	1.5E-32	0.469597	0.046	0.007	2.39E-28	Saliva_Peri	ENPEP
TTLL71	3.21E-32	0.947308	0.165	0.064	5.12E-28	Saliva_Peri	TTLL7
SERPING12	8.51E-32	0.970817	0.317	0.172	1.36E-27	Saliva_Peri	SERPING1
SMTN	1.64E-31	0.735066	0.126	0.042	2.61E-27	Saliva_Peri	SMTN
NCS1	1.73E-31	0.326988	0.055	0.011	2.75E-27	Saliva_Peri	NCS1
RPS174	2.25E-31	0.625098	0.382	0.221	3.58E-27	Saliva_Peri	RPS17
FADS3	2.83E-31	0.758342	0.124	0.041	4.51E-27	Saliva_Peri	FADS3
COL27A1	2.88E-31	0.565275	0.066	0.015	4.59E-27	Saliva_Peri	COL27A1
TGFBI1	6.99E-31	0.933197	0.162	0.063	1.11E-26	Saliva_Peri	TGFBI
ESAM1	7.03E-31	0.873265	0.174	0.071	1.12E-26	Saliva_Peri	ESAM
OLFML2A2	1.55E-30	0.607681	0.087	0.023	2.47E-26	Saliva_Peri	OLFML2A
MYLPF	1.98E-30	2.81806	0.065	0.014	3.16E-26	Saliva_Peri	MYLPF
NTN41	2.85E-30	0.916337	0.159	0.062	4.55E-26	Saliva_Peri	NTN4
PKIG2	2.86E-30	1.061871	0.212	0.099	4.56E-26	Saliva_Peri	PKIG
REEP1	3.36E-30	0.38353	0.054	0.01	5.36E-26	Saliva_Peri	REEP1
ROCK12	1.61E-29	1.103526	0.278	0.155	2.56E-25	Saliva_Peri	ROCK1
FRY1	2.42E-29	0.567418	0.079	0.02	3.85E-25	Saliva_Peri	FRY
DMPK	4.39E-29	0.504239	0.079	0.02	7E-25	Saliva_Peri	DMPK
CD1513	5.5E-29	1.221185	0.342	0.22	8.77E-25	Saliva_Peri	CD151
SLC2A4	7.24E-29	0.377396	0.043	0.007	1.16E-24	Saliva_Peri	SLC2A4
NUCKS12	1.1E-28	1.057975	0.404	0.283	1.76E-24	Saliva_Peri	NUCKS1
SORBS1	1.19E-28	0.586689	0.108	0.034	1.9E-24	Saliva_Peri	SORBS1
ADAMTS4	1.28E-28	1.087534	0.104	0.032	2.04E-24	Saliva_Peri	ADAMTS4
DGKH1	1.58E-28	0.757128	0.109	0.035	2.52E-24	Saliva_Peri	DGKH
SNTA1	1.82E-28	0.610501	0.091	0.026	2.9E-24	Saliva_Peri	SNTA1
NDUFB73	3.31E-28	1.123204	0.459	0.373	5.28E-24	Saliva_Peri	NDUFB7

LIMS22	4.81E-28	0.58319	0.102	0.032	7.67E-24	Saliva_Peri	LIMS2
CKMT2	1.58E-27	0.324562	0.043	0.007	2.52E-23	Saliva_Peri	CKMT2
C12orf572	1.61E-27	1.073505	0.456	0.357	2.57E-23	Saliva_Peri	C12orf57
FERMT22	2.87E-27	0.987352	0.181	0.081	4.57E-23	Saliva_Peri	FERMT2
MXRA72	2.96E-27	0.912517	0.185	0.083	4.72E-23	Saliva_Peri	MXRA7
ITGB11	3.24E-27	1.145905	0.45	0.357	5.16E-23	Saliva_Peri	ITGB1
CLMN	3.43E-27	1.029807	0.158	0.066	5.47E-23	Saliva_Peri	CLMN
ARHGAP1C	4.05E-27	1.037026	0.18	0.08	6.46E-23	Saliva_Peri	ARHGAP10
RHOB1	6.83E-27	1.211217	0.32	0.201	1.09E-22	Saliva_Peri	RHOB
RERG	2.26E-26	0.646432	0.082	0.023	3.61E-22	Saliva_Peri	RERG
SSPN1	1.31E-25	0.735444	0.16	0.067	2.09E-21	Saliva_Peri	SSPN
ACKR31	1.65E-25	0.532906	0.091	0.028	2.63E-21	Saliva_Peri	ACKR3
ADCY6	2.73E-25	0.679689	0.083	0.024	4.35E-21	Saliva_Peri	ADCY6
TPPP31	5.58E-25	0.851853	0.22	0.109	8.91E-21	Saliva_Peri	TPPP3
AKAP1	5.65E-25	0.580396	0.167	0.071	9.01E-21	Saliva_Peri	AKAP1
SELENBP1:	8.91E-25	1.068817	0.166	0.075	1.42E-20	Saliva_Peri	SELENBP1
JUND3	1.02E-24	1.037717	0.534	0.464	1.62E-20	Saliva_Peri	JUND
MT.ATP84	1.13E-24	1.009746	0.245	0.135	1.81E-20	Saliva_Peri	MT.ATP8
HSPB6	1.69E-24	0.537884	0.071	0.019	2.7E-20	Saliva_Peri	HSPB6
FRMD3	1.89E-24	0.758858	0.075	0.021	3.01E-20	Saliva_Peri	FRMD3
RBMS32	2.11E-24	0.882602	0.131	0.052	3.37E-20	Saliva_Peri	RBMS3
OLFML2B1	2.97E-24	0.600987	0.09	0.028	4.74E-20	Saliva_Peri	OLFML2B
EVA1B2	3.3E-24	1.022781	0.159	0.07	5.26E-20	Saliva_Peri	EVA1B
EID12	3.52E-24	0.949206	0.455	0.357	5.62E-20	Saliva_Peri	EID1
AGAP2	9.75E-24	0.40222	0.03	0.005	1.56E-19	Saliva_Peri	AGAP2
HEYL	1.77E-23	0.278292	0.037	0.007	2.83E-19	Saliva_Peri	HEYL
NFIB3	2.77E-23	0.922268	0.306	0.191	4.42E-19	Saliva_Peri	NFIB
DDIT4L	2.87E-23	0.460926	0.041	0.008	4.58E-19	Saliva_Peri	DDIT4L
LRRC321	8.1E-23	0.57127	0.098	0.033	1.29E-18	Saliva_Peri	LRRC32
FABP51	1.06E-22	1.284548	0.241	0.135	1.69E-18	Saliva_Peri	FABP5
CPED11	1.16E-22	0.476816	0.072	0.02	1.85E-18	Saliva_Peri	CPED1
CXCL122	1.33E-22	0.743891	0.219	0.109	2.13E-18	Saliva_Peri	CXCL12
COLEC11	1.74E-22	0.384638	0.029	0.004	2.78E-18	Saliva_Peri	COLEC11
FXYD62	1.75E-22	0.787939	0.089	0.029	2.8E-18	Saliva_Peri	FXYD6
SLC25A41	2.07E-22	1.887275	0.21	0.114	3.31E-18	Saliva_Peri	SLC25A4
BTG23	2.79E-22	1.20625	0.339	0.232	4.46E-18	Saliva_Peri	BTG2
MYOM1	2.97E-22	0.400948	0.064	0.017	4.73E-18	Saliva_Peri	MYOM1
CTGF1	4.4E-22	0.97122	0.214	0.112	7.02E-18	Saliva_Peri	CTGF
NEB	1.43E-21	0.678132	0.026	0.004	2.29E-17	Saliva_Peri	NEB
ACTN41	1.8E-21	0.985847	0.398	0.307	2.87E-17	Saliva_Peri	ACTN4
FITM1	2.17E-21	0.408603	0.022	0.003	3.46E-17	Saliva_Peri	FITM1
COX5B	3.16E-21	0.838708	0.573	0.566	5.04E-17	Saliva_Peri	COX5B
CRIP21	3.95E-21	1.020784	0.308	0.204	6.3E-17	Saliva_Peri	CRIP2
XIST4	4.91E-21	1.080864	0.223	0.122	7.83E-17	Saliva_Peri	XIST
RPL52	5.63E-21	0.276273	0.858	0.727	8.99E-17	Saliva_Peri	RPL5
PLCL1	6.95E-21	0.396205	0.041	0.009	1.11E-16	Saliva_Peri	PLCL1
CCL2	1.01E-20	1.815192	0.225	0.124	1.61E-16	Saliva_Peri	CCL2

IGFBP42	1.35E-20	0.765611	0.237	0.13	2.15E-16	Saliva_Peri	IGFBP4
TCF42	2.54E-20	0.95106	0.281	0.173	4.06E-16	Saliva_Peri	TCF4
FHL3	2.6E-20	0.83452	0.104	0.039	4.15E-16	Saliva_Peri	FHL3
MAP7D31	3.25E-20	0.75579	0.141	0.063	5.19E-16	Saliva_Peri	MAP7D3
SH3BGRL1	3.91E-20	0.988277	0.342	0.244	6.23E-16	Saliva_Peri	SH3BGRL
ROCK22	4.05E-20	0.90972	0.183	0.095	6.46E-16	Saliva_Peri	ROCK2
FAM181B	4.47E-20	0.299403	0.029	0.005	7.14E-16	Saliva_Peri	FAM181B
RPL7A3	7.36E-20	0.331063	0.881	0.814	1.17E-15	Saliva_Peri	RPL7A
GPRIN3	8.31E-20	0.470013	0.048	0.012	1.33E-15	Saliva_Peri	GPRIN3
LAMA51	1.26E-19	0.554521	0.083	0.028	2.01E-15	Saliva_Peri	LAMA5
DMD1	1.36E-19	0.787477	0.12	0.051	2.17E-15	Saliva_Peri	DMD
PEBP13	2.77E-19	0.757876	0.519	0.456	4.42E-15	Saliva_Peri	PEBP1
NDUFB103	3.07E-19	0.992675	0.444	0.385	4.9E-15	Saliva_Peri	NDUFB10
MAPRE2	3.49E-19	0.845275	0.159	0.078	5.57E-15	Saliva_Peri	MAPRE2
CBX61	5.5E-19	0.783876	0.167	0.084	8.78E-15	Saliva_Peri	CBX6
SLC25A33	6E-19	0.886464	0.495	0.456	9.57E-15	Saliva_Peri	SLC25A3
PMEPA12	6.72E-19	1.160861	0.207	0.119	1.07E-14	Saliva_Peri	PMEPA1
MBNL1.AS	6.76E-19	0.404056	0.044	0.01	1.08E-14	Saliva_Peri	MBNL1.AS1
ARHGAP42	8.17E-19	0.498709	0.064	0.019	1.3E-14	Saliva_Peri	ARHGAP42
PROCR1	8.33E-19	0.693987	0.086	0.031	1.33E-14	Saliva_Peri	PROCR
SYTL2	1.01E-18	0.730462	0.109	0.045	1.62E-14	Saliva_Peri	SYTL2
PGAM2	1.04E-18	1.322347	0.108	0.043	1.66E-14	Saliva_Peri	PGAM2
MAGED22	1.09E-18	1.021282	0.248	0.155	1.73E-14	Saliva_Peri	MAGED2
FBXL13	1.29E-18	0.524014	0.084	0.03	2.06E-14	Saliva_Peri	FBXL13
SKP11	1.61E-18	0.635088	0.577	0.571	2.57E-14	Saliva_Peri	SKP1
NDUFB84	2E-18	1.094202	0.267	0.178	3.2E-14	Saliva_Peri	NDUFB8
ADAP2	2.07E-18	0.560381	0.057	0.016	3.3E-14	Saliva_Peri	ADAP2
AMIGO21	2.07E-18	0.310984	0.047	0.012	3.3E-14	Saliva_Peri	AMIGO2
PCOLCE1	2.12E-18	0.801929	0.178	0.091	3.38E-14	Saliva_Peri	PCOLCE
PRRT21	3.84E-18	0.438959	0.046	0.011	6.13E-14	Saliva_Peri	PRRT2
KIAA0040	6.1E-18	0.789273	0.127	0.057	9.73E-14	Saliva_Peri	KIAA0040
C9orf31	6.31E-18	0.899792	0.181	0.098	1.01E-13	Saliva_Peri	C9orf3
FAM129A3	6.73E-18	0.795108	0.189	0.104	1.07E-13	Saliva_Peri	FAM129A
VEGFB3	7.85E-18	0.885552	0.183	0.099	1.25E-13	Saliva_Peri	VEGFB
C1QTNF22	1.12E-17	0.453893	0.071	0.023	1.79E-13	Saliva_Peri	C1QTNF2
PPP1R1A	1.85E-17	0.613197	0.05	0.013	2.95E-13	Saliva_Peri	PPP1R1A
PARM11	2.77E-17	0.83381	0.119	0.053	4.41E-13	Saliva_Peri	PARM1
GPAT2	5.49E-17	0.585081	0.055	0.016	8.75E-13	Saliva_Peri	GPAT2
MARVELD1	6.25E-17	0.531113	0.086	0.032	9.96E-13	Saliva_Peri	MARVELD1
CKB	6.76E-17	1.062456	0.326	0.245	1.08E-12	Saliva_Peri	CKB
TMEM74B	7.22E-17	0.327156	0.028	0.005	1.15E-12	Saliva_Peri	TMEM74B
COL6A11	8.24E-17	0.712855	0.239	0.141	1.31E-12	Saliva_Peri	COL6A1
PARK74	9.56E-17	0.772109	0.429	0.356	1.52E-12	Saliva_Peri	PARK7
DTX3	9.91E-17	0.510694	0.064	0.02	1.58E-12	Saliva_Peri	DTX3
CRTC3	1.76E-16	0.741235	0.126	0.059	2.8E-12	Saliva_Peri	CRTC3
HMGB12	1.79E-16	0.691854	0.575	0.576	2.85E-12	Saliva_Peri	HMGB1
ILK1	2.13E-16	0.955141	0.231	0.147	3.4E-12	Saliva_Peri	ILK

LGALSL	2.31E-16	0.749397	0.111	0.049	3.69E-12	Saliva_Peri	LGALSL
MGLL3	3.08E-16	1.152655	0.259	0.177	4.9E-12	Saliva_Peri	MGLL
ESD2	3.61E-16	1.006136	0.299	0.217	5.76E-12	Saliva_Peri	ESD
RNF150	4.37E-16	0.390046	0.054	0.016	6.97E-12	Saliva_Peri	RNF150
STAT32	4.94E-16	1.049777	0.274	0.19	7.88E-12	Saliva_Peri	STAT3
ENDOD12	4.98E-16	0.606287	0.086	0.033	7.95E-12	Saliva_Peri	ENDOD1
MYH91	7.08E-16	1.020198	0.268	0.186	1.13E-11	Saliva_Peri	MYH9
CALM14	8.76E-16	0.546836	0.631	0.652	1.4E-11	Saliva_Peri	CALM1
LUC7L33	1.58E-15	1.013601	0.337	0.266	2.52E-11	Saliva_Peri	LUC7L3
FAM13C1	1.79E-15	0.601312	0.066	0.023	2.86E-11	Saliva_Peri	FAM13C
CCDC102B	2.4E-15	0.617901	0.071	0.025	3.83E-11	Saliva_Peri	CCDC102B
TMOD11	2.42E-15	0.409551	0.047	0.013	3.87E-11	Saliva_Peri	TMOD1
CHRD1	3.3E-15	0.305662	0.035	0.008	5.26E-11	Saliva_Peri	CHRD
APBB21	3.9E-15	0.625858	0.082	0.032	6.22E-11	Saliva_Peri	APBB2
MT.ND64	4.2E-15	0.864541	0.189	0.111	6.7E-11	Saliva_Peri	MT.ND6
RPL302	4.32E-15	0.254558	0.905	0.844	6.88E-11	Saliva_Peri	RPL30
CCNI2	8.34E-15	0.674867	0.528	0.518	1.33E-10	Saliva_Peri	CCNI
SYNE21	1.01E-14	0.910488	0.217	0.137	1.61E-10	Saliva_Peri	SYNE2
SYT11	1.2E-14	0.398667	0.043	0.012	1.92E-10	Saliva_Peri	SYT11
PRELP1	1.73E-14	0.513759	0.095	0.04	2.75E-10	Saliva_Peri	PRELP
PHLDA2	1.95E-14	0.770475	0.29	0.207	3.11E-10	Saliva_Peri	PHLDA2
NFATC41	1.95E-14	0.438866	0.054	0.017	3.12E-10	Saliva_Peri	NFATC4
SLCO3A11	2.24E-14	0.753948	0.113	0.054	3.58E-10	Saliva_Peri	SLCO3A1
RILPL11	3.06E-14	0.381983	0.046	0.013	4.88E-10	Saliva_Peri	RILPL1
SLIT31	4.24E-14	0.756704	0.151	0.081	6.76E-10	Saliva_Peri	SLIT3
ACTN11	4.85E-14	0.897512	0.224	0.148	7.74E-10	Saliva_Peri	ACTN1
PLEKHH31	6.16E-14	0.818037	0.123	0.062	9.82E-10	Saliva_Peri	PLEKHH3
ATP5G32	7.2E-14	0.892087	0.408	0.362	1.15E-09	Saliva_Peri	ATP5G3
KLF23	7.5E-14	1.019166	0.275	0.191	1.2E-09	Saliva_Peri	KLF2
S1PR31	2.12E-13	0.312098	0.04	0.011	3.38E-09	Saliva_Peri	S1PR3
CCR101	2.31E-13	0.367108	0.046	0.014	3.68E-09	Saliva_Peri	CCR10
PTGER1	2.99E-13	0.281663	0.021	0.004	4.78E-09	Saliva_Peri	PTGER1
TNNC2	3.07E-13	3.127669	0.061	0.022	4.9E-09	Saliva_Peri	TNNC2
STEAP4	3.07E-13	1.347634	0.249	0.178	4.9E-09	Saliva_Peri	STEAP4
CBFA2T3	3.23E-13	0.448487	0.058	0.02	5.15E-09	Saliva_Peri	CBFA2T3
SOCS32	3.53E-13	0.95037	0.246	0.165	5.63E-09	Saliva_Peri	SOCS3
SEMA5A1	3.83E-13	0.641164	0.068	0.026	6.11E-09	Saliva_Peri	SEMA5A
SGCE1	4.05E-13	0.54224	0.09	0.039	6.46E-09	Saliva_Peri	SGCE
CYSTM12	4.13E-13	0.888157	0.299	0.226	6.58E-09	Saliva_Peri	CYSTM1
VSTM41	4.61E-13	0.576362	0.072	0.028	7.35E-09	Saliva_Peri	VSTM4
FGF71	8.79E-13	0.738176	0.1	0.046	1.4E-08	Saliva_Peri	FGF7
PLEKHG3	9.21E-13	0.408891	0.065	0.025	1.47E-08	Saliva_Peri	PLEKHG3
ARHGEF7	9.48E-13	0.679042	0.123	0.064	1.51E-08	Saliva_Peri	ARHGEF7
PLCB41	1.07E-12	0.707185	0.112	0.056	1.71E-08	Saliva_Peri	PLCB4
INPP4B	1.39E-12	0.739787	0.113	0.057	2.22E-08	Saliva_Peri	INPP4B
RAB341	1.73E-12	0.726116	0.17	0.102	2.76E-08	Saliva_Peri	RAB34
RHOQ2	1.79E-12	0.819841	0.151	0.086	2.85E-08	Saliva_Peri	RHOQ

AXL1	2.11E-12	0.622308	0.17	0.098	3.36E-08	Saliva_Peri	AXL
RPL242	2.69E-12	0.294565	0.809	0.802	4.29E-08	Saliva_Peri	RPL24
EMILIN11	2.74E-12	0.350068	0.057	0.02	4.36E-08	Saliva_Peri	EMILIN1
RGL3	2.91E-12	0.402158	0.037	0.011	4.64E-08	Saliva_Peri	RGL3
WNT6	2.99E-12	0.27117	0.021	0.004	4.77E-08	Saliva_Peri	WNT6
TXNIP2	3.98E-12	0.810865	0.438	0.377	6.34E-08	Saliva_Peri	TXNIP
TMEM471	5.36E-12	0.607481	0.109	0.055	8.55E-08	Saliva_Peri	TMEM47
PAWR	6.45E-12	1.056318	0.195	0.131	1.03E-07	Saliva_Peri	PAWR
ARHGAP11	7.87E-12	0.71905	0.116	0.06	1.25E-07	Saliva_Peri	ARHGAP1
RDH5	8.24E-12	0.355426	0.041	0.013	1.31E-07	Saliva_Peri	RDH5
LAMB22	1.05E-11	0.768156	0.145	0.083	1.67E-07	Saliva_Peri	LAMB2
NUPR12	1.06E-11	0.54812	0.337	0.26	1.69E-07	Saliva_Peri	NUPR1
CYGB1	1.08E-11	0.672836	0.064	0.025	1.72E-07	Saliva_Peri	CYGB
SORBS31	1.09E-11	0.83338	0.17	0.105	1.73E-07	Saliva_Peri	SORBS3
RUNX1T12	1.37E-11	0.656929	0.087	0.04	2.18E-07	Saliva_Peri	RUNX1T1
SOGA12	1.53E-11	0.573171	0.059	0.023	2.44E-07	Saliva_Peri	SOGA1
RP11.109G	2.65E-11	0.278475	0.033	0.009	4.23E-07	Saliva_Peri	RP11.109G23.3
LMO43	2.89E-11	0.937176	0.23	0.165	4.6E-07	Saliva_Peri	LMO4
DDR21	3E-11	0.453054	0.093	0.044	4.79E-07	Saliva_Peri	DDR2
RN7SL832I	3.03E-11	0.29357	0.026	0.006	4.83E-07	Saliva_Peri	RN7SL832P
MYADM2	4.38E-11	0.889049	0.219	0.15	6.99E-07	Saliva_Peri	MYADM
UQCRB	4.84E-11	0.735543	0.534	0.558	7.71E-07	Saliva_Peri	UQCRB
MAT2A	5.4E-11	0.953231	0.214	0.15	8.62E-07	Saliva_Peri	MAT2A
SDPR1	5.49E-11	0.571644	0.065	0.027	8.76E-07	Saliva_Peri	SDPR
ANXA52	5.51E-11	0.782538	0.375	0.318	8.79E-07	Saliva_Peri	ANXA5
MAGI2	6.96E-11	0.346272	0.043	0.014	1.11E-06	Saliva_Peri	MAGI2
CCDC1072	6.97E-11	0.783354	0.243	0.176	1.11E-06	Saliva_Peri	CCDC107
RPL223	8.37E-11	0.362248	0.692	0.694	1.34E-06	Saliva_Peri	RPL22
SGCA	1E-10	0.548168	0.1	0.049	1.6E-06	Saliva_Peri	SGCA
TUBA1B2	1.05E-10	0.709276	0.411	0.354	1.67E-06	Saliva_Peri	TUBA1B
PDE1A	1.39E-10	0.568183	0.083	0.039	2.21E-06	Saliva_Peri	PDE1A
N4BP2L23	1.42E-10	0.926011	0.339	0.293	2.26E-06	Saliva_Peri	N4BP2L2
COBLL12	1.89E-10	0.846609	0.158	0.099	3.02E-06	Saliva_Peri	COBLL1
HSPA1A2	1.96E-10	0.508036	0.4	0.326	3.13E-06	Saliva_Peri	HSPA1A
TACC12	1.97E-10	0.773731	0.231	0.167	3.13E-06	Saliva_Peri	TACC1
C3orf70	1.97E-10	0.341982	0.041	0.014	3.14E-06	Saliva_Peri	C3orf70
PSMB13	2.02E-10	0.817098	0.416	0.4	3.23E-06	Saliva_Peri	PSMB1
KLF92	2.34E-10	0.85869	0.167	0.106	3.73E-06	Saliva_Peri	KLF9
CD2481	3.21E-10	0.533787	0.079	0.036	5.12E-06	Saliva_Peri	CD248
C20orf27	3.45E-10	0.817375	0.123	0.069	5.49E-06	Saliva_Peri	C20orf27
ZBTB7C	3.48E-10	0.303547	0.047	0.017	5.56E-06	Saliva_Peri	ZBTB7C
CACNA1C	4.04E-10	0.358771	0.054	0.021	6.44E-06	Saliva_Peri	CACNA1C
PTPRG2	4.16E-10	0.640936	0.08	0.038	6.63E-06	Saliva_Peri	PTPRG
SLC8A11	4.69E-10	0.308819	0.043	0.015	7.48E-06	Saliva_Peri	SLC8A1
DKK32	5.27E-10	0.557652	0.089	0.044	8.4E-06	Saliva_Peri	DKK3
MKL21	5.46E-10	0.685982	0.094	0.048	8.7E-06	Saliva_Peri	MKL2
DDX173	6.54E-10	0.874803	0.378	0.354	1.04E-05	Saliva_Peri	DDX17

ZNF7031	9.49E-10	0.445619	0.089	0.044	1.51E-05	Saliva_Peri	ZNF703
LTBP11	1.02E-09	0.465524	0.101	0.053	1.63E-05	Saliva_Peri	LTBP1
AKAP6	1.34E-09	0.346057	0.048	0.018	2.13E-05	Saliva_Peri	AKAP6
STOM2	1.35E-09	0.88093	0.213	0.153	2.16E-05	Saliva_Peri	STOM
ATF31	1.55E-09	1.179659	0.296	0.245	2.48E-05	Saliva_Peri	ATF3
NRP12	1.58E-09	0.585056	0.105	0.056	2.53E-05	Saliva_Peri	NRP1
ITGA31	1.61E-09	0.454628	0.071	0.032	2.56E-05	Saliva_Peri	ITGA3
GAS62	1.67E-09	0.894829	0.151	0.095	2.67E-05	Saliva_Peri	GAS6
CRISPLD21	1.79E-09	0.509221	0.107	0.057	2.86E-05	Saliva_Peri	CRISPLD2
FAM150B	2.17E-09	0.286809	0.046	0.017	3.46E-05	Saliva_Peri	FAM150B
RP11.490N	2.63E-09	0.301639	0.029	0.009	4.19E-05	Saliva_Peri	RP11.490M8.1
PHLDB11	2.65E-09	0.678948	0.093	0.048	4.22E-05	Saliva_Peri	PHLDB1
EPB41L21	3.31E-09	0.902082	0.145	0.092	5.29E-05	Saliva_Peri	EPB41L2
RNF152	3.83E-09	0.631478	0.075	0.036	6.11E-05	Saliva_Peri	RNF152
MPDZ2	4.51E-09	0.461755	0.066	0.03	7.19E-05	Saliva_Peri	MPDZ
DTNA	5.3E-09	0.307	0.046	0.018	8.45E-05	Saliva_Peri	DTNA
SGIP1	6.49E-09	0.301025	0.025	0.007	0.000104	Saliva_Peri	SGIP1
PALLD1	8.4E-09	0.804605	0.214	0.16	0.000134	Saliva_Peri	PALLD
ATP5J3	8.74E-09	0.708518	0.436	0.437	0.000139	Saliva_Peri	ATP5J
INTS62	1.02E-08	0.960461	0.191	0.137	0.000162	Saliva_Peri	INTS6
KCNMA1	1.03E-08	0.447505	0.08	0.041	0.000165	Saliva_Peri	KCNMA1
SYP	1.5E-08	0.288939	0.028	0.008	0.00024	Saliva_Peri	SYP
CCND11	1.56E-08	0.727162	0.173	0.119	0.000249	Saliva_Peri	CCND1
IFITM21	1.67E-08	0.370293	0.382	0.305	0.000266	Saliva_Peri	IFITM2
CC2D2A1	2.1E-08	0.302043	0.041	0.016	0.000335	Saliva_Peri	CC2D2A
EDNRB2	2.2E-08	0.404821	0.057	0.025	0.000351	Saliva_Peri	EDNRB
DIXDC11	2.7E-08	0.49579	0.087	0.046	0.000431	Saliva_Peri	DIXDC1
COX6C	2.73E-08	0.580452	0.516	0.542	0.000436	Saliva_Peri	COX6C
ARHGAP17	2.76E-08	0.458554	0.073	0.036	0.00044	Saliva_Peri	ARHGAP17
TNFSF122	3.06E-08	0.587516	0.105	0.06	0.000488	Saliva_Peri	TNFSF12
ADARB11	3.73E-08	0.288505	0.053	0.023	0.000596	Saliva_Peri	ADARB1
ITPR11	3.76E-08	0.427163	0.064	0.03	0.0006	Saliva_Peri	ITPR1
DYNLL13	4.4E-08	0.392027	0.562	0.561	0.000702	Saliva_Peri	DYNLL1
MB	5.79E-08	3.207308	0.069	0.034	0.000923	Saliva_Peri	MB
GSTP12	5.82E-08	0.263159	0.585	0.557	0.000928	Saliva_Peri	GSTP1
THY11	6.59E-08	0.71917	0.093	0.051	0.001051	Saliva_Peri	THY1
LRRC171	7.43E-08	0.360561	0.041	0.016	0.001185	Saliva_Peri	LRRC17
GPM6B	9.86E-08	0.740379	0.08	0.043	0.001572	Saliva_Peri	GPM6B
PDE4C	1.16E-07	0.310812	0.039	0.015	0.001856	Saliva_Peri	PDE4C
PTEN	1.19E-07	0.847803	0.177	0.128	0.001901	Saliva_Peri	PTEN
ST51	1.27E-07	0.655313	0.102	0.06	0.002029	Saliva_Peri	ST5
ARPC1A	1.48E-07	0.909506	0.254	0.216	0.002367	Saliva_Peri	ARPC1A
APOLD11	1.51E-07	0.581402	0.064	0.031	0.002412	Saliva_Peri	APOLD1
COX14	1.52E-07	0.872454	0.271	0.232	0.002427	Saliva_Peri	COX14
SYNE33	1.54E-07	0.300577	0.044	0.018	0.00246	Saliva_Peri	SYNE3
SLC5A3	2.36E-07	0.470178	0.075	0.039	0.003765	Saliva_Peri	SLC5A3
BTF31	2.51E-07	0.319201	0.671	0.712	0.004	Saliva_Peri	BTF3

RP11.983P	2.55E-07	0.400948	0.061	0.029	0.004062	Saliva_Peri	RP11.983P16.4
NRARP1	2.92E-07	0.455726	0.076	0.04	0.00466	Saliva_Peri	NRARP
EPN21	2.93E-07	0.698372	0.112	0.07	0.004675	Saliva_Peri	EPN2
SSB2	4.42E-07	0.725232	0.243	0.198	0.007051	Saliva_Peri	SSB
SRSF73	4.85E-07	0.842339	0.36	0.343	0.007733	Saliva_Peri	SRSF7
TMEM38A	6.24E-07	0.374854	0.025	0.008	0.009954	Saliva_Peri	TMEM38A
EEF1A2	6.54E-07	0.609468	0.033	0.013	0.010434	Saliva_Peri	EEF1A2
STARD131	7.12E-07	0.358435	0.044	0.019	0.011364	Saliva_Peri	STARD13
C10orf101	7.53E-07	0.666024	0.112	0.07	0.012011	Saliva_Peri	C10orf10
GLRX5	8E-07	0.893514	0.213	0.171	0.012754	Saliva_Peri	GLRX5
CA3	8.05E-07	0.476484	0.022	0.007	0.012832	Saliva_Peri	CA3
ENTPD3	9.41E-07	0.430425	0.066	0.035	0.015016	Saliva_Peri	ENTPD3
SHPRH2	9.55E-07	0.71199	0.111	0.07	0.015239	Saliva_Peri	SHPRH
RASGRP2	1E-06	0.297858	0.107	0.063	0.015988	Saliva_Peri	RASGRP2
MAP91	1.03E-06	0.468156	0.073	0.04	0.016503	Saliva_Peri	MAP9
IDH21	1.13E-06	0.446024	0.107	0.195	0.017948	Saliva_Peri	IDH2
IER23	1.15E-06	0.716714	0.509	0.547	0.018288	Saliva_Peri	IER2
COX7B	1.15E-06	0.816494	0.4	0.41	0.018376	Saliva_Peri	COX7B
BOD1L13	1.23E-06	0.724221	0.173	0.128	0.019559	Saliva_Peri	BOD1L1
SMAP1	1.31E-06	0.256283	0.086	0.164	0.020836	Saliva_Peri	SMAP1
ST6GALNA	1.4E-06	0.523946	0.112	0.071	0.02234	Saliva_Peri	ST6GALNAC6
TNNI1	1.45E-06	0.611165	0.008	0.001	0.023167	Saliva_Peri	TNNI1
YME1L12	1.53E-06	0.266535	0.113	0.203	0.024457	Saliva_Peri	YME1L1
CYB5R32	1.77E-06	0.820171	0.259	0.222	0.028281	Saliva_Peri	CYB5R3
MAML21	1.92E-06	0.608147	0.086	0.05	0.03063	Saliva_Peri	MAML2
SIVA12	2.38E-06	0.692269	0.304	0.274	0.037951	Saliva_Peri	SIVA1
PKD21	2.46E-06	0.383964	0.053	0.026	0.039229	Saliva_Peri	PKD2
CHURC12	2.53E-06	0.835615	0.25	0.217	0.040397	Saliva_Peri	CHURC1
ENTPD12	2.54E-06	0.324412	0.082	0.046	0.040546	Saliva_Peri	ENTPD1
VIPR1	2.81E-06	0.50898	0.044	0.02	0.044782	Saliva_Peri	VIPR1
ENAH1	3.35E-06	0.734093	0.16	0.118	0.053378	Saliva_Peri	ENAH
MTHFD2	3.63E-06	0.661531	0.141	0.099	0.057852	Saliva_Peri	MTHFD2
PSIP12	3.65E-06	0.673616	0.169	0.124	0.058289	Saliva_Peri	PSIP1
GLT8D21	3.81E-06	0.398524	0.051	0.025	0.060743	Saliva_Peri	GLT8D2
NID2	3.94E-06	0.255064	0.022	0.007	0.062887	Saliva_Peri	NID2
RP13.188A	4.37E-06	0.270228	0.025	0.009	0.069768	Saliva_Peri	RP13.188A5.1
OAZ2	4.41E-06	0.846272	0.214	0.178	0.070374	Saliva_Peri	OAZ2
PCMTD11	4.99E-06	0.261521	0.101	0.181	0.079513	Saliva_Peri	PCMTD1
SH2D3C1	5.22E-06	0.377128	0.036	0.015	0.083316	Saliva_Peri	SH2D3C
INPP5A	5.79E-06	0.357281	0.069	0.038	0.092412	Saliva_Peri	INPP5A
SETBP11	6.23E-06	0.401587	0.066	0.036	0.099306	Saliva_Peri	SETBP1
ANXA6	6.66E-06	0.481265	0.154	0.108	0.106177	Saliva_Peri	ANXA6
PSMG22	7.06E-06	0.285727	0.136	0.231	0.112672	Saliva_Peri	PSMG2
PTS	7.1E-06	0.290284	0.072	0.138	0.11321	Saliva_Peri	PTS
PCSK7	8.58E-06	0.619161	0.154	0.112	0.136914	Saliva_Peri	PCSK7
NME33	8.78E-06	0.762718	0.243	0.209	0.140088	Saliva_Peri	NME3
ENPP11	8.84E-06	0.446542	0.037	0.017	0.141065	Saliva_Peri	ENPP1

LEPROT2	1E-05	0.841709	0.278	0.257	0.159967	Saliva_Peri	LEPROT
MOB2	1.14E-05	0.650679	0.177	0.137	0.181327	Saliva_Peri	MOB2
PNISR3	1.54E-05	0.791062	0.351	0.348	0.245092	Saliva_Peri	PNISR
RASAL21	1.57E-05	0.635015	0.13	0.092	0.2504	Saliva_Peri	RASAL2
TOX41	1.59E-05	0.263628	0.094	0.167	0.253672	Saliva_Peri	TOX4
SRRM1	1.7E-05	0.252653	0.207	0.329	0.271482	Saliva_Peri	SRRM1
COX7C	1.78E-05	0.443176	0.581	0.638	0.28353	Saliva_Peri	COX7C
RABGAP1	1.83E-05	0.690398	0.116	0.08	0.291321	Saliva_Peri	RABGAP1
C1orf541	2.04E-05	0.488372	0.079	0.048	0.325012	Saliva_Peri	C1orf54
SCARB23	2.06E-05	0.307267	0.089	0.158	0.328555	Saliva_Peri	SCARB2
METRN2	2.12E-05	0.651027	0.13	0.093	0.33852	Saliva_Peri	METRN
FADS1	2.25E-05	0.443484	0.053	0.028	0.359156	Saliva_Peri	FADS1
COPA2	2.33E-05	0.261002	0.082	0.147	0.372337	Saliva_Peri	COPA
NID11	2.36E-05	0.448796	0.053	0.028	0.376376	Saliva_Peri	NID1
ARID5B1	2.38E-05	0.565199	0.302	0.269	0.378852	Saliva_Peri	ARID5B
SEC31A2	2.41E-05	0.256971	0.131	0.218	0.384646	Saliva_Peri	SEC31A
ARL31	2.48E-05	0.676946	0.152	0.115	0.395205	Saliva_Peri	ARL3
NRBP2	2.51E-05	0.442312	0.051	0.027	0.399838	Saliva_Peri	NRBP2
HTRA11	2.73E-05	0.420516	0.155	0.112	0.434648	Saliva_Peri	HTRA1
UQCRFS1	2.84E-05	0.328157	0.169	0.272	0.452674	Saliva_Peri	UQCRFS1
NFIC3	2.92E-05	0.689748	0.207	0.172	0.465236	Saliva_Peri	NFIC
ALDH21	2.93E-05	0.642199	0.16	0.124	0.467527	Saliva_Peri	ALDH2
FAM20C1	2.96E-05	0.359033	0.054	0.029	0.471587	Saliva_Peri	FAM20C
DST1	3.08E-05	0.459754	0.224	0.184	0.490903	Saliva_Peri	DST
TMEM50B	3.15E-05	0.258601	0.087	0.154	0.501694	Saliva_Peri	TMEM50B
DYRK2	3.22E-05	0.363233	0.064	0.036	0.514336	Saliva_Peri	DYRK2
ASPH2	3.25E-05	0.298842	0.113	0.192	0.518026	Saliva_Peri	ASPH
GNB42	3.31E-05	0.470405	0.068	0.04	0.528107	Saliva_Peri	GNB4
SRP142	3.81E-05	0.286285	0.636	0.702	0.607192	Saliva_Peri	SRP14
CWC15	4.01E-05	0.255087	0.129	0.209	0.639794	Saliva_Peri	CWC15
LAMA3	4.12E-05	0.31656	0.039	0.019	0.656626	Saliva_Peri	LAMA3
RABAC14	4.15E-05	0.515766	0.407	0.405	0.662585	Saliva_Peri	RABAC1
FRMD4A1	4.29E-05	0.487872	0.082	0.051	0.683538	Saliva_Peri	FRMD4A
ENY22	4.69E-05	0.260884	0.206	0.323	0.748076	Saliva_Peri	ENY2
MLF2	4.7E-05	0.346879	0.138	0.226	0.749639	Saliva_Peri	MLF2
SPPL2A1	4.7E-05	0.257424	0.072	0.131	0.750242	Saliva_Peri	SPPL2A
TCF7L12	4.74E-05	0.325272	0.055	0.03	0.75577	Saliva_Peri	TCF7L1
FUS2	5.03E-05	0.742156	0.295	0.277	0.801517	Saliva_Peri	FUS
SERINC12	5.23E-05	0.273137	0.12	0.198	0.833713	Saliva_Peri	SERINC1
YBX32	5.32E-05	0.935597	0.472	0.496	0.84806	Saliva_Peri	YBX3
GABBR1	5.56E-05	0.276685	0.04	0.02	0.887234	Saliva_Peri	GABBR1
TNNC1	5.93E-05	0.703091	0.021	0.008	0.946122	Saliva_Peri	TNNC1
CTDSP21	6.21E-05	0.255549	0.101	0.17	0.991055	Saliva_Peri	CTDSP2
CAMK12	6.38E-05	0.346203	0.061	0.035	1	Saliva_Peri	CAMK1
DDX243	7E-05	0.684039	0.266	0.238	1	Saliva_Peri	DDX24
RBBP41	7.13E-05	0.261411	0.087	0.15	1	Saliva_Peri	RBBP4
CBX7	7.19E-05	0.307972	0.061	0.035	1	Saliva_Peri	CBX7

VMP1	7.31E-05	0.333363	0.202	0.312	1	Saliva_Peri	VMP1
CH25H	7.36E-05	0.325915	0.028	0.012	1	Saliva_Peri	CH25H
DDX52	7.68E-05	0.458192	0.55	0.603	1	Saliva_Peri	DDX5
CPNE31	7.74E-05	0.290216	0.118	0.194	1	Saliva_Peri	CPNE3
RGS6	7.8E-05	0.266178	0.044	0.023	1	Saliva_Peri	RGS6
TSPYL1	8.09E-05	0.271208	0.091	0.156	1	Saliva_Peri	TSPYL1
POLR2J33	8.5E-05	0.647433	0.131	0.097	1	Saliva_Peri	POLR2J3
UNC501	9.32E-05	0.282	0.069	0.125	1	Saliva_Peri	UNC50
KIF5B	9.5E-05	0.30132	0.187	0.292	1	Saliva_Peri	KIF5B
SSBP13	0.000106	0.706581	0.296	0.283	1	Saliva_Peri	SSBP1
PLXND11	0.000114	0.446005	0.054	0.03	1	Saliva_Peri	PLXND1
RNASEH2C	0.000132	0.726744	0.214	0.187	1	Saliva_Peri	RNASEH2C
ZNF641	0.000134	0.32409	0.036	0.018	1	Saliva_Peri	ZNF641
C11orf95	0.000137	0.285378	0.03	0.014	1	Saliva_Peri	C11orf95
TWIST11	0.000138	0.322313	0.076	0.047	1	Saliva_Peri	TWIST1
LCLAT1	0.000145	0.281913	0.044	0.023	1	Saliva_Peri	LCLAT1
CTDSPL2	0.000147	0.654847	0.116	0.085	1	Saliva_Peri	CTDSPL
RNF1152	0.000149	0.613759	0.14	0.107	1	Saliva_Peri	RNF115
SFPQ2	0.00017	0.314932	0.188	0.287	1	Saliva_Peri	SFPQ
PDAP1	0.000171	0.269961	0.14	0.219	1	Saliva_Peri	PDAP1
SVIL1	0.000172	0.673001	0.152	0.12	1	Saliva_Peri	SVIL
CHD93	0.000186	0.828024	0.195	0.167	1	Saliva_Peri	CHD9
RNF180	0.000191	0.376383	0.044	0.024	1	Saliva_Peri	RNF180
YIPF61	0.000194	0.297128	0.087	0.147	1	Saliva_Peri	YIPF6
EHBP1L11	0.000195	0.399251	0.083	0.054	1	Saliva_Peri	EHBP1L1
MSRB21	0.000199	0.685712	0.16	0.13	1	Saliva_Peri	MSRB2
CFL2	0.000212	0.806579	0.129	0.097	1	Saliva_Peri	CFL2
C8orf43	0.000224	0.827868	0.152	0.12	1	Saliva_Peri	C8orf4
RAB12	0.000238	0.293704	0.037	0.019	1	Saliva_Peri	RAB12
STAC3	0.000246	0.433253	0.024	0.01	1	Saliva_Peri	STAC3
GALNT161	0.000255	0.311511	0.043	0.023	1	Saliva_Peri	GALNT16
PRPF4B	0.00026	0.262596	0.1	0.162	1	Saliva_Peri	PRPF4B
SERPINB6	0.000261	0.285904	0.145	0.225	1	Saliva_Peri	SERPINB6
SOD11	0.000268	0.520361	0.408	0.416	1	Saliva_Peri	SOD1
ZFP36L1	0.000279	0.300351	0.476	0.469	1	Saliva_Peri	ZFP36L1
SSBP42	0.000279	0.540166	0.136	0.102	1	Saliva_Peri	SSBP4
ECHS1	0.000292	0.281619	0.13	0.205	1	Saliva_Peri	ECHS1
AK1	0.000303	1.006086	0.172	0.142	1	Saliva_Peri	AK1
EPHX12	0.000321	0.967584	0.151	0.122	1	Saliva_Peri	EPHX1
FAM127A1	0.000322	0.581139	0.122	0.091	1	Saliva_Peri	FAM127A
KDSR1	0.000323	0.279949	0.077	0.131	1	Saliva_Peri	KDSR
PUM1	0.000357	0.289936	0.087	0.144	1	Saliva_Peri	PUM1
CAMK2G	0.000365	0.408179	0.082	0.054	1	Saliva_Peri	CAMK2G
MME1	0.000381	0.3239	0.026	0.012	1	Saliva_Peri	MME
SMIM71	0.000383	0.30676	0.115	0.182	1	Saliva_Peri	SMIM7
RP11.326I1	0.000386	0.275723	0.029	0.014	1	Saliva_Peri	RP11.326I11.3
ATP5G12	0.000393	0.72295	0.274	0.261	1	Saliva_Peri	ATP5G1

DNAJC71	0.000401	0.268338	0.1	0.161	1	Saliva_Peri	DNAJC7
ZEB12	0.000413	0.452061	0.087	0.059	1	Saliva_Peri	ZEB1
MLEC3	0.000416	0.317451	0.12	0.19	1	Saliva_Peri	MLEC
CHD4	0.000416	0.292751	0.1	0.16	1	Saliva_Peri	CHD4
IER3IP11	0.000422	0.321866	0.087	0.144	1	Saliva_Peri	IER3IP1
NDUFAB12	0.000433	0.78474	0.257	0.247	1	Saliva_Peri	NDUFAB1
NDUFS2	0.000435	0.26056	0.08	0.133	1	Saliva_Peri	NDUFS2
TCEAL71	0.000448	0.266487	0.029	0.014	1	Saliva_Peri	TCEAL7
CYTH31	0.000455	0.428595	0.05	0.029	1	Saliva_Peri	CYTH3
RRAGA2	0.00046	0.714712	0.158	0.129	1	Saliva_Peri	RRAGA
HSPD11	0.000469	0.262285	0.173	0.259	1	Saliva_Peri	HSPD1
LZTS21	0.000492	0.439863	0.066	0.042	1	Saliva_Peri	LZTS2
RBPMS	0.00051	0.79998	0.205	0.184	1	Saliva_Peri	RBPMS
KLHDC8B1	0.000516	0.428682	0.072	0.047	1	Saliva_Peri	KLHDC8B
PFKM	0.000546	0.439739	0.054	0.033	1	Saliva_Peri	PFKM
PSMB21	0.000579	0.316881	0.134	0.207	1	Saliva_Peri	PSMB2
SGCB1	0.000588	0.562195	0.095	0.068	1	Saliva_Peri	SGCB
SF3B2	0.000596	0.29861	0.151	0.228	1	Saliva_Peri	SF3B2
HNRNPAB2	0.000599	0.265248	0.107	0.167	1	Saliva_Peri	HNRNPAB
TSEN34	0.000656	0.258106	0.08	0.132	1	Saliva_Peri	TSEN34
MYBPC11	0.000659	0.599744	0.036	0.019	1	Saliva_Peri	MYBPC1
PMP222	0.000665	0.581659	0.172	0.139	1	Saliva_Peri	PMP22
MAP2K22	0.000686	0.295941	0.13	0.199	1	Saliva_Peri	MAP2K2
EMC71	0.000785	0.335608	0.13	0.2	1	Saliva_Peri	EMC7
MRPS18C2	0.00079	0.258757	0.123	0.19	1	Saliva_Peri	MRPS18C
AHI11	0.000818	0.276169	0.068	0.113	1	Saliva_Peri	AHI1
ZNF37A3	0.00082	0.460369	0.082	0.056	1	Saliva_Peri	ZNF37A
CCDC471	0.00084	0.351766	0.087	0.14	1	Saliva_Peri	CCDC47
ADI13	0.000849	0.302535	0.147	0.223	1	Saliva_Peri	ADI1
NT5C3B	0.00087	0.609288	0.123	0.096	1	Saliva_Peri	NT5C3B
ECI12	0.000872	0.646951	0.165	0.139	1	Saliva_Peri	ECI1
ZYX	0.000889	0.577062	0.145	0.117	1	Saliva_Peri	ZYX
LAPTM4B	0.000892	0.307606	0.059	0.102	1	Saliva_Peri	LAPTM4B
POLD2	0.000893	0.292315	0.075	0.123	1	Saliva_Peri	POLD2
NDUFB5	0.000893	0.376563	0.162	0.246	1	Saliva_Peri	NDUFB5
ARPC5	0.000969	0.636234	0.382	0.414	1	Saliva_Peri	ARPC5
LIMA12	0.001017	0.390443	0.119	0.183	1	Saliva_Peri	LIMA1
SPAG91	0.001022	0.271247	0.127	0.194	1	Saliva_Peri	SPAG9
RGMA1	0.001035	0.340461	0.046	0.027	1	Saliva_Peri	RGMA
KMT2C1	0.001052	0.302669	0.098	0.155	1	Saliva_Peri	KMT2C
NDUFS51	0.001065	0.576058	0.416	0.456	1	Saliva_Peri	NDUFS5
DHX291	0.001096	0.29668	0.082	0.132	1	Saliva_Peri	DHX29
KLHL421	0.001114	0.349933	0.058	0.037	1	Saliva_Peri	KLHL42
SNW1	0.001123	0.259419	0.091	0.144	1	Saliva_Peri	SNW1
MRPS36	0.001153	0.396084	0.123	0.189	1	Saliva_Peri	MRPS36
PJA23	0.001223	0.670232	0.176	0.151	1	Saliva_Peri	PJA2
AKT32	0.001237	0.34666	0.061	0.039	1	Saliva_Peri	AKT3

SCAF112	0.001301	0.273405	0.156	0.233	1	Saliva_Peri	SCAF11
WDR61	0.001344	0.304704	0.082	0.13	1	Saliva_Peri	WDR61
PHPT13	0.001352	0.624661	0.321	0.325	1	Saliva_Peri	PHPT1
ZNF445	0.001366	0.315146	0.033	0.018	1	Saliva_Peri	ZNF445
KHDRBS1	0.001374	0.274082	0.145	0.217	1	Saliva_Peri	KHDRBS1
SNRPE	0.001376	0.276743	0.183	0.268	1	Saliva_Peri	SNRPE
C7orf501	0.001439	0.633216	0.16	0.135	1	Saliva_Peri	C7orf50
EAPP2	0.001484	0.308319	0.122	0.185	1	Saliva_Peri	EAPP
SLC25A11	0.001561	0.266521	0.071	0.114	1	Saliva_Peri	SLC25A11
SSBP21	0.001597	0.54778	0.097	0.072	1	Saliva_Peri	SSBP2
BIN1	0.001609	0.28012	0.075	0.118	1	Saliva_Peri	BIN1
LRPAP14	0.001689	0.280156	0.187	0.27	1	Saliva_Peri	LRPAP1
PSMD131	0.001695	0.299212	0.105	0.16	1	Saliva_Peri	PSMD13
ASAH11	0.001732	0.461947	0.187	0.28	1	Saliva_Peri	ASAH1
MSX12	0.001751	0.474119	0.058	0.037	1	Saliva_Peri	MSX1
IFT81	0.001826	0.386559	0.061	0.04	1	Saliva_Peri	IFT81
CNOT4	0.001861	0.255261	0.077	0.123	1	Saliva_Peri	CNOT4
TMED42	0.002005	0.262637	0.116	0.174	1	Saliva_Peri	TMED4
USP8	0.002075	0.351845	0.093	0.144	1	Saliva_Peri	USP8
DYNC1I2	0.002099	0.336839	0.142	0.212	1	Saliva_Peri	DYNC1I2
SYDE1	0.002243	0.259223	0.029	0.015	1	Saliva_Peri	SYDE1
ARMCX31	0.002311	0.313913	0.095	0.146	1	Saliva_Peri	ARMCX3
KIF1B	0.002369	0.307585	0.071	0.113	1	Saliva_Peri	KIF1B
CENPC2	0.002453	0.478289	0.086	0.062	1	Saliva_Peri	CENPC
TRAF5	0.002506	0.307969	0.059	0.039	1	Saliva_Peri	TRAF5
ANTXR11	0.002589	0.321995	0.072	0.05	1	Saliva_Peri	ANTXR1
DESI21	0.002617	0.259051	0.062	0.101	1	Saliva_Peri	DESI2
TCEA11	0.002939	0.313193	0.166	0.241	1	Saliva_Peri	TCEA1
FBXO3	0.002967	0.259836	0.064	0.103	1	Saliva_Peri	FBXO3
COX7A21	0.003117	0.261491	0.512	0.572	1	Saliva_Peri	COX7A2
PSMA1	0.003132	0.326609	0.174	0.255	1	Saliva_Peri	PSMA1
DCBLD1	0.003171	0.442031	0.037	0.022	1	Saliva_Peri	DCBLD1
THRA1	0.003243	0.350713	0.061	0.041	1	Saliva_Peri	THRA
SOX131	0.003247	0.3582	0.044	0.027	1	Saliva_Peri	SOX13
INF21	0.00332	0.371306	0.069	0.048	1	Saliva_Peri	INF2
TMEM126/	0.00333	0.284052	0.086	0.132	1	Saliva_Peri	TMEM126A
NKTR3	0.003403	0.32729	0.12	0.177	1	Saliva_Peri	NKTR
PSMA51	0.003418	0.306968	0.124	0.184	1	Saliva_Peri	PSMA5
TRAPPC4	0.003505	0.265668	0.087	0.133	1	Saliva_Peri	TRAPPC4
ADH51	0.003765	0.678162	0.158	0.136	1	Saliva_Peri	ADH5
RELA	0.003781	0.273752	0.068	0.107	1	Saliva_Peri	RELA
TGOLN23	0.003892	0.285706	0.118	0.173	1	Saliva_Peri	TGOLN2
MDM41	0.003942	0.325976	0.091	0.138	1	Saliva_Peri	MDM4
C1R1	0.00399	0.513454	0.138	0.111	1	Saliva_Peri	C1R
MPHOSPH	0.00401	0.313951	0.18	0.255	1	Saliva_Peri	MPHOSPH8
MAP2	0.004069	0.35228	0.048	0.031	1	Saliva_Peri	MAP2
PHAX1	0.004097	0.281854	0.062	0.099	1	Saliva_Peri	PHAX

RBBP6	0.004128	0.252621	0.115	0.168	1	Saliva_Peri	RBBP6
HDDC2	0.00422	0.313803	0.105	0.156	1	Saliva_Peri	HDDC2
CISD1	0.004256	0.341013	0.091	0.138	1	Saliva_Peri	CISD1
ZKSCAN12	0.004357	0.284226	0.076	0.117	1	Saliva_Peri	ZKSCAN1
ZNF24	0.004392	0.303444	0.093	0.14	1	Saliva_Peri	ZNF24
ITPK1	0.004417	0.298254	0.047	0.03	1	Saliva_Peri	ITPK1
RBM5	0.004473	0.327617	0.086	0.13	1	Saliva_Peri	RBM5
MBNL21	0.004598	0.298123	0.077	0.119	1	Saliva_Peri	MBNL2
SERTAD11	0.004669	0.75171	0.239	0.231	1	Saliva_Peri	SERTAD1
BSDC1	0.004765	0.278625	0.075	0.115	1	Saliva_Peri	BSDC1
MRPL13	0.004782	0.32736	0.084	0.129	1	Saliva_Peri	MRPL13
BCL7C2	0.004798	0.586986	0.141	0.118	1	Saliva_Peri	BCL7C
TMEM52	0.004872	0.323975	0.033	0.019	1	Saliva_Peri	TMEM52
HIPK22	0.004921	0.257184	0.048	0.08	1	Saliva_Peri	HIPK2
MICAL1	0.00495	0.285977	0.051	0.033	1	Saliva_Peri	MICAL1
OAT1	0.005073	0.318002	0.118	0.172	1	Saliva_Peri	OAT
VPS281	0.005118	0.307038	0.243	0.351	1	Saliva_Peri	VPS28
TMEM136	0.005354	0.250687	0.035	0.02	1	Saliva_Peri	TMEM136
OSBPL1A2	0.005363	0.6917	0.177	0.16	1	Saliva_Peri	OSBPL1A
PLEKHO1	0.005566	0.318689	0.109	0.085	1	Saliva_Peri	PLEKHO1
PPIL41	0.005925	0.614872	0.122	0.1	1	Saliva_Peri	PPIL4
MRPS11	0.005984	0.250406	0.072	0.11	1	Saliva_Peri	MRPS11
TRIM472	0.00601	0.332582	0.08	0.059	1	Saliva_Peri	TRIM47
MLH32	0.006243	0.431379	0.066	0.047	1	Saliva_Peri	MLH3
USP143	0.006335	0.265005	0.065	0.101	1	Saliva_Peri	USP14
RBM39	0.006433	0.282242	0.292	0.412	1	Saliva_Peri	RBM39
PRDX23	0.006515	0.505551	0.307	0.312	1	Saliva_Peri	PRDX2
ETFB1	0.006613	0.278673	0.192	0.273	1	Saliva_Peri	ETFB
TCF252	0.006668	0.354586	0.207	0.295	1	Saliva_Peri	TCF25
FAM127B1	0.006683	0.600096	0.101	0.08	1	Saliva_Peri	FAM127B
BOLA3	0.006746	0.349248	0.076	0.116	1	Saliva_Peri	BOLA3
TMEM126I	0.006775	0.250886	0.083	0.124	1	Saliva_Peri	TMEM126B
CNRIP12	0.006826	0.342323	0.057	0.039	1	Saliva_Peri	CNRIP1
CRTAP2	0.006887	0.549902	0.137	0.115	1	Saliva_Peri	CRTAP
RHOBTB32	0.006917	0.407602	0.079	0.119	1	Saliva_Peri	RHOBTB3
USP161	0.007339	0.343498	0.124	0.18	1	Saliva_Peri	USP16
CAMK2N1:	0.007673	0.727342	0.141	0.12	1	Saliva_Peri	CAMK2N1
NSD12	0.007714	0.25538	0.066	0.102	1	Saliva_Peri	NSD1
CCT81	0.00841	0.257942	0.147	0.206	1	Saliva_Peri	CCT8
PKN2	0.008509	0.361763	0.108	0.156	1	Saliva_Peri	PKN2
ATP5C1	0.00858	0.740838	0.272	0.282	1	Saliva_Peri	ATP5C1
VPS362	0.008664	0.28142	0.076	0.114	1	Saliva_Peri	VPS36
HSD17B12	0.008889	0.251124	0.076	0.114	1	Saliva_Peri	HSD17B12
TRAPPC2L	0.008942	0.291257	0.14	0.198	1	Saliva_Peri	TRAPPC2L
WBP4	0.009134	0.273916	0.064	0.097	1	Saliva_Peri	WBP4
LAMTOR1	0.009339	0.328794	0.205	0.288	1	Saliva_Peri	LAMTOR1
NCL3	0.009416	0.661586	0.272	0.271	1	Saliva_Peri	NCL

GOLGB12	0.00943	0.337648	0.141	0.2	1	Saliva_Peri	GOLGB1
CCL5	0	3.663779	0.652	0.051	0	Saliva_T	CCL5
PTPRC	0	2.957089	0.641	0.099	0	Saliva_T	PTPRC
CD2	0	2.674588	0.502	0.059	0	Saliva_T	CD2
GZMK	0	2.394294	0.191	0.012	0	Saliva_T	GZMK
GZMA	0	2.3841	0.377	0.034	0	Saliva_T	GZMA
GZMH	0	2.244713	0.24	0.017	0	Saliva_T	GZMH
CD3G	0	2.172764	0.352	0.037	0	Saliva_T	CD3G
CD3D	0	2.169061	0.581	0.118	0	Saliva_T	CD3D
CD8A	0	2.033302	0.252	0.022	0	Saliva_T	CD8A
KLRC1	0	1.85694	0.161	0.011	0	Saliva_T	KLRC1
CD52	0	1.846689	0.694	0.22	0	Saliva_T	CD52
APOBEC3G	0	1.845975	0.251	0.043	0	Saliva_T	APOBEC3G
IL32	0	1.842256	0.617	0.179	0	Saliva_T	IL32
HCST	0	1.802633	0.521	0.168	0	Saliva_T	HCST
PTPN7	0	1.637612	0.215	0.027	0	Saliva_T	PTPN7
S100A42	0	1.625446	0.863	0.459	0	Saliva_T	S100A4
NKG7	0	1.504294	0.356	0.044	0	Saliva_T	NKG7
IKZF31	0	1.385641	0.15	0.01	0	Saliva_T	IKZF3
MT.ATP66	0	1.16374	0.999	0.897	0	Saliva_T	MT.ATP6
MT.CYB6	0	1.142078	0.999	0.933	0	Saliva_T	MT.CYB
B2M2	0	1.032963	0.999	0.928	0	Saliva_T	B2M
THEMIS	0	1.024237	0.101	0.006	0	Saliva_T	THEMIS
MT.ND36	0	1.014674	0.994	0.791	0	Saliva_T	MT.ND3
MT.CO26	0	0.820473	0.999	0.959	0	Saliva_T	MT.CO2
RPL303	5.2E-306	1.162101	0.958	0.843	8.3E-302	Saliva_T	RPL30
CD226	6.7E-305	0.917195	0.089	0.006	1.1E-300	Saliva_T	CD226
MT.ND56	1.7E-280	1.158623	0.89	0.7	2.8E-276	Saliva_T	MT.ND5
RPL28	7E-277	1.007292	0.966	0.909	1.1E-272	Saliva_T	RPL28
FYB	1E-271	1.731358	0.302	0.069	1.6E-267	Saliva_T	FYB
CST7	4E-258	1.577012	0.279	0.06	6.4E-254	Saliva_T	CST7
ALOX5AP	8.8E-257	1.796393	0.332	0.087	1.4E-252	Saliva_T	ALOX5AP
KLRB1	7E-244	1.834129	0.205	0.036	1.1E-239	Saliva_T	KLRB1
CCL4	2.1E-241	2.166005	0.226	0.044	3.3E-237	Saliva_T	CCL4
RPL392	3.6E-233	1.117968	0.921	0.673	5.8E-229	Saliva_T	RPL39
GZMB	5.5E-219	1.283529	0.166	0.026	8.8E-215	Saliva_T	GZMB
GPR171	3.1E-202	0.845543	0.078	0.007	4.9E-198	Saliva_T	GPR171
MT.ND16	3.5E-201	0.808408	0.965	0.927	5.5E-197	Saliva_T	MT.ND1
MT.CO35	2E-200	0.776264	0.996	0.977	3.3E-196	Saliva_T	MT.CO3
MT.ND46	3E-199	0.612509	0.993	0.953	4.8E-195	Saliva_T	MT.ND4
RPS27A2	4.2E-197	0.837775	0.974	0.848	6.7E-193	Saliva_T	RPS27A
LCP1	4.5E-195	1.763902	0.326	0.105	7.3E-191	Saliva_T	LCP1
CLEC2D	2.9E-185	1.523756	0.2	0.044	4.6E-181	Saliva_T	CLEC2D
CD8B	7.4E-181	1.30995	0.187	0.038	1.2E-176	Saliva_T	CD8B
RUNX3	2E-180	1.320807	0.164	0.031	3.2E-176	Saliva_T	RUNX3
CD69	1.5E-176	1.636801	0.254	0.068	2.4E-172	Saliva_T	CD69
SH3BGRL3	6.6E-167	1.236742	0.763	0.631	1.1E-162	Saliva_T	SH3BGRL3

C16orf541	1E-165	0.850129	0.085	0.01	1.7E-161	Saliva_T	C16orf54
CORO1A	4.1E-161	1.180678	0.469	0.195	6.6E-157	Saliva_T	CORO1A
PTPN22	4.8E-161	1.020239	0.103	0.014	7.7E-157	Saliva_T	PTPN22
IL7R	2.7E-156	1.707983	0.223	0.059	4.3E-152	Saliva_T	IL7R
CD84	7.1E-150	1.15113	0.12	0.02	1.1E-145	Saliva_T	CD84
CD96	9.3E-150	1.069892	0.119	0.02	1.5E-145	Saliva_T	CD96
MT.ND26	9.3E-150	0.615212	0.961	0.906	1.5E-145	Saliva_T	MT.ND2
RPS12	7.6E-148	0.731024	0.972	0.86	1.2E-143	Saliva_T	RPS12
KLRD1	1.4E-142	1.127177	0.108	0.017	2.2E-138	Saliva_T	KLRD1
TPT13	8.8E-140	0.601559	0.977	0.958	1.4E-135	Saliva_T	TPT1
STK41	5E-139	1.650448	0.298	0.111	8E-135	Saliva_T	STK4
ITGA41	1.6E-138	1.152246	0.136	0.027	2.5E-134	Saliva_T	ITGA4
RPS241	8.6E-133	0.72834	0.915	0.838	1.4E-128	Saliva_T	RPS24
SRGN1	1.4E-132	0.920135	0.467	0.22	2.3E-128	Saliva_T	SRGN
APBB1IP	1.9E-131	1.240477	0.165	0.039	3E-127	Saliva_T	APBB1IP
CCL4L2	8.7E-127	1.576077	0.094	0.015	1.4E-122	Saliva_T	CCL4L2
GZMM	1.1E-125	1.273047	0.215	0.063	1.7E-121	Saliva_T	GZMM
HLA.C2	1.4E-125	0.83296	0.805	0.684	2.2E-121	Saliva_T	HLA.C
XCL1	3.2E-124	0.900739	0.077	0.01	5.2E-120	Saliva_T	XCL1
EMB1	3.2E-123	1.275149	0.161	0.039	5.1E-119	Saliva_T	EMB
CYBA1	3.5E-123	0.836372	0.656	0.438	5.5E-119	Saliva_T	CYBA
RASGRP1	1.5E-120	0.955341	0.094	0.015	2.4E-116	Saliva_T	RASGRP1
PYHIN1	4.9E-118	0.787512	0.084	0.013	7.8E-114	Saliva_T	PYHIN1
CD481	2E-117	1.219017	0.308	0.121	3.2E-113	Saliva_T	CD48
RPS15A1	4.6E-113	0.660493	0.938	0.848	7.3E-109	Saliva_T	RPS15A
CXCR4	7.5E-112	1.818033	0.297	0.121	1.2E-107	Saliva_T	CXCR4
RPLP11	2E-110	0.565632	0.978	0.914	3.2E-106	Saliva_T	RPLP1
APOBEC3H	5.7E-110	0.325388	0.026	0.001	9.2E-106	Saliva_T	APOBEC3H
MTRNR2L1	3E-108	0.939841	0.485	0.266	4.8E-104	Saliva_T	MTRNR2L12
TNFSF14	5.5E-106	0.746064	0.069	0.01	8.8E-102	Saliva_T	TNFSF14
MT.CO16	1.6E-102	0.657374	0.995	0.985	2.49E-98	Saliva_T	MT.CO1
KLRC2	3.1E-101	0.540784	0.034	0.003	4.87E-97	Saliva_T	KLRC2
ITGAL	1.14E-98	0.78666	0.08	0.013	1.82E-94	Saliva_T	ITGAL
HLA.B1	1.47E-98	0.49328	0.836	0.664	2.34E-94	Saliva_T	HLA.B
EOMES	3.83E-98	0.49029	0.032	0.002	6.11E-94	Saliva_T	EOMES
HLA.A1	6.04E-98	0.627319	0.797	0.667	9.63E-94	Saliva_T	HLA.A
TMSB4X	8.48E-98	0.310926	0.981	0.869	1.35E-93	Saliva_T	TMSB4X
CXCR3	9.82E-98	0.901911	0.096	0.019	1.57E-93	Saliva_T	CXCR3
RPS29	1.51E-97	0.734427	0.836	0.79	2.41E-93	Saliva_T	RPS29
IL2RB	1.48E-96	0.841519	0.084	0.015	2.36E-92	Saliva_T	IL2RB
H3F3A5	2.81E-95	0.909764	0.642	0.476	4.48E-91	Saliva_T	H3F3A
CCR5	1.03E-91	0.535645	0.042	0.004	1.64E-87	Saliva_T	CCR5
GPR65	9.34E-91	0.988878	0.104	0.023	1.49E-86	Saliva_T	GPR65
EMP32	2.2E-88	1.055544	0.524	0.349	3.51E-84	Saliva_T	EMP3
GPRIN31	4.45E-86	0.730252	0.068	0.011	7.1E-82	Saliva_T	GPRIN3
SH2D1A	7.56E-86	0.754383	0.076	0.013	1.21E-81	Saliva_T	SH2D1A
VIM4	9.21E-86	0.482302	0.75	0.491	1.47E-81	Saliva_T	VIM

ZNF683	3.11E-82	1.053897	0.113	0.028	4.95E-78	Saliva_T	ZNF683
SKAP1	7.93E-81	1.164476	0.169	0.057	1.27E-76	Saliva_T	SKAP1
LCK	1.27E-80	0.998649	0.217	0.082	2.02E-76	Saliva_T	LCK
ITGA12	3.79E-79	1.040747	0.098	0.023	6.04E-75	Saliva_T	ITGA1
RPL7A4	3.3E-78	0.512954	0.907	0.813	5.27E-74	Saliva_T	RPL7A
RPS232	3.36E-78	0.541552	0.93	0.812	5.36E-74	Saliva_T	RPS23
ACAP1	1.06E-77	1.050768	0.289	0.134	1.69E-73	Saliva_T	ACAP1
RPL19	1.11E-74	0.383062	0.955	0.847	1.78E-70	Saliva_T	RPL19
TIGIT	3.04E-74	0.7245	0.064	0.011	4.84E-70	Saliva_T	TIGIT
FAU1	8.37E-73	0.514369	0.891	0.855	1.34E-68	Saliva_T	FAU
SLFN52	1.21E-72	1.350564	0.183	0.07	1.93E-68	Saliva_T	SLFN5
CD3E	4.59E-72	0.734814	0.263	0.11	7.33E-68	Saliva_T	CD3E
CD531	1.04E-71	1.132287	0.262	0.121	1.66E-67	Saliva_T	CD53
RPS27	1.8E-71	0.378843	0.967	0.894	2.87E-67	Saliva_T	RPS27
GNLY	5.2E-70	0.382221	0.125	0.034	8.29E-66	Saliva_T	GNLY
LCP2	9.4E-70	0.920423	0.101	0.026	1.5E-65	Saliva_T	LCP2
ARL4C	1.69E-69	1.623866	0.324	0.187	2.7E-65	Saliva_T	ARL4C
CALM15	5.45E-69	0.950464	0.643	0.652	8.7E-65	Saliva_T	CALM1
PSMB92	7.61E-68	1.297731	0.307	0.167	1.21E-63	Saliva_T	PSMB9
GBP5	8.74E-68	0.612548	0.051	0.008	1.39E-63	Saliva_T	GBP5
RPS7	9.15E-67	0.430307	0.892	0.815	1.46E-62	Saliva_T	RPS7
SPN	8E-65	0.832734	0.092	0.023	1.28E-60	Saliva_T	SPN
RPL371	1.58E-63	0.562252	0.849	0.848	2.52E-59	Saliva_T	RPL37
LAG3	2.89E-62	0.677714	0.059	0.011	4.6E-58	Saliva_T	LAG3
DOCK81	1.43E-61	1.129541	0.144	0.051	2.29E-57	Saliva_T	DOCK8
SAMSN1	1.6E-61	0.965278	0.134	0.045	2.55E-57	Saliva_T	SAMSN1
RAC2	1.86E-60	0.899492	0.3	0.155	2.97E-56	Saliva_T	RAC2
DOCK10	1.16E-58	0.757333	0.074	0.017	1.85E-54	Saliva_T	DOCK10
BCL11B	2.23E-58	0.938256	0.095	0.026	3.56E-54	Saliva_T	BCL11B
LAT	5.98E-58	0.855668	0.167	0.064	9.54E-54	Saliva_T	LAT
MIAT1	1.83E-57	0.515204	0.043	0.007	2.91E-53	Saliva_T	MIAT
CD7	1.3E-56	0.95687	0.224	0.1	2.08E-52	Saliva_T	CD7
RPL91	1.37E-56	0.397604	0.884	0.748	2.18E-52	Saliva_T	RPL9
PSTPIP1	3.22E-56	0.749567	0.087	0.023	5.13E-52	Saliva_T	PSTPIP1
RPSA	1.9E-55	0.431139	0.769	0.677	3.04E-51	Saliva_T	RPSA
RPL14	3.32E-55	0.385939	0.873	0.795	5.3E-51	Saliva_T	RPL14
GRAP2	3.45E-52	0.659091	0.067	0.016	5.5E-48	Saliva_T	GRAP2
MS4A2	6.85E-52	0.25518	0.011	0.001	1.09E-47	Saliva_T	MS4A2
SAMD3	7.31E-52	0.696083	0.079	0.021	1.17E-47	Saliva_T	SAMD3
IKZF11	1.19E-51	0.904551	0.115	0.039	1.89E-47	Saliva_T	IKZF1
MYO1G	2.1E-51	0.79751	0.101	0.031	3.35E-47	Saliva_T	MYO1G
APOBEC3C	4.87E-51	1.167299	0.138	0.053	7.76E-47	Saliva_T	APOBEC3C
ANKRD44	7.58E-51	0.907275	0.107	0.035	1.21E-46	Saliva_T	ANKRD44
MYL12A1	1.47E-50	0.767637	0.66	0.658	2.35E-46	Saliva_T	MYL12A
CCL3L3	3.43E-50	0.348776	0.029	0.004	5.47E-46	Saliva_T	CCL3L3
ITK	1.12E-48	0.566722	0.059	0.013	1.78E-44	Saliva_T	ITK
CKLF1	3.53E-48	1.439537	0.257	0.149	5.63E-44	Saliva_T	CKLF

EVL	5.58E-48	1.11142	0.284	0.165	8.9E-44	Saliva_T	EVL
SLA	7.55E-48	0.696838	0.084	0.024	1.2E-43	Saliva_T	SLA
SLA2	1.28E-46	0.475849	0.042	0.008	2.04E-42	Saliva_T	SLA2
RASAL3	2.48E-45	0.751119	0.092	0.029	3.96E-41	Saliva_T	RASAL3
STK17B1	7.73E-45	1.24092	0.202	0.103	1.23E-40	Saliva_T	STK17B
ITM2A2	7.9E-45	1.179571	0.243	0.134	1.26E-40	Saliva_T	ITM2A
ATP5E	4.84E-44	0.704962	0.651	0.685	7.71E-40	Saliva_T	ATP5E
CASP81	7.24E-44	0.950733	0.109	0.039	1.16E-39	Saliva_T	CASP8
RPS21	8.35E-44	0.631229	0.701	0.673	1.33E-39	Saliva_T	RPS21
SH2D2A	1.15E-43	0.567884	0.057	0.014	1.83E-39	Saliva_T	SH2D2A
TBC1D10C	8.46E-43	0.872608	0.161	0.072	1.35E-38	Saliva_T	TBC1D10C
RPL243	1.19E-42	0.472356	0.802	0.802	1.9E-38	Saliva_T	RPL24
RPS19	2.09E-42	0.252458	0.938	0.875	3.33E-38	Saliva_T	RPS19
ICOS	2.52E-42	0.380029	0.033	0.005	4.02E-38	Saliva_T	ICOS
EEF1A12	2.57E-42	0.34788	0.951	0.915	4.1E-38	Saliva_T	EEF1A1
APOBEC3D	9.33E-42	0.412686	0.033	0.005	1.49E-37	Saliva_T	APOBEC3D
CD101	1.59E-41	0.281335	0.021	0.002	2.54E-37	Saliva_T	CD101
ZC3H12D	1.77E-41	0.295821	0.025	0.003	2.83E-37	Saliva_T	ZC3H12D
RPS175	1.9E-38	0.739229	0.331	0.221	3.03E-34	Saliva_T	RPS17
SLAMF11	3.59E-38	0.548576	0.051	0.012	5.73E-34	Saliva_T	SLAMF1
GMFG	4.67E-38	0.776829	0.325	0.208	7.45E-34	Saliva_T	GMFG
TRAF3IP3	5.91E-38	0.799925	0.191	0.096	9.43E-34	Saliva_T	TRAF3IP3
CD6	4.21E-37	0.655753	0.085	0.028	6.71E-33	Saliva_T	CD6
MYBL1	6.16E-37	0.528274	0.05	0.012	9.82E-33	Saliva_T	MYBL1
RPL181	3.45E-36	0.25835	0.904	0.878	5.5E-32	Saliva_T	RPL18
RPL224	1.62E-35	0.592094	0.689	0.694	2.58E-31	Saliva_T	RPL22
EVI2B1	3.25E-35	0.890559	0.138	0.063	5.18E-31	Saliva_T	EVI2B
SYNE22	6.67E-35	1.341355	0.225	0.136	1.06E-30	Saliva_T	SYNE2
NCKAP1L1	7.27E-35	0.657172	0.069	0.022	1.16E-30	Saliva_T	NCKAP1L
CLEC2B2	8.22E-35	1.201862	0.245	0.155	1.31E-30	Saliva_T	CLEC2B
HELB	4.67E-34	0.505756	0.047	0.012	7.44E-30	Saliva_T	HELB
KIAA15511	9.29E-34	1.245676	0.202	0.117	1.48E-29	Saliva_T	KIAA1551
P2RY10	4.74E-33	0.504857	0.047	0.012	7.56E-29	Saliva_T	P2RY10
ARPC1B1	4.95E-33	0.872618	0.376	0.292	7.9E-29	Saliva_T	ARPC1B
ETS11	2.36E-32	1.015901	0.151	0.076	3.77E-28	Saliva_T	ETS1
MTRNR2L1	2.38E-32	0.634926	0.22	0.125	3.79E-28	Saliva_T	MTRNR2L1
JAKMIP2	2.45E-32	0.405661	0.031	0.006	3.91E-28	Saliva_T	JAKMIP2
AKNA	4.23E-32	0.809517	0.093	0.036	6.75E-28	Saliva_T	AKNA
SEPT73	1.15E-31	1.157543	0.39	0.339	1.83E-27	Saliva_T	SEPT7
MATK	1.52E-31	0.669223	0.079	0.028	2.43E-27	Saliva_T	MATK
GIMAP41	2.72E-31	0.807089	0.151	0.074	4.33E-27	Saliva_T	GIMAP4
ARHGAP15	3.45E-31	0.857994	0.132	0.062	5.51E-27	Saliva_T	ARHGAP15
PREX1	8.38E-31	0.618918	0.065	0.021	1.34E-26	Saliva_T	PREX1
JUND4	1.32E-30	1.037942	0.477	0.464	2.1E-26	Saliva_T	JUND
TNF	5.18E-30	0.70622	0.059	0.018	8.25E-26	Saliva_T	TNF
SPOCK2	7.76E-30	0.743586	0.102	0.043	1.24E-25	Saliva_T	SPOCK2
HLA.E2	1E-29	0.662847	0.59	0.61	1.6E-25	Saliva_T	HLA.E

PARP15	2.43E-29	0.29851	0.025	0.004	3.87E-25	Saliva_T	PARP15
PRKCB	9.13E-29	0.693493	0.072	0.025	1.46E-24	Saliva_T	PRKCB
LAX11	9.7E-29	0.290369	0.027	0.005	1.55E-24	Saliva_T	LAX1
TGFB11	1.11E-28	0.958328	0.174	0.098	1.76E-24	Saliva_T	TGFB1
N4BP2L24	2.45E-28	1.384362	0.343	0.293	3.91E-24	Saliva_T	N4BP2L2
LAPTM5	2.62E-28	0.668007	0.268	0.172	4.18E-24	Saliva_T	LAPTM5
GAB3	3.13E-28	0.359467	0.033	0.007	4.99E-24	Saliva_T	GAB3
SEPT61	3.14E-28	1.007623	0.168	0.094	5.02E-24	Saliva_T	SEPT6
TOX	1.07E-27	0.398695	0.034	0.008	1.7E-23	Saliva_T	TOX
CRTAM	1.15E-27	0.40005	0.033	0.007	1.83E-23	Saliva_T	CRTAM
MYO1F	1.19E-27	0.682151	0.101	0.043	1.9E-23	Saliva_T	MYO1F
GIMAP71	3.32E-27	0.621146	0.209	0.12	5.29E-23	Saliva_T	GIMAP7
CD247	3.76E-27	0.551972	0.115	0.052	6E-23	Saliva_T	CD247
FYN2	4.5E-27	0.94213	0.132	0.066	7.17E-23	Saliva_T	FYN
ARHGAP3C	4.84E-27	0.769292	0.108	0.049	7.73E-23	Saliva_T	ARHGAP30
SCML4	5.48E-27	0.597584	0.06	0.02	8.74E-23	Saliva_T	SCML4
UBASH3A	5.77E-27	0.343108	0.034	0.008	9.21E-23	Saliva_T	UBASH3A
RPS281	5.65E-26	0.289343	0.891	0.881	9.01E-22	Saliva_T	RPS28
DUSP21	5.7E-26	1.265843	0.202	0.125	9.1E-22	Saliva_T	DUSP2
MBNL12	1.71E-25	1.147942	0.242	0.17	2.73E-21	Saliva_T	MBNL1
FAM101B1	4.46E-25	0.617916	0.057	0.019	7.12E-21	Saliva_T	FAM101B
CLIC11	2.2E-24	0.85247	0.477	0.476	3.51E-20	Saliva_T	CLIC1
ENY23	2.74E-24	0.264441	0.152	0.325	4.37E-20	Saliva_T	ENY2
SRSF74	5.02E-24	1.22546	0.372	0.343	8.01E-20	Saliva_T	SRSF7
CTD.2020K	5.62E-24	0.49398	0.047	0.015	8.97E-20	Saliva_T	CTD.2020K17.1
CCR6	1.13E-23	0.401524	0.038	0.011	1.81E-19	Saliva_T	CCR6
PARP8	1.41E-23	0.842246	0.086	0.038	2.24E-19	Saliva_T	PARP8
MPC12	2.52E-23	0.250524	0.086	0.213	4.01E-19	Saliva_T	MPC1
MS4A1	2.82E-23	0.830846	0.057	0.02	4.5E-19	Saliva_T	MS4A1
AIMP1	2.83E-23	0.257249	0.076	0.196	4.51E-19	Saliva_T	AIMP1
CYFIP2	5.56E-22	0.646815	0.072	0.03	8.87E-18	Saliva_T	CYFIP2
MRPL41	8.42E-22	0.275647	0.151	0.312	1.34E-17	Saliva_T	MRPL41
LSM32	1.38E-21	0.358355	0.146	0.307	2.21E-17	Saliva_T	LSM3
SYTL3	2.07E-21	0.629886	0.067	0.027	3.3E-17	Saliva_T	SYTL3
SASH3	2.92E-21	0.526149	0.059	0.022	4.66E-17	Saliva_T	SASH3
AGAP21	3.95E-21	0.274807	0.022	0.005	6.29E-17	Saliva_T	AGAP2
CD271	4.07E-21	0.603061	0.153	0.085	6.49E-17	Saliva_T	CD27
KLRG1	5.89E-21	0.686329	0.062	0.024	9.39E-17	Saliva_T	KLRG1
NDUFS61	7.03E-21	0.27508	0.161	0.327	1.12E-16	Saliva_T	NDUFS6
DOCK21	7.47E-21	0.515939	0.055	0.02	1.19E-16	Saliva_T	DOCK2
NLRC51	8.54E-21	0.523106	0.05	0.018	1.36E-16	Saliva_T	NLRC5
DOK2	1.44E-20	0.662607	0.118	0.062	2.3E-16	Saliva_T	DOK2
MGEA5	2.58E-20	0.292786	0.101	0.225	4.11E-16	Saliva_T	MGEA5
H2AFV	2.63E-20	0.267984	0.144	0.294	4.2E-16	Saliva_T	H2AFV
RNF7	3.1E-20	0.278882	0.151	0.306	4.95E-16	Saliva_T	RNF7
SELT1	7.46E-20	0.252949	0.105	0.229	1.19E-15	Saliva_T	SELT
SRSF111	8.73E-20	0.297353	0.152	0.306	1.39E-15	Saliva_T	SRSF11

CHCHD12	1.8E-19	0.260058	0.072	0.175	2.87E-15	Saliva_T	CHCHD1
ATP5C11	2.14E-19	0.261219	0.14	0.285	3.41E-15	Saliva_T	ATP5C1
OXNAD1	5.49E-19	0.673385	0.079	0.036	8.76E-15	Saliva_T	OXNAD1
MRPS18C3	9.19E-19	0.302394	0.083	0.191	1.47E-14	Saliva_T	MRPS18C
SNRPB2	1.5E-18	0.298258	0.121	0.251	2.39E-14	Saliva_T	SNRPB2
GNAI3	1.79E-18	0.25498	0.086	0.194	2.85E-14	Saliva_T	GNAI3
S100A102	2.77E-18	0.508909	0.68	0.676	4.42E-14	Saliva_T	S100A10
TOX42	4.01E-18	0.258614	0.07	0.168	6.39E-14	Saliva_T	TOX4
UBXN42	4.12E-18	0.257909	0.155	0.303	6.57E-14	Saliva_T	UBXN4
GCC2	6.44E-18	0.264363	0.138	0.274	1.03E-13	Saliva_T	GCC2
HMGN2	6.96E-18	0.289462	0.173	0.332	1.11E-13	Saliva_T	HMGN2
SRP191	8.29E-18	0.256447	0.079	0.18	1.32E-13	Saliva_T	SRP19
COTL1	1.41E-17	0.804913	0.249	0.184	2.25E-13	Saliva_T	COTL1
TAF1D	2.27E-17	0.27732	0.111	0.23	3.62E-13	Saliva_T	TAF1D
SYNCRIP1	2.32E-17	0.27115	0.084	0.188	3.7E-13	Saliva_T	SYNCRIP
ATG12	2.76E-17	0.262304	0.072	0.168	4.4E-13	Saliva_T	ATG12
GOLGA42	3.38E-17	0.333392	0.116	0.238	5.39E-13	Saliva_T	GOLGA4
TCF253	5.62E-17	0.363263	0.154	0.297	8.96E-13	Saliva_T	TCF25
TMEM134:	6.79E-17	0.257478	0.079	0.177	1.08E-12	Saliva_T	TMEM134
TMEM14B	7.42E-17	0.291897	0.128	0.255	1.18E-12	Saliva_T	TMEM14B
DDX3X1	7.96E-17	0.349254	0.159	0.304	1.27E-12	Saliva_T	DDX3X
ESD3	1.05E-16	0.252567	0.107	0.22	1.67E-12	Saliva_T	ESD
CFLAR2	1.12E-16	0.28003	0.142	0.274	1.78E-12	Saliva_T	CFLAR
USP81	1.24E-16	0.269177	0.059	0.145	1.98E-12	Saliva_T	USP8
MARCH91	1.31E-16	0.641181	0.064	0.028	2.09E-12	Saliva_T	MARCH9
FMNL11	1.53E-16	0.739294	0.113	0.063	2.45E-12	Saliva_T	FMNL1
CIR12	2.29E-16	0.298362	0.076	0.17	3.65E-12	Saliva_T	CIR1
RBM391	2.55E-16	0.300791	0.227	0.414	4.06E-12	Saliva_T	RBM39
MRPL203	2.98E-16	0.26083	0.142	0.273	4.76E-12	Saliva_T	MRPL20
STAT4	3.06E-16	0.561573	0.059	0.025	4.88E-12	Saliva_T	STAT4
ACTR3	3.12E-16	0.323194	0.193	0.359	4.98E-12	Saliva_T	ACTR3
PDCD101	3.48E-16	0.391744	0.098	0.206	5.54E-12	Saliva_T	PDCD10
ORMDL21	3.76E-16	0.369712	0.086	0.186	5.99E-12	Saliva_T	ORMDL2
ZNF207	4.52E-16	0.463725	0.136	0.266	7.21E-12	Saliva_T	ZNF207
CTSW	4.97E-16	0.486879	0.125	0.071	7.93E-12	Saliva_T	CTSW
OSBPL81	5.38E-16	0.255604	0.082	0.178	8.59E-12	Saliva_T	OSBPL8
EIF53	5.71E-16	0.28985	0.199	0.363	9.1E-12	Saliva_T	EIF5
GTF3C61	6.37E-16	0.357037	0.098	0.204	1.02E-11	Saliva_T	GTF3C6
KHDRBS11	6.65E-16	0.279673	0.108	0.218	1.06E-11	Saliva_T	KHDRBS1
RARRES3	6.73E-16	0.623067	0.28	0.22	1.07E-11	Saliva_T	RARRES3
UBE2I1	7.61E-16	0.322815	0.138	0.266	1.21E-11	Saliva_T	UBE2I
NSA22	8.09E-16	0.304176	0.13	0.252	1.29E-11	Saliva_T	NSA2
GGNBP2	8.96E-16	0.318126	0.1	0.205	1.43E-11	Saliva_T	GGNBP2
RNMT	8.99E-16	0.349784	0.101	0.207	1.43E-11	Saliva_T	RNMT
MAP4K1	1.43E-15	0.371038	0.045	0.017	2.28E-11	Saliva_T	MAP4K1
FAM133B1	2.43E-15	0.335388	0.098	0.201	3.88E-11	Saliva_T	FAM133B
MPLKIP	2.77E-15	0.289943	0.075	0.165	4.42E-11	Saliva_T	MPLKIP

PPP1R14B4	4.36E-15	0.284266	0.085	0.179	6.95E-11	Saliva_T	PPP1R14B
MGMT1	4.77E-15	0.266716	0.075	0.164	7.61E-11	Saliva_T	MGMT
MTDH3	5.68E-15	0.276115	0.171	0.312	9.06E-11	Saliva_T	MTDH
VPS282	6.5E-15	0.368136	0.193	0.352	1.04E-10	Saliva_T	VPS28
CHD41	7.07E-15	0.278583	0.074	0.161	1.13E-10	Saliva_T	CHD4
DCTN32	7.86E-15	0.338808	0.123	0.237	1.25E-10	Saliva_T	DCTN3
CD472	8.52E-15	0.292292	0.12	0.232	1.36E-10	Saliva_T	CD47
RNF2131	1.04E-14	1.141148	0.195	0.146	1.66E-10	Saliva_T	RNF213
FAM162A	1.09E-14	0.310661	0.096	0.196	1.75E-10	Saliva_T	FAM162A
SMAD2	1.23E-14	0.252945	0.053	0.129	1.96E-10	Saliva_T	SMAD2
ARID4B	1.31E-14	0.328563	0.128	0.244	2.09E-10	Saliva_T	ARID4B
COPA3	1.66E-14	0.258162	0.066	0.148	2.64E-10	Saliva_T	COPA
OASL	1.92E-14	0.422213	0.042	0.016	3.07E-10	Saliva_T	OASL
COX7B1	2.09E-14	0.33564	0.229	0.413	3.33E-10	Saliva_T	COX7B
BATF	2.09E-14	0.668803	0.083	0.044	3.34E-10	Saliva_T	BATF
UFD1L1	2.31E-14	0.301931	0.088	0.181	3.69E-10	Saliva_T	UFD1L
COX20	2.72E-14	0.267007	0.079	0.168	4.33E-10	Saliva_T	COX20
ACTR2	3.61E-14	0.360732	0.169	0.309	5.75E-10	Saliva_T	ACTR2
CD5	3.74E-14	0.423474	0.045	0.018	5.97E-10	Saliva_T	CD5
HNRNPU	5.01E-14	0.464683	0.195	0.352	8E-10	Saliva_T	HNRNPU
WIPF11	7.53E-14	0.818842	0.14	0.092	1.2E-09	Saliva_T	WIPF1
ABHD17A2	9.13E-14	1.075795	0.164	0.116	1.46E-09	Saliva_T	ABHD17A
MZT2A	1.05E-13	0.387655	0.174	0.314	1.67E-09	Saliva_T	MZT2A
UCP2	1.3E-13	0.819285	0.206	0.154	2.07E-09	Saliva_T	UCP2
HNRNPA31	1.31E-13	0.423324	0.206	0.368	2.09E-09	Saliva_T	HNRNPA3
IL2RG1	1.74E-13	0.676002	0.186	0.132	2.78E-09	Saliva_T	IL2RG
SIVA13	1.75E-13	0.291517	0.153	0.276	2.79E-09	Saliva_T	SIVA1
CYTIP1	1.77E-13	0.789755	0.131	0.084	2.83E-09	Saliva_T	CYTIP
PSMD82	1.77E-13	0.394747	0.188	0.335	2.83E-09	Saliva_T	PSMD8
PTBP3	1.89E-13	0.387623	0.077	0.162	3.02E-09	Saliva_T	PTBP3
EEF1B21	1.92E-13	0.394428	0.568	0.598	3.06E-09	Saliva_T	EEF1B2
BLOC1S1	2.1E-13	0.445188	0.191	0.345	3.35E-09	Saliva_T	BLOC1S1
MCTS11	2.45E-13	0.309687	0.072	0.152	3.9E-09	Saliva_T	MCTS1
ATP5J4	2.51E-13	0.294787	0.251	0.44	4E-09	Saliva_T	ATP5J
TERF2IP	2.91E-13	0.327812	0.12	0.227	4.64E-09	Saliva_T	TERF2IP
PHB21	3.37E-13	0.25658	0.103	0.198	5.37E-09	Saliva_T	PHB2
POLR2J34	3.43E-13	0.998989	0.142	0.097	5.47E-09	Saliva_T	POLR2J3
CCT6A1	3.98E-13	0.288342	0.091	0.181	6.35E-09	Saliva_T	CCT6A
DNAJC72	4.19E-13	0.258194	0.079	0.161	6.68E-09	Saliva_T	DNAJC7
LSM51	4.35E-13	0.332021	0.106	0.204	6.93E-09	Saliva_T	LSM5
DERL11	4.84E-13	0.274287	0.062	0.137	7.72E-09	Saliva_T	DERL1
ZNF241	5.03E-13	0.270712	0.065	0.141	8.02E-09	Saliva_T	ZNF24
CDV31	5.1E-13	0.265369	0.089	0.176	8.13E-09	Saliva_T	CDV3
HLA.F1	5.29E-13	0.929604	0.167	0.12	8.44E-09	Saliva_T	HLA.F
DNAJC11	9.97E-13	0.313207	0.082	0.165	1.59E-08	Saliva_T	DNAJC1
KMT2C2	1.15E-12	0.27515	0.076	0.155	1.83E-08	Saliva_T	KMT2C
CARD11	1.19E-12	0.253726	0.024	0.007	1.9E-08	Saliva_T	CARD11

WDR331	1.26E-12	0.284872	0.074	0.153	2E-08	Saliva_T	WDR33
MOB1A	1.42E-12	0.356893	0.097	0.188	2.26E-08	Saliva_T	MOB1A
ABI31	1.63E-12	0.552884	0.064	0.032	2.59E-08	Saliva_T	ABI3
TEX264	1.63E-12	0.280294	0.091	0.177	2.6E-08	Saliva_T	TEX264
SNRPF	1.75E-12	0.300119	0.132	0.24	2.8E-08	Saliva_T	SNRPF
RPS111	1.77E-12	0.278366	0.744	0.778	2.82E-08	Saliva_T	RPS11
PSMB14	1.86E-12	0.365395	0.232	0.403	2.96E-08	Saliva_T	PSMB1
TMC8	2.28E-12	0.515192	0.061	0.03	3.63E-08	Saliva_T	TMC8
RAB37	2.71E-12	0.334205	0.036	0.014	4.32E-08	Saliva_T	RAB37
LEPROTL1	3.17E-12	1.093205	0.211	0.17	5.05E-08	Saliva_T	LEPROTL1
SP1401	3.23E-12	0.393513	0.043	0.018	5.15E-08	Saliva_T	SP140
PAG1	3.24E-12	0.738655	0.085	0.048	5.16E-08	Saliva_T	PAG1
CGGBP1	3.51E-12	0.295009	0.075	0.152	5.6E-08	Saliva_T	CGGBP1
CDC262	3.57E-12	0.265929	0.059	0.128	5.7E-08	Saliva_T	CDC26
RPL22L11	3.79E-12	0.383206	0.12	0.221	6.05E-08	Saliva_T	RPL22L1
KPNA4	4.07E-12	0.340105	0.072	0.148	6.49E-08	Saliva_T	KPNA4
FGFR1OP2	4.37E-12	0.259417	0.078	0.156	6.96E-08	Saliva_T	FGFR1OP2
DNAJC19	5.11E-12	0.324992	0.084	0.166	8.15E-08	Saliva_T	DNAJC19
CMC1	5.46E-12	0.274473	0.05	0.114	8.71E-08	Saliva_T	CMC1
TAF7	5.57E-12	0.401436	0.136	0.245	8.88E-08	Saliva_T	TAF7
UXT1	5.68E-12	0.272623	0.219	0.373	9.05E-08	Saliva_T	UXT
BTN3A22	5.91E-12	0.680224	0.098	0.059	9.43E-08	Saliva_T	BTN3A2
ZDHHC12	6.59E-12	0.29703	0.065	0.136	1.05E-07	Saliva_T	ZDHHC12
CTSC1	7.21E-12	0.886031	0.166	0.121	1.15E-07	Saliva_T	CTSC
TRIM33	8.23E-12	0.254647	0.05	0.114	1.31E-07	Saliva_T	TRIM33
PGAM11	8.46E-12	0.406604	0.127	0.231	1.35E-07	Saliva_T	PGAM1
PDCD2	8.71E-12	0.252751	0.069	0.142	1.39E-07	Saliva_T	PDCD2
SECISBP21	1.2E-11	0.257323	0.066	0.136	1.91E-07	Saliva_T	SECISBP2
HCLS1	1.22E-11	0.631158	0.12	0.077	1.95E-07	Saliva_T	HCLS1
ANAPC161	1.23E-11	0.339355	0.22	0.374	1.96E-07	Saliva_T	ANAPC16
SNW11	1.54E-11	0.263022	0.072	0.144	2.45E-07	Saliva_T	SNW1
DNAJC151	1.54E-11	0.328306	0.103	0.191	2.46E-07	Saliva_T	DNAJC15
TTN1	1.58E-11	0.29557	0.028	0.01	2.52E-07	Saliva_T	TTN
PSMA2	1.75E-11	0.306692	0.059	0.125	2.79E-07	Saliva_T	PSMA2
SDF22	1.8E-11	0.297296	0.069	0.141	2.87E-07	Saliva_T	SDF2
REST	1.88E-11	0.304973	0.062	0.13	2.99E-07	Saliva_T	REST
MAP3K21	2.09E-11	0.259795	0.059	0.126	3.33E-07	Saliva_T	MAP3K2
NEDD8	2.16E-11	0.325118	0.25	0.426	3.44E-07	Saliva_T	NEDD8
KMT2E	2.25E-11	0.42018	0.174	0.302	3.59E-07	Saliva_T	KMT2E
PPP2R5C	2.35E-11	1.195008	0.22	0.186	3.74E-07	Saliva_T	PPP2R5C
NME34	2.45E-11	0.281975	0.118	0.211	3.91E-07	Saliva_T	NME3
PDE7A	2.52E-11	0.757844	0.086	0.051	4.01E-07	Saliva_T	PDE7A
SNRPB	2.7E-11	0.338596	0.197	0.336	4.3E-07	Saliva_T	SNRPB
PRPF38A	2.76E-11	0.283121	0.055	0.118	4.41E-07	Saliva_T	PRPF38A
ACAP21	2.78E-11	0.292292	0.072	0.145	4.43E-07	Saliva_T	ACAP2
TRIP12	3.07E-11	0.328735	0.067	0.136	4.89E-07	Saliva_T	TRIP12
UPF3A2	3.21E-11	0.255401	0.074	0.147	5.12E-07	Saliva_T	UPF3A

MRPL131	3.63E-11	0.324202	0.062	0.129	5.79E-07	Saliva_T	MRPL13
SNX20	3.64E-11	0.301207	0.031	0.012	5.81E-07	Saliva_T	SNX20
RPA31	4.21E-11	0.359526	0.075	0.148	6.71E-07	Saliva_T	RPA3
PRELID11	4.23E-11	0.310418	0.225	0.382	6.75E-07	Saliva_T	PRELID1
SUZ12	4.78E-11	0.287316	0.057	0.12	7.62E-07	Saliva_T	SUZ12
LMNA4	5.06E-11	0.267781	0.232	0.369	8.08E-07	Saliva_T	LMNA
TSTD12	5.1E-11	0.287918	0.16	0.275	8.14E-07	Saliva_T	TSTD1
ITGAE1	5.23E-11	0.25213	0.067	0.134	8.34E-07	Saliva_T	ITGAE
TRIP114	5.75E-11	0.333055	0.058	0.122	9.18E-07	Saliva_T	TRIP11
PIP4K2A	5.77E-11	0.738012	0.097	0.06	9.2E-07	Saliva_T	PIP4K2A
GPR183	6.01E-11	0.427414	0.119	0.076	9.59E-07	Saliva_T	GPR183
DYNC1H12	6.3E-11	0.335164	0.071	0.141	1E-06	Saliva_T	DYNC1H1
ZAP70	8.3E-11	0.414828	0.057	0.029	1.32E-06	Saliva_T	ZAP70
CD40LG	8.49E-11	0.318341	0.038	0.016	1.35E-06	Saliva_T	CD40LG
TRAPPC1	8.88E-11	0.251822	0.139	0.239	1.42E-06	Saliva_T	TRAPPC1
SRI1	9.52E-11	0.410509	0.14	0.244	1.52E-06	Saliva_T	SRI
ANAPC53	1.03E-10	0.359714	0.086	0.163	1.65E-06	Saliva_T	ANAPC5
OFD1	1.05E-10	0.32537	0.084	0.159	1.68E-06	Saliva_T	OFD1
TXNDC9	1.07E-10	0.289109	0.057	0.119	1.7E-06	Saliva_T	TXNDC9
FMNL31	1.08E-10	0.34331	0.03	0.011	1.72E-06	Saliva_T	FMNL3
NDUFAB13	1.1E-10	0.397158	0.142	0.248	1.75E-06	Saliva_T	NDUFAB1
NEMF	1.1E-10	0.377122	0.088	0.165	1.75E-06	Saliva_T	NEMF
ARPC21	1.11E-10	0.710338	0.48	0.576	1.77E-06	Saliva_T	ARPC2
PSMD132	1.28E-10	0.31766	0.086	0.161	2.03E-06	Saliva_T	PSMD13
ATP8A11	1.31E-10	0.422608	0.037	0.016	2.09E-06	Saliva_T	ATP8A1
HNRNPH1	1.67E-10	0.299325	0.181	0.3	2.66E-06	Saliva_T	HNRNPH1
RP11.347P	1.68E-10	0.407727	0.04	0.018	2.68E-06	Saliva_T	RP11.347P5.1
XRN21	1.99E-10	0.342207	0.08	0.152	3.18E-06	Saliva_T	XRN2
ADH52	2.15E-10	0.259321	0.07	0.137	3.43E-06	Saliva_T	ADH5
RORA1	2.35E-10	0.991065	0.159	0.121	3.75E-06	Saliva_T	RORA
USP144	2.41E-10	0.258926	0.046	0.102	3.84E-06	Saliva_T	USP14
DDX46	2.58E-10	0.351417	0.114	0.202	4.12E-06	Saliva_T	DDX46
SBNO1	2.8E-10	0.263096	0.055	0.114	4.47E-06	Saliva_T	SBNO1
SNRPN2	2.9E-10	0.272534	0.05	0.108	4.62E-06	Saliva_T	SNRPN
NCF11	3.2E-10	0.474832	0.073	0.041	5.11E-06	Saliva_T	NCF1
TMEM160	3.21E-10	0.365346	0.118	0.207	5.11E-06	Saliva_T	TMEM160
RAB8A1	3.27E-10	0.312386	0.072	0.139	5.22E-06	Saliva_T	RAB8A
RGS11	3.46E-10	0.668328	0.108	0.069	5.51E-06	Saliva_T	RGS1
ARPC4	3.65E-10	0.362545	0.135	0.234	5.83E-06	Saliva_T	ARPC4
SLTM2	3.9E-10	0.440662	0.11	0.197	6.21E-06	Saliva_T	SLTM
RFC13	3.96E-10	0.291734	0.057	0.117	6.32E-06	Saliva_T	RFC1
RBM51	4.1E-10	0.340146	0.067	0.131	6.54E-06	Saliva_T	RBM5
SIRT7	4.47E-10	0.253789	0.061	0.122	7.12E-06	Saliva_T	SIRT7
ARL6IP51	4.68E-10	0.933501	0.315	0.31	7.46E-06	Saliva_T	ARL6IP5
ITGB2	4.76E-10	0.545032	0.153	0.109	7.59E-06	Saliva_T	ITGB2
ANKRD122	5.8E-10	1.129804	0.316	0.324	9.25E-06	Saliva_T	ANKRD12
HAGH2	6.14E-10	0.318944	0.073	0.14	9.79E-06	Saliva_T	HAGH

SRRM11	6.23E-10	0.461415	0.195	0.33	9.93E-06	Saliva_T	SRRM1
MKLN11	6.26E-10	0.263021	0.054	0.111	9.98E-06	Saliva_T	MKLN1
COA4	6.72E-10	0.286785	0.059	0.118	1.07E-05	Saliva_T	COA4
STK10	7.42E-10	0.671469	0.086	0.052	1.18E-05	Saliva_T	STK10
SUPT16H1	7.43E-10	0.251602	0.053	0.11	1.19E-05	Saliva_T	SUPT16H
SRRM23	7.47E-10	0.431381	0.21	0.351	1.19E-05	Saliva_T	SRRM2
ILF3	7.64E-10	0.295799	0.071	0.136	1.22E-05	Saliva_T	ILF3
CWC25	7.77E-10	0.287626	0.06	0.12	1.24E-05	Saliva_T	CWC25
SELPLG	8.32E-10	0.446403	0.059	0.032	1.33E-05	Saliva_T	SELPLG
PTPN4	9.03E-10	0.68284	0.077	0.046	1.44E-05	Saliva_T	PTPN4
TMEM156:	9.18E-10	0.306222	0.029	0.012	1.46E-05	Saliva_T	TMEM156
ZFP36L22	9.29E-10	0.959677	0.386	0.393	1.48E-05	Saliva_T	ZFP36L2
AP3B11	1.09E-09	0.256512	0.04	0.09	1.75E-05	Saliva_T	AP3B1
PPP1CA1	1.09E-09	0.395687	0.191	0.316	1.75E-05	Saliva_T	PPP1CA
UBE2G2	1.17E-09	0.30106	0.052	0.107	1.87E-05	Saliva_T	UBE2G2
AHNAK4	1.19E-09	1.085016	0.364	0.387	1.9E-05	Saliva_T	AHNAK
FERMT3	1.25E-09	0.562249	0.081	0.049	2E-05	Saliva_T	FERMT3
TRAPPC41	1.47E-09	0.297829	0.07	0.134	2.35E-05	Saliva_T	TRAPPC4
EIF5A1	1.48E-09	0.449032	0.167	0.281	2.35E-05	Saliva_T	EIF5A
GTF2I4	1.65E-09	0.34929	0.085	0.155	2.63E-05	Saliva_T	GTF2I
NDUFB2	1.73E-09	0.315568	0.232	0.378	2.75E-05	Saliva_T	NDUFB2
ATPIF11	1.74E-09	0.278892	0.244	0.397	2.77E-05	Saliva_T	ATPIF1
ICAM31	1.8E-09	0.702529	0.185	0.145	2.87E-05	Saliva_T	ICAM3
PTP4A21	1.82E-09	0.496392	0.176	0.295	2.9E-05	Saliva_T	PTP4A2
BLOC1S2	1.82E-09	0.279159	0.057	0.115	2.91E-05	Saliva_T	BLOC1S2
ARFGEF1	1.86E-09	0.273244	0.042	0.092	2.97E-05	Saliva_T	ARFGEF1
PTPN21	1.93E-09	0.267678	0.06	0.118	3.07E-05	Saliva_T	PTPN2
MRPS341	1.93E-09	0.337197	0.115	0.199	3.08E-05	Saliva_T	MRPS34
BUB3	2E-09	0.362179	0.1	0.177	3.19E-05	Saliva_T	BUB3
LY91	2.14E-09	0.363068	0.042	0.02	3.42E-05	Saliva_T	LY9
IARS21	2.28E-09	0.26276	0.042	0.092	3.63E-05	Saliva_T	IARS2
TMEM259	2.37E-09	0.319759	0.077	0.143	3.79E-05	Saliva_T	TMEM259
EZR1	2.68E-09	0.373635	0.174	0.285	4.28E-05	Saliva_T	EZR
SUGT11	2.71E-09	0.316322	0.073	0.137	4.32E-05	Saliva_T	SUGT1
CD28	2.79E-09	0.250568	0.025	0.01	4.45E-05	Saliva_T	CD28
RAB27A	2.83E-09	0.802954	0.098	0.065	4.52E-05	Saliva_T	RAB27A
SUSD3	2.85E-09	0.468007	0.063	0.035	4.55E-05	Saliva_T	SUSD3
AKAP92	2.92E-09	0.524603	0.162	0.273	4.66E-05	Saliva_T	AKAP9
ATP5G33	2.93E-09	0.311072	0.222	0.365	4.67E-05	Saliva_T	ATP5G3
USP32	2.93E-09	0.303288	0.057	0.113	4.68E-05	Saliva_T	USP3
MT.ND65	2.95E-09	0.903809	0.147	0.111	4.7E-05	Saliva_T	MT.ND6
ERGIC21	2.97E-09	0.396455	0.087	0.158	4.73E-05	Saliva_T	ERGIC2
PRPF4B1	2.97E-09	0.40561	0.091	0.163	4.74E-05	Saliva_T	PRPF4B
DGUOK	3.16E-09	0.338677	0.113	0.194	5.03E-05	Saliva_T	DGUOK
PDLIM21	3.2E-09	0.319839	0.103	0.18	5.1E-05	Saliva_T	PDLIM2
JAK3	3.58E-09	0.251317	0.027	0.011	5.71E-05	Saliva_T	JAK3
MAGOH	3.59E-09	0.349113	0.102	0.179	5.73E-05	Saliva_T	MAGOH

MT.ND4L6	3.85E-09	0.657637	0.391	0.418	6.13E-05	Saliva_T	MT.ND4L
CHCHD103	4.06E-09	0.309779	0.149	0.247	6.47E-05	Saliva_T	CHCHD10
PRKAA1	4.31E-09	0.300498	0.057	0.112	6.88E-05	Saliva_T	PRKAA1
SMC5	4.46E-09	0.350186	0.058	0.114	7.11E-05	Saliva_T	SMC5
AP000769.	4.52E-09	0.375235	0.036	0.016	7.21E-05	Saliva_T	AP000769.1
EFCAB14	4.68E-09	0.319034	0.057	0.113	7.47E-05	Saliva_T	EFCAB14
CCDC88C1	4.76E-09	0.613088	0.071	0.042	7.59E-05	Saliva_T	CCDC88C
SRSF22	5.24E-09	0.457952	0.204	0.335	8.36E-05	Saliva_T	SRSF2
NDUFA121	5.31E-09	0.46271	0.166	0.277	8.46E-05	Saliva_T	NDUFA12
CTSA1	5.33E-09	0.309591	0.072	0.134	8.5E-05	Saliva_T	CTSA
IL10RA1	5.46E-09	0.503321	0.075	0.045	8.71E-05	Saliva_T	IL10RA
EPRS4	6.23E-09	0.387321	0.072	0.134	9.94E-05	Saliva_T	EPRS
SYS1	6.38E-09	0.288079	0.053	0.106	0.000102	Saliva_T	SYS1
LARS1	6.38E-09	0.354059	0.059	0.116	0.000102	Saliva_T	LARS
RSF11	6.54E-09	0.434437	0.106	0.183	0.000104	Saliva_T	RSF1
SNX5	7.43E-09	0.307181	0.057	0.112	0.000118	Saliva_T	SNX5
TC2N1	7.62E-09	0.413695	0.087	0.156	0.000121	Saliva_T	TC2N
PARK75	9.24E-09	0.272657	0.225	0.358	0.000147	Saliva_T	PARK7
RBPJ1	9.28E-09	0.436948	0.113	0.193	0.000148	Saliva_T	RBPJ
CHD94	9.3E-09	0.430022	0.096	0.169	0.000148	Saliva_T	CHD9
PHIP	9.96E-09	0.461698	0.098	0.171	0.000159	Saliva_T	PHIP
NTAN12	1.02E-08	0.292179	0.067	0.125	0.000163	Saliva_T	NTAN1
ARHGAP4	1.04E-08	0.430127	0.06	0.034	0.000165	Saliva_T	ARHGAP4
TIPRL	1.18E-08	0.313519	0.059	0.113	0.000188	Saliva_T	TIPRL
XIST5	1.2E-08	0.968245	0.159	0.123	0.000191	Saliva_T	XIST
ECHDC11	1.23E-08	0.339746	0.062	0.118	0.000196	Saliva_T	ECHDC1
HP1BP31	1.29E-08	0.488049	0.135	0.226	0.000206	Saliva_T	HP1BP3
ITSN2	1.32E-08	0.354745	0.08	0.144	0.00021	Saliva_T	ITSN2
SNRPA11	1.6E-08	0.326564	0.072	0.132	0.000256	Saliva_T	SNRPA1
C9orf78	1.68E-08	0.286144	0.103	0.175	0.000268	Saliva_T	C9orf78
MRPS6	1.68E-08	0.363439	0.108	0.183	0.000268	Saliva_T	MRPS6
RANBP2	1.71E-08	0.32424	0.062	0.118	0.000273	Saliva_T	RANBP2
RICTOR	1.85E-08	0.310984	0.057	0.11	0.000296	Saliva_T	RICTOR
CELF21	2E-08	0.839371	0.166	0.133	0.000319	Saliva_T	CELF2
TRAT1	2.06E-08	0.333946	0.047	0.025	0.000328	Saliva_T	TRAT1
NUDT51	2.08E-08	0.293911	0.059	0.113	0.000331	Saliva_T	NUDT5
CDC42	2.22E-08	0.315005	0.293	0.474	0.000355	Saliva_T	CDC42
TERF11	2.43E-08	0.256095	0.056	0.108	0.000387	Saliva_T	TERF1
LYST1	2.45E-08	0.257931	0.047	0.095	0.00039	Saliva_T	LYST
CHD1	2.52E-08	0.301715	0.074	0.133	0.000402	Saliva_T	CHD1
TNIP3	2.54E-08	0.251322	0.018	0.006	0.000406	Saliva_T	TNIP3
TAOK3	2.64E-08	0.366455	0.076	0.137	0.000421	Saliva_T	TAOK3
RAN1	2.78E-08	0.304784	0.233	0.37	0.000443	Saliva_T	RAN
UBE2M1	2.79E-08	0.380085	0.098	0.168	0.000444	Saliva_T	UBE2M
ESF11	2.8E-08	0.327375	0.062	0.117	0.000446	Saliva_T	ESF1
CNOT2	2.85E-08	0.357515	0.065	0.121	0.000454	Saliva_T	CNOT2
RPL7L12	2.91E-08	0.305949	0.064	0.119	0.000464	Saliva_T	RPL7L1

ZFYVE28	3.08E-08	0.437349	0.042	0.022	0.000491	Saliva_T	ZFYVE28
PSMA33	3.23E-08	0.46433	0.116	0.195	0.000516	Saliva_T	PSMA3
ZNF6382	3.42E-08	0.357979	0.073	0.132	0.000546	Saliva_T	ZNF638
PHKB2	3.57E-08	0.38375	0.056	0.107	0.00057	Saliva_T	PHKB
SON2	3.74E-08	0.426724	0.255	0.413	0.000597	Saliva_T	SON
USF21	4.13E-08	0.275622	0.064	0.119	0.000658	Saliva_T	USF2
ZNF800	4.22E-08	0.278702	0.052	0.101	0.000673	Saliva_T	ZNF800
CD82	4.49E-08	0.279185	0.058	0.11	0.000716	Saliva_T	CD82
RGL4	4.73E-08	0.346791	0.044	0.023	0.000755	Saliva_T	RGL4
LSM101	4.82E-08	0.267144	0.075	0.133	0.000769	Saliva_T	LSM10
PPHLN1	4.84E-08	0.368636	0.085	0.148	0.000771	Saliva_T	PPHLN1
CD37	5.35E-08	0.318208	0.248	0.203	0.000853	Saliva_T	CD37
DIS31	5.38E-08	0.269262	0.041	0.085	0.000859	Saliva_T	DIS3
FNBP11	5.49E-08	0.881755	0.159	0.126	0.000876	Saliva_T	FNBP1
SDAD11	5.54E-08	0.252979	0.042	0.087	0.000883	Saliva_T	SDAD1
LINC00649	5.93E-08	0.395969	0.042	0.021	0.000946	Saliva_T	LINC00649
TNFRSF1B1	6.52E-08	0.517922	0.076	0.048	0.001039	Saliva_T	TNFRSF1B
IVNS1ABP1	7.09E-08	0.445088	0.087	0.151	0.001131	Saliva_T	IVNS1ABP
DDX53	7.14E-08	0.588305	0.504	0.604	0.001139	Saliva_T	DDX5
ZNF644	7.69E-08	0.365157	0.066	0.12	0.001227	Saliva_T	ZNF644
USP47	8.03E-08	0.368823	0.059	0.11	0.00128	Saliva_T	USP47
UBAC2	8.42E-08	0.314445	0.057	0.106	0.001342	Saliva_T	UBAC2
METTL231	8.76E-08	0.288746	0.071	0.126	0.001397	Saliva_T	METTL23
NCOR1	9.35E-08	0.485937	0.115	0.191	0.001492	Saliva_T	NCOR1
MIS18BP1	1.1E-07	0.280483	0.055	0.104	0.001752	Saliva_T	MIS18BP1
XPO1	1.14E-07	0.281733	0.055	0.104	0.001816	Saliva_T	XPO1
ZNF1482	1.14E-07	0.364036	0.072	0.128	0.001816	Saliva_T	ZNF148
LNPEP2	1.27E-07	0.736861	0.097	0.067	0.002019	Saliva_T	LNPEP
FAM96B1	1.29E-07	0.458387	0.162	0.261	0.002062	Saliva_T	FAM96B
TRIM443	1.35E-07	0.254624	0.047	0.091	0.002156	Saliva_T	TRIM44
ERAP22	1.39E-07	0.508818	0.051	0.029	0.002212	Saliva_T	ERAP2
RBCK1	1.48E-07	0.320934	0.091	0.153	0.002354	Saliva_T	RBCK1
NCOA11	1.5E-07	0.259701	0.046	0.09	0.002388	Saliva_T	NCOA1
MORC3	1.58E-07	0.276432	0.047	0.091	0.002519	Saliva_T	MORC3
RSBN1L	1.7E-07	0.423621	0.106	0.176	0.002712	Saliva_T	RSBN1L
SCAF113	1.71E-07	0.525222	0.144	0.233	0.002731	Saliva_T	SCAF11
MPHOSPH	1.71E-07	0.43934	0.161	0.256	0.002734	Saliva_T	MPHOSPH8
SKA2	1.74E-07	0.301494	0.055	0.103	0.002771	Saliva_T	SKA2
ARID1A	2.18E-07	0.351661	0.067	0.12	0.003481	Saliva_T	ARID1A
M6PR	2.23E-07	0.367993	0.074	0.13	0.003559	Saliva_T	M6PR
TAGAP1	2.29E-07	0.441228	0.052	0.029	0.003648	Saliva_T	TAGAP
BAX	2.5E-07	0.30884	0.104	0.171	0.003986	Saliva_T	BAX
STAG21	2.59E-07	0.349376	0.062	0.112	0.004135	Saliva_T	STAG2
TLK1	2.68E-07	0.341192	0.055	0.101	0.004278	Saliva_T	TLK1
GGA1	2.69E-07	0.342022	0.068	0.12	0.004293	Saliva_T	GGA1
ADAR2	2.87E-07	0.360734	0.08	0.137	0.004582	Saliva_T	ADAR
TMED43	2.89E-07	0.468972	0.106	0.175	0.004613	Saliva_T	TMED4

MSN2	3.07E-07	0.891668	0.183	0.156	0.004898	Saliva_T	MSN
MBTPS12	3.09E-07	0.286362	0.045	0.087	0.004929	Saliva_T	MBTPS1
RBM262	3.11E-07	0.299564	0.053	0.099	0.004956	Saliva_T	RBM26
DDX27	3.12E-07	0.260914	0.053	0.099	0.004984	Saliva_T	DDX27
RBX12	3.16E-07	0.476999	0.22	0.352	0.005042	Saliva_T	RBX1
TSPAN21	3.18E-07	0.402938	0.047	0.026	0.005074	Saliva_T	TSPAN2
EFR3A	3.28E-07	0.31143	0.043	0.085	0.005228	Saliva_T	EFR3A
PCED1B	3.3E-07	0.28529	0.033	0.016	0.005268	Saliva_T	PCED1B
PCF11	3.3E-07	0.30792	0.062	0.111	0.005269	Saliva_T	PCF11
NIPBL	3.92E-07	0.470522	0.091	0.152	0.006247	Saliva_T	NIPBL
ECI22	3.95E-07	0.307511	0.052	0.097	0.006302	Saliva_T	ECI2
PLEC2	4.11E-07	0.425029	0.095	0.157	0.006548	Saliva_T	PLEC
GGA2	4.14E-07	0.252098	0.039	0.078	0.006603	Saliva_T	GGA2
RP11.356I2	4.17E-07	0.41048	0.037	0.019	0.006645	Saliva_T	RP11.356I2.4
SPG7	4.3E-07	0.258103	0.042	0.082	0.006855	Saliva_T	SPG7
GAPDH	4.58E-07	0.344267	0.653	0.766	0.00731	Saliva_T	GAPDH
CBX3	4.67E-07	0.466268	0.127	0.204	0.007442	Saliva_T	CBX3
FBXO22	4.78E-07	0.304106	0.036	0.074	0.00763	Saliva_T	FBXO22
HNRNPR2	4.79E-07	0.4529	0.12	0.194	0.007639	Saliva_T	HNRNPR
STAT33	4.88E-07	0.397804	0.12	0.192	0.007783	Saliva_T	STAT3
SUMF2	4.94E-07	0.348486	0.066	0.116	0.007876	Saliva_T	SUMF2
PRDM21	5.07E-07	0.309469	0.06	0.108	0.008085	Saliva_T	PRDM2
EIF1AY1	5.13E-07	0.269075	0.046	0.088	0.008187	Saliva_T	EIF1AY
DHX361	5.18E-07	0.464368	0.113	0.183	0.008258	Saliva_T	DHX36
UBE2S1	5.27E-07	0.360491	0.078	0.133	0.008401	Saliva_T	UBE2S
TXNIP3	5.4E-07	0.64333	0.368	0.377	0.008605	Saliva_T	TXNIP
GOLGB13	5.5E-07	0.466029	0.125	0.201	0.008775	Saliva_T	GOLGB1
CCDC12	5.83E-07	0.335706	0.101	0.164	0.009303	Saliva_T	CCDC12
GLOD4	5.99E-07	0.323326	0.07	0.121	0.009556	Saliva_T	GLOD4
FUBP1	6.02E-07	0.301818	0.055	0.101	0.009603	Saliva_T	FUBP1
TANK	6.27E-07	0.358906	0.075	0.129	0.009994	Saliva_T	TANK
GOSR11	6.48E-07	0.325159	0.055	0.1	0.01034	Saliva_T	GOSR1
KDM2A	6.53E-07	0.276048	0.045	0.086	0.010419	Saliva_T	KDM2A
RBM61	6.53E-07	0.31601	0.064	0.112	0.010422	Saliva_T	RBM6
TUBGCP2	6.57E-07	0.288881	0.048	0.09	0.010486	Saliva_T	TUBGCP2
MAP43	6.58E-07	0.332548	0.076	0.13	0.010487	Saliva_T	MAP4
ARL14EP1	6.84E-07	0.262784	0.045	0.086	0.010907	Saliva_T	ARL14EP
DNTTIP21	6.91E-07	0.295443	0.061	0.108	0.011021	Saliva_T	DNTTIP2
TTC373	7.14E-07	0.311135	0.045	0.086	0.011393	Saliva_T	TTC37
C14orf119	8.75E-07	0.372406	0.074	0.127	0.013957	Saliva_T	C14orf119
TMEM181	9.36E-07	0.272786	0.033	0.069	0.014926	Saliva_T	TMEM181
GPATCH8	9.39E-07	0.311045	0.057	0.102	0.014976	Saliva_T	GPATCH8
KANSL1	9.47E-07	0.327512	0.051	0.094	0.015096	Saliva_T	KANSL1
RBL2	1.02E-06	0.354515	0.076	0.128	0.016306	Saliva_T	RBL2
MBD21	1.04E-06	0.453292	0.084	0.14	0.016647	Saliva_T	MBD2
RGS10	1.07E-06	0.347304	0.14	0.218	0.017076	Saliva_T	RGS10
ARHGAP25	1.21E-06	0.343225	0.044	0.024	0.01925	Saliva_T	ARHGAP25

TOMM204	1.26E-06	0.474596	0.209	0.328	0.020083	Saliva_T	TOMM20
DDX244	1.35E-06	1.005549	0.246	0.238	0.02161	Saliva_T	DDX24
SNRPD31	1.36E-06	0.533407	0.148	0.234	0.021668	Saliva_T	SNRPD3
KLF61	1.38E-06	0.290542	0.271	0.405	0.021998	Saliva_T	KLF6
DPP81	1.44E-06	0.25833	0.038	0.075	0.022952	Saliva_T	DPP8
EPS15	1.47E-06	0.39027	0.064	0.111	0.02352	Saliva_T	EPS15
C8orf594	1.48E-06	0.604399	0.149	0.237	0.023561	Saliva_T	C8orf59
MED6	1.55E-06	0.254461	0.043	0.082	0.024706	Saliva_T	MED6
LRRFIP11	1.55E-06	0.448114	0.24	0.376	0.024708	Saliva_T	LRRFIP1
NUP210	1.59E-06	0.357334	0.042	0.023	0.025358	Saliva_T	NUP210
PPIL42	1.76E-06	0.355903	0.057	0.101	0.028021	Saliva_T	PPIL4
GDF11	1.76E-06	0.348498	0.03	0.015	0.028112	Saliva_T	GDF11
AKT11	1.8E-06	0.330649	0.049	0.089	0.028756	Saliva_T	AKT1
GNPTAB1	1.86E-06	0.354923	0.05	0.09	0.029624	Saliva_T	GNPTAB
CCAR1	1.87E-06	0.402284	0.06	0.105	0.029773	Saliva_T	CCAR1
DEK2	1.92E-06	0.48981	0.168	0.261	0.030601	Saliva_T	DEK
CCDC115	2E-06	0.290888	0.07	0.118	0.031882	Saliva_T	CCDC115
IAH11	2.14E-06	0.41992	0.101	0.162	0.03411	Saliva_T	IAH1
CENPC3	2.19E-06	0.734831	0.088	0.062	0.0349	Saliva_T	CENPC
INPP4A1	2.29E-06	0.486807	0.05	0.03	0.036532	Saliva_T	INPP4A
KIF21A2	2.42E-06	0.378336	0.044	0.082	0.038546	Saliva_T	KIF21A
VPS363	2.46E-06	0.36184	0.067	0.114	0.039186	Saliva_T	VPS36
TRPS1	2.56E-06	0.329802	0.049	0.089	0.040847	Saliva_T	TRPS1
KIAA11092	2.57E-06	0.297951	0.047	0.087	0.041053	Saliva_T	KIAA1109
BRIX11	2.7E-06	0.268729	0.052	0.092	0.043058	Saliva_T	BRIX1
COX6C1	2.85E-06	0.252625	0.348	0.545	0.045429	Saliva_T	COX6C
ATM1	2.92E-06	0.892699	0.118	0.091	0.0466	Saliva_T	ATM
G3BP2	2.99E-06	0.523149	0.11	0.176	0.047753	Saliva_T	G3BP2
XRN11	3.3E-06	0.326949	0.057	0.099	0.052671	Saliva_T	XRN1
RBM22	3.4E-06	0.299848	0.05	0.089	0.054201	Saliva_T	RBM22
AGGF1	3.43E-06	0.259448	0.04	0.075	0.054648	Saliva_T	AGGF1
POLR3GL	3.64E-06	0.313037	0.079	0.13	0.058113	Saliva_T	POLR3GL
POLR2B	3.8E-06	0.374247	0.057	0.099	0.060543	Saliva_T	POLR2B
CHURC13	4.03E-06	0.539046	0.14	0.218	0.064198	Saliva_T	CHURC1
MAP2K23	4.36E-06	0.476689	0.128	0.2	0.069486	Saliva_T	MAP2K2
NDUFB104	4.51E-06	0.392865	0.25	0.388	0.071937	Saliva_T	NDUFB10
STXBP3	4.8E-06	0.257378	0.045	0.081	0.076584	Saliva_T	STXBP3
PARP11	4.84E-06	0.315665	0.076	0.125	0.077158	Saliva_T	PARP1
MIA32	5.03E-06	0.326727	0.068	0.113	0.080229	Saliva_T	MIA3
GPR68	5.05E-06	0.272278	0.023	0.01	0.08051	Saliva_T	GPR68
SEC22C	5.27E-06	0.291736	0.043	0.079	0.084042	Saliva_T	SEC22C
ADCY7	5.35E-06	0.336322	0.035	0.019	0.08532	Saliva_T	ADCY7
SSB3	5.41E-06	0.414055	0.129	0.2	0.086328	Saliva_T	SSB
S1PR4	5.64E-06	0.337909	0.083	0.056	0.090029	Saliva_T	S1PR4
SENP61	5.7E-06	0.364973	0.065	0.11	0.090922	Saliva_T	SENP6
PHF11	6.08E-06	0.306994	0.07	0.116	0.096949	Saliva_T	PHF1
CDK6	6.42E-06	0.3451	0.047	0.085	0.102437	Saliva_T	CDK6

DHRS73	6.74E-06	0.450795	0.137	0.21	0.107425	Saliva_T	DHRS7
FRYL1	7.26E-06	0.268644	0.052	0.09	0.115742	Saliva_T	FRYL
PRPF40A1	7.4E-06	0.566419	0.121	0.189	0.11797	Saliva_T	PRPF40A
SFMBT2	7.5E-06	0.26657	0.022	0.01	0.119634	Saliva_T	SFMBT2
FNBP41	7.59E-06	0.44511	0.087	0.14	0.121033	Saliva_T	FNBP4
CCNH	7.85E-06	0.323592	0.07	0.115	0.125161	Saliva_T	CCNH
CDC42SE2:	8.05E-06	0.860728	0.144	0.12	0.128467	Saliva_T	CDC42SE2
ABHD14A2	8.53E-06	0.318391	0.062	0.104	0.136031	Saliva_T	ABHD14A
TMEM223	9.48E-06	0.263156	0.04	0.073	0.15119	Saliva_T	TMEM223
TOP2B	9.99E-06	0.307824	0.052	0.09	0.159256	Saliva_T	TOP2B
PDCD71	1.02E-05	0.261561	0.053	0.091	0.163189	Saliva_T	PDCD7
ITPR22	1.03E-05	0.270762	0.046	0.082	0.163989	Saliva_T	ITPR2
TAF101	1.07E-05	0.496261	0.115	0.178	0.171188	Saliva_T	TAF10
CEP3501	1.11E-05	0.325218	0.081	0.129	0.176737	Saliva_T	CEP350
EXOSC11	1.2E-05	0.292613	0.055	0.093	0.191844	Saliva_T	EXOSC1
ZNRD1	1.22E-05	0.392108	0.09	0.142	0.194048	Saliva_T	ZNRD1
GIMAP11	1.22E-05	0.438571	0.084	0.059	0.19475	Saliva_T	GIMAP1
LINC00861	1.32E-05	0.307306	0.04	0.023	0.211305	Saliva_T	LINC00861
SUB11	1.35E-05	0.798196	0.398	0.482	0.21464	Saliva_T	SUB1
NFATC3	1.35E-05	0.472147	0.071	0.116	0.215819	Saliva_T	NFATC3
YWHAB2	1.36E-05	0.878619	0.356	0.414	0.217342	Saliva_T	YWHAB
LSP1	1.38E-05	0.518147	0.25	0.231	0.220184	Saliva_T	LSP1
PACS1	1.41E-05	0.266479	0.042	0.076	0.225232	Saliva_T	PACS1
EIF2AK23	1.42E-05	0.336882	0.057	0.097	0.227112	Saliva_T	EIF2AK2
LSM6	1.46E-05	0.408316	0.091	0.143	0.232453	Saliva_T	LSM6
POP51	1.46E-05	0.322618	0.059	0.098	0.232777	Saliva_T	POP5
AGTRAP1	1.49E-05	0.314581	0.067	0.109	0.237941	Saliva_T	AGTRAP
AES	1.54E-05	0.313578	0.268	0.398	0.245859	Saliva_T	AES
NPIPB5	1.61E-05	0.43014	0.038	0.022	0.257197	Saliva_T	NPIPB5
KAT6B1	1.71E-05	0.28856	0.044	0.078	0.272205	Saliva_T	KAT6B
IFITM22	1.76E-05	0.463057	0.314	0.306	0.28057	Saliva_T	IFITM2
WHSC1L12	1.77E-05	0.521021	0.129	0.196	0.282354	Saliva_T	WHSC1L1
BIN2	1.78E-05	0.4653	0.104	0.078	0.283327	Saliva_T	BIN2
BTG1	1.9E-05	0.700213	0.498	0.579	0.302258	Saliva_T	BTG1
DOCK111	1.95E-05	0.391574	0.04	0.023	0.311739	Saliva_T	DOCK11
LSM7	1.96E-05	0.41453	0.193	0.288	0.312007	Saliva_T	LSM7
RIF12	1.96E-05	0.333376	0.055	0.093	0.312358	Saliva_T	RIF1
PRR41	2.16E-05	0.542624	0.138	0.111	0.344525	Saliva_T	PRR4
CDK12	2.17E-05	0.3885	0.064	0.105	0.345902	Saliva_T	CDK12
SNRNP200	2.21E-05	0.413239	0.063	0.104	0.352902	Saliva_T	SNRNP200
ZBTB8OS1	2.25E-05	0.493726	0.084	0.132	0.358587	Saliva_T	ZBTB8OS
MECP2	2.25E-05	0.34536	0.052	0.089	0.358927	Saliva_T	MECP2
CRBN1	2.41E-05	0.406099	0.093	0.144	0.384551	Saliva_T	CRBN
BTN3A12	2.46E-05	0.409862	0.039	0.023	0.392049	Saliva_T	BTN3A1
RNF216	2.46E-05	0.282686	0.051	0.087	0.392915	Saliva_T	RNF216
PIK3CD	2.48E-05	0.267784	0.032	0.017	0.39568	Saliva_T	PIK3CD
RASSF1	2.49E-05	0.271889	0.056	0.093	0.396641	Saliva_T	RASSF1

PJA24	2.64E-05	0.501683	0.098	0.153	0.420274	Saliva_T	PJA2
COMMD2	2.66E-05	0.325203	0.059	0.097	0.423683	Saliva_T	COMMD2
TCF7	2.76E-05	0.465742	0.07	0.048	0.439786	Saliva_T	TCF7
PHF32	2.79E-05	0.520722	0.085	0.134	0.445394	Saliva_T	PHF3
PTCD31	2.85E-05	0.287118	0.038	0.069	0.455051	Saliva_T	PTCD3
XPA1	2.91E-05	0.27372	0.052	0.088	0.464401	Saliva_T	XPA
XRCC52	2.95E-05	0.558232	0.165	0.249	0.469736	Saliva_T	XRCC5
B3GALT4	3.01E-05	0.286898	0.044	0.077	0.480179	Saliva_T	B3GALT4
PCSK71	3.04E-05	0.783673	0.135	0.112	0.485492	Saliva_T	PCSK7
C9orf142	3.21E-05	0.866647	0.166	0.146	0.511688	Saliva_T	C9orf142
SCAPER	3.38E-05	0.253437	0.038	0.068	0.53874	Saliva_T	SCAPER
CYB561D2	3.44E-05	0.250337	0.04	0.071	0.548817	Saliva_T	CYB561D2
IREB2	3.53E-05	0.271867	0.043	0.075	0.563135	Saliva_T	IREB2
UHMK1	3.67E-05	0.371258	0.055	0.092	0.585441	Saliva_T	UHMK1
LIMD2	3.73E-05	0.341882	0.207	0.174	0.594657	Saliva_T	LIMD2
PRPF8	3.82E-05	0.329365	0.063	0.102	0.609361	Saliva_T	PRPF8
NAP1L4	3.84E-05	0.56742	0.109	0.167	0.612008	Saliva_T	NAP1L4
USP162	3.88E-05	0.572484	0.118	0.18	0.618933	Saliva_T	USP16
ZC3H61	3.89E-05	0.262947	0.04	0.071	0.620035	Saliva_T	ZC3H6
EVI2A1	3.95E-05	0.451132	0.057	0.037	0.630385	Saliva_T	EVI2A
METTL51	3.97E-05	0.352206	0.078	0.122	0.632409	Saliva_T	METTL5
CD58	3.98E-05	0.259216	0.046	0.079	0.63421	Saliva_T	CD58
ARHGEF6	4.34E-05	0.34137	0.035	0.02	0.692302	Saliva_T	ARHGEF6
PPP1R12A	4.73E-05	0.482651	0.119	0.179	0.754415	Saliva_T	PPP1R12A
THOC21	4.88E-05	0.432823	0.081	0.127	0.777652	Saliva_T	THOC2
ILF3.AS1	4.97E-05	0.292417	0.068	0.107	0.792088	Saliva_T	ILF3.AS1
AHCTF1	5.11E-05	0.25106	0.031	0.058	0.814446	Saliva_T	AHCTF1
C4orf48	5.15E-05	0.378185	0.095	0.144	0.820762	Saliva_T	C4orf48
MRPL541	5.5E-05	0.55581	0.141	0.211	0.877991	Saliva_T	MRPL54
RSRC11	5.63E-05	0.34526	0.057	0.093	0.898569	Saliva_T	RSRC1
MBD41	5.76E-05	0.376167	0.059	0.096	0.918693	Saliva_T	MBD4
PARVG	5.77E-05	0.366704	0.048	0.031	0.920703	Saliva_T	PARVG
PSME1	6.47E-05	0.297095	0.267	0.386	1	Saliva_T	PSME1
TTC39C	6.76E-05	0.322261	0.064	0.102	1	Saliva_T	TTC39C
SPG11	6.95E-05	0.31582	0.042	0.072	1	Saliva_T	SPG11
HNRNPU.A	6.95E-05	0.253535	0.036	0.064	1	Saliva_T	HNRNPU.AS1
CUL51	7E-05	0.327049	0.047	0.079	1	Saliva_T	CUL5
ATXN7L3B	7.12E-05	0.259853	0.043	0.074	1	Saliva_T	ATXN7L3B
PLA2G163	8.01E-05	0.390374	0.082	0.126	1	Saliva_T	PLA2G16
TBCB	8.17E-05	0.432624	0.138	0.204	1	Saliva_T	TBCB
ASH1L1	8.35E-05	0.586742	0.113	0.171	1	Saliva_T	ASH1L
BAZ1B2	8.94E-05	0.314532	0.051	0.084	1	Saliva_T	BAZ1B
VASP	9.63E-05	0.357992	0.086	0.131	1	Saliva_T	VASP
SYNE34	9.69E-05	0.277466	0.032	0.018	1	Saliva_T	SYNE3
TMX41	9.79E-05	0.407277	0.109	0.161	1	Saliva_T	TMX4
SFPQ3	0.000102	0.563349	0.193	0.288	1	Saliva_T	SFPQ
PIK3R11	0.000107	0.882154	0.131	0.111	1	Saliva_T	PIK3R1

C19orf602	0.000108	0.439562	0.158	0.231	1	Saliva_T	C19orf60
RPL36AL1	0.000111	0.339481	0.574	0.733	1	Saliva_T	RPL36AL
KLHL36	0.000112	0.258963	0.038	0.065	1	Saliva_T	KLHL36
SMARCA52	0.000113	0.47435	0.086	0.131	1	Saliva_T	SMARCA5
CTCF	0.000113	0.326304	0.044	0.074	1	Saliva_T	CTCF
CHD3	0.000114	0.335294	0.055	0.088	1	Saliva_T	CHD3
WDR82	0.000116	0.378034	0.067	0.104	1	Saliva_T	WDR82
BIRC6	0.000118	0.482485	0.072	0.111	1	Saliva_T	BIRC6
ASB81	0.000119	0.300841	0.045	0.075	1	Saliva_T	ASB8
USP33	0.000122	0.356516	0.051	0.083	1	Saliva_T	USP33
YTHDC2	0.000124	0.279031	0.04	0.068	1	Saliva_T	YTHDC2
COX8A	0.000133	0.424364	0.288	0.439	1	Saliva_T	COX8A
RYK2	0.000134	0.336176	0.045	0.076	1	Saliva_T	RYK
SUPT20H	0.000135	0.328209	0.05	0.081	1	Saliva_T	SUPT20H
TBCC	0.000136	0.275494	0.062	0.097	1	Saliva_T	TBCC
PPIP5K2	0.000138	0.276003	0.04	0.069	1	Saliva_T	PPIP5K2
GSK3A	0.000143	0.261597	0.04	0.068	1	Saliva_T	GSK3A
NECAP1	0.000144	0.252801	0.033	0.059	1	Saliva_T	NECAP1
PURA3	0.000146	0.528065	0.083	0.126	1	Saliva_T	PURA
ARID4A1	0.000148	0.427957	0.071	0.11	1	Saliva_T	ARID4A
PAK21	0.000148	0.555897	0.123	0.183	1	Saliva_T	PAK2
FAM35A	0.00015	0.251558	0.03	0.055	1	Saliva_T	FAM35A
TAPBP2	0.000151	0.867415	0.178	0.164	1	Saliva_T	TAPBP
ITGB1BP12	0.000167	0.434378	0.103	0.153	1	Saliva_T	ITGB1BP1
PKN1	0.000168	0.262484	0.057	0.091	1	Saliva_T	PKN1
ZNF107	0.000168	0.38098	0.033	0.02	1	Saliva_T	ZNF107
OGT1	0.00018	0.381916	0.069	0.107	1	Saliva_T	OGT
HTATSF1	0.000181	0.258427	0.055	0.088	1	Saliva_T	HTATSF1
CAPG	0.000184	0.32196	0.116	0.168	1	Saliva_T	CAPG
NUB1	0.000184	0.339974	0.066	0.102	1	Saliva_T	NUB1
PYROXD1	0.000192	0.270907	0.032	0.057	1	Saliva_T	PYROXD1
ANXA2R	0.000195	0.340067	0.054	0.036	1	Saliva_T	ANXA2R
PHF142	0.000202	0.479631	0.084	0.126	1	Saliva_T	PHF14
SORL1	0.000206	0.274826	0.044	0.072	1	Saliva_T	SORL1
DDX174	0.000207	0.938027	0.312	0.355	1	Saliva_T	DDX17
AHSA2	0.000209	0.289295	0.044	0.073	1	Saliva_T	AHSA2
PPP6R1	0.000218	0.296561	0.05	0.08	1	Saliva_T	PPP6R1
LARP71	0.000224	0.493359	0.09	0.135	1	Saliva_T	LARP7
THRAP33	0.000231	0.525417	0.12	0.177	1	Saliva_T	THRAP3
TMEM71	0.000237	0.311616	0.028	0.016	1	Saliva_T	TMEM71
ANKRD13D	0.000245	0.727338	0.089	0.07	1	Saliva_T	ANKRD13D
USP15	0.000249	0.577899	0.106	0.156	1	Saliva_T	USP15
ST8SIA41	0.000269	0.378654	0.043	0.028	1	Saliva_T	ST8SIA4
RNASEH2B	0.000272	0.321939	0.069	0.105	1	Saliva_T	RNASEH2B
GNG2	0.00029	0.446551	0.091	0.07	1	Saliva_T	GNG2
NR3C12	0.000309	0.486178	0.102	0.15	1	Saliva_T	NR3C1
R3HDM2	0.000316	0.314637	0.05	0.08	1	Saliva_T	R3HDM2

LYAR	0.000318	0.60251	0.115	0.094	1	Saliva_T	LYAR
TOMM7	0.000328	0.469246	0.477	0.584	1	Saliva_T	TOMM7
LINC00152	0.000335	0.300791	0.126	0.18	1	Saliva_T	LINC00152
FAM89B1	0.000348	0.503287	0.121	0.176	1	Saliva_T	FAM89B
CEP85L	0.00036	0.291922	0.054	0.084	1	Saliva_T	CEP85L
DUS1L2	0.000368	0.444321	0.064	0.098	1	Saliva_T	DUS1L
BRD9	0.000377	0.319455	0.046	0.074	1	Saliva_T	BRD9
HELZ	0.000402	0.367202	0.053	0.083	1	Saliva_T	HELZ
RBM331	0.000408	0.401995	0.056	0.087	1	Saliva_T	RBM33
RAB30.AS1	0.000412	0.256483	0.036	0.061	1	Saliva_T	RAB30.AS1
KAT6A1	0.000414	0.30389	0.048	0.077	1	Saliva_T	KAT6A
PTPN1	0.000417	0.324185	0.053	0.083	1	Saliva_T	PTPN1
PLP22	0.00042	0.755373	0.233	0.232	1	Saliva_T	PLP2
TBCD	0.000434	0.273392	0.038	0.062	1	Saliva_T	TBCD
GSDMD1	0.000441	0.277162	0.067	0.1	1	Saliva_T	GSDMD
CD441	0.000443	0.563791	0.331	0.349	1	Saliva_T	CD44
TAF3	0.00046	0.270595	0.038	0.063	1	Saliva_T	TAF3
LSM21	0.000461	0.39407	0.118	0.17	1	Saliva_T	LSM2
DAXX	0.000474	0.332851	0.05	0.078	1	Saliva_T	DAXX
CYTH4	0.000474	0.328962	0.045	0.03	1	Saliva_T	CYTH4
C1orf186	0.000504	0.284011	0.014	0.007	1	Saliva_T	C1orf186
TTC143	0.00051	0.80804	0.12	0.102	1	Saliva_T	TTC14
KIAA2026	0.000511	0.435432	0.067	0.101	1	Saliva_T	KIAA2026
INO80D	0.000523	0.260153	0.038	0.063	1	Saliva_T	INO80D
DDX183	0.000533	0.549242	0.143	0.206	1	Saliva_T	DDX18
GPSM3	0.000536	0.33565	0.197	0.173	1	Saliva_T	GPSM3
PSMA73	0.000546	0.431028	0.312	0.467	1	Saliva_T	PSMA7
AAK1	0.00056	0.831683	0.129	0.112	1	Saliva_T	AAK1
ARAP2	0.000591	0.761312	0.088	0.07	1	Saliva_T	ARAP2
TPSAB1	0.000598	1.133219	0.021	0.011	1	Saliva_T	TPSAB1
TMEM432	0.000607	0.252458	0.044	0.07	1	Saliva_T	TMEM43
POLR3K	0.000633	0.259198	0.044	0.07	1	Saliva_T	POLR3K
LYRM2	0.000634	0.423929	0.062	0.094	1	Saliva_T	LYRM2
KLF12	0.000641	0.29387	0.031	0.019	1	Saliva_T	KLF12
SEL1L31	0.000654	0.52142	0.064	0.047	1	Saliva_T	SEL1L3
PTAR1	0.000667	0.324068	0.04	0.065	1	Saliva_T	PTAR1
PPIG3	0.000687	0.641491	0.174	0.251	1	Saliva_T	PPIG
SUMO22	0.00072	0.290644	0.393	0.605	1	Saliva_T	SUMO2
RASSF2	0.000722	0.325478	0.036	0.023	1	Saliva_T	RASSF2
IDS2	0.000747	0.573509	0.13	0.187	1	Saliva_T	IDS
SSBP43	0.000765	0.723875	0.12	0.102	1	Saliva_T	SSBP4
TNFAIP81	0.000771	0.660034	0.123	0.105	1	Saliva_T	TNFAIP8
MED101	0.000836	0.531751	0.13	0.186	1	Saliva_T	MED10
ANTXR22	0.000838	0.393526	0.048	0.033	1	Saliva_T	ANTXR2
ARHGAP18	0.000858	0.463393	0.064	0.097	1	Saliva_T	ARHGAP18
GLIPR21	0.000859	0.61671	0.103	0.085	1	Saliva_T	GLIPR2
RINL	0.000867	0.290104	0.031	0.019	1	Saliva_T	RINL

ELK41	0.000883	0.333369	0.044	0.069	1	Saliva_T	ELK4
SHISA51	0.000885	0.281132	0.072	0.105	1	Saliva_T	SHISA5
CIB1	0.000907	0.458139	0.244	0.353	1	Saliva_T	CIB1
NIP7	0.000921	0.270187	0.038	0.062	1	Saliva_T	NIP7
SRFBP12	0.000972	0.260585	0.039	0.063	1	Saliva_T	SRFBP1
PNKP1	0.001045	0.344819	0.052	0.08	1	Saliva_T	PNKP
CCDC57	0.001068	0.257903	0.053	0.08	1	Saliva_T	CCDC57
LUC7L22	0.001095	0.273877	0.031	0.052	1	Saliva_T	LUC7L2
RPS4Y12	0.001142	0.699861	0.265	0.274	1	Saliva_T	RPS4Y1
ZYX1	0.001189	0.359634	0.081	0.117	1	Saliva_T	ZYX
MSI21	0.00119	0.305582	0.046	0.071	1	Saliva_T	MSI2
VPS13C2	0.001199	0.812997	0.109	0.093	1	Saliva_T	VPS13C
GPATCH2L	0.001216	0.385324	0.055	0.083	1	Saliva_T	GPATCH2L
POU2F21	0.001232	0.45923	0.064	0.047	1	Saliva_T	POU2F2
ATP2B43	0.001251	0.656994	0.097	0.08	1	Saliva_T	ATP2B4
KMT2A3	0.001262	0.907396	0.143	0.131	1	Saliva_T	KMT2A
ST8SIA1	0.001274	0.280286	0.023	0.014	1	Saliva_T	ST8SIA1
DAD14	0.001285	0.553126	0.271	0.402	1	Saliva_T	DAD1
TPM31	0.001326	0.846659	0.296	0.333	1	Saliva_T	TPM3
SYNE13	0.001354	0.508399	0.074	0.057	1	Saliva_T	SYNE1
BCLAF13	0.001364	0.657321	0.141	0.201	1	Saliva_T	BCLAF1
ASCC31	0.001386	0.285596	0.038	0.061	1	Saliva_T	ASCC3
U2SURP3	0.001402	0.504838	0.123	0.173	1	Saliva_T	U2SURP
PCNXL21	0.001402	0.276281	0.027	0.016	1	Saliva_T	PCNXL2
RNF1251	0.00141	0.44186	0.051	0.036	1	Saliva_T	RNF125
KIN1	0.001415	0.273799	0.041	0.064	1	Saliva_T	KIN
RBM253	0.001439	0.639041	0.169	0.241	1	Saliva_T	RBM25
XPC3	0.001461	0.268105	0.039	0.062	1	Saliva_T	XPC
ARGLU12	0.001505	0.617595	0.215	0.311	1	Saliva_T	ARGLU1
VPS13A	0.001526	0.340081	0.047	0.073	1	Saliva_T	VPS13A
PPM1K1	0.001548	0.363608	0.069	0.1	1	Saliva_T	PPM1K
SMC1A1	0.001549	0.341705	0.049	0.074	1	Saliva_T	SMC1A
ZC3H132	0.001654	0.47138	0.086	0.123	1	Saliva_T	ZC3H13
ERN11	0.001705	0.264424	0.037	0.058	1	Saliva_T	ERN1
NFATC2IP	0.001706	0.379362	0.054	0.081	1	Saliva_T	NFATC2IP
PSMA52	0.001723	0.583802	0.13	0.184	1	Saliva_T	PSMA5
IRF31	0.001785	0.361978	0.069	0.101	1	Saliva_T	IRF3
DENND1B	0.001831	0.389158	0.046	0.07	1	Saliva_T	DENND1B
CHD6	0.00185	0.318186	0.053	0.079	1	Saliva_T	CHD6
SSBP14	0.001861	0.656374	0.198	0.285	1	Saliva_T	SSBP1
RCAN3	0.00189	0.636568	0.08	0.064	1	Saliva_T	RCAN3
ZNF721	0.001891	0.320264	0.043	0.067	1	Saliva_T	ZNF721
APRT1	0.001949	0.673678	0.326	0.371	1	Saliva_T	APRT
FAM107B1	0.002007	0.494596	0.212	0.3	1	Saliva_T	FAM107B
APOL6	0.00203	0.352975	0.053	0.079	1	Saliva_T	APOL6
RBM12	0.002117	0.253447	0.033	0.053	1	Saliva_T	RBM12
WAS	0.002184	0.384258	0.059	0.044	1	Saliva_T	WAS

TUBA4A	0.002267	0.449153	0.123	0.17	1	Saliva_T	TUBA4A
CPNE11	0.002281	0.390924	0.092	0.129	1	Saliva_T	CPNE1
RAB8B	0.002307	0.406661	0.068	0.098	1	Saliva_T	RAB8B
MGAT4A	0.002327	0.422363	0.073	0.104	1	Saliva_T	MGAT4A
FAM172A1	0.002404	0.302389	0.044	0.067	1	Saliva_T	FAM172A
ANKRD11	0.002442	0.608032	0.135	0.19	1	Saliva_T	ANKRD11
IFI27L22	0.002472	0.388427	0.101	0.141	1	Saliva_T	IFI27L2
NHLRC31	0.002491	0.345633	0.049	0.073	1	Saliva_T	NHLRC3
ZNF720	0.002526	0.275101	0.035	0.056	1	Saliva_T	ZNF720
MZT2B2	0.002527	0.392542	0.316	0.464	1	Saliva_T	MZT2B
MAST3	0.002624	0.255444	0.025	0.015	1	Saliva_T	MAST3
TRPM7	0.00264	0.253556	0.033	0.053	1	Saliva_T	TRPM7
MTRNR2L1	0.002647	0.326758	0.117	0.098	1	Saliva_T	MTRNR2L10
ZNF2921	0.002661	0.718802	0.123	0.174	1	Saliva_T	ZNF292
ZBTB7A1	0.002724	0.481176	0.088	0.124	1	Saliva_T	ZBTB7A
TPR2	0.002737	0.57755	0.147	0.204	1	Saliva_T	TPR
THUMPD1	0.002845	0.492617	0.072	0.103	1	Saliva_T	THUMPD1
C17orf621	0.002846	0.307064	0.064	0.092	1	Saliva_T	C17orf62
GPR155	0.002883	0.372575	0.04	0.028	1	Saliva_T	GPR155
KCNAB2	0.002888	0.304147	0.037	0.025	1	Saliva_T	KCNAB2
HINT12	0.003015	0.425712	0.468	0.572	1	Saliva_T	HINT1
TGOLN24	0.003041	0.603926	0.124	0.173	1	Saliva_T	TGOLN2
RABAC15	0.003134	0.319976	0.288	0.407	1	Saliva_T	RABAC1
FAM49B	0.003227	0.536615	0.125	0.174	1	Saliva_T	FAM49B
PHF6	0.003237	0.287426	0.036	0.056	1	Saliva_T	PHF6
MDFIC1	0.003303	0.345836	0.052	0.077	1	Saliva_T	MDFIC
COMMD71	0.003309	0.488418	0.093	0.13	1	Saliva_T	COMMD7
MYO9B	0.003321	0.28936	0.047	0.07	1	Saliva_T	MYO9B
NSD13	0.003397	0.458107	0.072	0.102	1	Saliva_T	NSD1
TRAPPC10	0.003492	0.276889	0.039	0.06	1	Saliva_T	TRAPPC10
ZNF37A4	0.003549	0.303346	0.037	0.057	1	Saliva_T	ZNF37A
NCOA2	0.003554	0.250924	0.032	0.05	1	Saliva_T	NCOA2
KAT7	0.00358	0.273222	0.035	0.055	1	Saliva_T	KAT7
IL161	0.003637	0.368158	0.069	0.053	1	Saliva_T	IL16
SLC38A12	0.003674	0.798305	0.113	0.099	1	Saliva_T	SLC38A1
PLEKHF1	0.003776	0.338635	0.057	0.082	1	Saliva_T	PLEKHF1
PPWD1	0.003808	0.259738	0.033	0.051	1	Saliva_T	PPWD1
ZBTB1	0.003865	0.325256	0.051	0.075	1	Saliva_T	ZBTB1
ZNF276	0.00395	0.406267	0.047	0.035	1	Saliva_T	ZNF276
RCSD1	0.003958	0.345334	0.068	0.053	1	Saliva_T	RCSD1
ZNF131	0.004002	0.385822	0.058	0.084	1	Saliva_T	ZNF131
ITGB71	0.004121	0.372365	0.051	0.038	1	Saliva_T	ITGB7
ZDHHC24	0.004159	0.28544	0.039	0.059	1	Saliva_T	ZDHHC24
DGKZ	0.004168	0.535189	0.069	0.054	1	Saliva_T	DGKZ
SNHG121	0.004236	0.32095	0.057	0.083	1	Saliva_T	SNHG12
NT5C1	0.004301	0.443692	0.091	0.126	1	Saliva_T	NT5C
SF3B12	0.004567	0.738662	0.181	0.255	1	Saliva_T	SF3B1

TCIRG1	0.004601	0.288768	0.051	0.074	1	Saliva_T	TCIRG1
LINC.PINT	0.004644	0.444652	0.057	0.043	1	Saliva_T	LINC.PINT
SEMA4D	0.004674	0.433378	0.052	0.039	1	Saliva_T	SEMA4D
FAM129A4	0.004714	0.756956	0.117	0.104	1	Saliva_T	FAM129A
ZNF830	0.004753	0.290425	0.043	0.064	1	Saliva_T	ZNF830
DNAJC212	0.004756	0.550064	0.087	0.122	1	Saliva_T	DNAJC21
KDM5A	0.004761	0.540338	0.088	0.123	1	Saliva_T	KDM5A
NR4A2	0.004766	0.423022	0.14	0.186	1	Saliva_T	NR4A2
SOS11	0.004901	0.346965	0.04	0.061	1	Saliva_T	SOS1
ANKRD49	0.004953	0.606611	0.066	0.052	1	Saliva_T	ANKRD49
FUS3	0.004995	0.889588	0.254	0.278	1	Saliva_T	FUS
METTL14	0.005095	0.254053	0.028	0.044	1	Saliva_T	METTL14
INTS63	0.005156	0.548028	0.1	0.138	1	Saliva_T	INTS6
SH3BP1	0.005161	0.501333	0.069	0.055	1	Saliva_T	SH3BP1
CDC401	0.005371	0.450528	0.074	0.103	1	Saliva_T	CDC40
ANKZF1	0.005548	0.286044	0.035	0.054	1	Saliva_T	ANKZF1
ZNF7081	0.005558	0.321872	0.03	0.02	1	Saliva_T	ZNF708
COX19	0.005977	0.328916	0.056	0.08	1	Saliva_T	COX19
MIER12	0.005984	0.549398	0.09	0.125	1	Saliva_T	MIER1
HSPB111	0.006047	0.574372	0.098	0.135	1	Saliva_T	HSPB11
CXorf38	0.006152	0.268598	0.033	0.05	1	Saliva_T	CXorf38
ADAM8	0.006248	0.321406	0.04	0.029	1	Saliva_T	ADAM8
NSUN61	0.006708	0.273066	0.035	0.053	1	Saliva_T	NSUN6
ITFG2	0.006717	0.250491	0.03	0.046	1	Saliva_T	ITFG2
FLNA3	0.006727	0.658539	0.159	0.15	1	Saliva_T	FLNA
UBR2	0.006816	0.309924	0.045	0.066	1	Saliva_T	UBR2
ZDHHC18	0.006956	0.257434	0.03	0.047	1	Saliva_T	ZDHHC18
SMCHD11	0.00703	0.828965	0.133	0.124	1	Saliva_T	SMCHD1
LPXN	0.007077	0.462808	0.06	0.047	1	Saliva_T	LPXN
ZCCHC11	0.007195	0.414068	0.064	0.089	1	Saliva_T	ZCCHC11
SP1002	0.007918	0.770089	0.147	0.14	1	Saliva_T	SP100
NKTR4	0.008171	0.963499	0.176	0.176	1	Saliva_T	NKTR
NIN	0.008264	0.253306	0.039	0.057	1	Saliva_T	NIN
SLC35E2B1	0.008368	0.252491	0.03	0.047	1	Saliva_T	SLC35E2B
ESCO11	0.008376	0.411694	0.06	0.085	1	Saliva_T	ESCO1
PGGT1B	0.008686	0.310194	0.033	0.05	1	Saliva_T	PGGT1B
SEPT1	0.009124	0.474405	0.113	0.1	1	Saliva_T	SEPT1
PBXIP11	0.009145	0.724899	0.125	0.115	1	Saliva_T	PBXIP1
PCNXL42	0.00917	0.339981	0.041	0.06	1	Saliva_T	PCNXL4
ARHGDIA2	0.009248	0.479261	0.147	0.198	1	Saliva_T	ARHGDIA
ERICH11	0.009889	0.488903	0.078	0.107	1	Saliva_T	ERICH1
LRBA	0.009956	0.353913	0.046	0.066	1	Saliva_T	LRBA
TFF3	0	5.794082	0.995	0.123	0	Saliva_Acir	TFF3
AGR2	0	5.45647	0.994	0.133	0	Saliva_Acir	AGR2
FCGBP	0	3.622953	0.89	0.127	0	Saliva_Acir	FCGBP
SCGB3A11	0	3.532732	0.833	0.126	0	Saliva_Acir	SCGB3A1
GOLM1	0	3.280301	0.908	0.073	0	Saliva_Acir	GOLM1

AZGP12	0	3.184836	0.994	0.19	0	Saliva_Acir	AZGP1
NKX3.1	0	2.669764	0.776	0.021	0	Saliva_Acir	NKX3.1
TSPAN8	0	2.639528	0.843	0.05	0	Saliva_Acir	TSPAN8
SPDEF1	0	2.553519	0.821	0.047	0	Saliva_Acir	SPDEF
FXYD32	0	2.500352	0.921	0.218	0	Saliva_Acir	FXYD3
SLC12A21	0	2.465215	0.831	0.097	0	Saliva_Acir	SLC12A2
SLC26A21	0	2.344671	0.816	0.062	0	Saliva_Acir	SLC26A2
MT.ND37	0	2.315447	0.999	0.79	0	Saliva_Acir	MT.ND3
MIA33	0	2.253765	0.821	0.097	0	Saliva_Acir	MIA3
PRSS231	0	2.19426	0.803	0.079	0	Saliva_Acir	PRSS23
GALNT7	0	2.190343	0.791	0.048	0	Saliva_Acir	GALNT7
FAM3D2	0	2.184397	0.936	0.225	0	Saliva_Acir	FAM3D
XBP13	0	2.158137	0.946	0.33	0	Saliva_Acir	XBP1
KIAA13242	0	2.104398	0.85	0.093	0	Saliva_Acir	KIAA1324
TM9SF3	0	2.095205	0.86	0.222	0	Saliva_Acir	TM9SF3
MT.ATP67	0	2.036396	0.999	0.896	0	Saliva_Acir	MT.ATP6
MSLN	0	2.010082	0.276	0.03	0	Saliva_Acir	MSLN
TSPAN1	0	1.996471	0.856	0.125	0	Saliva_Acir	TSPAN1
CPE2	0	1.964286	0.824	0.087	0	Saliva_Acir	CPE
C6orf582	0	1.955564	0.925	0.202	0	Saliva_Acir	C6orf58
MT.CYB7	0	1.925605	0.999	0.932	0	Saliva_Acir	MT.CYB
MT.CO27	0	1.907559	0.998	0.959	0	Saliva_Acir	MT.CO2
CDC42EP5	0	1.893869	0.822	0.184	0	Saliva_Acir	CDC42EP5
DNAJC101	0	1.886775	0.774	0.104	0	Saliva_Acir	DNAJC10
MT.ND47	0	1.88022	0.998	0.953	0	Saliva_Acir	MT.ND4
TSPAN132	0	1.878896	0.788	0.144	0	Saliva_Acir	TSPAN13
FAM3B	0	1.857634	0.676	0.061	0	Saliva_Acir	FAM3B
MT.ND57	0	1.853425	0.984	0.697	0	Saliva_Acir	MT.ND5
HDLBP3	0	1.853196	0.839	0.249	0	Saliva_Acir	HDLBP
STARD10	0	1.84639	0.805	0.124	0	Saliva_Acir	STARD10
P4HB2	0	1.800005	0.913	0.409	0	Saliva_Acir	P4HB
CPD1	0	1.793052	0.757	0.107	0	Saliva_Acir	CPD
TTC33	0	1.778325	0.836	0.221	0	Saliva_Acir	TTC3
MTRNR2L1	0	1.763305	0.861	0.257	0	Saliva_Acir	MTRNR2L12
TIMP13	0	1.758124	0.928	0.37	0	Saliva_Acir	TIMP1
GALNT6	0	1.735743	0.682	0.034	0	Saliva_Acir	GALNT6
GALNT12	0	1.724656	0.676	0.019	0	Saliva_Acir	GALNT12
GLYATL2	0	1.723544	0.636	0.011	0	Saliva_Acir	GLYATL2
KRT192	0	1.717785	0.798	0.108	0	Saliva_Acir	KRT19
MT.ND27	0	1.701698	0.993	0.905	0	Saliva_Acir	MT.ND2
GFPT12	0	1.699228	0.732	0.115	0	Saliva_Acir	GFPT1
MT.ND4L7	0	1.689696	0.941	0.406	0	Saliva_Acir	MT.ND4L
MT.ND17	0	1.676214	0.997	0.926	0	Saliva_Acir	MT.ND1
NPDC13	0	1.653354	0.819	0.159	0	Saliva_Acir	NPDC1
TSPAN32	0	1.653065	0.747	0.159	0	Saliva_Acir	TSPAN3
CREB3L11	0	1.648245	0.699	0.061	0	Saliva_Acir	CREB3L1
EDEM31	0	1.6473	0.703	0.056	0	Saliva_Acir	EDEM3

MT.CO17	0	1.630684	0.998	0.984	0	Saliva_Acir	MT.CO1
NUCB23	0	1.619431	0.855	0.258	0	Saliva_Acir	NUCB2
ANK31	0	1.591226	0.657	0.059	0	Saliva_Acir	ANK3
MTRNR2L1	0	1.589496	0.51	0.118	0	Saliva_Acir	MTRNR2L1
MT.CO36	0	1.584234	0.999	0.977	0	Saliva_Acir	MT.CO3
TCN12	0	1.578612	0.9	0.141	0	Saliva_Acir	TCN1
CYB5611	0	1.572814	0.683	0.075	0	Saliva_Acir	CYB561
ALCAM	0	1.56981	0.7	0.084	0	Saliva_Acir	ALCAM
APP2	0	1.562892	0.818	0.232	0	Saliva_Acir	APP
FAM129A5	0	1.557982	0.704	0.091	0	Saliva_Acir	FAM129A
KCNN41	0	1.557318	0.698	0.068	0	Saliva_Acir	KCNN4
TMED32	0	1.549297	0.835	0.267	0	Saliva_Acir	TMED3
NANS2	0	1.495978	0.765	0.219	0	Saliva_Acir	NANS
ASPH3	0	1.495843	0.74	0.179	0	Saliva_Acir	ASPH
PLCB42	0	1.495678	0.666	0.043	0	Saliva_Acir	PLCB4
KRT181	0	1.486797	0.911	0.29	0	Saliva_Acir	KRT18
TCEA31	0	1.461474	0.683	0.088	0	Saliva_Acir	TCEA3
XIST6	0	1.447967	0.492	0.115	0	Saliva_Acir	XIST
RRBP13	0	1.438762	0.835	0.301	0	Saliva_Acir	RRBP1
GALNT5	0	1.434523	0.624	0.036	0	Saliva_Acir	GALNT5
SPINT22	0	1.430993	0.861	0.302	0	Saliva_Acir	SPINT2
ALDH1A11	0	1.422578	0.605	0.065	0	Saliva_Acir	ALDH1A1
RABAC16	0	1.418165	0.878	0.395	0	Saliva_Acir	RABAC1
ZKSCAN13	0	1.395821	0.671	0.105	0	Saliva_Acir	ZKSCAN1
LMAN14	0	1.395447	0.761	0.182	0	Saliva_Acir	LMAN1
AGR3	0	1.390839	0.558	0.04	0	Saliva_Acir	AGR3
EPCAM2	0	1.388824	0.738	0.118	0	Saliva_Acir	EPCAM
CP1	0	1.376397	0.469	0.017	0	Saliva_Acir	CP
PART11	0	1.372111	0.636	0.06	0	Saliva_Acir	PART1
GOLGB14	0	1.36673	0.747	0.188	0	Saliva_Acir	GOLGB1
FUT2	0	1.351484	0.615	0.058	0	Saliva_Acir	FUT2
GALE	0	1.346288	0.703	0.137	0	Saliva_Acir	GALE
ANG2	0	1.340288	0.651	0.089	0	Saliva_Acir	ANG
CD1514	0	1.320488	0.776	0.209	0	Saliva_Acir	CD151
KRT81	0	1.319606	0.805	0.187	0	Saliva_Acir	KRT8
FAM114A1	0	1.312519	0.669	0.111	0	Saliva_Acir	FAM114A1
PTMS3	0	1.305548	0.788	0.233	0	Saliva_Acir	PTMS
SEC11C3	0	1.303572	0.837	0.229	0	Saliva_Acir	SEC11C
SORBS23	0	1.298192	0.668	0.101	0	Saliva_Acir	SORBS2
FOXP11	0	1.295256	0.745	0.208	0	Saliva_Acir	FOXP1
ACSL31	0	1.27875	0.688	0.148	0	Saliva_Acir	ACSL3
GALNT3	0	1.278412	0.658	0.11	0	Saliva_Acir	GALNT3
CREB3L41	0	1.273731	0.596	0.046	0	Saliva_Acir	CREB3L4
TMEM63A	0	1.270719	0.625	0.068	0	Saliva_Acir	TMEM63A
ERN2	0	1.264609	0.531	0.006	0	Saliva_Acir	ERN2
PDIA32	0	1.260408	0.813	0.312	0	Saliva_Acir	PDIA3
TPD52L13	0	1.258272	0.66	0.125	0	Saliva_Acir	TPD52L1

CD635	0	1.256056	0.955	0.618	0	Saliva_Acir	CD63
MTRNR2L8	0	1.253544	0.764	0.276	0	Saliva_Acir	MTRNR2L8
SEC16A	0	1.252638	0.584	0.075	0	Saliva_Acir	SEC16A
MTRNR2L1	0	1.243571	0.51	0.089	0	Saliva_Acir	MTRNR2L10
NUDT81	0	1.243456	0.522	0.047	0	Saliva_Acir	NUDT8
MAGED23	0	1.240446	0.696	0.144	0	Saliva_Acir	MAGED2
ATP2C2	0	1.239185	0.534	0.007	0	Saliva_Acir	ATP2C2
FGGY1	0	1.238588	0.594	0.074	0	Saliva_Acir	FGGY
CGREF11	0	1.226457	0.533	0.038	0	Saliva_Acir	CGREF1
ATP2A31	0	1.214009	0.549	0.044	0	Saliva_Acir	ATP2A3
CALR2	0	1.205142	0.865	0.414	0	Saliva_Acir	CALR
ITLN1	0	1.201214	0.205	0.004	0	Saliva_Acir	ITLN1
SLC44A4	0	1.198082	0.589	0.053	0	Saliva_Acir	SLC44A4
TECR2	0	1.194969	0.805	0.32	0	Saliva_Acir	TECR
RPS176	0	1.193333	0.816	0.21	0	Saliva_Acir	RPS17
BACE22	0	1.190672	0.657	0.152	0	Saliva_Acir	BACE2
SMIM12	0	1.180709	0.587	0.059	0	Saliva_Acir	SMIM1
GOLGA43	0	1.180501	0.722	0.225	0	Saliva_Acir	GOLGA4
ANKRD36C	0	1.17484	0.473	0.131	0	Saliva_Acir	ANKRD36C
SLC39A81	0	1.16408	0.617	0.083	0	Saliva_Acir	SLC39A8
SLC9A3R21	0	1.16396	0.671	0.156	0	Saliva_Acir	SLC9A3R2
SEC61A12	0	1.158598	0.665	0.141	0	Saliva_Acir	SEC61A1
NFIB4	0	1.157897	0.731	0.18	0	Saliva_Acir	NFIB
TMEM141	0	1.147722	0.662	0.154	0	Saliva_Acir	TMEM141
SMIM141	0	1.14737	0.726	0.24	0	Saliva_Acir	SMIM14
GPT21	0	1.146437	0.565	0.074	0	Saliva_Acir	GPT2
PRR42	0	1.139655	0.587	0.101	0	Saliva_Acir	PRR4
TSTA3	0	1.139319	0.67	0.166	0	Saliva_Acir	TSTA3
CKAP42	0	1.136378	0.658	0.155	0	Saliva_Acir	CKAP4
KDELR33	0	1.133537	0.582	0.075	0	Saliva_Acir	KDELR3
TF	0	1.130343	0.45	0.008	0	Saliva_Acir	TF
ATP1A12	0	1.112044	0.753	0.245	0	Saliva_Acir	ATP1A1
SEC31A3	0	1.109838	0.7	0.206	0	Saliva_Acir	SEC31A
KDELR22	0	1.092856	0.79	0.31	0	Saliva_Acir	KDELR2
SEL1L32	0	1.088131	0.548	0.036	0	Saliva_Acir	SEL1L3
MAGED11	0	1.086432	0.557	0.076	0	Saliva_Acir	MAGED1
SEC623	0	1.072138	0.835	0.393	0	Saliva_Acir	SEC62
MTURN1	0	1.066313	0.557	0.07	0	Saliva_Acir	MTURN
MUC16	0	1.063276	0.469	0.011	0	Saliva_Acir	MUC16
CLSTN12	0	1.06325	0.586	0.113	0	Saliva_Acir	CLSTN1
MANSC11	0	1.062168	0.549	0.071	0	Saliva_Acir	MANSC1
AKAP93	0	1.05761	0.735	0.261	0	Saliva_Acir	AKAP9
ANXA53	0	1.053334	0.798	0.308	0	Saliva_Acir	ANXA5
ANXA31	0	1.051226	0.636	0.145	0	Saliva_Acir	ANXA3
TMED103	0	1.041851	0.794	0.311	0	Saliva_Acir	TMED10
HSP90B11	0	1.040367	0.871	0.386	0	Saliva_Acir	HSP90B1
PTPRF2	0	1.037439	0.55	0.097	0	Saliva_Acir	PTPRF

CALU2	0	1.035309	0.646	0.166	0	Saliva_Acir	CALU
PHPT14	0	1.026379	0.781	0.315	0	Saliva_Acir	PHPT1
MT.ATP85	0	1.016802	0.621	0.125	0	Saliva_Acir	MT.ATP8
PAPSS2	0	1.016046	0.516	0.072	0	Saliva_Acir	PAPSS2
FAM177B	0	1.012018	0.46	0.007	0	Saliva_Acir	FAM177B
ST6GALNA	0	1.010913	0.485	0.033	0	Saliva_Acir	ST6GALNAC1
MT.ND66	0	0.99982	0.454	0.104	0	Saliva_Acir	MT.ND6
PHGDH2	0	0.99878	0.564	0.105	0	Saliva_Acir	PHGDH
FKBP112	0	0.994574	0.661	0.143	0	Saliva_Acir	FKBP11
PROM12	0	0.992279	0.518	0.073	0	Saliva_Acir	PROM1
STRA13	0	0.987521	0.66	0.185	0	Saliva_Acir	STRA13
ERGIC13	0	0.98336	0.591	0.124	0	Saliva_Acir	ERGIC1
CMTM82	0	0.983033	0.507	0.06	0	Saliva_Acir	CMTM8
ARFIP21	0	0.975406	0.555	0.097	0	Saliva_Acir	ARFIP2
CD164	0	0.975047	0.8	0.362	0	Saliva_Acir	CD164
OSBPL1A3	0	0.97401	0.602	0.151	0	Saliva_Acir	OSBPL1A
PRDX42	0	0.973003	0.755	0.246	0	Saliva_Acir	PRDX4
HM132	0	0.972011	0.673	0.194	0	Saliva_Acir	HM13
KDELR13	0	0.969314	0.712	0.241	0	Saliva_Acir	KDELR1
IBTK1	0	0.966577	0.519	0.084	0	Saliva_Acir	IBTK
COPA4	0	0.96298	0.61	0.136	0	Saliva_Acir	COPA
KIAA1147	0	0.962293	0.475	0.034	0	Saliva_Acir	KIAA1147
MTA1	0	0.961714	0.509	0.073	0	Saliva_Acir	MTA1
AQP3	0	0.961015	0.738	0.181	0	Saliva_Acir	AQP3
KRT71	0	0.957321	0.81	0.261	0	Saliva_Acir	KRT7
DPP72	0	0.956629	0.753	0.249	0	Saliva_Acir	DPP7
LMCD11	0	0.956034	0.559	0.074	0	Saliva_Acir	LMCD1
METRN3	0	0.955363	0.544	0.084	0	Saliva_Acir	METRN
QSOX1	0	0.953112	0.724	0.203	0	Saliva_Acir	QSOX1
PYCR13	0	0.947393	0.511	0.072	0	Saliva_Acir	PYCR1
FUT6	0	0.946296	0.473	0.053	0	Saliva_Acir	FUT6
MLEC4	0	0.943347	0.638	0.179	0	Saliva_Acir	MLEC
PDE4D	0	0.939076	0.436	0.048	0	Saliva_Acir	PDE4D
SLITRK61	0	0.935686	0.43	0.051	0	Saliva_Acir	SLITRK6
SELM5	0	0.933178	0.831	0.337	0	Saliva_Acir	SELM
LFNG1	0	0.931986	0.456	0.019	0	Saliva_Acir	LFNG
PITPNA1	0	0.930684	0.538	0.093	0	Saliva_Acir	PITPNA
COPB23	0	0.930161	0.605	0.146	0	Saliva_Acir	COPB2
SLC2A101	0	0.927281	0.463	0.027	0	Saliva_Acir	SLC2A10
SCNN1A	0	0.926058	0.531	0.081	0	Saliva_Acir	SCNN1A
LGALS3BP3	0	0.925946	0.659	0.16	0	Saliva_Acir	LGALS3BP
ENTPD8	0	0.925134	0.409	0.004	0	Saliva_Acir	ENTPD8
TPT14	0	0.914017	0.978	0.958	0	Saliva_Acir	TPT1
DDX175	0	0.911703	0.779	0.345	0	Saliva_Acir	DDX17
DCXR1	0	0.909902	0.704	0.21	0	Saliva_Acir	DCXR
SLC39A72	0	0.909344	0.551	0.104	0	Saliva_Acir	SLC39A7
GMDS2	0	0.907652	0.611	0.161	0	Saliva_Acir	GMDS

SERINC2	0	0.901879	0.696	0.203	0	Saliva_Acir	SERINC2
MSMB	0	0.900208	0.376	0.031	0	Saliva_Acir	MSMB
CANX3	0	0.890498	0.742	0.303	0	Saliva_Acir	CANX
LRPAP15	0	0.890419	0.718	0.26	0	Saliva_Acir	LRPAP1
GTF2I5	0	0.887235	0.607	0.144	0	Saliva_Acir	GTF2I
TPD522	0	0.88326	0.573	0.118	0	Saliva_Acir	TPD52
TMCO3	0	0.883017	0.487	0.064	0	Saliva_Acir	TMCO3
DUSP61	0	0.879901	0.526	0.12	0	Saliva_Acir	DUSP6
PDIA62	0	0.878829	0.741	0.27	0	Saliva_Acir	PDIA6
PPP3CA1	0	0.878468	0.61	0.146	0	Saliva_Acir	PPP3CA
CHPF3	0	0.877618	0.48	0.075	0	Saliva_Acir	CHPF
ACLY	0	0.877253	0.503	0.078	0	Saliva_Acir	ACLY
SNHG181	0	0.8746	0.476	0.053	0	Saliva_Acir	SNHG18
NOSTRIN1	0	0.873615	0.46	0.051	0	Saliva_Acir	NOSTRIN
NAGLU2	0	0.872488	0.474	0.061	0	Saliva_Acir	NAGLU
GGCT	0	0.869561	0.538	0.12	0	Saliva_Acir	GGCT
ARL13	0	0.869066	0.627	0.198	0	Saliva_Acir	ARL1
PODXL2	0	0.866515	0.45	0.065	0	Saliva_Acir	PODXL2
FKBP22	0	0.864206	0.8	0.342	0	Saliva_Acir	FKBP2
CCL282	0	0.861052	0.619	0.101	0	Saliva_Acir	CCL28
PERP	0	0.859306	0.839	0.357	0	Saliva_Acir	PERP
CANT11	0	0.855554	0.508	0.1	0	Saliva_Acir	CANT1
FOXA1	0	0.852232	0.446	0.039	0	Saliva_Acir	FOXA1
PPP1R16A1	0	0.849551	0.494	0.096	0	Saliva_Acir	PPP1R16A
SYNGR2	0	0.84925	0.649	0.226	0	Saliva_Acir	SYNGR2
C11orf241	0	0.848444	0.447	0.052	0	Saliva_Acir	C11orf24
HID12	0	0.847858	0.427	0.039	0	Saliva_Acir	HID1
JTB2	0	0.847527	0.783	0.362	0	Saliva_Acir	JTB
SSR43	0	0.847411	0.928	0.54	0	Saliva_Acir	SSR4
KCNQ1OT1	0	0.847219	0.519	0.134	0	Saliva_Acir	KCNQ1OT1
ZNF7042	0	0.834816	0.456	0.061	0	Saliva_Acir	ZNF704
CRYM	0	0.832617	0.557	0.109	0	Saliva_Acir	CRYM
ERLEC12	0	0.826635	0.582	0.174	0	Saliva_Acir	ERLEC1
NDUFB74	0	0.820603	0.781	0.365	0	Saliva_Acir	NDUFB7
SGSM31	0	0.815838	0.523	0.103	0	Saliva_Acir	SGSM3
FARP11	0	0.814248	0.47	0.092	0	Saliva_Acir	FARP1
CHCHD104	0	0.813658	0.71	0.235	0	Saliva_Acir	CHCHD10
MXD42	0	0.812261	0.532	0.111	0	Saliva_Acir	MXD4
ITGB51	0	0.80888	0.468	0.07	0	Saliva_Acir	ITGB5
TRPM4	0	0.80861	0.431	0.053	0	Saliva_Acir	TRPM4
YIF1A2	0	0.807239	0.594	0.164	0	Saliva_Acir	YIF1A
DSG2	0	0.807029	0.44	0.05	0	Saliva_Acir	DSG2
SELENBP14	0	0.8057	0.482	0.067	0	Saliva_Acir	SELENBP1
SCARB24	0	0.802852	0.559	0.149	0	Saliva_Acir	SCARB2
CCPG14	0	0.79947	0.605	0.159	0	Saliva_Acir	CCPG1
AGFG21	0	0.786997	0.53	0.129	0	Saliva_Acir	AGFG2
IDH1	0	0.786643	0.494	0.117	0	Saliva_Acir	IDH1

WIPI12	0	0.786157	0.472	0.091	0	Saliva_Acir	WIPI1
DNAJC33	0	0.78548	0.608	0.2	0	Saliva_Acir	DNAJC3
TMEM181	0	0.781749	0.433	0.06	0	Saliva_Acir	TMEM181
HOOK21	0	0.781354	0.443	0.096	0	Saliva_Acir	HOOK2
LINC00657	0	0.778825	0.603	0.195	0	Saliva_Acir	LINC00657
TNFRSF191	0	0.778279	0.44	0.058	0	Saliva_Acir	TNFRSF19
SLC30A71	0	0.77275	0.471	0.074	0	Saliva_Acir	SLC30A7
TMEM178A	0	0.768054	0.345	0.006	0	Saliva_Acir	TMEM178A
RP11.532F	0	0.767715	0.47	0.071	0	Saliva_Acir	RP11.532F12.5
MYO5C1	0	0.764458	0.408	0.042	0	Saliva_Acir	MYO5C
LRRC262	0	0.764402	0.425	0.071	0	Saliva_Acir	LRRC26
COPB11	0	0.7619	0.532	0.131	0	Saliva_Acir	COPB1
RPN22	0	0.759837	0.666	0.237	0	Saliva_Acir	RPN2
RASD11	0	0.75692	0.395	0.071	0	Saliva_Acir	RASD1
PCYOX12	0	0.756434	0.533	0.123	0	Saliva_Acir	PCYOX1
PGM32	0	0.75578	0.452	0.084	0	Saliva_Acir	PGM3
USO11	0	0.755281	0.503	0.121	0	Saliva_Acir	USO1
OXR1	0	0.755275	0.574	0.189	0	Saliva_Acir	OXR1
CHPT12	0	0.753561	0.613	0.198	0	Saliva_Acir	CHPT1
CBR11	0	0.752698	0.501	0.111	0	Saliva_Acir	CBR1
MYO1C2	0	0.751197	0.547	0.137	0	Saliva_Acir	MYO1C
PDIA41	0	0.748492	0.572	0.164	0	Saliva_Acir	PDIA4
SLC35A3	0	0.748099	0.538	0.127	0	Saliva_Acir	SLC35A3
KIAA0319L	0	0.746934	0.432	0.061	0	Saliva_Acir	KIAA0319L
CXCL17	0	0.74604	0.622	0.17	0	Saliva_Acir	CXCL17
SPATS2L	0	0.744481	0.616	0.216	0	Saliva_Acir	SPATS2L
SYNE23	0	0.741502	0.554	0.129	0	Saliva_Acir	SYNE2
RANBP21	0	0.740853	0.492	0.109	0	Saliva_Acir	RANBP2
TSTD13	0	0.739906	0.699	0.263	0	Saliva_Acir	TSTD1
B3GALT5	0	0.739163	0.368	0.028	0	Saliva_Acir	B3GALT5
ETV1	0	0.738394	0.356	0.043	0	Saliva_Acir	ETV1
FNDC3B2	0	0.737878	0.542	0.142	0	Saliva_Acir	FNDC3B
SEC633	0	0.737217	0.57	0.163	0	Saliva_Acir	SEC63
CDH11	0	0.730649	0.445	0.067	0	Saliva_Acir	CDH1
HERPUD11	0	0.730221	0.761	0.324	0	Saliva_Acir	HERPUD1
SH3BP42	0	0.729194	0.451	0.073	0	Saliva_Acir	SH3BP4
CLPTM1L2	0	0.726289	0.475	0.084	0	Saliva_Acir	CLPTM1L
SRPRB1	0	0.725222	0.478	0.104	0	Saliva_Acir	SRPRB
KCNK62	0	0.724039	0.445	0.094	0	Saliva_Acir	KCNK6
DAP2	0	0.723541	0.529	0.136	0	Saliva_Acir	DAP
MIPEP	0	0.721902	0.355	0.027	0	Saliva_Acir	MIPEP
COPG12	0	0.721461	0.467	0.088	0	Saliva_Acir	COPG1
SLC25A371	0	0.718479	0.614	0.166	0	Saliva_Acir	SLC25A37
TOM1L1	0	0.713307	0.402	0.051	0	Saliva_Acir	TOM1L1
SLC31A11	0	0.712303	0.415	0.073	0	Saliva_Acir	SLC31A1
ECI13	0	0.709985	0.51	0.131	0	Saliva_Acir	ECI1
PDXDC1	0	0.707724	0.537	0.146	0	Saliva_Acir	PDXDC1

MCFD22	0	0.70651	0.576	0.185	0	Saliva_Acir	MCFD2
SEC24D1	0	0.704567	0.445	0.082	0	Saliva_Acir	SEC24D
ARFGAP33	0	0.703195	0.528	0.151	0	Saliva_Acir	ARFGAP3
GLRX1	0	0.702794	0.659	0.204	0	Saliva_Acir	GLRX
TMED91	0	0.702087	0.704	0.283	0	Saliva_Acir	TMED9
PARM12	0	0.702078	0.401	0.046	0	Saliva_Acir	PARM1
S100A12	0	0.698345	0.502	0.096	0	Saliva_Acir	S100A1
PRRC11	0	0.698317	0.421	0.069	0	Saliva_Acir	PRRC1
SLC38A101	0	0.697285	0.457	0.092	0	Saliva_Acir	SLC38A10
SURF42	0	0.697009	0.543	0.17	0	Saliva_Acir	SURF4
DYNC1LI21	0	0.6968	0.527	0.155	0	Saliva_Acir	DYNC1LI2
CHRM3	0	0.696771	0.366	0.032	0	Saliva_Acir	CHRM3
TBC1D8B	0	0.696204	0.364	0.028	0	Saliva_Acir	TBC1D8B
ARHGEF28	0	0.694894	0.38	0.048	0	Saliva_Acir	ARHGEF28
ALDH3A21	0	0.694878	0.481	0.119	0	Saliva_Acir	ALDH3A2
EHF1	0	0.69155	0.656	0.235	0	Saliva_Acir	EHF
PDE8B	0	0.691454	0.349	0.022	0	Saliva_Acir	PDE8B
DDR1	0	0.690758	0.464	0.107	0	Saliva_Acir	DDR1
TMEM125:	0	0.690199	0.374	0.042	0	Saliva_Acir	TMEM125
SPATS21	0	0.687257	0.457	0.099	0	Saliva_Acir	SPATS2
CLCN31	0	0.682146	0.483	0.122	0	Saliva_Acir	CLCN3
DEGS2	0	0.682039	0.357	0.021	0	Saliva_Acir	DEGS2
ISOC21	0	0.681795	0.496	0.125	0	Saliva_Acir	ISOC2
SH3RF1	0	0.679204	0.374	0.048	0	Saliva_Acir	SH3RF1
NKTR5	0	0.679189	0.563	0.168	0	Saliva_Acir	NKTR
PACS11	0	0.678509	0.419	0.068	0	Saliva_Acir	PACS1
TTC374	0	0.678223	0.432	0.078	0	Saliva_Acir	TTC37
SOX21	0	0.677201	0.37	0.039	0	Saliva_Acir	SOX2
COBL1	0	0.674676	0.363	0.044	0	Saliva_Acir	COBL
SHROOM3	0	0.673997	0.362	0.044	0	Saliva_Acir	SHROOM3
CTSD	0	0.67385	0.791	0.283	0	Saliva_Acir	CTSD
GJC31	0	0.67327	0.391	0.054	0	Saliva_Acir	GJC3
TMEM87A	0	0.668825	0.526	0.164	0	Saliva_Acir	TMEM87A
SSR12	0	0.668454	0.554	0.184	0	Saliva_Acir	SSR1
RAB27B	0	0.667752	0.411	0.064	0	Saliva_Acir	RAB27B
SLC1A51	0	0.666441	0.407	0.07	0	Saliva_Acir	SLC1A5
CLMN1	0	0.663969	0.399	0.059	0	Saliva_Acir	CLMN
YIPF32	0	0.662992	0.605	0.226	0	Saliva_Acir	YIPF3
TMEM214:	0	0.662978	0.398	0.066	0	Saliva_Acir	TMEM214
PDCD42	0	0.661528	0.581	0.176	0	Saliva_Acir	PDCD4
RAP1GAP	0	0.659133	0.343	0.028	0	Saliva_Acir	RAP1GAP
SLC35A1	0	0.657157	0.397	0.045	0	Saliva_Acir	SLC35A1
KRTCAP31	0	0.655857	0.423	0.074	0	Saliva_Acir	KRTCAP3
EPRS5	0	0.654544	0.51	0.125	0	Saliva_Acir	EPRS
TMC5	0	0.651702	0.505	0.107	0	Saliva_Acir	TMC5
TGOLN25	0	0.650301	0.535	0.164	0	Saliva_Acir	TGOLN2
TACC2	0	0.64973	0.412	0.068	0	Saliva_Acir	TACC2

EIF4EBP11	0	0.648597	0.505	0.144	0	Saliva_Acir	EIF4EBP1
ARMCX32	0	0.648207	0.511	0.138	0	Saliva_Acir	ARMCX3
FAM120A1	0	0.647115	0.516	0.14	0	Saliva_Acir	FAM120A
NUDT4	0	0.645088	0.518	0.145	0	Saliva_Acir	NUDT4
CBR31	0	0.644429	0.406	0.076	0	Saliva_Acir	CBR3
PDLIM42	0	0.634498	0.469	0.098	0	Saliva_Acir	PDLIM4
SUCO1	0	0.634018	0.408	0.086	0	Saliva_Acir	SUCO
KMT2C3	0	0.630964	0.511	0.146	0	Saliva_Acir	KMT2C
CD814	0	0.630906	0.834	0.313	0	Saliva_Acir	CD81
PMEPA13	0	0.630568	0.478	0.112	0	Saliva_Acir	PMEPA1
PDLIM33	0	0.629012	0.342	0.052	0	Saliva_Acir	PDLIM3
ADI14	0	0.628456	0.598	0.214	0	Saliva_Acir	ADI1
CHDH	0	0.624794	0.324	0.034	0	Saliva_Acir	CHDH
NFIA2	0	0.624518	0.577	0.165	0	Saliva_Acir	NFIA
MAL2	0	0.622193	0.501	0.129	0	Saliva_Acir	MAL2
BNIP31	0	0.621014	0.489	0.143	0	Saliva_Acir	BNIP3
AKT12	0	0.620824	0.413	0.082	0	Saliva_Acir	AKT1
LIPH	0	0.620537	0.304	0.023	0	Saliva_Acir	LIPH
RAB4A1	0	0.619912	0.568	0.201	0	Saliva_Acir	RAB4A
MACF12	0	0.619162	0.525	0.161	0	Saliva_Acir	MACF1
KAZALD11	0	0.618745	0.35	0.024	0	Saliva_Acir	KAZALD1
VPS351	0	0.617015	0.53	0.177	0	Saliva_Acir	VPS35
SVIL2	0	0.613618	0.441	0.113	0	Saliva_Acir	SVIL
GOLGA31	0	0.613335	0.411	0.082	0	Saliva_Acir	GOLGA3
AKR1A12	0	0.612325	0.598	0.196	0	Saliva_Acir	AKR1A1
C1QTNF9B	0	0.609442	0.33	0.025	0	Saliva_Acir	C1QTNF9B.AS1
RHOBTB33	0	0.608959	0.458	0.112	0	Saliva_Acir	RHOBTB3
DPY19L3	0	0.608412	0.328	0.031	0	Saliva_Acir	DPY19L3
CLTC	0	0.607529	0.529	0.178	0	Saliva_Acir	CLTC
LIMCH12	0	0.604209	0.455	0.121	0	Saliva_Acir	LIMCH1
TRIP115	0	0.603912	0.457	0.114	0	Saliva_Acir	TRIP11
PCBD12	0	0.602309	0.52	0.151	0	Saliva_Acir	PCBD1
ENTPD31	0	0.599511	0.334	0.028	0	Saliva_Acir	ENTPD3
INADL1	0	0.598923	0.386	0.08	0	Saliva_Acir	INADL
TSPAN121	0	0.598249	0.355	0.046	0	Saliva_Acir	TSPAN12
CASC42	0	0.596727	0.47	0.115	0	Saliva_Acir	CASC4
FAAH	0	0.596551	0.318	0.037	0	Saliva_Acir	FAAH
MSI22	0	0.594826	0.376	0.064	0	Saliva_Acir	MSI2
CAPN9	0	0.594247	0.278	0.004	0	Saliva_Acir	CAPN9
PURA4	0	0.593477	0.455	0.118	0	Saliva_Acir	PURA
SBF1	0	0.593039	0.351	0.047	0	Saliva_Acir	SBF1
SLITRK5	0	0.591488	0.295	0.016	0	Saliva_Acir	SLITRK5
LLGL2	0	0.591027	0.449	0.11	0	Saliva_Acir	LLGL2
FNDC3A3	0	0.589397	0.43	0.11	0	Saliva_Acir	FNDC3A
SYTL11	0	0.589243	0.49	0.104	0	Saliva_Acir	SYTL1
ADAM9	0	0.588577	0.544	0.168	0	Saliva_Acir	ADAM9
B3GNT3	0	0.588022	0.357	0.05	0	Saliva_Acir	B3GNT3

NR2F62	0	0.587791	0.408	0.077	0	Saliva_Acir	NR2F6
SORL11	0	0.587768	0.394	0.065	0	Saliva_Acir	SORL1
TMC41	0	0.587211	0.375	0.074	0	Saliva_Acir	TMC4
CMTM4	0	0.586415	0.3	0.027	0	Saliva_Acir	CMTM4
ERGIC22	0	0.585219	0.501	0.149	0	Saliva_Acir	ERGIC2
ST6GAL12	0	0.582124	0.358	0.032	0	Saliva_Acir	ST6GAL1
CTNND11	0	0.581975	0.419	0.093	0	Saliva_Acir	CTNND1
LRRC592	0	0.581319	0.471	0.138	0	Saliva_Acir	LRRC59
SDR16C5	0	0.580913	0.353	0.048	0	Saliva_Acir	SDR16C5
PPP1R9A1	0	0.580572	0.323	0.045	0	Saliva_Acir	PPP1R9A
CLDN102	0	0.578263	0.399	0.089	0	Saliva_Acir	CLDN10
PTPRN21	0	0.576305	0.345	0.039	0	Saliva_Acir	PTPRN2
FAM195A	0	0.574975	0.453	0.115	0	Saliva_Acir	FAM195A
STEAP2	0	0.573353	0.328	0.024	0	Saliva_Acir	STEAP2
GMPPB2	0	0.571935	0.349	0.059	0	Saliva_Acir	GMPPB
GLG12	0	0.571585	0.475	0.12	0	Saliva_Acir	GLG1
P2RX4	0	0.570311	0.349	0.06	0	Saliva_Acir	P2RX4
ZC3H14	0	0.570293	0.461	0.106	0	Saliva_Acir	ZC3H14
PKP42	0	0.569964	0.369	0.072	0	Saliva_Acir	PKP4
GXYLT21	0	0.569678	0.316	0.027	0	Saliva_Acir	GXYLT2
CMBL1	0	0.569091	0.333	0.031	0	Saliva_Acir	CMBL
CYP27A11	0	0.568619	0.358	0.043	0	Saliva_Acir	CYP27A1
SLC44A3	0	0.568241	0.333	0.04	0	Saliva_Acir	SLC44A3
NUPR13	0	0.568169	0.709	0.25	0	Saliva_Acir	NUPR1
NFIX2	0	0.568078	0.442	0.103	0	Saliva_Acir	NFIX
CREB31	0	0.567261	0.454	0.125	0	Saliva_Acir	CREB3
MAN1B1	0	0.564758	0.371	0.068	0	Saliva_Acir	MAN1B1
MRPL542	0	0.564323	0.572	0.202	0	Saliva_Acir	MRPL54
PNKD	0	0.563902	0.561	0.186	0	Saliva_Acir	PNKD
PRKAA2	0	0.563405	0.309	0.03	0	Saliva_Acir	PRKAA2
GNPNAT1	0	0.562953	0.372	0.059	0	Saliva_Acir	GNPNAT1
HIPK23	0	0.560262	0.381	0.073	0	Saliva_Acir	HIPK2
SND11	0	0.558518	0.412	0.096	0	Saliva_Acir	SND1
FUCA11	0	0.557784	0.441	0.133	0	Saliva_Acir	FUCA1
TSC2	0	0.557446	0.36	0.062	0	Saliva_Acir	TSC2
LRIG31	0	0.557261	0.329	0.044	0	Saliva_Acir	LRIG3
GNAS2	0	0.555794	0.946	0.575	0	Saliva_Acir	GNAS
F2R2	0	0.554922	0.358	0.046	0	Saliva_Acir	F2R
ATP13A2	0	0.55391	0.346	0.049	0	Saliva_Acir	ATP13A2
CTBP2	0	0.553836	0.408	0.097	0	Saliva_Acir	CTBP2
SLC17A51	0	0.553191	0.375	0.077	0	Saliva_Acir	SLC17A5
TMEM131	0	0.552562	0.356	0.057	0	Saliva_Acir	TMEM131
SMARCA11	0	0.55215	0.361	0.052	0	Saliva_Acir	SMARCA1
SCD	0	0.55118	0.362	0.074	0	Saliva_Acir	SCD
C1orf1222	0	0.54824	0.524	0.176	0	Saliva_Acir	C1orf122
YIPF23	0	0.547508	0.412	0.102	0	Saliva_Acir	YIPF2
MON2	0	0.547166	0.363	0.063	0	Saliva_Acir	MON2

PCCA	0	0.546006	0.298	0.037	0	Saliva_Acir PCCA
ENTPD6	0	0.5459	0.353	0.055	0	Saliva_Acir ENTPD6
RNF1871	0	0.544832	0.471	0.118	0	Saliva_Acir RNF187
ALDH18A1	0	0.544047	0.349	0.051	0	Saliva_Acir ALDH18A1
STIM21	0	0.543892	0.347	0.048	0	Saliva_Acir STIM2
BCAS1	0	0.54371	0.499	0.115	0	Saliva_Acir BCAS1
STK39	0	0.54324	0.418	0.109	0	Saliva_Acir STK39
ARCN11	0	0.541546	0.434	0.109	0	Saliva_Acir ARCN1
STEAP1	0	0.540967	0.303	0.028	0	Saliva_Acir STEAP1
NFIC4	0	0.540416	0.531	0.164	0	Saliva_Acir NFIC
DDAH1	0	0.539397	0.342	0.042	0	Saliva_Acir DDAH1
ALDH9A11	0	0.538933	0.482	0.142	0	Saliva_Acir ALDH9A1
FAM174B	0	0.538435	0.333	0.055	0	Saliva_Acir FAM174B
SRA11	0	0.537723	0.517	0.175	0	Saliva_Acir SRA1
ALDH22	0	0.536392	0.461	0.117	0	Saliva_Acir ALDH2
LONP21	0	0.535897	0.389	0.081	0	Saliva_Acir LONP2
PHYHD11	0	0.535312	0.341	0.054	0	Saliva_Acir PHYHD1
DOPEY2	0	0.534805	0.316	0.048	0	Saliva_Acir DOPEY2
MBTPS13	0	0.534726	0.382	0.08	0	Saliva_Acir MBTPS1
MTUS11	0	0.533999	0.499	0.15	0	Saliva_Acir MTUS1
TMED7	0	0.53386	0.443	0.13	0	Saliva_Acir TMED7
NUS1	0	0.533726	0.39	0.089	0	Saliva_Acir NUS1
WWC11	0	0.533271	0.361	0.083	0	Saliva_Acir WWC1
ICA1	0	0.531953	0.438	0.105	0	Saliva_Acir ICA1
F5	0	0.53151	0.267	0.013	0	Saliva_Acir F5
ARSD	0	0.529118	0.327	0.053	0	Saliva_Acir ARSD
TNFSF101	0	0.52859	0.716	0.262	0	Saliva_Acir TNFSF10
RAB251	0	0.526925	0.432	0.128	0	Saliva_Acir RAB25
NPIPB15	0	0.526722	0.29	0.046	0	Saliva_Acir NPIPB15
MYO1D	0	0.525476	0.339	0.055	0	Saliva_Acir MYO1D
YIPF62	0	0.523622	0.46	0.14	0	Saliva_Acir YIPF6
GK52	0	0.523088	0.346	0.068	0	Saliva_Acir GK5
EIF2AK11	0	0.520519	0.399	0.097	0	Saliva_Acir EIF2AK1
CADM12	0	0.52011	0.381	0.098	0	Saliva_Acir CADM1
ZNF587	0	0.519627	0.26	0.03	0	Saliva_Acir ZNF587
IARS1	0	0.519561	0.344	0.066	0	Saliva_Acir IARS
SPTLC22	0	0.519334	0.349	0.076	0	Saliva_Acir SPTLC2
FAM73A	0	0.517776	0.319	0.052	0	Saliva_Acir FAM73A
PJA25	0	0.517562	0.482	0.144	0	Saliva_Acir PJA2
PHKB3	0	0.513954	0.404	0.1	0	Saliva_Acir PHKB
SLC25A43	0	0.512017	0.426	0.108	0	Saliva_Acir SLC25A4
MACROD1	0	0.511597	0.345	0.065	0	Saliva_Acir MACROD1
KCNE3	0	0.509956	0.27	0.008	0	Saliva_Acir KCNE3
DTX4	0	0.508536	0.294	0.031	0	Saliva_Acir DTX4
RCN12	0	0.508315	0.467	0.141	0	Saliva_Acir RCN1
TRPS11	0	0.507777	0.393	0.081	0	Saliva_Acir TRPS1
ADAM151	0	0.507183	0.376	0.069	0	Saliva_Acir ADAM15

UFL11	0	0.506654	0.419	0.11	0	Saliva_Acir	UFL1
PGM1	0	0.505722	0.329	0.05	0	Saliva_Acir	PGM1
AACS1	0	0.505132	0.333	0.058	0	Saliva_Acir	AACS
SEZ6L21	0	0.503521	0.273	0.041	0	Saliva_Acir	SEZ6L2
GOLGA21	0	0.500654	0.459	0.139	0	Saliva_Acir	GOLGA2
RNF1521	0	0.500397	0.301	0.03	0	Saliva_Acir	RNF152
SLC9A3R1	0	0.500384	0.5	0.147	0	Saliva_Acir	SLC9A3R1
UAP12	0	0.500258	0.534	0.176	0	Saliva_Acir	UAP1
RNF1301	0	0.500045	0.466	0.134	0	Saliva_Acir	RNF130
SEC131	0	0.499853	0.453	0.128	0	Saliva_Acir	SEC13
CTDSPL3	0	0.499442	0.353	0.079	0	Saliva_Acir	CTDSPL
RNF144B	0	0.499322	0.457	0.08	0	Saliva_Acir	RNF144B
MFSD4	0	0.496289	0.321	0.054	0	Saliva_Acir	MFSD4
CASZ1	0	0.495546	0.319	0.058	0	Saliva_Acir	CASZ1
ABHD17C	0	0.495506	0.313	0.03	0	Saliva_Acir	ABHD17C
ENOSF1	0	0.494924	0.309	0.059	0	Saliva_Acir	ENOSF1
PBX12	0	0.494905	0.41	0.108	0	Saliva_Acir	PBX1
SLC22A23	0	0.494154	0.314	0.054	0	Saliva_Acir	SLC22A23
NAALADL2	0	0.493302	0.272	0.045	0	Saliva_Acir	NAALADL2
CRNDE1	0	0.492624	0.521	0.148	0	Saliva_Acir	CRNDE
RAB3D1	0	0.492053	0.334	0.051	0	Saliva_Acir	RAB3D
CCND12	0	0.491813	0.436	0.112	0	Saliva_Acir	CCND1
SNX51	0	0.49029	0.42	0.104	0	Saliva_Acir	SNX5
RALGAPA2	0	0.490184	0.317	0.05	0	Saliva_Acir	RALGAPA2
WNK2	0	0.488978	0.267	0.026	0	Saliva_Acir	WNK2
PUS7L2	0	0.487246	0.346	0.064	0	Saliva_Acir	PUS7L
FLNB1	0	0.486441	0.346	0.073	0	Saliva_Acir	FLNB
C1GALT1	0	0.48633	0.377	0.093	0	Saliva_Acir	C1GALT1
ITGB41	0	0.485952	0.371	0.082	0	Saliva_Acir	ITGB4
SEC23IP	0	0.485587	0.32	0.049	0	Saliva_Acir	SEC23IP
ZNF6522	0	0.484587	0.376	0.09	0	Saliva_Acir	ZNF652
RNASET21	0	0.484139	0.641	0.228	0	Saliva_Acir	RNASET2
DPM21	0	0.483752	0.414	0.107	0	Saliva_Acir	DPM2
GMPPA1	0	0.480923	0.314	0.046	0	Saliva_Acir	GMPPA
ALDH6A1	0	0.479281	0.307	0.054	0	Saliva_Acir	ALDH6A1
SLC25A16	0	0.479232	0.285	0.03	0	Saliva_Acir	SLC25A16
IMPAD11	0	0.475573	0.371	0.086	0	Saliva_Acir	IMPAD1
FAM210B3	0	0.47361	0.391	0.087	0	Saliva_Acir	FAM210B
ESRP1	0	0.471945	0.335	0.075	0	Saliva_Acir	ESRP1
PSME4	0	0.47115	0.365	0.092	0	Saliva_Acir	PSME4
ERBB21	0	0.470587	0.293	0.054	0	Saliva_Acir	ERBB2
DLG3	0	0.469188	0.273	0.031	0	Saliva_Acir	DLG3
ENC1	0	0.467679	0.284	0.032	0	Saliva_Acir	ENC1
OGFRL12	0	0.466969	0.412	0.108	0	Saliva_Acir	OGFRL1
POLR2J35	0	0.464986	0.379	0.091	0	Saliva_Acir	POLR2J3
SCIN	0	0.464705	0.26	0.04	0	Saliva_Acir	SCIN
LEO1	0	0.463765	0.297	0.05	0	Saliva_Acir	LEO1

PYGB	0	0.463152	0.326	0.068	0	Saliva_Acir	PYGB
NPTN	0	0.462838	0.434	0.122	0	Saliva_Acir	NPTN
TMEM87B	0	0.462571	0.328	0.066	0	Saliva_Acir	TMEM87B
TBC1D161	0	0.462245	0.288	0.036	0	Saliva_Acir	TBC1D16
ZNF91	0	0.458497	0.397	0.1	0	Saliva_Acir	ZNF91
TMEM245:	0	0.456557	0.341	0.064	0	Saliva_Acir	TMEM245
PMM21	0	0.45549	0.271	0.036	0	Saliva_Acir	PMM2
TBCD1	0	0.454912	0.309	0.057	0	Saliva_Acir	TBCD
DUS1L3	0	0.453286	0.38	0.091	0	Saliva_Acir	DUS1L
PPM1K2	0	0.452568	0.405	0.093	0	Saliva_Acir	PPM1K
ROCK23	0	0.452394	0.376	0.089	0	Saliva_Acir	ROCK2
SLC35C1	0	0.449549	0.318	0.058	0	Saliva_Acir	SLC35C1
IRX32	0	0.449239	0.331	0.067	0	Saliva_Acir	IRX3
MKNK21	0	0.446616	0.401	0.111	0	Saliva_Acir	MKNK2
SLC33A11	0	0.445749	0.282	0.05	0	Saliva_Acir	SLC33A1
SYVN11	0	0.442459	0.324	0.07	0	Saliva_Acir	SYVN1
CACNB41	0	0.442417	0.245	0.013	0	Saliva_Acir	CACNB4
GMPR1	0	0.442092	0.277	0.04	0	Saliva_Acir	GMPR
SLC27A1	0	0.440481	0.27	0.03	0	Saliva_Acir	SLC27A1
PTPN18	0	0.439863	0.391	0.085	0	Saliva_Acir	PTPN18
AARS1	0	0.439602	0.282	0.047	0	Saliva_Acir	AARS
CTA.292E1	0	0.439581	0.256	0.041	0	Saliva_Acir	CTA.292E10.6
ENAH2	0	0.439102	0.412	0.112	0	Saliva_Acir	ENAH
PRKCI	0	0.438692	0.352	0.087	0	Saliva_Acir	PRKCI
STT3A1	0	0.436432	0.307	0.061	0	Saliva_Acir	STT3A
CCDC149	0	0.434992	0.243	0.029	0	Saliva_Acir	CCDC149
CYP2J2	0	0.431808	0.241	0.025	0	Saliva_Acir	CYP2J2
TMEM261	0	0.430028	0.457	0.133	0	Saliva_Acir	TMEM261
TMEM30B	0	0.429234	0.315	0.058	0	Saliva_Acir	TMEM30B
GLCE	0	0.426936	0.282	0.047	0	Saliva_Acir	GLCE
LRP31	0	0.426552	0.255	0.028	0	Saliva_Acir	LRP3
BCKDHB	0	0.42539	0.295	0.055	0	Saliva_Acir	BCKDHB
DHTKD1	0	0.424528	0.264	0.04	0	Saliva_Acir	DHTKD1
SLC39A111	0	0.423884	0.287	0.061	0	Saliva_Acir	SLC39A11
SATB1	0	0.423429	0.341	0.066	0	Saliva_Acir	SATB1
CDC42EP1:	0	0.423029	0.372	0.089	0	Saliva_Acir	CDC42EP1
DTNB	0	0.422664	0.26	0.042	0	Saliva_Acir	DTNB
HYOU11	0	0.422413	0.321	0.075	0	Saliva_Acir	HYOU1
CTSZ2	0	0.421286	0.592	0.183	0	Saliva_Acir	CTSZ
CAPS1	0	0.420814	0.329	0.067	0	Saliva_Acir	CAPS
TXNDC111	0	0.420037	0.318	0.069	0	Saliva_Acir	TXNDC11
ME1	0	0.419454	0.277	0.026	0	Saliva_Acir	ME1
SLC25A291	0	0.41919	0.261	0.052	0	Saliva_Acir	SLC25A29
PIK3R12	0	0.417436	0.391	0.105	0	Saliva_Acir	PIK3R1
HNMT	0	0.417347	0.388	0.103	0	Saliva_Acir	HNMT
C16orf58	0	0.416848	0.257	0.038	0	Saliva_Acir	C16orf58
ENPP3	0	0.414841	0.193	0.004	0	Saliva_Acir	ENPP3

DGUOK.AS	0	0.414751	0.248	0.014	0	Saliva_Acir	DGUOK.AS1
GLB1L2	0	0.414038	0.212	0.008	0	Saliva_Acir	GLB1L2
LPGAT1	0	0.413494	0.344	0.085	0	Saliva_Acir	LPGAT1
CDK61	0	0.413341	0.339	0.079	0	Saliva_Acir	CDK6
SHANK2	0	0.412798	0.224	0.021	0	Saliva_Acir	SHANK2
SETD71	0	0.411611	0.293	0.056	0	Saliva_Acir	SETD7
ZBTB7C1	0	0.408367	0.221	0.013	0	Saliva_Acir	ZBTB7C
NIPAL2	0	0.408271	0.268	0.032	0	Saliva_Acir	NIPAL2
LENG8	0	0.407945	0.322	0.074	0	Saliva_Acir	LENG8
ARFGAP1	0	0.406813	0.261	0.045	0	Saliva_Acir	ARFGAP1
GPX81	0	0.406511	0.28	0.034	0	Saliva_Acir	GPX8
KIAA1549	0	0.404408	0.214	0.01	0	Saliva_Acir	KIAA1549
MCF2L1	0	0.402856	0.256	0.024	0	Saliva_Acir	MCF2L
SLC16A9	0	0.402852	0.21	0.006	0	Saliva_Acir	SLC16A9
GATM1	0	0.40284	0.309	0.073	0	Saliva_Acir	GATM
MCTP2	0	0.401716	0.23	0.019	0	Saliva_Acir	MCTP2
IMPACT	0	0.399406	0.302	0.068	0	Saliva_Acir	IMPACT
PKN11	0	0.399295	0.379	0.084	0	Saliva_Acir	PKN1
CTAGE5	0	0.398939	0.269	0.051	0	Saliva_Acir	CTAGE5
ATP8B1	0	0.397623	0.302	0.066	0	Saliva_Acir	ATP8B1
KCNMA11	0	0.396899	0.241	0.037	0	Saliva_Acir	KCNMA1
PPM1L	0	0.396808	0.252	0.034	0	Saliva_Acir	PPM1L
SREBF2	0	0.395803	0.273	0.053	0	Saliva_Acir	SREBF2
DHCR7	0	0.395289	0.246	0.031	0	Saliva_Acir	DHCR7
PKIB	0	0.395278	0.289	0.052	0	Saliva_Acir	PKIB
MMAB	0	0.394914	0.328	0.079	0	Saliva_Acir	MMAB
ALDH1L21	0	0.394743	0.224	0.027	0	Saliva_Acir	ALDH1L2
SLIT32	0	0.39473	0.334	0.076	0	Saliva_Acir	SLIT3
ARHGEF37	0	0.393831	0.269	0.046	0	Saliva_Acir	ARHGEF37
RAD502	0	0.393675	0.336	0.083	0	Saliva_Acir	RAD50
CITED4	0	0.393648	0.336	0.078	0	Saliva_Acir	CITED4
ASAP3	0	0.39256	0.223	0.022	0	Saliva_Acir	ASAP3
DEPTOR1	0	0.391308	0.269	0.053	0	Saliva_Acir	DEPTOR
AGPS	0	0.388428	0.308	0.058	0	Saliva_Acir	AGPS
LRIG1	0	0.388055	0.24	0.038	0	Saliva_Acir	LRIG1
ARHGAP32	0	0.386468	0.325	0.076	0	Saliva_Acir	ARHGAP32
FOLH11	0	0.385392	0.226	0.031	0	Saliva_Acir	FOLH1
STARD4	0	0.384368	0.211	0.019	0	Saliva_Acir	STARD4
VEGFB4	0	0.383093	0.367	0.094	0	Saliva_Acir	VEGFB
RORC	0	0.381024	0.217	0.024	0	Saliva_Acir	RORC
CASK	0	0.380524	0.272	0.049	0	Saliva_Acir	CASK
ARHGAP26	0	0.380506	0.275	0.051	0	Saliva_Acir	ARHGAP26
SMARCD2	0	0.378525	0.332	0.082	0	Saliva_Acir	SMARCD2
ZNF552	0	0.376986	0.202	0.027	0	Saliva_Acir	ZNF552
ACP2	0	0.37659	0.275	0.053	0	Saliva_Acir	ACP2
KIAA16711	0	0.375268	0.256	0.042	0	Saliva_Acir	KIAA1671
GPR180	0	0.374862	0.214	0.029	0	Saliva_Acir	GPR180

MTMR10	0	0.373941	0.247	0.038	0	Saliva_Acir MTMR10
NEIL1	0	0.370781	0.224	0.039	0	Saliva_Acir NEIL1
LDOC1	0	0.370578	0.248	0.038	0	Saliva_Acir LDOC1
GCAT	0	0.368862	0.234	0.044	0	Saliva_Acir GCAT
LRP51	0	0.368585	0.235	0.039	0	Saliva_Acir LRP5
TRPV4	0	0.368366	0.191	0.012	0	Saliva_Acir TRPV4
GPD1L	0	0.36668	0.23	0.025	0	Saliva_Acir GPD1L
NBEA1	0	0.366105	0.239	0.044	0	Saliva_Acir NBEA
TLE2	0	0.366025	0.235	0.043	0	Saliva_Acir TLE2
FBXL8	0	0.364074	0.23	0.023	0	Saliva_Acir FBXL8
C2CD2	0	0.362924	0.228	0.033	0	Saliva_Acir C2CD2
SHMT21	0	0.361727	0.31	0.062	0	Saliva_Acir SHMT2
KIF3B	0	0.361612	0.25	0.049	0	Saliva_Acir KIF3B
SOSTDC1	0	0.361162	0.233	0.028	0	Saliva_Acir SOSTDC1
GCNT1	0	0.360522	0.215	0.016	0	Saliva_Acir GCNT1
CHPF21	0	0.359203	0.246	0.034	0	Saliva_Acir CHPF2
LRP11	0	0.357876	0.248	0.046	0	Saliva_Acir LRP11
ATP9A	0	0.357139	0.233	0.036	0	Saliva_Acir ATP9A
MAN2A11	0	0.3571	0.256	0.05	0	Saliva_Acir MAN2A1
IQGAP21	0	0.356551	0.418	0.11	0	Saliva_Acir IQGAP2
FAM83F	0	0.35539	0.223	0.028	0	Saliva_Acir FAM83F
LONRF2	0	0.354876	0.173	0.008	0	Saliva_Acir LONRF2
YIPF1	0	0.353965	0.277	0.058	0	Saliva_Acir YIPF1
DDI2	0	0.352555	0.248	0.038	0	Saliva_Acir DDI2
TMEM8B	0	0.352113	0.226	0.033	0	Saliva_Acir TMEM8B
IL20RA	0	0.351256	0.194	0.024	0	Saliva_Acir IL20RA
ANKZF11	0	0.350667	0.263	0.049	0	Saliva_Acir ANKZF1
UGGT1	0	0.350449	0.242	0.045	0	Saliva_Acir UGGT1
RAVER2	0	0.350329	0.211	0.031	0	Saliva_Acir RAVER2
GPR1601	0	0.347312	0.274	0.054	0	Saliva_Acir GPR160
SGSM2	0	0.346613	0.222	0.036	0	Saliva_Acir SGSM2
CTC.444N2	0	0.344851	0.252	0.042	0	Saliva_Acir CTC.444N24.11
EIF2AK31	0	0.344629	0.26	0.04	0	Saliva_Acir EIF2AK3
CADPS23	0	0.344502	0.232	0.041	0	Saliva_Acir CADPS2
DST2	0	0.343636	0.537	0.177	0	Saliva_Acir DST
ACSS1	0	0.342202	0.268	0.047	0	Saliva_Acir ACSS1
TBC1D30	0	0.341893	0.2	0.023	0	Saliva_Acir TBC1D30
DNAJC12	0	0.341383	0.195	0.018	0	Saliva_Acir DNAJC12
TMEM150	0	0.341085	0.2	0.016	0	Saliva_Acir TMEM150C
COG3	0	0.340309	0.27	0.055	0	Saliva_Acir COG3
MGAT5	0	0.33962	0.218	0.029	0	Saliva_Acir MGAT5
MB1	0	0.338965	0.276	0.029	0	Saliva_Acir MB
VSTM2L	0	0.338715	0.155	0.007	0	Saliva_Acir VSTM2L
TMEM41A	0	0.33825	0.262	0.054	0	Saliva_Acir TMEM41A
CCDC160	0	0.337753	0.196	0.026	0	Saliva_Acir CCDC160
GNMT	0	0.336848	0.197	0.02	0	Saliva_Acir GNMT
B4GALT21	0	0.336199	0.232	0.038	0	Saliva_Acir B4GALT2

CIPC	0	0.335525	0.212	0.027	0	Saliva_Acir	CIPC
RPS6KA21	0	0.332945	0.239	0.036	0	Saliva_Acir	RPS6KA2
C9orf91	0	0.331396	0.2	0.013	0	Saliva_Acir	C9orf91
UTP14C	0	0.331157	0.227	0.04	0	Saliva_Acir	UTP14C
FAN1	0	0.326513	0.173	0.023	0	Saliva_Acir	FAN1
FIGN	0	0.323905	0.196	0.022	0	Saliva_Acir	FIGN
PIGM	0	0.322931	0.199	0.031	0	Saliva_Acir	PIGM
FMN1	0	0.321448	0.184	0.025	0	Saliva_Acir	FMN1
CCDC125	0	0.32081	0.185	0.024	0	Saliva_Acir	CCDC125
PLEKHB1	0	0.319501	0.252	0.049	0	Saliva_Acir	PLEKHB1
UBXN10.A!	0	0.317301	0.193	0.008	0	Saliva_Acir	UBXN10.AS1
GALNT101	0	0.317188	0.203	0.025	0	Saliva_Acir	GALNT10
IRX1	0	0.315911	0.187	0.015	0	Saliva_Acir	IRX1
CAPN8	0	0.314852	0.161	0.003	0	Saliva_Acir	CAPN8
IMPDH11	0	0.314538	0.258	0.046	0	Saliva_Acir	IMPDH1
TRIQK	0	0.311908	0.239	0.035	0	Saliva_Acir	TRIQK
RAB17	0	0.310267	0.209	0.033	0	Saliva_Acir	RAB17
SOAT1	0	0.309621	0.266	0.053	0	Saliva_Acir	SOAT1
PLXNA3	0	0.303644	0.206	0.028	0	Saliva_Acir	PLXNA3
MYCN	0	0.303543	0.175	0.007	0	Saliva_Acir	MYCN
ASB91	0	0.300358	0.205	0.015	0	Saliva_Acir	ASB9
RABEP2	0	0.29979	0.214	0.035	0	Saliva_Acir	RABEP2
MAGIX	0	0.298455	0.188	0.025	0	Saliva_Acir	MAGIX
RP5.894A1	0	0.29713	0.154	0.007	0	Saliva_Acir	RP5.894A10.2
OVOL2	0	0.294438	0.167	0.02	0	Saliva_Acir	OVOL2
ALG1	0	0.293623	0.212	0.03	0	Saliva_Acir	ALG1
ZNF605	0	0.291538	0.178	0.024	0	Saliva_Acir	ZNF605
ARHGEF16	0	0.28877	0.177	0.022	0	Saliva_Acir	ARHGEF16
DMRTA1	0	0.288178	0.183	0.017	0	Saliva_Acir	DMRTA1
DGKD1	0	0.288084	0.204	0.029	0	Saliva_Acir	DGKD
HOOK1	0	0.287506	0.335	0.08	0	Saliva_Acir	HOOK1
URB1	0	0.286914	0.179	0.023	0	Saliva_Acir	URB1
TNFRSF11E	0	0.286123	0.167	0.013	0	Saliva_Acir	TNFRSF11B
SLC1A41	0	0.285465	0.183	0.026	0	Saliva_Acir	SLC1A4
MPC2	0	0.285455	0.664	0.25	0	Saliva_Acir	MPC2
CUEDC11	0	0.28497	0.194	0.028	0	Saliva_Acir	CUEDC1
ARHGEF38	0	0.282155	0.12	0.009	0	Saliva_Acir	ARHGEF38
RNF128	0	0.281918	0.169	0.017	0	Saliva_Acir	RNF128
PPM1H	0	0.279947	0.158	0.012	0	Saliva_Acir	PPM1H
ACPP	0	0.276157	0.143	0.008	0	Saliva_Acir	ACPP
PDIA52	0	0.273184	0.176	0.026	0	Saliva_Acir	PDIA5
PGAP3	0	0.272022	0.164	0.022	0	Saliva_Acir	PGAP3
TMEM246	0	0.270983	0.169	0.013	0	Saliva_Acir	TMEM246
HSF4	0	0.270191	0.142	0.014	0	Saliva_Acir	HSF4
FAM168A	0	0.268071	0.178	0.022	0	Saliva_Acir	FAM168A
IRX51	0	0.267812	0.189	0.025	0	Saliva_Acir	IRX5
SLC7A8	0	0.265228	0.159	0.021	0	Saliva_Acir	SLC7A8

VIPR11	0	0.265156	0.171	0.017	0	Saliva_Acir	VIPR1
LDLRAD2	0	0.263274	0.133	0.007	0	Saliva_Acir	LDLRAD2
SYNE4	0	0.261454	0.161	0.02	0	Saliva_Acir	SYNE4
GPT	0	0.260718	0.167	0.022	0	Saliva_Acir	GPT
SNCA1	0	0.259582	0.248	0.042	0	Saliva_Acir	SNCA
INHBB1	0	0.257256	0.161	0.018	0	Saliva_Acir	INHBB
OSBPL10	0	0.257203	0.284	0.06	0	Saliva_Acir	OSBPL10
LIMD11	0	0.254226	0.177	0.024	0	Saliva_Acir	LIMD1
LDLRAP1	0	0.251641	0.288	0.057	0	Saliva_Acir	LDLRAP1
GYLTL1B	0	0.250688	0.159	0.021	0	Saliva_Acir	GYLTL1B
CA12	5.8E-308	0.347119	0.255	0.051	9.2E-304	Saliva_Acir	CA12
ADCK3	8E-308	0.277479	0.239	0.046	1.3E-303	Saliva_Acir	ADCK3
RIC3	1.8E-307	0.458372	0.338	0.085	2.9E-303	Saliva_Acir	RIC3
CIB11	2.1E-307	0.719341	0.758	0.342	3.3E-303	Saliva_Acir	CIB1
SLC37A3	9E-307	0.302863	0.211	0.037	1.4E-302	Saliva_Acir	SLC37A3
GTF3C1	1.2E-306	0.338623	0.24	0.046	2E-302	Saliva_Acir	GTF3C1
MIS18BP1:	1.6E-306	0.450897	0.369	0.097	2.5E-302	Saliva_Acir	MIS18BP1
TEX91	4.8E-306	0.332488	0.204	0.035	7.6E-302	Saliva_Acir	TEX9
BRI33	5.9E-306	0.753931	0.75	0.338	9.4E-302	Saliva_Acir	BRI3
COG6	7.9E-306	0.290151	0.229	0.043	1.3E-301	Saliva_Acir	COG6
IER3IP12	1.1E-305	0.484302	0.447	0.137	1.7E-301	Saliva_Acir	IER3IP1
FRMD4B2	1.6E-305	0.525299	0.414	0.121	2.5E-301	Saliva_Acir	FRMD4B
S100A132	2.2E-305	0.532805	0.661	0.249	3.5E-301	Saliva_Acir	S100A13
RAB27A1	2.4E-305	0.298615	0.281	0.061	3.8E-301	Saliva_Acir	RAB27A
MINA	2.6E-305	0.300598	0.245	0.049	4.1E-301	Saliva_Acir	MINA
GBAS1	3.1E-305	0.435496	0.413	0.119	5E-301	Saliva_Acir	GBAS
MRPS262	2E-304	0.420109	0.466	0.143	3.2E-300	Saliva_Acir	MRPS26
ETNK1	2.7E-304	0.514543	0.447	0.135	4.4E-300	Saliva_Acir	ETNK1
GARS1	3.4E-304	0.416899	0.36	0.095	5.4E-300	Saliva_Acir	GARS
MGLL4	5.8E-304	0.546638	0.523	0.17	9.3E-300	Saliva_Acir	MGLL
YIPF52	6.9E-304	0.439509	0.397	0.112	1.1E-299	Saliva_Acir	YIPF5
SERF21	2.2E-302	0.808577	0.924	0.821	3.5E-298	Saliva_Acir	SERF2
IARS22	5.9E-302	0.387539	0.338	0.086	9.4E-298	Saliva_Acir	IARS2
COPZ11	7.9E-300	0.574733	0.572	0.21	1.3E-295	Saliva_Acir	COPZ1
ZNF280D2	8.1E-300	0.424353	0.322	0.079	1.3E-295	Saliva_Acir	ZNF280D
CLDN42	8.6E-300	0.467557	0.336	0.085	1.4E-295	Saliva_Acir	CLDN4
PTS1	1.6E-299	0.507055	0.433	0.131	2.5E-295	Saliva_Acir	PTS
TSPO2	2.3E-299	0.700589	0.846	0.458	3.6E-295	Saliva_Acir	TSPO
TOMM205	2.3E-298	0.753438	0.716	0.317	3.6E-294	Saliva_Acir	TOMM20
CHD95	9.9E-298	0.533147	0.492	0.16	1.6E-293	Saliva_Acir	CHD9
NIPAL3	3.4E-297	0.391607	0.283	0.064	5.4E-293	Saliva_Acir	NIPAL3
SUMF21	1.1E-294	0.459399	0.387	0.109	1.8E-290	Saliva_Acir	SUMF2
CDK5RAP3	1.4E-294	0.453051	0.415	0.123	2.2E-290	Saliva_Acir	CDK5RAP3
WDR6	4.1E-294	0.32553	0.287	0.065	6.5E-290	Saliva_Acir	WDR6
RBM47	5E-294	0.571805	0.575	0.21	7.9E-290	Saliva_Acir	RBM47
RPS6KA31	8.6E-294	0.403144	0.349	0.09	1.4E-289	Saliva_Acir	RPS6KA3
EIF4G1	2.1E-293	0.522545	0.485	0.162	3.4E-289	Saliva_Acir	EIF4G1

MRPL271	2.3E-293	0.417318	0.445	0.137	3.7E-289	Saliva_Acir	MRPL27
NET11	1.3E-292	0.401891	0.386	0.107	2.1E-288	Saliva_Acir	NET1
ATP1B1	4E-292	0.443292	0.753	0.316	6.5E-288	Saliva_Acir	ATP1B1
ACOT11	4.4E-292	0.267608	0.172	0.026	7.1E-288	Saliva_Acir	ACOT11
CHID12	6E-292	0.497155	0.496	0.164	9.5E-288	Saliva_Acir	CHID1
FBXO9	1.4E-291	0.35808	0.345	0.091	2.2E-287	Saliva_Acir	FBXO9
SLC30A9	1E-290	0.359826	0.28	0.064	1.7E-286	Saliva_Acir	SLC30A9
LRBA1	2.3E-290	0.34252	0.273	0.061	3.7E-286	Saliva_Acir	LRBA
METTL7A1	2.9E-290	0.363141	0.336	0.086	4.7E-286	Saliva_Acir	METTL7A
SLC35E11	1.6E-289	0.396323	0.294	0.07	2.5E-285	Saliva_Acir	SLC35E1
SLC30A51	3.9E-289	0.343866	0.286	0.066	6.2E-285	Saliva_Acir	SLC30A5
CREB3L22	8.1E-289	0.417846	0.353	0.095	1.3E-284	Saliva_Acir	CREB3L2
KIF5B1	1.8E-288	0.649247	0.673	0.282	2.9E-284	Saliva_Acir	KIF5B
SCYL11	5.4E-288	0.393848	0.349	0.093	8.7E-284	Saliva_Acir	SCYL1
COA3	1.5E-287	0.459118	0.503	0.171	2.4E-283	Saliva_Acir	COA3
OS92	2.6E-287	0.55132	0.587	0.218	4.1E-283	Saliva_Acir	OS9
TMF1	2.6E-287	0.526216	0.537	0.188	4.1E-283	Saliva_Acir	TMF1
RPN11	6.6E-287	0.523267	0.504	0.172	1.1E-282	Saliva_Acir	RPN1
MOGS1	3.5E-286	0.340639	0.265	0.058	5.6E-282	Saliva_Acir	MOGS
NFE2L11	7.1E-286	0.430873	0.334	0.086	1.1E-281	Saliva_Acir	NFE2L1
GORASP21	1.4E-285	0.444088	0.44	0.138	2.3E-281	Saliva_Acir	GORASP2
GSPT1	4.9E-285	0.521214	0.496	0.168	7.8E-281	Saliva_Acir	GSPT1
EMC10	2.3E-284	0.43316	0.459	0.147	3.7E-280	Saliva_Acir	EMC10
ZDHHC16	3.4E-284	0.302314	0.224	0.044	5.5E-280	Saliva_Acir	ZDHHC16
KIF21A3	4.9E-284	0.373565	0.308	0.076	7.9E-280	Saliva_Acir	KIF21A
SLC35C2	7.1E-284	0.310884	0.31	0.077	1.1E-279	Saliva_Acir	SLC35C2
TM7SF21	7.3E-284	0.441696	0.357	0.098	1.2E-279	Saliva_Acir	TM7SF2
SMCO41	8.6E-283	0.423378	0.387	0.113	1.4E-278	Saliva_Acir	SMCO4
MRPS342	1.1E-282	0.475328	0.54	0.19	1.8E-278	Saliva_Acir	MRPS34
CSRP11	1.4E-282	0.361209	0.467	0.149	2.2E-278	Saliva_Acir	CSRP1
LURAP1L	1.5E-282	0.302498	0.243	0.051	2.4E-278	Saliva_Acir	LURAP1L
NDUFB85	2.2E-282	0.51728	0.505	0.172	3.6E-278	Saliva_Acir	NDUFB8
ITGA63	7.7E-282	0.40531	0.428	0.13	1.2E-277	Saliva_Acir	ITGA6
PSD4	4.9E-281	0.279433	0.191	0.033	7.8E-277	Saliva_Acir	PSD4
IVD	8.2E-281	0.312438	0.231	0.047	1.3E-276	Saliva_Acir	IVD
UBE2J11	9E-281	0.363871	0.549	0.196	1.4E-276	Saliva_Acir	UBE2J1
LARS2	2.8E-280	0.421733	0.379	0.109	4.4E-276	Saliva_Acir	LARS
LINC00969	3.5E-280	0.468309	0.377	0.106	5.6E-276	Saliva_Acir	LINC00969
SLC25A362	8.6E-280	0.41119	0.4	0.118	1.4E-275	Saliva_Acir	SLC25A36
SLC7A11	4.4E-279	0.313518	0.23	0.046	7.1E-275	Saliva_Acir	SLC7A1
KIAA0368	1.1E-278	0.350335	0.261	0.058	1.8E-274	Saliva_Acir	KIAA0368
MROH1	2.1E-278	0.297218	0.198	0.036	3.4E-274	Saliva_Acir	MROH1
IKZF2	6.9E-278	0.309214	0.212	0.04	1.1E-273	Saliva_Acir	IKZF2
SSR32	7.4E-278	0.609777	0.678	0.293	1.2E-273	Saliva_Acir	SSR3
SLC39A62	2.9E-277	0.401434	0.343	0.094	4.7E-273	Saliva_Acir	SLC39A6
HSPA51	3.3E-277	0.689697	0.768	0.364	5.2E-273	Saliva_Acir	HSPA5
ATP6AP11	3.3E-277	0.465803	0.468	0.154	5.2E-273	Saliva_Acir	ATP6AP1

PPP1R14B5	1.4E-275	0.440369	0.502	0.171	2.2E-271	Saliva_Acir	PPP1R14B
SLFN13	2.3E-275	0.250922	0.155	0.023	3.6E-271	Saliva_Acir	SLFN13
ENTPD5	3.1E-275	0.262837	0.189	0.033	5E-271	Saliva_Acir	ENTPD5
GPAA12	6.2E-275	0.438913	0.435	0.137	9.9E-271	Saliva_Acir	GPAA1
ITPR23	1.6E-274	0.374962	0.303	0.076	2.5E-270	Saliva_Acir	ITPR2
TC2N2	1.6E-274	0.481511	0.456	0.148	2.5E-270	Saliva_Acir	TC2N
ATP5G13	2.4E-274	0.549498	0.638	0.253	3.8E-270	Saliva_Acir	ATP5G1
LINC00342	6.3E-274	0.511014	0.235	0.05	1E-269	Saliva_Acir	LINC00342
NEK6	2.3E-273	0.273242	0.187	0.033	3.6E-269	Saliva_Acir	NEK6
ATP2A2	3.1E-273	0.517223	0.489	0.169	5E-269	Saliva_Acir	ATP2A2
MARVELD3	3.5E-273	0.339465	0.216	0.042	5.5E-269	Saliva_Acir	MARVELD3
CRIP22	8.1E-273	0.379403	0.56	0.197	1.3E-268	Saliva_Acir	CRIP2
DCTN1	1.2E-272	0.408795	0.312	0.081	1.9E-268	Saliva_Acir	DCTN1
GNA112	1.3E-272	0.342436	0.274	0.064	2.1E-268	Saliva_Acir	GNA11
CLDN32	3.5E-272	0.311447	0.356	0.094	5.6E-268	Saliva_Acir	CLDN3
NOL3	4.4E-272	0.359538	0.298	0.074	7.1E-268	Saliva_Acir	NOL3
ERCC12	5.3E-272	0.437789	0.454	0.149	8.5E-268	Saliva_Acir	ERCC1
HYI2	8.4E-272	0.330869	0.317	0.082	1.3E-267	Saliva_Acir	HYI
EPM2AIP1	9.8E-272	0.32771	0.286	0.068	1.6E-267	Saliva_Acir	EPM2AIP1
GATAD12	3.3E-271	0.331262	0.293	0.072	5.2E-267	Saliva_Acir	GATAD1
NDUFV3	6.5E-271	0.362814	0.498	0.17	1E-266	Saliva_Acir	NDUFV3
NT5DC1	1.1E-270	0.315877	0.301	0.075	1.8E-266	Saliva_Acir	NT5DC1
GNE	2.1E-270	0.324979	0.285	0.069	3.4E-266	Saliva_Acir	GNE
MFSD6	1.6E-269	0.390076	0.295	0.074	2.6E-265	Saliva_Acir	MFSD6
FAM217B1	1.8E-269	0.262644	0.227	0.046	2.9E-265	Saliva_Acir	FAM217B
ARL6IP52	4E-269	0.514424	0.724	0.301	6.4E-265	Saliva_Acir	ARL6IP5
MRPL411	4.1E-269	0.647386	0.683	0.302	6.5E-265	Saliva_Acir	MRPL41
PACSIN3	5.9E-269	0.321511	0.23	0.048	9.5E-265	Saliva_Acir	PACSIN3
IRAK11	1.3E-268	0.28982	0.232	0.048	2.1E-264	Saliva_Acir	IRAK1
SMARCC1	2.7E-268	0.429133	0.376	0.109	4.3E-264	Saliva_Acir	SMARCC1
NDUFS81	2.3E-267	0.520249	0.64	0.257	3.7E-263	Saliva_Acir	NDUFS8
NCALD1	2.8E-267	0.292721	0.276	0.065	4.5E-263	Saliva_Acir	NCALD
NDUFB105	4.9E-267	0.689259	0.77	0.377	7.8E-263	Saliva_Acir	NDUFB10
MTMR6	8.3E-267	0.288115	0.261	0.06	1.3E-262	Saliva_Acir	MTMR6
GSTZ1	8.7E-267	0.350809	0.316	0.083	1.4E-262	Saliva_Acir	GSTZ1
CNKSR1	4E-266	0.279831	0.172	0.029	6.5E-262	Saliva_Acir	CNKSR1
MPZL12	1.1E-265	0.468261	0.423	0.135	1.8E-261	Saliva_Acir	MPZL1
SHPRH3	1.1E-265	0.354634	0.277	0.066	1.8E-261	Saliva_Acir	SHPRH
ZNF1061	3.6E-265	0.400405	0.421	0.133	5.7E-261	Saliva_Acir	ZNF106
OAT2	8.6E-265	0.469655	0.485	0.165	1.4E-260	Saliva_Acir	OAT
OSBP1	1.1E-264	0.354446	0.281	0.068	1.8E-260	Saliva_Acir	OSBP
FAM35A1	2.1E-264	0.2924	0.237	0.051	3.4E-260	Saliva_Acir	FAM35A
SCAMP4	4.1E-264	0.310725	0.254	0.057	6.5E-260	Saliva_Acir	SCAMP4
TRAK11	5.5E-264	0.339966	0.263	0.062	8.8E-260	Saliva_Acir	TRAK1
CD2AP1	6.6E-264	0.461251	0.416	0.132	1.1E-259	Saliva_Acir	CD2AP
TAPBP3	2.6E-263	0.335937	0.479	0.157	4.2E-259	Saliva_Acir	TAPBP
VCP1	3.3E-263	0.555395	0.595	0.238	5.3E-259	Saliva_Acir	VCP

ITGA2	3.4E-263	0.320587	0.323	0.084	5.5E-259	Saliva_Acir	ITGA2
TMEM208	4.1E-263	0.446082	0.523	0.19	6.5E-259	Saliva_Acir	TMEM208
SEC61B2	5.3E-263	0.776077	0.805	0.452	8.4E-259	Saliva_Acir	SEC61B
RAB3GAP2	7.4E-263	0.323432	0.246	0.055	1.2E-258	Saliva_Acir	RAB3GAP2
NDUFS11	1.4E-262	0.336743	0.317	0.084	2.2E-258	Saliva_Acir	NDUFS1
FAM84B2	1.7E-262	0.322115	0.301	0.077	2.7E-258	Saliva_Acir	FAM84B
ALDH7A1	2.4E-262	0.329067	0.26	0.061	3.8E-258	Saliva_Acir	ALDH7A1
INTS1	7.9E-262	0.307622	0.238	0.052	1.3E-257	Saliva_Acir	INTS1
QSER1	8.5E-262	0.35309	0.257	0.06	1.4E-257	Saliva_Acir	QSER1
UBE2Q2	1.4E-261	0.456189	0.373	0.112	2.2E-257	Saliva_Acir	UBE2Q2
LTBR2	2.6E-261	0.365726	0.318	0.084	4.2E-257	Saliva_Acir	LTBR
FASN	3.8E-261	0.29491	0.223	0.047	6.1E-257	Saliva_Acir	FASN
VIMP3	9.7E-261	0.580906	0.671	0.284	1.5E-256	Saliva_Acir	VIMP
ATRX3	3.3E-260	0.484963	0.543	0.2	5.2E-256	Saliva_Acir	ATRX
EEF2K1	1.6E-258	0.276926	0.211	0.042	2.6E-254	Saliva_Acir	EEF2K
ARHGAP12	1.8E-258	0.318317	0.302	0.078	2.8E-254	Saliva_Acir	ARHGAP12
PREPL	2.8E-258	0.285332	0.247	0.055	4.5E-254	Saliva_Acir	PREPL
SLC4A7	7.9E-258	0.278175	0.251	0.056	1.3E-253	Saliva_Acir	SLC4A7
LUC7L23	1E-257	0.273913	0.227	0.048	1.7E-253	Saliva_Acir	LUC7L2
BDH1	2E-257	0.259117	0.219	0.046	3.1E-253	Saliva_Acir	BDH1
SAT22	1.4E-256	0.426186	0.54	0.197	2.3E-252	Saliva_Acir	SAT2
AP1M2	2.8E-256	0.347618	0.274	0.068	4.4E-252	Saliva_Acir	AP1M2
PLD1	3E-256	0.288967	0.207	0.041	4.8E-252	Saliva_Acir	PLD1
RNF132	3.2E-256	0.45491	0.469	0.161	5E-252	Saliva_Acir	RNF13
PAPSS11	3.3E-256	0.397532	0.356	0.104	5.2E-252	Saliva_Acir	PAPSS1
TRAK2	1.5E-255	0.267222	0.21	0.042	2.4E-251	Saliva_Acir	TRAK2
LTBP31	1.8E-255	0.41947	0.44	0.145	2.8E-251	Saliva_Acir	LTBP3
EIF3B	1.9E-255	0.366784	0.354	0.103	3E-251	Saliva_Acir	EIF3B
KAT6B2	8.3E-255	0.355457	0.288	0.073	1.3E-250	Saliva_Acir	KAT6B
ERBB3	2.3E-254	0.403792	0.461	0.155	3.7E-250	Saliva_Acir	ERBB3
TM9SF22	3.5E-254	0.522581	0.509	0.188	5.6E-250	Saliva_Acir	TM9SF2
MAP7	5.6E-254	0.386435	0.316	0.085	8.9E-250	Saliva_Acir	MAP7
HSPA9	6.2E-254	0.417067	0.465	0.158	9.8E-250	Saliva_Acir	HSPA9
SDF2L12	1E-253	0.436314	0.578	0.223	1.7E-249	Saliva_Acir	SDF2L1
TRIM444	2.1E-253	0.366742	0.317	0.085	3.3E-249	Saliva_Acir	TRIM44
MRPL12	4.8E-253	0.358062	0.388	0.12	7.6E-249	Saliva_Acir	MRPL12
MOAP1	1.1E-252	0.28771	0.287	0.071	1.7E-248	Saliva_Acir	MOAP1
AGPAT3	1.1E-252	0.303769	0.261	0.062	1.7E-248	Saliva_Acir	AGPAT3
SEL1L1	2.4E-252	0.308089	0.324	0.088	3.8E-248	Saliva_Acir	SEL1L
RTCB	2.7E-252	0.374761	0.39	0.121	4.4E-248	Saliva_Acir	RTCB
SMAD41	2.9E-252	0.337064	0.261	0.062	4.7E-248	Saliva_Acir	SMAD4
EFCAB141	6.4E-252	0.378037	0.36	0.107	1E-247	Saliva_Acir	EFCAB14
MAGT11	1E-251	0.402445	0.397	0.125	1.6E-247	Saliva_Acir	MAGT1
MBNL22	4.7E-251	0.421344	0.375	0.113	7.5E-247	Saliva_Acir	MBNL2
SFXN4	4.8E-251	0.304077	0.231	0.051	7.6E-247	Saliva_Acir	SFXN4
NUMA12	8.8E-251	0.386911	0.38	0.115	1.4E-246	Saliva_Acir	NUMA1
VPS13C3	5.3E-250	0.299019	0.325	0.088	8.4E-246	Saliva_Acir	VPS13C

ENDOG1	9.8E-250	0.308349	0.256	0.06	1.6E-245	Saliva_Acir	ENDOG
PEBP14	1.1E-249	0.772526	0.818	0.448	1.8E-245	Saliva_Acir	PEBP1
ZMPSTE24	2.6E-249	0.283898	0.288	0.074	4.1E-245	Saliva_Acir	ZMPSTE24
SRGAP3	5E-249	0.283677	0.177	0.032	7.9E-245	Saliva_Acir	SRGAP3
GRN1	8.6E-249	0.530995	0.699	0.304	1.4E-244	Saliva_Acir	GRN
GAS2L11	1.7E-248	0.253965	0.224	0.048	2.6E-244	Saliva_Acir	GAS2L1
FAM134C	3.2E-248	0.331273	0.291	0.075	5.1E-244	Saliva_Acir	FAM134C
FUCA22	6.3E-248	0.295553	0.308	0.082	1E-243	Saliva_Acir	FUCA2
DGAT1	1.8E-247	0.337673	0.282	0.072	2.9E-243	Saliva_Acir	DGAT1
PLXNB2	2.1E-247	0.359441	0.451	0.15	3.4E-243	Saliva_Acir	PLXNB2
KIF13B1	3E-247	0.273578	0.227	0.05	4.8E-243	Saliva_Acir	KIF13B
REEP54	3.6E-247	0.53384	0.702	0.3	5.7E-243	Saliva_Acir	REEP5
BCLAF14	2.3E-246	0.465834	0.521	0.193	3.6E-242	Saliva_Acir	BCLAF1
PLOD1	2.8E-246	0.257635	0.2	0.04	4.5E-242	Saliva_Acir	PLOD1
URI1	6.3E-246	0.357958	0.413	0.132	1E-241	Saliva_Acir	URI1
DPM31	6.9E-246	0.4215	0.486	0.174	1.1E-241	Saliva_Acir	DPM3
PRDX24	2.8E-245	0.551058	0.698	0.304	4.5E-241	Saliva_Acir	PRDX2
HEXB2	5.9E-245	0.428905	0.459	0.156	9.4E-241	Saliva_Acir	HEXB
MAVS1	6.6E-245	0.294347	0.221	0.048	1.1E-240	Saliva_Acir	MAVS
NECAB3	7.8E-245	0.265187	0.199	0.04	1.2E-240	Saliva_Acir	NECAB3
NCOA4	9.3E-245	0.436793	0.459	0.161	1.5E-240	Saliva_Acir	NCOA4
BAIAP2	1.4E-244	0.396236	0.381	0.118	2.3E-240	Saliva_Acir	BAIAP2
C11orf54	2.1E-244	0.327892	0.247	0.058	3.3E-240	Saliva_Acir	C11orf54
COX8A1	2.8E-244	0.741087	0.783	0.429	4.5E-240	Saliva_Acir	COX8A
NAAA	2.9E-244	0.276357	0.312	0.084	4.6E-240	Saliva_Acir	NAAA
VPS51	3.7E-244	0.399701	0.452	0.155	5.9E-240	Saliva_Acir	VPS51
ATOX12	1.5E-243	0.567042	0.636	0.266	2.3E-239	Saliva_Acir	ATOX1
RUNX1	2.2E-243	0.36543	0.343	0.098	3.6E-239	Saliva_Acir	RUNX1
FAM111A1	2.3E-243	0.275597	0.219	0.047	3.6E-239	Saliva_Acir	FAM111A
TARS	6.3E-243	0.341654	0.301	0.082	1E-238	Saliva_Acir	TARS
SAR1B1	9.9E-243	0.478095	0.482	0.176	1.6E-238	Saliva_Acir	SAR1B
MRPL21	1.2E-242	0.407967	0.475	0.169	2E-238	Saliva_Acir	MRPL21
RBM263	1.3E-242	0.390119	0.328	0.093	2E-238	Saliva_Acir	RBM26
CCDC472	2.7E-242	0.423016	0.409	0.134	4.4E-238	Saliva_Acir	CCDC47
PNKP2	3E-242	0.321973	0.287	0.075	4.7E-238	Saliva_Acir	PNKP
JADE1	3.9E-242	0.268385	0.23	0.051	6.2E-238	Saliva_Acir	JADE1
UBR31	4.2E-242	0.281841	0.224	0.049	6.7E-238	Saliva_Acir	UBR3
ANKRD17	6.9E-242	0.385711	0.344	0.101	1.1E-237	Saliva_Acir	ANKRD17
CA21	1.4E-241	0.274196	0.337	0.093	2.3E-237	Saliva_Acir	CA2
ALG21	2E-241	0.3495	0.277	0.072	3.2E-237	Saliva_Acir	ALG2
PAM2	2.6E-241	0.338239	0.42	0.136	4.2E-237	Saliva_Acir	PAM
SRSF103	3.6E-241	0.467731	0.442	0.151	5.7E-237	Saliva_Acir	SRSF10
SNRNP25	1.4E-240	0.316315	0.324	0.092	2.3E-236	Saliva_Acir	SNRNP25
OSTC1	2.6E-240	0.567323	0.633	0.271	4.2E-236	Saliva_Acir	OSTC
PRKACB1	3.3E-240	0.325738	0.334	0.096	5.3E-236	Saliva_Acir	PRKACB
VKORC1L1	5.8E-240	0.294188	0.243	0.057	9.2E-236	Saliva_Acir	VKORC1L1
RPS6KB21	6E-239	0.359236	0.355	0.106	9.6E-235	Saliva_Acir	RPS6KB2

FAM107B2	6.4E-239	0.60693	0.664	0.291	1E-234	Saliva_Acir	FAM107B
AVL9	1.5E-238	0.257557	0.192	0.038	2.4E-234	Saliva_Acir	AVL9
PPP2R4	3.4E-238	0.34753	0.311	0.086	5.3E-234	Saliva_Acir	PPP2R4
SCFD12	3E-237	0.372042	0.358	0.109	4.8E-233	Saliva_Acir	SCFD1
TMEM192	6.5E-237	0.271975	0.237	0.055	1E-232	Saliva_Acir	TMEM192
SMAD52	2.7E-236	0.337454	0.317	0.09	4.4E-232	Saliva_Acir	SMAD5
ENTPD41	1.5E-235	0.296606	0.245	0.058	2.5E-231	Saliva_Acir	ENTPD4
TMED44	3E-235	0.358081	0.47	0.167	4.7E-231	Saliva_Acir	TMED4
ZFPL1	4.3E-235	0.32013	0.28	0.074	6.9E-231	Saliva_Acir	ZFPL1
MLXIP	6.5E-235	0.391297	0.331	0.097	1E-230	Saliva_Acir	MLXIP
TARBP1	6.8E-235	0.253174	0.179	0.034	1.1E-230	Saliva_Acir	TARBP1
NME35	8.5E-235	0.384327	0.538	0.203	1.3E-230	Saliva_Acir	NME3
TMEM184A	1.3E-234	0.310083	0.195	0.039	2.1E-230	Saliva_Acir	TMEM184A
PFKL	1.4E-234	0.342176	0.384	0.121	2.3E-230	Saliva_Acir	PFKL
GANAB1	2.2E-234	0.366102	0.336	0.099	3.6E-230	Saliva_Acir	GANAB
TMEM167A	1.7E-233	0.530199	0.558	0.23	2.6E-229	Saliva_Acir	TMEM167A
ZNF1483	2.6E-233	0.369373	0.38	0.122	4.1E-229	Saliva_Acir	ZNF148
DDB1	2.9E-233	0.347609	0.358	0.109	4.7E-229	Saliva_Acir	DDB1
IL17RC	6.4E-233	0.296869	0.246	0.06	1E-228	Saliva_Acir	IL17RC
TFCP2L11	8.3E-233	0.266804	0.209	0.045	1.3E-228	Saliva_Acir	TFCP2L1
TCEAL3	9.9E-233	0.299653	0.316	0.089	1.6E-228	Saliva_Acir	TCEAL3
TMEM251	2.5E-231	0.313986	0.283	0.075	4E-227	Saliva_Acir	TMEM251
NBAS	2.9E-231	0.267347	0.219	0.049	4.7E-227	Saliva_Acir	NBAS
MRPL521	6E-231	0.445154	0.552	0.218	9.6E-227	Saliva_Acir	MRPL52
MARCKSL1	7E-231	0.30081	0.536	0.199	1.1E-226	Saliva_Acir	MARCKSL1
HSD17B12	9.6E-231	0.352583	0.353	0.108	1.5E-226	Saliva_Acir	HSD17B12
ECHS11	2.1E-230	0.443909	0.513	0.198	3.4E-226	Saliva_Acir	ECHS1
TMEM981	3.7E-230	0.295938	0.279	0.072	6E-226	Saliva_Acir	TMEM98
TPCN1	5.6E-230	0.305292	0.222	0.05	8.9E-226	Saliva_Acir	TPCN1
COMTD11	1.5E-229	0.358649	0.414	0.139	2.4E-225	Saliva_Acir	COMTD1
CDK5RAP2	5.6E-229	0.304623	0.224	0.051	8.9E-225	Saliva_Acir	CDK5RAP2
C21orf21	2.3E-228	0.272913	0.317	0.09	3.6E-224	Saliva_Acir	C21orf2
FAM98A1	3.6E-228	0.272955	0.239	0.057	5.7E-224	Saliva_Acir	FAM98A
VPS364	3.8E-228	0.349151	0.352	0.108	6.1E-224	Saliva_Acir	VPS36
ARHGAP5	1.3E-227	0.479054	0.513	0.199	2.1E-223	Saliva_Acir	ARHGAP5
UBE2Z	1.5E-227	0.310076	0.312	0.089	2.4E-223	Saliva_Acir	UBE2Z
EIF4B2	2.1E-227	0.543272	0.656	0.293	3.3E-223	Saliva_Acir	EIF4B
FKBP142	2.7E-227	0.298286	0.196	0.04	4.3E-223	Saliva_Acir	FKBP14
DSP	4.2E-227	0.283381	0.472	0.168	6.8E-223	Saliva_Acir	DSP
PRKCSH	7.8E-227	0.396702	0.42	0.144	1.2E-222	Saliva_Acir	PRKCSH
MAPK131	1.1E-226	0.306232	0.343	0.105	1.7E-222	Saliva_Acir	MAPK13
ZNF3581	1.1E-226	0.2717	0.224	0.052	1.8E-222	Saliva_Acir	ZNF358
DYNC1H13	1.5E-226	0.371136	0.404	0.134	2.4E-222	Saliva_Acir	DYNC1H1
CLDN7	2.2E-226	0.337932	0.605	0.232	3.6E-222	Saliva_Acir	CLDN7
ZNF33A	2.4E-226	0.338881	0.316	0.092	3.8E-222	Saliva_Acir	ZNF33A
P4HA21	6.1E-226	0.293295	0.212	0.047	9.7E-222	Saliva_Acir	P4HA2
SMARCA21	9.5E-226	0.361788	0.352	0.109	1.5E-221	Saliva_Acir	SMARCA2

SEC23B1	2.6E-225	0.406449	0.343	0.106	4.1E-221	Saliva_Acir	SEC23B
SLC11A2	1.2E-224	0.347875	0.337	0.102	1.9E-220	Saliva_Acir	SLC11A2
NUCB11	3.2E-224	0.422566	0.52	0.2	5.1E-220	Saliva_Acir	NUCB1
ALG52	4.8E-224	0.380821	0.426	0.149	7.7E-220	Saliva_Acir	ALG5
SCAMP1	8.1E-224	0.313051	0.31	0.089	1.3E-219	Saliva_Acir	SCAMP1
SMPD1	1E-223	0.296853	0.303	0.085	1.6E-219	Saliva_Acir	SMPD1
ZNF37A5	4.8E-223	0.28036	0.226	0.053	7.6E-219	Saliva_Acir	ZNF37A
STT3B1	9.8E-223	0.375424	0.391	0.128	1.6E-218	Saliva_Acir	STT3B
PKP21	2E-222	0.273064	0.21	0.047	3.3E-218	Saliva_Acir	PKP2
ASH1L2	2.1E-222	0.436887	0.455	0.164	3.3E-218	Saliva_Acir	ASH1L
NEDD91	3.4E-222	0.288197	0.268	0.07	5.4E-218	Saliva_Acir	NEDD9
TRIM271	3.4E-222	0.28671	0.247	0.061	5.5E-218	Saliva_Acir	TRIM27
DCAF7	5.4E-222	0.29596	0.306	0.087	8.5E-218	Saliva_Acir	DCAF7
IVNS1ABP2	2E-221	0.38245	0.419	0.144	3.2E-217	Saliva_Acir	IVNS1ABP
KIAA11093	2.5E-221	0.367254	0.291	0.082	3.9E-217	Saliva_Acir	KIAA1109
USP331	3.2E-221	0.294694	0.286	0.078	5.1E-217	Saliva_Acir	USP33
ABHD17A3	7.4E-221	0.25108	0.358	0.111	1.2E-216	Saliva_Acir	ABHD17A
PIAS2	1.1E-220	0.299776	0.306	0.085	1.8E-216	Saliva_Acir	PIAS2
INTS64	1.2E-220	0.500351	0.394	0.131	1.8E-216	Saliva_Acir	INTS6
DHCR241	1.2E-220	0.3671	0.317	0.094	1.9E-216	Saliva_Acir	DHCR24
MPG2	1.4E-220	0.327226	0.463	0.167	2.3E-216	Saliva_Acir	MPG
BIRC61	2.3E-220	0.372951	0.343	0.106	3.7E-216	Saliva_Acir	BIRC6
DNAJC13	1.4E-219	0.354601	0.444	0.158	2.2E-215	Saliva_Acir	DNAJC1
SYNJ2BP1	1.8E-219	0.313146	0.258	0.067	2.9E-215	Saliva_Acir	SYNJ2BP
BPTF2	5.2E-219	0.35502	0.445	0.158	8.3E-215	Saliva_Acir	BPTF
IGF2R	1.7E-218	0.353971	0.247	0.062	2.7E-214	Saliva_Acir	IGF2R
PIGT2	3.5E-218	0.362677	0.443	0.158	5.6E-214	Saliva_Acir	PIGT
ATAD3A	5.3E-218	0.251027	0.166	0.031	8.4E-214	Saliva_Acir	ATAD3A
MKKS1	9.3E-218	0.367081	0.432	0.154	1.5E-213	Saliva_Acir	MKKS
HIF1A	1.3E-217	0.353948	0.447	0.16	2.1E-213	Saliva_Acir	HIF1A
VPS26B	7.5E-217	0.256256	0.253	0.065	1.2E-212	Saliva_Acir	VPS26B
PHF33	1.5E-215	0.378678	0.383	0.127	2.4E-211	Saliva_Acir	PHF3
PTGR11	8.7E-215	0.420216	0.325	0.099	1.4E-210	Saliva_Acir	PTGR1
SPCS32	1.5E-214	0.401301	0.581	0.244	2.3E-210	Saliva_Acir	SPCS3
SREK1	2E-214	0.391137	0.39	0.131	3.2E-210	Saliva_Acir	SREK1
SLC35F5	5.2E-214	0.328273	0.277	0.076	8.4E-210	Saliva_Acir	SLC35F5
PPFIBP2	6.5E-214	0.28147	0.192	0.042	1E-209	Saliva_Acir	PPFIBP2
PCMTD12	6.6E-214	0.410199	0.464	0.174	1E-209	Saliva_Acir	PCMTD1
NUDT31	7E-214	0.264552	0.328	0.098	1.1E-209	Saliva_Acir	NUDT3
VSIG2	9.9E-214	0.385042	0.448	0.151	1.6E-209	Saliva_Acir	VSIG2
PFN2	9.9E-214	0.285059	0.281	0.078	1.6E-209	Saliva_Acir	PFN2
LRRC41	2.8E-213	0.334399	0.293	0.084	4.5E-209	Saliva_Acir	LRRC41
DSC21	2.9E-213	0.308756	0.249	0.065	4.6E-209	Saliva_Acir	DSC2
UTRN3	3.8E-213	0.258502	0.36	0.111	6.1E-209	Saliva_Acir	UTRN
BTBD61	7.1E-213	0.284324	0.24	0.061	1.1E-208	Saliva_Acir	BTBD6
DNAJC16	1.6E-212	0.250172	0.179	0.037	2.5E-208	Saliva_Acir	DNAJC16
MANF1	1.8E-212	0.446485	0.553	0.228	2.9E-208	Saliva_Acir	MANF

TMEM123:	1.9E-212	0.488953	0.636	0.275	3E-208	Saliva_Acir	TMEM123
ANAPC54	2.1E-212	0.343248	0.436	0.156	3.3E-208	Saliva_Acir	ANAPC5
SH3YL1	2.1E-212	0.477878	0.508	0.203	3.3E-208	Saliva_Acir	SH3YL1
EFR3A1	5E-212	0.298433	0.285	0.08	8E-208	Saliva_Acir	EFR3A
MFSD6L	6.1E-212	0.250229	0.135	0.022	9.8E-208	Saliva_Acir	MFSD6L
MKLN12	7.5E-212	0.352119	0.338	0.105	1.2E-207	Saliva_Acir	MKLN1
TMEM259:	1.8E-211	0.341359	0.399	0.136	2.8E-207	Saliva_Acir	TMEM259
UBE4A	2.3E-211	0.28719	0.273	0.075	3.6E-207	Saliva_Acir	UBE4A
MPST	3.3E-211	0.397863	0.44	0.161	5.2E-207	Saliva_Acir	MPST
PLD31	1.1E-210	0.268914	0.447	0.159	1.7E-206	Saliva_Acir	PLD3
PER2	1.3E-210	0.311777	0.289	0.082	2E-206	Saliva_Acir	PER2
FDPS2	1.8E-210	0.435237	0.482	0.186	2.9E-206	Saliva_Acir	FDPS
CLINT11	5.2E-210	0.410513	0.454	0.169	8.4E-206	Saliva_Acir	CLINT1
ACBD31	8.3E-210	0.353362	0.359	0.118	1.3E-205	Saliva_Acir	ACBD3
SENP62	2.4E-209	0.29283	0.336	0.104	3.8E-205	Saliva_Acir	SENP6
TNRC6B2	4.2E-209	0.365	0.455	0.166	6.8E-205	Saliva_Acir	TNRC6B
AP3B12	9.4E-209	0.312103	0.291	0.084	1.5E-204	Saliva_Acir	AP3B1
CORO1B	1.2E-208	0.325587	0.513	0.2	2E-204	Saliva_Acir	CORO1B
MDH2	2.1E-208	0.388703	0.582	0.245	3.4E-204	Saliva_Acir	MDH2
IQGAP12	2.3E-208	0.452969	0.585	0.252	3.6E-204	Saliva_Acir	IQGAP1
CD46	3.6E-208	0.473498	0.676	0.305	5.7E-204	Saliva_Acir	CD46
MARCH6	4.7E-208	0.403803	0.436	0.159	7.5E-204	Saliva_Acir	MARCH6
PGLS	6.7E-208	0.377198	0.593	0.256	1.1E-203	Saliva_Acir	PGLS
MGAT4B	7.2E-207	0.278597	0.27	0.075	1.1E-202	Saliva_Acir	MGAT4B
ANKRD91	1.2E-205	0.331084	0.302	0.09	1.9E-201	Saliva_Acir	ANKRD9
INSR1	1.8E-205	0.386978	0.373	0.125	2.8E-201	Saliva_Acir	INSR
UROS1	2.3E-204	0.252761	0.315	0.095	3.7E-200	Saliva_Acir	UROS
IPO7	2.5E-204	0.299431	0.324	0.1	4E-200	Saliva_Acir	IPO7
IFRD2	6.6E-204	0.282705	0.308	0.092	1.1E-199	Saliva_Acir	IFRD2
LARP1B1	1.6E-203	0.287201	0.293	0.084	2.6E-199	Saliva_Acir	LARP1B
POLR2H	3.5E-203	0.317177	0.404	0.142	5.7E-199	Saliva_Acir	POLR2H
PLA2G12A	1.6E-202	0.304152	0.302	0.092	2.6E-198	Saliva_Acir	PLA2G12A
CHMP3	6.8E-202	0.325196	0.444	0.163	1.1E-197	Saliva_Acir	CHMP3
DHDDS	7.7E-202	0.256094	0.196	0.044	1.2E-197	Saliva_Acir	DHDDS
MGST21	1.1E-201	0.398271	0.509	0.203	1.7E-197	Saliva_Acir	MGST2
SGMS1	1.4E-201	0.284682	0.216	0.052	2.2E-197	Saliva_Acir	SGMS1
NSMCE4A2	3.4E-201	0.256302	0.276	0.078	5.4E-197	Saliva_Acir	NSMCE4A
C12orf573	4.6E-201	0.508729	0.717	0.35	7.3E-197	Saliva_Acir	C12orf57
SEC24A	5.6E-201	0.274948	0.269	0.075	9E-197	Saliva_Acir	SEC24A
ELP2	1.6E-200	0.290572	0.274	0.077	2.5E-196	Saliva_Acir	ELP2
SLC25A1	1.8E-200	0.317691	0.295	0.088	2.9E-196	Saliva_Acir	SLC25A1
NEAT1	1.9E-200	0.620167	0.946	0.677	3E-196	Saliva_Acir	NEAT1
RPL393	2.3E-200	0.768537	0.919	0.672	3.7E-196	Saliva_Acir	RPL39
NCKAP1	5.1E-200	0.339447	0.406	0.144	8.2E-196	Saliva_Acir	NCKAP1
KIAA20131	6.6E-200	0.321167	0.279	0.081	1E-195	Saliva_Acir	KIAA2013
THUMPD1.	1.3E-199	0.266906	0.32	0.098	2.1E-195	Saliva_Acir	THUMPD1
COG5	2.7E-199	0.256725	0.22	0.054	4.2E-195	Saliva_Acir	COG5

DNAJC213	2.9E-199	0.281923	0.352	0.116	4.6E-195	Saliva_Acir	DNAJC21
KCNK1	9.8E-199	0.314755	0.359	0.119	1.6E-194	Saliva_Acir	KCNK1
SLC39A9	1.2E-198	0.25616	0.225	0.057	2E-194	Saliva_Acir	SLC39A9
TM9SF4	2.8E-198	0.286007	0.239	0.063	4.5E-194	Saliva_Acir	TM9SF4
DHRSX	3.7E-198	0.292233	0.243	0.065	6E-194	Saliva_Acir	DHRSX
ZNF2922	6.4E-198	0.428866	0.441	0.167	1E-193	Saliva_Acir	ZNF292
RPS242	7.7E-198	0.606784	0.95	0.837	1.2E-193	Saliva_Acir	RPS24
ZDHHC9	1.1E-197	0.252014	0.223	0.056	1.8E-193	Saliva_Acir	ZDHHC9
PIGH	1.1E-197	0.291332	0.28	0.082	1.8E-193	Saliva_Acir	PIGH
DICER12	2.7E-197	0.286541	0.319	0.099	4.4E-193	Saliva_Acir	DICER1
MSRB22	3E-197	0.308968	0.367	0.125	4.9E-193	Saliva_Acir	MSRB2
COX7A13	4.1E-197	0.377002	0.39	0.134	6.6E-193	Saliva_Acir	COX7A1
ZBTB381	8.7E-197	0.35828	0.439	0.165	1.4E-192	Saliva_Acir	ZBTB38
CSNK1A11	2.8E-196	0.376567	0.611	0.265	4.5E-192	Saliva_Acir	CSNK1A1
FBXW4	3.2E-196	0.265454	0.223	0.056	5.1E-192	Saliva_Acir	FBXW4
TMED21	1.2E-195	0.475521	0.698	0.339	2E-191	Saliva_Acir	TMED2
RARS	1.9E-195	0.313871	0.279	0.082	3E-191	Saliva_Acir	RARS
ECHDC21	1.9E-195	0.390898	0.378	0.132	3E-191	Saliva_Acir	ECHDC2
PEMT	1.9E-195	0.307837	0.237	0.062	3.1E-191	Saliva_Acir	PEMT
SERP12	9.6E-195	0.651207	0.833	0.541	1.5E-190	Saliva_Acir	SERP1
NDUFA43	9.8E-195	0.642024	0.844	0.532	1.6E-190	Saliva_Acir	NDUFA4
CAPNS11	1.5E-194	0.411008	0.563	0.243	2.4E-190	Saliva_Acir	CAPNS1
C16orf13	9.4E-193	0.332789	0.51	0.206	1.5E-188	Saliva_Acir	C16orf13
GOLGA5	1.1E-192	0.308176	0.255	0.071	1.8E-188	Saliva_Acir	GOLGA5
CRELD21	2.2E-192	0.29236	0.405	0.145	3.5E-188	Saliva_Acir	CRELD2
TMEM592	8.5E-192	0.645347	0.881	0.604	1.4E-187	Saliva_Acir	TMEM59
TNKS2	1.6E-191	0.31336	0.344	0.114	2.5E-187	Saliva_Acir	TNKS2
RALGAPA1	4.3E-191	0.259942	0.256	0.071	6.8E-187	Saliva_Acir	RALGAPA1
GLT8D12	2.1E-190	0.267977	0.287	0.085	3.4E-186	Saliva_Acir	GLT8D1
NDUFA10	2.5E-190	0.338944	0.46	0.178	4E-186	Saliva_Acir	NDUFA10
MGST35	5.2E-190	0.41284	0.724	0.327	8.2E-186	Saliva_Acir	MGST3
EEF1A13	1.5E-189	0.609058	0.97	0.914	2.4E-185	Saliva_Acir	EEF1A1
N4BP2L25	2.1E-189	0.511162	0.629	0.287	3.3E-185	Saliva_Acir	N4BP2L2
ALG31	6.1E-189	0.251242	0.307	0.096	9.7E-185	Saliva_Acir	ALG3
LMF21	6.6E-189	0.278998	0.286	0.086	1.1E-184	Saliva_Acir	LMF2
GNAQ1	1.6E-188	0.347624	0.423	0.159	2.6E-184	Saliva_Acir	GNAQ
NDUFAB14	2E-188	0.396609	0.557	0.24	3.1E-184	Saliva_Acir	NDUFAB1
MESDC23	2E-188	0.350914	0.438	0.167	3.2E-184	Saliva_Acir	MESDC2
SLC35A4	5.2E-188	0.258535	0.267	0.077	8.3E-184	Saliva_Acir	SLC35A4
KIAA1522	8.1E-188	0.339818	0.24	0.066	1.3E-183	Saliva_Acir	KIAA1522
TXNDC152	9E-188	0.268095	0.351	0.118	1.4E-183	Saliva_Acir	TXNDC15
VKORC13	3E-187	0.318947	0.533	0.218	4.7E-183	Saliva_Acir	VKORC1
IFT201	9E-187	0.328595	0.414	0.154	1.4E-182	Saliva_Acir	IFT20
TMEM14B	1E-186	0.419154	0.566	0.246	1.6E-182	Saliva_Acir	TMEM14B
NDUFS62	1.6E-186	0.478195	0.658	0.317	2.5E-182	Saliva_Acir	NDUFS6
EVI52	1.7E-186	0.262093	0.261	0.075	2.7E-182	Saliva_Acir	EVI5
CYB5A2	7.8E-186	0.310181	0.462	0.177	1.3E-181	Saliva_Acir	CYB5A

PPIP5K21	1.3E-185	0.27964	0.238	0.065	2.1E-181	Saliva_Acir	PPIP5K2
NFATC2IP1	1.9E-185	0.255347	0.266	0.076	3E-181	Saliva_Acir	NFATC2IP
TMEM205:	4E-185	0.294355	0.499	0.199	6.3E-181	Saliva_Acir	TMEM205
VEZF11	4.7E-185	0.263492	0.28	0.084	7.5E-181	Saliva_Acir	VEZF1
STX16	5.2E-185	0.261232	0.272	0.08	8.4E-181	Saliva_Acir	STX16
PTP4A22	7.7E-185	0.404031	0.619	0.285	1.2E-180	Saliva_Acir	PTP4A2
CMPK1	1.1E-184	0.40102	0.634	0.294	1.7E-180	Saliva_Acir	CMPK1
MAN1A21	1.1E-184	0.299368	0.328	0.109	1.7E-180	Saliva_Acir	MAN1A2
FDFT1	5.8E-184	0.415452	0.496	0.206	9.3E-180	Saliva_Acir	FDFT1
UBA51	1.1E-183	0.250518	0.296	0.092	1.8E-179	Saliva_Acir	UBA5
TMX21	1.7E-183	0.265289	0.307	0.097	2.7E-179	Saliva_Acir	TMX2
SRP721	4.3E-183	0.293503	0.454	0.178	6.9E-179	Saliva_Acir	SRP72
SLC25A34	1.2E-182	0.584088	0.779	0.449	2E-178	Saliva_Acir	SLC25A3
TMBIM62	1.5E-182	0.576659	0.884	0.572	2.4E-178	Saliva_Acir	TMBIM6
SGMS2	2.5E-182	0.260475	0.212	0.054	4E-178	Saliva_Acir	SGMS2
MAP2K24	3.1E-182	0.268409	0.482	0.192	5E-178	Saliva_Acir	MAP2K2
COL4A3BP	3.5E-182	0.318064	0.343	0.117	5.5E-178	Saliva_Acir	COL4A3BP
THOC22	4.3E-182	0.273185	0.355	0.121	6.9E-178	Saliva_Acir	THOC2
MIOS	7.1E-182	0.279054	0.193	0.047	1.1E-177	Saliva_Acir	MIOS
GPD2	1.9E-181	0.252403	0.199	0.049	3E-177	Saliva_Acir	GPD2
ZNF242	3E-181	0.285291	0.374	0.134	4.8E-177	Saliva_Acir	ZNF24
CTBS2	4.1E-181	0.302428	0.304	0.099	6.5E-177	Saliva_Acir	CTBS
GDE1	4.8E-181	0.325483	0.304	0.098	7.6E-177	Saliva_Acir	GDE1
ATP5A11	8E-181	0.368853	0.662	0.306	1.3E-176	Saliva_Acir	ATP5A1
APIP1	2.1E-180	0.283937	0.294	0.093	3.4E-176	Saliva_Acir	APIP
G3BP21	2.5E-180	0.289148	0.442	0.169	4E-176	Saliva_Acir	G3BP2
ATP6V0B2	4E-180	0.481875	0.734	0.38	6.4E-176	Saliva_Acir	ATP6V0B
AHCYL11	7.2E-180	0.335653	0.416	0.16	1.2E-175	Saliva_Acir	AHCYL1
DDRGK11	8.9E-180	0.330407	0.395	0.147	1.4E-175	Saliva_Acir	DDRGK1
LSR	1.3E-179	0.374542	0.358	0.127	2E-175	Saliva_Acir	LSR
RBBP7	1.4E-179	0.288722	0.407	0.151	2.2E-175	Saliva_Acir	RBBP7
MPC13	2.3E-179	0.390521	0.489	0.204	3.7E-175	Saliva_Acir	MPC1
SIL11	3.3E-179	0.350922	0.327	0.11	5.3E-175	Saliva_Acir	SIL1
MRPL204	1.6E-178	0.362469	0.589	0.264	2.5E-174	Saliva_Acir	MRPL20
PARK76	4.8E-178	0.437328	0.724	0.348	7.7E-174	Saliva_Acir	PARK7
BEND7	1.2E-177	0.27606	0.212	0.055	1.9E-173	Saliva_Acir	BEND7
ACO21	2.9E-177	0.255655	0.297	0.095	4.6E-173	Saliva_Acir	ACO2
SYPL11	3E-177	0.367837	0.58	0.257	4.7E-173	Saliva_Acir	SYPL1
UHMK11	4.4E-177	0.278398	0.281	0.087	7E-173	Saliva_Acir	UHMK1
LARP1	4.7E-177	0.29238	0.33	0.112	7.5E-173	Saliva_Acir	LARP1
DERL12	5.9E-177	0.253026	0.364	0.13	9.4E-173	Saliva_Acir	DERL1
TMEM248	6.8E-177	0.354028	0.462	0.188	1.1E-172	Saliva_Acir	TMEM248
TTC17	1.3E-176	0.331543	0.291	0.092	2.1E-172	Saliva_Acir	TTC17
TTLL72	1.7E-176	0.279087	0.227	0.062	2.7E-172	Saliva_Acir	TTLL7
DNAJC152	2.1E-176	0.30954	0.467	0.184	3.3E-172	Saliva_Acir	DNAJC15
CHCHD13	1.1E-175	0.313362	0.427	0.168	1.8E-171	Saliva_Acir	CHCHD1
DDX1	1.3E-175	0.283397	0.309	0.101	2.1E-171	Saliva_Acir	DDX1

VPS13D	1.6E-175	0.269715	0.28	0.086	2.5E-171	Saliva_Acir	VPS13D
H3F3A6	2.5E-175	0.560213	0.857	0.471	4.1E-171	Saliva_Acir	H3F3A
EBP1	3.1E-175	0.304145	0.335	0.115	4.9E-171	Saliva_Acir	EBP
SRGAP1	4.1E-175	0.322253	0.322	0.107	6.6E-171	Saliva_Acir	SRGAP1
EI24	5.4E-175	0.339388	0.453	0.182	8.6E-171	Saliva_Acir	EI24
PLEKHJ1	7.2E-175	0.268083	0.421	0.162	1.1E-170	Saliva_Acir	PLEKHJ1
UQCR10	4.4E-174	0.565726	0.753	0.433	7E-170	Saliva_Acir	UQCR10
COQ4	5.1E-174	0.273429	0.286	0.09	8.2E-170	Saliva_Acir	COQ4
NDUFV11	8.6E-174	0.299789	0.43	0.167	1.4E-169	Saliva_Acir	NDUFV1
XAB21	2E-173	0.275281	0.287	0.092	3.2E-169	Saliva_Acir	XAB2
TCEAL8	1.3E-172	0.292201	0.419	0.161	2E-168	Saliva_Acir	TCEAL8
SPPL2A2	1.8E-172	0.294341	0.352	0.126	2.9E-168	Saliva_Acir	SPPL2A
TSPYL11	2.6E-172	0.283979	0.397	0.15	4.2E-168	Saliva_Acir	TSPYL1
COBLL13	1E-171	0.277562	0.295	0.095	1.6E-167	Saliva_Acir	COBLL1
MLF21	1.5E-171	0.36487	0.509	0.219	2.4E-167	Saliva_Acir	MLF2
PSMB52	9.8E-171	0.389871	0.531	0.235	1.6E-166	Saliva_Acir	PSMB5
ASAH12	1.4E-170	0.348459	0.597	0.272	2.3E-166	Saliva_Acir	ASAH1
SQLE	4.9E-170	0.300691	0.258	0.077	7.8E-166	Saliva_Acir	SQLE
PUM11	5E-170	0.280607	0.376	0.139	7.9E-166	Saliva_Acir	PUM1
BECN1	1.4E-169	0.280267	0.301	0.099	2.3E-165	Saliva_Acir	BECN1
BOD1L14	1.5E-169	0.295987	0.348	0.123	2.4E-165	Saliva_Acir	BOD1L1
NME42	7.7E-169	0.295873	0.376	0.138	1.2E-164	Saliva_Acir	NME4
ATP5G34	7.9E-169	0.472625	0.702	0.355	1.3E-164	Saliva_Acir	ATP5G3
PPIB3	1.8E-168	0.413193	0.754	0.387	2.8E-164	Saliva_Acir	PPIB
RAB3IP	2.4E-168	0.277056	0.263	0.079	3.8E-164	Saliva_Acir	RAB3IP
SPAG16	1.4E-167	0.287272	0.284	0.09	2.3E-163	Saliva_Acir	SPAG16
HMGCS1	4.6E-167	0.269481	0.283	0.09	7.3E-163	Saliva_Acir	HMGCS1
C14orf1	7.8E-167	0.302825	0.352	0.128	1.2E-162	Saliva_Acir	C14orf1
ARL32	2.4E-166	0.253549	0.324	0.111	3.9E-162	Saliva_Acir	ARL3
IDH22	2.6E-166	0.315037	0.459	0.189	4.2E-162	Saliva_Acir	IDH2
GOLPH3	4.1E-166	0.299061	0.418	0.165	6.5E-162	Saliva_Acir	GOLPH3
SSFA21	6E-166	0.361262	0.413	0.163	9.5E-162	Saliva_Acir	SSFA2
SETD51	9.1E-166	0.273872	0.329	0.114	1.5E-161	Saliva_Acir	SETD5
NDUFC1	1.2E-165	0.382005	0.597	0.275	1.9E-161	Saliva_Acir	NDUFC1
C8orf595	1.2E-165	0.347835	0.52	0.229	2E-161	Saliva_Acir	C8orf59
USP471	1.3E-165	0.260973	0.31	0.104	2.1E-161	Saliva_Acir	USP47
ATF6	4.9E-165	0.266252	0.293	0.096	7.8E-161	Saliva_Acir	ATF6
SERINC32	6.1E-165	0.341161	0.482	0.202	9.7E-161	Saliva_Acir	SERINC3
ATF7IP1	6.9E-164	0.258721	0.221	0.062	1.1E-159	Saliva_Acir	ATF7IP
EIF3A1	8.7E-164	0.399213	0.6	0.287	1.4E-159	Saliva_Acir	EIF3A
UBE2G21	1.5E-163	0.254596	0.304	0.102	2.4E-159	Saliva_Acir	UBE2G2
CHD61	3.7E-162	0.267321	0.251	0.075	5.9E-158	Saliva_Acir	CHD6
ARFGEF11	4.8E-162	0.298517	0.272	0.087	7.6E-158	Saliva_Acir	ARFGEF1
RAB131	5.5E-162	0.403044	0.491	0.213	8.8E-158	Saliva_Acir	RAB13
SRSF6	1.2E-161	0.273096	0.373	0.14	1.9E-157	Saliva_Acir	SRSF6
QARS	2.1E-161	0.279919	0.424	0.171	3.3E-157	Saliva_Acir	QARS
PRPSAP11	2.7E-161	0.296737	0.325	0.115	4.3E-157	Saliva_Acir	PRPSAP1

MRPS331	6E-161	0.273462	0.379	0.144	9.5E-157	Saliva_Acir	MRPS33
TFAP2A	1.3E-160	0.280977	0.191	0.05	2.1E-156	Saliva_Acir	TFAP2A
EXOC7	1.8E-160	0.27247	0.293	0.098	2.9E-156	Saliva_Acir	EXOC7
CCAR11	2E-160	0.297653	0.297	0.1	3.2E-156	Saliva_Acir	CCAR1
MAT2A1	3E-160	0.276625	0.385	0.145	4.9E-156	Saliva_Acir	MAT2A
SYNCRIP2	5.7E-160	0.325978	0.438	0.18	9.1E-156	Saliva_Acir	SYNCRIP
CCDC14	6E-159	0.288869	0.247	0.075	9.6E-155	Saliva_Acir	CCDC14
ANKIB1	6.9E-159	0.253477	0.245	0.074	1.1E-154	Saliva_Acir	ANKIB1
CEP412	1.1E-158	0.356574	0.293	0.098	1.8E-154	Saliva_Acir	CEP41
CERS41	6E-158	0.266944	0.224	0.065	9.6E-154	Saliva_Acir	CERS4
TUG12	6.9E-158	0.311562	0.348	0.128	1.1E-153	Saliva_Acir	TUG1
PPCS	1.7E-157	0.274923	0.453	0.19	2.7E-153	Saliva_Acir	PPCS
AHSA21	2.9E-157	0.264392	0.233	0.069	4.7E-153	Saliva_Acir	AHSA2
STRN	3.1E-157	0.258215	0.273	0.088	5E-153	Saliva_Acir	STRN
GADD45GI	4.2E-157	0.254781	0.599	0.273	6.8E-153	Saliva_Acir	GADD45GIP1
TRAPPC2L1	7.2E-157	0.262947	0.461	0.191	1.1E-152	Saliva_Acir	TRAPPC2L
TCF254	1.5E-156	0.35146	0.602	0.287	2.4E-152	Saliva_Acir	TCF25
CYC1	1.6E-155	0.276513	0.512	0.225	2.6E-151	Saliva_Acir	CYC1
FNIP11	2.3E-155	0.261681	0.258	0.081	3.6E-151	Saliva_Acir	FNIP1
ARPC1A1	2.9E-155	0.317167	0.487	0.211	4.7E-151	Saliva_Acir	ARPC1A
TNPO11	3.4E-155	0.277416	0.335	0.122	5.3E-151	Saliva_Acir	TNPO1
ATP5B	8.5E-155	0.41693	0.696	0.365	1.4E-150	Saliva_Acir	ATP5B
GNPAT	1.7E-154	0.257988	0.255	0.081	2.7E-150	Saliva_Acir	GNPAT
TRIM562	1.8E-154	0.273468	0.244	0.074	2.8E-150	Saliva_Acir	TRIM56
CREBZF1	1E-153	0.292077	0.235	0.071	1.7E-149	Saliva_Acir	CREBZF
SRSF112	3.7E-153	0.339992	0.621	0.296	5.9E-149	Saliva_Acir	SRSF11
OCIAD13	1.2E-152	0.297892	0.539	0.247	1.9E-148	Saliva_Acir	OCIAD1
TCTN31	1.6E-152	0.253173	0.231	0.069	2.6E-148	Saliva_Acir	TCTN3
AGO31	3E-152	0.257589	0.24	0.073	4.8E-148	Saliva_Acir	AGO3
CSDE11	5.6E-152	0.409624	0.674	0.352	8.9E-148	Saliva_Acir	CSDE1
AUP11	6.4E-152	0.257661	0.464	0.199	1E-147	Saliva_Acir	AUP1
SEC61G1	3.2E-151	0.530681	0.722	0.409	5.1E-147	Saliva_Acir	SEC61G
USP481	3.8E-151	0.265633	0.277	0.093	6.1E-147	Saliva_Acir	USP48
KMT2A4	7.8E-151	0.25619	0.343	0.126	1.2E-146	Saliva_Acir	KMT2A
AAMDC	4.7E-150	0.281238	0.38	0.149	7.5E-146	Saliva_Acir	AAMDC
ARHGEF10	2.2E-149	0.259601	0.19	0.051	3.5E-145	Saliva_Acir	ARHGEF10
SMDT11	9.3E-149	0.35099	0.693	0.366	1.5E-144	Saliva_Acir	SMDT1
AC159540.	1.2E-148	0.336744	0.207	0.059	1.9E-144	Saliva_Acir	AC159540.1
MRPS161	1.3E-148	0.250956	0.38	0.151	2.1E-144	Saliva_Acir	MRPS16
UQCRC1	2.3E-148	0.29278	0.445	0.188	3.7E-144	Saliva_Acir	UQCRC1
RPL304	4.5E-148	0.537397	0.951	0.842	7.2E-144	Saliva_Acir	RPL30
ERP441	5.3E-148	0.316179	0.415	0.173	8.4E-144	Saliva_Acir	ERP44
ETFB2	1.1E-147	0.260205	0.575	0.266	1.8E-143	Saliva_Acir	ETFB
TUBA1C1	1.3E-147	0.299588	0.361	0.138	2E-143	Saliva_Acir	TUBA1C
DHRS7B	1.6E-147	0.264591	0.233	0.072	2.5E-143	Saliva_Acir	DHRS7B
PRPF40A2	2.3E-147	0.254969	0.439	0.183	3.7E-143	Saliva_Acir	PRPF40A
TMEM33	1.5E-146	0.250466	0.301	0.107	2.3E-142	Saliva_Acir	TMEM33

CAPN2	3.2E-146	0.336901	0.631	0.306	5.1E-142	Saliva_Acir	CAPN2
RRAGD1	1.4E-145	0.295474	0.263	0.089	2.2E-141	Saliva_Acir	RRAGD
MRPL401	2.9E-145	0.251386	0.397	0.161	4.7E-141	Saliva_Acir	MRPL40
C1orf271	4E-145	0.252597	0.23	0.072	6.4E-141	Saliva_Acir	C1orf27
ZRANB23	4.5E-145	0.263763	0.434	0.182	7.1E-141	Saliva_Acir	ZRANB2
MDM42	4.6E-145	0.327886	0.349	0.133	7.3E-141	Saliva_Acir	MDM4
ABHD2	2.7E-144	0.266879	0.314	0.111	4.3E-140	Saliva_Acir	ABHD2
CD92	5.7E-144	0.290333	0.823	0.445	9E-140	Saliva_Acir	CD9
SUGP2	9.1E-144	0.268791	0.255	0.083	1.4E-139	Saliva_Acir	SUGP2
NENF3	1.6E-143	0.343954	0.644	0.322	2.6E-139	Saliva_Acir	NENF
ZFR	1.8E-143	0.257	0.296	0.106	2.8E-139	Saliva_Acir	ZFR
APRT2	5.2E-142	0.292006	0.712	0.362	8.4E-138	Saliva_Acir	APRT
DERL21	1.3E-141	0.273723	0.407	0.17	2.1E-137	Saliva_Acir	DERL2
FIS12	4E-141	0.30782	0.657	0.332	6.4E-137	Saliva_Acir	FIS1
SMC51	6.4E-141	0.25098	0.301	0.109	1E-136	Saliva_Acir	SMC5
LMAN21	9.8E-141	0.263288	0.539	0.25	1.6E-136	Saliva_Acir	LMAN2
RPS121	6.8E-140	0.498401	0.956	0.86	1.1E-135	Saliva_Acir	RPS12
SCAF114	8.6E-140	0.259998	0.5	0.226	1.4E-135	Saliva_Acir	SCAF11
HINT13	1.7E-139	0.528553	0.834	0.565	2.8E-135	Saliva_Acir	HINT1
PABPN11	7.3E-138	0.262443	0.369	0.146	1.2E-133	Saliva_Acir	PABPN1
WDR34	1E-137	0.25091	0.254	0.085	1.6E-133	Saliva_Acir	WDR34
SF3B13	2E-137	0.26277	0.53	0.247	3.3E-133	Saliva_Acir	SF3B1
UBE3A1	2.5E-137	0.260443	0.342	0.132	4E-133	Saliva_Acir	UBE3A
CCDC64B	2.8E-137	0.266654	0.293	0.104	4.4E-133	Saliva_Acir	CCDC64B
NARS	8.4E-135	0.252511	0.349	0.138	1.3E-130	Saliva_Acir	NARS
ARF41	2.8E-134	0.456367	0.73	0.411	4.4E-130	Saliva_Acir	ARF4
VGLL42	2.3E-133	0.25504	0.337	0.132	3.6E-129	Saliva_Acir	VGLL4
GNB11	3.5E-133	0.29155	0.459	0.209	5.6E-129	Saliva_Acir	GNB1
ZC3H11A	4.6E-133	0.289508	0.386	0.162	7.4E-129	Saliva_Acir	ZC3H11A
DDT1	1.2E-132	0.337871	0.637	0.335	1.8E-128	Saliva_Acir	DDT
VPS283	1.6E-132	0.295087	0.657	0.343	2.6E-128	Saliva_Acir	VPS28
ZMYM2	6.5E-131	0.280042	0.33	0.129	1E-126	Saliva_Acir	ZMYM2
MAP3K13	7.4E-131	0.351464	0.498	0.236	1.2E-126	Saliva_Acir	MAP3K13
SEPW13	4.1E-130	0.280771	0.672	0.352	6.6E-126	Saliva_Acir	SEPW1
SMAD21	9E-130	0.259787	0.318	0.123	1.4E-125	Saliva_Acir	SMAD2
FBXW2	1.3E-129	0.253591	0.253	0.087	2E-125	Saliva_Acir	FBXW2
SRRM24	1.7E-129	0.299177	0.661	0.341	2.7E-125	Saliva_Acir	SRRM2
LAMP23	6E-129	0.259793	0.501	0.231	9.6E-125	Saliva_Acir	LAMP2
PLEKHA1	8.5E-129	0.250222	0.321	0.123	1.4E-124	Saliva_Acir	PLEKHA1
MAGI11	9.3E-129	0.259518	0.21	0.066	1.5E-124	Saliva_Acir	MAGI1
PPP1R13B1	1.2E-127	0.27447	0.186	0.055	1.9E-123	Saliva_Acir	PPP1R13B
AHI12	1.5E-126	0.256556	0.296	0.109	2.3E-122	Saliva_Acir	AHI1
SSR21	4.4E-126	0.261997	0.764	0.422	7.1E-122	Saliva_Acir	SSR2
TMEM219	1.5E-125	0.272965	0.628	0.325	2.4E-121	Saliva_Acir	TMEM219
TXNL4A1	3.1E-123	0.272338	0.478	0.228	5E-119	Saliva_Acir	TXNL4A
DBI2	8.3E-122	0.285745	0.752	0.408	1.3E-117	Saliva_Acir	DBI
UBXN43	8.7E-122	0.258362	0.586	0.295	1.4E-117	Saliva_Acir	UBXN4

ATP5D1	2.5E-119	0.325946	0.758	0.44	4E-115	Saliva_Acir	ATP5D
EEF1B22	8.9E-118	0.350578	0.858	0.592	1.4E-113	Saliva_Acir	EEF1B2
TPTEP11	6.7E-116	0.281777	0.211	0.071	1.1E-111	Saliva_Acir	TPTEP1
RPL81	2.6E-115	0.406277	0.963	0.847	4.2E-111	Saliva_Acir	RPL8
NDUFB21	2.8E-115	0.256446	0.7	0.368	4.4E-111	Saliva_Acir	NDUFB2
TXNL11	1.7E-113	0.259782	0.496	0.245	2.7E-109	Saliva_Acir	TXNL1
OST41	2.7E-113	0.427036	0.792	0.495	4.3E-109	Saliva_Acir	OST4
LAMP1	9.9E-113	0.253355	0.571	0.293	1.6E-108	Saliva_Acir	LAMP1
RPL7A5	9.1E-109	0.423026	0.944	0.811	1.4E-104	Saliva_Acir	RPL7A
BAZ2B	1.3E-108	0.260466	0.335	0.144	2.1E-104	Saliva_Acir	BAZ2B
COPE1	1.1E-101	0.324654	0.719	0.435	1.73E-97	Saliva_Acir	COPE
RAB2A	1.1E-100	0.367172	0.663	0.386	1.74E-96	Saliva_Acir	RAB2A
MYEOV21	1.2E-99	0.251801	0.643	0.356	1.92E-95	Saliva_Acir	MYEOV2
PAWR1	9.1E-99	0.294078	0.299	0.127	1.45E-94	Saliva_Acir	PAWR
EEF2	4.99E-98	0.348431	0.864	0.603	7.97E-94	Saliva_Acir	EEF2
ATP5J5	1.01E-92	0.313428	0.709	0.431	1.6E-88	Saliva_Acir	ATP5J
PRDX13	1.58E-92	0.272374	0.746	0.446	2.52E-88	Saliva_Acir	PRDX1
UQCRQ2	1.67E-91	0.375793	0.765	0.494	2.66E-87	Saliva_Acir	UQCRQ
COX7B2	1.16E-90	0.301062	0.684	0.404	1.85E-86	Saliva_Acir	COX7B
RPL225	2.6E-84	0.363585	0.887	0.69	4.14E-80	Saliva_Acir	RPL22
GSTP13	2.2E-81	0.279612	0.822	0.552	3.51E-77	Saliva_Acir	GSTP1
EDF11	1.27E-77	0.309312	0.825	0.621	2.02E-73	Saliva_Acir	EDF1
RPL372	3.36E-75	0.31266	0.927	0.846	5.37E-71	Saliva_Acir	RPL37
CHCHD22	2.9E-63	0.285365	0.814	0.617	4.63E-59	Saliva_Acir	CHCHD2
RPL244	3.13E-61	0.260909	0.906	0.8	4.99E-57	Saliva_Acir	RPL24
FOSB5	7.52E-40	0.390909	0.388	0.249	1.2E-35	Saliva_Acir	FOSB
RNASE11	0	4.536628	0.543	0.091	0	Saliva_Ma	RNASE1
C1QA	0	4.328184	0.839	0.035	0	Saliva_Ma	C1QA
C1QB	0	4.029195	0.783	0.025	0	Saliva_Ma	C1QB
HLA.DPA1	0	3.831968	0.865	0.188	0	Saliva_Ma	HLA.DPA1
HLA.DRA1	0	3.600264	0.921	0.248	0	Saliva_Ma	HLA.DRA
LGMN	0	3.542903	0.608	0.125	0	Saliva_Ma	LGMN
C1QC	0	3.388076	0.726	0.025	0	Saliva_Ma	C1QC
MS4A6A	0	3.227864	0.774	0.043	0	Saliva_Ma	MS4A6A
HLA.DPB1	0	3.154108	0.868	0.215	0	Saliva_Ma	HLA.DPB1
HLA.DQB1	0	3.10073	0.735	0.128	0	Saliva_Ma	HLA.DQB1
FOLR2	0	3.0336	0.495	0.018	0	Saliva_Ma	FOLR2
DAB22	0	3.031895	0.538	0.092	0	Saliva_Ma	DAB2
HLA.DRB1	0	3.021573	0.892	0.215	0	Saliva_Ma	HLA.DRB1
SLC40A1	0	2.908339	0.482	0.075	0	Saliva_Ma	SLC40A1
CD141	0	2.890355	0.686	0.103	0	Saliva_Ma	CD14
MS4A7	0	2.720673	0.587	0.023	0	Saliva_Ma	MS4A7
CCL3L31	0	2.643319	0.137	0.003	0	Saliva_Ma	CCL3L3
CYBB	0	2.540877	0.551	0.018	0	Saliva_Ma	CYBB
CSF1R	0	2.528597	0.542	0.018	0	Saliva_Ma	CSF1R
HLA.DQA1	0	2.513443	0.662	0.104	0	Saliva_Ma	HLA.DQA1
STAB11	0	2.509788	0.406	0.013	0	Saliva_Ma	STAB1

HLA.DMA1	0	2.487036	0.708	0.137	0	Saliva_Ma	HLA.DMA
F13A1	0	2.439403	0.384	0.03	0	Saliva_Ma	F13A1
CD741	0	2.42994	0.962	0.359	0	Saliva_Ma	CD74
AIF1	0	2.399023	0.83	0.111	0	Saliva_Ma	AIF1
TGFBI2	0	2.351417	0.5	0.061	0	Saliva_Ma	TGFBI
VSIG4	0	2.346205	0.505	0.024	0	Saliva_Ma	VSIG4
MAFB1	0	2.30088	0.534	0.086	0	Saliva_Ma	MAFB
TYROBP	0	2.250959	0.894	0.12	0	Saliva_Ma	TYROBP
MS4A4A	0	2.169998	0.408	0.016	0	Saliva_Ma	MS4A4A
A2M2	0	2.158169	0.567	0.082	0	Saliva_Ma	A2M
ADAP21	0	2.125192	0.433	0.014	0	Saliva_Ma	ADAP2
FCER1G	0	2.119683	0.782	0.107	0	Saliva_Ma	FCER1G
FGL21	0	2.112529	0.606	0.096	0	Saliva_Ma	FGL2
MPEG1	0	1.973403	0.394	0.013	0	Saliva_Ma	MPEG1
HLA.DMB1	0	1.959165	0.523	0.065	0	Saliva_Ma	HLA.DMB
FCGR2B1	0	1.920783	0.375	0.028	0	Saliva_Ma	FCGR2B
MNDA	0	1.919622	0.395	0.026	0	Saliva_Ma	MNDA
LILRB5	0	1.887652	0.307	0.004	0	Saliva_Ma	LILRB5
EMB2	0	1.834886	0.368	0.039	0	Saliva_Ma	EMB
FCGR2A	0	1.778418	0.399	0.038	0	Saliva_Ma	FCGR2A
CD163	0	1.74161	0.301	0.018	0	Saliva_Ma	CD163
IGSF6	0	1.738459	0.394	0.028	0	Saliva_Ma	IGSF6
CLEC10A	0	1.713929	0.327	0.028	0	Saliva_Ma	CLEC10A
GPR34	0	1.674044	0.296	0.011	0	Saliva_Ma	GPR34
MSR1	0	1.606019	0.305	0.02	0	Saliva_Ma	MSR1
C3AR1	0	1.522844	0.283	0.013	0	Saliva_Ma	C3AR1
CD841	0	1.459289	0.285	0.02	0	Saliva_Ma	CD84
MARCH1	0	1.381517	0.267	0.016	0	Saliva_Ma	MARCH1
CX3CR1	0	1.379847	0.206	0.007	0	Saliva_Ma	CX3CR1
NCKAP1L2	0	1.35218	0.273	0.021	0	Saliva_Ma	NCKAP1L
HPGDS	0	1.338605	0.242	0.013	0	Saliva_Ma	HPGDS
SIGLEC1	0	1.308737	0.184	0.006	0	Saliva_Ma	SIGLEC1
P2RY14	0	1.304382	0.206	0.008	0	Saliva_Ma	P2RY14
SCN9A	0	1.277126	0.199	0.007	0	Saliva_Ma	SCN9A
CCR1	0	1.235297	0.204	0.009	0	Saliva_Ma	CCR1
P2RY13	0	1.095996	0.166	0.003	0	Saliva_Ma	P2RY13
PLD4	0	1.026408	0.17	0.009	0	Saliva_Ma	PLD4
CD209	0	0.953527	0.125	0.003	0	Saliva_Ma	CD209
LILRB4	3.2E-306	1.114502	0.199	0.013	5.1E-302	Saliva_Ma	LILRB4
SAMHD12	3.2E-306	1.706328	0.458	0.067	5.1E-302	Saliva_Ma	SAMHD1
CD68	5.7E-304	2.147915	0.588	0.116	9.1E-300	Saliva_Ma	CD68
PTPRC1	1.9E-303	1.742694	0.587	0.105	3E-299	Saliva_Ma	PTPRC
HLA.DQA2	4.1E-286	2.520554	0.462	0.072	6.5E-282	Saliva_Ma	HLA.DQA2
CLEC7A	2.3E-279	1.340725	0.278	0.027	3.6E-275	Saliva_Ma	CLEC7A
COLEC121	7.7E-278	1.815285	0.278	0.028	1.2E-273	Saliva_Ma	COLEC12
FPR3	2.8E-277	1.121176	0.22	0.017	4.4E-273	Saliva_Ma	FPR3
CMKLR11	3.1E-272	0.905149	0.152	0.008	5E-268	Saliva_Ma	CMKLR1

KCTD122	1.9E-271	1.893053	0.502	0.09	3.1E-267	Saliva_Ma(	KCTD12
ZEB22	1.5E-264	1.914516	0.487	0.086	2.4E-260	Saliva_Ma(	ZEB2
CST31	1.8E-264	2.468819	0.984	0.591	2.9E-260	Saliva_Ma(	CST3
CD4	2.9E-264	1.249334	0.285	0.03	4.6E-260	Saliva_Ma(	CD4
ITGAM	6E-263	1.050337	0.197	0.015	9.6E-259	Saliva_Ma(	ITGAM
CTSS1	1.3E-262	2.08299	0.736	0.213	2E-258	Saliva_Ma(	CTSS
CTSZ3	1.3E-261	2.216808	0.69	0.189	2.1E-257	Saliva_Ma(	CTSZ
LRRC25	1E-254	0.79541	0.152	0.009	1.6E-250	Saliva_Ma(	LRRC25
MAF	1.2E-249	2.124105	0.449	0.08	2E-245	Saliva_Ma(	MAF
LILRB3	9.3E-249	0.5895	0.114	0.005	1.5E-244	Saliva_Ma(	LILRB3
FTL1	3E-242	1.987457	0.993	0.895	4.8E-238	Saliva_Ma(	FTL
CD33	7.6E-236	0.84847	0.162	0.011	1.2E-231	Saliva_Ma(	CD33
GPRIN32	5.8E-235	0.838357	0.162	0.011	9.3E-231	Saliva_Ma(	GPRIN3
TFEC	3.4E-230	0.895828	0.15	0.01	5.4E-226	Saliva_Ma(	TFEC
HLA.DOA1	1.9E-229	1.011805	0.193	0.016	3E-225	Saliva_Ma(	HLA.DOA
SPI1	1.3E-228	1.402628	0.388	0.061	2.1E-224	Saliva_Ma(	SPI1
CIITA	2.2E-227	0.814159	0.148	0.009	3.5E-223	Saliva_Ma(	CIITA
MAMDC21	5.8E-223	1.760022	0.298	0.039	9.3E-219	Saliva_Ma(	MAMDC2
SLC15A3	3.5E-221	1.052547	0.204	0.018	5.6E-217	Saliva_Ma(	SLC15A3
TTN2	3.8E-221	1.238453	0.146	0.01	6.1E-217	Saliva_Ma(	TTN
ABCA61	1.6E-220	1.469148	0.24	0.025	2.6E-216	Saliva_Ma(	ABCA6
FCGRT2	1.5E-219	2.365832	0.751	0.275	2.4E-215	Saliva_Ma(	FCGRT
MEF2C3	5.2E-217	1.624554	0.43	0.079	8.4E-213	Saliva_Ma(	MEF2C
SLC8A12	6.2E-214	0.967193	0.175	0.014	9.9E-210	Saliva_Ma(	SLC8A1
HLA.DRB5	3.1E-208	2.354192	0.56	0.134	4.9E-204	Saliva_Ma(	HLA.DRB5
CPVL	3.3E-208	1.972615	0.384	0.067	5.3E-204	Saliva_Ma(	CPVL
LILRB2	1.9E-204	0.863085	0.148	0.011	3E-200	Saliva_Ma(	LILRB2
TLR42	3.5E-202	1.02743	0.168	0.014	5.6E-198	Saliva_Ma(	TLR4
CCL4L21	4.9E-197	2.497146	0.173	0.015	7.9E-193	Saliva_Ma(	CCL4L2
ALOX5AP1	1.4E-196	1.616844	0.448	0.089	2.3E-192	Saliva_Ma(	ALOX5AP
ADCY71	3.4E-193	0.973265	0.191	0.018	5.4E-189	Saliva_Ma(	ADCY7
TBXAS1	5.4E-192	1.092161	0.235	0.028	8.7E-188	Saliva_Ma(	TBXAS1
NPC21	2.1E-190	1.853976	0.85	0.438	3.3E-186	Saliva_Ma(	NPC2
RETN	5.6E-187	0.686526	0.106	0.006	8.9E-183	Saliva_Ma(	RETN
CYTH41	1.1E-178	1.136735	0.236	0.029	1.7E-174	Saliva_Ma(	CYTH4
MT.ATP68	1.7E-178	1.1368	0.998	0.898	2.7E-174	Saliva_Ma(	MT.ATP6
NCF2	1.4E-177	0.995517	0.206	0.023	2.2E-173	Saliva_Ma(	NCF2
GRN2	1.4E-177	1.831512	0.736	0.31	2.2E-173	Saliva_Ma(	GRN
FCGR3A	3.9E-177	1.667388	0.226	0.027	6.3E-173	Saliva_Ma(	FCGR3A
GLIPR1	3.3E-176	1.562338	0.417	0.088	5.3E-172	Saliva_Ma(	GLIPR1
CYBA2	1.1E-174	1.36911	0.904	0.438	1.8E-170	Saliva_Ma(	CYBA
LAPTM51	2.8E-173	1.429843	0.619	0.171	4.4E-169	Saliva_Ma(	LAPTM5
LTC4S1	3.4E-171	1.253494	0.236	0.031	5.5E-167	Saliva_Ma(	LTC4S
DOCK22	7.6E-171	0.915067	0.186	0.019	1.2E-166	Saliva_Ma(	DOCK2
FGD2	9.7E-170	0.846841	0.139	0.011	1.5E-165	Saliva_Ma(	FGD2
PTPRE1	3.4E-168	1.277216	0.31	0.052	5.4E-164	Saliva_Ma(	PTPRE
CTSC2	2.1E-167	1.820254	0.478	0.119	3.3E-163	Saliva_Ma(	CTSC

MT.CYB8	3.7E-167	0.988089	0.998	0.933	6E-163	Saliva_Ma(	MT.CYB
PLEK	2.1E-166	1.271085	0.316	0.053	3.4E-162	Saliva_Ma(	PLEK
RNASE6	1.4E-165	1.203281	0.267	0.039	2.2E-161	Saliva_Ma(	RNASE6
MT.ND38	4.6E-165	1.040091	1	0.793	7.4E-161	Saliva_Ma(	MT.ND3
RGL11	7.1E-165	1.230782	0.191	0.021	1.1E-160	Saliva_Ma(	RGL1
TREM2	5E-164	1.184165	0.15	0.013	8E-160	Saliva_Ma(	TREM2
ITGAX	4.4E-162	0.840753	0.153	0.014	6.9E-158	Saliva_Ma(	ITGAX
IL10RA2	1E-161	1.18162	0.282	0.044	1.7E-157	Saliva_Ma(	IL10RA
NCF41	3.6E-161	1.165671	0.269	0.041	5.7E-157	Saliva_Ma(	NCF4
MARCO	6.2E-159	1.624845	0.144	0.013	9.9E-155	Saliva_Ma(	MARCO
BMP2K	2.4E-156	1.27729	0.264	0.042	3.9E-152	Saliva_Ma(	BMP2K
VENTX	2.4E-154	0.339604	0.049	0.001	3.8E-150	Saliva_Ma(	VENTX
FAM105A	3.7E-153	1.129234	0.244	0.036	5.9E-149	Saliva_Ma(	FAM105A
P2RY6	3.4E-150	0.463019	0.072	0.003	5.4E-146	Saliva_Ma(	P2RY6
HAVCR2	2E-149	0.848643	0.175	0.02	3.2E-145	Saliva_Ma(	HAVCR2
MT.ND58	2.6E-149	1.177708	0.964	0.701	4.1E-145	Saliva_Ma(	MT.ND5
GNAI23	7.9E-147	1.648309	0.608	0.224	1.3E-142	Saliva_Ma(	GNAI2
PLXDC22	8.9E-147	1.331894	0.262	0.043	1.4E-142	Saliva_Ma(	PLXDC2
RASSF4	1.6E-146	1.08635	0.233	0.034	2.6E-142	Saliva_Ma(	RASSF4
PYCARD	1.7E-146	1.49778	0.536	0.165	2.7E-142	Saliva_Ma(	PYCARD
CD362	1.9E-146	1.194175	0.159	0.017	3E-142	Saliva_Ma(	CD36
FRMD4B3	1.9E-145	1.876238	0.446	0.126	3E-141	Saliva_Ma(	FRMD4B
RBPJ2	2.4E-145	1.887617	0.551	0.19	3.8E-141	Saliva_Ma(	RBPJ
PLEKHO11	1.4E-144	1.38119	0.374	0.083	2.3E-140	Saliva_Ma(	PLEKHO1
RAB321	2.3E-144	1.50095	0.377	0.088	3.6E-140	Saliva_Ma(	RAB32
GFRA2	5.1E-144	0.979659	0.159	0.017	8.1E-140	Saliva_Ma(	GFRA2
UNC93B11	1.1E-142	1.389173	0.321	0.065	1.7E-138	Saliva_Ma(	UNC93B1
CD86	1.3E-142	0.890275	0.184	0.022	2.2E-138	Saliva_Ma(	CD86
ITGB21	6.2E-142	1.207295	0.446	0.107	1E-137	Saliva_Ma(	ITGB2
FAM26F	9E-141	1.173508	0.265	0.044	1.4E-136	Saliva_Ma(	FAM26F
DOK21	2.7E-138	1.254601	0.318	0.061	4.3E-134	Saliva_Ma(	DOK2
IQGAP22	1.9E-136	1.750225	0.424	0.114	3.1E-132	Saliva_Ma(	IQGAP2
IFI30	4.4E-136	0.915801	0.166	0.019	7E-132	Saliva_Ma(	IFI30
CCL18	4.6E-136	1.671462	0.161	0.018	7.4E-132	Saliva_Ma(	CCL18
MT.ND18	1.3E-131	1.042252	0.995	0.927	2E-127	Saliva_Ma(	MT.ND1
NCF12	8.7E-131	1.023889	0.247	0.04	1.4E-126	Saliva_Ma(	NCF1
MT.CO28	1.1E-129	0.705011	0.998	0.959	1.8E-125	Saliva_Ma(	MT.CO2
ALOX51	8.8E-129	0.820041	0.162	0.019	1.4E-124	Saliva_Ma(	ALOX5
MT.ND48	1.7E-128	0.749414	0.998	0.953	2.6E-124	Saliva_Ma(	MT.ND4
ARHGAP1(	2.6E-127	1.579469	0.368	0.094	4.1E-123	Saliva_Ma(	ARHGAP18
MTRNR2L1	2.2E-126	1.480362	0.677	0.267	3.5E-122	Saliva_Ma(	MTRNR2L12
MT.CO37	3.7E-126	0.784541	0.998	0.977	6E-122	Saliva_Ma(	MT.CO3
PTAFR	6.2E-125	0.806161	0.148	0.017	9.9E-121	Saliva_Ma(	PTAFR
NLRP3	1.1E-124	0.576038	0.094	0.007	1.7E-120	Saliva_Ma(	NLRP3
RASSF21	1.5E-124	0.870989	0.173	0.022	2.3E-120	Saliva_Ma(	RASSF2
SH2B31	1.6E-123	1.050559	0.199	0.029	2.5E-119	Saliva_Ma(	SH2B3
ARPC1B2	5.1E-122	1.279467	0.688	0.291	8.1E-118	Saliva_Ma(	ARPC1B

S100A43	8.3E-122	1.306874	0.888	0.463	1.3E-117	Saliva_Ma	S100A4
BRI34	4.8E-121	1.623466	0.684	0.345	7.7E-117	Saliva_Ma	BRI3
SEPP11	2.4E-119	3.336406	0.572	0.264	3.9E-115	Saliva_Ma	SEPP1
RAB31	5.6E-117	1.364754	0.361	0.093	8.9E-113	Saliva_Ma	RAB31
TNFSF13B1	9.6E-116	0.974576	0.235	0.041	1.5E-111	Saliva_Ma	TNFSF13B
SRGN2	2.2E-115	0.984058	0.623	0.222	3.6E-111	Saliva_Ma	SRGN
NAIP	7.3E-115	0.943282	0.17	0.023	1.2E-110	Saliva_Ma	NAIP
RNF1302	9.2E-115	1.488194	0.439	0.14	1.5E-110	Saliva_Ma	RNF130
TPT15	2.3E-114	0.883567	0.991	0.958	3.6E-110	Saliva_Ma	TPT1
GPX1	2.9E-113	1.533077	0.758	0.426	4.7E-109	Saliva_Ma	GPX1
CCL31	1.2E-111	1.926186	0.249	0.047	2E-107	Saliva_Ma	CCL3
LILRB1	1.3E-111	0.476382	0.092	0.007	2E-107	Saliva_Ma	LILRB1
TLR7	5.2E-111	0.569074	0.083	0.006	8.2E-107	Saliva_Ma	TLR7
TNFRSF1B2	8.6E-109	0.977293	0.247	0.047	1.4E-104	Saliva_Ma	TNFRSF1B
ARRB2	1.6E-108	1.141301	0.345	0.086	2.6E-104	Saliva_Ma	ARRB2
ATP2B12	1.8E-107	1.583568	0.444	0.149	2.8E-103	Saliva_Ma	ATP2B1
SUCNR11	1.3E-106	0.386417	0.054	0.003	2.1E-102	Saliva_Ma	SUCNR1
CECR11	1.8E-106	1.025107	0.217	0.039	2.9E-102	Saliva_Ma	CECR1
EVI2B2	2.3E-106	1.09895	0.289	0.063	3.7E-102	Saliva_Ma	EVI2B
CTSD1	5.9E-106	1.754073	0.621	0.291	9.5E-102	Saliva_Ma	CTSD
RNASET22	1.4E-105	1.443431	0.569	0.235	2.3E-101	Saliva_Ma	RNASET2
MT.ND28	1.6E-105	0.860195	0.996	0.907	2.5E-101	Saliva_Ma	MT.ND2
LCP21	4.2E-105	0.799449	0.177	0.026	6.7E-101	Saliva_Ma	LCP2
LILRA6	5.5E-105	0.361877	0.054	0.003	8.8E-101	Saliva_Ma	LILRA6
CTSB	1.1E-103	1.493464	0.641	0.317	1.8E-99	Saliva_Ma	CTSB
GAS71	2.2E-103	1.093936	0.238	0.047	3.5E-99	Saliva_Ma	GAS7
LST1	1.4E-102	0.865675	0.37	0.096	2.28E-98	Saliva_Ma	LST1
ITM2B3	1.9E-102	1.242942	0.897	0.705	3.04E-98	Saliva_Ma	ITM2B
LCP11	3E-100	1.295605	0.374	0.107	4.81E-96	Saliva_Ma	LCP1
VIM5	5.2E-100	1.139877	0.894	0.492	8.27E-96	Saliva_Ma	VIM
GNB43	3.8E-99	1.005468	0.209	0.039	5.98E-95	Saliva_Ma	GNB4
THEMIS21	3.9E-99	0.913339	0.215	0.04	6.17E-95	Saliva_Ma	THEMIS2
PSAP1	4.02E-98	1.191707	0.738	0.434	6.41E-94	Saliva_Ma	PSAP
TPP1	9.33E-98	1.373452	0.339	0.096	1.49E-93	Saliva_Ma	TPP1
FYB1	1.86E-97	1.078835	0.303	0.072	2.97E-93	Saliva_Ma	FYB
FMNL32	1.95E-97	0.646793	0.106	0.011	3.1E-93	Saliva_Ma	FMNL3
PLEKHO2	1.31E-95	0.871343	0.168	0.026	2.09E-91	Saliva_Ma	PLEKHO2
HRH11	1.06E-94	1.15851	0.193	0.035	1.69E-90	Saliva_Ma	HRH1
SLC7A7	3.4E-94	0.780044	0.168	0.027	5.42E-90	Saliva_Ma	SLC7A7
MARCKS2	8.23E-94	1.63595	0.583	0.282	1.31E-89	Saliva_Ma	MARCKS
MT.CO18	1.35E-92	0.723704	0.998	0.985	2.16E-88	Saliva_Ma	MT.CO1
CPM	2.1E-92	1.321086	0.251	0.058	3.35E-88	Saliva_Ma	CPM
ATP8B4	7.77E-92	0.681646	0.101	0.011	1.24E-87	Saliva_Ma	ATP8B4
TLR1	3.37E-91	0.682046	0.094	0.009	5.37E-87	Saliva_Ma	TLR1
ADAM281	1.08E-90	0.862146	0.159	0.025	1.72E-86	Saliva_Ma	ADAM28
SCN1B2	6.14E-90	0.696808	0.11	0.013	9.79E-86	Saliva_Ma	SCN1B
RPS177	4.2E-89	0.888116	0.561	0.221	6.69E-85	Saliva_Ma	RPS17

BATF3	8.95E-89	0.600574	0.114	0.014	1.43E-84	Saliva_Ma(	BATF3
SYK	2.02E-87	0.909	0.184	0.034	3.22E-83	Saliva_Ma(	SYK
GPR651	2.73E-86	0.692113	0.152	0.023	4.35E-82	Saliva_Ma(	GPR65
TMEM176I	3.69E-86	1.201335	0.321	0.089	5.88E-82	Saliva_Ma(	TMEM176B
RGS101	3.91E-86	1.34301	0.509	0.215	6.23E-82	Saliva_Ma(	RGS10
TNFAIP8L2	1.89E-85	0.511813	0.114	0.014	3.01E-81	Saliva_Ma(	TNFAIP8L2
PTGS1	1.43E-84	0.632517	0.112	0.014	2.29E-80	Saliva_Ma(	PTGS1
LAIR1	2.23E-84	0.895678	0.168	0.029	3.56E-80	Saliva_Ma(	LAIR1
POU2F22	2.38E-84	0.914827	0.22	0.047	3.79E-80	Saliva_Ma(	POU2F2
PCDH121	4.28E-84	0.349094	0.045	0.002	6.82E-80	Saliva_Ma(	PCDH12
HCK	8.21E-81	0.631908	0.137	0.021	1.31E-76	Saliva_Ma(	HCK
SELPLG1	2.24E-80	0.785777	0.173	0.031	3.57E-76	Saliva_Ma(	SELPLG
HCST1	2.56E-80	0.86683	0.487	0.172	4.08E-76	Saliva_Ma(	HCST
LY86	3.5E-80	0.741485	0.195	0.038	5.58E-76	Saliva_Ma(	LY86
ENTPD13	4.64E-80	1.015176	0.211	0.046	7.4E-76	Saliva_Ma(	ENTPD1
UCP21	1.93E-79	1.146903	0.428	0.153	3.07E-75	Saliva_Ma(	UCP2
MYO1F1	2.02E-79	0.785573	0.208	0.043	3.23E-75	Saliva_Ma(	MYO1F
NRP13	2.26E-79	1.203288	0.235	0.056	3.61E-75	Saliva_Ma(	NRP1
KCNJ5	3.52E-79	0.407498	0.061	0.005	5.62E-75	Saliva_Ma(	KCNJ5
MAN1A12	1.01E-78	1.454102	0.296	0.085	1.61E-74	Saliva_Ma(	MAN1A1
CD3021	1.62E-78	1.114926	0.249	0.063	2.58E-74	Saliva_Ma(	CD302
IFI162	8.27E-78	1.217348	0.475	0.197	1.32E-73	Saliva_Ma(	IFI16
TNF1	2.04E-77	0.797955	0.126	0.018	3.25E-73	Saliva_Ma(	TNF
PLCB2	6.23E-77	0.511678	0.101	0.012	9.93E-73	Saliva_Ma(	PLCB2
C5AR1	1.76E-75	0.716649	0.126	0.019	2.81E-71	Saliva_Ma(	C5AR1
PLXND12	2.29E-74	0.917012	0.161	0.03	3.66E-70	Saliva_Ma(	PLXND1
MYO5A1	3.27E-73	0.893539	0.168	0.033	5.22E-69	Saliva_Ma(	MYO5A
VPS13C4	4.24E-73	1.146521	0.3	0.092	6.76E-69	Saliva_Ma(	VPS13C
CAMK1D1	5.71E-73	0.882715	0.19	0.04	9.11E-69	Saliva_Ma(	CAMK1D
TXNIP4	1.17E-72	1.158974	0.671	0.375	1.87E-68	Saliva_Ma(	TXNIP
SNX2	2.13E-72	1.415738	0.383	0.147	3.4E-68	Saliva_Ma(	SNX2
AHNAK5	9.23E-72	1.446152	0.635	0.385	1.47E-67	Saliva_Ma(	AHNAK
NRP21	2.92E-70	0.910059	0.157	0.03	4.66E-66	Saliva_Ma(	NRP2
CALM21	4.74E-70	0.938236	0.825	0.561	7.55E-66	Saliva_Ma(	CALM2
SLC43A2	1.03E-69	0.830306	0.166	0.033	1.64E-65	Saliva_Ma(	SLC43A2
DPYSL22	3.19E-69	1.042576	0.31	0.098	5.08E-65	Saliva_Ma(	DPYSL2
GAS63	3.43E-69	1.315562	0.298	0.094	5.48E-65	Saliva_Ma(	GAS6
ADRBK2	3.79E-69	0.77564	0.148	0.027	6.04E-65	Saliva_Ma(	ADRBK2
HLA.DQB2	8.54E-69	0.71665	0.144	0.025	1.36E-64	Saliva_Ma(	HLA.DQB2
SLC9A91	2.34E-68	0.646419	0.108	0.015	3.73E-64	Saliva_Ma(	SLC9A9
CCDC88A	8.14E-68	1.118534	0.3	0.095	1.3E-63	Saliva_Ma(	CCDC88A
PLTP2	1.14E-67	1.62695	0.291	0.094	1.81E-63	Saliva_Ma(	PLTP
LGALS2	1.46E-66	1.271654	0.193	0.044	2.33E-62	Saliva_Ma(	LGALS2
SLAMF8	2.43E-66	0.442388	0.074	0.008	3.88E-62	Saliva_Ma(	SLAMF8
TLR10	9.96E-66	0.312776	0.049	0.004	1.59E-61	Saliva_Ma(	TLR10
HCLS11	1.11E-65	0.868926	0.273	0.077	1.76E-61	Saliva_Ma(	HCLS1
TGFB12	7.21E-65	0.98026	0.307	0.098	1.15E-60	Saliva_Ma(	TGFB1

TM6SF11	1.01E-63	0.66226	0.114	0.018	1.62E-59	Saliva_Mac	TM6SF1
CD83	3.14E-63	1.445936	0.292	0.095	5E-59	Saliva_Mac	CD83
TAGLN23	1.11E-62	1.209475	0.653	0.408	1.78E-58	Saliva_Mac	TAGLN2
IGF12	1.89E-62	0.820287	0.17	0.036	3.01E-58	Saliva_Mac	IGF1
OSCAR	4.31E-62	0.367386	0.067	0.007	6.88E-58	Saliva_Mac	OSCAR
H3F3A7	1.33E-60	0.851567	0.778	0.477	2.12E-56	Saliva_Mac	H3F3A
DOCK101	1.44E-60	0.512389	0.11	0.017	2.3E-56	Saliva_Mac	DOCK10
PALD1	2.28E-60	0.414604	0.054	0.005	3.64E-56	Saliva_Mac	PALD1
EMP33	3.28E-60	0.907801	0.668	0.35	5.23E-56	Saliva_Mac	EMP3
IQGAP13	1.63E-59	1.345933	0.482	0.258	2.6E-55	Saliva_Mac	IQGAP1
COTL11	3.42E-59	1.062758	0.435	0.183	5.45E-55	Saliva_Mac	COTL1
EMILIN21	2.26E-58	0.739179	0.146	0.03	3.61E-54	Saliva_Mac	EMILIN2
ANKRD441	1.2E-57	0.723344	0.161	0.035	1.92E-53	Saliva_Mac	ANKRD44
DUSP62	1.47E-57	1.297863	0.329	0.127	2.35E-53	Saliva_Mac	DUSP6
FMNL12	1.95E-57	0.719265	0.229	0.063	3.11E-53	Saliva_Mac	FMNL1
BTK1	2.23E-57	0.550977	0.105	0.017	3.56E-53	Saliva_Mac	BTK
SH3BGRL2	1.33E-56	1.057552	0.489	0.243	2.13E-52	Saliva_Mac	SH3BGRL
LRP12	3.2E-56	0.897838	0.264	0.082	5.1E-52	Saliva_Mac	LRP1
RASGRP4	3.64E-56	0.266268	0.043	0.003	5.81E-52	Saliva_Mac	RASGRP4
BLVRB1	4.88E-56	1.23062	0.518	0.286	7.78E-52	Saliva_Mac	BLVRB
DOCK112	1.91E-55	0.600812	0.123	0.023	3.05E-51	Saliva_Mac	DOCK11
VASH1	4.69E-55	0.597005	0.103	0.017	7.48E-51	Saliva_Mac	VASH1
PPT1	6.95E-54	1.130245	0.327	0.129	1.11E-49	Saliva_Mac	PPT1
SPRED11	7.04E-54	0.931013	0.171	0.043	1.12E-49	Saliva_Mac	SPRED1
FCER1A	1.14E-53	1.368488	0.17	0.041	1.82E-49	Saliva_Mac	FCER1A
MTRNR2L1	4.1E-53	1.108485	0.33	0.125	6.53E-49	Saliva_Mac	MTRNR2L1
ARHGAP62	1.14E-52	0.506289	0.092	0.014	1.81E-48	Saliva_Mac	ARHGAP6
LY962	1.25E-52	0.909543	0.227	0.068	2E-48	Saliva_Mac	LY96
FPR1	6.42E-52	0.386237	0.07	0.009	1.02E-47	Saliva_Mac	FPR1
PLA2G15	7.72E-52	0.625176	0.11	0.02	1.23E-47	Saliva_Mac	PLA2G15
ARHGAP41	8.99E-52	0.739459	0.15	0.033	1.43E-47	Saliva_Mac	ARHGAP4
YWHAB3	1.04E-51	1.073174	0.608	0.412	1.67E-47	Saliva_Mac	YWHAB
CSF3R	1.11E-51	0.444406	0.096	0.015	1.77E-47	Saliva_Mac	CSF3R
OLR1	1.17E-51	0.6466	0.103	0.018	1.86E-47	Saliva_Mac	OLR1
HTR2B	1.38E-51	0.268982	0.032	0.002	2.2E-47	Saliva_Mac	HTR2B
ACP5	1.51E-51	0.988383	0.195	0.054	2.41E-47	Saliva_Mac	ACP5
SCIMP	3E-50	0.416256	0.07	0.009	4.78E-46	Saliva_Mac	SCIMP
AOAH	7.41E-50	0.420769	0.103	0.018	1.18E-45	Saliva_Mac	AOAH
MPP11	1.58E-49	0.90834	0.164	0.042	2.52E-45	Saliva_Mac	MPP1
ARHGAP24	1.71E-49	0.687218	0.152	0.036	2.72E-45	Saliva_Mac	ARHGAP24
MTSS1	5.9E-49	0.954729	0.2	0.059	9.41E-45	Saliva_Mac	MTSS1
PREX11	7.15E-49	0.51792	0.112	0.021	1.14E-44	Saliva_Mac	PREX1
CFP	1.12E-48	0.52358	0.13	0.027	1.79E-44	Saliva_Mac	CFP
FXYD63	4.01E-48	0.778768	0.132	0.029	6.4E-44	Saliva_Mac	FXYD6
GPR1551	1.02E-47	0.660884	0.128	0.028	1.63E-43	Saliva_Mac	GPR155
DOCK42	1.21E-47	0.609051	0.11	0.021	1.93E-43	Saliva_Mac	DOCK4
LMNA5	1.45E-47	1.427279	0.567	0.366	2.31E-43	Saliva_Mac	LMNA

LAT2	1.7E-47	0.675147	0.144	0.033	2.71E-43	Saliva_Ma(	LAT2
COLGALT1	1.98E-47	0.815246	0.15	0.037	3.16E-43	Saliva_Ma(	COLGALT1
PEPD2	3.01E-47	1.437527	0.269	0.101	4.8E-43	Saliva_Ma(	PEPD
APBB1IP1	3.75E-47	0.666856	0.162	0.041	5.97E-43	Saliva_Ma(	APBB1IP
CLTC1	5.46E-47	1.284883	0.374	0.184	8.7E-43	Saliva_Ma(	CLTC
MEF2A1	8.63E-47	1.050517	0.276	0.106	1.38E-42	Saliva_Ma(	MEF2A
CCL41	8.65E-47	1.822015	0.171	0.046	1.38E-42	Saliva_Ma(	CCL4
TGFBR11	1.05E-46	0.895142	0.2	0.061	1.68E-42	Saliva_Ma(	TGFBR1
ITPR24	1.4E-46	1.0433	0.233	0.08	2.24E-42	Saliva_Ma(	ITPR2
ANXA54	1.67E-46	0.944648	0.545	0.317	2.66E-42	Saliva_Ma(	ANXA5
TPM32	2.27E-46	1.073403	0.536	0.331	3.63E-42	Saliva_Ma(	TPM3
AP1B1	2.53E-46	0.93717	0.204	0.064	4.04E-42	Saliva_Ma(	AP1B1
PARP141	3.42E-46	0.938071	0.235	0.08	5.46E-42	Saliva_Ma(	PARP14
SLA1	3.83E-46	0.540787	0.119	0.024	6.11E-42	Saliva_Ma(	SLA
CD532	5.15E-46	0.747033	0.325	0.122	8.21E-42	Saliva_Ma(	CD53
SNX61	9.21E-46	1.25722	0.363	0.173	1.47E-41	Saliva_Ma(	SNX6
LPCAT22	9.54E-46	0.743574	0.152	0.038	1.52E-41	Saliva_Ma(	LPCAT2
TMEM176/	1.41E-45	0.94785	0.208	0.064	2.25E-41	Saliva_Ma(	TMEM176A
RPS243	2.26E-45	0.608288	0.958	0.839	3.6E-41	Saliva_Ma(	RPS24
TYMP1	8.82E-45	0.795324	0.394	0.184	1.41E-40	Saliva_Ma(	TYMP
FERMT31	9.55E-45	0.691099	0.177	0.049	1.52E-40	Saliva_Ma(	FERMT3
IRF5	1.09E-44	0.515908	0.083	0.014	1.74E-40	Saliva_Ma(	IRF5
MILR1	1.25E-44	0.379465	0.063	0.008	1.99E-40	Saliva_Ma(	MILR1
PARVB3	2.96E-44	0.690112	0.161	0.043	4.72E-40	Saliva_Ma(	PARVB
CD636	5.02E-44	0.613204	0.857	0.624	8.01E-40	Saliva_Ma(	CD63
ME11	5.09E-44	0.840878	0.13	0.03	8.12E-40	Saliva_Ma(	ME1
MBNL13	1E-43	0.976264	0.368	0.17	1.6E-39	Saliva_Ma(	MBNL1
CLEC4A	2.99E-43	0.456836	0.097	0.018	4.78E-39	Saliva_Ma(	CLEC4A
TMSB4X1	3.07E-43	0.312973	0.989	0.87	4.9E-39	Saliva_Ma(	TMSB4X
CCR51	4.16E-43	0.254635	0.045	0.005	6.63E-39	Saliva_Ma(	CCR5
ARPC51	1.45E-42	0.961379	0.596	0.412	2.31E-38	Saliva_Ma(	ARPC5
ZFHX32	1.57E-42	0.992483	0.242	0.089	2.5E-38	Saliva_Ma(	ZFHX3
LIPA1	1.63E-42	0.91162	0.218	0.073	2.61E-38	Saliva_Ma(	LIPA
TNNI21	1.83E-42	0.256611	0.069	0.01	2.91E-38	Saliva_Ma(	TNNI2
C16orf542	2.86E-42	0.349788	0.07	0.01	4.57E-38	Saliva_Ma(	C16orf54
NFKBID1	1.18E-41	0.748915	0.161	0.044	1.89E-37	Saliva_Ma(	NFKBID
GLIPR22	1.85E-41	0.901464	0.236	0.084	2.95E-37	Saliva_Ma(	GLIPR2
SMAP21	2.35E-41	0.744676	0.208	0.068	3.76E-37	Saliva_Ma(	SMAP2
WIPF12	6.08E-41	0.825262	0.251	0.092	9.7E-37	Saliva_Ma(	WIPF1
ATP6V0B3	8.45E-41	0.973741	0.565	0.387	1.35E-36	Saliva_Ma(	ATP6V0B
MT.ND4L8	8.77E-41	0.684862	0.619	0.416	1.4E-36	Saliva_Ma(	MT.ND4L
WSB12	2.13E-40	1.068931	0.44	0.249	3.4E-36	Saliva_Ma(	WSB1
TMEM37	2.79E-40	0.633429	0.092	0.018	4.45E-36	Saliva_Ma(	TMEM37
ARHGAP3(	3.57E-40	0.706039	0.17	0.049	5.7E-36	Saliva_Ma(	ARHGAP30
PTPN61	3.8E-40	0.699302	0.222	0.075	6.06E-36	Saliva_Ma(	PTPN6
GIMAP42	5.52E-40	0.523535	0.227	0.075	8.8E-36	Saliva_Ma(	GIMAP4
MTRNR2L1	6.49E-40	0.857557	0.26	0.097	1.03E-35	Saliva_Ma(	MTRNR2L10

IRF8	1.22E-39	0.602753	0.153	0.042	1.95E-35	Saliva_Ma	IRF8
ARHGEF61	1.48E-39	0.486158	0.097	0.02	2.36E-35	Saliva_Ma	ARHGEF6
CYP2S1	1.57E-39	0.306717	0.051	0.006	2.5E-35	Saliva_Ma	CYP2S1
APLP22	2.23E-39	1.138769	0.482	0.304	3.56E-35	Saliva_Ma	APLP2
TLN13	2.41E-39	0.993719	0.298	0.13	3.85E-35	Saliva_Ma	TLN1
PTPRO	5.48E-39	0.292826	0.047	0.005	8.74E-35	Saliva_Ma	PTPRO
TSPAN42	6.67E-39	1.125537	0.278	0.117	1.06E-34	Saliva_Ma	TSPAN4
RNF133	6.84E-39	1.080561	0.338	0.166	1.09E-34	Saliva_Ma	RNF13
PTPN181	7.05E-39	0.884478	0.238	0.09	1.12E-34	Saliva_Ma	PTPN18
SEPT111	7.57E-39	0.941271	0.231	0.085	1.21E-34	Saliva_Ma	SEPT11
AP1S21	1.74E-38	0.944454	0.294	0.125	2.77E-34	Saliva_Ma	AP1S2
ABCA11	2E-38	0.98626	0.195	0.066	3.19E-34	Saliva_Ma	ABCA1
TUBA1B3	4.58E-38	1.011393	0.561	0.353	7.31E-34	Saliva_Ma	TUBA1B
NAGA	6.32E-38	0.760319	0.15	0.043	1.01E-33	Saliva_Ma	NAGA
CEBPA	6.62E-38	0.575977	0.117	0.028	1.06E-33	Saliva_Ma	CEBPA
CSF2RA	2.18E-37	0.518124	0.117	0.028	3.47E-33	Saliva_Ma	CSF2RA
P2RX7	2.44E-37	0.435162	0.069	0.011	3.89E-33	Saliva_Ma	P2RX7
QKI2	3.37E-37	1.109286	0.341	0.171	5.38E-33	Saliva_Ma	QKI
ACSM5	5.94E-37	0.256306	0.036	0.003	9.47E-33	Saliva_Ma	ACSM5
FLI11	6.94E-37	0.621979	0.144	0.04	1.11E-32	Saliva_Ma	FLI1
IL1B	7.78E-37	1.722206	0.148	0.042	1.24E-32	Saliva_Ma	IL1B
TLR6	1.12E-36	0.257548	0.042	0.005	1.79E-32	Saliva_Ma	TLR6
KIAA10331	1.46E-36	0.953587	0.217	0.081	2.32E-32	Saliva_Ma	KIAA1033
GIMAP81	1.49E-36	0.468176	0.078	0.014	2.38E-32	Saliva_Ma	GIMAP8
ARL111	1.81E-36	0.266339	0.045	0.005	2.88E-32	Saliva_Ma	ARL11
PLXNC11	2.51E-36	0.424369	0.081	0.015	4.01E-32	Saliva_Ma	PLXNC1
DOCK82	3.03E-36	0.640039	0.168	0.052	4.83E-32	Saliva_Ma	DOCK8
CXorf211	3.15E-36	0.41578	0.067	0.011	5.02E-32	Saliva_Ma	CXorf21
DMXL2	4.28E-36	0.677041	0.125	0.032	6.83E-32	Saliva_Ma	DMXL2
LGALS13	5.18E-36	0.483386	0.688	0.409	8.26E-32	Saliva_Ma	LGALS1
BCAT1	6.12E-36	1.036375	0.2	0.072	9.76E-32	Saliva_Ma	BCAT1
SIGLEC10	7.54E-36	0.312021	0.045	0.005	1.2E-31	Saliva_Ma	SIGLEC10
ATM2	1.14E-35	0.909976	0.231	0.09	1.81E-31	Saliva_Ma	ATM
CD1C	1.48E-35	0.890017	0.097	0.021	2.35E-31	Saliva_Ma	CD1C
CKLF2	1.59E-35	1.023708	0.318	0.15	2.54E-31	Saliva_Ma	CKLF
TMEM106/	4.41E-35	0.527809	0.099	0.022	7.04E-31	Saliva_Ma	TMEM106A
MYO7A	6.57E-35	0.271072	0.034	0.003	1.05E-30	Saliva_Ma	MYO7A
VMO1	1.35E-34	0.662426	0.119	0.031	2.15E-30	Saliva_Ma	VMO1
SNX10	1.51E-34	0.664968	0.166	0.053	2.41E-30	Saliva_Ma	SNX10
CORO71	1.63E-34	0.625616	0.13	0.035	2.59E-30	Saliva_Ma	CORO7
NAGK1	5.15E-34	0.985954	0.274	0.125	8.21E-30	Saliva_Ma	NAGK
TLR2	5.18E-34	0.391949	0.07	0.013	8.26E-30	Saliva_Ma	TLR2
MTRNR2L8	7.15E-34	0.817906	0.487	0.286	1.14E-29	Saliva_Ma	MTRNR2L8
PHACTR11	1.08E-33	0.717049	0.126	0.034	1.72E-29	Saliva_Ma	PHACTR1
NUDT32	1.75E-33	0.845034	0.244	0.102	2.78E-29	Saliva_Ma	NUDT3
KIAA09301	1.97E-33	0.769131	0.193	0.069	3.15E-29	Saliva_Ma	KIAA0930
IFNGR11	4.61E-33	0.980509	0.287	0.134	7.35E-29	Saliva_Ma	IFNGR1

ARPC22	9.4E-33	0.678024	0.693	0.574	1.5E-28	Saliva_Mac	ARPC2
C1orf542	1.01E-32	0.820229	0.152	0.047	1.61E-28	Saliva_Mac	C1orf54
RYR1	1.35E-32	0.326799	0.052	0.008	2.16E-28	Saliva_Mac	RYR1
CTSH2	1.36E-32	1.136119	0.395	0.238	2.18E-28	Saliva_Mac	CTSH
VAMP81	1.88E-32	0.776932	0.579	0.409	3E-28	Saliva_Mac	VAMP8
TTYH3	2.91E-32	0.635583	0.116	0.031	4.64E-28	Saliva_Mac	TTYH3
CELF22	3.83E-32	0.781353	0.291	0.132	6.1E-28	Saliva_Mac	CELF2
ATP5E1	3.93E-32	0.580846	0.791	0.684	6.27E-28	Saliva_Mac	ATP5E
TSPAN331	5.15E-32	0.695691	0.143	0.044	8.22E-28	Saliva_Mac	TSPAN33
IL6R	9.64E-32	0.575649	0.135	0.04	1.54E-27	Saliva_Mac	IL6R
SLC12A9	9.69E-32	0.554456	0.121	0.033	1.55E-27	Saliva_Mac	SLC12A9
NINJ1	1.25E-31	1.015576	0.298	0.145	2E-27	Saliva_Mac	NINJ1
SLC4A71	1.26E-31	0.745402	0.171	0.059	2E-27	Saliva_Mac	SLC4A7
CNPY3	1.48E-31	0.771929	0.287	0.133	2.37E-27	Saliva_Mac	CNPY3
ACTR21	1.7E-31	0.988525	0.46	0.306	2.71E-27	Saliva_Mac	ACTR2
MFSD1	2.29E-31	0.995441	0.235	0.101	3.66E-27	Saliva_Mac	MFSD1
GPNMB1	3.66E-31	0.8508	0.282	0.126	5.84E-27	Saliva_Mac	GPNMB
RP11.67L2	3.78E-31	0.261573	0.042	0.005	6.03E-27	Saliva_Mac	RP11.67L2.2
RASA41	5.95E-31	0.43614	0.078	0.016	9.49E-27	Saliva_Mac	RASA4
RHOQ3	6.53E-31	0.899012	0.213	0.086	1.04E-26	Saliva_Mac	RHOQ
LTBP21	7.72E-31	0.490718	0.081	0.017	1.23E-26	Saliva_Mac	LTBP2
ETV5	8.45E-31	0.669202	0.125	0.036	1.35E-26	Saliva_Mac	ETV5
XIST7	1.43E-30	1.125068	0.267	0.122	2.29E-26	Saliva_Mac	XIST
ZFP36L11	2.04E-30	0.62623	0.652	0.468	3.25E-26	Saliva_Mac	ZFP36L1
CLEC2B3	2.65E-30	0.733732	0.319	0.155	4.23E-26	Saliva_Mac	CLEC2B
CMTM31	3E-30	0.665372	0.2	0.075	4.79E-26	Saliva_Mac	CMTM3
HTRA12	4.35E-30	0.969337	0.253	0.111	6.93E-26	Saliva_Mac	HTRA1
RPS6KA32	5.08E-30	0.911491	0.224	0.095	8.1E-26	Saliva_Mac	RPS6KA3
CTSL2	6.65E-30	0.898025	0.238	0.104	1.06E-25	Saliva_Mac	CTSL
LFNG2	6.85E-30	0.490362	0.106	0.028	1.09E-25	Saliva_Mac	LFNG
CD815	1.6E-29	0.721329	0.518	0.323	2.56E-25	Saliva_Mac	CD81
GAB31	2.21E-29	0.26811	0.049	0.007	3.53E-25	Saliva_Mac	GAB3
MSN3	4.07E-29	0.814681	0.31	0.156	6.49E-25	Saliva_Mac	MSN
IKZF12	4.08E-29	0.529948	0.132	0.039	6.51E-25	Saliva_Mac	IKZF1
DDX176	5.24E-29	0.861276	0.502	0.354	8.35E-25	Saliva_Mac	DDX17
TSPO3	5.6E-29	0.678422	0.601	0.466	8.94E-25	Saliva_Mac	TSPO
ZNF6411	6.04E-29	0.430482	0.079	0.017	9.64E-25	Saliva_Mac	ZNF641
PLD32	6.37E-29	1.146268	0.31	0.164	1.02E-24	Saliva_Mac	PLD3
CLECL11	8.38E-29	0.440936	0.067	0.013	1.34E-24	Saliva_Mac	CLECL1
TNFAIP21	1.08E-28	0.825272	0.289	0.14	1.73E-24	Saliva_Mac	TNFAIP2
BST22	3.88E-28	0.63629	0.354	0.181	6.2E-24	Saliva_Mac	BST2
DSE1	4.41E-28	0.637779	0.148	0.05	7.03E-24	Saliva_Mac	DSE
TBC1D9	6.73E-28	0.644833	0.135	0.043	1.07E-23	Saliva_Mac	TBC1D9
OGFRL13	7.45E-28	0.861868	0.245	0.114	1.19E-23	Saliva_Mac	OGFRL1
S100A111	1.48E-27	0.259385	0.866	0.666	2.36E-23	Saliva_Mac	S100A11
GPR1831	1.61E-27	0.471004	0.199	0.076	2.57E-23	Saliva_Mac	GPR183
ZFP36L23	3.14E-27	0.511116	0.59	0.392	5E-23	Saliva_Mac	ZFP36L2

GMFG1	3.54E-27	0.356769	0.413	0.209	5.65E-23	Saliva_Ma(	GMFG
C1orf162	3.8E-27	0.54143	0.224	0.091	6.05E-23	Saliva_Ma(	C1orf162
SFMBT21	4.43E-27	0.288659	0.056	0.01	7.06E-23	Saliva_Ma(	SFMBT2
RPL394	4.43E-27	0.356574	0.888	0.676	7.07E-23	Saliva_Ma(	RPL39
LYL11	7.24E-27	0.609716	0.186	0.071	1.15E-22	Saliva_Ma(	LYL1
MERTK	7.28E-27	0.407942	0.061	0.012	1.16E-22	Saliva_Ma(	MERTK
FCER2	8.88E-27	0.337911	0.052	0.009	1.42E-22	Saliva_Ma(	FCER2
HGF1	9.35E-27	0.33358	0.052	0.009	1.49E-22	Saliva_Ma(	HGF
TAGAP2	1.15E-26	0.409091	0.106	0.029	1.83E-22	Saliva_Ma(	TAGAP
CYSLTR12	1.6E-26	0.387285	0.076	0.017	2.55E-22	Saliva_Ma(	CYSLTR1
CD163L1	1.63E-26	0.486852	0.065	0.013	2.6E-22	Saliva_Ma(	CD163L1
PLA2G164	2.72E-26	0.800557	0.258	0.125	4.33E-22	Saliva_Ma(	PLA2G16
TGOLN26	4.05E-26	0.978157	0.309	0.171	6.47E-22	Saliva_Ma(	TGOLN2
RGS18	4.6E-26	0.282304	0.047	0.008	7.34E-22	Saliva_Ma(	RGS18
DYRK21	5.5E-26	0.63234	0.117	0.036	8.77E-22	Saliva_Ma(	DYRK2
SIGLEC9	1.06E-25	0.253343	0.034	0.004	1.7E-21	Saliva_Ma(	SIGLEC9
ARPC3	1.13E-25	0.626445	0.702	0.596	1.8E-21	Saliva_Ma(	ARPC3
MTPN2	1.14E-25	0.744374	0.289	0.149	1.82E-21	Saliva_Ma(	MTPN
IL13RA11	1.33E-25	0.900074	0.245	0.12	2.12E-21	Saliva_Ma(	IL13RA1
POLR2J36	1.85E-25	0.777328	0.217	0.097	2.95E-21	Saliva_Ma(	POLR2J3
HHEX1	2.19E-25	0.490875	0.106	0.031	3.49E-21	Saliva_Ma(	HHEX
FAM49B1	3.77E-25	0.891583	0.312	0.172	6.01E-21	Saliva_Ma(	FAM49B
SH3BGRL3	4.49E-25	0.517381	0.756	0.632	7.17E-21	Saliva_Ma(	SH3BGRL3
BTG24	4.68E-25	1.16655	0.381	0.232	7.46E-21	Saliva_Ma(	BTG2
UVRAG2	4.79E-25	0.597906	0.139	0.048	7.65E-21	Saliva_Ma(	UVRAG
SLC43A32	7.81E-25	0.512129	0.11	0.033	1.25E-20	Saliva_Ma(	SLC43A3
LRRFIP12	9.48E-25	0.757138	0.516	0.372	1.51E-20	Saliva_Ma(	LRRFIP1
GAA	1.14E-24	0.874555	0.186	0.079	1.81E-20	Saliva_Ma(	GAA
RGS13	1.89E-24	0.60788	0.179	0.069	3.01E-20	Saliva_Ma(	RGS1
ALDH23	1.95E-24	0.849582	0.247	0.123	3.1E-20	Saliva_Ma(	ALDH2
DPEP2	1.99E-24	0.445953	0.085	0.022	3.18E-20	Saliva_Ma(	DPEP2
PPP1R9B	2.03E-24	0.616373	0.103	0.03	3.23E-20	Saliva_Ma(	PPP1R9B
RNF135	3.54E-24	0.608355	0.112	0.035	5.65E-20	Saliva_Ma(	RNF135
MXD43	3.89E-24	0.828348	0.242	0.12	6.2E-20	Saliva_Ma(	MXD4
RTN1	5.8E-24	0.493485	0.083	0.021	9.25E-20	Saliva_Ma(	RTN1
RNH12	8.47E-24	0.837731	0.448	0.314	1.35E-19	Saliva_Ma(	RNH1
FOS4	1.16E-23	1.013444	0.569	0.425	1.84E-19	Saliva_Ma(	FOS
S100A103	1.37E-23	0.822728	0.782	0.676	2.19E-19	Saliva_Ma(	S100A10
FES1	2.6E-23	0.514992	0.114	0.036	4.15E-19	Saliva_Ma(	FES
CRTAP3	2.69E-23	0.80006	0.235	0.115	4.29E-19	Saliva_Ma(	CRTAP
SCAF115	2.82E-23	0.895261	0.363	0.231	4.49E-19	Saliva_Ma(	SCAF11
STX7	3.08E-23	0.837164	0.245	0.124	4.91E-19	Saliva_Ma(	STX7
FOSB6	3.23E-23	1.376389	0.384	0.251	5.15E-19	Saliva_Ma(	FOSB
FRMD4A2	3.51E-23	0.668956	0.139	0.051	5.6E-19	Saliva_Ma(	FRMD4A
YWHAH	3.71E-23	1.122975	0.336	0.207	5.91E-19	Saliva_Ma(	YWHAH
APPL13	3.91E-23	0.853949	0.244	0.123	6.24E-19	Saliva_Ma(	APPL1
MYADM3	4.56E-23	0.861526	0.282	0.15	7.27E-19	Saliva_Ma(	MYADM

SH3BP2	7.25E-23	0.754322	0.188	0.081	1.16E-18	Saliva_Ma(	SH3BP2
MAP3K14	8.43E-23	0.802959	0.164	0.067	1.34E-18	Saliva_Ma(	MAP3K1
HNMT1	9.22E-23	0.866672	0.222	0.108	1.47E-18	Saliva_Ma(	HNMT
DISC1	1.58E-22	0.332544	0.049	0.009	2.53E-18	Saliva_Ma(	DISC1
CLIC12	1.96E-22	0.591315	0.621	0.475	3.12E-18	Saliva_Ma(	CLIC1
HLA.E3	1.96E-22	0.444081	0.736	0.609	3.13E-18	Saliva_Ma(	HLA.E
RHOG	2.69E-22	0.718619	0.323	0.186	4.29E-18	Saliva_Ma(	RHOG
EIF2AK43	2.82E-22	0.800596	0.166	0.069	4.5E-18	Saliva_Ma(	EIF2AK4
WDFY21	3E-22	0.658535	0.134	0.049	4.78E-18	Saliva_Ma(	WDFY2
GSTO11	3.21E-22	0.81661	0.347	0.213	5.12E-18	Saliva_Ma(	GSTO1
YBX1	3.32E-22	0.26185	0.749	0.588	5.29E-18	Saliva_Ma(	YBX1
KCNMB11	9.08E-22	0.329214	0.067	0.016	1.45E-17	Saliva_Ma(	KCNMB1
LPAR5	1.28E-21	0.265211	0.051	0.01	2.04E-17	Saliva_Ma(	LPAR5
RAC11	1.28E-21	0.562986	0.659	0.548	2.05E-17	Saliva_Ma(	RAC1
FKBP15	1.52E-21	0.554717	0.11	0.036	2.43E-17	Saliva_Ma(	FKBP15
PIK3R5	2.08E-21	0.309464	0.054	0.011	3.32E-17	Saliva_Ma(	PIK3R5
RGS19	2.55E-21	0.448086	0.166	0.066	4.06E-17	Saliva_Ma(	RGS19
MYO9B1	2.76E-21	0.726338	0.164	0.069	4.4E-17	Saliva_Ma(	MYO9B
ACTB	3.97E-21	0.276825	0.977	0.872	6.33E-17	Saliva_Ma(	ACTB
TRPS12	5.31E-21	0.863618	0.19	0.087	8.46E-17	Saliva_Ma(	TRPS1
GCNT11	7.07E-21	0.436237	0.076	0.02	1.13E-16	Saliva_Ma(	GCNT1
HMHA1	8.05E-21	0.460734	0.168	0.067	1.28E-16	Saliva_Ma(	HMHA1
SGK1	9.32E-21	0.848612	0.226	0.112	1.49E-16	Saliva_Ma(	SGK1
M6PR1	1.12E-20	0.808549	0.242	0.128	1.78E-16	Saliva_Ma(	M6PR
OSM	1.65E-20	0.265879	0.038	0.006	2.63E-16	Saliva_Ma(	OSM
VEGFB5	1.77E-20	0.739661	0.206	0.099	2.82E-16	Saliva_Ma(	VEGFB
CCDC170	2.86E-20	0.378624	0.061	0.015	4.55E-16	Saliva_Ma(	CCDC170
CREG11	3.29E-20	1.026343	0.256	0.145	5.24E-16	Saliva_Ma(	CREG1
PLCL2	4.83E-20	0.306171	0.052	0.011	7.7E-16	Saliva_Ma(	PLCL2
CTTNBP2	4.9E-20	0.338085	0.054	0.012	7.82E-16	Saliva_Ma(	CTTNBP2
ST6GAL13	7.54E-20	0.495603	0.112	0.039	1.2E-15	Saliva_Ma(	ST6GAL1
OSBPL11	7.81E-20	0.570101	0.112	0.039	1.25E-15	Saliva_Ma(	OSBPL11
NR4A21	8.81E-20	1.375513	0.303	0.185	1.4E-15	Saliva_Ma(	NR4A2
SLC29A3	1.77E-19	0.318837	0.051	0.011	2.82E-15	Saliva_Ma(	SLC29A3
DENND4B	2.24E-19	0.383539	0.076	0.021	3.57E-15	Saliva_Ma(	DENND4B
RTN42	2.27E-19	0.704301	0.514	0.394	3.62E-15	Saliva_Ma(	RTN4
N4BP2L26	2.77E-19	0.771315	0.41	0.293	4.42E-15	Saliva_Ma(	N4BP2L2
SLC7A81	3E-19	0.485164	0.081	0.024	4.78E-15	Saliva_Ma(	SLC7A8
LMO21	4.47E-19	0.619506	0.191	0.09	7.13E-15	Saliva_Ma(	LMO2
FUOM1	4.64E-19	0.679886	0.171	0.078	7.4E-15	Saliva_Ma(	FUOM
EEF1B23	5.15E-19	0.341156	0.742	0.597	8.21E-15	Saliva_Ma(	EEF1B2
EPSTI11	5.32E-19	0.567227	0.123	0.046	8.49E-15	Saliva_Ma(	EPSTI1
WDFY4	5.59E-19	0.412326	0.056	0.013	8.91E-15	Saliva_Ma(	WDFY4
DEK3	7.05E-19	0.799199	0.375	0.259	1.12E-14	Saliva_Ma(	DEK
NCOA41	7.09E-19	0.893047	0.276	0.166	1.13E-14	Saliva_Ma(	NCOA4
DNASE1L1	7.42E-19	0.449006	0.085	0.026	1.18E-14	Saliva_Ma(	DNASE1L1
NPL	7.75E-19	0.468749	0.094	0.03	1.24E-14	Saliva_Ma(	NPL

PIK3CG	8.34E-19	0.265574	0.051	0.011	1.33E-14	Saliva_Ma	PIK3CG
C20orf194	8.51E-19	0.559226	0.11	0.039	1.36E-14	Saliva_Ma	C20orf194
NRIP11	9.94E-19	0.626999	0.128	0.05	1.59E-14	Saliva_Ma	NRIP1
PTMS4	1.1E-18	0.709207	0.374	0.244	1.76E-14	Saliva_Ma	PTMS
ERAP23	1.18E-18	0.414622	0.09	0.029	1.89E-14	Saliva_Ma	ERAP2
PTPRJ	1.31E-18	0.594914	0.101	0.035	2.08E-14	Saliva_Ma	PTPRJ
CHD96	1.46E-18	0.83794	0.28	0.167	2.33E-14	Saliva_Ma	CHD9
AP2A2	1.57E-18	0.879831	0.179	0.085	2.51E-14	Saliva_Ma	AP2A2
PAQR8	2.23E-18	0.310991	0.056	0.013	3.56E-14	Saliva_Ma	PAQR8
C2	2.41E-18	0.605125	0.083	0.026	3.84E-14	Saliva_Ma	C2
MKNK1	2.66E-18	0.649869	0.134	0.054	4.24E-14	Saliva_Ma	MKNK1
RB1	2.78E-18	0.777965	0.186	0.09	4.43E-14	Saliva_Ma	RB1
LYN	3.08E-18	0.744233	0.229	0.122	4.91E-14	Saliva_Ma	LYN
MTDH4	4.28E-18	0.695482	0.424	0.309	6.83E-14	Saliva_Ma	MTDH
WASF22	4.57E-18	0.876366	0.422	0.323	7.29E-14	Saliva_Ma	WASF2
CD200R1	4.9E-18	0.396304	0.072	0.02	7.81E-14	Saliva_Ma	CD200R1
ANKH1	5.18E-18	0.526104	0.103	0.036	8.26E-14	Saliva_Ma	ANKH
ENHO1	5.44E-18	0.307251	0.058	0.014	8.68E-14	Saliva_Ma	ENHO
SRGAP2	5.72E-18	0.577954	0.126	0.05	9.13E-14	Saliva_Ma	SRGAP2
CYFIP1	5.84E-18	0.778041	0.215	0.115	9.31E-14	Saliva_Ma	CYFIP1
GNS1	7.22E-18	0.780953	0.177	0.085	1.15E-13	Saliva_Ma	GNS
FEZ21	8.99E-18	0.785723	0.209	0.109	1.43E-13	Saliva_Ma	FEZ2
RIN2	9.9E-18	0.790141	0.161	0.073	1.58E-13	Saliva_Ma	RIN2
PIK3AP1	1.08E-17	0.638763	0.114	0.043	1.73E-13	Saliva_Ma	PIK3AP1
SULF21	1.16E-17	0.366382	0.096	0.032	1.85E-13	Saliva_Ma	SULF2
TRPV2	1.26E-17	0.306062	0.078	0.023	2.01E-13	Saliva_Ma	TRPV2
ARHGAP22	1.39E-17	0.398955	0.067	0.018	2.22E-13	Saliva_Ma	ARHGAP22
PMP223	1.47E-17	0.867228	0.251	0.138	2.35E-13	Saliva_Ma	PMP22
ADAP1	1.55E-17	0.357417	0.07	0.02	2.47E-13	Saliva_Ma	ADAP1
TNFSF123	1.72E-17	0.497259	0.143	0.06	2.75E-13	Saliva_Ma	TNFSF12
ASAP11	2.12E-17	0.661827	0.191	0.095	3.37E-13	Saliva_Ma	ASAP1
MCTP11	2.3E-17	0.482228	0.099	0.034	3.67E-13	Saliva_Ma	MCTP1
WAS1	2.45E-17	0.340774	0.119	0.044	3.91E-13	Saliva_Ma	WAS
EPB41L32	2.76E-17	0.619557	0.137	0.058	4.4E-13	Saliva_Ma	EPB41L3
ARHGAP31	2.8E-17	0.313599	0.06	0.015	4.46E-13	Saliva_Ma	ARHGAP31
EPB41L22	3.44E-17	0.637431	0.188	0.092	5.48E-13	Saliva_Ma	EPB41L2
ARL6IP53	4.1E-17	0.642849	0.431	0.31	6.54E-13	Saliva_Ma	ARL6IP5
MOB1A1	4.21E-17	0.739877	0.294	0.186	6.71E-13	Saliva_Ma	MOB1A
TRIM382	4.84E-17	0.57247	0.155	0.07	7.71E-13	Saliva_Ma	TRIM38
DDX54	5.8E-17	0.426434	0.688	0.602	9.26E-13	Saliva_Ma	DDX5
PLXDC11	5.89E-17	0.365298	0.065	0.018	9.39E-13	Saliva_Ma	PLXDC1
MT.ND67	7.73E-17	0.680067	0.211	0.111	1.23E-12	Saliva_Ma	MT.ND6
RPS6KA4	8.15E-17	0.38773	0.081	0.026	1.3E-12	Saliva_Ma	RPS6KA4
CD300A	8.57E-17	0.311054	0.072	0.021	1.37E-12	Saliva_Ma	CD300A
ANTXR23	8.96E-17	0.489869	0.096	0.033	1.43E-12	Saliva_Ma	ANTXR2
IL17RA	9.19E-17	0.417594	0.097	0.034	1.47E-12	Saliva_Ma	IL17RA
DOK1	9.87E-17	0.355912	0.07	0.021	1.57E-12	Saliva_Ma	DOK1

NFATC21	1.07E-16	0.532405	0.116	0.045	1.71E-12	Saliva_Ma	NFATC2
ENG1	1.27E-16	0.357431	0.139	0.058	2.03E-12	Saliva_Ma	ENG
TRIM14	1.29E-16	0.513668	0.097	0.035	2.05E-12	Saliva_Ma	TRIM14
CD931	1.51E-16	0.451212	0.094	0.032	2.4E-12	Saliva_Ma	CD93
GNPDA1	1.66E-16	0.66646	0.119	0.048	2.65E-12	Saliva_Ma	GNPDA1
UNC5B	2.34E-16	0.262609	0.042	0.009	3.73E-12	Saliva_Ma	UNC5B
ZFYVE161	2.51E-16	0.647628	0.157	0.073	4.01E-12	Saliva_Ma	ZFYVE16
FILIP1L3	2.59E-16	0.607007	0.177	0.084	4.13E-12	Saliva_Ma	FILIP1L
ELMO11	2.98E-16	0.403042	0.094	0.033	4.75E-12	Saliva_Ma	ELMO1
PSTPIP11	3.08E-16	0.291598	0.078	0.024	4.91E-12	Saliva_Ma	PSTPIP1
AKR1B11	3.4E-16	0.631403	0.211	0.111	5.43E-12	Saliva_Ma	AKR1B1
RHOA2	3.86E-16	0.595757	0.556	0.474	6.15E-12	Saliva_Ma	RHOA
COCH	4.02E-16	0.35678	0.065	0.019	6.41E-12	Saliva_Ma	COCH
PILRA	4.06E-16	0.323102	0.07	0.021	6.47E-12	Saliva_Ma	PILRA
ZNF385A1	4.62E-16	0.442473	0.101	0.037	7.37E-12	Saliva_Ma	ZNF385A
SLC25A24	4.73E-16	0.517769	0.112	0.044	7.54E-12	Saliva_Ma	SLC25A24
CBL	4.86E-16	0.477803	0.116	0.046	7.75E-12	Saliva_Ma	CBL
DBI3	6.24E-16	0.501141	0.551	0.414	9.94E-12	Saliva_Ma	DBI
ZYX2	6.48E-16	0.645465	0.215	0.116	1.03E-11	Saliva_Ma	ZYX
SETX3	8.43E-16	0.702651	0.193	0.102	1.34E-11	Saliva_Ma	SETX
WWP1	9.15E-16	0.943302	0.164	0.081	1.46E-11	Saliva_Ma	WWP1
RUNX31	9.36E-16	0.330897	0.094	0.033	1.49E-11	Saliva_Ma	RUNX3
RAP2A	1.04E-15	0.503725	0.114	0.045	1.66E-11	Saliva_Ma	RAP2A
C31	1.07E-15	1.124892	0.171	0.084	1.7E-11	Saliva_Ma	C3
CAP1	1.1E-15	0.640826	0.504	0.412	1.75E-11	Saliva_Ma	CAP1
GAS2L3	1.13E-15	0.256417	0.036	0.007	1.8E-11	Saliva_Ma	GAS2L3
GSAP	1.16E-15	0.448574	0.074	0.023	1.85E-11	Saliva_Ma	GSAP
LHFPL22	1.58E-15	0.663688	0.125	0.053	2.52E-11	Saliva_Ma	LHFPL2
CPED12	1.62E-15	0.328248	0.069	0.021	2.58E-11	Saliva_Ma	CPED1
RPL226	1.84E-15	0.367404	0.809	0.694	2.94E-11	Saliva_Ma	RPL22
ACAP22	2.14E-15	0.706698	0.242	0.143	3.41E-11	Saliva_Ma	ACAP2
TACC13	3.89E-15	0.758756	0.269	0.166	6.21E-11	Saliva_Ma	TACC1
FLNA4	4.19E-15	0.622632	0.258	0.149	6.69E-11	Saliva_Ma	FLNA
RCSD11	4.44E-15	0.349528	0.128	0.053	7.08E-11	Saliva_Ma	RCSD1
JAK2	5.74E-15	0.380097	0.074	0.024	9.15E-11	Saliva_Ma	JAK2
MOB1B	5.77E-15	0.486077	0.092	0.034	9.21E-11	Saliva_Ma	MOB1B
RPS233	6.13E-15	0.262896	0.968	0.813	9.78E-11	Saliva_Ma	RPS23
MT.ATP86	6.5E-15	0.676283	0.233	0.135	1.04E-10	Saliva_Ma	MT.ATP8
SNCA2	7.2E-15	0.463788	0.112	0.046	1.15E-10	Saliva_Ma	SNCA
AKAP132	8.92E-15	0.852681	0.314	0.217	1.42E-10	Saliva_Ma	AKAP13
CASP1	9.37E-15	0.434592	0.13	0.056	1.49E-10	Saliva_Ma	CASP1
EVI2A2	9.43E-15	0.404564	0.099	0.037	1.5E-10	Saliva_Ma	EVI2A
EVI53	1.43E-14	0.795052	0.157	0.078	2.28E-10	Saliva_Ma	EVI5
ARSB	1.47E-14	0.279647	0.047	0.012	2.34E-10	Saliva_Ma	ARSB
CCDC141	1.54E-14	0.273019	0.032	0.006	2.46E-10	Saliva_Ma	CCDC141
EGR14	1.81E-14	1.276279	0.271	0.173	2.89E-10	Saliva_Ma	EGR1
TNS13	2.14E-14	0.592459	0.132	0.059	3.42E-10	Saliva_Ma	TNS1

RPL305	3.15E-14	0.263075	0.944	0.844	5.03E-10	Saliva_Ma	RPL30
TNFAIP82	3.2E-14	0.482819	0.199	0.104	5.1E-10	Saliva_Ma	TNFAIP8
BHLHE412	3.85E-14	0.714757	0.108	0.045	6.13E-10	Saliva_Ma	BHLHE41
WDR81	3.89E-14	0.286326	0.051	0.013	6.2E-10	Saliva_Ma	WDR81
CCL81	4.2E-14	0.294	0.045	0.011	6.7E-10	Saliva_Ma	CCL8
STK42	4.66E-14	0.492894	0.209	0.113	7.44E-10	Saliva_Ma	STK4
CALHM22	5.62E-14	0.303088	0.092	0.034	8.96E-10	Saliva_Ma	CALHM2
C10orf128	6.51E-14	0.324286	0.067	0.021	1.04E-09	Saliva_Ma	C10orf128
DST3	6.88E-14	0.506856	0.287	0.184	1.1E-09	Saliva_Ma	DST
SGPP1	7.53E-14	0.391425	0.07	0.023	1.2E-09	Saliva_Ma	SGPP1
GPATCH2L	7.67E-14	0.614098	0.161	0.082	1.22E-09	Saliva_Ma	GPATCH2L
ORAI11	8.01E-14	0.603393	0.171	0.089	1.28E-09	Saliva_Ma	ORAI1
AKR1A13	8.96E-14	0.735901	0.298	0.204	1.43E-09	Saliva_Ma	AKR1A1
ST8SIA42	1.09E-13	0.410905	0.079	0.028	1.74E-09	Saliva_Ma	ST8SIA4
PARVG1	1.13E-13	0.318622	0.085	0.03	1.8E-09	Saliva_Ma	PARVG
PRKCB1	1.16E-13	0.314474	0.076	0.026	1.85E-09	Saliva_Ma	PRKCB
CXCL16	1.28E-13	0.292746	0.244	0.142	2.05E-09	Saliva_Ma	CXCL16
MGST36	1.49E-13	0.513391	0.451	0.335	2.38E-09	Saliva_Ma	MGST3
TBC1D2B1	2.14E-13	0.350255	0.087	0.032	3.41E-09	Saliva_Ma	TBC1D2B
SLC1A3	2.17E-13	0.361452	0.056	0.016	3.46E-09	Saliva_Ma	SLC1A3
LPAR61	2.54E-13	0.755272	0.233	0.141	4.06E-09	Saliva_Ma	LPAR6
PPM1F1	2.94E-13	0.395461	0.083	0.031	4.69E-09	Saliva_Ma	PPM1F
RBM12B1	3.13E-13	0.462835	0.085	0.032	4.99E-09	Saliva_Ma	RBM12B
TWF2	3.32E-13	0.60512	0.213	0.123	5.29E-09	Saliva_Ma	TWF2
LDLRAD4	3.65E-13	0.50129	0.121	0.054	5.82E-09	Saliva_Ma	LDLRAD4
SEC11A3	3.74E-13	0.661737	0.403	0.315	5.97E-09	Saliva_Ma	SEC11A
C10orf541	3.91E-13	0.540583	0.274	0.171	6.23E-09	Saliva_Ma	C10orf54
SYNE35	4.25E-13	0.262097	0.06	0.018	6.78E-09	Saliva_Ma	SYNE3
PLCG22	4.52E-13	0.382123	0.079	0.029	7.22E-09	Saliva_Ma	PLCG2
CAPG1	4.55E-13	0.78486	0.265	0.167	7.26E-09	Saliva_Ma	CAPG
INPP5D1	5.12E-13	0.565107	0.128	0.06	8.16E-09	Saliva_Ma	INPP5D
ST3GAL6	5.18E-13	0.450753	0.083	0.031	8.26E-09	Saliva_Ma	ST3GAL6
BOD1L15	5.52E-13	0.660295	0.215	0.128	8.8E-09	Saliva_Ma	BOD1L1
TTYH2	6.58E-13	0.308707	0.047	0.013	1.05E-08	Saliva_Ma	TTYH2
ITSN1	6.68E-13	0.665036	0.11	0.048	1.06E-08	Saliva_Ma	ITSN1
ADRBK1	6.81E-13	0.568748	0.162	0.084	1.09E-08	Saliva_Ma	ADRBK1
PDGFB1	7.3E-13	0.364631	0.061	0.02	1.16E-08	Saliva_Ma	PDGFB
PRPF40A3	1.02E-12	0.787306	0.274	0.188	1.62E-08	Saliva_Ma	PRPF40A
EIF54	1.22E-12	0.747084	0.435	0.36	1.95E-08	Saliva_Ma	EIF5
RENBP	1.27E-12	0.343679	0.063	0.021	2.03E-08	Saliva_Ma	RENBP
TPCN11	1.29E-12	0.553386	0.117	0.053	2.06E-08	Saliva_Ma	TPCN1
HMGB13	1.3E-12	0.40133	0.646	0.575	2.07E-08	Saliva_Ma	HMGB1
LACC1	1.68E-12	0.356392	0.061	0.02	2.67E-08	Saliva_Ma	LACC1
PAG11	1.7E-12	0.527979	0.11	0.048	2.7E-08	Saliva_Ma	PAG1
GAL3ST4	1.9E-12	0.355385	0.051	0.015	3.02E-08	Saliva_Ma	GAL3ST4
KCNK63	2E-12	0.721031	0.179	0.101	3.19E-08	Saliva_Ma	KCNK6
CELF1	2.01E-12	0.71975	0.208	0.125	3.21E-08	Saliva_Ma	CELF1

CDV32	2.31E-12	0.70832	0.264	0.174	3.68E-08	Saliva_Ma	CDV3
SRFBP13	3.1E-12	0.530071	0.128	0.062	4.94E-08	Saliva_Ma	SRFBP1
ITPRIPL2	3.48E-12	0.544652	0.188	0.105	5.56E-08	Saliva_Ma	ITPRIPL2
HDAC9	4.12E-12	0.486466	0.097	0.041	6.57E-08	Saliva_Ma	HDAC9
PDGFC	4.27E-12	0.602132	0.106	0.048	6.81E-08	Saliva_Ma	PDGFC
MIS18BP1	5.34E-12	0.563891	0.181	0.102	8.52E-08	Saliva_Ma	MIS18BP1
UAP1L1	5.77E-12	0.286234	0.043	0.012	9.2E-08	Saliva_Ma	UAP1L1
C6orf62	7.86E-12	0.802589	0.296	0.215	1.25E-07	Saliva_Ma	C6orf62
USF22	7.95E-12	0.601078	0.199	0.117	1.27E-07	Saliva_Ma	USF2
KLF24	9.03E-12	0.694262	0.291	0.191	1.44E-07	Saliva_Ma	KLF2
SGK3	9.38E-12	0.390592	0.063	0.022	1.5E-07	Saliva_Ma	SGK3
KMT2C4	1.28E-11	0.723725	0.235	0.154	2.03E-07	Saliva_Ma	KMT2C
AXL2	1.39E-11	0.366468	0.182	0.098	2.22E-07	Saliva_Ma	AXL
GDPD1	1.41E-11	0.344158	0.052	0.016	2.25E-07	Saliva_Ma	GDPD1
EFHD2	1.73E-11	0.562551	0.31	0.22	2.77E-07	Saliva_Ma	EFHD2
CREB51	1.8E-11	0.508609	0.119	0.056	2.88E-07	Saliva_Ma	CREB5
FOXN2	1.82E-11	0.407634	0.088	0.037	2.91E-07	Saliva_Ma	FOXN2
TCHH	1.84E-11	0.269697	0.042	0.011	2.93E-07	Saliva_Ma	TCHH
PABPC41	1.84E-11	0.652419	0.209	0.128	2.94E-07	Saliva_Ma	PABPC4
CNRIP13	1.86E-11	0.395328	0.092	0.038	2.96E-07	Saliva_Ma	CNRIP1
KLF62	1.99E-11	0.615654	0.489	0.402	3.18E-07	Saliva_Ma	KLF6
BASP11	2.1E-11	0.424923	0.159	0.084	3.34E-07	Saliva_Ma	BASP1
VASP1	2.23E-11	0.562758	0.213	0.129	3.56E-07	Saliva_Ma	VASP
TTL	2.25E-11	0.421076	0.069	0.025	3.6E-07	Saliva_Ma	TTL
ATG16L2	2.47E-11	0.333222	0.079	0.031	3.94E-07	Saliva_Ma	ATG16L2
TUBA1A3	2.67E-11	0.540381	0.338	0.235	4.25E-07	Saliva_Ma	TUBA1A
NAV2	3.05E-11	0.402148	0.054	0.018	4.86E-07	Saliva_Ma	NAV2
CHML	3.26E-11	0.347803	0.065	0.023	5.2E-07	Saliva_Ma	CHML
SNX291	3.27E-11	0.568668	0.126	0.063	5.22E-07	Saliva_Ma	SNX29
CSF2RB2	3.48E-11	0.421275	0.09	0.038	5.55E-07	Saliva_Ma	CSF2RB
SKAP2	3.63E-11	0.668614	0.188	0.112	5.79E-07	Saliva_Ma	SKAP2
SLCO3A12	3.76E-11	0.511715	0.114	0.054	6E-07	Saliva_Ma	SLCO3A1
DENND4C	3.88E-11	0.638916	0.135	0.07	6.19E-07	Saliva_Ma	DENND4C
HSP90AA1	3.92E-11	0.263666	0.767	0.713	6.25E-07	Saliva_Ma	HSP90AA1
TAOK11	4.38E-11	0.712989	0.186	0.111	6.98E-07	Saliva_Ma	TAOK1
C19orf603	6.25E-11	0.538776	0.319	0.229	9.96E-07	Saliva_Ma	C19orf60
TRIM445	6.59E-11	0.573654	0.161	0.09	1.05E-06	Saliva_Ma	TRIM44
ARHGEF40	6.71E-11	0.44534	0.088	0.038	1.07E-06	Saliva_Ma	ARHGEF40
PLEKHG5	8.3E-11	0.266606	0.034	0.009	1.32E-06	Saliva_Ma	PLEKHG5
TPPP	8.72E-11	0.28308	0.043	0.013	1.39E-06	Saliva_Ma	TPPP
MDM43	1.08E-10	0.664896	0.215	0.137	1.73E-06	Saliva_Ma	MDM4
SH3KBP1	1.12E-10	0.541188	0.161	0.088	1.78E-06	Saliva_Ma	SH3KBP1
ITGA42	1.18E-10	0.252165	0.074	0.028	1.88E-06	Saliva_Ma	ITGA4
MCOLN1	1.22E-10	0.4868	0.105	0.049	1.94E-06	Saliva_Ma	MCOLN1
WLS1	1.33E-10	0.502461	0.096	0.043	2.12E-06	Saliva_Ma	WLS
TAPBP4	1.35E-10	0.585132	0.247	0.164	2.15E-06	Saliva_Ma	TAPBP
TCIRG11	1.46E-10	0.543123	0.139	0.073	2.33E-06	Saliva_Ma	TCIRG1

HIVEP3	1.49E-10	0.33409	0.069	0.026	2.37E-06	Saliva_Ma(	HIVEP3
BCL2A1	1.5E-10	0.316409	0.085	0.035	2.39E-06	Saliva_Ma(	BCL2A1
VAV1	1.75E-10	0.376322	0.087	0.037	2.8E-06	Saliva_Ma(	VAV1
PPP1R103	2.39E-10	0.657882	0.231	0.152	3.82E-06	Saliva_Ma(	PPP1R10
ASAH13	2.65E-10	0.848605	0.345	0.278	4.22E-06	Saliva_Ma(	ASAH1
CCL21	2.7E-10	0.254272	0.208	0.124	4.31E-06	Saliva_Ma(	CCL2
RNF2132	3.03E-10	0.646739	0.226	0.147	4.83E-06	Saliva_Ma(	RNF213
ROCK13	3.31E-10	0.582388	0.236	0.156	5.27E-06	Saliva_Ma(	ROCK1
PEAK12	3.57E-10	0.495373	0.09	0.04	5.69E-06	Saliva_Ma(	PEAK1
PQLC32	3.86E-10	0.554878	0.161	0.092	6.16E-06	Saliva_Ma(	PQLC3
FNBP12	4.82E-10	0.498517	0.206	0.126	7.68E-06	Saliva_Ma(	FNBP1
FHOD1	4.86E-10	0.328384	0.06	0.022	7.74E-06	Saliva_Ma(	FHOD1
ATRX4	4.96E-10	0.690315	0.283	0.207	7.91E-06	Saliva_Ma(	ATRX
ZBTB37	5.03E-10	0.403621	0.07	0.028	8.02E-06	Saliva_Ma(	ZBTB37
GIMAP61	5.05E-10	0.336711	0.076	0.03	8.05E-06	Saliva_Ma(	GIMAP6
HSPA81	6.31E-10	0.410698	0.572	0.51	1.01E-05	Saliva_Ma(	HSPA8
DPYSL32	6.75E-10	0.572934	0.112	0.055	1.08E-05	Saliva_Ma(	DPYSL3
CMTM6	7.71E-10	0.569335	0.305	0.227	1.23E-05	Saliva_Ma(	CMTM6
MTHFR	7.9E-10	0.464462	0.088	0.04	1.26E-05	Saliva_Ma(	MTHFR
SAMD11	8.12E-10	0.391117	0.079	0.034	1.29E-05	Saliva_Ma(	SAMD1
TET2	8.3E-10	0.478362	0.114	0.057	1.32E-05	Saliva_Ma(	TET2
RNASEK	8.46E-10	0.340923	0.079	0.033	1.35E-05	Saliva_Ma(	RNASEK
CLTA1	9.04E-10	0.585734	0.439	0.386	1.44E-05	Saliva_Ma(	CLTA
PGLS1	9.68E-10	0.657514	0.33	0.263	1.54E-05	Saliva_Ma(	PGLS
PLEKHA2	1.18E-09	0.440025	0.105	0.051	1.88E-05	Saliva_Ma(	PLEKHA2
CXCL10	1.41E-09	0.67745	0.018	0.003	2.25E-05	Saliva_Ma(	CXCL10
IFI44L1	1.62E-09	0.408412	0.078	0.033	2.58E-05	Saliva_Ma(	IFI44L
NR3C13	1.83E-09	0.58097	0.224	0.149	2.92E-05	Saliva_Ma(	NR3C1
PARP42	2.04E-09	0.390652	0.09	0.041	3.26E-05	Saliva_Ma(	PARP4
MAT2A2	2.1E-09	0.677563	0.222	0.15	3.34E-05	Saliva_Ma(	MAT2A
SDC3	2.11E-09	0.32364	0.061	0.024	3.36E-05	Saliva_Ma(	SDC3
RNPEPL11	2.12E-09	0.561527	0.179	0.11	3.39E-05	Saliva_Ma(	RNPEPL1
GYPC2	2.17E-09	0.277214	0.307	0.203	3.46E-05	Saliva_Ma(	GYPC
GMIP	2.22E-09	0.263377	0.06	0.022	3.54E-05	Saliva_Ma(	GMIP
PLAUR	2.86E-09	0.427315	0.195	0.119	4.55E-05	Saliva_Ma(	PLAUR
RECQL2	2.95E-09	0.443474	0.112	0.056	4.7E-05	Saliva_Ma(	RECQL
PAK22	3E-09	0.629444	0.253	0.181	4.78E-05	Saliva_Ma(	PAK2
LIMS12	3.17E-09	0.609342	0.242	0.168	5.05E-05	Saliva_Ma(	LIMS1
CHCHD105	3.29E-09	0.549731	0.321	0.245	5.25E-05	Saliva_Ma(	CHCHD10
TLR5	3.31E-09	0.335323	0.056	0.021	5.28E-05	Saliva_Ma(	TLR5
DPP73	3.78E-09	0.573949	0.332	0.259	6.02E-05	Saliva_Ma(	DPP7
C10orf111	4.27E-09	0.530509	0.103	0.051	6.81E-05	Saliva_Ma(	C10orf11
VOPP11	4.33E-09	0.57297	0.182	0.115	6.91E-05	Saliva_Ma(	VOPP1
SLC25A35	4.57E-09	0.462807	0.502	0.456	7.28E-05	Saliva_Ma(	SLC25A3
EHBP1L12	4.69E-09	0.366211	0.108	0.054	7.48E-05	Saliva_Ma(	EHBP1L1
EGR21	4.74E-09	0.434948	0.078	0.034	7.55E-05	Saliva_Ma(	EGR2
ATG4C	5.04E-09	0.371788	0.074	0.032	8.04E-05	Saliva_Ma(	ATG4C

AP2S1	5.08E-09	0.659323	0.39	0.342	8.09E-05	Saliva_Ma(	AP2S1
KANK22	5.16E-09	0.44737	0.092	0.043	8.23E-05	Saliva_Ma(	KANK2
PHKB4	5.46E-09	0.638267	0.17	0.106	8.71E-05	Saliva_Ma(	PHKB
DNAJC131	5.72E-09	0.388079	0.081	0.037	9.12E-05	Saliva_Ma(	DNAJC13
SNX30	6.14E-09	0.308142	0.063	0.025	9.79E-05	Saliva_Ma(	SNX30
QPRT1	6.4E-09	0.340637	0.061	0.024	0.000102	Saliva_Ma(	QPRT
IL10	8.23E-09	0.275806	0.031	0.008	0.000131	Saliva_Ma(	IL10
PTGS2	8.27E-09	0.419342	0.087	0.04	0.000132	Saliva_Ma(	PTGS2
GNAQ2	8.37E-09	0.716446	0.231	0.164	0.000133	Saliva_Ma(	GNAQ
CNTRL	9.01E-09	0.362142	0.11	0.056	0.000144	Saliva_Ma(	CNTRL
CSK	1.2E-08	0.355519	0.137	0.075	0.000192	Saliva_Ma(	CSK
C20orf271	1.25E-08	0.359356	0.128	0.07	0.000199	Saliva_Ma(	C20orf27
ANXA41	1.82E-08	0.713852	0.204	0.138	0.00029	Saliva_Ma(	ANXA4
LAMTOR2	2.02E-08	0.579922	0.298	0.233	0.000323	Saliva_Ma(	LAMTOR2
LGALS9	2.2E-08	0.44817	0.181	0.113	0.000351	Saliva_Ma(	LGALS9
HOMER32	2.21E-08	0.526998	0.108	0.057	0.000352	Saliva_Ma(	HOMER3
FCGR1A	2.25E-08	0.273377	0.051	0.019	0.000358	Saliva_Ma(	FCGR1A
ZFP142	2.43E-08	0.339541	0.072	0.032	0.000388	Saliva_Ma(	ZFP14
PRKACB2	2.48E-08	0.670011	0.161	0.101	0.000395	Saliva_Ma(	PRKACB
HGSNAT2	2.51E-08	0.46181	0.126	0.07	0.0004	Saliva_Ma(	HGSNAT
APOE1	2.52E-08	0.627292	0.24	0.166	0.000402	Saliva_Ma(	APOE
ARAP11	2.57E-08	0.32819	0.085	0.04	0.000411	Saliva_Ma(	ARAP1
TCN22	2.6E-08	0.389847	0.078	0.035	0.000414	Saliva_Ma(	TCN2
NIN1	2.63E-08	0.443898	0.108	0.057	0.000419	Saliva_Ma(	NIN
NDUFB22	2.85E-08	0.480987	0.442	0.375	0.000455	Saliva_Ma(	NDUFB2
ADPGK	2.98E-08	0.441514	0.121	0.067	0.000476	Saliva_Ma(	ADPGK
MAML1	3.34E-08	0.417004	0.09	0.044	0.000532	Saliva_Ma(	MAML1
EFR3A2	3.35E-08	0.56081	0.141	0.084	0.000534	Saliva_Ma(	EFR3A
GRB21	3.63E-08	0.604513	0.238	0.172	0.000579	Saliva_Ma(	GRB2
PBX3	3.74E-08	0.315116	0.07	0.031	0.000596	Saliva_Ma(	PBX3
GNG21	3.83E-08	0.371617	0.128	0.07	0.000612	Saliva_Ma(	GNG2
LPXN1	3.86E-08	0.290973	0.096	0.047	0.000616	Saliva_Ma(	LPXN
DNM11	4.03E-08	0.350665	0.049	0.018	0.000642	Saliva_Ma(	DNM1
HSPA1A3	4.42E-08	0.554219	0.401	0.326	0.000705	Saliva_Ma(	HSPA1A
HERPUD12	4.52E-08	0.354963	0.395	0.333	0.00072	Saliva_Ma(	HERPUD1
DENND1A	4.61E-08	0.278692	0.056	0.022	0.000735	Saliva_Ma(	DENND1A
ATG7	4.63E-08	0.459769	0.083	0.04	0.000738	Saliva_Ma(	ATG7
ARRB11	4.95E-08	0.375406	0.078	0.036	0.00079	Saliva_Ma(	ARRB1
SH3TC1	5.28E-08	0.314141	0.061	0.026	0.000843	Saliva_Ma(	SH3TC1
FAM212B	5.28E-08	0.289808	0.051	0.019	0.000843	Saliva_Ma(	FAM212B
TNFRSF1A2	5.3E-08	0.63957	0.226	0.16	0.000845	Saliva_Ma(	TNFRSF1A
MARVELD1	5.5E-08	0.345639	0.072	0.032	0.000877	Saliva_Ma(	MARVELD1
SIPA1	5.52E-08	0.516019	0.148	0.089	0.000881	Saliva_Ma(	SIPA1
TRAK12	5.74E-08	0.53013	0.117	0.066	0.000915	Saliva_Ma(	TRAK1
DICER13	5.76E-08	0.645193	0.162	0.104	0.000919	Saliva_Ma(	DICER1
DENND4A1	6.26E-08	0.352591	0.085	0.041	0.000998	Saliva_Ma(	DENND4A
XRN22	6.27E-08	0.60246	0.215	0.151	0.001	Saliva_Ma(	XRN2

TBC1D13	6.75E-08	0.447935	0.108	0.058	0.001076	Saliva_Mac	TBC1D1
METTL7A2	6.8E-08	0.574767	0.148	0.091	0.001085	Saliva_Mac	METTL7A
TMEM55A	9.31E-08	0.352778	0.085	0.041	0.001485	Saliva_Mac	TMEM55A
GATAD13	1.02E-07	0.510405	0.13	0.076	0.001633	Saliva_Mac	GATAD1
LRRC8C1	1.04E-07	0.307165	0.072	0.033	0.001663	Saliva_Mac	LRRC8C
SDCCAG8	1.22E-07	0.525286	0.134	0.079	0.001952	Saliva_Mac	SDCCAG8
KLHL52	1.23E-07	0.272359	0.069	0.031	0.001964	Saliva_Mac	KLHL5
NABP1	1.36E-07	0.610227	0.233	0.167	0.002176	Saliva_Mac	NABP1
SMG1	1.4E-07	0.586318	0.181	0.121	0.002234	Saliva_Mac	SMG1
SESN31	1.42E-07	0.491452	0.135	0.08	0.002259	Saliva_Mac	SESN3
GPR137B	1.42E-07	0.496741	0.103	0.056	0.002269	Saliva_Mac	GPR137B
PIK3R13	1.43E-07	0.500991	0.171	0.11	0.002277	Saliva_Mac	PIK3R1
FZD1	1.75E-07	0.32465	0.054	0.022	0.002792	Saliva_Mac	FZD1
SMC1A2	1.87E-07	0.506295	0.126	0.074	0.00298	Saliva_Mac	SMC1A
SFXN31	2.26E-07	0.343495	0.087	0.043	0.00361	Saliva_Mac	SFXN3
PRMT2	2.28E-07	0.456532	0.226	0.158	0.003634	Saliva_Mac	PRMT2
HSPBAP1	2.34E-07	0.269715	0.06	0.026	0.003726	Saliva_Mac	HSPBAP1
MGAT51	2.58E-07	0.394162	0.07	0.033	0.004115	Saliva_Mac	MGAT5
LAMTOR11	2.62E-07	0.492211	0.345	0.286	0.00418	Saliva_Mac	LAMTOR1
HEXA	2.74E-07	0.599687	0.179	0.12	0.00437	Saliva_Mac	HEXA
SLC39A102	2.85E-07	0.466502	0.105	0.058	0.004542	Saliva_Mac	SLC39A10
UBAC1	2.92E-07	0.506769	0.143	0.089	0.004658	Saliva_Mac	UBAC1
PPP1R14B0	3.05E-07	0.624198	0.236	0.177	0.004862	Saliva_Mac	PPP1R14B
RNF166	3.19E-07	0.26617	0.096	0.049	0.005087	Saliva_Mac	RNF166
TTC144	3.23E-07	0.525111	0.159	0.102	0.005148	Saliva_Mac	TTC14
HECTD2	3.32E-07	0.303661	0.056	0.024	0.005287	Saliva_Mac	HECTD2
JUND5	3.36E-07	0.423009	0.496	0.464	0.005365	Saliva_Mac	JUND
AHR1	3.63E-07	0.49539	0.168	0.108	0.005796	Saliva_Mac	AHR
BNIP21	3.71E-07	0.603458	0.182	0.125	0.005912	Saliva_Mac	BNIP2
MAP3K22	3.97E-07	0.648608	0.181	0.124	0.006328	Saliva_Mac	MAP3K2
MGAT12	4.1E-07	0.611699	0.271	0.213	0.006535	Saliva_Mac	MGAT1
PRKDC3	4.25E-07	0.554604	0.153	0.098	0.00678	Saliva_Mac	PRKDC
SIRPA	4.55E-07	0.510578	0.135	0.083	0.007261	Saliva_Mac	SIRPA
STK101	4.88E-07	0.276105	0.099	0.053	0.007779	Saliva_Mac	STK10
CALM16	4.94E-07	0.289764	0.675	0.651	0.007879	Saliva_Mac	CALM1
CARD6	5.43E-07	0.271219	0.051	0.021	0.008658	Saliva_Mac	CARD6
MX2	5.63E-07	0.272257	0.072	0.034	0.008986	Saliva_Mac	MX2
IRAK12	6.07E-07	0.415322	0.096	0.052	0.009675	Saliva_Mac	IRAK1
FABP52	6.17E-07	0.574671	0.2	0.135	0.009842	Saliva_Mac	FABP5
MBP	6.17E-07	0.508464	0.215	0.154	0.009845	Saliva_Mac	MBP
NUP214	6.48E-07	0.431512	0.143	0.089	0.010329	Saliva_Mac	NUP214
LAP32	6.5E-07	0.548226	0.182	0.126	0.010364	Saliva_Mac	LAP3
ARID3A1	6.69E-07	0.46726	0.083	0.043	0.010674	Saliva_Mac	ARID3A
CARD81	6.74E-07	0.425817	0.099	0.054	0.010748	Saliva_Mac	CARD8
GCA	7.29E-07	0.374787	0.123	0.073	0.011625	Saliva_Mac	GCA
EPS83	7.88E-07	0.459415	0.13	0.079	0.012576	Saliva_Mac	EPS8
KIAA01411	7.91E-07	0.375933	0.079	0.04	0.012616	Saliva_Mac	KIAA0141

PLEC3	8.35E-07	0.569959	0.213	0.156	0.013318	Saliva_Mac	PLEC
JDP21	8.52E-07	0.404369	0.083	0.043	0.013582	Saliva_Mac	JDP2
ALDH1A12	8.67E-07	0.589025	0.126	0.076	0.013835	Saliva_Mac	ALDH1A1
STK17B2	9.85E-07	0.410375	0.162	0.105	0.015709	Saliva_Mac	STK17B
SPTLC23	1.08E-06	0.549776	0.13	0.081	0.017182	Saliva_Mac	SPTLC2
MAP3K31	1.13E-06	0.385896	0.072	0.035	0.018089	Saliva_Mac	MAP3K3
LPIN21	1.26E-06	0.419713	0.123	0.074	0.020114	Saliva_Mac	LPIN2
CCDC502	1.32E-06	0.599162	0.191	0.138	0.021113	Saliva_Mac	CCDC50
ANKRD123	1.42E-06	0.641796	0.363	0.323	0.02264	Saliva_Mac	ANKRD12
CLIC21	1.42E-06	0.402072	0.088	0.047	0.022646	Saliva_Mac	CLIC2
IRAK32	1.46E-06	0.355148	0.058	0.026	0.023252	Saliva_Mac	IRAK3
DPYD1	1.53E-06	0.359555	0.081	0.042	0.024462	Saliva_Mac	DPYD
GATM2	1.56E-06	0.550651	0.125	0.078	0.024959	Saliva_Mac	GATM
MBD22	1.62E-06	0.560014	0.193	0.138	0.025838	Saliva_Mac	MBD2
ARHGEF2	1.64E-06	0.379706	0.117	0.069	0.026188	Saliva_Mac	ARHGEF2
CANX4	1.64E-06	0.555272	0.352	0.312	0.026202	Saliva_Mac	CANX
MCM5	1.7E-06	0.359893	0.069	0.033	0.02716	Saliva_Mac	MCM5
LRRK1	1.77E-06	0.288399	0.049	0.02	0.028187	Saliva_Mac	LRRK1
ADCK2	1.85E-06	0.405803	0.076	0.039	0.029505	Saliva_Mac	ADCK2
ME21	1.91E-06	0.355416	0.087	0.046	0.030506	Saliva_Mac	ME2
SLC18B1	1.96E-06	0.30797	0.06	0.027	0.031247	Saliva_Mac	SLC18B1
NCOA31	2.08E-06	0.548223	0.152	0.1	0.033099	Saliva_Mac	NCOA3
FOXN31	2.11E-06	0.533234	0.181	0.125	0.033593	Saliva_Mac	FOXN3
CTSA2	2.32E-06	0.518697	0.188	0.132	0.036977	Saliva_Mac	CTSA
PCNXL43	2.48E-06	0.429086	0.103	0.059	0.039533	Saliva_Mac	PCNXL4
KIDINS220	2.66E-06	0.495941	0.153	0.102	0.042499	Saliva_Mac	KIDINS220
TPPP32	2.67E-06	0.404258	0.168	0.109	0.04256	Saliva_Mac	TPPP3
EPB41L1	3.23E-06	0.444872	0.063	0.031	0.051588	Saliva_Mac	EPB41L1
TGFBR22	3.26E-06	0.395851	0.171	0.116	0.051989	Saliva_Mac	TGFBR2
SON3	3.74E-06	0.427335	0.444	0.41	0.0596	Saliva_Mac	SON
BICD2	3.88E-06	0.423793	0.088	0.049	0.061824	Saliva_Mac	BICD2
P2RX41	4.03E-06	0.504015	0.11	0.066	0.064259	Saliva_Mac	P2RX4
PAPD4	4.18E-06	0.495674	0.108	0.065	0.066588	Saliva_Mac	PAPD4
FUCA12	4.19E-06	0.74183	0.188	0.139	0.066898	Saliva_Mac	FUCA1
FUS4	4.27E-06	0.534226	0.329	0.277	0.068113	Saliva_Mac	FUS
ACSL4	4.56E-06	0.286741	0.065	0.032	0.072753	Saliva_Mac	ACSL4
SGMS11	4.73E-06	0.533694	0.096	0.055	0.075384	Saliva_Mac	SGMS1
DDAH21	5.11E-06	0.591863	0.264	0.211	0.081521	Saliva_Mac	DDAH2
HNRNPU.A	5.16E-06	0.488367	0.106	0.064	0.082333	Saliva_Mac	HNRNPU.AS1
TKT	5.22E-06	0.433402	0.231	0.174	0.083321	Saliva_Mac	TKT
SPPL2A3	5.42E-06	0.577242	0.181	0.13	0.086497	Saliva_Mac	SPPL2A
BRI3BP	5.53E-06	0.278902	0.07	0.036	0.088184	Saliva_Mac	BRI3BP
STAT5A	5.57E-06	0.291241	0.081	0.043	0.088834	Saliva_Mac	STAT5A
CAT1	5.59E-06	0.515284	0.166	0.115	0.089169	Saliva_Mac	CAT
EHD41	6.19E-06	0.476088	0.139	0.091	0.098656	Saliva_Mac	EHD4
ALCAM1	6.76E-06	0.551353	0.144	0.097	0.107851	Saliva_Mac	ALCAM
HADHA1	6.77E-06	0.560972	0.251	0.2	0.107944	Saliva_Mac	HADHA

STAT11	7.21E-06	0.539395	0.134	0.087	0.115	Saliva_Ma	STAT1
BLNK1	7.27E-06	0.412384	0.087	0.048	0.115887	Saliva_Ma	BLNK
SEMA4A1	7.68E-06	0.28547	0.072	0.037	0.122554	Saliva_Ma	SEMA4A
DRAM2	7.71E-06	0.544611	0.179	0.128	0.12297	Saliva_Ma	DRAM2
ZMYM6	7.86E-06	0.298309	0.056	0.026	0.125411	Saliva_Ma	ZMYM6
VPS352	8.48E-06	0.605914	0.231	0.184	0.135173	Saliva_Ma	VPS35
AGO4	8.99E-06	0.286652	0.06	0.029	0.14332	Saliva_Ma	AGO4
LAMP24	9.21E-06	0.678974	0.276	0.236	0.146826	Saliva_Ma	LAMP2
INPPL1	9.33E-06	0.375131	0.078	0.042	0.148754	Saliva_Ma	INPPL1
LINC00936	9.5E-06	0.259959	0.123	0.075	0.151496	Saliva_Ma	LINC00936
MR1	9.62E-06	0.288617	0.052	0.024	0.153434	Saliva_Ma	MR1
SNX52	9.63E-06	0.58527	0.159	0.111	0.153525	Saliva_Ma	SNX5
EPS151	9.91E-06	0.574027	0.157	0.11	0.158068	Saliva_Ma	EPS15
AKT33	1E-05	0.273957	0.074	0.039	0.159541	Saliva_Ma	AKT3
SF3B14	1.05E-05	0.592404	0.294	0.253	0.167866	Saliva_Ma	SF3B1
CHD42	1.06E-05	0.572136	0.208	0.16	0.168601	Saliva_Ma	CHD4
TBC1D14	1.08E-05	0.421938	0.097	0.058	0.17158	Saliva_Ma	TBC1D14
SRRM25	1.11E-05	0.488009	0.381	0.348	0.177206	Saliva_Ma	SRRM2
MAML22	1.16E-05	0.392236	0.088	0.05	0.185459	Saliva_Ma	MAML2
CNTLN2	1.19E-05	0.320295	0.07	0.037	0.190108	Saliva_Ma	CNTLN
ATXN1	1.25E-05	0.413664	0.116	0.072	0.199571	Saliva_Ma	ATXN1
FAM120A2	1.29E-05	0.628777	0.195	0.148	0.205018	Saliva_Ma	FAM120A
TRMT1	1.29E-05	0.380004	0.094	0.054	0.205025	Saliva_Ma	TRMT1
ITSN21	1.29E-05	0.572213	0.191	0.142	0.205528	Saliva_Ma	ITSN2
XAF12	1.38E-05	0.286788	0.079	0.043	0.220662	Saliva_Ma	XAF1
PAK1	1.4E-05	0.383981	0.106	0.065	0.222991	Saliva_Ma	PAK1
WDR11	1.42E-05	0.381572	0.088	0.051	0.226216	Saliva_Ma	WDR11
TATDN3	1.49E-05	0.348581	0.072	0.039	0.237052	Saliva_Ma	TATDN3
ATP6AP12	1.5E-05	0.553159	0.208	0.161	0.238618	Saliva_Ma	ATP6AP1
KIAA0100	1.5E-05	0.382101	0.087	0.05	0.24	Saliva_Ma	KIAA0100
MTMR14	1.59E-05	0.372057	0.11	0.068	0.253104	Saliva_Ma	MTMR14
TFRC	1.71E-05	0.390824	0.092	0.054	0.272914	Saliva_Ma	TFRC
RELT	1.72E-05	0.250137	0.061	0.031	0.274083	Saliva_Ma	RELT
TBC1D22A	1.75E-05	0.384971	0.092	0.054	0.279587	Saliva_Ma	TBC1D22A
ANGEL2	1.81E-05	0.312895	0.069	0.036	0.289088	Saliva_Ma	ANGEL2
IKBKB1	1.83E-05	0.446559	0.088	0.051	0.291182	Saliva_Ma	IKBKB
BLVRA	1.93E-05	0.560025	0.159	0.113	0.307542	Saliva_Ma	BLVRA
FNIP12	1.93E-05	0.48592	0.128	0.085	0.308114	Saliva_Ma	FNIP1
MANBA	1.93E-05	0.34264	0.074	0.04	0.308612	Saliva_Ma	MANBA
HNRNPA2E	1.94E-05	0.303269	0.527	0.492	0.308692	Saliva_Ma	HNRNPA2B1
ITGA52	2.01E-05	0.276074	0.074	0.04	0.320302	Saliva_Ma	ITGA5
HEXDC1	2.09E-05	0.303438	0.072	0.039	0.333372	Saliva_Ma	HEXDC
RP11.488L	2.11E-05	0.260455	0.043	0.019	0.335898	Saliva_Ma	RP11.488L18.10
RBM254	2.14E-05	0.516435	0.283	0.24	0.341557	Saliva_Ma	RBM25
GTF2I6	2.24E-05	0.594993	0.2	0.154	0.357664	Saliva_Ma	GTF2I
SFPQ4	2.37E-05	0.51915	0.325	0.286	0.377673	Saliva_Ma	SFPQ
RAB342	2.42E-05	0.389718	0.15	0.102	0.386172	Saliva_Ma	RAB34

ZNF1062	2.51E-05	0.603707	0.184	0.139	0.400517	Saliva_Mac	ZNF106
NDUFB86	2.53E-05	0.50659	0.224	0.178	0.402956	Saliva_Mac	NDUFB8
STAG22	2.62E-05	0.483337	0.157	0.111	0.418587	Saliva_Mac	STAG2
HERC12	2.63E-05	0.384064	0.106	0.066	0.419853	Saliva_Mac	HERC1
PCF111	2.67E-05	0.492839	0.155	0.11	0.426514	Saliva_Mac	PCF11
MMD	2.7E-05	0.274312	0.058	0.029	0.430205	Saliva_Mac	MMD
UEVLD	2.74E-05	0.291345	0.049	0.023	0.436896	Saliva_Mac	UEVLD
SLC12A71	2.8E-05	0.310879	0.051	0.024	0.44677	Saliva_Mac	SLC12A7
RGS21	2.84E-05	0.42841	0.146	0.097	0.452477	Saliva_Mac	RGS2
KIF20B	2.85E-05	0.298712	0.072	0.039	0.453801	Saliva_Mac	KIF20B
KIF16B	2.95E-05	0.274105	0.065	0.034	0.46992	Saliva_Mac	KIF16B
CEP3502	3.47E-05	0.388772	0.177	0.128	0.553352	Saliva_Mac	CEP350
FAM91A1	3.52E-05	0.465329	0.108	0.069	0.561816	Saliva_Mac	FAM91A1
STON2	3.66E-05	0.263845	0.038	0.016	0.584389	Saliva_Mac	STON2
MOB3C	3.86E-05	0.260236	0.045	0.021	0.615591	Saliva_Mac	MOB3C
LIMD12	3.94E-05	0.286936	0.054	0.027	0.627766	Saliva_Mac	LIMD1
RAP2B	4.08E-05	0.431501	0.197	0.148	0.651032	Saliva_Mac	RAP2B
ATP6V0A1	4.22E-05	0.425827	0.094	0.057	0.673157	Saliva_Mac	ATP6V0A1
EGFL71	4.25E-05	0.477033	0.121	0.079	0.677309	Saliva_Mac	EGFL7
KCNQ1	4.37E-05	0.339751	0.065	0.035	0.696242	Saliva_Mac	KCNQ1
SP1003	4.41E-05	0.419728	0.19	0.139	0.702834	Saliva_Mac	SP100
BTN2A21	4.47E-05	0.272611	0.047	0.022	0.713411	Saliva_Mac	BTN2A2
RGCC3	4.68E-05	0.413737	0.276	0.214	0.745804	Saliva_Mac	RGCC
RAB3IL11	4.73E-05	0.311747	0.063	0.033	0.754581	Saliva_Mac	RAB3IL1
AGPAT2	4.74E-05	0.527623	0.171	0.127	0.755607	Saliva_Mac	AGPAT2
NIPBL1	4.99E-05	0.577298	0.195	0.151	0.79602	Saliva_Mac	NIPBL
RUFY1	5.27E-05	0.399521	0.088	0.053	0.840033	Saliva_Mac	RUFY1
GNB12	5.34E-05	0.643152	0.251	0.214	0.851645	Saliva_Mac	GNB1
SERINC13	5.38E-05	0.612171	0.238	0.197	0.858565	Saliva_Mac	SERINC1
ELF1	5.61E-05	0.49473	0.224	0.178	0.894293	Saliva_Mac	ELF1
MBD31	5.71E-05	0.388407	0.076	0.043	0.910635	Saliva_Mac	MBD3
WDR36	6.13E-05	0.387715	0.072	0.041	0.977717	Saliva_Mac	WDR36
NUFIP21	6.15E-05	0.57818	0.195	0.152	0.980279	Saliva_Mac	NUFIP2
FAM53B	6.21E-05	0.256936	0.056	0.028	0.98998	Saliva_Mac	FAM53B
IFI27L23	6.41E-05	0.273836	0.193	0.14	1	Saliva_Mac	IFI27L2
PPP6R11	6.8E-05	0.457533	0.119	0.079	1	Saliva_Mac	PPP6R1
SRSF75	6.93E-05	0.396347	0.379	0.343	1	Saliva_Mac	SRSF7
AMDHD2	7.12E-05	0.35717	0.083	0.049	1	Saliva_Mac	AMDHD2
MTM1	7.79E-05	0.312075	0.06	0.031	1	Saliva_Mac	MTM1
RPS6KA1	8.2E-05	0.301317	0.081	0.047	1	Saliva_Mac	RPS6KA1
ATP2B44	8.29E-05	0.412593	0.121	0.08	1	Saliva_Mac	ATP2B4
SEL1L2	8.54E-05	0.45805	0.134	0.093	1	Saliva_Mac	SEL1L
POMP1	8.82E-05	0.41447	0.424	0.403	1	Saliva_Mac	POMP
NCOR21	8.84E-05	0.51235	0.143	0.101	1	Saliva_Mac	NCOR2
TRIM222	9E-05	0.258843	0.088	0.053	1	Saliva_Mac	TRIM22
FAM20C2	9.06E-05	0.258776	0.056	0.029	1	Saliva_Mac	FAM20C
PRNP1	9.09E-05	0.475823	0.208	0.16	1	Saliva_Mac	PRNP

KDM7A	9.11E-05	0.35014	0.083	0.049	1	Saliva_Ma(	KDM7A
FAM49A	9.48E-05	0.295615	0.072	0.041	1	Saliva_Ma(	FAM49A
KDM5A1	9.48E-05	0.482805	0.164	0.122	1	Saliva_Ma(	KDM5A
POLK2	9.81E-05	0.404037	0.108	0.071	1	Saliva_Ma(	POLK
ZCCHC242	0.000104	0.360303	0.063	0.034	1	Saliva_Ma(	ZCCHC24
RIOK2	0.000109	0.348922	0.072	0.041	1	Saliva_Ma(	RIOK2
NUDT1	0.000113	0.428565	0.137	0.096	1	Saliva_Ma(	NUDT1
TCOF1	0.000115	0.274049	0.079	0.047	1	Saliva_Ma(	TCOF1
STARD132	0.000116	0.287132	0.042	0.019	1	Saliva_Ma(	STARD13
CCDC1121	0.000117	0.428684	0.096	0.06	1	Saliva_Ma(	CCDC112
FGD4	0.000117	0.450396	0.106	0.07	1	Saliva_Ma(	FGD4
PIAS1	0.000119	0.501151	0.146	0.106	1	Saliva_Ma(	PIAS1
PRKX	0.000121	0.356954	0.074	0.043	1	Saliva_Ma(	PRKX
RP2	0.000122	0.270259	0.054	0.028	1	Saliva_Ma(	RP2
TNRC6B3	0.000126	0.560567	0.213	0.172	1	Saliva_Ma(	TNRC6B
KIAA15512	0.000136	0.388758	0.162	0.118	1	Saliva_Ma(	KIAA1551
PPIA	0.000136	0.291894	0.579	0.59	1	Saliva_Ma(	PPIA
DIAPH22	0.000146	0.366368	0.088	0.055	1	Saliva_Ma(	DIAPH2
FAM111A2	0.000146	0.37699	0.083	0.05	1	Saliva_Ma(	FAM111A
SORL12	0.000149	0.531038	0.108	0.072	1	Saliva_Ma(	SORL1
PSMA74	0.000151	0.262519	0.484	0.464	1	Saliva_Ma(	PSMA7
CSDE12	0.000158	0.451337	0.377	0.359	1	Saliva_Ma(	CSDE1
ADAM152	0.000158	0.489794	0.112	0.075	1	Saliva_Ma(	ADAM15
CAPZA2	0.000161	0.522788	0.309	0.279	1	Saliva_Ma(	CAPZA2
LAMTOR4	0.000166	0.28893	0.417	0.389	1	Saliva_Ma(	LAMTOR4
LRRC581	0.000166	0.406712	0.11	0.073	1	Saliva_Ma(	LRRC58
MARCH2	0.000168	0.369353	0.11	0.073	1	Saliva_Ma(	MARCH2
MGAT4A1	0.000171	0.477451	0.144	0.104	1	Saliva_Ma(	MGAT4A
AHSA22	0.000176	0.402979	0.108	0.072	1	Saliva_Ma(	AHSA2
PKN12	0.000185	0.35097	0.13	0.09	1	Saliva_Ma(	PKN1
CHM2	0.000187	0.36294	0.072	0.042	1	Saliva_Ma(	CHM
RAB20	0.000189	0.296199	0.047	0.024	1	Saliva_Ma(	RAB20
PGGT1B1	0.000203	0.382094	0.081	0.05	1	Saliva_Ma(	PGGT1B
GLS2	0.000203	0.458607	0.159	0.117	1	Saliva_Ma(	GLS
C17orf622	0.000207	0.359222	0.132	0.091	1	Saliva_Ma(	C17orf62
MACF13	0.000211	0.509905	0.208	0.168	1	Saliva_Ma(	MACF1
CRYL1	0.000213	0.449406	0.134	0.096	1	Saliva_Ma(	CRYL1
CCAR12	0.000214	0.500402	0.143	0.104	1	Saliva_Ma(	CCAR1
RAPH1	0.000214	0.421616	0.085	0.053	1	Saliva_Ma(	RAPH1
SOCS6	0.000224	0.361357	0.076	0.045	1	Saliva_Ma(	SOCS6
STT3B2	0.000247	0.550444	0.171	0.133	1	Saliva_Ma(	STT3B
REL	0.000269	0.266222	0.235	0.186	1	Saliva_Ma(	REL
NPTX2	0.000269	0.291723	0.051	0.026	1	Saliva_Ma(	NPTX2
FADS11	0.000269	0.286262	0.052	0.028	1	Saliva_Ma(	FADS1
AP3B13	0.000277	0.500421	0.125	0.089	1	Saliva_Ma(	AP3B1
BID	0.000291	0.313675	0.164	0.121	1	Saliva_Ma(	BID
GDI2	0.000293	0.496964	0.296	0.268	1	Saliva_Ma(	GDI2

PRPF38B	0.000301	0.452941	0.233	0.193	1	Saliva_Ma	PRPF38B
SSBP44	0.000307	0.29898	0.144	0.102	1	Saliva_Ma	SSBP4
ATP6V1B2	0.000311	0.451747	0.125	0.088	1	Saliva_Ma	ATP6V1B2
STIM22	0.000313	0.345663	0.087	0.054	1	Saliva_Ma	STIM2
EP300	0.000334	0.419154	0.106	0.072	1	Saliva_Ma	EP300
ARHGAP21	0.000356	0.487222	0.132	0.095	1	Saliva_Ma	ARHGAP21
PLEKHM2	0.000358	0.394045	0.097	0.064	1	Saliva_Ma	PLEKHM2
C4orf481	0.000393	0.444824	0.184	0.143	1	Saliva_Ma	C4orf48
ELOVL11	0.000393	0.386063	0.135	0.098	1	Saliva_Ma	ELOVL1
AGPS1	0.000417	0.381794	0.096	0.063	1	Saliva_Ma	AGPS
BAZ2B1	0.000432	0.633657	0.182	0.148	1	Saliva_Ma	BAZ2B
ARHGAP17	0.000441	0.283894	0.063	0.036	1	Saliva_Ma	ARHGAP17
RASGEF1B	0.000466	0.323964	0.099	0.066	1	Saliva_Ma	RASGEF1B
UTP6	0.000468	0.301912	0.092	0.06	1	Saliva_Ma	UTP6
HEXB3	0.000482	0.459624	0.199	0.162	1	Saliva_Ma	HEXB
RCBTB2	0.000505	0.351609	0.083	0.053	1	Saliva_Ma	RCBTB2
APC2	0.000524	0.390684	0.092	0.061	1	Saliva_Ma	APC
ARPC41	0.000536	0.458932	0.265	0.232	1	Saliva_Ma	ARPC4
FKBP5	0.000553	0.283239	0.079	0.049	1	Saliva_Ma	FKBP5
TRIM563	0.000563	0.362807	0.112	0.078	1	Saliva_Ma	TRIM56
ATP10D	0.000587	0.29064	0.052	0.029	1	Saliva_Ma	ATP10D
DUSP231	0.000627	0.40018	0.19	0.153	1	Saliva_Ma	DUSP23
NEK61	0.000627	0.257635	0.061	0.036	1	Saliva_Ma	NEK6
TNS3	0.000642	0.305187	0.054	0.03	1	Saliva_Ma	TNS3
DUSP24	0.000648	0.45414	0.168	0.126	1	Saliva_Ma	DUSP2
SUPT16H2	0.000651	0.528031	0.144	0.109	1	Saliva_Ma	SUPT16H
ANKRD13D	0.000652	0.335238	0.103	0.07	1	Saliva_Ma	ANKRD13D
DDX3X2	0.000662	0.46152	0.323	0.301	1	Saliva_Ma	DDX3X
PPIG4	0.000729	0.458724	0.278	0.25	1	Saliva_Ma	PPIG
HPS1	0.000734	0.415194	0.13	0.095	1	Saliva_Ma	HPS1
PHYKPL2	0.000757	0.315863	0.103	0.07	1	Saliva_Ma	PHYKPL
RBM264	0.000764	0.484016	0.132	0.098	1	Saliva_Ma	RBM26
ATP6V1F	0.000775	0.502216	0.415	0.419	1	Saliva_Ma	ATP6V1F
PPIP5K22	0.000791	0.433675	0.099	0.068	1	Saliva_Ma	PPIP5K2
ADAR3	0.000805	0.475391	0.171	0.136	1	Saliva_Ma	ADAR
CEPT1	0.000829	0.322526	0.069	0.042	1	Saliva_Ma	CEPT1
COMMD9	0.000843	0.359611	0.116	0.082	1	Saliva_Ma	COMMD9
ZMAT11	0.000856	0.282549	0.058	0.033	1	Saliva_Ma	ZMAT1
CPQ1	0.00087	0.299319	0.11	0.075	1	Saliva_Ma	CPQ
MYCBP23	0.000891	0.385773	0.182	0.145	1	Saliva_Ma	MYCBP2
RAB8A2	0.000902	0.441324	0.173	0.137	1	Saliva_Ma	RAB8A
ABCC5	0.000902	0.29428	0.056	0.032	1	Saliva_Ma	ABCC5
VCPIP1	0.000924	0.399112	0.078	0.05	1	Saliva_Ma	VCPIP1
CYP20A13	0.000932	0.446001	0.13	0.096	1	Saliva_Ma	CYP20A1
VIPR12	0.000939	0.265009	0.04	0.02	1	Saliva_Ma	VIPR1
MTR1	0.000982	0.331643	0.065	0.039	1	Saliva_Ma	MTR
RBM332	0.000983	0.442877	0.119	0.086	1	Saliva_Ma	RBM33

SET1	0.000992	0.318085	0.341	0.311	1	Saliva_Ma(	SET
FAM96A1	0.001009	0.360926	0.134	0.099	1	Saliva_Ma(	FAM96A
USP24	0.001045	0.284768	0.061	0.037	1	Saliva_Ma(	USP24
FAM20A	0.001046	0.311399	0.063	0.038	1	Saliva_Ma(	FAM20A
FAM126A2	0.001118	0.314919	0.092	0.062	1	Saliva_Ma(	FAM126A
TAF11	0.001126	0.30577	0.06	0.035	1	Saliva_Ma(	TAF1
PLEKHB2	0.001139	0.575781	0.181	0.149	1	Saliva_Ma(	PLEKHB2
NT5C2	0.001139	0.344366	0.162	0.125	1	Saliva_Ma(	NT5C
LPGAT11	0.001147	0.445663	0.123	0.09	1	Saliva_Ma(	LPGAT1
TPST22	0.001161	0.290547	0.103	0.07	1	Saliva_Ma(	TPST2
SLC27A32	0.001162	0.265202	0.051	0.028	1	Saliva_Ma(	SLC27A3
LMO44	0.001181	0.466869	0.199	0.165	1	Saliva_Ma(	LMO4
EGR31	0.001203	0.344129	0.058	0.034	1	Saliva_Ma(	EGR3
ARHGDIA3	0.001206	0.335588	0.236	0.197	1	Saliva_Ma(	ARHGDIA
MAVS2	0.001227	0.286989	0.079	0.052	1	Saliva_Ma(	MAVS
SMCR8	0.001234	0.287813	0.056	0.033	1	Saliva_Ma(	SMCR8
TAF102	0.001267	0.423448	0.209	0.176	1	Saliva_Ma(	TAF10
FAM133B2	0.001291	0.560908	0.227	0.199	1	Saliva_Ma(	FAM133B
BCLAF15	0.001321	0.476966	0.229	0.2	1	Saliva_Ma(	BCLAF1
CTNND12	0.001333	0.447282	0.132	0.1	1	Saliva_Ma(	CTNND1
TOMM206	0.001366	0.394873	0.345	0.326	1	Saliva_Ma(	TOMM20
DNASE1L3	0.001467	0.348074	0.029	0.013	1	Saliva_Ma(	DNASE1L3
OAS11	0.001513	0.344961	0.112	0.08	1	Saliva_Ma(	OAS1
CREBL2	0.001557	0.443528	0.101	0.071	1	Saliva_Ma(	CREBL2
FGD5.AS11	0.001611	0.456111	0.143	0.11	1	Saliva_Ma(	FGD5.AS1
SSR13	0.001688	0.507562	0.22	0.191	1	Saliva_Ma(	SSR1
PLXNB21	0.001706	0.516637	0.188	0.156	1	Saliva_Ma(	PLXNB2
ZSWIM6	0.001775	0.476486	0.096	0.067	1	Saliva_Ma(	ZSWIM6
NHLRC32	0.001786	0.374006	0.103	0.073	1	Saliva_Ma(	NHLRC3
RSRC12	0.001801	0.467931	0.123	0.092	1	Saliva_Ma(	RSRC1
C2orf742	0.001815	0.306718	0.088	0.06	1	Saliva_Ma(	C2orf74
DNM1L1	0.001833	0.451127	0.11	0.08	1	Saliva_Ma(	DNM1L
CPPED1	0.001865	0.304158	0.092	0.064	1	Saliva_Ma(	CPPED1
FAM50A	0.001926	0.426366	0.164	0.131	1	Saliva_Ma(	FAM50A
SENP63	0.001927	0.389799	0.141	0.109	1	Saliva_Ma(	SENP6
IMMT	0.002045	0.36402	0.132	0.1	1	Saliva_Ma(	IMMT
SSB4	0.002083	0.377791	0.233	0.198	1	Saliva_Ma(	SSB
GLB1	0.002096	0.356804	0.101	0.072	1	Saliva_Ma(	GLB1
SESN1	0.00215	0.405929	0.081	0.055	1	Saliva_Ma(	SESN1
ACOT92	0.00216	0.318575	0.088	0.06	1	Saliva_Ma(	ACOT9
AP2M1	0.002161	0.418263	0.319	0.305	1	Saliva_Ma(	AP2M1
TMEM14C:	0.002174	0.506345	0.274	0.256	1	Saliva_Ma(	TMEM14C
PJA26	0.00229	0.482855	0.182	0.151	1	Saliva_Ma(	PJA2
MEPCE	0.002291	0.344206	0.09	0.062	1	Saliva_Ma(	MEPCE
PAPD7	0.002315	0.280183	0.049	0.028	1	Saliva_Ma(	PAPD7
B3GNT7	0.002349	0.253187	0.045	0.025	1	Saliva_Ma(	B3GNT7
CXCL9	0.002509	0.317876	0.011	0.003	1	Saliva_Ma(	CXCL9

PIP4K2A1	0.002574	0.291892	0.088	0.06	1	Saliva_Ma	PIP4K2A
MOSPD2	0.002599	0.264271	0.051	0.03	1	Saliva_Ma	MOSPD2
SLC38A9	0.002669	0.291895	0.054	0.033	1	Saliva_Ma	SLC38A9
LBR1	0.002702	0.28841	0.094	0.065	1	Saliva_Ma	LBR
RABEP1	0.002799	0.349388	0.106	0.078	1	Saliva_Ma	RABEP1
GON4L2	0.002915	0.338397	0.11	0.081	1	Saliva_Ma	GON4L
CREBRF	0.003226	0.462353	0.17	0.14	1	Saliva_Ma	CREBRF
C9orf72	0.003231	0.254046	0.072	0.047	1	Saliva_Ma	C9orf72
NKTR6	0.003271	0.4861	0.206	0.176	1	Saliva_Ma	NKTR
TAOK31	0.003384	0.446703	0.166	0.136	1	Saliva_Ma	TAOK3
PEX26	0.003424	0.254176	0.058	0.036	1	Saliva_Ma	PEX26
CTDNEP11	0.003755	0.417719	0.164	0.135	1	Saliva_Ma	CTDNEP1
DOCK5	0.003824	0.250313	0.051	0.03	1	Saliva_Ma	DOCK5
ZNF7913	0.003831	0.37968	0.097	0.07	1	Saliva_Ma	ZNF791
MCL11	0.003872	0.504622	0.381	0.382	1	Saliva_Ma	MCL1
SLC35F61	0.00391	0.327372	0.078	0.053	1	Saliva_Ma	SLC35F6
MLXIP1	0.003949	0.423004	0.13	0.101	1	Saliva_Ma	MLXIP
PNISR4	0.003965	0.347107	0.365	0.348	1	Saliva_Ma	PNISR
RAB11FIP2	0.003966	0.292808	0.067	0.044	1	Saliva_Ma	RAB11FIP2
STAT6	0.004129	0.36394	0.101	0.073	1	Saliva_Ma	STAT6
RIF13	0.004158	0.400001	0.121	0.092	1	Saliva_Ma	RIF1
MTMR101	0.004194	0.315418	0.065	0.043	1	Saliva_Ma	MTMR10
MFSD121	0.004274	0.394963	0.121	0.093	1	Saliva_Ma	MFSD12
DPP9	0.004305	0.27781	0.058	0.036	1	Saliva_Ma	DPP9
BPTF3	0.004306	0.482601	0.191	0.164	1	Saliva_Ma	BPTF
DYNC1LI1	0.004331	0.311235	0.116	0.087	1	Saliva_Ma	DYNC1LI1
HTT	0.004426	0.278258	0.069	0.045	1	Saliva_Ma	HTT
BNIP3L	0.004569	0.434606	0.305	0.297	1	Saliva_Ma	BNIP3L
FAM65A1	0.00466	0.272693	0.072	0.048	1	Saliva_Ma	FAM65A
MIF4GD	0.004676	0.262398	0.096	0.068	1	Saliva_Ma	MIF4GD
SGMS21	0.004684	0.420219	0.081	0.057	1	Saliva_Ma	SGMS2
RILP	0.004693	0.34835	0.081	0.057	1	Saliva_Ma	RILP
CTBS3	0.004767	0.471234	0.13	0.103	1	Saliva_Ma	CTBS
SERINC5	0.004776	0.333873	0.078	0.053	1	Saliva_Ma	SERINC5
MRE11A1	0.004901	0.287894	0.056	0.035	1	Saliva_Ma	MRE11A
SCAMP21	0.004945	0.47155	0.213	0.189	1	Saliva_Ma	SCAMP2
STAT2	0.005309	0.361904	0.097	0.071	1	Saliva_Ma	STAT2
LNPEP3	0.005324	0.292514	0.094	0.067	1	Saliva_Ma	LNPEP
GOLIM41	0.005429	0.405435	0.112	0.084	1	Saliva_Ma	GOLIM4
IQSEC11	0.005543	0.326922	0.076	0.052	1	Saliva_Ma	IQSEC1
CABIN11	0.005596	0.289855	0.079	0.055	1	Saliva_Ma	CABIN1
ATF7IP2	0.005627	0.375811	0.09	0.065	1	Saliva_Ma	ATF7IP
PBRM1	0.005637	0.468775	0.152	0.125	1	Saliva_Ma	PBRM1
DUSP221	0.005792	0.407777	0.117	0.09	1	Saliva_Ma	DUSP22
CHD11	0.005859	0.408498	0.161	0.132	1	Saliva_Ma	CHD1
ATP6AP21	0.00587	0.405492	0.262	0.239	1	Saliva_Ma	ATP6AP2
PTBP31	0.00596	0.527866	0.184	0.16	1	Saliva_Ma	PTBP3

EVA1B3	0.005987	0.296044	0.097	0.071	1	Saliva_Mac	EVA1B
FMNL21	0.006029	0.276489	0.061	0.04	1	Saliva_Mac	FMNL2
SNX271	0.006625	0.303038	0.078	0.054	1	Saliva_Mac	SNX27
PIK3CA2	0.006639	0.309579	0.061	0.04	1	Saliva_Mac	PIK3CA
UBR21	0.00669	0.356961	0.09	0.066	1	Saliva_Mac	UBR2
KMT2A5	0.006747	0.481744	0.157	0.131	1	Saliva_Mac	KMT2A
NTAN13	0.006851	0.518533	0.15	0.124	1	Saliva_Mac	NTAN1
KPNA41	0.006991	0.513589	0.171	0.147	1	Saliva_Mac	KPNA4
NSD14	0.007	0.354621	0.128	0.101	1	Saliva_Mac	NSD1
YTHDC21	0.007114	0.368716	0.092	0.067	1	Saliva_Mac	YTHDC2
PTEN1	0.007286	0.401195	0.155	0.128	1	Saliva_Mac	PTEN
BACH1	0.007438	0.476166	0.134	0.107	1	Saliva_Mac	BACH1
UNC119	0.007496	0.383127	0.097	0.072	1	Saliva_Mac	UNC119
LARP72	0.007545	0.416042	0.161	0.134	1	Saliva_Mac	LARP7
SMYD4	0.007638	0.257502	0.054	0.035	1	Saliva_Mac	SMYD4
TMX1	0.008087	0.414537	0.132	0.105	1	Saliva_Mac	TMX1
IVNS1ABP3	0.008179	0.468207	0.175	0.15	1	Saliva_Mac	IVNS1ABP
ZDHHC7	0.008265	0.281097	0.063	0.042	1	Saliva_Mac	ZDHHC7
CLCN7	0.00828	0.329917	0.061	0.041	1	Saliva_Mac	CLCN7
CUL52	0.008344	0.39192	0.103	0.078	1	Saliva_Mac	CUL5
SEPT21	0.008548	0.495485	0.249	0.236	1	Saliva_Mac	SEPT2
PRDX14	0.008663	0.279825	0.453	0.453	1	Saliva_Mac	PRDX1
TBC1D121	0.008716	0.276121	0.067	0.045	1	Saliva_Mac	TBC1D12
SYNGR21	0.008727	0.484828	0.249	0.235	1	Saliva_Mac	SYNGR2
CYP4V21	0.008834	0.253174	0.042	0.025	1	Saliva_Mac	CYP4V2
MSL3	0.009243	0.322089	0.09	0.066	1	Saliva_Mac	MSL3
FAM208A	0.009407	0.323648	0.087	0.063	1	Saliva_Mac	FAM208A
EPM2AIP1	0.009438	0.331565	0.097	0.073	1	Saliva_Mac	EPM2AIP1
DDX184	0.00948	0.41908	0.227	0.204	1	Saliva_Mac	DDX18
PHF20	0.009533	0.399006	0.171	0.145	1	Saliva_Mac	PHF20
WDFY31	0.009721	0.338493	0.072	0.051	1	Saliva_Mac	WDFY3
XPO11	0.009944	0.405959	0.128	0.103	1	Saliva_Mac	XPO1
KRT193	0	3.962137	0.981	0.118	0	Saliva_Acir	KRT19
TFCP2L12	0	3.108096	0.87	0.043	0	Saliva_Acir	TFCP2L1
KRT82	0	1.72651	0.854	0.196	0	Saliva_Acir	KRT8
SORBS24	0	1.678728	0.637	0.11	0	Saliva_Acir	SORBS2
KLK1	0	1.55333	0.27	0.009	0	Saliva_Acir	KLK1
MT.ATP87	0	1.551755	0.707	0.132	0	Saliva_Acir	MT.ATP8
SEMA3C1	0	1.497855	0.563	0.032	0	Saliva_Acir	SEMA3C
FOXC12	0	1.423899	0.609	0.109	0	Saliva_Acir	FOXC1
TMEM213	0	1.416594	0.442	0.004	0	Saliva_Acir	TMEM213
PKP22	0	1.352263	0.514	0.048	0	Saliva_Acir	PKP2
SCPEP11	0	1.323697	0.578	0.082	0	Saliva_Acir	SCPEP1
DSG21	0	1.261171	0.49	0.055	0	Saliva_Acir	DSG2
HSD11B2	0	1.177329	0.399	0.008	0	Saliva_Acir	HSD11B2
RNF1501	0	1.115562	0.427	0.014	0	Saliva_Acir	RNF150
PYGB1	0	1.070298	0.501	0.071	0	Saliva_Acir	PYGB

ERMP1	0	1.045398	0.422	0.025	0	Saliva_Acir	ERMP1
S100A2	0	1.044958	0.832	0.186	0	Saliva_Acir	S100A2
CLCNKB	0	1.033379	0.297	0.007	0	Saliva_Acir	CLCNKB
SHROOM3	0	1.007271	0.418	0.048	0	Saliva_Acir	SHROOM3
SLC4A11	0	1.006448	0.367	0.023	0	Saliva_Acir	SLC4A11
KCNK51	0	0.948143	0.357	0.009	0	Saliva_Acir	KCNK5
CLNK	0	0.941628	0.18	0.004	0	Saliva_Acir	CLNK
SCD5	0	0.876022	0.399	0.038	0	Saliva_Acir	SCD5
TF1	0	0.827319	0.297	0.016	0	Saliva_Acir	TF
MAML23	0	0.822816	0.408	0.048	0	Saliva_Acir	MAML2
PLLP1	0	0.821386	0.329	0.021	0	Saliva_Acir	PLLP
RASSF6	0	0.791553	0.28	0.014	0	Saliva_Acir	RASSF6
COLCA1	0	0.745223	0.289	0.017	0	Saliva_Acir	COLCA1
WDR72	0	0.698012	0.312	0.017	0	Saliva_Acir	WDR72
ADAM282	0	0.599022	0.302	0.024	0	Saliva_Acir	ADAM28
SLIT21	0	0.5873	0.287	0.023	0	Saliva_Acir	SLIT2
TMEM150(	0	0.552945	0.284	0.018	0	Saliva_Acir	TMEM150C
ZBTB7C2	0	0.507277	0.263	0.016	0	Saliva_Acir	ZBTB7C
IL19	0	0.486527	0.221	0.014	0	Saliva_Acir	IL19
NAV21	0	0.452307	0.238	0.016	0	Saliva_Acir	NAV2
CNTNAP3E	0	0.404117	0.225	0.014	0	Saliva_Acir	CNTNAP3B
TPPP1	0	0.401949	0.2	0.012	0	Saliva_Acir	TPPP
DLK2	0	0.391134	0.144	0.004	0	Saliva_Acir	DLK2
BSND	0	0.386527	0.095	0	0	Saliva_Acir	BSND
CHL11	0	0.382182	0.204	0.013	0	Saliva_Acir	CHL1
SUSD4	0	0.371526	0.198	0.011	0	Saliva_Acir	SUSD4
GABRB2	0	0.318286	0.142	0.002	0	Saliva_Acir	GABRB2
NWD1	0	0.278948	0.142	0.006	0	Saliva_Acir	NWD1
MTRNR2L1	1.4E-296	2.39395	0.9	0.266	2.2E-292	Saliva_Acir	MTRNR2L12
PRICKLE11	1.8E-293	0.620868	0.295	0.028	2.9E-289	Saliva_Acir	PRICKLE1
MT.CYB9	2.3E-291	2.43785	0.998	0.933	3.7E-287	Saliva_Acir	MT.CYB
MT.CO38	5.3E-285	2.556485	1	0.977	8.4E-281	Saliva_Acir	MT.CO3
SYNE24	5.9E-285	1.483113	0.654	0.135	9.5E-281	Saliva_Acir	SYNE2
ATP1A13	1E-284	2.111754	0.822	0.253	1.6E-280	Saliva_Acir	ATP1A1
RNF1522	1.2E-283	0.90993	0.325	0.034	1.9E-279	Saliva_Acir	RNF152
MT.ND39	1.8E-282	2.39522	1	0.794	2.8E-278	Saliva_Acir	MT.ND3
MTRNR2L1	6E-279	2.05764	0.541	0.096	9.5E-275	Saliva_Acir	MTRNR2L10
OGDHL	2E-278	0.286209	0.151	0.008	3.2E-274	Saliva_Acir	OGDHL
MT.ATP69	3.8E-278	2.353858	1	0.898	6E-274	Saliva_Acir	MT.ATP6
MT.CO29	5.3E-278	2.119483	1	0.959	8.5E-274	Saliva_Acir	MT.CO2
WFDC21	2.4E-276	1.924009	0.853	0.234	3.8E-272	Saliva_Acir	WFDC2
MT.ND49	7.1E-275	2.167439	0.998	0.953	1.1E-270	Saliva_Acir	MT.ND4
ATP6V1C2	3.4E-274	1.216393	0.422	0.058	5.4E-270	Saliva_Acir	ATP6V1C2
MEIS21	5.8E-273	1.213266	0.55	0.101	9.2E-269	Saliva_Acir	MEIS2
MUC161	3.8E-272	0.81408	0.24	0.02	6E-268	Saliva_Acir	MUC16
KRT72	3.9E-269	2.035219	0.885	0.269	6.3E-265	Saliva_Acir	KRT7
CX3CL11	1.1E-267	0.543685	0.253	0.022	1.8E-263	Saliva_Acir	CX3CL1

MT.ND59	7.8E-265	2.153184	0.996	0.701	1.2E-260	Saliva_Acir	MT.ND5
DMRT2	1.5E-261	0.298794	0.095	0.003	2.4E-257	Saliva_Acir	DMRT2
CLDN43	1.9E-257	1.254961	0.503	0.088	3E-253	Saliva_Acir	CLDN4
MT.ND19	7.4E-256	2.168578	1	0.927	1.2E-251	Saliva_Acir	MT.ND1
NFIB5	3.7E-255	1.530441	0.735	0.188	5.8E-251	Saliva_Acir	NFIB
RERG1	4.9E-254	0.513323	0.248	0.022	7.9E-250	Saliva_Acir	RERG
CHCHD106	2.8E-252	1.52501	0.819	0.242	4.4E-248	Saliva_Acir	CHCHD10
ENC11	1.2E-250	0.767411	0.31	0.035	1.9E-246	Saliva_Acir	ENC1
MT.ND29	1.3E-247	2.068837	0.998	0.907	2.1E-243	Saliva_Acir	MT.ND2
PBX13	8.9E-246	1.181894	0.558	0.112	1.4E-241	Saliva_Acir	PBX1
EPDR1	3E-244	0.346602	0.197	0.015	4.8E-240	Saliva_Acir	EPDR1
MT.CO19	3.5E-242	1.873283	1	0.985	5.5E-238	Saliva_Acir	MT.CO1
FXYD2	2.8E-237	0.588841	0.259	0.025	4.5E-233	Saliva_Acir	FXYD2
SYNJ2	8.8E-236	0.832462	0.359	0.049	1.4E-231	Saliva_Acir	SYNJ2
RP1.244F2	1.6E-235	0.321717	0.121	0.006	2.6E-231	Saliva_Acir	RP1.244F24.1
INHBA	1.8E-234	0.443088	0.242	0.022	2.8E-230	Saliva_Acir	INHBA
FUT21	1.1E-233	0.923506	0.425	0.068	1.7E-229	Saliva_Acir	FUT2
SOX22	7.4E-233	0.720379	0.335	0.044	1.2E-228	Saliva_Acir	SOX2
PLCL11	1.3E-230	0.327601	0.142	0.008	2E-226	Saliva_Acir	PLCL1
LAMB3	6.6E-228	0.965017	0.399	0.06	1E-223	Saliva_Acir	LAMB3
MT.ND4L9	3.2E-227	1.869453	0.924	0.414	5.2E-223	Saliva_Acir	MT.ND4L
ZKSCAN14	1.4E-224	1.084256	0.544	0.114	2.3E-220	Saliva_Acir	ZKSCAN1
COL17A1	6.9E-224	0.646713	0.284	0.032	1.1E-219	Saliva_Acir	COL17A1
KCNJ15	1.2E-222	0.308609	0.172	0.012	1.8E-218	Saliva_Acir	KCNJ15
RARRES12	5.8E-221	1.295441	0.308	0.039	9.3E-217	Saliva_Acir	RARRES1
RNF207	5.3E-219	0.310006	0.181	0.014	8.5E-215	Saliva_Acir	RNF207
PPM1L1	7.4E-218	0.696785	0.299	0.037	1.2E-213	Saliva_Acir	PPM1L
GPRC5C1	7.8E-218	0.708176	0.348	0.049	1.2E-213	Saliva_Acir	GPRC5C
SLC25A12	1.5E-216	0.404886	0.236	0.023	2.4E-212	Saliva_Acir	SLC25A12
LRFN4	1.4E-215	0.346952	0.17	0.012	2.3E-211	Saliva_Acir	LRFN4
AKAP61	3.7E-215	0.446694	0.202	0.018	5.8E-211	Saliva_Acir	AKAP6
CCL283	1.7E-212	1.216662	0.544	0.109	2.6E-208	Saliva_Acir	CCL28
KRT14	2.6E-212	0.309456	0.647	0.147	4.1E-208	Saliva_Acir	KRT14
ITGB42	6E-212	0.931058	0.457	0.086	9.6E-208	Saliva_Acir	ITGB4
NCKAP51	1.2E-211	0.520113	0.187	0.015	2E-207	Saliva_Acir	NCKAP5
KRT5	2.4E-210	0.924989	0.448	0.077	3.9E-206	Saliva_Acir	KRT5
ANK32	3.3E-209	0.795839	0.41	0.07	5.2E-205	Saliva_Acir	ANK3
PERP1	4E-208	1.441692	0.898	0.364	6.4E-204	Saliva_Acir	PERP
DST4	7.5E-207	1.460954	0.669	0.182	1.2E-202	Saliva_Acir	DST
ECI14	4.4E-204	1.128388	0.573	0.136	7.1E-200	Saliva_Acir	ECI1
MTRNR2L1	5.9E-204	2.691278	0.539	0.124	9.3E-200	Saliva_Acir	MTRNR2L1
GTF2I7	2E-202	1.149665	0.603	0.151	3.3E-198	Saliva_Acir	GTF2I
JAG21	3E-202	0.331977	0.161	0.012	4.9E-198	Saliva_Acir	JAG2
NR3C2	7E-202	0.461764	0.217	0.021	1.1E-197	Saliva_Acir	NR3C2
EPCAM3	2.8E-199	1.193249	0.571	0.128	4.4E-195	Saliva_Acir	EPCAM
PDLIM11	5.8E-197	1.464062	0.715	0.222	9.2E-193	Saliva_Acir	PDLIM1
CLCNKA	1E-195	0.382987	0.17	0.014	1.6E-191	Saliva_Acir	CLCNKA

FZD11	3.3E-195	0.368232	0.214	0.021	5.3E-191	Saliva_Acir	FZD1
CTSD2	1.1E-194	1.269447	0.82	0.29	1.8E-190	Saliva_Acir	CTSD
XIST8	3.7E-193	1.754014	0.516	0.121	5.8E-189	Saliva_Acir	XIST
TLE21	1.5E-189	0.584193	0.314	0.045	2.4E-185	Saliva_Acir	TLE2
CLU2	4.9E-189	1.949102	0.669	0.198	7.9E-185	Saliva_Acir	CLU
NAALADL2	4.3E-187	0.68726	0.321	0.048	6.8E-183	Saliva_Acir	NAALADL2
NGFR	6.8E-185	0.372143	0.136	0.009	1.1E-180	Saliva_Acir	NGFR
CD94	4.3E-184	1.479951	0.941	0.45	6.9E-180	Saliva_Acir	CD9
SCNN1A1	1.8E-183	0.882549	0.448	0.089	2.8E-179	Saliva_Acir	SCNN1A
ENDOD13	1.2E-182	0.51423	0.259	0.032	2E-178	Saliva_Acir	ENDOD1
CRISPLD1	4.7E-182	0.331652	0.164	0.014	7.5E-178	Saliva_Acir	CRISPLD1
CA121	2.9E-181	0.758491	0.338	0.054	4.7E-177	Saliva_Acir	CA12
WNT5B2	3E-181	0.277915	0.174	0.015	4.8E-177	Saliva_Acir	WNT5B
ATP1B11	1.1E-179	1.682887	0.805	0.323	1.7E-175	Saliva_Acir	ATP1B1
CMTM41	4.3E-179	0.511134	0.251	0.032	6.8E-175	Saliva_Acir	CMTM4
RUNX21	5.4E-179	0.576165	0.284	0.039	8.5E-175	Saliva_Acir	RUNX2
CTTNBP21	6.7E-179	0.345828	0.147	0.011	1.1E-174	Saliva_Acir	CTTNBP2
DSP1	4E-178	1.091806	0.622	0.172	6.4E-174	Saliva_Acir	DSP
OBSCN	4.8E-177	0.257559	0.13	0.009	7.7E-173	Saliva_Acir	OBSCN
FST1	6E-177	0.511693	0.225	0.025	9.6E-173	Saliva_Acir	FST
COL9A3	1.6E-176	0.546303	0.246	0.03	2.6E-172	Saliva_Acir	COL9A3
NNT	3.9E-176	0.575679	0.285	0.04	6.2E-172	Saliva_Acir	NNT
MCC	8.6E-176	0.471806	0.265	0.034	1.4E-171	Saliva_Acir	MCC
SLC6A61	2.2E-174	0.641776	0.28	0.039	3.5E-170	Saliva_Acir	SLC6A6
TGFB2	3E-174	0.423837	0.195	0.02	4.8E-170	Saliva_Acir	TGFB2
DSC22	2E-173	0.783928	0.369	0.067	3.3E-169	Saliva_Acir	DSC2
EFNA5	6.8E-173	0.509253	0.253	0.032	1.1E-168	Saliva_Acir	EFNA5
MTRNR2L8	4E-172	1.768304	0.781	0.284	6.4E-168	Saliva_Acir	MTRNR2L8
MTRNR2L6	6.9E-171	0.475134	0.2	0.021	1.1E-166	Saliva_Acir	MTRNR2L6
PTPRF3	1.7E-170	0.858308	0.467	0.105	2.7E-166	Saliva_Acir	PTPRF
LINC00969	4.1E-168	0.988898	0.48	0.109	6.6E-164	Saliva_Acir	LINC00969
LOXL4	6E-167	0.316646	0.161	0.014	9.5E-163	Saliva_Acir	LOXL4
NFIX3	1.6E-166	0.855912	0.474	0.108	2.6E-162	Saliva_Acir	NFIX
DAPL1	7.2E-166	0.505241	0.234	0.029	1.2E-161	Saliva_Acir	DAPL1
PDLIM43	3.2E-165	0.882157	0.469	0.103	5E-161	Saliva_Acir	PDLIM4
COL7A1	3.4E-165	0.332813	0.172	0.016	5.4E-161	Saliva_Acir	COL7A1
MAOB1	2.1E-163	0.347252	0.197	0.021	3.3E-159	Saliva_Acir	MAOB
MMP7	1.6E-161	0.727876	0.253	0.034	2.6E-157	Saliva_Acir	MMP7
PRKAA21	1.8E-161	0.452257	0.251	0.034	2.9E-157	Saliva_Acir	PRKAA2
IKZF21	6.3E-161	0.47775	0.284	0.042	1E-156	Saliva_Acir	IKZF2
NEBL	1.3E-160	0.780331	0.397	0.08	2E-156	Saliva_Acir	NEBL
TRIM564	2E-160	0.717938	0.389	0.076	3.2E-156	Saliva_Acir	TRIM56
TMEM63A	2.6E-159	0.73585	0.393	0.078	4.2E-155	Saliva_Acir	TMEM63A
COL9A2	1.5E-156	0.367699	0.163	0.015	2.4E-152	Saliva_Acir	COL9A2
MYOF2	1.6E-155	0.654987	0.329	0.057	2.6E-151	Saliva_Acir	MYOF
TACSTD2	4.2E-155	1.05634	0.851	0.324	6.7E-151	Saliva_Acir	TACSTD2
RPS178	5.9E-154	0.810872	0.72	0.22	9.5E-150	Saliva_Acir	RPS17

PCLO	8.2E-154	0.455354	0.195	0.022	1.3E-149	Saliva_Acir	PCLO
SLC14A11	7.7E-153	0.260167	0.066	0.003	1.2E-148	Saliva_Acir	SLC14A1
ZMAT12	2.6E-152	0.513274	0.238	0.032	4.1E-148	Saliva_Acir	ZMAT1
GNAL	6.1E-152	0.336898	0.164	0.016	9.7E-148	Saliva_Acir	GNAL
C5orf421	1.2E-151	0.54884	0.27	0.041	1.8E-147	Saliva_Acir	C5orf42
ANGPT11	2E-150	0.27335	0.142	0.012	3.1E-146	Saliva_Acir	ANGPT1
HIST3H2A	5.7E-150	0.310173	0.147	0.013	9E-146	Saliva_Acir	HIST3H2A
MYO19	3.1E-149	0.454424	0.259	0.038	5E-145	Saliva_Acir	MYO19
PPP1R12B1	2.8E-148	0.676449	0.293	0.048	4.5E-144	Saliva_Acir	PPP1R12B
HOOK22	5.7E-148	0.811972	0.435	0.101	9.2E-144	Saliva_Acir	HOOK2
DGKH2	6E-148	0.438674	0.246	0.035	9.6E-144	Saliva_Acir	DGKH
KIAA1217	1.4E-147	0.694458	0.363	0.072	2.3E-143	Saliva_Acir	KIAA1217
KRTCAP32	1.4E-145	0.693807	0.378	0.079	2.2E-141	Saliva_Acir	KRTCAP3
DDR11	6.3E-141	0.778099	0.456	0.113	1E-136	Saliva_Acir	DDR1
ITGA64	3.2E-140	0.849708	0.505	0.134	5.1E-136	Saliva_Acir	ITGA6
HIPK24	4E-140	0.695626	0.371	0.078	6.4E-136	Saliva_Acir	HIPK2
ENAH3	6.1E-140	0.847878	0.463	0.117	9.7E-136	Saliva_Acir	ENAH
BARX21	7.5E-138	0.666888	0.321	0.062	1.2E-133	Saliva_Acir	BARX2
KCNC4	2.2E-134	0.331598	0.172	0.02	3.5E-130	Saliva_Acir	KCNC4
ARHGAP18	1.3E-132	1.028896	0.397	0.094	2.1E-128	Saliva_Acir	ARHGAP18
FAXDC21	2.1E-132	0.35413	0.212	0.029	3.4E-128	Saliva_Acir	FAXDC2
CALCRL1	6.5E-132	0.339308	0.229	0.033	1E-127	Saliva_Acir	CALCRL
JADE11	1.1E-131	0.608779	0.295	0.053	1.8E-127	Saliva_Acir	JADE1
INPP5J	2.6E-130	0.250036	0.119	0.01	4.1E-126	Saliva_Acir	INPP5J
DAB2IP1	2.4E-129	0.348397	0.198	0.026	3.8E-125	Saliva_Acir	DAB2IP
MT.ND68	1.5E-128	0.833665	0.437	0.109	2.4E-124	Saliva_Acir	MT.ND6
RUNX11	1.6E-128	0.668149	0.422	0.102	2.6E-124	Saliva_Acir	RUNX1
NET12	6.3E-127	0.733734	0.437	0.111	1E-122	Saliva_Acir	NET1
CDK62	8E-127	0.707006	0.371	0.083	1.3E-122	Saliva_Acir	CDK6
ZNF827	1.7E-126	0.408752	0.219	0.033	2.8E-122	Saliva_Acir	ZNF827
WNK21	4.1E-125	0.351478	0.21	0.03	6.5E-121	Saliva_Acir	WNK2
ERBB22	8.4E-124	0.571204	0.299	0.058	1.3E-119	Saliva_Acir	ERBB2
NDUFS12	1.7E-123	0.65821	0.376	0.087	2.7E-119	Saliva_Acir	NDUFS1
FBXO2	7.5E-123	0.373292	0.185	0.024	1.2E-118	Saliva_Acir	FBXO2
IRX11	1.9E-122	0.251828	0.157	0.018	3E-118	Saliva_Acir	IRX1
SLC25A44	3.2E-122	0.736704	0.431	0.113	5E-118	Saliva_Acir	SLC25A4
BCL11B1	4.7E-122	0.274248	0.197	0.026	7.6E-118	Saliva_Acir	BCL11B
GLYATL21	1.1E-121	0.322513	0.181	0.023	1.8E-117	Saliva_Acir	GLYATL2
IQGAP23	1.6E-121	0.738702	0.442	0.114	2.5E-117	Saliva_Acir	IQGAP2
IGFBP23	1.3E-120	0.570574	0.282	0.052	2.1E-116	Saliva_Acir	IGFBP2
PLCB11	4.1E-120	0.45358	0.236	0.039	6.5E-116	Saliva_Acir	PLCB1
ZNF4931	4.2E-120	0.426083	0.248	0.041	6.8E-116	Saliva_Acir	ZNF493
THBS1	1.5E-119	0.605543	0.361	0.079	2.4E-115	Saliva_Acir	THBS1
SLC27A11	3.4E-119	0.362153	0.219	0.034	5.4E-115	Saliva_Acir	SLC27A1
GBAS2	5.4E-118	0.698758	0.45	0.124	8.6E-114	Saliva_Acir	GBAS
LAMA52	1.2E-117	0.303708	0.197	0.028	1.9E-113	Saliva_Acir	LAMA5
SRGAP31	7.7E-117	0.411261	0.217	0.034	1.2E-112	Saliva_Acir	SRGAP3

AGL1	6.6E-116	0.407852	0.242	0.041	1.1E-111	Saliva_Acir	AGL
DBN11	1.5E-115	0.411515	0.276	0.051	2.3E-111	Saliva_Acir	DBN1
INADL2	1.5E-115	0.615607	0.359	0.085	2.4E-111	Saliva_Acir	INADL
LNPEP4	2E-115	0.500361	0.318	0.066	3.1E-111	Saliva_Acir	LNPEP
RIC31	4.2E-115	0.657866	0.371	0.089	6.7E-111	Saliva_Acir	RIC3
TFEB	1.1E-114	0.334952	0.195	0.028	1.8E-110	Saliva_Acir	TFEB
MEIS1	1.7E-114	0.496861	0.261	0.048	2.7E-110	Saliva_Acir	MEIS1
ATP5B1	3.5E-113	1.029715	0.773	0.369	5.6E-109	Saliva_Acir	ATP5B
CLDN33	6E-113	0.840583	0.395	0.098	9.5E-109	Saliva_Acir	CLDN3
MGAT52	2.9E-112	0.355449	0.208	0.032	4.6E-108	Saliva_Acir	MGAT5
LGALS3BP4	4E-111	0.695514	0.548	0.169	6.4E-107	Saliva_Acir	LGALS3BP
PTN1	6.3E-111	0.470002	0.319	0.068	1E-106	Saliva_Acir	PTN
CD816	8.7E-111	0.689489	0.8	0.321	1.4E-106	Saliva_Acir	CD81
KLHL231	8.8E-111	0.274475	0.149	0.018	1.4E-106	Saliva_Acir	KLHL23
NNT.AS1	1.4E-110	0.310379	0.202	0.031	2.3E-106	Saliva_Acir	NNT.AS1
FXYD33	1.9E-110	0.632408	0.69	0.23	3E-106	Saliva_Acir	FXYD3
POLR2J37	3.1E-110	0.569951	0.384	0.096	5E-106	Saliva_Acir	POLR2J3
CLSTN13	7.9E-110	0.666613	0.433	0.121	1.3E-105	Saliva_Acir	CLSTN1
ATP5G35	1.8E-109	1.049213	0.773	0.36	2.9E-105	Saliva_Acir	ATP5G3
KREMEN11	3.1E-109	0.343491	0.251	0.046	5E-105	Saliva_Acir	KREMEN1
CUEDC12	5.3E-109	0.373839	0.198	0.03	8.5E-105	Saliva_Acir	CUEDC1
ABCA7	5.8E-109	0.261572	0.172	0.023	9.2E-105	Saliva_Acir	ABCA7
FGFR21	6.5E-109	0.382633	0.219	0.037	1E-104	Saliva_Acir	FGFR2
MARC21	2.2E-108	0.320443	0.178	0.025	3.5E-104	Saliva_Acir	MARC2
PPP1R9A2	1.1E-107	0.519975	0.257	0.05	1.7E-103	Saliva_Acir	PPP1R9A
TRIM29	2.3E-107	0.645212	0.391	0.099	3.6E-103	Saliva_Acir	TRIM29
ENOSF11	7.9E-107	0.427721	0.297	0.063	1.3E-102	Saliva_Acir	ENOSF1
TSTD14	8.1E-107	0.869512	0.696	0.27	1.3E-102	Saliva_Acir	TSTD1
PKP43	1.4E-105	0.543715	0.327	0.077	2.3E-101	Saliva_Acir	PKP4
ANXA32	3.7E-105	0.95472	0.486	0.153	5.9E-101	Saliva_Acir	ANXA3
ACOT111	2.4E-104	0.330372	0.189	0.029	3.8E-100	Saliva_Acir	ACOT11
FXYD64	4.3E-104	0.277157	0.191	0.028	6.9E-100	Saliva_Acir	FXYD6
MGST13	1.3E-103	0.984818	0.56	0.192	2E-99	Saliva_Acir	MGST1
TMPRSS13	6.2E-103	0.310186	0.164	0.023	9.9E-99	Saliva_Acir	TMPRSS13
CP2	1.5E-102	0.307661	0.18	0.026	2.44E-98	Saliva_Acir	CP
GMDS3	2.4E-102	0.922866	0.507	0.169	3.9E-98	Saliva_Acir	GMDS
RBBP8	2.7E-102	0.452162	0.263	0.052	4.31E-98	Saliva_Acir	RBBP8
FAM3B1	3E-102	0.511448	0.319	0.072	4.73E-98	Saliva_Acir	FAM3B
IVNS1ABP4	4.9E-102	0.850969	0.467	0.148	7.86E-98	Saliva_Acir	IVNS1ABP
MTR2	6.6E-102	0.356426	0.221	0.038	1.05E-97	Saliva_Acir	MTR
PPP1R14B1	9.3E-102	0.747637	0.531	0.176	1.49E-97	Saliva_Acir	PPP1R14B
NIPAL1	1.1E-101	0.255796	0.123	0.013	1.81E-97	Saliva_Acir	NIPAL1
CEP682	3.8E-101	0.357059	0.231	0.041	6E-97	Saliva_Acir	CEP68
SYNJ2BP2	7.6E-101	0.47927	0.306	0.069	1.21E-96	Saliva_Acir	SYNJ2BP
ARHGAP51	4.1E-100	0.880197	0.554	0.203	6.55E-96	Saliva_Acir	ARHGAP5
CDH12	2E-99	0.514062	0.314	0.074	3.21E-95	Saliva_Acir	CDH1
METTL7A3	2.5E-99	0.511334	0.355	0.089	4.05E-95	Saliva_Acir	METTL7A

SH3RF11	1.08E-98	0.444152	0.261	0.053	1.73E-94	Saliva_Acir	SH3RF1
AUTS21	1.2E-98	0.45686	0.27	0.057	1.92E-94	Saliva_Acir	AUTS2
CHD97	1.28E-98	0.76218	0.501	0.165	2.04E-94	Saliva_Acir	CHD9
GNA113	3.97E-98	0.5535	0.297	0.067	6.33E-94	Saliva_Acir	GNA11
PM20D2	5.09E-98	0.35931	0.223	0.04	8.12E-94	Saliva_Acir	PM20D2
TP63	9.89E-98	0.26476	0.164	0.023	1.58E-93	Saliva_Acir	TP63
ZNF7032	1.54E-97	0.263662	0.234	0.043	2.46E-93	Saliva_Acir	ZNF703
CLMN2	2.38E-97	0.434983	0.293	0.065	3.8E-93	Saliva_Acir	CLMN
LGR41	2.55E-97	0.336816	0.206	0.035	4.06E-93	Saliva_Acir	LGR4
AFG3L2	5.03E-97	0.501771	0.301	0.069	8.03E-93	Saliva_Acir	AFG3L2
TMPRSS3	6.37E-97	0.329043	0.108	0.011	1.02E-92	Saliva_Acir	TMPRSS3
VGLL43	1.11E-96	0.6882	0.439	0.134	1.78E-92	Saliva_Acir	VGLL4
ITPR25	1.44E-96	0.930371	0.321	0.08	2.29E-92	Saliva_Acir	ITPR2
RARRES22	1.61E-96	1.842801	0.344	0.087	2.57E-92	Saliva_Acir	RARRES2
TNS31	1.62E-96	0.317764	0.185	0.03	2.58E-92	Saliva_Acir	TNS3
C9orf32	1.96E-96	0.612689	0.371	0.097	3.13E-92	Saliva_Acir	C9orf3
TET11	2.89E-96	0.307763	0.178	0.027	4.61E-92	Saliva_Acir	TET1
ZBTB202	1.55E-95	0.671691	0.44	0.133	2.48E-91	Saliva_Acir	ZBTB20
LRPPRC	2.57E-95	0.474255	0.302	0.07	4.1E-91	Saliva_Acir	LRPPRC
PTGFRN1	5.1E-95	0.301621	0.219	0.04	8.13E-91	Saliva_Acir	PTGFRN
DAG11	6.32E-95	0.398996	0.278	0.061	1.01E-90	Saliva_Acir	DAG1
PTPN141	2.07E-94	0.455704	0.265	0.056	3.31E-90	Saliva_Acir	PTPN14
CCND13	4.36E-94	0.683381	0.41	0.118	6.95E-90	Saliva_Acir	CCND1
ALCAM2	4.44E-94	0.650682	0.359	0.095	7.09E-90	Saliva_Acir	ALCAM
FLNB2	6.77E-94	0.481004	0.318	0.078	1.08E-89	Saliva_Acir	FLNB
PLXDC23	3.09E-93	0.267203	0.231	0.043	4.92E-89	Saliva_Acir	PLXDC2
CAMK1D2	4.22E-93	0.300557	0.221	0.04	6.74E-89	Saliva_Acir	CAMK1D
SCGB3A12	9.14E-93	0.73251	0.474	0.139	1.46E-88	Saliva_Acir	SCGB3A1
ZNF4621	1.16E-92	0.410754	0.251	0.053	1.85E-88	Saliva_Acir	ZNF462
TRAK13	2.22E-92	0.442816	0.284	0.065	3.54E-88	Saliva_Acir	TRAK1
RRAGD2	2.88E-91	0.578643	0.34	0.091	4.59E-87	Saliva_Acir	RRAGD
TPD52L14	3.19E-91	0.625713	0.439	0.134	5.09E-87	Saliva_Acir	TPD52L1
NT5DC11	5.11E-91	0.470187	0.318	0.079	8.15E-87	Saliva_Acir	NT5DC1
FASTKD1	1.1E-90	0.265662	0.142	0.019	1.75E-86	Saliva_Acir	FASTKD1
TMEM245:	2.62E-90	0.461064	0.293	0.069	4.17E-86	Saliva_Acir	TMEM245
KRT182	2.84E-90	0.465327	0.76	0.301	4.52E-86	Saliva_Acir	KRT18
GNPTAB2	4.08E-90	0.56361	0.335	0.088	6.51E-86	Saliva_Acir	GNPTAB
DDX177	5.62E-90	0.906746	0.722	0.352	8.96E-86	Saliva_Acir	DDX17
LAMB23	1.07E-89	0.401484	0.331	0.082	1.71E-85	Saliva_Acir	LAMB2
SLITRK62	2.81E-89	0.551604	0.261	0.058	4.48E-85	Saliva_Acir	SLITRK6
APPL14	7.35E-89	0.555732	0.406	0.122	1.17E-84	Saliva_Acir	APPL1
APLP23	2.35E-88	0.874758	0.681	0.302	3.76E-84	Saliva_Acir	APLP2
EPB41L11	3.06E-88	0.263902	0.18	0.03	4.89E-84	Saliva_Acir	EPB41L1
ARHGEF28	3.87E-88	0.458462	0.248	0.054	6.17E-84	Saliva_Acir	ARHGEF28
FGD41	5.96E-88	0.433768	0.291	0.069	9.51E-84	Saliva_Acir	FGD4
LXN2	1.86E-87	0.446026	0.253	0.055	2.96E-83	Saliva_Acir	LXN
CHDH1	3.69E-87	0.35575	0.206	0.039	5.89E-83	Saliva_Acir	CHDH

VPS365	2.4E-86	0.539116	0.382	0.112	3.83E-82	Saliva_Acir	VPS36
BOD1L16	2.67E-86	0.554088	0.414	0.127	4.26E-82	Saliva_Acir	BOD1L1
SH3PXD2A	8.42E-86	0.400821	0.327	0.083	1.34E-81	Saliva_Acir	SH3PXD2A
EHF2	1.28E-85	0.739504	0.614	0.242	2.03E-81	Saliva_Acir	EHF
VPS13C5	1.62E-85	0.507691	0.342	0.092	2.59E-81	Saliva_Acir	VPS13C
PEBP15	3.38E-85	0.865618	0.853	0.454	5.38E-81	Saliva_Acir	PEBP1
SLC25A5	4.04E-85	0.86907	0.756	0.367	6.44E-81	Saliva_Acir	SLC25A5
BCL11A	6E-85	0.32624	0.219	0.044	9.57E-81	Saliva_Acir	BCL11A
HOMER21	2.27E-84	0.400482	0.233	0.049	3.62E-80	Saliva_Acir	HOMER2
GATAD14	2.83E-84	0.416558	0.301	0.075	4.51E-80	Saliva_Acir	GATAD1
ARL4C1	3.71E-84	0.576927	0.535	0.187	5.91E-80	Saliva_Acir	ARL4C
SHPRH4	5.17E-84	0.472913	0.285	0.069	8.24E-80	Saliva_Acir	SHPRH
WLS2	5.33E-84	0.398277	0.216	0.042	8.51E-80	Saliva_Acir	WLS
COX5B1	7.4E-84	0.868414	0.898	0.564	1.18E-79	Saliva_Acir	COX5B
HEXDC2	1E-83	0.294856	0.202	0.038	1.59E-79	Saliva_Acir	HEXDC
PDE7A1	1.36E-83	0.331562	0.238	0.05	2.17E-79	Saliva_Acir	PDE7A
DLD	1.49E-83	0.4497	0.318	0.083	2.37E-79	Saliva_Acir	DLD
N4BP2L27	3.96E-83	0.854182	0.656	0.292	6.32E-79	Saliva_Acir	N4BP2L2
UQCRB1	4.33E-83	0.866713	0.887	0.556	6.9E-79	Saliva_Acir	UQCRB
ATP5A12	5.63E-83	0.754338	0.69	0.311	8.98E-79	Saliva_Acir	ATP5A1
GOT1	5.91E-83	0.484434	0.295	0.075	9.43E-79	Saliva_Acir	GOT1
PHYHD12	9.6E-83	0.419944	0.257	0.059	1.53E-78	Saliva_Acir	PHYHD1
ASH1L3	1.39E-82	0.691122	0.478	0.168	2.21E-78	Saliva_Acir	ASH1L
PTER	9.22E-82	0.321945	0.212	0.042	1.47E-77	Saliva_Acir	PTER
GK53	1.1E-81	0.430198	0.289	0.073	1.76E-77	Saliva_Acir	GK5
PDLIM34	2.13E-81	0.484285	0.253	0.057	3.39E-77	Saliva_Acir	PDLIM3
KIAA11094	2.25E-81	0.460671	0.318	0.085	3.59E-77	Saliva_Acir	KIAA1109
NFIC5	2.33E-81	0.627571	0.484	0.17	3.72E-77	Saliva_Acir	NFIC
SOX4	3.29E-81	0.812073	0.62	0.257	5.24E-77	Saliva_Acir	SOX4
PURA5	3.82E-81	0.550215	0.397	0.124	6.09E-77	Saliva_Acir	PURA
COX7B3	3.87E-81	0.934067	0.749	0.407	6.18E-77	Saliva_Acir	COX7B
VEGFB6	9.27E-81	0.468485	0.348	0.098	1.48E-76	Saliva_Acir	VEGFB
SPINT23	1.71E-80	0.693116	0.722	0.311	2.73E-76	Saliva_Acir	SPINT2
SLC5A31	6.74E-80	0.287405	0.2	0.039	1.08E-75	Saliva_Acir	SLC5A3
TRIP116	7.15E-80	0.610003	0.384	0.119	1.14E-75	Saliva_Acir	TRIP11
LUC7L24	1.03E-79	0.38191	0.233	0.051	1.64E-75	Saliva_Acir	LUC7L2
MDH1	1.55E-79	0.642251	0.51	0.189	2.47E-75	Saliva_Acir	MDH1
RP11.532F	1.77E-79	0.540185	0.299	0.078	2.82E-75	Saliva_Acir	RP11.532F12.5
DDI21	2.14E-79	0.332276	0.206	0.041	3.41E-75	Saliva_Acir	DDI2
MARVELD2	2.73E-79	0.260766	0.13	0.018	4.35E-75	Saliva_Acir	MARVELD2
H3F3A8	3.06E-79	0.81847	0.898	0.477	4.87E-75	Saliva_Acir	H3F3A
RYK3	8.95E-79	0.424477	0.289	0.074	1.43E-74	Saliva_Acir	RYK
TNRC6B4	1.06E-78	0.592084	0.48	0.171	1.69E-74	Saliva_Acir	TNRC6B
NUCKS13	1.27E-78	0.726345	0.645	0.282	2.02E-74	Saliva_Acir	NUCKS1
DYNC1H14	2.26E-78	0.600319	0.418	0.138	3.61E-74	Saliva_Acir	DYNC1H1
ATP9A1	3.22E-78	0.346416	0.198	0.039	5.14E-74	Saliva_Acir	ATP9A
ZNF2923	4.68E-78	0.681029	0.471	0.171	7.46E-74	Saliva_Acir	ZNF292

LGALS31	1.63E-77	0.82971	0.921	0.569	2.6E-73	Saliva_Acir	LGALS3
KMT2A6	1.98E-77	0.525951	0.405	0.129	3.15E-73	Saliva_Acir	KMT2A
PRKDC4	2.08E-77	0.480836	0.34	0.097	3.32E-73	Saliva_Acir	PRKDC
HMGN32	3.49E-77	0.708215	0.635	0.277	5.57E-73	Saliva_Acir	HMGN3
PRPSAP12	4.99E-77	0.551437	0.376	0.118	7.95E-73	Saliva_Acir	PRPSAP1
PRDX51	6.1E-77	0.82829	0.849	0.489	9.73E-73	Saliva_Acir	PRDX5
NDUFA44	6.96E-77	0.777568	0.902	0.536	1.11E-72	Saliva_Acir	NDUFA4
SERTAD4.A	1.06E-76	0.356977	0.219	0.047	1.69E-72	Saliva_Acir	SERTAD4.AS1
MPZL2	2.7E-76	0.431316	0.34	0.096	4.31E-72	Saliva_Acir	MPZL2
MAVS3	3.42E-76	0.319002	0.229	0.051	5.45E-72	Saliva_Acir	MAVS
TBC1D15	4.18E-76	0.39447	0.248	0.057	6.66E-72	Saliva_Acir	TBC1D1
MBTPS14	4.2E-76	0.455769	0.31	0.085	6.7E-72	Saliva_Acir	MBTPS1
APP3	8.39E-76	0.672762	0.586	0.242	1.34E-71	Saliva_Acir	APP
NBEAL2	1.14E-75	0.28262	0.18	0.033	1.81E-71	Saliva_Acir	NBEAL2
PHF34	1.49E-75	0.615635	0.399	0.131	2.37E-71	Saliva_Acir	PHF3
PPP1R16A3	1.7E-75	0.528152	0.346	0.103	2.72E-71	Saliva_Acir	PPP1R16A
HADHB	2.3E-75	0.718942	0.48	0.18	3.66E-71	Saliva_Acir	HADHB
PGRMC11	3.44E-75	0.572843	0.463	0.165	5.49E-71	Saliva_Acir	PGRMC1
ZNF3261	3.7E-75	0.383783	0.312	0.085	5.9E-71	Saliva_Acir	ZNF326
SLFN53	6.69E-75	0.370367	0.28	0.07	1.07E-70	Saliva_Acir	SLFN5
CTC.444N2	9.74E-75	0.279056	0.214	0.045	1.55E-70	Saliva_Acir	CTC.444N24.11
LRIG11	1.75E-74	0.301303	0.202	0.042	2.79E-70	Saliva_Acir	LRIG1
IARS23	2.26E-74	0.433771	0.318	0.09	3.61E-70	Saliva_Acir	IARS2
MUC1	4.94E-74	0.539898	0.543	0.205	7.87E-70	Saliva_Acir	MUC1
LARS3	5.99E-74	0.492974	0.365	0.113	9.55E-70	Saliva_Acir	LARS
MUM11	6.12E-74	0.510004	0.314	0.087	9.77E-70	Saliva_Acir	MUM1
ZFPM11	7.51E-74	0.275387	0.155	0.026	1.2E-69	Saliva_Acir	ZFPM1
SLC1A52	8.49E-74	0.437855	0.287	0.076	1.35E-69	Saliva_Acir	SLC1A5
FAM73A1	8.83E-74	0.34418	0.24	0.056	1.41E-69	Saliva_Acir	FAM73A
NUDT33	1.69E-73	0.437051	0.346	0.101	2.69E-69	Saliva_Acir	NUDT3
ANKH2	2.24E-73	0.255803	0.185	0.035	3.58E-69	Saliva_Acir	ANKH
REPS2	2.8E-73	0.260673	0.149	0.025	4.46E-69	Saliva_Acir	REPS2
FAM60A	3.99E-73	0.480437	0.35	0.105	6.36E-69	Saliva_Acir	FAM60A
PPTC7	4.3E-73	0.298593	0.259	0.063	6.85E-69	Saliva_Acir	PPTC7
ZFP143	1.25E-72	0.261241	0.17	0.031	2E-68	Saliva_Acir	ZFP14
ZC3H62	2.12E-72	0.276865	0.274	0.07	3.38E-68	Saliva_Acir	ZC3H6
GTF3C4	5.64E-72	0.270332	0.17	0.032	8.99E-68	Saliva_Acir	GTF3C4
ARSD1	1.69E-71	0.342777	0.24	0.058	2.7E-67	Saliva_Acir	ARSD
RP11.553L	1.72E-71	0.293007	0.227	0.051	2.74E-67	Saliva_Acir	RP11.553L6.5
RAD503	2.05E-71	0.42802	0.308	0.087	3.27E-67	Saliva_Acir	RAD50
PRRC2B2	2.4E-71	0.378106	0.31	0.087	3.83E-67	Saliva_Acir	PRRC2B
FOXP4	5.82E-71	0.268928	0.144	0.024	9.28E-67	Saliva_Acir	FOXP4
AHNAK21	6.88E-71	0.35306	0.259	0.064	1.1E-66	Saliva_Acir	AHNAK2
MSLN1	7.91E-71	0.40434	0.178	0.034	1.26E-66	Saliva_Acir	MSLN
SESTD11	8.78E-71	0.467359	0.308	0.087	1.4E-66	Saliva_Acir	SESTD1
WDR62	8.96E-71	0.312161	0.268	0.069	1.43E-66	Saliva_Acir	WDR6
CRIM12	9.4E-71	0.429199	0.289	0.078	1.5E-66	Saliva_Acir	CRIM1

INSR2	1.38E-70	0.467963	0.391	0.129	2.19E-66	Saliva_Acir	INSR
TMEM982	2.8E-70	0.374889	0.284	0.075	4.47E-66	Saliva_Acir	TMEM98
PLEKHA51	2.94E-70	0.4536	0.301	0.085	4.69E-66	Saliva_Acir	PLEKHA5
SMAD42	4.18E-70	0.372506	0.257	0.065	6.66E-66	Saliva_Acir	SMAD4
CLUAP11	7.11E-70	0.347028	0.244	0.059	1.13E-65	Saliva_Acir	CLUAP1
OCLN	8.59E-70	0.328055	0.187	0.038	1.37E-65	Saliva_Acir	OCLN
ZZZ3	1.12E-69	0.317871	0.198	0.042	1.79E-65	Saliva_Acir	ZZZ3
BPTF4	1.91E-69	0.580732	0.444	0.162	3.04E-65	Saliva_Acir	BPTF
RALGAPA1	2.17E-69	0.371288	0.278	0.074	3.46E-65	Saliva_Acir	RALGAPA1
GOLGB15	2.67E-69	0.669413	0.499	0.198	4.26E-65	Saliva_Acir	GOLGB1
ST6GAL14	3.18E-69	0.255716	0.189	0.038	5.07E-65	Saliva_Acir	ST6GAL1
RCAN22	4.75E-69	0.506731	0.161	0.029	7.58E-65	Saliva_Acir	RCAN2
PCNXL44	7.2E-69	0.394012	0.24	0.059	1.15E-64	Saliva_Acir	PCNXL4
CTBP21	7.93E-69	0.435596	0.335	0.102	1.26E-64	Saliva_Acir	CTBP2
IRX33	8.18E-69	0.423541	0.268	0.072	1.3E-64	Saliva_Acir	IRX3
VEZF12	9.84E-69	0.433336	0.302	0.086	1.57E-64	Saliva_Acir	VEZF1
RBM41	1.05E-68	0.296843	0.176	0.035	1.67E-64	Saliva_Acir	RBM41
CENPC4	1.15E-68	0.346285	0.248	0.061	1.84E-64	Saliva_Acir	CENPC
MIB11	1.22E-68	0.363419	0.255	0.065	1.94E-64	Saliva_Acir	MIB1
HOOK11	2.02E-68	0.322158	0.301	0.084	3.22E-64	Saliva_Acir	HOOK1
ZNF7043	5.15E-68	0.396366	0.261	0.068	8.22E-64	Saliva_Acir	ZNF704
MKLN13	5.32E-68	0.530517	0.344	0.109	8.48E-64	Saliva_Acir	MKLN1
MACF14	7.49E-68	0.532428	0.45	0.167	1.19E-63	Saliva_Acir	MACF1
UQCRH1	7.76E-68	0.739719	0.809	0.477	1.24E-63	Saliva_Acir	UQCRH
ZSCAN182	8.87E-68	0.346185	0.289	0.079	1.42E-63	Saliva_Acir	ZSCAN18
GMPR3	9.27E-68	0.370225	0.2	0.044	1.48E-63	Saliva_Acir	GMPR
NDUFB75	1.09E-67	0.642158	0.733	0.371	1.73E-63	Saliva_Acir	NDUFB7
KIAA15221	1.3E-67	0.440047	0.259	0.069	2.07E-63	Saliva_Acir	KIAA1522
ZNF37A6	1.56E-67	0.309573	0.231	0.055	2.49E-63	Saliva_Acir	ZNF37A
DICER14	4.3E-67	0.438114	0.333	0.103	6.86E-63	Saliva_Acir	DICER1
RABGAP11	5.36E-67	0.426388	0.284	0.08	8.55E-63	Saliva_Acir	RABGAP1
BMPR1A1	5.45E-67	0.274522	0.168	0.033	8.7E-63	Saliva_Acir	BMPR1A
ANKRD171	1.02E-66	0.403704	0.338	0.105	1.63E-62	Saliva_Acir	ANKRD17
ATP1B3	1.15E-66	0.652551	0.558	0.235	1.83E-62	Saliva_Acir	ATP1B3
ZNF6383	1.99E-66	0.4963	0.384	0.129	3.17E-62	Saliva_Acir	ZNF638
AKAP94	2.35E-66	0.678273	0.595	0.269	3.74E-62	Saliva_Acir	AKAP9
CTSL3	2.94E-66	0.384682	0.34	0.103	4.69E-62	Saliva_Acir	CTSL
TMC42	4.25E-66	0.433528	0.282	0.08	6.78E-62	Saliva_Acir	TMC4
ZNHIT62	4.6E-66	0.279457	0.174	0.035	7.34E-62	Saliva_Acir	ZNHIT6
NBEA2	4.71E-66	0.401961	0.206	0.047	7.51E-62	Saliva_Acir	NBEA
NKTR7	5.02E-66	0.471991	0.467	0.174	8E-62	Saliva_Acir	NKTR
KCTD1	6.11E-66	0.262288	0.161	0.03	9.74E-62	Saliva_Acir	KCTD1
DGCR2	7.24E-66	0.279348	0.219	0.052	1.15E-61	Saliva_Acir	DGCR2
CENPB1	7.32E-66	0.257665	0.208	0.047	1.17E-61	Saliva_Acir	CENPB
HS3ST1	1.16E-65	0.346261	0.227	0.054	1.86E-61	Saliva_Acir	HS3ST1
SRSF104	1.43E-65	0.52546	0.427	0.156	2.28E-61	Saliva_Acir	SRSF10
SLC25A363	1.7E-65	0.493	0.367	0.122	2.71E-61	Saliva_Acir	SLC25A36

ETV6	2.43E-65	0.463624	0.34	0.109	3.87E-61	Saliva_Acir	ETV6
NAP1L41	2.86E-65	0.559232	0.439	0.164	4.55E-61	Saliva_Acir	NAP1L4
MGST37	3.08E-65	0.585261	0.722	0.333	4.92E-61	Saliva_Acir	MGST3
GBA2	3.29E-65	0.274002	0.176	0.036	5.24E-61	Saliva_Acir	GBA2
ROGDI	4.37E-65	0.310329	0.234	0.058	6.96E-61	Saliva_Acir	ROGDI
ATRX5	4.43E-65	0.604055	0.505	0.206	7.07E-61	Saliva_Acir	ATRX
ZNF254	4.89E-65	0.258958	0.172	0.034	7.8E-61	Saliva_Acir	ZNF254
TSHZ23	5.63E-65	0.341471	0.348	0.109	8.98E-61	Saliva_Acir	TSHZ2
MGAT4B1	5.88E-65	0.347176	0.278	0.078	9.39E-61	Saliva_Acir	MGAT4B
ZMYM4	6.95E-65	0.299958	0.231	0.057	1.11E-60	Saliva_Acir	ZMYM4
SGSM21	1.13E-64	0.282613	0.185	0.039	1.81E-60	Saliva_Acir	SGSM2
NHLRC21	1.26E-64	0.321433	0.189	0.041	2.01E-60	Saliva_Acir	NHLRC2
ZNF33B	1.34E-64	0.339605	0.223	0.055	2.13E-60	Saliva_Acir	ZNF33B
ATP6V1A	1.39E-64	0.596933	0.389	0.135	2.22E-60	Saliva_Acir	ATP6V1A
GCSH2	1.51E-64	0.538293	0.367	0.125	2.41E-60	Saliva_Acir	GCSH
RNF217	1.66E-64	0.301056	0.2	0.045	2.64E-60	Saliva_Acir	RNF217
PCM11	1.68E-64	0.460977	0.435	0.156	2.68E-60	Saliva_Acir	PCM1
SRFBP14	1.84E-64	0.364025	0.24	0.061	2.93E-60	Saliva_Acir	SRFBP1
ARHGAP21	2.02E-64	0.388439	0.314	0.094	3.23E-60	Saliva_Acir	ARHGAP21
ITGA21	2.6E-64	0.580578	0.297	0.088	4.14E-60	Saliva_Acir	ITGA2
DLG1	5.1E-64	0.434311	0.299	0.089	8.13E-60	Saliva_Acir	DLG1
TBC1D52	5.2E-64	0.319818	0.233	0.058	8.29E-60	Saliva_Acir	TBC1D5
OGDH	6.44E-64	0.331841	0.259	0.069	1.03E-59	Saliva_Acir	OGDH
RHOBTB34	6.71E-64	0.421052	0.359	0.117	1.07E-59	Saliva_Acir	RHOBTB3
PROM13	9.74E-64	0.486407	0.282	0.082	1.55E-59	Saliva_Acir	PROM1
ATP5D2	9.8E-64	0.647503	0.798	0.445	1.56E-59	Saliva_Acir	ATP5D
TOMM207	1.48E-63	0.692062	0.654	0.324	2.37E-59	Saliva_Acir	TOMM20
HSD17B12	2.15E-63	0.432011	0.344	0.112	3.43E-59	Saliva_Acir	HSD17B12
AGO22	3.52E-63	0.289639	0.216	0.051	5.62E-59	Saliva_Acir	AGO2
PHIP1	3.59E-63	0.477582	0.444	0.168	5.73E-59	Saliva_Acir	PHIP
MLH33	5.23E-63	0.286135	0.202	0.046	8.35E-59	Saliva_Acir	MLH3
SMARCA4	6.4E-63	0.446572	0.335	0.108	1.02E-58	Saliva_Acir	SMARCA4
CASD1	6.76E-63	0.269316	0.161	0.032	1.08E-58	Saliva_Acir	CASD1
KRR12	7.08E-63	0.464888	0.378	0.131	1.13E-58	Saliva_Acir	KRR1
SLC38A13	7.19E-63	0.39304	0.318	0.098	1.15E-58	Saliva_Acir	SLC38A1
PRDX25	8.47E-63	0.625554	0.656	0.31	1.35E-58	Saliva_Acir	PRDX2
CHD62	8.62E-63	0.397694	0.276	0.078	1.37E-58	Saliva_Acir	CHD6
ANKRD92	1.06E-62	0.429719	0.306	0.094	1.69E-58	Saliva_Acir	ANKRD9
TMTC31	1.48E-62	0.299813	0.176	0.037	2.36E-58	Saliva_Acir	TMTC3
FARP12	1.57E-62	0.478095	0.314	0.098	2.5E-58	Saliva_Acir	FARP1
UTRN4	1.85E-62	0.357738	0.357	0.115	2.94E-58	Saliva_Acir	UTRN
PRKX1	2.27E-62	0.2872	0.191	0.042	3.62E-58	Saliva_Acir	PRKX
EYA2	3.4E-62	0.250235	0.216	0.051	5.43E-58	Saliva_Acir	EYA2
NDUFB87	7.51E-62	0.527916	0.456	0.177	1.2E-57	Saliva_Acir	NDUFB8
TSC22D12	1.14E-61	0.433493	0.597	0.267	1.82E-57	Saliva_Acir	TSC22D1
MRPL351	1.24E-61	0.36243	0.219	0.055	1.98E-57	Saliva_Acir	MRPL35
RBM265	1.31E-61	0.321375	0.314	0.097	2.09E-57	Saliva_Acir	RBM26

ALDH24	1.37E-61	0.407285	0.363	0.123	2.19E-57	Saliva_Acir	ALDH2
CBR12	1.42E-61	0.546031	0.352	0.118	2.26E-57	Saliva_Acir	CBR1
PRRG4	2.1E-61	0.402352	0.274	0.079	3.35E-57	Saliva_Acir	PRRG4
NT5C3	2.29E-61	0.344023	0.372	0.124	3.65E-57	Saliva_Acir	NT5C
SCNN1B	2.37E-61	0.397877	0.338	0.105	3.78E-57	Saliva_Acir	SCNN1B
MDK1	2.38E-61	0.566049	0.405	0.148	3.79E-57	Saliva_Acir	MDK
MACC1	2.51E-61	0.456344	0.319	0.099	4.01E-57	Saliva_Acir	MACC1
ATL2	3.9E-61	0.402404	0.233	0.061	6.22E-57	Saliva_Acir	ATL2
SNHG81	4.84E-61	0.71425	0.618	0.295	7.72E-57	Saliva_Acir	SNHG8
ATP5J6	6.06E-61	0.705581	0.766	0.435	9.67E-57	Saliva_Acir	ATP5J
PDHA11	6.51E-61	0.434196	0.325	0.104	1.04E-56	Saliva_Acir	PDHA1
SPG111	8.5E-61	0.368434	0.255	0.071	1.36E-56	Saliva_Acir	SPG11
TFDP21	1.57E-60	0.306732	0.335	0.107	2.51E-56	Saliva_Acir	TFDP2
MRPL151	1.81E-60	0.397968	0.297	0.091	2.88E-56	Saliva_Acir	MRPL15
C14orf159	1.94E-60	0.256073	0.202	0.048	3.09E-56	Saliva_Acir	C14orf159
PUM12	2.26E-60	0.510998	0.388	0.142	3.61E-56	Saliva_Acir	PUM1
AP5M1	2.46E-60	0.415043	0.25	0.068	3.92E-56	Saliva_Acir	AP5M1
NPTN1	2.67E-60	0.398808	0.369	0.127	4.26E-56	Saliva_Acir	NPTN
BAZ2B2	2.76E-60	0.539318	0.399	0.146	4.4E-56	Saliva_Acir	BAZ2B
ILVBL	3.03E-60	0.353119	0.21	0.051	4.83E-56	Saliva_Acir	ILVBL
TFAP2A1	3.09E-60	0.302176	0.21	0.052	4.93E-56	Saliva_Acir	TFAP2A
CCAR13	3.14E-60	0.468745	0.321	0.103	5E-56	Saliva_Acir	CCAR1
GAMT	5.08E-60	0.376832	0.278	0.082	8.1E-56	Saliva_Acir	GAMT
EXPH5	9.16E-60	0.352713	0.253	0.069	1.46E-55	Saliva_Acir	EXPH5
LRBA2	9.92E-60	0.371598	0.24	0.065	1.58E-55	Saliva_Acir	LRBA
SMARCA22	2.11E-59	0.467766	0.336	0.113	3.37E-55	Saliva_Acir	SMARCA2
PTPRK	2.34E-59	0.367572	0.255	0.071	3.73E-55	Saliva_Acir	PTPRK
SSH3	2.66E-59	0.299264	0.18	0.04	4.24E-55	Saliva_Acir	SSH3
AHNAK6	3.26E-59	0.601875	0.716	0.384	5.19E-55	Saliva_Acir	AHNAK
ATP5G14	4.05E-59	0.581784	0.575	0.259	6.46E-55	Saliva_Acir	ATP5G1
CASZ11	4.15E-59	0.35459	0.234	0.063	6.63E-55	Saliva_Acir	CASZ1
GALNT51	4.92E-59	0.340598	0.198	0.047	7.85E-55	Saliva_Acir	GALNT5
NCOA12	5.87E-59	0.337377	0.291	0.088	9.37E-55	Saliva_Acir	NCOA1
NFAT52	8.37E-59	0.378057	0.369	0.127	1.34E-54	Saliva_Acir	NFAT5
PNN3	8.58E-59	0.531005	0.503	0.21	1.37E-54	Saliva_Acir	PNN
CLDN71	1.1E-58	0.596392	0.556	0.238	1.75E-54	Saliva_Acir	CLDN7
SNRNP200	1.14E-58	0.351125	0.318	0.102	1.81E-54	Saliva_Acir	SNRNP200
ATP7A	1.33E-58	0.369999	0.136	0.025	2.12E-54	Saliva_Acir	ATP7A
IFT811	1.34E-58	0.352498	0.178	0.039	2.14E-54	Saliva_Acir	IFT81
MED24	2.75E-58	0.25938	0.181	0.041	4.39E-54	Saliva_Acir	MED24
TAPBP5	2.81E-58	0.395633	0.433	0.163	4.48E-54	Saliva_Acir	TAPBP
DBT	2.85E-58	0.2622	0.161	0.033	4.55E-54	Saliva_Acir	DBT
FBXO91	3E-58	0.359579	0.302	0.095	4.78E-54	Saliva_Acir	FBXO9
SLC25A111	3.11E-58	0.406735	0.338	0.112	4.97E-54	Saliva_Acir	SLC25A11
PCBD13	3.29E-58	0.483038	0.414	0.157	5.25E-54	Saliva_Acir	PCBD1
SLC25A38	3.45E-58	0.695888	0.773	0.454	5.5E-54	Saliva_Acir	SLC25A3
RTP4	3.95E-58	0.300972	0.136	0.025	6.31E-54	Saliva_Acir	RTP4

LINC00657	4.03E-58	0.495543	0.482	0.202	6.43E-54	Saliva_Acir	LINC00657
PCYOX13	4.18E-58	0.433311	0.369	0.13	6.67E-54	Saliva_Acir	PCYOX1
UQCRC21	4.28E-58	0.50369	0.491	0.208	6.82E-54	Saliva_Acir	UQCRC2
NRIP12	5.68E-58	0.268313	0.204	0.05	9.05E-54	Saliva_Acir	NRIP1
EPN22	7.95E-58	0.349222	0.248	0.069	1.27E-53	Saliva_Acir	EPN2
SF3B3	9.18E-58	0.253419	0.225	0.058	1.46E-53	Saliva_Acir	SF3B3
PER31	1.24E-57	0.331371	0.242	0.066	1.98E-53	Saliva_Acir	PER3
RAB3IP1	1.52E-57	0.423741	0.276	0.082	2.42E-53	Saliva_Acir	RAB3IP
RCOR3	2.19E-57	0.319023	0.25	0.07	3.5E-53	Saliva_Acir	RCOR3
DAAM11	2.37E-57	0.469577	0.433	0.168	3.78E-53	Saliva_Acir	DAAM1
FOXP12	2.41E-57	0.642296	0.507	0.217	3.84E-53	Saliva_Acir	FOXP1
LMAN15	2.58E-57	0.406412	0.476	0.192	4.12E-53	Saliva_Acir	LMAN1
IGF1R	2.99E-57	0.325401	0.257	0.074	4.76E-53	Saliva_Acir	IGF1R
SNTB1	3.84E-57	0.354176	0.134	0.025	6.13E-53	Saliva_Acir	SNTB1
CIPC1	3.94E-57	0.275098	0.149	0.03	6.29E-53	Saliva_Acir	CIPC
HSPA91	4.37E-57	0.495709	0.422	0.164	6.97E-53	Saliva_Acir	HSPA9
MTUS12	4.55E-57	0.488346	0.41	0.156	7.26E-53	Saliva_Acir	MTUS1
LLGL21	4.92E-57	0.534532	0.336	0.116	7.84E-53	Saliva_Acir	LLGL2
LATS11	8.15E-57	0.278122	0.183	0.042	1.3E-52	Saliva_Acir	LATS1
HYI3	2.34E-56	0.398759	0.28	0.086	3.74E-52	Saliva_Acir	HYI
NME36	2.49E-56	0.45247	0.499	0.208	3.98E-52	Saliva_Acir	NME3
THUMPD1	3.36E-56	0.355321	0.314	0.102	5.36E-52	Saliva_Acir	THUMPD1
TMEM237	3.84E-56	0.290428	0.181	0.042	6.12E-52	Saliva_Acir	TMEM237
MOB1B1	3.91E-56	0.266855	0.159	0.033	6.23E-52	Saliva_Acir	MOB1B
SERINC51	4.01E-56	0.321998	0.208	0.053	6.39E-52	Saliva_Acir	SERINC5
MBOAT2	4.78E-56	0.422249	0.219	0.057	7.62E-52	Saliva_Acir	MBOAT2
TET21	8.31E-56	0.299126	0.217	0.056	1.32E-51	Saliva_Acir	TET2
CD1515	1.14E-55	0.472897	0.52	0.219	1.82E-51	Saliva_Acir	CD151
NUMA13	1.2E-55	0.365446	0.346	0.12	1.92E-51	Saliva_Acir	NUMA1
EVA1C1	2.29E-55	0.300481	0.251	0.071	3.65E-51	Saliva_Acir	EVA1C
FGGY2	2.36E-55	0.38661	0.274	0.084	3.76E-51	Saliva_Acir	FGGY
CARS	2.43E-55	0.415174	0.289	0.091	3.88E-51	Saliva_Acir	CARS
CASK1	2.45E-55	0.252633	0.208	0.053	3.9E-51	Saliva_Acir	CASK
ARRDC2	3.45E-55	0.273637	0.206	0.051	5.51E-51	Saliva_Acir	ARRDC2
ALDH7A11	3.78E-55	0.355032	0.231	0.064	6.03E-51	Saliva_Acir	ALDH7A1
CCPG15	4.1E-55	0.507173	0.425	0.167	6.53E-51	Saliva_Acir	CCPG1
KIDINS220	5.09E-55	0.326399	0.312	0.101	8.12E-51	Saliva_Acir	KIDINS220
MRPS27	5.49E-55	0.273978	0.185	0.044	8.75E-51	Saliva_Acir	MRPS27
GOLGA44	6.23E-55	0.642948	0.52	0.234	9.94E-51	Saliva_Acir	GOLGA4
EPM2AIP1	6.45E-55	0.257536	0.251	0.072	1.03E-50	Saliva_Acir	EPM2AIP1
SYNGR11	7.44E-55	0.429203	0.378	0.141	1.19E-50	Saliva_Acir	SYNGR1
NUDT82	8.31E-55	0.299044	0.216	0.057	1.32E-50	Saliva_Acir	NUDT8
CBX51	9.22E-55	0.467813	0.327	0.112	1.47E-50	Saliva_Acir	CBX5
LARP11	9.92E-55	0.390184	0.335	0.115	1.58E-50	Saliva_Acir	LARP1
PNISR5	1.3E-54	0.615797	0.669	0.346	2.08E-50	Saliva_Acir	PNISR
AIG12	1.41E-54	0.408445	0.321	0.108	2.25E-50	Saliva_Acir	AIG1
CYP20A14	1.71E-54	0.285741	0.299	0.095	2.73E-50	Saliva_Acir	CYP20A1

UHMK12	2.11E-54	0.354876	0.285	0.09	3.37E-50	Saliva_Acir	UHMK1
RNPC3	2.55E-54	0.323406	0.204	0.052	4.07E-50	Saliva_Acir	RNPC3
LTBP32	2.72E-54	0.434452	0.395	0.15	4.34E-50	Saliva_Acir	LTBP3
TIA1	2.85E-54	0.326392	0.285	0.089	4.54E-50	Saliva_Acir	TIA1
PDZD8	3.06E-54	0.275481	0.242	0.067	4.89E-50	Saliva_Acir	PDZD8
ZMYND11	3.54E-54	0.360646	0.263	0.079	5.64E-50	Saliva_Acir	ZMYND11
TTC171	4.05E-54	0.349565	0.295	0.095	6.46E-50	Saliva_Acir	TTC17
CYC11	6.18E-54	0.510324	0.516	0.229	9.85E-50	Saliva_Acir	CYC1
NUDT41	7.24E-54	0.496858	0.393	0.152	1.15E-49	Saliva_Acir	NUDT4
SLC15A2	8.13E-54	0.306931	0.202	0.052	1.3E-49	Saliva_Acir	SLC15A2
ITGB8	8.54E-54	0.313931	0.406	0.15	1.36E-49	Saliva_Acir	ITGB8
TSPO4	1.06E-53	0.497949	0.809	0.464	1.7E-49	Saliva_Acir	TSPO
ESRRA	1.25E-53	0.392827	0.312	0.104	2E-49	Saliva_Acir	ESRRA
CISD11	1.39E-53	0.416048	0.372	0.136	2.21E-49	Saliva_Acir	CISD1
LONP22	1.53E-53	0.344887	0.278	0.087	2.44E-49	Saliva_Acir	LONP2
CCNB1IP1	2.22E-53	0.367517	0.301	0.099	3.53E-49	Saliva_Acir	CCNB1IP1
IRF6	2.35E-53	0.254258	0.272	0.082	3.76E-49	Saliva_Acir	IRF6
CLPTM1L3	2.4E-53	0.290565	0.287	0.091	3.82E-49	Saliva_Acir	CLPTM1L
CAV22	2.56E-53	0.277464	0.35	0.119	4.08E-49	Saliva_Acir	CAV2
TRIM446	2.59E-53	0.32739	0.284	0.089	4.13E-49	Saliva_Acir	TRIM44
ACO22	3.79E-53	0.319292	0.299	0.098	6.04E-49	Saliva_Acir	ACO2
AQP31	4.2E-53	0.358822	0.473	0.191	6.7E-49	Saliva_Acir	AQP3
LRIG32	4.55E-53	0.261021	0.195	0.049	7.26E-49	Saliva_Acir	LRIG3
POGZ1	6.81E-53	0.345185	0.28	0.088	1.09E-48	Saliva_Acir	POGZ
C8orf82	6.96E-53	0.374689	0.24	0.07	1.11E-48	Saliva_Acir	C8orf82
ENO1	8.22E-53	0.603506	0.733	0.41	1.31E-48	Saliva_Acir	ENO1
CHM3	1.15E-52	0.284705	0.176	0.042	1.84E-48	Saliva_Acir	CHM
CHD31	1.26E-52	0.328915	0.276	0.086	2.01E-48	Saliva_Acir	CHD3
RIF14	1.28E-52	0.340678	0.287	0.091	2.03E-48	Saliva_Acir	RIF1
TUG13	1.43E-52	0.412032	0.359	0.132	2.29E-48	Saliva_Acir	TUG1
SFT2D21	1.62E-52	0.417454	0.316	0.108	2.59E-48	Saliva_Acir	SFT2D2
PARVA1	1.75E-52	0.312573	0.217	0.059	2.79E-48	Saliva_Acir	PARVA
SOS12	1.84E-52	0.312235	0.219	0.059	2.94E-48	Saliva_Acir	SOS1
TRAK21	1.85E-52	0.288986	0.183	0.045	2.95E-48	Saliva_Acir	TRAK2
SCAF116	1.89E-52	0.503519	0.512	0.23	3.02E-48	Saliva_Acir	SCAF11
COQ9	2.43E-52	0.324728	0.244	0.072	3.88E-48	Saliva_Acir	COQ9
NFIA3	2.78E-52	0.378026	0.433	0.172	4.43E-48	Saliva_Acir	NFIA
DHX9	2.92E-52	0.327188	0.318	0.108	4.66E-48	Saliva_Acir	DHX9
UQCRC11	3.21E-52	0.485176	0.454	0.192	5.12E-48	Saliva_Acir	UQCRC1
PLA2G165	4.03E-52	0.441397	0.348	0.124	6.43E-48	Saliva_Acir	PLA2G16
BRWD1	4.04E-52	0.493794	0.397	0.156	6.44E-48	Saliva_Acir	BRWD1
FAM210B4	4.51E-52	0.303836	0.285	0.093	7.2E-48	Saliva_Acir	FAM210B
CTDSPL4	4.59E-52	0.344603	0.268	0.084	7.32E-48	Saliva_Acir	CTDSPL
LAPTM4B1	5.19E-52	0.361754	0.304	0.101	8.28E-48	Saliva_Acir	LAPTM4B
HADHA2	1.07E-51	0.494604	0.463	0.199	1.71E-47	Saliva_Acir	HADHA
PDGFC1	1.21E-51	0.31164	0.187	0.047	1.93E-47	Saliva_Acir	PDGFC
ZNF1484	1.28E-51	0.382885	0.344	0.126	2.05E-47	Saliva_Acir	ZNF148

SUCLG11	1.35E-51	0.454388	0.427	0.176	2.16E-47	Saliva_Acir	SUCLG1
S100A61	1.6E-51	0.387564	0.987	0.712	2.55E-47	Saliva_Acir	S100A6
MXD44	2.32E-51	0.357257	0.336	0.119	3.7E-47	Saliva_Acir	MXD4
PRKAG2	2.47E-51	0.285688	0.227	0.063	3.95E-47	Saliva_Acir	PRKAG2
FAM213A1	3.04E-51	0.471285	0.344	0.127	4.85E-47	Saliva_Acir	FAM213A
SLC2A4RG:	6.03E-51	0.32182	0.297	0.098	9.61E-47	Saliva_Acir	SLC2A4RG
PHC31	6.11E-51	0.266895	0.206	0.055	9.74E-47	Saliva_Acir	PHC3
TMEM168:	6.77E-51	0.259524	0.161	0.036	1.08E-46	Saliva_Acir	TMEM168
COX7C1	7.05E-51	0.581283	0.909	0.636	1.13E-46	Saliva_Acir	COX7C
SET2	8.5E-51	0.537164	0.614	0.309	1.36E-46	Saliva_Acir	SET
CTNNB1	1.09E-50	0.34021	0.425	0.171	1.74E-46	Saliva_Acir	CTNNB1
ZNF280D3	1.2E-50	0.372244	0.265	0.083	1.92E-46	Saliva_Acir	ZNF280D
SFRP12	1.28E-50	0.361099	0.291	0.095	2.05E-46	Saliva_Acir	SFRP1
TANC2	1.42E-50	0.308511	0.181	0.045	2.27E-46	Saliva_Acir	TANC2
EXOSC62	1.55E-50	0.256137	0.187	0.047	2.47E-46	Saliva_Acir	EXOSC6
ATP2A32	1.78E-50	0.300899	0.204	0.054	2.83E-46	Saliva_Acir	ATP2A3
CUL53	2.11E-50	0.320555	0.253	0.077	3.37E-46	Saliva_Acir	CUL5
SMG11	2.19E-50	0.373853	0.336	0.12	3.5E-46	Saliva_Acir	SMG1
HERC13	2.5E-50	0.253062	0.229	0.065	3.99E-46	Saliva_Acir	HERC1
MBD6	2.68E-50	0.277202	0.206	0.055	4.27E-46	Saliva_Acir	MBD6
PKM	3.36E-50	0.574781	0.692	0.372	5.36E-46	Saliva_Acir	PKM
TOP2B1	3.62E-50	0.28687	0.276	0.088	5.77E-46	Saliva_Acir	TOP2B
TMEM181:	4.3E-50	0.281478	0.231	0.067	6.85E-46	Saliva_Acir	TMEM181
NDUFB51	5.48E-50	0.507655	0.524	0.243	8.74E-46	Saliva_Acir	NDUFB5
STARD7	7.96E-50	0.284405	0.285	0.093	1.27E-45	Saliva_Acir	STARD7
KIF13A1	2.32E-49	0.274027	0.193	0.05	3.69E-45	Saliva_Acir	KIF13A
CEP95	2.66E-49	0.262809	0.191	0.05	4.25E-45	Saliva_Acir	CEP95
PTPN182	3.64E-49	0.365091	0.276	0.09	5.8E-45	Saliva_Acir	PTPN18
NDUFA101	5.06E-49	0.421308	0.433	0.183	8.06E-45	Saliva_Acir	NDUFA10
TMEM141:	5.14E-49	0.458762	0.401	0.164	8.2E-45	Saliva_Acir	TMEM141
TCEA32	5.59E-49	0.314206	0.297	0.099	8.92E-45	Saliva_Acir	TCEA3
NDUFS41	6E-49	0.524581	0.482	0.217	9.57E-45	Saliva_Acir	NDUFS4
PRDX31	8.12E-49	0.368318	0.393	0.155	1.29E-44	Saliva_Acir	PRDX3
NCOA72	9.51E-49	0.365223	0.312	0.107	1.52E-44	Saliva_Acir	NCOA7
ZNF431	1.3E-48	0.257577	0.217	0.061	2.07E-44	Saliva_Acir	ZNF431
CTNND13	1.4E-48	0.34074	0.291	0.099	2.24E-44	Saliva_Acir	CTNND1
ZNF7211	1.79E-48	0.275958	0.225	0.065	2.85E-44	Saliva_Acir	ZNF721
TLK11	2.17E-48	0.34197	0.293	0.099	3.47E-44	Saliva_Acir	TLK1
DHRS74	2.18E-48	0.522171	0.476	0.207	3.47E-44	Saliva_Acir	DHRS7
KDSR2	2.24E-48	0.323258	0.35	0.129	3.57E-44	Saliva_Acir	KDSR
TNFSF102	2.3E-48	0.250189	0.584	0.27	3.66E-44	Saliva_Acir	TNFSF10
NDUFAB15	2.71E-48	0.476122	0.524	0.245	4.32E-44	Saliva_Acir	NDUFAB1
PHF10	3.15E-48	0.319803	0.238	0.072	5.03E-44	Saliva_Acir	PHF10
APOBEC3C	3.63E-48	0.358543	0.2	0.053	5.79E-44	Saliva_Acir	APOBEC3C
SFXN41	4.09E-48	0.287841	0.198	0.054	6.52E-44	Saliva_Acir	SFXN4
RAB252	4.74E-48	0.424872	0.35	0.133	7.57E-44	Saliva_Acir	RAB25
FAM195A1	4.76E-48	0.376913	0.333	0.121	7.6E-44	Saliva_Acir	FAM195A

COMMD21	5.8E-48	0.303329	0.285	0.095	9.25E-44	Saliva_Acir	COMMD2
IL6ST2	6.63E-48	0.464896	0.49	0.221	1.06E-43	Saliva_Acir	IL6ST
SNRPN3	6.78E-48	0.440776	0.299	0.106	1.08E-43	Saliva_Acir	SNRPN
ACYP11	9.18E-48	0.251566	0.221	0.063	1.46E-43	Saliva_Acir	ACYP1
LUC7L34	1.01E-47	0.431731	0.558	0.265	1.62E-43	Saliva_Acir	LUC7L3
PRDX61	1.02E-47	0.614034	0.671	0.373	1.63E-43	Saliva_Acir	PRDX6
GLG13	1.19E-47	0.403547	0.338	0.126	1.9E-43	Saliva_Acir	GLG1
BIRC62	1.25E-47	0.371419	0.308	0.11	1.99E-43	Saliva_Acir	BIRC6
IPO71	1.29E-47	0.342335	0.299	0.103	2.06E-43	Saliva_Acir	IPO7
ANKRD26	1.57E-47	0.274031	0.178	0.045	2.51E-43	Saliva_Acir	ANKRD26
TPBG1	1.72E-47	0.271333	0.248	0.077	2.74E-43	Saliva_Acir	TPBG
ADD31	1.72E-47	0.325207	0.405	0.159	2.74E-43	Saliva_Acir	ADD3
MYO1D1	1.93E-47	0.251885	0.214	0.061	3.07E-43	Saliva_Acir	MYO1D
NIPBL2	2.88E-47	0.437671	0.376	0.15	4.59E-43	Saliva_Acir	NIPBL
MAST4	4.13E-47	0.397423	0.348	0.131	6.59E-43	Saliva_Acir	MAST4
DOCK7	4.57E-47	0.300972	0.18	0.047	7.29E-43	Saliva_Acir	DOCK7
SMC6	4.83E-47	0.351359	0.242	0.074	7.7E-43	Saliva_Acir	SMC6
SLC22A18	5.04E-47	0.267453	0.219	0.063	8.04E-43	Saliva_Acir	SLC22A18
XPR1	7.75E-47	0.289229	0.142	0.031	1.24E-42	Saliva_Acir	XPR1
PHKB5	2.67E-46	0.372729	0.297	0.105	4.26E-42	Saliva_Acir	PHKB
SETD2	4.32E-46	0.345509	0.255	0.083	6.9E-42	Saliva_Acir	SETD2
TMEM261	5.13E-46	0.32239	0.361	0.139	8.18E-42	Saliva_Acir	TMEM261
WWP11	6.31E-46	0.269806	0.251	0.081	1.01E-41	Saliva_Acir	WWP1
IVD1	6.52E-46	0.278214	0.185	0.05	1.04E-41	Saliva_Acir	IVD
MAGED24	6.59E-46	0.322326	0.389	0.154	1.05E-41	Saliva_Acir	MAGED2
SMAD53	6.72E-46	0.293172	0.276	0.093	1.07E-41	Saliva_Acir	SMAD5
UBE3A2	6.9E-46	0.362851	0.352	0.135	1.1E-41	Saliva_Acir	UBE3A
SDC4	7.66E-46	0.410907	0.406	0.165	1.22E-41	Saliva_Acir	SDC4
PLEKHA11	7.92E-46	0.358625	0.336	0.126	1.26E-41	Saliva_Acir	PLEKHA1
CASC43	9.19E-46	0.326427	0.327	0.121	1.47E-41	Saliva_Acir	CASC4
MAP3K23	1.39E-45	0.319167	0.333	0.124	2.21E-41	Saliva_Acir	MAP3K2
NF11	1.53E-45	0.311581	0.261	0.086	2.45E-41	Saliva_Acir	NF1
NDUFB106	1.64E-45	0.468891	0.699	0.383	2.61E-41	Saliva_Acir	NDUFB10
MDH21	2.06E-45	0.449406	0.522	0.251	3.28E-41	Saliva_Acir	MDH2
DNAJB142	2.69E-45	0.31178	0.293	0.102	4.29E-41	Saliva_Acir	DNAJB14
TTC145	2.88E-45	0.264585	0.293	0.101	4.6E-41	Saliva_Acir	TTC14
RPS6KA33	2.99E-45	0.324553	0.28	0.095	4.77E-41	Saliva_Acir	RPS6KA3
USP332	3.43E-45	0.382465	0.251	0.082	5.46E-41	Saliva_Acir	USP33
ANXA42	3.91E-45	0.321924	0.357	0.137	6.23E-41	Saliva_Acir	ANXA4
MAFB2	5E-45	0.258012	0.268	0.088	7.97E-41	Saliva_Acir	MAFB
ITGAV1	5.74E-45	0.335814	0.278	0.095	9.15E-41	Saliva_Acir	ITGAV
RBM255	5.79E-45	0.384518	0.516	0.238	9.24E-41	Saliva_Acir	RBM25
MFSD61	6.92E-45	0.32439	0.242	0.078	1.1E-40	Saliva_Acir	MFSD6
ATP5C12	7.82E-45	0.450467	0.565	0.28	1.25E-40	Saliva_Acir	ATP5C1
METTL52	8.44E-45	0.310206	0.325	0.12	1.35E-40	Saliva_Acir	METTL5
TRIM2	8.45E-45	0.411403	0.299	0.108	1.35E-40	Saliva_Acir	TRIM2
MLLT61	8.98E-45	0.275843	0.293	0.102	1.43E-40	Saliva_Acir	MLLT6

LRP61	9.97E-45	0.274199	0.198	0.056	1.59E-40	Saliva_Acir	LRP6
DDAH22	1.61E-44	0.372833	0.474	0.209	2.57E-40	Saliva_Acir	DDAH2
U2SURP4	1.74E-44	0.338055	0.408	0.171	2.77E-40	Saliva_Acir	U2SURP
UNC93B12	1.9E-44	0.263103	0.219	0.066	3.03E-40	Saliva_Acir	UNC93B1
ESD4	2.27E-44	0.434934	0.476	0.216	3.63E-40	Saliva_Acir	ESD
TTC191	2.97E-44	0.326497	0.323	0.121	4.74E-40	Saliva_Acir	TTC19
MSH3	2.97E-44	0.260393	0.163	0.041	4.74E-40	Saliva_Acir	MSH3
ZC3H133	3.31E-44	0.330479	0.325	0.121	5.28E-40	Saliva_Acir	ZC3H13
ZYG11B1	3.34E-44	0.260364	0.183	0.05	5.32E-40	Saliva_Acir	ZYG11B
NR2F63	3.41E-44	0.336424	0.25	0.083	5.45E-40	Saliva_Acir	NR2F6
RERE	3.75E-44	0.354794	0.344	0.134	5.99E-40	Saliva_Acir	RERE
CYB5A3	5.96E-44	0.43383	0.422	0.181	9.5E-40	Saliva_Acir	CYB5A
GAS64	7.33E-44	0.337639	0.278	0.095	1.17E-39	Saliva_Acir	GAS6
TEX2641	8.21E-44	0.382677	0.41	0.174	1.31E-39	Saliva_Acir	TEX264
HNRNPU.A	8.93E-44	0.299827	0.212	0.063	1.42E-39	Saliva_Acir	HNRNPU.AS1
ADI15	9.53E-44	0.453997	0.478	0.22	1.52E-39	Saliva_Acir	ADI1
DDRGK12	9.62E-44	0.306674	0.371	0.151	1.53E-39	Saliva_Acir	DDRGK1
PHB22	1.15E-43	0.307609	0.442	0.195	1.84E-39	Saliva_Acir	PHB2
RSF12	1.29E-43	0.397605	0.416	0.181	2.05E-39	Saliva_Acir	RSF1
ZNF6441	1.3E-43	0.352576	0.314	0.118	2.08E-39	Saliva_Acir	ZNF644
RASD12	1.34E-43	0.285616	0.242	0.077	2.13E-39	Saliva_Acir	RASD1
SENP64	1.43E-43	0.293092	0.299	0.108	2.27E-39	Saliva_Acir	SENP6
RNF134	1.87E-43	0.38819	0.395	0.166	2.98E-39	Saliva_Acir	RNF13
MYO1C3	2.64E-43	0.347833	0.361	0.145	4.2E-39	Saliva_Acir	MYO1C
ORC4	3.14E-43	0.281756	0.208	0.062	5.01E-39	Saliva_Acir	ORC4
CANX5	3.5E-43	0.470551	0.588	0.311	5.59E-39	Saliva_Acir	CANX
KIF13B2	5.7E-43	0.261107	0.187	0.053	9.09E-39	Saliva_Acir	KIF13B
SPTLC24	6.16E-43	0.285712	0.244	0.08	9.82E-39	Saliva_Acir	SPTLC2
INTS65	1.03E-42	0.431552	0.342	0.136	1.64E-38	Saliva_Acir	INTS6
ADH53	1.14E-42	0.340317	0.344	0.135	1.81E-38	Saliva_Acir	ADH5
ABI21	1.38E-42	0.254431	0.261	0.087	2.2E-38	Saliva_Acir	ABI2
ENPP4	1.5E-42	0.268282	0.202	0.059	2.39E-38	Saliva_Acir	ENPP4
NARS1	1.68E-42	0.330158	0.353	0.141	2.67E-38	Saliva_Acir	NARS
NR1D2	1.95E-42	0.387226	0.357	0.143	3.11E-38	Saliva_Acir	NR1D2
MAN2A12	3.68E-42	0.295289	0.189	0.054	5.86E-38	Saliva_Acir	MAN2A1
PALLD2	3.88E-42	0.431568	0.378	0.16	6.19E-38	Saliva_Acir	PALLD
TRAP1	4.63E-42	0.263263	0.216	0.066	7.38E-38	Saliva_Acir	TRAP1
HINT14	6.58E-42	0.540626	0.843	0.569	1.05E-37	Saliva_Acir	HINT1
EBP2	6.74E-42	0.360691	0.312	0.118	1.08E-37	Saliva_Acir	EBP
ATP2C1	1.14E-41	0.300279	0.229	0.073	1.82E-37	Saliva_Acir	ATP2C1
LMO45	1.15E-41	0.337946	0.388	0.164	1.83E-37	Saliva_Acir	LMO4
DYNC1LI22	1.46E-41	0.424811	0.374	0.162	2.32E-37	Saliva_Acir	DYNC1LI2
ATXN7L3B:	1.49E-41	0.311575	0.227	0.072	2.38E-37	Saliva_Acir	ATXN7L3B
PHF20L11	1.55E-41	0.35868	0.329	0.129	2.48E-37	Saliva_Acir	PHF20L1
STAU2	1.71E-41	0.283103	0.227	0.072	2.73E-37	Saliva_Acir	STAU2
IMPAD12	1.79E-41	0.296168	0.263	0.091	2.86E-37	Saliva_Acir	IMPAD1
EP3001	1.83E-41	0.310224	0.225	0.071	2.91E-37	Saliva_Acir	EP300

ECHDC22	2.48E-41	0.322799	0.34	0.136	3.95E-37	Saliva_Acir	ECHDC2
SBF21	2.84E-41	0.286405	0.21	0.065	4.53E-37	Saliva_Acir	SBF2
DDX11	3.18E-41	0.353189	0.285	0.104	5.07E-37	Saliva_Acir	DDX1
EXOC71	3.9E-41	0.335257	0.28	0.101	6.22E-37	Saliva_Acir	EXOC7
MSRB23	3.92E-41	0.305928	0.327	0.129	6.26E-37	Saliva_Acir	MSRB2
AHCYL12	4.65E-41	0.374974	0.38	0.164	7.42E-37	Saliva_Acir	AHCYL1
THOC23	5.35E-41	0.272047	0.325	0.125	8.53E-37	Saliva_Acir	THOC2
EBPL1	5.58E-41	0.292837	0.342	0.134	8.9E-37	Saliva_Acir	EBPL
NDUFS63	5.71E-41	0.462227	0.601	0.322	9.1E-37	Saliva_Acir	NDUFS6
DCAF71	6.96E-41	0.263999	0.261	0.091	1.11E-36	Saliva_Acir	DCAF7
ESF12	7.01E-41	0.2983	0.306	0.115	1.12E-36	Saliva_Acir	ESF1
RBMX	8.58E-41	0.274073	0.448	0.198	1.37E-36	Saliva_Acir	RBMX
RPL7L13	8.8E-41	0.259355	0.312	0.117	1.4E-36	Saliva_Acir	RPL7L1
SLC38A2	1.09E-40	0.307982	0.509	0.24	1.74E-36	Saliva_Acir	SLC38A2
SNX62	2.02E-40	0.399914	0.397	0.173	3.23E-36	Saliva_Acir	SNX6
MAL21	2.17E-40	0.376118	0.338	0.136	3.47E-36	Saliva_Acir	MAL2
PNKD1	2.46E-40	0.319115	0.431	0.193	3.92E-36	Saliva_Acir	PNKD
ILF31	2.46E-40	0.317801	0.338	0.134	3.93E-36	Saliva_Acir	ILF3
DECR11	2.58E-40	0.345042	0.38	0.162	4.11E-36	Saliva_Acir	DECR1
SMARCA53	2.7E-40	0.340612	0.327	0.129	4.31E-36	Saliva_Acir	SMARCA5
SPEN	3.09E-40	0.253535	0.285	0.104	4.92E-36	Saliva_Acir	SPEN
TRIM8	3.42E-40	0.261696	0.276	0.098	5.45E-36	Saliva_Acir	TRIM8
AES1	7.23E-40	0.294033	0.699	0.394	1.15E-35	Saliva_Acir	AES
SF12	8.39E-40	0.324685	0.49	0.233	1.34E-35	Saliva_Acir	SF1
VPS353	8.95E-40	0.374823	0.408	0.183	1.43E-35	Saliva_Acir	VPS35
FAM120A3	1.26E-39	0.359012	0.353	0.147	2.01E-35	Saliva_Acir	FAM120A
CXCL171	1.41E-39	0.354275	0.422	0.178	2.26E-35	Saliva_Acir	CXCL17
ZBTB44	1.56E-39	0.314721	0.282	0.103	2.49E-35	Saliva_Acir	ZBTB44
VPS13A1	2.08E-39	0.320619	0.221	0.071	3.31E-35	Saliva_Acir	VPS13A
STX161	2.14E-39	0.255429	0.244	0.083	3.41E-35	Saliva_Acir	STX16
APIP2	2.44E-39	0.341796	0.265	0.096	3.89E-35	Saliva_Acir	APIP
SREK11	2.69E-39	0.290283	0.336	0.135	4.28E-35	Saliva_Acir	SREK1
ISOC22	3.66E-39	0.340307	0.327	0.131	5.83E-35	Saliva_Acir	ISOC2
WDR341	4.32E-39	0.355347	0.251	0.088	6.89E-35	Saliva_Acir	WDR34
USP9X	5.1E-39	0.281385	0.287	0.107	8.13E-35	Saliva_Acir	USP9X
SCAPER1	5.3E-39	0.296691	0.21	0.066	8.46E-35	Saliva_Acir	SCAPER
SEPW14	5.74E-39	0.404589	0.647	0.358	9.16E-35	Saliva_Acir	SEPW1
DARS	7.54E-39	0.25958	0.355	0.146	1.2E-34	Saliva_Acir	DARS
MPC14	7.66E-39	0.460735	0.439	0.209	1.22E-34	Saliva_Acir	MPC1
CPNE32	7.75E-39	0.378152	0.422	0.191	1.24E-34	Saliva_Acir	CPNE3
CAPN1	1.02E-38	0.363863	0.435	0.199	1.62E-34	Saliva_Acir	CAPN1
ASS1	1.08E-38	0.31374	0.284	0.106	1.72E-34	Saliva_Acir	ASS1
NDUFC11	1.32E-38	0.43259	0.544	0.28	2.11E-34	Saliva_Acir	NDUFC1
ADSS	1.42E-38	0.277456	0.301	0.115	2.27E-34	Saliva_Acir	ADSS
TAOK12	1.44E-38	0.25089	0.293	0.11	2.3E-34	Saliva_Acir	TAOK1
BCLAF16	1.94E-38	0.368001	0.429	0.198	3.1E-34	Saliva_Acir	BCLAF1
OPA1	2.14E-38	0.318868	0.202	0.064	3.42E-34	Saliva_Acir	OPA1

TPR3	2.85E-38	0.278468	0.444	0.202	4.54E-34	Saliva_Acir	TPR
PON21	3.39E-38	0.274851	0.217	0.071	5.41E-34	Saliva_Acir	PON2
NDUFS82	3.86E-38	0.322189	0.535	0.263	6.15E-34	Saliva_Acir	NDUFS8
CCDC6	5.11E-38	0.339989	0.302	0.118	8.15E-34	Saliva_Acir	CCDC6
MDM44	5.51E-38	0.297278	0.335	0.137	8.8E-34	Saliva_Acir	MDM4
KCNQ1OT1	6.02E-38	0.371292	0.34	0.141	9.61E-34	Saliva_Acir	KCNQ1OT1
SEPT22	6.24E-38	0.392476	0.478	0.235	9.95E-34	Saliva_Acir	SEPT2
ARID1B	6.44E-38	0.310179	0.24	0.084	1.03E-33	Saliva_Acir	ARID1B
NDUFA9	7.5E-38	0.274627	0.344	0.141	1.2E-33	Saliva_Acir	NDUFA9
RPS244	1.05E-37	0.600732	0.968	0.839	1.68E-33	Saliva_Acir	RPS24
DNM1L2	1.16E-37	0.295841	0.233	0.079	1.86E-33	Saliva_Acir	DNM1L
KMT2C5	1.23E-37	0.28563	0.361	0.153	1.96E-33	Saliva_Acir	KMT2C
GSTP14	1.39E-37	0.397369	0.873	0.556	2.21E-33	Saliva_Acir	GSTP1
DDB11	1.75E-37	0.274572	0.295	0.113	2.78E-33	Saliva_Acir	DDB1
TGOLN27	2.27E-37	0.320799	0.388	0.171	3.62E-33	Saliva_Acir	TGOLN2
ASPH4	2.88E-37	0.355934	0.416	0.19	4.6E-33	Saliva_Acir	ASPH
NFE2L12	2.9E-37	0.272592	0.253	0.091	4.62E-33	Saliva_Acir	NFE2L1
TMEM205	3.59E-37	0.333504	0.442	0.204	5.72E-33	Saliva_Acir	TMEM205
PRPF40A4	3.95E-37	0.334075	0.41	0.187	6.29E-33	Saliva_Acir	PRPF40A
SNRPD32	4.27E-37	0.383292	0.471	0.231	6.8E-33	Saliva_Acir	SNRPD3
EPB41L4A.	4.8E-37	0.454264	0.423	0.198	7.66E-33	Saliva_Acir	EPB41L4A.AS1
NDUFA122	5.22E-37	0.395196	0.529	0.274	8.33E-33	Saliva_Acir	NDUFA12
OSBPL1A4	5.23E-37	0.351072	0.367	0.159	8.35E-33	Saliva_Acir	OSBPL1A
MAN1A22	5.37E-37	0.287682	0.289	0.113	8.56E-33	Saliva_Acir	MAN1A2
COX8A2	6.22E-37	0.493065	0.72	0.434	9.91E-33	Saliva_Acir	COX8A
EFCAB142	6.49E-37	0.293979	0.287	0.111	1.03E-32	Saliva_Acir	EFCAB14
SCARB25	6.96E-37	0.34319	0.363	0.156	1.11E-32	Saliva_Acir	SCARB2
NCOR11	1.05E-36	0.327366	0.414	0.189	1.67E-32	Saliva_Acir	NCOR1
IMMT1	1.2E-36	0.253573	0.268	0.099	1.91E-32	Saliva_Acir	IMMT
LPGAT12	1.29E-36	0.292292	0.248	0.089	2.05E-32	Saliva_Acir	LPGAT1
NDUFS31	1.32E-36	0.270853	0.41	0.187	2.1E-32	Saliva_Acir	NDUFS3
FAM208A1	1.46E-36	0.258732	0.198	0.063	2.33E-32	Saliva_Acir	FAM208A
RBM52	1.65E-36	0.335355	0.316	0.129	2.63E-32	Saliva_Acir	RBM5
SLTM3	2.02E-36	0.312631	0.42	0.194	3.22E-32	Saliva_Acir	SLTM
TUBA1C2	2.05E-36	0.39842	0.336	0.142	3.27E-32	Saliva_Acir	TUBA1C
NPY	2.14E-36	0.589418	0.038	0.004	3.42E-32	Saliva_Acir	NPY
TRPM41	2.42E-36	0.307214	0.193	0.061	3.86E-32	Saliva_Acir	TRPM4
MRPS332	3.33E-36	0.294846	0.346	0.148	5.32E-32	Saliva_Acir	MRPS33
MRPL432	5E-36	0.334175	0.412	0.191	7.97E-32	Saliva_Acir	MRPL43
PPIG5	5.4E-36	0.356622	0.497	0.249	8.6E-32	Saliva_Acir	PPIG
SELENBP15	5.95E-36	0.382396	0.219	0.075	9.48E-32	Saliva_Acir	SELENBP1
M6PR2	8.19E-36	0.261127	0.316	0.128	1.31E-31	Saliva_Acir	M6PR
SMAD22	8.31E-36	0.306595	0.31	0.126	1.33E-31	Saliva_Acir	SMAD2
LIMA13	1.17E-35	0.365175	0.401	0.181	1.87E-31	Saliva_Acir	LIMA1
TRMT111	1.21E-35	0.250593	0.144	0.038	1.93E-31	Saliva_Acir	TRMT11
KIAA20261	1.41E-35	0.280826	0.265	0.099	2.25E-31	Saliva_Acir	KIAA2026
DUSP232	1.43E-35	0.289319	0.353	0.152	2.28E-31	Saliva_Acir	DUSP23

MRPL4	1.54E-35	0.292279	0.299	0.119	2.45E-31	Saliva_Acir	MRPL4
MRPL412	1.66E-35	0.398716	0.571	0.308	2.64E-31	Saliva_Acir	MRPL41
LGALS8	2.45E-35	0.288591	0.278	0.106	3.91E-31	Saliva_Acir	LGALS8
HNRNPR3	2.46E-35	0.339416	0.416	0.192	3.92E-31	Saliva_Acir	HNRNPR
TMEM123	2.96E-35	0.38731	0.546	0.281	4.73E-31	Saliva_Acir	TMEM123
PRKCI1	3.06E-35	0.293748	0.25	0.092	4.88E-31	Saliva_Acir	PRKCI
DHCR242	3.46E-35	0.294865	0.259	0.098	5.52E-31	Saliva_Acir	DHCR24
UFL12	3.68E-35	0.285827	0.289	0.115	5.86E-31	Saliva_Acir	UFL1
NDUFB23	3.8E-35	0.374264	0.667	0.374	6.06E-31	Saliva_Acir	NDUFB2
SH3YL11	4.41E-35	0.440248	0.427	0.208	7.04E-31	Saliva_Acir	SH3YL1
NBN	5.35E-35	0.328712	0.212	0.072	8.53E-31	Saliva_Acir	NBN
CLCN32	5.57E-35	0.322469	0.312	0.129	8.89E-31	Saliva_Acir	CLCN3
EPRS6	5.86E-35	0.267386	0.319	0.132	9.35E-31	Saliva_Acir	EPRS
STK38	6.54E-35	0.250053	0.27	0.101	1.04E-30	Saliva_Acir	STK38
NDUFV12	7.42E-35	0.321068	0.38	0.172	1.18E-30	Saliva_Acir	NDUFV1
TNRC6A1	7.64E-35	0.257446	0.257	0.096	1.22E-30	Saliva_Acir	TNRC6A
PABPN12	7.67E-35	0.269216	0.348	0.15	1.22E-30	Saliva_Acir	PABPN1
KTN11	7.78E-35	0.515577	0.658	0.396	1.24E-30	Saliva_Acir	KTN1
BTBD71	1.34E-34	0.288305	0.259	0.098	2.13E-30	Saliva_Acir	BTBD7
SYNCRIP3	1.37E-34	0.337867	0.397	0.185	2.19E-30	Saliva_Acir	SYNCRIP
PFN21	1.58E-34	0.326076	0.229	0.081	2.52E-30	Saliva_Acir	PFN2
EEF1A14	3E-34	0.482812	0.992	0.915	4.78E-30	Saliva_Acir	EEF1A1
MRPL402	3.03E-34	0.323874	0.367	0.165	4.83E-30	Saliva_Acir	MRPL40
MBD23	3.87E-34	0.279074	0.325	0.138	6.17E-30	Saliva_Acir	MBD2
RFC14	5.19E-34	0.269376	0.285	0.114	8.28E-30	Saliva_Acir	RFC1
NDUFS52	6.13E-34	0.450217	0.735	0.454	9.77E-30	Saliva_Acir	NDUFS5
USP34	7.26E-34	0.337807	0.321	0.135	1.16E-29	Saliva_Acir	USP34
INTS11	7.82E-34	0.250722	0.176	0.055	1.25E-29	Saliva_Acir	INTS1
FAM162A1	8.44E-34	0.317985	0.406	0.193	1.35E-29	Saliva_Acir	FAM162A
KIAA03681	1.01E-33	0.277491	0.189	0.062	1.61E-29	Saliva_Acir	KIAA0368
CITED41	1.03E-33	0.34131	0.231	0.083	1.64E-29	Saliva_Acir	CITED4
TMEM14C	1.25E-33	0.35087	0.499	0.255	1.99E-29	Saliva_Acir	TMEM14C
COX7A14	1.44E-33	0.341957	0.327	0.138	2.29E-29	Saliva_Acir	COX7A1
FNBP1L	1.66E-33	0.308046	0.31	0.132	2.65E-29	Saliva_Acir	FNBP1L
PLA2G12A	1.68E-33	0.27351	0.25	0.095	2.67E-29	Saliva_Acir	PLA2G12A
MRPS263	2.08E-33	0.273964	0.342	0.149	3.32E-29	Saliva_Acir	MRPS26
SNX14	2.76E-33	0.258287	0.204	0.069	4.4E-29	Saliva_Acir	SNX14
OMA1	2.89E-33	0.276178	0.134	0.036	4.6E-29	Saliva_Acir	OMA1
GRHPR	3.22E-33	0.285841	0.323	0.139	5.14E-29	Saliva_Acir	GRHPR
ROCK24	3.96E-33	0.275181	0.25	0.095	6.31E-29	Saliva_Acir	ROCK2
ZFR1	6.8E-33	0.273085	0.274	0.109	1.08E-28	Saliva_Acir	ZFR
RUSC1	7.04E-33	0.255402	0.185	0.06	1.12E-28	Saliva_Acir	RUSC1
GNAQ3	7.09E-33	0.325955	0.361	0.163	1.13E-28	Saliva_Acir	GNAQ
ZNF6523	1.07E-32	0.266334	0.25	0.095	1.71E-28	Saliva_Acir	ZNF652
TATDN1	1.22E-32	0.293515	0.274	0.11	1.94E-28	Saliva_Acir	TATDN1
AKR1A14	1.32E-32	0.30463	0.425	0.203	2.1E-28	Saliva_Acir	AKR1A1
CLN52	1.43E-32	0.264809	0.219	0.078	2.28E-28	Saliva_Acir	CLN5

HSPD12	1.44E-32	0.312805	0.501	0.257	2.29E-28	Saliva_Acir	HSPD1
NEDD4L	1.47E-32	0.524689	0.287	0.12	2.34E-28	Saliva_Acir	NEDD4L
TAOK32	1.56E-32	0.252316	0.318	0.135	2.49E-28	Saliva_Acir	TAOK3
UBAC11	1.59E-32	0.300268	0.236	0.088	2.53E-28	Saliva_Acir	UBAC1
PCMTD13	2.12E-32	0.346879	0.382	0.179	3.38E-28	Saliva_Acir	PCMTD1
XRCC53	2.61E-32	0.399452	0.474	0.246	4.17E-28	Saliva_Acir	XRCC5
CEP2902	3.1E-32	0.250229	0.161	0.048	4.95E-28	Saliva_Acir	CEP290
MRPL272	4.64E-32	0.324196	0.325	0.143	7.39E-28	Saliva_Acir	MRPL27
KIF21A4	4.73E-32	0.35446	0.219	0.081	7.54E-28	Saliva_Acir	KIF21A
KCNMA12	7.02E-32	0.263264	0.142	0.04	1.12E-27	Saliva_Acir	KCNMA1
RANBP22	8.03E-32	0.280188	0.282	0.116	1.28E-27	Saliva_Acir	RANBP2
ATP6AP22	8.32E-32	0.347843	0.474	0.238	1.33E-27	Saliva_Acir	ATP6AP2
CAPN21	8.37E-32	0.434181	0.556	0.311	1.33E-27	Saliva_Acir	CAPN2
RPL306	9.69E-32	0.49323	0.966	0.844	1.55E-27	Saliva_Acir	RPL30
SPPL2A4	9.96E-32	0.263686	0.304	0.13	1.59E-27	Saliva_Acir	SPPL2A
RPL395	1.01E-31	0.372156	0.957	0.676	1.62E-27	Saliva_Acir	RPL39
GOSR12	1.36E-31	0.26107	0.253	0.099	2.17E-27	Saliva_Acir	GOSR1
TRIM331	1.4E-31	0.253567	0.276	0.112	2.24E-27	Saliva_Acir	TRIM33
BLCAP	1.41E-31	0.297214	0.319	0.138	2.24E-27	Saliva_Acir	BLCAP
FH	3.23E-31	0.274494	0.208	0.074	5.15E-27	Saliva_Acir	FH
GPAA13	3.78E-31	0.281055	0.323	0.142	6.02E-27	Saliva_Acir	GPAA1
AP1M21	4.32E-31	0.361621	0.2	0.072	6.89E-27	Saliva_Acir	AP1M2
USP82	4.55E-31	0.296934	0.321	0.142	7.25E-27	Saliva_Acir	USP8
NSD15	5.41E-31	0.277545	0.253	0.1	8.63E-27	Saliva_Acir	NSD1
MRPL121	6.02E-31	0.281101	0.293	0.125	9.6E-27	Saliva_Acir	MRPL12
CCDC473	6.71E-31	0.33764	0.316	0.139	1.07E-26	Saliva_Acir	CCDC47
CMTM83	6.72E-31	0.273262	0.197	0.069	1.07E-26	Saliva_Acir	CMTM8
FRMD4B4	6.86E-31	0.32538	0.295	0.127	1.09E-26	Saliva_Acir	FRMD4B
FUS5	7.78E-31	0.332797	0.516	0.276	1.24E-26	Saliva_Acir	FUS
UBXN44	9.49E-31	0.323424	0.546	0.299	1.51E-26	Saliva_Acir	UBXN4
UBE2M2	1.01E-30	0.251254	0.363	0.165	1.6E-26	Saliva_Acir	UBE2M
KAT6B3	1.01E-30	0.252846	0.212	0.077	1.61E-26	Saliva_Acir	KAT6B
ARHGAP32	1.05E-30	0.271697	0.219	0.081	1.67E-26	Saliva_Acir	ARHGAP32
ECHDC12	1.4E-30	0.252747	0.28	0.116	2.24E-26	Saliva_Acir	ECHDC1
EIF4B3	1.42E-30	0.389502	0.533	0.299	2.26E-26	Saliva_Acir	EIF4B
USP145	2.42E-30	0.302655	0.251	0.1	3.86E-26	Saliva_Acir	USP14
DNAJC214	2.61E-30	0.256464	0.285	0.12	4.15E-26	Saliva_Acir	DNAJC21
PUS7L3	3.49E-30	0.280057	0.197	0.069	5.57E-26	Saliva_Acir	PUS7L
HNRNPD1	3.81E-30	0.322754	0.457	0.239	6.08E-26	Saliva_Acir	HNRNPD
NSA23	4.47E-30	0.37553	0.474	0.249	7.13E-26	Saliva_Acir	NSA2
ALDH3A22	5.92E-30	0.301919	0.293	0.126	9.44E-26	Saliva_Acir	ALDH3A2
ATP6V0B4	7.92E-30	0.763938	0.631	0.387	1.26E-25	Saliva_Acir	ATP6V0B
ST14	8.01E-30	0.352452	0.318	0.142	1.28E-25	Saliva_Acir	ST14
ZBTB382	8.59E-30	0.269392	0.363	0.169	1.37E-25	Saliva_Acir	ZBTB38
PRRC2C3	8.9E-30	0.377981	0.503	0.278	1.42E-25	Saliva_Acir	PRRC2C
VDAC31	9.12E-30	0.276189	0.388	0.185	1.45E-25	Saliva_Acir	VDAC3
BMS1	1.06E-29	0.263245	0.189	0.066	1.69E-25	Saliva_Acir	BMS1

SPATS2L1	1.21E-29	0.37813	0.429	0.223	1.92E-25	Saliva_Acir	SPATS2L
TM9SF31	1.38E-29	0.311757	0.452	0.235	2.21E-25	Saliva_Acir	TM9SF3
DHX362	1.43E-29	0.293548	0.382	0.181	2.28E-25	Saliva_Acir	DHX36
NEMF1	1.57E-29	0.321675	0.348	0.163	2.5E-25	Saliva_Acir	NEMF
ECHS12	1.63E-29	0.394251	0.403	0.203	2.6E-25	Saliva_Acir	ECHS1
COX5A1	1.83E-29	0.428232	0.662	0.411	2.92E-25	Saliva_Acir	COX5A
MARCKSL1	2.05E-29	0.284884	0.418	0.204	3.26E-25	Saliva_Acir	MARCKSL1
BDP11	2.94E-29	0.265685	0.257	0.104	4.69E-25	Saliva_Acir	BDP1
MLLT4	3.46E-29	0.262774	0.439	0.225	5.52E-25	Saliva_Acir	MLLT4
PAWR2	3.92E-29	0.291857	0.297	0.13	6.26E-25	Saliva_Acir	PAWR
SF3B15	4.06E-29	0.308289	0.474	0.252	6.48E-25	Saliva_Acir	SF3B1
AGPAT21	6.92E-29	0.282651	0.291	0.126	1.1E-24	Saliva_Acir	AGPAT2
NDUFB9	7.55E-29	0.26524	0.611	0.345	1.2E-24	Saliva_Acir	NDUFB9
MPP5	7.95E-29	0.268165	0.212	0.079	1.27E-24	Saliva_Acir	MPP5
RAB132	9.51E-29	0.381749	0.42	0.218	1.52E-24	Saliva_Acir	RAB13
SRRM26	1E-28	0.378533	0.584	0.347	1.6E-24	Saliva_Acir	SRRM2
UBE2H	1.01E-28	0.326258	0.318	0.146	1.61E-24	Saliva_Acir	UBE2H
MBNL23	1.67E-28	0.262197	0.278	0.118	2.67E-24	Saliva_Acir	MBNL2
NCKAP11	1.88E-28	0.273714	0.325	0.149	3E-24	Saliva_Acir	NCKAP1
PKN21	1.93E-28	0.31656	0.335	0.155	3.08E-24	Saliva_Acir	PKN2
BRD7	2.63E-28	0.328601	0.278	0.12	4.19E-24	Saliva_Acir	BRD7
SAA21	2.71E-28	0.325638	0.163	0.053	4.33E-24	Saliva_Acir	SAA2
MRPL34	3.51E-28	0.269756	0.369	0.178	5.6E-24	Saliva_Acir	MRPL34
PTK21	4.87E-28	0.285056	0.265	0.112	7.77E-24	Saliva_Acir	PTK2
EIF1AX1	5.19E-28	0.312917	0.577	0.336	8.27E-24	Saliva_Acir	EIF1AX
UACA2	5.29E-28	0.258777	0.265	0.111	8.44E-24	Saliva_Acir	UACA
EMC101	6.29E-28	0.283076	0.327	0.152	1E-23	Saliva_Acir	EMC10
PPP6R3	6.48E-28	0.256394	0.233	0.093	1.03E-23	Saliva_Acir	PPP6R3
NDUFA5	7.89E-28	0.316178	0.471	0.251	1.26E-23	Saliva_Acir	NDUFA5
MRPL205	8.02E-28	0.280888	0.493	0.27	1.28E-23	Saliva_Acir	MRPL20
VDAC1	1.13E-27	0.299011	0.457	0.241	1.8E-23	Saliva_Acir	VDAC1
GMPR2	1.19E-27	0.255221	0.219	0.085	1.9E-23	Saliva_Acir	GMPR2
TUFM1	1.22E-27	0.28444	0.527	0.291	1.95E-23	Saliva_Acir	TUFM
EIF55	1.7E-27	0.285999	0.611	0.359	2.71E-23	Saliva_Acir	EIF5
GAPDH1	2.77E-27	0.341129	0.94	0.763	4.42E-23	Saliva_Acir	GAPDH
TMX22	2.86E-27	0.251664	0.246	0.101	4.56E-23	Saliva_Acir	TMX2
UQCRFS11	3.02E-27	0.316833	0.488	0.27	4.81E-23	Saliva_Acir	UQCRFS1
CSNK1A12	3.22E-27	0.29605	0.497	0.271	5.13E-23	Saliva_Acir	CSNK1A1
HP1BP32	3.38E-27	0.28223	0.427	0.224	5.39E-23	Saliva_Acir	HP1BP3
HNRNPAB3	4.51E-27	0.304993	0.348	0.166	7.19E-23	Saliva_Acir	HNRNPAB
CLTC2	7.42E-27	0.305823	0.367	0.184	1.18E-22	Saliva_Acir	CLTC
IQGAP14	7.53E-27	0.311566	0.469	0.258	1.2E-22	Saliva_Acir	IQGAP1
HSP90AB1	8.35E-27	0.461916	0.807	0.569	1.33E-22	Saliva_Acir	HSP90AB1
OARD1	9.74E-27	0.260478	0.276	0.12	1.55E-22	Saliva_Acir	OARD1
CXADR	1.19E-26	0.28991	0.348	0.167	1.9E-22	Saliva_Acir	CXADR
ZFYVE212	3.75E-26	0.288116	0.314	0.148	5.99E-22	Saliva_Acir	ZFYVE21
OCIAD14	5.27E-26	0.265428	0.465	0.252	8.4E-22	Saliva_Acir	OCIAD1

RTN31	5.57E-26	0.389103	0.467	0.262	8.88E-22	Saliva_Acir	RTN3
ACADVL1	6.29E-26	0.288183	0.435	0.231	1E-21	Saliva_Acir	ACADVL
GOPC1	7.29E-26	0.273104	0.24	0.101	1.16E-21	Saliva_Acir	GOPC
EIF4G11	8.35E-26	0.259529	0.342	0.167	1.33E-21	Saliva_Acir	EIF4G1
ZC3H11A1	1.68E-25	0.296054	0.338	0.165	2.68E-21	Saliva_Acir	ZC3H11A
KIF5B2	1.74E-25	0.260365	0.512	0.289	2.77E-21	Saliva_Acir	KIF5B
LSM52	2.13E-25	0.263109	0.393	0.201	3.39E-21	Saliva_Acir	LSM5
RPS122	2.39E-25	0.401988	0.981	0.861	3.81E-21	Saliva_Acir	RPS12
BCAP31	2.56E-25	0.297105	0.512	0.288	4.09E-21	Saliva_Acir	BCAP31
GTF2H5	2.6E-25	0.303245	0.388	0.199	4.14E-21	Saliva_Acir	GTF2H5
COX6C2	2.82E-25	0.397413	0.756	0.541	4.5E-21	Saliva_Acir	COX6C
CLIP1	8.88E-25	0.269335	0.348	0.173	1.42E-20	Saliva_Acir	CLIP1
ITIH53	1.34E-24	0.322921	0.127	0.039	2.13E-20	Saliva_Acir	ITIH5
MPZL13	1.37E-24	0.394173	0.295	0.14	2.19E-20	Saliva_Acir	MPZL1
CSDE13	2.29E-24	0.322983	0.575	0.358	3.65E-20	Saliva_Acir	CSDE1
C8orf596	2.99E-24	0.296262	0.429	0.234	4.77E-20	Saliva_Acir	C8orf59
HDGF	3.54E-24	0.257025	0.295	0.138	5.65E-20	Saliva_Acir	HDGF
DHRS7B1	5.87E-24	0.250241	0.191	0.075	9.35E-20	Saliva_Acir	DHRS7B
FAM13C2	7.29E-24	0.32684	0.089	0.023	1.16E-19	Saliva_Acir	FAM13C
UBA3	8.56E-24	0.267428	0.212	0.087	1.36E-19	Saliva_Acir	UBA3
HMGB14	1.03E-23	0.318758	0.805	0.574	1.65E-19	Saliva_Acir	HMGB1
FAM3C1	1.23E-23	0.259484	0.38	0.196	1.96E-19	Saliva_Acir	FAM3C
RAB141	1.52E-23	0.263291	0.363	0.187	2.43E-19	Saliva_Acir	RAB14
PHLDB21	2.22E-23	0.254112	0.151	0.052	3.54E-19	Saliva_Acir	PHLDB2
RPL227	3.29E-23	0.398116	0.913	0.693	5.25E-19	Saliva_Acir	RPL22
WBP41	3.61E-23	0.259993	0.227	0.096	5.76E-19	Saliva_Acir	WBP4
IRF2BP2	4.6E-23	0.260629	0.454	0.258	7.33E-19	Saliva_Acir	IRF2BP2
NCALD2	4.61E-23	0.482032	0.178	0.069	7.35E-19	Saliva_Acir	NCALD
CCNI3	6.95E-23	0.317381	0.741	0.517	1.11E-18	Saliva_Acir	CCNI
TMEM9B	1.6E-22	0.27637	0.312	0.156	2.55E-18	Saliva_Acir	TMEM9B
PRDX15	1.69E-22	0.272367	0.709	0.451	2.7E-18	Saliva_Acir	PRDX1
C12orf574	2.43E-22	0.275997	0.588	0.356	3.87E-18	Saliva_Acir	C12orf57
ATP5O	4.05E-22	0.250481	0.673	0.429	6.46E-18	Saliva_Acir	ATP5O
CLINT12	6.15E-22	0.264739	0.336	0.174	9.81E-18	Saliva_Acir	CLINT1
MRPS162	7.74E-22	0.254127	0.306	0.155	1.23E-17	Saliva_Acir	MRPS16
MKKS2	9.14E-22	0.257395	0.316	0.159	1.46E-17	Saliva_Acir	MKKS
JMJD1C	3.1E-21	0.261608	0.42	0.234	4.94E-17	Saliva_Acir	JMJD1C
MRPS361	1.68E-20	0.258044	0.35	0.188	2.67E-16	Saliva_Acir	MRPS36
RPL53	6.84E-20	0.29772	0.934	0.727	1.09E-15	Saliva_Acir	RPL5
NDUFA6	7.84E-20	0.263356	0.524	0.321	1.25E-15	Saliva_Acir	NDUFA6
ALDOA	1.91E-19	0.282626	0.858	0.641	3.04E-15	Saliva_Acir	ALDOA
SSFA22	2.18E-19	0.460175	0.312	0.167	3.48E-15	Saliva_Acir	SSFA2
EIF3A2	2.28E-19	0.270087	0.48	0.293	3.64E-15	Saliva_Acir	EIF3A
CIRBP1	6.05E-18	0.260273	0.76	0.545	9.64E-14	Saliva_Acir	CIRBP
C1orf1223	9.35E-18	0.300334	0.333	0.183	1.49E-13	Saliva_Acir	C1orf122
COX6A11	4.43E-17	0.277116	0.76	0.53	7.06E-13	Saliva_Acir	COX6A1
RPL7A6	1.76E-15	0.289678	0.947	0.813	2.81E-11	Saliva_Acir	RPL7A

RPLP12	3.47E-14	0.285751	0.983	0.915	5.53E-10	Saliva_Acir	RPLP1
KRT231	1.72E-13	0.280254	0.321	0.19	2.74E-09	Saliva_Acir	KRT23
RPL245	1.88E-12	0.27259	0.936	0.802	2.99E-08	Saliva_Acir	RPL24
FOSB7	3.1E-10	0.411079	0.365	0.251	4.95E-06	Saliva_Acir	FOSB
JUN4	1.87E-09	0.28112	0.641	0.487	2.98E-05	Saliva_Acir	JUN
TAGLN1	0	4.950558	0.984	0.082	0	Saliva_Duc	TAGLN
ACTA21	0	4.871766	0.989	0.063	0	Saliva_Duc	ACTA2
TPM21	0	4.840636	0.984	0.083	0	Saliva_Duc	TPM2
MYLK1	0	4.246225	0.935	0.053	0	Saliva_Duc	MYLK
ACTG21	0	4.099608	0.876	0.014	0	Saliva_Duc	ACTG2
MYH111	0	4.086534	0.946	0.037	0	Saliva_Duc	MYH11
C2orf402	0	3.857546	0.876	0.038	0	Saliva_Duc	C2orf40
MYL92	0	3.626262	0.957	0.117	0	Saliva_Duc	MYL9
SFRP13	0	3.384557	0.881	0.094	0	Saliva_Duc	SFRP1
SYNM1	0	2.766283	0.605	0.018	0	Saliva_Duc	SYNM
PPP1R14A1	0	2.653714	0.686	0.045	0	Saliva_Duc	PPP1R14A
KCNMB12	0	1.908936	0.454	0.015	0	Saliva_Duc	KCNMB1
STAC2	0	0.8274	0.141	0.002	0	Saliva_Duc	STAC2
S100B	7.2E-306	1.727231	0.486	0.028	1.2E-301	Saliva_Duc	S100B
KRT51	1.6E-299	2.47027	0.795	0.078	2.6E-295	Saliva_Duc	KRT5
COL17A11	5.3E-288	2.016519	0.503	0.032	8.4E-284	Saliva_Duc	COL17A1
GAS65	2E-283	3.179635	0.811	0.094	3.3E-279	Saliva_Duc	GAS6
KRT141	3.6E-268	2.924766	0.984	0.148	5.8E-264	Saliva_Duc	KRT14
MEG31	1.7E-260	2.720693	0.795	0.092	2.7E-256	Saliva_Duc	MEG3
LAMA31	7.8E-252	1.536537	0.351	0.018	1.2E-247	Saliva_Duc	LAMA3
PDLIM35	7.8E-247	2.311593	0.611	0.057	1.2E-242	Saliva_Duc	PDLIM3
A2M3	9.8E-247	2.795198	0.746	0.084	1.6E-242	Saliva_Duc	A2M
IGFBP24	5.9E-225	2.654517	0.562	0.052	9.4E-221	Saliva_Duc	IGFBP2
NEXN1	1.4E-221	1.68747	0.362	0.022	2.3E-217	Saliva_Duc	NEXN
COL9A31	2.4E-221	1.766735	0.427	0.03	3.8E-217	Saliva_Duc	COL9A3
CALD12	7.9E-209	3.47266	0.951	0.194	1.3E-204	Saliva_Duc	CALD1
CSRP12	3.5E-207	3.273921	0.859	0.154	5.6E-203	Saliva_Duc	CSRP1
TPM13	7.3E-196	3.485547	0.908	0.186	1.2E-191	Saliva_Duc	TPM1
TIMP33	1.1E-192	3.016986	0.897	0.165	1.7E-188	Saliva_Duc	TIMP3
DST5	1.5E-181	3.262737	0.881	0.183	2.4E-177	Saliva_Duc	DST
SYNPO22	1.6E-181	1.485527	0.362	0.026	2.6E-177	Saliva_Duc	SYNPO2
MOXD1	2.2E-168	1.334786	0.281	0.017	3.5E-164	Saliva_Duc	MOXD1
ITPR12	5E-166	1.600749	0.368	0.029	7.9E-162	Saliva_Duc	ITPR1
MAP1B2	5.4E-166	2.199205	0.638	0.088	8.6E-162	Saliva_Duc	MAP1B
TGFB1I13	1.6E-165	1.91531	0.486	0.052	2.5E-161	Saliva_Duc	TGFB1I1
FLNA5	1.4E-164	2.533043	0.795	0.148	2.2E-160	Saliva_Duc	FLNA
DLK21	4.3E-162	0.771404	0.141	0.004	6.8E-158	Saliva_Duc	DLK2
FHOD3	2E-159	0.889525	0.168	0.006	3.2E-155	Saliva_Duc	FHOD3
SSPN2	3.2E-157	1.895551	0.546	0.067	5.1E-153	Saliva_Duc	SSPN
FBXO21	8.1E-155	1.624205	0.324	0.025	1.3E-150	Saliva_Duc	FBXO2
EFEMP11	2.5E-154	2.063587	0.551	0.07	4.1E-150	Saliva_Duc	EFEMP1
MIA	3.2E-153	0.812553	0.114	0.003	5E-149	Saliva_Duc	MIA

TUBB2B	6.3E-146	1.381142	0.281	0.019	1E-141	Saliva_Duc	TUBB2B
MFGE82	3.8E-142	2.064468	0.627	0.099	6E-138	Saliva_Duc	MFGE8
COL4A23	5.4E-142	1.779494	0.465	0.054	8.7E-138	Saliva_Duc	COL4A2
SORBS11	1.2E-141	1.642521	0.368	0.034	1.9E-137	Saliva_Duc	SORBS1
PPP1R12B2	4.5E-137	1.901029	0.432	0.049	7.2E-133	Saliva_Duc	PPP1R12B
DKK33	1.5E-135	1.702242	0.405	0.043	2.4E-131	Saliva_Duc	DKK3
FST2	6.9E-130	1.530338	0.308	0.026	1.1E-125	Saliva_Duc	FST
CTGF2	5.5E-129	2.460167	0.638	0.112	8.8E-125	Saliva_Duc	CTGF
MATN22	1.3E-128	1.49323	0.389	0.042	2.1E-124	Saliva_Duc	MATN2
PDLIM44	5.1E-128	2.050267	0.605	0.104	8.1E-124	Saliva_Duc	PDLIM4
ACTN12	2E-125	2.311089	0.697	0.147	3.2E-121	Saliva_Duc	ACTN1
NCS11	5.6E-123	1.023446	0.189	0.01	8.9E-119	Saliva_Duc	NCS1
NFIB6	5.8E-114	2.232271	0.762	0.19	9.2E-110	Saliva_Duc	NFIB
FILIP13	2.9E-112	1.20491	0.265	0.022	4.6E-108	Saliva_Duc	FILIP1
CRISPLD11	1.3E-111	1.086465	0.211	0.014	2.1E-107	Saliva_Duc	CRISPLD1
TCEAL6	2E-93	0.368172	0.07	0.002	3.2E-89	Saliva_Duc	TCEAL6
IGFBP52	3.77E-93	1.761535	0.649	0.136	6.01E-89	Saliva_Duc	IGFBP5
CLMP1	4.47E-93	0.919536	0.189	0.014	7.13E-89	Saliva_Duc	CLMP
CPE3	1.26E-91	1.574486	0.546	0.102	2.01E-87	Saliva_Duc	CPE
MTRNR2L1	2.76E-89	2.324943	0.827	0.269	4.4E-85	Saliva_Duc	MTRNR2L12
PPP1R12A4	4.13E-87	2.125149	0.654	0.177	6.59E-83	Saliva_Duc	PPP1R12A
GPC6	6.43E-87	0.694046	0.135	0.008	1.02E-82	Saliva_Duc	GPC6
TNS14	7.78E-86	1.394643	0.389	0.059	1.24E-81	Saliva_Duc	TNS1
SAMD5	1.19E-85	0.583639	0.108	0.005	1.89E-81	Saliva_Duc	SAMD5
AQP12	1.76E-85	1.583284	0.514	0.095	2.8E-81	Saliva_Duc	AQP1
LGALS14	4.46E-85	1.721777	0.989	0.41	7.12E-81	Saliva_Duc	LGALS1
MT.CYB10	8.54E-85	1.509485	1	0.934	1.36E-80	Saliva_Duc	MT.CYB
COL8A21	3.04E-84	0.670657	0.151	0.01	4.86E-80	Saliva_Duc	COL8A2
PALLD3	4.95E-84	1.9052	0.616	0.16	7.9E-80	Saliva_Duc	PALLD
MXRA73	1.59E-83	1.495313	0.459	0.083	2.53E-79	Saliva_Duc	MXRA7
IRX12	5.52E-81	1.022198	0.205	0.018	8.81E-77	Saliva_Duc	IRX1
NTRK22	3.42E-80	1.402812	0.362	0.053	5.45E-76	Saliva_Duc	NTRK2
C9orf33	6.56E-77	1.776479	0.476	0.098	1.05E-72	Saliva_Duc	C9orf3
MT.ATP61(	9.08E-77	1.424615	1	0.898	1.45E-72	Saliva_Duc	MT.ATP6
MT.ND310	4.06E-75	1.446468	1	0.794	6.47E-71	Saliva_Duc	MT.ND3
MT.ND110	6.03E-73	1.390293	1	0.927	9.62E-69	Saliva_Duc	MT.ND1
LAMB31	1.04E-71	1.367569	0.368	0.062	1.66E-67	Saliva_Duc	LAMB3
NGFR1	2.22E-71	0.84265	0.141	0.01	3.53E-67	Saliva_Duc	NGFR
MT.CO39	3.7E-71	1.255499	1	0.977	5.9E-67	Saliva_Duc	MT.CO3
LPP2	8.53E-71	1.898819	0.665	0.211	1.36E-66	Saliva_Duc	LPP
ANXA33	2.49E-70	1.966756	0.562	0.154	3.97E-66	Saliva_Duc	ANXA3
COL9A21	7.55E-70	0.873005	0.178	0.016	1.2E-65	Saliva_Duc	COL9A2
MT.ND510	4.1E-69	1.369251	1	0.702	6.54E-65	Saliva_Duc	MT.ND5
MT.ND410	5.87E-69	1.177985	1	0.954	9.36E-65	Saliva_Duc	MT.ND4
TCEAL21	4.52E-68	1.301235	0.281	0.039	7.21E-64	Saliva_Duc	TCEAL2
FGFR11	1.25E-67	1.393682	0.438	0.087	1.99E-63	Saliva_Duc	FGFR1
MTRNR2L1	2.29E-65	1.61761	0.459	0.097	3.65E-61	Saliva_Duc	MTRNR2L10

SMOC1	2.76E-65	0.59583	0.114	0.007	4.39E-61	Saliva_Duc	SMOC1
MSRB33	4.65E-65	1.237024	0.259	0.035	7.42E-61	Saliva_Duc	MSRB3
LTBP12	9.5E-65	1.28856	0.324	0.052	1.52E-60	Saliva_Duc	LTBP1
MT.CO210	7.44E-63	1.005453	1	0.96	1.19E-58	Saliva_Duc	MT.CO2
PDLIM73	1.61E-62	1.314486	0.351	0.064	2.57E-58	Saliva_Duc	PDLIM7
CST32	7.67E-62	2.97541	0.924	0.593	1.22E-57	Saliva_Duc	CST3
CD817	8.46E-62	1.317051	0.832	0.323	1.35E-57	Saliva_Duc	CD81
GAPDH2	8.49E-62	1.449546	0.984	0.764	1.35E-57	Saliva_Duc	GAPDH
SELM6	2.33E-61	1.53614	0.822	0.347	3.72E-57	Saliva_Duc	SELM
RBBP81	8.42E-61	1.241409	0.314	0.053	1.34E-56	Saliva_Duc	RBBP8
KRT83	1.05E-60	1.643955	0.638	0.2	1.68E-56	Saliva_Duc	KRT8
FHL2	2.52E-60	1.705229	0.557	0.159	4.02E-56	Saliva_Duc	FHL2
JAG22	1.36E-59	0.700382	0.146	0.012	2.17E-55	Saliva_Duc	JAG2
PIK3R14	2.8E-59	1.779796	0.454	0.11	4.46E-55	Saliva_Duc	PIK3R1
VWA11	3.39E-59	1.476228	0.384	0.08	5.4E-55	Saliva_Duc	VWA1
CCDC81	4.07E-58	0.75076	0.135	0.011	6.5E-54	Saliva_Duc	CCDC8
KRT73	1.27E-57	1.736323	0.741	0.272	2.02E-53	Saliva_Duc	KRT7
BHLHE413	3.8E-57	1.128336	0.281	0.045	6.06E-53	Saliva_Duc	BHLHE41
CAV13	1.62E-56	1.213813	0.665	0.198	2.58E-52	Saliva_Duc	CAV1
DSTN2	6.61E-56	1.492297	0.892	0.563	1.05E-51	Saliva_Duc	DSTN
MT.ND210	6.87E-56	0.977746	1	0.907	1.1E-51	Saliva_Duc	MT.ND2
PCSK1N	1.26E-55	1.393636	0.33	0.061	2.01E-51	Saliva_Duc	PCSK1N
FBXO32	5.33E-54	1.776493	0.578	0.184	8.5E-50	Saliva_Duc	FBXO32
JAM31	6.13E-54	0.97757	0.195	0.024	9.78E-50	Saliva_Duc	JAM3
THBS11	1.17E-52	1.231164	0.378	0.08	1.87E-48	Saliva_Duc	THBS1
MYL61	1.16E-51	1.17863	0.968	0.805	1.85E-47	Saliva_Duc	MYL6
KRT232	2.26E-51	1.564282	0.578	0.19	3.61E-47	Saliva_Duc	KRT23
PDGFA1	3E-50	0.986264	0.232	0.035	4.79E-46	Saliva_Duc	PDGFA
MTRNR2L1	1.58E-49	1.911113	0.465	0.125	2.52E-45	Saliva_Duc	MTRNR2L1
MARVELD1	1.67E-49	0.929719	0.222	0.032	2.67E-45	Saliva_Duc	MARVELD1
CD1516	6.41E-48	1.561672	0.589	0.22	1.02E-43	Saliva_Duc	CD151
FERMT23	6.79E-47	1.14955	0.357	0.082	1.08E-42	Saliva_Duc	FERMT2
PITX2	1.6E-46	0.599426	0.114	0.01	2.55E-42	Saliva_Duc	PITX2
MTRNR2L8	1.86E-46	1.30752	0.708	0.286	2.97E-42	Saliva_Duc	MTRNR2L8
PTRF3	1.03E-45	1.365239	0.524	0.163	1.65E-41	Saliva_Duc	PTRF
DMD2	1.72E-45	1.205736	0.27	0.051	2.75E-41	Saliva_Duc	DMD
MCAM2	2.24E-45	0.910934	0.205	0.03	3.58E-41	Saliva_Duc	MCAM
FBXL221	5.57E-45	0.460245	0.092	0.007	8.88E-41	Saliva_Duc	FBXL22
XIST9	1.42E-44	1.456217	0.438	0.123	2.27E-40	Saliva_Duc	XIST
LIMS23	1.67E-44	1.000169	0.211	0.032	2.66E-40	Saliva_Duc	LIMS2
DGKG1	3.08E-44	0.342754	0.059	0.003	4.91E-40	Saliva_Duc	DGKG
TGFBR32	1.92E-43	1.380407	0.368	0.091	3.07E-39	Saliva_Duc	TGFBR3
GPC31	4.3E-43	0.785408	0.195	0.028	6.86E-39	Saliva_Duc	GPC3
RGS9	4.24E-42	0.313749	0.065	0.004	6.75E-38	Saliva_Duc	RGS9
TRIM9	3.38E-41	0.34216	0.07	0.004	5.39E-37	Saliva_Duc	TRIM9
MT.ND4L1	3.44E-41	1.210608	0.784	0.416	5.49E-37	Saliva_Duc	MT.ND4L
BOC1	8.79E-41	0.776517	0.178	0.025	1.4E-36	Saliva_Duc	BOC

FAM198B1	1.65E-40	1.039832	0.211	0.035	2.64E-36	Saliva_Duc	FAM198B
MT1X	1.65E-40	0.665509	0.568	0.2	2.64E-36	Saliva_Duc	MT1X
PDLIM12	1.99E-40	1.490202	0.562	0.224	3.18E-36	Saliva_Duc	PDLIM1
BCAM2	4.63E-40	1.083266	0.324	0.076	7.39E-36	Saliva_Duc	BCAM
FAXC	1.36E-39	0.52075	0.097	0.008	2.16E-35	Saliva_Duc	FAXC
MGST38	1.49E-39	1.286722	0.708	0.335	2.38E-35	Saliva_Duc	MGST3
ITM2C1	3.31E-38	1.049905	0.416	0.121	5.29E-34	Saliva_Duc	ITM2C
SCPEP12	8.16E-38	1.211973	0.335	0.085	1.3E-33	Saliva_Duc	SCPEP1
LTBP22	1.43E-37	0.631638	0.141	0.017	2.27E-33	Saliva_Duc	LTBP2
SEMA3C2	3.59E-37	0.94031	0.205	0.035	5.72E-33	Saliva_Duc	SEMA3C
MT.CO110	4.26E-37	0.730829	1	0.985	6.8E-33	Saliva_Duc	MT.CO1
ITGA22	5.08E-37	1.364912	0.335	0.088	8.1E-33	Saliva_Duc	ITGA2
CD637	1.12E-35	1.037367	0.924	0.624	1.79E-31	Saliva_Duc	CD63
NRTN	1.76E-35	0.600689	0.124	0.015	2.8E-31	Saliva_Duc	NRTN
RPLP13	1.83E-35	0.800574	1	0.915	2.92E-31	Saliva_Duc	RPLP1
ITGB12	1.94E-35	1.435224	0.67	0.357	3.09E-31	Saliva_Duc	ITGB1
HTRA13	2.19E-35	1.08328	0.389	0.112	3.5E-31	Saliva_Duc	HTRA1
SVIL3	6.38E-35	1.339925	0.384	0.12	1.02E-30	Saliva_Duc	SVIL
PLCB12	1.01E-34	1.028195	0.211	0.04	1.6E-30	Saliva_Duc	PLCB1
CDH3	2.04E-34	0.678579	0.124	0.015	3.26E-30	Saliva_Duc	CDH3
TCEAL72	2.71E-34	0.57484	0.119	0.014	4.32E-30	Saliva_Duc	TCEAL7
CD2001	3.86E-34	0.647677	0.146	0.02	6.15E-30	Saliva_Duc	CD200
KCNQ5	4.32E-34	0.293925	0.038	0.002	6.89E-30	Saliva_Duc	KCNQ5
EEF1A15	4.37E-34	0.774286	0.995	0.915	6.97E-30	Saliva_Duc	EEF1A1
ADCY51	4.49E-34	0.54151	0.081	0.007	7.17E-30	Saliva_Duc	ADCY5
EPHB6	5.29E-34	0.770595	0.195	0.034	8.44E-30	Saliva_Duc	EPHB6
ANTXR12	7.71E-34	1.019357	0.238	0.049	1.23E-29	Saliva_Duc	ANTXR1
ATP2A21	7.86E-34	1.420931	0.459	0.175	1.25E-29	Saliva_Duc	ATP2A2
PTMS5	9.26E-34	1.30928	0.568	0.244	1.48E-29	Saliva_Duc	PTMS
DSG22	1.35E-33	1.018243	0.254	0.058	2.16E-29	Saliva_Duc	DSG2
RPL228	3E-33	0.983262	0.946	0.694	4.79E-29	Saliva_Duc	RPL22
MTSS11	5.06E-33	1.056561	0.259	0.059	8.06E-29	Saliva_Duc	MTSS1
FGF72	1.21E-32	1.058876	0.227	0.046	1.93E-28	Saliva_Duc	FGF7
CHL12	5.21E-32	0.660965	0.114	0.014	8.31E-28	Saliva_Duc	CHL1
MRGPRF2	6.58E-32	0.73961	0.162	0.026	1.05E-27	Saliva_Duc	MRGPRF
ACTN42	1.24E-31	1.172661	0.627	0.307	1.98E-27	Saliva_Duc	ACTN4
RPS245	1.94E-31	0.817448	1	0.839	3.09E-27	Saliva_Duc	RPS24
C1QTNF4	2.09E-31	0.786182	0.151	0.023	3.33E-27	Saliva_Duc	C1QTNF4
PEBP16	1.01E-30	1.12696	0.757	0.456	1.61E-26	Saliva_Duc	PEBP1
C12orf575	1.2E-30	1.27427	0.67	0.357	1.91E-26	Saliva_Duc	C12orf57
SEPW15	3.11E-30	1.17088	0.659	0.359	4.96E-26	Saliva_Duc	SEPW1
RAI141	4.47E-30	1.185376	0.297	0.083	7.13E-26	Saliva_Duc	RAI14
SPTBN12	8.73E-30	1.153244	0.486	0.198	1.39E-25	Saliva_Duc	SPTBN1
KLHL422	1.17E-29	0.847345	0.189	0.037	1.86E-25	Saliva_Duc	KLHL42
ISM1	1.24E-29	0.579356	0.092	0.01	1.97E-25	Saliva_Duc	ISM1
PYGB2	1.41E-29	1.111595	0.276	0.073	2.25E-25	Saliva_Duc	PYGB
S100A62	1.98E-29	0.592083	0.989	0.713	3.15E-25	Saliva_Duc	S100A6

SLC22A172	4.28E-29	0.934154	0.2	0.041	6.82E-25	Saliva_Duc	SLC22A17
RPS179	8.83E-29	0.812992	0.557	0.222	1.41E-24	Saliva_Duc	RPS17
FAM129A6	1.2E-28	1.081613	0.335	0.104	1.92E-24	Saliva_Duc	FAM129A
GLT8D22	1.26E-28	0.687311	0.151	0.025	2.01E-24	Saliva_Duc	GLT8D2
ZNF483	1.61E-28	0.565546	0.097	0.011	2.57E-24	Saliva_Duc	ZNF483
SBSPON1	2.44E-28	0.572138	0.13	0.019	3.89E-24	Saliva_Duc	SBSPON
EPB41L23	2.64E-28	1.011841	0.314	0.092	4.2E-24	Saliva_Duc	EPB41L2
RPL54	6.49E-28	0.662966	0.978	0.728	1.03E-23	Saliva_Duc	RPL5
TMEM158	1.23E-27	0.538551	0.097	0.012	1.96E-23	Saliva_Duc	TMEM158
SH3BGR	2.06E-27	0.774724	0.157	0.028	3.28E-23	Saliva_Duc	SH3BGR
RPL246	5.05E-27	0.745095	0.957	0.802	8.05E-23	Saliva_Duc	RPL24
PERP2	7.71E-27	0.888883	0.676	0.367	1.23E-22	Saliva_Duc	PERP
PLD5	1.91E-26	0.368088	0.059	0.005	3.04E-22	Saliva_Duc	PLD5
MPDZ3	2.1E-26	0.71529	0.162	0.03	3.35E-22	Saliva_Duc	MPDZ
CENPW	2.37E-26	1.108948	0.227	0.056	3.78E-22	Saliva_Duc	CENPW
SKP12	4.69E-26	0.851409	0.822	0.571	7.47E-22	Saliva_Duc	SKP1
ESD5	5.14E-26	1.168987	0.486	0.217	8.2E-22	Saliva_Duc	ESD
SAA22	1.47E-25	1.063658	0.222	0.053	2.35E-21	Saliva_Duc	SAA2
TMEM25	1.73E-25	0.730265	0.141	0.024	2.76E-21	Saliva_Duc	TMEM25
VIM6	1.96E-25	0.463023	0.919	0.494	3.12E-21	Saliva_Duc	VIM
MT.ATP88	2.05E-25	1.029811	0.373	0.135	3.27E-21	Saliva_Duc	MT.ATP8
RPL307	2.81E-25	0.705788	0.995	0.844	4.48E-21	Saliva_Duc	RPL30
NNAT	4.32E-25	0.600298	0.086	0.01	6.89E-21	Saliva_Duc	NNAT
PDGFB2	4.65E-25	0.58396	0.124	0.02	7.42E-21	Saliva_Duc	PDGFB
H3F3A9	5.07E-25	0.759297	0.865	0.478	8.08E-21	Saliva_Duc	H3F3A
EPHA41	5.24E-25	0.510544	0.108	0.015	8.35E-21	Saliva_Duc	EPHA4
CCND21	7.57E-25	1.087631	0.314	0.102	1.21E-20	Saliva_Duc	CCND2
RASSF61	1.08E-24	0.578358	0.108	0.016	1.72E-20	Saliva_Duc	RASSF6
PRUNE2	1.18E-24	0.547701	0.097	0.013	1.89E-20	Saliva_Duc	PRUNE2
CHRM31	1.22E-24	0.901148	0.178	0.039	1.95E-20	Saliva_Duc	CHRM3
ADH54	1.56E-24	1.064269	0.368	0.135	2.49E-20	Saliva_Duc	ADH5
IGFBP61	1.81E-24	0.464825	0.4	0.138	2.88E-20	Saliva_Duc	IGFBP6
SPHK11	3.11E-24	1.041848	0.238	0.064	4.96E-20	Saliva_Duc	SPHK1
MT1E	3.31E-24	0.278613	0.373	0.129	5.28E-20	Saliva_Duc	MT1E
ITGA65	3.58E-24	1.179164	0.362	0.136	5.71E-20	Saliva_Duc	ITGA6
RPS27A3	4.39E-24	0.645949	1	0.849	7E-20	Saliva_Duc	RPS27A
CD95	6.2E-24	0.790907	0.768	0.453	9.89E-20	Saliva_Duc	CD9
IFT812	2.79E-23	0.856814	0.178	0.04	4.44E-19	Saliva_Duc	IFT81
PLCH2	3.59E-23	0.382791	0.07	0.007	5.72E-19	Saliva_Duc	PLCH2
CMYA52	7.36E-23	0.730206	0.162	0.034	1.17E-18	Saliva_Duc	CMYA5
MYH92	1.77E-22	1.209991	0.422	0.186	2.82E-18	Saliva_Duc	MYH9
WLS3	2.12E-22	0.825147	0.184	0.043	3.39E-18	Saliva_Duc	WLS
PVRL1	2.92E-22	0.623102	0.146	0.029	4.65E-18	Saliva_Duc	PVRL1
KLHL29	5.17E-22	0.302143	0.054	0.005	8.25E-18	Saliva_Duc	KLHL29
ZFYVE213	9.17E-22	1.198481	0.362	0.149	1.46E-17	Saliva_Duc	ZFYVE21
CAP21	9.34E-22	0.393916	0.081	0.01	1.49E-17	Saliva_Duc	CAP2
SLC25A45	2.08E-21	1.016081	0.314	0.114	3.32E-17	Saliva_Duc	SLC25A4

DTNA1	2.91E-21	0.578365	0.108	0.018	4.64E-17	Saliva_Duc	DTNA
NDUFA45	5.24E-21	0.824602	0.784	0.538	8.35E-17	Saliva_Duc	NDUFA4
POPDC2	5.47E-21	0.290003	0.054	0.005	8.72E-17	Saliva_Duc	POPDC2
C12orf75	6.98E-21	0.905601	0.362	0.137	1.11E-16	Saliva_Duc	C12orf75
FAM60A1	1.18E-20	0.972074	0.297	0.106	1.88E-16	Saliva_Duc	FAM60A
TP73	1.34E-20	0.308755	0.054	0.005	2.14E-16	Saliva_Duc	TP73
FERMT1	1.44E-20	0.49202	0.097	0.015	2.3E-16	Saliva_Duc	FERMT1
PNISR6	1.88E-20	1.093407	0.595	0.348	2.99E-16	Saliva_Duc	PNISR
ZYX3	2.03E-20	0.913515	0.319	0.116	3.24E-16	Saliva_Duc	ZYX
SNCA3	2.19E-20	0.699735	0.184	0.046	3.49E-16	Saliva_Duc	SNCA
CRABP21	3.26E-20	0.891942	0.265	0.083	5.21E-16	Saliva_Duc	CRABP2
TUBA1A4	4.26E-20	0.937035	0.497	0.235	6.79E-16	Saliva_Duc	TUBA1A
ST52	9.9E-20	0.886532	0.211	0.06	1.58E-15	Saliva_Duc	ST5
RGN	1.02E-19	0.566187	0.097	0.015	1.63E-15	Saliva_Duc	RGN
SPEG1	1.09E-19	0.495767	0.092	0.014	1.74E-15	Saliva_Duc	SPEG
TP631	1.17E-19	0.580546	0.124	0.024	1.87E-15	Saliva_Duc	TP63
RPL92	1.26E-19	0.515151	0.973	0.75	2.01E-15	Saliva_Duc	RPL9
ZNRF3	1.75E-19	0.626823	0.108	0.019	2.78E-15	Saliva_Duc	ZNRF3
CYR612	1.91E-19	1.11874	0.314	0.113	3.04E-15	Saliva_Duc	CYR61
TCEAL5	2.88E-19	0.291687	0.054	0.005	4.59E-15	Saliva_Duc	TCEAL5
KIRREL1	3.11E-19	0.321319	0.059	0.006	4.96E-15	Saliva_Duc	KIRREL
COL6A22	4.25E-19	0.298941	0.422	0.16	6.79E-15	Saliva_Duc	COL6A2
TCF7L13	5.97E-19	0.688649	0.141	0.03	9.52E-15	Saliva_Duc	TCF7L1
PPIA1	8.45E-19	0.751188	0.805	0.59	1.35E-14	Saliva_Duc	PPIA
BGN2	8.88E-19	0.528707	0.232	0.067	1.42E-14	Saliva_Duc	BGN
CAPN61	3.53E-18	0.309175	0.059	0.007	5.63E-14	Saliva_Duc	CAPN6
RBMS33	4.67E-18	0.807669	0.189	0.052	7.45E-14	Saliva_Duc	RBMS3
RILPL12	5.17E-18	0.390419	0.086	0.013	8.24E-14	Saliva_Duc	RILPL1
PTGFRN2	6.05E-18	0.704697	0.162	0.041	9.65E-14	Saliva_Duc	PTGFRN
SGIP11	1.15E-17	0.341199	0.059	0.007	1.84E-13	Saliva_Duc	SGIP1
PAWR3	1.43E-17	1.02424	0.314	0.131	2.27E-13	Saliva_Duc	PAWR
APLP24	1.51E-17	1.031605	0.519	0.304	2.4E-13	Saliva_Duc	APLP2
TLE22	1.72E-17	0.815825	0.173	0.047	2.74E-13	Saliva_Duc	TLE2
VCL2	1.73E-17	1.038756	0.292	0.113	2.76E-13	Saliva_Duc	VCL
RPS234	1.86E-17	0.513879	0.995	0.814	2.96E-13	Saliva_Duc	RPS23
BAMBI	2.08E-17	0.484842	0.108	0.021	3.31E-13	Saliva_Duc	BAMBI
PLP23	2.12E-17	0.934488	0.459	0.232	3.39E-13	Saliva_Duc	PLP2
DDX245	2.13E-17	0.903282	0.465	0.237	3.39E-13	Saliva_Duc	DDX24
CALM22	2.19E-17	0.656873	0.8	0.563	3.5E-13	Saliva_Duc	CALM2
ITGB43	2.26E-17	1.024504	0.249	0.087	3.6E-13	Saliva_Duc	ITGB4
UQCRB2	4.94E-17	0.738425	0.778	0.557	7.88E-13	Saliva_Duc	UQCRB
SSBP22	5.17E-17	0.841549	0.222	0.072	8.25E-13	Saliva_Duc	SSBP2
N4BP2L28	5.29E-17	0.983613	0.503	0.293	8.44E-13	Saliva_Duc	N4BP2L2
GRAMD3	6.01E-17	0.761881	0.178	0.05	9.58E-13	Saliva_Duc	GRAMD3
PRSS232	6.11E-17	0.677723	0.27	0.094	9.74E-13	Saliva_Duc	PRSS23
PARK77	8.76E-17	0.87114	0.584	0.356	1.4E-12	Saliva_Duc	PARK7
KIAA12171	9.03E-17	0.938864	0.222	0.074	1.44E-12	Saliva_Duc	KIAA1217

NUCKS14	9.35E-17	0.93849	0.503	0.284	1.49E-12	Saliva_Duc	NUCKS1
PBX14	1.27E-16	1.034716	0.286	0.115	2.03E-12	Saliva_Duc	PBX1
HINT15	4.6E-16	0.653799	0.789	0.57	7.34E-12	Saliva_Duc	HINT1
RPS4X	5.71E-16	0.3939	0.978	0.771	9.11E-12	Saliva_Duc	RPS4X
ITGA32	6.29E-16	0.669819	0.135	0.032	1E-11	Saliva_Duc	ITGA3
RPL7A7	6.32E-16	0.474595	0.978	0.814	1.01E-11	Saliva_Duc	RPL7A
GAS11	8.56E-16	0.571268	0.146	0.036	1.37E-11	Saliva_Duc	GAS1
ALDH7A12	9.65E-16	0.867883	0.2	0.065	1.54E-11	Saliva_Duc	ALDH7A1
NFIX4	1E-15	0.979092	0.276	0.11	1.59E-11	Saliva_Duc	NFIX
PRDX16	1.64E-15	0.763218	0.665	0.452	2.61E-11	Saliva_Duc	PRDX1
SGCE2	1.96E-15	0.578504	0.151	0.04	3.12E-11	Saliva_Duc	SGCE
TLN14	2.2E-15	0.904405	0.308	0.13	3.5E-11	Saliva_Duc	TLN1
HSP90AB1	3.18E-15	0.723323	0.762	0.57	5.08E-11	Saliva_Duc	HSP90AB1
TPT1.AS12	6.76E-15	1.026625	0.243	0.093	1.08E-10	Saliva_Duc	TPT1.AS1
DMKN	7.08E-15	0.260796	0.351	0.157	1.13E-10	Saliva_Duc	DMKN
LAPTM4A1	7.3E-15	0.60794	0.73	0.519	1.16E-10	Saliva_Duc	LAPTM4A
CCND14	9.04E-15	0.994224	0.286	0.119	1.44E-10	Saliva_Duc	CCND1
NCOA73	1.16E-14	0.815669	0.27	0.108	1.85E-10	Saliva_Duc	NCOA7
NRG2	1.8E-14	0.441244	0.081	0.014	2.88E-10	Saliva_Duc	NRG2
PRKG12	1.89E-14	0.517281	0.103	0.022	3.01E-10	Saliva_Duc	PRKG1
TRIM291	2.65E-14	0.87183	0.254	0.101	4.22E-10	Saliva_Duc	TRIM29
FAM184A1	2.72E-14	0.514935	0.103	0.022	4.35E-10	Saliva_Duc	FAM184A
RPL182	3.03E-14	0.39774	0.968	0.878	4.84E-10	Saliva_Duc	RPL18
FXYD34	3.4E-14	0.531695	0.47	0.233	5.42E-10	Saliva_Duc	FXYD3
RBM381	4.29E-14	0.519265	0.141	0.037	6.85E-10	Saliva_Duc	RBM38
CITED42	4.74E-14	0.842859	0.227	0.083	7.56E-10	Saliva_Duc	CITED4
NUPR14	5.23E-14	0.692205	0.486	0.26	8.34E-10	Saliva_Duc	NUPR1
TUBB2A	5.7E-14	0.944807	0.27	0.113	9.09E-10	Saliva_Duc	TUBB2A
SLC35F3	6.48E-14	0.337497	0.054	0.007	1.03E-09	Saliva_Duc	SLC35F3
BOD1L17	6.61E-14	0.832418	0.292	0.128	1.05E-09	Saliva_Duc	BOD1L1
HSP90AA1	6.65E-14	0.281788	0.892	0.713	1.06E-09	Saliva_Duc	HSP90AA1
OGFRL14	7.61E-14	0.983087	0.27	0.115	1.21E-09	Saliva_Duc	OGFRL1
ARL4C2	1.07E-13	0.673408	0.389	0.188	1.7E-09	Saliva_Duc	ARL4C
ARHGAP20	1.48E-13	0.353621	0.07	0.012	2.36E-09	Saliva_Duc	ARHGAP20
EEF1B24	1.56E-13	0.438849	0.859	0.597	2.49E-09	Saliva_Duc	EEF1B2
AMIGO22	2.32E-13	0.381318	0.07	0.012	3.7E-09	Saliva_Duc	AMIGO2
LINC00665	2.48E-13	0.413926	0.081	0.015	3.95E-09	Saliva_Duc	LINC00665
ZC2HC1A2	2.52E-13	0.641726	0.157	0.047	4.01E-09	Saliva_Duc	ZC2HC1A
LEPROT3	2.59E-13	0.959095	0.432	0.257	4.12E-09	Saliva_Duc	LEPROT
ANKRD292	2.81E-13	0.529693	0.119	0.029	4.48E-09	Saliva_Duc	ANKRD29
ZNF4622	3.02E-13	0.769473	0.168	0.054	4.81E-09	Saliva_Duc	ZNF462
TINAGL12	3.44E-13	0.743825	0.222	0.083	5.48E-09	Saliva_Duc	TINAGL1
ROCK25	3.67E-13	0.900409	0.238	0.095	5.85E-09	Saliva_Duc	ROCK2
PRNP2	4.01E-13	0.823432	0.335	0.16	6.4E-09	Saliva_Duc	PRNP
FSTL11	4.21E-13	0.592335	0.341	0.154	6.71E-09	Saliva_Duc	FSTL1
PRDX26	4.67E-13	0.730884	0.503	0.312	7.45E-09	Saliva_Duc	PRDX2
NDUFS53	5.02E-13	0.808658	0.611	0.455	8.01E-09	Saliva_Duc	NDUFS5

NACA2	5.25E-13	0.352183	0.968	0.831	8.38E-09	Saliva_Duc	NACA
NFASC1	5.27E-13	0.307615	0.059	0.009	8.41E-09	Saliva_Duc	NFASC
RPL141	6.69E-13	0.335054	0.968	0.796	1.07E-08	Saliva_Duc	RPL14
CAV23	6.69E-13	0.958199	0.276	0.12	1.07E-08	Saliva_Duc	CAV2
C3orf701	6.88E-13	0.375617	0.076	0.014	1.1E-08	Saliva_Duc	C3orf70
ITIH54	7.11E-13	0.581741	0.141	0.039	1.13E-08	Saliva_Duc	ITIH5
CIRBP2	8.73E-13	0.69697	0.703	0.546	1.39E-08	Saliva_Duc	CIRBP
TMEM983	1.18E-12	0.856094	0.205	0.076	1.88E-08	Saliva_Duc	TMEM98
CSAD	1.2E-12	0.768669	0.173	0.058	1.92E-08	Saliva_Duc	CSAD
BRI35	1.21E-12	0.776612	0.524	0.347	1.93E-08	Saliva_Duc	BRI3
NFIC6	1.22E-12	0.872964	0.341	0.172	1.94E-08	Saliva_Duc	NFIC
TMEM121	1.22E-12	0.32233	0.07	0.012	1.95E-08	Saliva_Duc	TMEM121
EHD23	1.23E-12	0.603686	0.151	0.045	1.96E-08	Saliva_Duc	EHD2
SPINT24	1.31E-12	0.85264	0.503	0.314	2.1E-08	Saliva_Duc	SPINT2
RPL10A	1.35E-12	0.351058	0.978	0.735	2.15E-08	Saliva_Duc	RPL10A
CDKN2C	1.58E-12	0.695648	0.135	0.038	2.52E-08	Saliva_Duc	CDKN2C
RIC32	1.74E-12	0.76186	0.227	0.09	2.77E-08	Saliva_Duc	RIC3
FAM169A	1.89E-12	0.383883	0.076	0.014	3.02E-08	Saliva_Duc	FAM169A
NDN2	2.08E-12	0.635464	0.184	0.063	3.32E-08	Saliva_Duc	NDN
SPARC3	2.1E-12	0.317519	0.357	0.162	3.35E-08	Saliva_Duc	SPARC
RPS3A	2.36E-12	0.359255	0.984	0.831	3.77E-08	Saliva_Duc	RPS3A
FZD71	2.64E-12	0.60831	0.13	0.036	4.21E-08	Saliva_Duc	FZD7
NPTN2	3.21E-12	1.001235	0.276	0.128	5.11E-08	Saliva_Duc	NPTN
CRNDE2	3.3E-12	0.778207	0.319	0.155	5.27E-08	Saliva_Duc	CRNDE
KMT2A7	4.79E-12	0.898733	0.281	0.131	7.63E-08	Saliva_Duc	KMT2A
CHD98	5.39E-12	0.954997	0.324	0.167	8.6E-08	Saliva_Duc	CHD9
TCEAL42	5.43E-12	0.868339	0.319	0.16	8.66E-08	Saliva_Duc	TCEAL4
RRAS3	5.79E-12	0.822985	0.238	0.099	9.23E-08	Saliva_Duc	RRAS
SOD12	9.16E-12	0.624112	0.611	0.415	1.46E-07	Saliva_Duc	SOD1
SHPRH5	9.35E-12	0.776052	0.189	0.07	1.49E-07	Saliva_Duc	SHPRH
SERPINI13	1.18E-11	0.364386	0.097	0.023	1.89E-07	Saliva_Duc	SERPINI1
PGRMC12	1.22E-11	1.008154	0.319	0.167	1.94E-07	Saliva_Duc	PGRMC1
HMGB15	1.47E-11	0.63184	0.73	0.576	2.35E-07	Saliva_Duc	HMGB1
MGST14	2.13E-11	0.825023	0.357	0.194	3.4E-07	Saliva_Duc	MGST1
DIXDC12	2.26E-11	0.617919	0.146	0.047	3.6E-07	Saliva_Duc	DIXDC1
NKTR8	2.52E-11	0.807471	0.335	0.176	4.02E-07	Saliva_Duc	NKTR
RPL31	2.69E-11	0.267387	0.973	0.843	4.29E-07	Saliva_Duc	RPL3
GNG71	2.9E-11	0.655649	0.173	0.061	4.62E-07	Saliva_Duc	GNG7
TSPAN43	2.94E-11	0.687878	0.265	0.118	4.7E-07	Saliva_Duc	TSPAN4
TOMM208	3.21E-11	0.822341	0.486	0.326	5.12E-07	Saliva_Duc	TOMM20
MSRB24	3.47E-11	0.873853	0.27	0.13	5.53E-07	Saliva_Duc	MSRB2
NR2F23	3.54E-11	0.501983	0.222	0.087	5.64E-07	Saliva_Duc	NR2F2
CAPN121	3.81E-11	0.497992	0.092	0.022	6.08E-07	Saliva_Duc	CAPN12
NGFRAP11	4.24E-11	0.804691	0.368	0.207	6.76E-07	Saliva_Duc	NGFRAP1
NHSL1	4.38E-11	0.576764	0.13	0.039	6.99E-07	Saliva_Duc	NHSL1
FAU2	5.27E-11	0.325524	0.968	0.855	8.4E-07	Saliva_Duc	FAU
NBEA3	5.27E-11	0.76652	0.146	0.048	8.41E-07	Saliva_Duc	NBEA

ANKH3	5.65E-11	0.502942	0.124	0.036	9.01E-07	Saliva_Duc	ANKH
ASB2	5.97E-11	0.345683	0.054	0.009	9.52E-07	Saliva_Duc	ASB2
SOX23	6.13E-11	0.71586	0.141	0.045	9.78E-07	Saliva_Duc	SOX2
MTR3	7.11E-11	0.531624	0.13	0.039	1.13E-06	Saliva_Duc	MTR
MAML24	7.41E-11	0.565704	0.151	0.05	1.18E-06	Saliva_Duc	MAML2
DTWD12	1.05E-10	0.796377	0.2	0.081	1.68E-06	Saliva_Duc	DTWD1
USP311	1.13E-10	0.555519	0.124	0.037	1.81E-06	Saliva_Duc	USP31
RPS71	1.38E-10	0.270362	0.984	0.816	2.19E-06	Saliva_Duc	RPS7
CBR13	1.44E-10	0.865062	0.254	0.119	2.3E-06	Saliva_Duc	CBR1
RPS211	1.48E-10	0.509606	0.838	0.673	2.35E-06	Saliva_Duc	RPS21
SLC25A31(	1.71E-10	0.642916	0.6	0.456	2.72E-06	Saliva_Duc	SLC25A3
FRY2	1.72E-10	0.408806	0.086	0.021	2.74E-06	Saliva_Duc	FRY
KRT194	1.97E-10	0.641133	0.27	0.123	3.15E-06	Saliva_Duc	KRT19
AHNAK7	1.98E-10	0.51373	0.573	0.386	3.15E-06	Saliva_Duc	AHNAK
PPIG6	2.15E-10	0.796911	0.405	0.25	3.43E-06	Saliva_Duc	PPIG
SLC2A4RG:	2.32E-10	0.713835	0.227	0.099	3.7E-06	Saliva_Duc	SLC2A4RG
SLC16A71	2.35E-10	0.519207	0.13	0.04	3.75E-06	Saliva_Duc	SLC16A7
RPL396	2.54E-10	0.257	0.946	0.677	4.06E-06	Saliva_Duc	RPL39
ANGPTL21	2.68E-10	0.422966	0.114	0.032	4.27E-06	Saliva_Duc	ANGPTL2
GNAL1	4.11E-10	0.341779	0.076	0.017	6.56E-06	Saliva_Duc	GNAL
MYBPC12	4.45E-10	0.472761	0.081	0.019	7.09E-06	Saliva_Duc	MYBPC1
SLMAP	4.53E-10	0.829842	0.27	0.137	7.23E-06	Saliva_Duc	SLMAP
SDC31	4.8E-10	0.473141	0.092	0.024	7.66E-06	Saliva_Duc	SDC3
BEX2	4.88E-10	0.824477	0.216	0.094	7.78E-06	Saliva_Duc	BEX2
CCNI4	5.8E-10	0.536848	0.67	0.518	9.25E-06	Saliva_Duc	CCNI
ENDOD14	6.14E-10	0.502782	0.114	0.034	9.8E-06	Saliva_Duc	ENDOD1
MAGED25	6.63E-10	0.749929	0.297	0.155	1.06E-05	Saliva_Duc	MAGED2
SEMA5A2	6.67E-10	0.450488	0.097	0.026	1.06E-05	Saliva_Duc	SEMA5A
SNHG82	7.03E-10	0.686452	0.465	0.297	1.12E-05	Saliva_Duc	SNHG8
ANO61	8.25E-10	0.630569	0.173	0.067	1.32E-05	Saliva_Duc	ANO6
TMEM237:	8.87E-10	0.617714	0.13	0.042	1.42E-05	Saliva_Duc	TMEM237
EID13	9.45E-10	0.743041	0.519	0.358	1.51E-05	Saliva_Duc	EID1
TMEM8B1	9.68E-10	0.511153	0.119	0.037	1.54E-05	Saliva_Duc	TMEM8B
ITGA13	9.83E-10	0.314528	0.092	0.024	1.57E-05	Saliva_Duc	ITGA1
FAM210B5	1.01E-09	0.794376	0.211	0.094	1.6E-05	Saliva_Duc	FAM210B
MPPED2	1.02E-09	0.313624	0.07	0.015	1.62E-05	Saliva_Duc	MPPED2
COL6A12	1.09E-09	0.435083	0.297	0.142	1.75E-05	Saliva_Duc	COL6A1
NT5C4	1.11E-09	0.707776	0.259	0.125	1.77E-05	Saliva_Duc	NT5C
RABAC17	1.13E-09	0.61576	0.568	0.405	1.81E-05	Saliva_Duc	RABAC1
DHRS31	1.26E-09	0.642532	0.292	0.149	2.01E-05	Saliva_Duc	DHRS3
RPS6KA51	1.37E-09	0.641765	0.173	0.068	2.18E-05	Saliva_Duc	RPS6KA5
JADE12	1.39E-09	0.56866	0.151	0.055	2.22E-05	Saliva_Duc	JADE1
FHL31	1.61E-09	0.559301	0.124	0.04	2.56E-05	Saliva_Duc	FHL3
C16orf451	1.86E-09	0.627302	0.168	0.064	2.97E-05	Saliva_Duc	C16orf45
BEX41	1.87E-09	1.011694	0.303	0.17	2.99E-05	Saliva_Duc	BEX4
C1R2	1.96E-09	0.395087	0.254	0.111	3.12E-05	Saliva_Duc	C1R
PLCB43	2.33E-09	0.683974	0.151	0.056	3.71E-05	Saliva_Duc	PLCB4

BOK	2.41E-09	0.282142	0.059	0.012	3.84E-05	Saliva_Duc	BOK
RPL82	2.6E-09	0.314965	0.984	0.849	4.14E-05	Saliva_Duc	RPL8
PKP44	3.02E-09	0.767235	0.184	0.078	4.82E-05	Saliva_Duc	PKP4
FIGN1	3.86E-09	0.437874	0.092	0.025	6.16E-05	Saliva_Duc	FIGN
RPS131	4.44E-09	0.314723	0.978	0.817	7.09E-05	Saliva_Duc	RPS13
IRX52	4.58E-09	0.507749	0.097	0.028	7.31E-05	Saliva_Duc	IRX5
ARID5B2	4.97E-09	0.524179	0.438	0.269	7.93E-05	Saliva_Duc	ARID5B
DSP2	5.61E-09	0.658393	0.314	0.174	8.94E-05	Saliva_Duc	DSP
TFCP2L13	6.22E-09	0.677346	0.135	0.048	9.92E-05	Saliva_Duc	TFCP2L1
DDX178	6.39E-09	0.860012	0.481	0.354	0.000102	Saliva_Duc	DDX17
WNK3	7.15E-09	0.340246	0.07	0.017	0.000114	Saliva_Duc	WNK3
EPPK1	7.45E-09	0.540958	0.097	0.028	0.000119	Saliva_Duc	EPPK1
LMNA6	7.86E-09	0.382889	0.551	0.366	0.000125	Saliva_Duc	LMNA
RARB	7.87E-09	0.275274	0.049	0.009	0.000126	Saliva_Duc	RARB
PSMB15	7.92E-09	0.631014	0.535	0.4	0.000126	Saliva_Duc	PSMB1
KDSR3	8.09E-09	0.71244	0.254	0.13	0.000129	Saliva_Duc	KDSR
BTF32	8.22E-09	0.402645	0.838	0.712	0.000131	Saliva_Duc	BTF3
WTIP1	9.3E-09	0.313899	0.081	0.021	0.000148	Saliva_Duc	WTIP
GSTO12	1E-08	0.675703	0.357	0.213	0.00016	Saliva_Duc	GSTO1
BEX1	1.1E-08	0.400864	0.076	0.019	0.000176	Saliva_Duc	BEX1
GNAQ4	1.42E-08	0.841899	0.292	0.164	0.000227	Saliva_Duc	GNAQ
CD592	1.43E-08	0.411033	0.697	0.515	0.000228	Saliva_Duc	CD59
RAB27A2	1.56E-08	0.56998	0.162	0.065	0.000249	Saliva_Duc	RAB27A
KRTCAP33	1.59E-08	0.637859	0.184	0.081	0.000254	Saliva_Duc	KRTCAP3
RPL373	1.61E-08	0.362441	0.962	0.848	0.000257	Saliva_Duc	RPL37
RAB343	2.01E-08	0.708078	0.216	0.102	0.000321	Saliva_Duc	RAB34
FGF22	2.09E-08	0.340522	0.076	0.019	0.000333	Saliva_Duc	FGF2
RPL34	2.12E-08	0.261112	1	0.919	0.000339	Saliva_Duc	RPL34
SSR33	2.22E-08	0.686015	0.427	0.301	0.000353	Saliva_Duc	SSR3
AARD	2.35E-08	0.315846	0.065	0.015	0.000376	Saliva_Duc	AARD
SRP143	2.44E-08	0.431605	0.8	0.701	0.000389	Saliva_Duc	SRP14
HSPG22	2.72E-08	0.656444	0.232	0.114	0.000433	Saliva_Duc	HSPG2
FZD12	3.03E-08	0.448499	0.081	0.022	0.000484	Saliva_Duc	FZD1
CTSD3	3.13E-08	0.410994	0.443	0.293	0.0005	Saliva_Duc	CTSD
GTF2I8	3.48E-08	0.787865	0.276	0.154	0.000556	Saliva_Duc	GTF2I
NPAS3	3.68E-08	0.26881	0.049	0.01	0.000586	Saliva_Duc	NPAS3
PUF60	3.68E-08	0.735089	0.281	0.157	0.000587	Saliva_Duc	PUF60
ATP5J7	4.15E-08	0.544046	0.557	0.437	0.000662	Saliva_Duc	ATP5J
CCDC1073	4.15E-08	0.728839	0.308	0.176	0.000662	Saliva_Duc	CCDC107
INTU1	4.24E-08	0.45451	0.086	0.025	0.000677	Saliva_Duc	INTU
LIFR1	4.42E-08	0.655456	0.157	0.063	0.000705	Saliva_Duc	LIFR
RPS123	4.79E-08	0.256313	0.984	0.861	0.000763	Saliva_Duc	RPS12
IRX34	5.12E-08	0.862285	0.168	0.073	0.000816	Saliva_Duc	IRX3
PAK31	6.51E-08	0.281136	0.054	0.012	0.001039	Saliva_Duc	PAK3
CELSR2	6.57E-08	0.260725	0.059	0.014	0.001048	Saliva_Duc	CELSR2
MAP44	6.62E-08	0.720856	0.243	0.129	0.001056	Saliva_Duc	MAP4
TOMM71	6.98E-08	0.43467	0.724	0.582	0.001113	Saliva_Duc	TOMM7

ARHGEF25	7.42E-08	0.30641	0.049	0.01	0.001184	Saliva_Duc	ARHGEF25
PHLDA31	7.52E-08	0.60541	0.232	0.117	0.001199	Saliva_Duc	PHLDA3
GSTP15	7.98E-08	0.361704	0.73	0.557	0.001272	Saliva_Duc	GSTP1
NDUFS64	8.09E-08	0.746948	0.438	0.324	0.00129	Saliva_Duc	NDUFS6
CHCHD107	8.14E-08	0.593498	0.384	0.245	0.001297	Saliva_Duc	CHCHD10
DTX31	9.53E-08	0.479777	0.076	0.021	0.00152	Saliva_Duc	DTX3
SERPING13	1.01E-07	0.385931	0.319	0.173	0.001605	Saliva_Duc	SERPING1
COX7A15	1.02E-07	0.642474	0.259	0.139	0.001621	Saliva_Duc	COX7A1
ZNF5031	1.06E-07	0.612584	0.162	0.069	0.001689	Saliva_Duc	ZNF503
SYNE14	1.11E-07	0.44955	0.146	0.057	0.001772	Saliva_Duc	SYNE1
WIPF3	1.17E-07	0.496447	0.081	0.024	0.001866	Saliva_Duc	WIPF3
FGFR22	1.18E-07	0.602909	0.108	0.037	0.001876	Saliva_Duc	FGFR2
HCFC1R12	1.22E-07	0.68094	0.254	0.136	0.001949	Saliva_Duc	HCFC1R1
NTN42	1.57E-07	0.644966	0.151	0.063	0.002508	Saliva_Duc	NTN4
FER1	1.58E-07	0.448414	0.119	0.043	0.002517	Saliva_Duc	FER
EBF4	1.8E-07	0.294387	0.038	0.007	0.002874	Saliva_Duc	EBF4
NREP1	1.83E-07	0.510478	0.114	0.04	0.002921	Saliva_Duc	NREP
RPL292	2.07E-07	0.268063	0.957	0.829	0.003296	Saliva_Duc	RPL29
NSG1	2.14E-07	0.438176	0.108	0.038	0.003406	Saliva_Duc	NSG1
FBLIM1	2.31E-07	0.651109	0.146	0.061	0.003682	Saliva_Duc	FBLIM1
PARD6G	2.39E-07	0.421096	0.065	0.017	0.003818	Saliva_Duc	PARD6G
ZRANB24	2.67E-07	0.855018	0.303	0.187	0.004258	Saliva_Duc	ZRANB2
FOXC13	2.82E-07	0.668704	0.216	0.112	0.004504	Saliva_Duc	FOXC1
ID41	2.83E-07	0.609865	0.173	0.078	0.004515	Saliva_Duc	ID4
MID1	2.84E-07	0.363115	0.081	0.024	0.004523	Saliva_Duc	MID1
SERTAD4.A	2.95E-07	0.565228	0.124	0.048	0.004708	Saliva_Duc	SERTAD4.AS1
NDUFB88	3.51E-07	0.731724	0.292	0.178	0.005598	Saliva_Duc	NDUFB8
NCL4	3.68E-07	0.595998	0.405	0.271	0.005874	Saliva_Duc	NCL
COL7A11	3.89E-07	0.367413	0.065	0.017	0.006209	Saliva_Duc	COL7A1
FJX1	4.03E-07	0.25991	0.038	0.007	0.006425	Saliva_Duc	FJX1
MYADM4	4.11E-07	0.606414	0.27	0.15	0.00655	Saliva_Duc	MYADM
SSB5	4.15E-07	0.588217	0.324	0.198	0.006621	Saliva_Duc	SSB
RSBN1L1	4.16E-07	0.678636	0.292	0.175	0.006631	Saliva_Duc	RSBN1L
MBNL1.AS	4.25E-07	0.325477	0.049	0.011	0.006778	Saliva_Duc	MBNL1.AS1
SMARCD31	4.51E-07	0.387532	0.086	0.027	0.007199	Saliva_Duc	SMARCD3
PLXNA11	4.61E-07	0.323926	0.076	0.022	0.007347	Saliva_Duc	PLXNA1
IRF2BPL	4.79E-07	0.625973	0.168	0.076	0.007644	Saliva_Duc	IRF2BPL
PRELP2	5.35E-07	0.343098	0.114	0.041	0.00853	Saliva_Duc	PRELP
DPYSL23	5.45E-07	0.475971	0.205	0.099	0.008685	Saliva_Duc	DPYSL2
TBC1D2	5.72E-07	0.468979	0.092	0.03	0.009118	Saliva_Duc	TBC1D2
MTRNR2L6	5.73E-07	0.344526	0.076	0.022	0.009132	Saliva_Duc	MTRNR2L6
COMMD6	5.75E-07	0.466905	0.616	0.474	0.009172	Saliva_Duc	COMMD6
DOCK51	5.91E-07	0.394697	0.092	0.03	0.009425	Saliva_Duc	DOCK5
AGO23	6.02E-07	0.540809	0.13	0.052	0.009594	Saliva_Duc	AGO2
GABBR11	6.21E-07	0.287668	0.07	0.02	0.009911	Saliva_Duc	GABBR1
FHL12	6.47E-07	0.442612	0.249	0.129	0.010315	Saliva_Duc	FHL1
LAMB24	8.03E-07	0.495505	0.178	0.083	0.012802	Saliva_Duc	LAMB2

CHCHD23	8.06E-07	0.425941	0.735	0.621	0.012853	Saliva_Duc	CHCHD2
ECI15	8.4E-07	0.635692	0.243	0.139	0.013402	Saliva_Duc	ECI1
ANKS1A	8.61E-07	0.542193	0.119	0.046	0.013724	Saliva_Duc	ANKS1A
ITM2A3	9.28E-07	0.398861	0.259	0.136	0.014804	Saliva_Duc	ITM2A
CASKIN21	9.29E-07	0.295799	0.065	0.018	0.014819	Saliva_Duc	CASKIN2
HRAS	9.34E-07	0.674159	0.184	0.09	0.014899	Saliva_Duc	HRAS
NME37	9.63E-07	0.572612	0.33	0.209	0.015353	Saliva_Duc	NME3
CHD63	9.64E-07	0.653769	0.168	0.079	0.015371	Saliva_Duc	CHD6
PTPN142	1.1E-06	0.678505	0.135	0.058	0.017577	Saliva_Duc	PTPN14
U2SURP5	1.17E-06	0.738129	0.281	0.172	0.018684	Saliva_Duc	U2SURP
NDUFA51	1.36E-06	0.670303	0.362	0.252	0.02168	Saliva_Duc	NDUFA5
HSPA4L	1.36E-06	0.521784	0.141	0.06	0.021761	Saliva_Duc	HSPA4L
IL34	1.42E-06	0.393032	0.081	0.026	0.022673	Saliva_Duc	IL34
SMIM103	1.47E-06	0.335956	0.07	0.021	0.023421	Saliva_Duc	SMIM10
FUS6	1.99E-06	0.635475	0.395	0.277	0.031767	Saliva_Duc	FUS
SRSF105	2.1E-06	0.725686	0.259	0.157	0.033562	Saliva_Duc	SRSF10
RBPMS1	2.16E-06	0.837744	0.286	0.184	0.034438	Saliva_Duc	RBPMS
HPS5	2.23E-06	0.592699	0.135	0.059	0.035563	Saliva_Duc	HPS5
PSMA75	2.3E-06	0.456392	0.568	0.464	0.036758	Saliva_Duc	PSMA7
EIF3E1	2.34E-06	0.391217	0.578	0.449	0.037388	Saliva_Duc	EIF3E
ANKRD65	2.37E-06	0.351968	0.092	0.032	0.037865	Saliva_Duc	ANKRD65
TTC34	2.4E-06	0.591338	0.351	0.234	0.038226	Saliva_Duc	TTC3
PROS12	2.5E-06	0.346912	0.114	0.044	0.039816	Saliva_Duc	PROS1
CDKN1C1	2.61E-06	0.601331	0.27	0.161	0.041573	Saliva_Duc	CDKN1C
ATL11	2.64E-06	0.474483	0.103	0.039	0.042053	Saliva_Duc	ATL1
PLK21	2.78E-06	0.68128	0.173	0.086	0.04429	Saliva_Duc	PLK2
NDUFB76	2.82E-06	0.632051	0.459	0.373	0.044952	Saliva_Duc	NDUFB7
PSTPIP2	2.92E-06	0.42854	0.065	0.019	0.046588	Saliva_Duc	PSTPIP2
RNF1502	2.95E-06	0.271839	0.059	0.016	0.047025	Saliva_Duc	RNF150
TRMT113	3.07E-06	0.494106	0.103	0.039	0.048899	Saliva_Duc	TRMT11
MYOF3	3.09E-06	0.476506	0.135	0.059	0.049286	Saliva_Duc	MYOF
LPCAT23	3.11E-06	0.470687	0.103	0.039	0.04957	Saliva_Duc	LPCAT2
ATRX6	3.13E-06	0.68164	0.314	0.207	0.049861	Saliva_Duc	ATRX
MTMR11	3.24E-06	0.265656	0.049	0.012	0.051622	Saliva_Duc	MTMR11
ZC3H63	3.63E-06	0.570299	0.151	0.071	0.057893	Saliva_Duc	ZC3H6
TSTD15	3.67E-06	0.524768	0.389	0.272	0.058457	Saliva_Duc	TSTD1
ETV51	3.84E-06	0.470395	0.097	0.036	0.061267	Saliva_Duc	ETV5
CHPT13	3.91E-06	0.59854	0.314	0.206	0.062312	Saliva_Duc	CHPT1
ZNF37A7	4.04E-06	0.583336	0.13	0.056	0.064374	Saliva_Duc	ZNF37A
LUC7L35	4.19E-06	0.698438	0.373	0.266	0.066867	Saliva_Duc	LUC7L3
MRVI11	4.22E-06	0.256776	0.059	0.017	0.067276	Saliva_Duc	MRVI1
TRAF51	4.22E-06	0.408736	0.103	0.039	0.067367	Saliva_Duc	TRAF5
TNS32	4.65E-06	0.403164	0.086	0.03	0.074086	Saliva_Duc	TNS3
PRPSAP13	4.79E-06	0.71511	0.211	0.119	0.076321	Saliva_Duc	PRPSAP1
SLC38A14	4.9E-06	0.649882	0.189	0.099	0.078149	Saliva_Duc	SLC38A1
CRIM13	5.09E-06	0.591254	0.162	0.079	0.081121	Saliva_Duc	CRIM1
NRG1	5.86E-06	0.346503	0.065	0.02	0.093412	Saliva_Duc	NRG1

TMEM168:	6.75E-06	0.467983	0.097	0.037	0.107612	Saliva_Duc	TMEM168
MEF2C4	7.37E-06	0.398826	0.168	0.081	0.117466	Saliva_Duc	MEF2C
MZT2B3	7.48E-06	0.440428	0.568	0.461	0.119231	Saliva_Duc	MZT2B
ASPH5	7.57E-06	0.586178	0.292	0.191	0.120785	Saliva_Duc	ASPH
LTBR3	8.18E-06	0.572801	0.173	0.089	0.130413	Saliva_Duc	LTBR
MANBAL	8.36E-06	0.636944	0.178	0.094	0.13332	Saliva_Duc	MANBAL
TUBB61	8.5E-06	0.490874	0.303	0.191	0.135504	Saliva_Duc	TUBB6
HOOK23	9.6E-06	0.635132	0.189	0.103	0.153184	Saliva_Duc	HOOK2
LYST2	1.08E-05	0.568455	0.178	0.094	0.172969	Saliva_Duc	LYST
LGALS3BP5	1.09E-05	0.404341	0.281	0.171	0.17358	Saliva_Duc	LGALS3BP
TNRC6B5	1.21E-05	0.714794	0.27	0.172	0.193303	Saliva_Duc	TNRC6B
SORBS25	1.23E-05	0.860816	0.2	0.113	0.196549	Saliva_Duc	SORBS2
KANK23	1.27E-05	0.342458	0.108	0.044	0.203045	Saliva_Duc	KANK2
CENPC5	1.41E-05	0.477259	0.135	0.062	0.224389	Saliva_Duc	CENPC
PLD33	1.42E-05	0.556586	0.265	0.165	0.227105	Saliva_Duc	PLD3
MICAL31	1.48E-05	0.399128	0.081	0.029	0.236348	Saliva_Duc	MICAL3
PRMT21	1.51E-05	0.537264	0.259	0.158	0.240613	Saliva_Duc	PRMT2
CHADL	1.63E-05	0.293586	0.054	0.015	0.260379	Saliva_Duc	CHADL
PCNXL45	1.64E-05	0.517108	0.13	0.059	0.261049	Saliva_Duc	PCNXL4
ACSL41	1.65E-05	0.287383	0.086	0.032	0.262525	Saliva_Duc	ACSL4
NDUFB107	1.66E-05	0.429335	0.486	0.385	0.264011	Saliva_Duc	NDUFB10
CTSF2	1.87E-05	0.683482	0.249	0.157	0.298954	Saliva_Duc	CTSF
ABI22	1.94E-05	0.606727	0.168	0.088	0.309494	Saliva_Duc	ABI2
SIVA14	1.95E-05	0.608096	0.373	0.274	0.31171	Saliva_Duc	SIVA1
AKAP14	2.14E-05	0.261145	0.151	0.072	0.341806	Saliva_Duc	AKAP1
SRRM27	2.18E-05	0.570535	0.438	0.348	0.347409	Saliva_Duc	SRRM2
CEP683	2.23E-05	0.46508	0.103	0.042	0.354907	Saliva_Duc	CEP68
FAM127B2	2.33E-05	0.523961	0.157	0.08	0.372112	Saliva_Duc	FAM127B
NDUFC12	2.34E-05	0.623612	0.373	0.281	0.372782	Saliva_Duc	NDUFC1
RCN2	2.36E-05	0.594071	0.249	0.155	0.376169	Saliva_Duc	RCN2
SHROOM3	2.4E-05	0.511163	0.114	0.05	0.382655	Saliva_Duc	SHROOM3
MIEF2	2.47E-05	0.316314	0.049	0.013	0.394527	Saliva_Duc	MIEF2
ZC3H134	2.5E-05	0.569274	0.211	0.122	0.398927	Saliva_Duc	ZC3H13
ATP5G36	2.54E-05	0.426646	0.465	0.362	0.404357	Saliva_Duc	ATP5G3
TMEM139	2.61E-05	0.421393	0.054	0.016	0.416829	Saliva_Duc	TMEM139
EFEMP21	2.88E-05	0.469725	0.141	0.068	0.459729	Saliva_Duc	EFEMP2
MAP92	3.02E-05	0.478122	0.097	0.04	0.480881	Saliva_Duc	MAP9
SCOC	3.18E-05	0.652132	0.259	0.172	0.507898	Saliva_Duc	SCOC
PNN4	3.58E-05	0.668469	0.303	0.212	0.570705	Saliva_Duc	PNN
CIC	4.32E-05	0.513576	0.103	0.044	0.689546	Saliva_Duc	CIC
KIF13A2	4.43E-05	0.473129	0.114	0.051	0.706659	Saliva_Duc	KIF13A
SLITRK51	4.43E-05	0.364604	0.065	0.022	0.706992	Saliva_Duc	SLITRK5
CBX62	4.44E-05	0.469788	0.162	0.084	0.70772	Saliva_Duc	CBX6
NTRK32	4.56E-05	0.385048	0.07	0.025	0.727122	Saliva_Duc	NTRK3
GAMT1	4.92E-05	0.58365	0.157	0.083	0.784303	Saliva_Duc	GAMT
SUMO23	5.08E-05	0.418499	0.665	0.602	0.809644	Saliva_Duc	SUMO2
MICU32	5.1E-05	0.347852	0.076	0.028	0.813373	Saliva_Duc	MICU3

BCL11B2	5.19E-05	0.266857	0.076	0.027	0.827813	Saliva_Duc	BCL11B
NDUFAB16	5.19E-05	0.555134	0.335	0.246	0.828266	Saliva_Duc	NDUFAB1
EOGT	5.29E-05	0.378456	0.076	0.028	0.843653	Saliva_Duc	EOGT
PJA27	5.42E-05	0.656777	0.238	0.152	0.865099	Saliva_Duc	PJA2
SSBP15	5.67E-05	0.599075	0.368	0.283	0.904743	Saliva_Duc	SSBP1
PSMD11	5.76E-05	0.54092	0.184	0.104	0.919233	Saliva_Duc	PSMD1
C8orf597	5.85E-05	0.79885	0.314	0.235	0.932947	Saliva_Duc	C8orf59
ARHGAP52	5.94E-05	0.825487	0.286	0.205	0.947944	Saliva_Duc	ARHGAP5
TMEM472	5.97E-05	0.439644	0.119	0.055	0.951839	Saliva_Duc	TMEM47
GLG14	6.12E-05	0.615152	0.211	0.127	0.976152	Saliva_Duc	GLG1
HNRNPR4	6.24E-05	0.638675	0.281	0.193	0.99572	Saliva_Duc	HNRNPR
WFDC11	6.39E-05	0.382271	0.081	0.032	1	Saliva_Duc	WFDC1
ATP5G15	6.88E-05	0.59991	0.351	0.261	1	Saliva_Duc	ATP5G1
LAYN1	7.53E-05	0.359263	0.076	0.028	1	Saliva_Duc	LAYN
CFL21	7.55E-05	0.616701	0.173	0.097	1	Saliva_Duc	CFL2
PLEKHA4	7.76E-05	0.36677	0.076	0.028	1	Saliva_Duc	PLEKHA4
SEPT74	7.82E-05	0.412277	0.438	0.34	1	Saliva_Duc	SEPT7
ICA1L	7.84E-05	0.257513	0.065	0.023	1	Saliva_Duc	ICA1L
SLC27A33	8.19E-05	0.287457	0.076	0.028	1	Saliva_Duc	SLC27A3
CCDC148	9.21E-05	0.312623	0.043	0.012	1	Saliva_Duc	CCDC148
AKAP7	9.38E-05	0.294302	0.07	0.026	1	Saliva_Duc	AKAP7
ADARB12	9.52E-05	0.276395	0.065	0.023	1	Saliva_Duc	ADARB1
LINC00662	9.95E-05	0.428813	0.108	0.049	1	Saliva_Duc	LINC00662
RALGAPB	0.0001	0.490577	0.119	0.057	1	Saliva_Duc	RALGAPB
AKAP95	0.000101	0.607387	0.351	0.271	1	Saliva_Duc	AKAP9
UBE2M3	0.000101	0.63198	0.249	0.167	1	Saliva_Duc	UBE2M
SUN2	0.00011	0.318988	0.216	0.125	1	Saliva_Duc	SUN2
DPYSL33	0.000112	0.312812	0.119	0.055	1	Saliva_Duc	DPYSL3
TBX11	0.000115	0.271322	0.065	0.023	1	Saliva_Duc	TBX1
PEG10	0.000117	0.259874	0.065	0.023	1	Saliva_Duc	PEG10
PAPSS12	0.000121	0.613558	0.184	0.11	1	Saliva_Duc	PAPSS1
C14orf80	0.000125	0.251847	0.065	0.023	1	Saliva_Duc	C14orf80
IQCA1	0.000129	0.264047	0.049	0.015	1	Saliva_Duc	IQCA1
FBXO92	0.000135	0.586668	0.168	0.096	1	Saliva_Duc	FBXO9
COX6C3	0.000138	0.323531	0.627	0.542	1	Saliva_Duc	COX6C
LRP52	0.000138	0.412137	0.097	0.043	1	Saliva_Duc	LRP5
TSHZ24	0.000142	0.524716	0.189	0.11	1	Saliva_Duc	TSHZ2
SOCS5	0.000143	0.399278	0.092	0.04	1	Saliva_Duc	SOCS5
CAMSAP2	0.00015	0.475945	0.141	0.074	1	Saliva_Duc	CAMSAP2
CDH13	0.000154	0.498762	0.141	0.075	1	Saliva_Duc	CDH1
VPS366	0.000155	0.541496	0.189	0.113	1	Saliva_Duc	VPS36
PHF35	0.000158	0.567781	0.211	0.133	1	Saliva_Duc	PHF3
FN3KRP	0.000159	0.403699	0.108	0.051	1	Saliva_Duc	FN3KRP
MARCKS3	0.000164	0.413876	0.378	0.284	1	Saliva_Duc	MARCKS
NDUFB91	0.000165	0.483892	0.432	0.346	1	Saliva_Duc	NDUFB9
DOCK71	0.000166	0.428104	0.103	0.047	1	Saliva_Duc	DOCK7
MT.ND69	0.000169	0.411705	0.189	0.111	1	Saliva_Duc	MT.ND6

PCNX	0.000172	0.377395	0.103	0.047	1	Saliva_Duc	PCNX
ERBB23	0.000179	0.658537	0.119	0.059	1	Saliva_Duc	ERBB2
HECTD21	0.000183	0.285999	0.065	0.024	1	Saliva_Duc	HECTD2
RNF1801	0.000185	0.342635	0.065	0.024	1	Saliva_Duc	RNF180
FAM13B	0.000193	0.344599	0.103	0.047	1	Saliva_Duc	FAM13B
CEP97	0.00021	0.259147	0.059	0.021	1	Saliva_Duc	CEP97
CSF12	0.000214	0.315106	0.081	0.033	1	Saliva_Duc	CSF1
MFSD101	0.000216	0.587708	0.2	0.124	1	Saliva_Duc	MFSD10
CEP78	0.000222	0.340138	0.086	0.037	1	Saliva_Duc	CEP78
C9orf401	0.000227	0.294263	0.054	0.018	1	Saliva_Duc	C9orf40
IFFO2	0.000246	0.309774	0.065	0.024	1	Saliva_Duc	IFFO2
SGCB2	0.000246	0.582934	0.13	0.068	1	Saliva_Duc	SGCB
FOXP41	0.000247	0.316084	0.065	0.024	1	Saliva_Duc	FOXP4
CENPB2	0.00025	0.425563	0.103	0.048	1	Saliva_Duc	CENPB
FGFRL1	0.000273	0.369787	0.07	0.028	1	Saliva_Duc	FGFRL1
ZMYND11	0.000277	0.460456	0.146	0.08	1	Saliva_Duc	ZMYND11
PSMA34	0.000287	0.582038	0.27	0.194	1	Saliva_Duc	PSMA3
HLF1	0.000287	0.35924	0.076	0.031	1	Saliva_Duc	HLF
LRPAP16	0.000293	0.552209	0.351	0.27	1	Saliva_Duc	LRPAP1
MRFAP11	0.000301	0.583208	0.346	0.267	1	Saliva_Duc	MRFAP1
PRDX32	0.000303	0.521437	0.232	0.157	1	Saliva_Duc	PRDX3
BMP41	0.000303	0.293864	0.059	0.021	1	Saliva_Duc	BMP4
DSTYK1	0.000305	0.383162	0.092	0.041	1	Saliva_Duc	DSTYK
MRPS264	0.000322	0.549503	0.227	0.15	1	Saliva_Duc	MRPS26
SMAD54	0.000332	0.516464	0.162	0.094	1	Saliva_Duc	SMAD5
MT1F	0.000333	0.302228	0.124	0.063	1	Saliva_Duc	MT1F
MBNL24	0.000342	0.617507	0.189	0.119	1	Saliva_Duc	MBNL2
PUM13	0.000344	0.614179	0.216	0.144	1	Saliva_Duc	PUM1
PCM12	0.000354	0.502855	0.238	0.158	1	Saliva_Duc	PCM1
APPL15	0.00036	0.65362	0.195	0.124	1	Saliva_Duc	APPL1
TTC146	0.000373	0.536751	0.173	0.102	1	Saliva_Duc	TTC14
KIF5B3	0.000374	0.639101	0.357	0.291	1	Saliva_Duc	KIF5B
PRKDC5	0.000378	0.465709	0.168	0.099	1	Saliva_Duc	PRKDC
COX7C2	0.000379	0.300611	0.714	0.638	1	Saliva_Duc	COX7C
TACC14	0.000401	0.43686	0.249	0.167	1	Saliva_Duc	TACC1
PPIB4	0.000437	0.304731	0.486	0.395	1	Saliva_Duc	PPIB
SON4	0.000438	0.408251	0.486	0.41	1	Saliva_Duc	SON
CBX71	0.000445	0.380853	0.081	0.035	1	Saliva_Duc	CBX7
SLC25A364	0.000478	0.488638	0.195	0.123	1	Saliva_Duc	SLC25A36
ZFP144	0.000489	0.348967	0.076	0.032	1	Saliva_Duc	ZFP14
ERH1	0.000489	0.51457	0.384	0.312	1	Saliva_Duc	ERH
PHIP2	0.000493	0.599316	0.243	0.17	1	Saliva_Duc	PHIP
CYB5A4	0.000507	0.550146	0.259	0.183	1	Saliva_Duc	CYB5A
BCLAF17	0.000517	0.610994	0.27	0.2	1	Saliva_Duc	BCLAF1
FLNB3	0.00054	0.469811	0.141	0.079	1	Saliva_Duc	FLNB
TRIP6	0.0006	0.524719	0.211	0.14	1	Saliva_Duc	TRIP6
AFAP1L2	0.000624	0.302454	0.07	0.029	1	Saliva_Duc	AFAP1L2

TUSC3	0.000641	0.40454	0.114	0.059	1	Saliva_Duc	TUSC3
CSNK1G3	0.000659	0.409454	0.114	0.058	1	Saliva_Duc	CSNK1G3
FBLN51	0.000679	0.280695	0.13	0.068	1	Saliva_Duc	FBLN5
MLH34	0.000697	0.387272	0.097	0.047	1	Saliva_Duc	MLH3
SPRED12	0.000744	0.341072	0.092	0.043	1	Saliva_Duc	SPRED1
NEDD81	0.000758	0.411302	0.481	0.423	1	Saliva_Duc	NEDD8
LUC7L25	0.000769	0.435106	0.103	0.052	1	Saliva_Duc	LUC7L2
PTPRE2	0.000774	0.252217	0.108	0.054	1	Saliva_Duc	PTPRE
PHC21	0.000807	0.471691	0.124	0.068	1	Saliva_Duc	PHC2
CRBN2	0.000816	0.484021	0.216	0.143	1	Saliva_Duc	CRBN
PHYHD13	0.000823	0.452286	0.114	0.06	1	Saliva_Duc	PHYHD1
CTNNAL11	0.000831	0.509943	0.151	0.09	1	Saliva_Duc	CTNNAL1
THUMPD1	0.000834	0.489925	0.168	0.103	1	Saliva_Duc	THUMPD1
C5orf24	0.000839	0.477466	0.178	0.112	1	Saliva_Duc	C5orf24
HMGN33	0.000851	0.348268	0.362	0.279	1	Saliva_Duc	HMGN3
GNA114	0.000875	0.526444	0.124	0.069	1	Saliva_Duc	GNA11
SETBP12	0.000902	0.295467	0.081	0.037	1	Saliva_Duc	SETBP1
ACP51	0.000913	0.371285	0.108	0.055	1	Saliva_Duc	ACP5
ZBTB203	0.000926	0.45288	0.205	0.135	1	Saliva_Duc	ZBTB20
DICER15	0.00098	0.467984	0.168	0.104	1	Saliva_Duc	DICER1
SENP65	0.000995	0.571423	0.173	0.109	1	Saliva_Duc	SENP6
MTDH5	0.001029	0.435269	0.384	0.309	1	Saliva_Duc	MTDH
ZSCAN183	0.001045	0.385986	0.141	0.081	1	Saliva_Duc	ZSCAN18
NF12	0.00105	0.551214	0.146	0.087	1	Saliva_Duc	NF1
OCIAD15	0.001077	0.521707	0.319	0.253	1	Saliva_Duc	OCIAD1
CERS6	0.001092	0.330498	0.092	0.045	1	Saliva_Duc	CERS6
RAB4A2	0.001095	0.588526	0.27	0.209	1	Saliva_Duc	RAB4A
MPHOSPH	0.001111	0.534084	0.324	0.254	1	Saliva_Duc	MPHOSPH8
NENF4	0.001112	0.455459	0.395	0.329	1	Saliva_Duc	NENF
TMTC32	0.001141	0.346643	0.081	0.038	1	Saliva_Duc	TMTC3
ZFPM12	0.001152	0.292362	0.065	0.027	1	Saliva_Duc	ZFPM1
DAB2IP2	0.00116	0.316148	0.065	0.027	1	Saliva_Duc	DAB2IP
RSF13	0.001214	0.550376	0.249	0.182	1	Saliva_Duc	RSF1
PHLDB12	0.001234	0.398411	0.097	0.049	1	Saliva_Duc	PHLDB1
AES2	0.001245	0.317952	0.465	0.396	1	Saliva_Duc	AES
ANXA55	0.001266	0.396544	0.4	0.318	1	Saliva_Duc	ANXA5
PTPRF4	0.001267	0.540744	0.168	0.107	1	Saliva_Duc	PTPRF
SLC25A11	0.001307	0.474934	0.178	0.114	1	Saliva_Duc	SLC25A11
CREBZF2	0.001329	0.564854	0.13	0.074	1	Saliva_Duc	CREBZF
VEGFB7	0.00133	0.43956	0.162	0.1	1	Saliva_Duc	VEGFB
CSRP22	0.00137	0.380199	0.141	0.081	1	Saliva_Duc	CSRP2
CTPS1	0.001397	0.286499	0.065	0.028	1	Saliva_Duc	CTPS1
ATP1A14	0.001397	0.523519	0.324	0.256	1	Saliva_Duc	ATP1A1
INPP4B1	0.001403	0.408881	0.108	0.057	1	Saliva_Duc	INPP4B
IGF1R1	0.001415	0.447215	0.13	0.075	1	Saliva_Duc	IGF1R
LHFP3	0.001417	0.266256	0.151	0.087	1	Saliva_Duc	LHFP
ZNF135	0.001489	0.260848	0.038	0.012	1	Saliva_Duc	ZNF135

EIF4A2	0.001495	0.277747	0.551	0.486	1	Saliva_Duc	EIF4A2
LAPTM4B2	0.001551	0.483194	0.162	0.102	1	Saliva_Duc	LAPTM4B
BDP12	0.001563	0.393723	0.168	0.105	1	Saliva_Duc	BDP1
TYW5	0.001648	0.255459	0.07	0.031	1	Saliva_Duc	TYW5
SERINC14	0.001661	0.681328	0.259	0.197	1	Saliva_Duc	SERINC1
BMS11	0.001676	0.397342	0.119	0.066	1	Saliva_Duc	BMS1
ANAPC162	0.001688	0.464921	0.427	0.371	1	Saliva_Duc	ANAPC16
UBR4	0.001693	0.435251	0.146	0.088	1	Saliva_Duc	UBR4
SEC624	0.001704	0.398954	0.459	0.402	1	Saliva_Duc	SEC62
C14orf132	0.001714	0.260717	0.049	0.018	1	Saliva_Duc	C14orf132
PRMT12	0.001728	0.473567	0.265	0.192	1	Saliva_Duc	PRMT1
COPZ21	0.001779	0.423605	0.141	0.083	1	Saliva_Duc	COPZ2
SMTN1	0.001783	0.354727	0.086	0.042	1	Saliva_Duc	SMTN
RYK4	0.001803	0.398808	0.13	0.075	1	Saliva_Duc	RYK
CYBRD11	0.001819	0.349208	0.184	0.115	1	Saliva_Duc	CYBRD1
CCDC84	0.001835	0.303975	0.076	0.035	1	Saliva_Duc	CCDC84
DNAJC191	0.001884	0.636173	0.227	0.164	1	Saliva_Duc	DNAJC19
ARL6IP41	0.00189	0.295328	0.481	0.422	1	Saliva_Duc	ARL6IP4
LZTS22	0.001891	0.312475	0.086	0.042	1	Saliva_Duc	LZTS2
DDX185	0.001904	0.481372	0.276	0.204	1	Saliva_Duc	DDX18
PRPF81	0.001904	0.410062	0.162	0.101	1	Saliva_Duc	PRPF8
ORC41	0.001942	0.392827	0.114	0.062	1	Saliva_Duc	ORC4
CNN2	0.001956	0.415662	0.249	0.175	1	Saliva_Duc	CNN2
FAM127C	0.002004	0.30526	0.065	0.028	1	Saliva_Duc	FAM127C
ZDHHC172	0.002038	0.310674	0.076	0.035	1	Saliva_Duc	ZDHHC17
CARF	0.002132	0.288329	0.054	0.022	1	Saliva_Duc	CARF
PKM1	0.0022	0.386154	0.438	0.374	1	Saliva_Duc	PKM
ROGDI1	0.002223	0.427672	0.108	0.059	1	Saliva_Duc	ROGDI
KIDINS220	0.002223	0.40336	0.162	0.102	1	Saliva_Duc	KIDINS220
CLUAP12	0.002228	0.507142	0.108	0.06	1	Saliva_Duc	CLUAP1
RDX1	0.002236	0.55409	0.249	0.187	1	Saliva_Duc	RDX
BPTF5	0.002253	0.491359	0.227	0.164	1	Saliva_Duc	BPTF
TCEAL1	0.002317	0.287522	0.103	0.054	1	Saliva_Duc	TCEAL1
LAMA53	0.002446	0.260839	0.065	0.029	1	Saliva_Duc	LAMA5
MPHOSPH	0.002459	0.484685	0.146	0.09	1	Saliva_Duc	MPHOSPH10
PFN22	0.002503	0.549261	0.135	0.082	1	Saliva_Duc	PFN2
SRRM12	0.002516	0.501097	0.378	0.328	1	Saliva_Duc	SRRM1
NFATC2IP2	0.002517	0.352941	0.135	0.08	1	Saliva_Duc	NFATC2IP
SBDS	0.00258	0.473641	0.395	0.342	1	Saliva_Duc	SBDS
DGKH3	0.002624	0.284339	0.076	0.036	1	Saliva_Duc	DGKH
SLC1A53	0.002659	0.46454	0.13	0.078	1	Saliva_Duc	SLC1A5
PRICKLE12	0.002744	0.26431	0.065	0.029	1	Saliva_Duc	PRICKLE1
ARL4D	0.00275	0.446583	0.151	0.095	1	Saliva_Duc	ARL4D
PPP1R13L	0.002778	0.489146	0.124	0.072	1	Saliva_Duc	PPP1R13L
FAM20C3	0.002797	0.26265	0.065	0.029	1	Saliva_Duc	FAM20C
EIF56	0.002805	0.404456	0.416	0.36	1	Saliva_Duc	EIF5
TSLP	0.002902	0.348787	0.043	0.016	1	Saliva_Duc	TSLP

PSMB41	0.002997	0.563709	0.238	0.18	1	Saliva_Duc	PSMB4
MACF15	0.003002	0.413061	0.232	0.168	1	Saliva_Duc	MACF1
TUSC11	0.003012	0.405114	0.119	0.068	1	Saliva_Duc	TUSC1
RHOQ4	0.003105	0.557974	0.141	0.087	1	Saliva_Duc	RHOQ
PCSK72	0.003145	0.408722	0.173	0.112	1	Saliva_Duc	PCSK7
TTC5	0.003274	0.271314	0.07	0.033	1	Saliva_Duc	TTC5
FNDC3A4	0.003286	0.55581	0.173	0.117	1	Saliva_Duc	FNDC3A
ZNF6384	0.00348	0.584955	0.189	0.13	1	Saliva_Duc	ZNF638
LRP62	0.003575	0.461582	0.103	0.057	1	Saliva_Duc	LRP6
RBM256	0.003592	0.511203	0.297	0.24	1	Saliva_Duc	RBM25
FAM133B3	0.003595	0.476283	0.259	0.199	1	Saliva_Duc	FAM133B
MEF2A2	0.003606	0.598167	0.162	0.107	1	Saliva_Duc	MEF2A
TRPS13	0.003725	0.496104	0.141	0.088	1	Saliva_Duc	TRPS1
TTC282	0.003826	0.329762	0.07	0.034	1	Saliva_Duc	TTC28
BRD71	0.003849	0.435163	0.178	0.121	1	Saliva_Duc	BRD7
SEPT23	0.00385	0.57418	0.286	0.236	1	Saliva_Duc	SEPT2
YIF1A3	0.003863	0.476426	0.232	0.173	1	Saliva_Duc	YIF1A
MIB12	0.003981	0.430652	0.114	0.066	1	Saliva_Duc	MIB1
RNPC31	0.004082	0.315132	0.097	0.053	1	Saliva_Duc	RNPC3
PCLO1	0.004124	0.291751	0.054	0.023	1	Saliva_Duc	PCLO
FAM195B1	0.004134	0.513083	0.189	0.132	1	Saliva_Duc	FAM195B
MYO9A2	0.004239	0.382437	0.124	0.074	1	Saliva_Duc	MYO9A
PRDX62	0.004288	0.501114	0.411	0.374	1	Saliva_Duc	PRDX6
TBCA1	0.004293	0.502812	0.389	0.352	1	Saliva_Duc	TBCA
KRR13	0.004318	0.478441	0.189	0.133	1	Saliva_Duc	KRR1
TES	0.00433	0.530877	0.184	0.127	1	Saliva_Duc	TES
NKAP1	0.00433	0.490254	0.151	0.097	1	Saliva_Duc	NKAP
GOLGA45	0.004359	0.393893	0.297	0.236	1	Saliva_Duc	GOLGA4
CBX52	0.00439	0.57212	0.168	0.114	1	Saliva_Duc	CBX5
SDCCAG81	0.004446	0.463741	0.13	0.08	1	Saliva_Duc	SDCCAG8
RBM432	0.004492	0.293208	0.059	0.027	1	Saliva_Duc	RBM43
ANKRD13D	0.004527	0.364087	0.119	0.07	1	Saliva_Duc	ANKRD13D
HIRIP3	0.004561	0.319409	0.092	0.049	1	Saliva_Duc	HIRIP3
NAE1	0.00457	0.418126	0.157	0.101	1	Saliva_Duc	NAE1
DNM1L3	0.004879	0.423475	0.13	0.08	1	Saliva_Duc	DNM1L
SH3BGRL4	0.004891	0.511603	0.308	0.245	1	Saliva_Duc	SH3BGRL
NT5DC12	0.004963	0.513674	0.13	0.08	1	Saliva_Duc	NT5DC1
LANCL1	0.005107	0.397107	0.092	0.05	1	Saliva_Duc	LANCL1
FAR1	0.005141	0.430473	0.135	0.085	1	Saliva_Duc	FAR1
ANKRD124	0.005148	0.401908	0.373	0.324	1	Saliva_Duc	ANKRD12
NSA24	0.00515	0.50235	0.303	0.25	1	Saliva_Duc	NSA2
HLTF1	0.005264	0.291206	0.092	0.05	1	Saliva_Duc	HLTF
PGAM12	0.00529	0.531206	0.281	0.229	1	Saliva_Duc	PGAM1
GCSH3	0.005293	0.584463	0.178	0.126	1	Saliva_Duc	GCSH
SOBP1	0.005385	0.382622	0.076	0.038	1	Saliva_Duc	SOBP
GLIPR11	0.005463	0.344313	0.146	0.09	1	Saliva_Duc	GLIPR1
WSB13	0.005635	0.43606	0.308	0.25	1	Saliva_Duc	WSB1

FAM162A2	0.005786	0.543362	0.249	0.194	1	Saliva_Duc	FAM162A
PER32	0.005808	0.333629	0.114	0.067	1	Saliva_Duc	PER3
EZH12	0.005814	0.299586	0.081	0.042	1	Saliva_Duc	EZH1
C5orf422	0.005899	0.334045	0.081	0.043	1	Saliva_Duc	C5orf42
GTF2H51	0.005962	0.678532	0.249	0.2	1	Saliva_Duc	GTF2H5
ATP5B2	0.006027	0.478944	0.411	0.372	1	Saliva_Duc	ATP5B
C1orf61	0.006118	0.299776	0.054	0.024	1	Saliva_Duc	C1orf61
AGO32	0.006146	0.440558	0.124	0.077	1	Saliva_Duc	AGO3
ARL33	0.006371	0.487255	0.168	0.115	1	Saliva_Duc	ARL3
TNPO12	0.006406	0.48166	0.178	0.126	1	Saliva_Duc	TNPO1
THUMPD2	0.006477	0.301685	0.07	0.035	1	Saliva_Duc	THUMPD2
COX5B2	0.00655	0.28759	0.595	0.566	1	Saliva_Duc	COX5B
ZNF3262	0.00668	0.518408	0.135	0.086	1	Saliva_Duc	ZNF326
TRIP117	0.00679	0.452262	0.173	0.121	1	Saliva_Duc	TRIP11
SYPL12	0.006849	0.417043	0.319	0.264	1	Saliva_Duc	SYPL1
LMAN16	0.006911	0.454458	0.249	0.194	1	Saliva_Duc	LMAN1
NFE2L13	0.00695	0.427195	0.141	0.092	1	Saliva_Duc	NFE2L1
MRPL206	0.007031	0.472728	0.319	0.271	1	Saliva_Duc	MRPL20
RSU1	0.00707	0.370913	0.178	0.123	1	Saliva_Duc	RSU1
CHDH2	0.007557	0.354163	0.076	0.04	1	Saliva_Duc	CHDH
PSMD83	0.007623	0.399123	0.378	0.332	1	Saliva_Duc	PSMD8
PCMTD14	0.007798	0.543411	0.232	0.18	1	Saliva_Duc	PCMTD1
PPP1R104	0.007831	0.505023	0.205	0.153	1	Saliva_Duc	PPP1R10
EIF2AK24	0.007922	0.398395	0.146	0.096	1	Saliva_Duc	EIF2AK2
PDGFC2	0.008037	0.377978	0.086	0.048	1	Saliva_Duc	PDGFC
BACH2	0.008064	0.274688	0.043	0.018	1	Saliva_Duc	BACH2
EIF6	0.008179	0.413658	0.319	0.269	1	Saliva_Duc	EIF6
RBFOX21	0.00836	0.508413	0.157	0.107	1	Saliva_Duc	RBFOX2
CHURC14	0.008561	0.538825	0.265	0.217	1	Saliva_Duc	CHURC1
RAB133	0.008601	0.498172	0.265	0.219	1	Saliva_Duc	RAB13
MFAP1	0.008654	0.465976	0.124	0.079	1	Saliva_Duc	MFAP1
PMVK1	0.008694	0.461149	0.184	0.132	1	Saliva_Duc	PMVK
SCO1	0.008721	0.298807	0.086	0.048	1	Saliva_Duc	SCO1
TERF12	0.008902	0.462858	0.157	0.107	1	Saliva_Duc	TERF1
PLCD3	0.009123	0.312807	0.07	0.036	1	Saliva_Duc	PLCD3
PLXDC24	0.009231	0.445451	0.081	0.044	1	Saliva_Duc	PLXDC2
RCAN31	0.009304	0.321587	0.108	0.064	1	Saliva_Duc	RCAN3
SLC6A62	0.009361	0.27917	0.076	0.04	1	Saliva_Duc	SLC6A6
DPF21	0.009594	0.41327	0.119	0.075	1	Saliva_Duc	DPF2
LIMK2	0.009696	0.34889	0.081	0.045	1	Saliva_Duc	LIMK2
IRGQ	0.009766	0.276336	0.059	0.029	1	Saliva_Duc	IRGQ
ZNF609	0.009809	0.304715	0.081	0.044	1	Saliva_Duc	ZNF609
IMMP2L	0.009813	0.327493	0.086	0.048	1	Saliva_Duc	IMMP2L
RGS121	0.009911	0.258671	0.076	0.04	1	Saliva_Duc	RGS12
PKP3	0.009993	0.380598	0.108	0.066	1	Saliva_Duc	PKP3
SCGB3A13	0	3.419539	0.97	0.118	0	Saliva_Acir	SCGB3A1
PRR43	0	3.354356	0.624	0.097	0	Saliva_Acir	PRR4

LYZ2	0	3.274155	1	0.32	0	Saliva_Acir	LYZ
ZG16B2	0	3.202024	1	0.399	0	Saliva_Acir	ZG16B
C6orf583	0	3.174271	1	0.196	0	Saliva_Acir	C6orf58
SLPI2	0	3.105403	1	0.481	0	Saliva_Acir	SLPI
TCN13	0	3.070273	0.865	0.137	0	Saliva_Acir	TCN1
RP11.1143	0	2.802019	0.988	0.209	0	Saliva_Acir	RP11.1143G9.4
AZGP13	0	2.743217	0.949	0.186	0	Saliva_Acir	AZGP1
TFF31	0	2.144733	0.83	0.123	0	Saliva_Acir	TFF3
AGR21	0	2.111121	0.683	0.137	0	Saliva_Acir	AGR2
FAM3D3	0	1.95633	0.546	0.232	0	Saliva_Acir	FAM3D
SSR44	0	1.717667	0.837	0.541	0	Saliva_Acir	SSR4
FCGBP1	0	1.577284	0.422	0.136	0	Saliva_Acir	FCGBP
WFDC22	0	1.572547	0.768	0.223	0	Saliva_Acir	WFDC2
GNAS3	0	1.487992	0.911	0.574	0	Saliva_Acir	GNAS
TPT16	0	1.287637	0.998	0.957	0	Saliva_Acir	TPT1
MT.ND311	0	1.018019	0.989	0.789	0	Saliva_Acir	MT.ND3
MT.ATP61:	0	0.798324	0.991	0.896	0	Saliva_Acir	MT.ATP6
MT.CO211	0	0.715031	0.991	0.959	0	Saliva_Acir	MT.CO2
MT.CYB11	0	0.651363	0.983	0.932	0	Saliva_Acir	MT.CYB
FXYD35	1.3E-303	1.912089	0.472	0.226	2.1E-299	Saliva_Acir	FXYD3
XBP14	2.3E-269	1.671866	0.558	0.337	3.6E-265	Saliva_Acir	XBP1
S100A14	3.3E-255	1.909477	0.293	0.099	5.2E-251	Saliva_Acir	S100A1
RPL397	4.3E-198	0.701406	0.858	0.672	6.9E-194	Saliva_Acir	RPL39
LRRC263	1.4E-195	1.872974	0.227	0.074	2.3E-191	Saliva_Acir	LRRC26
KRT195	3.4E-153	1.405204	0.28	0.119	5.5E-149	Saliva_Acir	KRT19
KIAA13243	4.2E-114	1.557094	0.233	0.106	6.6E-110	Saliva_Acir	KIAA1324
RPS246	2.9E-104	0.530801	0.86	0.839	4.7E-100	Saliva_Acir	RPS24
CCL284	3.7E-97	1.447139	0.226	0.109	5.91E-93	Saliva_Acir	CCL28
MT.ND411	7.16E-85	0.356377	0.95	0.954	1.14E-80	Saliva_Acir	MT.ND4
PHKG1	1.24E-80	1.011853	0.073	0.019	1.98E-76	Saliva_Acir	PHKG1
CLDN103	5.36E-72	1.412743	0.189	0.094	8.55E-68	Saliva_Acir	CLDN10
UQCR101	3.63E-63	0.272149	0.187	0.446	5.79E-59	Saliva_Acir	UQCR10
DDT2	8.28E-63	0.253134	0.134	0.347	1.32E-58	Saliva_Acir	DDT
TOMM209	7.34E-59	0.297703	0.129	0.332	1.17E-54	Saliva_Acir	TOMM20
C8orf598	2.88E-56	0.271006	0.079	0.24	4.6E-52	Saliva_Acir	C8orf59
RAB4A3	2.12E-54	0.260236	0.066	0.213	3.38E-50	Saliva_Acir	RAB4A
COPZ12	3.91E-54	0.262339	0.071	0.221	6.23E-50	Saliva_Acir	COPZ1
NENF5	3.44E-53	0.286649	0.137	0.334	5.48E-49	Saliva_Acir	NENF
OSTC2	2.07E-52	0.343638	0.109	0.283	3.3E-48	Saliva_Acir	OSTC
ETFB3	3.45E-51	0.332453	0.107	0.277	5.5E-47	Saliva_Acir	ETFB
ATP5G16	5.83E-51	0.254146	0.101	0.266	9.3E-47	Saliva_Acir	ATP5G1
MRPL207	6.23E-51	0.325894	0.106	0.276	9.93E-47	Saliva_Acir	MRPL20
ADI16	2.92E-50	0.264175	0.078	0.226	4.66E-46	Saliva_Acir	ADI1
SRA12	2.01E-49	0.284102	0.055	0.186	3.21E-45	Saliva_Acir	SRA1
PTRHD1	4.31E-49	0.251189	0.062	0.196	6.88E-45	Saliva_Acir	PTRHD1
TIMP14	1.44E-48	0.857947	0.429	0.381	2.3E-44	Saliva_Acir	TIMP1
MGST22	3.84E-48	0.333645	0.073	0.213	6.13E-44	Saliva_Acir	MGST2

EIF5A2	5.97E-48	0.431455	0.114	0.284	9.52E-44	Saliva_Acir	EIF5A
MT.ND511	6.44E-47	0.590117	0.633	0.705	1.03E-42	Saliva_Acir	MT.ND5
PRDX27	1.75E-46	0.28139	0.136	0.317	2.8E-42	Saliva_Acir	PRDX2
ATP2A22	2.52E-46	0.263009	0.055	0.179	4.02E-42	Saliva_Acir	ATP2A2
CANX6	8.68E-46	0.399076	0.136	0.317	1.38E-41	Saliva_Acir	CANX
LRPAP17	1.44E-45	0.277556	0.112	0.274	2.3E-41	Saliva_Acir	LRPAP1
SERINC21	1.48E-45	0.251456	0.079	0.217	2.35E-41	Saliva_Acir	SERINC2
EEF1A16	9.25E-45	0.251153	0.945	0.914	1.48E-40	Saliva_Acir	EEF1A1
SSR14	1.99E-44	0.261774	0.067	0.195	3.18E-40	Saliva_Acir	SSR1
FAM107B3	4.25E-44	0.284737	0.131	0.304	6.79E-40	Saliva_Acir	FAM107B
SEC11C4	5.33E-44	0.97732	0.311	0.24	8.5E-40	Saliva_Acir	SEC11C
PPP1R14B8	6.74E-44	0.257224	0.059	0.181	1.07E-39	Saliva_Acir	PPP1R14B
MRPL543	8.02E-44	0.314695	0.079	0.214	1.28E-39	Saliva_Acir	MRPL54
TMED33	8.86E-44	1.460887	0.328	0.278	1.41E-39	Saliva_Acir	TMED3
STRA131	6.65E-43	0.312267	0.07	0.199	1.06E-38	Saliva_Acir	STRA13
ATOX13	1.17E-42	0.398865	0.118	0.279	1.87E-38	Saliva_Acir	ATOX1
NDUFB77	1.67E-42	0.420963	0.175	0.379	2.67E-38	Saliva_Acir	NDUFB7
ARL14	3.54E-42	0.40154	0.078	0.21	5.65E-38	Saliva_Acir	ARL1
ATRAID3	4.53E-42	0.334176	0.129	0.296	7.22E-38	Saliva_Acir	ATRAID
TSTA31	6.93E-41	0.303164	0.061	0.18	1.1E-36	Saliva_Acir	TSTA3
TMEM179I	9.27E-41	0.309838	0.075	0.202	1.48E-36	Saliva_Acir	TMEM179B
CD1517	1.46E-40	0.278848	0.089	0.224	2.32E-36	Saliva_Acir	CD151
HM133	1.69E-40	0.322998	0.079	0.208	2.69E-36	Saliva_Acir	HM13
TMEM208	2.13E-40	0.312801	0.075	0.2	3.39E-36	Saliva_Acir	TMEM208
NANS3	5.42E-40	0.46778	0.095	0.235	8.65E-36	Saliva_Acir	NANS
RPL293	2.17E-39	0.277035	0.848	0.829	3.45E-35	Saliva_Acir	RPL29
GALE1	4.4E-38	0.25604	0.048	0.152	7.01E-34	Saliva_Acir	GALE
SSR34	5.61E-38	0.429657	0.141	0.306	8.94E-34	Saliva_Acir	SSR3
TSPAN34	8.31E-38	0.305396	0.062	0.175	1.33E-33	Saliva_Acir	TSPAN3
SEC61G2	1.78E-37	0.37603	0.206	0.421	2.85E-33	Saliva_Acir	SEC61G
ACSL32	2.88E-37	0.338061	0.055	0.163	4.59E-33	Saliva_Acir	ACSL3
CHCHD14	3.23E-37	0.386438	0.064	0.177	5.15E-33	Saliva_Acir	CHCHD1
KDELR23	7.59E-37	0.408169	0.152	0.325	1.21E-32	Saliva_Acir	KDELR2
HEXB4	1.78E-36	0.275335	0.058	0.165	2.84E-32	Saliva_Acir	HEXB
VAMP82	1.84E-36	0.264078	0.207	0.415	2.94E-32	Saliva_Acir	VAMP8
FUCA13	1.9E-36	0.268496	0.044	0.142	3.03E-32	Saliva_Acir	FUCA1
DHRS75	3.05E-36	0.342335	0.087	0.212	4.86E-32	Saliva_Acir	DHRS7
ATP5G37	3.15E-36	0.43704	0.177	0.368	5.02E-32	Saliva_Acir	ATP5G3
TECR3	1.07E-35	0.481726	0.16	0.336	1.7E-31	Saliva_Acir	TECR
COPB24	2.1E-35	0.349837	0.055	0.159	3.35E-31	Saliva_Acir	COPB2
ITM2B4	8.36E-35	0.534307	0.603	0.71	1.33E-30	Saliva_Acir	ITM2B
SPCS33	8.58E-35	0.432017	0.115	0.255	1.37E-30	Saliva_Acir	SPCS3
RPL7A8	1.72E-34	0.299063	0.819	0.814	2.75E-30	Saliva_Acir	RPL7A
SAA23	2.6E-34	0.968847	0.105	0.052	4.15E-30	Saliva_Acir	SAA2
DPP74	2.84E-34	0.397341	0.121	0.264	4.53E-30	Saliva_Acir	DPP7
PDCD43	5.14E-34	0.340636	0.075	0.188	8.2E-30	Saliva_Acir	PDCD4
NDUFA46	1.16E-33	0.927601	0.474	0.54	1.84E-29	Saliva_Acir	NDUFA4

AC023590.	1.28E-33	0.520823	0.032	0.008	2.04E-29	Saliva_Acir	AC023590.1
RPL308	3.05E-33	0.263835	0.844	0.845	4.87E-29	Saliva_Acir	RPL30
ALG53	5.48E-33	0.324876	0.058	0.157	8.75E-29	Saliva_Acir	ALG5
VKORC14	6.82E-33	0.368178	0.102	0.228	1.09E-28	Saliva_Acir	VKORC1
CLCN33	8.7E-33	0.272333	0.043	0.132	1.39E-28	Saliva_Acir	CLCN3
ATP1A15	8.97E-33	0.412859	0.121	0.26	1.43E-28	Saliva_Acir	ATP1A1
TMEM141	9.47E-33	0.361273	0.064	0.168	1.51E-28	Saliva_Acir	TMEM141
SCARB26	1.05E-32	0.345	0.06	0.16	1.68E-28	Saliva_Acir	SCARB2
S100A133	1.33E-32	0.25796	0.124	0.261	2.12E-28	Saliva_Acir	S100A13
ALDH9A12	3.29E-32	0.318811	0.055	0.152	5.25E-28	Saliva_Acir	ALDH9A1
AKR1A15	3.68E-32	0.439207	0.09	0.208	5.86E-28	Saliva_Acir	AKR1A1
YIF1A4	7.1E-32	0.375281	0.07	0.176	1.13E-27	Saliva_Acir	YIF1A
TTC35	9.14E-32	0.390569	0.108	0.237	1.46E-27	Saliva_Acir	TTC3
TM9SF23	1.16E-31	0.506706	0.084	0.198	1.84E-27	Saliva_Acir	TM9SF2
RNF1303	1.19E-31	0.25444	0.051	0.144	1.9E-27	Saliva_Acir	RNF130
SURF43	3.94E-31	0.42816	0.074	0.181	6.29E-27	Saliva_Acir	SURF4
PDIA63	5.6E-31	0.399139	0.138	0.284	8.93E-27	Saliva_Acir	PDIA6
DAP3	6.03E-31	0.355837	0.054	0.147	9.61E-27	Saliva_Acir	DAP
PSMA21	1.58E-30	0.283837	0.042	0.127	2.52E-26	Saliva_Acir	PSMA2
EPRS7	1.67E-30	0.285128	0.047	0.136	2.67E-26	Saliva_Acir	EPRS
BNIP32	4.48E-30	0.376249	0.058	0.153	7.15E-26	Saliva_Acir	BNIP3
CHID13	6.2E-30	0.416379	0.072	0.174	9.88E-26	Saliva_Acir	CHID1
MSRB25	2.47E-29	0.261395	0.047	0.132	3.94E-25	Saliva_Acir	MSRB2
SLC9A3R1	2.92E-29	0.306432	0.062	0.157	4.66E-25	Saliva_Acir	SLC9A3R1
C1orf1224	4.35E-29	0.421118	0.081	0.187	6.94E-25	Saliva_Acir	C1orf122
PAPSS13	4.43E-29	0.256111	0.035	0.112	7.07E-25	Saliva_Acir	PAPSS1
SSR22	6.6E-29	0.359633	0.23	0.435	1.05E-24	Saliva_Acir	SSR2
CKAP43	6.94E-29	0.34334	0.07	0.168	1.11E-24	Saliva_Acir	CKAP4
TM9SF32	1.9E-28	0.584243	0.114	0.239	3.03E-24	Saliva_Acir	TM9SF3
LAGE3	2.4E-28	0.27559	0.056	0.145	3.83E-24	Saliva_Acir	LAGE3
PCYOX14	4.22E-28	0.352831	0.05	0.134	6.72E-24	Saliva_Acir	PCYOX1
FAM114A1	5.35E-28	0.254016	0.044	0.126	8.54E-24	Saliva_Acir	FAM114A1
TMEM205	5.48E-28	0.439651	0.096	0.208	8.74E-24	Saliva_Acir	TMEM205
GPAA14	7.93E-28	0.341116	0.057	0.146	1.26E-23	Saliva_Acir	GPAA1
MRPS265	1.53E-27	0.340011	0.062	0.153	2.43E-23	Saliva_Acir	MRPS26
SRPRB2	2.16E-27	0.255823	0.038	0.115	3.45E-23	Saliva_Acir	SRPRB
BACE23	2.36E-26	0.480499	0.071	0.165	3.77E-22	Saliva_Acir	BACE2
TPD523	2.39E-26	0.334027	0.049	0.13	3.81E-22	Saliva_Acir	TPD52
TXNDC153	2.76E-26	0.262242	0.047	0.125	4.4E-22	Saliva_Acir	TXNDC15
LAMP25	7.97E-26	0.65903	0.118	0.24	1.27E-21	Saliva_Acir	LAMP2
ECI16	1.15E-25	0.4391	0.057	0.141	1.84E-21	Saliva_Acir	ECI1
IDH11	1.67E-25	0.37372	0.049	0.127	2.66E-21	Saliva_Acir	IDH1
SMCO42	3.12E-25	0.350489	0.045	0.121	4.98E-21	Saliva_Acir	SMCO4
MPZL14	4.48E-25	0.445054	0.059	0.143	7.14E-21	Saliva_Acir	MPZL1
MXD45	5.15E-25	0.306448	0.047	0.123	8.21E-21	Saliva_Acir	MXD4
PITPNA2	7.33E-25	0.298165	0.035	0.104	1.17E-20	Saliva_Acir	PITPNA
ARFIP22	1.13E-24	0.280711	0.038	0.108	1.8E-20	Saliva_Acir	ARFIP2

DPM22	2.33E-24	0.327126	0.043	0.115	3.71E-20	Saliva_Acir	DPM2
ISOC23	4.83E-24	0.374455	0.055	0.135	7.7E-20	Saliva_Acir	ISOC2
DNAJC102	5.13E-24	0.288482	0.047	0.12	8.18E-20	Saliva_Acir	DNAJC10
DAD15	2.29E-23	0.596848	0.219	0.404	3.66E-19	Saliva_Acir	DAD1
COPG13	5.02E-23	0.279982	0.033	0.097	8.01E-19	Saliva_Acir	COPG1
DBI4	6.81E-23	0.961712	0.394	0.416	1.09E-18	Saliva_Acir	DBI
RAB253	7.52E-23	0.455154	0.058	0.136	1.2E-18	Saliva_Acir	RAB25
TMEM45A	9.07E-23	0.27971	0.037	0.102	1.45E-18	Saliva_Acir	TMEM45A
PRDX43	9.63E-23	1.127886	0.286	0.256	1.54E-18	Saliva_Acir	PRDX4
RDH111	1.22E-22	0.335477	0.044	0.113	1.95E-18	Saliva_Acir	RDH11
TMEM123	4.03E-22	0.583722	0.153	0.286	6.43E-18	Saliva_Acir	TMEM123
PRKACB3	4.74E-22	0.31257	0.038	0.103	7.55E-18	Saliva_Acir	PRKACB
IBTK2	4.84E-22	0.272677	0.033	0.095	7.72E-18	Saliva_Acir	IBTK
ALCAM3	5.28E-22	0.270493	0.035	0.099	8.42E-18	Saliva_Acir	ALCAM
UQCRQ3	9.52E-22	0.498944	0.275	0.506	1.52E-17	Saliva_Acir	UQCRQ
MIA34	2.54E-21	0.265998	0.047	0.114	4.06E-17	Saliva_Acir	MIA3
PGM33	5.14E-21	0.291049	0.034	0.094	8.2E-17	Saliva_Acir	PGM3
ANXA34	6.8E-21	0.506722	0.075	0.158	1.08E-16	Saliva_Acir	ANXA3
MCFD23	7.23E-21	0.609091	0.099	0.196	1.15E-16	Saliva_Acir	MCFD2
XAB22	7.31E-21	0.322913	0.036	0.098	1.17E-16	Saliva_Acir	XAB2
CNPY22	1.14E-20	0.560125	0.108	0.209	1.82E-16	Saliva_Acir	CNPY2
SNRPN4	1.19E-20	0.353157	0.044	0.109	1.9E-16	Saliva_Acir	SNRPN
AGFG22	2.06E-20	0.443056	0.064	0.14	3.29E-16	Saliva_Acir	AGFG2
CTNNB11	3.87E-20	0.480203	0.087	0.175	6.17E-16	Saliva_Acir	CTNNB1
NME43	5.44E-20	0.483685	0.068	0.145	8.67E-16	Saliva_Acir	NME4
FAM84B3	5.53E-20	0.252621	0.029	0.084	8.82E-16	Saliva_Acir	FAM84B
SERF22	1.16E-19	0.340389	0.668	0.827	1.85E-15	Saliva_Acir	SERF2
FAM210B6	5.61E-19	0.289665	0.038	0.095	8.95E-15	Saliva_Acir	FAM210B
HIPK25	7E-19	0.26704	0.029	0.081	1.12E-14	Saliva_Acir	HIPK2
PAM3	1.57E-18	0.384195	0.07	0.144	2.51E-14	Saliva_Acir	PAM
CLPTM1L4	2.83E-18	0.292324	0.038	0.094	4.52E-14	Saliva_Acir	CLPTM1L
SLC31A12	3.12E-18	0.320893	0.03	0.082	4.98E-14	Saliva_Acir	SLC31A1
GFPT13	6.29E-18	0.50763	0.061	0.13	1E-13	Saliva_Acir	GFPT1
SLC1A54	7.86E-18	0.274665	0.029	0.079	1.25E-13	Saliva_Acir	SLC1A5
CDH14	8.85E-18	0.262265	0.027	0.076	1.41E-13	Saliva_Acir	CDH1
SEC61A13	9.91E-18	0.545615	0.078	0.154	1.58E-13	Saliva_Acir	SEC61A1
TMED45	1.46E-17	0.58962	0.093	0.176	2.33E-13	Saliva_Acir	TMED4
OAT3	1.46E-17	0.587611	0.091	0.174	2.33E-13	Saliva_Acir	OAT
PTPN183	2.51E-17	0.262181	0.038	0.093	4E-13	Saliva_Acir	PTPN18
DPM32	5.43E-17	0.618786	0.098	0.183	8.66E-13	Saliva_Acir	DPM3
PHPT15	9.24E-17	0.604409	0.189	0.329	1.47E-12	Saliva_Acir	PHPT1
REEP55	3.23E-16	0.590244	0.181	0.313	5.16E-12	Saliva_Acir	REEP5
COX8A3	4.26E-16	0.661758	0.251	0.441	6.79E-12	Saliva_Acir	COX8A
MTA11	4.74E-16	0.331566	0.034	0.084	7.56E-12	Saliva_Acir	MTA1
DHCR243	4.94E-16	0.429691	0.045	0.101	7.88E-12	Saliva_Acir	DHCR24
SUMF22	2.23E-15	0.438834	0.057	0.117	3.55E-11	Saliva_Acir	SUMF2
SPDEF2	2.77E-15	1.027742	0.098	0.063	4.42E-11	Saliva_Acir	SPDEF

GPT22	3.49E-15	0.369817	0.037	0.086	5.57E-11	Saliva_Acir	GPT2
PTPRF5	8.5E-15	0.477306	0.052	0.108	1.36E-10	Saliva_Acir	PTPRF
P2RX42	1.9E-14	0.257607	0.026	0.068	3.03E-10	Saliva_Acir	P2RX4
MAGED12	2.58E-14	0.394235	0.039	0.087	4.11E-10	Saliva_Acir	MAGED1
RPS1710	3.67E-14	0.744416	0.251	0.222	5.85E-10	Saliva_Acir	RPS17
OGFRL15	4.19E-14	0.438909	0.059	0.116	6.68E-10	Saliva_Acir	OGFRL1
VEGFB8	4.5E-14	0.400349	0.049	0.101	7.18E-10	Saliva_Acir	VEGFB
TSTD16	5.1E-14	0.652734	0.163	0.276	8.13E-10	Saliva_Acir	TSTD1
MTURN2	5.13E-14	0.383733	0.036	0.082	8.17E-10	Saliva_Acir	MTURN
DSC23	1.38E-13	0.327246	0.029	0.07	2.2E-09	Saliva_Acir	DSC2
FKBP113	1.49E-13	0.28193	0.089	0.156	2.37E-09	Saliva_Acir	FKBP11
CD638	1.75E-13	0.424658	0.554	0.627	2.8E-09	Saliva_Acir	CD63
RASSF3	3.67E-13	0.328172	0.034	0.077	5.86E-09	Saliva_Acir	RASSF3
JTB3	4.02E-13	0.689263	0.223	0.375	6.41E-09	Saliva_Acir	JTB
SERP13	4.1E-13	0.479066	0.32	0.553	6.53E-09	Saliva_Acir	SERP1
PART12	4.78E-13	1.084828	0.103	0.071	7.63E-09	Saliva_Acir	PART1
TMEM63A	5.12E-13	0.31827	0.038	0.082	8.17E-09	Saliva_Acir	TMEM63A
PEBP17	5.94E-13	0.454471	0.275	0.461	9.48E-09	Saliva_Acir	PEBP1
LGALS3BP6	9.94E-13	1.208245	0.196	0.17	1.59E-08	Saliva_Acir	LGALS3BP
C8orf44	1.02E-12	0.273251	0.066	0.122	1.63E-08	Saliva_Acir	C8orf4
BARX22	1.17E-12	0.292723	0.026	0.064	1.87E-08	Saliva_Acir	BARX2
RGCC4	1.75E-12	0.906164	0.236	0.214	2.79E-08	Saliva_Acir	RGCC
INSR3	1.84E-12	0.592144	0.073	0.132	2.94E-08	Saliva_Acir	INSR
IL17RC1	3.21E-12	0.268026	0.027	0.065	5.12E-08	Saliva_Acir	IL17RC
ENTPD61	5.83E-12	0.26984	0.026	0.063	9.3E-08	Saliva_Acir	ENTPD6
HPSE1	5.89E-12	0.321915	0.028	0.065	9.39E-08	Saliva_Acir	HPSE
FAM174B1	6.34E-12	0.273349	0.026	0.061	1.01E-07	Saliva_Acir	FAM174B
CHPF4	1.1E-11	0.408825	0.042	0.085	1.75E-07	Saliva_Acir	CHPF
C11orf242	1.12E-11	0.268887	0.026	0.062	1.79E-07	Saliva_Acir	C11orf24
TMEM258	1.28E-11	0.54958	0.275	0.458	2.04E-07	Saliva_Acir	TMEM258
NFIB7	1.56E-11	0.507682	0.118	0.194	2.49E-07	Saliva_Acir	NFIB
NR2F64	1.59E-11	0.42137	0.042	0.085	2.54E-07	Saliva_Acir	NR2F6
NDUFC22	1.68E-11	0.772119	0.235	0.391	2.68E-07	Saliva_Acir	NDUFC2
ATP6V0E2	1.86E-11	0.278128	0.038	0.078	2.97E-07	Saliva_Acir	ATP6V0E2
CCDC152	2.79E-11	0.303419	0.027	0.062	4.46E-07	Saliva_Acir	CCDC15
TNFRSF192	2.85E-11	0.324376	0.031	0.068	4.55E-07	Saliva_Acir	TNFRSF19
EIF4EBP12	3.5E-11	0.655969	0.09	0.153	5.57E-07	Saliva_Acir	EIF4EBP1
KRT84	3.67E-11	0.309214	0.127	0.202	5.86E-07	Saliva_Acir	KRT8
HDLBP4	3.83E-11	0.783914	0.163	0.264	6.1E-07	Saliva_Acir	HDLBP
SLC12A22	5.11E-11	1.183899	0.141	0.112	8.15E-07	Saliva_Acir	SLC12A2
SEC61B3	7.82E-11	0.548055	0.282	0.465	1.25E-06	Saliva_Acir	SEC61B
DCXR2	1.7E-10	0.65968	0.139	0.223	2.71E-06	Saliva_Acir	DCXR
MESP11	1.9E-10	0.279844	0.024	0.056	3.03E-06	Saliva_Acir	MESP1
PHGDH3	2.33E-10	0.579113	0.067	0.117	3.72E-06	Saliva_Acir	PHGDH
CMTM84	5.26E-10	0.405102	0.035	0.071	8.39E-06	Saliva_Acir	CMTM8
TMEM214	6.28E-10	0.414255	0.038	0.074	1E-05	Saliva_Acir	TMEM214
PKP23	6.39E-10	0.251179	0.022	0.051	1.02E-05	Saliva_Acir	PKP2

ACTR3B2	7.31E-10	0.397301	0.034	0.069	1.17E-05	Saliva_Acir	ACTR3B
NUCB24	8.89E-10	1.071743	0.266	0.271	1.42E-05	Saliva_Acir	NUCB2
ATP13A21	9.5E-10	0.288784	0.026	0.056	1.52E-05	Saliva_Acir	ATP13A2
STARD101	1.05E-09	0.44225	0.085	0.14	1.67E-05	Saliva_Acir	STARD10
TPD52L15	1.08E-09	0.668932	0.083	0.138	1.72E-05	Saliva_Acir	TPD52L1
GOLM11	1.18E-09	0.906131	0.118	0.09	1.88E-05	Saliva_Acir	GOLM1
PHYHD14	1.32E-09	0.340562	0.029	0.061	2.11E-05	Saliva_Acir	PHYHD1
P4HB3	1.57E-09	1.027496	0.358	0.421	2.5E-05	Saliva_Acir	P4HB
BSPRY1	2.26E-09	0.299893	0.023	0.051	3.61E-05	Saliva_Acir	BSPRY
KDELR34	2.7E-09	0.446697	0.048	0.087	4.3E-05	Saliva_Acir	KDELR3
SLC31A21	3.69E-09	0.583659	0.066	0.112	5.88E-05	Saliva_Acir	SLC31A2
SFRP14	4.08E-09	0.317951	0.056	0.097	6.5E-05	Saliva_Acir	SFRP1
RPL247	5.68E-09	0.25689	0.68	0.806	9.05E-05	Saliva_Acir	RPL24
CANT12	6.65E-09	0.654281	0.065	0.11	0.000106	Saliva_Acir	CANT1
CPD2	6.72E-09	0.643694	0.074	0.122	0.000107	Saliva_Acir	CPD
ANG3	7.47E-09	0.532587	0.06	0.102	0.000119	Saliva_Acir	ANG
ITGA66	8.17E-09	0.607069	0.085	0.137	0.00013	Saliva_Acir	ITGA6
BIK	1.03E-08	0.290148	0.026	0.054	0.000164	Saliva_Acir	BIK
SOX24	1.14E-08	0.262154	0.02	0.046	0.000182	Saliva_Acir	SOX2
SEZ6L22	1.4E-08	0.300302	0.021	0.047	0.000223	Saliva_Acir	SEZ6L2
CALR3	2.56E-08	0.663779	0.268	0.428	0.000408	Saliva_Acir	CALR
GMPPB3	2.86E-08	0.371964	0.035	0.066	0.000456	Saliva_Acir	GMPPB
FUT22	5.35E-08	0.416556	0.039	0.071	0.000853	Saliva_Acir	FUT2
MGST39	6.13E-08	0.533416	0.22	0.339	0.000978	Saliva_Acir	MGST3
HINT16	6.89E-08	0.30545	0.364	0.576	0.0011	Saliva_Acir	HINT1
PHB3	7.23E-08	1.105348	0.239	0.242	0.001153	Saliva_Acir	PHB
FAM3B2	8.76E-08	0.394704	0.042	0.075	0.001397	Saliva_Acir	FAM3B
TESC3	1.26E-07	0.457073	0.072	0.115	0.002014	Saliva_Acir	TESC
FAM46A2	1.48E-07	1.03383	0.18	0.165	0.002355	Saliva_Acir	FAM46A
NAGLU3	2.56E-07	0.426276	0.04	0.07	0.004089	Saliva_Acir	NAGLU
NDUFB89	2.9E-07	0.801027	0.119	0.18	0.004625	Saliva_Acir	NDUFB8
RAB3D2	3.77E-07	0.379601	0.031	0.058	0.006013	Saliva_Acir	RAB3D
SMIM13	3.97E-07	0.445547	0.041	0.071	0.006333	Saliva_Acir	SMIM1
CLDN44	6.36E-07	0.4911	0.056	0.091	0.010147	Saliva_Acir	CLDN4
METRN4	9.07E-07	0.543652	0.059	0.095	0.014468	Saliva_Acir	METRN
FBXO6	9.99E-07	0.263927	0.023	0.046	0.015931	Saliva_Acir	FBXO6
PARM13	1.15E-06	0.362921	0.03	0.054	0.018286	Saliva_Acir	PARM1
SLC39A82	1.16E-06	0.542957	0.06	0.095	0.018564	Saliva_Acir	SLC39A8
CREB3L12	1.19E-06	0.46243	0.045	0.075	0.018903	Saliva_Acir	CREB3L1
SLITRK63	1.45E-06	0.402135	0.034	0.06	0.023196	Saliva_Acir	SLITRK6
GALNT71	1.54E-06	0.361159	0.038	0.064	0.0245	Saliva_Acir	GALNT7
SELENBP1(	1.79E-06	0.455195	0.047	0.076	0.028592	Saliva_Acir	SELENBP1
YIPF24	2.03E-06	0.68078	0.071	0.11	0.032332	Saliva_Acir	YIPF2
PKDCC2	2.32E-06	0.340928	0.029	0.053	0.037012	Saliva_Acir	PKDCC
SLC26A22	2.71E-06	0.478623	0.049	0.079	0.043258	Saliva_Acir	SLC26A2
PLA2R11	3.86E-06	0.389764	0.032	0.056	0.061565	Saliva_Acir	PLA2R1
CYB5612	6.1E-06	0.557221	0.057	0.089	0.097289	Saliva_Acir	CYB561

RABAC18	7.5E-06	0.605896	0.269	0.409	0.119604	Saliva_Acir	RABAC1
GGT61	7.72E-06	0.29416	0.021	0.04	0.123162	Saliva_Acir	GGT6
CREB3L42	8.61E-06	0.38929	0.035	0.058	0.13728	Saliva_Acir	CREB3L4
NKX3.11	9.3E-06	0.571638	0.053	0.037	0.148286	Saliva_Acir	NKX3.1
CHPT14	9.54E-06	0.822464	0.143	0.209	0.152214	Saliva_Acir	CHPT1
SH3BP43	1.23E-05	0.579752	0.053	0.082	0.196151	Saliva_Acir	SH3BP4
CEP413	1.25E-05	0.613006	0.069	0.103	0.199894	Saliva_Acir	CEP41
CGREF12	1.4E-05	0.374646	0.028	0.049	0.222653	Saliva_Acir	CGREF1
APIP3	1.47E-05	0.659633	0.065	0.098	0.23518	Saliva_Acir	APIP
TMBIM63	2.57E-05	0.438975	0.366	0.585	0.41001	Saliva_Acir	TMBIM6
TM7SF22	2.92E-05	0.668246	0.07	0.104	0.465672	Saliva_Acir	TM7SF2
PPP1R16A4	3.67E-05	0.694505	0.071	0.105	0.585925	Saliva_Acir	PPP1R16A
CLDN34	5.7E-05	0.951219	0.114	0.099	0.908659	Saliva_Acir	CLDN3
C1orf56	6.24E-05	1.020704	0.134	0.122	0.994492	Saliva_Acir	C1orf56
TFCP2L14	6.56E-05	0.326358	0.029	0.049	1	Saliva_Acir	TFCP2L1
TMEM125:	8.71E-05	0.41579	0.031	0.05	1	Saliva_Acir	TMEM125
GALNT61	0.000108	0.321044	0.03	0.049	1	Saliva_Acir	GALNT6
NPDC14	0.000133	0.96516	0.177	0.173	1	Saliva_Acir	NPDC1
ATP2A33	0.000135	0.350905	0.035	0.056	1	Saliva_Acir	ATP2A3
ARG21	0.000145	0.552785	0.046	0.069	1	Saliva_Acir	ARG2
GLYATL22	0.000214	0.466349	0.035	0.024	1	Saliva_Acir	GLYATL2
FGGY3	0.000251	1.039336	0.098	0.085	1	Saliva_Acir	FGGY
ANKRD36C	0.000554	0.559632	0.102	0.139	1	Saliva_Acir	ANKRD36C
FOLH12	0.000575	0.310676	0.021	0.035	1	Saliva_Acir	FOLH1
SPCS12	0.000618	0.591751	0.309	0.467	1	Saliva_Acir	SPCS1
HID13	0.000764	0.417425	0.031	0.048	1	Saliva_Acir	HID1
AFMID	0.000874	0.387084	0.03	0.046	1	Saliva_Acir	AFMID
TSPAN133	0.001254	0.824703	0.117	0.159	1	Saliva_Acir	TSPAN13
CDC42EP5:	0.001772	0.714335	0.147	0.199	1	Saliva_Acir	CDC42EP5
MSMB1	0.001801	0.339754	0.025	0.039	1	Saliva_Acir	MSMB
NUDT83	0.001876	0.470708	0.041	0.058	1	Saliva_Acir	NUDT8
PTH2R	0.001969	0.31209	0.019	0.03	1	Saliva_Acir	PTH2R
SNHG182	0.00236	0.495428	0.045	0.063	1	Saliva_Acir	SNHG18
PNPT1	0.003359	0.701832	0.059	0.048	1	Saliva_Acir	PNPT1
CHCHD108	0.003657	0.817683	0.181	0.247	1	Saliva_Acir	CHCHD10
NUPR15	0.004116	0.570947	0.195	0.262	1	Saliva_Acir	NUPR1
CD818	0.005267	0.60526	0.29	0.325	1	Saliva_Acir	CD81
GMDS4	0.006659	0.89456	0.129	0.172	1	Saliva_Acir	GMDS
MDK2	0.007438	0.836887	0.114	0.151	1	Saliva_Acir	MDK
GJC32	0.007645	0.825175	0.07	0.061	1	Saliva_Acir	GJC3
RARRES13	0.009042	0.629296	0.05	0.041	1	Saliva_Acir	RARRES1
MYBPC13	0.00993	0.320697	0.026	0.019	1	Saliva_Acir	MYBPC1
GNAS4	0	3.520328	1	0.569	0	Saliva_Acir	GNAS
ZG16B3	0	3.336481	1	0.396	0	Saliva_Acir	ZG16B
LYZ3	0	3.154803	1	0.317	0	Saliva_Acir	LYZ
C6orf584	0	3.019572	0.999	0.191	0	Saliva_Acir	C6orf58
SLPI3	0	2.775829	1	0.478	0	Saliva_Acir	SLPI

TCN14	0	2.676407	0.998	0.129	0	Saliva_Acir	TCN1
AZGP14	0	2.473456	1	0.18	0	Saliva_Acir	AZGP1
RP11.1143	0	2.386688	0.996	0.205	0	Saliva_Acir	RP11.1143G9.4
FAM3D4	0	2.245858	1	0.215	0	Saliva_Acir	FAM3D
S100A15	0	2.120752	0.981	0.075	0	Saliva_Acir	S100A1
CLDN104	0	2.085648	0.948	0.068	0	Saliva_Acir	CLDN10
DBI5	0	1.962894	0.998	0.396	0	Saliva_Acir	DBI
FAM46A3	0	1.895497	0.975	0.138	0	Saliva_Acir	FAM46A
CCL285	0	1.891112	0.983	0.083	0	Saliva_Acir	CCL28
TMED34	0	1.868993	0.992	0.255	0	Saliva_Acir	TMED3
KIAA13244	0	1.860208	0.987	0.08	0	Saliva_Acir	KIAA1324
FXYD36	0	1.853307	0.996	0.208	0	Saliva_Acir	FXYD3
XBP15	0	1.769541	0.997	0.321	0	Saliva_Acir	XBP1
PHB4	0	1.688583	0.988	0.217	0	Saliva_Acir	PHB
SPINT25	0	1.666069	0.989	0.291	0	Saliva_Acir	SPINT2
TESC4	0	1.628116	0.896	0.088	0	Saliva_Acir	TESC
C8orf45	0	1.589766	0.936	0.093	0	Saliva_Acir	C8orf4
P4HB4	0	1.502749	0.997	0.4	0	Saliva_Acir	P4HB
SEC11C5	0	1.479114	0.988	0.217	0	Saliva_Acir	SEC11C
NDUFA47	0	1.440719	0.997	0.523	0	Saliva_Acir	NDUFA4
MTRNR2L1	0	1.410588	0.996	0.245	0	Saliva_Acir	MTRNR2L12
RGCC5	0	1.400017	0.943	0.19	0	Saliva_Acir	RGCC
SSR45	0	1.398838	1	0.534	0	Saliva_Acir	SSR4
CA22	0	1.386156	0.855	0.073	0	Saliva_Acir	CA2
EPCAM4	0	1.367896	0.948	0.104	0	Saliva_Acir	EPCAM
MCFD24	0	1.33082	0.937	0.169	0	Saliva_Acir	MCFD2
CLDN35	0	1.320599	0.912	0.072	0	Saliva_Acir	CLDN3
NPDC15	0	1.304837	0.951	0.147	0	Saliva_Acir	NPDC1
NUCB25	0	1.296154	0.976	0.247	0	Saliva_Acir	NUCB2
ELF52	0	1.289534	0.902	0.043	0	Saliva_Acir	ELF5
TMED104	0	1.229051	0.983	0.299	0	Saliva_Acir	TMED10
MGST15	0	1.175205	0.939	0.17	0	Saliva_Acir	MGST1
PRDX44	0	1.169473	0.966	0.233	0	Saliva_Acir	PRDX4
MGLL5	0	1.143832	0.93	0.152	0	Saliva_Acir	MGLL
PEBP18	0	1.116145	0.988	0.438	0	Saliva_Acir	PEBP1
XIST10	0	1.110577	0.937	0.096	0	Saliva_Acir	XIST
TPD52L16	0	1.109402	0.898	0.111	0	Saliva_Acir	TPD52L1
TPT17	0	1.08561	1	0.957	0	Saliva_Acir	TPT1
SLC31A22	0	1.0627	0.84	0.087	0	Saliva_Acir	SLC31A2
LPO2	0	1.045106	0.69	0.028	0	Saliva_Acir	LPO
ANKRD36C	0	1.03242	0.859	0.114	0	Saliva_Acir	ANKRD36C
CEP414	0	1.030443	0.836	0.078	0	Saliva_Acir	CEP41
TSPAN134	0	1.021695	0.902	0.133	0	Saliva_Acir	TSPAN13
OGFRL16	0	1.016486	0.88	0.089	0	Saliva_Acir	OGFRL1
MTRNR2L1	0	1.003743	0.899	0.1	0	Saliva_Acir	MTRNR2L1
GMDS5	0	0.995035	0.908	0.146	0	Saliva_Acir	GMDS
REEP56	0	0.981326	0.966	0.287	0	Saliva_Acir	REEP5

CLDN45	0	0.974873	0.771	0.067	0	Saliva_Acir	CLDN4
CD639	0	0.960931	0.998	0.613	0	Saliva_Acir	CD63
SLITRK64	0	0.948439	0.782	0.035	0	Saliva_Acir	SLITRK6
LGALS3BP7	0	0.942346	0.93	0.146	0	Saliva_Acir	LGALS3BP
TSTD17	0	0.925411	0.959	0.25	0	Saliva_Acir	TSTD1
TMEM258:	0	0.920319	0.979	0.436	0	Saliva_Acir	TMEM258
SFRP15	0	0.915783	0.839	0.071	0	Saliva_Acir	SFRP1
MT.ND312	0	0.899994	1	0.788	0	Saliva_Acir	MT.ND3
NUPR16	0	0.888952	0.967	0.237	0	Saliva_Acir	NUPR1
MANSC12	0	0.887365	0.821	0.056	0	Saliva_Acir	MANSC1
UQCRQ4	0	0.882173	0.981	0.484	0	Saliva_Acir	UQCRQ
KRT196	0	0.881531	0.916	0.097	0	Saliva_Acir	KRT19
TM7SF23	0	0.868872	0.814	0.08	0	Saliva_Acir	TM7SF2
PPP1R16A!	0	0.861105	0.823	0.08	0	Saliva_Acir	PPP1R16A
SLC12A23	0	0.85725	0.793	0.09	0	Saliva_Acir	SLC12A2
GJC33	0	0.856633	0.766	0.037	0	Saliva_Acir	GJC3
PART13	0	0.856427	0.796	0.048	0	Saliva_Acir	PART1
DCXR3	0	0.850521	0.931	0.197	0	Saliva_Acir	DCXR
METRN5	0	0.85036	0.815	0.07	0	Saliva_Acir	METRN
RPS1711	0	0.8399	0.978	0.197	0	Saliva_Acir	RPS17
CXCL52	0	0.83942	0.446	0.016	0	Saliva_Acir	CXCL5
NCALD3	0	0.836867	0.776	0.046	0	Saliva_Acir	NCALD
TFF32	0	0.836757	0.556	0.128	0	Saliva_Acir	TFF3
ALDH9A13	0	0.826264	0.868	0.125	0	Saliva_Acir	ALDH9A1
PTPRF6	0	0.825265	0.806	0.083	0	Saliva_Acir	PTPRF
TMBIM64	0	0.817941	0.997	0.565	0	Saliva_Acir	TMBIM6
PDIA64	0	0.814889	0.947	0.257	0	Saliva_Acir	PDIA6
HDLBP5	0	0.810302	0.934	0.239	0	Saliva_Acir	HDLBP
MTRNR2L1	0	0.807989	0.928	0.071	0	Saliva_Acir	MTRNR2L10
FKBP23	0	0.806627	0.971	0.331	0	Saliva_Acir	FKBP2
CHI3L21	0	0.79891	0.684	0.037	0	Saliva_Acir	CHI3L2
MGST310	0	0.796836	0.97	0.314	0	Saliva_Acir	MGST3
CHPT15	0	0.795118	0.886	0.184	0	Saliva_Acir	CHPT1
CD1518	0	0.782572	0.934	0.197	0	Saliva_Acir	CD151
RP11.532F	0	0.781963	0.787	0.056	0	Saliva_Acir	RP11.532F12.5
PHGDH4	0	0.781145	0.813	0.092	0	Saliva_Acir	PHGDH
LAMP26	0	0.77989	0.917	0.214	0	Saliva_Acir	LAMP2
SH3BP44	0	0.775732	0.753	0.059	0	Saliva_Acir	SH3BP4
ATOX14	0	0.774136	0.924	0.252	0	Saliva_Acir	ATOX1
LRPAP18	0	0.772795	0.917	0.248	0	Saliva_Acir	LRPAP1
PLA2R12	0	0.767312	0.736	0.033	0	Saliva_Acir	PLA2R1
SLC39A83	0	0.755515	0.801	0.07	0	Saliva_Acir	SLC39A8
CCDC153	0	0.755174	0.653	0.041	0	Saliva_Acir	CCDC15
SELM7	0	0.754508	0.984	0.326	0	Saliva_Acir	SELM
TMEM123	0	0.733352	0.949	0.26	0	Saliva_Acir	TMEM123
MT.CYB12	0	0.732284	1	0.932	0	Saliva_Acir	MT.CYB
KCNN42	0	0.730737	0.773	0.059	0	Saliva_Acir	KCNN4

PDCD44	0	0.730485	0.881	0.162	0	Saliva_Acir	PDCD4
OAT4	0	0.729933	0.876	0.148	0	Saliva_Acir	OAT
PYCR14	0	0.72357	0.762	0.058	0	Saliva_Acir	PYCR1
LIMCH13	0	0.72197	0.794	0.106	0	Saliva_Acir	LIMCH1
SEC61B4	0	0.721316	0.98	0.443	0	Saliva_Acir	SEC61B
DPM33	0	0.717013	0.848	0.159	0	Saliva_Acir	DPM3
RAB4A4	0	0.716815	0.865	0.187	0	Saliva_Acir	RAB4A
JTB4	0	0.71145	0.963	0.351	0	Saliva_Acir	JTB
FAM107B4	0	0.711042	0.907	0.279	0	Saliva_Acir	FAM107B
YIPF25	0	0.705378	0.778	0.087	0	Saliva_Acir	YIPF2
RDH112	0	0.704349	0.787	0.088	0	Saliva_Acir	RDH11
MT.ATP61:	0	0.701435	1	0.895	0	Saliva_Acir	MT.ATP6
SPCS13	0	0.699749	0.981	0.445	0	Saliva_Acir	SPCS1
TTC36	0	0.699556	0.918	0.211	0	Saliva_Acir	TTC3
MPZL15	0	0.693681	0.816	0.118	0	Saliva_Acir	MPZL1
BNIP33	0	0.683865	0.808	0.128	0	Saliva_Acir	BNIP3
ETFB4	0	0.680214	0.917	0.251	0	Saliva_Acir	ETFB
DHCR244	0	0.67862	0.727	0.078	0	Saliva_Acir	DHCR24
FOXC14	0	0.673875	0.762	0.09	0	Saliva_Acir	FOXC1
FAM114A1	0	0.673657	0.807	0.1	0	Saliva_Acir	FAM114A1
CD819	0	0.666652	0.988	0.302	0	Saliva_Acir	CD81
HPSE2	0	0.665304	0.694	0.043	0	Saliva_Acir	HPSE
BACE24	0	0.665	0.82	0.141	0	Saliva_Acir	BACE2
TOMM201	0	0.663088	0.939	0.306	0	Saliva_Acir	TOMM20
PLIN51	0	0.66298	0.666	0.034	0	Saliva_Acir	PLIN5
ITGA67	0	0.661727	0.829	0.113	0	Saliva_Acir	ITGA6
ITM2B5	0	0.656217	0.998	0.697	0	Saliva_Acir	ITM2B
PHPT16	0	0.652641	0.948	0.305	0	Saliva_Acir	PHPT1
DUSP63	0	0.650808	0.765	0.107	0	Saliva_Acir	DUSP6
FGGY4	0	0.649356	0.659	0.066	0	Saliva_Acir	FGGY
ATRAID4	0	0.644403	0.928	0.27	0	Saliva_Acir	ATRAID
KRTCAP34	0	0.641273	0.733	0.059	0	Saliva_Acir	KRTCAP3
RHOBTB35	0	0.635697	0.783	0.097	0	Saliva_Acir	RHOBTB3
TMEM45A	0	0.629208	0.759	0.079	0	Saliva_Acir	TMEM45A
GK54	0	0.629041	0.695	0.053	0	Saliva_Acir	GK5
ELL22	0	0.628923	0.759	0.105	0	Saliva_Acir	ELL2
LRRC264	0	0.623869	0.776	0.055	0	Saliva_Acir	LRRC26
ANK33	0	0.621991	0.692	0.051	0	Saliva_Acir	ANK3
AKR1A16	0	0.606935	0.868	0.183	0	Saliva_Acir	AKR1A1
MT.CO212	0	0.606774	1	0.958	0	Saliva_Acir	MT.CO2
SPCS34	0	0.60431	0.884	0.23	0	Saliva_Acir	SPCS3
ETV11	0	0.6035	0.628	0.03	0	Saliva_Acir	ETV1
RPS27L2	0	0.593831	0.979	0.428	0	Saliva_Acir	RPS27L
CPD3	0	0.58749	0.752	0.1	0	Saliva_Acir	CPD
CDC42EP5:	0	0.584066	0.89	0.174	0	Saliva_Acir	CDC42EP5
PRKAR2B1	0	0.581879	0.626	0.03	0	Saliva_Acir	PRKAR2B
CD142	0	0.581462	0.776	0.085	0	Saliva_Acir	CD14

MEIS22	0	0.578375	0.717	0.083	0	Saliva_Acir	MEIS2
KDELR24	0	0.57395	0.933	0.299	0	Saliva_Acir	KDELR2
ECHDC13	0	0.573633	0.741	0.097	0	Saliva_Acir	ECHDC1
ACTR3B3	0	0.572506	0.636	0.049	0	Saliva_Acir	ACTR3B
KDELR35	0	0.563144	0.71	0.065	0	Saliva_Acir	KDELR3
H3F3A10	0	0.560168	0.996	0.462	0	Saliva_Acir	H3F3A
PROM14	0	0.553266	0.668	0.063	0	Saliva_Acir	PROM1
SERF23	0	0.552012	1	0.817	0	Saliva_Acir	SERF2
SPDEF3	0	0.549361	0.685	0.043	0	Saliva_Acir	SPDEF
ATP1A16	0	0.547336	0.914	0.234	0	Saliva_Acir	ATP1A1
SERP14	0	0.54653	0.981	0.532	0	Saliva_Acir	SERP1
DSC24	0	0.545759	0.647	0.049	0	Saliva_Acir	DSC2
KDELR14	0	0.543927	0.889	0.23	0	Saliva_Acir	KDELR1
SCD1	0	0.542264	0.629	0.062	0	Saliva_Acir	SCD
DHRS76	0	0.541361	0.874	0.187	0	Saliva_Acir	DHRS7
TPD524	0	0.540301	0.768	0.106	0	Saliva_Acir	TPD52
CREB3L13	0	0.538704	0.663	0.055	0	Saliva_Acir	CREB3L1
ANG4	0	0.537848	0.725	0.08	0	Saliva_Acir	ANG
ASCL21	0	0.537667	0.613	0.033	0	Saliva_Acir	ASCL2
SMIM15	0	0.535867	0.656	0.051	0	Saliva_Acir	SMIM1
EPRS8	0	0.535229	0.774	0.112	0	Saliva_Acir	EPRS
AGFG23	0	0.531345	0.753	0.117	0	Saliva_Acir	AGFG2
EIF4EBP13	0	0.529376	0.784	0.13	0	Saliva_Acir	EIF4EBP1
PRKACB4	0	0.523206	0.717	0.081	0	Saliva_Acir	PRKACB
NENF6	0	0.52208	0.932	0.309	0	Saliva_Acir	NENF
INPP5D2	0	0.521178	0.653	0.04	0	Saliva_Acir	INPP5D
TMEM63A	0	0.517877	0.67	0.061	0	Saliva_Acir	TMEM63A
TM9SF24	0	0.514972	0.83	0.174	0	Saliva_Acir	TM9SF2
GRN3	0	0.513922	0.941	0.291	0	Saliva_Acir	GRN
AC159540.	0	0.511607	0.568	0.046	0	Saliva_Acir	AC159540.1
OS93	0	0.509267	0.879	0.205	0	Saliva_Acir	OS9
ITPR26	0	0.508803	0.664	0.062	0	Saliva_Acir	ITPR2
RRAGD3	0	0.508795	0.657	0.074	0	Saliva_Acir	RRAGD
C1orf1225	0	0.508648	0.819	0.163	0	Saliva_Acir	C1orf122
MESP12	0	0.506684	0.582	0.037	0	Saliva_Acir	MESP1
LINC00342	0	0.505676	0.56	0.037	0	Saliva_Acir	LINC00342
MAGED13	0	0.504944	0.683	0.066	0	Saliva_Acir	MAGED1
TCEA33	0	0.502954	0.704	0.08	0	Saliva_Acir	TCEA3
RPN23	0	0.500273	0.897	0.225	0	Saliva_Acir	RPN2
RABAC19	0	0.496322	0.968	0.386	0	Saliva_Acir	RABAC1
BARX23	0	0.494672	0.593	0.046	0	Saliva_Acir	BARX2
EHF3	0	0.490921	0.897	0.222	0	Saliva_Acir	EHF
SSFA23	0	0.486253	0.778	0.148	0	Saliva_Acir	SSFA2
IRX35	0	0.484691	0.631	0.054	0	Saliva_Acir	IRX3
NDRG2	0	0.48367	0.99	0.346	0	Saliva_Acir	NDRG2
CKAP44	0	0.481283	0.786	0.145	0	Saliva_Acir	CKAP4
CCPG16	0	0.479991	0.823	0.147	0	Saliva_Acir	CCPG1

PKDCC3	0	0.478938	0.552	0.036	0	Saliva_Acir	PKDCC
PAIP2B2	0	0.478539	0.613	0.044	0	Saliva_Acir	PAIP2B
GOLGA46	0	0.477764	0.867	0.215	0	Saliva_Acir	GOLGA4
SMCO43	0	0.47664	0.71	0.099	0	Saliva_Acir	SMCO4
CANT13	0	0.474999	0.7	0.089	0	Saliva_Acir	CANT1
TMEM593	0	0.474618	0.991	0.597	0	Saliva_Acir	TMEM59
GPT23	0	0.474174	0.633	0.066	0	Saliva_Acir	GPT2
C8orf599	0	0.473917	0.841	0.215	0	Saliva_Acir	C8orf59
ZNF6524	0	0.473472	0.677	0.076	0	Saliva_Acir	ZNF652
YIPF33	0	0.472883	0.842	0.214	0	Saliva_Acir	YIPF3
NFIB8	0	0.47165	0.847	0.17	0	Saliva_Acir	NFIB
INADL3	0	0.468274	0.624	0.069	0	Saliva_Acir	INADL
FASN1	0	0.467526	0.543	0.034	0	Saliva_Acir	FASN
PKP24	0	0.467391	0.558	0.033	0	Saliva_Acir	PKP2
FARP13	0	0.466247	0.662	0.081	0	Saliva_Acir	FARP1
APIP4	0	0.464757	0.641	0.079	0	Saliva_Acir	APIP
MXD46	0	0.464612	0.732	0.1	0	Saliva_Acir	MXD4
PTH2R1	0	0.459367	0.533	0.013	0	Saliva_Acir	PTH2R
FAM129A7	0	0.457814	0.67	0.086	0	Saliva_Acir	FAM129A
SMIM19	0	0.455824	0.798	0.179	0	Saliva_Acir	SMIM19
MAPK132	0	0.454202	0.68	0.091	0	Saliva_Acir	MAPK13
ARHGAP18	0	0.451918	0.673	0.077	0	Saliva_Acir	ARHGAP18
TMEM87A	0	0.448901	0.782	0.151	0	Saliva_Acir	TMEM87A
SCGB3A14	0	0.44854	0.67	0.123	0	Saliva_Acir	SCGB3A1
QDPR1	0	0.444805	0.678	0.075	0	Saliva_Acir	QDPR
TECR4	0	0.442784	0.934	0.311	0	Saliva_Acir	TECR
SCARB27	0	0.442551	0.775	0.137	0	Saliva_Acir	SCARB2
RAB3D3	0	0.442387	0.583	0.039	0	Saliva_Acir	RAB3D
PCYOX15	0	0.441103	0.73	0.112	0	Saliva_Acir	PCYOX1
TSTA32	0	0.439492	0.791	0.156	0	Saliva_Acir	TSTA3
SREBF11	0	0.439115	0.631	0.074	0	Saliva_Acir	SREBF1
NDUFC23	0	0.43899	0.932	0.368	0	Saliva_Acir	NDUFC2
CHID14	0	0.438425	0.803	0.15	0	Saliva_Acir	CHID1
CADM13	0	0.437612	0.677	0.085	0	Saliva_Acir	CADM1
MSMB2	0	0.437267	0.465	0.024	0	Saliva_Acir	MSMB
PAM4	0	0.436394	0.783	0.121	0	Saliva_Acir	PAM
COBL2	0	0.436315	0.546	0.034	0	Saliva_Acir	COBL
CHRM32	0	0.436102	0.512	0.023	0	Saliva_Acir	CHRM3
BSPRY2	0	0.435723	0.553	0.034	0	Saliva_Acir	BSPRY
ARL16	0	0.434773	0.828	0.186	0	Saliva_Acir	ARL1
SOX25	0	0.433418	0.544	0.029	0	Saliva_Acir	SOX2
NAGLU4	0	0.432074	0.623	0.051	0	Saliva_Acir	NAGLU
GTF2I9	0	0.428602	0.757	0.134	0	Saliva_Acir	GTF2I
VAMP83	0	0.428335	0.976	0.391	0	Saliva_Acir	VAMP8
FADS2	0	0.426488	0.477	0.015	0	Saliva_Acir	FADS2
MTRNR2L8	0	0.425618	0.986	0.264	0	Saliva_Acir	MTRNR2L8
TNFRSF193	0	0.425435	0.576	0.049	0	Saliva_Acir	TNFRSF19

MTURN3	0	0.423806	0.623	0.062	0	Saliva_Acir	MTURN
GNPTAB3	0	0.423771	0.643	0.071	0	Saliva_Acir	GNPTAB
CTBS4	0	0.422676	0.661	0.085	0	Saliva_Acir	CTBS
STK391	0	0.422477	0.67	0.097	0	Saliva_Acir	STK39
AGR22	0	0.420856	0.622	0.136	0	Saliva_Acir	AGR2
ANXA35	0	0.418969	0.762	0.135	0	Saliva_Acir	ANXA3
FMO51	0	0.418077	0.509	0.024	0	Saliva_Acir	FMO5
ACSL33	0	0.415243	0.74	0.14	0	Saliva_Acir	ACSL3
SNHG183	0	0.41361	0.587	0.045	0	Saliva_Acir	SNHG18
PPIB5	0	0.411343	0.954	0.376	0	Saliva_Acir	PPIB
PRR44	0	0.411332	0.713	0.091	0	Saliva_Acir	PRR4
SFT2D11	0	0.408246	0.797	0.184	0	Saliva_Acir	SFT2D1
SEZ6L23	0	0.408221	0.531	0.03	0	Saliva_Acir	SEZ6L2
NDUFC13	0	0.406021	0.874	0.262	0	Saliva_Acir	NDUFC1
GATM3	0	0.404979	0.57	0.061	0	Saliva_Acir	GATM
CHCHD109	0	0.401613	0.895	0.224	0	Saliva_Acir	CHCHD10
FNDC3A5	0	0.39966	0.674	0.098	0	Saliva_Acir	FNDC3A
CRNDE3	0	0.398846	0.776	0.135	0	Saliva_Acir	CRNDE
CGREF13	0	0.398814	0.524	0.033	0	Saliva_Acir	CGREF1
CTDSPL5	0	0.398626	0.613	0.068	0	Saliva_Acir	CTDSPL
DPP4	0	0.398399	0.569	0.041	0	Saliva_Acir	DPP4
MT.ND4L1	0	0.397324	0.985	0.398	0	Saliva_Acir	MT.ND4L
FKBP114	0	0.396141	0.842	0.131	0	Saliva_Acir	FKBP11
TNFRSF11A	0	0.395115	0.552	0.04	0	Saliva_Acir	TNFRSF11A
CLPTM1L5	0	0.394839	0.668	0.073	0	Saliva_Acir	CLPTM1L
FAM84B4	0	0.3947	0.615	0.064	0	Saliva_Acir	FAM84B
TMED46	0	0.393852	0.801	0.153	0	Saliva_Acir	TMED4
ANAPC55	0	0.392517	0.769	0.141	0	Saliva_Acir	ANAPC5
FUCA14	0	0.391881	0.712	0.121	0	Saliva_Acir	FUCA1
SLC25A311	0	0.390395	0.961	0.439	0	Saliva_Acir	SLC25A3
CYB5613	0	0.389925	0.612	0.071	0	Saliva_Acir	CYB561
SYNGR12	0	0.389076	0.707	0.124	0	Saliva_Acir	SYNGR1
TC2N3	0	0.388942	0.763	0.134	0	Saliva_Acir	TC2N
HOOK24	0	0.385512	0.647	0.085	0	Saliva_Acir	HOOK2
ATP5G38	0	0.382446	0.924	0.344	0	Saliva_Acir	ATP5G3
DEPTOR2	0	0.381987	0.532	0.042	0	Saliva_Acir	DEPTOR
CLSTN14	0	0.381328	0.678	0.104	0	Saliva_Acir	CLSTN1
VKORC15	0	0.37996	0.835	0.204	0	Saliva_Acir	VKORC1
FOLH13	0	0.378889	0.48	0.02	0	Saliva_Acir	FOLH1
PKP45	0	0.378407	0.565	0.062	0	Saliva_Acir	PKP4
CD593	0	0.376449	0.991	0.5	0	Saliva_Acir	CD59
MYEOV22	0	0.37592	0.917	0.343	0	Saliva_Acir	MYEOV2
TMC43	0	0.375345	0.582	0.064	0	Saliva_Acir	TMC4
ALG54	0	0.374559	0.747	0.135	0	Saliva_Acir	ALG5
TMEM205	0	0.374359	0.83	0.184	0	Saliva_Acir	TMEM205
MT.CO310	0	0.374345	1	0.977	0	Saliva_Acir	MT.CO3
CES31	0	0.374266	0.468	0.015	0	Saliva_Acir	CES3

RAD51B1	0	0.373551	0.502	0.026	0	Saliva_Acir	RAD51B
LRP53	0	0.373243	0.504	0.028	0	Saliva_Acir	LRP5
NME44	0	0.369779	0.726	0.124	0	Saliva_Acir	NME4
HOMER22	0	0.368914	0.515	0.035	0	Saliva_Acir	HOMER2
DAP4	0	0.368863	0.72	0.125	0	Saliva_Acir	DAP
BTG25	0	0.368847	0.629	0.22	0	Saliva_Acir	BTG2
RBM471	0	0.367721	0.827	0.198	0	Saliva_Acir	RBM47
SLC39A91	0	0.363243	0.528	0.045	0	Saliva_Acir	SLC39A9
COPB25	0	0.362668	0.721	0.137	0	Saliva_Acir	COPB2
CTSZ4	0	0.360714	0.855	0.17	0	Saliva_Acir	CTSZ
SEC634	0	0.359987	0.762	0.152	0	Saliva_Acir	SEC63
WWC12	0	0.358526	0.585	0.072	0	Saliva_Acir	WWC1
HSBP1	0	0.357888	0.95	0.415	0	Saliva_Acir	HSBP1
LMAN17	0	0.356936	0.81	0.173	0	Saliva_Acir	LMAN1
CERS42	0	0.356853	0.571	0.052	0	Saliva_Acir	CERS4
BEX42	0	0.355258	0.753	0.151	0	Saliva_Acir	BEX4
C1orf1151	0	0.354484	0.496	0.032	0	Saliva_Acir	C1orf115
NDUFB78	0	0.353934	0.942	0.354	0	Saliva_Acir	NDUFB7
MLEC5	0	0.351732	0.774	0.17	0	Saliva_Acir	MLEC
GPAA15	0	0.350209	0.73	0.124	0	Saliva_Acir	GPAA1
PDIA33	0	0.348495	0.912	0.303	0	Saliva_Acir	PDIA3
TBC1D241	0	0.346389	0.48	0.026	0	Saliva_Acir	TBC1D24
MT.ATP89	0	0.34623	0.772	0.115	0	Saliva_Acir	MT.ATP8
AACS2	0	0.344399	0.514	0.049	0	Saliva_Acir	AACS
ITGB44	0	0.343519	0.599	0.071	0	Saliva_Acir	ITGB4
PBX15	0	0.341262	0.639	0.097	0	Saliva_Acir	PBX1
HIPK26	0	0.341169	0.542	0.064	0	Saliva_Acir	HIPK2
IDH12	0	0.340961	0.643	0.108	0	Saliva_Acir	IDH1
KIF21A5	0	0.340326	0.571	0.065	0	Saliva_Acir	KIF21A
HEXB5	0	0.337263	0.759	0.142	0	Saliva_Acir	HEXB
TPT1.AS13	0	0.337018	0.486	0.08	0	Saliva_Acir	TPT1.AS1
KRT74	0	0.336871	0.918	0.251	0	Saliva_Acir	KRT7
MT.ND412	0	0.33651	0.999	0.952	0	Saliva_Acir	MT.ND4
SH3YL12	0	0.33573	0.771	0.191	0	Saliva_Acir	SH3YL1
ARHGEF28	0	0.333843	0.493	0.04	0	Saliva_Acir	ARHGEF28
NR2F65	0	0.333662	0.59	0.067	0	Saliva_Acir	NR2F6
PLA2G12A	0	0.332247	0.59	0.08	0	Saliva_Acir	PLA2G12A
GFPT14	0	0.331621	0.651	0.11	0	Saliva_Acir	GFPT1
WIPI13	0	0.33055	0.607	0.082	0	Saliva_Acir	WIPI1
NDUFB108	0	0.330361	0.943	0.367	0	Saliva_Acir	NDUFB10
MRPL208	0	0.330227	0.865	0.251	0	Saliva_Acir	MRPL20
PPP1R9A3	0	0.32948	0.467	0.037	0	Saliva_Acir	PPP1R9A
NDUFB24	0	0.329223	0.947	0.356	0	Saliva_Acir	NDUFB2
ALDH18A1	0	0.328198	0.511	0.042	0	Saliva_Acir	ALDH18A1
ERGIC14	0	0.327669	0.677	0.116	0	Saliva_Acir	ERGIC1
SEC61G3	0	0.327319	0.932	0.398	0	Saliva_Acir	SEC61G
ARSD2	0	0.326783	0.503	0.044	0	Saliva_Acir	ARSD

CHCHD15	0	0.326635	0.736	0.155	0	Saliva_Acir	CHCHD1
XAB23	0	0.326361	0.586	0.08	0	Saliva_Acir	XAB2
PXMP41	0	0.324981	0.473	0.035	0	Saliva_Acir	PXMP4
TM9SF33	0	0.323388	0.805	0.217	0	Saliva_Acir	TM9SF3
SEC61A14	0	0.323357	0.714	0.133	0	Saliva_Acir	SEC61A1
MKNK22	0	0.322381	0.652	0.1	0	Saliva_Acir	MKNK2
CANX7	0	0.32084	0.895	0.293	0	Saliva_Acir	CANX
SURF44	0	0.320418	0.741	0.159	0	Saliva_Acir	SURF4
CREB3L43	0	0.318894	0.506	0.043	0	Saliva_Acir	CREB3L4
PAPSS14	0	0.318883	0.623	0.093	0	Saliva_Acir	PAPSS1
CD1641	0	0.316845	0.923	0.353	0	Saliva_Acir	CD164
PHKB6	0	0.31604	0.613	0.089	0	Saliva_Acir	PHKB
COPG14	0	0.316001	0.571	0.08	0	Saliva_Acir	COPG1
YIF1A5	0	0.314757	0.756	0.154	0	Saliva_Acir	YIF1A
CTA.293F1	0	0.314646	0.45	0.027	0	Saliva_Acir	CTA.293F17.1
SMDT12	0	0.314515	0.926	0.355	0	Saliva_Acir	SMDT1
SORBS26	0	0.313163	0.645	0.095	0	Saliva_Acir	SORBS2
SLC31A13	0	0.309419	0.53	0.066	0	Saliva_Acir	SLC31A1
OGFOD31	0	0.308807	0.576	0.067	0	Saliva_Acir	OGFOD3
RAP1GAP1	0	0.308194	0.431	0.022	0	Saliva_Acir	RAP1GAP
FRMD4B5	0	0.307705	0.649	0.11	0	Saliva_Acir	FRMD4B
AKT13	0	0.307084	0.583	0.072	0	Saliva_Acir	AKT1
ERLEC13	0	0.30642	0.745	0.164	0	Saliva_Acir	ERLEC1
MTA12	0	0.30587	0.549	0.067	0	Saliva_Acir	MTA1
SELENBP17	0	0.305851	0.578	0.059	0	Saliva_Acir	SELENBP1
DMD3	0	0.305642	0.477	0.037	0	Saliva_Acir	DMD
TCEAL22	0	0.304498	0.434	0.027	0	Saliva_Acir	TCEAL2
MAOA	0	0.303942	0.474	0.038	0	Saliva_Acir	MAOA
TSPAN122	0	0.302511	0.469	0.038	0	Saliva_Acir	TSPAN12
SLC26A23	0	0.301551	0.468	0.065	0	Saliva_Acir	SLC26A2
MCEE1	0	0.299381	0.517	0.041	0	Saliva_Acir	MCEE
CREG12	0	0.297434	0.694	0.127	0	Saliva_Acir	CREG1
FAHD2A1	0	0.296936	0.52	0.073	0	Saliva_Acir	FAHD2A
TMEM1254	0	0.296936	0.458	0.036	0	Saliva_Acir	TMEM125
GOLGB16	0	0.296396	0.768	0.181	0	Saliva_Acir	GOLGB1
PODXL3	0	0.29603	0.499	0.036	0	Saliva_Acir	PODXL
CYP2J21	0	0.295708	0.407	0.017	0	Saliva_Acir	CYP2J2
TMEM561	0	0.293011	0.438	0.03	0	Saliva_Acir	TMEM56
ACAT1	0	0.290345	0.667	0.117	0	Saliva_Acir	ACAT1
SLC37A21	0	0.290208	0.404	0.025	0	Saliva_Acir	SLC37A2
TXNDC154	0	0.288701	0.681	0.104	0	Saliva_Acir	TXNDC15
NANS4	0	0.288395	0.811	0.212	0	Saliva_Acir	NANS
GGT62	0	0.288309	0.422	0.027	0	Saliva_Acir	GGT6
PDE3B1	0	0.287674	0.444	0.029	0	Saliva_Acir	PDE3B
CCNC1	0	0.287498	0.764	0.186	0	Saliva_Acir	CCNC
MGST23	0	0.286512	0.779	0.191	0	Saliva_Acir	MGST2
DPP75	0	0.285807	0.874	0.239	0	Saliva_Acir	DPP7

FAM120A4	0	0.283805	0.67	0.131	0	Saliva_Acir	FAM120A
PERP3	0	0.282623	0.967	0.347	0	Saliva_Acir	PERP
PIK3AP11	0	0.280073	0.446	0.03	0	Saliva_Acir	PIK3AP1
TSPAN332	0	0.279705	0.451	0.031	0	Saliva_Acir	TSPAN33
TMED22	0	0.279175	0.917	0.327	0	Saliva_Acir	TMED2
BOLA31	0	0.278865	0.617	0.099	0	Saliva_Acir	BOLA3
KCNQ1OT1	0	0.278349	0.645	0.125	0	Saliva_Acir	KCNQ1OT1
KCNK64	0	0.277979	0.591	0.085	0	Saliva_Acir	KCNK6
SGSM32	0	0.277706	0.637	0.095	0	Saliva_Acir	SGSM3
SYPL13	0	0.277403	0.858	0.244	0	Saliva_Acir	SYPL1
DNAJC14	0	0.277176	0.715	0.146	0	Saliva_Acir	DNAJC1
NDUFB810	0	0.273957	0.74	0.16	0	Saliva_Acir	NDUFB8
NFIA4	0	0.273937	0.758	0.154	0	Saliva_Acir	NFIA
SLC39A63	0	0.271869	0.557	0.084	0	Saliva_Acir	SLC39A6
NFIX5	0	0.271526	0.6	0.094	0	Saliva_Acir	NFIX
FAM171B1	0	0.269108	0.417	0.024	0	Saliva_Acir	FAM171B
MRPL544	0	0.268454	0.796	0.191	0	Saliva_Acir	MRPL54
MSRB26	0	0.268398	0.635	0.113	0	Saliva_Acir	MSRB2
DTNB1	0	0.267672	0.426	0.034	0	Saliva_Acir	DTNB
CDH15	0	0.267641	0.503	0.061	0	Saliva_Acir	CDH1
DUS1L4	0	0.267147	0.595	0.081	0	Saliva_Acir	DUS1L
CHPF5	0	0.266472	0.544	0.068	0	Saliva_Acir	CHPF
GBAS3	0	0.266207	0.648	0.108	0	Saliva_Acir	GBAS
ECI17	0	0.266037	0.665	0.121	0	Saliva_Acir	ECI1
MACF16	0	0.265864	0.714	0.15	0	Saliva_Acir	MACF1
SNRNP251	0	0.265451	0.585	0.081	0	Saliva_Acir	SNRNP25
ERBB31	0	0.265237	0.715	0.143	0	Saliva_Acir	ERBB3
NDUFAB17	0	0.264873	0.823	0.227	0	Saliva_Acir	NDUFAB1
AUTS22	0	0.26449	0.463	0.045	0	Saliva_Acir	AUTS2
TMEM214	0	0.26336	0.515	0.058	0	Saliva_Acir	TMEM214
ADI17	0	0.261841	0.802	0.203	0	Saliva_Acir	ADI1
SLC25A46	0	0.261819	0.627	0.098	0	Saliva_Acir	SLC25A4
PITPNA3	0	0.261681	0.562	0.087	0	Saliva_Acir	PITPNA
KIAA15222	0	0.260166	0.459	0.057	0	Saliva_Acir	KIAA1522
CADPS24	0	0.259394	0.414	0.033	0	Saliva_Acir	CADPS2
HID14	0	0.258998	0.438	0.034	0	Saliva_Acir	HID1
ZNF7044	0	0.258502	0.489	0.056	0	Saliva_Acir	ZNF704
DAD16	0	0.258347	0.938	0.381	0	Saliva_Acir	DAD1
CLCN34	0	0.257384	0.616	0.114	0	Saliva_Acir	CLCN3
CMTM85	0	0.256981	0.497	0.055	0	Saliva_Acir	CMTM8
TFCP2L15	0	0.25696	0.397	0.036	0	Saliva_Acir	TFCP2L1
OSTC3	0	0.256519	0.853	0.259	0	Saliva_Acir	OSTC
COMT	0	0.254873	0.744	0.164	0	Saliva_Acir	COMT
TRAK14	0	0.25438	0.483	0.052	0	Saliva_Acir	TRAK1
SSR35	0	0.25416	0.87	0.283	0	Saliva_Acir	SSR3
SECISBP2L	0	0.253174	0.713	0.167	0	Saliva_Acir	SECISBP2L
MDK3	0	0.2531	0.695	0.131	0	Saliva_Acir	MDK

OSBPL1A5	0	0.252085	0.671	0.144	0	Saliva_Acir	OSBPL1A
CMYA53	0	0.251698	0.369	0.023	0	Saliva_Acir	CMYA5
FAM210B7	0	0.251164	0.579	0.078	0	Saliva_Acir	FAM210B
ATP6AP13	0	0.250968	0.702	0.143	0	Saliva_Acir	ATP6AP1
NAALADL2	0	0.250835	0.404	0.038	0	Saliva_Acir	NAALADL2
UQCR102	9.9E-299	0.266739	0.945	0.422	1.6E-294	Saliva_Acir	UQCR10
OST42	9.6E-251	0.338273	0.972	0.486	1.5E-246	Saliva_Acir	OST4
RPL7A9	1.8E-195	0.396746	1	0.808	2.9E-191	Saliva_Acir	RPL7A
RPL398	1.3E-165	0.330742	0.999	0.667	2E-161	Saliva_Acir	RPL39
HINT17	1.9E-160	0.258224	0.981	0.557	3.1E-156	Saliva_Acir	HINT1
EEF1A17	6.9E-102	0.274909	1	0.912	1.11E-97	Saliva_Acir	EEF1A1
RPL309	2.84E-96	0.304042	1	0.84	4.52E-92	Saliva_Acir	RPL30
TFF33	0	3.000427	0.985	0.135	0	Saliva_Acir	TFF3
LYZ4	0	2.963945	1	0.333	0	Saliva_Acir	LYZ
C6orf585	0	2.781021	1	0.211	0	Saliva_Acir	C6orf58
AGR23	0	2.646624	0.897	0.145	0	Saliva_Acir	AGR2
ZG16B4	0	2.455788	1	0.411	0	Saliva_Acir	ZG16B
GNAS5	0	2.298352	0.999	0.579	0	Saliva_Acir	GNAS
AZGP15	0	2.098356	0.946	0.201	0	Saliva_Acir	AZGP1
SLPI4	0	1.98028	1	0.491	0	Saliva_Acir	SLPI
TCN15	0	1.815246	0.709	0.152	0	Saliva_Acir	TCN1
MTRNR2L1	0	1.721499	0.628	0.122	0	Saliva_Acir	MTRNR2L1
RP11.1143	0	1.567642	0.951	0.224	0	Saliva_Acir	RP11.1143G9.4
MT.CYB13	0	1.485997	1	0.933	0	Saliva_Acir	MT.CYB
MTRNR2L1	0	1.451058	0.841	0.265	0	Saliva_Acir	MTRNR2L12
SCGB3A15	0	1.387279	0.812	0.135	0	Saliva_Acir	SCGB3A1
MT.CO311	0	1.332183	1	0.977	0	Saliva_Acir	MT.CO3
MT.CO213	0	1.304729	1	0.959	0	Saliva_Acir	MT.CO2
MT.ATP61	1.2E-305	1.349846	1	0.897	1.9E-301	Saliva_Acir	MT.ATP6
MT.ND313	2.5E-299	1.440627	1	0.793	4E-295	Saliva_Acir	MT.ND3
MT.ND413	2.2E-234	0.949819	1	0.953	3.6E-230	Saliva_Acir	MT.ND4
TPT18	2.6E-224	0.974354	1	0.958	4.1E-220	Saliva_Acir	TPT1
MT.ND211	5.3E-213	0.941765	0.997	0.906	8.5E-209	Saliva_Acir	MT.ND2
MT.ND111	2.9E-199	0.877237	0.993	0.927	4.6E-195	Saliva_Acir	MT.ND1
XBP16	3E-172	1.857085	0.691	0.34	4.8E-168	Saliva_Acir	XBP1
MT.ND512	1.1E-138	1.041873	0.875	0.701	1.7E-134	Saliva_Acir	MT.ND5
MT.CO111	6E-128	0.427095	1	0.985	9.6E-124	Saliva_Acir	MT.CO1
SSR46	2.9E-117	1.040952	0.779	0.547	4.7E-113	Saliva_Acir	SSR4
FAM3D5	3.6E-107	1.518636	0.53	0.238	5.8E-103	Saliva_Acir	FAM3D
FXYD37	4E-87	1.572227	0.473	0.231	6.37E-83	Saliva_Acir	FXYD3
CLDN105	2.87E-86	1.63146	0.288	0.094	4.58E-82	Saliva_Acir	CLDN10
DBI6	6.55E-70	1.415369	0.578	0.414	1.05E-65	Saliva_Acir	DBI
CCL286	2.6E-68	1.557412	0.291	0.11	4.14E-64	Saliva_Acir	CCL28
S100A17	3.19E-61	1.434801	0.271	0.103	5.08E-57	Saliva_Acir	S100A1
C8orf46	1.3E-56	1.60416	0.283	0.119	2.07E-52	Saliva_Acir	C8orf4
GOLM12	4.17E-50	1.386703	0.235	0.089	6.65E-46	Saliva_Acir	GOLM1
NDUFA48	1E-49	1.034245	0.615	0.538	1.6E-45	Saliva_Acir	NDUFA4

MT.ND4L1	2.12E-48	1.260851	0.532	0.416	3.38E-44	Saliva_Acir	MT.ND4L
SEC11C6	3.53E-47	1.138171	0.408	0.241	5.62E-43	Saliva_Acir	SEC11C
MTRNR2L8	3.92E-45	0.680474	0.474	0.285	6.25E-41	Saliva_Acir	MTRNR2L8
MSLN2	2.1E-44	1.134492	0.126	0.034	3.34E-40	Saliva_Acir	MSLN
NKX3.12	2.47E-43	1.135082	0.13	0.037	3.94E-39	Saliva_Acir	NKX3.1
KIAA13245	1.18E-40	1.373105	0.243	0.109	1.88E-36	Saliva_Acir	KIAA1324
XIST11	1.19E-40	1.403269	0.261	0.122	1.9E-36	Saliva_Acir	XIST
MTRNR2L1	9.12E-39	0.97077	0.231	0.097	1.46E-34	Saliva_Acir	MTRNR2L10
FAM46A4	2.94E-36	1.394941	0.298	0.164	4.7E-32	Saliva_Acir	FAM46A
TMED35	4.09E-31	1.386447	0.385	0.278	6.52E-27	Saliva_Acir	TMED3
SLC12A24	2.86E-29	1.469668	0.223	0.112	4.56E-25	Saliva_Acir	SLC12A2
EEF1A18	4.38E-28	0.320183	0.982	0.915	6.99E-24	Saliva_Acir	EEF1A1
PRDX45	4.69E-26	1.228768	0.359	0.256	7.48E-22	Saliva_Acir	PRDX4
ITM2B6	1.11E-24	0.563894	0.687	0.707	1.77E-20	Saliva_Acir	ITM2B
EPCAM5	1.72E-23	1.301722	0.231	0.13	2.74E-19	Saliva_Acir	EPCAM
H3F3A11	1.05E-22	0.634301	0.569	0.478	1.67E-18	Saliva_Acir	H3F3A
KRT197	1.33E-22	0.854198	0.228	0.122	2.11E-18	Saliva_Acir	KRT19
GLYATL23	9.24E-19	0.768902	0.073	0.024	1.47E-14	Saliva_Acir	GLYATL2
TSPAN81	6.16E-17	0.974068	0.137	0.066	9.82E-13	Saliva_Acir	TSPAN8
NPDC16	7E-17	1.236245	0.254	0.173	1.12E-12	Saliva_Acir	NPDC1
SPDEF4	4.31E-16	1.063689	0.13	0.063	6.88E-12	Saliva_Acir	SPDEF
RPS1712	6.75E-16	0.746141	0.309	0.222	1.08E-11	Saliva_Acir	RPS17
HID15	1.12E-14	1.005134	0.103	0.047	1.78E-10	Saliva_Acir	HID1
SMIM142	1.62E-14	0.338674	0.102	0.251	2.58E-10	Saliva_Acir	SMIM14
CA23	1.91E-14	1.222376	0.168	0.098	3.04E-10	Saliva_Acir	CA2
SLC26A24	1.14E-13	1.095883	0.141	0.077	1.82E-09	Saliva_Acir	SLC26A2
SH3YL13	1.14E-13	0.26485	0.081	0.211	1.82E-09	Saliva_Acir	SH3YL1
SRP9	3.03E-13	0.327567	0.161	0.344	4.83E-09	Saliva_Acir	SRP9
RRBP14	4.95E-13	0.297104	0.146	0.313	7.9E-09	Saliva_Acir	RRBP1
ARHGAP53	5.04E-13	0.308871	0.081	0.207	8.03E-09	Saliva_Acir	ARHGAP5
SFT2D12	7.47E-13	0.261222	0.081	0.205	1.19E-08	Saliva_Acir	SFT2D1
P4HB5	1.26E-12	0.915427	0.422	0.419	2E-08	Saliva_Acir	P4HB
SPINT26	1.36E-12	1.106082	0.348	0.314	2.17E-08	Saliva_Acir	SPINT2
EIF4B4	1.66E-12	0.308311	0.14	0.302	2.65E-08	Saliva_Acir	EIF4B
CLDN46	2.11E-12	1.122418	0.152	0.09	3.37E-08	Saliva_Acir	CLDN4
NDUFC24	2.66E-12	0.26931	0.191	0.388	4.25E-08	Saliva_Acir	NDUFC2
GALNT72	3.81E-12	1.043133	0.12	0.063	6.08E-08	Saliva_Acir	GALNT7
IL6ST3	4.74E-12	0.264837	0.096	0.224	7.57E-08	Saliva_Acir	IL6ST
CAPN22	5.19E-12	0.285496	0.15	0.314	8.28E-08	Saliva_Acir	CAPN2
ATP5B3	6.03E-12	0.3162	0.184	0.373	9.62E-08	Saliva_Acir	ATP5B
YIPF63	6.05E-12	0.258176	0.049	0.147	9.65E-08	Saliva_Acir	YIPF6
YIPF34	6.93E-12	0.385964	0.103	0.235	1.1E-07	Saliva_Acir	YIPF3
DERL22	7.33E-12	0.287287	0.067	0.176	1.17E-07	Saliva_Acir	DERL2
SF3B16	8.01E-12	0.33854	0.115	0.255	1.28E-07	Saliva_Acir	SF3B1
AKAP96	1.04E-11	0.330665	0.126	0.272	1.66E-07	Saliva_Acir	AKAP9
TMED23	1.19E-11	0.303762	0.172	0.348	1.89E-07	Saliva_Acir	TMED2
PHB5	1.38E-11	1.112216	0.291	0.241	2.21E-07	Saliva_Acir	PHB

COPZ13	1.75E-11	0.377822	0.095	0.218	2.8E-07	Saliva_Acir	COPZ1
TFG1	1.92E-11	0.360427	0.077	0.189	3.06E-07	Saliva_Acir	TFG
HMGN34	2.3E-11	0.290373	0.133	0.28	3.67E-07	Saliva_Acir	HMGN3
CD6310	2.36E-11	0.394665	0.625	0.625	3.76E-07	Saliva_Acir	CD63
NDUFC14	2.97E-11	0.332042	0.135	0.283	4.73E-07	Saliva_Acir	NDUFC1
GTF3C62	3.06E-11	0.308781	0.087	0.203	4.87E-07	Saliva_Acir	GTF3C6
ELF53	3.37E-11	1.057302	0.125	0.07	5.37E-07	Saliva_Acir	ELF5
MRPL413	4.8E-11	0.388367	0.152	0.311	7.66E-07	Saliva_Acir	MRPL41
ATP2A23	5.92E-11	0.336876	0.071	0.177	9.44E-07	Saliva_Acir	ATP2A2
SSR36	9.85E-11	0.274396	0.151	0.303	1.57E-06	Saliva_Acir	SSR3
TSPAN135	1.06E-10	1.282229	0.214	0.157	1.69E-06	Saliva_Acir	TSPAN13
UBE2E3	1.11E-10	0.262883	0.071	0.175	1.77E-06	Saliva_Acir	UBE2E3
ERLEC14	1.14E-10	0.287429	0.077	0.183	1.82E-06	Saliva_Acir	ERLEC1
DNAJC34	1.36E-10	0.346488	0.093	0.209	2.18E-06	Saliva_Acir	DNAJC3
PART14	1.61E-10	1.082654	0.125	0.072	2.57E-06	Saliva_Acir	PART1
PRSS233	1.62E-10	0.958504	0.152	0.094	2.59E-06	Saliva_Acir	PRSS23
OCIAD16	1.63E-10	0.361666	0.121	0.254	2.59E-06	Saliva_Acir	OCIAD1
SCOC1	2.46E-10	0.333807	0.071	0.173	3.92E-06	Saliva_Acir	SCOC
PGRMC13	2.68E-10	0.291532	0.069	0.168	4.28E-06	Saliva_Acir	PGRMC1
CD461	2.92E-10	0.344615	0.159	0.314	4.65E-06	Saliva_Acir	CD46
BEX43	3.38E-10	0.298437	0.071	0.171	5.39E-06	Saliva_Acir	BEX4
OXR11	3.61E-10	0.395931	0.088	0.198	5.76E-06	Saliva_Acir	OXR1
SMIM191	3.93E-10	0.384608	0.089	0.2	6.26E-06	Saliva_Acir	SMIM19
NDUFAB18	4.73E-10	0.34956	0.12	0.248	7.54E-06	Saliva_Acir	NDUFAB1
RNF136	4.77E-10	0.290374	0.07	0.168	7.6E-06	Saliva_Acir	RNF13
MRPL209	4.98E-10	0.343646	0.135	0.272	7.95E-06	Saliva_Acir	MRPL20
PTS2	6.94E-10	0.270199	0.052	0.139	1.11E-05	Saliva_Acir	PTS
SRSF113	8.31E-10	0.311293	0.155	0.304	1.32E-05	Saliva_Acir	SRSF11
PPA2	9.56E-10	0.274113	0.045	0.127	1.53E-05	Saliva_Acir	PPA2
OS94	9.81E-10	0.30483	0.109	0.227	1.56E-05	Saliva_Acir	OS9
CLCN35	1.08E-09	0.251517	0.048	0.131	1.71E-05	Saliva_Acir	CLCN3
PIGP1	1.42E-09	0.255514	0.058	0.145	2.26E-05	Saliva_Acir	PIGP
SCARB28	1.42E-09	0.291926	0.066	0.158	2.27E-05	Saliva_Acir	SCARB2
TMED47	1.71E-09	0.279043	0.077	0.175	2.72E-05	Saliva_Acir	TMED4
GALNT121	1.72E-09	0.704782	0.07	0.032	2.74E-05	Saliva_Acir	GALNT12
GOLGB17	1.87E-09	0.321784	0.093	0.201	2.98E-05	Saliva_Acir	GOLGB1
ADI18	1.96E-09	0.385118	0.107	0.223	3.12E-05	Saliva_Acir	ADI1
SAR1B2	2.11E-09	0.403764	0.082	0.184	3.37E-05	Saliva_Acir	SAR1B
TECR5	2.73E-09	0.345653	0.174	0.332	4.36E-05	Saliva_Acir	TECR
URI11	2.9E-09	0.251057	0.055	0.139	4.63E-05	Saliva_Acir	URI1
ANXA72	3.43E-09	0.32696	0.092	0.197	5.47E-05	Saliva_Acir	ANXA7
ZRANB25	3.54E-09	0.288574	0.087	0.188	5.64E-05	Saliva_Acir	ZRANB2
COX7B4	4.38E-09	0.329641	0.22	0.411	6.99E-05	Saliva_Acir	COX7B
ACAT11	4.74E-09	0.276201	0.054	0.135	7.55E-05	Saliva_Acir	ACAT1
MPC15	4.76E-09	0.403915	0.102	0.212	7.6E-05	Saliva_Acir	MPC1
LRRC69	6.04E-09	0.568431	0.048	0.019	9.63E-05	Saliva_Acir	LRRC69
PSMA35	6.35E-09	0.368842	0.092	0.195	0.000101	Saliva_Acir	PSMA3

SURF45	9E-09	0.34935	0.082	0.179	0.000144	Saliva_Acir	SURF4
CEP415	9.05E-09	1.085977	0.152	0.102	0.000144	Saliva_Acir	CEP41
PURA6	9.98E-09	0.270621	0.049	0.126	0.000159	Saliva_Acir	PURA
NR1D21	1.25E-08	0.288906	0.062	0.145	0.0002	Saliva_Acir	NR1D2
RAB4A5	1.73E-08	0.451167	0.103	0.21	0.000276	Saliva_Acir	RAB4A
ATP5G39	1.85E-08	0.308476	0.198	0.364	0.000295	Saliva_Acir	ATP5G3
CIB12	2.01E-08	0.303822	0.194	0.353	0.00032	Saliva_Acir	CIB1
ANAPC13	2.17E-08	0.356871	0.058	0.137	0.000347	Saliva_Acir	ANAPC13
HIF1A1	2.18E-08	0.370183	0.077	0.167	0.000347	Saliva_Acir	HIF1A
FRMD4B6	2.23E-08	0.295561	0.052	0.128	0.000356	Saliva_Acir	FRMD4B
MRPL522	2.31E-08	0.427405	0.114	0.226	0.000369	Saliva_Acir	MRPL52
COPB12	2.75E-08	0.308125	0.06	0.141	0.000439	Saliva_Acir	COPB1
FNDC3B3	2.8E-08	0.308412	0.067	0.151	0.000447	Saliva_Acir	FNDC3B
SYPL14	2.88E-08	0.457962	0.139	0.265	0.000459	Saliva_Acir	SYPL1
SEC23B2	3.38E-08	0.256514	0.043	0.112	0.000539	Saliva_Acir	SEC23B
CREG13	3.64E-08	0.325218	0.065	0.146	0.000581	Saliva_Acir	CREG1
NKTR9	4.01E-08	0.254864	0.085	0.177	0.000639	Saliva_Acir	NKTR
LSM53	5.15E-08	0.369021	0.102	0.203	0.000822	Saliva_Acir	LSM5
BET11	5.21E-08	0.269138	0.043	0.111	0.00083	Saliva_Acir	BET1
MYO1C4	5.31E-08	0.284202	0.066	0.147	0.000846	Saliva_Acir	MYO1C
C1orf1226	5.57E-08	0.323894	0.091	0.185	0.000889	Saliva_Acir	C1orf122
RBM472	5.66E-08	0.377169	0.113	0.219	0.000902	Saliva_Acir	RBM47
FDFT11	6.74E-08	0.496972	0.109	0.213	0.001075	Saliva_Acir	FDFT1
SECISBP2L:	9.03E-08	0.398557	0.092	0.186	0.00144	Saliva_Acir	SECISBP2L
CALU3	9.45E-08	0.366555	0.087	0.177	0.001507	Saliva_Acir	CALU
PDXDC11	9.68E-08	0.337371	0.073	0.156	0.001543	Saliva_Acir	PDXDC1
OSTC4	1.13E-07	0.473014	0.151	0.28	0.001802	Saliva_Acir	OSTC
PJA28	1.34E-07	0.381359	0.071	0.152	0.002138	Saliva_Acir	PJA2
ALDH25	1.36E-07	0.299929	0.054	0.125	0.002176	Saliva_Acir	ALDH2
CLTC3	1.72E-07	0.464947	0.093	0.186	0.002746	Saliva_Acir	CLTC
ETNK11	2E-07	0.368758	0.066	0.143	0.003192	Saliva_Acir	ETNK1
SELENBP18	2.01E-07	1.025747	0.118	0.075	0.0032	Saliva_Acir	SELENBP1
SLITRK65	2.33E-07	0.927293	0.099	0.059	0.003717	Saliva_Acir	SLITRK6
NDUFV13	2.39E-07	0.385607	0.087	0.174	0.003805	Saliva_Acir	NDUFV1
BCKDK1	2.39E-07	0.282712	0.041	0.104	0.003809	Saliva_Acir	BCKDK
ISOC24	2.57E-07	0.295065	0.06	0.133	0.004095	Saliva_Acir	ISOC2
VPS367	2.69E-07	0.280064	0.048	0.114	0.004289	Saliva_Acir	VPS36
MKNK23	2.81E-07	0.252341	0.051	0.118	0.004479	Saliva_Acir	MKNK2
AZI2	3.26E-07	0.299952	0.043	0.105	0.005204	Saliva_Acir	AZI2
CREB32	3.47E-07	0.332771	0.06	0.132	0.005534	Saliva_Acir	CREB3
SERINC22	3.58E-07	0.407431	0.114	0.214	0.005705	Saliva_Acir	SERINC2
ALG55	3.64E-07	0.351033	0.076	0.155	0.005801	Saliva_Acir	ALG5
ARFGAP34	3.84E-07	0.379093	0.078	0.159	0.006129	Saliva_Acir	ARFGAP3
RGL2	4.05E-07	0.250729	0.047	0.111	0.006456	Saliva_Acir	RGL2
FUCA15	4.05E-07	0.385563	0.066	0.14	0.00646	Saliva_Acir	FUCA1
BACE25	4.38E-07	0.411526	0.081	0.163	0.00698	Saliva_Acir	BACE2
MAP3K24	4.43E-07	0.326338	0.056	0.125	0.007063	Saliva_Acir	MAP3K2

MAPK133	4.52E-07	0.320088	0.047	0.111	0.007215	Saliva_Acir	MAPK13
KCNK11	4.9E-07	0.282576	0.056	0.125	0.007822	Saliva_Acir	KCNK1
RBM53	5.71E-07	0.303415	0.06	0.131	0.009103	Saliva_Acir	RBM5
SLC35A31	6.27E-07	0.369006	0.065	0.137	0.010007	Saliva_Acir	SLC35A3
TMEM141:	6.27E-07	0.396156	0.084	0.166	0.010008	Saliva_Acir	TMEM141
UAP13	7.19E-07	0.389279	0.096	0.184	0.011464	Saliva_Acir	UAP1
LARS4	7.58E-07	0.284498	0.051	0.115	0.012094	Saliva_Acir	LARS
TIMP15	7.79E-07	0.471297	0.397	0.382	0.012426	Saliva_Acir	TIMP1
SPPL2A5	9.09E-07	0.361821	0.062	0.131	0.014493	Saliva_Acir	SPPL2A
ENTPD81	9.16E-07	0.449195	0.033	0.013	0.014603	Saliva_Acir	ENTPD8
ATP5A13	1E-06	0.340878	0.18	0.315	0.015977	Saliva_Acir	ATP5A1
NUCB26	1.1E-06	0.919987	0.291	0.27	0.01755	Saliva_Acir	NUCB2
CAT2	1.12E-06	0.266215	0.052	0.116	0.01787	Saliva_Acir	CAT
TMEM331	1.13E-06	0.329055	0.049	0.112	0.018056	Saliva_Acir	TMEM33
SNX53	1.18E-06	0.258971	0.049	0.112	0.01879	Saliva_Acir	SNX5
WDR131	1.24E-06	0.294963	0.049	0.112	0.019709	Saliva_Acir	WDR13
PEX2	1.33E-06	0.261245	0.043	0.101	0.021197	Saliva_Acir	PEX2
VEGFB9	1.5E-06	0.250788	0.043	0.1	0.023932	Saliva_Acir	VEGFB
SELM8	1.51E-06	0.714997	0.354	0.348	0.024074	Saliva_Acir	SELM
CADM14	1.54E-06	0.282537	0.045	0.105	0.024534	Saliva_Acir	CADM1
EIF4EBP14	1.57E-06	0.374326	0.077	0.152	0.025079	Saliva_Acir	EIF4EBP1
NPTN3	1.84E-06	0.384199	0.062	0.129	0.029271	Saliva_Acir	NPTN
CTBS5	2.01E-06	0.317441	0.045	0.104	0.032111	Saliva_Acir	CTBS
LAMP27	2.02E-06	0.542929	0.132	0.237	0.032286	Saliva_Acir	LAMP2
TMEM87A	2.05E-06	0.478395	0.091	0.173	0.032754	Saliva_Acir	TMEM87A
TM9SF25	2.21E-06	0.521186	0.106	0.196	0.035264	Saliva_Acir	TM9SF2
SNRNP70	2.35E-06	0.482589	0.099	0.184	0.037479	Saliva_Acir	SNRNP70
PPP3CA2	2.49E-06	0.389164	0.081	0.156	0.039792	Saliva_Acir	PPP3CA
JKAMP	2.5E-06	0.387275	0.048	0.107	0.039813	Saliva_Acir	JKAMP
ZNF664	2.61E-06	0.27992	0.045	0.103	0.041629	Saliva_Acir	ZNF664
SEC132	3.24E-06	0.404077	0.067	0.135	0.051676	Saliva_Acir	SEC13
ANKRD93	4.03E-06	0.311806	0.041	0.095	0.064248	Saliva_Acir	ANKRD9
WIPI14	4.48E-06	0.316449	0.044	0.099	0.071434	Saliva_Acir	WIPI1
ZC3H11A2	4.82E-06	0.497978	0.089	0.167	0.076802	Saliva_Acir	ZC3H11A
ERN21	5.09E-06	0.442738	0.038	0.017	0.081149	Saliva_Acir	ERN2
LTBP33	5.28E-06	0.372384	0.08	0.152	0.084204	Saliva_Acir	LTBP3
ANAPC56	5.3E-06	0.46183	0.087	0.162	0.084504	Saliva_Acir	ANAPC5
MAGT12	5.44E-06	0.408069	0.066	0.131	0.086763	Saliva_Acir	MAGT1
LINC00657	5.63E-06	0.5126	0.114	0.204	0.089824	Saliva_Acir	LINC00657
ANXA56	6.22E-06	0.916513	0.317	0.318	0.099228	Saliva_Acir	ANXA5
PDCL	6.3E-06	0.283784	0.036	0.086	0.100467	Saliva_Acir	PDCL
RGCC6	6.46E-06	0.73026	0.249	0.214	0.102958	Saliva_Acir	RGCC
PAPSS15	6.8E-06	0.351104	0.052	0.11	0.10845	Saliva_Acir	PAPSS1
KCNK65	6.83E-06	0.315709	0.047	0.102	0.108996	Saliva_Acir	KCNK6
ATOX15	6.98E-06	0.482705	0.159	0.275	0.111282	Saliva_Acir	ATOX1
ZFR2	7.02E-06	0.370064	0.052	0.11	0.11193	Saliva_Acir	ZFR
GNPAT1	7.09E-06	0.268214	0.036	0.085	0.113078	Saliva_Acir	GNPAT

MACF17	7.54E-06	0.417141	0.092	0.169	0.120295	Saliva_Acir	MACF1
C8orf5910	1.13E-05	0.551552	0.136	0.236	0.180876	Saliva_Acir	C8orf59
IVNS1ABP5	1.36E-05	0.459274	0.081	0.15	0.217296	Saliva_Acir	IVNS1ABP
ZNF770	1.41E-05	0.250452	0.032	0.077	0.225039	Saliva_Acir	ZNF770
MBNL25	1.55E-05	0.366016	0.06	0.12	0.247606	Saliva_Acir	MBNL2
RANBP23	1.67E-05	0.374523	0.059	0.117	0.266262	Saliva_Acir	RANBP2
FOXP13	1.7E-05	0.474973	0.128	0.22	0.271024	Saliva_Acir	FOXP1
OGFRL17	1.74E-05	1.058822	0.15	0.115	0.277171	Saliva_Acir	OGFRL1
SRPRB3	1.75E-05	0.355916	0.056	0.113	0.2787	Saliva_Acir	SRPRB
KCNN43	1.8E-05	0.943508	0.117	0.082	0.28739	Saliva_Acir	KCNN4
SVIL4	1.92E-05	0.343054	0.062	0.121	0.30621	Saliva_Acir	SVIL
FAM210B8	1.94E-05	0.28204	0.044	0.094	0.309334	Saliva_Acir	FAM210B
COPA5	2.07E-05	0.478478	0.08	0.147	0.330065	Saliva_Acir	COPA
HSBP11	2.08E-05	0.391811	0.258	0.434	0.33143	Saliva_Acir	HSBP1
MGST16	2.18E-05	1.11049	0.22	0.194	0.347667	Saliva_Acir	MGST1
FLNB4	2.21E-05	0.26134	0.034	0.08	0.352114	Saliva_Acir	FLNB
CMPK11	2.28E-05	0.515332	0.18	0.302	0.363478	Saliva_Acir	CMPK1
GALNT31	2.35E-05	0.378354	0.063	0.122	0.374362	Saliva_Acir	GALNT3
CKAP45	2.4E-05	0.436404	0.093	0.166	0.383465	Saliva_Acir	CKAP4
CCNC2	2.55E-05	0.618738	0.118	0.205	0.407129	Saliva_Acir	CCNC
TNRC6A2	2.78E-05	0.28436	0.047	0.097	0.443855	Saliva_Acir	TNRC6A
IER3IP13	2.81E-05	0.487943	0.078	0.144	0.448835	Saliva_Acir	IER3IP1
CDC42EP1:	2.83E-05	0.281703	0.045	0.095	0.450629	Saliva_Acir	CDC42EP1
USO12	2.94E-05	0.448446	0.069	0.13	0.469462	Saliva_Acir	USO1
MPZL16	2.98E-05	0.485238	0.077	0.142	0.475734	Saliva_Acir	MPZL1
ATP1A17	3E-05	0.435236	0.154	0.257	0.478327	Saliva_Acir	ATP1A1
RPS27L3	3E-05	0.673065	0.398	0.446	0.478849	Saliva_Acir	RPS27L
SMAD55	3.06E-05	0.312587	0.045	0.095	0.488786	Saliva_Acir	SMAD5
SEC635	3.15E-05	0.521653	0.098	0.172	0.501985	Saliva_Acir	SEC63
UBA52	3.29E-05	0.340677	0.047	0.097	0.524176	Saliva_Acir	UBA5
COPG15	3.9E-05	0.352675	0.047	0.096	0.622749	Saliva_Acir	COPG1
RPN24	3.93E-05	0.444857	0.148	0.247	0.626237	Saliva_Acir	RPN2
TMEM167/	3.93E-05	0.649753	0.14	0.238	0.62682	Saliva_Acir	TMEM167A
PHPT17	4.16E-05	0.430743	0.198	0.326	0.662716	Saliva_Acir	PHPT1
TXNDC155	4.31E-05	0.394745	0.066	0.124	0.686715	Saliva_Acir	TXNDC15
NBN1	4.34E-05	0.254582	0.032	0.073	0.691413	Saliva_Acir	NBN
CCDC142	4.52E-05	0.253456	0.036	0.079	0.720486	Saliva_Acir	CCDC14
PRPSAP14	4.55E-05	0.434472	0.063	0.12	0.726142	Saliva_Acir	PRPSAP1
TMED105	4.61E-05	1.015482	0.308	0.322	0.735254	Saliva_Acir	TMED10
ARHGAP12	4.75E-05	0.262361	0.038	0.083	0.75808	Saliva_Acir	ARHGAP12
FNDC3A6	5.01E-05	0.420557	0.062	0.117	0.799612	Saliva_Acir	FNDC3A
AKT14	5.07E-05	0.308208	0.043	0.089	0.809088	Saliva_Acir	AKT1
DHRS77	5.7E-05	0.413093	0.125	0.21	0.909744	Saliva_Acir	DHRS7
SEC61A15	5.87E-05	0.479363	0.087	0.153	0.93591	Saliva_Acir	SEC61A1
DAP5	6.06E-05	0.526367	0.081	0.145	0.966345	Saliva_Acir	DAP
MBTPS15	6.18E-05	0.30954	0.041	0.086	0.985066	Saliva_Acir	MBTPS1
FAM120A5	6.26E-05	0.51113	0.084	0.149	0.998259	Saliva_Acir	FAM120A

DAD17	6.27E-05	0.498313	0.242	0.401	1	Saliva_Acir	DAD1
CTNND14	6.58E-05	0.365659	0.051	0.1	1	Saliva_Acir	CTNND1
TMEM123!	7.18E-05	1.055472	0.28	0.282	1	Saliva_Acir	TMEM123
RBM62	7.22E-05	0.335887	0.059	0.112	1	Saliva_Acir	RBM6
NUMA14	7.54E-05	0.346892	0.066	0.122	1	Saliva_Acir	NUMA1
ZKSCAN15	8.42E-05	0.400075	0.063	0.117	1	Saliva_Acir	ZKSCAN1
SLC39A64	8.62E-05	0.363163	0.051	0.099	1	Saliva_Acir	SLC39A6
GPCPD1	9.3E-05	0.465886	0.103	0.175	1	Saliva_Acir	GPCPD1
ACSL1	0.000101	0.283313	0.041	0.085	1	Saliva_Acir	ACSL1
MTA13	0.000103	0.288427	0.04	0.083	1	Saliva_Acir	MTA1
MGAT4A2	0.000104	0.287541	0.055	0.104	1	Saliva_Acir	MGAT4A
ITGB45	0.000121	0.299765	0.044	0.088	1	Saliva_Acir	ITGB4
NUDT9	0.000122	0.265689	0.032	0.07	1	Saliva_Acir	NUDT9
ACSL34	0.000125	0.588758	0.093	0.16	1	Saliva_Acir	ACSL3
KIAA15223	0.00013	0.280035	0.032	0.07	1	Saliva_Acir	KIAA1522
GALE2	0.000132	0.544421	0.087	0.15	1	Saliva_Acir	GALE
DUS1L5	0.000136	0.35777	0.051	0.098	1	Saliva_Acir	DUS1L
TMED71	0.000146	0.532934	0.078	0.137	1	Saliva_Acir	TMED7
SLC30A72	0.000158	0.274589	0.041	0.083	1	Saliva_Acir	SLC30A7
PANK3	0.000161	0.288337	0.029	0.065	1	Saliva_Acir	PANK3
PHKB7	0.00017	0.465903	0.058	0.107	1	Saliva_Acir	PHKB
TMC44	0.000173	0.335917	0.04	0.081	1	Saliva_Acir	TMC4
TMEM63A	0.000184	0.934735	0.11	0.08	1	Saliva_Acir	TMEM63A
PCYOX16	0.000188	0.476496	0.076	0.132	1	Saliva_Acir	PCYOX1
PRRC12	0.000203	0.313888	0.037	0.077	1	Saliva_Acir	PRRC1
GOLGA47	0.000211	0.551975	0.146	0.237	1	Saliva_Acir	GOLGA4
JTB5	0.000216	0.457223	0.231	0.372	1	Saliva_Acir	JTB
CHD32	0.000224	0.319603	0.045	0.088	1	Saliva_Acir	CHD3
LMAN18	0.000238	0.482106	0.12	0.195	1	Saliva_Acir	LMAN1
INADL4	0.000285	0.385665	0.045	0.087	1	Saliva_Acir	INADL
SND12	0.000286	0.368791	0.056	0.103	1	Saliva_Acir	SND1
FOXC15	0.000291	0.414041	0.063	0.113	1	Saliva_Acir	FOXC1
SLC30A52	0.000295	0.299007	0.034	0.071	1	Saliva_Acir	SLC30A5
GBAS4	0.000306	0.468915	0.073	0.126	1	Saliva_Acir	GBAS
ALG141	0.000311	0.276086	0.03	0.065	1	Saliva_Acir	ALG14
C8orf821	0.000312	0.261871	0.034	0.071	1	Saliva_Acir	C8orf82
OPA11	0.000328	0.259319	0.03	0.065	1	Saliva_Acir	OPA1
CXCL53	0.000334	0.565179	0.051	0.03	1	Saliva_Acir	CXCL5
ZNF6525	0.000358	0.399581	0.052	0.096	1	Saliva_Acir	ZNF652
ATP2A34	0.000359	0.705473	0.081	0.055	1	Saliva_Acir	ATP2A3
PRR45	0.000362	0.436247	0.144	0.111	1	Saliva_Acir	PRR4
SMCO44	0.000362	0.433446	0.069	0.119	1	Saliva_Acir	SMCO4
CD1642	0.000372	0.594353	0.229	0.372	1	Saliva_Acir	CD164
PRDX33	0.000386	0.60369	0.095	0.157	1	Saliva_Acir	PRDX3
ATP5J8	0.000392	0.460198	0.268	0.438	1	Saliva_Acir	ATP5J
CASC44	0.000397	0.437202	0.071	0.123	1	Saliva_Acir	CASC4
SPCS14	0.000403	0.67738	0.393	0.463	1	Saliva_Acir	SPCS1

EPB41L5	0.000416	0.273419	0.026	0.058	1	Saliva_Acir	EPB41L5
TFCP2L16	0.000468	0.68798	0.073	0.048	1	Saliva_Acir	TFCP2L1
GNE1	0.000479	0.281866	0.037	0.074	1	Saliva_Acir	GNE
CLPTM1L6	0.000501	0.34838	0.051	0.093	1	Saliva_Acir	CLPTM1L
CCPG17	0.000504	0.496911	0.104	0.169	1	Saliva_Acir	CCPG1
SLC35E12	0.000521	0.325549	0.038	0.075	1	Saliva_Acir	SLC35E1
PAM5	0.000526	0.400863	0.087	0.143	1	Saliva_Acir	PAM
ZNF280D4	0.000543	0.357656	0.045	0.085	1	Saliva_Acir	ZNF280D
PGM34	0.000548	0.398927	0.051	0.093	1	Saliva_Acir	PGM3
CTSZ5	0.00055	0.263418	0.124	0.193	1	Saliva_Acir	CTSZ
GGCT1	0.000557	0.502948	0.077	0.13	1	Saliva_Acir	GGCT
TRIM21	0.000599	0.425091	0.063	0.11	1	Saliva_Acir	TRIM2
KIAA1109!	0.000608	0.34482	0.047	0.086	1	Saliva_Acir	KIAA1109
ZC3H141	0.000627	0.310723	0.067	0.114	1	Saliva_Acir	ZC3H14
IARS24	0.000638	0.34834	0.051	0.092	1	Saliva_Acir	IARS2
PBX16	0.000639	0.438837	0.067	0.115	1	Saliva_Acir	PBX1
CHKA1	0.000729	0.448077	0.052	0.094	1	Saliva_Acir	CHKA
SATB11	0.000736	0.257086	0.037	0.072	1	Saliva_Acir	SATB1
FGGY5	0.000748	0.910453	0.111	0.085	1	Saliva_Acir	FGGY
GGA21	0.000771	0.317556	0.041	0.078	1	Saliva_Acir	GGA2
CTDSPL6	0.000781	0.38992	0.047	0.085	1	Saliva_Acir	CTDSPL
ITPR27	0.000806	0.355645	0.044	0.081	1	Saliva_Acir	ITPR2
RRAGD4	0.000824	0.456725	0.052	0.093	1	Saliva_Acir	RRAGD
TMEM30B	0.000829	0.251661	0.032	0.064	1	Saliva_Acir	TMEM30B
PPP3CB	0.00086	0.305247	0.038	0.074	1	Saliva_Acir	PPP3CB
TRPM42	0.000871	0.256199	0.03	0.062	1	Saliva_Acir	TRPM4
ECHDC14	0.000892	0.512345	0.07	0.118	1	Saliva_Acir	ECHDC1
MFSD62	0.000907	0.347108	0.043	0.079	1	Saliva_Acir	MFSD6
CCS2	0.000915	0.413083	0.066	0.112	1	Saliva_Acir	CCS
SREBF12	0.000937	0.37119	0.052	0.092	1	Saliva_Acir	SREBF1
STX162	0.000942	0.343955	0.047	0.085	1	Saliva_Acir	STX16
PTCD32	0.00106	0.293828	0.036	0.069	1	Saliva_Acir	PTCD3
MT.ATP81(	0.00107	0.967275	0.159	0.136	1	Saliva_Acir	MT.ATP8
CLDN36	0.001074	0.821808	0.125	0.099	1	Saliva_Acir	CLDN3
RARS1	0.001098	0.367965	0.048	0.086	1	Saliva_Acir	RARS
SRSF61	0.001245	0.567663	0.091	0.146	1	Saliva_Acir	SRSF6
FAM114A1	0.001335	0.48792	0.076	0.124	1	Saliva_Acir	FAM114A1
SLC39A84	0.001405	0.910277	0.118	0.094	1	Saliva_Acir	SLC39A8
CSRP13	0.001427	0.497652	0.099	0.156	1	Saliva_Acir	CSRP1
IMPAD13	0.001431	0.418094	0.054	0.093	1	Saliva_Acir	IMPAD1
WWC13	0.001436	0.418051	0.051	0.089	1	Saliva_Acir	WWC1
KIAA0319L	0.001517	0.305427	0.037	0.07	1	Saliva_Acir	KIAA0319L
GLCE1	0.001519	0.26473	0.025	0.052	1	Saliva_Acir	GLCE
TMEM214!	0.001539	0.296595	0.04	0.073	1	Saliva_Acir	TMEM214
ERBB24	0.001599	0.283817	0.03	0.06	1	Saliva_Acir	ERBB2
NCALD4	0.001607	0.796556	0.093	0.069	1	Saliva_Acir	NCALD
TTC375	0.001875	0.362584	0.049	0.086	1	Saliva_Acir	TTC37

TUSC31	0.001915	0.272208	0.03	0.059	1	Saliva_Acir	TUSC3
SYNJ21	0.001971	0.27897	0.025	0.051	1	Saliva_Acir	SYNJ2
CDK5RAP3	0.002128	0.541147	0.081	0.129	1	Saliva_Acir	CDK5RAP3
MAGED26	0.002165	0.495116	0.1	0.156	1	Saliva_Acir	MAGED2
TSC21	0.002202	0.282971	0.037	0.068	1	Saliva_Acir	TSC2
SSFA24	0.002227	0.604972	0.109	0.169	1	Saliva_Acir	SSFA2
ACLY1	0.002295	0.385578	0.051	0.087	1	Saliva_Acir	ACLY
CPD4	0.002542	1.048724	0.141	0.121	1	Saliva_Acir	CPD
AGFG24	0.002651	0.542075	0.088	0.138	1	Saliva_Acir	AGFG2
NSUN5	0.002696	0.291288	0.04	0.071	1	Saliva_Acir	NSUN5
PEBP19	0.002717	0.700296	0.38	0.457	1	Saliva_Acir	PEBP1
ESRP11	0.002811	0.410913	0.047	0.081	1	Saliva_Acir	ESRP1
APIP5	0.002874	0.474067	0.059	0.097	1	Saliva_Acir	APIP
ARFIP23	0.002975	0.488701	0.066	0.107	1	Saliva_Acir	ARFIP2
ITGB52	0.002988	0.37361	0.045	0.078	1	Saliva_Acir	ITGB5
WDR77	0.003017	0.26039	0.03	0.058	1	Saliva_Acir	WDR77
RHOBTB36	0.003335	0.40526	0.076	0.119	1	Saliva_Acir	RHOBTB3
PITPNA4	0.003411	0.49692	0.063	0.102	1	Saliva_Acir	PITPNA
ACTR3B4	0.003617	0.357272	0.038	0.068	1	Saliva_Acir	ACTR3B
AGA1	0.003885	0.431876	0.049	0.083	1	Saliva_Acir	AGA
IMPACT1	0.003902	0.388568	0.043	0.074	1	Saliva_Acir	IMPACT
SH3GLB21	0.003906	0.504721	0.069	0.109	1	Saliva_Acir	SH3GLB2
TM7SF24	0.003915	0.494032	0.065	0.104	1	Saliva_Acir	TM7SF2
DCAF8	0.003971	0.300618	0.036	0.064	1	Saliva_Acir	DCAF8
IL17RC2	0.004217	0.29405	0.036	0.064	1	Saliva_Acir	IL17RC
MTRNR2L6	0.004296	0.383498	0.037	0.022	1	Saliva_Acir	MTRNR2L6
TMEM178/	0.004635	0.317277	0.025	0.013	1	Saliva_Acir	TMEM178A
NR2F66	0.004827	0.433591	0.051	0.084	1	Saliva_Acir	NR2F6
CERS43	0.004914	0.314916	0.04	0.069	1	Saliva_Acir	CERS4
COPB26	0.00505	0.698717	0.103	0.156	1	Saliva_Acir	COPB2
ENOSF12	0.005303	0.330014	0.037	0.065	1	Saliva_Acir	ENOSF1
SLC25A47	0.005615	0.505886	0.074	0.115	1	Saliva_Acir	SLC25A4
LIMCH14	0.005718	0.587562	0.084	0.129	1	Saliva_Acir	LIMCH1
ASPH6	0.005723	0.706532	0.128	0.192	1	Saliva_Acir	ASPH
TMEM594	0.005737	0.437231	0.481	0.611	1	Saliva_Acir	TMEM59
FARP14	0.006016	0.493381	0.063	0.1	1	Saliva_Acir	FARP1
SERP15	0.006391	0.371702	0.345	0.549	1	Saliva_Acir	SERP1
ANG5	0.006422	0.478084	0.065	0.101	1	Saliva_Acir	ANG
PPP1R9A4	0.006445	0.337267	0.027	0.051	1	Saliva_Acir	PPP1R9A
FAM3B3	0.006875	0.818955	0.093	0.074	1	Saliva_Acir	FAM3B
LINC00342	0.007125	0.756315	0.073	0.054	1	Saliva_Acir	LINC00342
SHROOM3	0.00753	0.276448	0.027	0.051	1	Saliva_Acir	SHROOM3
AC009501.	0.007538	0.560168	0.049	0.033	1	Saliva_Acir	AC009501.4
AKR1A17	0.007695	0.633927	0.139	0.205	1	Saliva_Acir	AKR1A1
HIPK27	0.007779	0.450066	0.049	0.08	1	Saliva_Acir	HIPK2
TMEM131	0.007929	0.310227	0.037	0.063	1	Saliva_Acir	TMEM131
CCNL2	0.008016	0.460539	0.076	0.115	1	Saliva_Acir	CCNL2

CYB5614	0.008065	0.833491	0.107	0.088	1	Saliva_Acir	CYB561
ITGA68	0.008255	0.971735	0.151	0.136	1	Saliva_Acir	ITGA6
SLC9A3R12	0.008456	0.601179	0.104	0.155	1	Saliva_Acir	SLC9A3R1
LHFPL23	0.008536	0.29763	0.03	0.054	1	Saliva_Acir	LHFPL2
RAPGEF21	0.008618	0.296536	0.033	0.058	1	Saliva_Acir	RAPGEF2
SORL13	0.008644	0.320248	0.044	0.072	1	Saliva_Acir	SORL1
KRTCAP35	0.00866	0.432142	0.051	0.081	1	Saliva_Acir	KRTCAP3
PACS12	0.008692	0.392567	0.047	0.076	1	Saliva_Acir	PACS1
TF2	0.008705	0.359696	0.03	0.018	1	Saliva_Acir	TF
GALNT62	0.008767	0.655405	0.066	0.048	1	Saliva_Acir	GALNT6
TRAK22	0.009465	0.257025	0.025	0.046	1	Saliva_Acir	TRAK2
LPAR62	0.009517	0.472765	0.096	0.142	1	Saliva_Acir	LPAR6
KRT75	0.009545	0.360467	0.283	0.272	1	Saliva_Acir	KRT7
SEC24D2	0.009714	0.447792	0.058	0.09	1	Saliva_Acir	SEC24D
PTPN184	0.009782	0.378002	0.059	0.092	1	Saliva_Acir	PTPN18
CREM	0	2.940851	0.761	0.191	0	Skin_T	CREM
CD691	0	2.78235	0.634	0.063	0	Skin_T	CD69
SRGN3	0	2.675955	0.933	0.214	0	Skin_T	SRGN
DUSP25	0	2.533984	0.68	0.118	0	Skin_T	DUSP2
IL321	0	2.510874	0.862	0.177	0	Skin_T	IL32
SAMSN11	0	2.505519	0.616	0.038	0	Skin_T	SAMSN1
CXCR41	0	2.476164	0.684	0.115	0	Skin_T	CXCR4
STK17B3	0	2.19886	0.624	0.098	0	Skin_T	STK17B
RPL41	0	2.158822	1	0.86	0	Skin_T	RPL41
BIRC31	0	2.155588	0.597	0.148	0	Skin_T	BIRC3
RGS14	0	2.078868	0.382	0.066	0	Skin_T	RGS1
RGCC7	0	2.068056	0.683	0.208	0	Skin_T	RGCC
TMSB4X2	0	2.065969	1	0.869	0	Skin_T	TMSB4X
TRAT11	0	2.063435	0.467	0.019	0	Skin_T	TRAT1
SLC2A31	0	2.01166	0.637	0.123	0	Skin_T	SLC2A3
KLRB11	0	2.00848	0.287	0.035	0	Skin_T	KLRB1
CYTIP2	0	1.982597	0.528	0.078	0	Skin_T	CYTIP
STK43	0	1.98237	0.595	0.107	0	Skin_T	STK4
ALOX5AP2	0	1.9501	0.588	0.084	0	Skin_T	ALOX5AP
CD521	0	1.924518	0.873	0.219	0	Skin_T	CD52
S100A44	0	1.920998	0.965	0.458	0	Skin_T	S100A4
RPS291	0	1.918931	0.999	0.788	0	Skin_T	RPS29
ZFP36L24	0	1.914259	0.819	0.387	0	Skin_T	ZFP36L2
EML4	0	1.912573	0.509	0.115	0	Skin_T	EML4
CDC42SE2	0	1.872702	0.528	0.115	0	Skin_T	CDC42SE2
ZNF331	0	1.84901	0.498	0.099	0	Skin_T	ZNF331
PTPRC2	0	1.842566	0.628	0.101	0	Skin_T	PTPRC
LEPROTL11	0	1.835921	0.58	0.165	0	Skin_T	LEPROTL1
BTG11	0	1.800074	0.923	0.573	0	Skin_T	BTG1
RPL21	0	1.790324	1	0.812	0	Skin_T	RPL21
RPS4Y13	0	1.789179	0.785	0.266	0	Skin_T	RPS4Y1
PTGER4	0	1.757332	0.397	0.046	0	Skin_T	PTGER4

RPL399	0	1.723228	0.998	0.673	0	Skin_T	RPL39
NR4A22	0	1.722619	0.588	0.18	0	Skin_T	NR4A2
CD3D1	0	1.677883	0.703	0.117	0	Skin_T	CD3D
TNFRSF181	0	1.654788	0.367	0.044	0	Skin_T	TNFRSF18
LGALS15	0	1.606523	0.95	0.403	0	Skin_T	LGALS1
RPS271	0	1.585654	1	0.893	0	Skin_T	RPS27
RHOH	0	1.540775	0.407	0.05	0	Skin_T	RHOH
CD442	0	1.491484	0.816	0.343	0	Skin_T	CD44
NBEAL1	0	1.44608	0.79	0.314	0	Skin_T	NBEAL1
HCST2	0	1.445951	0.675	0.167	0	Skin_T	HCST
RPS26	0	1.439376	0.992	0.739	0	Skin_T	RPS26
RP11.138A	0	1.432802	0.311	0.043	0	Skin_T	RP11.138A9.2
RPL341	0	1.370652	1	0.918	0	Skin_T	RPL34
GPR1832	0	1.365471	0.404	0.072	0	Skin_T	GPR183
CD40LG1	0	1.333743	0.263	0.013	0	Skin_T	CD40LG
IL7R1	0	1.332429	0.396	0.057	0	Skin_T	IL7R
CLEC2D1	0	1.300386	0.285	0.043	0	Skin_T	CLEC2D
CD961	0	1.229016	0.274	0.018	0	Skin_T	CD96
SYTL31	0	1.217674	0.24	0.024	0	Skin_T	SYTL3
RUNX32	0	1.182777	0.271	0.03	0	Skin_T	RUNX3
TNFRSF1B3	0	1.172617	0.292	0.045	0	Skin_T	TNFRSF1B
RPS212	0	1.163045	0.983	0.669	0	Skin_T	RPS21
CST71	0	1.162877	0.355	0.059	0	Skin_T	CST7
SPOCK21	0	1.142656	0.295	0.04	0	Skin_T	SPOCK2
B2M3	0	1.141982	0.993	0.928	0	Skin_T	B2M
CD21	0	1.140313	0.36	0.062	0	Skin_T	CD2
RP11.347P	0	1.113186	0.177	0.016	0	Skin_T	RP11.347P5.1
RPS27A4	0	1.107594	0.999	0.848	0	Skin_T	RPS27A
BCL11B3	0	1.105406	0.245	0.024	0	Skin_T	BCL11B
CD3G1	0	1.076365	0.3	0.039	0	Skin_T	CD3G
RPS282	0	1.054405	0.999	0.88	0	Skin_T	RPS28
RPL23A	0	1.037788	0.999	0.801	0	Skin_T	RPL23A
RPL36	0	1.010333	0.998	0.839	0	Skin_T	RPL36
GPR652	0	0.966681	0.195	0.022	0	Skin_T	GPR65
RPL281	0	0.958108	0.999	0.908	0	Skin_T	RPL28
PTPN71	0	0.945148	0.221	0.028	0	Skin_T	PTPN7
PTPN221	0	0.926251	0.178	0.013	0	Skin_T	PTPN22
CD281	0	0.878854	0.174	0.008	0	Skin_T	CD28
TIGIT1	0	0.86156	0.142	0.01	0	Skin_T	TIGIT
ITK1	0	0.841698	0.172	0.012	0	Skin_T	ITK
RASGRP11	0	0.827437	0.155	0.015	0	Skin_T	RASGRP1
SH2D1A1	0	0.809418	0.152	0.013	0	Skin_T	SH2D1A
FXYD7	0	0.75211	0.142	0.009	0	Skin_T	FXYD7
ICOS1	0	0.743708	0.125	0.004	0	Skin_T	ICOS
P2RY101	0	0.636021	0.132	0.011	0	Skin_T	P2RY10
FFAR3	0	0.30325	0.041	0.001	0	Skin_T	FFAR3
FYB2	7.7E-305	1.149544	0.351	0.069	1.2E-300	Skin_T	FYB

MPZL3	6.5E-304	1.132948	0.24	0.037	1E-299	Skin_T	MPZL3
TAGAP3	7.5E-298	0.911656	0.207	0.027	1.2E-293	Skin_T	TAGAP
TNFRSF41	2.2E-297	1.058278	0.202	0.026	3.5E-293	Skin_T	TNFRSF4
FXYD5	1.9E-296	1.179459	0.76	0.293	3E-292	Skin_T	FXYD5
THEMIS1	2.3E-294	0.531665	0.1	0.007	3.6E-290	Skin_T	THEMIS
CNOT6L1	6.5E-294	1.528167	0.387	0.096	1E-289	Skin_T	CNOT6L
RPL311	3E-287	1.026873	0.998	0.765	4.9E-283	Skin_T	RPL31
RPS20	4E-285	1.004669	0.992	0.793	6.3E-281	Skin_T	RPS20
CD8A1	4.2E-282	0.819743	0.191	0.024	6.7E-278	Skin_T	CD8A
CCR61	1.9E-280	0.661802	0.117	0.009	3E-276	Skin_T	CCR6
EZR2	2.3E-279	1.769663	0.639	0.278	3.6E-275	Skin_T	EZR
RPLP2	1.5E-277	0.934122	0.998	0.842	2.4E-273	Skin_T	RPLP2
LAPTM52	1.7E-272	1.072021	0.567	0.168	2.8E-268	Skin_T	LAPTM5
ZC3HAV1	3.3E-272	1.482095	0.374	0.094	5.2E-268	Skin_T	ZC3HAV1
RPS25	2E-271	0.912189	0.993	0.822	3.1E-267	Skin_T	RPS25
RPS18	2.7E-265	0.854727	1	0.915	4.3E-261	Skin_T	RPS18
IL2RA	1.2E-263	0.510469	0.077	0.004	2E-259	Skin_T	IL2RA
LMNA7	2.3E-263	1.409294	0.758	0.361	3.7E-259	Skin_T	LMNA
PTMA1	1.1E-262	1.003067	0.997	0.817	1.8E-258	Skin_T	PTMA
FNBP13	6.1E-262	1.361021	0.43	0.122	9.8E-258	Skin_T	FNBP1
CTSW1	2.7E-260	1.331075	0.327	0.068	4.3E-256	Skin_T	CTSW
PFDN5	5.1E-260	0.981839	0.964	0.724	8.2E-256	Skin_T	PFDN5
RPL27A	1.9E-257	0.917299	0.996	0.834	3.1E-253	Skin_T	RPL27A
CD3E1	9.1E-256	0.697933	0.449	0.107	1.5E-251	Skin_T	CD3E
ANKRD281	5.2E-252	1.55338	0.388	0.106	8.3E-248	Skin_T	ANKRD28
EIF4A1	3E-251	1.20154	0.774	0.348	4.8E-247	Skin_T	EIF4A1
RPL38	1.9E-249	0.985189	0.972	0.76	3.1E-245	Skin_T	RPL38
CCL51	1.4E-248	1.369713	0.291	0.057	2.2E-244	Skin_T	CCL5
RPS15A2	2.5E-247	0.853681	0.998	0.848	4E-243	Skin_T	RPS15A
TNF2	1.5E-246	1.025163	0.149	0.017	2.4E-242	Skin_T	TNF
ARF6	4.1E-246	1.464689	0.724	0.372	6.6E-242	Skin_T	ARF6
ID2	3.7E-245	1.650427	0.613	0.25	5.9E-241	Skin_T	ID2
RPL32	8.9E-244	0.835432	1	0.87	1.4E-239	Skin_T	RPL32
RPL35A	1.1E-243	0.82557	0.997	0.862	1.7E-239	Skin_T	RPL35A
EEF1A19	1.2E-238	0.854813	0.998	0.914	2E-234	Skin_T	EEF1A1
RPLP14	1.4E-233	0.813984	1	0.914	2.2E-229	Skin_T	RPLP1
RPL101	5.3E-233	0.791527	1	0.876	8.4E-229	Skin_T	RPL10
RPS15	1.6E-232	0.794187	0.999	0.879	2.6E-228	Skin_T	RPS15
SH3BGRL3:	5.8E-232	1.080116	0.911	0.629	9.2E-228	Skin_T	SH3BGRL3
XCL11	4.7E-231	2.136294	0.109	0.01	7.5E-227	Skin_T	XCL1
LCK1	9E-231	0.824188	0.354	0.08	1.4E-226	Skin_T	LCK
RPL35	3.7E-230	0.825848	0.994	0.834	6E-226	Skin_T	RPL35
EVI2B3	4.4E-228	0.977246	0.288	0.061	7E-224	Skin_T	EVI2B
COTL12	2E-227	1.145973	0.529	0.18	3.2E-223	Skin_T	COTL1
REL1	2.2E-226	1.463893	0.504	0.182	3.4E-222	Skin_T	REL
ITM2A4	2.2E-225	1.296372	0.438	0.132	3.6E-221	Skin_T	ITM2A
RPL26	3E-223	0.845142	0.999	0.833	4.8E-219	Skin_T	RPL26

JUNB	9.9E-221	1.343826	0.936	0.687	1.6E-216	Skin_T	JUNB
PIP4K2A2	6.9E-220	1.06272	0.267	0.058	1.1E-215	Skin_T	PIP4K2A
FYN3	9.3E-220	1.074929	0.288	0.064	1.5E-215	Skin_T	FYN
TUBA4A1	8E-217	1.462878	0.471	0.165	1.3E-212	Skin_T	TUBA4A
PLP24	6.8E-213	1.180445	0.567	0.227	1.1E-208	Skin_T	PLP2
RPS141	2.8E-212	0.760372	1	0.881	4.5E-208	Skin_T	RPS14
RPS191	4.1E-206	0.783816	1	0.874	6.5E-202	Skin_T	RPS19
RPL374	2.2E-205	0.777174	0.981	0.846	3.5E-201	Skin_T	RPL37
RPL13A	9E-205	0.765017	1	0.88	1.4E-200	Skin_T	RPL13A
SFXN1	6.1E-204	0.996217	0.233	0.048	9.7E-200	Skin_T	SFXN1
EVI2A3	1.2E-202	0.839122	0.201	0.035	1.9E-198	Skin_T	EVI2A
RPL27	2.7E-200	0.827415	0.964	0.747	4.3E-196	Skin_T	RPL27
TNFSF141	7.7E-200	0.516431	0.098	0.009	1.2E-195	Skin_T	TNFSF14
SELK1	3.7E-198	1.502738	0.735	0.466	5.9E-194	Skin_T	SELK
GPR1711	1.3E-197	0.443606	0.083	0.007	2.1E-193	Skin_T	GPR171
LTB	1.1E-196	0.376755	0.547	0.171	1.8E-192	Skin_T	LTB
RORA2	1.4E-196	1.374864	0.374	0.118	2.2E-192	Skin_T	RORA
TSC22D31	4.9E-196	1.348758	0.61	0.278	7.8E-192	Skin_T	TSC22D3
TSPYL21	5.9E-195	1.2377	0.315	0.086	9.3E-191	Skin_T	TSPYL2
RPL18A	8.9E-195	0.724854	0.999	0.821	1.4E-190	Skin_T	RPL18A
ARHGAP15	1.1E-194	0.908642	0.267	0.06	1.7E-190	Skin_T	ARHGAP15
HSPA82	1.4E-194	1.183621	0.796	0.507	2.3E-190	Skin_T	HSPA8
RPL7	8.7E-187	0.744896	0.994	0.794	1.4E-182	Skin_T	RPL7
RPL23	2.6E-186	0.880873	0.932	0.68	4.1E-182	Skin_T	RPL23
CLIC13	2.4E-185	1.078247	0.794	0.471	3.9E-181	Skin_T	CLIC1
DOK22	1.1E-180	0.839464	0.262	0.06	1.8E-176	Skin_T	DOK2
WIPF13	6.7E-179	1.044081	0.32	0.09	1.1E-174	Skin_T	WIPF1
SLAMF12	3.3E-178	0.519304	0.105	0.012	5.2E-174	Skin_T	SLAMF1
ATP5L1	1.1E-175	0.948854	0.881	0.657	1.8E-171	Skin_T	ATP5L
CCDC1074	2.9E-175	1.288249	0.444	0.173	4.6E-171	Skin_T	CCDC107
RGS22	8.9E-175	1.309059	0.324	0.094	1.4E-170	Skin_T	RGS2
PFN1	6.9E-174	0.746665	0.917	0.581	1.1E-169	Skin_T	PFN1
RP4.594I10	2.3E-173	0.521182	0.085	0.008	3.7E-169	Skin_T	RP4.594I10.3
CD8B1	2.5E-172	0.654337	0.201	0.038	4E-168	Skin_T	CD8B
RPL3010	8.5E-171	0.678357	0.993	0.843	1.4E-166	Skin_T	RPL30
ELF11	3.1E-170	1.337227	0.436	0.175	5E-166	Skin_T	ELF1
ACAP11	2.3E-168	0.681783	0.418	0.133	3.7E-164	Skin_T	ACAP1
ZNF90	2.7E-168	1.022128	0.317	0.092	4.4E-164	Skin_T	ZNF90
SERPINB9	1.8E-166	0.893496	0.248	0.06	2.8E-162	Skin_T	SERPINB9
CD482	3.5E-166	0.764065	0.395	0.12	5.5E-162	Skin_T	CD48
PRNP3	1.7E-165	1.16355	0.418	0.156	2.7E-161	Skin_T	PRNP
ARHGDIB	1E-164	0.760133	0.807	0.443	1.7E-160	Skin_T	ARHGDIB
GLIPR12	1.1E-164	1.014754	0.305	0.087	1.8E-160	Skin_T	GLIPR1
RP11.138A	3E-164	0.990554	0.207	0.045	4.8E-160	Skin_T	RP11.138A9.1
IL10RA3	7.6E-164	0.74373	0.207	0.043	1.2E-159	Skin_T	IL10RA
RPL12	4.3E-163	0.673782	1	0.851	6.9E-159	Skin_T	RPL12
LAT1	7.1E-163	0.693516	0.266	0.063	1.1E-158	Skin_T	LAT

CORO1A1	3.4E-161	0.590893	0.547	0.195	5.4E-157	Skin_T	CORO1A
GRAP21	1.4E-160	0.504947	0.114	0.015	2.2E-156	Skin_T	GRAP2
LCP12	3E-160	0.97317	0.339	0.105	4.8E-156	Skin_T	LCP1
CALM23	1.6E-159	0.944871	0.862	0.559	2.6E-155	Skin_T	CALM2
VIM7	5E-159	0.441525	0.923	0.489	8E-155	Skin_T	VIM
RPL37A	3E-158	0.658776	0.988	0.854	4.8E-154	Skin_T	RPL37A
CD371	1E-157	0.486888	0.539	0.199	1.6E-153	Skin_T	CD37
GAPDH3	1.2E-157	1.252592	0.935	0.762	1.9E-153	Skin_T	GAPDH
EMP34	1.3E-156	0.770308	0.721	0.347	2.1E-152	Skin_T	EMP3
RPS16	2.1E-156	0.627741	0.993	0.834	3.3E-152	Skin_T	RPS16
IFITM1	2.5E-153	1.022797	0.432	0.156	4E-149	Skin_T	IFITM1
GZMK1	1.2E-149	1.021078	0.104	0.013	1.9E-145	Skin_T	GZMK
HNRNPA1	1.5E-149	0.767609	0.86	0.541	2.4E-145	Skin_T	HNRNPA1
RPL11	4.7E-149	0.566763	0.998	0.852	7.5E-145	Skin_T	RPL11
RPS2	5.8E-147	0.451626	0.999	0.798	9.3E-143	Skin_T	RPS2
ZFP36	2.8E-145	0.743456	0.852	0.538	4.4E-141	Skin_T	ZFP36
RPS3	1.2E-143	0.417591	0.998	0.814	2E-139	Skin_T	RPS3
RPL191	1.7E-143	0.524421	0.998	0.847	2.7E-139	Skin_T	RPL19
WNT10A1	1.6E-142	0.497428	0.094	0.012	2.5E-138	Skin_T	WNT10A
LDHA1	4.8E-142	1.070313	0.716	0.437	7.7E-138	Skin_T	LDHA
H2AFZ	1.6E-141	1.075727	0.695	0.436	2.5E-137	Skin_T	H2AFZ
RPS6	4.2E-140	0.419624	1	0.822	6.8E-136	Skin_T	RPS6
TMSB101	9.9E-140	0.63327	0.998	0.877	1.6E-135	Skin_T	TMSB10
FMNL13	3.1E-137	0.795617	0.237	0.062	4.9E-133	Skin_T	FMNL1
FAU3	4.9E-137	0.558382	0.989	0.854	7.8E-133	Skin_T	FAU
SLA3	6.1E-136	0.637768	0.135	0.023	9.8E-132	Skin_T	SLA
MCL12	1.1E-135	1.236144	0.619	0.378	1.8E-131	Skin_T	MCL1
BATF1	1.6E-135	0.794766	0.187	0.042	2.6E-131	Skin_T	BATF
RPL33	3.9E-135	0.492592	1	0.841	6.2E-131	Skin_T	RPL3
PTTG1	5.2E-135	0.907733	0.272	0.082	8.2E-131	Skin_T	PTTG1
PPP1R16B1	1.3E-134	0.319585	0.066	0.006	2.1E-130	Skin_T	PPP1R16B
ATP5E2	2.1E-134	0.735863	0.893	0.682	3.3E-130	Skin_T	ATP5E
LPAR63	4E-134	1.111943	0.362	0.138	6.5E-130	Skin_T	LPAR6
STAT41	1.2E-133	0.679638	0.136	0.024	2E-129	Skin_T	STAT4
ETS12	2.5E-132	0.864763	0.258	0.074	4E-128	Skin_T	ETS1
RPS124	3.9E-132	0.559798	0.994	0.86	6.3E-128	Skin_T	RPS12
RPL36A	6.9E-132	0.64499	0.833	0.507	1.1E-127	Skin_T	RPL36A
COMMD61	6.1E-130	0.898672	0.736	0.471	9.8E-126	Skin_T	COMMD6
LPXN2	1.7E-129	0.762864	0.191	0.045	2.8E-125	Skin_T	LPXN
NPM1	6.5E-129	0.703948	0.83	0.53	1E-124	Skin_T	NPM1
RPL229	4.5E-128	0.72053	0.909	0.691	7.1E-124	Skin_T	RPL22
ZC3H12D1	1.1E-127	0.27154	0.044	0.003	1.8E-123	Skin_T	ZC3H12D
GNG22	1.1E-127	0.760045	0.244	0.068	1.8E-123	Skin_T	GNG2
CLEC2B4	2.5E-127	1.263261	0.379	0.153	4E-123	Skin_T	CLEC2B
ELMSAN1	4.9E-127	1.167709	0.251	0.076	7.8E-123	Skin_T	ELMSAN1
FOS5	2.2E-126	0.787661	0.736	0.421	3.5E-122	Skin_T	FOS
IFITM23	3E-125	0.759752	0.612	0.301	4.7E-121	Skin_T	IFITM2

CD51	8.5E-125	0.512141	0.109	0.017	1.4E-120	Skin_T	CD5
NR3C14	1.8E-121	1.221293	0.357	0.146	2.8E-117	Skin_T	NR3C1
EMB3	7.8E-120	0.658669	0.173	0.04	1.2E-115	Skin_T	EMB
AC016831.	1E-119	0.570785	0.109	0.018	1.6E-115	Skin_T	AC016831.7
TUBA1B4	4.9E-118	0.922218	0.623	0.351	7.7E-114	Skin_T	TUBA1B
HLA.C3	8.1E-116	0.511733	0.909	0.683	1.3E-111	Skin_T	HLA.C
IKZF13	7E-115	0.612251	0.166	0.038	1.1E-110	Skin_T	IKZF1
UQCRB3	5.5E-114	0.918715	0.769	0.555	8.8E-110	Skin_T	UQCRB
BEST1	9.4E-114	0.780252	0.17	0.041	1.5E-109	Skin_T	BEST1
ARL4C3	1.2E-113	0.937903	0.42	0.186	1.9E-109	Skin_T	ARL4C
CTLA4	1.7E-112	0.323733	0.04	0.003	2.7E-108	Skin_T	CTLA4
GATA3	4.2E-112	0.978929	0.25	0.082	6.7E-108	Skin_T	GATA3
HLA.B2	4.6E-112	0.340407	0.937	0.663	7.4E-108	Skin_T	HLA.B
TGFB13	7.8E-112	0.892878	0.284	0.096	1.2E-107	Skin_T	TGFB1
OST43	1.8E-111	0.786914	0.745	0.498	2.9E-107	Skin_T	OST4
HNRNPA0	1.8E-110	0.966683	0.575	0.332	2.8E-106	Skin_T	HNRNPA0
GLUD1	3.3E-110	1.186392	0.364	0.161	5.2E-106	Skin_T	GLUD1
KB.1208A1	9.7E-110	0.387661	0.074	0.009	1.5E-105	Skin_T	KB.1208A12.3
TNFRSF9	1.5E-109	0.372428	0.049	0.004	2.4E-105	Skin_T	TNFRSF9
EEF1D1	3.7E-109	0.428345	0.941	0.744	5.8E-105	Skin_T	EEF1D
TOX1	1.3E-108	0.35899	0.065	0.007	2.2E-104	Skin_T	TOX
RPS132	2.1E-108	0.51996	0.988	0.815	3.3E-104	Skin_T	RPS13
TRAF1	1.4E-107	0.57131	0.124	0.024	2.3E-103	Skin_T	TRAF1
RPL36AL2	7.2E-105	0.632959	0.892	0.728	1.2E-100	Skin_T	RPL36AL
SUB12	8.6E-105	0.822195	0.677	0.478	1.4E-100	Skin_T	SUB1
CFL1	9.1E-105	0.606248	0.885	0.707	1.4E-100	Skin_T	CFL1
SNRPD2	9.4E-103	0.797874	0.673	0.43	1.5E-98	Skin_T	SNRPD2
HSPA1A4	9.5E-103	1.053565	0.575	0.323	1.52E-98	Skin_T	HSPA1A
MYL62	4.2E-100	0.552399	0.949	0.804	6.63E-96	Skin_T	MYL6
RPS10	8.4E-99	0.57213	0.848	0.605	1.34E-94	Skin_T	RPS10
CKS2	1.25E-98	1.030478	0.333	0.14	1.99E-94	Skin_T	CKS2
PRPF38B1	1.12E-97	1.06816	0.389	0.19	1.78E-93	Skin_T	PRPF38B
OASL1	1.49E-97	0.465995	0.092	0.015	2.37E-93	Skin_T	OASL
DUSP4	8.62E-96	1.105339	0.404	0.194	1.37E-91	Skin_T	DUSP4
HLA.A2	2.21E-95	0.310191	0.911	0.665	3.53E-91	Skin_T	HLA.A
IL2RG2	5.08E-95	0.618059	0.339	0.13	8.1E-91	Skin_T	IL2RG
TNFRSF25	2.24E-94	0.475064	0.108	0.021	3.57E-90	Skin_T	TNFRSF25
RPL15	8.07E-94	0.434133	0.998	0.894	1.29E-89	Skin_T	RPL15
RPS72	2.44E-93	0.398111	0.981	0.814	3.89E-89	Skin_T	RPS7
DDIT4	2.96E-93	0.955365	0.463	0.24	4.72E-89	Skin_T	DDIT4
PNRC1	9.42E-93	0.725205	0.72	0.508	1.5E-88	Skin_T	PNRC1
STMN1	8.12E-92	0.77721	0.407	0.188	1.3E-87	Skin_T	STMN1
TUBB2	9.44E-91	0.887293	0.535	0.308	1.51E-86	Skin_T	TUBB
PDCL3	3.78E-90	0.902948	0.232	0.082	6.03E-86	Skin_T	PDCL3
RAB8B1	2.19E-89	0.877047	0.254	0.096	3.49E-85	Skin_T	RAB8B
RPL10A1	3.94E-89	0.378986	0.992	0.732	6.28E-85	Skin_T	RPL10A
RPL142	1.48E-88	0.38333	0.975	0.794	2.36E-84	Skin_T	RPL14

SRSF23	1.38E-87	0.981621	0.527	0.33	2.2E-83	Skin_T	SRSF2
CLK1	1.68E-86	0.951682	0.444	0.249	2.68E-82	Skin_T	CLK1
ZFAS1	2.49E-86	0.668902	0.686	0.463	3.97E-82	Skin_T	ZFAS1
RPL13	2.02E-85	0.257401	1	0.917	3.22E-81	Skin_T	RPL13
PCSK73	2.58E-85	0.921216	0.273	0.11	4.11E-81	Skin_T	PCSK7
FAM129A8	4.44E-85	0.906298	0.26	0.102	7.08E-81	Skin_T	FAM129A
IL2RB1	5.25E-85	0.361839	0.086	0.015	8.38E-81	Skin_T	IL2RB
SRSF76	2.13E-84	0.898675	0.538	0.341	3.39E-80	Skin_T	SRSF7
METTL15	3.65E-84	0.883252	0.162	0.047	5.82E-80	Skin_T	METTL15
LAX12	4.27E-84	0.271973	0.046	0.005	6.81E-80	Skin_T	LAX1
ANXA61	5.59E-84	0.720723	0.275	0.106	8.91E-80	Skin_T	ANXA6
RPS112	1.47E-83	0.464781	0.965	0.775	2.35E-79	Skin_T	RPS11
RSL24D1	2.33E-83	0.912397	0.5	0.304	3.72E-79	Skin_T	RSL24D1
HNRNPH11	3.94E-82	1.030604	0.488	0.296	6.29E-78	Skin_T	HNRNPH1
CD991	8.52E-81	0.665704	0.691	0.49	1.36E-76	Skin_T	CD99
CDC421	1.56E-80	0.855387	0.637	0.468	2.49E-76	Skin_T	CDC42
PDE4B1	2.71E-80	0.723065	0.166	0.05	4.33E-76	Skin_T	PDE4B
SOCS11	2.96E-80	0.651251	0.161	0.047	4.72E-76	Skin_T	SOCS1
ISG201	8.95E-80	0.757309	0.407	0.2	1.43E-75	Skin_T	ISG20
NFKBID2	1.44E-79	0.664337	0.154	0.043	2.3E-75	Skin_T	NFKBID
TNFAIP3	5.57E-79	0.815192	0.347	0.159	8.88E-75	Skin_T	TNFAIP3
CYCS1	2.79E-78	0.782873	0.531	0.339	4.45E-74	Skin_T	CYCS
HINT18	3.08E-78	0.649977	0.776	0.568	4.92E-74	Skin_T	HINT1
RPSA1	3.26E-78	0.278591	0.935	0.675	5.21E-74	Skin_T	RPSA
FGFR1OP2	5.57E-78	1.031167	0.319	0.152	8.88E-74	Skin_T	FGFR1OP2
ITGB22	6.33E-78	0.481313	0.283	0.107	1.01E-73	Skin_T	ITGB2
MYL12A2	7.31E-78	0.631984	0.83	0.656	1.17E-73	Skin_T	MYL12A
CRIP11	1.15E-77	0.709946	0.5	0.274	1.84E-73	Skin_T	CRIP1
CD61	4.41E-77	0.454026	0.12	0.028	7.03E-73	Skin_T	CD6
FAM177A1	4.9E-77	1.162409	0.384	0.213	7.81E-73	Skin_T	FAM177A1
CCND22	1.25E-76	0.886298	0.249	0.1	1.99E-72	Skin_T	CCND2
RNF19A	3.43E-76	0.964508	0.277	0.122	5.48E-72	Skin_T	RNF19A
MIF	8.5E-76	0.747395	0.67	0.479	1.36E-71	Skin_T	MIF
CD533	8.84E-76	0.498863	0.305	0.121	1.41E-71	Skin_T	CD53
CLDND1	1.83E-75	1.020727	0.359	0.189	2.92E-71	Skin_T	CLDND1
RPL6	3.94E-75	0.300217	0.972	0.791	6.28E-71	Skin_T	RPL6
GPSM31	3.24E-74	0.400026	0.386	0.17	5.17E-70	Skin_T	GPSM3
HNRNPDL	5.18E-74	0.700045	0.548	0.334	8.26E-70	Skin_T	HNRNPDL
MYO1G1	7.6E-74	0.416586	0.125	0.031	1.21E-69	Skin_T	MYO1G
C16orf543	1.17E-73	0.324798	0.064	0.01	1.86E-69	Skin_T	C16orf54
KPNA2	2.59E-73	0.818509	0.255	0.105	4.13E-69	Skin_T	KPNA2
SNHG15	3.7E-73	0.791318	0.262	0.109	5.91E-69	Skin_T	SNHG15
AIM1	1.35E-72	0.835685	0.253	0.105	2.15E-68	Skin_T	AIM1
AHR2	1.54E-72	0.838742	0.255	0.106	2.46E-68	Skin_T	AHR
ADAM19	1.84E-72	0.336198	0.058	0.009	2.94E-68	Skin_T	ADAM19
ACTB1	5.18E-72	0.661368	0.974	0.871	8.26E-68	Skin_T	ACTB
EIF4A21	1.01E-70	0.761034	0.644	0.484	1.61E-66	Skin_T	EIF4A2

RPS9	1.66E-70	0.342188	0.993	0.835	2.65E-66	Skin_T	RPS9
NCR3	1.85E-70	0.420854	0.097	0.021	2.95E-66	Skin_T	NCR3
AP003419.	2.38E-70	0.266028	0.047	0.006	3.79E-66	Skin_T	AP003419.16
COX7C3	2.58E-70	0.698534	0.805	0.636	4.12E-66	Skin_T	COX7C
CAPZA1	3.64E-70	1.000429	0.439	0.276	5.81E-66	Skin_T	CAPZA1
PBX4	4.28E-70	0.371312	0.069	0.012	6.83E-66	Skin_T	PBX4
ICAM32	7.8E-70	0.54712	0.323	0.143	1.24E-65	Skin_T	ICAM3
GNA13	1.03E-69	0.760429	0.216	0.083	1.64E-65	Skin_T	GNA13
RPL93	1.09E-69	0.31436	0.975	0.747	1.73E-65	Skin_T	RPL9
RAC21	3.21E-69	0.383541	0.357	0.154	5.12E-65	Skin_T	RAC2
CRTAM1	1.08E-68	0.452924	0.051	0.007	1.73E-64	Skin_T	CRTAM
RP11.345J4	2.07E-68	0.511787	0.112	0.028	3.3E-64	Skin_T	RP11.345J4.5
TPSAB11	2.17E-68	0.346243	0.063	0.01	3.46E-64	Skin_T	TPSAB1
PRR7	6.19E-68	0.64941	0.155	0.049	9.87E-64	Skin_T	PRR7
SKAP11	2.05E-67	0.549897	0.174	0.057	3.28E-63	Skin_T	SKAP1
RCBTB21	3.84E-66	0.726796	0.157	0.051	6.12E-62	Skin_T	RCBTB2
RPS3A1	1.91E-65	0.278257	0.993	0.829	3.04E-61	Skin_T	RPS3A
FLNA6	4.36E-65	0.639475	0.314	0.147	6.95E-61	Skin_T	FLNA
RBM392	4.87E-65	0.793052	0.565	0.409	7.77E-61	Skin_T	RBM39
CSRNP1	8.98E-65	0.842037	0.286	0.136	1.43E-60	Skin_T	CSRNP1
RPL248	8.06E-64	0.384999	0.95	0.8	1.29E-59	Skin_T	RPL24
TOMM72	1.04E-63	0.528895	0.765	0.58	1.66E-59	Skin_T	TOMM7
H2AFV1	2.25E-63	0.903224	0.445	0.289	3.58E-59	Skin_T	H2AFV
G3BP22	5.5E-63	0.976352	0.322	0.173	8.78E-59	Skin_T	G3BP2
PPP2R5C1	5.28E-62	0.98041	0.335	0.185	8.43E-58	Skin_T	PPP2R5C
RP5.940J5.	7.38E-62	0.353542	0.064	0.012	1.18E-57	Skin_T	RP5.940J5.9
H3F3A12	1.7E-61	0.41516	0.774	0.475	2.7E-57	Skin_T	H3F3A
CHST11	2.15E-61	0.4411	0.1	0.025	3.43E-57	Skin_T	CHST11
TERF2IP1	4.74E-61	0.870885	0.38	0.223	7.55E-57	Skin_T	TERF2IP
ARPC23	6.48E-61	0.622852	0.704	0.573	1.03E-56	Skin_T	ARPC2
S100A104	2.98E-60	0.261585	0.906	0.673	4.76E-56	Skin_T	S100A10
PPP1CB	4.05E-60	0.74033	0.62	0.454	6.45E-56	Skin_T	PPP1CB
NR4A3	4.94E-60	0.60185	0.156	0.053	7.89E-56	Skin_T	NR4A3
NDUFS54	6.23E-60	0.696079	0.6	0.453	9.93E-56	Skin_T	NDUFS5
SPN1	8.71E-59	0.372293	0.095	0.023	1.39E-54	Skin_T	SPN
EIF3E2	1.1E-58	0.597963	0.626	0.447	1.76E-54	Skin_T	EIF3E
SEPT112	1.29E-58	0.726799	0.207	0.085	2.05E-54	Skin_T	SEPT11
RAP1A	3.35E-58	0.846444	0.421	0.273	5.35E-54	Skin_T	RAP1A
TESPA1	6.4E-58	0.291565	0.067	0.013	1.02E-53	Skin_T	TESPA1
EIF11	1.05E-57	0.345964	0.994	0.908	1.68E-53	Skin_T	EIF1
CTD.2020K	3.46E-57	0.345158	0.071	0.015	5.52E-53	Skin_T	CTD.2020K17.1
ANP32E	4.55E-56	0.750328	0.221	0.096	7.26E-52	Skin_T	ANP32E
GZMA1	4.91E-55	0.3877	0.129	0.038	7.84E-51	Skin_T	GZMA
SLC25A51	5.35E-55	0.783299	0.526	0.367	8.52E-51	Skin_T	SLC25A5
RPS235	7.23E-55	0.317023	0.99	0.811	1.15E-50	Skin_T	RPS23
RAN2	1.49E-54	0.66522	0.528	0.365	2.37E-50	Skin_T	RAN
GMFG2	1.73E-54	0.324286	0.411	0.207	2.76E-50	Skin_T	GMFG

ARID5B3	2.96E-54	0.674344	0.434	0.267	4.72E-50	Skin_T	ARID5B
ITPKB1	3.14E-54	0.504297	0.123	0.038	5.01E-50	Skin_T	ITPKB
RBM3	7.95E-54	0.635825	0.59	0.438	1.27E-49	Skin_T	RBM3
ZBTB11	2.6E-53	0.670035	0.182	0.073	4.15E-49	Skin_T	ZBTB1
AKNA1	1.11E-52	0.493311	0.118	0.036	1.77E-48	Skin_T	AKNA
MAF1	1.88E-52	0.733233	0.194	0.081	3E-48	Skin_T	MAF
PRKX2	2.19E-52	0.559485	0.128	0.042	3.5E-48	Skin_T	PRKX
SIAH21	1.19E-51	0.726892	0.228	0.104	1.89E-47	Skin_T	SIAH2
MECP21	1.82E-51	0.715379	0.2	0.087	2.91E-47	Skin_T	MECP2
PSTPIP12	2.47E-51	0.333882	0.091	0.023	3.94E-47	Skin_T	PSTPIP1
RCAN32	2.71E-51	0.621441	0.165	0.063	4.31E-47	Skin_T	RCAN3
YPEL5	8.07E-51	0.84886	0.483	0.345	1.29E-46	Skin_T	YPEL5
KMT2E1	1.56E-50	0.783551	0.44	0.298	2.48E-46	Skin_T	KMT2E
U2AF1L4	1.62E-50	0.542108	0.165	0.063	2.59E-46	Skin_T	U2AF1L4
SH2D2A1	3.91E-50	0.304467	0.065	0.014	6.24E-46	Skin_T	SH2D2A
TPI1	4.04E-50	0.671307	0.647	0.534	6.45E-46	Skin_T	TPI1
CHD2	7.12E-50	0.856605	0.342	0.206	1.14E-45	Skin_T	CHD2
SCML41	8.05E-50	0.336171	0.081	0.02	1.28E-45	Skin_T	SCML4
HNRNPA1L	9.91E-50	0.554679	0.137	0.048	1.58E-45	Skin_T	HNRNPA1L2
RILPL21	1.36E-49	0.750953	0.212	0.096	2.16E-45	Skin_T	RILPL2
CAPG2	3E-49	0.627809	0.309	0.165	4.78E-45	Skin_T	CAPG
ZAP701	1.33E-48	0.28171	0.101	0.028	2.13E-44	Skin_T	ZAP70
CA6	7.43E-48	0.287004	0.021	0.002	1.18E-43	Skin_T	CA6
RNF1252	1.15E-47	0.477556	0.113	0.036	1.83E-43	Skin_T	RNF125
CCNH1	7E-47	0.791622	0.23	0.113	1.12E-42	Skin_T	CCNH
HMCES	5.14E-46	0.596589	0.144	0.054	8.19E-42	Skin_T	HMCES
GEM	5.61E-46	0.591794	0.199	0.085	8.94E-42	Skin_T	GEM
USP36	5.68E-46	0.651519	0.157	0.063	9.05E-42	Skin_T	USP36
B3GAT2	1.13E-45	0.458125	0.095	0.028	1.8E-41	Skin_T	B3GAT2
CCL42	1.77E-45	2.130311	0.129	0.045	2.83E-41	Skin_T	CCL4
PHTF2	2.68E-45	0.595022	0.137	0.051	4.27E-41	Skin_T	PHTF2
C7orf55.LU	2.86E-45	0.472697	0.129	0.045	4.56E-41	Skin_T	C7orf55.LUC7L2
CDKN2D	5.84E-45	0.595842	0.189	0.082	9.31E-41	Skin_T	CDKN2D
ARHGAP3C	8.03E-45	0.417035	0.136	0.048	1.28E-40	Skin_T	ARHGAP30
RHOF	1.03E-44	0.548778	0.188	0.081	1.64E-40	Skin_T	RHOF
TMA7	2.16E-44	0.516874	0.748	0.66	3.45E-40	Skin_T	TMA7
SNRPG	2.17E-44	0.703189	0.474	0.353	3.46E-40	Skin_T	SNRPG
TTC39C1	2.97E-44	0.714952	0.212	0.1	4.74E-40	Skin_T	TTC39C
SOD13	9.37E-44	0.603019	0.563	0.414	1.49E-39	Skin_T	SOD1
TIPIN	9.95E-44	0.642524	0.17	0.073	1.59E-39	Skin_T	TIPIN
AC009501.	1.04E-43	0.458487	0.102	0.032	1.65E-39	Skin_T	AC009501.4
RNF139	1.77E-43	0.733535	0.252	0.13	2.82E-39	Skin_T	RNF139
EVL1	1.92E-43	0.438001	0.314	0.165	3.06E-39	Skin_T	EVL
SP1402	1.14E-42	0.319714	0.072	0.018	1.82E-38	Skin_T	SP140
DDX246	1.63E-42	0.691223	0.372	0.236	2.59E-38	Skin_T	DDX24
CALM17	3.14E-42	0.416941	0.787	0.65	5.02E-38	Skin_T	CALM1
DOCK102	5.78E-42	0.326746	0.069	0.017	9.21E-38	Skin_T	DOCK10

PPIA2	2.1E-41	0.480501	0.706	0.588	3.35E-37	Skin_T	PPIA
RP11.51J9.	2.69E-41	0.508503	0.133	0.049	4.29E-37	Skin_T	RP11.51J9.5
A1BG1	5.11E-41	0.507639	0.153	0.062	8.15E-37	Skin_T	A1BG
PPP1R2	7.16E-41	0.807834	0.388	0.271	1.14E-36	Skin_T	PPP1R2
CKLF3	1.48E-40	0.699161	0.271	0.149	2.36E-36	Skin_T	CKLF
FAM46C1	2E-40	0.502556	0.19	0.086	3.19E-36	Skin_T	FAM46C
HSP90AB1	2.77E-40	0.487895	0.711	0.569	4.42E-36	Skin_T	HSP90AB1
PREX12	3.76E-40	0.330815	0.077	0.021	6E-36	Skin_T	PREX1
MOB4	4.43E-40	0.668579	0.193	0.091	7.06E-36	Skin_T	MOB4
TAGLN24	4.51E-40	0.541717	0.564	0.407	7.19E-36	Skin_T	TAGLN2
FXYD21	5.42E-40	0.61685	0.086	0.026	8.65E-36	Skin_T	FXYD2
SS18L21	6.54E-40	0.716041	0.24	0.129	1.04E-35	Skin_T	SS18L2
AMPD2	3.18E-39	0.375098	0.091	0.028	5.07E-35	Skin_T	AMPD2
TSPAN22	3.45E-39	0.41038	0.085	0.025	5.5E-35	Skin_T	TSPAN2
CARD16	2.21E-38	0.644441	0.2	0.095	3.53E-34	Skin_T	CARD16
COX7B5	2.28E-38	0.672839	0.514	0.408	3.64E-34	Skin_T	COX7B
APOBEC3C	2.45E-38	0.518657	0.135	0.053	3.9E-34	Skin_T	APOBEC3C
TPM33	1.67E-37	0.645547	0.449	0.331	2.66E-33	Skin_T	TPM3
JUND6	2.25E-37	0.455655	0.588	0.463	3.59E-33	Skin_T	JUND
HSPE11	2.91E-37	0.68601	0.486	0.371	4.64E-33	Skin_T	HSPE1
RBM8A	5.46E-37	0.6256	0.449	0.342	8.7E-33	Skin_T	RBM8A
TGIF1	7.63E-37	0.799945	0.309	0.195	1.22E-32	Skin_T	TGIF1
ATP1B31	1.54E-36	0.725885	0.357	0.235	2.45E-32	Skin_T	ATP1B3
AMD1	2.06E-36	0.780717	0.306	0.193	3.28E-32	Skin_T	AMD1
SLC38A15	2.13E-36	0.709808	0.195	0.098	3.39E-32	Skin_T	SLC38A1
ARID5A	4.13E-36	0.540832	0.2	0.097	6.59E-32	Skin_T	ARID5A
ATP2B13	5.51E-36	0.694988	0.261	0.149	8.78E-32	Skin_T	ATP2B1
AC093673.	1.07E-35	0.291581	0.062	0.016	1.71E-31	Skin_T	AC093673.5
PGAP1	1.21E-35	0.493119	0.085	0.027	1.93E-31	Skin_T	PGAP1
ANKRD442	1.45E-35	0.393412	0.101	0.035	2.31E-31	Skin_T	ANKRD44
PIM21	2.71E-35	0.409843	0.112	0.041	4.32E-31	Skin_T	PIM2
SELT2	5.16E-35	0.748399	0.334	0.226	8.22E-31	Skin_T	SELT
SET3	5.3E-35	0.705331	0.421	0.31	8.45E-31	Skin_T	SET
SH3KBP11	2.12E-34	0.495408	0.183	0.087	3.38E-30	Skin_T	SH3KBP1
PBXIP12	2.41E-34	0.587975	0.216	0.114	3.84E-30	Skin_T	PBXIP1
RHOG1	3.35E-34	0.59197	0.302	0.185	5.34E-30	Skin_T	RHOG
SRSF32	5.36E-34	0.63327	0.534	0.432	8.55E-30	Skin_T	SRSF3
PAG12	8.05E-34	0.509624	0.12	0.048	1.28E-29	Skin_T	PAG1
ZFYVE281	9.66E-34	0.362485	0.072	0.021	1.54E-29	Skin_T	ZFYVE28
KLF63	1.19E-33	0.549491	0.532	0.401	1.91E-29	Skin_T	KLF6
MATK1	1.2E-33	0.274728	0.086	0.028	1.91E-29	Skin_T	MATK
MAP4K11	1.68E-33	0.251011	0.063	0.017	2.68E-29	Skin_T	MAP4K1
CCDC88C2	2.01E-33	0.41894	0.109	0.041	3.2E-29	Skin_T	CCDC88C
CD842	2.16E-33	0.298177	0.072	0.021	3.44E-29	Skin_T	CD84
EEF21	7.37E-33	0.305137	0.765	0.606	1.18E-28	Skin_T	EEF2
FOSL2	1.8E-32	0.70805	0.33	0.218	2.88E-28	Skin_T	FOSL2
AAED12	1.9E-32	0.605863	0.16	0.075	3.04E-28	Skin_T	AAED1

APRT3	1.93E-32	0.400867	0.5	0.368	3.07E-28	Skin_T		APRT
DNAJA11	1.98E-32	0.656833	0.466	0.36	3.16E-28	Skin_T		DNAJA1
SMCHD12	2.32E-32	0.72424	0.221	0.123	3.69E-28	Skin_T		SMCHD1
BTG26	2.66E-32	0.4644	0.36	0.231	4.25E-28	Skin_T		BTG2
OAZ1	3.92E-32	0.276427	0.867	0.782	6.26E-28	Skin_T		OAZ1
SMAP22	1.71E-31	0.493305	0.149	0.067	2.72E-27	Skin_T		SMAP2
NAP1L1	1.8E-31	0.452994	0.524	0.4	2.88E-27	Skin_T		NAP1L1
CBX31	2.19E-31	0.638534	0.307	0.201	3.49E-27	Skin_T		CBX3
STK17A	2.24E-31	0.573968	0.253	0.147	3.57E-27	Skin_T		STK17A
MFHAS1	2.41E-31	0.459963	0.105	0.04	3.85E-27	Skin_T		MFHAS1
GZMB1	3.54E-31	0.395839	0.083	0.027	5.64E-27	Skin_T		GZMB
WAS2	4.21E-31	0.277393	0.114	0.044	6.72E-27	Skin_T		WAS
SON5	7.2E-31	0.58682	0.503	0.409	1.15E-26	Skin_T		SON
TNFAIP83	8.29E-31	0.463358	0.202	0.104	1.32E-26	Skin_T		TNFAIP8
AC010642.	8.4E-31	0.358283	0.098	0.036	1.34E-26	Skin_T		AC010642.1
INSIG1	4.92E-30	0.358894	0.314	0.206	7.84E-26	Skin_T		INSIG1
CCDC85B1	1.15E-29	0.452503	0.395	0.271	1.84E-25	Skin_T		CCDC85B
SEPT12	1.23E-29	0.350066	0.198	0.098	1.96E-25	Skin_T		SEPT1
SLFN111	1.52E-29	0.3552	0.087	0.031	2.42E-25	Skin_T		SLFN11
LAG31	1.58E-29	0.289216	0.047	0.012	2.53E-25	Skin_T		LAG3
SDCBP1	1.61E-29	0.497984	0.523	0.411	2.57E-25	Skin_T		SDCBP
SSR23	1.62E-29	0.331918	0.548	0.427	2.59E-25	Skin_T		SSR2
TSEN54	3.02E-29	0.384724	0.104	0.041	4.82E-25	Skin_T		TSEN54
NDUFA61	3.99E-29	0.72334	0.402	0.321	6.37E-25	Skin_T		NDUFA6
ZBTB25	4.51E-29	0.436706	0.091	0.034	7.2E-25	Skin_T		ZBTB25
MATR3	6.57E-29	0.515576	0.209	0.115	1.05E-24	Skin_T		MATR3
IL27RA	9.51E-29	0.34158	0.1	0.038	1.52E-24	Skin_T		IL27RA
SNRPB21	1.27E-28	0.711141	0.341	0.247	2.03E-24	Skin_T		SNRPB2
PHF191	1.3E-28	0.348027	0.08	0.028	2.07E-24	Skin_T		PHF19
CDK2AP21	1.63E-28	0.684464	0.262	0.164	2.59E-24	Skin_T		CDK2AP2
JMJD6	2.86E-28	0.581286	0.172	0.09	4.56E-24	Skin_T		JMJD6
CTSC3	3.4E-28	0.462337	0.219	0.12	5.43E-24	Skin_T		CTSC
LSP11	5.94E-28	0.292672	0.368	0.229	9.48E-24	Skin_T		LSP1
RAP1B	1.06E-27	0.604372	0.368	0.268	1.69E-23	Skin_T		RAP1B
JADE2	1.09E-27	0.323663	0.072	0.024	1.73E-23	Skin_T		JADE2
C9orf781	1.33E-27	0.564347	0.274	0.173	2.13E-23	Skin_T		C9orf78
HERPUD13	1.65E-27	0.409696	0.431	0.332	2.63E-23	Skin_T		HERPUD1
ATP5G2	1.76E-27	0.268626	0.688	0.572	2.8E-23	Skin_T		ATP5G2
LINC.PINT1	2.98E-27	0.455857	0.105	0.043	4.75E-23	Skin_T		LINC.PINT
PCNP	4.69E-27	0.65005	0.353	0.259	7.48E-23	Skin_T		PCNP
APOBEC3G	2.28E-26	0.348737	0.109	0.045	3.64E-22	Skin_T		APOBEC3G
LIX1L1	2.28E-26	0.432779	0.108	0.046	3.64E-22	Skin_T		LIX1L
PARP81	2.46E-26	0.447724	0.095	0.038	3.93E-22	Skin_T		PARP8
CUTA1	4.56E-26	0.458966	0.472	0.375	7.27E-22	Skin_T		CUTA
SGTB	5.03E-26	0.279506	0.063	0.02	8.03E-22	Skin_T		SGTB
GZMH1	8.45E-26	0.256582	0.064	0.02	1.35E-21	Skin_T		GZMH
ADAM81	1.21E-25	0.256891	0.079	0.028	1.93E-21	Skin_T		ADAM8

CMPK12	1.36E-25	0.593595	0.388	0.3	2.17E-21	Skin_T	CMPK1
LBH1	2.17E-25	0.410145	0.182	0.095	3.46E-21	Skin_T	LBH
CDC263	2.17E-25	0.679641	0.207	0.126	3.47E-21	Skin_T	CDC26
TCF71	2.62E-25	0.33405	0.112	0.048	4.18E-21	Skin_T	TCF7
CDKN1B	7.72E-25	0.605051	0.252	0.161	1.23E-20	Skin_T	CDKN1B
MBNL14	1.14E-24	0.523796	0.266	0.17	1.81E-20	Skin_T	MBNL1
PDE4A	1.75E-24	0.506593	0.113	0.051	2.79E-20	Skin_T	PDE4A
MOB3A	1.96E-24	0.363104	0.107	0.046	3.13E-20	Skin_T	MOB3A
CD200R11	1.97E-24	0.251547	0.062	0.02	3.15E-20	Skin_T	CD200R1
SEPT62	3.86E-24	0.395805	0.176	0.094	6.15E-20	Skin_T	SEPT6
EGR22	5.94E-24	0.438793	0.085	0.034	9.47E-20	Skin_T	EGR2
SKP13	7.02E-24	0.401768	0.649	0.57	1.12E-19	Skin_T	SKP1
MTHFD21	1.08E-23	0.543479	0.175	0.098	1.72E-19	Skin_T	MTHFD2
HIGD2A	1.08E-23	0.435299	0.438	0.351	1.72E-19	Skin_T	HIGD2A
ARL6IP54	2.15E-23	0.458417	0.407	0.309	3.42E-19	Skin_T	ARL6IP5
ASF1A	4.9E-23	0.482922	0.131	0.065	7.81E-19	Skin_T	ASF1A
IL162	5.33E-23	0.296437	0.118	0.053	8.5E-19	Skin_T	IL16
DUSP10	5.98E-23	0.531909	0.131	0.066	9.53E-19	Skin_T	DUSP10
SLC7A5	8.63E-23	0.499099	0.115	0.055	1.38E-18	Skin_T	SLC7A5
TMEM21	8.68E-23	0.482718	0.121	0.058	1.38E-18	Skin_T	TMEM2
VPS37B	1.43E-22	0.678219	0.233	0.152	2.28E-18	Skin_T	VPS37B
HECA	1.85E-22	0.653662	0.228	0.147	2.95E-18	Skin_T	HECA
U2AF1	1.96E-22	0.433201	0.356	0.255	3.13E-18	Skin_T	U2AF1
TRA2A	2.86E-22	0.692643	0.244	0.164	4.56E-18	Skin_T	TRA2A
STX11	5.93E-22	0.485264	0.129	0.065	9.45E-18	Skin_T	STX11
GPBP1	9.98E-22	0.653093	0.393	0.32	1.59E-17	Skin_T	GPBP1
SNHG83	1.33E-21	0.482183	0.388	0.296	2.12E-17	Skin_T	SNHG8
DNAJB1	1.94E-21	0.852889	0.511	0.429	3.1E-17	Skin_T	DNAJB1
CDK171	2.28E-21	0.384763	0.091	0.04	3.64E-17	Skin_T	CDK17
HMGB16	2.5E-21	0.28481	0.663	0.575	3.99E-17	Skin_T	HMGB1
DNAJB91	2.71E-21	0.583266	0.29	0.207	4.32E-17	Skin_T	DNAJB9
ARPP19	4.19E-21	0.302586	0.305	0.217	6.68E-17	Skin_T	ARPP19
RNF1661	5.15E-21	0.290339	0.107	0.049	8.21E-17	Skin_T	RNF166
PKM2	6.49E-21	0.446827	0.463	0.372	1.03E-16	Skin_T	PKM
CXCR31	7.42E-21	0.252382	0.057	0.019	1.18E-16	Skin_T	CXCR3
CMTM7	2.23E-20	0.349353	0.135	0.069	3.55E-16	Skin_T	CMTM7
METTL9	2.23E-20	0.634186	0.296	0.221	3.56E-16	Skin_T	METTL9
CYLD1	2.4E-20	0.569799	0.199	0.124	3.83E-16	Skin_T	CYLD
BAZ1A	1.11E-19	0.614028	0.322	0.244	1.76E-15	Skin_T	BAZ1A
ANKRD125	1.13E-19	0.62413	0.388	0.323	1.81E-15	Skin_T	ANKRD12
ARHGDIA4	2.35E-19	0.405237	0.284	0.196	3.74E-15	Skin_T	ARHGDIA
C1orf561	4.23E-19	0.657686	0.193	0.121	6.74E-15	Skin_T	C1orf56
UBE2D3	4.91E-19	0.365543	0.602	0.533	7.83E-15	Skin_T	UBE2D3
C6orf48	1.61E-18	0.25772	0.418	0.312	2.57E-14	Skin_T	C6orf48
FAM118A	1.93E-18	0.377481	0.135	0.073	3.08E-14	Skin_T	FAM118A
TRMT1121	1.97E-18	0.416437	0.447	0.374	3.15E-14	Skin_T	TRMT112
DNAJB6	2.56E-18	0.454432	0.486	0.419	4.09E-14	Skin_T	DNAJB6

GLIPR23	6.7E-18	0.396215	0.15	0.085	1.07E-13	Skin_T	GLIPR2
CIRBP3	9.24E-18	0.280002	0.621	0.545	1.47E-13	Skin_T	CIRBP
HNRNPA32	1.01E-17	0.47987	0.424	0.365	1.62E-13	Skin_T	HNRNPA3
OSTF1	1.38E-17	0.54342	0.312	0.241	2.21E-13	Skin_T	OSTF1
ENO11	1.47E-17	0.457318	0.481	0.411	2.34E-13	Skin_T	ENO1
TFRC1	1.52E-17	0.434463	0.105	0.053	2.43E-13	Skin_T	TFRC
GABARAPL	1.61E-17	0.613171	0.235	0.167	2.57E-13	Skin_T	GABARAPL1
NKRF	1.75E-17	0.275006	0.053	0.019	2.79E-13	Skin_T	NKRF
ZNF6831	2.17E-17	0.304037	0.07	0.029	3.46E-13	Skin_T	ZNF683
CTH	2.37E-17	0.323692	0.048	0.017	3.77E-13	Skin_T	CTH
DDX21	2.45E-17	0.507664	0.275	0.199	3.91E-13	Skin_T	DDX21
PRMT7	2.47E-17	0.314196	0.063	0.025	3.94E-13	Skin_T	PRMT7
SNRPF1	2.77E-17	0.512245	0.31	0.237	4.41E-13	Skin_T	SNRPF
BRD21	3.82E-17	0.58917	0.333	0.271	6.09E-13	Skin_T	BRD2
ITGB13	3.98E-17	0.466059	0.421	0.357	6.35E-13	Skin_T	ITGB1
CHD12	4.15E-17	0.535515	0.2	0.131	6.63E-13	Skin_T	CHD1
PDE7A2	5.77E-17	0.402052	0.101	0.051	9.2E-13	Skin_T	PDE7A
GGA11	6.39E-17	0.579239	0.184	0.118	1.02E-12	Skin_T	GGA1
FUS7	9.19E-17	0.489838	0.348	0.276	1.47E-12	Skin_T	FUS
RARA	1.32E-16	0.436992	0.106	0.055	2.11E-12	Skin_T	RARA
NFATC1	2.59E-16	0.333406	0.087	0.041	4.14E-12	Skin_T	NFATC1
VASP2	3.21E-16	0.476316	0.197	0.129	5.12E-12	Skin_T	VASP
PTGES32	3.37E-16	0.419044	0.453	0.393	5.38E-12	Skin_T	PTGES3
HNRNPLL	3.64E-16	0.525321	0.119	0.065	5.81E-12	Skin_T	HNRNPLL
NBAS1	4.15E-16	0.476971	0.1	0.052	6.61E-12	Skin_T	NBAS
MBP1	4.2E-16	0.543568	0.221	0.153	6.7E-12	Skin_T	MBP
RBM382	5.23E-16	0.275147	0.08	0.036	8.35E-12	Skin_T	RBM38
RAB27A3	7.71E-16	0.429366	0.118	0.065	1.23E-11	Skin_T	RAB27A
ELOVL5	7.88E-16	0.48129	0.17	0.107	1.26E-11	Skin_T	ELOVL5
TAP11	1.16E-15	0.360696	0.158	0.095	1.85E-11	Skin_T	TAP1
SUMO24	1.29E-15	0.277631	0.661	0.601	2.06E-11	Skin_T	SUMO2
NAB2	1.4E-15	0.300967	0.065	0.028	2.24E-11	Skin_T	NAB2
ETV3	1.54E-15	0.348875	0.095	0.048	2.46E-11	Skin_T	ETV3
YWHAZ	1.68E-15	0.342437	0.568	0.509	2.68E-11	Skin_T	YWHAZ
RGS102	1.9E-15	0.391492	0.291	0.215	3.03E-11	Skin_T	RGS10
PAN3	2.49E-15	0.462856	0.137	0.081	3.97E-11	Skin_T	PAN3
FAM105A1	3.35E-15	0.350242	0.078	0.036	5.35E-11	Skin_T	FAM105A
MORF4L1	4.75E-15	0.348728	0.514	0.458	7.57E-11	Skin_T	MORF4L1
DCXR4	5.71E-15	0.513639	0.284	0.22	9.1E-11	Skin_T	DCXR
PCNX1	6.39E-15	0.362431	0.091	0.046	1.02E-10	Skin_T	PCNX
PSMB93	6.71E-15	0.269278	0.249	0.169	1.07E-10	Skin_T	PSMB9
CMC2	7.13E-15	0.512229	0.179	0.119	1.14E-10	Skin_T	CMC2
C1orf35	1.07E-14	0.465184	0.157	0.099	1.7E-10	Skin_T	C1orf35
IL4R	1.07E-14	0.26984	0.083	0.04	1.71E-10	Skin_T	IL4R
ITM2C2	1.44E-14	0.356894	0.186	0.12	2.3E-10	Skin_T	ITM2C
SNRPB1	1.96E-14	0.38531	0.394	0.333	3.13E-10	Skin_T	SNRPB
USMG51	1.97E-14	0.529331	0.477	0.436	3.15E-10	Skin_T	USMG5

TBCC1	2.72E-14	0.433609	0.154	0.095	4.34E-10	Skin_T	TBCC
CORO72	2.89E-14	0.280559	0.075	0.035	4.61E-10	Skin_T	CORO7
PDCD45	5.47E-14	0.685893	0.243	0.184	8.72E-10	Skin_T	PDCD4
TMEM123(	5.72E-14	0.418797	0.343	0.282	9.12E-10	Skin_T	TMEM123
PSMB81	6.64E-14	0.326412	0.263	0.191	1.06E-09	Skin_T	PSMB8
CCNI5	7.56E-14	0.25972	0.569	0.518	1.21E-09	Skin_T	CCNI
DOCK83	8.3E-14	0.299265	0.098	0.052	1.32E-09	Skin_T	DOCK8
CRLF3	1.03E-13	0.284354	0.082	0.041	1.65E-09	Skin_T	CRLF3
C19orf53	1.14E-13	0.402668	0.457	0.422	1.81E-09	Skin_T	C19orf53
CNBP	1.36E-13	0.3047	0.486	0.427	2.18E-09	Skin_T	CNBP
PFDN2	1.64E-13	0.445362	0.336	0.274	2.62E-09	Skin_T	PFDN2
BTG3	1.65E-13	0.528718	0.219	0.159	2.64E-09	Skin_T	BTG3
NFATC22	1.67E-13	0.303352	0.087	0.045	2.66E-09	Skin_T	NFATC2
SUMO1	1.71E-13	0.443871	0.391	0.347	2.73E-09	Skin_T	SUMO1
METTL12	2.33E-13	0.377553	0.09	0.047	3.72E-09	Skin_T	METTL12
HNRNPK	2.9E-13	0.380839	0.482	0.446	4.63E-09	Skin_T	HNRNPK
CASP82	3.18E-13	0.330471	0.08	0.04	5.08E-09	Skin_T	CASP8
RNF168	3.29E-13	0.475115	0.151	0.097	5.25E-09	Skin_T	RNF168
DHX363	3.97E-13	0.53786	0.24	0.181	6.34E-09	Skin_T	DHX36
AP3M2	4.15E-13	0.371404	0.084	0.043	6.62E-09	Skin_T	AP3M2
SACM1L	5.21E-13	0.332012	0.086	0.045	8.32E-09	Skin_T	SACM1L
MYADM5	6.14E-13	0.421998	0.213	0.15	9.8E-09	Skin_T	MYADM
SPNS3	6.39E-13	0.27946	0.076	0.037	1.02E-08	Skin_T	SPNS3
MDFIC2	7.4E-13	0.465959	0.125	0.076	1.18E-08	Skin_T	MDFIC
PHF12	1.18E-12	0.420377	0.17	0.115	1.87E-08	Skin_T	PHF1
RABGGTB	1.59E-12	0.482281	0.152	0.101	2.54E-08	Skin_T	RABGGTB
DCK	1.67E-12	0.323628	0.106	0.06	2.66E-08	Skin_T	DCK
TLK12	1.71E-12	0.467402	0.151	0.1	2.72E-08	Skin_T	TLK1
LRRFIP13	2.24E-12	0.407785	0.419	0.373	3.57E-08	Skin_T	LRRFIP1
ATG2A	2.47E-12	0.322512	0.083	0.044	3.94E-08	Skin_T	ATG2A
MRPS61	2.58E-12	0.527713	0.238	0.181	4.12E-08	Skin_T	MRPS6
PTP4A1	3.41E-12	0.431577	0.337	0.284	5.45E-08	Skin_T	PTP4A1
MOB1A2	3.82E-12	0.550412	0.238	0.186	6.09E-08	Skin_T	MOB1A
IVNS1ABP(	4.28E-12	0.513473	0.202	0.149	6.83E-08	Skin_T	IVNS1ABP
TOB2	4.94E-12	0.485289	0.151	0.1	7.88E-08	Skin_T	TOB2
CNIH1	5.64E-12	0.426818	0.184	0.129	9E-08	Skin_T	CNIH1
TAF1D1	6.54E-12	0.510831	0.279	0.227	1.04E-07	Skin_T	TAF1D
SEPT9	6.75E-12	0.342457	0.226	0.163	1.08E-07	Skin_T	SEPT9
ARL5B	1.15E-11	0.48881	0.148	0.098	1.83E-07	Skin_T	ARL5B
DRAP1	1.26E-11	0.318749	0.392	0.335	2.01E-07	Skin_T	DRAP1
PCMT11	1.28E-11	0.446274	0.272	0.217	2.05E-07	Skin_T	PCMT1
TANK1	2.06E-11	0.509077	0.179	0.127	3.29E-07	Skin_T	TANK
NXT1	3.19E-11	0.430823	0.207	0.153	5.09E-07	Skin_T	NXT1
HERPUD2	4.21E-11	0.403991	0.139	0.09	6.72E-07	Skin_T	HERPUD2
UBXN1	4.36E-11	0.284055	0.439	0.391	6.95E-07	Skin_T	UBXN1
CD821	5.83E-11	0.416782	0.157	0.108	9.3E-07	Skin_T	CD82
MSN4	7.26E-11	0.316045	0.215	0.156	1.16E-06	Skin_T	MSN

NDUFV21	7.99E-11	0.502432	0.409	0.386	1.27E-06	Skin_T	NDUFV2
YWHAH1	8.85E-11	0.437472	0.261	0.207	1.41E-06	Skin_T	YWHAH
SLC38A21	1.09E-10	0.316143	0.302	0.241	1.73E-06	Skin_T	SLC38A2
HSPA1B	1.51E-10	0.430013	0.374	0.31	2.41E-06	Skin_T	HSPA1B
TMED24	1.79E-10	0.344414	0.387	0.346	2.86E-06	Skin_T	TMED2
CBX63	1.92E-10	0.347227	0.13	0.084	3.06E-06	Skin_T	CBX6
PRPS1	2.41E-10	0.288005	0.102	0.061	3.84E-06	Skin_T	PRPS1
WHSC1L13	2.7E-10	0.532948	0.241	0.194	4.3E-06	Skin_T	WHSC1L1
HNRNPC	2.89E-10	0.361012	0.462	0.43	4.6E-06	Skin_T	HNRNPC
RBX13	4.35E-10	0.394793	0.382	0.349	6.94E-06	Skin_T	RBX1
YWHAB4	4.74E-10	0.354184	0.44	0.413	7.56E-06	Skin_T	YWHAB
HMGB2	5.8E-10	0.470321	0.295	0.246	9.25E-06	Skin_T	HMGB2
RHOC2	6.28E-10	0.353127	0.277	0.224	1E-05	Skin_T	RHOC
YWHAQ	7.05E-10	0.432026	0.393	0.365	1.12E-05	Skin_T	YWHAQ
NDUFA11	7.26E-10	0.419191	0.439	0.412	1.16E-05	Skin_T	NDUFA1
GSTK1	7.71E-10	0.291834	0.35	0.297	1.23E-05	Skin_T	GSTK1
TOB1	7.83E-10	0.383189	0.328	0.282	1.25E-05	Skin_T	TOB1
GLS3	8.24E-10	0.427466	0.165	0.117	1.31E-05	Skin_T	GLS
IFI163	8.32E-10	0.393807	0.253	0.198	1.33E-05	Skin_T	IFI16
ATXN11	9.09E-10	0.383358	0.113	0.072	1.45E-05	Skin_T	ATXN1
RBMX1	9.14E-10	0.407876	0.249	0.198	1.46E-05	Skin_T	RBMX
FAM102B1	1.11E-09	0.294867	0.058	0.03	1.78E-05	Skin_T	FAM102B
COX6C4	1.39E-09	0.261768	0.578	0.541	2.22E-05	Skin_T	COX6C
C2orf82	1.52E-09	0.369919	0.026	0.009	2.42E-05	Skin_T	C2orf82
KDSR4	1.63E-09	0.462031	0.175	0.129	2.6E-05	Skin_T	KDSR
UXT2	1.63E-09	0.262475	0.41	0.37	2.61E-05	Skin_T	UXT
CITED2	1.84E-09	0.367689	0.308	0.253	2.93E-05	Skin_T	CITED2
TBPL1	1.96E-09	0.399457	0.135	0.091	3.12E-05	Skin_T	TBPL1
PAFAH1B2	2.49E-09	0.53184	0.17	0.126	3.98E-05	Skin_T	PAFAH1B2
TPD525	2.51E-09	0.461199	0.17	0.127	4E-05	Skin_T	TPD52
CSGALNAC	3.17E-09	0.279346	0.072	0.04	5.05E-05	Skin_T	CSGALNACT2
PLEKHA21	3.62E-09	0.304292	0.086	0.051	5.77E-05	Skin_T	PLEKHA2
NCK11	3.83E-09	0.397157	0.152	0.108	6.1E-05	Skin_T	NCK1
MTFP1	4.7E-09	0.252208	0.069	0.038	7.49E-05	Skin_T	MTFP1
TRAM11	5.31E-09	0.414879	0.332	0.3	8.46E-05	Skin_T	TRAM1
RBPJ3	5.71E-09	0.524478	0.235	0.192	9.11E-05	Skin_T	RBPJ
ARL6IP6	5.72E-09	0.303727	0.111	0.072	9.12E-05	Skin_T	ARL6IP6
RSBN1	5.81E-09	0.366143	0.152	0.107	9.27E-05	Skin_T	RSBN1
CARD82	7.16E-09	0.254729	0.09	0.054	0.000114	Skin_T	CARD8
TGFBR33	8.88E-09	0.337798	0.134	0.091	0.000142	Skin_T	TGFBR3
CELF23	1.09E-08	0.313842	0.182	0.133	0.000175	Skin_T	CELF2
MANF2	1.12E-08	0.374142	0.274	0.234	0.000179	Skin_T	MANF
PLIN2	1.23E-08	0.44111	0.17	0.126	0.000196	Skin_T	PLIN2
IDS3	1.5E-08	0.466011	0.227	0.185	0.00024	Skin_T	IDS
SEPT75	1.77E-08	0.251605	0.385	0.339	0.000283	Skin_T	SEPT7
RAP2C	1.81E-08	0.344315	0.086	0.053	0.000288	Skin_T	RAP2C
NACA21	2.04E-08	0.275961	0.111	0.072	0.000325	Skin_T	NACA2

TBCA2	2.54E-08	0.334234	0.38	0.351	0.000405	Skin_T	TBCA
NEDD82	2.65E-08	0.296976	0.448	0.423	0.000423	Skin_T	NEDD8
ARID4B1	3.47E-08	0.479553	0.278	0.242	0.000553	Skin_T	ARID4B
EIF5A3	3.63E-08	0.473049	0.308	0.279	0.000579	Skin_T	EIF5A
HNRNPF	5.71E-08	0.395167	0.337	0.308	0.000911	Skin_T	HNRNPF
ATXN7	5.72E-08	0.33734	0.102	0.067	0.000913	Skin_T	ATXN7
BUB31	7.42E-08	0.418422	0.216	0.175	0.001183	Skin_T	BUB3
EED	7.48E-08	0.283865	0.07	0.041	0.001193	Skin_T	EED
VAPA	1.1E-07	0.324398	0.417	0.4	0.001762	Skin_T	VAPA
C4orf321	1.11E-07	0.362644	0.118	0.081	0.001774	Skin_T	C4orf32
SRSF114	1.8E-07	0.402782	0.33	0.303	0.002863	Skin_T	SRSF11
SF13	1.97E-07	0.370031	0.27	0.234	0.003142	Skin_T	SF1
CYSTM13	2.13E-07	0.343813	0.269	0.226	0.00339	Skin_T	CYSTM1
POLR3E	2.45E-07	0.338902	0.08	0.05	0.003908	Skin_T	POLR3E
CDC42EP3:	2.53E-07	0.325388	0.133	0.095	0.004038	Skin_T	CDC42EP3
NECAP11	2.75E-07	0.316807	0.09	0.058	0.004383	Skin_T	NECAP1
CDC371	2.93E-07	0.40565	0.303	0.274	0.004679	Skin_T	CDC37
CCL20	2.98E-07	0.942515	0.05	0.027	0.004759	Skin_T	CCL20
COX141	3.43E-07	0.41268	0.265	0.232	0.005473	Skin_T	COX14
TBC1D10A	3.77E-07	0.273679	0.093	0.061	0.006009	Skin_T	TBC1D10A
CHST121	4.92E-07	0.316966	0.117	0.081	0.007841	Skin_T	CHST12
POLR2K1	5.36E-07	0.444065	0.312	0.293	0.008556	Skin_T	POLR2K
UBE2I2	6.22E-07	0.359509	0.292	0.263	0.009917	Skin_T	UBE2I
ACTR31	7.03E-07	0.375757	0.372	0.356	0.011218	Skin_T	ACTR3
RAD9A1	8.06E-07	0.305758	0.086	0.056	0.012854	Skin_T	RAD9A
NDEL1	8.19E-07	0.359025	0.114	0.08	0.01307	Skin_T	NDEL1
CYTH1	8.91E-07	0.371947	0.179	0.141	0.014203	Skin_T	CYTH1
RANBP1	1.06E-06	0.313798	0.303	0.267	0.016912	Skin_T	RANBP1
MAPRE21	1.07E-06	0.41135	0.111	0.079	0.017114	Skin_T	MAPRE2
LINC00152	1.08E-06	0.2984	0.221	0.178	0.017209	Skin_T	LINC00152
ZMYM21	1.26E-06	0.48132	0.165	0.132	0.020141	Skin_T	ZMYM2
PFKFB3	1.27E-06	0.31219	0.131	0.096	0.020329	Skin_T	PFKFB3
JOSD1	1.51E-06	0.406772	0.16	0.125	0.024086	Skin_T	JOSD1
DYNLT3	1.61E-06	0.330031	0.164	0.126	0.025609	Skin_T	DYNLT3
NKTR10	2.16E-06	0.40416	0.21	0.176	0.034401	Skin_T	NKTR
TUBB2A1	2.53E-06	0.409833	0.147	0.113	0.040345	Skin_T	TUBB2A
ACOT7	2.6E-06	0.271133	0.074	0.047	0.041451	Skin_T	ACOT7
SLBP	2.86E-06	0.383482	0.179	0.145	0.045608	Skin_T	SLBP
CPNE12	3.04E-06	0.343304	0.164	0.128	0.048463	Skin_T	CPNE1
HPRT1	3.07E-06	0.337227	0.124	0.091	0.048943	Skin_T	HPRT1
DNAJC9	3.67E-06	0.337296	0.104	0.073	0.058526	Skin_T	DNAJC9
COX201	3.73E-06	0.422767	0.197	0.166	0.059475	Skin_T	COX20
MTF2	3.81E-06	0.353969	0.119	0.088	0.060764	Skin_T	MTF2
GTPBP1	3.99E-06	0.315166	0.11	0.079	0.063627	Skin_T	GTPBP1
DNTTIP22	4.35E-06	0.392396	0.139	0.107	0.069374	Skin_T	DNTTIP2
NCL5	5.33E-06	0.294482	0.305	0.271	0.085079	Skin_T	NCL
SLC20A1	5.74E-06	0.357612	0.103	0.073	0.091547	Skin_T	SLC20A1

NFE2L2	6.54E-06	0.421486	0.306	0.288	0.104264	Skin_T	NFE2L2
PSMD5.AS	6.58E-06	0.260912	0.091	0.063	0.10487	Skin_T	PSMD5.AS1
PIM31	6.64E-06	0.3827	0.315	0.291	0.105876	Skin_T	PIM3
RNF2133	6.69E-06	0.372971	0.18	0.147	0.10673	Skin_T	RNF213
USP151	7.03E-06	0.415488	0.185	0.155	0.112174	Skin_T	USP15
RNF149	7.04E-06	0.440313	0.188	0.159	0.112256	Skin_T	RNF149
PPP3CA3	7.81E-06	0.393833	0.188	0.155	0.124511	Skin_T	PPP3CA
RALGAPA1	8.35E-06	0.347845	0.104	0.075	0.133178	Skin_T	RALGAPA1
KCTD20	1.11E-05	0.344501	0.106	0.077	0.177066	Skin_T	KCTD20
HSPD13	1.25E-05	0.363213	0.284	0.258	0.199468	Skin_T	HSPD1
SDF41	1.27E-05	0.375517	0.235	0.209	0.202752	Skin_T	SDF4
ZNRD11	1.27E-05	0.303183	0.172	0.141	0.202972	Skin_T	ZNRD1
TBCB1	1.39E-05	0.308246	0.235	0.202	0.222319	Skin_T	TBCB
SLC4A72	1.43E-05	0.279283	0.086	0.059	0.227887	Skin_T	SLC4A7
C14orf2	1.45E-05	0.310343	0.451	0.449	0.230805	Skin_T	C14orf2
PRKCH1	1.54E-05	0.298194	0.117	0.087	0.245957	Skin_T	PRKCH
TNRC6B6	1.57E-05	0.478698	0.2	0.172	0.250679	Skin_T	TNRC6B
TMEM120I	1.59E-05	0.29825	0.067	0.044	0.253202	Skin_T	TMEM120B
MAPRE1	1.94E-05	0.421847	0.202	0.175	0.309755	Skin_T	MAPRE1
PHF201	2.14E-05	0.406434	0.175	0.145	0.341478	Skin_T	PHF20
CCL32	2.48E-05	0.253364	0.074	0.048	0.395232	Skin_T	CCL3
MEAF6	2.95E-05	0.383071	0.249	0.225	0.470747	Skin_T	MEAF6
ATF41	2.96E-05	0.341676	0.35	0.338	0.472851	Skin_T	ATF4
C20orf24	3.28E-05	0.276067	0.187	0.155	0.523664	Skin_T	C20orf24
SIK3	3.76E-05	0.359564	0.095	0.07	0.600026	Skin_T	SIK3
EHD42	4E-05	0.280399	0.119	0.091	0.638089	Skin_T	EHD4
WSB14	4.03E-05	0.358149	0.273	0.25	0.642763	Skin_T	WSB1
TIPARP	4.04E-05	0.33679	0.23	0.202	0.644874	Skin_T	TIPARP
SAR1A	4.11E-05	0.450886	0.25	0.233	0.655427	Skin_T	SAR1A
ZNF267	4.64E-05	0.348339	0.104	0.077	0.740181	Skin_T	ZNF267
GADD45G	4.81E-05	0.281685	0.094	0.067	0.766856	Skin_T	GADD45G
PNMA1	6E-05	0.265073	0.074	0.051	0.957632	Skin_T	PNMA1
RNF5	6.94E-05	0.385505	0.17	0.143	1	Skin_T	RNF5
EMD	7.04E-05	0.369844	0.152	0.125	1	Skin_T	EMD
RBM63	8.48E-05	0.357581	0.138	0.111	1	Skin_T	RBM6
PGM2L11	8.67E-05	0.25486	0.066	0.044	1	Skin_T	PGM2L1
HNRNPUL1	9.58E-05	0.352757	0.188	0.163	1	Skin_T	HNRNPUL1
LMO46	9.7E-05	0.415701	0.189	0.165	1	Skin_T	LMO4
B3GNT2	0.000101	0.285631	0.094	0.069	1	Skin_T	B3GNT2
GNAI31	0.000124	0.414041	0.212	0.192	1	Skin_T	GNAI3
CHRAC1	0.00013	0.294026	0.097	0.073	1	Skin_T	CHRAC1
CBWD2	0.000139	0.279142	0.072	0.05	1	Skin_T	CBWD2
IRF2BP21	0.000146	0.405435	0.272	0.259	1	Skin_T	IRF2BP2
CBLB1	0.000153	0.277213	0.077	0.055	1	Skin_T	CBLB
ISCA1	0.000162	0.271094	0.168	0.141	1	Skin_T	ISCA1
FAM49B2	0.000185	0.349946	0.198	0.173	1	Skin_T	FAM49B
ZC3H12A	0.000186	0.354001	0.128	0.101	1	Skin_T	ZC3H12A

ZNF394	0.000197	0.308846	0.11	0.086	1	Skin_T	ZNF394
MAP3K8	0.000208	0.321695	0.143	0.116	1	Skin_T	MAP3K8
SRRM13	0.000308	0.351382	0.33	0.328	1	Skin_T	SRRM1
PMF1	0.000313	0.328346	0.152	0.128	1	Skin_T	PMF1
NEK7	0.000323	0.321886	0.102	0.079	1	Skin_T	NEK7
MYH93	0.000358	0.295904	0.209	0.187	1	Skin_T	MYH9
MED30	0.000361	0.287829	0.124	0.1	1	Skin_T	MED30
TRABD1	0.000413	0.313747	0.142	0.117	1	Skin_T	TRABD
CKS1B1	0.000465	0.364492	0.163	0.14	1	Skin_T	CKS1B
RBM257	0.000559	0.311494	0.256	0.24	1	Skin_T	RBM25
ASXL2	0.0006	0.289254	0.079	0.059	1	Skin_T	ASXL2
NDUFA123	0.000641	0.306305	0.283	0.275	1	Skin_T	NDUFA12
KDM6B	0.000715	0.307316	0.142	0.118	1	Skin_T	KDM6B
NOP58	0.000819	0.316527	0.137	0.115	1	Skin_T	NOP58
USP35	0.000922	0.329732	0.133	0.112	1	Skin_T	USP3
DEDD2	0.000964	0.325614	0.144	0.122	1	Skin_T	DEDD2
PGAM13	0.001022	0.351973	0.241	0.229	1	Skin_T	PGAM1
ZNF1311	0.00119	0.295739	0.104	0.083	1	Skin_T	ZNF131
WDR332	0.00119	0.345194	0.17	0.152	1	Skin_T	WDR33
EIF1AY2	0.001261	0.306426	0.107	0.087	1	Skin_T	EIF1AY
LYRM4	0.001293	0.311524	0.134	0.113	1	Skin_T	LYRM4
STAT34	0.001319	0.300283	0.211	0.191	1	Skin_T	STAT3
CSNK1D	0.001417	0.367175	0.197	0.181	1	Skin_T	CSNK1D
GCLM	0.001437	0.274989	0.084	0.065	1	Skin_T	GCLM
MRPL1	0.001438	0.30315	0.092	0.073	1	Skin_T	MRPL1
KLHL28	0.001449	0.274178	0.075	0.056	1	Skin_T	KLHL28
SRSF62	0.001576	0.433469	0.162	0.145	1	Skin_T	SRSF6
DNAJB143	0.001649	0.320652	0.123	0.103	1	Skin_T	DNAJB14
DIAPH1	0.001814	0.324107	0.172	0.153	1	Skin_T	DIAPH1
UBE2S2	0.001922	0.338217	0.151	0.132	1	Skin_T	UBE2S
LINC00969	0.002161	0.31646	0.131	0.111	1	Skin_T	LINC00969
GLO1	0.002167	0.343654	0.156	0.137	1	Skin_T	GLO1
TIMM10	0.002173	0.319687	0.173	0.155	1	Skin_T	TIMM10
CBFB	0.002245	0.319544	0.112	0.093	1	Skin_T	CBFB
SC5D	0.002264	0.279939	0.067	0.051	1	Skin_T	SC5D
AKAP133	0.002375	0.332069	0.23	0.217	1	Skin_T	AKAP13
RASGEF1B:	0.002389	0.300966	0.084	0.065	1	Skin_T	RASGEF1B
PQBP11	0.002392	0.272443	0.174	0.154	1	Skin_T	PQBP1
OGT2	0.002542	0.345814	0.125	0.106	1	Skin_T	OGT
POLE3	0.002728	0.262587	0.115	0.095	1	Skin_T	POLE3
ATP5B4	0.002751	0.365179	0.36	0.372	1	Skin_T	ATP5B
IRF2BPL1	0.003654	0.300222	0.095	0.076	1	Skin_T	IRF2BPL
AKIRIN2	0.003688	0.45292	0.192	0.182	1	Skin_T	AKIRIN2
CCNT1	0.003863	0.307096	0.086	0.069	1	Skin_T	CCNT1
LNPEP5	0.004464	0.254556	0.085	0.067	1	Skin_T	LNPEP
ASXL1	0.004722	0.30219	0.096	0.079	1	Skin_T	ASXL1
EIF4A3	0.004971	0.258328	0.212	0.199	1	Skin_T	EIF4A3

YAF2	0.006754	0.291831	0.114	0.098	1	Skin_T	YAF2
GSPT11	0.007672	0.361776	0.184	0.175	1	Skin_T	GSPT1
MED28	0.007963	0.348065	0.167	0.155	1	Skin_T	MED28
EIF1B1	0.008247	0.251165	0.258	0.25	1	Skin_T	EIF1B
RPS6KA34	0.008258	0.279839	0.112	0.096	1	Skin_T	RPS6KA3
AKAP17A1	0.00841	0.28308	0.11	0.094	1	Skin_T	AKAP17A
VPS291	0.009356	0.350761	0.276	0.282	1	Skin_T	VPS29
PDE4D1	0.009761	0.327854	0.071	0.057	1	Skin_T	PDE4D
HLA.DRA2	0	4.336277	0.995	0.232	0	Skin_Macr	HLA.DRA
HLA.DPB11	0	4.185871	0.985	0.198	0	Skin_Macr	HLA.DPB1
INSIG11	0	4.051891	0.851	0.191	0	Skin_Macr	INSIG1
G0S2	0	3.943333	0.702	0.107	0	Skin_Macr	G0S2
HLA.DRB12	0	3.889561	0.989	0.198	0	Skin_Macr	HLA.DRB1
HLA.DPA12	0	3.867613	0.986	0.171	0	Skin_Macr	HLA.DPA1
HLA.DQB11	0	3.839174	0.959	0.109	0	Skin_Macr	HLA.DQB1
HLA.DQA1	0	3.827694	0.956	0.085	0	Skin_Macr	HLA.DQA1
GPR1833	0	3.789578	0.853	0.055	0	Skin_Macr	GPR183
SRGN4	0	3.719809	0.955	0.204	0	Skin_Macr	SRGN
CD742	0	3.606881	0.996	0.346	0	Skin_Macr	CD74
FCER1A1	0	3.468638	0.705	0.024	0	Skin_Macr	FCER1A
CST33	0	3.378907	0.991	0.583	0	Skin_Macr	CST3
SERPINB91	0	3.375262	0.693	0.045	0	Skin_Macr	SERPINB9
HLA.DRB51	0	3.23349	0.906	0.116	0	Skin_Macr	HLA.DRB5
REL2	0	3.126232	0.844	0.169	0	Skin_Macr	REL
PLAUR1	0	3.031335	0.806	0.101	0	Skin_Macr	PLAUR
CD831	0	2.934406	0.75	0.079	0	Skin_Macr	CD83
IL1B1	0	2.884082	0.554	0.029	0	Skin_Macr	IL1B
IL1R2	0	2.872845	0.686	0.024	0	Skin_Macr	IL1R2
CXCL3	0	2.76494	0.524	0.124	0	Skin_Macr	CXCL3
SGK11	0	2.699251	0.632	0.099	0	Skin_Macr	SGK1
ID21	0	2.581349	0.758	0.241	0	Skin_Macr	ID2
LGALS21	0	2.560806	0.704	0.027	0	Skin_Macr	LGALS2
HLA.DQB21	0	2.525707	0.553	0.012	0	Skin_Macr	HLA.DQB2
TMSB4X3	0	2.403276	1	0.868	0	Skin_Macr	TMSB4X
CLEC10A1	0	2.379823	0.592	0.015	0	Skin_Macr	CLEC10A
HLA.DMA2	0	2.379619	0.823	0.122	0	Skin_Macr	HLA.DMA
RGS15	0	2.353868	0.513	0.058	0	Skin_Macr	RGS1
BIRC32	0	2.345412	0.713	0.139	0	Skin_Macr	BIRC3
HLA.DQA2	0	2.336847	0.564	0.061	0	Skin_Macr	HLA.DQA2
MMP9	0	2.289059	0.383	0.018	0	Skin_Macr	MMP9
CXCL2	0	2.27637	0.624	0.151	0	Skin_Macr	CXCL2
LST11	0	2.198771	0.779	0.08	0	Skin_Macr	LST1
LPAR64	0	2.171896	0.621	0.129	0	Skin_Macr	LPAR6
CD1E	0	2.15397	0.445	0.005	0	Skin_Macr	CD1E
CTSZ6	0	2.146929	0.759	0.177	0	Skin_Macr	CTSZ
FCER1G1	0	2.107444	0.821	0.092	0	Skin_Macr	FCER1G
CMTM61	0	2.019437	0.737	0.214	0	Skin_Macr	CMTM6

NINJ11	0	2.002718	0.625	0.133	0	Skin_Macr NINJ1
BID1	0	1.989492	0.607	0.108	0	Skin_Macr BID
CD443	0	1.987688	0.876	0.335	0	Skin_Macr CD44
GNA131	0	1.977804	0.572	0.072	0	Skin_Macr GNA13
AIF11	0	1.96939	0.764	0.098	0	Skin_Macr AIF1
NFKB1	0	1.952336	0.596	0.107	0	Skin_Macr NFKB1
HSPA1A5	0	1.950088	0.72	0.316	0	Skin_Macr HSPA1A
CD1C1	0	1.923181	0.447	0.01	0	Skin_Macr CD1C
TYROBP1	0	1.906625	0.89	0.104	0	Skin_Macr TYROBP
BCL2A11	0	1.902771	0.429	0.025	0	Skin_Macr BCL2A1
CREM1	0	1.857434	0.673	0.186	0	Skin_Macr CREM
SELK2	0	1.840886	0.798	0.461	0	Skin_Macr SELK
BASP12	0	1.821196	0.498	0.073	0	Skin_Macr BASP1
TIMP16	0	1.801649	0.781	0.372	0	Skin_Macr TIMP1
DUSP27	0	1.798294	0.613	0.113	0	Skin_Macr DUSP2
STK44	0	1.793155	0.591	0.101	0	Skin_Macr STK4
CSF2RA1	0	1.76611	0.489	0.016	0	Skin_Macr CSF2RA
EMP35	0	1.762243	0.906	0.337	0	Skin_Macr EMP3
SPI11	0	1.757712	0.574	0.049	0	Skin_Macr SPI1
NFKBIA1	0	1.755838	0.951	0.505	0	Skin_Macr NFKBIA
NFKBID3	0	1.70131	0.48	0.033	0	Skin_Macr NFKBID
RGS23	0	1.699791	0.445	0.088	0	Skin_Macr RGS2
ATP13A3	0	1.68408	0.464	0.084	0	Skin_Macr ATP13A3
CCL33	0	1.670988	0.237	0.043	0	Skin_Macr CCL3
AP1S22	0	1.657395	0.603	0.113	0	Skin_Macr AP1S2
LGALS16	0	1.649715	0.982	0.396	0	Skin_Macr LGALS1
MS4A6A1	0	1.630257	0.479	0.036	0	Skin_Macr MS4A6A
PLEK1	0	1.620865	0.467	0.044	0	Skin_Macr PLEK
ETV31	0	1.619935	0.431	0.039	0	Skin_Macr ETV3
COTL13	0	1.607067	0.711	0.171	0	Skin_Macr COTL1
CPVL1	0	1.606386	0.418	0.06	0	Skin_Macr CPVL
NR4A31	0	1.599259	0.391	0.045	0	Skin_Macr NR4A3
CCR7	0	1.590117	0.315	0.038	0	Skin_Macr CCR7
S100A45	0	1.586863	0.967	0.452	0	Skin_Macr S100A4
FGL22	0	1.583982	0.537	0.087	0	Skin_Macr FGL2
NR4A23	0	1.57555	0.592	0.175	0	Skin_Macr NR4A2
H2AFZ1	0	1.570859	0.812	0.43	0	Skin_Macr H2AFZ
FTH1	0	1.542088	0.999	0.935	0	Skin_Macr FTH1
TRAF11	0	1.541313	0.382	0.016	0	Skin_Macr TRAF1
FCGR2A1	0	1.540193	0.399	0.031	0	Skin_Macr FCGR2A
CYTIP3	0	1.537364	0.515	0.073	0	Skin_Macr CYTIP
ATP1B32	0	1.513373	0.585	0.228	0	Skin_Macr ATP1B3
GPR157	0	1.507417	0.327	0.018	0	Skin_Macr GPR157
FAM49A1	0	1.490195	0.384	0.032	0	Skin_Macr FAM49A
SLC2A32	0	1.477486	0.575	0.118	0	Skin_Macr SLC2A3
HLA.DMB2	0	1.469765	0.49	0.056	0	Skin_Macr HLA.DMB
IL4I1	0	1.456054	0.313	0.027	0	Skin_Macr IL4I1

PTPRE3	0	1.454049	0.429	0.044	0	Skin_Macr PTPRE
PNRC11	0	1.448946	0.873	0.502	0	Skin_Macr PNRC1
EZR3	0	1.446211	0.654	0.273	0	Skin_Macr EZR
IRF81	0	1.445199	0.345	0.034	0	Skin_Macr IRF8
RPL411	0	1.427633	1	0.858	0	Skin_Macr RPL41
CST72	0	1.419592	0.506	0.051	0	Skin_Macr CST7
FNIP21	0	1.415939	0.376	0.083	0	Skin_Macr FNIP2
RASGEF1B:	0	1.412673	0.298	0.059	0	Skin_Macr RASGEF1B
KDM6B1	0	1.406154	0.472	0.109	0	Skin_Macr KDM6B
GLIPR13	0	1.399521	0.46	0.08	0	Skin_Macr GLIPR1
PTGS21	0	1.399081	0.314	0.033	0	Skin_Macr PTGS2
PTTG11	0	1.38976	0.413	0.075	0	Skin_Macr PTTG1
FPR31	0	1.389687	0.325	0.01	0	Skin_Macr FPR3
CLEC7A1	0	1.385346	0.379	0.019	0	Skin_Macr CLEC7A
SAT1	0	1.358754	0.962	0.603	0	Skin_Macr SAT1
ZNF901	0	1.354694	0.453	0.086	0	Skin_Macr ZNF90
BTG12	0	1.346463	0.866	0.57	0	Skin_Macr BTG1
LMNA8	0	1.343119	0.824	0.354	0	Skin_Macr LMNA
FABP53	0	1.33734	0.469	0.127	0	Skin_Macr FABP5
ATP2B14	0	1.337196	0.497	0.142	0	Skin_Macr ATP2B1
RPL3910	0	1.330415	0.993	0.669	0	Skin_Macr RPL39
SAMSN12	0	1.328665	0.342	0.038	0	Skin_Macr SAMSN1
ARL5B1	0	1.325761	0.351	0.092	0	Skin_Macr ARL5B
NBEAL11	0	1.323702	0.809	0.307	0	Skin_Macr NBEAL1
FNBP14	0	1.322025	0.486	0.117	0	Skin_Macr FNBP1
MARCKS4	0	1.275697	0.606	0.275	0	Skin_Macr MARCKS
CFLAR3	0	1.273787	0.606	0.263	0	Skin_Macr CFLAR
TNFRSF1B4	0	1.26491	0.358	0.04	0	Skin_Macr TNFRSF1B
EIF4A11	0	1.258568	0.801	0.341	0	Skin_Macr EIF4A1
FTL2	0	1.251429	0.998	0.893	0	Skin_Macr FTL
BCAT11	0	1.250378	0.356	0.065	0	Skin_Macr BCAT1
OTUD1	0	1.250096	0.321	0.072	0	Skin_Macr OTUD1
TUBA1B5	0	1.238131	0.713	0.345	0	Skin_Macr TUBA1B
RPS292	0	1.237224	0.994	0.785	0	Skin_Macr RPS29
SDCBP2	0	1.230103	0.735	0.404	0	Skin_Macr SDCBP
PKIB1	0	1.229637	0.314	0.05	0	Skin_Macr PKIB
VMO11	0	1.228771	0.259	0.025	0	Skin_Macr VMO1
PPIF	0	1.225622	0.372	0.096	0	Skin_Macr PPIF
FCGR2B2	0	1.225264	0.313	0.022	0	Skin_Macr FCGR2B
GPBP11	0	1.217036	0.642	0.313	0	Skin_Macr GPBP1
MCL13	0	1.211361	0.689	0.373	0	Skin_Macr MCL1
CLIC14	0	1.205558	0.853	0.465	0	Skin_Macr CLIC1
RBPJ4	0	1.202512	0.521	0.183	0	Skin_Macr RBPJ
BEST11	0	1.199246	0.344	0.035	0	Skin_Macr BEST1
CKS21	0	1.185274	0.431	0.135	0	Skin_Macr CKS2
TNFAIP84	0	1.183869	0.414	0.097	0	Skin_Macr TNFAIP8
STX111	0	1.179563	0.365	0.058	0	Skin_Macr STX11

PTMA2	0	1.175328	0.998	0.815	0	Skin_Macr PTMA
OPN3	0	1.169073	0.281	0.048	0	Skin_Macr OPN3
ZEB23	0	1.154741	0.399	0.081	0	Skin_Macr ZEB2
TNFSF13B2	0	1.151321	0.326	0.035	0	Skin_Macr TNFSF13B
MNDA1	0	1.15035	0.255	0.022	0	Skin_Macr MNDA
NFE2L21	0	1.14477	0.586	0.28	0	Skin_Macr NFE2L2
ARL4C4	0	1.135577	0.529	0.18	0	Skin_Macr ARL4C
C1orf543	0	1.134837	0.274	0.042	0	Skin_Macr C1orf54
NAMPT	0	1.123229	0.752	0.372	0	Skin_Macr NAMPT
F13A11	0	1.121487	0.265	0.026	0	Skin_Macr F13A1
CARD161	0	1.09519	0.397	0.088	0	Skin_Macr CARD16
RP11.138A	0	1.085098	0.282	0.04	0	Skin_Macr RP11.138A9.2
CD681	0	1.082888	0.432	0.111	0	Skin_Macr CD68
PTGIR1	0	1.063337	0.271	0.014	0	Skin_Macr PTGIR
PPT11	0	1.062093	0.412	0.122	0	Skin_Macr PPT1
CCND23	0	1.057203	0.338	0.096	0	Skin_Macr CCND2
ATP5E3	0	1.044366	0.953	0.677	0	Skin_Macr ATP5E
PID11	0	1.037383	0.295	0.031	0	Skin_Macr PID1
TNFAIP31	0	1.02587	0.472	0.153	0	Skin_Macr TNFAIP3
PDE4A1	0	1.025818	0.299	0.045	0	Skin_Macr PDE4A
RNF144B1	0	1.022592	0.326	0.082	0	Skin_Macr RNF144B
C1QA1	0	1.01082	0.285	0.034	0	Skin_Macr C1QA
MTHFD22	0	1.009813	0.336	0.093	0	Skin_Macr MTHFD2
PDE4B2	0	1.005727	0.273	0.045	0	Skin_Macr PDE4B
CALM24	0	1.003332	0.893	0.554	0	Skin_Macr CALM2
TGFBI3	0	1.000398	0.341	0.056	0	Skin_Macr TGFBI
ALOX5AP3	0	0.998456	0.419	0.082	0	Skin_Macr ALOX5AP
CDC42EP3	0	0.991045	0.323	0.089	0	Skin_Macr CDC42EP3
ACTB2	0	0.989076	0.989	0.869	0	Skin_Macr ACTB
YBX11	0	0.988932	0.93	0.58	0	Skin_Macr YBX1
GLA1	0	0.982191	0.301	0.083	0	Skin_Macr GLA
CD1A	0	0.970224	0.193	0.002	0	Skin_Macr CD1A
H3F3A13	0	0.963411	0.906	0.467	0	Skin_Macr H3F3A
RPS4Y14	0	0.958138	0.667	0.263	0	Skin_Macr RPS4Y1
DOK23	0	0.956521	0.353	0.055	0	Skin_Macr DOK2
CD1B	0	0.955946	0.187	0.001	0	Skin_Macr CD1B
KYNU	0	0.955384	0.276	0.059	0	Skin_Macr KYNU
ARID3A2	0	0.946139	0.236	0.038	0	Skin_Macr ARID3A
AHR3	0	0.940153	0.355	0.102	0	Skin_Macr AHR
VEGFA1	0	0.938823	0.293	0.079	0	Skin_Macr VEGFA
CD861	0	0.938237	0.245	0.017	0	Skin_Macr CD86
C1QC1	0	0.934932	0.213	0.025	0	Skin_Macr C1QC
OAZ11	0	0.930483	0.959	0.778	0	Skin_Macr OAZ1
RHOG2	0	0.914243	0.475	0.179	0	Skin_Macr RHOG
CXCL161	0	0.901481	0.468	0.134	0	Skin_Macr CXCL16
LPXN3	0	0.901037	0.222	0.043	0	Skin_Macr LPXN
RNASE61	0	0.896821	0.273	0.034	0	Skin_Macr RNASE6

CXCR42	0	0.892948	0.444	0.115	0	Skin_Macr CXCR4
CD581	0	0.889831	0.291	0.073	0	Skin_Macr CD58
LCP13	0	0.888151	0.4	0.1	0	Skin_Macr LCP1
IL1R12	0	0.885518	0.287	0.07	0	Skin_Macr IL1R1
RAB311	0	0.885183	0.323	0.088	0	Skin_Macr RAB31
RHOF1	0	0.874633	0.324	0.076	0	Skin_Macr RHOF
SOD2	0	0.869398	0.738	0.402	0	Skin_Macr SOD2
ST8SIA43	0	0.869026	0.233	0.023	0	Skin_Macr ST8SIA4
GLIPR24	0	0.868376	0.316	0.079	0	Skin_Macr GLIPR2
RPS272	0	0.862835	1	0.892	0	Skin_Macr RPS27
TMSB102	0	0.862575	0.998	0.876	0	Skin_Macr TMSB10
NRP22	0	0.858518	0.21	0.026	0	Skin_Macr NRP2
S100A105	0	0.852136	0.966	0.668	0	Skin_Macr S100A10
S100A63	0	0.849934	0.992	0.706	0	Skin_Macr S100A6
SH3BGRL3:	0	0.846421	0.911	0.625	0	Skin_Macr SH3BGRL3
SLC25A19	0	0.833056	0.231	0.029	0	Skin_Macr SLC25A19
C5AR11	0	0.817762	0.174	0.015	0	Skin_Macr C5AR1
RPL231	0	0.81017	0.936	0.677	0	Skin_Macr RPL23
MTRNR2L8	0	0.807812	0.664	0.277	0	Skin_Macr MTRNR2L8
ENTPD14	0	0.806532	0.25	0.041	0	Skin_Macr ENTPD1
IRF41	0	0.794367	0.167	0.008	0	Skin_Macr IRF4
C9orf721	0	0.792552	0.22	0.043	0	Skin_Macr C9orf72
PFN11	0	0.791854	0.942	0.576	0	Skin_Macr PFN1
TNFRSF42	0	0.788712	0.163	0.025	0	Skin_Macr TNFRSF4
LDLRAD41	0	0.788112	0.242	0.049	0	Skin_Macr LDLRAD4
CLEC4A1	0	0.783053	0.208	0.014	0	Skin_Macr CLEC4A
HCST3	0	0.777573	0.589	0.162	0	Skin_Macr HCST
CSGALNAC	0	0.770712	0.214	0.036	0	Skin_Macr CSGALNACT2
IFI301	0	0.764167	0.217	0.014	0	Skin_Macr IFI30
RPL342	0	0.758848	0.999	0.917	0	Skin_Macr RPL34
MMP19	0	0.757836	0.129	0.013	0	Skin_Macr MMP19
RP11.138A	0	0.75367	0.216	0.043	0	Skin_Macr RP11.138A9.1
SH2B32	0	0.748593	0.216	0.025	0	Skin_Macr SH2B3
HCAR2	0	0.744728	0.17	0.017	0	Skin_Macr HCAR2
TYMP2	0	0.712943	0.483	0.178	0	Skin_Macr TYMP
SNX101	0	0.699897	0.227	0.049	0	Skin_Macr SNX10
MCOLN2	0	0.697539	0.169	0.011	0	Skin_Macr MCOLN2
CFP1	0	0.682739	0.24	0.022	0	Skin_Macr CFP
CLIC22	0	0.682445	0.218	0.043	0	Skin_Macr CLIC2
VIM8	0	0.676768	0.954	0.482	0	Skin_Macr VIM
RUNX33	0	0.666705	0.205	0.028	0	Skin_Macr RUNX3
NCF21	0	0.65043	0.207	0.019	0	Skin_Macr NCF2
DNASE1L3:	0	0.649101	0.121	0.011	0	Skin_Macr DNASE1L3
ADAM82	0	0.628866	0.208	0.024	0	Skin_Macr ADAM8
VSIG41	0	0.622253	0.165	0.023	0	Skin_Macr VSIG4
IGSF61	0	0.605392	0.209	0.026	0	Skin_Macr IGSF6
EMILIN22	0	0.597284	0.17	0.027	0	Skin_Macr EMILIN2

IL10RA4	0	0.591584	0.229	0.04	0	Skin_Macr	IL10RA
EVI2B4	0	0.586584	0.264	0.059	0	Skin_Macr	EVI2B
MB21D2	0	0.586519	0.155	0.022	0	Skin_Macr	MB21D2
LAPTM53	0	0.581885	0.521	0.164	0	Skin_Macr	LAPTM5
CECR12	0	0.58178	0.191	0.036	0	Skin_Macr	CECR1
EVI2A4	0	0.538596	0.188	0.034	0	Skin_Macr	EVI2A
ITGAX1	0	0.537605	0.132	0.012	0	Skin_Macr	ITGAX
CLEC5A	0	0.526855	0.124	0.004	0	Skin_Macr	CLEC5A
CYP2S11	0	0.523788	0.129	0.003	0	Skin_Macr	CYP2S1
PTAFR1	0	0.521612	0.126	0.015	0	Skin_Macr	PTAFR
CTD.2020K	0	0.496959	0.133	0.012	0	Skin_Macr	CTD.2020K17.1
CSF1R1	0	0.489948	0.137	0.018	0	Skin_Macr	CSF1R
CD274	0	0.476957	0.092	0.006	0	Skin_Macr	CD274
ALOX15	0	0.471411	0.119	0.004	0	Skin_Macr	ALOX15
HCK1	0	0.462227	0.156	0.018	0	Skin_Macr	HCK
LILRB21	0	0.458707	0.106	0.009	0	Skin_Macr	LILRB2
TVP23A	0	0.455849	0.089	0.003	0	Skin_Macr	TVP23A
CLECL12	0	0.453462	0.123	0.01	0	Skin_Macr	CLECL1
P2RY141	0	0.448868	0.108	0.007	0	Skin_Macr	P2RY14
KMO	0	0.446556	0.084	0.007	0	Skin_Macr	KMO
MIR181A1	0	0.435062	0.097	0.006	0	Skin_Macr	MIR181A1HG
SLAMF81	0	0.419369	0.111	0.005	0	Skin_Macr	SLAMF8
CYBA3	0	0.397574	0.843	0.431	0	Skin_Macr	CYBA
SLAMF9	0	0.386722	0.099	0.006	0	Skin_Macr	SLAMF9
UTF1	0	0.383807	0.079	0.005	0	Skin_Macr	UTF1
CIITA1	0	0.371278	0.099	0.008	0	Skin_Macr	CIITA
GPR132	0	0.362245	0.09	0.005	0	Skin_Macr	GPR132
SCN9A1	0	0.358149	0.081	0.006	0	Skin_Macr	SCN9A
KRT142	0	0.353103	0.693	0.135	0	Skin_Macr	KRT14
RP11.1143	0	0.301344	0.723	0.217	0	Skin_Macr	RP11.1143G9.4
GPR35	0	0.26378	0.064	0.003	0	Skin_Macr	GPR35
CCR52	0	0.263157	0.06	0.003	0	Skin_Macr	CCR5
AVPI1	7.2E-308	1.102012	0.369	0.121	1.2E-303	Skin_Macr	AVPI1
ANXA57	1.5E-307	0.937002	0.652	0.309	2.4E-303	Skin_Macr	ANXA5
LRRFIP14	5.9E-307	1.050104	0.684	0.365	9.4E-303	Skin_Macr	LRRFIP1
CD401	1.2E-306	0.884513	0.271	0.07	1.9E-302	Skin_Macr	CD40
PFDN51	1.5E-306	0.717204	0.951	0.721	2.4E-302	Skin_Macr	PFDN5
MSR11	1.2E-303	0.655463	0.133	0.019	2E-299	Skin_Macr	MSR1
PAK11	1.5E-300	0.76382	0.248	0.061	2.4E-296	Skin_Macr	PAK1
RASSF22	2.2E-297	0.51355	0.138	0.02	3.4E-293	Skin_Macr	RASSF2
LY861	6.5E-295	0.416137	0.189	0.035	1E-290	Skin_Macr	LY86
LY963	1.9E-290	0.742291	0.259	0.064	3E-286	Skin_Macr	LY96
UBASH3B	1E-289	0.365984	0.092	0.01	1.6E-285	Skin_Macr	UBASH3B
ZC3H12A1	2.4E-288	0.826793	0.322	0.096	3.8E-284	Skin_Macr	ZC3H12A
RB11	6.5E-286	1.040291	0.291	0.085	1E-281	Skin_Macr	RB1
CAPG3	2.4E-284	0.778121	0.44	0.16	3.8E-280	Skin_Macr	CAPG
ADAM191	9.3E-283	0.368945	0.081	0.007	1.5E-278	Skin_Macr	ADAM19

MAP4K41	2.5E-282	1.05967	0.329	0.106	4E-278	Skin_Macr	MAP4K4
SOCS33	1.6E-281	0.578016	0.447	0.158	2.5E-277	Skin_Macr	SOCS3
RPL211	2E-281	0.667118	0.997	0.809	3.3E-277	Skin_Macr	RPL21
CRIP12	5.1E-281	0.912091	0.606	0.269	8.2E-277	Skin_Macr	CRIP1
EDEM11	7.1E-280	0.836577	0.287	0.082	1.1E-275	Skin_Macr	EDEM1
GADD45B	2.9E-278	0.792779	0.716	0.368	4.7E-274	Skin_Macr	GADD45B
MS4A71	4.1E-276	0.56575	0.145	0.024	6.6E-272	Skin_Macr	MS4A7
HLA.DOA2	4.8E-276	0.345851	0.112	0.015	7.6E-272	Skin_Macr	HLA.DOA
PIM22	9.3E-276	0.590565	0.189	0.038	1.5E-271	Skin_Macr	PIM2
FUT1	1.2E-275	0.316256	0.066	0.005	2E-271	Skin_Macr	FUT1
S100B1	5E-272	0.735011	0.15	0.025	8E-268	Skin_Macr	S100B
TTYH21	2.1E-271	0.374963	0.095	0.011	3.3E-267	Skin_Macr	TTYH2
GLS4	1.9E-270	0.938663	0.34	0.111	3E-266	Skin_Macr	GLS
C1orf1621	3.3E-268	0.535899	0.309	0.086	5.3E-264	Skin_Macr	C1orf162
HNRNPH1:	8.6E-268	1.100599	0.585	0.291	1.4E-263	Skin_Macr	HNRNPH1
NLRP31	1E-267	0.304995	0.07	0.006	1.6E-263	Skin_Macr	NLRP3
DUSP1	2.1E-264	0.896329	0.858	0.554	3.4E-260	Skin_Macr	DUSP1
STK17B4	4.7E-264	0.77445	0.32	0.099	7.5E-260	Skin_Macr	STK17B
TAGLN25	8.9E-264	0.788801	0.733	0.401	1.4E-259	Skin_Macr	TAGLN2
RPS213	2.1E-263	0.712948	0.953	0.665	3.4E-259	Skin_Macr	RPS21
RAB8B2	4.9E-263	0.887729	0.301	0.092	7.8E-259	Skin_Macr	RAB8B
FOLR21	8.3E-261	0.726192	0.124	0.019	1.3E-256	Skin_Macr	FOLR2
CD363	2E-260	0.49436	0.11	0.015	3.1E-256	Skin_Macr	CD36
HAVCR21	5.7E-260	0.42647	0.121	0.018	9.1E-256	Skin_Macr	HAVCR2
RASSF41	7.2E-260	0.588371	0.165	0.032	1.1E-255	Skin_Macr	RASSF4
ARPC24	1.4E-258	0.835608	0.811	0.568	2.2E-254	Skin_Macr	ARPC2
FGD21	3.1E-258	0.342001	0.089	0.01	4.9E-254	Skin_Macr	FGD2
ARPC52	7.3E-258	0.944592	0.691	0.406	1.2E-253	Skin_Macr	ARPC5
PILRA1	1.2E-256	0.455758	0.123	0.019	1.9E-252	Skin_Macr	PILRA
DNAJB61	1.7E-254	1.032974	0.692	0.413	2.7E-250	Skin_Macr	DNAJB6
QPCT1	1.7E-254	0.584206	0.242	0.062	2.7E-250	Skin_Macr	QPCT
CYTH42	7.8E-252	0.418705	0.152	0.027	1.2E-247	Skin_Macr	CYTH4
RPL27A1	9E-252	0.578057	0.986	0.832	1.4E-247	Skin_Macr	RPL27A
ARF61	4E-250	0.944991	0.664	0.369	6.3E-246	Skin_Macr	ARF6
DPYSL24	1.1E-249	0.703859	0.307	0.094	1.8E-245	Skin_Macr	DPYSL2
ABL2	2.8E-248	0.869248	0.281	0.085	4.5E-244	Skin_Macr	ABL2
ARHGAP31	3.5E-248	0.351673	0.102	0.013	5.6E-244	Skin_Macr	ARHGAP31
LSP12	6.9E-248	0.605709	0.546	0.223	1.1E-243	Skin_Macr	LSP1
GPX4	7.5E-248	0.266311	0.841	0.537	1.2E-243	Skin_Macr	GPX4
GPR137B1	3.4E-247	0.69381	0.21	0.052	5.4E-243	Skin_Macr	GPR137B
GPX11	1.6E-246	0.608084	0.733	0.419	2.6E-242	Skin_Macr	GPX1
SH3BGRL5	8.8E-245	0.809073	0.525	0.237	1.4E-240	Skin_Macr	SH3BGRL
SNHG151	4E-244	0.703172	0.324	0.106	6.3E-240	Skin_Macr	SNHG15
USP12	1.2E-243	0.750333	0.24	0.065	1.9E-239	Skin_Macr	USP12
LDHA2	1.1E-242	0.859649	0.73	0.433	1.8E-238	Skin_Macr	LDHA
RPL261	1.3E-242	0.602107	0.992	0.831	2.1E-238	Skin_Macr	RPL26
PTGER41	7.2E-242	0.561889	0.203	0.047	1.1E-237	Skin_Macr	PTGER4

SLC43A21	1.8E-240	0.579212	0.156	0.031	2.8E-236	Skin_Macr	SLC43A2
FMNL14	4.1E-239	0.490643	0.235	0.06	6.5E-235	Skin_Macr	FMNL1
TUBB3	5.1E-238	0.832157	0.606	0.303	8.2E-234	Skin_Macr	TUBB
RPL381	1.9E-237	0.618481	0.958	0.757	3.1E-233	Skin_Macr	RPL38
CDC422	2.1E-237	1.025847	0.733	0.464	3.4E-233	Skin_Macr	CDC42
CDKN1A	7.9E-237	0.642074	0.563	0.252	1.3E-232	Skin_Macr	CDKN1A
SNN	1.1E-236	0.667197	0.177	0.039	1.8E-232	Skin_Macr	SNN
SNX3	1.1E-234	0.894369	0.684	0.409	1.8E-230	Skin_Macr	SNX3
ATP6V1B2	3E-232	0.929462	0.269	0.083	4.8E-228	Skin_Macr	ATP6V1B2
DAPP1	4.3E-232	0.875941	0.265	0.08	6.9E-228	Skin_Macr	DAPP1
PTPRC3	5.5E-231	0.427111	0.33	0.102	8.7E-227	Skin_Macr	PTPRC
SMAP23	1.6E-229	0.715275	0.234	0.064	2.6E-225	Skin_Macr	SMAP2
MARCH11	2.5E-229	0.376579	0.106	0.016	4E-225	Skin_Macr	MARCH1
PTPN11	1.5E-228	0.857261	0.258	0.078	2.4E-224	Skin_Macr	PTPN1
HCAR3	2.5E-227	0.387479	0.096	0.013	4.1E-223	Skin_Macr	HCAR3
JARID2	8.4E-224	0.852357	0.287	0.096	1.3E-219	Skin_Macr	JARID2
CD41	1.3E-221	0.386104	0.146	0.028	2E-217	Skin_Macr	CD4
LITAF	2E-221	1.057175	0.623	0.359	3.2E-217	Skin_Macr	LITAF
VASP3	2.7E-219	0.725934	0.34	0.124	4.2E-215	Skin_Macr	VASP
ITGB23	1.4E-218	0.365775	0.33	0.103	2.2E-214	Skin_Macr	ITGB2
RAP1B1	7.9E-217	0.907576	0.526	0.263	1.3E-212	Skin_Macr	RAP1B
ZFP36L12	3E-216	0.813313	0.761	0.462	4.7E-212	Skin_Macr	ZFP36L1
RPLP15	1.1E-215	0.46608	0.998	0.913	1.8E-211	Skin_Macr	RPLP1
ARRB21	7.1E-215	0.500286	0.274	0.082	1.1E-210	Skin_Macr	ARRB2
BATF31	8.4E-215	0.335117	0.091	0.012	1.3E-210	Skin_Macr	BATF3
GPR653	2E-213	0.480687	0.123	0.022	3.3E-209	Skin_Macr	GPR65
POMP2	1E-212	0.870383	0.662	0.396	1.6E-208	Skin_Macr	POMP
MTSS12	1.3E-208	0.635634	0.207	0.056	2.1E-204	Skin_Macr	MTSS1
PIK3R51	1.5E-208	0.285326	0.079	0.01	2.4E-204	Skin_Macr	PIK3R5
RTN11	4.6E-208	0.439082	0.113	0.019	7.3E-204	Skin_Macr	RTN1
CREB52	1.1E-207	0.602159	0.202	0.053	1.8E-203	Skin_Macr	CREB5
RPS151	2.6E-207	0.51029	0.996	0.878	4.2E-203	Skin_Macr	RPS15
PLEKHO12	6.4E-207	0.672208	0.26	0.08	1E-202	Skin_Macr	PLEKHO1
PPP1CB1	3.8E-206	0.969127	0.72	0.449	6.1E-202	Skin_Macr	PPP1CB
CYCS2	6E-205	0.771676	0.6	0.335	9.5E-201	Skin_Macr	CYCS
NBPF10	2.1E-204	0.546232	0.183	0.045	3.4E-200	Skin_Macr	NBPF10
PTGER31	2.2E-203	0.473072	0.138	0.028	3.5E-199	Skin_Macr	PTGER3
ISG202	5.9E-203	1.06763	0.44	0.197	9.3E-199	Skin_Macr	ISG20
RPL361	3.8E-202	0.512212	0.988	0.837	6.1E-198	Skin_Macr	RPL36
WTAP	5.3E-202	0.909397	0.528	0.275	8.5E-198	Skin_Macr	WTAP
ANXA21	4.2E-201	0.592303	0.87	0.591	6.7E-197	Skin_Macr	ANXA2
RASSF31	4.5E-201	0.873646	0.232	0.072	7.1E-197	Skin_Macr	RASSF3
RPS283	8.8E-201	0.525582	0.996	0.878	1.4E-196	Skin_Macr	RPS28
RGCC8	2.1E-200	0.831388	0.471	0.208	3.4E-196	Skin_Macr	RGCC
RP21	6.6E-200	0.525633	0.13	0.025	1E-195	Skin_Macr	RP2
EIF3E3	8.3E-200	0.76427	0.72	0.442	1.3E-195	Skin_Macr	EIF3E
ASAP12	9.4E-200	0.708998	0.273	0.091	1.5E-195	Skin_Macr	ASAP1

THBD1	1.7E-199	0.609394	0.168	0.039	2.7E-195	Skin_Macr	THBD
ALCAM4	6E-199	0.743093	0.271	0.092	9.5E-195	Skin_Macr	ALCAM
CASP11	3E-198	0.559859	0.199	0.053	4.9E-194	Skin_Macr	CASP1
CD331	1.2E-196	0.253206	0.079	0.01	2E-192	Skin_Macr	CD33
TMEM123	5.4E-196	0.84538	0.527	0.276	8.6E-192	Skin_Macr	TMEM123
ETS21	2E-195	0.8299	0.372	0.152	3.1E-191	Skin_Macr	ETS2
ATF51	2.4E-192	0.765217	0.237	0.075	3.8E-188	Skin_Macr	ATF5
MAP3K81	3.5E-192	0.761425	0.306	0.112	5.6E-188	Skin_Macr	MAP3K8
C1orf562	1.4E-190	1.037643	0.309	0.117	2.2E-186	Skin_Macr	C1orf56
LAT21	2E-190	0.361418	0.145	0.031	3.3E-186	Skin_Macr	LAT2
RPS161	8.6E-190	0.443965	0.983	0.832	1.4E-185	Skin_Macr	RPS16
FCGRT3	3E-189	0.644045	0.531	0.271	4.8E-185	Skin_Macr	FCGRT
NABP11	5.2E-188	0.768948	0.382	0.162	8.4E-184	Skin_Macr	NABP1
EREG	1.2E-185	0.96041	0.14	0.031	2E-181	Skin_Macr	EREG
RPS192	8.2E-185	0.416908	0.998	0.872	1.3E-180	Skin_Macr	RPS19
RAC12	3.7E-184	0.691247	0.79	0.542	5.9E-180	Skin_Macr	RAC1
NR4A1	1.9E-183	0.685869	0.508	0.251	3.1E-179	Skin_Macr	NR4A1
KLF64	2.5E-183	0.776745	0.668	0.395	3.9E-179	Skin_Macr	KLF6
PRDX17	7.9E-182	0.959017	0.685	0.447	1.3E-177	Skin_Macr	PRDX1
MTPN3	1.6E-181	0.764648	0.348	0.145	2.5E-177	Skin_Macr	MTPN
RPL351	8.4E-181	0.460021	0.987	0.832	1.3E-176	Skin_Macr	RPL35
ZFP361	9.1E-180	0.490588	0.83	0.535	1.4E-175	Skin_Macr	ZFP36
MPEG11	4.4E-178	0.303375	0.089	0.014	7E-174	Skin_Macr	MPEG1
POGLUT1	3.1E-177	0.597187	0.126	0.026	4.9E-173	Skin_Macr	POGLUT1
PFDN21	5.6E-177	0.748762	0.511	0.269	8.9E-173	Skin_Macr	PFDN2
NFKBIE	9.8E-177	0.659081	0.231	0.075	1.6E-172	Skin_Macr	NFKBIE
RPL271	1.7E-176	0.463855	0.946	0.745	2.7E-172	Skin_Macr	RPL27
NRARP2	3.3E-176	0.517662	0.155	0.038	5.3E-172	Skin_Macr	NRARP
IVNS1ABP	1.2E-175	0.886267	0.337	0.145	1.9E-171	Skin_Macr	IVNS1ABP
FAM49B3	5.5E-174	0.737367	0.377	0.168	8.8E-170	Skin_Macr	FAM49B
RPL36A1	1.4E-173	0.435404	0.823	0.503	2.2E-169	Skin_Macr	RPL36A
TMEM120I	4.3E-173	0.648793	0.16	0.041	6.9E-169	Skin_Macr	TMEM120B
RP11.347P	2.6E-172	0.500846	0.096	0.016	4.2E-168	Skin_Macr	RP11.347P5.1
MARCO1	8.2E-172	0.251878	0.081	0.012	1.3E-167	Skin_Macr	MARCO
CDC42SE2	1.1E-171	0.690769	0.3	0.116	1.7E-167	Skin_Macr	CDC42SE2
POU2F23	1.2E-171	0.456481	0.171	0.044	1.9E-167	Skin_Macr	POU2F2
FLNA7	6.7E-171	0.521089	0.359	0.144	1.1E-166	Skin_Macr	FLNA
IFNGR12	1.8E-170	0.798219	0.319	0.13	2.9E-166	Skin_Macr	IFNGR1
PYCARD1	2.4E-170	0.46687	0.379	0.162	3.8E-166	Skin_Macr	PYCARD
TSPAN333	4E-170	0.571782	0.159	0.042	6.4E-166	Skin_Macr	TSPAN33
ZNF3311	8.4E-170	0.770947	0.276	0.1	1.3E-165	Skin_Macr	ZNF331
TOR3A1	1.3E-169	0.568256	0.18	0.05	2E-165	Skin_Macr	TOR3A
EMB4	1.3E-169	0.429671	0.157	0.038	2.1E-165	Skin_Macr	EMB
RPS113	5E-169	0.486273	0.957	0.773	8E-165	Skin_Macr	RPS11
RPL18A1	5.2E-168	0.408297	0.993	0.819	8.4E-164	Skin_Macr	RPL18A
SLC43A33	1.2E-167	0.437555	0.135	0.031	1.9E-163	Skin_Macr	SLC43A3
TPM34	2.1E-167	0.701127	0.568	0.326	3.3E-163	Skin_Macr	TPM3

RIPK2	3.7E-165	0.598933	0.193	0.058	5.9E-161	Skin_Macr	RIPK2
PTP4A23	1.8E-163	0.824149	0.507	0.287	2.8E-159	Skin_Macr	PTP4A2
ITGAM1	6.9E-163	0.3038	0.085	0.014	1.1E-158	Skin_Macr	ITGAM
SRC	2.6E-162	0.292486	0.087	0.014	4.2E-158	Skin_Macr	SRC
NAP1L11	2.6E-162	0.693332	0.647	0.395	4.2E-158	Skin_Macr	NAP1L1
GK	1.4E-161	0.757754	0.191	0.059	2.2E-157	Skin_Macr	GK
CORO1C	3.5E-161	0.730728	0.287	0.114	5.5E-157	Skin_Macr	CORO1C
DBI7	8.5E-161	0.725461	0.69	0.408	1.4E-156	Skin_Macr	DBI
SYNGR22	6.8E-160	0.814724	0.437	0.229	1.1E-155	Skin_Macr	SYNGR2
TANK2	1E-159	0.718548	0.301	0.123	1.6E-155	Skin_Macr	TANK
ZNF2671	3E-159	0.617251	0.22	0.074	4.8E-155	Skin_Macr	ZNF267
RGS103	4.9E-158	0.600187	0.433	0.211	7.8E-154	Skin_Macr	RGS10
ARRDC3	1.6E-155	0.83521	0.454	0.239	2.6E-151	Skin_Macr	ARRDC3
RBM31	2.4E-154	0.64241	0.668	0.434	3.9E-150	Skin_Macr	RBM3
LIMS13	5.3E-154	0.755696	0.356	0.164	8.4E-150	Skin_Macr	LIMS1
RPS181	6.9E-154	0.385964	1	0.914	1.1E-149	Skin_Macr	RPS18
MOB1A3	2.6E-152	0.758776	0.375	0.182	4.1E-148	Skin_Macr	MOB1A
RPS201	8.7E-152	0.402939	0.966	0.791	1.4E-147	Skin_Macr	RPS20
FGR	4E-151	0.274112	0.12	0.026	6.4E-147	Skin_Macr	FGR
SLCO3A13	7.4E-151	0.551594	0.174	0.051	1.2E-146	Skin_Macr	SLCO3A1
RPLP21	7.8E-151	0.274379	0.993	0.84	1.2E-146	Skin_Macr	RPLP2
ZFHX33	2.9E-150	0.700588	0.236	0.086	4.7E-146	Skin_Macr	ZFHX3
CHD21	9E-150	0.81668	0.402	0.202	1.4E-145	Skin_Macr	CHD2
B2M4	1.1E-149	0.310838	1	0.927	1.7E-145	Skin_Macr	B2M
SET4	1.2E-149	0.751106	0.53	0.305	1.9E-145	Skin_Macr	SET
ATP1B12	1.9E-148	0.873759	0.553	0.319	3E-144	Skin_Macr	ATP1B1
RPS247	1E-147	0.428556	0.979	0.836	1.6E-143	Skin_Macr	RPS24
U2AF11	1.4E-146	0.579777	0.483	0.25	2.3E-142	Skin_Macr	U2AF1
KBTBD8	1.1E-145	0.266657	0.063	0.009	1.8E-141	Skin_Macr	KBTBD8
DDIT41	1.8E-145	0.585319	0.466	0.237	2.9E-141	Skin_Macr	DDIT4
CAPZA11	1.7E-144	0.883068	0.477	0.273	2.7E-140	Skin_Macr	CAPZA1
ICOSLG	1.9E-144	0.283724	0.076	0.012	3E-140	Skin_Macr	ICOSLG
SCARF11	7.2E-144	0.273077	0.07	0.011	1.1E-139	Skin_Macr	SCARF1
ARL8B	2.4E-143	0.867811	0.364	0.182	3.8E-139	Skin_Macr	ARL8B
MOB3A1	2.7E-143	0.428561	0.158	0.044	4.3E-139	Skin_Macr	MOB3A
TM6SF12	3.9E-143	0.375603	0.089	0.017	6.2E-139	Skin_Macr	TM6SF1
FSCN11	5.2E-143	0.60286	0.196	0.064	8.2E-139	Skin_Macr	FSCN1
DDAH23	7.3E-143	0.701692	0.407	0.206	1.2E-138	Skin_Macr	DDAH2
NPC22	8.7E-143	0.600097	0.663	0.434	1.4E-138	Skin_Macr	NPC2
P2RX71	3.1E-142	0.277105	0.067	0.01	5E-138	Skin_Macr	P2RX7
MCTP12	3.9E-141	0.388763	0.131	0.032	6.2E-137	Skin_Macr	MCTP1
ADCY72	4.6E-141	0.279419	0.092	0.017	7.4E-137	Skin_Macr	ADCY7
ANKRD282	2.5E-140	0.726674	0.266	0.106	4E-136	Skin_Macr	ANKRD28
GRB22	1.2E-139	0.70651	0.35	0.168	1.9E-135	Skin_Macr	GRB2
SSR15	1.2E-139	0.702823	0.374	0.187	2E-135	Skin_Macr	SSR1
DRAP11	2.1E-139	0.550137	0.567	0.329	3.4E-135	Skin_Macr	DRAP1
TGFB14	2.4E-139	0.526525	0.255	0.095	3.8E-135	Skin_Macr	TGFB1

PAFAH1B2	4.1E-138	0.773433	0.284	0.122	6.5E-134	Skin_Macr	PAFAH1B2
KCTD201	9.8E-137	0.608886	0.209	0.074	1.6E-132	Skin_Macr	KCTD20
KDM7A1	1.8E-136	0.518643	0.158	0.047	2.9E-132	Skin_Macr	KDM7A
BHLHE40	2.6E-136	0.704512	0.381	0.183	4.1E-132	Skin_Macr	BHLHE40
FERMT32	2.8E-136	0.295091	0.163	0.046	4.5E-132	Skin_Macr	FERMT3
MARCKSL1	5E-136	0.85683	0.393	0.201	8E-132	Skin_Macr	MARCKSL1
HSP90AB1	1.1E-135	0.609905	0.786	0.565	1.7E-131	Skin_Macr	HSP90AB1
CD55	1.9E-135	0.623831	0.475	0.261	3E-131	Skin_Macr	CD55
ICAM11	3.5E-135	0.408617	0.315	0.131	5.6E-131	Skin_Macr	ICAM1
DDX211	8.5E-135	0.63931	0.393	0.194	1.4E-130	Skin_Macr	DDX21
CCL43	1.7E-133	1.193228	0.153	0.044	2.8E-129	Skin_Macr	CCL4
AP1S3	2E-133	0.322066	0.074	0.013	3.1E-129	Skin_Macr	AP1S3
RPS142	5.4E-133	0.367603	0.997	0.879	8.6E-129	Skin_Macr	RPS14
IL6R1	5.8E-133	0.446045	0.139	0.038	9.2E-129	Skin_Macr	IL6R
ARL6IP1	1.7E-132	0.827194	0.511	0.313	2.7E-128	Skin_Macr	ARL6IP1
PHACTR12	1.7E-132	0.478623	0.126	0.032	2.8E-128	Skin_Macr	PHACTR1
SLC25A52	2.6E-132	0.599874	0.589	0.364	4.2E-128	Skin_Macr	SLC25A5
PPP1R18	2.8E-132	0.414132	0.285	0.114	4.5E-128	Skin_Macr	PPP1R18
FOXN32	1.2E-130	0.65881	0.283	0.121	1.9E-126	Skin_Macr	FOXN3
FAU4	1.3E-130	0.319183	0.984	0.852	2.1E-126	Skin_Macr	FAU
NFKB21	1.5E-130	0.50955	0.21	0.075	2.5E-126	Skin_Macr	NFKB2
IL101	2.5E-130	0.299011	0.054	0.007	4E-126	Skin_Macr	IL10
EEF1A110	3.9E-130	0.405784	0.992	0.913	6.2E-126	Skin_Macr	EEF1A1
ACOT93	4.9E-130	0.487608	0.177	0.057	7.8E-126	Skin_Macr	ACOT9
PSAP2	1.2E-129	0.391206	0.65	0.43	1.9E-125	Skin_Macr	PSAP
MAP2K1	2.8E-129	0.717643	0.368	0.184	4.5E-125	Skin_Macr	MAP2K1
RPL2210	2.8E-129	0.468328	0.904	0.689	4.5E-125	Skin_Macr	RPL22
RAPGEF11	3.2E-129	0.499948	0.186	0.062	5.1E-125	Skin_Macr	RAPGEF1
FAM105A2	6.7E-129	0.377944	0.131	0.035	1.1E-124	Skin_Macr	FAM105A
CDC42EP2	6.2E-128	0.435531	0.129	0.034	9.8E-124	Skin_Macr	CDC42EP2
BCL11A1	8.4E-128	0.619106	0.144	0.042	1.3E-123	Skin_Macr	BCL11A
CTSS2	1.1E-127	0.427179	0.421	0.21	1.7E-123	Skin_Macr	CTSS
SRSF24	1.3E-127	0.622198	0.538	0.327	2.1E-123	Skin_Macr	SRSF2
SPRED2	1.5E-127	0.458387	0.127	0.034	2.5E-123	Skin_Macr	SPRED2
RPL71	7.1E-127	0.282655	0.976	0.792	1.1E-122	Skin_Macr	RPL7
RTN43	2.5E-125	0.580802	0.612	0.389	4E-121	Skin_Macr	RTN4
ST3GAL61	3.1E-125	0.486637	0.115	0.029	4.9E-121	Skin_Macr	ST3GAL6
CD3022	6.4E-125	0.493373	0.181	0.061	1E-120	Skin_Macr	CD302
TLR21	6.8E-125	0.271018	0.068	0.011	1.1E-120	Skin_Macr	TLR2
CCRL2	1.5E-124	0.259965	0.065	0.01	2.4E-120	Skin_Macr	CCRL2
PEA15	9.7E-124	0.664322	0.34	0.169	1.6E-119	Skin_Macr	PEA15
HSPD14	1.1E-123	0.79603	0.441	0.253	1.7E-119	Skin_Macr	HSPD1
N4BP2L1	5.9E-122	0.494884	0.181	0.061	9.4E-118	Skin_Macr	N4BP2L1
PMAIP1	1.5E-121	0.600686	0.404	0.202	2.3E-117	Skin_Macr	PMAIP1
RSL24D11	1.5E-121	0.616747	0.509	0.302	2.4E-117	Skin_Macr	RSL24D1
FOSL21	2E-121	0.738301	0.402	0.215	3.1E-117	Skin_Macr	FOSL2
TET22	8.5E-121	0.533988	0.166	0.054	1.3E-116	Skin_Macr	TET2

TNF3	2.7E-120	0.402755	0.085	0.017	4.3E-116	Skin_Macr	TNF
HSPH1	3.3E-120	0.779088	0.418	0.228	5.2E-116	Skin_Macr	HSPH1
SPAG92	3.8E-119	0.729426	0.363	0.189	6E-115	Skin_Macr	SPAG9
CKLF4	9.9E-119	0.549393	0.316	0.146	1.6E-114	Skin_Macr	CKLF
SLC7A111	7.4E-118	0.385703	0.09	0.02	1.2E-113	Skin_Macr	SLC7A11
AC016831.	2.7E-117	0.35254	0.084	0.017	4.2E-113	Skin_Macr	AC016831.7
TIMM17A1	4.9E-117	0.678724	0.281	0.132	7.8E-113	Skin_Macr	TIMM17A
TXN	2.5E-116	0.625733	0.741	0.522	4E-112	Skin_Macr	TXN
IL3RA1	6.2E-116	0.322907	0.089	0.019	9.9E-112	Skin_Macr	IL3RA
PPTC71	2.3E-115	0.4916	0.176	0.061	3.6E-111	Skin_Macr	PPTC7
RP11.345J4	3.2E-115	0.302676	0.109	0.027	5.2E-111	Skin_Macr	RP11.345J4.5
NUP62	2E-114	0.543452	0.206	0.079	3.1E-110	Skin_Macr	NUP62
MFSD2A	2.5E-114	0.299237	0.082	0.017	4.1E-110	Skin_Macr	MFSD2A
RPL37A1	4.4E-114	0.320966	0.984	0.853	7E-110	Skin_Macr	RPL37A
HSPB1	4.5E-114	0.587909	0.805	0.559	7.1E-110	Skin_Macr	HSPB1
GNAI24	1.9E-113	0.49626	0.414	0.221	3.1E-109	Skin_Macr	GNAI2
CHMP4B	2.1E-113	0.702711	0.498	0.302	3.4E-109	Skin_Macr	CHMP4B
EIF4E	1.3E-112	0.659412	0.42	0.239	2.1E-108	Skin_Macr	EIF4E
PTPN62	5E-112	0.323961	0.202	0.073	7.9E-108	Skin_Macr	PTPN6
CSF2RB3	7.7E-112	0.457317	0.126	0.036	1.2E-107	Skin_Macr	CSF2RB
GAPDH4	1.2E-111	0.383557	0.907	0.76	1.8E-107	Skin_Macr	GAPDH
NFAT53	2.3E-111	0.657437	0.272	0.124	3.7E-107	Skin_Macr	NFAT5
PPP3R1	3.7E-111	0.560129	0.184	0.067	5.9E-107	Skin_Macr	PPP3R1
RPL121	6.2E-111	0.294713	0.995	0.849	9.8E-107	Skin_Macr	RPL12
MSN5	6.7E-111	0.444256	0.326	0.152	1.1E-106	Skin_Macr	MSN
NUBP1	3.4E-110	0.513742	0.164	0.056	5.4E-106	Skin_Macr	NUBP1
TNFRSF182	8.7E-110	0.364768	0.148	0.046	1.4E-105	Skin_Macr	TNFRSF18
UBE2D31	3.1E-109	0.544141	0.723	0.529	4.9E-105	Skin_Macr	UBE2D3
MEF2C5	7.3E-109	0.457417	0.206	0.078	1.2E-104	Skin_Macr	MEF2C
TPRA1	2E-108	0.782585	0.222	0.093	3.2E-104	Skin_Macr	TPRA1
CD6311	1.1E-107	0.345594	0.883	0.618	1.8E-103	Skin_Macr	CD63
RPS133	1.7E-107	0.327057	0.976	0.814	2.7E-103	Skin_Macr	RPS13
RGL12	7.7E-107	0.439705	0.089	0.021	1.2E-102	Skin_Macr	RGL1
FOS6	7.8E-107	0.512953	0.681	0.419	1.3E-102	Skin_Macr	FOS
ABCA12	9.3E-107	0.679716	0.175	0.063	1.5E-102	Skin_Macr	ABCA1
STX4	2.7E-106	0.590109	0.23	0.097	4.4E-102	Skin_Macr	STX4
OAZ21	4.4E-106	0.620693	0.335	0.174	7.1E-102	Skin_Macr	OAZ2
CMTM71	4.8E-106	0.312342	0.186	0.067	7.7E-102	Skin_Macr	CMTM7
G3BP1	3.3E-105	0.57271	0.316	0.157	5.3E-101	Skin_Macr	G3BP1
GNA12	1.3E-104	0.399945	0.121	0.036	2E-100	Skin_Macr	GNA12
ACSL5	5.1E-104	0.330118	0.107	0.029	8.1E-100	Skin_Macr	ACSL5
HSPA1B1	5.9E-104	1.023412	0.499	0.306	9.4E-100	Skin_Macr	HSPA1B
PIM32	6.1E-104	0.611681	0.467	0.287	9.7E-100	Skin_Macr	PIM3
TMED25	1.2E-103	0.59804	0.528	0.341	1.9E-99	Skin_Macr	TMED2
AXL3	1E-102	0.497964	0.232	0.095	1.66E-98	Skin_Macr	AXL
BMP2K1	1.5E-102	0.434467	0.131	0.041	2.33E-98	Skin_Macr	BMP2K
TIAM1	1.5E-102	0.428361	0.136	0.043	2.42E-98	Skin_Macr	TIAM1

VOPP12	3.4E-102	0.593037	0.246	0.112	5.43E-98	Skin_Macr	VOPP1
RPS236	5.3E-102	0.332779	0.985	0.809	8.43E-98	Skin_Macr	RPS23
ANPEP1	8.4E-102	0.327362	0.114	0.032	1.35E-97	Skin_Macr	ANPEP
AKAP134	2.5E-101	0.634095	0.377	0.213	3.91E-97	Skin_Macr	AKAP13
HSPA6	8.9E-101	1.014713	0.216	0.087	1.42E-96	Skin_Macr	HSPA6
OLR11	2.3E-100	0.566585	0.076	0.017	3.71E-96	Skin_Macr	OLR1
RPL321	1.1E-99	0.25792	0.995	0.869	1.78E-95	Skin_Macr	RPL32
TPP11	1.05E-98	0.557377	0.22	0.094	1.67E-94	Skin_Macr	TPP1
MORF4L11	3.46E-98	0.520168	0.65	0.454	5.52E-94	Skin_Macr	MORF4L1
HSP90AA1	2.62E-97	0.592473	0.846	0.71	4.18E-93	Skin_Macr	HSP90AA1
ACSL42	2.79E-97	0.410216	0.107	0.03	4.45E-93	Skin_Macr	ACSL4
ATP5L2	1.93E-96	0.370611	0.835	0.656	3.07E-92	Skin_Macr	ATP5L
CXCL1	2.82E-96	0.412283	0.294	0.138	4.49E-92	Skin_Macr	CXCL1
HSD17B11	9.51E-96	0.455696	0.286	0.135	1.52E-91	Skin_Macr	HSD17B11
AMD11	1.34E-95	0.604045	0.348	0.19	2.14E-91	Skin_Macr	AMD1
HSPE12	3.21E-95	0.604119	0.55	0.368	5.12E-91	Skin_Macr	HSPE1
WIPF14	8.16E-95	0.381607	0.218	0.089	1.3E-90	Skin_Macr	WIPF1
PQLC33	8.65E-95	0.471108	0.211	0.089	1.38E-90	Skin_Macr	PQLC3
RHOC3	1.7E-94	0.483456	0.395	0.22	2.71E-90	Skin_Macr	RHOC
LYPD3	3.22E-94	0.316847	0.208	0.085	5.13E-90	Skin_Macr	LYPD3
ARL6IP55	8.24E-94	0.42287	0.503	0.305	1.31E-89	Skin_Macr	ARL6IP5
SUB13	6.84E-93	0.44605	0.649	0.476	1.09E-88	Skin_Macr	SUB1
ETV61	3.67E-92	0.64672	0.231	0.108	5.85E-88	Skin_Macr	ETV6
RPL151	3.99E-92	0.278478	0.996	0.892	6.37E-88	Skin_Macr	RPL15
RNF51	8.33E-92	0.602624	0.279	0.14	1.33E-87	Skin_Macr	RNF5
AP2S11	3.21E-91	0.480109	0.505	0.337	5.12E-87	Skin_Macr	AP2S1
DEK4	5.43E-91	0.564051	0.422	0.255	8.66E-87	Skin_Macr	DEK
CFL11	7.35E-91	0.273605	0.857	0.706	1.17E-86	Skin_Macr	CFL1
PLEK2	1.12E-89	0.325315	0.103	0.03	1.78E-85	Skin_Macr	PLEK2
CSNK1A13	3.3E-89	0.545126	0.436	0.268	5.26E-85	Skin_Macr	CSNK1A1
COMMD62	5.31E-89	0.355689	0.691	0.468	8.47E-85	Skin_Macr	COMMD6
LCP22	9E-89	0.2546	0.095	0.026	1.44E-84	Skin_Macr	LCP2
OGFRL18	4.22E-88	0.546136	0.238	0.112	6.73E-84	Skin_Macr	OGFRL1
BLOC1S62	5.88E-88	0.527961	0.21	0.093	9.39E-84	Skin_Macr	BLOC1S6
ELMO12	6.36E-88	0.279144	0.106	0.031	1.01E-83	Skin_Macr	ELMO1
RAP2A1	7.09E-88	0.429928	0.129	0.044	1.13E-83	Skin_Macr	RAP2A
RNF1304	8.02E-88	0.464025	0.274	0.138	1.28E-83	Skin_Macr	RNF130
KMT2E2	3.23E-87	0.583739	0.461	0.296	5.15E-83	Skin_Macr	KMT2E
HDAC91	6.27E-87	0.369709	0.122	0.039	1E-82	Skin_Macr	HDAC9
CDC372	1E-86	0.567703	0.431	0.271	1.6E-82	Skin_Macr	CDC37
CCDC88A1	1.33E-86	0.367583	0.216	0.093	2.12E-82	Skin_Macr	CCDC88A
RFTN11	1.76E-86	0.344255	0.115	0.036	2.81E-82	Skin_Macr	RFTN1
RPL375	3.81E-86	0.282536	0.965	0.845	6.08E-82	Skin_Macr	RPL37
PFKFB31	9.44E-86	0.484485	0.212	0.093	1.51E-81	Skin_Macr	PFKFB3
KDM2B	1.86E-85	0.334474	0.111	0.034	2.96E-81	Skin_Macr	KDM2B
UBE2E1	2.95E-85	0.543032	0.266	0.135	4.71E-81	Skin_Macr	UBE2E1
ARID5A1	3.56E-85	0.374928	0.22	0.096	5.68E-81	Skin_Macr	ARID5A

ATP6AP23	5.1E-85	0.548926	0.394	0.235	8.13E-81	Skin_Macr	ATP6AP2
CHST7	1.11E-84	0.28625	0.079	0.02	1.77E-80	Skin_Macr	CHST7
SDC21	1.26E-84	0.330844	0.236	0.106	2.01E-80	Skin_Macr	SDC2
FKBP1A1	1.85E-84	0.471848	0.508	0.333	2.95E-80	Skin_Macr	FKBP1A
PVRL2	1.95E-84	0.601392	0.32	0.178	3.11E-80	Skin_Macr	PVRL2
TNFRSF10E	4.3E-84	0.354707	0.124	0.042	6.85E-80	Skin_Macr	TNFRSF10B
DSE2	9.15E-84	0.38544	0.136	0.048	1.46E-79	Skin_Macr	DSE
TNFAIP22	2.05E-83	0.46987	0.277	0.137	3.27E-79	Skin_Macr	TNFAIP2
HOMER23	3.06E-83	0.467961	0.133	0.048	4.88E-79	Skin_Macr	HOMER2
POLR2E	6.45E-83	0.485063	0.345	0.196	1.03E-78	Skin_Macr	POLR2E
ACTR32	9.69E-83	0.535224	0.514	0.352	1.55E-78	Skin_Macr	ACTR3
AAED13	1.39E-82	0.424494	0.18	0.074	2.22E-78	Skin_Macr	AAED1
CEBPB	1.51E-82	0.300078	0.447	0.269	2.41E-78	Skin_Macr	CEBPB
MBNL15	1.83E-82	0.432795	0.318	0.167	2.92E-78	Skin_Macr	MBNL1
RALA	2.02E-82	0.614796	0.387	0.235	3.22E-78	Skin_Macr	RALA
SLC25A6	3.42E-82	0.288296	0.784	0.562	5.45E-78	Skin_Macr	SLC25A6
CBX64	4.59E-82	0.488603	0.191	0.081	7.32E-78	Skin_Macr	CBX6
DENND5A	1.37E-81	0.387767	0.12	0.04	2.19E-77	Skin_Macr	DENND5A
PURB	5.42E-81	0.552344	0.188	0.083	8.64E-77	Skin_Macr	PURB
SLC38A22	1.11E-80	0.379993	0.407	0.237	1.77E-76	Skin_Macr	SLC38A2
ZNF516	1.41E-80	0.40521	0.124	0.043	2.25E-76	Skin_Macr	ZNF516
QKI3	1.46E-80	0.567229	0.308	0.169	2.32E-76	Skin_Macr	QKI
HNRNPLL1	3.27E-80	0.44917	0.16	0.064	5.21E-76	Skin_Macr	HNRNPLL
GNAQ5	6.36E-80	0.646402	0.291	0.161	1.01E-75	Skin_Macr	GNAQ
MYO9B2	8.23E-80	0.45583	0.165	0.067	1.31E-75	Skin_Macr	MYO9B
H2AFV2	8.57E-80	0.582053	0.44	0.287	1.37E-75	Skin_Macr	H2AFV
SELT3	1.18E-79	0.542896	0.371	0.223	1.89E-75	Skin_Macr	SELT
SLC46A3	2.89E-79	0.279732	0.073	0.018	4.61E-75	Skin_Macr	SLC46A3
MYADM6	6.24E-79	0.494476	0.284	0.147	9.96E-75	Skin_Macr	MYADM
YWHAH2	1.14E-78	0.529779	0.354	0.204	1.83E-74	Skin_Macr	YWHAH
ELF12	1.45E-78	0.592941	0.312	0.175	2.31E-74	Skin_Macr	ELF1
LPCAT24	5.39E-78	0.315653	0.113	0.037	8.59E-74	Skin_Macr	LPCAT2
ZFAS11	9.4E-78	0.300821	0.661	0.461	1.5E-73	Skin_Macr	ZFAS1
RAB10	1.06E-77	0.646441	0.387	0.247	1.7E-73	Skin_Macr	RAB10
PLEKHB21	1.13E-77	0.55834	0.273	0.146	1.81E-73	Skin_Macr	PLEKHB2
CA24	1.34E-77	0.750877	0.205	0.095	2.14E-73	Skin_Macr	CA2
PKM3	1.61E-77	0.397882	0.552	0.369	2.56E-73	Skin_Macr	PKM
RPL102	1.01E-76	0.256857	0.998	0.875	1.61E-72	Skin_Macr	RPL10
HNRNPC1	6.38E-76	0.487939	0.589	0.426	1.02E-71	Skin_Macr	HNRNPC
IL13RA12	1.17E-75	0.528084	0.234	0.118	1.86E-71	Skin_Macr	IL13RA1
PRNP4	1.3E-75	0.446735	0.297	0.156	2.07E-71	Skin_Macr	PRNP
HSPA83	1.58E-75	0.552876	0.654	0.507	2.51E-71	Skin_Macr	HSPA8
SEPT113	1.61E-75	0.46572	0.19	0.083	2.57E-71	Skin_Macr	SEPT11
FYN4	1.73E-75	0.264958	0.164	0.065	2.76E-71	Skin_Macr	FYN
CD843	7.03E-75	0.288736	0.077	0.02	1.12E-70	Skin_Macr	CD84
ZMIZ2	2.3E-74	0.449173	0.126	0.046	3.67E-70	Skin_Macr	ZMIZ2
TNIP2	2.45E-74	0.434349	0.194	0.088	3.9E-70	Skin_Macr	TNIP2

CTNNB12	6.37E-74	0.679733	0.299	0.169	1.02E-69	Skin_Macr	CTNNB1
DOCK52	1.02E-73	0.351528	0.094	0.029	1.62E-69	Skin_Macr	DOCK5
CSRNP11	1.18E-73	0.404083	0.264	0.135	1.89E-69	Skin_Macr	CSRNP1
LAMP3	3.62E-73	0.336127	0.064	0.015	5.78E-69	Skin_Macr	LAMP3
IRAK2	1.93E-72	0.283926	0.081	0.023	3.08E-68	Skin_Macr	IRAK2
RILPL22	2.97E-72	0.491193	0.202	0.095	4.73E-68	Skin_Macr	RILPL2
TUBB2A2	3.8E-72	0.462139	0.227	0.11	6.05E-68	Skin_Macr	TUBB2A
VAPA1	4.97E-72	0.540419	0.546	0.397	7.93E-68	Skin_Macr	VAPA
SLC6A63	1.36E-71	0.319682	0.111	0.038	2.16E-67	Skin_Macr	SLC6A6
HNRNPDL1	1.52E-71	0.285158	0.528	0.332	2.42E-67	Skin_Macr	HNRNPDL
ARHGAP18	1.69E-71	0.48528	0.196	0.093	2.69E-67	Skin_Macr	ARHGAP18
PDE4DIP1	4.09E-71	0.438073	0.154	0.064	6.53E-67	Skin_Macr	PDE4DIP
PHLDA21	6.66E-71	0.404709	0.351	0.204	1.06E-66	Skin_Macr	PHLDA2
PSMA6	1.79E-70	0.419309	0.161	0.067	2.85E-66	Skin_Macr	PSMA6
ZNF385A2	2.19E-70	0.288102	0.106	0.036	3.49E-66	Skin_Macr	ZNF385A
CTSC4	1.07E-69	0.632451	0.24	0.118	1.71E-65	Skin_Macr	CTSC
THAP2	1.21E-69	0.347476	0.137	0.053	1.93E-65	Skin_Macr	THAP2
PIP4K2A3	1.94E-69	0.324212	0.146	0.058	3.09E-65	Skin_Macr	PIP4K2A
SERF24	2.87E-69	0.260376	0.951	0.82	4.57E-65	Skin_Macr	SERF2
ZC3HAV11	7.91E-69	0.501278	0.2	0.095	1.26E-64	Skin_Macr	ZC3HAV1
C3orf58	2.56E-68	0.464145	0.139	0.056	4.08E-64	Skin_Macr	C3orf58
CTTNBP2N	2.96E-68	0.500304	0.214	0.106	4.71E-64	Skin_Macr	CTTNBP2NL
PTMS6	3.49E-68	0.35673	0.398	0.241	5.56E-64	Skin_Macr	PTMS
MYL63	3.61E-68	0.253994	0.914	0.803	5.75E-64	Skin_Macr	MYL6
TGIF11	3.91E-68	0.483977	0.33	0.193	6.24E-64	Skin_Macr	TGIF1
IKZF14	5.65E-68	0.2559	0.111	0.038	9.01E-64	Skin_Macr	IKZF1
WSB15	8.24E-68	0.53614	0.389	0.247	1.31E-63	Skin_Macr	WSB1
CAMK1D3	9.38E-68	0.305266	0.112	0.039	1.5E-63	Skin_Macr	CAMK1D
KPNA21	1.67E-67	0.29985	0.218	0.104	2.66E-63	Skin_Macr	KPNA2
RBM393	3.92E-67	0.476329	0.557	0.407	6.25E-63	Skin_Macr	RBM39
ATP6V0B5	7.82E-66	0.4039	0.54	0.384	1.25E-61	Skin_Macr	ATP6V0B
MAP2K3	8.56E-66	0.417961	0.38	0.238	1.37E-61	Skin_Macr	MAP2K3
CLEC2B5	9.61E-66	0.305377	0.291	0.153	1.53E-61	Skin_Macr	CLEC2B
PTPN121	1.04E-65	0.519244	0.211	0.105	1.66E-61	Skin_Macr	PTPN12
VDR	1.88E-65	0.267769	0.081	0.024	3E-61	Skin_Macr	VDR
HNRNPA01	5.26E-65	0.334489	0.5	0.331	8.39E-61	Skin_Macr	HNRNPA0
RHEB1	5.62E-65	0.550868	0.475	0.335	8.96E-61	Skin_Macr	RHEB
RNF145	2.36E-64	0.559069	0.359	0.225	3.76E-60	Skin_Macr	RNF145
YWHAZ1	3.01E-64	0.372751	0.677	0.506	4.81E-60	Skin_Macr	YWHAZ
RUNX12	6.82E-64	0.406893	0.205	0.101	1.09E-59	Skin_Macr	RUNX1
NAB1	8.1E-64	0.394515	0.143	0.06	1.29E-59	Skin_Macr	NAB1
MALT1	9.56E-64	0.446402	0.169	0.077	1.52E-59	Skin_Macr	MALT1
SUPT4H1	2.46E-63	0.475965	0.371	0.24	3.93E-59	Skin_Macr	SUPT4H1
VPS292	3.01E-63	0.487549	0.409	0.279	4.81E-59	Skin_Macr	VPS29
TIPIN1	3.18E-63	0.403132	0.162	0.072	5.07E-59	Skin_Macr	TIPIN
H2AFY	3.57E-63	0.426157	0.469	0.322	5.69E-59	Skin_Macr	H2AFY
CDK2	5.33E-63	0.278165	0.085	0.027	8.5E-59	Skin_Macr	CDK2

B3GNT5	7.46E-63	0.532761	0.185	0.09	1.19E-58	Skin_Macr	B3GNT5
NFKBIZ	1.39E-62	0.489531	0.379	0.242	2.22E-58	Skin_Macr	NFKBIZ
CHMP1B	2.1E-62	0.646127	0.453	0.308	3.35E-58	Skin_Macr	CHMP1B
CHD13	2.55E-62	0.454549	0.241	0.129	4.07E-58	Skin_Macr	CHD1
SLFN112	2.59E-62	0.307476	0.092	0.03	4.13E-58	Skin_Macr	SLFN11
GLRX2	4.89E-62	0.429615	0.349	0.21	7.79E-58	Skin_Macr	GLRX
THEMIS22	5.93E-62	0.250961	0.109	0.039	9.45E-58	Skin_Macr	THEMIS2
CAPZA21	7.24E-62	0.571392	0.407	0.276	1.15E-57	Skin_Macr	CAPZA2
YWHAE	7.44E-62	0.506991	0.532	0.394	1.19E-57	Skin_Macr	YWHAE
TPI11	8.66E-62	0.372725	0.68	0.531	1.38E-57	Skin_Macr	TPI1
UNC1191	2.18E-61	0.346712	0.157	0.07	3.47E-57	Skin_Macr	UNC119
INHBA1	2.85E-61	0.364302	0.075	0.022	4.55E-57	Skin_Macr	INHBA
MKNK24	3.09E-61	0.503754	0.218	0.115	4.92E-57	Skin_Macr	MKNK2
SRSF33	3.61E-61	0.38657	0.584	0.43	5.76E-57	Skin_Macr	SRSF3
GDI21	4.27E-61	0.476154	0.394	0.265	6.8E-57	Skin_Macr	GDI2
NFIL3	4.44E-61	0.312529	0.138	0.057	7.09E-57	Skin_Macr	NFIL3
CREBL21	4.83E-61	0.407529	0.155	0.069	7.7E-57	Skin_Macr	CREBL2
TRPS14	5.46E-61	0.478914	0.178	0.086	8.71E-57	Skin_Macr	TRPS1
RAP2B1	5.75E-61	0.504777	0.262	0.145	9.17E-57	Skin_Macr	RAP2B
ITGA53	1.07E-60	0.255349	0.106	0.038	1.71E-56	Skin_Macr	ITGA5
UBAC21	2.34E-60	0.480368	0.202	0.103	3.73E-56	Skin_Macr	UBAC2
RNASEK1	2.48E-60	0.276483	0.094	0.032	3.96E-56	Skin_Macr	RNASEK
TBCA3	8.54E-60	0.424986	0.488	0.348	1.36E-55	Skin_Macr	TBCA
RHBDF21	9.38E-60	0.295346	0.079	0.025	1.5E-55	Skin_Macr	RHBDF2
TOB21	9.56E-60	0.436902	0.197	0.098	1.52E-55	Skin_Macr	TOB2
PPP1R15B	2.32E-59	0.487249	0.265	0.15	3.7E-55	Skin_Macr	PPP1R15B
ACTR22	5.05E-59	0.476914	0.435	0.303	8.05E-55	Skin_Macr	ACTR2
RPS6KA35	7.12E-59	0.447803	0.188	0.093	1.14E-54	Skin_Macr	RPS6KA3
RNF137	7.62E-59	0.512423	0.277	0.164	1.22E-54	Skin_Macr	RNF13
GRN4	2.33E-57	0.367734	0.449	0.309	3.71E-53	Skin_Macr	GRN
SUMO11	3.41E-57	0.465766	0.475	0.344	5.43E-53	Skin_Macr	SUMO1
ATP6V1C1	6.13E-57	0.539387	0.172	0.085	9.77E-53	Skin_Macr	ATP6V1C1
RAB21	6.57E-57	0.50151	0.277	0.163	1.05E-52	Skin_Macr	RAB21
KLF10	1E-56	0.522857	0.248	0.139	1.6E-52	Skin_Macr	KLF10
PCBP2	1.43E-56	0.355186	0.565	0.417	2.27E-52	Skin_Macr	PCBP2
DNAJB12	1.62E-56	1.0651	0.558	0.427	2.59E-52	Skin_Macr	DNAJB1
DENND1B1	1.67E-56	0.431929	0.149	0.068	2.66E-52	Skin_Macr	DENND1B
SEC11A4	2.26E-56	0.367587	0.453	0.312	3.6E-52	Skin_Macr	SEC11A
GSTO13	2.6E-56	0.330177	0.342	0.21	4.15E-52	Skin_Macr	GSTO1
YY11	2.64E-56	0.513411	0.343	0.224	4.22E-52	Skin_Macr	YY1
CLK11	3.2E-56	0.509516	0.377	0.249	5.1E-52	Skin_Macr	CLK1
GLUD11	3.29E-56	0.527366	0.272	0.161	5.24E-52	Skin_Macr	GLUD1
PPP1R15A	3.57E-56	0.44914	0.62	0.445	5.69E-52	Skin_Macr	PPP1R15A
CTSH3	3.63E-56	0.441959	0.356	0.235	5.79E-52	Skin_Macr	CTSH
GADD45G1	4.13E-56	0.488291	0.147	0.066	6.59E-52	Skin_Macr	GADD45G
FMNL22	1.76E-55	0.3172	0.102	0.038	2.81E-51	Skin_Macr	FMNL2
MAP7D1	6.51E-55	0.373186	0.211	0.11	1.04E-50	Skin_Macr	MAP7D1

MATR31	7.42E-55	0.320476	0.219	0.113	1.18E-50	Skin_Macr	MATR3
TMEM106/	1.01E-54	0.250229	0.07	0.021	1.61E-50	Skin_Macr	TMEM106A
NAAA1	1.13E-54	0.355723	0.177	0.086	1.8E-50	Skin_Macr	NAAA
BAZ1A1	1.22E-54	0.431349	0.371	0.242	1.95E-50	Skin_Macr	BAZ1A
PRPF38B2	3.33E-54	0.443305	0.309	0.19	5.31E-50	Skin_Macr	PRPF38B
RARA1	6.49E-54	0.28596	0.128	0.054	1.04E-49	Skin_Macr	RARA
COX171	9.27E-54	0.593482	0.389	0.273	1.48E-49	Skin_Macr	COX17
PRKD3	1.65E-53	0.372606	0.16	0.077	2.63E-49	Skin_Macr	PRKD3
TWISTNB	3.59E-53	0.649012	0.181	0.092	5.73E-49	Skin_Macr	TWISTNB
TFRC2	1.17E-52	0.466917	0.123	0.052	1.86E-48	Skin_Macr	TFRC
BCL3	1.91E-52	0.400662	0.193	0.099	3.05E-48	Skin_Macr	BCL3
AC009501.	2.07E-52	0.251655	0.089	0.032	3.31E-48	Skin_Macr	AC009501.4
SRRM14	2.32E-52	0.498525	0.445	0.325	3.7E-48	Skin_Macr	SRRM1
ERN12	2.35E-52	0.406322	0.129	0.056	3.75E-48	Skin_Macr	ERN1
SH3KBP12	3.63E-52	0.277504	0.178	0.086	5.78E-48	Skin_Macr	SH3KBP1
TRIB12	5.87E-52	0.30949	0.112	0.045	9.36E-48	Skin_Macr	TRIB1
POLR2L	8.59E-52	0.295576	0.632	0.469	1.37E-47	Skin_Macr	POLR2L
CBWD21	1.04E-51	0.389442	0.116	0.048	1.66E-47	Skin_Macr	CBWD2
RAN3	1.93E-51	0.283059	0.518	0.364	3.08E-47	Skin_Macr	RAN
PBX2	3.51E-51	0.255792	0.106	0.042	5.6E-47	Skin_Macr	PBX2
ENO12	4.38E-51	0.281762	0.556	0.409	6.98E-47	Skin_Macr	ENO1
DCK1	7.27E-51	0.314363	0.134	0.059	1.16E-46	Skin_Macr	DCK
HECA1	7.63E-51	0.48945	0.249	0.145	1.22E-46	Skin_Macr	HECA
FAM102B2	1.52E-50	0.316321	0.082	0.029	2.42E-46	Skin_Macr	FAM102B
CCDC1075	1.53E-50	0.396326	0.285	0.174	2.43E-46	Skin_Macr	CCDC107
VDAC11	2.53E-50	0.445812	0.354	0.239	4.03E-46	Skin_Macr	VDAC1
SRGAP21	3.39E-50	0.320146	0.116	0.048	5.41E-46	Skin_Macr	SRGAP2
EIF4A31	3.94E-50	0.491986	0.308	0.197	6.28E-46	Skin_Macr	EIF4A3
CTSB1	4.53E-50	0.600076	0.431	0.316	7.23E-46	Skin_Macr	CTSB
SH3BP52	1.39E-49	0.355596	0.211	0.113	2.22E-45	Skin_Macr	SH3BP5
RAB322	2.28E-49	0.321341	0.174	0.087	3.64E-45	Skin_Macr	RAB32
ATXN12	3.68E-49	0.35622	0.148	0.07	5.88E-45	Skin_Macr	ATXN1
BACH11	6.52E-49	0.441225	0.195	0.105	1.04E-44	Skin_Macr	BACH1
PAN31	1.03E-48	0.403467	0.16	0.079	1.65E-44	Skin_Macr	PAN3
SNCA4	1.36E-48	0.25933	0.108	0.044	2.17E-44	Skin_Macr	SNCA
GNA15	1.42E-48	0.26765	0.273	0.158	2.27E-44	Skin_Macr	GNA15
RASSF5	3.75E-48	0.296232	0.218	0.119	5.98E-44	Skin_Macr	RASSF5
ELMSAN11	4.19E-48	0.357863	0.157	0.077	6.68E-44	Skin_Macr	ELMSAN1
STARD71	5.98E-48	0.376686	0.177	0.092	9.54E-44	Skin_Macr	STARD7
CLN8	7.53E-48	0.464763	0.214	0.121	1.2E-43	Skin_Macr	CLN8
HMGN21	1.03E-47	0.561327	0.442	0.326	1.64E-43	Skin_Macr	HMGN2
ELOVL51	1.3E-47	0.343529	0.197	0.106	2.07E-43	Skin_Macr	ELOVL5
YWHAG	2.47E-47	0.456456	0.34	0.228	3.94E-43	Skin_Macr	YWHAG
CHORDC1	3.31E-47	0.406613	0.185	0.098	5.28E-43	Skin_Macr	CHORDC1
LTC4S2	1.07E-46	0.263154	0.084	0.031	1.71E-42	Skin_Macr	LTC4S
NOTCH2NI	1.86E-46	0.308336	0.168	0.085	2.96E-42	Skin_Macr	NOTCH2NL
IRF2BP22	2.52E-46	0.467942	0.365	0.256	4.02E-42	Skin_Macr	IRF2BP2

FILIP1L4	2.85E-46	0.309774	0.167	0.083	4.55E-42	Skin_Macr	FILIP1L
RNF1681	5.05E-46	0.355187	0.181	0.095	8.05E-42	Skin_Macr	RNF168
RELB1	1.38E-45	0.28942	0.137	0.064	2.21E-41	Skin_Macr	RELB
SPG21	2.61E-45	0.461315	0.251	0.155	4.16E-41	Skin_Macr	SPG21
MRPL23	3.44E-45	0.28362	0.323	0.209	5.49E-41	Skin_Macr	MRPL23
PCNP1	4.19E-45	0.345515	0.374	0.258	6.68E-41	Skin_Macr	PCNP
RAP1A1	4.79E-45	0.362882	0.385	0.272	7.63E-41	Skin_Macr	RAP1A
ZMIZ12	2.07E-44	0.380978	0.153	0.077	3.3E-40	Skin_Macr	ZMIZ1
SIPA1L1	2.19E-44	0.409321	0.124	0.058	3.49E-40	Skin_Macr	SIPA1L1
ANP32E1	2.32E-44	0.346104	0.18	0.095	3.7E-40	Skin_Macr	ANP32E
ATF42	5.66E-43	0.32418	0.456	0.335	9.03E-39	Skin_Macr	ATF4
MKNK11	5.77E-43	0.338886	0.117	0.053	9.2E-39	Skin_Macr	MKNK1
HNRNPA33	7.28E-43	0.337724	0.482	0.362	1.16E-38	Skin_Macr	HNRNPA3
RNH13	2.14E-42	0.281249	0.429	0.312	3.42E-38	Skin_Macr	RNH1
ARF5	3.13E-42	0.319416	0.326	0.22	4.99E-38	Skin_Macr	ARF5
SNX8	4.75E-42	0.341627	0.137	0.067	7.57E-38	Skin_Macr	SNX8
CMIP1	1.07E-41	0.444612	0.197	0.113	1.7E-37	Skin_Macr	CMIP
ATP6V0D1	1.08E-41	0.440743	0.298	0.201	1.72E-37	Skin_Macr	ATP6V0D1
KLF42	2.13E-41	0.408678	0.338	0.224	3.4E-37	Skin_Macr	KLF4
B3GNT71	2.64E-41	0.271915	0.069	0.024	4.21E-37	Skin_Macr	B3GNT7
DUOX1	3.15E-41	0.297554	0.076	0.029	5.02E-37	Skin_Macr	DUOX1
MRPS62	8.55E-41	0.409843	0.279	0.179	1.36E-36	Skin_Macr	MRPS6
SEC61G4	1.43E-40	0.292597	0.546	0.412	2.28E-36	Skin_Macr	SEC61G
EPB41L33	1.77E-40	0.378222	0.121	0.057	2.82E-36	Skin_Macr	EPB41L3
USP37	3.41E-40	0.394864	0.191	0.11	5.43E-36	Skin_Macr	USP3
PLEKHA22	4.49E-40	0.333775	0.11	0.049	7.16E-36	Skin_Macr	PLEKHA2
TACC15	4.85E-40	0.336905	0.266	0.164	7.74E-36	Skin_Macr	TACC1
KIAA0101	5.52E-40	0.301292	0.073	0.027	8.8E-36	Skin_Macr	KIAA0101
SIAH1	8.68E-40	0.459402	0.173	0.097	1.38E-35	Skin_Macr	SIAH1
CBX32	2.08E-39	0.320835	0.302	0.2	3.31E-35	Skin_Macr	CBX3
TMEM39A	2.5E-39	0.285743	0.104	0.047	3.99E-35	Skin_Macr	TMEM39A
GAS66	3.15E-39	0.252258	0.174	0.094	5.02E-35	Skin_Macr	GAS6
PRCP1	3.53E-39	0.372297	0.207	0.123	5.64E-35	Skin_Macr	PRCP
RAB7A	4.62E-39	0.362531	0.484	0.37	7.37E-35	Skin_Macr	RAB7A
SYAP1	5.01E-39	0.574853	0.336	0.243	7.99E-35	Skin_Macr	SYAP1
CELF24	8.25E-39	0.269099	0.226	0.131	1.32E-34	Skin_Macr	CELF2
CYLD2	1.01E-38	0.399825	0.207	0.123	1.61E-34	Skin_Macr	CYLD
USP361	1.8E-38	0.348344	0.127	0.062	2.86E-34	Skin_Macr	USP36
SMG12	1.93E-38	0.392549	0.201	0.119	3.08E-34	Skin_Macr	SMG1
FOXP14	4.57E-38	0.444196	0.314	0.217	7.29E-34	Skin_Macr	FOXP1
SAR1A1	5.77E-38	0.573459	0.319	0.23	9.2E-34	Skin_Macr	SAR1A
SFT2D13	7.46E-38	0.470929	0.291	0.202	1.19E-33	Skin_Macr	SFT2D1
FAM110A	8.39E-38	0.340392	0.122	0.06	1.34E-33	Skin_Macr	FAM110A
CYTH11	2.13E-37	0.356954	0.227	0.139	3.39E-33	Skin_Macr	CYTH1
AGAP3	2.93E-37	0.273025	0.088	0.037	4.67E-33	Skin_Macr	AGAP3
BRI36	4.73E-37	0.39162	0.452	0.344	7.54E-33	Skin_Macr	BRI3
GATM4	4.79E-37	0.476499	0.141	0.076	7.63E-33	Skin_Macr	GATM

CERS61	7.59E-37	0.350001	0.097	0.043	1.21E-32	Skin_Macr	CERS6
RBX14	7.8E-37	0.344978	0.453	0.347	1.24E-32	Skin_Macr	RBX1
IER51	2.05E-36	0.334213	0.316	0.208	3.26E-32	Skin_Macr	IER5
RNF241	7.62E-36	0.297553	0.127	0.064	1.21E-31	Skin_Macr	RNF24
GRAMD1A	9.34E-36	0.260642	0.145	0.076	1.49E-31	Skin_Macr	GRAMD1A
NUMB	1.43E-35	0.402091	0.194	0.117	2.28E-31	Skin_Macr	NUMB
MAN1A13	2.29E-35	0.2991	0.158	0.085	3.65E-31	Skin_Macr	MAN1A1
MAP3K25	3.09E-35	0.384684	0.201	0.123	4.92E-31	Skin_Macr	MAP3K2
TRA2B	3.97E-35	0.456154	0.385	0.287	6.32E-31	Skin_Macr	TRA2B
ALDH26	1.29E-34	0.34697	0.199	0.122	2.05E-30	Skin_Macr	ALDH2
DENND4A2	1.92E-34	0.265432	0.09	0.04	3.07E-30	Skin_Macr	DENND4A
BNIP3L1	2.4E-34	0.50673	0.379	0.295	3.83E-30	Skin_Macr	BNIP3L
UBE2A	4.08E-34	0.420533	0.299	0.211	6.5E-30	Skin_Macr	UBE2A
EIF5A4	8.63E-34	0.35938	0.373	0.276	1.38E-29	Skin_Macr	EIF5A
UBE2W	1.13E-33	0.458028	0.193	0.119	1.8E-29	Skin_Macr	UBE2W
TES1	2.2E-33	0.372375	0.203	0.125	3.51E-29	Skin_Macr	TES
RNF2	3.47E-33	0.304966	0.105	0.051	5.53E-29	Skin_Macr	RNF2
ACOT71	4.49E-33	0.544327	0.099	0.046	7.17E-29	Skin_Macr	ACOT7
METTL21A	7.99E-33	0.356808	0.135	0.073	1.27E-28	Skin_Macr	METTL21A
MOB1B2	1.1E-32	0.266155	0.078	0.033	1.76E-28	Skin_Macr	MOB1B
SRSF91	1.56E-32	0.384969	0.444	0.352	2.49E-28	Skin_Macr	SRSF9
RAP2C1	2.36E-32	0.262807	0.106	0.052	3.76E-28	Skin_Macr	RAP2C
ACP52	4.16E-32	0.299648	0.109	0.053	6.64E-28	Skin_Macr	ACP5
SLC36A4	4.93E-32	0.301558	0.091	0.042	7.87E-28	Skin_Macr	SLC36A4
C16orf87	8.11E-32	0.296874	0.136	0.073	1.29E-27	Skin_Macr	C16orf87
TCOF11	1.03E-31	0.310351	0.096	0.046	1.65E-27	Skin_Macr	TCOF1
RNASE12	2.14E-31	0.816169	0.16	0.092	3.41E-27	Skin_Macr	RNASE1
MMD1	2.44E-31	0.276928	0.068	0.028	3.88E-27	Skin_Macr	MMD
CLDND11	3.29E-31	0.444895	0.273	0.189	5.25E-27	Skin_Macr	CLDND1
COX7B6	3.3E-31	0.255796	0.51	0.407	5.27E-27	Skin_Macr	COX7B
CDC42SE1	3.66E-31	0.628975	0.277	0.197	5.84E-27	Skin_Macr	CDC42SE1
MOB41	4.22E-31	0.330732	0.159	0.091	6.73E-27	Skin_Macr	MOB4
SLC4A73	4.44E-31	0.336534	0.114	0.058	7.08E-27	Skin_Macr	SLC4A7
CMPK13	4.76E-31	0.270211	0.397	0.299	7.6E-27	Skin_Macr	CMPK1
FRMD4B7	1.2E-30	0.406161	0.196	0.126	1.91E-26	Skin_Macr	FRMD4B
UBE2S3	1.21E-30	0.323401	0.207	0.13	1.93E-26	Skin_Macr	UBE2S
ZFYVE162	1.86E-30	0.336993	0.132	0.072	2.96E-26	Skin_Macr	ZFYVE16
ETF1	2.63E-30	0.382967	0.25	0.171	4.19E-26	Skin_Macr	ETF1
DNAJA12	3.41E-30	0.422372	0.454	0.359	5.44E-26	Skin_Macr	DNAJA1
MFSD11	3.58E-30	0.43009	0.166	0.101	5.71E-26	Skin_Macr	MFSD1
FUS8	3.82E-30	0.312927	0.371	0.275	6.09E-26	Skin_Macr	FUS
CD473	8.38E-30	0.270191	0.319	0.228	1.34E-25	Skin_Macr	CD47
AKIRIN21	9.42E-30	0.424062	0.258	0.18	1.5E-25	Skin_Macr	AKIRIN2
DDX3X3	1.03E-29	0.353608	0.387	0.299	1.64E-25	Skin_Macr	DDX3X
BNIP22	1.66E-29	0.335142	0.196	0.123	2.64E-25	Skin_Macr	BNIP2
MAT2A3	1.7E-29	0.378134	0.224	0.149	2.72E-25	Skin_Macr	MAT2A
TRAM12	2.15E-29	0.341262	0.386	0.298	3.42E-25	Skin_Macr	TRAM1

UBE2E2	2.29E-29	0.331979	0.139	0.078	3.66E-25	Skin_Macr	UBE2E2
ANKRD10	3.62E-29	0.281452	0.183	0.111	5.77E-25	Skin_Macr	ANKRD10
RNF1491	4.71E-29	0.441618	0.232	0.158	7.51E-25	Skin_Macr	RNF149
PPP3CA4	7.75E-29	0.330699	0.232	0.154	1.24E-24	Skin_Macr	PPP3CA
HNRNPH2	3.91E-28	0.270294	0.142	0.08	6.23E-24	Skin_Macr	HNRNPH2
IRF2BPL2	4.64E-28	0.310208	0.134	0.075	7.4E-24	Skin_Macr	IRF2BPL
KCNN44	1.42E-27	0.294264	0.139	0.08	2.26E-23	Skin_Macr	KCNN4
APMAP	1.94E-27	0.267108	0.169	0.103	3.1E-23	Skin_Macr	APMAP
ZBTB10	4.47E-27	0.435347	0.13	0.075	7.13E-23	Skin_Macr	ZBTB10
MDM45	7.32E-27	0.464668	0.203	0.136	1.17E-22	Skin_Macr	MDM4
PRDM11	8.49E-27	0.312417	0.255	0.168	1.35E-22	Skin_Macr	PRDM1
ADAM17	1.24E-26	0.316334	0.149	0.088	1.97E-22	Skin_Macr	ADAM17
RAB5C	1.27E-26	0.31984	0.335	0.254	2.02E-22	Skin_Macr	RAB5C
DNAJB92	1.46E-26	0.301084	0.287	0.206	2.33E-22	Skin_Macr	DNAJB9
HNMT2	1.65E-26	0.336714	0.171	0.108	2.63E-22	Skin_Macr	HNMT
UGCG	1.71E-26	0.333796	0.176	0.11	2.72E-22	Skin_Macr	UGCG
ZBTB12	1.79E-26	0.275767	0.13	0.073	2.85E-22	Skin_Macr	ZBTB1
P4HA11	5.9E-26	0.343967	0.14	0.082	9.41E-22	Skin_Macr	P4HA1
NQO21	6.65E-26	0.390399	0.173	0.111	1.06E-21	Skin_Macr	NQO2
LAMTOR3	1.25E-25	0.369589	0.169	0.108	1.99E-21	Skin_Macr	LAMTOR3
PICALM	1.36E-25	0.285609	0.33	0.246	2.18E-21	Skin_Macr	PICALM
IPO72	1.92E-25	0.357175	0.164	0.103	3.06E-21	Skin_Macr	IPO7
RABGGTB1	2.06E-25	0.309027	0.161	0.1	3.29E-21	Skin_Macr	RABGGTB
RCC2	3.6E-25	0.306078	0.125	0.072	5.74E-21	Skin_Macr	RCC2
SLC20A11	3.74E-25	0.340213	0.127	0.072	5.96E-21	Skin_Macr	SLC20A1
UPF2	6.2E-25	0.425585	0.214	0.146	9.89E-21	Skin_Macr	UPF2
RQCD1	7.74E-25	0.319044	0.116	0.065	1.23E-20	Skin_Macr	RQCD1
VMA21	1.85E-24	0.299374	0.166	0.105	2.95E-20	Skin_Macr	VMA21
STARD3NL	1.87E-24	0.37644	0.14	0.085	2.98E-20	Skin_Macr	STARD3NL
CACUL1	2.55E-24	0.435543	0.223	0.157	4.06E-20	Skin_Macr	CACUL1
RAPH11	3.01E-24	0.287233	0.098	0.052	4.79E-20	Skin_Macr	RAPH1
TSC22D2	3.48E-24	0.340337	0.168	0.106	5.56E-20	Skin_Macr	TSC22D2
TMEM14C	6.51E-24	0.293428	0.33	0.254	1.04E-19	Skin_Macr	TMEM14C
DYNC1H15	1.74E-23	0.314593	0.202	0.138	2.78E-19	Skin_Macr	DYNC1H1
CPNE81	2.91E-23	0.251437	0.081	0.04	4.65E-19	Skin_Macr	CPNE8
RHOQ5	4.02E-23	0.293368	0.14	0.085	6.41E-19	Skin_Macr	RHOQ
EFHD21	8.96E-23	0.271123	0.297	0.219	1.43E-18	Skin_Macr	EFHD2
PABPN13	1.12E-22	0.301957	0.215	0.149	1.78E-18	Skin_Macr	PABPN1
PPP2CA	2.46E-22	0.325576	0.302	0.231	3.92E-18	Skin_Macr	PPP2CA
PXDC1	2.64E-22	0.325169	0.137	0.083	4.21E-18	Skin_Macr	PXDC1
ATP6V1H	2.73E-22	0.361387	0.169	0.111	4.35E-18	Skin_Macr	ATP6V1H
ACER3	9.59E-22	0.275537	0.127	0.076	1.53E-17	Skin_Macr	ACER3
PIGA	1.48E-21	0.262304	0.082	0.042	2.36E-17	Skin_Macr	PIGA
CCL17	1.52E-21	1.195897	0.042	0.016	2.42E-17	Skin_Macr	CCL17
MORF4L2	3.15E-21	0.370756	0.366	0.3	5.02E-17	Skin_Macr	MORF4L2
RPS19BP1	4.46E-21	0.269894	0.349	0.278	7.11E-17	Skin_Macr	RPS19BP1
METTL151	1.91E-20	0.296012	0.088	0.047	3.04E-16	Skin_Macr	METTL15

LYST3	2.21E-20	0.328347	0.144	0.092	3.53E-16	Skin_Macr	LYST
SEPP12	2.88E-20	0.901517	0.328	0.264	4.59E-16	Skin_Macr	SEPP1
FAM133B4	3.13E-20	0.341936	0.262	0.197	4.99E-16	Skin_Macr	FAM133B
PNPLA8	4.06E-20	0.316334	0.158	0.104	6.47E-16	Skin_Macr	PNPLA8
JOSD11	4.14E-20	0.261265	0.183	0.124	6.6E-16	Skin_Macr	JOSD1
ATP1A18	1.2E-19	0.263519	0.329	0.254	1.92E-15	Skin_Macr	ATP1A1
NSUN62	1.22E-19	0.251795	0.092	0.051	1.94E-15	Skin_Macr	NSUN6
NOP10	1.54E-19	0.2557	0.446	0.367	2.45E-15	Skin_Macr	NOP10
RAB5A	2.1E-19	0.342721	0.267	0.206	3.36E-15	Skin_Macr	RAB5A
C12orf451	5.07E-19	0.281143	0.124	0.077	8.09E-15	Skin_Macr	C12orf45
LRRFIP2	6.02E-19	0.375912	0.213	0.154	9.6E-15	Skin_Macr	LRRFIP2
ARL5A	1.07E-18	0.281503	0.256	0.195	1.7E-14	Skin_Macr	ARL5A
EYA3	1.15E-18	0.28645	0.089	0.05	1.83E-14	Skin_Macr	EYA3
TMX11	6.97E-18	0.257551	0.156	0.104	1.11E-13	Skin_Macr	TMX1
SLC41A2	1.38E-17	0.257323	0.064	0.033	2.2E-13	Skin_Macr	SLC41A2
PTRHD11	2.25E-17	0.250022	0.248	0.191	3.59E-13	Skin_Macr	PTRHD1
CELF11	4.03E-17	0.328796	0.175	0.124	6.43E-13	Skin_Macr	CELF1
DNAJB111	5.55E-17	0.266549	0.22	0.165	8.84E-13	Skin_Macr	DNAJB11
RIT1	1.87E-16	0.408827	0.174	0.125	2.98E-12	Skin_Macr	RIT1
ERGIC15	2.05E-16	0.330786	0.182	0.133	3.28E-12	Skin_Macr	ERGIC1
FBXO11	4.4E-16	0.291714	0.15	0.103	7.02E-12	Skin_Macr	FBXO11
NOTCH21	4.7E-16	0.268382	0.14	0.093	7.49E-12	Skin_Macr	NOTCH2
ITSN22	5.31E-16	0.28419	0.194	0.141	8.46E-12	Skin_Macr	ITSN2
PLEKHA52	5.31E-16	0.386956	0.128	0.085	8.47E-12	Skin_Macr	PLEKHA5
CISD21	5.59E-16	0.351122	0.22	0.17	8.92E-12	Skin_Macr	CISD2
PTEN2	1.36E-15	0.276527	0.177	0.127	2.17E-11	Skin_Macr	PTEN
MAP3K131	1.51E-15	0.349576	0.288	0.24	2.41E-11	Skin_Macr	MAP3K13
NDUFA62	2.12E-15	0.258737	0.376	0.32	3.39E-11	Skin_Macr	NDUFA6
TRA2A1	1.1E-14	0.358644	0.213	0.164	1.75E-10	Skin_Macr	TRA2A
FAM60A2	2.01E-14	0.313402	0.149	0.105	3.21E-10	Skin_Macr	FAM60A
STK38L	3.31E-14	0.253018	0.135	0.092	5.28E-10	Skin_Macr	STK38L
ZMYM22	4.78E-14	0.330857	0.177	0.132	7.62E-10	Skin_Macr	ZMYM2
NAA50	7.28E-14	0.343908	0.247	0.201	1.16E-09	Skin_Macr	NAA50
NMB	8.61E-14	0.359055	0.11	0.073	1.37E-09	Skin_Macr	NMB
CDC264	8.92E-14	0.296115	0.17	0.126	1.42E-09	Skin_Macr	CDC26
WAC	1.53E-13	0.310456	0.259	0.212	2.43E-09	Skin_Macr	WAC
NUDT34	2.74E-13	0.259935	0.144	0.102	4.38E-09	Skin_Macr	NUDT3
CRK1	3.53E-13	0.256187	0.163	0.119	5.64E-09	Skin_Macr	CRK
CCL181	7.44E-13	0.606426	0.039	0.018	1.19E-08	Skin_Macr	CCL18
SCAF117	1.06E-12	0.265593	0.28	0.231	1.69E-08	Skin_Macr	SCAF11
AZIN1	1.15E-12	0.319727	0.216	0.172	1.84E-08	Skin_Macr	AZIN1
JMJD1C1	8.3E-12	0.288067	0.279	0.234	1.32E-07	Skin_Macr	JMJD1C
RHOU2	1.69E-11	0.255267	0.073	0.045	2.69E-07	Skin_Macr	RHOU
NUFIP22	2.48E-11	0.298121	0.193	0.152	3.96E-07	Skin_Macr	NUFIP2
SFT2D22	4.7E-11	0.265124	0.146	0.109	7.5E-07	Skin_Macr	SFT2D2
AREG	1.63E-10	0.290514	0.164	0.125	2.59E-06	Skin_Macr	AREG
MRPL14	3.23E-10	0.263921	0.266	0.227	5.16E-06	Skin_Macr	MRPL14

OSBPL82	4.42E-10	0.253911	0.216	0.176	7.05E-06	Skin_Macr	OSBPL8
PRKAR1A	6.45E-10	0.251297	0.278	0.238	1.03E-05	Skin_Macr	PRKAR1A
MAFG	1.4E-09	0.289623	0.159	0.124	2.23E-05	Skin_Macr	MAFG
TSPYL12	1.86E-09	0.323274	0.189	0.154	2.97E-05	Skin_Macr	TSPYL1
MAFB3	1.87E-09	0.313926	0.122	0.088	2.98E-05	Skin_Macr	MAFB
TRIM13	1.27E-08	0.264248	0.117	0.087	0.000203	Skin_Macr	TRIM13
TAB2	3E-08	0.26028	0.15	0.118	0.000479	Skin_Macr	TAB2
SLC39A85	1.15E-07	0.251991	0.121	0.093	0.001831	Skin_Macr	SLC39A8
HIPK1	1.24E-07	0.25706	0.143	0.114	0.001982	Skin_Macr	HIPK1
HIF1A2	8.97E-07	0.251429	0.194	0.166	0.014304	Skin_Macr	HIF1A
CCL201	1.54E-06	0.620927	0.043	0.027	0.024541	Skin_Macr	CCL20
UFM11	1.95E-05	0.271659	0.25	0.23	0.310935	Skin_Macr	UFM1
CTSL4	0.000225	0.398941	0.124	0.104	1	Skin_Macr	CTSL
CDYL	0.000596	0.250025	0.105	0.088	1	Skin_Macr	CDYL
TAGLN3	0	4.427248	0.965	0.072	0	Skin_Peric	TAGLN
CCL22	0	4.165349	0.791	0.116	0	Skin_Peric	CCL2
IL6	0	4.057122	0.522	0.038	0	Skin_Peric	IL6
ACTA22	0	4.043279	0.877	0.054	0	Skin_Peric	ACTA2
MYL93	0	4.022758	0.945	0.107	0	Skin_Peric	MYL9
C11orf961	0	3.925035	0.833	0.094	0	Skin_Peric	C11orf96
TPM22	0	3.920888	0.885	0.074	0	Skin_Peric	TPM2
CYCS3	0	3.9169	0.771	0.336	0	Skin_Peric	CYCS
RGS162	0	3.663984	0.723	0.08	0	Skin_Peric	RGS16
RGS51	0	3.275257	0.437	0.023	0	Skin_Peric	RGS5
CRISPLD22	0	3.144196	0.665	0.049	0	Skin_Peric	CRISPLD2
ID42	0	3.000704	0.65	0.07	0	Skin_Peric	ID4
GADD45B1	0	2.941242	0.899	0.37	0	Skin_Peric	GADD45B
GEM1	0	2.914592	0.644	0.079	0	Skin_Peric	GEM
MYH112	0	2.882796	0.616	0.031	0	Skin_Peric	MYH11
CALD13	0	2.716176	0.943	0.185	0	Skin_Peric	CALD1
ADIRF3	0	2.673217	0.993	0.428	0	Skin_Peric	ADIRF
MYLK2	0	2.647998	0.622	0.047	0	Skin_Peric	MYLK
ADRA2A1	0	2.632317	0.529	0.013	0	Skin_Peric	ADRA2A
CEBPD	0	2.609727	0.834	0.331	0	Skin_Peric	CEBPD
CPE4	0	2.595965	0.794	0.094	0	Skin_Peric	CPE
GJA42	0	2.508357	0.434	0.012	0	Skin_Peric	GJA4
NDUFA4L2	0	2.472876	0.488	0.07	0	Skin_Peric	NDUFA4L2
ADAMTS12	0	2.417043	0.612	0.054	0	Skin_Peric	ADAMTS1
CCL82	0	2.377971	0.189	0.009	0	Skin_Peric	CCL8
ZNF3312	0	2.344415	0.503	0.1	0	Skin_Peric	ZNF331
ADAMTS41	0	2.315104	0.437	0.027	0	Skin_Peric	ADAMTS4
IGFBP73	0	2.269737	0.978	0.256	0	Skin_Peric	IGFBP7
RRAD2	0	2.243632	0.337	0.031	0	Skin_Peric	RRAD
TPM14	0	2.22956	0.736	0.18	0	Skin_Peric	TPM1
DSTN3	0	2.125298	0.886	0.559	0	Skin_Peric	DSTN
MT2A	0	2.122718	0.9	0.3	0	Skin_Peric	MT2A
RHOB2	0	2.074195	0.602	0.196	0	Skin_Peric	RHOB

MFGE83	0	2.028551	0.655	0.092	0	Skin_Peric	MFGE8
TINAGL13	0	2.014557	0.598	0.076	0	Skin_Peric	TINAGL1
SORBS27	0	2.009444	0.524	0.108	0	Skin_Peric	SORBS2
PDGFA2	0	2.006912	0.403	0.03	0	Skin_Peric	PDGFA
MT1M	0	1.957241	0.563	0.066	0	Skin_Peric	MT1M
AXL4	0	1.956974	0.606	0.092	0	Skin_Peric	AXL
CDKN1A1	0	1.925955	0.745	0.254	0	Skin_Peric	CDKN1A
LBH2	0	1.920628	0.574	0.09	0	Skin_Peric	LBH
CYR613	0	1.919804	0.46	0.109	0	Skin_Peric	CYR61
ACTG22	0	1.919026	0.205	0.013	0	Skin_Peric	ACTG2
MCAM3	0	1.914545	0.512	0.023	0	Skin_Peric	MCAM
BCAM3	0	1.904736	0.517	0.07	0	Skin_Peric	BCAM
HSPA1A6	0	1.888364	0.793	0.32	0	Skin_Peric	HSPA1A
PLN1	0	1.873921	0.377	0.014	0	Skin_Peric	PLN
JUNB1	0	1.852175	0.972	0.687	0	Skin_Peric	JUNB
NOTCH32	0	1.84904	0.506	0.031	0	Skin_Peric	NOTCH3
SYNPO23	0	1.813426	0.427	0.021	0	Skin_Peric	SYNPO2
SNHG152	0	1.812561	0.497	0.106	0	Skin_Peric	SNHG15
NR4A24	0	1.795534	0.638	0.18	0	Skin_Peric	NR4A2
MTHFD23	0	1.793879	0.523	0.094	0	Skin_Peric	MTHFD2
RPL412	0	1.789196	1	0.86	0	Skin_Peric	RPL41
MYC	0	1.77216	0.551	0.124	0	Skin_Peric	MYC
MAP1B3	0	1.765312	0.581	0.083	0	Skin_Peric	MAP1B
FLNA8	0	1.765261	0.574	0.144	0	Skin_Peric	FLNA
PPP1R14A:	0	1.763518	0.439	0.041	0	Skin_Peric	PPP1R14A
SNCG2	0	1.760449	0.497	0.114	0	Skin_Peric	SNCG
NR4A11	0	1.758449	0.721	0.251	0	Skin_Peric	NR4A1
SOCS34	0	1.752347	0.553	0.16	0	Skin_Peric	SOCS3
CAV14	0	1.746332	0.792	0.191	0	Skin_Peric	CAV1
IFITM33	0	1.731832	0.903	0.303	0	Skin_Peric	IFITM3
CALM25	0	1.730616	0.972	0.557	0	Skin_Peric	CALM2
COX4I21	0	1.728633	0.39	0.01	0	Skin_Peric	COX4I2
APOLD12	0	1.696008	0.359	0.027	0	Skin_Peric	APOLD1
INHBA2	0	1.659439	0.189	0.022	0	Skin_Peric	INHBA
LDHA3	0	1.649466	0.837	0.435	0	Skin_Peric	LDHA
ARID5A2	0	1.634171	0.552	0.093	0	Skin_Peric	ARID5A
PDGFRB2	0	1.628459	0.508	0.062	0	Skin_Peric	PDGFRB
NR4A32	0	1.62424	0.4	0.049	0	Skin_Peric	NR4A3
PTP4A32	0	1.5552	0.351	0.028	0	Skin_Peric	PTP4A3
NR2F24	0	1.551797	0.44	0.083	0	Skin_Peric	NR2F2
SPARC4	0	1.550187	0.67	0.156	0	Skin_Peric	SPARC
LHFP4	0	1.544022	0.473	0.082	0	Skin_Peric	LHFP
CSRP23	0	1.536934	0.393	0.077	0	Skin_Peric	CSRP2
PTRF4	0	1.533086	0.626	0.158	0	Skin_Peric	PTRF
FXYD12	0	1.531695	0.535	0.08	0	Skin_Peric	FXYD1
PCSK74	0	1.525005	0.492	0.107	0	Skin_Peric	PCSK7
SLC2A33	0	1.521895	0.55	0.124	0	Skin_Peric	SLC2A3

HES41	0	1.514928	0.611	0.19	0	Skin_Peric	HES4
MIR22HG1	0	1.512847	0.399	0.081	0	Skin_Peric	MIR22HG
BGN3	0	1.478167	0.449	0.063	0	Skin_Peric	BGN
PTMA3	0	1.476179	0.999	0.817	0	Skin_Peric	PTMA
CCDC32	0	1.43953	0.35	0.022	0	Skin_Peric	CCDC3
NTRK23	0	1.439354	0.439	0.049	0	Skin_Peric	NTRK2
ISYNA11	0	1.431782	0.395	0.055	0	Skin_Peric	ISYNA1
LGALS17	0	1.427643	0.983	0.403	0	Skin_Peric	LGALS1
KLF93	0	1.420607	0.438	0.102	0	Skin_Peric	KLF9
RPS4Y15	0	1.414578	0.784	0.267	0	Skin_Peric	RPS4Y1
C9orf34	0	1.407516	0.45	0.094	0	Skin_Peric	C9orf3
HSPB12	0	1.400368	0.971	0.56	0	Skin_Peric	HSPB1
RERG2	0	1.399005	0.307	0.02	0	Skin_Peric	RERG
PRKCDBP3	0	1.384071	0.512	0.124	0	Skin_Peric	PRKCDBP
ANGPT22	0	1.37885	0.318	0.025	0	Skin_Peric	ANGPT2
PHLDB22	0	1.372675	0.298	0.05	0	Skin_Peric	PHLDB2
FRZB1	0	1.323843	0.296	0.009	0	Skin_Peric	FRZB
TNS15	0	1.323698	0.382	0.055	0	Skin_Peric	TNS1
ZFHX34	0	1.302542	0.398	0.086	0	Skin_Peric	ZFHX3
EPAS12	0	1.289491	0.446	0.086	0	Skin_Peric	EPAS1
TIMP34	0	1.288295	0.665	0.16	0	Skin_Peric	TIMP3
LGI41	0	1.270908	0.28	0.008	0	Skin_Peric	LGI4
TGFBI4	0	1.244369	0.334	0.06	0	Skin_Peric	TGFBI
EBF13	0	1.239384	0.388	0.058	0	Skin_Peric	EBF1
PGAP11	0	1.220798	0.214	0.025	0	Skin_Peric	PGAP1
S100A46	0	1.203813	0.967	0.459	0	Skin_Peric	S100A4
COL18A13	0	1.201605	0.353	0.07	0	Skin_Peric	COL18A1
SLFN113	0	1.187313	0.249	0.029	0	Skin_Peric	SLFN11
JAG13	0	1.163774	0.324	0.047	0	Skin_Peric	JAG1
COL4A24	0	1.152077	0.308	0.052	0	Skin_Peric	COL4A2
IGFBP53	0	1.146381	0.624	0.131	0	Skin_Peric	IGFBP5
NFIL31	0	1.124344	0.323	0.055	0	Skin_Peric	NFIL3
RCAN23	0	1.12098	0.293	0.027	0	Skin_Peric	RCAN2
MAP3K7CL	0	1.082161	0.22	0.012	0	Skin_Peric	MAP3K7CL
VIM9	0	1.08128	0.993	0.488	0	Skin_Peric	VIM
PLEKHA41	0	1.023709	0.245	0.026	0	Skin_Peric	PLEKHA4
C1QTNF12	0	1.014317	0.262	0.032	0	Skin_Peric	C1QTNF1
ITGA71	0	1.003396	0.245	0.007	0	Skin_Peric	ITGA7
EFHD12	0	0.993543	0.266	0.026	0	Skin_Peric	EFHD1
RAMP11	0	0.98344	0.247	0.022	0	Skin_Peric	RAMP1
RASL11A1	0	0.975074	0.223	0.008	0	Skin_Peric	RASL11A
SYNM2	0	0.973815	0.198	0.017	0	Skin_Peric	SYNM
MRGPRF3	0	0.971722	0.245	0.023	0	Skin_Peric	MRGPRF
KANK24	0	0.965955	0.267	0.041	0	Skin_Peric	KANK2
SEPT41	0	0.952022	0.214	0.022	0	Skin_Peric	SEPT4
FADS31	0	0.951607	0.258	0.039	0	Skin_Peric	FADS3
GUCY1A32	0	0.929986	0.252	0.031	0	Skin_Peric	GUCY1A3

MSRB34	0	0.922226	0.247	0.032	0	Skin_Perici	MSRB3
AOC32	0	0.849772	0.208	0.01	0	Skin_Perici	AOC3
PDE5A2	0	0.836802	0.198	0.019	0	Skin_Perici	PDE5A
MRVI12	0	0.762079	0.177	0.015	0	Skin_Perici	MRVI1
DMPK1	0	0.746876	0.182	0.019	0	Skin_Perici	DMPK
ENPEP1	0	0.710781	0.153	0.006	0	Skin_Perici	ENPEP
RASL122	0	0.706848	0.163	0.01	0	Skin_Perici	RASL12
AVPR1A1	0	0.670415	0.115	0.002	0	Skin_Perici	AVPR1A
CAP22	0	0.615405	0.142	0.009	0	Skin_Perici	CAP2
RBPMS21	0	0.614162	0.143	0.008	0	Skin_Perici	RBPMS2
ATP1B21	0	0.601175	0.129	0.006	0	Skin_Perici	ATP1B2
EGFL6	0	0.573687	0.099	0.001	0	Skin_Perici	EGFL6
KCNA5	0	0.571546	0.115	0.001	0	Skin_Perici	KCNA5
KCNJ82	0	0.56445	0.11	0.006	0	Skin_Perici	KCNJ8
HRH21	0	0.542558	0.123	0.007	0	Skin_Perici	HRH2
RBM241	0	0.533814	0.106	0.005	0	Skin_Perici	RBM24
LDB31	0	0.494143	0.107	0.007	0	Skin_Perici	LDB3
PDE3A1	0	0.471463	0.103	0.003	0	Skin_Perici	PDE3A
HRC1	0	0.395444	0.082	0.002	0	Skin_Perici	HRC
NBEAL12	2.2E-307	1.20642	0.798	0.314	3.5E-303	Skin_Perici	NBEAL1
DDIT42	3.9E-307	2.389236	0.638	0.238	6.3E-303	Skin_Perici	DDIT4
EIF4A12	3E-305	1.405246	0.815	0.347	4.8E-301	Skin_Perici	EIF4A1
ARID5B4	4.1E-304	1.49826	0.691	0.264	6.6E-300	Skin_Perici	ARID5B
COL6A23	9.3E-302	0.845554	0.583	0.155	1.5E-297	Skin_Perici	COL6A2
EIF4A32	2.6E-301	2.383458	0.563	0.195	4.1E-297	Skin_Perici	EIF4A3
SELM9	9.2E-297	1.312059	0.8	0.341	1.5E-292	Skin_Perici	SELM
RPL72	5.1E-296	1.103352	0.99	0.795	8.1E-292	Skin_Perici	RPL7
KCNAB11	4.6E-292	0.569333	0.131	0.011	7.4E-288	Skin_Perici	KCNAB1
PPP1R15A1	1.3E-291	1.613904	0.845	0.445	2.1E-287	Skin_Perici	PPP1R15A
SH3BGRL6	1.1E-290	1.38932	0.646	0.239	1.7E-286	Skin_Perici	SH3BGRL
MT1X1	3E-290	0.966764	0.61	0.196	4.8E-286	Skin_Perici	MT1X
RPL3911	2.2E-282	1.123273	0.984	0.673	3.4E-278	Skin_Perici	RPL39
LMNA9	9.7E-282	1.136722	0.823	0.361	1.5E-277	Skin_Perici	LMNA
COX7A16	9.5E-281	1.277298	0.478	0.135	1.5E-276	Skin_Perici	COX7A1
ZNF7033	5.2E-280	0.99424	0.254	0.041	8.3E-276	Skin_Perici	ZNF703
MTRNR2L8	1.7E-277	1.058768	0.746	0.281	2.8E-273	Skin_Perici	MTRNR2L8
PGF2	2E-277	0.81779	0.204	0.027	3.3E-273	Skin_Perici	PGF
ZEB24	5.3E-275	1.099127	0.379	0.085	8.4E-271	Skin_Perici	ZEB2
RPL212	2.5E-274	1.041239	0.997	0.812	3.9E-270	Skin_Perici	RPL21
MPRIP.AS1	2.8E-272	0.615455	0.13	0.012	4.4E-268	Skin_Perici	MPRIP.AS1
MYL64	1.2E-271	1.152068	0.97	0.804	1.9E-267	Skin_Perici	MYL6
FOS7	3.9E-268	1.586597	0.847	0.42	6.2E-264	Skin_Perici	FOS
THBS12	2E-267	1.324971	0.352	0.077	3.1E-263	Skin_Perici	THBS1
TGFB1I14	1.9E-264	0.916719	0.275	0.049	3.1E-260	Skin_Perici	TGFB1I1
A2M4	9.1E-261	0.760051	0.37	0.081	1.5E-256	Skin_Perici	A2M
ID33	2.6E-260	1.211676	0.551	0.168	4.1E-256	Skin_Perici	ID3
UBA21	3E-259	1.171201	0.419	0.112	4.8E-255	Skin_Perici	UBA2

SERPINI14	3.6E-258	0.833484	0.172	0.021	5.7E-254	Skin_Peric	SERPINI1
CEBPB1	1.9E-256	1.158417	0.677	0.268	3E-252	Skin_Peric	CEBPB
SRSF34	6E-252	1.403582	0.787	0.429	9.6E-248	Skin_Peric	SRSF3
SEPT76	2.2E-249	1.39767	0.72	0.335	3.5E-245	Skin_Peric	SEPT7
CH25H1	4.1E-249	0.840087	0.119	0.011	6.6E-245	Skin_Peric	CH25H
GJC11	4E-248	0.590635	0.127	0.012	6.3E-244	Skin_Peric	GJC1
SLC38A112	1.3E-246	0.287586	0.05	0.002	2.1E-242	Skin_Peric	SLC38A11
ARHGEF25	5.6E-246	0.426204	0.106	0.009	8.9E-242	Skin_Peric	ARHGEF25
RARRES23	2.9E-244	0.976913	0.364	0.085	4.6E-240	Skin_Peric	RARRES2
CNRIP14	1.3E-243	0.798612	0.225	0.036	2E-239	Skin_Peric	CNRIP1
CABP11	1.9E-242	0.304922	0.058	0.002	3.1E-238	Skin_Peric	CABP1
HNRNPA11	1.5E-241	1.090518	0.912	0.541	2.3E-237	Skin_Peric	HNRNPA1
C2orf403	7.8E-241	0.801151	0.229	0.037	1.2E-236	Skin_Peric	C2orf40
SBSPON2	7.5E-238	0.622978	0.151	0.018	1.2E-233	Skin_Peric	SBSPON
PGM5.AS1	3.3E-237	0.289464	0.048	0.002	5.3E-233	Skin_Peric	PGM5.AS1
HEY21	7.1E-237	0.690116	0.159	0.02	1.1E-232	Skin_Peric	HEY2
NUDT42	4.5E-236	1.355958	0.461	0.149	7.2E-232	Skin_Peric	NUDT4
PNRC12	9.3E-235	1.384398	0.828	0.507	1.5E-230	Skin_Peric	PNRC1
CSRNP12	3.7E-234	1.287651	0.441	0.134	5.9E-230	Skin_Peric	CSRNP1
SPEG2	6.7E-233	0.566068	0.125	0.013	1.1E-228	Skin_Peric	SPEG
CHD14	1.1E-232	1.408396	0.426	0.128	1.7E-228	Skin_Peric	CHD1
ZNF141	2.4E-229	0.744747	0.156	0.019	3.8E-225	Skin_Peric	ZNF141
NFASC2	6.7E-228	0.452335	0.099	0.008	1.1E-223	Skin_Peric	NFASC
PDK4	1.2E-226	1.643948	0.221	0.038	1.9E-222	Skin_Peric	PDK4
HSPE13	2.2E-226	1.49994	0.711	0.368	3.6E-222	Skin_Peric	HSPE1
HSPB61	2.7E-226	0.619644	0.148	0.018	4.3E-222	Skin_Peric	HSPB6
AEBP12	8.7E-226	0.858133	0.337	0.077	1.4E-221	Skin_Peric	AEBP1
ARHGEF17	9.8E-226	0.554553	0.134	0.015	1.6E-221	Skin_Peric	ARHGEF17
RPL343	5.4E-225	0.824523	0.998	0.918	8.7E-221	Skin_Peric	RPL34
MEF2C6	1.2E-222	0.979859	0.33	0.078	1.9E-218	Skin_Peric	MEF2C
SLC2A41	3.6E-222	0.402109	0.087	0.006	5.7E-218	Skin_Peric	SLC2A4
ARHGAP29	3.8E-222	1.118737	0.393	0.111	6.1E-218	Skin_Peric	ARHGAP29
SMTN2	9.4E-222	1.104791	0.225	0.04	1.5E-217	Skin_Peric	SMTN
CREM2	1.5E-219	1.889064	0.523	0.195	2.4E-215	Skin_Peric	CREM
EEF1A111	3.9E-218	0.78763	0.996	0.914	6.2E-214	Skin_Peric	EEF1A1
THAP21	5.2E-218	1.879769	0.255	0.052	8.4E-214	Skin_Peric	THAP2
CRIM14	5.3E-218	1.114784	0.315	0.076	8.5E-214	Skin_Peric	CRIM1
MGST311	1.3E-217	1.118165	0.72	0.33	2.1E-213	Skin_Peric	MGST3
CSRP14	6.2E-214	1.64126	0.446	0.152	9.9E-210	Skin_Peric	CSRP1
IFITM24	2.1E-207	0.911004	0.725	0.3	3.4E-203	Skin_Peric	IFITM2
LPP3	2.1E-205	1.390778	0.53	0.208	3.3E-201	Skin_Peric	LPP
PTGIR2	4.2E-205	0.599796	0.148	0.019	6.7E-201	Skin_Peric	PTGIR
PLEKHO13	3.2E-204	1.301141	0.323	0.082	5.2E-200	Skin_Peric	PLEKHO1
STOM3	5.7E-202	1.070981	0.454	0.149	9E-198	Skin_Peric	STOM
ZFP362	7.5E-201	1.493747	0.875	0.538	1.2E-196	Skin_Peric	ZFP36
OAZ22	1.6E-200	1.282885	0.473	0.174	2.6E-196	Skin_Peric	OAZ2
CTH1	4.1E-200	0.770433	0.13	0.016	6.6E-196	Skin_Peric	CTH

MAT2A4	5.6E-200	1.695702	0.426	0.147	8.9E-196	Skin_Peric	MAT2A
CBX65	7.8E-199	1.02959	0.318	0.081	1.2E-194	Skin_Peric	CBX6
ITIH55	1.4E-198	0.741963	0.209	0.037	2.3E-194	Skin_Peric	ITIH5
FILIP1L5	1.6E-197	0.982647	0.324	0.082	2.5E-193	Skin_Peric	FILIP1L
RPL310	2.3E-197	0.733251	0.998	0.841	3.7E-193	Skin_Peric	RPL3
CIRBP4	2E-196	0.997339	0.859	0.542	3.1E-192	Skin_Peric	CIRBP
TBC1D17	2.9E-196	0.853786	0.256	0.056	4.6E-192	Skin_Peric	TBC1D1
CSPG41	7.5E-196	0.322888	0.071	0.005	1.2E-191	Skin_Peric	CSPG4
GLA2	1E-194	1.626942	0.319	0.086	1.6E-190	Skin_Peric	GLA
GPX31	6.4E-194	1.136218	0.4	0.123	1E-189	Skin_Peric	GPX3
PPP1R12A!	3.1E-192	1.141041	0.474	0.174	5E-188	Skin_Peric	PPP1R12A
EPS84	1.1E-191	1.033328	0.301	0.077	1.7E-187	Skin_Peric	EPS8
PTMS7	9.6E-191	1.031427	0.582	0.24	1.5E-186	Skin_Peric	PTMS
ADCY32	1.7E-190	0.611729	0.148	0.02	2.7E-186	Skin_Peric	ADCY3
PDLIM36	3.5E-189	0.940282	0.253	0.055	5.5E-185	Skin_Peric	PDLIM3
C7orf55.LL	2.5E-188	0.724328	0.225	0.044	4E-184	Skin_Peric	C7orf55.LUC7L2
ZNF5032	2.9E-188	0.952667	0.278	0.067	4.6E-184	Skin_Peric	ZNF503
ATP1B33	3.7E-188	1.934295	0.531	0.233	6E-184	Skin_Peric	ATP1B3
ZFP36L13	4.5E-188	0.960676	0.836	0.465	7.2E-184	Skin_Peric	ZFP36L1
B3GNT21	1.1E-186	0.962149	0.277	0.067	1.8E-182	Skin_Peric	B3GNT2
CACNA1H1	1.6E-186	0.376479	0.078	0.006	2.5E-182	Skin_Peric	CACNA1H
SDC22	8.9E-186	0.961809	0.368	0.106	1.4E-181	Skin_Peric	SDC2
ATP1A21	3.9E-185	0.275265	0.067	0.004	6.2E-181	Skin_Peric	ATP1A2
TUBA1B6	3.2E-182	1.125757	0.701	0.35	5E-178	Skin_Peric	TUBA1B
CPM1	5.9E-182	0.919009	0.248	0.056	9.3E-178	Skin_Peric	CPM
CAV24	2E-181	0.992028	0.379	0.116	3.2E-177	Skin_Peric	CAV2
PDLIM74	2.2E-180	0.955886	0.261	0.062	3.5E-176	Skin_Peric	PDLIM7
HSPA1B2	1.2E-177	1.38978	0.644	0.307	1.9E-173	Skin_Peric	HSPA1B
CXCL21	3.5E-176	1.498471	0.457	0.16	5.5E-172	Skin_Peric	CXCL2
S100A64	1.3E-175	0.616027	0.989	0.71	2.1E-171	Skin_Peric	S100A6
H2AFZ2	2.8E-175	1.235272	0.748	0.435	4.5E-171	Skin_Peric	H2AFZ
RPL23A1	6.8E-175	0.743372	0.989	0.801	1.1E-170	Skin_Peric	RPL23A
CNN31	7.3E-175	1.095988	0.527	0.221	1.2E-170	Skin_Peric	CNN3
TSC22D13	2.7E-171	1.219855	0.58	0.264	4.3E-167	Skin_Peric	TSC22D1
ESAM2	3.5E-170	0.789688	0.276	0.069	5.5E-166	Skin_Peric	ESAM
RPL10A2	1.2E-168	0.681958	0.99	0.732	2E-164	Skin_Peric	RPL10A
HAPLN31	5.9E-167	0.7167	0.182	0.033	9.3E-163	Skin_Peric	HAPLN3
CLIC43	1.4E-165	1.087883	0.37	0.122	2.3E-161	Skin_Peric	CLIC4
HSP90AB1(	3.2E-164	0.913442	0.873	0.566	5.1E-160	Skin_Peric	HSP90AB1
YBX12	8E-164	0.676968	0.891	0.585	1.3E-159	Skin_Peric	YBX1
NPM11	1.1E-163	0.820889	0.871	0.529	1.7E-159	Skin_Peric	NPM1
RPL27A2	4.9E-161	0.686811	0.99	0.834	7.8E-157	Skin_Peric	RPL27A
RAB3A	2.5E-160	0.531716	0.102	0.012	4E-156	Skin_Peric	RAB3A
ATF32	2.9E-159	1.249419	0.547	0.241	4.6E-155	Skin_Peric	ATF3
TUBB4	5.9E-159	0.948683	0.644	0.307	9.4E-155	Skin_Peric	TUBB
CYP26B11	8.8E-159	0.916003	0.153	0.025	1.4E-154	Skin_Peric	CYP26B1
RPS273	1.4E-157	0.67906	0.999	0.893	2.2E-153	Skin_Peric	RPS27

FILIP14	2.5E-157	0.529431	0.138	0.021	4E-153	Skin_Peric	FILIP1
HNRNPA02	2.8E-157	0.925784	0.661	0.331	4.4E-153	Skin_Peric	HNRNPA0
RPL37A2	2.4E-156	0.667176	0.989	0.854	3.9E-152	Skin_Peric	RPL37A
C10orf102	1.4E-154	1.414646	0.26	0.067	2.2E-150	Skin_Peric	C10orf10
MT1E1	2.4E-154	0.374257	0.384	0.126	3.8E-150	Skin_Peric	MT1E
ZNF902	3E-154	0.834701	0.318	0.093	4.8E-150	Skin_Peric	ZNF90
WFDC12	3.1E-154	0.694933	0.163	0.03	4.9E-150	Skin_Peric	WFDC1
HCFC1R13	9.9E-154	0.955288	0.384	0.133	1.6E-149	Skin_Peric	HCFC1R1
RPL35A1	5.3E-153	0.651761	0.99	0.862	8.4E-149	Skin_Peric	RPL35A
MFHAS11	1E-152	0.699336	0.19	0.039	1.6E-148	Skin_Peric	MFHAS1
HNRNPH3	2.4E-152	0.987114	0.503	0.218	3.8E-148	Skin_Peric	HNRNPH3
RPL232	2.8E-152	0.764129	0.944	0.68	4.5E-148	Skin_Peric	RPL23
HNRNPH13	5.9E-152	1.216019	0.59	0.295	9.4E-148	Skin_Peric	HNRNPH1
COL6A13	2.1E-150	0.730542	0.412	0.138	3.4E-146	Skin_Peric	COL6A1
RPS251	5.4E-149	0.62846	0.99	0.822	8.6E-145	Skin_Peric	RPS25
NFKBIA2	1E-148	0.929446	0.854	0.512	1.7E-144	Skin_Peric	NFKBIA
KLHL232	1.6E-148	0.530963	0.119	0.017	2.6E-144	Skin_Peric	KLHL23
TPPP33	2.9E-148	0.933698	0.344	0.106	4.7E-144	Skin_Peric	TPPP3
SGCA1	5.7E-148	0.630288	0.215	0.048	9.1E-144	Skin_Peric	SGCA
CHN11	7.3E-147	0.763005	0.133	0.021	1.2E-142	Skin_Peric	CHN1
PKIG3	1.2E-146	0.914815	0.311	0.097	2E-142	Skin_Peric	PKIG
GADD45G2	1.6E-146	1.349637	0.248	0.065	2.5E-142	Skin_Peric	GADD45G
ITGB14	3.9E-146	1.023297	0.647	0.354	6.1E-142	Skin_Peric	ITGB1
BTG27	4.7E-145	1.092717	0.526	0.229	7.5E-141	Skin_Peric	BTG2
BHLHE401	7.5E-145	1.012188	0.458	0.184	1.2E-140	Skin_Peric	BHLHE40
PCOLCE3	1.9E-144	0.741778	0.309	0.089	3E-140	Skin_Peric	PCOLCE
NRGN2	4.1E-144	0.433598	0.107	0.014	6.5E-140	Skin_Peric	NRGN
RPLP16	9.6E-144	0.566319	0.997	0.914	1.5E-139	Skin_Peric	RPLP1
RSL24D12	6.2E-143	0.989761	0.596	0.303	9.9E-139	Skin_Peric	RSL24D1
EGR15	7.5E-143	1.516668	0.426	0.17	1.2E-138	Skin_Peric	EGR1
SEMA4C1	1.9E-142	0.6506	0.142	0.024	3E-138	Skin_Peric	SEMA4C
SLC25A61	2.8E-142	0.731513	0.881	0.564	4.4E-138	Skin_Peric	SLC25A6
RPS82	3.2E-142	0.659131	0.99	0.848	5.1E-138	Skin_Peric	RPS8
IFITM11	4.9E-142	0.962202	0.433	0.156	7.8E-138	Skin_Peric	IFITM1
DNAJB93	2.4E-141	1.703035	0.452	0.205	3.8E-137	Skin_Peric	DNAJB9
PPP1R12B3	4.7E-141	0.804914	0.205	0.048	7.6E-137	Skin_Peric	PPP1R12B
GAPDH5	8.2E-141	0.682997	0.949	0.762	1.3E-136	Skin_Peric	GAPDH
C1orf544	1E-140	0.626369	0.202	0.046	1.6E-136	Skin_Peric	C1orf54
ADAMTS51	8.6E-139	0.615906	0.123	0.019	1.4E-134	Skin_Peric	ADAMTS5
CCNH2	5.8E-138	1.158134	0.328	0.112	9.3E-134	Skin_Peric	CCNH
IFRD1	2.7E-136	1.141895	0.477	0.217	4.3E-132	Skin_Peric	IFRD1
UBB1	3.9E-136	0.665043	0.943	0.727	6.2E-132	Skin_Peric	UBB
IGFBP43	7.1E-136	0.715496	0.38	0.127	1.1E-131	Skin_Peric	IGFBP4
HES7	4.3E-135	0.261719	0.052	0.004	6.9E-131	Skin_Peric	HES7
FAU5	7.3E-135	0.577424	0.991	0.854	1.2E-130	Skin_Peric	FAU
ANGPT12	1.1E-134	0.403397	0.094	0.012	1.8E-130	Skin_Peric	ANGPT1
NET13	2.7E-134	1.143624	0.321	0.11	4.3E-130	Skin_Peric	NET1

EIF3E4	1.9E-133	0.821038	0.758	0.445	3E-129	Skin_Perici	EIF3E
GPM6B1	3.6E-132	0.946814	0.183	0.041	5.8E-128	Skin_Perici	GPM6B
STAT35	2E-131	0.937567	0.442	0.188	3.2E-127	Skin_Perici	STAT3
EIF1B2	5.6E-131	1.078915	0.51	0.247	8.9E-127	Skin_Perici	EIF1B
ATF43	1.6E-130	0.956675	0.606	0.334	2.6E-126	Skin_Perici	ATF4
IER3	1.7E-130	1.427525	0.669	0.385	2.7E-126	Skin_Perici	IER3
MRPL32	2.1E-130	0.955748	0.358	0.135	3.4E-126	Skin_Perici	MRPL32
PRKG13	3.8E-130	0.448903	0.125	0.02	6.1E-126	Skin_Perici	PRKG1
ARC	5.5E-130	0.433	0.08	0.009	8.8E-126	Skin_Perici	ARC
SLC25A53	6.4E-130	0.885798	0.661	0.366	1E-125	Skin_Perici	SLC25A5
HNRNPDL2	8.2E-130	0.852231	0.646	0.333	1.3E-125	Skin_Perici	HNRNPDL
U2AF12	8.9E-130	0.857114	0.537	0.252	1.4E-125	Skin_Perici	U2AF1
SLC38A23	2.2E-129	0.915803	0.518	0.238	3.5E-125	Skin_Perici	SLC38A2
EHD24	5.9E-129	0.655301	0.19	0.044	9.4E-125	Skin_Perici	EHD2
FOSL22	2E-128	1.11418	0.475	0.217	3.1E-124	Skin_Perici	FOSL2
RBM32	4.3E-128	0.867883	0.716	0.437	6.8E-124	Skin_Perici	RBM3
HEYL1	1.2E-127	0.302056	0.065	0.006	1.9E-123	Skin_Perici	HEYL
PFDN52	5.8E-127	0.632483	0.95	0.724	9.3E-123	Skin_Perici	PFDN5
PGM52	1.2E-126	0.387245	0.087	0.011	1.9E-122	Skin_Perici	PGM5
SEPHS2	8.8E-126	0.894449	0.229	0.062	1.4E-121	Skin_Perici	SEPHS2
ENAH4	2.2E-125	0.957184	0.324	0.116	3.6E-121	Skin_Perici	ENAH
CCDC1076	1.2E-124	0.963228	0.415	0.173	2E-120	Skin_Perici	CCDC107
JUN5	2.8E-123	1.064889	0.773	0.484	4.5E-119	Skin_Perici	JUN
NUPR17	2.9E-123	0.723607	0.558	0.256	4.7E-119	Skin_Perici	NUPR1
CCND15	3.2E-123	1.012769	0.325	0.117	5.2E-119	Skin_Perici	CCND1
EEF22	9.8E-123	0.682296	0.876	0.605	1.6E-118	Skin_Perici	EEF2
FHL13	1.7E-121	0.829253	0.356	0.127	2.8E-117	Skin_Perici	FHL1
HNRNPA2E	8.3E-121	0.88131	0.757	0.489	1.3E-116	Skin_Perici	HNRNPA2B1
CRTAP4	1.1E-120	0.836945	0.319	0.113	1.7E-116	Skin_Perici	CRTAP
PCNP2	1.6E-120	0.958217	0.51	0.257	2.6E-116	Skin_Perici	PCNP
RPS261	2E-120	0.591632	0.955	0.74	3.2E-116	Skin_Perici	RPS26
HMGB17	3.1E-120	0.724386	0.832	0.572	4.9E-116	Skin_Perici	HMGB1
RPL312	1.3E-119	0.474298	0.984	0.766	2E-115	Skin_Perici	RPL31
SERPINF11	2.3E-119	0.466517	0.387	0.136	3.6E-115	Skin_Perici	SERPINF1
SCN4B1	5.4E-119	0.26892	0.048	0.004	8.6E-115	Skin_Perici	SCN4B
BMP2	8.7E-119	0.619581	0.16	0.034	1.4E-114	Skin_Perici	BMP2
LAPTM4A2	1.2E-118	0.909088	0.773	0.516	1.9E-114	Skin_Perici	LAPTM4A
RPS27A5	1.5E-118	0.562503	0.994	0.848	2.4E-114	Skin_Perici	RPS27A
PMP224	1.4E-117	0.67497	0.374	0.136	2.2E-113	Skin_Perici	PMP22
C12orf576	1.7E-116	0.826939	0.629	0.354	2.7E-112	Skin_Perici	C12orf57
DLC13	2.8E-115	0.651842	0.192	0.048	4.5E-111	Skin_Perici	DLC1
EID14	6.2E-115	0.789527	0.639	0.354	9.8E-111	Skin_Perici	EID1
CDC42EP4	1.2E-114	0.72572	0.204	0.054	1.9E-110	Skin_Perici	CDC42EP4
EIF4A22	6.8E-114	0.760352	0.747	0.483	1.1E-109	Skin_Perici	EIF4A2
BTF33	1.5E-112	0.600882	0.921	0.709	2.3E-108	Skin_Perici	BTF3
CA41	2.2E-112	0.486596	0.06	0.006	3.5E-108	Skin_Perici	CA4
CHSY11	3.1E-112	0.758723	0.199	0.053	4.9E-108	Skin_Perici	CHSY1

RAB201	6.6E-112	0.509374	0.122	0.022	1.1E-107	Skin_Peric	RAB20
RPS182	1.4E-111	0.510491	1	0.915	2.2E-107	Skin_Peric	RPS18
RPS143	1.7E-111	0.528007	0.997	0.881	2.7E-107	Skin_Peric	RPS14
KIAA00401	2.1E-111	0.741723	0.205	0.056	3.3E-107	Skin_Peric	KIAA0040
ERF	3.4E-111	0.807503	0.237	0.071	5.4E-107	Skin_Peric	ERF
RASD13	1.4E-110	1.365911	0.243	0.076	2.2E-106	Skin_Peric	RASD1
SBDS1	3.8E-110	0.871908	0.587	0.339	6.1E-106	Skin_Peric	SBDS
CTNNB13	5.3E-110	1.12165	0.388	0.169	8.4E-106	Skin_Peric	CTNNB1
UQCRB4	1E-109	0.805517	0.787	0.555	1.6E-105	Skin_Peric	UQCRB
RPL152	3.2E-109	0.492735	0.994	0.894	5.1E-105	Skin_Peric	RPL15
RPL2211	3.7E-109	0.612817	0.935	0.691	5.8E-105	Skin_Peric	RPL22
ARHGAP63	8.1E-109	0.382751	0.093	0.014	1.3E-104	Skin_Peric	ARHGAP6
ELL23	3.5E-108	1.099348	0.315	0.123	5.5E-104	Skin_Peric	ELL2
RPL13A1	3.9E-108	0.421008	0.999	0.88	6.1E-104	Skin_Peric	RPL13A
RPS61	1.1E-107	0.335042	0.993	0.823	1.8E-103	Skin_Peric	RPS6
RNF1523	2.1E-106	0.685853	0.152	0.035	3.4E-102	Skin_Peric	RNF152
SORBS12	2.4E-106	0.666494	0.15	0.033	3.8E-102	Skin_Peric	SORBS1
NEXN2	2.4E-106	0.474271	0.116	0.021	3.9E-102	Skin_Peric	NEXN
HSPD15	4.2E-106	1.088486	0.485	0.255	6.7E-102	Skin_Peric	HSPD1
COL5A32	2E-105	0.378611	0.074	0.009	3.3E-101	Skin_Peric	COL5A3
LIF	2.3E-105	0.456109	0.079	0.011	3.6E-101	Skin_Peric	LIF
HNRNPA1L	4.3E-105	0.576783	0.184	0.048	6.8E-101	Skin_Peric	HNRNPA1L2
RPS284	1E-104	0.520462	0.99	0.88	1.6E-100	Skin_Peric	RPS28
BIRC33	1.4E-104	1.613902	0.363	0.152	2.3E-100	Skin_Peric	BIRC3
TWSG1	1.7E-104	0.711923	0.224	0.067	2.7E-100	Skin_Peric	TWSG1
CRIP23	2.6E-103	0.800514	0.437	0.202	4.2E-99	Skin_Peric	CRIP2
COX6C5	4.9E-103	0.729763	0.781	0.539	7.8E-99	Skin_Peric	COX6C
RPL382	7.8E-103	0.558536	0.95	0.76	1.24E-98	Skin_Peric	RPL38
PROCR2	1.1E-102	0.61621	0.138	0.03	1.77E-98	Skin_Peric	PROCR
SERPINH12	2.7E-102	0.819061	0.305	0.113	4.29E-98	Skin_Peric	SERPINH1
RPL272	2.9E-102	0.520211	0.954	0.748	4.7E-98	Skin_Peric	RPL27
MFAP41	9.4E-102	0.377689	0.297	0.097	1.5E-97	Skin_Peric	MFAP4
YBX33	2.7E-100	1.059919	0.712	0.493	4.23E-96	Skin_Peric	YBX3
TOB22	3.8E-100	0.844879	0.274	0.099	6.14E-96	Skin_Peric	TOB2
TMEM38B	1.4E-99	0.706034	0.225	0.069	2.24E-95	Skin_Peric	TMEM38B
SOD14	1.8E-99	0.703523	0.689	0.412	2.83E-95	Skin_Peric	SOD1
COL27A11	9E-99	0.378394	0.089	0.014	1.43E-94	Skin_Peric	COL27A1
KDM6B2	2.67E-98	0.896128	0.304	0.116	4.26E-94	Skin_Peric	KDM6B
SELK3	4.06E-98	1.117182	0.684	0.467	6.48E-94	Skin_Peric	SELK
GUCY1B32	3.35E-96	0.473379	0.108	0.02	5.34E-92	Skin_Peric	GUCY1B3
HMGN22	8.23E-96	0.827702	0.553	0.326	1.31E-91	Skin_Peric	HMGN2
MLLT11	1.21E-95	0.753757	0.157	0.039	1.93E-91	Skin_Peric	MLLT11
PHLDA22	1.24E-95	0.723753	0.433	0.205	1.98E-91	Skin_Peric	PHLDA2
CPED13	2.15E-95	0.39555	0.107	0.02	3.43E-91	Skin_Peric	CPED1
SIK1	4.39E-95	0.843138	0.354	0.147	7E-91	Skin_Peric	SIK1
RPS202	5.18E-94	0.46206	0.97	0.793	8.27E-90	Skin_Peric	RPS20
SMARCD32	3.63E-93	0.455415	0.124	0.026	5.79E-89	Skin_Peric	SMARCD3

RPL352	4.26E-93	0.458307	0.987	0.834	6.79E-89	Skin_Peric	RPL35
ILK2	4.28E-93	0.885401	0.336	0.145	6.83E-89	Skin_Peric	ILK
RP11.294J2	4.57E-93	0.478345	0.128	0.028	7.29E-89	Skin_Peric	RP11.294J22.6
THBD2	8.69E-93	0.613004	0.162	0.041	1.39E-88	Skin_Peric	THBD
YWHAH3	3.46E-92	0.906475	0.42	0.205	5.52E-88	Skin_Peric	YWHAH
AAED14	3.64E-92	0.713917	0.228	0.074	5.8E-88	Skin_Peric	AAED1
RPS193	3.07E-91	0.419347	0.997	0.874	4.9E-87	Skin_Peric	RPS19
RPS51	4.3E-91	0.423267	0.99	0.783	6.86E-87	Skin_Peric	RPS5
PTK22	8.64E-91	0.916069	0.28	0.111	1.38E-86	Skin_Peric	PTK2
CXCL123	1.89E-90	0.469898	0.302	0.108	3.02E-86	Skin_Peric	CXCL12
NUCKS15	7.42E-90	0.79559	0.51	0.281	1.18E-85	Skin_Peric	NUCKS1
NMD3	1.01E-89	0.741344	0.225	0.075	1.62E-85	Skin_Peric	NMD3
H3F3A14	3.49E-89	0.512507	0.843	0.474	5.56E-85	Skin_Peric	H3F3A
RPL262	1.85E-88	0.448681	0.985	0.833	2.95E-84	Skin_Peric	RPL26
REL3	3.7E-88	0.870357	0.394	0.184	5.9E-84	Skin_Peric	REL
FERMT24	7.09E-88	0.702474	0.234	0.08	1.13E-83	Skin_Peric	FERMT2
NDN3	8.73E-88	0.608272	0.201	0.061	1.39E-83	Skin_Peric	NDN
EDNRB3	1.51E-87	0.529734	0.115	0.024	2.41E-83	Skin_Peric	EDNRB
BTG13	1.55E-87	0.583316	0.823	0.574	2.47E-83	Skin_Peric	BTG1
C1QTNF23	3.03E-87	0.396859	0.111	0.022	4.84E-83	Skin_Peric	C1QTNF2
RPL103	4.57E-87	0.455509	0.998	0.876	7.29E-83	Skin_Peric	RPL10
KCNMB13	1.31E-86	0.35361	0.088	0.015	2.09E-82	Skin_Peric	KCNMB1
RPS134	1.47E-86	0.44933	0.98	0.816	2.34E-82	Skin_Peric	RPS13
TCEAL73	2.08E-86	0.598759	0.081	0.013	3.31E-82	Skin_Peric	TCEAL7
WSB16	2.31E-86	0.784859	0.465	0.248	3.69E-82	Skin_Peric	WSB1
RPL322	3.03E-86	0.414908	0.995	0.871	4.83E-82	Skin_Peric	RPL32
DDR22	3.89E-86	0.485224	0.162	0.042	6.21E-82	Skin_Peric	DDR2
ESD6	4.51E-86	0.812947	0.423	0.215	7.2E-82	Skin_Peric	ESD
MID1IP11	9.56E-86	0.825801	0.221	0.074	1.52E-81	Skin_Peric	MID1IP1
HNRNPU1	1.38E-85	0.959397	0.548	0.347	2.19E-81	Skin_Peric	HNRNPU
RPL4	2.76E-85	0.261088	0.941	0.697	4.4E-81	Skin_Peric	RPL4
FAM150B1	3.17E-85	0.625525	0.091	0.016	5.06E-81	Skin_Peric	FAM150B
ELMSAN12	1.26E-84	0.682878	0.226	0.077	2.02E-80	Skin_Peric	ELMSAN1
IRF2BPL3	1.46E-83	0.75621	0.22	0.075	2.33E-79	Skin_Peric	IRF2BPL
EPOR	1.86E-83	0.408024	0.077	0.012	2.97E-79	Skin_Peric	EPOR
ARMCX11	4.83E-83	0.622297	0.183	0.055	7.7E-79	Skin_Peric	ARMCX1
DNAJB13	1.36E-82	0.827971	0.658	0.427	2.16E-78	Skin_Peric	DNAJB1
CLMN3	4.02E-82	0.637598	0.199	0.065	6.41E-78	Skin_Peric	CLMN
ACTN13	6.56E-82	0.849371	0.325	0.146	1.05E-77	Skin_Peric	ACTN1
TWIST12	5.54E-81	0.455852	0.167	0.046	8.84E-77	Skin_Peric	TWIST1
LRRC322	1.47E-80	0.446597	0.134	0.033	2.34E-76	Skin_Peric	LRRC32
IRF11	4E-80	1.008358	0.416	0.213	6.38E-76	Skin_Peric	IRF1
NDUFS55	4.04E-80	0.665194	0.67	0.453	6.44E-76	Skin_Peric	NDUFS5
RPL55	4.54E-80	0.355239	0.971	0.725	7.23E-76	Skin_Peric	RPL5
HOXB.AS1	5.6E-80	0.413787	0.101	0.02	8.94E-76	Skin_Peric	HOXB.AS1
TNFRSF12A	5.9E-80	0.672187	0.305	0.125	9.4E-76	Skin_Peric	TNFRSF12A
AKAP121	2.01E-79	0.718327	0.216	0.073	3.21E-75	Skin_Peric	AKAP12

VAPA2	4.78E-79	0.780261	0.603	0.398	7.63E-75	Skin_Peric	VAPA
RPL249	1.15E-78	0.44467	0.951	0.8	1.83E-74	Skin_Peric	RPL24
MATR32	1.29E-78	0.640851	0.285	0.114	2.06E-74	Skin_Peric	MATR3
P2RY142	1.98E-78	0.357016	0.062	0.009	3.15E-74	Skin_Peric	P2RY14
MAFF	2.87E-78	0.909809	0.437	0.236	4.58E-74	Skin_Peric	MAFF
BANP	3.09E-78	0.78557	0.16	0.046	4.93E-74	Skin_Peric	BANP
EMX21	6.36E-78	0.492737	0.125	0.03	1.01E-73	Skin_Peric	EMX2
NFIB9	1.14E-77	0.699857	0.391	0.189	1.81E-73	Skin_Peric	NFIB
ANKRD293	1.38E-77	0.497969	0.12	0.028	2.2E-73	Skin_Peric	ANKRD29
APCDD1	1.57E-77	0.636299	0.145	0.038	2.5E-73	Skin_Peric	APCDD1
KCTD202	1.58E-77	0.705409	0.215	0.076	2.52E-73	Skin_Peric	KCTD20
ELOVL52	2.75E-77	0.690264	0.266	0.106	4.38E-73	Skin_Peric	ELOVL5
ZFAS12	2.99E-77	0.536683	0.709	0.463	4.76E-73	Skin_Peric	ZFAS1
N4BP2L11	3.35E-77	0.605131	0.194	0.062	5.34E-73	Skin_Peric	N4BP2L1
HNRNPA34	1.01E-76	0.712784	0.568	0.363	1.61E-72	Skin_Peric	HNRNPA3
DES1	3.74E-76	1.722051	0.086	0.016	5.96E-72	Skin_Peric	DES
AC010642.	5.76E-75	0.408882	0.138	0.035	9.19E-71	Skin_Peric	AC010642.1
BCL6	7.91E-75	0.659585	0.21	0.073	1.26E-70	Skin_Peric	BCL6
SLC25A48	8.59E-75	0.79717	0.267	0.113	1.37E-70	Skin_Peric	SLC25A4
RAB344	1.45E-74	0.624563	0.256	0.1	2.31E-70	Skin_Peric	RAB34
MORF4L12	3.02E-74	0.621096	0.672	0.456	4.82E-70	Skin_Peric	MORF4L1
TSPYL22	3.53E-74	0.692645	0.234	0.087	5.64E-70	Skin_Peric	TSPYL2
OLFML2B2	4.59E-74	0.422558	0.117	0.028	7.32E-70	Skin_Peric	OLFML2B
CYGB2	6.87E-74	0.574595	0.108	0.024	1.1E-69	Skin_Peric	CYGB
TGIF12	2.59E-73	0.977681	0.375	0.195	4.14E-69	Skin_Peric	TGIF1
MYH94	5.7E-73	0.749267	0.368	0.184	9.08E-69	Skin_Peric	MYH9
C20orf272	6.51E-73	0.653614	0.2	0.068	1.04E-68	Skin_Peric	C20orf27
GUCY1A21	7.18E-73	0.366758	0.066	0.01	1.15E-68	Skin_Peric	GUCY1A2
YWHAQ1	1.82E-72	0.749706	0.56	0.363	2.9E-68	Skin_Peric	YWHAQ
NAP1L12	1.94E-72	0.58729	0.634	0.399	3.09E-68	Skin_Peric	NAP1L1
DNAJA13	1.42E-71	0.787835	0.557	0.359	2.27E-67	Skin_Peric	DNAJA1
SATB2	2.24E-71	0.262968	0.059	0.009	3.58E-67	Skin_Peric	SATB2
SLIT33	2.98E-71	0.586319	0.22	0.08	4.75E-67	Skin_Peric	SLIT3
RPS91	1.6E-70	0.354091	0.988	0.835	2.55E-66	Skin_Peric	RPS9
ERH2	1.63E-70	0.656384	0.513	0.309	2.6E-66	Skin_Peric	ERH
MYO1B1	2E-70	0.696003	0.2	0.071	3.18E-66	Skin_Peric	MYO1B
COL23A1	2.2E-70	0.265482	0.049	0.006	3.51E-66	Skin_Peric	COL23A1
RPL94	2.22E-70	0.323322	0.978	0.747	3.54E-66	Skin_Peric	RPL9
CYB5R33	3.87E-70	0.767752	0.407	0.22	6.17E-66	Skin_Peric	CYB5R3
ANKRD101	1.23E-69	0.728752	0.266	0.111	1.96E-65	Skin_Peric	ANKRD10
CD1519	1.55E-69	0.744515	0.406	0.218	2.47E-65	Skin_Peric	CD151
DNAJB4	1.58E-69	0.739689	0.254	0.104	2.51E-65	Skin_Peric	DNAJB4
DKK34	2.49E-69	0.504113	0.147	0.043	3.97E-65	Skin_Peric	DKK3
SKP14	1.2E-68	0.550796	0.775	0.569	1.92E-64	Skin_Peric	SKP1
CTGF3	1.46E-68	0.824159	0.271	0.111	2.34E-64	Skin_Peric	CTGF
CNBP1	4.24E-68	0.570647	0.638	0.425	6.76E-64	Skin_Peric	CNBP
CAMTA1	7.31E-68	0.770885	0.51	0.332	1.17E-63	Skin_Peric	CAMTA1

SPRY21	7.35E-68	0.376055	0.112	0.027	1.17E-63	Skin_Perici	SPRY2
SET5	8.26E-68	0.717112	0.503	0.309	1.32E-63	Skin_Perici	SET
H3F3B	1.32E-67	0.319941	0.982	0.861	2.11E-63	Skin_Perici	H3F3B
RAN4	4.2E-67	0.642236	0.574	0.365	6.7E-63	Skin_Perici	RAN
TUBA1A5	6.27E-67	0.565288	0.446	0.233	1E-62	Skin_Perici	TUBA1A
TLN15	8E-67	0.654418	0.289	0.129	1.28E-62	Skin_Perici	TLN1
BEST12	2E-66	0.450589	0.144	0.041	3.18E-62	Skin_Perici	BEST1
RPS3A2	2.01E-66	0.30274	0.986	0.829	3.21E-62	Skin_Perici	RPS3A
SDPR2	4.42E-66	0.43853	0.107	0.026	7.04E-62	Skin_Perici	SDPR
WTIP2	4.69E-66	0.321147	0.093	0.02	7.49E-62	Skin_Perici	WTIP
RGS24	5.04E-66	0.701252	0.243	0.095	8.04E-62	Skin_Perici	RGS2
TIPIN2	6.77E-66	0.618774	0.199	0.073	1.08E-61	Skin_Perici	TIPIN
NT5DC22	7.34E-66	0.580824	0.148	0.045	1.17E-61	Skin_Perici	NT5DC2
DDIT4L1	8.77E-66	0.262461	0.054	0.008	1.4E-61	Skin_Perici	DDIT4L
SFPQ5	1.42E-65	0.737399	0.467	0.284	2.26E-61	Skin_Perici	SFPQ
DDX55	2.11E-65	0.442933	0.799	0.6	3.37E-61	Skin_Perici	DDX5
RHOC4	2.37E-65	0.604832	0.416	0.222	3.79E-61	Skin_Perici	RHOC
COL16A11	2.5E-65	0.415426	0.122	0.032	3.98E-61	Skin_Perici	COL16A1
SVIL5	5.4E-65	0.800911	0.266	0.118	8.61E-61	Skin_Perici	SVIL
SYNPO1	1.14E-64	0.316364	0.078	0.015	1.82E-60	Skin_Perici	SYNPO
SLC7A51	1.9E-64	0.720773	0.163	0.054	3.02E-60	Skin_Perici	SLC7A5
TOMM73	1.11E-63	0.444702	0.809	0.579	1.77E-59	Skin_Perici	TOMM7
PTGES33	1.13E-63	0.628515	0.584	0.391	1.8E-59	Skin_Perici	PTGES3
RPS248	1.82E-63	0.360194	0.977	0.838	2.9E-59	Skin_Perici	RPS24
PRNP5	2.03E-63	0.676572	0.329	0.158	3.24E-59	Skin_Perici	PRNP
PPP1CB2	3.46E-63	0.574321	0.66	0.453	5.52E-59	Skin_Perici	PPP1CB
RPL18A2	3.61E-63	0.296743	0.997	0.821	5.77E-59	Skin_Perici	RPL18A
KB.1208A1	4.22E-63	0.265193	0.06	0.01	6.73E-59	Skin_Perici	KB.1208A12.3
MOB42	7.21E-63	0.592525	0.227	0.091	1.15E-58	Skin_Perici	MOB4
TGFB31	8.16E-63	0.316663	0.082	0.017	1.3E-58	Skin_Perici	TGFB3
FBXL131	1.63E-62	0.474403	0.112	0.029	2.6E-58	Skin_Perici	FBXL13
C1orf198	2.44E-62	0.52873	0.17	0.058	3.9E-58	Skin_Perici	C1orf198
MED22	2.5E-62	0.463857	0.106	0.027	3.98E-58	Skin_Perici	MED22
PNP1	3.02E-62	0.750828	0.196	0.074	4.82E-58	Skin_Perici	PNP
TFPI2	5.43E-62	0.819295	0.207	0.079	8.66E-58	Skin_Perici	TFPI
PNMA11	1.6E-61	0.49304	0.154	0.05	2.56E-57	Skin_Perici	PNMA1
ACTB3	1.69E-61	0.485923	0.988	0.871	2.7E-57	Skin_Perici	ACTB
HNRNPK1	2.12E-61	0.620658	0.625	0.444	3.38E-57	Skin_Perici	HNRNPK
SERPING14	2.46E-61	0.4227	0.359	0.171	3.93E-57	Skin_Perici	SERPING1
ETS22	4.84E-61	0.613712	0.323	0.156	7.72E-57	Skin_Perici	ETS2
COL6A31	8.75E-61	0.589978	0.232	0.091	1.4E-56	Skin_Perici	COL6A3
PIGA1	1.75E-60	0.562479	0.138	0.042	2.79E-56	Skin_Perici	PIGA
ANXA58	1.92E-60	0.540345	0.529	0.315	3.07E-56	Skin_Perici	ANXA5
SRSF25	8.42E-60	0.661495	0.515	0.33	1.34E-55	Skin_Perici	SRSF2
ATP2B45	2.14E-59	0.525695	0.205	0.079	3.42E-55	Skin_Perici	ATP2B4
APEX1	2.31E-59	0.881279	0.321	0.163	3.68E-55	Skin_Perici	APEX1
TNFAIP32	2.36E-59	1.017179	0.324	0.16	3.76E-55	Skin_Perici	TNFAIP3

WBP5	3.81E-59	0.740925	0.34	0.179	6.08E-55	Skin_Peric	WBP5
ESYT2	1.07E-58	0.821793	0.298	0.148	1.71E-54	Skin_Peric	ESYT2
KLHL15	1.08E-58	0.358221	0.098	0.024	1.72E-54	Skin_Peric	KLHL15
EIF5A5	1.38E-58	0.709239	0.448	0.277	2.2E-54	Skin_Peric	EIF5A
RPS152	1.45E-58	0.325327	0.997	0.88	2.32E-54	Skin_Peric	RPS15
OLFML2A3	1.47E-58	0.311983	0.095	0.023	2.34E-54	Skin_Peric	OLFML2A
AC009501.	2.48E-58	0.432198	0.116	0.032	3.95E-54	Skin_Peric	AC009501.4
RASGRP21	3.25E-58	0.319146	0.181	0.062	5.18E-54	Skin_Peric	RASGRP2
RPS293	4.23E-58	0.320104	0.961	0.788	6.74E-54	Skin_Peric	RPS29
GPRC5C2	1.14E-57	0.544369	0.148	0.049	1.82E-53	Skin_Peric	GPRC5C
SOD21	1.75E-57	0.487054	0.605	0.408	2.78E-53	Skin_Peric	SOD2
PIP4K2A4	2.09E-57	0.521318	0.169	0.059	3.33E-53	Skin_Peric	PIP4K2A
LAMC12	5.7E-57	0.552808	0.197	0.076	9.09E-53	Skin_Peric	LAMC1
LURAP1L1	1.03E-56	0.639338	0.155	0.054	1.64E-52	Skin_Peric	LURAP1L
PTPRG3	1.18E-56	0.40984	0.125	0.037	1.88E-52	Skin_Peric	PTPRG
HOMER1	1.28E-56	0.448806	0.107	0.029	2.05E-52	Skin_Peric	HOMER1
CXorf40B	1.29E-56	0.595307	0.134	0.042	2.06E-52	Skin_Peric	CXorf40B
DDX212	4.05E-56	0.723061	0.362	0.197	6.47E-52	Skin_Peric	DDX21
PFDN22	4.09E-56	0.698443	0.447	0.273	6.53E-52	Skin_Peric	PFDN2
GLIS21	4.87E-56	0.296065	0.082	0.018	7.76E-52	Skin_Peric	GLIS2
PARM14	1.91E-55	0.607295	0.151	0.052	3.05E-51	Skin_Peric	PARM1
LIX1L2	2.69E-55	0.431711	0.141	0.045	4.29E-51	Skin_Peric	LIX1L
AL592183.	3.72E-55	0.361369	0.091	0.022	5.93E-51	Skin_Peric	AL592183.1
CCNI6	1.43E-54	0.51185	0.69	0.516	2.27E-50	Skin_Peric	CCNI
AMD12	2.67E-54	0.705977	0.349	0.192	4.26E-50	Skin_Peric	AMD1
CD6312	2.9E-54	0.34417	0.888	0.622	4.63E-50	Skin_Peric	CD63
CCDC102B	3.73E-54	0.42606	0.096	0.025	5.94E-50	Skin_Peric	CCDC102B
ROCK14	5.07E-54	0.609499	0.307	0.154	8.08E-50	Skin_Peric	ROCK1
CDKN2C1	5.58E-54	0.57355	0.123	0.037	8.89E-50	Skin_Peric	CDKN2C
VCL3	6.6E-54	0.65375	0.245	0.112	1.05E-49	Skin_Peric	VCL
POMP3	7.11E-54	0.540471	0.578	0.401	1.13E-49	Skin_Peric	POMP
RPS162	1.44E-53	0.252755	0.981	0.834	2.3E-49	Skin_Peric	RPS16
ZBTB101	1.57E-53	0.625377	0.188	0.074	2.5E-49	Skin_Peric	ZBTB10
MORF4L21	2.03E-53	0.706959	0.458	0.3	3.24E-49	Skin_Peric	MORF4L2
PRKAR1A1	2.27E-53	0.726694	0.393	0.237	3.62E-49	Skin_Peric	PRKAR1A
SMIM104	2.96E-53	0.324728	0.084	0.02	4.72E-49	Skin_Peric	SMIM10
TPI12	3.33E-53	0.518362	0.711	0.533	5.31E-49	Skin_Peric	TPI1
NINJ12	5.39E-53	0.603916	0.292	0.144	8.6E-49	Skin_Peric	NINJ1
USP2	6.71E-53	0.407874	0.139	0.045	1.07E-48	Skin_Peric	USP2
RBMX2	7.19E-53	0.634286	0.357	0.197	1.15E-48	Skin_Peric	RBMX
CAMK13	9.01E-53	0.344485	0.116	0.034	1.44E-48	Skin_Peric	CAMK1
ZNRD12	1.43E-52	2.197852	0.272	0.139	2.27E-48	Skin_Peric	ZNRD1
NBL12	2.7E-52	0.421028	0.253	0.109	4.31E-48	Skin_Peric	NBL1
JMJD61	3.8E-52	0.56371	0.21	0.089	6.07E-48	Skin_Peric	JMJD6
TUBA1C3	4.2E-52	0.686221	0.279	0.141	6.7E-48	Skin_Peric	TUBA1C
TMEM55B	7.19E-52	0.637697	0.213	0.092	1.15E-47	Skin_Peric	TMEM55B
ST6GALNA	7.33E-52	0.486956	0.181	0.07	1.17E-47	Skin_Peric	ST6GALNAC6

CHCHD101	8.29E-52	0.522873	0.416	0.243	1.32E-47	Skin_Peric	CHCHD10
NDUFA49	1.02E-51	0.710043	0.696	0.536	1.63E-47	Skin_Peric	NDUFA4
NCKAP52	1.16E-51	0.316635	0.072	0.016	1.85E-47	Skin_Peric	NCKAP5
SELENBP19	2.17E-51	0.580959	0.184	0.074	3.46E-47	Skin_Peric	SELENBP1
KLF101	2.4E-51	0.617397	0.284	0.14	3.82E-47	Skin_Peric	KLF10
CLK12	5.1E-51	0.667113	0.41	0.25	8.14E-47	Skin_Peric	CLK1
OPA3	5.8E-51	0.458765	0.112	0.033	9.24E-47	Skin_Peric	OPA3
NFATC42	9.75E-51	0.303226	0.074	0.017	1.56E-46	Skin_Peric	NFATC4
SMYD41	1.48E-50	0.456243	0.112	0.034	2.36E-46	Skin_Peric	SMYD4
BATF32	1.48E-50	0.286672	0.066	0.014	2.37E-46	Skin_Peric	BATF3
ID22	1.52E-50	0.651056	0.428	0.253	2.43E-46	Skin_Peric	ID2
FGFR12	1.53E-50	0.409823	0.211	0.086	2.44E-46	Skin_Peric	FGFR1
COX7A22	1.95E-50	0.301933	0.75	0.569	3.11E-46	Skin_Peric	COX7A2
TCEB1	2.51E-50	0.849951	0.506	0.357	4.01E-46	Skin_Peric	TCEB1
CRIP13	3.94E-50	0.345359	0.48	0.275	6.28E-46	Skin_Peric	CRIP1
TCEAL11	4.59E-50	0.498658	0.15	0.053	7.32E-46	Skin_Peric	TCEAL1
RPL83	5.72E-50	0.292569	0.992	0.847	9.12E-46	Skin_Peric	RPL8
RCAN11	7.07E-50	0.581812	0.183	0.072	1.13E-45	Skin_Peric	RCAN1
TMEM473	9E-50	0.500634	0.151	0.054	1.43E-45	Skin_Peric	TMEM47
ANKRD40	1.28E-49	0.481561	0.14	0.049	2.04E-45	Skin_Peric	ANKRD40
SLIRP	2.64E-49	0.631768	0.41	0.257	4.21E-45	Skin_Peric	SLIRP
SKI2	3.66E-49	0.509085	0.159	0.059	5.84E-45	Skin_Peric	SKI
HNRNPM	1.16E-48	0.498991	0.38	0.215	1.86E-44	Skin_Peric	HNRNPM
ROCK26	1.41E-48	0.622823	0.211	0.094	2.25E-44	Skin_Peric	ROCK2
NEURL1B2	2.56E-48	0.344035	0.088	0.023	4.08E-44	Skin_Peric	NEURL1B
BST23	3.41E-48	0.383146	0.354	0.18	5.43E-44	Skin_Peric	BST2
ZBTB16	3.48E-48	0.336361	0.104	0.03	5.56E-44	Skin_Peric	ZBTB16
NNMT1	4.25E-48	0.424459	0.248	0.109	6.77E-44	Skin_Peric	NNMT
GGT53	4.4E-48	0.356396	0.091	0.024	7.01E-44	Skin_Peric	GGT5
CSNK1A14	5.09E-48	0.643919	0.426	0.27	8.12E-44	Skin_Peric	CSNK1A1
RAP1B2	1.18E-47	0.578717	0.43	0.268	1.88E-43	Skin_Peric	RAP1B
USP121	1.18E-47	0.533997	0.173	0.069	1.88E-43	Skin_Peric	USP12
GRK53	1.62E-47	0.288524	0.08	0.02	2.58E-43	Skin_Peric	GRK5
SSBP23	1.64E-47	0.528626	0.176	0.07	2.62E-43	Skin_Peric	SSBP2
CD97	3.31E-47	0.260655	0.689	0.45	5.28E-43	Skin_Peric	CD9
RASSF11	6.36E-47	0.559089	0.208	0.091	1.01E-42	Skin_Peric	RASSF1
HES1	1.35E-46	0.697893	0.374	0.219	2.15E-42	Skin_Peric	HES1
NCL6	1.48E-46	0.555656	0.436	0.269	2.36E-42	Skin_Peric	NCL
CYSTM14	1.51E-46	0.506019	0.387	0.224	2.4E-42	Skin_Peric	CYSTM1
PTEN3	1.99E-46	0.652717	0.253	0.127	3.17E-42	Skin_Peric	PTEN
PARK78	2.09E-46	0.44582	0.545	0.354	3.34E-42	Skin_Peric	PARK7
TACC16	2.68E-46	0.587879	0.309	0.165	4.27E-42	Skin_Peric	TACC1
IRF2BP23	3.28E-46	0.775893	0.399	0.257	5.24E-42	Skin_Peric	IRF2BP2
MGLL6	3.4E-46	0.602717	0.316	0.176	5.42E-42	Skin_Peric	MGLL
CKS22	3.43E-46	0.603487	0.279	0.141	5.48E-42	Skin_Peric	CKS2
STRAP	5.05E-46	0.597037	0.361	0.216	8.06E-42	Skin_Peric	STRAP
SGCE3	9.68E-46	0.398455	0.119	0.039	1.54E-41	Skin_Peric	SGCE

RNF52	1.17E-45	0.619115	0.273	0.142	1.86E-41	Skin_Peric	RNF5
TTLL73	1.33E-45	0.505754	0.162	0.064	2.13E-41	Skin_Peric	TTLL7
LAMB25	1.56E-45	0.53836	0.193	0.082	2.49E-41	Skin_Peric	LAMB2
ATP5G21	2.27E-45	0.311931	0.76	0.571	3.62E-41	Skin_Peric	ATP5G2
NAF1	2.47E-45	0.449547	0.112	0.036	3.94E-41	Skin_Peric	NAF1
ATP5B5	3.15E-45	0.544599	0.521	0.37	5.02E-41	Skin_Peric	ATP5B
RBM8A1	4.45E-45	0.619956	0.486	0.341	7.1E-41	Skin_Peric	RBM8A
LAMA54	5.62E-45	0.349111	0.096	0.028	8.97E-41	Skin_Peric	LAMA5
GNAI1	8.03E-45	0.573768	0.156	0.061	1.28E-40	Skin_Peric	GNAI1
GOLIM42	9.52E-45	0.551999	0.193	0.083	1.52E-40	Skin_Peric	GOLIM4
GNG112	1.09E-44	0.295116	0.247	0.113	1.73E-40	Skin_Peric	GNG11
EIF3L	1.39E-44	0.398818	0.562	0.386	2.22E-40	Skin_Peric	EIF3L
RP11.345J4	1.52E-44	0.343815	0.098	0.029	2.43E-40	Skin_Peric	RP11.345J4.5
FUS9	3.53E-44	0.54144	0.431	0.275	5.63E-40	Skin_Peric	FUS
MAGED27	3.73E-44	0.580605	0.288	0.154	5.96E-40	Skin_Peric	MAGED2
COMMD63	4.22E-44	0.349553	0.683	0.471	6.73E-40	Skin_Peric	COMMD6
AEBP2	4.92E-44	0.534678	0.157	0.062	7.85E-40	Skin_Peric	AEBP2
CCNL11	6.31E-44	0.56612	0.493	0.338	1.01E-39	Skin_Peric	CCNL1
LMO47	7.7E-44	0.627592	0.297	0.163	1.23E-39	Skin_Peric	LMO4
NOP16	8.72E-44	0.509133	0.171	0.071	1.39E-39	Skin_Peric	NOP16
NXT11	8.84E-44	0.596908	0.285	0.152	1.41E-39	Skin_Peric	NXT1
C3orf702	1.02E-43	0.306761	0.061	0.014	1.63E-39	Skin_Peric	C3orf70
GUK12	1.33E-43	0.673074	0.682	0.556	2.13E-39	Skin_Peric	GUK1
SERTAD12	3.45E-43	0.620194	0.377	0.229	5.5E-39	Skin_Peric	SERTAD1
SNX71	3.58E-43	0.456648	0.162	0.065	5.7E-39	Skin_Peric	SNX7
LIMS24	4.12E-43	0.297766	0.103	0.031	6.58E-39	Skin_Peric	LIMS2
GAS67	4.34E-43	0.557719	0.208	0.094	6.92E-39	Skin_Peric	GAS6
FRY3	1.43E-42	0.285173	0.077	0.02	2.28E-38	Skin_Peric	FRY
NOLC1	2.06E-42	0.505383	0.183	0.08	3.29E-38	Skin_Peric	NOLC1
TMEM119	2.13E-42	0.258752	0.059	0.013	3.4E-38	Skin_Peric	TMEM119
OAT5	2.39E-42	0.654184	0.297	0.17	3.82E-38	Skin_Peric	OAT
ID11	2.48E-42	0.350384	0.232	0.107	3.96E-38	Skin_Peric	ID1
TUBB2A3	2.85E-42	0.586243	0.23	0.112	4.54E-38	Skin_Peric	TUBB2A
THY12	3.5E-42	0.456994	0.139	0.05	5.58E-38	Skin_Peric	THY1
HEXIM1	4.02E-42	0.540745	0.304	0.167	6.41E-38	Skin_Peric	HEXIM1
DNAJC27	5.26E-42	0.397139	0.09	0.026	8.38E-38	Skin_Peric	DNAJC27
MOB21	6.19E-42	0.588935	0.259	0.136	9.88E-38	Skin_Peric	MOB2
RQCD11	6.92E-42	0.563602	0.159	0.065	1.1E-37	Skin_Peric	RQCD1
ARL5B2	7.31E-42	0.583795	0.208	0.097	1.17E-37	Skin_Peric	ARL5B
STARD133	9.06E-42	0.309483	0.074	0.019	1.44E-37	Skin_Peric	STARD13
COQ10B	9.3E-42	0.614813	0.269	0.145	1.48E-37	Skin_Peric	COQ10B
CAPZA22	9.67E-42	0.596985	0.418	0.278	1.54E-37	Skin_Peric	CAPZA2
POLR2L1	1.14E-41	0.417587	0.644	0.471	1.82E-37	Skin_Peric	POLR2L
EFNA11	1.46E-41	0.615024	0.194	0.089	2.32E-37	Skin_Peric	EFNA1
RPL36AL3	1.72E-41	0.344617	0.868	0.728	2.75E-37	Skin_Peric	RPL36AL
SLC22A3	2.67E-41	0.299849	0.077	0.021	4.26E-37	Skin_Peric	SLC22A3
ALKBH2	4.61E-41	0.396654	0.126	0.045	7.36E-37	Skin_Peric	ALKBH2

WTAP1	7.17E-41	0.422944	0.427	0.28	1.14E-36	Skin_Peric	WTAP
FAM122A	8.55E-41	0.458523	0.139	0.053	1.36E-36	Skin_Peric	FAM122A
SORBS32	1.03E-40	0.564355	0.215	0.104	1.64E-36	Skin_Peric	SORBS3
NEK1	1.06E-40	0.52878	0.126	0.046	1.7E-36	Skin_Peric	NEK1
NDEL11	1.3E-40	0.51177	0.181	0.08	2.07E-36	Skin_Peric	NDEL1
SON6	1.39E-40	0.480873	0.561	0.408	2.22E-36	Skin_Peric	SON
IMPDH2	1.6E-40	0.537514	0.287	0.156	2.56E-36	Skin_Peric	IMPDH2
SLC8A13	1.77E-40	0.293368	0.062	0.015	2.83E-36	Skin_Peric	SLC8A1
OLFM2	1.92E-40	0.445125	0.112	0.038	3.06E-36	Skin_Peric	OLFM2
DYNLT31	2.96E-40	0.51724	0.247	0.125	4.72E-36	Skin_Peric	DYNLT3
SPRY12	3.04E-40	0.257224	0.205	0.091	4.85E-36	Skin_Peric	SPRY1
PALLD4	3.7E-40	0.692151	0.282	0.159	5.91E-36	Skin_Peric	PALLD
TMEM14A	3.8E-40	0.498594	0.187	0.084	6.06E-36	Skin_Peric	TMEM14A
ARRDC31	4.11E-40	0.632442	0.384	0.243	6.55E-36	Skin_Peric	ARRDC3
ECM22	5.07E-40	0.311212	0.105	0.033	8.09E-36	Skin_Peric	ECM2
MSN6	5.25E-40	0.471865	0.291	0.155	8.37E-36	Skin_Peric	MSN
TCF43	7.15E-40	0.368628	0.317	0.172	1.14E-35	Skin_Peric	TCF4
FKBP101	9.96E-40	0.317492	0.106	0.034	1.59E-35	Skin_Peric	FKBP10
RHOQ6	1.06E-39	0.523008	0.188	0.085	1.69E-35	Skin_Peric	RHOQ
EIF3I	1.28E-39	0.669944	0.375	0.243	2.04E-35	Skin_Peric	EIF3I
C4orf322	1.32E-39	0.50397	0.181	0.08	2.1E-35	Skin_Peric	C4orf32
NFIA5	1.32E-39	0.561398	0.303	0.172	2.11E-35	Skin_Peric	NFIA
PTP4A11	2.37E-39	0.607784	0.421	0.283	3.77E-35	Skin_Peric	PTP4A1
PRKAR2B2	3.18E-39	0.589618	0.127	0.048	5.07E-35	Skin_Peric	PRKAR2B
TBCA4	3.42E-39	0.497195	0.495	0.35	5.45E-35	Skin_Peric	TBCA
USP371	6.52E-39	0.538208	0.136	0.053	1.04E-34	Skin_Peric	USP37
MOB3B1	6.82E-39	0.346067	0.087	0.026	1.09E-34	Skin_Peric	MOB3B
SYTL21	7.22E-39	0.407941	0.122	0.044	1.15E-34	Skin_Peric	SYTL2
CTC.444N2	9.36E-39	0.451226	0.123	0.045	1.49E-34	Skin_Peric	CTC.444N24.11
MYLIP1	9.45E-39	0.542998	0.293	0.163	1.51E-34	Skin_Peric	MYLIP
SEPW16	1.17E-38	0.424237	0.51	0.357	1.86E-34	Skin_Peric	SEPW1
PM20D21	1.28E-38	0.416528	0.114	0.04	2.05E-34	Skin_Peric	PM20D2
CTSL5	1.52E-38	0.489555	0.215	0.103	2.42E-34	Skin_Peric	CTSL
A1BG2	1.95E-38	0.372557	0.154	0.062	3.11E-34	Skin_Peric	A1BG
ARL34	2.21E-38	0.504435	0.225	0.114	3.52E-34	Skin_Peric	ARL3
FGF73	2.91E-38	0.520506	0.125	0.045	4.64E-34	Skin_Peric	FGF7
ANP32E2	3.29E-38	0.525335	0.203	0.096	5.25E-34	Skin_Peric	ANP32E
CAMK2G1	3.72E-38	0.388204	0.138	0.053	5.94E-34	Skin_Peric	CAMK2G
PLP25	7.01E-38	0.325831	0.393	0.23	1.12E-33	Skin_Peric	PLP2
CD444	8.82E-38	0.520963	0.516	0.347	1.41E-33	Skin_Peric	CD44
PPP1R15B1	1.01E-37	0.622824	0.271	0.151	1.61E-33	Skin_Peric	PPP1R15B
SH3PXD2A	1.1E-37	0.572398	0.182	0.083	1.76E-33	Skin_Peric	SH3PXD2A
BCAR1	1.18E-37	0.559899	0.166	0.074	1.89E-33	Skin_Peric	BCAR1
KLHL21	1.5E-37	0.55366	0.171	0.077	2.39E-33	Skin_Peric	KLHL21
RELT1	1.51E-37	0.335958	0.094	0.03	2.41E-33	Skin_Peric	RELT
PPP1R181	1.58E-37	0.386154	0.238	0.117	2.52E-33	Skin_Peric	PPP1R18
ZFAND1	4.57E-37	0.501139	0.222	0.112	7.29E-33	Skin_Peric	ZFAND1

BRD22	4.91E-37	0.656596	0.395	0.27	7.84E-33	Skin_Perici	BRD2
RSU11	5.14E-37	0.538389	0.234	0.122	8.21E-33	Skin_Perici	RSU1
RP11.983P	5.58E-37	0.325577	0.092	0.029	8.91E-33	Skin_Perici	RP11.983P16.4
NRARP3	5.79E-37	0.369764	0.112	0.04	9.23E-33	Skin_Perici	NRARP
TSPYL13	8.35E-37	0.572528	0.27	0.154	1.33E-32	Skin_Perici	TSPYL1
SNRPD21	9.29E-37	0.314082	0.615	0.431	1.48E-32	Skin_Perici	SNRPD2
DTX32	1.51E-36	0.291205	0.073	0.02	2.41E-32	Skin_Perici	DTX3
GADD45A	1.64E-36	0.355105	0.363	0.213	2.61E-32	Skin_Perici	GADD45A
ACKR32	1.9E-36	0.342664	0.089	0.027	3.03E-32	Skin_Perici	ACKR3
SNX31	2.2E-36	0.411895	0.562	0.415	3.52E-32	Skin_Perici	SNX3
UAP14	9.08E-36	1.097039	0.296	0.182	1.45E-31	Skin_Perici	UAP1
ZNF10	9.09E-36	0.295053	0.068	0.019	1.45E-31	Skin_Perici	ZNF10
TWISTNB1	9.54E-36	0.689179	0.193	0.093	1.52E-31	Skin_Perici	TWISTNB
PLAUR2	1.49E-35	0.277773	0.236	0.118	2.38E-31	Skin_Perici	PLAUR
RSL1D1	1.8E-35	0.457235	0.387	0.247	2.87E-31	Skin_Perici	RSL1D1
MAPRE22	7.8E-35	0.438099	0.17	0.078	1.24E-30	Skin_Perici	MAPRE2
NDUFB79	1.42E-34	0.473504	0.504	0.372	2.26E-30	Skin_Perici	NDUFB7
SLC25A25	1.44E-34	0.470246	0.144	0.061	2.3E-30	Skin_Perici	SLC25A25
UGDH1	2.92E-34	0.441754	0.163	0.072	4.65E-30	Skin_Perici	UGDH
EIF12	3.19E-34	0.279658	0.979	0.908	5.09E-30	Skin_Perici	EIF1
SLC39A14	3.92E-34	0.386036	0.13	0.051	6.25E-30	Skin_Perici	SLC39A14
MAP3K82	5.44E-34	0.468415	0.224	0.115	8.67E-30	Skin_Perici	MAP3K8
ARPC1A2	1.61E-33	0.628025	0.329	0.215	2.56E-29	Skin_Perici	ARPC1A
RHEB2	1.82E-33	0.564637	0.458	0.337	2.9E-29	Skin_Perici	RHEB
PHLDB13	2.37E-33	0.361772	0.123	0.048	3.78E-29	Skin_Perici	PHLDB1
NAB11	3.55E-33	0.462013	0.143	0.061	5.67E-29	Skin_Perici	NAB1
ANXA62	4.3E-33	0.33473	0.218	0.107	6.85E-29	Skin_Perici	ANXA6
SERBP11	4.61E-33	0.449489	0.496	0.353	7.36E-29	Skin_Perici	SERBP1
TRA2B1	5.38E-33	0.489746	0.418	0.288	8.58E-29	Skin_Perici	TRA2B
CALHM23	9.54E-33	0.296745	0.098	0.034	1.52E-28	Skin_Perici	CALHM2
ATP5O1	1.08E-32	0.40554	0.561	0.429	1.73E-28	Skin_Perici	ATP5O
VCAM12	1.26E-32	0.543481	0.094	0.032	2.01E-28	Skin_Perici	VCAM1
FN3K	1.63E-32	0.345013	0.082	0.027	2.6E-28	Skin_Perici	FN3K
ANTXR13	2.04E-32	0.335371	0.124	0.049	3.26E-28	Skin_Perici	ANTXR1
HSPA84	2.33E-32	0.356591	0.654	0.509	3.72E-28	Skin_Perici	HSPA8
MIF1	2.39E-32	0.297166	0.643	0.48	3.82E-28	Skin_Perici	MIF
SNTA11	4.7E-32	0.309597	0.081	0.026	7.49E-28	Skin_Perici	SNTA1
VDAC12	6.8E-32	0.774664	0.348	0.241	1.08E-27	Skin_Perici	VDAC1
KLF11	8.38E-32	0.44639	0.153	0.07	1.34E-27	Skin_Perici	KLF11
USMG52	2.16E-31	0.438992	0.563	0.435	3.44E-27	Skin_Perici	USMG5
COX7C4	2.3E-31	0.30431	0.8	0.636	3.67E-27	Skin_Perici	COX7C
VASN	2.36E-31	0.472106	0.234	0.127	3.77E-27	Skin_Perici	VASN
COX7B7	3.03E-31	0.467779	0.53	0.408	4.84E-27	Skin_Perici	COX7B
TRIM473	3.69E-31	0.353737	0.137	0.058	5.88E-27	Skin_Perici	TRIM47
CCDC85B2	3.91E-31	0.329023	0.418	0.271	6.24E-27	Skin_Perici	CCDC85B
RBM7	6.2E-31	0.564714	0.215	0.117	9.89E-27	Skin_Perici	RBM7
MYADM7	7.03E-31	0.437877	0.264	0.149	1.12E-26	Skin_Perici	MYADM

PIP5K1B	3.39E-30	0.390899	0.09	0.032	5.4E-26	Skin_Peric	PIP5K1B
UBXN11	3.79E-30	0.344549	0.526	0.39	6.04E-26	Skin_Peric	UBXN1
BAG3	3.91E-30	0.481831	0.256	0.148	6.24E-26	Skin_Peric	BAG3
PITPNB	4.71E-30	0.518701	0.213	0.116	7.51E-26	Skin_Peric	PITPNB
DDAH24	6.92E-30	0.435124	0.33	0.209	1.1E-25	Skin_Peric	DDAH2
FLNC2	1.05E-29	0.3256	0.05	0.012	1.67E-25	Skin_Peric	FLNC
SCML1	1.49E-29	0.38063	0.152	0.07	2.37E-25	Skin_Peric	SCML1
FXYD65	1.58E-29	0.303274	0.085	0.029	2.51E-25	Skin_Peric	FXYD6
FKBP4	2.35E-29	0.56891	0.272	0.169	3.75E-25	Skin_Peric	FKBP4
PDLIM13	2.65E-29	0.486184	0.341	0.223	4.23E-25	Skin_Peric	PDLIM1
CXCL31	4.5E-29	0.440248	0.243	0.133	7.18E-25	Skin_Peric	CXCL3
G3BP11	5.33E-29	0.488893	0.267	0.16	8.5E-25	Skin_Peric	G3BP1
ATP5I	5.96E-29	0.405071	0.497	0.371	9.51E-25	Skin_Peric	ATP5I
WDR74	7.03E-29	0.518146	0.196	0.101	1.12E-24	Skin_Peric	WDR74
CDK18	7.1E-29	0.252736	0.058	0.016	1.13E-24	Skin_Peric	CDK18
SLCO3A14	9.58E-29	0.428419	0.125	0.053	1.53E-24	Skin_Peric	SLCO3A1
ZNF263	1E-28	0.396182	0.108	0.043	1.6E-24	Skin_Peric	ZNF263
SARS1	2.02E-28	0.478357	0.259	0.155	3.22E-24	Skin_Peric	SARS
BCL31	4.14E-28	0.502407	0.191	0.1	6.6E-24	Skin_Peric	BCL3
RAD23A	4.63E-28	0.431327	0.319	0.205	7.38E-24	Skin_Peric	RAD23A
MRFAP12	6.66E-28	0.476874	0.377	0.265	1.06E-23	Skin_Peric	MRFAP1
DNTTIP23	6.75E-28	0.493204	0.197	0.106	1.08E-23	Skin_Peric	DNTTIP2
COPS8	7.94E-28	0.456459	0.219	0.123	1.27E-23	Skin_Peric	COPS8
SPTSSA	8.28E-28	0.49915	0.356	0.244	1.32E-23	Skin_Peric	SPTSSA
FRMD31	1.18E-27	0.32189	0.067	0.021	1.87E-23	Skin_Peric	FRMD3
EIF57	1.41E-27	0.504995	0.467	0.359	2.25E-23	Skin_Peric	EIF5
DHX15	1.47E-27	0.652859	0.2	0.111	2.34E-23	Skin_Peric	DHX15
DEK5	1.72E-27	0.451277	0.374	0.258	2.74E-23	Skin_Peric	DEK
SEMA5A3	1.73E-27	0.311015	0.076	0.026	2.76E-23	Skin_Peric	SEMA5A
UBE2D32	1.88E-27	0.421237	0.657	0.532	2.99E-23	Skin_Peric	UBE2D3
PSMB61	2.25E-27	0.405945	0.432	0.321	3.59E-23	Skin_Peric	PSMB6
ALKBH5	2.76E-27	0.527089	0.223	0.129	4.39E-23	Skin_Peric	ALKBH5
MIDN	3.22E-27	0.354837	0.402	0.277	5.14E-23	Skin_Peric	MIDN
RP11.138A	4.25E-27	0.271903	0.113	0.046	6.78E-23	Skin_Peric	RP11.138A9.2
DPYSL25	5.58E-27	0.402532	0.19	0.098	8.9E-23	Skin_Peric	DPYSL2
GABARAPL	7.63E-27	0.678068	0.264	0.167	1.22E-22	Skin_Peric	GABARAPL1
CXorf40A	1.39E-26	0.298182	0.066	0.021	2.22E-22	Skin_Peric	CXorf40A
MAFK	4.59E-26	0.414811	0.127	0.057	7.32E-22	Skin_Peric	MAFK
GLS5	4.73E-26	0.434811	0.21	0.116	7.55E-22	Skin_Peric	GLS
SHFM1	5.02E-26	0.418269	0.535	0.43	8.01E-22	Skin_Peric	SHFM1
COX7A2L	6.02E-26	0.402678	0.436	0.32	9.59E-22	Skin_Peric	COX7A2L
C12orf751	1.08E-25	0.359716	0.24	0.136	1.73E-21	Skin_Peric	C12orf75
SRP91	1.21E-25	0.461587	0.443	0.341	1.93E-21	Skin_Peric	SRP9
TUBB2B1	1.32E-25	0.322196	0.062	0.019	2.11E-21	Skin_Peric	TUBB2B
NFKB11	1.44E-25	0.567313	0.21	0.119	2.29E-21	Skin_Peric	NFKB1
RBPMS3	1.53E-25	0.530603	0.279	0.183	2.45E-21	Skin_Peric	RBPMS
RUNX1T13	1.7E-25	0.296165	0.099	0.039	2.71E-21	Skin_Peric	RUNX1T1

RBMS34	2.13E-25	0.304337	0.119	0.051	3.39E-21	Skin_Peric	RBMS3
AP2M11	2.98E-25	0.399372	0.411	0.303	4.75E-21	Skin_Peric	AP2M1
ARL4D1	2.98E-25	0.93229	0.176	0.094	4.76E-21	Skin_Peric	ARL4D
TRMT1122	3.08E-25	0.352908	0.496	0.373	4.91E-21	Skin_Peric	TRMT112
UBE2S4	3.38E-25	0.503945	0.224	0.131	5.39E-21	Skin_Peric	UBE2S
SNN1	3.83E-25	0.352238	0.102	0.042	6.11E-21	Skin_Peric	SNN
RABGGTB2	5.2E-25	0.446571	0.183	0.1	8.3E-21	Skin_Peric	RABGGTB
SRSF63	6.93E-25	0.555014	0.234	0.144	1.1E-20	Skin_Peric	SRSF6
KMT2E3	7.78E-25	0.498244	0.401	0.299	1.24E-20	Skin_Peric	KMT2E
AHR4	8.7E-25	0.433978	0.197	0.107	1.39E-20	Skin_Peric	AHR
FRMD4A3	9.89E-25	0.360673	0.114	0.051	1.58E-20	Skin_Peric	FRMD4A
MRPS63	1.22E-24	0.402999	0.284	0.181	1.95E-20	Skin_Peric	MRPS6
NR3C15	2.4E-24	0.450702	0.244	0.148	3.83E-20	Skin_Peric	NR3C1
RANBP24	2.65E-24	0.58969	0.199	0.116	4.22E-20	Skin_Peric	RANBP2
SLC25A312	2.74E-24	0.308708	0.578	0.454	4.37E-20	Skin_Peric	SLC25A3
CKS1B2	3.49E-24	0.487073	0.231	0.139	5.56E-20	Skin_Peric	CKS1B
NFE2L22	4.58E-24	0.524002	0.387	0.287	7.31E-20	Skin_Peric	NFE2L2
RBM394	5.23E-24	0.371004	0.518	0.41	8.35E-20	Skin_Peric	RBM39
C11orf581	5.33E-24	0.416151	0.468	0.367	8.51E-20	Skin_Peric	C11orf58
MICAL11	6.77E-24	0.27151	0.086	0.033	1.08E-19	Skin_Peric	MICAL1
ZNF1312	1.01E-23	0.422383	0.158	0.082	1.61E-19	Skin_Peric	ZNF131
PQBP12	1.05E-23	0.373365	0.25	0.153	1.67E-19	Skin_Peric	PQBP1
EIF1AX2	1.15E-23	0.481291	0.433	0.336	1.84E-19	Skin_Peric	EIF1AX
RGS31	1.36E-23	0.307009	0.101	0.042	2.18E-19	Skin_Peric	RGS3
ETS13	2.12E-23	0.291316	0.154	0.076	3.38E-19	Skin_Peric	ETS1
PLK22	2.44E-23	0.498197	0.161	0.085	3.9E-19	Skin_Peric	PLK2
FBL	2.94E-23	0.293836	0.353	0.235	4.69E-19	Skin_Peric	FBL
COX142	3.59E-23	0.436366	0.33	0.231	5.73E-19	Skin_Peric	COX14
CFL22	3.91E-23	0.501235	0.175	0.097	6.24E-19	Skin_Peric	CFL2
UTRN5	3.96E-23	0.377868	0.203	0.115	6.31E-19	Skin_Peric	UTRN
SNHG84	4.6E-23	0.476593	0.411	0.296	7.34E-19	Skin_Peric	SNHG8
UQCRQ5	4.67E-23	0.302756	0.619	0.498	7.45E-19	Skin_Peric	UQCRQ
IFI164	8.65E-23	0.440311	0.301	0.198	1.38E-18	Skin_Peric	IFI16
USP362	8.75E-23	0.392453	0.131	0.063	1.4E-18	Skin_Peric	USP36
DUT	9.22E-23	0.409149	0.337	0.235	1.47E-18	Skin_Peric	DUT
RAB134	1.02E-22	0.533934	0.306	0.218	1.63E-18	Skin_Peric	RAB13
EPHX13	1.15E-22	0.595372	0.204	0.121	1.83E-18	Skin_Peric	EPHX1
FBLIM11	1.19E-22	0.451415	0.125	0.061	1.89E-18	Skin_Peric	FBLIM1
CSF13	1.42E-22	0.257671	0.084	0.033	2.27E-18	Skin_Peric	CSF1
CRTC31	2.47E-22	0.380991	0.123	0.059	3.94E-18	Skin_Peric	CRTC3
BCAS21	3.03E-22	0.506886	0.239	0.152	4.84E-18	Skin_Peric	BCAS2
ARHGEF71	3.39E-22	0.434363	0.129	0.064	5.41E-18	Skin_Peric	ARHGEF7
LINC00998	3.41E-22	0.390431	0.135	0.067	5.44E-18	Skin_Peric	LINC00998
ICAM12	3.62E-22	0.50123	0.228	0.135	5.77E-18	Skin_Peric	ICAM1
NDUFAF4	5.47E-22	0.395414	0.154	0.081	8.72E-18	Skin_Peric	NDUFAF4
TMED26	5.51E-22	0.375701	0.448	0.345	8.79E-18	Skin_Peric	TMED2
HNRNPAB4	7.56E-22	0.440837	0.257	0.166	1.21E-17	Skin_Peric	HNRNPAB

HIF1A3	7.77E-22	0.467929	0.253	0.165	1.24E-17	Skin_Peric	HIF1A
CYTH32	8.21E-22	0.276849	0.075	0.029	1.31E-17	Skin_Peric	CYTH3
ANGPTL4	9.71E-22	0.390381	0.163	0.086	1.55E-17	Skin_Peric	ANGPTL4
COX5B3	1.01E-21	0.30621	0.676	0.565	1.61E-17	Skin_Peric	COX5B
STRN3	1.22E-21	0.429612	0.167	0.092	1.94E-17	Skin_Peric	STRN3
SNRPB3	1.5E-21	0.368887	0.438	0.332	2.39E-17	Skin_Peric	SNRPB
EMD1	1.77E-21	0.440599	0.207	0.125	2.83E-17	Skin_Peric	EMD
EIF4G2	2.97E-21	0.297379	0.56	0.459	4.74E-17	Skin_Peric	EIF4G2
PRMT13	4.22E-21	0.391993	0.288	0.191	6.73E-17	Skin_Peric	PRMT1
SLC25A191	4.68E-21	0.268246	0.084	0.034	7.46E-17	Skin_Peric	SLC25A19
RWDD1	7.99E-21	0.456177	0.4	0.307	1.27E-16	Skin_Peric	RWDD1
METTL152	8.52E-21	0.339531	0.105	0.048	1.36E-16	Skin_Peric	METTL15
REV3L1	1.25E-20	0.445969	0.165	0.091	1.99E-16	Skin_Peric	REV3L
MINOS1	1.48E-20	0.366192	0.406	0.306	2.36E-16	Skin_Peric	MINOS1
HSPA21	1.5E-20	0.453283	0.134	0.069	2.39E-16	Skin_Peric	HSPA2
DEGS1	1.64E-20	0.377317	0.208	0.126	2.62E-16	Skin_Peric	DEGS1
HNRNPC2	2.55E-20	0.37122	0.527	0.429	4.06E-16	Skin_Peric	HNRNPC
ATP1A19	2.92E-20	0.391569	0.35	0.255	4.66E-16	Skin_Peric	ATP1A1
NTN43	3.12E-20	0.404905	0.125	0.062	4.97E-16	Skin_Peric	NTN4
PPP2CA1	3.18E-20	0.53611	0.316	0.232	5.07E-16	Skin_Peric	PPP2CA
C7orf502	3.24E-20	0.389864	0.215	0.134	5.16E-16	Skin_Peric	C7orf50
RAP2A2	5.88E-20	0.316796	0.099	0.045	9.37E-16	Skin_Peric	RAP2A
EIF1AY3	6.39E-20	0.37331	0.156	0.086	1.02E-15	Skin_Peric	EIF1AY
CTA.29F11	9.66E-20	0.292749	0.137	0.07	1.54E-15	Skin_Peric	CTA.29F11.1
SEPT24	1.12E-19	0.500334	0.317	0.235	1.79E-15	Skin_Peric	SEPT2
ACTN43	1.21E-19	0.343556	0.404	0.307	1.93E-15	Skin_Peric	ACTN4
EIF4B5	1.28E-19	0.374054	0.389	0.299	2.04E-15	Skin_Peric	EIF4B
DENND5A1	1.47E-19	0.281293	0.093	0.041	2.34E-15	Skin_Peric	DENND5A
ATP5C13	1.49E-19	0.366901	0.371	0.281	2.37E-15	Skin_Peric	ATP5C1
APP4	1.99E-19	0.384081	0.337	0.243	3.17E-15	Skin_Peric	APP
PRKRA	2.08E-19	0.399583	0.203	0.125	3.33E-15	Skin_Peric	PRKRA
C5orf241	2.39E-19	0.466589	0.184	0.111	3.81E-15	Skin_Peric	C5orf24
CDS2	2.51E-19	0.420884	0.136	0.072	4E-15	Skin_Peric	CDS2
MARCH61	2.9E-19	0.478424	0.244	0.164	4.62E-15	Skin_Peric	MARCH6
KLHL423	3.45E-19	0.276678	0.085	0.036	5.51E-15	Skin_Peric	KLHL42
PYURF	5.5E-19	0.414607	0.334	0.247	8.77E-15	Skin_Peric	PYURF
RANBP11	6.19E-19	0.300276	0.369	0.266	9.88E-15	Skin_Peric	RANBP1
SSB6	7.47E-19	0.327665	0.291	0.197	1.19E-14	Skin_Peric	SSB
PAWR4	9.08E-19	0.508311	0.202	0.13	1.45E-14	Skin_Peric	PAWR
IPO73	1.29E-18	0.439996	0.174	0.104	2.06E-14	Skin_Peric	IPO7
NGFRAP12	1.36E-18	0.431014	0.292	0.206	2.17E-14	Skin_Peric	NGFRAP1
FN11	1.49E-18	0.321734	0.176	0.098	2.38E-14	Skin_Peric	FN1
CYC12	1.59E-18	0.431033	0.313	0.23	2.54E-14	Skin_Peric	CYC1
TMEM165	1.68E-18	0.436132	0.287	0.204	2.69E-14	Skin_Peric	TMEM165
YWHAE1	1.88E-18	0.370221	0.484	0.397	3E-14	Skin_Peric	YWHAE
ZC3HAV12	1.91E-18	0.431025	0.168	0.097	3.05E-14	Skin_Peric	ZC3HAV1
MAP45	2.32E-18	0.446193	0.203	0.128	3.71E-14	Skin_Peric	MAP4

FAM127A2	2.7E-18	0.364704	0.159	0.091	4.3E-14	Skin_Peric	FAM127A
RAB211	2.72E-18	0.413764	0.247	0.165	4.34E-14	Skin_Peric	RAB21
GSTM4	3.01E-18	0.280451	0.085	0.037	4.8E-14	Skin_Peric	GSTM4
DCUN1D3	3.77E-18	0.261571	0.079	0.033	6.01E-14	Skin_Peric	DCUN1D3
AP006222.	4.75E-18	0.271061	0.089	0.04	7.57E-14	Skin_Peric	AP006222.2
PAN32	5.01E-18	0.389767	0.145	0.081	8E-14	Skin_Peric	PAN3
B3GALNT2	5.73E-18	0.308065	0.091	0.042	9.14E-14	Skin_Peric	B3GALNT2
PMVK2	6.47E-18	0.392594	0.207	0.131	1.03E-13	Skin_Peric	PMVK
INPP5A1	7.45E-18	0.25694	0.086	0.038	1.19E-13	Skin_Peric	INPP5A
ARPC53	7.55E-18	0.351133	0.509	0.412	1.2E-13	Skin_Peric	ARPC5
SUMO12	7.8E-18	0.391429	0.425	0.346	1.24E-13	Skin_Peric	SUMO1
AHSA1	7.93E-18	0.404781	0.221	0.144	1.26E-13	Skin_Peric	AHSA1
NFKBIZ1	9.16E-18	0.448809	0.331	0.245	1.46E-13	Skin_Peric	NFKBIZ
CHMP4B1	9.94E-18	0.354809	0.401	0.306	1.58E-13	Skin_Peric	CHMP4B
GPATCH4	1.55E-17	0.375264	0.124	0.065	2.47E-13	Skin_Peric	GPATCH4
ARHGAP1C	1.63E-17	0.316807	0.145	0.08	2.6E-13	Skin_Peric	ARHGAP10
ARHGEF21	1.69E-17	0.345177	0.13	0.069	2.69E-13	Skin_Peric	ARHGEF2
PMEPA14	1.84E-17	0.404714	0.191	0.119	2.93E-13	Skin_Peric	PMEPA1
ALG13	1.95E-17	0.883418	0.176	0.11	3.1E-13	Skin_Peric	ALG13
SRRM28	2.19E-17	0.329384	0.431	0.347	3.49E-13	Skin_Peric	SRRM2
CAMLG	3.23E-17	0.293608	0.341	0.248	5.15E-13	Skin_Peric	CAMLG
LUC7L36	3.67E-17	0.385138	0.349	0.265	5.85E-13	Skin_Peric	LUC7L3
HSPA92	4.39E-17	0.432499	0.239	0.164	7E-13	Skin_Peric	HSPA9
KIF5B4	6.13E-17	0.455484	0.364	0.29	9.78E-13	Skin_Peric	KIF5B
GPBP12	6.63E-17	0.444996	0.401	0.32	1.06E-12	Skin_Peric	GPBP1
TCEAL43	6.85E-17	0.362661	0.236	0.159	1.09E-12	Skin_Peric	TCEAL4
BTBD72	7.17E-17	0.449692	0.163	0.098	1.14E-12	Skin_Peric	BTBD7
TMEM173	8.78E-17	0.29991	0.184	0.11	1.4E-12	Skin_Peric	TMEM173
PHPT18	9.22E-17	0.325241	0.414	0.324	1.47E-12	Skin_Peric	PHPT1
UBE2E11	1.04E-16	0.45181	0.208	0.138	1.66E-12	Skin_Peric	UBE2E1
MAP7D32	1.16E-16	0.329742	0.12	0.063	1.85E-12	Skin_Peric	MAP7D3
ATP5A14	1.44E-16	0.250456	0.407	0.312	2.3E-12	Skin_Peric	ATP5A1
NOP561	1.49E-16	0.367659	0.197	0.124	2.37E-12	Skin_Peric	NOP56
SNRPE1	1.52E-16	0.361495	0.351	0.266	2.43E-12	Skin_Peric	SNRPE
BCOR	1.55E-16	0.320047	0.113	0.058	2.47E-12	Skin_Peric	BCOR
ST53	1.55E-16	0.349582	0.114	0.06	2.48E-12	Skin_Peric	ST5
RASSF32	1.6E-16	0.412118	0.134	0.075	2.55E-12	Skin_Peric	RASSF3
EPB41L4A.	1.7E-16	0.401155	0.278	0.198	2.71E-12	Skin_Peric	EPB41L4A.AS1
ARHGAP17	1.72E-16	0.257619	0.08	0.036	2.74E-12	Skin_Peric	ARHGAP17
QTRT11	2.28E-16	0.322126	0.134	0.074	3.63E-12	Skin_Peric	QTRT1
VEGFA2	2.41E-16	0.41695	0.146	0.083	3.84E-12	Skin_Peric	VEGFA
RELB2	2.43E-16	0.346391	0.122	0.065	3.88E-12	Skin_Peric	RELB
STARD72	3.03E-16	0.392293	0.157	0.094	4.84E-12	Skin_Peric	STARD7
COPS6	3.37E-16	0.374708	0.288	0.211	5.37E-12	Skin_Peric	COPS6
BNIP3L2	3.4E-16	0.560353	0.361	0.296	5.42E-12	Skin_Peric	BNIP3L
NRP14	3.82E-16	0.27151	0.111	0.056	6.1E-12	Skin_Peric	NRP1
PTPRS1	4.44E-16	0.29273	0.118	0.062	7.08E-12	Skin_Peric	PTPRS

PPP1CC	7.33E-16	0.338643	0.331	0.248	1.17E-11	Skin_Peric	PPP1CC
GNL31	9.84E-16	0.565996	0.273	0.204	1.57E-11	Skin_Peric	GNL3
C8orf47	1E-15	0.693759	0.188	0.119	1.6E-11	Skin_Peric	C8orf4
CHURC15	1.13E-15	0.407194	0.291	0.216	1.81E-11	Skin_Peric	CHURC1
ZC3H12A2	1.64E-15	0.362291	0.167	0.101	2.61E-11	Skin_Peric	ZC3H12A
TGIF2	2.22E-15	0.266835	0.077	0.035	3.54E-11	Skin_Peric	TGIF2
LZTS23	2.5E-15	0.279119	0.087	0.042	3.99E-11	Skin_Peric	LZTS2
ZEB13	2.53E-15	0.270701	0.112	0.059	4.03E-11	Skin_Peric	ZEB1
RARA2	2.58E-15	0.270816	0.106	0.055	4.12E-11	Skin_Peric	RARA
FAM110B1	3.32E-15	0.285639	0.082	0.039	5.29E-11	Skin_Peric	FAM110B
NEDD83	3.5E-15	0.257781	0.501	0.422	5.59E-11	Skin_Peric	NEDD8
NUDC1	3.5E-15	0.330218	0.33	0.25	5.59E-11	Skin_Peric	NUDC
UBE2E21	3.53E-15	0.364154	0.137	0.079	5.63E-11	Skin_Peric	UBE2E2
SRSF77	3.55E-15	0.285677	0.426	0.342	5.67E-11	Skin_Peric	SRSF7
SAV11	3.6E-15	0.403269	0.17	0.106	5.74E-11	Skin_Peric	SAV1
EIF3D	3.75E-15	0.288812	0.366	0.284	5.97E-11	Skin_Peric	EIF3D
MEF2A3	4.34E-15	0.409589	0.17	0.106	6.92E-11	Skin_Peric	MEF2A
MCL14	6.12E-15	0.268081	0.466	0.38	9.76E-11	Skin_Peric	MCL1
SMIM4	6.85E-15	0.424979	0.133	0.078	1.09E-10	Skin_Peric	SMIM4
ARMCX33	7.87E-15	0.411719	0.212	0.145	1.25E-10	Skin_Peric	ARMCX3
SEC23A1	7.94E-15	0.284945	0.125	0.07	1.27E-10	Skin_Peric	SEC23A
ATXN13	1.04E-14	0.309288	0.127	0.072	1.66E-10	Skin_Peric	ATXN1
RP11.553L	1.15E-14	0.29758	0.1	0.051	1.84E-10	Skin_Peric	RP11.553L6.5
CSDE14	1.32E-14	0.364564	0.422	0.358	2.11E-10	Skin_Peric	CSDE1
DDX3X4	1.34E-14	0.356956	0.373	0.301	2.14E-10	Skin_Peric	DDX3X
NACA22	1.39E-14	0.270165	0.129	0.072	2.22E-10	Skin_Peric	NACA2
SMIM121	1.48E-14	0.366063	0.168	0.106	2.37E-10	Skin_Peric	SMIM12
THAP9.AS1	1.54E-14	0.340834	0.136	0.079	2.46E-10	Skin_Peric	THAP9.AS1
HMGN35	1.69E-14	0.306277	0.359	0.278	2.69E-10	Skin_Peric	HMGN3
DDIT31	1.73E-14	0.307102	0.227	0.154	2.76E-10	Skin_Peric	DDIT3
ECE12	1.95E-14	0.274308	0.114	0.062	3.11E-10	Skin_Peric	ECE1
ZNF428	2.17E-14	0.306542	0.239	0.166	3.46E-10	Skin_Peric	ZNF428
PDS5B1	3.9E-14	0.34896	0.14	0.083	6.23E-10	Skin_Peric	PDS5B
PRDX63	3.92E-14	0.272562	0.448	0.374	6.25E-10	Skin_Peric	PRDX6
SNRPF2	4.08E-14	0.31604	0.317	0.237	6.51E-10	Skin_Peric	SNRPF
LSM54	4.12E-14	0.376173	0.271	0.201	6.57E-10	Skin_Peric	LSM5
SLC3A22	6.69E-14	0.326598	0.271	0.197	1.07E-09	Skin_Peric	SLC3A2
CDC423	6.74E-14	0.258068	0.545	0.47	1.08E-09	Skin_Peric	CDC42
TLE3	6.86E-14	0.393629	0.16	0.101	1.09E-09	Skin_Peric	TLE3
POLE41	8.48E-14	0.308716	0.236	0.165	1.35E-09	Skin_Peric	POLE4
RBM64	8.52E-14	0.374546	0.172	0.111	1.36E-09	Skin_Peric	RBM6
UBE2W1	9.19E-14	0.46279	0.18	0.12	1.47E-09	Skin_Peric	UBE2W
OPTN	1.22E-13	0.30843	0.266	0.193	1.95E-09	Skin_Peric	OPTN
LMCD12	1.47E-13	0.451202	0.14	0.084	2.34E-09	Skin_Peric	LMCD1
CDK172	1.47E-13	0.285057	0.081	0.04	2.34E-09	Skin_Peric	CDK17
FOXK1	1.69E-13	0.308546	0.099	0.053	2.69E-09	Skin_Peric	FOXK1
PDCL31	1.69E-13	0.267637	0.14	0.083	2.7E-09	Skin_Peric	PDCL3

ATP6V0A1	2.01E-13	0.325316	0.104	0.056	3.2E-09	Skin_Peric	ATP6V0A1
COX202	2.19E-13	0.399258	0.23	0.165	3.49E-09	Skin_Peric	COX20
VASP4	2.21E-13	0.312419	0.195	0.129	3.53E-09	Skin_Peric	VASP
TNFRSF1A3	2.64E-13	0.427886	0.226	0.16	4.21E-09	Skin_Peric	TNFRSF1A
STIP1	2.64E-13	0.371592	0.183	0.122	4.21E-09	Skin_Peric	STIP1
ATN1	2.69E-13	0.305304	0.095	0.05	4.29E-09	Skin_Peric	ATN1
NDUFV14	2.81E-13	0.39921	0.237	0.172	4.48E-09	Skin_Peric	NDUFV1
C1orf563	4.69E-13	0.477887	0.183	0.121	7.47E-09	Skin_Peric	C1orf56
CRY2	6.21E-13	0.335338	0.1	0.055	9.9E-09	Skin_Peric	CRY2
ARL13B	7.58E-13	0.254739	0.093	0.049	1.21E-08	Skin_Peric	ARL13B
TAF9	7.83E-13	0.350838	0.285	0.216	1.25E-08	Skin_Peric	TAF9
DIMT1	8.61E-13	0.294478	0.111	0.063	1.37E-08	Skin_Peric	DIMT1
ST13	9.11E-13	0.255507	0.475	0.406	1.45E-08	Skin_Peric	ST13
PRPS11	1.05E-12	0.290087	0.109	0.061	1.68E-08	Skin_Peric	PRPS1
CDC373	1.92E-12	0.382895	0.337	0.274	3.06E-08	Skin_Peric	CDC37
NME45	2.09E-12	0.374767	0.202	0.142	3.33E-08	Skin_Peric	NME4
INPP4B2	2.09E-12	0.257628	0.103	0.057	3.34E-08	Skin_Peric	INPP4B
MRPL24	2.16E-12	0.307908	0.158	0.102	3.44E-08	Skin_Peric	MRPL24
PLEKHA3	2.23E-12	0.407201	0.183	0.124	3.55E-08	Skin_Peric	PLEKHA3
HIPK31	2.61E-12	0.326632	0.177	0.119	4.17E-08	Skin_Peric	HIPK3
EIF2S3	3.07E-12	0.339895	0.219	0.156	4.9E-08	Skin_Peric	EIF2S3
PABPC42	3.29E-12	0.312476	0.189	0.128	5.24E-08	Skin_Peric	PABPC4
SRSF1	3.54E-12	0.397825	0.214	0.154	5.65E-08	Skin_Peric	SRSF1
FKBP71	6.09E-12	0.272469	0.101	0.057	9.71E-08	Skin_Peric	FKBP7
CDK2AP22	7.34E-12	0.423844	0.227	0.165	1.17E-07	Skin_Peric	CDK2AP2
FAM129A9	7.69E-12	0.286134	0.16	0.104	1.23E-07	Skin_Peric	FAM129A
TROVE2	8.06E-12	0.33115	0.194	0.134	1.28E-07	Skin_Peric	TROVE2
NSRP1	8.15E-12	0.406455	0.206	0.148	1.3E-07	Skin_Peric	NSRP1
DCTN2	8.49E-12	0.35377	0.253	0.194	1.35E-07	Skin_Peric	DCTN2
SPG201	8.58E-12	0.318214	0.147	0.094	1.37E-07	Skin_Peric	SPG20
NDUFA52	9.85E-12	0.380305	0.308	0.251	1.57E-07	Skin_Peric	NDUFA5
UGCG1	1.04E-11	0.439479	0.166	0.111	1.65E-07	Skin_Peric	UGCG
SLMAP1	1.09E-11	0.584856	0.19	0.136	1.73E-07	Skin_Peric	SLMAP
YY12	1.51E-11	0.32345	0.288	0.227	2.41E-07	Skin_Peric	YY1
LRCH11	1.74E-11	0.268492	0.077	0.04	2.78E-07	Skin_Peric	LRCH1
YWHAG1	1.96E-11	0.356249	0.292	0.23	3.13E-07	Skin_Peric	YWHAG
LGALSL1	2.03E-11	0.314131	0.089	0.049	3.24E-07	Skin_Peric	LGALSL
HNRNPD2	2.63E-11	0.342266	0.298	0.24	4.19E-07	Skin_Peric	HNRNPD
NAA15	2.81E-11	0.300651	0.123	0.075	4.47E-07	Skin_Peric	NAA15
ITGB1BP13	2.97E-11	0.254757	0.215	0.151	4.74E-07	Skin_Peric	ITGB1BP1
PER21	3.99E-11	0.36213	0.134	0.085	6.37E-07	Skin_Peric	PER2
PRDX34	4.05E-11	0.42054	0.211	0.156	6.46E-07	Skin_Peric	PRDX3
RNPS1	4.57E-11	0.26417	0.265	0.198	7.28E-07	Skin_Peric	RNPS1
CDC73	6.09E-11	0.315816	0.128	0.08	9.72E-07	Skin_Peric	CDC73
EIF2A1	7.32E-11	0.324995	0.209	0.153	1.17E-06	Skin_Peric	EIF2A
SEPT114	7.99E-11	0.266295	0.137	0.086	1.27E-06	Skin_Peric	SEPT11
VPS37B1	8.66E-11	0.439618	0.208	0.153	1.38E-06	Skin_Peric	VPS37B

NDUFS42	8.93E-11	0.371576	0.272	0.218	1.42E-06	Skin_Peric	NDUFS4
RAB312	9.12E-11	0.2788	0.146	0.094	1.45E-06	Skin_Peric	RAB31
TAX1BP3	9.46E-11	0.284654	0.137	0.086	1.51E-06	Skin_Peric	TAX1BP3
UBE2D1	1.28E-10	0.323112	0.243	0.181	2.04E-06	Skin_Peric	UBE2D1
SOX41	1.28E-10	0.447366	0.327	0.258	2.05E-06	Skin_Peric	SOX4
IRF21	1.46E-10	0.279489	0.138	0.088	2.33E-06	Skin_Peric	IRF2
CTTN	1.46E-10	0.38908	0.261	0.207	2.34E-06	Skin_Peric	CTTN
MAPRE11	1.48E-10	0.37152	0.229	0.175	2.36E-06	Skin_Peric	MAPRE1
PGRMC14	1.64E-10	0.364066	0.221	0.166	2.61E-06	Skin_Peric	PGRMC1
BANF1	2.17E-10	0.303585	0.294	0.233	3.46E-06	Skin_Peric	BANF1
PNO1	2.77E-10	0.260151	0.097	0.056	4.43E-06	Skin_Peric	PNO1
RBPJ5	2.9E-10	0.32339	0.25	0.191	4.63E-06	Skin_Peric	RBPJ
TMEM51	3.07E-10	0.257995	0.075	0.04	4.9E-06	Skin_Peric	TMEM51
MBNL16	3.16E-10	0.257134	0.234	0.17	5.04E-06	Skin_Peric	MBNL1
ZNF3022	3.56E-10	0.296367	0.124	0.078	5.69E-06	Skin_Peric	ZNF302
RRAGC	4.07E-10	0.309764	0.151	0.102	6.49E-06	Skin_Peric	RRAGC
H2AFX1	5.83E-10	0.276986	0.107	0.065	9.3E-06	Skin_Peric	H2AFX
ROMO1	5.9E-10	0.317119	0.359	0.31	9.41E-06	Skin_Peric	ROMO1
TSC22D21	6.4E-10	0.259523	0.158	0.107	1.02E-05	Skin_Peric	TSC22D2
CDC42BPA	6.5E-10	0.292426	0.112	0.07	1.04E-05	Skin_Peric	CDC42BPA
CHORDC11	6.81E-10	0.307769	0.149	0.1	1.09E-05	Skin_Peric	CHORDC1
LINC00969	8.76E-10	0.348862	0.16	0.111	1.4E-05	Skin_Peric	LINC00969
GLRX51	8.81E-10	0.347773	0.222	0.171	1.41E-05	Skin_Peric	GLRX5
DTWD13	1.07E-09	0.28897	0.125	0.08	1.71E-05	Skin_Peric	DTWD1
IL6ST4	1.08E-09	0.338629	0.276	0.222	1.72E-05	Skin_Peric	IL6ST
HIPK28	1.16E-09	0.35294	0.122	0.079	1.85E-05	Skin_Peric	HIPK2
PSMC3	1.23E-09	0.332082	0.255	0.204	1.96E-05	Skin_Peric	PSMC3
FAM177A1	1.32E-09	0.368974	0.267	0.215	2.1E-05	Skin_Peric	FAM177A1
BTG31	1.61E-09	0.297401	0.215	0.159	2.57E-05	Skin_Peric	BTG3
DAPK3	1.83E-09	0.298246	0.131	0.086	2.92E-05	Skin_Peric	DAPK3
SRSF115	1.93E-09	0.253235	0.359	0.302	3.07E-05	Skin_Peric	SRSF11
NFIC7	2.33E-09	0.278622	0.225	0.171	3.72E-05	Skin_Peric	NFIC
NMB1	2.43E-09	0.41253	0.114	0.073	3.88E-05	Skin_Peric	NMB
PA2G4	2.57E-09	0.257683	0.304	0.244	4.1E-05	Skin_Peric	PA2G4
LDLR	2.66E-09	0.303774	0.149	0.102	4.23E-05	Skin_Peric	LDLR
RBM258	2.96E-09	0.280617	0.295	0.239	4.73E-05	Skin_Peric	RBM25
NUTF2	3.4E-09	0.321812	0.181	0.132	5.43E-05	Skin_Peric	NUTF2
CEBPZ	3.42E-09	0.284974	0.17	0.12	5.46E-05	Skin_Peric	CEBPZ
POLR2F	3.68E-09	0.297752	0.304	0.252	5.87E-05	Skin_Peric	POLR2F
ARL5A1	3.81E-09	0.327171	0.247	0.196	6.07E-05	Skin_Peric	ARL5A
PPIG7	3.83E-09	0.292371	0.302	0.249	6.11E-05	Skin_Peric	PPIG
SUCLG2	3.93E-09	0.316238	0.167	0.119	6.26E-05	Skin_Peric	SUCLG2
SYF2	4.01E-09	0.266419	0.378	0.327	6.4E-05	Skin_Peric	SYF2
BCL7B	4.62E-09	0.272595	0.148	0.101	7.36E-05	Skin_Peric	BCL7B
GBE1	5.11E-09	0.271076	0.093	0.056	8.14E-05	Skin_Peric	GBE1
UBR1	7.62E-09	0.253279	0.07	0.039	0.000122	Skin_Peric	UBR1
SYPL15	8.19E-09	0.271902	0.314	0.263	0.000131	Skin_Peric	SYPL1

PSMA4	9.88E-09	0.281417	0.309	0.256	0.000158	Skin_Perio	PSMA4
CREB33	1E-08	0.31411	0.177	0.131	0.00016	Skin_Perio	CREB3
MPRIP	1.21E-08	0.271187	0.224	0.171	0.000193	Skin_Perio	MPRIP
HYI4	1.22E-08	0.30055	0.128	0.086	0.000194	Skin_Perio	HYI
NOC2L	1.3E-08	0.255363	0.127	0.085	0.000207	Skin_Perio	NOC2L
PSMD12	1.38E-08	0.384913	0.197	0.152	0.000221	Skin_Perio	PSMD12
TFRC3	1.4E-08	0.283382	0.089	0.054	0.000223	Skin_Perio	TFRC
PABPN14	1.41E-08	0.323976	0.198	0.151	0.000225	Skin_Perio	PABPN1
ABI23	1.42E-08	0.301777	0.13	0.088	0.000227	Skin_Perio	ABI2
NFKB22	1.5E-08	0.265994	0.119	0.078	0.00024	Skin_Perio	NFKB2
AK11	1.51E-08	0.306645	0.189	0.142	0.000241	Skin_Perio	AK1
TERF2IP2	1.53E-08	0.275588	0.278	0.224	0.000244	Skin_Perio	TERF2IP
NEK71	1.55E-08	0.250429	0.12	0.079	0.000247	Skin_Perio	NEK7
FGFR1OP2	1.72E-08	0.284194	0.204	0.154	0.000275	Skin_Perio	FGFR1OP2
SENP66	1.77E-08	0.317199	0.152	0.108	0.000282	Skin_Perio	SENP6
ASXL11	1.94E-08	0.312086	0.119	0.079	0.00031	Skin_Perio	ASXL1
PLOD2	2.23E-08	0.263141	0.07	0.039	0.000355	Skin_Perio	PLOD2
TRA2A2	3.24E-08	0.366077	0.21	0.164	0.000517	Skin_Perio	TRA2A
NT5C3B1	4.76E-08	0.316773	0.136	0.096	0.000759	Skin_Perio	NT5C3B
NDUFA63	4.89E-08	0.300936	0.359	0.321	0.00078	Skin_Perio	NDUFA6
PURA7	5.51E-08	0.344098	0.168	0.125	0.000878	Skin_Perio	PURA
FBXL3	6.76E-08	0.323529	0.146	0.105	0.001078	Skin_Perio	FBXL3
ABHD21	7.47E-08	0.345589	0.157	0.115	0.001191	Skin_Perio	ABHD2
TCP1	7.62E-08	0.303427	0.291	0.246	0.001216	Skin_Perio	TCP1
CRK2	8.85E-08	0.303417	0.162	0.119	0.001412	Skin_Perio	CRK
AAMDC1	1E-07	0.308177	0.196	0.153	0.001595	Skin_Perio	AAMDC
RBBP61	1.14E-07	0.28371	0.215	0.167	0.001824	Skin_Perio	RBBP6
YAF21	1.34E-07	0.30955	0.138	0.097	0.002142	Skin_Perio	YAF2
LONRF1	1.41E-07	0.255944	0.093	0.06	0.002249	Skin_Perio	LONRF1
NONO	1.43E-07	0.308898	0.234	0.191	0.002274	Skin_Perio	NONO
CDC37L1	1.45E-07	0.328229	0.121	0.083	0.002305	Skin_Perio	CDC37L1
ATP2A24	1.99E-07	0.380163	0.215	0.175	0.003171	Skin_Perio	ATP2A2
IMP31	2.56E-07	0.261905	0.234	0.187	0.004088	Skin_Perio	IMP3
CAMSAP21	2.96E-07	0.271393	0.109	0.073	0.004727	Skin_Perio	CAMSAP2
PSMB53	3.02E-07	0.337787	0.275	0.241	0.004811	Skin_Perio	PSMB5
HIVEP2	3.03E-07	0.271588	0.131	0.092	0.004838	Skin_Perio	HIVEP2
C1orf211	3.95E-07	0.323094	0.14	0.101	0.0063	Skin_Perio	C1orf21
FOXO31	6.23E-07	0.363764	0.212	0.173	0.009931	Skin_Perio	FOXO3
MAP1LC3A	7.76E-07	0.313361	0.251	0.211	0.012374	Skin_Perio	MAP1LC3A
PJA29	8.08E-07	0.288657	0.191	0.151	0.012894	Skin_Perio	PJA2
CHD22	8.09E-07	0.291163	0.25	0.207	0.0129	Skin_Perio	CHD2
DMD4	8.47E-07	0.255679	0.08	0.051	0.013511	Skin_Perio	DMD
SAR1A2	8.98E-07	0.290759	0.27	0.232	0.014326	Skin_Perio	SAR1A
RAP1A2	9.15E-07	0.324222	0.311	0.275	0.014587	Skin_Perio	RAP1A
HIBADH	9.42E-07	0.293941	0.111	0.077	0.015027	Skin_Perio	HIBADH
MTRF1L	9.89E-07	0.268595	0.092	0.06	0.015774	Skin_Perio	MTRF1L
HNMT3	1.18E-06	0.30619	0.145	0.109	0.01888	Skin_Perio	HNMT

BRD4	1.81E-06	0.326686	0.202	0.164	0.028869	Skin_Peric	BRD4
TCEA12	1.99E-06	0.254259	0.278	0.24	0.031729	Skin_Peric	TCEA1
PPIL43	2.32E-06	0.282694	0.136	0.1	0.037052	Skin_Peric	PPIL4
RBFOX22	2.39E-06	0.296067	0.143	0.107	0.038161	Skin_Peric	RBFOX2
UQCRC22	3.23E-06	0.258394	0.246	0.209	0.051506	Skin_Peric	UQCRC2
CORO1C1	3.23E-06	0.307457	0.154	0.118	0.051583	Skin_Peric	CORO1C
SNRNP701	4.13E-06	0.258925	0.221	0.183	0.065814	Skin_Peric	SNRNP70
ASH1L4	4.22E-06	0.297803	0.206	0.17	0.067343	Skin_Peric	ASH1L
GBAS5	4.67E-06	0.310352	0.159	0.125	0.074536	Skin_Peric	GBAS
TLE1	4.82E-06	0.258934	0.099	0.069	0.076949	Skin_Peric	TLE1
RBM27	5E-06	0.269001	0.109	0.077	0.079722	Skin_Peric	RBM27
GRPEL1	5.04E-06	0.277567	0.165	0.13	0.080373	Skin_Peric	GRPEL1
ASAP13	5.08E-06	0.297547	0.13	0.095	0.081074	Skin_Peric	ASAP1
ETV62	8.44E-06	0.336369	0.143	0.11	0.134586	Skin_Peric	ETV6
TARDBP	8.92E-06	0.274653	0.146	0.113	0.142262	Skin_Peric	TARDBP
HSPB112	9.98E-06	0.270789	0.169	0.134	0.159198	Skin_Peric	HSPB11
SDHD	1.03E-05	0.301184	0.247	0.215	0.16364	Skin_Peric	SDHD
TSEN341	1.12E-05	0.309045	0.163	0.131	0.17893	Skin_Peric	TSEN34
SMG13	1.13E-05	0.27151	0.155	0.121	0.179827	Skin_Peric	SMG1
AP3S1	1.39E-05	0.273201	0.316	0.292	0.222051	Skin_Peric	AP3S1
FBXO211	1.4E-05	0.260892	0.114	0.084	0.22266	Skin_Peric	FBXO21
BRD3	1.46E-05	0.297713	0.099	0.071	0.233216	Skin_Peric	BRD3
CKB1	1.77E-05	0.348111	0.283	0.245	0.282633	Skin_Peric	CKB
SH3RF12	2.42E-05	0.270216	0.08	0.054	0.385771	Skin_Peric	SH3RF1
STARD3NL	2.47E-05	0.260844	0.116	0.086	0.393639	Skin_Peric	STARD3NL
TRIP61	2.86E-05	0.300457	0.171	0.14	0.456275	Skin_Peric	TRIP6
MEIS23	3.52E-05	0.277502	0.133	0.103	0.561377	Skin_Peric	MEIS2
MAPKAPK2	3.62E-05	0.27615	0.152	0.121	0.576972	Skin_Peric	MAPKAPK2
ZFYVE214	3.84E-05	0.330452	0.178	0.149	0.611895	Skin_Peric	ZFYVE21
PPP2R2A	3.88E-05	0.280571	0.16	0.129	0.619093	Skin_Peric	PPP2R2A
TAF1D2	4.49E-05	0.251804	0.26	0.227	0.716859	Skin_Peric	TAF1D
TMEM701	5.19E-05	0.253978	0.144	0.113	0.828428	Skin_Peric	TMEM70
TTC192	6.66E-05	0.301437	0.151	0.122	1	Skin_Peric	TTC19
FAM133B5	8.58E-05	0.260664	0.228	0.199	1	Skin_Peric	FAM133B
PAFAH1B2	9.61E-05	0.312332	0.155	0.126	1	Skin_Peric	PAFAH1B2
PVRL21	0.000108	0.320734	0.209	0.182	1	Skin_Peric	PVRL2
AHCYL13	0.000109	0.326712	0.191	0.165	1	Skin_Peric	AHCYL1
ADNP	0.000117	0.253569	0.121	0.094	1	Skin_Peric	ADNP
FAM63B1	0.000177	0.299414	0.13	0.103	1	Skin_Peric	FAM63B
SERTAD3	0.000184	0.253799	0.152	0.124	1	Skin_Peric	SERTAD3
BIRC2	0.000213	0.33047	0.178	0.152	1	Skin_Peric	BIRC2
SLC9A3R2	0.000215	0.367467	0.194	0.167	1	Skin_Peric	SLC9A3R2
CCDC71L1	0.000261	0.284071	0.089	0.065	1	Skin_Peric	CCDC71L
RBBP71	0.000275	0.25141	0.183	0.156	1	Skin_Peric	RBBP7
INSR4	0.000288	0.283571	0.157	0.13	1	Skin_Peric	INSR
CTBP22	0.000362	0.27725	0.128	0.103	1	Skin_Peric	CTBP2
UBE2A1	0.000386	0.269828	0.235	0.213	1	Skin_Peric	UBE2A

GCSH4	0.000397	0.281039	0.151	0.126	1	Skin_Pericy	GCSH
SENP5	0.00041	0.252119	0.102	0.078	1	Skin_Pericy	SENP5
AMOTL2	0.000414	0.273209	0.114	0.089	1	Skin_Pericy	AMOTL2
PGRMC21	0.000515	0.264952	0.166	0.142	1	Skin_Pericy	PGRMC2
YPEL21	0.00053	0.268431	0.13	0.104	1	Skin_Pericy	YPEL2
CCDC474	0.000556	0.301905	0.163	0.14	1	Skin_Pericy	CCDC47
DYNC1LI23	0.00082	0.299272	0.183	0.163	1	Skin_Pericy	DYNC1LI2
TIMM101	0.000925	0.254418	0.179	0.155	1	Skin_Pericy	TIMM10
MYO1C5	0.001696	0.286217	0.167	0.146	1	Skin_Pericy	MYO1C
RCHY1	0.001892	0.257546	0.096	0.076	1	Skin_Pericy	RCHY1
PTGR12	0.002087	0.359797	0.124	0.104	1	Skin_Pericy	PTGR1
SYNCRIP4	0.003192	0.25845	0.202	0.186	1	Skin_Pericy	SYNCRIP
MDM46	0.005532	0.261255	0.156	0.138	1	Skin_Pericy	MDM4
COL1A11	0	4.458562	0.82	0.086	0	Skin_Fibro	COL1A1
COL1A21	0	4.277884	0.896	0.102	0	Skin_Fibro	COL1A2
DCN1	0	4.151348	0.988	0.14	0	Skin_Fibro	DCN
FBLN11	0	3.535722	0.81	0.09	0	Skin_Fibro	FBLN1
LUM1	0	3.474851	0.813	0.067	0	Skin_Fibro	LUM
TNFAIP61	0	3.408463	0.598	0.046	0	Skin_Fibro	TNFAIP6
COL6A24	0	3.376541	0.919	0.104	0	Skin_Fibro	COL6A2
MMP21	0	3.33546	0.781	0.074	0	Skin_Fibro	MMP2
CXCL22	0	3.261174	0.668	0.126	0	Skin_Fibro	CXCL2
SOD22	0	3.140248	0.87	0.376	0	Skin_Fibro	SOD2
CEBPB2	0	3.129065	0.872	0.229	0	Skin_Fibro	CEBPB
CFD1	0	3.110472	0.96	0.292	0	Skin_Fibro	CFD
CTSK1	0	2.957686	0.743	0.051	0	Skin_Fibro	CTSK
GEM2	0	2.952002	0.627	0.046	0	Skin_Fibro	GEM
SPARC5	0	2.922148	0.714	0.121	0	Skin_Fibro	SPARC
CXCL124	0	2.759885	0.668	0.068	0	Skin_Fibro	CXCL12
SERPINF12	0	2.756805	0.775	0.092	0	Skin_Fibro	SERPINF1
NNMT2	0	2.710455	0.727	0.065	0	Skin_Fibro	NNMT
COL6A14	0	2.693725	0.725	0.098	0	Skin_Fibro	COL6A1
CXCL11	0	2.670011	0.461	0.118	0	Skin_Fibro	CXCL1
GSN2	0	2.665724	0.94	0.335	0	Skin_Fibro	GSN
CCL24	0	2.645314	0.619	0.088	0	Skin_Fibro	CCL2
WTAP2	0	2.62853	0.696	0.251	0	Skin_Fibro	WTAP
CCDC801	0	2.609092	0.777	0.104	0	Skin_Fibro	CCDC80
ID34	0	2.608533	0.713	0.132	0	Skin_Fibro	ID3
IGFBP54	0	2.590206	0.735	0.092	0	Skin_Fibro	IGFBP5
CEBPD1	0	2.557131	0.853	0.299	0	Skin_Fibro	CEBPD
MFAP42	0	2.532517	0.707	0.054	0	Skin_Fibro	MFAP4
C1S1	0	2.491893	0.754	0.072	0	Skin_Fibro	C1S
LGALS18	0	2.463949	0.99	0.368	0	Skin_Fibro	LGALS1
APOD1	0	2.459966	0.73	0.097	0	Skin_Fibro	APOD
C11orf962	0	2.393563	0.494	0.075	0	Skin_Fibro	C11orf96
VCAN1	0	2.366066	0.659	0.051	0	Skin_Fibro	VCAN
AXL5	0	2.361974	0.609	0.06	0	Skin_Fibro	AXL

FSTL12	0	2.353817	0.682	0.115	0	Skin_Fibro	FSTL1
THBS13	0	2.349106	0.461	0.052	0	Skin_Fibro	THBS1
CXCL32	0	2.26737	0.471	0.109	0	Skin_Fibro	CXCL3
COL6A32	0	2.252788	0.608	0.054	0	Skin_Fibro	COL6A3
TPPP34	0	2.23433	0.562	0.076	0	Skin_Fibro	TPPP3
HSPA1A7	0	2.230982	0.787	0.292	0	Skin_Fibro	HSPA1A
CYBRD12	0	2.223467	0.609	0.078	0	Skin_Fibro	CYBRD1
MEG32	0	2.216078	0.583	0.056	0	Skin_Fibro	MEG3
SEPP13	0	2.200437	0.818	0.225	0	Skin_Fibro	SEPP1
LEPR1	0	2.19773	0.469	0.038	0	Skin_Fibro	LEPR
WISP21	0	2.185992	0.391	0.033	0	Skin_Fibro	WISP2
CTHRC1	0	2.183476	0.366	0.04	0	Skin_Fibro	CTHRC1
CYR614	0	2.150159	0.492	0.085	0	Skin_Fibro	CYR61
FBLN22	0	2.078463	0.574	0.056	0	Skin_Fibro	FBLN2
C1R3	0	2.077011	0.639	0.072	0	Skin_Fibro	C1R
SERPING15	0	2.037048	0.714	0.133	0	Skin_Fibro	SERPING1
PCOLCE4	0	2.029828	0.551	0.057	0	Skin_Fibro	PCOLCE
NBL13	0	2.019825	0.549	0.078	0	Skin_Fibro	NBL1
ARID5B5	0	2.013065	0.684	0.238	0	Skin_Fibro	ARID5B
HTRA14	0	1.99888	0.536	0.08	0	Skin_Fibro	HTRA1
LAPTM4A3	0	1.994255	0.925	0.489	0	Skin_Fibro	LAPTM4A
ZFP36L25	0	1.977357	0.852	0.358	0	Skin_Fibro	ZFP36L2
S100A65	0	1.969738	0.998	0.692	0	Skin_Fibro	S100A6
CRABP22	0	1.963148	0.5	0.052	0	Skin_Fibro	CRABP2
PMP225	0	1.95309	0.629	0.102	0	Skin_Fibro	PMP22
FN12	0	1.951429	0.548	0.065	0	Skin_Fibro	FN1
MFAP51	0	1.931034	0.386	0.027	0	Skin_Fibro	MFAP5
ZFP36L14	0	1.929654	0.898	0.437	0	Skin_Fibro	ZFP36L1
RGS163	0	1.925769	0.425	0.063	0	Skin_Fibro	RGS16
TIMP21	0	1.905209	0.751	0.321	0	Skin_Fibro	TIMP2
NFKBIA3	0	1.890674	0.926	0.486	0	Skin_Fibro	NFKBIA
SDC23	0	1.877435	0.536	0.078	0	Skin_Fibro	SDC2
NBEAL13	0	1.864968	0.831	0.282	0	Skin_Fibro	NBEAL1
HSPA1B3	0	1.862047	0.681	0.283	0	Skin_Fibro	HSPA1B
IGFBP62	0	1.860276	0.539	0.109	0	Skin_Fibro	IGFBP6
NR4A12	0	1.838853	0.641	0.229	0	Skin_Fibro	NR4A1
AEBP13	0	1.8345	0.49	0.049	0	Skin_Fibro	AEBP1
TWIST13	0	1.792655	0.411	0.02	0	Skin_Fibro	TWIST1
BGN4	0	1.788907	0.401	0.043	0	Skin_Fibro	BGN
CD445	0	1.746788	0.819	0.314	0	Skin_Fibro	CD44
PI161	0	1.733096	0.328	0.03	0	Skin_Fibro	PI16
ZFP363	0	1.730354	0.873	0.518	0	Skin_Fibro	ZFP36
PTRF5	0	1.728316	0.623	0.13	0	Skin_Fibro	PTRF
PAM6	0	1.725033	0.487	0.116	0	Skin_Fibro	PAM
RPL413	0	1.706627	1	0.851	0	Skin_Fibro	RPL41
NR4A25	0	1.698959	0.51	0.161	0	Skin_Fibro	NR4A2
APCDD11	0	1.668086	0.288	0.021	0	Skin_Fibro	APCDD1

PNRC13	0	1.667885	0.839	0.487	0	Skin_Fibro	PNRC1
TUBB5	0	1.667846	0.729	0.28	0	Skin_Fibro	TUBB
IGFBP44	0	1.648777	0.564	0.098	0	Skin_Fibro	IGFBP4
TNFAIP23	0	1.637165	0.459	0.117	0	Skin_Fibro	TNFAIP2
TCF44	0	1.625846	0.54	0.147	0	Skin_Fibro	TCF4
SELM10	0	1.624529	0.806	0.313	0	Skin_Fibro	SELM
KLF43	0	1.616902	0.558	0.202	0	Skin_Fibro	KLF4
HES11	0	1.603761	0.501	0.2	0	Skin_Fibro	HES1
PDGFRA1	0	1.596929	0.401	0.03	0	Skin_Fibro	PDGFRA
GPNMB2	0	1.596284	0.577	0.093	0	Skin_Fibro	GPNMB
CFH1	0	1.593171	0.598	0.089	0	Skin_Fibro	CFH
LMNA10	0	1.578538	0.825	0.332	0	Skin_Fibro	LMNA
CST34	0	1.576528	0.954	0.567	0	Skin_Fibro	CST3
APOE2	0	1.574999	0.55	0.138	0	Skin_Fibro	APOE
IGFBP74	0	1.566308	0.822	0.224	0	Skin_Fibro	IGFBP7
TNXB2	0	1.566123	0.43	0.047	0	Skin_Fibro	TNXB
BICC11	0	1.558764	0.349	0.018	0	Skin_Fibro	BICC1
MYC1	0	1.555101	0.464	0.105	0	Skin_Fibro	MYC
VIM10	0	1.553842	0.988	0.458	0	Skin_Fibro	VIM
MAP1B4	0	1.552346	0.417	0.065	0	Skin_Fibro	MAP1B
COL5A21	0	1.551984	0.328	0.021	0	Skin_Fibro	COL5A2
ICAM13	0	1.551549	0.478	0.11	0	Skin_Fibro	ICAM1
FBN11	0	1.53957	0.429	0.047	0	Skin_Fibro	FBN1
MTRNR2L8	0	1.539233	0.759	0.251	0	Skin_Fibro	MTRNR2L8
IRF12	0	1.536296	0.531	0.192	0	Skin_Fibro	IRF1
FXYD13	0	1.535275	0.455	0.058	0	Skin_Fibro	FXYD1
OLFML31	0	1.534672	0.38	0.022	0	Skin_Fibro	OLFML3
FOS8	0	1.528633	0.765	0.4	0	Skin_Fibro	FOS
CYP1B1	0	1.527886	0.284	0.034	0	Skin_Fibro	CYP1B1
DUSP11	0	1.525537	0.835	0.542	0	Skin_Fibro	DUSP1
LOX	0	1.517108	0.32	0.017	0	Skin_Fibro	LOX
CALD14	0	1.50992	0.689	0.158	0	Skin_Fibro	CALD1
SOCS35	0	1.50197	0.461	0.143	0	Skin_Fibro	SOCS3
IFITM34	0	1.49411	0.826	0.273	0	Skin_Fibro	IFITM3
S100A134	0	1.489219	0.634	0.229	0	Skin_Fibro	S100A13
CD992	0	1.486528	0.825	0.468	0	Skin_Fibro	CD99
SLIT34	0	1.48086	0.396	0.058	0	Skin_Fibro	SLIT3
CD98	0	1.463857	0.787	0.428	0	Skin_Fibro	CD9
HNRNPH14	0	1.452162	0.578	0.277	0	Skin_Fibro	HNRNPH1
FGFR13	0	1.447557	0.419	0.063	0	Skin_Fibro	FGFR1
MYADM8	0	1.446818	0.468	0.127	0	Skin_Fibro	MYADM
ANXA22	0	1.446228	0.938	0.573	0	Skin_Fibro	ANXA2
CNN32	0	1.436036	0.528	0.202	0	Skin_Fibro	CNN3
CD8110	0	1.43381	0.652	0.299	0	Skin_Fibro	CD81
CYB5R34	0	1.433391	0.527	0.199	0	Skin_Fibro	CYB5R3
CAV15	0	1.430644	0.656	0.165	0	Skin_Fibro	CAV1
PRKCDBP4	0	1.417699	0.471	0.104	0	Skin_Fibro	PRKCDBP

MAN1A14	0	1.412272	0.377	0.065	0	Skin_Fibro	MAN1A1
EFEMP12	0	1.406155	0.337	0.051	0	Skin_Fibro	EFEMP1
THY13	0	1.402774	0.337	0.03	0	Skin_Fibro	THY1
POLR2L2	0	1.394754	0.782	0.451	0	Skin_Fibro	POLR2L
SAR1A3	0	1.390744	0.445	0.217	0	Skin_Fibro	SAR1A
SERPINH13	0	1.382866	0.401	0.094	0	Skin_Fibro	SERPINH1
NFIA6	0	1.382134	0.464	0.152	0	Skin_Fibro	NFIA
TNFAIP33	0	1.380543	0.41	0.143	0	Skin_Fibro	TNFAIP3
FHL14	0	1.380154	0.468	0.104	0	Skin_Fibro	FHL1
TMED27	0	1.37839	0.653	0.323	0	Skin_Fibro	TMED2
GADD45B2	0	1.36843	0.692	0.353	0	Skin_Fibro	GADD45B
LDHA4	0	1.362251	0.776	0.415	0	Skin_Fibro	LDHA
ARRDC32	0	1.343762	0.475	0.227	0	Skin_Fibro	ARRDC3
TSPAN82	0	1.339538	0.27	0.051	0	Skin_Fibro	TSPAN8
MGP3	0	1.337118	0.77	0.171	0	Skin_Fibro	MGP
PTN2	0	1.337012	0.295	0.053	0	Skin_Fibro	PTN
C32	0	1.334971	0.344	0.065	0	Skin_Fibro	C3
MT1M1	0	1.330811	0.379	0.05	0	Skin_Fibro	MT1M
COX7A17	0	1.330603	0.417	0.119	0	Skin_Fibro	COX7A1
LHFP5	0	1.323982	0.404	0.063	0	Skin_Fibro	LHFP
RARRES24	0	1.316671	0.348	0.069	0	Skin_Fibro	RARRES2
RTN44	0	1.296716	0.723	0.37	0	Skin_Fibro	RTN4
RPS4Y16	0	1.294037	0.668	0.244	0	Skin_Fibro	RPS4Y1
ELN2	0	1.293604	0.282	0.04	0	Skin_Fibro	ELN
MT2A1	0	1.28362	0.809	0.27	0	Skin_Fibro	MT2A
RPL3912	0	1.279366	0.994	0.654	0	Skin_Fibro	RPL39
DPYSL26	0	1.277392	0.378	0.079	0	Skin_Fibro	DPYSL2
FCGRT4	0	1.271012	0.587	0.255	0	Skin_Fibro	FCGRT
SLC39A141	0	1.268672	0.285	0.035	0	Skin_Fibro	SLC39A14
CALM26	0	1.258088	0.913	0.537	0	Skin_Fibro	CALM2
TPM15	0	1.25177	0.489	0.165	0	Skin_Fibro	TPM1
PDGFRB3	0	1.251199	0.366	0.045	0	Skin_Fibro	PDGFRB
TIMP17	0	1.249676	0.801	0.351	0	Skin_Fibro	TIMP1
QPCT2	0	1.245868	0.305	0.049	0	Skin_Fibro	QPCT
MFGE84	0	1.241618	0.384	0.078	0	Skin_Fibro	MFGE8
TSPAN44	0	1.239052	0.382	0.098	0	Skin_Fibro	TSPAN4
EID15	0	1.23815	0.675	0.334	0	Skin_Fibro	EID1
CLEC11A1	0	1.236786	0.333	0.061	0	Skin_Fibro	CLEC11A
MORF4L13	0	1.23566	0.753	0.437	0	Skin_Fibro	MORF4L1
CTSL6	0	1.215705	0.334	0.087	0	Skin_Fibro	CTSL
H2AFZ3	0	1.2132	0.701	0.42	0	Skin_Fibro	H2AFZ
FST3	0	1.20694	0.21	0.012	0	Skin_Fibro	FST
PTPRS2	0	1.205692	0.253	0.048	0	Skin_Fibro	PTPRS
DDAH25	0	1.196313	0.475	0.191	0	Skin_Fibro	DDAH2
LRP13	0	1.195819	0.382	0.061	0	Skin_Fibro	LRP1
KLF102	0	1.195033	0.367	0.125	0	Skin_Fibro	KLF10
PRNP6	0	1.193373	0.44	0.139	0	Skin_Fibro	PRNP

NUPR18	0	1.191672	0.599	0.235	0	Skin_Fibro	NUPR1
CD474	0	1.191155	0.474	0.212	0	Skin_Fibro	CD47
CLU3	0	1.190563	0.457	0.181	0	Skin_Fibro	CLU
NUCKS16	0	1.188864	0.556	0.264	0	Skin_Fibro	NUCKS1
AQP13	0	1.186094	0.407	0.073	0	Skin_Fibro	AQP1
OSR21	0	1.185028	0.281	0.028	0	Skin_Fibro	OSR2
CTGF4	0	1.18441	0.389	0.092	0	Skin_Fibro	CTGF
EIF4A13	0	1.184064	0.739	0.325	0	Skin_Fibro	EIF4A1
ZEB25	0	1.18041	0.347	0.07	0	Skin_Fibro	ZEB2
MXRA81	0	1.173667	0.327	0.038	0	Skin_Fibro	MXRA8
FKBP92	0	1.167487	0.309	0.061	0	Skin_Fibro	FKBP9
IER31	0	1.165877	0.736	0.363	0	Skin_Fibro	IER3
MEDAG1	0	1.161936	0.231	0.017	0	Skin_Fibro	MEDAG
DNAJB15	0	1.15902	0.675	0.412	0	Skin_Fibro	DNAJB1
ANXA59	0	1.158921	0.643	0.294	0	Skin_Fibro	ANXA5
EGR16	0	1.157459	0.419	0.155	0	Skin_Fibro	EGR1
S100A47	0	1.153089	0.922	0.431	0	Skin_Fibro	S100A4
FGF74	0	1.14993	0.269	0.03	0	Skin_Fibro	FGF7
TGFBR34	0	1.148508	0.301	0.076	0	Skin_Fibro	TGFBR3
TIMP35	0	1.147725	0.549	0.138	0	Skin_Fibro	TIMP3
AMD13	0	1.141337	0.404	0.179	0	Skin_Fibro	AMD1
WBP51	0	1.139589	0.406	0.164	0	Skin_Fibro	WBP5
CALU4	0	1.139396	0.398	0.159	0	Skin_Fibro	CALU
EMP36	0	1.13931	0.751	0.322	0	Skin_Fibro	EMP3
CD551	0	1.134498	0.455	0.253	0	Skin_Fibro	CD55
MMP23B	0	1.134162	0.258	0.019	0	Skin_Fibro	MMP23B
ZNF903	0	1.134083	0.32	0.079	0	Skin_Fibro	ZNF90
UAP15	0	1.1337	0.368	0.17	0	Skin_Fibro	UAP1
CHIC2	0	1.132565	0.387	0.173	0	Skin_Fibro	CHIC2
UGDH2	0	1.130493	0.28	0.058	0	Skin_Fibro	UGDH
TUBA1B7	0	1.123418	0.646	0.333	0	Skin_Fibro	TUBA1B
SERPINE21	0	1.118503	0.244	0.046	0	Skin_Fibro	SERPINE2
ABL21	0	1.099544	0.284	0.076	0	Skin_Fibro	ABL2
ABI3BP1	0	1.098788	0.295	0.036	0	Skin_Fibro	ABI3BP
ANTXR14	0	1.095209	0.258	0.034	0	Skin_Fibro	ANTXR1
PSAP3	0	1.093452	0.773	0.41	0	Skin_Fibro	PSAP
MMP14	0	1.09294	0.252	0.041	0	Skin_Fibro	MMP14
CES1	0	1.089235	0.251	0.04	0	Skin_Fibro	CES1
SPSB1	0	1.08709	0.245	0.045	0	Skin_Fibro	SPSB1
PRELP3	0	1.086971	0.259	0.024	0	Skin_Fibro	PRELP
ECM23	0	1.086055	0.251	0.018	0	Skin_Fibro	ECM2
NFKB12	0	1.08262	0.299	0.106	0	Skin_Fibro	NFKB1
MYLIP2	0	1.080801	0.381	0.148	0	Skin_Fibro	MYLIP
FBLN52	0	1.080291	0.31	0.05	0	Skin_Fibro	FBLN5
MORF4L22	0	1.076716	0.523	0.285	0	Skin_Fibro	MORF4L2
APP5	0	1.075581	0.481	0.226	0	Skin_Fibro	APP
AP3S11	0	1.075154	0.483	0.278	0	Skin_Fibro	AP3S1

ADIRF4	0	1.071656	0.892	0.402	0	Skin_Fibro	ADIRF
CCNI7	0	1.071138	0.775	0.499	0	Skin_Fibro	CCNI
LTBP42	0	1.070522	0.423	0.108	0	Skin_Fibro	LTBP4
JUNB2	0	1.067138	0.919	0.673	0	Skin_Fibro	JUNB
RSL24D13	0	1.060625	0.557	0.288	0	Skin_Fibro	RSL24D1
EFEMP22	0	1.056296	0.275	0.052	0	Skin_Fibro	EFEMP2
PDGFRL1	0	1.055808	0.269	0.035	0	Skin_Fibro	PDGFRL
RUNX13	0	1.055689	0.27	0.091	0	Skin_Fibro	RUNX1
RIN21	0	1.055669	0.216	0.063	0	Skin_Fibro	RIN2
BASP13	0	1.055492	0.287	0.069	0	Skin_Fibro	BASP1
CD6313	0	1.054502	0.929	0.602	0	Skin_Fibro	CD63
DAB23	0	1.052659	0.32	0.078	0	Skin_Fibro	DAB2
HNRNPA03	0	1.050751	0.616	0.314	0	Skin_Fibro	HNRNPA0
RCN31	0	1.050313	0.251	0.022	0	Skin_Fibro	RCN3
CRISPLD23	0	1.044194	0.281	0.041	0	Skin_Fibro	CRISPLD2
LPAR11	0	1.043636	0.246	0.024	0	Skin_Fibro	LPAR1
HSP90AB1	0	1.0389	0.827	0.551	0	Skin_Fibro	HSP90AB1
GOLIM43	0	1.032799	0.266	0.07	0	Skin_Fibro	GOLIM4
IL6ST5	0	1.030924	0.418	0.208	0	Skin_Fibro	IL6ST
CLIC44	0	1.023445	0.32	0.111	0	Skin_Fibro	CLIC4
SDCBP3	0	1.022082	0.674	0.393	0	Skin_Fibro	SDCBP
IL11RA1	0	1.017092	0.257	0.038	0	Skin_Fibro	IL11RA
S100A106	0	1.016193	0.954	0.655	0	Skin_Fibro	S100A10
RBPJ6	0	1.012529	0.393	0.177	0	Skin_Fibro	RBPJ
SNX32	0	1.00992	0.655	0.399	0	Skin_Fibro	SNX3
TNFRSF12A	0	1.008888	0.336	0.112	0	Skin_Fibro	TNFRSF12A
REV3L2	0	1.006825	0.279	0.078	0	Skin_Fibro	REV3L
REXO2	0	1.00682	0.426	0.247	0	Skin_Fibro	REXO2
YWHAG2	0	1.005543	0.411	0.217	0	Skin_Fibro	YWHAG
SLC38A24	0	1.004209	0.494	0.223	0	Skin_Fibro	SLC38A2
SPRY13	0	1.003974	0.292	0.077	0	Skin_Fibro	SPRY1
SLC2A34	0	1.001839	0.388	0.111	0	Skin_Fibro	SLC2A3
ADD32	0	0.997561	0.364	0.146	0	Skin_Fibro	ADD3
AKR1C12	0	0.992981	0.195	0.034	0	Skin_Fibro	AKR1C1
LIMA14	0	0.990631	0.386	0.167	0	Skin_Fibro	LIMA1
WSB17	0	0.99037	0.47	0.234	0	Skin_Fibro	WSB1
PTGS22	0	0.989094	0.187	0.029	0	Skin_Fibro	PTGS2
RP11.138A	0	0.985482	0.202	0.035	0	Skin_Fibro	RP11.138A9.2
RP11.553L	0	0.984361	0.233	0.039	0	Skin_Fibro	RP11.553L6.5
COL5A11	0	0.971183	0.219	0.018	0	Skin_Fibro	COL5A1
CIRBP5	0	0.968342	0.811	0.526	0	Skin_Fibro	CIRBP
LTBP13	0	0.967126	0.236	0.039	0	Skin_Fibro	LTBP1
PTMA4	0	0.964083	0.997	0.806	0	Skin_Fibro	PTMA
CPE5	0	0.960201	0.302	0.088	0	Skin_Fibro	CPE
NOVA11	0	0.960185	0.249	0.041	0	Skin_Fibro	NOVA1
PCNP3	0	0.953832	0.454	0.246	0	Skin_Fibro	PCNP
GAS12	0	0.951672	0.214	0.023	0	Skin_Fibro	GAS1

COPZ22	0	0.948953	0.243	0.071	0	Skin_Fibro	COPZ2
SEC61G5	0	0.947972	0.642	0.398	0	Skin_Fibro	SEC61G
IFI27L24	0	0.947583	0.366	0.124	0	Skin_Fibro	IFI27L2
NOTCH22	0	0.946234	0.26	0.082	0	Skin_Fibro	NOTCH2
SRPX1	0	0.944946	0.214	0.018	0	Skin_Fibro	SRPX
PTGES	0	0.944496	0.264	0.076	0	Skin_Fibro	PTGES
CLEC3B1	0	0.943045	0.177	0.011	0	Skin_Fibro	CLEC3B
C1orf1981	0	0.937117	0.217	0.047	0	Skin_Fibro	C1orf198
COX6C6	0	0.934869	0.739	0.527	0	Skin_Fibro	COX6C
GAS68	0	0.930695	0.274	0.082	0	Skin_Fibro	GAS6
SCARA51	0	0.929514	0.247	0.027	0	Skin_Fibro	SCARA5
KDM6B3	0	0.92928	0.284	0.106	0	Skin_Fibro	KDM6B
LAMB26	0	0.928286	0.263	0.07	0	Skin_Fibro	LAMB2
STOM4	0	0.924609	0.366	0.137	0	Skin_Fibro	STOM
TGIF13	0	0.924044	0.363	0.184	0	Skin_Fibro	TGIF1
TWSG11	0	0.924026	0.24	0.057	0	Skin_Fibro	TWSG1
PTMS8	0	0.923657	0.489	0.227	0	Skin_Fibro	PTMS
NFKBIZ2	0	0.923368	0.42	0.233	0	Skin_Fibro	NFKBIZ
SGCA2	0	0.922481	0.216	0.037	0	Skin_Fibro	SGCA
KLF94	0	0.91809	0.272	0.094	0	Skin_Fibro	KLF9
HCFC1R14	0	0.917133	0.336	0.121	0	Skin_Fibro	HCFC1R1
PPP3CA5	0	0.917051	0.312	0.144	0	Skin_Fibro	PPP3CA
RBM34	0	0.914479	0.667	0.423	0	Skin_Fibro	RBM3
NENF7	0	0.913189	0.539	0.313	0	Skin_Fibro	NENF
LAMC13	0	0.912833	0.254	0.064	0	Skin_Fibro	LAMC1
RPS274	0	0.911051	1	0.887	0	Skin_Fibro	RPS27
COMP	0	0.910404	0.093	0.014	0	Skin_Fibro	COMP
STAT36	0	0.908991	0.377	0.177	0	Skin_Fibro	STAT3
COL18A14	0	0.908325	0.219	0.063	0	Skin_Fibro	COL18A1
YWHAE2	0	0.90736	0.573	0.385	0	Skin_Fibro	YWHAE
PTGDS	0	0.904891	0.441	0.088	0	Skin_Fibro	PTGDS
TCF7L21	0	0.90258	0.248	0.076	0	Skin_Fibro	TCF7L2
SPTBN13	0	0.901784	0.402	0.183	0	Skin_Fibro	SPTBN1
NUDT43	0	0.901164	0.307	0.142	0	Skin_Fibro	NUDT4
TUBB2A4	0	0.900602	0.292	0.1	0	Skin_Fibro	TUBB2A
SEPT115	0	0.898496	0.271	0.072	0	Skin_Fibro	SEPT11
VASN1	0	0.897034	0.3	0.116	0	Skin_Fibro	VASN
PROS13	0	0.895169	0.22	0.031	0	Skin_Fibro	PROS1
FGL23	0	0.893349	0.332	0.081	0	Skin_Fibro	FGL2
NFIL32	0	0.892399	0.216	0.047	0	Skin_Fibro	NFIL3
RPL344	0	0.891805	0.999	0.913	0	Skin_Fibro	RPL34
PGRMC15	0	0.891112	0.33	0.155	0	Skin_Fibro	PGRMC1
TAX1BP31	0	0.887862	0.271	0.073	0	Skin_Fibro	TAX1BP3
SET6	0	0.886833	0.489	0.298	0	Skin_Fibro	SET
ARL6IP56	0	0.882825	0.541	0.293	0	Skin_Fibro	ARL6IP5
LY6E1	0	0.879219	0.609	0.311	0	Skin_Fibro	LY6E
AHNAK8	0	0.877514	0.66	0.366	0	Skin_Fibro	AHNAK

ANKRD102	0	0.876537	0.279	0.101	0	Skin_Fibro	ANKRD10
SNX72	0	0.874404	0.231	0.054	0	Skin_Fibro	SNX7
MAP3K83	0	0.874216	0.287	0.104	0	Skin_Fibro	MAP3K8
ID23	0	0.869963	0.466	0.239	0	Skin_Fibro	ID2
PDLIM22	0	0.864002	0.366	0.165	0	Skin_Fibro	PDLIM2
MAMDC22	0	0.862881	0.175	0.03	0	Skin_Fibro	MAMDC2
MARCKS5	0	0.862148	0.47	0.27	0	Skin_Fibro	MARCKS
GLUD12	0	0.857679	0.329	0.151	0	Skin_Fibro	GLUD1
EBF14	0	0.857524	0.24	0.049	0	Skin_Fibro	EBF1
VKORC16	0	0.856562	0.392	0.212	0	Skin_Fibro	VKORC1
FKBP102	0	0.856096	0.207	0.022	0	Skin_Fibro	FKBP10
CKS1B3	0	0.853083	0.307	0.127	0	Skin_Fibro	CKS1B
ID12	0	0.851007	0.288	0.096	0	Skin_Fibro	ID1
REL4	0	0.850897	0.388	0.172	0	Skin_Fibro	REL
SAV12	0	0.85003	0.251	0.096	0	Skin_Fibro	SAV1
TFPI3	0	0.848909	0.258	0.067	0	Skin_Fibro	TFPI
GADD45A1	0	0.848379	0.407	0.2	0	Skin_Fibro	GADD45A
SLIT22	0	0.843448	0.183	0.012	0	Skin_Fibro	SLIT2
PLEKHH21	0	0.843354	0.163	0.029	0	Skin_Fibro	PLEKHH2
CILP1	0	0.841456	0.165	0.018	0	Skin_Fibro	CILP
PLD34	0	0.840116	0.327	0.153	0	Skin_Fibro	PLD3
MIR22HG2	0	0.839085	0.236	0.074	0	Skin_Fibro	MIR22HG
CERCAM2	0	0.839019	0.2	0.047	0	Skin_Fibro	CERCAM
DDR23	0	0.838625	0.222	0.031	0	Skin_Fibro	DDR2
ERF1	0	0.837131	0.213	0.063	0	Skin_Fibro	ERF
FILIP1L6	0	0.835876	0.232	0.074	0	Skin_Fibro	FILIP1L
PCOLCE21	0	0.835658	0.188	0.024	0	Skin_Fibro	PCOLCE2
SBDS2	0	0.835327	0.538	0.327	0	Skin_Fibro	SBDS
YWHAQ2	0	0.834162	0.538	0.352	0	Skin_Fibro	YWHAQ
PLAGL11	0	0.83298	0.252	0.083	0	Skin_Fibro	PLAGL1
CRTAP5	0	0.831562	0.283	0.103	0	Skin_Fibro	CRTAP
SLC44A12	0	0.831306	0.235	0.068	0	Skin_Fibro	SLC44A1
COL14A12	0	0.828474	0.211	0.029	0	Skin_Fibro	COL14A1
CELF25	0	0.824647	0.309	0.12	0	Skin_Fibro	CELF2
RASD14	0	0.823616	0.203	0.069	0	Skin_Fibro	RASD1
PXDC11	0	0.820891	0.211	0.075	0	Skin_Fibro	PXDC1
F13A12	0	0.817818	0.177	0.021	0	Skin_Fibro	F13A1
CPQ2	0	0.81692	0.242	0.063	0	Skin_Fibro	CPQ
SEP151	0	0.815905	0.471	0.294	0	Skin_Fibro	SEP15
EIF3E5	0	0.814905	0.688	0.432	0	Skin_Fibro	EIF3E
KLF65	0	0.813937	0.624	0.386	0	Skin_Fibro	KLF6
TPST11	0	0.812221	0.201	0.044	0	Skin_Fibro	TPST1
TMEM984	0	0.811274	0.226	0.065	0	Skin_Fibro	TMEM98
RP11.14N7	0	0.810851	0.201	0.027	0	Skin_Fibro	RP11.14N7.2
ITIH56	0	0.806933	0.197	0.027	0	Skin_Fibro	ITIH5
SRSF35	0	0.806183	0.607	0.421	0	Skin_Fibro	SRSF3
PLTP3	0	0.802771	0.234	0.085	0	Skin_Fibro	PLTP

NFIC8	0	0.801098	0.338	0.16	0	Skin_Fibro NFIC
TMEM14A	0	0.800984	0.236	0.074	0	Skin_Fibro TMEM14A
FERMT25	0	0.797623	0.232	0.071	0	Skin_Fibro FERMT2
EPHX14	0	0.797455	0.265	0.111	0	Skin_Fibro EPHX1
MAF2	0	0.7946	0.229	0.071	0	Skin_Fibro MAF
RAB345	0	0.792836	0.271	0.09	0	Skin_Fibro RAB34
CD2482	0	0.791566	0.204	0.024	0	Skin_Fibro CD248
AHR5	0	0.786976	0.264	0.097	0	Skin_Fibro AHR
RECK1	0	0.786276	0.184	0.035	0	Skin_Fibro RECK
SEC625	0	0.784716	0.583	0.389	0	Skin_Fibro SEC62
SEMA3C3	0	0.784448	0.156	0.027	0	Skin_Fibro SEMA3C
FKBP1A2	0	0.780908	0.523	0.324	0	Skin_Fibro FKBP1A
ANGPTL22	0	0.778096	0.19	0.02	0	Skin_Fibro ANGPTL2
TOB23	0	0.775166	0.237	0.091	0	Skin_Fibro TOB2
ITGB15	0	0.775051	0.539	0.344	0	Skin_Fibro ITGB1
PPP1R15A1	0	0.774931	0.706	0.431	0	Skin_Fibro PPP1R15A
U2AF13	0	0.773383	0.469	0.24	0	Skin_Fibro U2AF1
MBNL17	0	0.772302	0.344	0.158	0	Skin_Fibro MBNL1
NBPF101	0	0.771689	0.189	0.038	0	Skin_Fibro NBPF10
NFIX6	0	0.771257	0.245	0.1	0	Skin_Fibro NFIX
ANXA43	0	0.770334	0.284	0.127	0	Skin_Fibro ANXA4
CADM31	0	0.766783	0.153	0.013	0	Skin_Fibro CADM3
RBMS35	0	0.764842	0.193	0.042	0	Skin_Fibro RBMS3
MFAP21	0	0.764326	0.172	0.016	0	Skin_Fibro MFAP2
XG1	0	0.761713	0.175	0.02	0	Skin_Fibro XG
NEGR11	0	0.7613	0.175	0.017	0	Skin_Fibro NEGR1
PDK41	0	0.760741	0.145	0.033	0	Skin_Fibro PDK4
CDKN1A2	0	0.756795	0.493	0.243	0	Skin_Fibro CDKN1A
NDN4	0	0.755142	0.214	0.052	0	Skin_Fibro NDN
KCTD123	0	0.752475	0.26	0.08	0	Skin_Fibro KCTD12
ACKR33	0	0.750212	0.149	0.019	0	Skin_Fibro ACKR3
ELMSAN13	0	0.739192	0.205	0.069	0	Skin_Fibro ELMSAN1
RUNX1T14	0	0.731664	0.18	0.03	0	Skin_Fibro RUNX1T1
HTRA31	0	0.726396	0.158	0.017	0	Skin_Fibro HTRA3
COL16A12	0	0.726381	0.165	0.024	0	Skin_Fibro COL16A1
EMX22	0	0.725726	0.16	0.022	0	Skin_Fibro EMX2
GPC32	0	0.722432	0.116	0.022	0	Skin_Fibro GPC3
CYP26B12	0	0.71452	0.134	0.019	0	Skin_Fibro CYP26B1
RP11.138A	0	0.71442	0.158	0.039	0	Skin_Fibro RP11.138A9.1
CSF14	0	0.71138	0.16	0.024	0	Skin_Fibro CSF1
PLSCR42	0	0.709795	0.179	0.038	0	Skin_Fibro PLSCR4
KIAA09302	0	0.70979	0.204	0.06	0	Skin_Fibro KIAA0930
RHOC5	0	0.703998	0.401	0.211	0	Skin_Fibro RHOC
MATN4	0	0.701383	0.108	0.01	0	Skin_Fibro MATN4
CREB53	0	0.699787	0.175	0.048	0	Skin_Fibro CREB5
MATR33	0	0.696123	0.271	0.104	0	Skin_Fibro MATR3
COL15A12	0	0.69512	0.211	0.045	0	Skin_Fibro COL15A1

TMEM176I	0	0.688535	0.263	0.078	0	Skin_Fibro	TMEM176B
ARMCX12	0	0.686569	0.185	0.047	0	Skin_Fibro	ARMCX1
CRIP14	0	0.680455	0.479	0.263	0	Skin_Fibro	CRIP1
LOXL11	0	0.67843	0.154	0.023	0	Skin_Fibro	LOXL1
H3F3A15	0	0.675365	0.823	0.453	0	Skin_Fibro	H3F3A
MRC21	0	0.66697	0.175	0.035	0	Skin_Fibro	MRC2
MAP1A1	0	0.665208	0.161	0.027	0	Skin_Fibro	MAP1A
CORIN	0	0.663217	0.135	0.005	0	Skin_Fibro	CORIN
RGS32	0	0.662972	0.141	0.036	0	Skin_Fibro	RGS3
VCAM13	0	0.660069	0.135	0.025	0	Skin_Fibro	VCAM1
C16orf452	0	0.65908	0.188	0.055	0	Skin_Fibro	C16orf45
PID12	0	0.658862	0.172	0.027	0	Skin_Fibro	PID1
GSTM51	0	0.657496	0.155	0.019	0	Skin_Fibro	GSTM5
ZFAS13	0	0.656827	0.676	0.451	0	Skin_Fibro	ZFAS1
RPL122	0	0.656022	0.996	0.842	0	Skin_Fibro	RPL12
MITF	0	0.651604	0.157	0.034	0	Skin_Fibro	MITF
RPL73	0	0.6508	0.991	0.783	0	Skin_Fibro	RPL7
LAMC3	0	0.641624	0.136	0.011	0	Skin_Fibro	LAMC3
DIRAS3	0	0.640329	0.089	0.006	0	Skin_Fibro	DIRAS3
CD1092	0	0.636649	0.163	0.037	0	Skin_Fibro	CD109
NTRK24	0	0.636398	0.194	0.043	0	Skin_Fibro	NTRK2
VAMP52	0	0.634344	0.35	0.156	0	Skin_Fibro	VAMP5
FKBP72	0	0.634216	0.171	0.049	0	Skin_Fibro	FKBP7
PLEKHA42	0	0.630803	0.137	0.02	0	Skin_Fibro	PLEKHA4
ACVRL12	0	0.629232	0.156	0.018	0	Skin_Fibro	ACVRL1
LRRN4CL1	0	0.622589	0.145	0.015	0	Skin_Fibro	LRRN4CL
ANPEP2	0	0.621418	0.159	0.025	0	Skin_Fibro	ANPEP
HMGB18	0	0.621248	0.746	0.563	0	Skin_Fibro	HMGB1
C1QTNF24	0	0.615915	0.138	0.015	0	Skin_Fibro	C1QTNF2
NPC23	0	0.613009	0.621	0.427	0	Skin_Fibro	NPC2
FTL3	0	0.6099	0.999	0.888	0	Skin_Fibro	FTL
EEF23	0	0.607274	0.819	0.592	0	Skin_Fibro	EEF2
RPL233	0	0.606266	0.9	0.668	0	Skin_Fibro	RPL23
UBB2	0	0.602639	0.905	0.717	0	Skin_Fibro	UBB
PTPRG4	0	0.593055	0.139	0.031	0	Skin_Fibro	PTPRG
TRIM474	0	0.591127	0.168	0.051	0	Skin_Fibro	TRIM47
HSPB13	0	0.587684	0.891	0.541	0	Skin_Fibro	HSPB1
SSPN3	0	0.586065	0.203	0.058	0	Skin_Fibro	SSPN
EEF1A112	0	0.581357	0.996	0.909	0	Skin_Fibro	EEF1A1
CCBE1	0	0.578989	0.111	0.005	0	Skin_Fibro	CCBE1
PROCR3	0	0.573602	0.128	0.024	0	Skin_Fibro	PROCR
CLIC15	0	0.57319	0.681	0.46	0	Skin_Fibro	CLIC1
DPYSL34	0	0.571947	0.166	0.047	0	Skin_Fibro	DPYSL3
TMEM176/	0	0.564599	0.183	0.056	0	Skin_Fibro	TMEM176A
SNAI11	0	0.563885	0.108	0.014	0	Skin_Fibro	SNAI1
IL61	0	0.563678	0.188	0.033	0	Skin_Fibro	IL6
AKR1C21	0	0.563568	0.126	0.02	0	Skin_Fibro	AKR1C2

DYNLL14	0	0.560695	0.745	0.548	0	Skin_Fibro	DYNLL1
CYGB3	0	0.558236	0.118	0.018	0	Skin_Fibro	CYGB
ZCCHC243	0	0.558062	0.135	0.027	0	Skin_Fibro	ZCCHC24
NR4A33	0	0.557991	0.158	0.046	0	Skin_Fibro	NR4A3
NKD2	0	0.552613	0.096	0.006	0	Skin_Fibro	NKD2
PCDH181	0	0.551333	0.125	0.014	0	Skin_Fibro	PCDH18
GPX82	0	0.549214	0.143	0.032	0	Skin_Fibro	GPX8
OLFML11	0	0.547209	0.128	0.012	0	Skin_Fibro	OLFML1
MSX13	0	0.546381	0.146	0.029	0	Skin_Fibro	MSX1
DLC14	0	0.541999	0.153	0.042	0	Skin_Fibro	DLC1
NRN11	0	0.538331	0.159	0.028	0	Skin_Fibro	NRN1
LRRC323	0	0.537352	0.137	0.026	0	Skin_Fibro	LRRC32
NID12	0	0.532661	0.127	0.02	0	Skin_Fibro	NID1
GFPT21	0	0.531084	0.11	0.013	0	Skin_Fibro	GFPT2
TMSB4X4	0	0.526031	1	0.862	0	Skin_Fibro	TMSB4X
FYN5	0	0.523843	0.189	0.058	0	Skin_Fibro	FYN
RPS153	0	0.523686	0.999	0.872	0	Skin_Fibro	RPS15
RPL104	0	0.520493	0.999	0.869	0	Skin_Fibro	RPL10
ITGA54	0	0.52011	0.143	0.032	0	Skin_Fibro	ITGA5
RPL362	0	0.518341	0.989	0.83	0	Skin_Fibro	RPL36
GNG113	0	0.517338	0.305	0.1	0	Skin_Fibro	GNG11
C7orf55.LU	0	0.515384	0.154	0.038	0	Skin_Fibro	C7orf55.LUC7L2
ADAM331	0	0.514781	0.109	0.009	0	Skin_Fibro	ADAM33
IFITM12	0	0.508002	0.346	0.146	0	Skin_Fibro	IFITM1
CD70	0	0.506205	0.101	0.016	0	Skin_Fibro	CD70
FEZ11	0	0.495976	0.123	0.03	0	Skin_Fibro	FEZ1
CP3	0	0.493876	0.11	0.021	0	Skin_Fibro	CP
MRGPRF4	0	0.491644	0.125	0.019	0	Skin_Fibro	MRGPRF
PFDN53	0	0.490769	0.899	0.715	0	Skin_Fibro	PFDN5
BEST13	0	0.489036	0.142	0.035	0	Skin_Fibro	BEST1
RPS294	0	0.48858	0.965	0.778	0	Skin_Fibro	RPS29
YBX13	0	0.486037	0.827	0.571	0	Skin_Fibro	YBX1
RPL35A2	0	0.483942	0.99	0.854	0	Skin_Fibro	RPL35A
SAMD4A	0	0.483084	0.23	0.075	0	Skin_Fibro	SAMD4A
RPL213	0	0.477483	0.996	0.801	0	Skin_Fibro	RPL21
TGFB1I15	0	0.476706	0.156	0.045	0	Skin_Fibro	TGFB1I1
GPX41	0	0.4762	0.822	0.524	0	Skin_Fibro	GPX4
BMP42	0	0.474617	0.098	0.016	0	Skin_Fibro	BMP4
PLAUR3	0	0.468946	0.271	0.108	0	Skin_Fibro	PLAUR
IFITM25	0	0.467785	0.586	0.285	0	Skin_Fibro	IFITM2
RPS203	0	0.467226	0.965	0.783	0	Skin_Fibro	RPS20
RPS183	0	0.464356	1	0.91	0	Skin_Fibro	RPS18
NT5E1	0	0.459027	0.116	0.021	0	Skin_Fibro	NT5E
ASIP	0	0.456656	0.087	0.006	0	Skin_Fibro	ASIP
GLI31	0	0.455322	0.106	0.015	0	Skin_Fibro	GLI3
SEMA3D	0	0.455104	0.1	0.015	0	Skin_Fibro	SEMA3D
CPED14	0	0.454685	0.097	0.015	0	Skin_Fibro	CPED1

TMSB103	0	0.454323	0.995	0.87	0	Skin_Fibro	TMSB10
PAPPA	0	0.453772	0.078	0.011	0	Skin_Fibro	PAPPA
ENHO2	0	0.452512	0.09	0.009	0	Skin_Fibro	ENHO
CDON1	0	0.451341	0.099	0.012	0	Skin_Fibro	CDON
CHRDL11	0	0.449255	0.116	0.02	0	Skin_Fibro	CHRDL1
BTG14	0	0.448425	0.779	0.563	0	Skin_Fibro	BTG1
ADAMTS42	0	0.448419	0.12	0.026	0	Skin_Fibro	ADAMTS4
TMEM119	0	0.444462	0.093	0.007	0	Skin_Fibro	TMEM119
IRX13	0	0.437656	0.081	0.014	0	Skin_Fibro	IRX1
MCOLN31	0	0.436933	0.09	0.017	0	Skin_Fibro	MCOLN3
AXIN2	0	0.43502	0.089	0.015	0	Skin_Fibro	AXIN2
CNRIP15	0	0.430173	0.128	0.032	0	Skin_Fibro	CNRIP1
LRRC2	0	0.428799	0.087	0.005	0	Skin_Fibro	LRRC2
RPS285	0	0.428045	0.993	0.873	0	Skin_Fibro	RPS28
EMID1	0	0.427251	0.074	0.007	0	Skin_Fibro	EMID1
ADAMTS52	0	0.425756	0.095	0.015	0	Skin_Fibro	ADAMTS5
NTN1	0	0.418796	0.09	0.016	0	Skin_Fibro	NTN1
BOC2	0	0.412111	0.1	0.02	0	Skin_Fibro	BOC
EMILIN12	0	0.407667	0.099	0.015	0	Skin_Fibro	EMILIN1
MYL94	0	0.406279	0.334	0.103	0	Skin_Fibro	MYL9
UST	0	0.397606	0.08	0.01	0	Skin_Fibro	UST
ISM11	0	0.396214	0.08	0.005	0	Skin_Fibro	ISM1
RPL313	0	0.3956	0.981	0.753	0	Skin_Fibro	RPL31
FBLN7	0	0.380541	0.079	0.007	0	Skin_Fibro	FBLN7
IL15RA1	0	0.377704	0.079	0.013	0	Skin_Fibro	IL15RA
RPS83	0	0.377246	0.993	0.839	0	Skin_Fibro	RPS8
TSHZ31	0	0.376036	0.077	0.01	0	Skin_Fibro	TSHZ3
S100A3	0	0.371524	0.087	0.012	0	Skin_Fibro	S100A3
RPL13A2	0	0.35816	0.999	0.873	0	Skin_Fibro	RPL13A
NFATC43	0	0.341106	0.078	0.013	0	Skin_Fibro	NFATC4
DNM12	0	0.337998	0.081	0.013	0	Skin_Fibro	DNM1
ADAMTS21	0	0.334368	0.071	0.007	0	Skin_Fibro	ADAMTS2
COL13A1	0	0.305899	0.056	0.002	0	Skin_Fibro	COL13A1
CHRD2	0	0.26935	0.055	0.005	0	Skin_Fibro	CHRD
COL23A11	0	0.266235	0.046	0.004	0	Skin_Fibro	COL23A1
NEAT11	0	0.265918	0.962	0.662	0	Skin_Fibro	NEAT1
CPXM1	0	0.260008	0.054	0.006	0	Skin_Fibro	CPXM1
SPON21	5.1E-308	0.400194	0.178	0.054	8.2E-304	Skin_Fibro	SPON2
MGST312	5.1E-307	0.607231	0.538	0.32	8.2E-303	Skin_Fibro	MGST3
RPL10A3	1.2E-305	0.280221	0.982	0.717	2E-301	Skin_Fibro	RPL10A
G3BP12	3.5E-304	0.825565	0.312	0.15	5.7E-300	Skin_Fibro	G3BP1
PPIB6	4.5E-303	0.648691	0.58	0.381	7.1E-299	Skin_Fibro	PPIB
CXXC52	9.1E-302	0.855617	0.302	0.142	1.4E-297	Skin_Fibro	CXXC5
SNX211	3.7E-301	0.435043	0.114	0.027	5.9E-297	Skin_Fibro	SNX21
ROR2	1.3E-300	0.27921	0.059	0.007	2E-296	Skin_Fibro	ROR2
IGDCC41	1.5E-299	0.28131	0.056	0.007	2.5E-295	Skin_Fibro	IGDCC4
RPL23A2	9.2E-299	0.26666	0.985	0.79	1.5E-294	Skin_Fibro	RPL23A

NRP15	9.5E-299	0.5243	0.162	0.049	1.5E-294	Skin_Fibro	NRP1
C1QTNF31	2.1E-298	0.609596	0.131	0.035	3.3E-294	Skin_Fibro	C1QTNF3
RBM395	3.2E-297	0.75649	0.57	0.399	5.2E-293	Skin_Fibro	RBM39
RPL353	3.9E-297	0.33131	0.984	0.825	6.2E-293	Skin_Fibro	RPL35
HNRNPA12	1.2E-296	0.470297	0.756	0.53	1.9E-292	Skin_Fibro	HNRNPA1
ESD7	1.5E-296	0.823589	0.377	0.206	2.4E-292	Skin_Fibro	ESD
BIRC34	1.6E-296	0.924576	0.311	0.143	2.6E-292	Skin_Fibro	BIRC3
CTSF3	2.8E-295	0.865943	0.301	0.147	4.4E-291	Skin_Fibro	CTSF
CH25H2	7.4E-295	0.317164	0.061	0.008	1.2E-290	Skin_Fibro	CH25H
OLFM21	2.9E-294	0.513973	0.126	0.032	4.6E-290	Skin_Fibro	OLFM2
PEAK13	2.2E-293	0.517219	0.128	0.034	3.4E-289	Skin_Fibro	PEAK1
ARF42	5.3E-293	0.768844	0.569	0.407	8.5E-289	Skin_Fibro	ARF4
ADAMTS13	3.2E-290	0.51969	0.169	0.053	5.1E-286	Skin_Fibro	ADAMTS1
NOV2	9.2E-290	0.469881	0.098	0.021	1.5E-285	Skin_Fibro	NOV
MYL65	1.3E-289	0.383469	0.921	0.797	2.1E-285	Skin_Fibro	MYL6
SH3PXD2A	2.8E-288	0.701853	0.203	0.075	4.5E-284	Skin_Fibro	SH3PXD2A
SUMO13	1.1E-287	0.801271	0.501	0.336	1.7E-283	Skin_Fibro	SUMO1
IFI165	1.2E-287	0.786654	0.36	0.187	1.9E-283	Skin_Fibro	IFI16
CHD15	1.8E-286	0.824332	0.27	0.122	2.8E-282	Skin_Fibro	CHD1
SGCD1	2.4E-286	0.364077	0.084	0.016	3.9E-282	Skin_Fibro	SGCD
RPL263	2.5E-286	0.306254	0.989	0.824	4E-282	Skin_Fibro	RPL26
RPS52	2.6E-286	0.264399	0.974	0.772	4.2E-282	Skin_Fibro	RPS5
RGCC9	7.3E-286	0.691904	0.394	0.201	1.2E-281	Skin_Fibro	RGCC
PRSS12	3.6E-285	0.355948	0.068	0.011	5.7E-281	Skin_Fibro	PRSS12
SSC5D1	1.2E-282	0.306446	0.072	0.012	1.9E-278	Skin_Fibro	SSC5D
C4orf46	5.1E-282	0.510372	0.1	0.022	8.2E-278	Skin_Fibro	C4orf46
VAPA3	4.5E-280	0.763028	0.553	0.389	7.1E-276	Skin_Fibro	VAPA
SLITRK41	4.5E-280	0.379844	0.09	0.018	7.1E-276	Skin_Fibro	SLITRK4
CMPK14	7.1E-280	0.788735	0.457	0.289	1.1E-275	Skin_Fibro	CMPK1
SLC25A62	1.1E-278	0.405268	0.784	0.552	1.7E-274	Skin_Fibro	SLC25A6
SCARF21	2.9E-278	0.272572	0.059	0.008	4.7E-274	Skin_Fibro	SCARF2
GLT8D23	6.4E-278	0.357185	0.094	0.02	1E-273	Skin_Fibro	GLT8D2
PBX17	8.3E-277	0.797109	0.241	0.105	1.3E-272	Skin_Fibro	PBX1
SGCE4	1.6E-276	0.506586	0.124	0.033	2.6E-272	Skin_Fibro	SGCE
ENAH5	2.6E-276	0.891078	0.246	0.109	4.2E-272	Skin_Fibro	ENAH
C12orf577	1.8E-275	0.596299	0.534	0.345	2.8E-271	Skin_Fibro	C12orf57
PLXDC25	2.8E-275	0.499928	0.133	0.037	4.5E-271	Skin_Fibro	PLXDC2
RPL323	4.1E-275	0.283193	0.997	0.863	6.5E-271	Skin_Fibro	RPL32
FBXO171	2.1E-274	0.438726	0.111	0.028	3.4E-270	Skin_Fibro	FBXO17
RAI21	3.8E-273	0.373177	0.087	0.018	6.1E-269	Skin_Fibro	RAI2
ABCA13	6.8E-272	0.708544	0.171	0.059	1.1E-267	Skin_Fibro	ABCA1
SLC29A12	8.9E-272	0.519953	0.138	0.041	1.4E-267	Skin_Fibro	SLC29A1
TAGLN26	3.2E-271	0.491446	0.615	0.394	5.1E-267	Skin_Fibro	TAGLN2
TRAM13	6.7E-271	0.79958	0.448	0.289	1.1E-266	Skin_Fibro	TRAM1
ROBO11	7.9E-271	0.44542	0.102	0.024	1.3E-266	Skin_Fibro	ROBO1
CD2761	5.1E-270	0.556716	0.141	0.043	8.1E-266	Skin_Fibro	CD276
HSD3B71	1E-269	0.442747	0.114	0.029	1.6E-265	Skin_Fibro	HSD3B7

FAT41	3.1E-269	0.25081	0.052	0.006	4.9E-265	Skin_Fibro	FAT4
YBX34	8.9E-269	0.570261	0.67	0.483	1.4E-264	Skin_Fibro	YBX3
MGST17	4.5E-268	0.91215	0.339	0.184	7.2E-264	Skin_Fibro	MGST1
MAFB4	5.7E-268	0.742813	0.207	0.08	9.2E-264	Skin_Fibro	MAFB
LRRC172	9.8E-268	0.321071	0.072	0.012	1.6E-263	Skin_Fibro	LRRC17
ZNF5212	6.6E-267	0.385281	0.088	0.018	1.1E-262	Skin_Fibro	ZNF521
WWTR11	1.2E-266	0.780712	0.261	0.12	1.9E-262	Skin_Fibro	WWTR1
CLEC2B6	1.7E-265	0.63028	0.308	0.145	2.8E-261	Skin_Fibro	CLEC2B
TSC22D22	2.4E-265	0.767225	0.231	0.098	3.8E-261	Skin_Fibro	TSC22D2
NFATC23	2.4E-265	0.591902	0.133	0.039	3.9E-261	Skin_Fibro	NFATC2
TMEM433	1.4E-264	0.559144	0.176	0.062	2.2E-260	Skin_Fibro	TMEM43
PPP1CC1	5.3E-264	0.76426	0.403	0.238	8.4E-260	Skin_Fibro	PPP1CC
CD342	2.1E-263	0.464787	0.177	0.06	3.3E-259	Skin_Fibro	CD34
TCF7L14	5.5E-263	0.410455	0.104	0.025	8.7E-259	Skin_Fibro	TCF7L1
C12orf752	6.5E-262	0.544548	0.282	0.126	1E-257	Skin_Fibro	C12orf75
PLXDC12	3.1E-260	0.333447	0.075	0.014	4.9E-256	Skin_Fibro	PLXDC1
PLIN21	4E-259	0.987888	0.254	0.117	6.4E-255	Skin_Fibro	PLIN2
SCPEP13	1.7E-258	0.593913	0.2	0.077	2.7E-254	Skin_Fibro	SCPEP1
ETS14	2.8E-257	0.568223	0.189	0.069	4.5E-253	Skin_Fibro	ETS1
HNRNPDL3	4.6E-257	0.54359	0.528	0.323	7.3E-253	Skin_Fibro	HNRNPDL
FGFBP21	4.6E-255	0.285103	0.121	0.032	7.3E-251	Skin_Fibro	FGFBP2
TMEM474	7.9E-255	0.583903	0.148	0.048	1.3E-250	Skin_Fibro	TMEM47
FRMD61	1.9E-254	0.644829	0.2	0.078	3.1E-250	Skin_Fibro	FRMD6
SOD15	4.4E-254	0.573312	0.591	0.403	7.1E-250	Skin_Fibro	SOD1
GAS72	5.5E-254	0.467546	0.137	0.041	8.7E-250	Skin_Fibro	GAS7
HSPH11	8.6E-254	0.708617	0.388	0.222	1.4E-249	Skin_Fibro	HSPH1
FAM3C2	1.2E-251	0.768387	0.339	0.187	1.9E-247	Skin_Fibro	FAM3C
PDLIM45	2.3E-249	0.667532	0.225	0.097	3.7E-245	Skin_Fibro	PDLIM4
GALNT162	2.5E-249	0.382189	0.085	0.019	3.9E-245	Skin_Fibro	GALNT16
CBX66	7.4E-249	0.622694	0.195	0.076	1.2E-244	Skin_Fibro	CBX6
CNTN1	1.2E-247	0.369282	0.079	0.016	1.9E-243	Skin_Fibro	CNTN1
PHLDB14	6.2E-247	0.508446	0.137	0.042	9.9E-243	Skin_Fibro	PHLDB1
DDX213	3.6E-246	0.780736	0.345	0.189	5.8E-242	Skin_Fibro	DDX21
DST6	1.3E-245	0.413083	0.331	0.174	2E-241	Skin_Fibro	DST
FSCN12	1.8E-245	0.512166	0.168	0.06	2.8E-241	Skin_Fibro	FSCN1
RPL383	3.6E-245	0.346901	0.915	0.751	5.7E-241	Skin_Fibro	RPL38
SNED11	1.1E-244	0.307515	0.074	0.014	1.8E-240	Skin_Fibro	SNED1
ABCA62	1.4E-244	0.336798	0.094	0.022	2.3E-240	Skin_Fibro	ABCA6
TPBG2	1.5E-244	0.679166	0.182	0.07	2.4E-240	Skin_Fibro	TPBG
HMGN23	1.7E-243	0.714693	0.473	0.318	2.7E-239	Skin_Fibro	HMGN2
CLMP2	2.5E-243	0.25253	0.062	0.01	4.1E-239	Skin_Fibro	CLMP
OAZ12	6.8E-243	0.392746	0.883	0.776	1.1E-238	Skin_Fibro	OAZ1
COPS81	8.5E-243	0.70543	0.247	0.115	1.4E-238	Skin_Fibro	COPS8
RPS92	1.4E-242	0.263694	0.983	0.826	2.2E-238	Skin_Fibro	RPS9
TNFRSF1A4	6.9E-242	0.728296	0.295	0.151	1.1E-237	Skin_Fibro	TNFRSF1A
GGT54	9.6E-242	0.351375	0.089	0.02	1.5E-237	Skin_Fibro	GGT5
ANKRD283	1.5E-241	0.689843	0.231	0.101	2.3E-237	Skin_Fibro	ANKRD28

RAN5	7.3E-241	0.637591	0.527	0.356	1.2E-236	Skin_Fibro	RAN
ZBTB204	9.5E-241	0.711441	0.261	0.125	1.5E-236	Skin_Fibro	ZBTB20
RPS144	1.5E-240	0.271952	0.997	0.874	2.4E-236	Skin_Fibro	RPS14
HSPE14	7.9E-240	0.665301	0.524	0.362	1.3E-235	Skin_Fibro	HSPE1
KDELR25	9.4E-240	0.644004	0.472	0.308	1.5E-235	Skin_Fibro	KDELR2
TUBA1A6	1.8E-239	0.555226	0.403	0.223	2.9E-235	Skin_Fibro	TUBA1A
SEC23A2	3.5E-239	0.57868	0.171	0.063	5.6E-235	Skin_Fibro	SEC23A
CSRNP13	7.3E-239	0.722334	0.268	0.129	1.2E-234	Skin_Fibro	CSRNP1
RBFOX3	2.1E-238	0.397229	0.057	0.009	3.4E-234	Skin_Fibro	RBFOX3
STRN31	1.6E-236	0.786847	0.201	0.085	2.6E-232	Skin_Fibro	STRN3
ITGB1BP14	6.1E-236	0.621193	0.286	0.142	9.7E-232	Skin_Fibro	ITGB1BP1
RCN13	4.5E-235	0.721877	0.273	0.139	7.1E-231	Skin_Fibro	RCN1
SRSF116	8.6E-235	0.715058	0.448	0.292	1.4E-230	Skin_Fibro	SRSF11
IL1R13	6.9E-234	0.645349	0.177	0.068	1.1E-229	Skin_Fibro	IL1R1
MUC12	3.5E-233	0.361112	0.076	0.016	5.5E-229	Skin_Fibro	MUC12
PCBP21	4E-233	0.601388	0.567	0.41	6.5E-229	Skin_Fibro	PCBP2
CCNL12	4.6E-233	0.713068	0.485	0.329	7.3E-229	Skin_Fibro	CCNL1
DSTN4	1.1E-232	0.423425	0.736	0.551	1.8E-228	Skin_Fibro	DSTN
COMMD64	1.5E-232	0.477391	0.655	0.461	2.3E-228	Skin_Fibro	COMMD6
STEAP21	2E-232	0.520641	0.098	0.025	3.2E-228	Skin_Fibro	STEAP2
ABCA91	5E-231	0.281745	0.065	0.012	8E-227	Skin_Fibro	ABCA9
ANTXR24	1.8E-230	0.35931	0.106	0.028	2.9E-226	Skin_Fibro	ANTXR2
PDGFD2	1.3E-229	0.375351	0.083	0.018	2E-225	Skin_Fibro	PDGFD
MSC	1.6E-229	0.37158	0.08	0.017	2.5E-225	Skin_Fibro	MSC
CYB5A5	5E-229	0.724343	0.316	0.173	7.9E-225	Skin_Fibro	CYB5A
ROR1	5.8E-229	0.281308	0.061	0.01	9.3E-225	Skin_Fibro	ROR1
IFRD11	8.4E-229	0.752451	0.36	0.21	1.3E-224	Skin_Fibro	IFRD1
ARID5A3	1.2E-228	0.595673	0.215	0.09	1.9E-224	Skin_Fibro	ARID5A
CTTN1	1.9E-227	0.832893	0.337	0.198	3E-223	Skin_Fibro	CTTN
ZC3H12A3	5.2E-227	0.65879	0.215	0.093	8.2E-223	Skin_Fibro	ZC3H12A
EFNA51	5.9E-227	0.498767	0.104	0.028	9.4E-223	Skin_Fibro	EFNA5
SNTB22	9E-227	0.643465	0.183	0.073	1.4E-222	Skin_Fibro	SNTB2
KDELC21	2.2E-226	0.431596	0.11	0.031	3.5E-222	Skin_Fibro	KDELC2
RAP1B3	1.2E-225	0.729702	0.413	0.259	1.9E-221	Skin_Fibro	RAP1B
ATOH81	8.1E-225	0.288788	0.066	0.012	1.3E-220	Skin_Fibro	ATOH8
RBMS11	1.1E-224	0.717336	0.355	0.204	1.7E-220	Skin_Fibro	RBMS1
UBE2S5	2.3E-223	0.777877	0.253	0.123	3.6E-219	Skin_Fibro	UBE2S
TNFSF142	4.9E-223	0.298646	0.051	0.007	7.8E-219	Skin_Fibro	TNFSF14
MBOAT1	2.4E-222	0.336911	0.074	0.016	3.8E-218	Skin_Fibro	MBOAT1
IRF2BPL4	2.5E-222	0.68451	0.176	0.069	4E-218	Skin_Fibro	IRF2BPL
SDF42	1.7E-221	0.71189	0.341	0.199	2.7E-217	Skin_Fibro	SDF4
GAPDH6	3.1E-221	0.3388	0.887	0.755	5E-217	Skin_Fibro	GAPDH
SERF25	5.8E-220	0.415061	0.922	0.816	9.2E-216	Skin_Fibro	SERF2
NFE2L23	5.4E-219	0.782099	0.421	0.278	8.7E-215	Skin_Fibro	NFE2L2
FOSB8	6.2E-219	0.458632	0.416	0.24	9.9E-215	Skin_Fibro	FOSB
TSKU1	1E-218	0.416507	0.107	0.03	1.6E-214	Skin_Fibro	TSKU
ST6GALNA	6E-218	0.535528	0.168	0.064	9.6E-214	Skin_Fibro	ST6GALNAC6

C6orf481	7.8E-218	0.44814	0.489	0.3	1.3E-213	Skin_Fibro	C6orf48
ITGAV2	2E-217	0.697232	0.201	0.088	3.2E-213	Skin_Fibro	ITGAV
ID43	5.8E-217	0.635908	0.178	0.071	9.3E-213	Skin_Fibro	ID4
SOX42	2.1E-215	0.721925	0.411	0.248	3.3E-211	Skin_Fibro	SOX4
GLS6	9.9E-215	0.671049	0.233	0.109	1.6E-210	Skin_Fibro	GLS
CSNK1A15	3.9E-214	0.716418	0.409	0.262	6.2E-210	Skin_Fibro	CSNK1A1
AKR1C32	6E-214	0.422636	0.151	0.053	9.6E-210	Skin_Fibro	AKR1C3
RP11.345J4	9.3E-214	0.339184	0.094	0.025	1.5E-209	Skin_Fibro	RP11.345J4.5
RHEB3	9.7E-213	0.704898	0.471	0.328	1.5E-208	Skin_Fibro	RHEB
OAF1	2.1E-212	0.500946	0.137	0.047	3.3E-208	Skin_Fibro	OAF
C14orf132	2.1E-212	0.281896	0.07	0.014	3.4E-208	Skin_Fibro	C14orf132
PTGIS1	4.5E-212	0.307861	0.086	0.021	7.1E-208	Skin_Fibro	PTGIS
DKK35	1.7E-210	0.482157	0.121	0.038	2.7E-206	Skin_Fibro	DKK3
FAM126A3	2.2E-210	0.527022	0.151	0.055	3.6E-206	Skin_Fibro	FAM126A
AAED15	1.2E-209	0.548972	0.174	0.069	1.9E-205	Skin_Fibro	AAED1
PALM2	6.5E-209	0.313834	0.081	0.019	1E-204	Skin_Fibro	PALM
RPS262	6.7E-209	0.274712	0.886	0.732	1.1E-204	Skin_Fibro	RPS26
TMEM258	1.2E-208	0.501929	0.605	0.442	1.9E-204	Skin_Fibro	TMEM258
VSTM42	4.1E-208	0.350316	0.092	0.024	6.5E-204	Skin_Fibro	VSTM4
NCKAP53	6E-208	0.302882	0.064	0.013	9.6E-204	Skin_Fibro	NCKAP5
RPL37A3	1E-207	0.26599	0.975	0.847	1.7E-203	Skin_Fibro	RPL37A
SRSF26	1.7E-207	0.646857	0.473	0.322	2.7E-203	Skin_Fibro	SRSF2
SNHG85	1.9E-207	0.591176	0.448	0.286	3E-203	Skin_Fibro	SNHG8
INHBB2	1.9E-206	0.370122	0.075	0.017	3E-202	Skin_Fibro	INHBB
MN11	5.1E-206	0.291439	0.063	0.012	8.2E-202	Skin_Fibro	MN1
ENPP22	1.2E-205	0.379454	0.098	0.027	1.9E-201	Skin_Fibro	ENPP2
SFPQ6	2.3E-205	0.719829	0.418	0.276	3.7E-201	Skin_Fibro	SFPQ
MTCH11	1.2E-204	0.68537	0.37	0.234	1.9E-200	Skin_Fibro	MTCH1
AOX11	2.2E-203	0.292867	0.065	0.013	3.4E-199	Skin_Fibro	AOX1
NAV12	2.7E-202	0.338337	0.093	0.025	4.3E-198	Skin_Fibro	NAV1
F2R3	4.8E-202	0.502838	0.134	0.046	7.6E-198	Skin_Fibro	F2R
RAB23	6E-202	0.253897	0.061	0.012	9.6E-198	Skin_Fibro	RAB23
ERH3	1.2E-201	0.645294	0.446	0.302	1.9E-197	Skin_Fibro	ERH
ATP6AP24	1.2E-201	0.664667	0.372	0.23	2E-197	Skin_Fibro	ATP6AP2
INSIG12	5.7E-201	0.337747	0.342	0.198	9.1E-197	Skin_Fibro	INSIG1
PQLC34	7.6E-201	0.596614	0.194	0.085	1.2E-196	Skin_Fibro	PQLC3
DSEL1	1.8E-200	0.419198	0.103	0.03	2.9E-196	Skin_Fibro	DSEL
JUND7	5.3E-200	0.45383	0.611	0.453	8.4E-196	Skin_Fibro	JUND
MMP191	2.9E-199	0.268175	0.063	0.012	4.6E-195	Skin_Fibro	MMP19
RPL153	3.3E-199	0.252557	0.994	0.888	5.3E-195	Skin_Fibro	RPL15
HEXB6	3.6E-199	0.689888	0.28	0.153	5.7E-195	Skin_Fibro	HEXB
MEGF6	4.7E-199	0.301586	0.068	0.014	7.4E-195	Skin_Fibro	MEGF6
CYP7B1	9.7E-199	0.283087	0.062	0.012	1.5E-194	Skin_Fibro	CYP7B1
MFHAS12	1.3E-198	0.483305	0.113	0.036	2.1E-194	Skin_Fibro	MFHAS1
RHOA3	8.2E-197	0.617107	0.589	0.466	1.3E-192	Skin_Fibro	RHOA
TMSB15B	2.1E-195	0.263894	0.064	0.013	3.3E-191	Skin_Fibro	TMSB15B
DNAJA14	4.5E-195	0.671113	0.492	0.352	7.2E-191	Skin_Fibro	DNAJA1

OLFML2B3	4.9E-194	0.321045	0.09	0.024	7.8E-190	Skin_Fibro	OLFML2B
TOMM74	2.8E-193	0.377208	0.742	0.571	4.5E-189	Skin_Fibro	TOMM7
BHLHE402	8.8E-193	0.659686	0.319	0.178	1.4E-188	Skin_Fibro	BHLHE40
ANP32E3	2.3E-192	0.638149	0.199	0.09	3.7E-188	Skin_Fibro	ANP32E
TSC22D32	2.4E-192	0.424691	0.441	0.27	3.9E-188	Skin_Fibro	TSC22D3
HMCN12	7.6E-192	0.266471	0.058	0.011	1.2E-187	Skin_Fibro	HMCN1
OSTC5	3.8E-191	0.645632	0.404	0.269	6.1E-187	Skin_Fibro	OSTC
BCL61	4.2E-191	0.565173	0.167	0.068	6.7E-187	Skin_Fibro	BCL6
SCML11	2.4E-190	0.476795	0.161	0.064	3.8E-186	Skin_Fibro	SCML1
EIF1B3	2.4E-190	0.647107	0.38	0.241	3.8E-186	Skin_Fibro	EIF1B
MPRIP.AS1	5.4E-190	0.296496	0.054	0.01	8.6E-186	Skin_Fibro	MPRIP.AS1
TMEM230	6.2E-190	0.656515	0.395	0.258	9.8E-186	Skin_Fibro	TMEM230
TYMP3	1E-188	0.374385	0.318	0.176	1.6E-184	Skin_Fibro	TYMP
FSTL31	1.4E-188	0.389862	0.091	0.025	2.3E-184	Skin_Fibro	FSTL3
SIDT2	4.5E-188	0.606788	0.127	0.045	7.1E-184	Skin_Fibro	SIDT2
GLIS22	3.6E-187	0.290019	0.069	0.016	5.7E-183	Skin_Fibro	GLIS2
AL592183.	1.3E-186	0.313008	0.077	0.019	2.1E-182	Skin_Fibro	AL592183.1
CALHM24	5.7E-186	0.317742	0.1	0.03	9.1E-182	Skin_Fibro	CALHM2
RP11.983P	1E-185	0.38116	0.09	0.025	1.6E-181	Skin_Fibro	RP11.983P16.4
P4HA12	8.2E-185	0.627882	0.176	0.077	1.3E-180	Skin_Fibro	P4HA1
SVIL6	3.9E-183	0.60556	0.225	0.112	6.3E-179	Skin_Fibro	SVIL
TMEM55A	1.6E-182	0.372622	0.111	0.036	2.6E-178	Skin_Fibro	TMEM55A
RAC13	1.2E-181	0.434374	0.685	0.538	1.9E-177	Skin_Fibro	RAC1
NFIB10	5.6E-181	0.593388	0.319	0.182	9E-177	Skin_Fibro	NFIB
NTM1	6.3E-180	0.303701	0.053	0.01	1E-175	Skin_Fibro	NTM
SH3KBP13	7.3E-180	0.462984	0.187	0.081	1.2E-175	Skin_Fibro	SH3KBP1
BCL32	1.1E-179	0.650036	0.2	0.094	1.8E-175	Skin_Fibro	BCL3
CPNE82	4.3E-179	0.480429	0.109	0.036	6.9E-175	Skin_Fibro	CPNE8
COCH1	9.7E-179	1.774143	0.067	0.015	1.5E-174	Skin_Fibro	COCH
PPTC72	1.2E-178	0.555029	0.146	0.058	1.9E-174	Skin_Fibro	PPTC7
RPS114	5.8E-178	0.256233	0.932	0.766	9.2E-174	Skin_Fibro	RPS11
ARL22	4.4E-177	0.547423	0.338	0.201	7E-173	Skin_Fibro	ARL2
OAT6	3.6E-176	0.663687	0.282	0.163	5.7E-172	Skin_Fibro	OAT
RNF242	7.4E-176	0.539552	0.148	0.059	1.2E-171	Skin_Fibro	RNF24
BAD	1.4E-175	0.680103	0.301	0.182	2.3E-171	Skin_Fibro	BAD
ATF44	2E-175	0.603284	0.457	0.329	3.1E-171	Skin_Fibro	ATF4
PIGT3	2.3E-175	0.737158	0.273	0.156	3.6E-171	Skin_Fibro	PIGT
LAMA21	1.1E-174	0.265731	0.082	0.022	1.7E-170	Skin_Fibro	LAMA2
BTG32	2.3E-174	0.647761	0.273	0.152	3.6E-170	Skin_Fibro	BTG3
LIX1L3	5.2E-174	0.415101	0.118	0.041	8.3E-170	Skin_Fibro	LIX1L
CBR32	5.3E-174	0.589025	0.173	0.076	8.5E-170	Skin_Fibro	CBR3
CLIC23	1.4E-173	0.406548	0.12	0.042	2.2E-169	Skin_Fibro	CLIC2
HOXB.AS3	2.6E-173	0.287103	0.055	0.011	4.2E-169	Skin_Fibro	HOXB.AS3
ROMO11	7E-173	0.712984	0.422	0.302	1.1E-168	Skin_Fibro	ROMO1
ZNF7034	1.1E-172	0.419045	0.114	0.039	1.7E-168	Skin_Fibro	ZNF703
QKI4	1.2E-172	0.661474	0.285	0.164	1.9E-168	Skin_Fibro	QKI
AC010642.	2.9E-171	0.290967	0.102	0.032	4.6E-167	Skin_Fibro	AC010642.1

CHMP4B2	5.6E-171	0.647093	0.434	0.297	9E-167	Skin_Fibro	CHMP4B
NAB12	6.3E-170	0.547678	0.141	0.056	1E-165	Skin_Fibro	NAB1
PTBP2	2.9E-169	0.502778	0.104	0.034	4.6E-165	Skin_Fibro	PTBP2
SON7	8.2E-169	0.585782	0.529	0.401	1.3E-164	Skin_Fibro	SON
RAPH12	8.3E-168	0.525755	0.126	0.048	1.3E-163	Skin_Fibro	RAPH1
SPRED13	1.1E-166	0.441591	0.111	0.038	1.7E-162	Skin_Fibro	SPRED1
CDC42EP4:	5.3E-166	0.478609	0.132	0.051	8.5E-162	Skin_Fibro	CDC42EP4
SKI3	5.7E-166	0.46039	0.139	0.055	9.1E-162	Skin_Fibro	SKI
COL4A25	1.9E-165	0.419876	0.131	0.049	3E-161	Skin_Fibro	COL4A2
ITM2C3	1.9E-165	0.346038	0.229	0.113	3E-161	Skin_Fibro	ITM2C
HOXA10	3.2E-165	0.403082	0.094	0.029	5E-161	Skin_Fibro	HOXA10
ADAM171	4.3E-165	0.613982	0.179	0.083	6.8E-161	Skin_Fibro	ADAM17
ATP13A31	4.8E-165	0.653441	0.185	0.087	7.6E-161	Skin_Fibro	ATP13A3
CDC424	1.7E-164	0.607352	0.577	0.463	2.7E-160	Skin_Fibro	CDC42
C5orf15	2.5E-164	0.595455	0.166	0.075	3.9E-160	Skin_Fibro	C5orf15
HNRNPA1L	7.9E-164	0.481283	0.121	0.044	1.3E-159	Skin_Fibro	HNRNPA1L2
DBN12	1.7E-163	0.448792	0.125	0.047	2.7E-159	Skin_Fibro	DBN1
MEF2A4	2.6E-163	0.594142	0.202	0.1	4.2E-159	Skin_Fibro	MEF2A
NEDD84	4.9E-163	0.503743	0.539	0.415	7.8E-159	Skin_Fibro	NEDD8
ARL13B1	2.2E-162	0.492957	0.119	0.044	3.5E-158	Skin_Fibro	ARL13B
ATXN14	2.7E-162	0.522964	0.155	0.066	4.3E-158	Skin_Fibro	ATXN1
PCSK1N1	5.7E-162	0.875589	0.139	0.056	9.1E-158	Skin_Fibro	PCSK1N
OLFM12	5.8E-162	0.297163	0.076	0.021	9.3E-158	Skin_Fibro	OLFM1
NAP1L13	6.6E-162	0.487561	0.537	0.392	1E-157	Skin_Fibro	NAP1L1
GNAI11	8.4E-162	0.47324	0.139	0.056	1.3E-157	Skin_Fibro	GNAI1
RNH14	1.8E-161	0.565685	0.431	0.306	2.9E-157	Skin_Fibro	RNH1
SPCS22	2E-161	0.485575	0.515	0.399	3.1E-157	Skin_Fibro	SPCS2
PTGIR3	3.7E-161	0.308116	0.069	0.017	6E-157	Skin_Fibro	PTGIR
EVA1B4	1.4E-160	0.520778	0.153	0.065	2.3E-156	Skin_Fibro	EVA1B
SMTN3	1.4E-160	0.373853	0.108	0.037	2.3E-156	Skin_Fibro	SMTN
ERRFI1	2.3E-160	0.640593	0.283	0.163	3.6E-156	Skin_Fibro	ERRFI1
ITM2A5	8.6E-160	0.483424	0.251	0.128	1.4E-155	Skin_Fibro	ITM2A
OLFML2A4	1.6E-159	0.281662	0.074	0.02	2.5E-155	Skin_Fibro	OLFML2A
TACC17	3.2E-159	0.579172	0.279	0.159	5.1E-155	Skin_Fibro	TACC1
SH3BGRL7	2.5E-158	0.517663	0.373	0.235	3.9E-154	Skin_Fibro	SH3BGRL
SPAG93	5.7E-158	0.686589	0.301	0.186	9.1E-154	Skin_Fibro	SPAG9
NDUFS56	1.3E-156	0.46539	0.571	0.447	2.1E-152	Skin_Fibro	NDUFS5
TMEM45A	9.4E-156	0.618546	0.19	0.094	1.5E-151	Skin_Fibro	TMEM45A
SYNE15	1.1E-155	0.39011	0.133	0.052	1.7E-151	Skin_Fibro	SYNE1
C1QTNF13	9.9E-155	0.384501	0.094	0.03	1.6E-150	Skin_Fibro	C1QTNF1
LDB22	1E-154	0.351255	0.109	0.038	1.6E-150	Skin_Fibro	LDB2
JAZF1	7.3E-154	0.422265	0.112	0.041	1.2E-149	Skin_Fibro	JAZF1
LSAMP1	2.4E-153	0.33029	0.065	0.017	3.8E-149	Skin_Fibro	LSAMP
HNRNPK2	2.9E-153	0.518993	0.552	0.439	4.6E-149	Skin_Fibro	HNRNPK
RPL2212	3.5E-153	0.296719	0.859	0.682	5.7E-149	Skin_Fibro	RPL22
RP11.294J:	6E-153	0.349351	0.083	0.025	9.6E-149	Skin_Fibro	RP11.294J22.6
CRIP24	8.3E-153	0.534323	0.322	0.196	1.3E-148	Skin_Fibro	CRIP2

MOXD11	1.6E-152	0.264945	0.06	0.014	2.6E-148	Skin_Fibro	MOXD1
SRI2	2.1E-152	0.571312	0.357	0.234	3.3E-148	Skin_Fibro	SRI
RIPK21	6.8E-152	0.527897	0.136	0.056	1.1E-147	Skin_Fibro	RIPK2
ZNF670	9.4E-152	0.304689	0.058	0.013	1.5E-147	Skin_Fibro	ZNF670
NINJ13	1.2E-150	0.554447	0.25	0.138	1.8E-146	Skin_Fibro	NINJ1
UGCG2	2.6E-150	0.759005	0.203	0.105	4.2E-146	Skin_Fibro	UGCG
ABL11	3.8E-150	0.410548	0.113	0.042	6.1E-146	Skin_Fibro	ABL1
ITGB53	2.1E-149	0.539958	0.159	0.072	3.3E-145	Skin_Fibro	ITGB5
NDRG12	1.5E-148	0.466181	0.25	0.136	2.5E-144	Skin_Fibro	NDRG1
TMEM237	3.1E-148	0.403102	0.105	0.038	5E-144	Skin_Fibro	TMEM237
EIF1AX3	3.5E-148	0.636374	0.44	0.33	5.6E-144	Skin_Fibro	EIF1AX
TUSC12	2.7E-147	0.521413	0.145	0.063	4.3E-143	Skin_Fibro	TUSC1
TMA71	1E-146	0.368769	0.76	0.654	1.7E-142	Skin_Fibro	TMA7
PGF3	1.4E-146	0.338954	0.083	0.025	2.3E-142	Skin_Fibro	PGF
RABAC110	8.8E-146	0.359704	0.539	0.395	1.4E-141	Skin_Fibro	RABAC1
AIDA2	1.6E-145	0.555494	0.192	0.097	2.6E-141	Skin_Fibro	AIDA
TMEM165	1.7E-145	0.661389	0.309	0.198	2.7E-141	Skin_Fibro	TMEM165
AK31	1.7E-145	0.656653	0.256	0.151	2.7E-141	Skin_Fibro	AK3
OST44	4.3E-144	0.374964	0.644	0.491	6.8E-140	Skin_Fibro	OST4
NOTCH2NL	4.6E-144	0.495329	0.171	0.081	7.3E-140	Skin_Fibro	NOTCH2NL
NCL7	3.6E-143	0.495266	0.393	0.262	5.7E-139	Skin_Fibro	NCL
CHSY12	3.8E-143	0.469814	0.124	0.05	6.1E-139	Skin_Fibro	CHSY1
JUN6	4.2E-143	0.388537	0.637	0.477	6.7E-139	Skin_Fibro	JUN
AKAP122	1.5E-142	0.446663	0.156	0.069	2.3E-138	Skin_Fibro	AKAP12
MINOS11	6E-141	0.600565	0.415	0.3	9.5E-137	Skin_Fibro	MINOS1
LARP61	6.9E-141	0.453708	0.163	0.075	1.1E-136	Skin_Fibro	LARP6
HMGN36	8.7E-141	0.583406	0.39	0.27	1.4E-136	Skin_Fibro	HMGN3
ARL4D2	1.7E-140	0.586437	0.18	0.089	2.7E-136	Skin_Fibro	ARL4D
FNDC3B4	2.1E-140	0.686155	0.244	0.143	3.4E-136	Skin_Fibro	FNDC3B
GPBP13	2.2E-140	0.625146	0.425	0.313	3.6E-136	Skin_Fibro	GPBP1
CNBP2	3.7E-140	0.394778	0.546	0.419	5.9E-136	Skin_Fibro	CNBP
CTSO1	2E-139	0.502772	0.135	0.057	3.2E-135	Skin_Fibro	CTSO
ZNF423	1.6E-138	0.266962	0.061	0.016	2.5E-134	Skin_Fibro	ZNF423
CTNNB14	4.6E-138	0.771709	0.27	0.165	7.4E-134	Skin_Fibro	CTNNB1
SLFN114	1.1E-136	0.319752	0.086	0.028	1.8E-132	Skin_Fibro	SLFN11
C9orf35	3E-136	0.544328	0.184	0.093	4.8E-132	Skin_Fibro	C9orf3
PAN33	6.5E-136	0.571159	0.16	0.076	1E-131	Skin_Fibro	PAN3
NR3C16	1E-135	0.530843	0.248	0.142	1.6E-131	Skin_Fibro	NR3C1
NME1.NM	1.6E-135	0.250339	0.074	0.022	2.6E-131	Skin_Fibro	NME1.NME2
HSPA85	2.1E-135	0.375844	0.618	0.503	3.4E-131	Skin_Fibro	HSPA8
FLNA9	4.8E-135	0.278967	0.26	0.142	7.6E-131	Skin_Fibro	FLNA
NGFRAP13	8.5E-135	0.552177	0.311	0.2	1.4E-130	Skin_Fibro	NGFRAP1
ATP2B46	4.8E-134	0.419219	0.16	0.074	7.6E-130	Skin_Fibro	ATP2B4
SQSTM1	4.9E-134	0.320521	0.603	0.471	7.9E-130	Skin_Fibro	SQSTM1
KDELR36	7.9E-134	0.525705	0.165	0.08	1.3E-129	Skin_Fibro	KDELR3
NXT12	2.2E-132	0.529807	0.251	0.146	3.5E-128	Skin_Fibro	NXT1
PYURF1	4.9E-132	0.561999	0.35	0.24	7.9E-128	Skin_Fibro	PYURF

WIPF15	9.2E-132	0.371315	0.18	0.086	1.5E-127	Skin_Fibro	WIPF1
PPP1R15B2	2.9E-131	0.674613	0.246	0.146	4.6E-127	Skin_Fibro	PPP1R15B
FOSL1	4.6E-131	0.471847	0.163	0.078	7.3E-127	Skin_Fibro	FOSL1
FAM127A3	3.3E-130	0.492314	0.172	0.086	5.3E-126	Skin_Fibro	FAM127A
RHOB3	3.8E-130	0.52361	0.306	0.194	6.1E-126	Skin_Fibro	RHOB
MICA	6.6E-130	0.43886	0.125	0.053	1E-125	Skin_Fibro	MICA
GSTA41	1.6E-129	0.328852	0.101	0.038	2.5E-125	Skin_Fibro	GSTA4
SPRY22	3E-129	0.328781	0.077	0.025	4.9E-125	Skin_Fibro	SPRY2
ZBTB161	5.5E-129	0.276751	0.082	0.027	8.8E-125	Skin_Fibro	ZBTB16
MOB3B2	9.8E-129	0.338442	0.074	0.023	1.6E-124	Skin_Fibro	MOB3B
RBM71	1.1E-128	0.602429	0.204	0.112	1.8E-124	Skin_Fibro	RBM7
ABI24	2.5E-128	0.539986	0.166	0.082	3.9E-124	Skin_Fibro	ABI2
LATS2	6.5E-128	0.556669	0.152	0.072	1E-123	Skin_Fibro	LATS2
SLC4A74	4.8E-127	0.453404	0.127	0.055	7.6E-123	Skin_Fibro	SLC4A7
PIP4K2A5	6.8E-126	0.378975	0.129	0.056	1.1E-121	Skin_Fibro	PIP4K2A
NFAT54	7.4E-126	0.599856	0.215	0.122	1.2E-121	Skin_Fibro	NFAT5
RWDD11	1.4E-125	0.566367	0.406	0.301	2.2E-121	Skin_Fibro	RWDD1
COX16	2.4E-125	0.46165	0.21	0.114	3.8E-121	Skin_Fibro	COX16
ATP5I1	4.5E-125	0.517889	0.474	0.365	7.2E-121	Skin_Fibro	ATP5I
CASC45	5.5E-125	0.604394	0.207	0.116	8.8E-121	Skin_Fibro	CASC4
PTP4A24	9.1E-125	0.597501	0.387	0.286	1.4E-120	Skin_Fibro	PTP4A2
KANK25	5.6E-124	0.318945	0.103	0.039	8.9E-120	Skin_Fibro	KANK2
PRKAR1A2	1.8E-123	0.638236	0.332	0.232	2.9E-119	Skin_Fibro	PRKAR1A
KPNA22	7.7E-123	0.433931	0.193	0.101	1.2E-118	Skin_Fibro	KPNA2
RNF2171	1.6E-122	0.442918	0.104	0.041	2.5E-118	Skin_Fibro	RNF217
CYCS4	1.8E-122	0.333827	0.449	0.334	2.9E-118	Skin_Fibro	CYCS
CNIH11	2.7E-122	0.512974	0.218	0.123	4.2E-118	Skin_Fibro	CNIH1
MAGEH13	4.4E-122	0.28721	0.106	0.042	7E-118	Skin_Fibro	MAGEH1
SUMO25	6.5E-122	0.370755	0.694	0.595	1E-117	Skin_Fibro	SUMO2
ARL5B3	4.4E-121	0.527285	0.178	0.093	7E-117	Skin_Fibro	ARL5B
HNRNPA2E	1.5E-120	0.33712	0.608	0.483	2.4E-116	Skin_Fibro	HNRNPA2B1
CMTM32	3.8E-120	0.312232	0.152	0.071	6.1E-116	Skin_Fibro	CMTM3
DENND5A2	1.3E-119	0.363879	0.098	0.038	2E-115	Skin_Fibro	DENND5A
UBE2D33	3.4E-119	0.426085	0.63	0.526	5.4E-115	Skin_Fibro	UBE2D3
CAPZA23	4.6E-119	0.604576	0.372	0.273	7.4E-115	Skin_Fibro	CAPZA2
TRMT1123	1.4E-118	0.422059	0.483	0.367	2.2E-114	Skin_Fibro	TRMT112
MAT2A5	1.5E-118	0.619603	0.238	0.144	2.4E-114	Skin_Fibro	MAT2A
CAV25	1.9E-118	0.443093	0.208	0.113	3E-114	Skin_Fibro	CAV2
AHNAK22	3.9E-118	0.380909	0.133	0.06	6.2E-114	Skin_Fibro	AHNAK2
MSRB35	1E-117	0.311284	0.087	0.031	1.7E-113	Skin_Fibro	MSRB3
SRP144	1.4E-117	0.286333	0.804	0.694	2.2E-113	Skin_Fibro	SRP14
DSE3	2.8E-117	0.397734	0.111	0.046	4.5E-113	Skin_Fibro	DSE
PTGER42	2.9E-117	0.321684	0.114	0.047	4.5E-113	Skin_Fibro	PTGER4
SIAH11	3.6E-117	0.648058	0.175	0.093	5.8E-113	Skin_Fibro	SIAH1
EXT11	5.2E-117	0.343693	0.088	0.033	8.3E-113	Skin_Fibro	EXT1
FOSL23	7.9E-117	0.587957	0.319	0.213	1.3E-112	Skin_Fibro	FOSL2
FAM198B2	2E-116	0.317955	0.087	0.031	3.2E-112	Skin_Fibro	FAM198B

IGF13	4E-116	0.288869	0.091	0.033	6.3E-112	Skin_Fibro	IGF1
NAB21	4.7E-116	0.302962	0.075	0.025	7.5E-112	Skin_Fibro	NAB2
EHD25	9.2E-116	0.293681	0.104	0.041	1.5E-111	Skin_Fibro	EHD2
ZEB14	1.2E-115	0.378982	0.125	0.055	2E-111	Skin_Fibro	ZEB1
HNRNPH31	1.5E-115	0.557937	0.318	0.214	2.4E-111	Skin_Fibro	HNRNPH3
ATRAID5	2.5E-115	0.488781	0.391	0.284	3.9E-111	Skin_Fibro	ATRAID
CLK13	1.6E-114	0.528543	0.35	0.245	2.6E-110	Skin_Fibro	CLK1
TGFBI5	2E-114	0.328933	0.132	0.058	3.2E-110	Skin_Fibro	TGFBI
MTDH6	2.1E-114	0.457692	0.41	0.302	3.4E-110	Skin_Fibro	MTDH
SETBP13	2.4E-114	0.35279	0.088	0.033	3.8E-110	Skin_Fibro	SETBP1
LMO48	2.4E-114	0.566567	0.254	0.158	3.8E-110	Skin_Fibro	LMO4
IFI63	4E-114	0.370799	0.255	0.148	6.3E-110	Skin_Fibro	IFI6
TBL1XR11	9.5E-114	0.620194	0.236	0.145	1.5E-109	Skin_Fibro	TBL1XR1
SELK4	1.4E-113	0.365329	0.562	0.463	2.2E-109	Skin_Fibro	SELK
ZFAND5	1.4E-113	0.313747	0.508	0.387	2.2E-109	Skin_Fibro	ZFAND5
ARL6IP11	1.6E-113	0.636371	0.406	0.312	2.5E-109	Skin_Fibro	ARL6IP1
STEAP11	2E-113	0.380352	0.083	0.03	3.2E-109	Skin_Fibro	STEAP1
C11orf582	1.5E-112	0.494752	0.457	0.362	2.4E-108	Skin_Fibro	C11orf58
SH3D191	1.7E-112	0.510048	0.177	0.095	2.7E-108	Skin_Fibro	SH3D19
SLC41A21	3.1E-112	0.401814	0.083	0.03	5E-108	Skin_Fibro	SLC41A2
LIFR2	3.8E-112	0.442917	0.129	0.059	6E-108	Skin_Fibro	LIFR
NOP161	4.3E-112	0.424924	0.14	0.067	6.9E-108	Skin_Fibro	NOP16
ARHGEF10	6.6E-112	0.410762	0.113	0.049	1.1E-107	Skin_Fibro	ARHGEF10
DNAJB41	7.9E-112	0.517064	0.185	0.1	1.3E-107	Skin_Fibro	DNAJB4
VMP11	2E-111	0.577894	0.404	0.304	3.1E-107	Skin_Fibro	VMP1
FUCA23	7.7E-111	0.475422	0.158	0.082	1.2E-106	Skin_Fibro	FUCA2
BDKRB2	8.5E-111	0.315161	0.064	0.02	1.4E-106	Skin_Fibro	BDKRB2
LAMP11	1.6E-110	0.569211	0.384	0.292	2.5E-106	Skin_Fibro	LAMP1
BRD23	1.9E-110	0.61536	0.359	0.265	3E-106	Skin_Fibro	BRD2
MARVELD1	2.3E-110	0.287068	0.081	0.029	3.7E-106	Skin_Fibro	MARVELD1
RAB313	1.9E-109	0.462729	0.171	0.089	3.1E-105	Skin_Fibro	RAB31
BAG31	2E-109	0.450633	0.237	0.143	3.2E-105	Skin_Fibro	BAG3
EIF5A6	3.8E-109	0.563964	0.369	0.272	6.1E-105	Skin_Fibro	EIF5A
ANKRD351	6.1E-109	0.32404	0.091	0.035	9.8E-105	Skin_Fibro	ANKRD35
BRI37	7.5E-109	0.597544	0.433	0.34	1.2E-104	Skin_Fibro	BRI3
HSPG23	1.4E-108	0.441691	0.195	0.108	2.3E-104	Skin_Fibro	HSPG2
PER22	3.2E-108	0.589381	0.155	0.081	5.1E-104	Skin_Fibro	PER2
FUS10	2.2E-107	0.508252	0.372	0.27	3.4E-103	Skin_Fibro	FUS
EGR32	1.9E-106	0.315993	0.082	0.031	3E-102	Skin_Fibro	EGR3
ATP6V1B2	4.1E-106	0.522696	0.158	0.083	6.6E-102	Skin_Fibro	ATP6V1B2
TCN23	7E-106	0.324438	0.085	0.032	1.1E-101	Skin_Fibro	TCN2
NTAN14	8.9E-105	0.499927	0.202	0.118	1.4E-100	Skin_Fibro	NTAN1
RBFOX23	2.9E-104	0.542946	0.181	0.102	4.6E-100	Skin_Fibro	RBFOX2
PTGES34	3.4E-104	0.443472	0.482	0.387	5.4E-100	Skin_Fibro	PTGES3
BAG2	3.6E-104	0.309934	0.088	0.034	5.7E-100	Skin_Fibro	BAG2
RRAGC1	4.4E-104	0.511161	0.176	0.098	6.9E-100	Skin_Fibro	RRAGC
JMJD62	6E-104	0.449484	0.162	0.086	9.6E-100	Skin_Fibro	JMJD6

MOB43	7.2E-104	0.432573	0.165	0.087	1.1E-99	Skin_Fibro	MOB4
HNRNPAB5	7.6E-104	0.559592	0.251	0.161	1.2E-99	Skin_Fibro	HNRNPAB
NUCB12	1.2E-103	0.570042	0.292	0.2	1.8E-99	Skin_Fibro	NUCB1
SLC25A372	2E-103	0.412481	0.263	0.17	3.2E-99	Skin_Fibro	SLC25A37
EIF4A23	7.5E-103	0.353657	0.582	0.479	1.2E-98	Skin_Fibro	EIF4A2
CCDC85B3	8.9E-103	0.317506	0.383	0.264	1.42E-98	Skin_Fibro	CCDC85B
KIF1B1	1.9E-102	0.59672	0.186	0.108	3.06E-98	Skin_Fibro	KIF1B
TWISTNB2	3.9E-101	0.555175	0.165	0.089	6.19E-97	Skin_Fibro	TWISTNB
PDIA34	3.9E-101	0.438838	0.417	0.316	6.25E-97	Skin_Fibro	PDIA3
PKD11	4.7E-101	0.378517	0.103	0.044	7.49E-97	Skin_Fibro	PKD1
NUAK11	9.6E-101	0.314728	0.085	0.033	1.53E-96	Skin_Fibro	NUAK1
SOCS51	1.4E-100	0.364299	0.089	0.036	2.25E-96	Skin_Fibro	SOCS5
CDC14B1	1.7E-100	0.316837	0.074	0.027	2.78E-96	Skin_Fibro	CDC14B
PTK7	2.1E-100	0.397639	0.1	0.043	3.43E-96	Skin_Fibro	PTK7
RORA3	6.8E-100	0.453122	0.201	0.115	1.08E-95	Skin_Fibro	RORA
RAB11FIP2	2.1E-99	0.359382	0.095	0.04	3.34E-95	Skin_Fibro	RAB11FIP2
APLP25	2.7E-99	0.479807	0.392	0.298	4.38E-95	Skin_Fibro	APLP2
ELK31	3.9E-99	0.343336	0.122	0.056	6.29E-95	Skin_Fibro	ELK3
BSG	1.02E-98	0.453059	0.469	0.382	1.62E-94	Skin_Fibro	BSG
PARVA2	1.47E-98	0.403335	0.119	0.056	2.34E-94	Skin_Fibro	PARVA
KLHL151	1.07E-97	0.284601	0.065	0.022	1.71E-93	Skin_Fibro	KLHL15
TNRC6C1	1.95E-97	0.423207	0.123	0.058	3.11E-93	Skin_Fibro	TNRC6C
NAMPT1	2.24E-97	0.281381	0.495	0.374	3.57E-93	Skin_Fibro	NAMPT
MYO1B2	2.72E-97	0.395604	0.136	0.068	4.34E-93	Skin_Fibro	MYO1B
EPB41L24	3.38E-97	0.399255	0.164	0.087	5.38E-93	Skin_Fibro	EPB41L2
SRGAP22	4.08E-97	0.415252	0.105	0.046	6.51E-93	Skin_Fibro	SRGAP2
SMARCA12	2.17E-96	0.445415	0.116	0.054	3.45E-92	Skin_Fibro	SMARCA1
YWHAZ2	2.22E-95	0.324582	0.609	0.503	3.55E-91	Skin_Fibro	YWHAZ
KDM7A2	3.23E-95	0.392105	0.103	0.046	5.16E-91	Skin_Fibro	KDM7A
PELI2	5.01E-95	0.280284	0.069	0.024	7.98E-91	Skin_Fibro	PELI2
SMG14	5.21E-95	0.525196	0.194	0.115	8.3E-91	Skin_Fibro	SMG1
PHLDB23	9.15E-95	0.385578	0.108	0.049	1.46E-90	Skin_Fibro	PHLDB2
MXRA74	2.94E-94	0.388744	0.151	0.079	4.69E-90	Skin_Fibro	MXRA7
KAZALD12	3.83E-94	0.292934	0.074	0.028	6.11E-90	Skin_Fibro	KAZALD1
CRK3	9.69E-94	0.51807	0.192	0.115	1.54E-89	Skin_Fibro	CRK
KRTCAP21	2.76E-93	0.496226	0.395	0.308	4.41E-89	Skin_Fibro	KRTCAP2
NCK12	8.08E-93	0.489404	0.18	0.103	1.29E-88	Skin_Fibro	NCK1
ZNF532	1.17E-92	0.311273	0.072	0.027	1.87E-88	Skin_Fibro	ZNF532
CD1643	1.51E-92	0.487443	0.445	0.366	2.41E-88	Skin_Fibro	CD164
EMP22	2.25E-92	0.384398	0.282	0.184	3.59E-88	Skin_Fibro	EMP2
BRK1	2.64E-92	0.318017	0.534	0.45	4.2E-88	Skin_Fibro	BRK1
FAS	3.15E-92	0.350519	0.097	0.042	5.02E-88	Skin_Fibro	FAS
ATF1	9.95E-92	0.381244	0.125	0.061	1.59E-87	Skin_Fibro	ATF1
TCF121	1.41E-91	0.452422	0.134	0.069	2.25E-87	Skin_Fibro	TCF12
DDX3X5	2.39E-91	0.55894	0.378	0.296	3.82E-87	Skin_Fibro	DDX3X
PPP1R182	2.86E-91	0.29392	0.199	0.112	4.56E-87	Skin_Fibro	PPP1R18
DUT1	4.4E-91	0.416971	0.324	0.23	7.01E-87	Skin_Fibro	DUT

C17orf581	8.5E-91	0.410469	0.086	0.036	1.36E-86	Skin_Fibro	C17orf58
MTPN4	9.02E-91	0.455933	0.229	0.144	1.44E-86	Skin_Fibro	MTPN
IFNGR13	1.72E-90	0.496604	0.21	0.129	2.74E-86	Skin_Fibro	IFNGR1
YWHAH4	3.67E-90	0.481137	0.292	0.201	5.86E-86	Skin_Fibro	YWHAH
CREB3L14	7.27E-90	0.431091	0.135	0.07	1.16E-85	Skin_Fibro	CREB3L1
MAP46	9.7E-90	0.513039	0.202	0.124	1.55E-85	Skin_Fibro	MAP4
PARD3B	1.46E-89	0.285521	0.069	0.026	2.33E-85	Skin_Fibro	PARD3B
USP122	2.25E-89	0.427921	0.13	0.066	3.58E-85	Skin_Fibro	USP12
TCEAL44	2.88E-89	0.41003	0.238	0.154	4.59E-85	Skin_Fibro	TCEAL4
PALLD5	3.41E-89	0.592789	0.235	0.155	5.43E-85	Skin_Fibro	PALLD
SORBS13	9.49E-89	0.347688	0.079	0.032	1.51E-84	Skin_Fibro	SORBS1
AC009501.	1.62E-88	0.288435	0.077	0.03	2.59E-84	Skin_Fibro	AC009501.4
ARNT	1.93E-88	0.371472	0.1	0.045	3.07E-84	Skin_Fibro	ARNT
TMEM173	9.3E-88	0.352378	0.186	0.105	1.48E-83	Skin_Fibro	TMEM173
SRSF64	1.2E-87	0.567974	0.217	0.14	1.92E-83	Skin_Fibro	SRSF6
JDP22	1.87E-87	0.383014	0.091	0.039	2.98E-83	Skin_Fibro	JDP2
FLCN	2.86E-87	0.342732	0.084	0.035	4.56E-83	Skin_Fibro	FLCN
PANX1	3.45E-87	0.273765	0.071	0.027	5.5E-83	Skin_Fibro	PANX1
EIF4A33	4.67E-87	0.582627	0.277	0.194	7.44E-83	Skin_Fibro	EIF4A3
ARMCX34	5.32E-87	0.511359	0.218	0.14	8.48E-83	Skin_Fibro	ARMCX3
LINC00969	7.37E-87	0.456196	0.181	0.106	1.17E-82	Skin_Fibro	LINC00969
LACTB1	1.03E-86	0.485661	0.148	0.081	1.65E-82	Skin_Fibro	LACTB
PARK79	2.21E-86	0.269302	0.463	0.348	3.52E-82	Skin_Fibro	PARK7
NREP2	5.45E-86	0.343311	0.087	0.037	8.68E-82	Skin_Fibro	NREP
TSPYL14	7.2E-86	0.59479	0.226	0.15	1.15E-81	Skin_Fibro	TSPYL1
UBE2E22	8.16E-86	0.466456	0.14	0.075	1.3E-81	Skin_Fibro	UBE2E2
SH3BP53	3.14E-85	0.364488	0.189	0.11	5.01E-81	Skin_Fibro	SH3BP5
SEMA4C2	3.21E-85	0.261861	0.064	0.023	5.12E-81	Skin_Fibro	SEMA4C
TGFBR12	4.71E-85	0.449881	0.117	0.058	7.51E-81	Skin_Fibro	TGFBR1
SGMS22	5.01E-85	0.436399	0.11	0.053	7.99E-81	Skin_Fibro	SGMS2
RAB323	1.67E-84	0.378409	0.154	0.085	2.66E-80	Skin_Fibro	RAB32
PFDN23	1.9E-84	0.444848	0.359	0.269	3.03E-80	Skin_Fibro	PFDN2
NFATC11	2.1E-84	0.307142	0.089	0.039	3.35E-80	Skin_Fibro	NFATC1
LINC00657	3.52E-84	0.52378	0.277	0.198	5.61E-80	Skin_Fibro	LINC00657
TRPS15	1.26E-83	0.537217	0.149	0.083	2.01E-79	Skin_Fibro	TRPS1
PRR51	1.56E-83	0.289734	0.093	0.041	2.49E-79	Skin_Fibro	PRR5
ATF33	1.83E-83	0.384211	0.338	0.238	2.92E-79	Skin_Fibro	ATF3
TMEM50B.	1.9E-83	0.53204	0.225	0.149	3.03E-79	Skin_Fibro	TMEM50B
HIBADH1	1.97E-83	0.454962	0.136	0.073	3.14E-79	Skin_Fibro	HIBADH
EXTL21	3.93E-83	0.306985	0.085	0.037	6.27E-79	Skin_Fibro	EXTL2
GADD45G3	5.19E-83	0.461183	0.125	0.064	8.27E-79	Skin_Fibro	GADD45G
SGCB3	8.56E-83	0.421891	0.125	0.064	1.36E-78	Skin_Fibro	SGCB
UBTD2	1.18E-82	0.417991	0.118	0.06	1.89E-78	Skin_Fibro	UBTD2
MCC1	2.14E-82	0.309639	0.079	0.033	3.41E-78	Skin_Fibro	MCC
RBX15	2.94E-82	0.446115	0.422	0.344	4.69E-78	Skin_Fibro	RBX1
TIPIN3	4.4E-82	0.423978	0.133	0.07	7.01E-78	Skin_Fibro	TIPIN
FAM46A5	7.96E-82	0.345714	0.251	0.159	1.27E-77	Skin_Fibro	FAM46A

PDS5B2	5.01E-81	0.471769	0.144	0.079	7.99E-77	Skin_Fibro	PDS5B
TGFBR23	8.58E-81	0.360639	0.188	0.111	1.37E-76	Skin_Fibro	TGFBR2
SFRP41	2.12E-80	0.44215	0.043	0.013	3.39E-76	Skin_Fibro	SFRP4
CAMSAP22	2.67E-80	0.425926	0.131	0.069	4.25E-76	Skin_Fibro	CAMSAP2
FAM133B6	5.19E-80	0.555044	0.271	0.194	8.27E-76	Skin_Fibro	FAM133B
MATN23	5.62E-80	0.30191	0.088	0.039	8.97E-76	Skin_Fibro	MATN2
CORO1C2	1.54E-79	0.477599	0.185	0.113	2.46E-75	Skin_Fibro	CORO1C
MRPL231	1.73E-79	0.429255	0.29	0.206	2.75E-75	Skin_Fibro	MRPL23
PGRMC22	1.99E-79	0.551857	0.208	0.137	3.17E-75	Skin_Fibro	PGRMC2
GPX32	2.07E-79	0.62376	0.195	0.121	3.3E-75	Skin_Fibro	GPX3
RAB3IL12	3.33E-79	0.282931	0.075	0.03	5.3E-75	Skin_Fibro	RAB3IL1
NDFIP1	5.35E-79	0.440772	0.454	0.382	8.53E-75	Skin_Fibro	NDFIP1
SEC11A5	6.7E-79	0.400745	0.393	0.31	1.07E-74	Skin_Fibro	SEC11A
DRAP12	9.81E-79	0.336819	0.424	0.329	1.56E-74	Skin_Fibro	DRAP1
SNRPE2	1.35E-78	0.431302	0.347	0.261	2.16E-74	Skin_Fibro	SNRPE
FZD72	1.54E-78	0.328414	0.079	0.033	2.46E-74	Skin_Fibro	FZD7
SRP92	4.46E-78	0.479802	0.407	0.338	7.11E-74	Skin_Fibro	SRP9
IRS1	1.83E-77	0.259065	0.077	0.032	2.91E-73	Skin_Fibro	IRS1
RHOQ7	1.84E-77	0.422495	0.146	0.082	2.94E-73	Skin_Fibro	RHOQ
PMEPA15	2.92E-77	0.545347	0.185	0.115	4.65E-73	Skin_Fibro	PMEPA1
B3GAT21	3.04E-77	0.314798	0.066	0.026	4.85E-73	Skin_Fibro	B3GAT2
VAT11	3.45E-77	0.478335	0.206	0.134	5.51E-73	Skin_Fibro	VAT1
CCND16	4.4E-77	0.477423	0.186	0.114	7.02E-73	Skin_Fibro	CCND1
DAD18	7.96E-77	0.351868	0.469	0.394	1.27E-72	Skin_Fibro	DAD1
LEPROT4	1.42E-76	0.48431	0.329	0.252	2.26E-72	Skin_Fibro	LEPROT
ST3GAL51	1.51E-76	0.290021	0.066	0.026	2.41E-72	Skin_Fibro	ST3GAL5
SLC16A1	2.07E-76	0.33964	0.09	0.041	3.3E-72	Skin_Fibro	SLC16A1
HEXA1	2.27E-76	0.472379	0.185	0.116	3.62E-72	Skin_Fibro	HEXA
PKD22	5.16E-76	0.260622	0.062	0.023	8.22E-72	Skin_Fibro	PKD2
HYPK	5.78E-76	0.283135	0.064	0.025	9.22E-72	Skin_Fibro	HYPK
WLS4	1.11E-75	0.31237	0.088	0.04	1.77E-71	Skin_Fibro	WLS
HSPD16	1.11E-75	0.472122	0.333	0.252	1.77E-71	Skin_Fibro	HSPD1
SLC39A12	1.17E-75	0.430136	0.172	0.104	1.87E-71	Skin_Fibro	SLC39A1
SETD52	3.51E-75	0.499006	0.182	0.114	5.6E-71	Skin_Fibro	SETD5
TNS16	6.73E-75	0.283394	0.112	0.056	1.07E-70	Skin_Fibro	TNS1
RNASEK2	7.38E-75	0.26098	0.074	0.031	1.18E-70	Skin_Fibro	RNASEK
EIF4E1	1.08E-74	0.379168	0.319	0.238	1.72E-70	Skin_Fibro	EIF4E
TPI13	1.27E-74	0.307467	0.602	0.53	2.03E-70	Skin_Fibro	TPI1
HSD17B11	1.32E-74	0.360149	0.213	0.133	2.1E-70	Skin_Fibro	HSD17B11
TNFSF13B3	1.94E-74	0.292185	0.088	0.039	3.1E-70	Skin_Fibro	TNFSF13B
EEA12	3.26E-74	0.440351	0.16	0.095	5.2E-70	Skin_Fibro	EEA1
HNRNPC3	7.19E-74	0.431842	0.497	0.425	1.15E-69	Skin_Fibro	HNRNPC
SEMA5A4	1.39E-73	0.331361	0.062	0.024	2.22E-69	Skin_Fibro	SEMA5A
NR1D1	2.48E-73	0.264957	0.094	0.044	3.96E-69	Skin_Fibro	NR1D1
PTEN4	3.21E-73	0.528905	0.192	0.124	5.12E-69	Skin_Fibro	PTEN
SEC636	5.79E-73	0.490861	0.238	0.167	9.24E-69	Skin_Fibro	SEC63
SKIL	6.67E-73	0.589268	0.224	0.153	1.06E-68	Skin_Fibro	SKIL

NUP153	7.06E-73	0.413252	0.114	0.06	1.13E-68	Skin_Fibro	NUP153
CCDC28B	7.67E-73	0.255308	0.087	0.039	1.22E-68	Skin_Fibro	CCDC28B
TLE4	8.84E-73	0.34601	0.236	0.154	1.41E-68	Skin_Fibro	TLE4
CDC42EP2:	1.01E-72	0.253463	0.078	0.034	1.62E-68	Skin_Fibro	CDC42EP2
MSN7	1.1E-72	0.300107	0.235	0.151	1.76E-68	Skin_Fibro	MSN
APOL2	9.78E-72	0.29558	0.074	0.032	1.56E-67	Skin_Fibro	APOL2
RAB1A	1.13E-71	0.455792	0.426	0.363	1.81E-67	Skin_Fibro	RAB1A
ARHGAP12	1.47E-71	0.466247	0.138	0.079	2.34E-67	Skin_Fibro	ARHGAP12
RRAS4	1.65E-71	0.449438	0.159	0.095	2.63E-67	Skin_Fibro	RRAS
ANKRD401	1.23E-70	0.34093	0.095	0.046	1.96E-66	Skin_Fibro	ANKRD40
NUFIP23	1.5E-70	0.542151	0.217	0.148	2.39E-66	Skin_Fibro	NUFIP2
TIMM13	3.82E-70	0.423206	0.318	0.242	6.09E-66	Skin_Fibro	TIMM13
RNF1391	7.06E-70	0.330768	0.201	0.126	1.13E-65	Skin_Fibro	RNF139
UBE2E31	7.67E-70	0.455945	0.24	0.169	1.22E-65	Skin_Fibro	UBE2E3
PTPN122	7.84E-70	0.459075	0.168	0.103	1.25E-65	Skin_Fibro	PTPN12
CSGALNAC	8.4E-70	0.264722	0.083	0.038	1.34E-65	Skin_Fibro	CSGALNACT2
C15orf611	8.55E-70	0.414066	0.175	0.109	1.36E-65	Skin_Fibro	C15orf61
ZNF385D1	1.57E-69	0.296946	0.1	0.049	2.5E-65	Skin_Fibro	ZNF385D
BMPR1A2	1.8E-69	0.275475	0.072	0.031	2.87E-65	Skin_Fibro	BMPR1A
AVPI11	2.98E-69	0.474978	0.192	0.123	4.75E-65	Skin_Fibro	AVPI1
TCEAL81	3.19E-69	0.504995	0.231	0.162	5.09E-65	Skin_Fibro	TCEAL8
CBLB2	9.32E-69	0.336555	0.103	0.052	1.49E-64	Skin_Fibro	CBLB
CYSTM15	1.38E-68	0.313527	0.306	0.22	2.2E-64	Skin_Fibro	CYSTM1
ZNF4281	2.22E-68	0.372447	0.236	0.161	3.54E-64	Skin_Fibro	ZNF428
ZDHHC91	4.62E-68	0.386897	0.107	0.056	7.38E-64	Skin_Fibro	ZDHHC9
P4HA22	4.66E-68	0.348192	0.095	0.047	7.43E-64	Skin_Fibro	P4HA2
HNRNPH2:	5.77E-68	0.328048	0.137	0.078	9.21E-64	Skin_Fibro	HNRNPH2
SLC19A2	3.1E-67	0.299409	0.065	0.027	4.94E-63	Skin_Fibro	SLC19A2
PSMA41	3.98E-67	0.443217	0.326	0.251	6.35E-63	Skin_Fibro	PSMA4
TMEM50A	4.28E-67	0.433237	0.474	0.413	6.82E-63	Skin_Fibro	TMEM50A
SUB14	4.4E-67	0.255671	0.542	0.476	7.02E-63	Skin_Fibro	SUB1
COQ10B1	4.51E-67	0.494051	0.21	0.142	7.2E-63	Skin_Fibro	COQ10B
HNMT4	1.19E-66	0.462684	0.167	0.105	1.89E-62	Skin_Fibro	HNMT
CREBRF1	1.78E-66	0.573815	0.201	0.136	2.85E-62	Skin_Fibro	CREBRF
POLR2F1	2.28E-66	0.43732	0.319	0.248	3.63E-62	Skin_Fibro	POLR2F
ARL5A2	2.47E-66	0.469194	0.261	0.191	3.94E-62	Skin_Fibro	ARL5A
SPEN1	4.36E-66	0.475621	0.162	0.1	6.96E-62	Skin_Fibro	SPEN
STRAP1	4.9E-66	0.495361	0.282	0.213	7.81E-62	Skin_Fibro	STRAP
MAP7D11	6.29E-66	0.385818	0.174	0.108	1E-61	Skin_Fibro	MAP7D1
CDC265	6.79E-66	0.473913	0.185	0.123	1.08E-61	Skin_Fibro	CDC26
SERBP12	1.29E-65	0.344573	0.432	0.349	2.06E-61	Skin_Fibro	SERBP1
METTL7A4	1.34E-65	0.419946	0.146	0.087	2.14E-61	Skin_Fibro	METTL7A
FAM114A1	2.21E-65	0.438587	0.183	0.119	3.52E-61	Skin_Fibro	FAM114A1
SP31	2.58E-65	0.441711	0.149	0.089	4.12E-61	Skin_Fibro	SP3
VEGFA3	2.67E-65	0.416045	0.139	0.08	4.26E-61	Skin_Fibro	VEGFA
PABPN15	2.82E-65	0.460088	0.214	0.146	4.5E-61	Skin_Fibro	PABPN1
PPP3R11	3.46E-65	0.411936	0.121	0.067	5.51E-61	Skin_Fibro	PPP3R1

SH3PXD2B	3.78E-65	0.301839	0.082	0.039	6.03E-61	Skin_Fibro	SH3PXD2B
ZBTB7A2	1.09E-64	0.492571	0.183	0.119	1.73E-60	Skin_Fibro	ZBTB7A
MED221	2.26E-64	0.28647	0.062	0.025	3.61E-60	Skin_Fibro	MED22
JAG14	2.94E-64	0.270277	0.096	0.047	4.68E-60	Skin_Fibro	JAG1
ILK3	1.94E-63	0.426713	0.209	0.143	3.1E-59	Skin_Fibro	ILK
TSHZ11	4.02E-63	0.272968	0.078	0.036	6.4E-59	Skin_Fibro	TSHZ1
DDX247	5.65E-63	0.363034	0.311	0.232	9.02E-59	Skin_Fibro	DDX24
PTGER32	1.24E-62	0.298948	0.066	0.028	1.98E-58	Skin_Fibro	PTGER3
SERINC15	2E-62	0.491598	0.259	0.193	3.19E-58	Skin_Fibro	SERINC1
KDSR5	2.52E-62	0.435941	0.19	0.126	4.02E-58	Skin_Fibro	KDSR
HP1BP33	3.11E-62	0.481809	0.287	0.22	4.96E-58	Skin_Fibro	HP1BP3
MID1IP12	3.19E-62	0.346089	0.127	0.072	5.09E-58	Skin_Fibro	MID1IP1
ACVR2A	4.15E-62	0.310623	0.083	0.04	6.62E-58	Skin_Fibro	ACVR2A
SIRT1	4.67E-62	0.376099	0.11	0.059	7.45E-58	Skin_Fibro	SIRT1
SEPW17	7.22E-62	0.258522	0.433	0.354	1.15E-57	Skin_Fibro	SEPW1
EPB41L4A.	1.9E-61	0.388321	0.267	0.194	3.04E-57	Skin_Fibro	EPB41L4A.AS1
FAM134B	2.37E-61	0.330336	0.125	0.069	3.78E-57	Skin_Fibro	FAM134B
TCEAL12	3.28E-61	0.288004	0.099	0.051	5.23E-57	Skin_Fibro	TCEAL1
YY13	4.37E-61	0.52747	0.287	0.223	6.97E-57	Skin_Fibro	YY1
TM2D12	1.04E-60	0.518413	0.216	0.153	1.67E-56	Skin_Fibro	TM2D1
LSM71	1.21E-60	0.2599	0.364	0.28	1.94E-56	Skin_Fibro	LSM7
NDUFS43	1.94E-60	0.464456	0.278	0.214	3.09E-56	Skin_Fibro	NDUFS4
MARCH62	2.25E-60	0.543919	0.222	0.161	3.59E-56	Skin_Fibro	MARCH6
OAZ23	4.63E-60	0.412821	0.241	0.173	7.39E-56	Skin_Fibro	OAZ2
CCND24	5.32E-60	0.390631	0.158	0.098	8.49E-56	Skin_Fibro	CCND2
TRAM22	5.33E-60	0.252143	0.074	0.034	8.5E-56	Skin_Fibro	TRAM2
RRAD3	5.93E-60	0.284772	0.072	0.032	9.46E-56	Skin_Fibro	RRAD
LUZP11	9.52E-60	0.441413	0.182	0.12	1.52E-55	Skin_Fibro	LUZP1
TBCB2	2.41E-59	0.337099	0.273	0.197	3.85E-55	Skin_Fibro	TBCB
HIVEP11	2.92E-59	0.291981	0.078	0.037	4.65E-55	Skin_Fibro	HIVEP1
SUMO3	2.92E-59	0.44651	0.282	0.217	4.66E-55	Skin_Fibro	SUMO3
PFN23	1.64E-58	0.434419	0.131	0.079	2.61E-54	Skin_Fibro	PFN2
FGFR1OP2	2.14E-58	0.436128	0.215	0.15	3.41E-54	Skin_Fibro	FGFR1OP2
DNAJB62	2.92E-58	0.286254	0.486	0.415	4.66E-54	Skin_Fibro	DNAJB6
YAF22	3E-58	0.447442	0.15	0.094	4.79E-54	Skin_Fibro	YAF2
SLC37A31	5.04E-58	0.304725	0.078	0.038	8.03E-54	Skin_Fibro	SLC37A3
STX41	7.9E-58	0.356822	0.156	0.097	1.26E-53	Skin_Fibro	STX4
AP1S23	8.68E-58	0.25043	0.19	0.121	1.38E-53	Skin_Fibro	AP1S2
SPTSSA1	9.44E-58	0.453829	0.307	0.241	1.51E-53	Skin_Fibro	SPTSSA
TMEM14C	1.54E-57	0.404621	0.315	0.252	2.45E-53	Skin_Fibro	TMEM14C
WDR83OS	1.95E-57	0.311263	0.428	0.364	3.11E-53	Skin_Fibro	WDR83OS
ZC3HAV13	1.97E-57	0.320548	0.152	0.093	3.15E-53	Skin_Fibro	ZC3HAV1
CAMLG1	3.01E-57	0.306371	0.319	0.244	4.81E-53	Skin_Fibro	CAMLG
RNF146	7.78E-57	0.399319	0.118	0.068	1.24E-52	Skin_Fibro	RNF146
IMPDH21	1.96E-56	0.327525	0.222	0.153	3.13E-52	Skin_Fibro	IMPDH2
GNA132	2.45E-56	0.329665	0.136	0.081	3.91E-52	Skin_Fibro	GNA13
FTO	5.53E-56	0.257053	0.072	0.034	8.82E-52	Skin_Fibro	FTO

MPZL17	2.15E-55	0.461612	0.194	0.137	3.43E-51	Skin_Fibro	MPZL1
LRRC582	3.7E-55	0.351863	0.12	0.07	5.91E-51	Skin_Fibro	LRRC58
NBPF1	4.47E-55	0.289839	0.072	0.034	7.13E-51	Skin_Fibro	NBPF1
RBM259	1.19E-54	0.446589	0.299	0.236	1.89E-50	Skin_Fibro	RBM25
GINM12	3.63E-54	0.442948	0.204	0.144	5.79E-50	Skin_Fibro	GINM1
PRCP2	8.61E-54	0.42416	0.179	0.122	1.37E-49	Skin_Fibro	PRCP
CRNDE4	2.42E-53	0.287777	0.215	0.151	3.87E-49	Skin_Fibro	CRNDE
MAOB2	2.67E-53	0.261335	0.05	0.02	4.26E-49	Skin_Fibro	MAOB
FOXO12	3.02E-53	0.268102	0.087	0.045	4.82E-49	Skin_Fibro	FOXO1
SEC24D3	5.02E-53	0.389397	0.138	0.086	8E-49	Skin_Fibro	SEC24D
LGALS3BP8	5.79E-53	0.361913	0.233	0.166	9.24E-49	Skin_Fibro	LGALS3BP
STK3	7.95E-53	0.398362	0.121	0.072	1.27E-48	Skin_Fibro	STK3
SPG211	1.33E-52	0.447047	0.212	0.153	2.12E-48	Skin_Fibro	SPG21
ING1	2.13E-52	0.369815	0.115	0.067	3.4E-48	Skin_Fibro	ING1
SPATS2L2	2.9E-52	0.586738	0.274	0.22	4.62E-48	Skin_Fibro	SPATS2L
KMT2A8	3.22E-52	0.439852	0.184	0.127	5.13E-48	Skin_Fibro	KMT2A
TAF4B	5.53E-52	0.269627	0.065	0.03	8.82E-48	Skin_Fibro	TAF4B
BCL7C3	5.99E-52	0.352404	0.172	0.114	9.56E-48	Skin_Fibro	BCL7C
RSL1D11	6.75E-52	0.31253	0.315	0.244	1.08E-47	Skin_Fibro	RSL1D1
SYPL16	6.93E-52	0.435531	0.318	0.26	1.11E-47	Skin_Fibro	SYPL1
ERGIC31	1.17E-51	0.318444	0.371	0.307	1.87E-47	Skin_Fibro	ERGIC3
KIF13A3	1.2E-51	0.337039	0.09	0.048	1.91E-47	Skin_Fibro	KIF13A
RNF1682	1.52E-51	0.401562	0.148	0.094	2.43E-47	Skin_Fibro	RNF168
ZNF5033	1.53E-51	0.322603	0.114	0.066	2.45E-47	Skin_Fibro	ZNF503
PDLIM37	1.68E-51	0.344018	0.1	0.055	2.68E-47	Skin_Fibro	PDLIM3
SERTAD13	1.9E-51	0.373472	0.295	0.226	3.04E-47	Skin_Fibro	SERTAD1
NOLC11	1.97E-51	0.364714	0.128	0.078	3.14E-47	Skin_Fibro	NOLC1
PHPT19	2.97E-51	0.292727	0.392	0.32	4.73E-47	Skin_Fibro	PHPT1
RARG1	4.04E-51	0.272309	0.106	0.059	6.45E-47	Skin_Fibro	RARG
INO80D1	6.44E-51	0.340343	0.105	0.059	1.03E-46	Skin_Fibro	INO80D
OSBPL83	6.6E-51	0.503279	0.23	0.173	1.05E-46	Skin_Fibro	OSBPL8
TRA2B2	8.26E-51	0.402155	0.346	0.285	1.32E-46	Skin_Fibro	TRA2B
A4GALT2	1.67E-50	0.342686	0.135	0.083	2.67E-46	Skin_Fibro	A4GALT
TMEM109	1.75E-50	0.296462	0.185	0.124	2.79E-46	Skin_Fibro	TMEM109
VIMP4	1.96E-50	0.302749	0.352	0.288	3.13E-46	Skin_Fibro	VIMP
DNAJC35	2.88E-50	0.517288	0.259	0.205	4.59E-46	Skin_Fibro	DNAJC3
MRFAP13	3.4E-50	0.357053	0.324	0.262	5.43E-46	Skin_Fibro	MRFAP1
REEP57	4.12E-50	0.266678	0.377	0.304	6.57E-46	Skin_Fibro	REEP5
COMT1	9.64E-50	0.400058	0.237	0.179	1.54E-45	Skin_Fibro	COMT
ENTPD15	1.79E-49	0.265865	0.084	0.044	2.86E-45	Skin_Fibro	ENTPD1
ASH1L5	2.74E-49	0.457099	0.222	0.166	4.37E-45	Skin_Fibro	ASH1L
SLIRP1	4.19E-49	0.423791	0.311	0.255	6.68E-45	Skin_Fibro	SLIRP
TMEM64	1.07E-48	0.264628	0.063	0.03	1.7E-44	Skin_Fibro	TMEM64
PRSS234	1.13E-48	0.337543	0.146	0.091	1.8E-44	Skin_Fibro	PRSS23
NCOA74	2.01E-48	0.351124	0.159	0.104	3.2E-44	Skin_Fibro	NCOA7
CHST122	3.32E-48	0.271043	0.129	0.078	5.3E-44	Skin_Fibro	CHST12
AMOTL12	6.34E-48	0.323751	0.095	0.054	1.01E-43	Skin_Fibro	AMOTL1

CDK42	1.65E-47	0.330072	0.164	0.11	2.63E-43	Skin_Fibro	CDK4
SPG202	2.03E-47	0.361987	0.142	0.091	3.23E-43	Skin_Fibro	SPG20
BST24	2.2E-47	0.365854	0.247	0.177	3.51E-43	Skin_Fibro	BST2
N4BP2L12	3.2E-47	0.266581	0.106	0.061	5.1E-43	Skin_Fibro	N4BP2L1
EML41	5.69E-47	0.461417	0.169	0.117	9.07E-43	Skin_Fibro	EML4
MAP4K51	6.83E-47	0.389233	0.132	0.084	1.09E-42	Skin_Fibro	MAP4K5
HDGF1	7.02E-47	0.440187	0.189	0.135	1.12E-42	Skin_Fibro	HDGF
PHACTR22	9.73E-47	0.393714	0.238	0.178	1.55E-42	Skin_Fibro	PHACTR2
MIR4458H	2.31E-46	0.291788	0.072	0.036	3.69E-42	Skin_Fibro	MIR4458HG
FADS32	2.78E-46	0.267801	0.076	0.039	4.44E-42	Skin_Fibro	FADS3
ATP5G17	3.85E-46	0.273511	0.32	0.257	6.14E-42	Skin_Fibro	ATP5G1
LMCD13	4.2E-46	0.253419	0.131	0.081	6.69E-42	Skin_Fibro	LMCD1
RNF138	4.63E-46	0.430165	0.218	0.164	7.38E-42	Skin_Fibro	RNF13
LHFPL24	1.95E-45	0.336162	0.09	0.051	3.11E-41	Skin_Fibro	LHFPL2
FOXN33	2.13E-45	0.40257	0.176	0.122	3.4E-41	Skin_Fibro	FOXN3
NFKBIE1	2.39E-45	0.38617	0.122	0.076	3.81E-41	Skin_Fibro	NFKBIE
TANK3	3.32E-45	0.385914	0.177	0.124	5.3E-41	Skin_Fibro	TANK
SLC39A73	3.82E-45	0.367018	0.161	0.11	6.1E-41	Skin_Fibro	SLC39A7
MAZ1	4.95E-45	0.269365	0.135	0.085	7.9E-41	Skin_Fibro	MAZ
PAFAH1B2	7.81E-45	0.461098	0.174	0.123	1.25E-40	Skin_Fibro	PAFAH1B2
PKIG4	1.27E-44	0.345033	0.146	0.096	2.02E-40	Skin_Fibro	PKIG
HIF1A4	3.6E-44	0.488869	0.214	0.163	5.74E-40	Skin_Fibro	HIF1A
HNRNPA35	3.8E-44	0.338554	0.412	0.362	6.07E-40	Skin_Fibro	HNRNPA3
NPM3	4.02E-44	0.280109	0.131	0.082	6.4E-40	Skin_Fibro	NPM3
HNRNPU2	4.82E-44	0.383326	0.394	0.346	7.69E-40	Skin_Fibro	HNRNPU
CLTB	5.29E-44	0.282667	0.422	0.36	8.44E-40	Skin_Fibro	CLTB
PAQR3	7.38E-44	0.266241	0.07	0.036	1.18E-39	Skin_Fibro	PAQR3
RBMX3	8.83E-44	0.304883	0.256	0.195	1.41E-39	Skin_Fibro	RBMX
LRRC16A1	1.54E-43	0.31531	0.073	0.038	2.46E-39	Skin_Fibro	LRRC16A
ZBTB21	2.04E-43	0.307264	0.082	0.045	3.25E-39	Skin_Fibro	ZBTB21
RFX21	2.08E-43	0.28169	0.084	0.046	3.32E-39	Skin_Fibro	RFX2
MESDC11	2.98E-43	0.324776	0.121	0.076	4.75E-39	Skin_Fibro	MESDC1
RAB8B3	4.58E-43	0.33362	0.144	0.094	7.3E-39	Skin_Fibro	RAB8B
PXK1	4.73E-43	0.251475	0.077	0.041	7.55E-39	Skin_Fibro	PXK
CERS2	5.05E-43	0.297179	0.126	0.08	8.05E-39	Skin_Fibro	CERS2
LINC00152	6.49E-43	0.37167	0.237	0.174	1.04E-38	Skin_Fibro	LINC00152
LRRC593	6.68E-43	0.43321	0.191	0.141	1.07E-38	Skin_Fibro	LRRC59
NPHP32	9.75E-43	0.288165	0.08	0.043	1.56E-38	Skin_Fibro	NPHP3
EIF58	1.3E-42	0.348085	0.405	0.357	2.07E-38	Skin_Fibro	EIF5
LRCH12	1.5E-42	0.25924	0.072	0.038	2.39E-38	Skin_Fibro	LRCH1
DCTD	1.76E-42	0.302203	0.123	0.077	2.81E-38	Skin_Fibro	DCTD
RYBP	2.1E-42	0.486644	0.192	0.143	3.36E-38	Skin_Fibro	RYBP
RANBP12	2.19E-42	0.283028	0.327	0.263	3.49E-38	Skin_Fibro	RANBP1
DDIT43	2.31E-42	0.312859	0.309	0.238	3.68E-38	Skin_Fibro	DDIT4
RAP2A3	3.07E-42	0.297719	0.079	0.043	4.9E-38	Skin_Fibro	RAP2A
RAB6A	6.08E-42	0.466627	0.216	0.167	9.7E-38	Skin_Fibro	RAB6A
GBE11	1.06E-41	0.31144	0.093	0.054	1.69E-37	Skin_Fibro	GBE1

NFKB23	1.27E-41	0.303082	0.12	0.075	2.03E-37	Skin_Fibro	NFKB2
FEZ22	1.29E-41	0.380663	0.155	0.107	2.05E-37	Skin_Fibro	FEZ2
ASXL12	1.61E-41	0.341158	0.12	0.076	2.56E-37	Skin_Fibro	ASXL1
RBM8A2	2.04E-41	0.364023	0.385	0.34	3.26E-37	Skin_Fibro	RBM8A
ST131	2.79E-41	0.28725	0.453	0.403	4.44E-37	Skin_Fibro	ST13
TTC38	4.31E-41	0.286449	0.291	0.23	6.88E-37	Skin_Fibro	TTC3
RBBP62	8.41E-41	0.390361	0.217	0.164	1.34E-36	Skin_Fibro	RBBP6
SLC30A73	1.19E-40	0.401813	0.123	0.08	1.9E-36	Skin_Fibro	SLC30A7
GDI22	1.36E-40	0.379033	0.313	0.265	2.17E-36	Skin_Fibro	GDI2
LPP4	2.77E-40	0.289058	0.266	0.208	4.42E-36	Skin_Fibro	LPP
CAMK2D1	3.45E-40	0.263754	0.096	0.057	5.51E-36	Skin_Fibro	CAMK2D
MED301	3.48E-40	0.330076	0.144	0.097	5.55E-36	Skin_Fibro	MED30
SLC25A441	3.96E-40	0.251906	0.073	0.039	6.31E-36	Skin_Fibro	SLC25A44
ZNF5161	4.11E-40	0.28576	0.077	0.042	6.55E-36	Skin_Fibro	ZNF516
SESTD12	4.25E-40	0.384199	0.129	0.085	6.78E-36	Skin_Fibro	SESTD1
DNAJB94	1.73E-39	0.360677	0.257	0.204	2.76E-35	Skin_Fibro	DNAJB9
C1orf212	1.82E-39	0.307269	0.146	0.098	2.9E-35	Skin_Fibro	C1orf21
METAP2	1.02E-38	0.393776	0.231	0.179	1.62E-34	Skin_Fibro	METAP2
NDUFA13	1.8E-38	0.258362	0.457	0.409	2.87E-34	Skin_Fibro	NDUFA1
PGAM14	2.62E-38	0.33501	0.276	0.226	4.18E-34	Skin_Fibro	PGAM1
ZNF7045	2.77E-38	0.347682	0.106	0.067	4.42E-34	Skin_Fibro	ZNF704
PHLDA32	3.59E-38	0.325202	0.163	0.114	5.73E-34	Skin_Fibro	PHLDA3
UBE2A2	3.65E-38	0.463339	0.255	0.21	5.82E-34	Skin_Fibro	UBE2A
DEXI1	3.82E-38	0.29256	0.112	0.071	6.09E-34	Skin_Fibro	DEXI
LRIG33	6.49E-38	0.271028	0.082	0.047	1.03E-33	Skin_Fibro	LRIG3
ALKBH51	8.08E-38	0.39179	0.174	0.127	1.29E-33	Skin_Fibro	ALKBH5
AHDC1	8.58E-38	0.298456	0.102	0.063	1.37E-33	Skin_Fibro	AHDC1
UBL3	9.11E-38	0.410534	0.288	0.241	1.45E-33	Skin_Fibro	UBL3
ARGLU13	1.28E-37	0.367021	0.353	0.306	2.05E-33	Skin_Fibro	ARGLU1
TP53I131	1.64E-37	0.271976	0.111	0.07	2.62E-33	Skin_Fibro	TP53I13
ACTN14	2.55E-37	0.326507	0.194	0.145	4.07E-33	Skin_Fibro	ACTN1
C2orf76	3.55E-37	0.257791	0.077	0.043	5.66E-33	Skin_Fibro	C2orf76
DDAH11	4.37E-37	0.325112	0.08	0.046	6.97E-33	Skin_Fibro	DDAH1
MTHFD24	4.71E-37	0.352688	0.141	0.096	7.51E-33	Skin_Fibro	MTHFD2
ELF2	5.06E-37	0.43534	0.168	0.122	8.07E-33	Skin_Fibro	ELF2
SMARCA23	5.89E-37	0.425165	0.155	0.111	9.4E-33	Skin_Fibro	SMARCA2
RSU12	7.31E-37	0.31178	0.168	0.12	1.17E-32	Skin_Fibro	RSU1
FBXL31	8.79E-37	0.379522	0.146	0.102	1.4E-32	Skin_Fibro	FBXL3
TMEM702	1.27E-36	0.351648	0.155	0.11	2.03E-32	Skin_Fibro	TMEM70
IAH12	2.49E-36	0.346508	0.208	0.157	3.97E-32	Skin_Fibro	IAH1
PCMT12	4.89E-36	0.28588	0.266	0.214	7.79E-32	Skin_Fibro	PCMT1
BNIP23	5.63E-36	0.348945	0.168	0.122	8.98E-32	Skin_Fibro	BNIP2
UBXN7	5.92E-36	0.339549	0.106	0.067	9.44E-32	Skin_Fibro	UBXN7
TRA2A3	1.79E-35	0.447015	0.208	0.162	2.85E-31	Skin_Fibro	TRA2A
NMD31	2.65E-35	0.309552	0.114	0.074	4.22E-31	Skin_Fibro	NMD3
LGR42	2.68E-35	0.271472	0.064	0.034	4.28E-31	Skin_Fibro	LGR4
LINC00998	3.32E-35	0.317979	0.103	0.065	5.29E-31	Skin_Fibro	LINC00998

RAB28	3.54E-35	0.263697	0.09	0.054	5.64E-31	Skin_Fibro	RAB28
KLHL211	4.04E-35	0.315645	0.115	0.075	6.44E-31	Skin_Fibro	KLHL21
YPEL22	5.45E-35	0.362553	0.145	0.102	8.69E-31	Skin_Fibro	YPEL2
TNRC6B7	6.97E-35	0.448214	0.216	0.169	1.11E-30	Skin_Fibro	TNRC6B
PLEKHA31	7.03E-35	0.435131	0.166	0.122	1.12E-30	Skin_Fibro	PLEKHA3
DEGS11	7.62E-35	0.327921	0.17	0.124	1.22E-30	Skin_Fibro	DEGS1
C1orf52	9.42E-35	0.340784	0.156	0.112	1.5E-30	Skin_Fibro	C1orf52
TM9SF34	1.1E-34	0.383712	0.277	0.233	1.76E-30	Skin_Fibro	TM9SF3
BANF11	1.32E-34	0.261013	0.284	0.23	2.11E-30	Skin_Fibro	BANF1
PSD31	1.88E-34	0.297234	0.124	0.082	3E-30	Skin_Fibro	PSD3
MED13	2.11E-34	0.458677	0.168	0.126	3.37E-30	Skin_Fibro	MED13
UBE2D11	2.93E-34	0.425441	0.227	0.179	4.67E-30	Skin_Fibro	UBE2D1
LONRF11	3.52E-34	0.327241	0.093	0.058	5.62E-30	Skin_Fibro	LONRF1
ATN11	4.28E-34	0.274127	0.082	0.049	6.83E-30	Skin_Fibro	ATN1
CD3023	4.47E-34	0.270407	0.099	0.062	7.13E-30	Skin_Fibro	CD302
IMPAD14	4.49E-34	0.400809	0.129	0.09	7.17E-30	Skin_Fibro	IMPAD1
HIPK32	6.94E-34	0.391383	0.16	0.116	1.11E-29	Skin_Fibro	HIPK3
SMURF21	9.98E-34	0.327148	0.09	0.055	1.59E-29	Skin_Fibro	SMURF2
CACYBP	1.27E-33	0.333662	0.273	0.227	2.03E-29	Skin_Fibro	CACYBP
AEBP21	1.5E-33	0.358624	0.096	0.061	2.39E-29	Skin_Fibro	AEBP2
TSHZ25	1.85E-33	0.333878	0.151	0.107	2.94E-29	Skin_Fibro	TSHZ2
ATP2B15	2.6E-33	0.263394	0.198	0.147	4.15E-29	Skin_Fibro	ATP2B1
ANXA73	3.46E-33	0.346422	0.238	0.193	5.51E-29	Skin_Fibro	ANXA7
TSPYL23	4.64E-33	0.256133	0.128	0.086	7.4E-29	Skin_Fibro	TSPYL2
SEPT25	1.06E-32	0.383093	0.274	0.234	1.69E-28	Skin_Fibro	SEPT2
CHORDC12	1.73E-32	0.321282	0.139	0.098	2.76E-28	Skin_Fibro	CHORDC1
DEK6	2.6E-32	0.320657	0.302	0.256	4.15E-28	Skin_Fibro	DEK
WAC1	3.37E-32	0.461972	0.249	0.21	5.37E-28	Skin_Fibro	WAC
HDAC92	6.1E-32	0.275534	0.07	0.04	9.73E-28	Skin_Fibro	HDAC9
TMX12	1.91E-31	0.372397	0.143	0.103	3.05E-27	Skin_Fibro	TMX1
SLC3A23	2.04E-31	0.287449	0.243	0.195	3.26E-27	Skin_Fibro	SLC3A2
GOLT1B1	2.24E-31	0.331551	0.133	0.094	3.58E-27	Skin_Fibro	GOLT1B
PQBP13	2.96E-31	0.273549	0.198	0.151	4.73E-27	Skin_Fibro	PQBP1
RNF53	4.14E-31	0.344859	0.184	0.14	6.6E-27	Skin_Fibro	RNF5
KMT2E4	6.01E-31	0.340279	0.337	0.297	9.58E-27	Skin_Fibro	KMT2E
NAA501	6.18E-31	0.474887	0.237	0.2	9.85E-27	Skin_Fibro	NAA50
HNRNPF1	6.47E-31	0.326379	0.345	0.306	1.03E-26	Skin_Fibro	HNRNPF
TERF2IP3	9.53E-31	0.314087	0.268	0.222	1.52E-26	Skin_Fibro	TERF2IP
RABGGTB3	1.25E-30	0.339149	0.138	0.099	1.99E-26	Skin_Fibro	RABGGTB
SLC25A365	1.4E-30	0.389983	0.161	0.121	2.24E-26	Skin_Fibro	SLC25A36
IMP32	2.24E-30	0.277858	0.232	0.184	3.57E-26	Skin_Fibro	IMP3
ZBTB102	2.68E-30	0.339005	0.109	0.073	4.27E-26	Skin_Fibro	ZBTB10
FBXO111	3.39E-30	0.375936	0.141	0.101	5.41E-26	Skin_Fibro	FBXO11
ENY24	4.17E-30	0.299889	0.355	0.32	6.66E-26	Skin_Fibro	ENY2
TOB11	6.26E-30	0.283942	0.326	0.28	9.98E-26	Skin_Fibro	TOB1
ARL35	9.55E-30	0.343042	0.152	0.113	1.52E-25	Skin_Fibro	ARL3
SCO2	1.06E-29	0.29587	0.127	0.089	1.69E-25	Skin_Fibro	SCO2

KHDRBS31	1.47E-29	0.335547	0.107	0.071	2.35E-25	Skin_Fibro	KHDRBS3
TNKS1BP1:	2.11E-29	0.286709	0.096	0.063	3.36E-25	Skin_Fibro	TNKS1BP1
LTBR4	2.36E-29	0.324191	0.123	0.086	3.77E-25	Skin_Fibro	LTBR
CD15110	2.9E-29	0.253359	0.265	0.217	4.62E-25	Skin_Fibro	CD151
EIF2AK32	5.12E-29	0.253125	0.072	0.043	8.16E-25	Skin_Fibro	EIF2AK3
RAP2C2	6.34E-29	0.284422	0.082	0.051	1.01E-24	Skin_Fibro	RAP2C
ARHGAP21	6.44E-29	0.316349	0.131	0.093	1.03E-24	Skin_Fibro	ARHGAP21
KDELR15	6.62E-29	0.292402	0.289	0.249	1.06E-24	Skin_Fibro	KDELR1
C4orf272	7.62E-29	0.269977	0.112	0.076	1.22E-24	Skin_Fibro	C4orf27
ASPH7	8.62E-29	0.351215	0.229	0.188	1.37E-24	Skin_Fibro	ASPH
BDH21	8.69E-29	0.26951	0.112	0.076	1.39E-24	Skin_Fibro	BDH2
PRDX35	2.48E-28	0.364116	0.193	0.154	3.96E-24	Skin_Fibro	PRDX3
ARID2	4.21E-28	0.304699	0.11	0.074	6.72E-24	Skin_Fibro	ARID2
TUSC32	4.5E-28	0.257927	0.088	0.057	7.18E-24	Skin_Fibro	TUSC3
TAF13	5.09E-28	0.272732	0.078	0.049	8.13E-24	Skin_Fibro	TAF13
ANO62	8.47E-28	0.291767	0.098	0.065	1.35E-23	Skin_Fibro	ANO6
ATP6V0A1	1.02E-27	0.250842	0.086	0.055	1.62E-23	Skin_Fibro	ATP6V0A1
C5orf242	1.03E-27	0.368509	0.147	0.11	1.65E-23	Skin_Fibro	C5orf24
POLR2E1	1.73E-27	0.278294	0.24	0.197	2.76E-23	Skin_Fibro	POLR2E
TCEA13	2.4E-27	0.376631	0.274	0.238	3.82E-23	Skin_Fibro	TCEA1
PPP3CB1	5.42E-27	0.299192	0.104	0.071	8.64E-23	Skin_Fibro	PPP3CB
TMCO12	6.83E-27	0.374428	0.265	0.23	1.09E-22	Skin_Fibro	TMCO1
SF14	7.56E-27	0.280864	0.273	0.232	1.21E-22	Skin_Fibro	SF1
DCP1A	7.99E-27	0.288656	0.111	0.077	1.27E-22	Skin_Fibro	DCP1A
RPS6KA36	8.03E-27	0.340441	0.13	0.093	1.28E-22	Skin_Fibro	RPS6KA3
HDAC2	8.62E-27	0.386709	0.222	0.184	1.38E-22	Skin_Fibro	HDAC2
CNOT41	9.43E-27	0.386757	0.157	0.12	1.5E-22	Skin_Fibro	CNOT4
IPO74	9.81E-27	0.377505	0.138	0.102	1.56E-22	Skin_Fibro	IPO7
PRKD31	1.26E-26	0.299281	0.111	0.076	2.02E-22	Skin_Fibro	PRKD3
PNKD2	2.57E-26	0.301042	0.23	0.191	4.09E-22	Skin_Fibro	PNKD
B3GNT22	2.59E-26	0.287908	0.1	0.067	4.13E-22	Skin_Fibro	B3GNT2
NAA151	3.41E-26	0.294404	0.107	0.073	5.44E-22	Skin_Fibro	NAA15
SMAD56	3.83E-26	0.346277	0.127	0.092	6.11E-22	Skin_Fibro	SMAD5
DRAM1	3.86E-26	0.298037	0.083	0.054	6.15E-22	Skin_Fibro	DRAM1
SYNC	4.77E-26	0.257711	0.073	0.045	7.6E-22	Skin_Fibro	SYNC
BAZ1A2	9.55E-26	0.367792	0.281	0.243	1.52E-21	Skin_Fibro	BAZ1A
VCL4	1.32E-25	0.294391	0.148	0.111	2.1E-21	Skin_Fibro	VCL
NACA23	1.44E-25	0.279874	0.104	0.07	2.3E-21	Skin_Fibro	NACA2
ZFAND11	4.01E-25	0.314198	0.148	0.111	6.4E-21	Skin_Fibro	ZFAND1
KIDINS220	7.08E-25	0.344296	0.135	0.1	1.13E-20	Skin_Fibro	KIDINS220
PAPD5	7.28E-25	0.314425	0.094	0.064	1.16E-20	Skin_Fibro	PAPD5
CDC37L11	9.49E-25	0.318924	0.114	0.082	1.51E-20	Skin_Fibro	CDC37L1
KCTD9	1E-24	0.322759	0.103	0.072	1.6E-20	Skin_Fibro	KCTD9
TCEB11	1.03E-24	0.309566	0.387	0.357	1.65E-20	Skin_Fibro	TCEB1
TCEA2	1.72E-24	0.285066	0.126	0.091	2.75E-20	Skin_Fibro	TCEA2
NPTN4	2.26E-24	0.399265	0.161	0.126	3.61E-20	Skin_Fibro	NPTN
VDAC13	2.9E-24	0.312865	0.274	0.24	4.62E-20	Skin_Fibro	VDAC1

RNF19A1	3.74E-24	0.376569	0.157	0.122	5.96E-20	Skin_Fibro	RNF19A
COPRS1	3.8E-24	0.332724	0.184	0.148	6.06E-20	Skin_Fibro	COPRS
GNG12	4.14E-24	0.363932	0.228	0.193	6.6E-20	Skin_Fibro	GNG12
IRF2BP24	5.29E-24	0.335176	0.289	0.257	8.44E-20	Skin_Fibro	IRF2BP2
SHFM11	6E-24	0.260219	0.451	0.43	9.58E-20	Skin_Fibro	SHFM1
JMJD1C2	6.31E-24	0.393744	0.264	0.233	1.01E-19	Skin_Fibro	JMJD1C
KLF111	6.88E-24	0.289875	0.099	0.069	1.1E-19	Skin_Fibro	KLF11
PLEKHF11	1.01E-23	0.255712	0.113	0.079	1.6E-19	Skin_Fibro	PLEKHF1
INSR5	1.34E-23	0.409783	0.162	0.128	2.14E-19	Skin_Fibro	INSR
IPMK	1.5E-23	0.256178	0.074	0.047	2.39E-19	Skin_Fibro	IPMK
UPF3A3	1.74E-23	0.311323	0.181	0.143	2.78E-19	Skin_Fibro	UPF3A
TFG2	1.91E-23	0.38108	0.216	0.186	3.05E-19	Skin_Fibro	TFG
HIVEP21	2.22E-23	0.310643	0.124	0.09	3.54E-19	Skin_Fibro	HIVEP2
MSL1	3.17E-23	0.329236	0.128	0.096	5.06E-19	Skin_Fibro	MSL1
NEK72	4.22E-23	0.318113	0.109	0.077	6.73E-19	Skin_Fibro	NEK7
SSR16	5.08E-23	0.320246	0.223	0.189	8.1E-19	Skin_Fibro	SSR1
SRSF92	7.79E-23	0.3085	0.375	0.353	1.24E-18	Skin_Fibro	SRSF9
RAB7A1	8.17E-23	0.269529	0.398	0.371	1.3E-18	Skin_Fibro	RAB7A
MAGED28	8.21E-23	0.299349	0.19	0.153	1.31E-18	Skin_Fibro	MAGED2
MKLN14	8.54E-23	0.366909	0.141	0.108	1.36E-18	Skin_Fibro	MKLN1
TAOK13	1.11E-22	0.351392	0.142	0.109	1.77E-18	Skin_Fibro	TAOK1
ERCC13	1.15E-22	0.303096	0.187	0.153	1.83E-18	Skin_Fibro	ERCC1
UGP2	1.26E-22	0.361717	0.321	0.299	2.02E-18	Skin_Fibro	UGP2
KHDRBS12	1.48E-22	0.29498	0.249	0.213	2.36E-18	Skin_Fibro	KHDRBS1
FOXK11	1.88E-22	0.260339	0.079	0.052	2.99E-18	Skin_Fibro	FOXK1
UBR5	1.96E-22	0.32191	0.129	0.096	3.13E-18	Skin_Fibro	UBR5
AZIN11	2.23E-22	0.413892	0.203	0.171	3.56E-18	Skin_Fibro	AZIN1
CTBP1	3.56E-22	0.286265	0.108	0.078	5.68E-18	Skin_Fibro	CTBP1
OS95	4.09E-22	0.341885	0.254	0.224	6.53E-18	Skin_Fibro	OS9
FAM120A6	5.6E-22	0.383247	0.178	0.146	8.94E-18	Skin_Fibro	FAM120A
RBM271	7.34E-22	0.285204	0.106	0.075	1.17E-17	Skin_Fibro	RBM27
IFNGR2	7.7E-22	0.33945	0.235	0.203	1.23E-17	Skin_Fibro	IFNGR2
SNHG184	8.23E-22	0.282214	0.088	0.061	1.31E-17	Skin_Fibro	SNHG18
PHF23	1.14E-21	0.354878	0.126	0.095	1.82E-17	Skin_Fibro	PHF23
RHOBTB37	1.43E-21	0.328701	0.149	0.117	2.28E-17	Skin_Fibro	RHOBTB3
KREMEN12	1.65E-21	0.251423	0.071	0.046	2.64E-17	Skin_Fibro	KREMEN1
SDHD1	1.69E-21	0.352514	0.243	0.214	2.69E-17	Skin_Fibro	SDHD
CHD23	3.29E-21	0.366106	0.238	0.205	5.25E-17	Skin_Fibro	CHD2
BMPR22	4.22E-21	0.316462	0.133	0.101	6.74E-17	Skin_Fibro	BMPR2
GPATCH81	7.67E-21	0.312349	0.13	0.099	1.22E-16	Skin_Fibro	GPATCH8
LDLR1	8.56E-21	0.326418	0.132	0.1	1.37E-16	Skin_Fibro	LDLR
NUDT35	1.04E-20	0.297456	0.132	0.101	1.66E-16	Skin_Fibro	NUDT3
RCN21	1.07E-20	0.288338	0.187	0.153	1.7E-16	Skin_Fibro	RCN2
SETD72	1.08E-20	0.253749	0.086	0.059	1.72E-16	Skin_Fibro	SETD7
SFT2D23	1.37E-20	0.362959	0.138	0.108	2.19E-16	Skin_Fibro	SFT2D2
AHSA11	1.74E-20	0.293266	0.176	0.143	2.78E-16	Skin_Fibro	AHSA1
RNF1872	2.38E-20	0.268603	0.157	0.124	3.8E-16	Skin_Fibro	RNF187

CBFB1	3.65E-20	0.307866	0.121	0.091	5.82E-16	Skin_Fibro	CBFB
NMB2	6.14E-20	0.357472	0.099	0.072	9.79E-16	Skin_Fibro	NMB
PPIL3	8.62E-20	0.250701	0.092	0.065	1.37E-15	Skin_Fibro	PPIL3
ARID4B2	8.99E-20	0.341883	0.27	0.24	1.43E-15	Skin_Fibro	ARID4B
MEAF61	1.15E-19	0.276762	0.255	0.223	1.84E-15	Skin_Fibro	MEAF6
SRRM15	1.58E-19	0.317899	0.346	0.326	2.52E-15	Skin_Fibro	SRRM1
AGO33	1.73E-19	0.277937	0.103	0.075	2.77E-15	Skin_Fibro	AGO3
ZFP91	2.26E-19	0.375074	0.119	0.091	3.61E-15	Skin_Fibro	ZFP91
MRPL321	2.58E-19	0.317892	0.166	0.135	4.12E-15	Skin_Fibro	MRPL32
RYK5	3.81E-19	0.28835	0.1	0.073	6.08E-15	Skin_Fibro	RYK
LSM55	5.78E-19	0.3029	0.23	0.2	9.21E-15	Skin_Fibro	LSM5
RAD23B	8.14E-19	0.267679	0.222	0.19	1.3E-14	Skin_Fibro	RAD23B
AHCYL14	9.06E-19	0.398352	0.19	0.164	1.45E-14	Skin_Fibro	AHCYL1
ZFYVE215	3.53E-18	0.360874	0.175	0.147	5.62E-14	Skin_Fibro	ZFYVE21
SERTAD31	5.38E-18	0.306809	0.152	0.122	8.58E-14	Skin_Fibro	SERTAD3
MANBAL1	5.96E-18	0.292457	0.12	0.092	9.5E-14	Skin_Fibro	MANBAL
TCTN1	6.73E-18	0.278439	0.087	0.062	1.07E-13	Skin_Fibro	TCTN1
PRKRA1	6.73E-18	0.283856	0.154	0.124	1.07E-13	Skin_Fibro	PRKRA
CBX33	8.38E-18	0.260509	0.232	0.2	1.34E-13	Skin_Fibro	CBX3
STX8	8.78E-18	0.255916	0.19	0.158	1.4E-13	Skin_Fibro	STX8
PAIP1	1.15E-17	0.339298	0.177	0.15	1.83E-13	Skin_Fibro	PAIP1
FNIP13	1.39E-17	0.301165	0.11	0.083	2.21E-13	Skin_Fibro	FNIP1
ATL32	1.53E-17	0.269999	0.148	0.118	2.44E-13	Skin_Fibro	ATL3
STAG12	1.64E-17	0.261108	0.095	0.07	2.61E-13	Skin_Fibro	STAG1
MYCBP24	1.67E-17	0.254286	0.174	0.143	2.67E-13	Skin_Fibro	MYCBP2
PNPLA81	1.83E-17	0.310654	0.132	0.104	2.93E-13	Skin_Fibro	PNPLA8
GMFB	2.47E-17	0.263215	0.09	0.065	3.94E-13	Skin_Fibro	GMFB
CCDC71L2	2.56E-17	0.284587	0.089	0.064	4.09E-13	Skin_Fibro	CCDC71L
RQCD12	2.69E-17	0.259392	0.09	0.065	4.3E-13	Skin_Fibro	RQCD1
PFKFB32	2.88E-17	0.266177	0.123	0.094	4.59E-13	Skin_Fibro	PFKFB3
FAM76A	5.64E-17	0.278284	0.081	0.058	8.99E-13	Skin_Fibro	FAM76A
NCOA13	6.48E-17	0.259313	0.114	0.088	1.03E-12	Skin_Fibro	NCOA1
COPS61	7.82E-17	0.273429	0.236	0.21	1.25E-12	Skin_Fibro	COPS6
AK12	8.85E-17	0.265159	0.17	0.141	1.41E-12	Skin_Fibro	AK1
FNIP22	9.81E-17	0.313349	0.115	0.089	1.56E-12	Skin_Fibro	FNIP2
RBM17	9.84E-17	0.269849	0.209	0.179	1.57E-12	Skin_Fibro	RBM17
GOLPH31	1.07E-16	0.388161	0.193	0.169	1.71E-12	Skin_Fibro	GOLPH3
PIM33	1.32E-16	0.275	0.314	0.29	2.1E-12	Skin_Fibro	PIM3
CHMP31	1.39E-16	0.281719	0.195	0.167	2.21E-12	Skin_Fibro	CHMP3
BNIP3L3	1.69E-16	0.31879	0.31	0.296	2.69E-12	Skin_Fibro	BNIP3L
BOD1	2.15E-16	0.262754	0.129	0.102	3.42E-12	Skin_Fibro	BOD1
GCSH5	8.4E-16	0.360669	0.149	0.125	1.34E-11	Skin_Fibro	GCSH
SCAF118	1E-15	0.33611	0.252	0.23	1.6E-11	Skin_Fibro	SCAF11
PRPF38B3	1.06E-15	0.260242	0.22	0.191	1.69E-11	Skin_Fibro	PRPF38B
METTL153	1.18E-15	0.29682	0.068	0.047	1.88E-11	Skin_Fibro	METTL15
SELT4	1.55E-15	0.290752	0.249	0.226	2.48E-11	Skin_Fibro	SELT
ZSWIM61	1.7E-15	0.265775	0.089	0.066	2.72E-11	Skin_Fibro	ZSWIM6

FGD5.AS12	1.98E-15	0.293436	0.134	0.108	3.16E-11	Skin_Fibro	FGD5.AS1
PPP1R14B9	3.47E-15	0.350889	0.2	0.176	5.54E-11	Skin_Fibro	PPP1R14B
RTF1	3.78E-15	0.355191	0.263	0.245	6.03E-11	Skin_Fibro	RTF1
GUSB1	4.41E-15	0.31605	0.16	0.136	7.03E-11	Skin_Fibro	GUSB
C4orf323	5.49E-15	0.297318	0.105	0.08	8.75E-11	Skin_Fibro	C4orf32
POGZ2	6.25E-15	0.281577	0.112	0.088	9.97E-11	Skin_Fibro	POGZ
ZFHX35	6.97E-15	0.277921	0.113	0.088	1.11E-10	Skin_Fibro	ZFHX3
RNF1153	1.44E-14	0.272263	0.13	0.105	2.3E-10	Skin_Fibro	RNF115
ADNP1	1.77E-14	0.300091	0.116	0.092	2.83E-10	Skin_Fibro	ADNP
C1orf564	2.22E-14	0.311566	0.147	0.12	3.53E-10	Skin_Fibro	C1orf56
DHX151	2.79E-14	0.312589	0.135	0.111	4.46E-10	Skin_Fibro	DHX15
FAM204A	6.12E-14	0.290595	0.188	0.162	9.76E-10	Skin_Fibro	FAM204A
ELF13	8.63E-14	0.29932	0.201	0.177	1.38E-09	Skin_Fibro	ELF1
CREB1	1E-13	0.288724	0.149	0.125	1.6E-09	Skin_Fibro	CREB1
KCNQ1OT1	1.02E-13	0.252888	0.166	0.14	1.63E-09	Skin_Fibro	KCNQ1OT1
CCNG1	1.21E-13	0.259253	0.181	0.157	1.93E-09	Skin_Fibro	CCNG1
MAFG1	1.27E-13	0.333749	0.147	0.123	2.03E-09	Skin_Fibro	MAFG
DNAJC8	1.6E-13	0.261098	0.242	0.218	2.55E-09	Skin_Fibro	DNAJC8
CYLD3	1.72E-13	0.306992	0.148	0.124	2.74E-09	Skin_Fibro	CYLD
NCKAP12	1.73E-13	0.367122	0.17	0.148	2.76E-09	Skin_Fibro	NCKAP1
CANX8	1.75E-13	0.259685	0.324	0.312	2.79E-09	Skin_Fibro	CANX
TAF1D3	1.89E-13	0.260088	0.249	0.226	3.01E-09	Skin_Fibro	TAF1D
NONO1	2.16E-13	0.292688	0.212	0.19	3.45E-09	Skin_Fibro	NONO
FOXO32	2.45E-13	0.370269	0.192	0.172	3.91E-09	Skin_Fibro	FOXO3
TM2D3	2.46E-13	0.27883	0.135	0.112	3.92E-09	Skin_Fibro	TM2D3
ATF2	2.49E-13	0.276991	0.097	0.076	3.98E-09	Skin_Fibro	ATF2
LTBP34	2.87E-13	0.269433	0.173	0.15	4.58E-09	Skin_Fibro	LTBP3
MOCS2	3.07E-13	0.312036	0.122	0.1	4.9E-09	Skin_Fibro	MOCS2
ANG6	3.19E-13	0.255298	0.122	0.099	5.09E-09	Skin_Fibro	ANG
SARS2	4.78E-13	0.253365	0.179	0.155	7.63E-09	Skin_Fibro	SARS
UBE2Q21	4.97E-13	0.314505	0.138	0.116	7.92E-09	Skin_Fibro	UBE2Q2
VGLL44	7.48E-13	0.281092	0.157	0.134	1.19E-08	Skin_Fibro	VGLL4
SRSF12	1.14E-12	0.279761	0.175	0.153	1.82E-08	Skin_Fibro	SRSF1
MGAT13	1.22E-12	0.259922	0.232	0.212	1.94E-08	Skin_Fibro	MGAT1
CAMTA11	1.5E-12	0.30722	0.337	0.334	2.4E-08	Skin_Fibro	CAMTA1
PSMB54	1.52E-12	0.284608	0.254	0.24	2.43E-08	Skin_Fibro	PSMB5
MED13L	1.63E-12	0.331076	0.152	0.13	2.6E-08	Skin_Fibro	MED13L
WBP42	2.29E-12	0.300619	0.117	0.096	3.66E-08	Skin_Fibro	WBP4
TCEAL31	3.81E-12	0.277274	0.113	0.092	6.07E-08	Skin_Fibro	TCEAL3
HYI5	3.88E-12	0.263611	0.106	0.085	6.19E-08	Skin_Fibro	HYI
ANKRD111	4.16E-12	0.340005	0.206	0.188	6.64E-08	Skin_Fibro	ANKRD11
EIF2S31	4.2E-12	0.286842	0.178	0.155	6.7E-08	Skin_Fibro	EIF2S3
PPIL44	5.52E-12	0.26415	0.121	0.099	8.8E-08	Skin_Fibro	PPIL4
MORC31	6.04E-12	0.286895	0.11	0.089	9.63E-08	Skin_Fibro	MORC3
CELF12	6.47E-12	0.276552	0.146	0.124	1.03E-07	Skin_Fibro	CELF1
SUMF23	8.4E-12	0.259445	0.135	0.114	1.34E-07	Skin_Fibro	SUMF2
ZNF667.AS	1.1E-11	0.275644	0.123	0.101	1.75E-07	Skin_Fibro	ZNF667.AS1

GOPC2	1.14E-11	0.274373	0.121	0.1	1.82E-07	Skin_Fibro	GOPC
AKAP135	1.41E-11	0.259221	0.235	0.216	2.26E-07	Skin_Fibro	AKAP13
CMTM62	1.71E-11	0.258964	0.245	0.227	2.72E-07	Skin_Fibro	CMTM6
COPS2	1.71E-11	0.292286	0.145	0.124	2.73E-07	Skin_Fibro	COPS2
MAP3K26	1.31E-10	0.280568	0.143	0.123	2.08E-06	Skin_Fibro	MAP3K2
VEZF13	1.41E-10	0.262293	0.106	0.086	2.24E-06	Skin_Fibro	VEZF1
ACBD32	1.47E-10	0.330193	0.14	0.122	2.34E-06	Skin_Fibro	ACBD3
HNRNPD3	1.64E-10	0.298261	0.252	0.24	2.61E-06	Skin_Fibro	HNRNPD
WDR451	2.05E-10	0.256088	0.157	0.137	3.27E-06	Skin_Fibro	WDR45
SLC25A26	3.01E-10	0.272179	0.105	0.086	4.8E-06	Skin_Fibro	SLC25A26
TROVE21	6.97E-10	0.260888	0.153	0.134	1.11E-05	Skin_Fibro	TROVE2
ZNF451	8.58E-10	0.268888	0.123	0.104	1.37E-05	Skin_Fibro	ZNF451
TGOLN28	9.99E-10	0.265592	0.188	0.171	1.59E-05	Skin_Fibro	TGOLN2
INTS66	1.02E-09	0.25005	0.155	0.136	1.62E-05	Skin_Fibro	INTS6
SPOP2	1.06E-09	0.251541	0.132	0.113	1.69E-05	Skin_Fibro	SPOP
KHSRP	1.18E-09	0.257645	0.102	0.084	1.89E-05	Skin_Fibro	KHSRP
TMEM167/	1.82E-09	0.333423	0.242	0.236	2.9E-05	Skin_Fibro	TMEM167A
MEIS24	2.01E-09	0.323591	0.119	0.102	3.2E-05	Skin_Fibro	MEIS2
PJA210	2.14E-09	0.251748	0.168	0.15	3.41E-05	Skin_Fibro	PJA2
TRIP62	3.04E-09	0.25551	0.156	0.139	4.85E-05	Skin_Fibro	TRIP6
ZBTB441	3.3E-09	0.315586	0.12	0.103	5.27E-05	Skin_Fibro	ZBTB44
EI241	4.09E-09	0.322621	0.199	0.187	6.52E-05	Skin_Fibro	EI24
CDC272	4.94E-09	0.251643	0.092	0.076	7.88E-05	Skin_Fibro	CDC27
IRS2	1.07E-08	0.304734	0.121	0.105	0.00017	Skin_Fibro	IRS2
KLF3	1.15E-08	0.289939	0.203	0.189	0.000184	Skin_Fibro	KLF3
UBE2H1	1.18E-08	0.30999	0.16	0.146	0.000189	Skin_Fibro	UBE2H
EPS152	2.45E-08	0.278138	0.125	0.109	0.000391	Skin_Fibro	EPS15
UBE2E12	2.5E-08	0.255431	0.153	0.138	0.000399	Skin_Fibro	UBE2E1
ATG121	2.8E-08	0.306749	0.179	0.166	0.000447	Skin_Fibro	ATG12
ZC3H11A3	6.5E-08	0.303081	0.178	0.166	0.001036	Skin_Fibro	ZC3H11A
PITPNB1	8.64E-08	0.289998	0.132	0.117	0.001379	Skin_Fibro	PITPNB
OTUD4	1E-07	0.29127	0.098	0.084	0.001598	Skin_Fibro	OTUD4
PPP2R5E	1.1E-07	0.266851	0.128	0.113	0.001761	Skin_Fibro	PPP2R5E
PCBD14	3.42E-07	0.258574	0.17	0.158	0.005449	Skin_Fibro	PCBD1
PPP4R2	3.52E-07	0.285376	0.14	0.127	0.005618	Skin_Fibro	PPP4R2
CLDND12	4.04E-07	0.250194	0.203	0.191	0.006449	Skin_Fibro	CLDND1
STAU1	4.38E-07	0.327034	0.211	0.205	0.006982	Skin_Fibro	STAU1
SPIN1	8.59E-07	0.257429	0.11	0.097	0.013702	Skin_Fibro	SPIN1
NDUFA53	1.05E-06	0.251084	0.254	0.252	0.016786	Skin_Fibro	NDUFA5
RBBP42	1.6E-06	0.294915	0.16	0.149	0.025472	Skin_Fibro	RBBP4
PPP2CA2	1.7E-06	0.258573	0.238	0.233	0.027157	Skin_Fibro	PPP2CA
GSPT12	1.86E-06	0.275826	0.183	0.174	0.029649	Skin_Fibro	GSPT1
UFM12	3.97E-06	0.282956	0.232	0.231	0.063332	Skin_Fibro	UFM1
ABCA5	4.4E-06	0.263284	0.115	0.103	0.07014	Skin_Fibro	ABCA5
ACADVL2	5.27E-06	0.282956	0.233	0.232	0.08399	Skin_Fibro	ACADVL
CLOCK	6.91E-06	0.28476	0.081	0.07	0.110179	Skin_Fibro	CLOCK
PPP2CB	8.42E-06	0.314192	0.17	0.163	0.134277	Skin_Fibro	PPP2CB

EIF3A3	9.44E-06	0.263608	0.29	0.294	0.150483	Skin_Fibro	EIF3A
TMED72	1.38E-05	0.291018	0.145	0.136	0.219377	Skin_Fibro	TMED7
SCARB29	4.82E-05	0.276296	0.163	0.157	0.769148	Skin_Fibro	SCARB2
PVRL22	5.64E-05	0.266024	0.188	0.182	0.898852	Skin_Fibro	PVRL2
BRD41	6.12E-05	0.270909	0.171	0.164	0.976399	Skin_Fibro	BRD4
C16orf72	0.000111	0.27429	0.167	0.16	1	Skin_Fibro	C16orf72
RNF1492	0.00013	0.263516	0.166	0.159	1	Skin_Fibro	RNF149
TNPO13	0.000212	0.257489	0.133	0.126	1	Skin_Fibro	TNPO1
RNF11	0.000587	0.260251	0.208	0.209	1	Skin_Fibro	RNF11
GNAQ6	0.000998	0.267389	0.167	0.164	1	Skin_Fibro	GNAQ
RSF14	0.001003	0.250098	0.184	0.182	1	Skin_Fibro	RSF1
SREK12	0.00103	0.257706	0.142	0.136	1	Skin_Fibro	SREK1
RAB142	0.001377	0.258926	0.188	0.188	1	Skin_Fibro	RAB14
BROX	0.001384	0.255818	0.115	0.109	1	Skin_Fibro	BROX
CTBP23	0.001426	0.265565	0.109	0.103	1	Skin_Fibro	CTBP2
BIRC21	0.008037	0.253016	0.153	0.152	1	Skin_Fibro	BIRC2
KRT143	0	6.099064	0.98	0.122	0	Skin_Kerat	KRT14
SFN	0	5.46145	0.999	0.12	0	Skin_Kerat	SFN
KRT52	0	4.614943	0.885	0.053	0	Skin_Kerat	KRT5
DMKN1	0	4.587826	0.874	0.134	0	Skin_Kerat	DMKN
S100A21	0	4.096581	0.919	0.166	0	Skin_Kerat	S100A2
KRTDAP	0	4.088045	0.654	0.047	0	Skin_Kerat	KRTDAP
KRT10	0	3.857951	0.868	0.399	0	Skin_Kerat	KRT10
PERP4	0	3.449633	0.992	0.347	0	Skin_Kerat	PERP
AQP32	0	3.324878	0.944	0.168	0	Skin_Kerat	AQP3
DST7	0	3.251764	0.81	0.164	0	Skin_Kerat	DST
LYPD31	0	3.243286	0.75	0.066	0	Skin_Kerat	LYPD3
DSP3	0	2.994906	0.909	0.15	0	Skin_Kerat	DSP
RNF144B2	0	2.936211	0.729	0.067	0	Skin_Kerat	RNF144B
ERRFI11	0	2.553613	0.793	0.151	0	Skin_Kerat	ERRFI1
LGALS7	0	2.407715	0.497	0.003	0	Skin_Kerat	LGALS7
MYC2	0	2.35989	0.797	0.108	0	Skin_Kerat	MYC
AREG1	0	2.318378	0.495	0.114	0	Skin_Kerat	AREG
SLC38A25	0	2.317882	0.901	0.22	0	Skin_Kerat	SLC38A2
RPL414	0	2.264642	1	0.857	0	Skin_Kerat	RPL41
COL17A12	0	1.98574	0.509	0.018	0	Skin_Kerat	COL17A1
LDHA5	0	1.959507	0.958	0.423	0	Skin_Kerat	LDHA
MPZL21	0	1.934203	0.702	0.078	0	Skin_Kerat	MPZL2
IRF61	0	1.933737	0.712	0.062	0	Skin_Kerat	IRF6
CSNK1A16	0	1.921336	0.875	0.252	0	Skin_Kerat	CSNK1A1
NDRG13	0	1.916616	0.728	0.125	0	Skin_Kerat	NDRG1
RPLP17	0	1.8811	1	0.913	0	Skin_Kerat	RPLP1
TNFRSF12/	0	1.852148	0.74	0.107	0	Skin_Kerat	TNFRSF12A
EMP23	0	1.843241	0.708	0.174	0	Skin_Kerat	EMP2
H2AFZ4	0	1.808914	0.922	0.424	0	Skin_Kerat	H2AFZ
TNFAIP34	0	1.744847	0.73	0.143	0	Skin_Kerat	TNFAIP3
DDX214	0	1.743927	0.799	0.18	0	Skin_Kerat	DDX21

TACSTD21	0	1.711475	0.932	0.307	0	Skin_Kerat	TACSTD2
RPS184	0	1.710535	1	0.914	0	Skin_Kerat	RPS18
RPL3913	0	1.690211	0.997	0.667	0	Skin_Kerat	RPL39
PMAIP11	0	1.679975	0.797	0.188	0	Skin_Kerat	PMAIP1
DRAP13	0	1.673303	0.873	0.318	0	Skin_Kerat	DRAP1
MT1X2	0	1.663022	0.83	0.18	0	Skin_Kerat	MT1X
ADRB21	0	1.64633	0.555	0.028	0	Skin_Kerat	ADRB2
PFDN24	0	1.640477	0.835	0.257	0	Skin_Kerat	PFDN2
EIF4A14	0	1.63693	0.94	0.334	0	Skin_Kerat	EIF4A1
RTN45	0	1.624637	0.923	0.378	0	Skin_Kerat	RTN4
TP632	0	1.622129	0.552	0.006	0	Skin_Kerat	TP63
CD552	0	1.621577	0.811	0.249	0	Skin_Kerat	CD55
DAAM12	0	1.615005	0.684	0.152	0	Skin_Kerat	DAAM1
SDC41	0	1.612205	0.67	0.15	0	Skin_Kerat	SDC4
MAST41	0	1.607826	0.664	0.115	0	Skin_Kerat	MAST4
NBEAL14	0	1.603991	0.908	0.301	0	Skin_Kerat	NBEAL1
CYR615	0	1.598179	0.562	0.099	0	Skin_Kerat	CYR61
PPP1R14C	0	1.581857	0.608	0.049	0	Skin_Kerat	PPP1R14C
KLF5	0	1.568879	0.768	0.204	0	Skin_Kerat	KLF5
HNRNPH15	0	1.561695	0.782	0.282	0	Skin_Kerat	HNRNPH1
LIMA15	0	1.554199	0.685	0.166	0	Skin_Kerat	LIMA1
IGFBP31	0	1.55091	0.453	0.054	0	Skin_Kerat	IGFBP3
TRIM292	0	1.542799	0.645	0.083	0	Skin_Kerat	TRIM29
ZFP36L15	0	1.529265	0.894	0.455	0	Skin_Kerat	ZFP36L1
CAV26	0	1.523445	0.656	0.102	0	Skin_Kerat	CAV2
ZFP36L26	0	1.501697	0.906	0.376	0	Skin_Kerat	ZFP36L2
RPS194	0	1.498401	1	0.871	0	Skin_Kerat	RPS19
CAV16	0	1.495583	0.799	0.179	0	Skin_Kerat	CAV1
SLC2A1	0	1.495409	0.544	0.045	0	Skin_Kerat	SLC2A1
NFKBIA4	0	1.493796	0.931	0.503	0	Skin_Kerat	NFKBIA
FOSL11	0	1.492654	0.612	0.066	0	Skin_Kerat	FOSL1
ETS23	0	1.481355	0.729	0.139	0	Skin_Kerat	ETS2
BTG15	0	1.472506	0.974	0.565	0	Skin_Kerat	BTG1
S100A161	0	1.471129	0.832	0.264	0	Skin_Kerat	S100A16
ARID5B6	0	1.458418	0.77	0.253	0	Skin_Kerat	ARID5B
RPL363	0	1.454666	0.999	0.836	0	Skin_Kerat	RPL36
EPPK11	0	1.437455	0.394	0.016	0	Skin_Kerat	EPPK1
SET7	0	1.436898	0.797	0.295	0	Skin_Kerat	SET
CXADR1	0	1.43095	0.664	0.151	0	Skin_Kerat	CXADR
CD446	0	1.430226	0.915	0.33	0	Skin_Kerat	CD44
EZR4	0	1.413386	0.814	0.265	0	Skin_Kerat	EZR
RPS4Y17	0	1.410802	0.856	0.254	0	Skin_Kerat	RPS4Y1
PTP4A12	0	1.402143	0.816	0.268	0	Skin_Kerat	PTP4A1
EIF3E6	0	1.375194	0.924	0.434	0	Skin_Kerat	EIF3E
TUBB2A5	0	1.368364	0.614	0.097	0	Skin_Kerat	TUBB2A
RPL345	0	1.359866	1	0.916	0	Skin_Kerat	RPL34
UBR41	0	1.352946	0.52	0.074	0	Skin_Kerat	UBR4

RPS286	0	1.352753	0.999	0.878	0	Skin_Kerat	RPS28
RPL74	0	1.344179	0.999	0.791	0	Skin_Kerat	RPL7
PTMA5	0	1.343902	0.999	0.814	0	Skin_Kerat	PTMA
DSG3	0	1.338993	0.476	0.005	0	Skin_Kerat	DSG3
RSL24D14	0	1.320842	0.819	0.29	0	Skin_Kerat	RSL24D1
CLTB1	0	1.31233	0.851	0.348	0	Skin_Kerat	CLTB
CHIC21	0	1.29817	0.66	0.172	0	Skin_Kerat	CHIC2
DNAJB63	0	1.292742	0.875	0.405	0	Skin_Kerat	DNAJB6
ANXA23	0	1.284483	0.987	0.585	0	Skin_Kerat	ANXA2
KLF44	0	1.261314	0.704	0.211	0	Skin_Kerat	KLF4
GNAI12	0	1.259283	0.543	0.046	0	Skin_Kerat	GNAI1
DEGS12	0	1.25587	0.569	0.112	0	Skin_Kerat	DEGS1
RPS84	0	1.254229	0.998	0.845	0	Skin_Kerat	RPS8
RORA4	0	1.251801	0.584	0.106	0	Skin_Kerat	RORA
SPTSSA2	0	1.25088	0.721	0.23	0	Skin_Kerat	SPTSSA
RPL234	0	1.244382	0.988	0.674	0	Skin_Kerat	RPL23
CALM27	0	1.243674	0.979	0.549	0	Skin_Kerat	CALM2
TPPP35	0	1.230069	0.598	0.093	0	Skin_Kerat	TPPP3
SOX15	0	1.228756	0.46	0.027	0	Skin_Kerat	SOX15
FXYD38	0	1.226912	0.823	0.214	0	Skin_Kerat	FXYD3
SERBP13	0	1.22502	0.83	0.339	0	Skin_Kerat	SERBP1
GADD45A2	0	1.217985	0.658	0.2	0	Skin_Kerat	GADD45A
KTN12	0	1.21682	0.847	0.383	0	Skin_Kerat	KTN1
RPS154	0	1.21668	1	0.877	0	Skin_Kerat	RPS15
PRNP7	0	1.215791	0.653	0.144	0	Skin_Kerat	PRNP
RAB101	0	1.204156	0.7	0.236	0	Skin_Kerat	RAB10
PHLDB24	0	1.20077	0.444	0.04	0	Skin_Kerat	PHLDB2
YWHAG3	0	1.200663	0.691	0.215	0	Skin_Kerat	YWHAG
YWHAZ3	0	1.199176	0.93	0.496	0	Skin_Kerat	YWHAZ
FHL21	0	1.194608	0.647	0.144	0	Skin_Kerat	FHL2
PHLDA23	0	1.191265	0.634	0.194	0	Skin_Kerat	PHLDA2
SLC25A54	0	1.187712	0.822	0.355	0	Skin_Kerat	SLC25A5
CA122	0	1.186744	0.483	0.041	0	Skin_Kerat	CA12
VAPA4	0	1.180098	0.848	0.386	0	Skin_Kerat	VAPA
NOP162	0	1.16645	0.53	0.057	0	Skin_Kerat	NOP16
MARCKS6	0	1.160967	0.762	0.268	0	Skin_Kerat	MARCKS
BTG33	0	1.146908	0.613	0.145	0	Skin_Kerat	BTG3
RPL354	0	1.146729	0.998	0.831	0	Skin_Kerat	RPL35
HAS3	0	1.145451	0.352	0.007	0	Skin_Kerat	HAS3
CEBPB3	0	1.142111	0.824	0.255	0	Skin_Kerat	CEBPB
REL5	0	1.140412	0.708	0.169	0	Skin_Kerat	REL
RPS295	0	1.139087	0.991	0.784	0	Skin_Kerat	RPS29
NRARP4	0	1.137499	0.385	0.029	0	Skin_Kerat	NRARP
RPS53	0	1.128752	0.997	0.779	0	Skin_Kerat	RPS5
MAL22	0	1.125462	0.501	0.125	0	Skin_Kerat	MAL2
RPL37A4	0	1.12207	0.997	0.851	0	Skin_Kerat	RPL37A
RPS275	0	1.112512	1	0.891	0	Skin_Kerat	RPS27

EIF1AX4	0	1.109012	0.751	0.324	0	Skin_Kerat	EIF1AX
EGLN3	0	1.107539	0.496	0.078	0	Skin_Kerat	EGLN3
SIK11	0	1.105778	0.612	0.134	0	Skin_Kerat	SIK1
KLC3	0	1.095481	0.444	0.024	0	Skin_Kerat	KLC3
EIF4E2	0	1.090902	0.731	0.228	0	Skin_Kerat	EIF4E
ATP1B34	0	1.08959	0.711	0.221	0	Skin_Kerat	ATP1B3
ZNF904	0	1.086444	0.523	0.081	0	Skin_Kerat	ZNF90
CYCS5	0	1.085328	0.839	0.326	0	Skin_Kerat	CYCS
RPS214	0	1.079362	0.995	0.662	0	Skin_Kerat	RPS21
HNRNPAB6	0	1.078074	0.588	0.153	0	Skin_Kerat	HNRNPAB
ARL4D3	0	1.057019	0.487	0.082	0	Skin_Kerat	ARL4D
WEE1	0	1.053912	0.461	0.084	0	Skin_Kerat	WEE1
BAZ1A3	0	1.045425	0.683	0.231	0	Skin_Kerat	BAZ1A
U2AF14	0	1.042939	0.737	0.24	0	Skin_Kerat	U2AF1
YWHAE3	0	1.042252	0.806	0.385	0	Skin_Kerat	YWHAE
RPS145	0	1.039739	1	0.879	0	Skin_Kerat	RPS14
AMD14	0	1.034949	0.626	0.18	0	Skin_Kerat	AMD1
NDFIP2	0	1.029302	0.699	0.226	0	Skin_Kerat	NDFIP2
ARF62	0	1.015926	0.821	0.362	0	Skin_Kerat	ARF6
NCL8	0	1.011609	0.731	0.256	0	Skin_Kerat	NCL
FBXW71	0	1.009711	0.479	0.085	0	Skin_Kerat	FBXW7
FSCN13	0	1.006401	0.437	0.055	0	Skin_Kerat	FSCN1
HBEGF1	0	1.005996	0.374	0.039	0	Skin_Kerat	HBEGF
G3BP13	0	1.000732	0.587	0.148	0	Skin_Kerat	G3BP1
CCND17	0	1.000655	0.512	0.106	0	Skin_Kerat	CCND1
CD910	0	0.991502	0.914	0.438	0	Skin_Kerat	CD9
GSPT13	0	0.982393	0.565	0.162	0	Skin_Kerat	GSPT1
SMG15	0	0.982389	0.518	0.108	0	Skin_Kerat	SMG1
PA2G41	0	0.980922	0.678	0.231	0	Skin_Kerat	PA2G4
TNFRSF183	0	0.968559	0.448	0.035	0	Skin_Kerat	TNFRSF18
RPL314	0	0.966197	1	0.838	0	Skin_Kerat	RPL3
PLP26	0	0.964644	0.699	0.217	0	Skin_Kerat	PLP2
RPL10A4	0	0.960815	0.997	0.727	0	Skin_Kerat	RPL10A
ARPC5L	0	0.960576	0.675	0.253	0	Skin_Kerat	ARPC5L
TGIF14	0	0.959596	0.589	0.184	0	Skin_Kerat	TGIF1
YWHAH5	0	0.956329	0.617	0.194	0	Skin_Kerat	YWHAH
TMED28	0	0.953362	0.782	0.332	0	Skin_Kerat	TMED2
F2RL1	0	0.949012	0.383	0.022	0	Skin_Kerat	F2RL1
RPL18A3	0	0.947624	1	0.818	0	Skin_Kerat	RPL18A
RHOV	0	0.946633	0.345	0.045	0	Skin_Kerat	RHOV
RPLP01	0	0.944887	0.993	0.792	0	Skin_Kerat	RPLP0
BAG1	0	0.944225	0.757	0.282	0	Skin_Kerat	BAG1
PAWR5	0	0.943072	0.485	0.119	0	Skin_Kerat	PAWR
FAM60A3	0	0.938321	0.469	0.094	0	Skin_Kerat	FAM60A
MRPS64	0	0.936692	0.574	0.169	0	Skin_Kerat	MRPS6
GLTP	0	0.935547	0.52	0.19	0	Skin_Kerat	GLTP
ZC3H12A4	0	0.930949	0.485	0.089	0	Skin_Kerat	ZC3H12A

RP11.295G	0	0.929232	0.398	0.048	0	Skin_Kerat	RP11.295G20.2
WDR43	0	0.921835	0.434	0.075	0	Skin_Kerat	WDR43
RBM396	0	0.920904	0.808	0.398	0	Skin_Kerat	RBM39
SUMO14	0	0.920879	0.755	0.334	0	Skin_Kerat	SUMO1
TOR1AIP2	0	0.920503	0.55	0.182	0	Skin_Kerat	TOR1AIP2
CKS23	0	0.911806	0.569	0.129	0	Skin_Kerat	CKS2
TMEM154	0	0.910027	0.379	0.028	0	Skin_Kerat	TMEM154
CD3EAP	0	0.905563	0.328	0.017	0	Skin_Kerat	CD3EAP
TMEM45A	0	0.905485	0.389	0.091	0	Skin_Kerat	TMEM45A
RPL214	0	0.902855	0.999	0.808	0	Skin_Kerat	RPL21
MORF4L23	0	0.901647	0.682	0.289	0	Skin_Kerat	MORF4L2
BMP21	0	0.901366	0.379	0.025	0	Skin_Kerat	BMP2
RPL264	0	0.892669	0.996	0.83	0	Skin_Kerat	RPL26
GPNMB3	0	0.88941	0.652	0.11	0	Skin_Kerat	GPNMB
HNRNPDL4	0	0.889387	0.788	0.322	0	Skin_Kerat	HNRNPDL
ZNF7062	0	0.886306	0.736	0.332	0	Skin_Kerat	ZNF706
EIF4G21	0	0.885823	0.824	0.448	0	Skin_Kerat	EIF4G2
IRF2BP25	0	0.884348	0.64	0.246	0	Skin_Kerat	IRF2BP2
RPL84	0	0.881473	0.997	0.844	0	Skin_Kerat	RPL8
PNRC14	0	0.88143	0.893	0.499	0	Skin_Kerat	PNRC1
METAP21	0	0.879363	0.562	0.17	0	Skin_Kerat	METAP2
TMEM165	0	0.879124	0.583	0.193	0	Skin_Kerat	TMEM165
RAN6	0	0.877955	0.786	0.354	0	Skin_Kerat	RAN
EIF5B1	0	0.877491	0.649	0.266	0	Skin_Kerat	EIF5B
HMGCS11	0	0.873925	0.414	0.084	0	Skin_Kerat	HMGCS1
SNRPB4	0	0.87166	0.743	0.32	0	Skin_Kerat	SNRPB
CHMP1B1	0	0.871213	0.716	0.299	0	Skin_Kerat	CHMP1B
PTTG12	0	0.864161	0.44	0.073	0	Skin_Kerat	PTTG1
SAR1A4	0	0.863137	0.582	0.221	0	Skin_Kerat	SAR1A
RPS62	0	0.863121	1	0.819	0	Skin_Kerat	RPS6
RPL13A3	0	0.862918	1	0.878	0	Skin_Kerat	RPL13A
EIF4A34	0	0.858131	0.561	0.188	0	Skin_Kerat	EIF4A3
EIF61	0	0.856987	0.625	0.257	0	Skin_Kerat	EIF6
CLIC16	0	0.85674	0.898	0.462	0	Skin_Kerat	CLIC1
TFAP2A2	0	0.852301	0.378	0.042	0	Skin_Kerat	TFAP2A
RPS27A6	0	0.852248	0.999	0.845	0	Skin_Kerat	RPS27A
RPS93	0	0.851368	0.998	0.832	0	Skin_Kerat	RPS9
PLEK21	0	0.850491	0.337	0.021	0	Skin_Kerat	PLEK2
KDM6B4	0	0.850239	0.497	0.106	0	Skin_Kerat	KDM6B
DUSP7	0	0.849372	0.35	0.018	0	Skin_Kerat	DUSP7
EFNA12	0	0.848899	0.393	0.08	0	Skin_Kerat	EFNA1
HNRNPA04	0	0.845939	0.773	0.321	0	Skin_Kerat	HNRNPA0
LAD1	0	0.84146	0.37	0.039	0	Skin_Kerat	LAD1
RPL384	0	0.840349	0.988	0.755	0	Skin_Kerat	RPL38
TSC22D14	0	0.840204	0.667	0.255	0	Skin_Kerat	TSC22D1
SOWAHC	0	0.839574	0.435	0.078	0	Skin_Kerat	SOWAHC
EFNB11	0	0.839001	0.37	0.024	0	Skin_Kerat	EFNB1

UBE2D34	0	0.837072	0.885	0.522	0	Skin_Kerat	UBE2D3
NXT13	0	0.836182	0.532	0.141	0	Skin_Kerat	NXT1
ABI1	0	0.835152	0.586	0.207	0	Skin_Kerat	ABI1
HNRNPH32	0	0.834212	0.616	0.208	0	Skin_Kerat	HNRNPH3
DAPL11	0	0.831933	0.336	0.02	0	Skin_Kerat	DAPL1
RND3	0	0.829363	0.819	0.312	0	Skin_Kerat	RND3
CBWD22	0	0.821057	0.369	0.04	0	Skin_Kerat	CBWD2
SFPQ7	0	0.820971	0.658	0.274	0	Skin_Kerat	SFPQ
ZNF5931	0	0.818156	0.474	0.118	0	Skin_Kerat	ZNF593
RAC14	0	0.813868	0.902	0.537	0	Skin_Kerat	RAC1
ELL24	0	0.813833	0.444	0.115	0	Skin_Kerat	ELL2
CYB5R1	0	0.813792	0.453	0.108	0	Skin_Kerat	CYB5R1
AHNAK23	0	0.811655	0.399	0.054	0	Skin_Kerat	AHNAK2
ADAM172	0	0.810456	0.408	0.079	0	Skin_Kerat	ADAM17
CKS1B4	0	0.805755	0.49	0.128	0	Skin_Kerat	CKS1B
OLFM22	0	0.805385	0.361	0.028	0	Skin_Kerat	OLFM2
DENND2C	0	0.804607	0.396	0.063	0	Skin_Kerat	DENND2C
EIF5A7	0	0.804429	0.593	0.269	0	Skin_Kerat	EIF5A
ETF11	0	0.802943	0.51	0.162	0	Skin_Kerat	ETF1
ITGA69	0	0.801798	0.42	0.127	0	Skin_Kerat	ITGA6
DR1	0	0.80162	0.478	0.116	0	Skin_Kerat	DR1
ARL5B4	0	0.800345	0.451	0.087	0	Skin_Kerat	ARL5B
TUBA1C4	0	0.798269	0.477	0.132	0	Skin_Kerat	TUBA1C
PPP1R14B1	0	0.79431	0.527	0.166	0	Skin_Kerat	PPP1R14B
BCL10	0	0.793522	0.468	0.146	0	Skin_Kerat	BCL10
PRRG41	0	0.790717	0.389	0.07	0	Skin_Kerat	PRRG4
SRSF27	0	0.790447	0.716	0.32	0	Skin_Kerat	SRSF2
EDN11	0	0.789522	0.281	0.031	0	Skin_Kerat	EDN1
LDLR2	0	0.788656	0.448	0.091	0	Skin_Kerat	LDLR
CTNND15	0	0.785454	0.408	0.09	0	Skin_Kerat	CTNND1
HCAR31	0	0.785259	0.292	0.006	0	Skin_Kerat	HCAR3
CTNNBIP1	0	0.785063	0.442	0.162	0	Skin_Kerat	CTNNBIP1
PRPF38B4	0	0.781701	0.576	0.181	0	Skin_Kerat	PRPF38B
RPL154	0	0.778538	0.998	0.892	0	Skin_Kerat	RPL15
NSG11	0	0.776601	0.355	0.027	0	Skin_Kerat	NSG1
RPL324	0	0.76903	0.999	0.868	0	Skin_Kerat	RPL32
GLS7	0	0.76831	0.453	0.106	0	Skin_Kerat	GLS
ZFAS14	0	0.763342	0.854	0.454	0	Skin_Kerat	ZFAS1
MED302	0	0.762433	0.446	0.089	0	Skin_Kerat	MED30
EIF3A4	0	0.75971	0.635	0.283	0	Skin_Kerat	EIF3A
ERF2	0	0.758855	0.397	0.063	0	Skin_Kerat	ERF
RPS204	0	0.758783	0.991	0.789	0	Skin_Kerat	RPS20
GAN	0	0.758172	0.333	0.049	0	Skin_Kerat	GAN
IFFO21	0	0.758057	0.313	0.015	0	Skin_Kerat	IFFO2
CDKN1A3	0	0.75626	0.73	0.245	0	Skin_Kerat	CDKN1A
MRPL141	0	0.755266	0.562	0.217	0	Skin_Kerat	MRPL14
DSTN5	0	0.753518	0.917	0.552	0	Skin_Kerat	DSTN

MCL15	0	0.753128	0.733	0.37	0	Skin_Kerat MCL1
RPL35A3	0	0.751096	0.995	0.859	0	Skin_Kerat RPL35A
ETNK12	0	0.750227	0.452	0.132	0	Skin_Kerat ETNK1
RAP2B2	0	0.750052	0.479	0.138	0	Skin_Kerat RAP2B
RPS22	0	0.740549	0.999	0.794	0	Skin_Kerat RPS2
IVNS1ABP8	0	0.740096	0.466	0.139	0	Skin_Kerat IVNS1ABP
IPO75	0	0.739783	0.409	0.095	0	Skin_Kerat IPO7
EEF1A113	0	0.73975	0.999	0.912	0	Skin_Kerat EEF1A1
NDUFA4L2	0	0.737712	0.389	0.065	0	Skin_Kerat NDUFA4L2
TUBB7	0	0.736325	0.723	0.298	0	Skin_Kerat TUBB
WTAP3	0	0.735882	0.688	0.268	0	Skin_Kerat WTAP
DSC1	0	0.733042	0.219	0.001	0	Skin_Kerat DSC1
IMPA2	0	0.732093	0.354	0.051	0	Skin_Kerat IMPA2
HNRNPC4	0	0.730422	0.774	0.419	0	Skin_Kerat HNRNPC
JUP	0	0.730197	0.555	0.196	0	Skin_Kerat JUP
RHEB4	0	0.728812	0.691	0.327	0	Skin_Kerat RHEB
PMEPA16	0	0.727814	0.428	0.11	0	Skin_Kerat PMEPA1
SBDS3	0	0.726754	0.714	0.33	0	Skin_Kerat SBDS
RPL23A3	0	0.726122	0.998	0.797	0	Skin_Kerat RPL23A
HRAS1	0	0.726093	0.393	0.081	0	Skin_Kerat HRAS
STRAP2	0	0.722495	0.546	0.207	0	Skin_Kerat STRAP
TIAM11	0	0.721888	0.323	0.036	0	Skin_Kerat TIAM1
CERS62	0	0.719603	0.334	0.035	0	Skin_Kerat CERS6
ARL6IP12	0	0.719325	0.642	0.308	0	Skin_Kerat ARL6IP1
UBE2G1	0	0.718412	0.59	0.225	0	Skin_Kerat UBE2G1
FGFR3	0	0.717868	0.274	0.012	0	Skin_Kerat FGFR3
BTF34	0	0.712895	0.96	0.704	0	Skin_Kerat BTF3
RPS15A3	0	0.712415	0.996	0.845	0	Skin_Kerat RPS15A
ISOC1	0	0.711777	0.341	0.06	0	Skin_Kerat ISOC1
RAP1B4	0	0.708843	0.641	0.257	0	Skin_Kerat RAP1B
PPP3CA6	0	0.70796	0.433	0.147	0	Skin_Kerat PPP3CA
HINT19	0	0.707573	0.918	0.559	0	Skin_Kerat HINT1
SRSF117	0	0.707516	0.657	0.291	0	Skin_Kerat SRSF11
RPL123	0	0.706283	0.996	0.848	0	Skin_Kerat RPL12
MIF2	0	0.705961	0.854	0.47	0	Skin_Kerat MIF
H3F3A16	0	0.705583	0.936	0.464	0	Skin_Kerat H3F3A
TIMM8A	0	0.70511	0.303	0.025	0	Skin_Kerat TIMM8A
HCAR21	0	0.704898	0.298	0.012	0	Skin_Kerat HCAR2
NAB13	0	0.702559	0.352	0.052	0	Skin_Kerat NAB1
CHD16	0	0.700343	0.471	0.121	0	Skin_Kerat CHD1
TSPYL15	0	0.696523	0.428	0.146	0	Skin_Kerat TSPYL1
RGS122	0	0.694779	0.321	0.031	0	Skin_Kerat RGS12
CORO1C3	0	0.69463	0.417	0.108	0	Skin_Kerat CORO1C
CRK4	0	0.69058	0.427	0.11	0	Skin_Kerat CRK
WDR47	0	0.690135	0.334	0.036	0	Skin_Kerat WDR47
RPL273	0	0.687716	0.981	0.743	0	Skin_Kerat RPL27
CDH16	0	0.687093	0.331	0.067	0	Skin_Kerat CDH1

HNRNPA13	0	0.684092	0.922	0.533	0	Skin_Kerat	HNRNPA1
CENPP	0	0.683982	0.275	0.016	0	Skin_Kerat	CENPP
SRRM16	0	0.682878	0.657	0.317	0	Skin_Kerat	SRRM1
RABGGTB4	0	0.682482	0.404	0.091	0	Skin_Kerat	RABGGTB
C1orf213	0	0.68082	0.409	0.091	0	Skin_Kerat	C1orf21
YBX35	0	0.680635	0.854	0.484	0	Skin_Kerat	YBX3
ARPC25	0	0.679714	0.881	0.565	0	Skin_Kerat	ARPC2
RPS249	0	0.67894	0.996	0.834	0	Skin_Kerat	RPS24
NUPR19	0	0.678817	0.674	0.247	0	Skin_Kerat	NUPR1
MBNL18	0	0.678686	0.549	0.159	0	Skin_Kerat	MBNL1
MEAF62	0	0.677671	0.578	0.214	0	Skin_Kerat	MEAF6
FAM46B	0	0.67636	0.314	0.055	0	Skin_Kerat	FAM46B
RSL1D12	0	0.674242	0.622	0.236	0	Skin_Kerat	RSL1D1
APOE3	0	0.674007	0.804	0.146	0	Skin_Kerat	APOE
STX112	0	0.669846	0.356	0.056	0	Skin_Kerat	STX11
MIR22HG3	0	0.669067	0.391	0.076	0	Skin_Kerat	MIR22HG
PTPN131	0	0.668462	0.328	0.048	0	Skin_Kerat	PTPN13
BBC3	0	0.668024	0.365	0.059	0	Skin_Kerat	BBC3
PPP2CA3	0	0.667801	0.568	0.222	0	Skin_Kerat	PPP2CA
RAD23B1	0	0.667072	0.53	0.181	0	Skin_Kerat	RAD23B
PDLIM14	0	0.666331	0.54	0.214	0	Skin_Kerat	PDLIM1
EEF24	0	0.665806	0.952	0.597	0	Skin_Kerat	EEF2
FAM213A2	0	0.665273	0.386	0.12	0	Skin_Kerat	FAM213A
PKM4	0	0.664999	0.755	0.361	0	Skin_Kerat	PKM
HNRNPF2	0	0.664643	0.65	0.297	0	Skin_Kerat	HNRNPF
TES2	0	0.664631	0.417	0.118	0	Skin_Kerat	TES
RPL376	0	0.664326	0.993	0.843	0	Skin_Kerat	RPL37
YY14	0	0.663831	0.546	0.217	0	Skin_Kerat	YY1
EFNA3	0	0.661548	0.284	0.011	0	Skin_Kerat	EFNA3
RPS252	0	0.660996	0.997	0.818	0	Skin_Kerat	RPS25
NFAT55	0	0.660287	0.433	0.118	0	Skin_Kerat	NFAT5
EIF2S1	0	0.66005	0.407	0.112	0	Skin_Kerat	EIF2S1
MACF18	0	0.659898	0.471	0.159	0	Skin_Kerat	MACF1
C9orf36	0	0.659696	0.397	0.089	0	Skin_Kerat	C9orf3
GATA31	0	0.658529	0.346	0.075	0	Skin_Kerat	GATA3
RPL27A3	0	0.656918	0.995	0.831	0	Skin_Kerat	RPL27A
HDAC21	0	0.655268	0.493	0.176	0	Skin_Kerat	HDAC2
GNG121	0	0.654282	0.508	0.185	0	Skin_Kerat	GNG12
UBALD1	0	0.653484	0.349	0.07	0	Skin_Kerat	UBALD1
NPM12	0	0.652901	0.899	0.522	0	Skin_Kerat	NPM1
DDX3X6	0	0.650914	0.627	0.291	0	Skin_Kerat	DDX3X
RPS115	0	0.649886	0.989	0.771	0	Skin_Kerat	RPS11
EIF3J1	0	0.648612	0.477	0.186	0	Skin_Kerat	EIF3J
IMP33	0	0.648558	0.514	0.177	0	Skin_Kerat	IMP3
FBL1	0	0.644898	0.626	0.224	0	Skin_Kerat	FBL
JAG15	0	0.644311	0.333	0.041	0	Skin_Kerat	JAG1
CEBPG	0	0.643858	0.398	0.101	0	Skin_Kerat	CEBPG

ZNF217	0	0.642155	0.335	0.099	0	Skin_Kerat	ZNF217
LRRC8A	0	0.641379	0.366	0.072	0	Skin_Kerat	LRRC8A
RPS125	0	0.640536	0.996	0.857	0	Skin_Kerat	RPS12
SNHG153	0	0.640528	0.446	0.1	0	Skin_Kerat	SNHG15
NOLC12	0	0.639692	0.367	0.072	0	Skin_Kerat	NOLC1
KDSR6	0	0.638243	0.431	0.12	0	Skin_Kerat	KDSR
MAP7D12	0	0.636453	0.41	0.103	0	Skin_Kerat	MAP7D1
RAB143	0	0.636175	0.494	0.178	0	Skin_Kerat	RAB14
PPP2R2A1	0	0.634981	0.426	0.12	0	Skin_Kerat	PPP2R2A
SNRPF3	0	0.633362	0.581	0.227	0	Skin_Kerat	SNRPF
LGALSL2	0	0.633264	0.277	0.042	0	Skin_Kerat	LGALSL
CEBPA1	0	0.633023	0.25	0.021	0	Skin_Kerat	CEBPA
HDGF2	0	0.632589	0.421	0.13	0	Skin_Kerat	HDGF
ATP2B16	0	0.631525	0.483	0.14	0	Skin_Kerat	ATP2B1
PLIN22	0	0.631268	0.369	0.119	0	Skin_Kerat	PLIN2
BICD21	0	0.629414	0.307	0.04	0	Skin_Kerat	BICD2
HMGA1	0	0.62922	0.653	0.274	0	Skin_Kerat	HMGA1
PLK23	0	0.629158	0.349	0.077	0	Skin_Kerat	PLK2
PANX11	0	0.627754	0.277	0.022	0	Skin_Kerat	PANX1
RPS237	0	0.626462	0.996	0.808	0	Skin_Kerat	RPS23
RPS135	0	0.626415	0.995	0.812	0	Skin_Kerat	RPS13
SELT5	0	0.625221	0.535	0.217	0	Skin_Kerat	SELT
NAA502	0	0.621125	0.476	0.193	0	Skin_Kerat	NAA50
PABPN16	0	0.620106	0.447	0.141	0	Skin_Kerat	PABPN1
MED31	0	0.618901	0.373	0.109	0	Skin_Kerat	MED31
ARL6IP61	0	0.618481	0.35	0.063	0	Skin_Kerat	ARL6IP6
C1orf521	0	0.617885	0.394	0.106	0	Skin_Kerat	C1orf52
NR4A13	0	0.616332	0.607	0.246	0	Skin_Kerat	NR4A1
KIAA12172	0	0.616229	0.283	0.067	0	Skin_Kerat	KIAA1217
TLE31	0	0.616133	0.368	0.093	0	Skin_Kerat	TLE3
TSC22D23	0	0.616097	0.379	0.099	0	Skin_Kerat	TSC22D2
RPS4X1	0	0.614184	0.997	0.764	0	Skin_Kerat	RPS4X
TWISTNB3	0	0.611692	0.368	0.085	0	Skin_Kerat	TWISTNB
ITGAV3	0	0.610991	0.36	0.087	0	Skin_Kerat	ITGAV
PYURF2	0	0.610269	0.553	0.238	0	Skin_Kerat	PYURF
SIAH22	0	0.610024	0.406	0.096	0	Skin_Kerat	SIAH2
FRMD62	0	0.609863	0.377	0.077	0	Skin_Kerat	FRMD6
NBL14	0	0.609311	0.489	0.099	0	Skin_Kerat	NBL1
UBE2D12	0	0.607707	0.473	0.173	0	Skin_Kerat	UBE2D1
ENAH6	0	0.607705	0.391	0.11	0	Skin_Kerat	ENAH
CPEB2	0	0.607068	0.318	0.071	0	Skin_Kerat	CPEB2
RAB5A1	0	0.606012	0.506	0.198	0	Skin_Kerat	RAB5A
FAM174A1	0	0.605632	0.334	0.076	0	Skin_Kerat	FAM174A
UBE2H2	0	0.604585	0.415	0.138	0	Skin_Kerat	UBE2H
STRN32	0	0.603235	0.334	0.085	0	Skin_Kerat	STRN3
VIMP5	0	0.6021	0.62	0.282	0	Skin_Kerat	VIMP
RP11.345J4	0	0.601432	0.29	0.021	0	Skin_Kerat	RP11.345J4.5

PVRL11	0	0.60117	0.267	0.021	0	Skin_Kerat PVRL1
GNAI32	0	0.600092	0.491	0.182	0	Skin_Kerat GNAI3
ATP2B47	0	0.599432	0.369	0.071	0	Skin_Kerat ATP2B4
GRPEL11	0	0.599343	0.4	0.121	0	Skin_Kerat GRPEL1
CLEC2B7	0	0.597481	0.493	0.145	0	Skin_Kerat CLEC2B
ATP1A110	0	0.596648	0.604	0.245	0	Skin_Kerat ATP1A1
FAU6	0	0.594336	0.995	0.851	0	Skin_Kerat FAU
PCNP4	0	0.593105	0.596	0.249	0	Skin_Kerat PCNP
NFKB13	0	0.592449	0.413	0.11	0	Skin_Kerat NFKB1
LRRC594	0	0.592442	0.401	0.136	0	Skin_Kerat LRRC59
TIMM131	0	0.591495	0.563	0.237	0	Skin_Kerat TIMM13
NAA152	0	0.590693	0.331	0.067	0	Skin_Kerat NAA15
TUBA4A2	0	0.589434	0.507	0.158	0	Skin_Kerat TUBA4A
HNRNPA1L	0	0.585276	0.308	0.041	0	Skin_Kerat HNRNPA1L2
ANKRD22	0	0.584071	0.218	0.008	0	Skin_Kerat ANKRD22
FAM83G	0	0.583312	0.249	0.007	0	Skin_Kerat FAM83G
TOB24	0	0.583122	0.361	0.092	0	Skin_Kerat TOB2
WSB18	0	0.582758	0.571	0.24	0	Skin_Kerat WSB1
MIB2	0	0.58254	0.374	0.086	0	Skin_Kerat MIB2
RRAGC2	0	0.580672	0.36	0.094	0	Skin_Kerat RRAGC
IL1R21	0	0.580621	0.28	0.034	0	Skin_Kerat IL1R2
RPL42	0	0.578498	0.985	0.69	0	Skin_Kerat RPL4
DUSP14	0	0.577823	0.372	0.118	0	Skin_Kerat DUSP14
BLCAP1	0	0.576478	0.4	0.131	0	Skin_Kerat BLCAP
RNF1683	0	0.57621	0.355	0.089	0	Skin_Kerat RNF168
C19orf33	0	0.57555	0.598	0.185	0	Skin_Kerat C19orf33
EXPH51	0	0.574423	0.303	0.062	0	Skin_Kerat EXPH5
NDUFAF41	0	0.572918	0.327	0.074	0	Skin_Kerat NDUFAF4
CBWD51	0	0.572189	0.27	0.033	0	Skin_Kerat CBWD5
TAF1D4	0	0.571669	0.529	0.218	0	Skin_Kerat TAF1D
S100A112	0	0.568355	0.989	0.657	0	Skin_Kerat S100A11
IMPDH22	0	0.566468	0.463	0.148	0	Skin_Kerat IMPDH2
GSTA42	0	0.56639	0.273	0.035	0	Skin_Kerat GSTA4
AHSA12	0	0.566192	0.422	0.136	0	Skin_Kerat AHSA1
PELI1	0	0.565609	0.518	0.187	0	Skin_Kerat PELI1
DUSP12	0	0.565595	0.914	0.551	0	Skin_Kerat DUSP1
LRRFIP21	0	0.563897	0.434	0.147	0	Skin_Kerat LRRFIP2
C7orf55.LU	0	0.56257	0.324	0.037	0	Skin_Kerat C7orf55.LUC7L2
DKC1	0	0.561919	0.332	0.072	0	Skin_Kerat DKC1
SNHG86	0	0.561496	0.65	0.285	0	Skin_Kerat SNHG8
RPLP22	0	0.559205	0.999	0.839	0	Skin_Kerat RPLP2
EPHB61	0	0.559141	0.272	0.027	0	Skin_Kerat EPHB6
GATM5	0	0.558312	0.282	0.071	0	Skin_Kerat GATM
TARDBP1	0	0.557113	0.371	0.105	0	Skin_Kerat TARDBP
PNPLA82	0	0.556613	0.358	0.097	0	Skin_Kerat PNPLA8
CYLD4	0	0.55605	0.397	0.116	0	Skin_Kerat CYLD
TAF4B1	0	0.556013	0.264	0.025	0	Skin_Kerat TAF4B

TMX13	0	0.55562	0.361	0.097	0	Skin_Kerat	TMX1
NFATC31	0	0.553111	0.348	0.107	0	Skin_Kerat	NFATC3
TAF131	0	0.552881	0.273	0.043	0	Skin_Kerat	TAF13
IRS21	0	0.551102	0.324	0.098	0	Skin_Kerat	IRS2
RPS73	0	0.54893	0.997	0.81	0	Skin_Kerat	RPS7
AEBP22	0	0.548759	0.284	0.056	0	Skin_Kerat	AEBP2
PAFAH1B2	0	0.548727	0.373	0.119	0	Skin_Kerat	PAFAH1B2
SLC38A16	0	0.547717	0.353	0.091	0	Skin_Kerat	SLC38A1
ADORA2B	0	0.546936	0.24	0.005	0	Skin_Kerat	ADORA2B
TXN1	0	0.546681	0.932	0.515	0	Skin_Kerat	TXN
CMIP2	0	0.543804	0.362	0.107	0	Skin_Kerat	CMIP
BASP14	0	0.543489	0.305	0.077	0	Skin_Kerat	BASP1
C4orf324	0	0.541524	0.328	0.074	0	Skin_Kerat	C4orf32
RANBP13	0	0.539309	0.594	0.257	0	Skin_Kerat	RANBP1
C1QBP	0	0.536088	0.554	0.228	0	Skin_Kerat	C1QBP
RBM2510	0	0.532935	0.549	0.23	0	Skin_Kerat	RBM25
SDCBP21	0	0.532192	0.253	0.027	0	Skin_Kerat	SDCBP2
RAPGEFL1	0	0.532124	0.235	0.012	0	Skin_Kerat	RAPGEFL1
CLDND13	0	0.530896	0.479	0.182	0	Skin_Kerat	CLDND1
TNFAIP85	0	0.530494	0.391	0.096	0	Skin_Kerat	TNFAIP8
JARID21	0	0.529786	0.328	0.094	0	Skin_Kerat	JARID2
C20orf241	0	0.528957	0.467	0.145	0	Skin_Kerat	C20orf24
TNKS1BP1:	0	0.528304	0.287	0.058	0	Skin_Kerat	TNKS1BP1
MT2A2	0	0.525738	0.815	0.291	0	Skin_Kerat	MT2A
BRIX12	0	0.525082	0.331	0.084	0	Skin_Kerat	BRIX1
MRPL50	0	0.524735	0.332	0.092	0	Skin_Kerat	MRPL50
LURAP1L2	0	0.518785	0.247	0.049	0	Skin_Kerat	LURAP1L
TTC193	0	0.517342	0.357	0.115	0	Skin_Kerat	TTC19
RNF126	0	0.516007	0.432	0.133	0	Skin_Kerat	RNF126
SPINT27	0	0.515861	0.686	0.302	0	Skin_Kerat	SPINT2
NDEL12	0	0.513172	0.324	0.073	0	Skin_Kerat	NDEL1
RNPS11	0	0.512618	0.505	0.189	0	Skin_Kerat	RNPS1
MBIP	0	0.511565	0.283	0.061	0	Skin_Kerat	MBIP
PPTC73	0	0.511323	0.299	0.056	0	Skin_Kerat	PPTC7
DAPK31	0	0.508052	0.297	0.079	0	Skin_Kerat	DAPK3
BARD1	0	0.507612	0.255	0.035	0	Skin_Kerat	BARD1
TRIB13	0	0.506755	0.273	0.039	0	Skin_Kerat	TRIB1
PRMT14	0	0.504795	0.496	0.182	0	Skin_Kerat	PRMT1
SLC16A11	0	0.503129	0.228	0.038	0	Skin_Kerat	SLC16A1
PTMS9	0	0.502631	0.582	0.234	0	Skin_Kerat	PTMS
RAB38	0	0.502111	0.233	0.014	0	Skin_Kerat	RAB38
SLC20A2	0	0.500145	0.286	0.069	0	Skin_Kerat	SLC20A2
TIPIN4	0	0.497286	0.296	0.067	0	Skin_Kerat	TIPIN
RPS31	0	0.493541	0.999	0.811	0	Skin_Kerat	RPS3
RPL315	0	0.493269	0.996	0.761	0	Skin_Kerat	RPL31
NUP1531	0	0.491855	0.278	0.056	0	Skin_Kerat	NUP153
CDCP1	0	0.49099	0.313	0.09	0	Skin_Kerat	CDCP1

RGS141	0	0.488414	0.281	0.039	0	Skin_Kerat	RGS14
FBLIM12	0	0.488055	0.256	0.055	0	Skin_Kerat	FBLIM1
CDHR1	0	0.487869	0.198	0.002	0	Skin_Kerat	CDHR1
GPR87	0	0.48717	0.233	0.033	0	Skin_Kerat	GPR87
DUSP111	0	0.485754	0.297	0.077	0	Skin_Kerat	DUSP11
PCDH71	0	0.48562	0.211	0.016	0	Skin_Kerat	PCDH7
DHX152	0	0.485329	0.338	0.105	0	Skin_Kerat	DHX15
SEMA4B	0	0.484665	0.222	0.03	0	Skin_Kerat	SEMA4B
SDC11	0	0.483475	0.408	0.129	0	Skin_Kerat	SDC1
PKP31	0	0.480437	0.276	0.059	0	Skin_Kerat	PKP3
MSN8	0	0.478351	0.454	0.147	0	Skin_Kerat	MSN
RPL131	0	0.478045	1	0.916	0	Skin_Kerat	RPL13
HOMER11	0	0.476698	0.214	0.024	0	Skin_Kerat	HOMER1
RRP1	0	0.476087	0.258	0.046	0	Skin_Kerat	RRP1
MAF3	0	0.473391	0.329	0.074	0	Skin_Kerat	MAF
EFNB21	0	0.470662	0.258	0.044	0	Skin_Kerat	EFNB2
PTGS23	0	0.46921	0.188	0.035	0	Skin_Kerat	PTGS2
SLC20A12	0	0.467578	0.277	0.067	0	Skin_Kerat	SLC20A1
NFIB11	0	0.466343	0.494	0.182	0	Skin_Kerat	NFIB
BCL11B4	0	0.465604	0.236	0.021	0	Skin_Kerat	BCL11B
NBPF102	0	0.464387	0.265	0.041	0	Skin_Kerat	NBPF10
TDG	0	0.464002	0.3	0.081	0	Skin_Kerat	TDG
NAA25	0	0.463823	0.247	0.043	0	Skin_Kerat	NAA25
COL7A12	0	0.462286	0.176	0.012	0	Skin_Kerat	COL7A1
USP123	0	0.460486	0.289	0.063	0	Skin_Kerat	USP12
RRP9	0	0.459691	0.223	0.032	0	Skin_Kerat	RRP9
LPAR65	0	0.459653	0.395	0.133	0	Skin_Kerat	LPAR6
ETV32	0	0.45953	0.264	0.042	0	Skin_Kerat	ETV3
YBX14	0	0.458322	0.926	0.578	0	Skin_Kerat	YBX1
SMTN4	0	0.457791	0.239	0.036	0	Skin_Kerat	SMTN
SULT2B1	0	0.456162	0.216	0.05	0	Skin_Kerat	SULT2B1
ZC3H12C	0	0.455614	0.213	0.025	0	Skin_Kerat	ZC3H12C
CBR14	0	0.455442	0.348	0.111	0	Skin_Kerat	CBR1
PAN34	0	0.451215	0.278	0.075	0	Skin_Kerat	PAN3
NIFK2	0	0.451182	0.324	0.089	0	Skin_Kerat	NIFK
IFNGR14	0	0.448699	0.386	0.127	0	Skin_Kerat	IFNGR1
TET3	0	0.448512	0.222	0.027	0	Skin_Kerat	TET3
SGK12	0	0.448419	0.37	0.105	0	Skin_Kerat	SGK1
SCML12	0	0.447204	0.335	0.062	0	Skin_Kerat	SCML1
KCNK7	0	0.444489	0.184	0.002	0	Skin_Kerat	KCNK7
MFSD2A1	0	0.444114	0.195	0.013	0	Skin_Kerat	MFSD2A
SPEN2	0	0.443246	0.327	0.097	0	Skin_Kerat	SPEN
KIAA15224	0	0.44293	0.261	0.063	0	Skin_Kerat	KIAA1522
PNO11	0	0.442883	0.246	0.051	0	Skin_Kerat	PNO1
TMEM30B	0	0.439079	0.243	0.058	0	Skin_Kerat	TMEM30B
HMGCR	0	0.438737	0.233	0.049	0	Skin_Kerat	HMGCR
CRY1	0	0.437509	0.246	0.058	0	Skin_Kerat	CRY1

PRSS3	0	0.43733	0.249	0.059	0	Skin_Kerat	PRSS3
WDR361	0	0.436765	0.22	0.035	0	Skin_Kerat	WDR36
DCAF13	0	0.43435	0.28	0.076	0	Skin_Kerat	DCAF13
PSMG1	0	0.434095	0.341	0.095	0	Skin_Kerat	PSMG1
DIP2B	0	0.433406	0.223	0.04	0	Skin_Kerat	DIP2B
TRMT10C	0	0.430948	0.315	0.088	0	Skin_Kerat	TRMT10C
SGMS23	0	0.430903	0.233	0.051	0	Skin_Kerat	SGMS2
SGMS12	0	0.429739	0.241	0.049	0	Skin_Kerat	SGMS1
FAM57A	0	0.429046	0.203	0.025	0	Skin_Kerat	FAM57A
LMNA11	0	0.428265	0.808	0.352	0	Skin_Kerat	LMNA
FAM83C	0	0.428229	0.164	0.002	0	Skin_Kerat	FAM83C
ARMC10	0	0.427421	0.304	0.086	0	Skin_Kerat	ARMC10
AJUBA	0	0.426864	0.192	0.014	0	Skin_Kerat	AJUBA
POLE31	0	0.425686	0.316	0.088	0	Skin_Kerat	POLE3
PPP3R12	0	0.425531	0.273	0.064	0	Skin_Kerat	PPP3R1
MAPKBP1	0	0.422218	0.209	0.028	0	Skin_Kerat	MAPKBP1
ZNF7701	0	0.422068	0.27	0.07	0	Skin_Kerat	ZNF770
FEM1B	0	0.42088	0.331	0.096	0	Skin_Kerat	FEM1B
TINCR	0	0.420877	0.24	0.047	0	Skin_Kerat	TINCR
SNAPC1	0	0.419556	0.272	0.062	0	Skin_Kerat	SNAPC1
HIVEP22	0	0.416325	0.305	0.085	0	Skin_Kerat	HIVEP2
KLF112	0	0.415913	0.259	0.065	0	Skin_Kerat	KLF11
GCH1	0	0.412513	0.268	0.051	0	Skin_Kerat	GCH1
SRF	0	0.408585	0.24	0.045	0	Skin_Kerat	SRF
CRABP23	0	0.407835	0.307	0.076	0	Skin_Kerat	CRABP2
TNNT1	0	0.40699	0.169	0.011	0	Skin_Kerat	TNNT1
ANP32E4	0	0.405207	0.315	0.09	0	Skin_Kerat	ANP32E
WDR741	0	0.40353	0.351	0.094	0	Skin_Kerat	WDR74
DLL11	0	0.402946	0.148	0.011	0	Skin_Kerat	DLL1
IARS3	0	0.402759	0.255	0.066	0	Skin_Kerat	IARS
SIDT21	0	0.399378	0.22	0.045	0	Skin_Kerat	SIDT2
C1orf109	0	0.398763	0.217	0.026	0	Skin_Kerat	C1orf109
AK4	0	0.398015	0.227	0.052	0	Skin_Kerat	AK4
SMIM131	0	0.395744	0.206	0.035	0	Skin_Kerat	SMIM13
SEH1L	0	0.395203	0.223	0.037	0	Skin_Kerat	SEH1L
FOSB9	0	0.395006	0.62	0.24	0	Skin_Kerat	FOSB
CDH31	0	0.393413	0.164	0.01	0	Skin_Kerat	CDH3
BCL11A2	0	0.393146	0.202	0.04	0	Skin_Kerat	BCL11A
SDR16C51	0	0.391078	0.227	0.049	0	Skin_Kerat	SDR16C5
RRS1	0	0.389336	0.232	0.032	0	Skin_Kerat	RRS1
TFB2M	0	0.389257	0.192	0.032	0	Skin_Kerat	TFB2M
SCO21	0	0.387677	0.304	0.084	0	Skin_Kerat	SCO2
CDCA4	0	0.385523	0.237	0.047	0	Skin_Kerat	CDCA4
ZBTB211	0	0.38518	0.222	0.041	0	Skin_Kerat	ZBTB21
USP312	0	0.384898	0.18	0.033	0	Skin_Kerat	USP31
GRHL3	0	0.384119	0.154	0.01	0	Skin_Kerat	GRHL3
LONRF12	0	0.382063	0.243	0.054	0	Skin_Kerat	LONRF1

B3GNT23	0	0.381616	0.251	0.063	0	Skin_Kerat	B3GNT2
TGFBI6	0	0.381438	0.267	0.057	0	Skin_Kerat	TGFBI
NACA24	0	0.380873	0.262	0.067	0	Skin_Kerat	NACA2
NR1D11	0	0.380004	0.232	0.042	0	Skin_Kerat	NR1D1
TMEM237	0	0.37726	0.211	0.037	0	Skin_Kerat	TMEM237
SLC35F21	0	0.376789	0.186	0.025	0	Skin_Kerat	SLC35F2
PTGFRN3	0	0.374259	0.196	0.036	0	Skin_Kerat	PTGFRN
TMEM41A	0	0.372772	0.23	0.052	0	Skin_Kerat	TMEM41A
CEBPD2	0	0.372314	0.751	0.324	0	Skin_Kerat	CEBPD
PTRF6	0	0.371031	0.477	0.154	0	Skin_Kerat	PTRF
POLR1C	0	0.37053	0.203	0.044	0	Skin_Kerat	POLR1C
SPSB11	0	0.369321	0.246	0.053	0	Skin_Kerat	SPSB1
CYP3A5	0	0.368932	0.193	0.034	0	Skin_Kerat	CYP3A5
CRKL	0	0.366126	0.203	0.034	0	Skin_Kerat	CRKL
MATR34	0	0.366059	0.363	0.108	0	Skin_Kerat	MATR3
PARD6G1	0	0.365721	0.179	0.011	0	Skin_Kerat	PARD6G
MINK1	0	0.365462	0.188	0.03	0	Skin_Kerat	MINK1
RP3.512B1	0	0.364762	0.168	0.008	0	Skin_Kerat	RP3.512B11.3
STXBP61	0	0.361361	0.201	0.027	0	Skin_Kerat	STXBP6
C6orf141	0	0.359354	0.155	0.018	0	Skin_Kerat	C6orf141
TSLP1	0	0.35771	0.143	0.012	0	Skin_Kerat	TSLP
PPAN	0	0.355622	0.209	0.038	0	Skin_Kerat	PPAN
IPMK1	0	0.355469	0.216	0.044	0	Skin_Kerat	IPMK
NHSL11	0	0.355182	0.184	0.034	0	Skin_Kerat	NHSL1
CALD15	0	0.353647	0.558	0.183	0	Skin_Kerat	CALD1
SRPK2	0	0.352386	0.42	0.145	0	Skin_Kerat	SRPK2
ARPP191	0	0.350236	0.545	0.208	0	Skin_Kerat	ARPP19
HSPB14	0	0.348399	0.956	0.553	0	Skin_Kerat	HSPB1
FRMD6.AS	0	0.348263	0.098	0.002	0	Skin_Kerat	FRMD6.AS2
EXT12	0	0.342381	0.197	0.031	0	Skin_Kerat	EXT1
ETS15	0	0.342133	0.286	0.07	0	Skin_Kerat	ETS1
LYPD6B	0	0.341917	0.181	0.024	0	Skin_Kerat	LYPD6B
LYAR1	0	0.341549	0.341	0.086	0	Skin_Kerat	LYAR
PSAT11	0	0.341118	0.178	0.024	0	Skin_Kerat	PSAT1
SERPINB8	0	0.340513	0.183	0.033	0	Skin_Kerat	SERPINB8
F12	0	0.339288	0.164	0.016	0	Skin_Kerat	F12
DGKH4	0	0.339264	0.187	0.031	0	Skin_Kerat	DGKH
SOX71	0	0.33789	0.182	0.016	0	Skin_Kerat	SOX7
MPZL31	0	0.336413	0.192	0.035	0	Skin_Kerat	MPZL3
PTPRE4	0	0.33295	0.219	0.048	0	Skin_Kerat	PTPRE
FAM83B	0	0.332168	0.155	0.017	0	Skin_Kerat	FAM83B
ATF11	0	0.331194	0.246	0.06	0	Skin_Kerat	ATF1
FZD61	0	0.330939	0.181	0.034	0	Skin_Kerat	FZD6
NMU	0	0.323692	0.152	0.018	0	Skin_Kerat	NMU
ZXDB	0	0.3219	0.147	0.012	0	Skin_Kerat	ZXDB
CHSY13	0	0.320526	0.223	0.049	0	Skin_Kerat	CHSY1
ARL13B2	0	0.317872	0.202	0.044	0	Skin_Kerat	ARL13B

ALKBH21	0	0.317541	0.209	0.041	0	Skin_Kerat	ALKBH2
INPP11	0	0.312743	0.298	0.077	0	Skin_Kerat	INPP1
ACTG1	0	0.311735	0.995	0.82	0	Skin_Kerat	ACTG1
PPRC1	0	0.311184	0.176	0.025	0	Skin_Kerat	PPRC1
ZNF7035	0	0.309376	0.203	0.039	0	Skin_Kerat	ZNF703
FERMT11	0	0.308689	0.151	0.011	0	Skin_Kerat	FERMT1
AC025335.	0	0.308578	0.139	0.006	0	Skin_Kerat	AC025335.1
ITGA33	0	0.306238	0.162	0.028	0	Skin_Kerat	ITGA3
SLC19A21	0	0.303405	0.15	0.026	0	Skin_Kerat	SLC19A2
RNMTL1	0	0.301022	0.191	0.04	0	Skin_Kerat	RNMTL1
NIPAL4	0	0.300213	0.132	0.001	0	Skin_Kerat	NIPAL4
CTD.2090I:	0	0.298672	0.132	0.012	0	Skin_Kerat	CTD.2090I13.1
SRCAP	0	0.297764	0.154	0.02	0	Skin_Kerat	SRCAP
ZNF385A3	0	0.283794	0.187	0.033	0	Skin_Kerat	ZNF385A
C3orf52	0	0.280766	0.137	0.021	0	Skin_Kerat	C3orf52
NR4A34	0	0.280192	0.227	0.048	0	Skin_Kerat	NR4A3
MCC2	0	0.276588	0.169	0.031	0	Skin_Kerat	MCC
DNMBP	0	0.276034	0.16	0.027	0	Skin_Kerat	DNMBP
LRRC8C2	0	0.275964	0.176	0.028	0	Skin_Kerat	LRRC8C
MMP281	0	0.275459	0.137	0.011	0	Skin_Kerat	MMP28
POU3F1	0	0.272863	0.131	0.004	0	Skin_Kerat	POU3F1
SERPINE11	0	0.269351	0.12	0.016	0	Skin_Kerat	SERPINE1
LY6G6C	0	0.265593	0.094	0.007	0	Skin_Kerat	LY6G6C
PRR71	0	0.264742	0.208	0.045	0	Skin_Kerat	PRR7
WNT3	0	0.264575	0.116	0.009	0	Skin_Kerat	WNT3
KLK11	0	0.262277	0.124	0.006	0	Skin_Kerat	KLK1
ELOVL71	0	0.262199	0.138	0.018	0	Skin_Kerat	ELOVL7
RCAN12	0	0.255809	0.27	0.067	0	Skin_Kerat	RCAN1
VDR1	0	0.253882	0.148	0.022	0	Skin_Kerat	VDR
POU2F3	0	0.252732	0.118	0.008	0	Skin_Kerat	POU2F3
NMD32	7.5E-308	0.372905	0.265	0.071	1.2E-303	Skin_Kerat	NMD3
ATXN15	1.1E-307	0.391343	0.255	0.066	1.8E-303	Skin_Kerat	ATXN1
EIF2S32	2.2E-307	0.486393	0.415	0.148	3.5E-303	Skin_Kerat	EIF2S3
RPS3A3	2.9E-307	0.508615	0.997	0.826	4.6E-303	Skin_Kerat	RPS3A
SDE2	4.8E-307	0.341545	0.199	0.044	7.6E-303	Skin_Kerat	SDE2
MRPL232	3.8E-306	0.464502	0.506	0.202	6E-302	Skin_Kerat	MRPL23
ARL4A1	4.6E-306	0.37076	0.539	0.222	7.4E-302	Skin_Kerat	ARL4A
ADSS1	1.1E-305	0.45455	0.341	0.108	1.8E-301	Skin_Kerat	ADSS
ATF45	3.3E-305	0.64332	0.661	0.327	5.2E-301	Skin_Kerat	ATF4
SNRPD1	3.9E-305	0.477298	0.527	0.212	6.1E-301	Skin_Kerat	SNRPD1
SMPDL3A	4.1E-305	0.395029	0.229	0.056	6.5E-301	Skin_Kerat	SMPDL3A
NXF1	1.1E-304	0.393688	0.268	0.072	1.8E-300	Skin_Kerat	NXF1
C1orf106	2.1E-304	0.301988	0.187	0.039	3.3E-300	Skin_Kerat	C1orf106
POLR2L3	2.7E-304	0.620249	0.827	0.462	4.3E-300	Skin_Kerat	POLR2L
STX12	7.9E-304	0.513248	0.344	0.113	1.3E-299	Skin_Kerat	STX12
STK17A1	1.8E-303	0.398904	0.408	0.14	2.8E-299	Skin_Kerat	STK17A
HNRNPD4	3.8E-303	0.611029	0.53	0.231	6.1E-299	Skin_Kerat	HNRNPD

RPL36A2	2.5E-301	0.505436	0.897	0.498	4E-297	Skin_Kerat	RPL36A
RPL111	5.7E-301	0.467245	1	0.849	9.1E-297	Skin_Kerat	RPL11
TOMM40	7.9E-301	0.420835	0.289	0.083	1.3E-296	Skin_Kerat	TOMM40
RPL2213	3.8E-300	0.599693	0.978	0.685	6E-296	Skin_Kerat	RPL22
ACTR33	9.5E-300	0.614376	0.692	0.345	1.5E-295	Skin_Kerat	ACTR3
CAPZA24	2.7E-299	0.612427	0.595	0.269	4.2E-295	Skin_Kerat	CAPZA2
SS18L22	7.5E-299	0.393307	0.365	0.123	1.2E-294	Skin_Kerat	SS18L2
OSER1	5.4E-298	0.469833	0.525	0.218	8.6E-294	Skin_Kerat	OSER1
RNF2172	7.2E-298	0.315712	0.19	0.041	1.2E-293	Skin_Kerat	RNF217
CYP24A1	1.4E-297	0.321225	0.055	0.004	2.2E-293	Skin_Kerat	CYP24A1
ENSA	3.3E-297	0.536674	0.529	0.226	5.3E-293	Skin_Kerat	ENSA
GPATCH41	2.6E-296	0.36091	0.237	0.06	4.2E-292	Skin_Kerat	GPATCH4
DDX186	7.8E-296	0.528002	0.489	0.195	1.2E-291	Skin_Kerat	DDX18
VDAC14	1E-295	0.561233	0.536	0.233	1.7E-291	Skin_Kerat	VDAC1
HYPK1	3E-295	0.290346	0.14	0.024	4.8E-291	Skin_Kerat	HYPK
RPS101	2.8E-294	0.535616	0.936	0.597	4.5E-290	Skin_Kerat	RPS10
THBD3	1.1E-293	0.262782	0.184	0.038	1.7E-289	Skin_Kerat	THBD
HECA2	2.9E-293	0.447187	0.398	0.14	4.6E-289	Skin_Kerat	HECA
TNFRSF19	4.7E-293	0.398532	0.234	0.061	7.5E-289	Skin_Kerat	TNFRSF19
HNRNPH2	8.3E-293	0.356546	0.272	0.076	1.3E-288	Skin_Kerat	HNRNPH2
PAK1IP1	1.1E-292	0.360858	0.209	0.049	1.7E-288	Skin_Kerat	PAK1IP1
CERS3	1.1E-292	0.263451	0.135	0.022	1.7E-288	Skin_Kerat	CERS3
NFE2L24	5.3E-291	0.59938	0.595	0.278	8.5E-287	Skin_Kerat	NFE2L2
PTS3	6.7E-291	0.558431	0.366	0.13	1.1E-286	Skin_Kerat	PTS
SRSF13	1.3E-290	0.468368	0.402	0.146	2.1E-286	Skin_Kerat	SRSF1
MARCH63	2.1E-290	0.542497	0.415	0.157	3.4E-286	Skin_Kerat	MARCH6
LAMB32	2.5E-290	0.443874	0.229	0.057	4.1E-286	Skin_Kerat	LAMB3
AMOTL21	1.1E-289	0.427285	0.287	0.083	1.8E-285	Skin_Kerat	AMOTL2
KPNA42	1.6E-288	0.476814	0.387	0.139	2.5E-284	Skin_Kerat	KPNA4
DSC25	1.3E-287	0.488554	0.236	0.063	2.1E-283	Skin_Kerat	DSC2
MOB44	1.6E-287	0.352685	0.293	0.086	2.5E-283	Skin_Kerat	MOB4
MAP4K52	1.6E-287	0.403672	0.279	0.081	2.5E-283	Skin_Kerat	MAP4K5
THOC6	3.6E-287	0.296788	0.216	0.052	5.8E-283	Skin_Kerat	THOC6
DCUN1D5	1.7E-286	0.424632	0.285	0.084	2.8E-282	Skin_Kerat	DCUN1D5
MAX	3.1E-286	0.528242	0.438	0.171	5E-282	Skin_Kerat	MAX
MAFK1	1.2E-285	0.333499	0.218	0.053	1.9E-281	Skin_Kerat	MAFK
PPP4R21	1.3E-285	0.455285	0.352	0.12	2.1E-281	Skin_Kerat	PPP4R2
MIPEP1	1.6E-285	0.279377	0.155	0.03	2.5E-281	Skin_Kerat	MIPEP
EIF4A24	6.3E-285	0.606305	0.815	0.475	1E-280	Skin_Kerat	EIF4A2
RBM8A3	3.5E-284	0.586607	0.66	0.333	5.5E-280	Skin_Kerat	RBM8A
PITHD1	5.3E-284	0.439397	0.385	0.138	8.4E-280	Skin_Kerat	PITHD1
PURB1	7.2E-284	0.406529	0.273	0.079	1.2E-279	Skin_Kerat	PURB
PPP1R13L1	1E-283	0.383706	0.248	0.067	1.6E-279	Skin_Kerat	PPP1R13L
TOMM5	1.6E-283	0.57071	0.382	0.139	2.6E-279	Skin_Kerat	TOMM5
RIOK1	1.8E-283	0.318622	0.217	0.053	2.8E-279	Skin_Kerat	RIOK1
ZC3H15	9.1E-282	0.543282	0.511	0.22	1.5E-277	Skin_Kerat	ZC3H15
ZBTB43	6.1E-281	0.448593	0.316	0.101	9.8E-277	Skin_Kerat	ZBTB43

PHF231	9.4E-281	0.413702	0.296	0.091	1.5E-276	Skin_Kerat	PHF23
SLC44A13	1.3E-280	0.297295	0.267	0.074	2.1E-276	Skin_Kerat	SLC44A1
FOXN34	2.4E-280	0.407557	0.352	0.118	3.8E-276	Skin_Kerat	FOXN3
SPOPL	3.3E-280	0.341239	0.201	0.048	5.3E-276	Skin_Kerat	SPOPL
SLC25A63	9.3E-280	0.50883	0.914	0.557	1.5E-275	Skin_Kerat	SLC25A6
NHP2	1.4E-279	0.500392	0.521	0.222	2.2E-275	Skin_Kerat	NHP2
SUB15	1.8E-279	0.535773	0.813	0.47	2.8E-275	Skin_Kerat	SUB1
KLF103	4.3E-279	0.447935	0.384	0.134	6.9E-275	Skin_Kerat	KLF10
RPL183	1.5E-278	0.399624	0.996	0.875	2.3E-274	Skin_Kerat	RPL18
LRRC42	5.5E-277	0.29815	0.174	0.037	8.8E-273	Skin_Kerat	LRRC42
KCTD91	1E-276	0.397567	0.247	0.068	1.6E-272	Skin_Kerat	KCTD9
CHMP4B3	2.5E-276	0.544501	0.633	0.296	4E-272	Skin_Kerat	CHMP4B
STX6	2.5E-275	0.285961	0.166	0.034	3.9E-271	Skin_Kerat	STX6
CEBPZ1	7.7E-275	0.471974	0.338	0.114	1.2E-270	Skin_Kerat	CEBPZ
CNIH12	9.5E-275	0.392581	0.358	0.122	1.5E-270	Skin_Kerat	CNIH1
UGDH3	1E-274	0.442561	0.247	0.068	1.6E-270	Skin_Kerat	UGDH
SLC7A52	1.7E-274	0.353213	0.207	0.05	2.8E-270	Skin_Kerat	SLC7A5
ISG203	5.7E-274	0.397835	0.493	0.193	9.1E-270	Skin_Kerat	ISG20
EBNA1BP2	4.1E-273	0.507624	0.338	0.116	6.5E-269	Skin_Kerat	EBNA1BP2
LSS	1.9E-272	0.298097	0.167	0.035	3E-268	Skin_Kerat	LSS
YTHDF21	3.6E-272	0.510525	0.423	0.168	5.8E-268	Skin_Kerat	YTHDF2
DEK7	1.4E-271	0.505914	0.555	0.25	2.2E-267	Skin_Kerat	DEK
IMP41	1.4E-271	0.404338	0.36	0.127	2.2E-267	Skin_Kerat	IMP4
MED19	1.1E-270	0.43466	0.296	0.093	1.8E-266	Skin_Kerat	MED19
EHD43	2E-270	0.3723	0.283	0.085	3.2E-266	Skin_Kerat	EHD4
SON8	2.3E-270	0.572524	0.742	0.399	3.7E-266	Skin_Kerat	SON
ILF21	2.9E-270	0.489889	0.476	0.197	4.6E-266	Skin_Kerat	ILF2
PDLIM46	3.3E-270	0.357794	0.314	0.099	5.2E-266	Skin_Kerat	PDLIM4
POMP4	4.3E-270	0.558685	0.744	0.392	6.8E-266	Skin_Kerat	POMP
SPAG94	5.9E-270	0.512746	0.458	0.185	9.4E-266	Skin_Kerat	SPAG9
LAPTM4A4	1.4E-269	0.431448	0.866	0.508	2.2E-265	Skin_Kerat	LAPTM4A
VEZT1	1.4E-269	0.38374	0.26	0.075	2.3E-265	Skin_Kerat	VEZT
MAP2K31	3.9E-269	0.457645	0.531	0.232	6.2E-265	Skin_Kerat	MAP2K3
WAC2	1.3E-268	0.516709	0.478	0.204	2E-264	Skin_Kerat	WAC
JMJD63	3.3E-268	0.355614	0.281	0.085	5.2E-264	Skin_Kerat	JMJD6
NUP50	1.6E-267	0.427843	0.298	0.095	2.5E-263	Skin_Kerat	NUP50
RPL22L12	1.7E-267	0.461506	0.495	0.21	2.7E-263	Skin_Kerat	RPL22L1
PLEKHB22	1E-266	0.494986	0.383	0.142	1.7E-262	Skin_Kerat	PLEKHB2
TOMM22	1.9E-265	0.458101	0.483	0.204	3E-261	Skin_Kerat	TOMM22
HNRNPK3	2E-265	0.579426	0.77	0.436	3.2E-261	Skin_Kerat	HNRNPK
CCDC86	2.2E-265	0.266228	0.146	0.028	3.5E-261	Skin_Kerat	CCDC86
CISD12	2.7E-265	0.461368	0.362	0.13	4.3E-261	Skin_Kerat	CISD1
KIF13A4	5.4E-265	0.317203	0.194	0.047	8.6E-261	Skin_Kerat	KIF13A
PNP2	3E-264	0.334386	0.25	0.07	4.8E-260	Skin_Kerat	PNP
CELF13	3.1E-264	0.396006	0.341	0.118	4.9E-260	Skin_Kerat	CELF1
MGME1	4.9E-264	0.275168	0.184	0.042	7.9E-260	Skin_Kerat	MGME1
AHNAK9	4E-262	0.417012	0.729	0.375	6.4E-258	Skin_Kerat	AHNAK

ITGA23	4E-262	0.445884	0.273	0.083	6.4E-258	Skin_Kerat	ITGA2
PDCD46	1.2E-261	0.433747	0.441	0.176	1.8E-257	Skin_Kerat	PDCD4
DNAJB95	1.5E-261	0.461648	0.479	0.199	2.4E-257	Skin_Kerat	DNAJB9
CNBP3	2.5E-261	0.491174	0.758	0.417	4.1E-257	Skin_Kerat	CNBP
FGFR1OP2	3.9E-261	0.422589	0.394	0.147	6.1E-257	Skin_Kerat	FGFR1OP2
SSFA25	2.4E-260	0.577761	0.402	0.16	3.9E-256	Skin_Kerat	SSFA2
QKI5	2.7E-260	0.442792	0.423	0.164	4.3E-256	Skin_Kerat	QKI
PSMA42	3.4E-260	0.503701	0.548	0.247	5.5E-256	Skin_Kerat	PSMA4
KDM7A3	4.5E-260	0.312346	0.19	0.045	7.2E-256	Skin_Kerat	KDM7A
TRA2B3	5.6E-260	0.492375	0.593	0.28	8.9E-256	Skin_Kerat	TRA2B
RPF2	8.7E-260	0.394391	0.26	0.077	1.4E-255	Skin_Kerat	RPF2
DYNLL15	1.3E-259	0.640917	0.846	0.552	2.1E-255	Skin_Kerat	DYNLL1
MTPN5	3.1E-259	0.365058	0.389	0.142	5E-255	Skin_Kerat	MTPN
FAM210A	4.3E-259	0.313108	0.207	0.052	6.8E-255	Skin_Kerat	FAM210A
RPS163	5.1E-259	0.428033	0.996	0.831	8.1E-255	Skin_Kerat	RPS16
UBE2S6	5.4E-259	0.418691	0.354	0.125	8.5E-255	Skin_Kerat	UBE2S
SNRPE3	2E-258	0.48116	0.566	0.257	3.3E-254	Skin_Kerat	SNRPE
RPL294	4.5E-258	0.453882	0.993	0.824	7.2E-254	Skin_Kerat	RPL29
HNRNPA2E	1.5E-257	0.532149	0.835	0.481	2.4E-253	Skin_Kerat	HNRNPA2B1
RALGDS1	3.9E-257	0.343338	0.245	0.069	6.3E-253	Skin_Kerat	RALGDS
BHLHE403	1E-256	0.354855	0.461	0.179	1.7E-252	Skin_Kerat	BHLHE40
RCOR1	1.5E-256	0.378371	0.272	0.082	2.4E-252	Skin_Kerat	RCOR1
MRPL322	1.8E-256	0.414226	0.359	0.13	2.9E-252	Skin_Kerat	MRPL32
RLF	2.1E-256	0.358857	0.237	0.066	3.4E-252	Skin_Kerat	RLF
OGT3	1.2E-255	0.395506	0.304	0.1	1.8E-251	Skin_Kerat	OGT
CTNNB15	1.6E-255	0.715744	0.409	0.164	2.6E-251	Skin_Kerat	CTNNB1
PER23	5.3E-255	0.434594	0.263	0.08	8.4E-251	Skin_Kerat	PER2
VCL5	1.6E-254	0.391676	0.32	0.107	2.5E-250	Skin_Kerat	VCL
ARL8B1	1.7E-254	0.474352	0.438	0.179	2.7E-250	Skin_Kerat	ARL8B
GNL32	6.1E-254	0.532439	0.46	0.196	9.7E-250	Skin_Kerat	GNL3
GRWD1	1.9E-253	0.264381	0.181	0.042	3.1E-249	Skin_Kerat	GRWD1
YRDC	2.2E-253	0.254663	0.196	0.048	3.5E-249	Skin_Kerat	YRDC
TRIP63	1E-252	0.475485	0.361	0.133	1.6E-248	Skin_Kerat	TRIP6
MBP2	2.9E-251	0.398779	0.39	0.146	4.6E-247	Skin_Kerat	MBP
TERF2IP4	5.5E-251	0.433105	0.501	0.216	8.8E-247	Skin_Kerat	TERF2IP
MORC32	3.8E-250	0.362033	0.273	0.084	6E-246	Skin_Kerat	MORC3
SYNCRIP5	2.4E-249	0.519667	0.427	0.178	3.8E-245	Skin_Kerat	SYNCRIP
KPNA23	3.8E-249	0.272463	0.312	0.101	6.1E-245	Skin_Kerat	KPNA2
TPD526	6.4E-249	0.372192	0.338	0.12	1E-244	Skin_Kerat	TPD52
OAT7	1.2E-248	0.475776	0.41	0.164	2E-244	Skin_Kerat	OAT
RPL95	2.2E-248	0.418447	0.994	0.743	3.5E-244	Skin_Kerat	RPL9
CTR9	6.6E-248	0.409264	0.277	0.088	1E-243	Skin_Kerat	CTR9
HSPE15	7.7E-248	0.463157	0.7	0.362	1.2E-243	Skin_Kerat	HSPE1
HSP90AB1:	6.8E-247	0.510334	0.884	0.56	1.1E-242	Skin_Kerat	HSP90AB1
EIF2A2	1.1E-246	0.395175	0.382	0.146	1.8E-242	Skin_Kerat	EIF2A
FKBP41	1.8E-246	0.488769	0.405	0.163	2.8E-242	Skin_Kerat	FKBP4
ZBTB111	2.7E-246	0.28973	0.189	0.046	4.4E-242	Skin_Kerat	ZBTB11

GTF3C41	2.9E-246	0.253568	0.143	0.029	4.6E-242	Skin_Kerat	GTF3C4
ARHGEF35	1.3E-245	0.357161	0.182	0.045	2.1E-241	Skin_Kerat	ARHGEF35
CDK7	3.5E-245	0.354015	0.188	0.047	5.5E-241	Skin_Kerat	CDK7
ERH4	6.7E-245	0.471416	0.62	0.302	1.1E-240	Skin_Kerat	ERH
THAP9.AS1	5E-243	0.361281	0.25	0.074	8.1E-239	Skin_Kerat	THAP9.AS1
UGCG3	7.9E-243	0.352644	0.312	0.105	1.3E-238	Skin_Kerat	UGCG
SLC3A24	1.6E-242	0.44956	0.453	0.189	2.6E-238	Skin_Kerat	SLC3A2
PITPNB2	2.1E-242	0.440207	0.318	0.111	3.3E-238	Skin_Kerat	PITPNB
NOM1	6.9E-242	0.281629	0.158	0.035	1.1E-237	Skin_Kerat	NOM1
ZNF581	7.2E-242	0.263338	0.262	0.079	1.1E-237	Skin_Kerat	ZNF581
LARP4	2.8E-241	0.39521	0.26	0.081	4.5E-237	Skin_Kerat	LARP4
IGSF82	2E-240	0.251243	0.203	0.053	3.2E-236	Skin_Kerat	IGSF8
RRP15	4.1E-240	0.38159	0.22	0.061	6.5E-236	Skin_Kerat	RRP15
PPIA3	1.5E-239	0.537497	0.875	0.581	2.4E-235	Skin_Kerat	PPIA
EBPL2	3.9E-239	0.308676	0.351	0.128	6.1E-235	Skin_Kerat	EBPL
TMTC33	4.6E-239	0.253361	0.155	0.034	7.4E-235	Skin_Kerat	TMTC3
ADK	5.3E-239	0.390017	0.31	0.107	8.4E-235	Skin_Kerat	ADK
FAS1	1.1E-238	0.270316	0.176	0.041	1.8E-234	Skin_Kerat	FAS
ELMSAN14	2.4E-238	0.369055	0.247	0.073	3.9E-234	Skin_Kerat	ELMSAN1
EI242	5.5E-238	0.469046	0.425	0.18	8.8E-234	Skin_Kerat	EI24
SIAH12	9.9E-238	0.379602	0.284	0.093	1.6E-233	Skin_Kerat	SIAH1
RBX16	2.3E-237	0.477422	0.656	0.339	3.7E-233	Skin_Kerat	RBX1
KLF66	4.6E-237	0.330497	0.742	0.391	7.4E-233	Skin_Kerat	KLF6
CAMSAP23	9E-237	0.349358	0.235	0.068	1.4E-232	Skin_Kerat	CAMSAP2
AK2	9.2E-237	0.476983	0.369	0.146	1.5E-232	Skin_Kerat	AK2
GNL2	9.2E-236	0.393379	0.224	0.064	1.5E-231	Skin_Kerat	GNL2
COBLL14	2.3E-235	0.439445	0.281	0.094	3.7E-231	Skin_Kerat	COBLL1
ARL5A3	5.8E-235	0.423176	0.442	0.188	9.2E-231	Skin_Kerat	ARL5A
C19orf48	1.1E-234	0.299244	0.215	0.059	1.7E-230	Skin_Kerat	C19orf48
PTGES35	2.3E-233	0.49615	0.708	0.383	3.6E-229	Skin_Kerat	PTGES3
PIGA2	3.7E-233	0.272444	0.168	0.039	5.9E-229	Skin_Kerat	PIGA
PPP1CC2	6.8E-233	0.399489	0.526	0.24	1.1E-228	Skin_Kerat	PPP1CC
PUM2	1.4E-231	0.409418	0.309	0.109	2.3E-227	Skin_Kerat	PUM2
TIMM102	1.2E-230	0.433423	0.378	0.148	1.9E-226	Skin_Kerat	TIMM10
KLHL212	4E-229	0.337185	0.241	0.073	6.4E-225	Skin_Kerat	KLHL21
COPS21	4.6E-229	0.396324	0.326	0.119	7.3E-225	Skin_Kerat	COPS2
CA25	1.2E-228	0.377988	0.281	0.092	2E-224	Skin_Kerat	CA2
LAMTOR41	7.7E-228	0.484723	0.689	0.38	1.2E-223	Skin_Kerat	LAMTOR4
POLR1D	8.2E-228	0.498461	0.614	0.323	1.3E-223	Skin_Kerat	POLR1D
SELK5	1.5E-227	0.396912	0.787	0.459	2.3E-223	Skin_Kerat	SELK
ZFAND12	1.7E-227	0.341586	0.307	0.107	2.7E-223	Skin_Kerat	ZFAND1
NOC2L1	1.2E-226	0.314539	0.254	0.08	1.9E-222	Skin_Kerat	NOC2L
TAGLN27	2.2E-226	0.30993	0.775	0.397	3.5E-222	Skin_Kerat	TAGLN2
POLR2F2	3.8E-226	0.470946	0.516	0.244	6.1E-222	Skin_Kerat	POLR2F
PGAM15	1.1E-225	0.437469	0.486	0.221	1.8E-221	Skin_Kerat	PGAM1
PFKFB33	2E-225	0.346648	0.278	0.09	3.1E-221	Skin_Kerat	PFKFB3
MAP3K84	1.4E-224	0.304716	0.32	0.11	2.2E-220	Skin_Kerat	MAP3K8

ENOPH1	1.9E-224	0.308556	0.246	0.076	3.1E-220	Skin_Kerat	ENOPH1
CHD24	1.4E-222	0.417776	0.457	0.199	2.3E-218	Skin_Kerat	CHD2
GART	2.3E-222	0.284206	0.182	0.047	3.7E-218	Skin_Kerat	GART
B3GNT51	2.8E-222	0.326972	0.266	0.086	4.4E-218	Skin_Kerat	B3GNT5
BARX24	5.1E-222	0.385716	0.205	0.059	8.2E-218	Skin_Kerat	BARX2
SARS3	1.1E-221	0.370926	0.375	0.149	1.7E-217	Skin_Kerat	SARS
PLEKHF2	1.2E-221	0.306482	0.233	0.07	1.9E-217	Skin_Kerat	PLEKHF2
AFTPH	4.3E-221	0.431005	0.371	0.149	6.9E-217	Skin_Kerat	AFTPH
CAMTA12	5.2E-220	0.509842	0.613	0.325	8.3E-216	Skin_Kerat	CAMTA1
TAX1BP32	1.1E-219	0.286286	0.259	0.081	1.8E-215	Skin_Kerat	TAX1BP3
PLS3	3.8E-219	0.361573	0.397	0.156	6.1E-215	Skin_Kerat	PLS3
YWHAQ3	4.5E-219	0.493964	0.666	0.355	7.1E-215	Skin_Kerat	YWHAQ
RAB212	8E-219	0.335027	0.393	0.159	1.3E-214	Skin_Kerat	RAB21
SRRM29	2.1E-218	0.442522	0.637	0.339	3.4E-214	Skin_Kerat	SRRM2
POP71	2.6E-218	0.369861	0.343	0.133	4.1E-214	Skin_Kerat	POP7
PGRMC23	7.8E-218	0.430668	0.345	0.135	1.2E-213	Skin_Kerat	PGRMC2
RNF1524	1.2E-217	0.26023	0.146	0.033	1.9E-213	Skin_Kerat	RNF152
TNKS21	4.1E-217	0.382264	0.309	0.113	6.6E-213	Skin_Kerat	TNKS2
TRMT1124	1.4E-216	0.438756	0.685	0.364	2.2E-212	Skin_Kerat	TRMT112
DNAJA2	2.6E-216	0.430861	0.379	0.155	4.2E-212	Skin_Kerat	DNAJA2
PRPF40A5	3.6E-216	0.412024	0.42	0.181	5.7E-212	Skin_Kerat	PRPF40A
CTTN2	4.5E-216	0.526608	0.444	0.2	7.2E-212	Skin_Kerat	CTTN
KLF31	4.8E-215	0.450184	0.422	0.182	7.7E-211	Skin_Kerat	KLF3
RNF111	4.9E-215	0.423219	0.451	0.201	7.8E-211	Skin_Kerat	RNF11
LSM56	7.8E-215	0.405045	0.443	0.194	1.2E-210	Skin_Kerat	LSM5
ATP13A32	4.8E-214	0.310273	0.267	0.088	7.7E-210	Skin_Kerat	ATP13A3
UBQLN1	1.3E-213	0.377411	0.324	0.123	2E-209	Skin_Kerat	UBQLN1
PVRL23	2.2E-213	0.430057	0.413	0.175	3.5E-209	Skin_Kerat	PVRL2
FUBP11	4.1E-213	0.360347	0.275	0.094	6.5E-209	Skin_Kerat	FUBP1
GAR1	1.1E-211	0.308481	0.223	0.067	1.7E-207	Skin_Kerat	GAR1
ANKRD50	1.6E-211	0.261037	0.144	0.033	2.5E-207	Skin_Kerat	ANKRD50
IL1RAP	2E-211	0.253723	0.171	0.042	3.1E-207	Skin_Kerat	IL1RAP
NME11	2.6E-211	0.387189	0.382	0.158	4.2E-207	Skin_Kerat	NME1
RALBP1	1.9E-210	0.451458	0.492	0.233	3E-206	Skin_Kerat	RALBP1
ECE2	3.4E-210	0.304506	0.16	0.039	5.4E-206	Skin_Kerat	ECE2
COQ10B2	8.3E-210	0.363641	0.356	0.14	1.3E-205	Skin_Kerat	COQ10B
ANKRD172	8.6E-210	0.36098	0.283	0.101	1.4E-205	Skin_Kerat	ANKRD17
MED13L1	1.6E-209	0.390125	0.328	0.125	2.5E-205	Skin_Kerat	MED13L
PSMD111	2.6E-209	0.403639	0.379	0.157	4.1E-205	Skin_Kerat	PSMD11
TNFRSF1A!	2.8E-209	0.311928	0.383	0.153	4.5E-205	Skin_Kerat	TNFRSF1A
EXOSC3	5.6E-209	0.2738	0.208	0.06	9E-205	Skin_Kerat	EXOSC3
LUZP12	7.5E-209	0.395169	0.318	0.118	1.2E-204	Skin_Kerat	LUZP1
EXOSC71	8.4E-209	0.271308	0.255	0.084	1.3E-204	Skin_Kerat	EXOSC7
EPB41L4A.	9.8E-209	0.332955	0.44	0.191	1.6E-204	Skin_Kerat	EPB41L4A.AS1
NGDN	1.7E-208	0.278205	0.252	0.082	2.8E-204	Skin_Kerat	NGDN
MKNK25	1.8E-208	0.339689	0.305	0.111	2.8E-204	Skin_Kerat	MKNK2
DCTPP1	4.7E-208	0.309111	0.286	0.101	7.5E-204	Skin_Kerat	DCTPP1

MPLKIP1	6.5E-208	0.389451	0.377	0.157	1E-203	Skin_Kerat	MPLKIP
ARGLU14	7.8E-208	0.476458	0.582	0.3	1.2E-203	Skin_Kerat	ARGLU1
MRPL42	8.2E-208	0.33692	0.308	0.114	1.3E-203	Skin_Kerat	MRPL4
NIP71	9.8E-208	0.292042	0.199	0.057	1.6E-203	Skin_Kerat	NIP7
RBM272	2.7E-207	0.29804	0.232	0.072	4.3E-203	Skin_Kerat	RBM27
NFKBIZ3	9.6E-207	0.461564	0.503	0.237	1.5E-202	Skin_Kerat	NFKBIZ
BUB32	3.5E-206	0.30988	0.404	0.168	5.5E-202	Skin_Kerat	BUB3
CMC21	4.9E-206	0.322657	0.309	0.114	7.8E-202	Skin_Kerat	CMC2
WDR333	9.2E-206	0.339596	0.359	0.145	1.5E-201	Skin_Kerat	WDR33
DNAJB112	2.1E-205	0.445844	0.378	0.16	3.4E-201	Skin_Kerat	DNAJB11
SNRPG1	3.7E-205	0.467228	0.646	0.345	5.9E-201	Skin_Kerat	SNRPG
CHORDC13	4.2E-205	0.346493	0.276	0.095	6.7E-201	Skin_Kerat	CHORDC1
TUBA1B8	6.6E-205	0.345077	0.674	0.344	1E-200	Skin_Kerat	TUBA1B
HNRNPA36	1.2E-204	0.456153	0.651	0.356	1.9E-200	Skin_Kerat	HNRNPA3
NCK13	1.3E-204	0.292729	0.291	0.103	2.1E-200	Skin_Kerat	NCK1
SLIRP2	3.2E-203	0.472257	0.516	0.251	5.1E-199	Skin_Kerat	SLIRP
SEC61G6	1.2E-202	0.459513	0.724	0.405	1.9E-198	Skin_Kerat	SEC61G
RAB18	1.4E-202	0.460474	0.518	0.256	2.2E-198	Skin_Kerat	RAB18
MAP3K15	1.5E-202	0.316224	0.21	0.063	2.4E-198	Skin_Kerat	MAP3K1
UBA22	2E-202	0.276879	0.305	0.11	3.2E-198	Skin_Kerat	UBA2
BTF3L4	2.3E-201	0.439674	0.408	0.181	3.7E-197	Skin_Kerat	BTF3L4
MAT2A6	2.6E-201	0.392439	0.355	0.144	4.1E-197	Skin_Kerat	MAT2A
HSPD17	3.2E-201	0.360467	0.524	0.249	5.1E-197	Skin_Kerat	HSPD1
DPM1	3.3E-201	0.421415	0.359	0.148	5.3E-197	Skin_Kerat	DPM1
RAPH13	4.2E-201	0.27865	0.18	0.049	6.8E-197	Skin_Kerat	RAPH1
EIF3M	2.5E-200	0.311163	0.526	0.251	4E-196	Skin_Kerat	EIF3M
PRDX18	3.3E-200	0.431835	0.764	0.443	5.3E-196	Skin_Kerat	PRDX1
PRELID12	6.6E-200	0.449896	0.665	0.37	1.1E-195	Skin_Kerat	PRELID1
BOD11	1.7E-199	0.316787	0.277	0.098	2.7E-195	Skin_Kerat	BOD1
TIMM17A2	2.6E-199	0.373163	0.328	0.129	4.1E-195	Skin_Kerat	TIMM17A
WNK11	3.6E-198	0.306374	0.373	0.154	5.7E-194	Skin_Kerat	WNK1
USP472	5.8E-198	0.341595	0.283	0.103	9.2E-194	Skin_Kerat	USP47
RPL105	1.3E-197	0.418592	1	0.874	2.1E-193	Skin_Kerat	RPL10
KDM5B	2.8E-197	0.357168	0.3	0.112	4.4E-193	Skin_Kerat	KDM5B
CYB5A6	7.1E-197	0.323854	0.408	0.175	1.1E-192	Skin_Kerat	CYB5A
TOP1	8E-197	0.386024	0.552	0.275	1.3E-192	Skin_Kerat	TOP1
GNB2L1	1.5E-196	0.286504	0.988	0.745	2.3E-192	Skin_Kerat	GNB2L1
AP3S12	1.3E-195	0.432517	0.55	0.284	2E-191	Skin_Kerat	AP3S1
SLCO3A15	1.4E-195	0.254568	0.182	0.05	2.2E-191	Skin_Kerat	SLCO3A1
GUK13	1.5E-195	0.465931	0.838	0.548	2.4E-191	Skin_Kerat	GUK1
PCMT13	2.8E-195	0.315048	0.46	0.209	4.5E-191	Skin_Kerat	PCMT1
ABHD13	3.7E-195	0.279943	0.185	0.052	5.9E-191	Skin_Kerat	ABHD13
FAM177A1	8.3E-195	0.374745	0.452	0.208	1.3E-190	Skin_Kerat	FAM177A1
PPP2R5E1	1.1E-194	0.352324	0.292	0.108	1.7E-190	Skin_Kerat	PPP2R5E
EXOSC4	2.5E-194	0.344289	0.31	0.119	4.1E-190	Skin_Kerat	EXOSC4
MYL66	3.6E-194	0.415644	0.98	0.8	5.8E-190	Skin_Kerat	MYL6
HERC3	5.5E-194	0.25447	0.173	0.047	8.8E-190	Skin_Kerat	HERC3

PSMB62	2.3E-193	0.401642	0.591	0.313	3.7E-189	Skin_Kerat	PSMB6
MAGOH1	2.3E-193	0.355059	0.395	0.17	3.7E-189	Skin_Kerat	MAGOH
SNRNP702	5.1E-193	0.363617	0.402	0.177	8.2E-189	Skin_Kerat	SNRNP70
RPL143	5.1E-192	0.291595	0.991	0.79	8.1E-188	Skin_Kerat	RPL14
PQBP14	2E-191	0.261591	0.363	0.147	3.1E-187	Skin_Kerat	PQBP1
ZBTB7A3	4.9E-191	0.306285	0.309	0.117	7.9E-187	Skin_Kerat	ZBTB7A
CCDC61	1.3E-190	0.362953	0.297	0.114	2.1E-186	Skin_Kerat	CCDC6
CAPZA12	1.8E-190	0.472832	0.535	0.27	2.9E-186	Skin_Kerat	CAPZA1
SLC7A12	2.6E-190	0.271414	0.169	0.046	4.1E-186	Skin_Kerat	SLC7A1
SETD53	3.4E-190	0.361734	0.296	0.113	5.3E-186	Skin_Kerat	SETD5
TFRC4	3.6E-190	0.266466	0.179	0.05	5.8E-186	Skin_Kerat	TFRC
RPL192	7.7E-190	0.276077	0.997	0.844	1.2E-185	Skin_Kerat	RPL19
MED21	1E-189	0.434506	0.336	0.139	1.7E-185	Skin_Kerat	MED21
CDC37L12	1E-189	0.321262	0.237	0.079	1.7E-185	Skin_Kerat	CDC37L1
FAM120A7	1.7E-189	0.357958	0.344	0.142	2.8E-185	Skin_Kerat	FAM120A
BAG4	2.2E-189	0.281089	0.162	0.043	3.4E-185	Skin_Kerat	BAG4
TOB12	3.5E-189	0.342849	0.55	0.274	5.6E-185	Skin_Kerat	TOB1
SLC30A74	4.2E-189	0.314673	0.234	0.078	6.6E-185	Skin_Kerat	SLC30A7
TPI14	6.4E-189	0.499926	0.812	0.526	1E-184	Skin_Kerat	TPI1
PSMD121	6.4E-189	0.372803	0.35	0.146	1E-184	Skin_Kerat	PSMD12
SRSF36	1.1E-188	0.404447	0.73	0.424	1.7E-184	Skin_Kerat	SRSF3
APRT4	3.3E-188	0.286041	0.683	0.359	5.2E-184	Skin_Kerat	APRT
PTPN15	6E-186	0.295385	0.234	0.078	9.6E-182	Skin_Kerat	PTPN1
PHF5A	1E-185	0.309699	0.291	0.11	1.6E-181	Skin_Kerat	PHF5A
MTDH7	1.3E-185	0.339792	0.582	0.301	2.1E-181	Skin_Kerat	MTDH
GPSM2	5.1E-185	0.273622	0.196	0.059	8.2E-181	Skin_Kerat	GPSM2
SRP93	8.1E-185	0.408888	0.613	0.333	1.3E-180	Skin_Kerat	SRP9
CDV33	9.3E-185	0.305414	0.389	0.168	1.5E-180	Skin_Kerat	CDV3
STAT37	1.3E-184	0.275246	0.418	0.184	2E-180	Skin_Kerat	STAT3
CBWD1	1.1E-183	0.267236	0.191	0.057	1.7E-179	Skin_Kerat	CBWD1
FAM126B	1.6E-183	0.279519	0.228	0.075	2.5E-179	Skin_Kerat	FAM126B
ITGB16	1.8E-183	0.71588	0.609	0.349	2.9E-179	Skin_Kerat	ITGB1
MZT1	7.1E-183	0.255755	0.215	0.068	1.1E-178	Skin_Kerat	MZT1
GMFB1	7.1E-183	0.287875	0.202	0.062	1.1E-178	Skin_Kerat	GMFB
SLAIN2	4.9E-181	0.355201	0.302	0.118	7.8E-177	Skin_Kerat	SLAIN2
SNRPD22	1.3E-180	0.283642	0.769	0.423	2.1E-176	Skin_Kerat	SNRPD2
BTG28	2.5E-180	0.34573	0.485	0.225	3.9E-176	Skin_Kerat	BTG2
BNIP24	1E-179	0.259285	0.307	0.119	1.6E-175	Skin_Kerat	BNIP2
SRSF65	1.4E-179	0.364158	0.334	0.139	2.2E-175	Skin_Kerat	SRSF6
STRA132	1.5E-179	0.332378	0.412	0.188	2.4E-175	Skin_Kerat	STRA13
ANKRD103	3.6E-179	0.251313	0.288	0.107	5.7E-175	Skin_Kerat	ANKRD10
PTPN143	5.8E-179	0.266812	0.182	0.054	9.3E-175	Skin_Kerat	PTPN14
EIF3L1	1.2E-178	0.261595	0.684	0.378	1.9E-174	Skin_Kerat	EIF3L
TANC11	1.5E-178	0.25412	0.184	0.054	2.3E-174	Skin_Kerat	TANC1
GTPBP4	4.5E-178	0.284272	0.233	0.08	7.2E-174	Skin_Kerat	GTPBP4
CDC425	1.4E-177	0.541089	0.757	0.461	2.2E-173	Skin_Kerat	CDC42
ESRP12	1.5E-177	0.312742	0.224	0.076	2.4E-173	Skin_Kerat	ESRP1

MOB1A4	1.9E-177	0.34787	0.399	0.18	3E-173	Skin_Kerat	MOB1A
TP53RK	4.2E-177	0.288694	0.216	0.071	6.8E-173	Skin_Kerat	TP53RK
PSME41	1.3E-176	0.340084	0.254	0.093	2E-172	Skin_Kerat	PSME4
SF15	2.3E-176	0.278627	0.473	0.227	3.7E-172	Skin_Kerat	SF1
TNIP21	2.6E-176	0.265098	0.246	0.086	4.1E-172	Skin_Kerat	TNIP2
SPPL3	3E-176	0.305011	0.239	0.084	4.8E-172	Skin_Kerat	SPPL3
FYTTD1	3.7E-176	0.31222	0.306	0.121	5.9E-172	Skin_Kerat	FYTTD1
AAMP	1.1E-175	0.332867	0.323	0.133	1.7E-171	Skin_Kerat	AAMP
LSM72	1.7E-174	0.260809	0.554	0.277	2.7E-170	Skin_Kerat	LSM7
ZBTB442	1.7E-174	0.299198	0.266	0.099	2.8E-170	Skin_Kerat	ZBTB44
SENP51	1.8E-174	0.320603	0.22	0.074	2.8E-170	Skin_Kerat	SENP5
PIP5K1A	1.1E-173	0.287617	0.211	0.069	1.7E-169	Skin_Kerat	PIP5K1A
SLBP1	3.5E-172	0.28659	0.336	0.14	5.5E-168	Skin_Kerat	SLBP
PDK11	1.7E-171	0.264613	0.189	0.058	2.7E-167	Skin_Kerat	PDK1
TIPARP1	2.2E-170	0.311692	0.428	0.195	3.5E-166	Skin_Kerat	TIPARP
C1orf351	2.7E-170	0.273548	0.258	0.094	4.3E-166	Skin_Kerat	C1orf35
BRD42	1.3E-169	0.33468	0.359	0.158	2.1E-165	Skin_Kerat	BRD4
METTL154	3.1E-169	0.25173	0.16	0.045	4.9E-165	Skin_Kerat	METTL15
IFRD21	1.3E-168	0.29871	0.25	0.092	2.1E-164	Skin_Kerat	IFRD2
HIGD1A	1.7E-168	0.379609	0.462	0.225	2.7E-164	Skin_Kerat	HIGD1A
RCC21	1.9E-168	0.269611	0.207	0.069	3.1E-164	Skin_Kerat	RCC2
MTHFD25	5.7E-168	0.302088	0.255	0.094	9.1E-164	Skin_Kerat	MTHFD2
SMIM151	2.3E-167	0.353383	0.294	0.12	3.7E-163	Skin_Kerat	SMIM15
SNX33	5.7E-166	0.355436	0.699	0.407	9.1E-162	Skin_Kerat	SNX3
RAB1A1	8.2E-166	0.41521	0.628	0.359	1.3E-161	Skin_Kerat	RAB1A
TAF91	9E-166	0.280918	0.442	0.209	1.4E-161	Skin_Kerat	TAF9
ARHGEF12	3.7E-165	0.334106	0.316	0.132	5.9E-161	Skin_Kerat	ARHGEF12
LIMK21	8E-165	0.263642	0.149	0.041	1.3E-160	Skin_Kerat	LIMK2
CPSF6	1.1E-164	0.298656	0.249	0.092	1.7E-160	Skin_Kerat	CPSF6
ATP5I2	2.7E-164	0.347089	0.656	0.363	4.4E-160	Skin_Kerat	ATP5I
LETM1	3.2E-164	0.268563	0.174	0.053	5.2E-160	Skin_Kerat	LETM1
MRPS121	5.7E-164	0.278577	0.32	0.134	9.2E-160	Skin_Kerat	MRPS12
ATP5G18	2.7E-162	0.266004	0.506	0.253	4.3E-158	Skin_Kerat	ATP5G1
GSTP16	3.3E-162	0.275246	0.878	0.547	5.2E-158	Skin_Kerat	GSTP1
NOP562	1.3E-161	0.274594	0.298	0.119	2.1E-157	Skin_Kerat	NOP56
CHMP5	3.9E-161	0.369963	0.544	0.286	6.2E-157	Skin_Kerat	CHMP5
MYH95	4E-161	0.267561	0.394	0.18	6.3E-157	Skin_Kerat	MYH9
TCEB12	7.5E-161	0.413821	0.62	0.35	1.2E-156	Skin_Kerat	TCEB1
PAICS	9E-161	0.26672	0.201	0.067	1.4E-156	Skin_Kerat	PAICS
SSRP1	1.4E-160	0.251508	0.266	0.102	2.2E-156	Skin_Kerat	SSRP1
LYST4	1.9E-160	0.285378	0.243	0.089	3E-156	Skin_Kerat	LYST
PUF601	2.7E-160	0.287188	0.345	0.151	4.3E-156	Skin_Kerat	PUF60
NUCKS17	3.3E-160	0.29726	0.536	0.276	5.3E-156	Skin_Kerat	NUCKS1
QTRT12	7.2E-160	0.25695	0.209	0.071	1.2E-155	Skin_Kerat	QTRT1
NTMT1	1E-159	0.269782	0.326	0.138	1.6E-155	Skin_Kerat	NTMT1
MSMO1	1.4E-159	0.303781	0.227	0.081	2.2E-155	Skin_Kerat	MSMO1
CYB5D2	1.8E-159	0.315645	0.198	0.066	2.9E-155	Skin_Kerat	CYB5D2

UTP11L	4E-159	0.32485	0.241	0.09	6.4E-155	Skin_Kerat	UTP11L
VDAC2	4.6E-159	0.373971	0.682	0.403	7.3E-155	Skin_Kerat	VDAC2
VEGFA4	7.7E-159	0.295232	0.226	0.08	1.2E-154	Skin_Kerat	VEGFA
PTPN123	8.6E-159	0.25178	0.268	0.103	1.4E-154	Skin_Kerat	PTPN12
TMEM11	1.7E-158	0.32888	0.295	0.122	2.7E-154	Skin_Kerat	TMEM11
NOP581	1.3E-157	0.257004	0.279	0.11	2.1E-153	Skin_Kerat	NOP58
GLUD13	4.2E-157	0.256119	0.356	0.157	6.7E-153	Skin_Kerat	GLUD1
ABCF1	6.2E-157	0.336645	0.307	0.13	9.8E-153	Skin_Kerat	ABCF1
RQCD13	2.3E-156	0.258242	0.191	0.062	3.7E-152	Skin_Kerat	RQCD1
LAMTOR5	2.5E-156	0.375097	0.634	0.365	4E-152	Skin_Kerat	LAMTOR5
TOMM75	4.2E-156	0.324004	0.879	0.573	6.6E-152	Skin_Kerat	TOMM7
ATP6V1B2:	8.1E-156	0.254571	0.231	0.083	1.3E-151	Skin_Kerat	ATP6V1B2
GTF2F2	9.4E-156	0.300709	0.25	0.095	1.5E-151	Skin_Kerat	GTF2F2
EIF3B1	3.4E-155	0.277181	0.262	0.103	5.5E-151	Skin_Kerat	EIF3B
DLG11	2.4E-154	0.292394	0.231	0.086	3.8E-150	Skin_Kerat	DLG1
VPS26A	1.1E-153	0.367821	0.375	0.177	1.8E-149	Skin_Kerat	VPS26A
CMSS1	1.5E-153	0.263821	0.247	0.092	2.4E-149	Skin_Kerat	CMSS1
FKBP1A3	2.8E-153	0.272391	0.605	0.329	4.5E-149	Skin_Kerat	FKBP1A
TFG3	3.9E-153	0.381707	0.378	0.182	6.3E-149	Skin_Kerat	TFG
GPBP14	4.4E-153	0.30981	0.576	0.313	7E-149	Skin_Kerat	GPBP1
TBL1XR12	8.9E-153	0.320673	0.331	0.145	1.4E-148	Skin_Kerat	TBL1XR1
CRB3	8.9E-152	0.28106	0.324	0.138	1.4E-147	Skin_Kerat	CRB3
OTUD41	2.5E-151	0.256717	0.223	0.08	4E-147	Skin_Kerat	OTUD4
KRAS	2.9E-151	0.333108	0.383	0.182	4.6E-147	Skin_Kerat	KRAS
KPNA6	2.3E-150	0.294514	0.264	0.105	3.6E-146	Skin_Kerat	KPNA6
ACTB4	2.4E-150	0.306456	0.997	0.868	3.9E-146	Skin_Kerat	ACTB
NOTCH23	6.2E-150	0.250123	0.24	0.09	9.8E-146	Skin_Kerat	NOTCH2
ATL21	5.2E-149	0.277144	0.178	0.058	8.3E-145	Skin_Kerat	ATL2
NCKAP13	5.5E-149	0.320904	0.324	0.144	8.8E-145	Skin_Kerat	NCKAP1
IFRD12	8.9E-149	0.308384	0.434	0.213	1.4E-144	Skin_Kerat	IFRD1
TFDP1	2.1E-147	0.26812	0.232	0.088	3.3E-143	Skin_Kerat	TFDP1
HSPB113	4.5E-147	0.285396	0.301	0.129	7.1E-143	Skin_Kerat	HSPB11
LUC7L37	1.7E-146	0.3123	0.492	0.259	2.7E-142	Skin_Kerat	LUC7L3
STX3	2.8E-146	0.255173	0.208	0.074	4.5E-142	Skin_Kerat	STX3
HCCS	3.4E-146	0.251865	0.195	0.067	5.4E-142	Skin_Kerat	HCCS
RBM171	1.6E-145	0.26594	0.376	0.175	2.6E-141	Skin_Kerat	RBM17
PTPRF7	2.7E-145	0.268659	0.256	0.102	4.3E-141	Skin_Kerat	PTPRF
MAPK6	4.1E-145	0.363425	0.352	0.163	6.5E-141	Skin_Kerat	MAPK6
ARID1A1	1.8E-144	0.275128	0.274	0.114	2.9E-140	Skin_Kerat	ARID1A
CDC266	1.9E-144	0.273052	0.286	0.122	3.1E-140	Skin_Kerat	CDC26
TMEM134:	3.6E-143	0.308324	0.355	0.169	5.7E-139	Skin_Kerat	TMEM134
SYPL17	8.3E-143	0.278724	0.492	0.256	1.3E-138	Skin_Kerat	SYPL1
USP38	1.4E-142	0.253777	0.21	0.075	2.3E-138	Skin_Kerat	USP38
UFM13	2.7E-142	0.365292	0.431	0.224	4.3E-138	Skin_Kerat	UFM1
RPL2410	2.9E-142	0.31009	0.986	0.796	4.6E-138	Skin_Kerat	RPL24
CCL202	3E-142	0.416964	0.105	0.025	4.8E-138	Skin_Kerat	CCL20
NDUFS57	9.1E-142	0.313138	0.73	0.446	1.5E-137	Skin_Kerat	NDUFS5

CCDC475	9.5E-142	0.316543	0.303	0.135	1.5E-137	Skin_Kerat	CCDC47
GOLGA7	2.8E-141	0.266045	0.362	0.169	4.5E-137	Skin_Kerat	GOLGA7
RBM35	2.8E-141	0.297341	0.71	0.431	4.5E-137	Skin_Kerat	RBM3
RASGEF1B3	7.8E-141	0.258408	0.184	0.062	1.2E-136	Skin_Kerat	RASGEF1B
SMNDC1	3E-140	0.270334	0.275	0.114	4.7E-136	Skin_Kerat	SMNDC1
ATP5B6	5.1E-140	0.340843	0.613	0.364	8.1E-136	Skin_Kerat	ATP5B
ENO13	9.8E-140	0.288546	0.685	0.403	1.6E-135	Skin_Kerat	ENO1
SMU1	2.8E-139	0.256914	0.215	0.08	4.5E-135	Skin_Kerat	SMU1
MAPRE12	3.2E-139	0.317516	0.357	0.17	5E-135	Skin_Kerat	MAPRE1
ZC3H11A4	3.9E-139	0.309923	0.343	0.161	6.2E-135	Skin_Kerat	ZC3H11A
RAB254	4.2E-139	0.349532	0.292	0.129	6.7E-135	Skin_Kerat	RAB25
CCND25	7.4E-139	0.2634	0.247	0.098	1.2E-134	Skin_Kerat	CCND2
LRRC1	1.2E-138	0.269398	0.209	0.077	2E-134	Skin_Kerat	LRRC1
IRF2BPL5	1.8E-138	0.287893	0.202	0.072	2.9E-134	Skin_Kerat	IRF2BPL
NEDD4L1	1.8E-138	0.346686	0.271	0.116	2.9E-134	Skin_Kerat	NEDD4L
ALDH27	3.3E-138	0.263777	0.28	0.119	5.3E-134	Skin_Kerat	ALDH2
TMEM332	5E-138	0.3335	0.257	0.107	7.9E-134	Skin_Kerat	TMEM33
ABCE12	1.6E-136	0.262622	0.235	0.092	2.5E-132	Skin_Kerat	ABCE1
TRAF4	1.6E-136	0.332365	0.341	0.162	2.6E-132	Skin_Kerat	TRAF4
LSM14A	2.9E-136	0.261344	0.387	0.189	4.6E-132	Skin_Kerat	LSM14A
NUFIP24	4E-136	0.282951	0.322	0.147	6.5E-132	Skin_Kerat	NUFIP2
ASH1L6	6.4E-136	0.272372	0.348	0.164	1E-131	Skin_Kerat	ASH1L
CCDC124	1E-135	0.273424	0.332	0.154	1.6E-131	Skin_Kerat	CCDC124
RYBP1	1.1E-135	0.299733	0.312	0.141	1.8E-131	Skin_Kerat	RYBP
GOLGA48	4.7E-135	0.263806	0.451	0.229	7.6E-131	Skin_Kerat	GOLGA4
MED131	2.3E-134	0.289476	0.284	0.124	3.7E-130	Skin_Kerat	MED13
STIP11	3E-133	0.264016	0.277	0.118	4.8E-129	Skin_Kerat	STIP1
RAB7A2	4.3E-133	0.319647	0.627	0.364	6.9E-129	Skin_Kerat	RAB7A
LARP12	9.1E-133	0.265157	0.264	0.112	1.5E-128	Skin_Kerat	LARP1
NUDT44	1.4E-132	0.26199	0.322	0.148	2.3E-128	Skin_Kerat	NUDT4
NONO2	2.5E-131	0.262974	0.377	0.185	3.9E-127	Skin_Kerat	NONO
MPST1	6E-130	0.291233	0.337	0.162	9.5E-126	Skin_Kerat	MPST
PDCD102	1E-129	0.316254	0.389	0.198	1.6E-125	Skin_Kerat	PDCD10
SRSF93	1.2E-129	0.322457	0.586	0.347	1.9E-125	Skin_Kerat	SRSF9
UBE2I3	1.3E-129	0.258263	0.477	0.257	2.1E-125	Skin_Kerat	UBE2I
BRD24	2E-129	0.269268	0.486	0.265	3.1E-125	Skin_Kerat	BRD2
MYO1B3	4.4E-129	0.251009	0.191	0.069	7E-125	Skin_Kerat	MYO1B
ATP6V0B6	5.7E-129	0.297813	0.635	0.38	9E-125	Skin_Kerat	ATP6V0B
KIF5B5	3.8E-128	0.326303	0.504	0.284	6.1E-124	Skin_Kerat	KIF5B
PRKCI2	1.5E-127	0.289795	0.222	0.088	2.4E-123	Skin_Kerat	PRKCI
WDR26	4.1E-127	0.266265	0.352	0.169	6.6E-123	Skin_Kerat	WDR26
CASZ12	8.7E-127	0.261134	0.172	0.06	1.4E-122	Skin_Kerat	CASZ1
PUM14	1.8E-126	0.255861	0.302	0.139	2.8E-122	Skin_Kerat	PUM1
ATP5G310	6.7E-126	0.253789	0.616	0.354	1.1E-121	Skin_Kerat	ATP5G3
HNRNPU3	6.8E-126	0.296951	0.584	0.342	1.1E-121	Skin_Kerat	HNRNPU
SKP15	1.9E-125	0.358463	0.825	0.563	3E-121	Skin_Kerat	SKP1
TRA2A4	3.3E-125	0.333789	0.332	0.159	5.2E-121	Skin_Kerat	TRA2A

UCHL3	6.7E-125	0.271893	0.266	0.117	1.1E-120	Skin_Kerat	UCHL3
FAM162A3	1.1E-124	0.277114	0.372	0.188	1.8E-120	Skin_Kerat	FAM162A
HMGB19	4.8E-124	0.303411	0.836	0.567	7.6E-120	Skin_Kerat	HMGB1
SLMAP2	5.5E-124	0.281261	0.29	0.132	8.8E-120	Skin_Kerat	SLMAP
HIF1A5	6.1E-124	0.258305	0.337	0.161	9.8E-120	Skin_Kerat	HIF1A
LIN7C	9.6E-124	0.256194	0.181	0.066	1.5E-119	Skin_Kerat	LIN7C
NSFL1C	1.2E-123	0.263526	0.286	0.128	1.9E-119	Skin_Kerat	NSFL1C
PCBP22	6.3E-123	0.250686	0.674	0.413	1E-118	Skin_Kerat	PCBP2
ARF43	2.1E-121	0.32545	0.664	0.41	3.4E-117	Skin_Kerat	ARF4
TMEM41B	3.9E-121	0.288767	0.251	0.108	6.3E-117	Skin_Kerat	TMEM41B
RRAS21	1E-120	0.291716	0.22	0.09	1.6E-116	Skin_Kerat	RRAS2
RTF11	1.2E-119	0.273429	0.444	0.239	2E-115	Skin_Kerat	RTF1
GJB2	2.5E-119	0.359915	0.135	0.043	4E-115	Skin_Kerat	GJB2
POLR2H1	7.2E-119	0.299787	0.3	0.142	1.2E-114	Skin_Kerat	POLR2H
PSMB55	5.5E-117	0.258059	0.432	0.235	8.8E-113	Skin_Kerat	PSMB5
SUDS3	3E-114	0.258714	0.264	0.118	4.7E-110	Skin_Kerat	SUDS3
MAP3K132	3.4E-114	0.315012	0.428	0.235	5.4E-110	Skin_Kerat	MAP3K13
NUS11	1.4E-113	0.270137	0.219	0.092	2.3E-109	Skin_Kerat	NUS1
ATP2A25	4E-113	0.271544	0.339	0.171	6.4E-109	Skin_Kerat	ATP2A2
MRPL361	2.7E-112	0.255988	0.362	0.185	4.3E-108	Skin_Kerat	MRPL36
RSRC2	9.8E-107	0.283361	0.504	0.294	1.6E-102	Skin_Kerat	RSRC2
HAT1	1.5E-105	0.261254	0.241	0.109	2.4E-101	Skin_Kerat	HAT1
CLIP11	3E-105	0.274801	0.331	0.169	4.8E-101	Skin_Kerat	CLIP1
CTNNAL12	2.4E-104	0.25352	0.207	0.086	3.8E-100	Skin_Kerat	CTNNAL1
NARS2	9.6E-103	0.256098	0.284	0.138	1.53E-98	Skin_Kerat	NARS
STAU11	1.1E-102	0.25207	0.374	0.2	1.83E-98	Skin_Kerat	STAU1
AHCYL15	2E-102	0.250576	0.316	0.161	3.11E-98	Skin_Kerat	AHCYL1
JMJD1C3	5.7E-100	0.258085	0.413	0.229	9.15E-96	Skin_Kerat	JMJD1C
COX7B8	5.9E-99	0.257338	0.647	0.402	9.43E-95	Skin_Kerat	COX7B
C14orf21	2.21E-95	0.253327	0.697	0.441	3.53E-91	Skin_Kerat	C14orf2
UBE2K	1.74E-94	0.272239	0.396	0.224	2.77E-90	Skin_Kerat	UBE2K
TMA72	4.77E-92	0.261813	0.893	0.653	7.61E-88	Skin_Kerat	TMA7
SUMO26	1.31E-82	0.254336	0.816	0.595	2.09E-78	Skin_Kerat	SUMO2
LOR	4.17E-61	1.006188	0.023	0.003	6.64E-57	Skin_Kerat	LOR
SPRY14	0	3.466555	0.821	0.079	0	Skin_Vascl	SPRY1
TSC22D15	0	3.338086	0.912	0.257	0	Skin_Vascl	TSC22D1
G0S21	0	3.295276	0.665	0.113	0	Skin_Vascl	G0S2
TM4SF11	0	3.221479	0.983	0.303	0	Skin_Vascl	TM4SF1
IL62	0	3.154458	0.592	0.034	0	Skin_Vascl	IL6
IFI271	0	3.109398	0.979	0.185	0	Skin_Vascl	IFI27
RCAN13	0	3.024737	0.725	0.062	0	Skin_Vascl	RCAN1
CTGF5	0	2.949905	0.705	0.102	0	Skin_Vascl	CTGF
SOCS36	0	2.873459	0.839	0.153	0	Skin_Vascl	SOCS3
AQP14	0	2.782608	0.872	0.082	0	Skin_Vascl	AQP1
ICAM14	0	2.519036	0.77	0.124	0	Skin_Vascl	ICAM1
CLDN51	0	2.48404	0.702	0.029	0	Skin_Vascl	CLDN5
GNG114	0	2.458809	0.823	0.102	0	Skin_Vascl	GNG11

MCTP13	0	2.441296	0.718	0.023	0	Skin_Vascl	MCTP1
ARRDC33	0	2.347709	0.826	0.234	0	Skin_Vascl	ARRDC3
TCF45	0	2.334758	0.834	0.162	0	Skin_Vascl	TCF4
RGS164	0	2.330944	0.679	0.078	0	Skin_Vascl	RGS16
SLC2A35	0	2.320213	0.805	0.118	0	Skin_Vascl	SLC2A3
IFITM35	0	2.286207	0.978	0.299	0	Skin_Vascl	IFITM3
RAMP22	0	2.270106	0.737	0.063	0	Skin_Vascl	RAMP2
PLVAP1	0	2.263038	0.682	0.02	0	Skin_Vascl	PLVAP
TNFAIP35	0	2.197784	0.692	0.152	0	Skin_Vascl	TNFAIP3
CAV17	0	2.167324	0.923	0.186	0	Skin_Vascl	CAV1
LMCD14	0	2.159772	0.687	0.074	0	Skin_Vascl	LMCD1
C2CD4B1	0	2.069799	0.469	0.012	0	Skin_Vascl	C2CD4B
ZFP364	0	2.053115	0.925	0.536	0	Skin_Vascl	ZFP36
NFKBIA5	0	2.015791	0.973	0.509	0	Skin_Vascl	NFKBIA
ETS24	0	2.013132	0.758	0.147	0	Skin_Vascl	ETS2
ADAMTS14	0	2.008646	0.578	0.052	0	Skin_Vascl	ADAMTS1
EMCN2	0	1.988443	0.664	0.028	0	Skin_Vascl	EMCN
SOD23	0	1.900947	0.887	0.402	0	Skin_Vascl	SOD2
STOM5	0	1.887986	0.743	0.143	0	Skin_Vascl	STOM
ADAMTS43	0	1.876109	0.55	0.024	0	Skin_Vascl	ADAMTS4
RND1	0	1.860402	0.483	0.053	0	Skin_Vascl	RND1
PDLIM15	0	1.845949	0.793	0.215	0	Skin_Vascl	PDLIM1
ARHGAP29	0	1.845296	0.665	0.105	0	Skin_Vascl	ARHGAP29
CCL25	0	1.810594	0.664	0.115	0	Skin_Vascl	CCL2
NDRG14	0	1.81001	0.618	0.136	0	Skin_Vascl	NDRG1
CDKN1A4	0	1.807678	0.832	0.25	0	Skin_Vascl	CDKN1A
PTMA6	0	1.790353	1	0.816	0	Skin_Vascl	PTMA
IFITM13	0	1.788761	0.776	0.148	0	Skin_Vascl	IFITM1
NCOA75	0	1.777011	0.572	0.1	0	Skin_Vascl	NCOA7
CXCL23	0	1.765619	0.614	0.156	0	Skin_Vascl	CXCL2
NEDD92	0	1.754152	0.588	0.065	0	Skin_Vascl	NEDD9
SDCBP4	0	1.753334	0.921	0.403	0	Skin_Vascl	SDCBP
ADIRF5	0	1.744962	0.98	0.426	0	Skin_Vascl	ADIRF
CRIP25	0	1.728627	0.758	0.195	0	Skin_Vascl	CRIP2
APOLD13	0	1.686529	0.475	0.023	0	Skin_Vascl	APOLD1
HBEGF2	0	1.683793	0.472	0.042	0	Skin_Vascl	HBEGF
CD932	0	1.681675	0.556	0.023	0	Skin_Vascl	CD93
MYCT11	0	1.679198	0.548	0.016	0	Skin_Vascl	MYCT1
RPS4Y18	0	1.674556	0.856	0.263	0	Skin_Vascl	RPS4Y1
MED241	0	1.672582	0.327	0.036	0	Skin_Vascl	MED24
SOCS21	0	1.670603	0.528	0.051	0	Skin_Vascl	SOCS2
ARID5A4	0	1.66153	0.595	0.09	0	Skin_Vascl	ARID5A
CSRP24	0	1.658313	0.509	0.074	0	Skin_Vascl	CSRP2
NRN12	0	1.653353	0.561	0.028	0	Skin_Vascl	NRN1
KCTD124	0	1.649454	0.596	0.084	0	Skin_Vascl	KCTD12
ZNF385D2	0	1.638359	0.532	0.044	0	Skin_Vascl	ZNF385D
FKBP1A4	0	1.633847	0.834	0.329	0	Skin_Vascl	FKBP1A

RPL415	0	1.633136	1	0.859	0	Skin_Vascl	RPL41
MAST42	0	1.599812	0.54	0.125	0	Skin_Vascl	MAST4
NR4A14	0	1.598593	0.719	0.249	0	Skin_Vascl	NR4A1
RAMP31	0	1.592767	0.508	0.025	0	Skin_Vascl	RAMP3
ESAM3	0	1.57847	0.588	0.063	0	Skin_Vascl	ESAM
TSPAN71	0	1.562557	0.507	0.021	0	Skin_Vascl	TSPAN7
ARID5B7	0	1.535637	0.775	0.26	0	Skin_Vascl	ARID5B
BCAM4	0	1.535364	0.575	0.067	0	Skin_Vascl	BCAM
NBEAL15	0	1.524592	0.89	0.31	0	Skin_Vascl	NBEAL1
C4orf325	0	1.524255	0.517	0.074	0	Skin_Vascl	C4orf32
ITM2A6	0	1.521017	0.63	0.127	0	Skin_Vascl	ITM2A
EPAS13	0	1.520702	0.572	0.082	0	Skin_Vascl	EPAS1
NR4A26	0	1.515152	0.664	0.177	0	Skin_Vascl	NR4A2
CLEC2B8	0	1.50811	0.647	0.148	0	Skin_Vascl	CLEC2B
CD743	0	1.498995	0.993	0.352	0	Skin_Vascl	CD74
FOS9	0	1.483342	0.86	0.418	0	Skin_Vascl	FOS
CAV27	0	1.475206	0.627	0.111	0	Skin_Vascl	CAV2
NNMT3	0	1.467627	0.62	0.102	0	Skin_Vascl	NNMT
SAV13	0	1.459877	0.525	0.099	0	Skin_Vascl	SAV1
BIRC35	0	1.450207	0.599	0.147	0	Skin_Vascl	BIRC3
CD343	0	1.446187	0.548	0.059	0	Skin_Vascl	CD34
FAM198B3	0	1.444847	0.469	0.027	0	Skin_Vascl	FAM198B
ETS16	0	1.440562	0.544	0.068	0	Skin_Vascl	ETS1
TSHZ26	0	1.431453	0.574	0.102	0	Skin_Vascl	TSHZ2
H2AFZ5	0	1.430374	0.854	0.432	0	Skin_Vascl	H2AFZ
C10orf103	0	1.427898	0.432	0.063	0	Skin_Vascl	C10orf10
KMT2E5	0	1.424584	0.709	0.293	0	Skin_Vascl	KMT2E
YBX36	0	1.423674	0.884	0.489	0	Skin_Vascl	YBX3
UGCG4	0	1.423498	0.508	0.105	0	Skin_Vascl	UGCG
CLU4	0	1.420394	0.66	0.192	0	Skin_Vascl	CLU
HIPK33	0	1.417425	0.565	0.111	0	Skin_Vascl	HIPK3
THBD4	0	1.413806	0.466	0.035	0	Skin_Vascl	THBD
DUSP13	0	1.411175	0.932	0.556	0	Skin_Vascl	DUSP1
EVA1C2	0	1.398859	0.493	0.065	0	Skin_Vascl	EVA1C
YWHAH6	0	1.392041	0.643	0.2	0	Skin_Vascl	YWHAH
COL15A13	0	1.386518	0.495	0.049	0	Skin_Vascl	COL15A1
PTRF7	0	1.38368	0.693	0.155	0	Skin_Vascl	PTRF
BTG16	0	1.380875	0.934	0.571	0	Skin_Vascl	BTG1
TMSB4X5	0	1.373904	1	0.869	0	Skin_Vascl	TMSB4X
WWTR12	0	1.353264	0.541	0.122	0	Skin_Vascl	WWTR1
LDHA6	0	1.345952	0.874	0.433	0	Skin_Vascl	LDHA
PMP226	0	1.345922	0.664	0.13	0	Skin_Vascl	PMP22
JAM21	0	1.3377	0.488	0.03	0	Skin_Vascl	JAM2
EDN12	0	1.333127	0.288	0.034	0	Skin_Vascl	EDN1
ANGPT23	0	1.327457	0.365	0.022	0	Skin_Vascl	ANGPT2
IFI166	0	1.31797	0.667	0.191	0	Skin_Vascl	IFI16
A2M5	0	1.310753	0.574	0.076	0	Skin_Vascl	A2M

TMEM703	0	1.309745	0.484	0.107	0	Skin_Vascl TMEM70
TGFBR24	0	1.307408	0.572	0.108	0	Skin_Vascl TGFBR2
DNAJB42	0	1.304462	0.49	0.099	0	Skin_Vascl DNAJB4
IGFBP45	0	1.286662	0.654	0.121	0	Skin_Vascl IGFBP4
HSPA1A8	0	1.285848	0.777	0.319	0	Skin_Vascl HSPA1A
RPL346	0	1.284922	1	0.917	0	Skin_Vascl RPL34
UPP1	0	1.28336	0.542	0.117	0	Skin_Vascl UPP1
ID35	0	1.278534	0.659	0.164	0	Skin_Vascl ID3
RBP71	0	1.277607	0.413	0.08	0	Skin_Vascl RBP7
C9orf37	0	1.267951	0.514	0.091	0	Skin_Vascl C9orf3
HIF1A6	0	1.26161	0.525	0.16	0	Skin_Vascl HIF1A
CLIC45	0	1.260358	0.537	0.118	0	Skin_Vascl CLIC4
SEPW18	0	1.255486	0.791	0.352	0	Skin_Vascl SEPW1
PPP3CA7	0	1.252381	0.548	0.149	0	Skin_Vascl PPP3CA
LIFR3	0	1.242477	0.466	0.056	0	Skin_Vascl LIFR
CRIM15	0	1.241787	0.479	0.072	0	Skin_Vascl CRIM1
EIF4A15	0	1.235302	0.868	0.344	0	Skin_Vascl EIF4A1
ELF14	0	1.218595	0.565	0.172	0	Skin_Vascl ELF1
CALCRL2	0	1.21759	0.426	0.027	0	Skin_Vascl CALCRL
FOXP15	0	1.2152	0.605	0.212	0	Skin_Vascl FOXP1
TMEM173	0	1.205366	0.541	0.103	0	Skin_Vascl TMEM173
IFITM26	0	1.200103	0.866	0.296	0	Skin_Vascl IFITM2
HSD17B11	0	1.192793	0.559	0.131	0	Skin_Vascl HSD17B11
CYYR11	0	1.190801	0.414	0.015	0	Skin_Vascl CYYR1
ACTN15	0	1.189745	0.53	0.141	0	Skin_Vascl ACTN1
ADRB22	0	1.178903	0.406	0.039	0	Skin_Vascl ADRB2
ARL5B5	0	1.175508	0.445	0.093	0	Skin_Vascl ARL5B
MSN9	0	1.171621	0.576	0.149	0	Skin_Vascl MSN
BMP22	0	1.16894	0.325	0.031	0	Skin_Vascl BMP2
PVRL24	0	1.165775	0.565	0.175	0	Skin_Vascl PVRL2
EGFL72	0	1.161719	0.43	0.073	0	Skin_Vascl EGFL7
CNN33	0	1.159197	0.633	0.218	0	Skin_Vascl CNN3
MYC3	0	1.156745	0.552	0.122	0	Skin_Vascl MYC
CNKSR3	0	1.155521	0.378	0.052	0	Skin_Vascl CNKSR3
VIM11	0	1.145406	0.981	0.486	0	Skin_Vascl VIM
HLA.E4	0	1.134404	0.948	0.604	0	Skin_Vascl HLA.E
ELMSAN15	0	1.134343	0.388	0.073	0	Skin_Vascl ELMSAN1
ATP1A111	0	1.134126	0.649	0.249	0	Skin_Vascl ATP1A1
S1PR11	0	1.114737	0.343	0.014	0	Skin_Vascl S1PR1
RAC15	0	1.10691	0.892	0.542	0	Skin_Vascl RAC1
RALGAPA2	0	1.101159	0.385	0.05	0	Skin_Vascl RALGAPA2
HLA.DRB52	0	1.09928	0.714	0.126	0	Skin_Vascl HLA.DRB5
INSIG13	0	1.096121	0.624	0.2	0	Skin_Vascl INSIG1
RPS185	0	1.091041	1	0.915	0	Skin_Vascl RPS18
GADD45A3	0	1.085068	0.642	0.207	0	Skin_Vascl GADD45A
VAMP53	0	1.08174	0.594	0.162	0	Skin_Vascl VAMP5
TFPI4	0	1.08134	0.44	0.074	0	Skin_Vascl TFPI

CREM3	0	1.072176	0.606	0.192	0	Skin_Vascl CREM
OAZ24	0	1.071239	0.548	0.172	0	Skin_Vascl OAZ2
DDX215	0	1.070791	0.572	0.193	0	Skin_Vascl DDX21
RPL3914	0	1.067898	0.991	0.672	0	Skin_Vascl RPL39
HOXD1	0	1.067647	0.332	0.012	0	Skin_Vascl HOXD1
ITGA610	0	1.062454	0.476	0.13	0	Skin_Vascl ITGA6
CCDC85B4	0	1.061926	0.715	0.265	0	Skin_Vascl CCDC85B
CSF2RB4	0	1.058619	0.333	0.033	0	Skin_Vascl CSF2RB
ADM51	0	1.052621	0.283	0.012	0	Skin_Vascl ADM5
SPARC6	0	1.050644	0.686	0.153	0	Skin_Vascl SPARC
NPDC17	0	1.046559	0.593	0.166	0	Skin_Vascl NPDC1
REL6	0	1.044534	0.602	0.179	0	Skin_Vascl REL
LPAR66	0	1.041587	0.515	0.135	0	Skin_Vascl LPAR6
EFNB22	0	1.038246	0.362	0.046	0	Skin_Vascl EFNB2
TAGLN28	0	1.023517	0.849	0.401	0	Skin_Vascl TAGLN2
IGFBP75	0	1.01423	0.886	0.254	0	Skin_Vascl IGFBP7
CDC42EP3:	0	1.014156	0.436	0.089	0	Skin_Vascl CDC42EP3
PTMS10	0	1.011721	0.666	0.237	0	Skin_Vascl PTMS
TMEM22	0	1.010261	0.372	0.054	0	Skin_Vascl TMEM2
HLA.DMA3	0	1.007746	0.628	0.131	0	Skin_Vascl HLA.DMA
SNCG3	0	1.004874	0.484	0.112	0	Skin_Vascl SNCG
POLE42	0	1.002645	0.551	0.159	0	Skin_Vascl POLE4
RNASE13	0	1.001849	0.546	0.086	0	Skin_Vascl RNASE1
RPS287	0	1.001113	0.999	0.879	0	Skin_Vascl RPS28
FNIP23	0	0.997656	0.367	0.086	0	Skin_Vascl FNIP2
ABL22	0	0.996949	0.377	0.085	0	Skin_Vascl ABL2
CYR616	0	0.994892	0.426	0.108	0	Skin_Vascl CYR61
KLF25	0	0.976424	0.594	0.185	0	Skin_Vascl KLF2
CYSTM16	0	0.974685	0.613	0.22	0	Skin_Vascl CYSTM1
IL3RA2	0	0.973613	0.334	0.015	0	Skin_Vascl IL3RA
ENPP23	0	0.966179	0.296	0.027	0	Skin_Vascl ENPP2
U2AF15	0	0.963968	0.675	0.248	0	Skin_Vascl U2AF1
SOX72	0	0.963519	0.296	0.017	0	Skin_Vascl SOX7
TMSB104	0	0.961241	1	0.877	0	Skin_Vascl TMSB10
SPTBN14	0	0.960416	0.577	0.192	0	Skin_Vascl SPTBN1
CEBPD3	0	0.953801	0.77	0.33	0	Skin_Vascl CEBPD
GBP21	0	0.948874	0.43	0.105	0	Skin_Vascl GBP2
KLF71	0	0.942986	0.34	0.043	0	Skin_Vascl KLF7
DLC15	0	0.942754	0.359	0.044	0	Skin_Vascl DLC1
ENG2	0	0.942487	0.409	0.052	0	Skin_Vascl ENG
ELK32	0	0.938643	0.392	0.055	0	Skin_Vascl ELK3
CNRIP16	0	0.935733	0.359	0.033	0	Skin_Vascl CNRIP1
CD3201	0	0.929236	0.421	0.09	0	Skin_Vascl CD320
SHC11	0	0.919095	0.365	0.062	0	Skin_Vascl SHC1
CTHRC11	0	0.909978	0.403	0.056	0	Skin_Vascl CTHRC1
RIN22	0	0.899919	0.324	0.069	0	Skin_Vascl RIN2
GIMAP62	0	0.896331	0.336	0.025	0	Skin_Vascl GIMAP6

KLF95	0	0.894725	0.43	0.101	0	Skin_Vascl KLF9
TIMP36	0	0.893941	0.67	0.158	0	Skin_Vascl TIMP3
MEF2C7	0	0.893894	0.431	0.075	0	Skin_Vascl MEF2C
EFNA13	0	0.891249	0.369	0.085	0	Skin_Vascl EFNA1
LMNA12	0	0.886311	0.837	0.358	0	Skin_Vascl LMNA
EDNRB4	0	0.882937	0.274	0.021	0	Skin_Vascl EDNRB
ZNF905	0	0.880943	0.393	0.09	0	Skin_Vascl ZNF90
IL1R14	0	0.880276	0.365	0.07	0	Skin_Vascl IL1R1
CTNNAL13	0	0.876707	0.375	0.085	0	Skin_Vascl CTNNAL1
PNP3	0	0.873156	0.36	0.071	0	Skin_Vascl PNP
MAP3K85	0	0.866632	0.446	0.111	0	Skin_Vascl MAP3K8
PTPRK1	0	0.864452	0.345	0.067	0	Skin_Vascl PTPRK
PDK42	0	0.862246	0.269	0.036	0	Skin_Vascl PDK4
AHR6	0	0.854164	0.421	0.103	0	Skin_Vascl AHR
CDH51	0	0.853914	0.311	0.021	0	Skin_Vascl CDH5
PRKCDBP5	0	0.850788	0.466	0.123	0	Skin_Vascl PRKCDBP
PODXL4	0	0.848192	0.306	0.046	0	Skin_Vascl PODXL
RP11.138A	0	0.842934	0.266	0.043	0	Skin_Vascl RP11.138A9.2
NRP16	0	0.833981	0.355	0.052	0	Skin_Vascl NRP1
HLA.DRA3	0	0.833293	0.942	0.24	0	Skin_Vascl HLA.DRA
EEF1A114	0	0.832962	0.998	0.914	0	Skin_Vascl EEF1A1
HLA.DRB13	0	0.830982	0.912	0.207	0	Skin_Vascl HLA.DRB1
RPS146	0	0.829185	0.999	0.88	0	Skin_Vascl RPS14
B2M5	0	0.825655	1	0.928	0	Skin_Vascl B2M
CPNE83	0	0.819079	0.276	0.037	0	Skin_Vascl CPNE8
HSPA22	0	0.816738	0.336	0.065	0	Skin_Vascl HSPA2
CLIC24	0	0.815903	0.337	0.042	0	Skin_Vascl CLIC2
ST8SIA44	0	0.810928	0.29	0.024	0	Skin_Vascl ST8SIA4
CD2002	0	0.8101	0.22	0.017	0	Skin_Vascl CD200
SNTB23	0	0.807002	0.34	0.076	0	Skin_Vascl SNTB2
NFIL33	0	0.806619	0.33	0.054	0	Skin_Vascl NFIL3
SEMA6A1	0	0.80637	0.264	0.025	0	Skin_Vascl SEMA6A
HLA.DPA13	0	0.806218	0.81	0.181	0	Skin_Vascl HLA.DPA1
TMEM255I	0	0.800498	0.274	0.016	0	Skin_Vascl TMEM255B
SPHK12	0	0.793	0.329	0.06	0	Skin_Vascl SPHK1
EBF15	0	0.785504	0.359	0.057	0	Skin_Vascl EBF1
GIMAP43	0	0.784651	0.418	0.069	0	Skin_Vascl GIMAP4
ELOVL72	0	0.782618	0.267	0.017	0	Skin_Vascl ELOVL7
LRRC324	0	0.781073	0.292	0.029	0	Skin_Vascl LRRC32
SSH1	0	0.771054	0.261	0.024	0	Skin_Vascl SSH1
ABCD41	0	0.769233	0.33	0.061	0	Skin_Vascl ABCD4
FAM13C3	0	0.768955	0.24	0.019	0	Skin_Vascl FAM13C
AP1S24	0	0.756879	0.469	0.12	0	Skin_Vascl AP1S2
HSPB15	0	0.752439	0.957	0.559	0	Skin_Vascl HSPB1
AAED16	0	0.745263	0.339	0.072	0	Skin_Vascl AAED1
MYLK3	0	0.735804	0.344	0.049	0	Skin_Vascl MYLK
AKR1C33	0	0.733457	0.304	0.056	0	Skin_Vascl AKR1C3

TM4SF181	0	0.7325	0.264	0.014	0	Skin_Vascl TM4SF18
NFKBID4	0	0.728378	0.31	0.04	0	Skin_Vascl NFKBID
HAPLN32	0	0.727079	0.274	0.031	0	Skin_Vascl HAPLN3
ITGA55	0	0.726809	0.3	0.035	0	Skin_Vascl ITGA5
THSD7A1	0	0.72411	0.237	0.013	0	Skin_Vascl THSD7A
KRT144	0	0.719081	0.757	0.139	0	Skin_Vascl KRT14
MSX14	0	0.705452	0.298	0.032	0	Skin_Vascl MSX1
ARHGEF31	0	0.685712	0.264	0.043	0	Skin_Vascl ARHGEF3
MCAM4	0	0.683303	0.279	0.025	0	Skin_Vascl MCAM
COL4A26	0	0.679773	0.282	0.051	0	Skin_Vascl COL4A2
VWF1	0	0.679416	0.308	0.035	0	Skin_Vascl VWF
HHEX2	0	0.677232	0.256	0.027	0	Skin_Vascl HHEX
TNFRSF10E	0	0.671886	0.256	0.04	0	Skin_Vascl TNFRSF10B
MMRN21	0	0.669787	0.255	0.018	0	Skin_Vascl MMRN2
IGFBP25	0	0.668276	0.3	0.049	0	Skin_Vascl IGFBP2
PTPRB1	0	0.665135	0.251	0.014	0	Skin_Vascl PTPRB
FLI12	0	0.660394	0.296	0.036	0	Skin_Vascl FLI1
IGF22	0	0.654932	0.245	0.03	0	Skin_Vascl IGF2
CD1093	0	0.650941	0.277	0.041	0	Skin_Vascl CD109
RP11.138A	0	0.645703	0.252	0.044	0	Skin_Vascl RP11.138A9.1
NUAK12	0	0.643816	0.245	0.033	0	Skin_Vascl NUAK1
NBL15	0	0.643136	0.426	0.106	0	Skin_Vascl NBL1
KDR1	0	0.639756	0.225	0.007	0	Skin_Vascl KDR
HLX1	0	0.63881	0.199	0.011	0	Skin_Vascl HLX
CLEC1A1	0	0.631401	0.217	0.006	0	Skin_Vascl CLEC1A
HLA.DPB12	0	0.631115	0.843	0.208	0	Skin_Vascl HLA.DPB1
NFATC24	0	0.618773	0.264	0.041	0	Skin_Vascl NFATC2
PLEKHG11	0	0.615704	0.204	0.019	0	Skin_Vascl PLEKHG1
LDB23	0	0.612943	0.258	0.039	0	Skin_Vascl LDB2
HLA.DQB1	0	0.598961	0.582	0.124	0	Skin_Vascl HLA.DQB1
LRRC8C3	0	0.598228	0.247	0.029	0	Skin_Vascl LRRC8C
HOXB7	0	0.594619	0.216	0.02	0	Skin_Vascl HOXB7
TSHZ12	0	0.586737	0.226	0.036	0	Skin_Vascl TSHZ1
ADCY41	0	0.583348	0.209	0.011	0	Skin_Vascl ADCY4
C10orf128	0	0.581267	0.228	0.018	0	Skin_Vascl C10orf128
ITPR13	0	0.580154	0.203	0.027	0	Skin_Vascl ITPR1
SHROOM4	0	0.575385	0.195	0.007	0	Skin_Vascl SHROOM4
FAM110D1	0	0.574488	0.184	0.009	0	Skin_Vascl FAM110D
FLT11	0	0.573644	0.25	0.035	0	Skin_Vascl FLT1
ANKRD294	0	0.567197	0.232	0.026	0	Skin_Vascl ANKRD29
CCL231	0	0.560999	0.14	0.005	0	Skin_Vascl CCL23
PTPRM1	0	0.550118	0.207	0.025	0	Skin_Vascl PTPRM
TCTEX1D1	0	0.517953	0.14	0.002	0	Skin_Vascl TCTEX1D1
RASIP11	0	0.51629	0.183	0.017	0	Skin_Vascl RASIP1
RNF122	0	0.513144	0.124	0.007	0	Skin_Vascl RNF122
GATA21	0	0.503525	0.184	0.022	0	Skin_Vascl GATA2
CLDN151	0	0.494323	0.178	0.02	0	Skin_Vascl CLDN15

LIMS25	0	0.481876	0.208	0.029	0	Skin_Vascl	LIMS2
SDPR3	0	0.478901	0.19	0.024	0	Skin_Vascl	SDPR
PCDH171	0	0.476884	0.169	0.015	0	Skin_Vascl	PCDH17
EMP1	0	0.471616	0.915	0.37	0	Skin_Vascl	EMP1
SHISA2	0	0.470634	0.129	0.006	0	Skin_Vascl	SHISA2
FAM72A	0	0.462486	0.132	0.011	0	Skin_Vascl	FAM72A
CYGB4	0	0.449481	0.182	0.023	0	Skin_Vascl	CYGB
KCNK52	0	0.412396	0.132	0.008	0	Skin_Vascl	KCNK5
GPR681	0	0.407021	0.144	0.008	0	Skin_Vascl	GPR68
HLA.DQB2	0	0.399645	0.215	0.023	0	Skin_Vascl	HLA.DQB2
DLL41	0	0.396792	0.119	0.005	0	Skin_Vascl	DLL4
KANK31	0	0.394887	0.158	0.015	0	Skin_Vascl	KANK3
TMTC11	0	0.394081	0.158	0.017	0	Skin_Vascl	TMTC1
ARHGAP31	0	0.392436	0.151	0.013	0	Skin_Vascl	ARHGAP31
PREX22	0	0.389683	0.163	0.019	0	Skin_Vascl	PREX2
GIMAP82	0	0.389148	0.145	0.012	0	Skin_Vascl	GIMAP8
CACHD1	0	0.376926	0.143	0.014	0	Skin_Vascl	CACHD1
TIE11	0	0.372744	0.134	0.009	0	Skin_Vascl	TIE1
PDE2A1	0	0.365523	0.127	0.01	0	Skin_Vascl	PDE2A
KRT53	0	0.355124	0.435	0.073	0	Skin_Vascl	KRT5
BCL6B1	0	0.346427	0.105	0.003	0	Skin_Vascl	BCL6B
SYT151	0	0.338552	0.117	0.007	0	Skin_Vascl	SYT15
RAPGEF41	0	0.335454	0.123	0.007	0	Skin_Vascl	RAPGEF4
SCARF12	0	0.314976	0.128	0.01	0	Skin_Vascl	SCARF1
SFN1	0	0.307399	0.57	0.14	0	Skin_Vascl	SFN
TEK1	0	0.296288	0.108	0.006	0	Skin_Vascl	TEK
HLA.DQA1	0	0.271145	0.476	0.101	0	Skin_Vascl	HLA.DQA1
LUCAT1	0	0.263596	0.085	0.005	0	Skin_Vascl	LUCAT1
SERTAD4.A	7.4E-308	0.681331	0.247	0.044	1.2E-303	Skin_Vascl	SERTAD4.AS1
TNFRSF10I	9.7E-308	0.366901	0.142	0.015	1.5E-303	Skin_Vascl	TNFRSF10D
WTAP4	7.6E-307	1.175342	0.666	0.275	1.2E-302	Skin_Vascl	WTAP
ANKRD284	2.1E-305	0.890947	0.403	0.105	3.3E-301	Skin_Vascl	ANKRD28
RUNDC3B1	4E-305	0.457712	0.149	0.017	6.3E-301	Skin_Vascl	RUNDC3B
CYTL11	8.9E-305	0.452919	0.155	0.018	1.4E-300	Skin_Vascl	CYTL1
CDC42EP2	1.1E-304	0.54428	0.215	0.034	1.8E-300	Skin_Vascl	CDC42EP2
SWAP701	1.1E-302	0.944003	0.455	0.135	1.7E-298	Skin_Vascl	SWAP70
MT2A3	5.6E-302	0.745606	0.741	0.3	8.9E-298	Skin_Vascl	MT2A
SAT11	2.3E-301	1.009899	0.973	0.606	3.7E-297	Skin_Vascl	SAT1
GADD45B3	1.2E-298	1.207622	0.79	0.37	1.9E-294	Skin_Vascl	GADD45B
DCUN1D31	1.6E-297	0.538902	0.203	0.031	2.6E-293	Skin_Vascl	DCUN1D3
LUZP13	2.5E-296	0.844573	0.424	0.119	4E-292	Skin_Vascl	LUZP1
DDIT4L2	1.1E-295	0.320785	0.093	0.007	1.8E-291	Skin_Vascl	DDIT4L
RHOC6	2.3E-295	0.93417	0.602	0.218	3.7E-291	Skin_Vascl	RHOC
OLFML2A5	4.5E-295	0.364068	0.167	0.021	7.3E-291	Skin_Vascl	OLFML2A
STAT38	5.2E-295	0.918085	0.542	0.185	8.3E-291	Skin_Vascl	STAT3
GRASP1	1E-293	0.377963	0.16	0.02	1.6E-289	Skin_Vascl	GRASP
BAALC1	1.6E-293	0.362338	0.133	0.014	2.6E-289	Skin_Vascl	BAALC

C8orf48	2.3E-293	1.518265	0.414	0.115	3.7E-289	Skin_Vascl	C8orf4
FZD62	1.6E-291	0.694997	0.212	0.035	2.6E-287	Skin_Vascl	FZD6
BHLHE404	5.6E-291	0.959231	0.545	0.182	8.9E-287	Skin_Vascl	BHLHE40
ELMO13	7.5E-291	0.426348	0.2	0.03	1.2E-286	Skin_Vascl	ELMO1
KLF67	1.9E-290	1.072833	0.8	0.395	3E-286	Skin_Vascl	KLF6
RPL316	2.4E-289	0.770993	1	0.841	3.8E-285	Skin_Vascl	RPL3
MKL22	3E-289	0.624915	0.243	0.045	4.8E-285	Skin_Vascl	MKL2
SERPINE12	5.3E-289	0.856675	0.146	0.017	8.5E-285	Skin_Vascl	SERPINE1
SGK13	1.4E-288	1.03434	0.397	0.108	2.3E-284	Skin_Vascl	SGK1
KDM6B5	4.3E-288	0.841483	0.411	0.114	6.9E-284	Skin_Vascl	KDM6B
DUSP233	1.3E-287	0.989046	0.464	0.147	2E-283	Skin_Vascl	DUSP23
PTPN124	3.7E-287	0.883375	0.384	0.103	5.9E-283	Skin_Vascl	PTPN12
RALGDS2	4.7E-286	0.741353	0.313	0.07	7.5E-282	Skin_Vascl	RALGDS
HNRNPH16	3.1E-285	1.256294	0.668	0.292	4.9E-281	Skin_Vascl	HNRNPH1
CD911	3.1E-285	0.982039	0.868	0.446	5E-281	Skin_Vascl	CD9
ANKRD104	3.2E-285	0.811592	0.398	0.108	5.2E-281	Skin_Vascl	ANKRD10
DOCK43	1.9E-284	0.408329	0.155	0.019	3E-280	Skin_Vascl	DOCK4
CYP1B11	5E-284	0.806278	0.254	0.048	8E-280	Skin_Vascl	CYP1B1
RPL75	2.1E-281	0.831127	0.998	0.794	3.3E-277	Skin_Vascl	RPL7
ID13	7.8E-278	0.656005	0.396	0.104	1.2E-273	Skin_Vascl	ID1
LMO22	3.9E-277	0.782105	0.347	0.086	6.3E-273	Skin_Vascl	LMO2
FOSL24	6.8E-277	1.031502	0.576	0.214	1.1E-272	Skin_Vascl	FOSL2
YWHAE4	1E-276	1.083653	0.764	0.392	1.6E-272	Skin_Vascl	YWHAE
RPL325	1E-276	0.755879	1	0.87	1.6E-272	Skin_Vascl	RPL32
AMD15	2.5E-276	1.008416	0.528	0.189	4E-272	Skin_Vascl	AMD1
ERF3	2.3E-275	0.70382	0.307	0.069	3.7E-271	Skin_Vascl	ERF
ATP11C1	3.7E-275	0.566544	0.223	0.039	6E-271	Skin_Vascl	ATP11C
KIAA14621	5.6E-275	0.260648	0.106	0.009	9E-271	Skin_Vascl	KIAA1462
KLF45	4E-274	1.080457	0.582	0.22	6.3E-270	Skin_Vascl	KLF4
SLC9A3R23	1.2E-273	1.201984	0.479	0.161	1.9E-269	Skin_Vascl	SLC9A3R2
S100A66	2.2E-273	0.621151	0.992	0.708	3.5E-269	Skin_Vascl	S100A6
CTSC5	2.4E-272	0.725606	0.42	0.116	3.8E-268	Skin_Vascl	CTSC
RP11.345J4	7.6E-272	0.418062	0.182	0.027	1.2E-267	Skin_Vascl	RP11.345J4.5
G3BP14	1.4E-271	0.937284	0.477	0.156	2.2E-267	Skin_Vascl	G3BP1
ICAM22	1.2E-270	0.541706	0.303	0.064	2E-266	Skin_Vascl	ICAM2
RPS210	1.8E-268	0.694768	1	0.797	2.9E-264	Skin_Vascl	RPS2
SNHG71	2.6E-267	0.743581	0.598	0.217	4.1E-263	Skin_Vascl	SNHG7
CHIC22	2.9E-265	1.045691	0.512	0.182	4.7E-261	Skin_Vascl	CHIC2
MYLIP3	8.2E-264	0.836035	0.483	0.159	1.3E-259	Skin_Vascl	MYLIP
HOXB4	1.2E-262	0.306885	0.125	0.014	1.9E-258	Skin_Vascl	HOXB4
CCDC33	2.9E-262	0.414804	0.167	0.024	4.7E-258	Skin_Vascl	CCDC3
CALM28	2.2E-261	0.820725	0.931	0.556	3.5E-257	Skin_Vascl	CALM2
SNHG154	3.8E-261	0.681621	0.389	0.106	6E-257	Skin_Vascl	SNHG15
HNRNPA1L	3.9E-259	0.582184	0.239	0.046	6.2E-255	Skin_Vascl	HNRNPA1L2
FGD52	7.7E-259	0.346522	0.134	0.016	1.2E-254	Skin_Vascl	FGD5
ATF46	1.2E-258	1.035043	0.691	0.331	2E-254	Skin_Vascl	ATF4
TSPAN136	1.9E-257	0.907446	0.461	0.152	3.1E-253	Skin_Vascl	TSPAN13

LY6E2	2.4E-257	0.930946	0.734	0.324	3.7E-253	Skin_Vascl	LY6E
CXCL33	3.3E-257	0.811372	0.436	0.129	5.3E-253	Skin_Vascl	CXCL3
NXT14	6E-257	0.785007	0.46	0.148	9.6E-253	Skin_Vascl	NXT1
RPS195	2.4E-255	0.733476	1	0.873	3.9E-251	Skin_Vascl	RPS19
SET8	3.6E-253	1.028983	0.671	0.305	5.8E-249	Skin_Vascl	SET
EIF3E7	1.1E-252	0.89122	0.838	0.443	1.8E-248	Skin_Vascl	EIF3E
TGFBR35	1.1E-251	0.614906	0.341	0.087	1.7E-247	Skin_Vascl	TGFBR3
MYH96	1.4E-249	0.840415	0.505	0.181	2.3E-245	Skin_Vascl	MYH9
H3F3A17	4.9E-249	0.861312	0.906	0.471	7.9E-245	Skin_Vascl	H3F3A
OLFM13	3.5E-247	0.35114	0.157	0.022	5.6E-243	Skin_Vascl	OLFM1
PLK24	3.8E-247	0.772484	0.321	0.082	6.1E-243	Skin_Vascl	PLK2
S100A162	5.3E-247	0.757944	0.665	0.276	8.4E-243	Skin_Vascl	S100A16
RILPL23	6E-247	0.666532	0.346	0.093	9.5E-243	Skin_Vascl	RILPL2
CDC374	1.1E-246	0.934565	0.624	0.269	1.8E-242	Skin_Vascl	CDC37
VAPA5	1.8E-246	1.011857	0.753	0.394	2.9E-242	Skin_Vascl	VAPA
RP11.798M	4.5E-244	0.382143	0.143	0.019	7.2E-240	Skin_Vascl	RP11.798M19.6
TPM35	1.2E-243	0.95466	0.689	0.326	1.9E-239	Skin_Vascl	TPM3
FLOT11	1.7E-243	0.785742	0.428	0.138	2.7E-239	Skin_Vascl	FLOT1
MECOM1	2.2E-243	0.537493	0.176	0.028	3.6E-239	Skin_Vascl	MECOM
TUBB8	7.1E-243	0.797178	0.704	0.304	1.1E-238	Skin_Vascl	TUBB
RPL106	9.2E-243	0.688202	1	0.876	1.5E-238	Skin_Vascl	RPL10
FGL24	4.9E-242	0.612948	0.361	0.094	7.8E-238	Skin_Vascl	FGL2
CDK173	1E-241	0.529777	0.205	0.037	1.6E-237	Skin_Vascl	CDK17
RPS94	2.6E-241	0.693677	0.994	0.834	4.1E-237	Skin_Vascl	RPS9
COX7A18	3.2E-241	0.702007	0.431	0.134	5.1E-237	Skin_Vascl	COX7A1
EPS85	8.3E-241	0.646535	0.304	0.076	1.3E-236	Skin_Vascl	EPS8
AC010642.	1.1E-240	0.370547	0.198	0.034	1.8E-236	Skin_Vascl	AC010642.1
AKAP136	2.5E-240	0.943039	0.537	0.212	3.9E-236	Skin_Vascl	AKAP13
LY964	1.8E-239	0.537182	0.286	0.065	2.9E-235	Skin_Vascl	LY96
PKIG5	2.3E-239	0.834793	0.344	0.096	3.7E-235	Skin_Vascl	PKIG
PRMT15	1.6E-236	0.833947	0.513	0.187	2.5E-232	Skin_Vascl	PRMT1
RSL24D15	7.7E-236	0.904675	0.671	0.301	1.2E-231	Skin_Vascl	RSL24D1
HRH12	3.9E-235	0.462997	0.19	0.033	6.2E-231	Skin_Vascl	HRH1
CRK5	1.3E-234	0.77211	0.38	0.115	2.1E-230	Skin_Vascl	CRK
SYNE25	1.8E-234	0.714698	0.42	0.133	2.9E-230	Skin_Vascl	SYNE2
CKS1B5	3.8E-234	0.772273	0.421	0.135	6.1E-230	Skin_Vascl	CKS1B
FSTL13	6.9E-234	0.574506	0.466	0.149	1.1E-229	Skin_Vascl	FSTL1
CH25H3	1E-233	0.296782	0.102	0.01	1.7E-229	Skin_Vascl	CH25H
MBNL19	1.1E-233	0.709263	0.488	0.166	1.7E-229	Skin_Vascl	MBNL1
NCK14	1.6E-233	0.718995	0.362	0.104	2.5E-229	Skin_Vascl	NCK1
GPR1461	2.9E-233	0.388721	0.136	0.018	4.6E-229	Skin_Vascl	GPR146
NFIB12	3E-233	0.755811	0.518	0.186	4.7E-229	Skin_Vascl	NFIB
INPP12	1.4E-231	0.579575	0.315	0.08	2.3E-227	Skin_Vascl	INPP1
RBM36	2.1E-231	0.903694	0.791	0.434	3.3E-227	Skin_Vascl	RBM3
ZFP36L16	2.2E-231	0.872799	0.831	0.463	3.5E-227	Skin_Vascl	ZFP36L1
GFOD11	2.5E-231	0.405667	0.157	0.024	4E-227	Skin_Vascl	GFOD1
CLIC17	1.1E-230	0.849191	0.848	0.469	1.8E-226	Skin_Vascl	CLIC1

RAP1B5	1.6E-230	0.898567	0.616	0.264	2.6E-226	Skin_Vascl	RAP1B
SERPINB92	2.1E-230	0.400081	0.265	0.059	3.3E-226	Skin_Vascl	SERPINB9
ABI32	1.8E-229	0.371065	0.18	0.03	2.8E-225	Skin_Vascl	ABI3
CST35	1.1E-228	0.315944	0.939	0.588	1.7E-224	Skin_Vascl	CST3
CHSY14	2.2E-228	0.54195	0.239	0.052	3.5E-224	Skin_Vascl	CHSY1
PFDN25	5.3E-227	0.880591	0.622	0.269	8.5E-223	Skin_Vascl	PFDN2
RASA42	2.6E-226	0.376899	0.119	0.014	4.1E-222	Skin_Vascl	RASA4
UBE2D35	1.7E-225	0.935316	0.848	0.528	2.7E-221	Skin_Vascl	UBE2D3
ZEB15	3.2E-225	0.544538	0.253	0.056	5.1E-221	Skin_Vascl	ZEB1
TUBA1B9	5.2E-225	0.833253	0.732	0.348	8.3E-221	Skin_Vascl	TUBA1B
IFI44L2	1.7E-224	0.42277	0.179	0.03	2.7E-220	Skin_Vascl	IFI44L
NINJ14	1.7E-224	0.710585	0.43	0.141	2.8E-220	Skin_Vascl	NINJ1
ARL23	3.7E-222	0.776236	0.534	0.205	5.9E-218	Skin_Vascl	ARL2
SERBP14	1.4E-220	0.890986	0.711	0.349	2.3E-216	Skin_Vascl	SERBP1
HLA.DQA2.	3.1E-220	0.27374	0.295	0.07	5E-216	Skin_Vascl	HLA.DQA2
CD402	3.2E-220	0.579837	0.285	0.071	5.1E-216	Skin_Vascl	CD40
FABP54	7E-220	0.817366	0.409	0.131	1.1E-215	Skin_Vascl	FABP5
RPS63	7.7E-220	0.531957	0.999	0.822	1.2E-215	Skin_Vascl	RPS6
DPYSL27	1.8E-219	0.553023	0.346	0.095	2.9E-215	Skin_Vascl	DPYSL2
PRCP3	5.7E-219	0.867916	0.376	0.121	9.1E-215	Skin_Vascl	PRCP
MGST24	8.4E-219	0.960157	0.502	0.204	1.3E-214	Skin_Vascl	MGST2
DMKN2	1.8E-218	0.273621	0.444	0.152	2.8E-214	Skin_Vascl	DMKN
CMIP3	1.8E-218	0.74795	0.364	0.111	2.9E-214	Skin_Vascl	CMIP
SNHG87	1.2E-217	0.809019	0.656	0.291	1.9E-213	Skin_Vascl	SNHG8
CBR33	8.9E-216	0.630113	0.298	0.079	1.4E-211	Skin_Vascl	CBR3
DSTN6	9.8E-216	0.812434	0.896	0.558	1.6E-211	Skin_Vascl	DSTN
S100A135	1.5E-215	0.707913	0.605	0.251	2.4E-211	Skin_Vascl	S100A13
NSUN63	2.1E-215	0.583437	0.226	0.049	3.3E-211	Skin_Vascl	NSUN6
RPL124	2.6E-215	0.685007	0.999	0.85	4.1E-211	Skin_Vascl	RPL12
LEPR2	8.4E-215	0.451611	0.274	0.064	1.3E-210	Skin_Vascl	LEPR
FOXO13	1.3E-214	0.501486	0.216	0.045	2.1E-210	Skin_Vascl	FOXO1
TNFRSF10A	1.4E-214	0.271075	0.105	0.012	2.2E-210	Skin_Vascl	TNFRSF10A
PRNP8	2.2E-214	0.859648	0.449	0.155	3.6E-210	Skin_Vascl	PRNP
MID11	2E-213	0.390153	0.146	0.022	3.2E-209	Skin_Vascl	MID1
USP124	4.8E-213	0.591321	0.27	0.066	7.6E-209	Skin_Vascl	USP12
TNFRSF1A(	2.3E-212	0.758668	0.445	0.156	3.7E-208	Skin_Vascl	TNFRSF1A
SERPINH14	9.2E-211	0.579357	0.37	0.111	1.5E-206	Skin_Vascl	SERPINH1
C11orf963	2.7E-210	0.264812	0.357	0.1	4.3E-206	Skin_Vascl	C11orf96
RPL364	2.8E-210	0.648774	0.997	0.838	4.5E-206	Skin_Vascl	RPL36
PLSCR11	4.6E-210	0.860076	0.522	0.207	7.3E-206	Skin_Vascl	PLSCR1
HNRNPA37	6.5E-210	0.873962	0.7	0.36	1E-205	Skin_Vascl	HNRNPA3
PHLDA1	1.5E-208	0.661733	0.738	0.352	2.3E-204	Skin_Vascl	PHLDA1
BMPR23	1.6E-208	0.713674	0.336	0.099	2.5E-204	Skin_Vascl	BMPR2
POLR2L4	2.4E-208	0.82977	0.812	0.468	3.9E-204	Skin_Vascl	POLR2L
ZFAS15	2.5E-208	0.750141	0.82	0.46	4E-204	Skin_Vascl	ZFAS1
PROX11	4.3E-208	0.358614	0.113	0.014	6.9E-204	Skin_Vascl	PROX1
CSRNP14	8E-208	0.697376	0.407	0.134	1.3E-203	Skin_Vascl	CSRNP1

HSPA1B4	9.2E-208	1.008743	0.658	0.305	1.5E-203	Skin_Vascl	HSPA1B
HSP90AB1!	2.8E-207	0.805063	0.882	0.565	4.5E-203	Skin_Vascl	HSP90AB1
CSNK1A17	7.7E-207	0.814325	0.601	0.266	1.2E-202	Skin_Vascl	CSNK1A1
ADAM153	1.8E-206	0.606651	0.277	0.072	2.8E-202	Skin_Vascl	ADAM15
LRCH13	1.4E-205	0.417054	0.191	0.037	2.2E-201	Skin_Vascl	LRCH1
RPS276	3.6E-205	0.66885	1	0.893	5.7E-201	Skin_Vascl	RPS27
ECE13	6.6E-205	0.506839	0.249	0.059	1E-200	Skin_Vascl	ECE1
ZNF5213	6.8E-205	0.308953	0.14	0.021	1.1E-200	Skin_Vascl	ZNF521
CHD17	5.5E-204	0.742558	0.388	0.127	8.8E-200	Skin_Vascl	CHD1
PSMB94	3.4E-203	0.483646	0.48	0.164	5.4E-199	Skin_Vascl	PSMB9
ARL151	3.8E-203	0.413774	0.141	0.022	6.1E-199	Skin_Vascl	ARL15
CTNND16	5.8E-203	0.704646	0.322	0.096	9.3E-199	Skin_Vascl	CTNND1
TSPAN45	1.1E-202	0.578132	0.37	0.113	1.8E-198	Skin_Vascl	TSPAN4
SHB	1.1E-202	0.587168	0.215	0.047	1.8E-198	Skin_Vascl	SHB
FRY4	2.4E-202	0.301748	0.13	0.019	3.9E-198	Skin_Vascl	FRY
TIFA1	2.5E-202	0.611799	0.241	0.057	4E-198	Skin_Vascl	TIFA
C7orf55.LU	4.5E-202	0.387481	0.212	0.043	7.3E-198	Skin_Vascl	C7orf55.LUC7L2
CKS24	1.6E-201	0.643609	0.416	0.138	2.5E-197	Skin_Vascl	CKS2
POMP5	2.7E-201	0.900249	0.742	0.397	4.3E-197	Skin_Vascl	POMP
TNFRSF1B!	2.7E-201	0.399826	0.217	0.045	4.4E-197	Skin_Vascl	TNFRSF1B
TGIF21	3.5E-201	0.410741	0.177	0.033	5.6E-197	Skin_Vascl	TGIF2
SLC38A26	4.2E-201	0.784487	0.563	0.236	6.6E-197	Skin_Vascl	SLC38A2
YPEL23	8.4E-201	0.66092	0.334	0.101	1.3E-196	Skin_Vascl	YPEL2
SEPHS21	3.1E-200	0.543444	0.253	0.061	5E-196	Skin_Vascl	SEPHS2
SCML13	3.7E-199	0.475352	0.268	0.068	5.9E-195	Skin_Vascl	SCML1
KTN13	1.6E-198	0.914472	0.713	0.392	2.5E-194	Skin_Vascl	KTN1
CFLAR4	3E-198	0.891151	0.586	0.266	4.8E-194	Skin_Vascl	CFLAR
NFE2L25	3.9E-198	0.899	0.603	0.283	6.1E-194	Skin_Vascl	NFE2L2
NME1.NM	5.5E-198	0.282231	0.147	0.023	8.8E-194	Skin_Vascl	NME1.NME2
GBE12	1.2E-197	0.583749	0.23	0.053	2E-193	Skin_Vascl	GBE1
PDIA53	5.6E-197	0.387035	0.157	0.027	8.9E-193	Skin_Vascl	PDIA5
CBLB3	7.5E-197	0.559977	0.228	0.052	1.2E-192	Skin_Vascl	CBLB
UBE2W2	8.6E-197	0.847947	0.356	0.117	1.4E-192	Skin_Vascl	UBE2W
RPL85	8.7E-197	0.613181	0.993	0.847	1.4E-192	Skin_Vascl	RPL8
LHFP6	1.6E-196	0.454504	0.31	0.083	2.5E-192	Skin_Vascl	LHFP
ARGLU15	2E-196	0.928214	0.622	0.304	3.2E-192	Skin_Vascl	ARGLU1
RPL155	2.3E-196	0.557992	0.997	0.893	3.6E-192	Skin_Vascl	RPL15
PIM34	2.3E-196	0.862267	0.614	0.286	3.7E-192	Skin_Vascl	PIM3
HSPE16	5.2E-196	0.789008	0.708	0.367	8.3E-192	Skin_Vascl	HSPE1
USP363	9.5E-196	0.577361	0.247	0.061	1.5E-191	Skin_Vascl	USP36
TRIB14	1.3E-195	0.461954	0.207	0.044	2.1E-191	Skin_Vascl	TRIB1
YBX15	1.4E-195	0.514022	0.904	0.584	2.2E-191	Skin_Vascl	YBX1
PITPNC11	1.2E-194	0.472142	0.224	0.051	1.8E-190	Skin_Vascl	PITPNC1
GBP41	1.8E-194	0.341972	0.142	0.022	2.9E-190	Skin_Vascl	GBP4
SYBU	4E-193	0.286302	0.125	0.018	6.4E-189	Skin_Vascl	SYBU
CNTNAP3E	5.2E-193	0.31176	0.106	0.013	8.3E-189	Skin_Vascl	CNTNAP3B
JUN7	2.3E-192	0.93507	0.818	0.482	3.7E-188	Skin_Vascl	JUN

CHN12	2.4E-192	0.327948	0.134	0.021	3.8E-188	Skin_Vascl	CHN1
BST25	4.4E-192	0.516066	0.5	0.176	7E-188	Skin_Vascl	BST2
TGIF15	1.1E-191	0.791334	0.485	0.192	1.8E-187	Skin_Vascl	TGIF1
MATR35	1.2E-191	0.565033	0.361	0.112	1.8E-187	Skin_Vascl	MATR3
RASGRP31	3.2E-191	0.517264	0.205	0.045	5.1E-187	Skin_Vascl	RASGRP3
PTP4A33	7.7E-191	0.382171	0.165	0.03	1.2E-186	Skin_Vascl	PTP4A3
RCAN24	1.4E-189	0.360387	0.158	0.028	2.3E-185	Skin_Vascl	RCAN2
PDLIM38	8.1E-189	0.567088	0.231	0.055	1.3E-184	Skin_Vascl	PDLIM3
LPCAT25	1.4E-188	0.466667	0.182	0.036	2.3E-184	Skin_Vascl	LPCAT2
TAP12	1.5E-188	0.504804	0.318	0.092	2.3E-184	Skin_Vascl	TAP1
DNAJA15	1.5E-188	0.849421	0.683	0.356	2.4E-184	Skin_Vascl	DNAJA1
APOL31	1.5E-188	0.300585	0.163	0.029	2.5E-184	Skin_Vascl	APOL3
FEZ23	2E-188	0.650507	0.337	0.106	3.2E-184	Skin_Vascl	FEZ2
RPL215	6.4E-188	0.627195	1	0.811	1E-183	Skin_Vascl	RPL21
HYAL21	1.3E-187	0.496108	0.226	0.053	2E-183	Skin_Vascl	HYAL2
PPP3R13	1.8E-187	0.555549	0.258	0.067	2.9E-183	Skin_Vascl	PPP3R1
CYB5R35	2.3E-187	0.697104	0.522	0.217	3.6E-183	Skin_Vascl	CYB5R3
RPL18A4	5.1E-186	0.560923	0.998	0.82	8.2E-182	Skin_Vascl	RPL18A
PAFAH1B2	9.3E-186	0.704721	0.364	0.122	1.5E-181	Skin_Vascl	PAFAH1B2
GIMAP12	3.2E-185	0.272012	0.243	0.056	5.2E-181	Skin_Vascl	GIMAP1
TINAGL14	4.6E-185	0.469194	0.287	0.08	7.4E-181	Skin_Vascl	TINAGL1
HNRNPA05	3.1E-184	0.712919	0.681	0.329	4.9E-180	Skin_Vascl	HNRNPA0
MTUS13	7.5E-184	0.871059	0.41	0.153	1.2E-179	Skin_Vascl	MTUS1
SAMD4A1	1.4E-183	0.34658	0.299	0.082	2.3E-179	Skin_Vascl	SAMD4A
KCNJ2	3.5E-183	0.340142	0.061	0.005	5.6E-179	Skin_Vascl	KCNJ2
SSFA26	5.4E-182	0.809123	0.421	0.164	8.7E-178	Skin_Vascl	SSFA2
RPL265	3.7E-181	0.62136	0.997	0.833	5.9E-177	Skin_Vascl	RPL26
ENY25	1.8E-180	0.785218	0.628	0.317	2.8E-176	Skin_Vascl	ENY2
SIK21	1.8E-180	0.413674	0.174	0.035	2.9E-176	Skin_Vascl	SIK2
VMP12	1.9E-180	0.925311	0.62	0.305	3.1E-176	Skin_Vascl	VMP1
MPRIP.AS1	1.7E-179	0.294643	0.096	0.012	2.7E-175	Skin_Vascl	MPRIP.AS1
PCNP5	1.2E-178	0.693179	0.565	0.255	1.9E-174	Skin_Vascl	PCNP
WBP52	5.6E-178	0.772909	0.449	0.176	8.9E-174	Skin_Vascl	WBP5
RPL35A4	5.7E-178	0.573118	0.997	0.861	9E-174	Skin_Vascl	RPL35A
RDX2	1.1E-177	0.779748	0.45	0.182	1.7E-173	Skin_Vascl	RDX
MAT2A7	1.9E-177	0.807727	0.394	0.146	3.1E-173	Skin_Vascl	MAT2A
ZC3H12A5	2.3E-177	0.614654	0.32	0.098	3.6E-173	Skin_Vascl	ZC3H12A
SNRPB5	2.6E-177	0.7126	0.658	0.328	4.1E-173	Skin_Vascl	SNRPB
APP6	5.5E-177	0.745735	0.546	0.239	8.8E-173	Skin_Vascl	APP
ITPKB2	1.2E-175	0.410385	0.178	0.037	1.9E-171	Skin_Vascl	ITPKB
HNRNPA14	3.6E-175	0.615035	0.882	0.54	5.7E-171	Skin_Vascl	HNRNPA1
CALD16	1.2E-174	0.51142	0.499	0.19	1.9E-170	Skin_Vascl	CALD1
CASKIN22	1.5E-174	0.295783	0.112	0.016	2.4E-170	Skin_Vascl	CASKIN2
FADS33	6.7E-174	0.435386	0.184	0.039	1.1E-169	Skin_Vascl	FADS3
MIR22HG4	7E-174	0.576321	0.284	0.082	1.1E-169	Skin_Vascl	MIR22HG
SRP145	1.4E-173	0.834443	0.924	0.698	2.2E-169	Skin_Vascl	SRP14
RPL235	4E-173	0.627909	0.96	0.679	6.3E-169	Skin_Vascl	RPL23

BNIP25	1E-172	0.615027	0.356	0.121	1.6E-168	Skin_Vascl	BNIP2
B3GNT24	1.3E-172	0.495283	0.249	0.066	2.1E-168	Skin_Vascl	B3GNT2
NUDT14	5.8E-172	0.559692	0.266	0.076	9.3E-168	Skin_Vascl	NUDT14
CD832	8.8E-172	0.454668	0.308	0.093	1.4E-167	Skin_Vascl	CD83
RP11.294J2	1.8E-171	0.343054	0.148	0.027	2.8E-167	Skin_Vascl	RP11.294J22.6
LAMC14	2.9E-171	0.544025	0.267	0.074	4.6E-167	Skin_Vascl	LAMC1
CYSLTR13	9.2E-171	0.274611	0.11	0.016	1.5E-166	Skin_Vascl	CYSLTR1
C1orf214	2.2E-170	0.568244	0.313	0.098	3.5E-166	Skin_Vascl	C1orf21
RPL355	2.5E-170	0.552281	0.996	0.833	4E-166	Skin_Vascl	RPL35
KCNJ151	1.1E-169	0.360656	0.094	0.012	1.8E-165	Skin_Vascl	KCNJ15
CHD7	1.5E-169	0.482899	0.194	0.045	2.5E-165	Skin_Vascl	CHD7
ITPRIP	1.6E-169	0.540317	0.239	0.063	2.6E-165	Skin_Vascl	ITPRIP
RPS85	2.3E-169	0.566496	0.999	0.847	3.6E-165	Skin_Vascl	RPS8
RPS155	5.4E-169	0.547697	0.999	0.879	8.6E-165	Skin_Vascl	RPS15
SRSF37	4E-168	0.768545	0.744	0.428	6.3E-164	Skin_Vascl	SRSF3
PHLDB25	6E-168	0.519347	0.21	0.05	9.6E-164	Skin_Vascl	PHLDB2
RPL317	1.3E-167	0.485947	0.995	0.765	2.2E-163	Skin_Vascl	RPL31
CCDC503	2E-167	0.68468	0.37	0.134	3.2E-163	Skin_Vascl	CCDC50
RPLP18	3.2E-167	0.481901	1	0.914	5E-163	Skin_Vascl	RPLP1
RYBP2	7E-167	0.693138	0.38	0.142	1.1E-162	Skin_Vascl	RYBP
GLA3	2.9E-166	0.742736	0.285	0.085	4.7E-162	Skin_Vascl	GLA
C21orf91	3.2E-165	0.489672	0.219	0.055	5.1E-161	Skin_Vascl	C21orf91
PLXNA21	9.2E-165	0.297227	0.122	0.02	1.5E-160	Skin_Vascl	PLXNA2
NFKBIZ4	1.8E-163	0.706648	0.534	0.241	2.9E-159	Skin_Vascl	NFKBIZ
MSRB36	2.1E-163	0.313184	0.163	0.033	3.4E-159	Skin_Vascl	MSRB3
SPAG95	4.2E-163	0.814189	0.449	0.189	6.8E-159	Skin_Vascl	SPAG9
NRARP5	7.3E-163	0.403561	0.178	0.038	1.2E-158	Skin_Vascl	NRARP
CAPZA25	8.3E-162	0.818343	0.565	0.275	1.3E-157	Skin_Vascl	CAPZA2
TSPAN123	6.8E-161	0.557842	0.2	0.05	1.1E-156	Skin_Vascl	TSPAN12
ZNF281	9.4E-161	0.505067	0.211	0.053	1.5E-156	Skin_Vascl	ZNF281
TCEB13	3.5E-159	0.824623	0.652	0.354	5.5E-155	Skin_Vascl	TCEB1
TBPL11	4.5E-159	0.483707	0.289	0.088	7.2E-155	Skin_Vascl	TBPL1
FKBP93	2.7E-158	0.517114	0.261	0.075	4.4E-154	Skin_Vascl	FKBP9
EGLN31	1.9E-157	0.609491	0.284	0.088	3.1E-153	Skin_Vascl	EGLN3
RPS296	2E-157	0.565319	0.984	0.787	3.3E-153	Skin_Vascl	RPS29
SUMO15	4E-157	0.75105	0.639	0.342	6.4E-153	Skin_Vascl	SUMO1
CNOT6L2	1.7E-156	0.596253	0.297	0.096	2.6E-152	Skin_Vascl	CNOT6L
PROS14	4.3E-156	0.337127	0.184	0.041	6.8E-152	Skin_Vascl	PROS1
NR4A35	1.1E-155	0.507986	0.208	0.051	1.7E-151	Skin_Vascl	NR4A3
PYURF3	2.3E-155	0.678011	0.523	0.243	3.7E-151	Skin_Vascl	PYURF
JUNB3	5.6E-155	0.79805	0.945	0.686	8.9E-151	Skin_Vascl	JUNB
CD993	7.5E-155	0.619242	0.799	0.487	1.2E-150	Skin_Vascl	CD99
ARL4A2	3.4E-154	0.449401	0.517	0.227	5.4E-150	Skin_Vascl	ARL4A
ROCK27	3.5E-154	0.606937	0.287	0.092	5.6E-150	Skin_Vascl	ROCK2
RALB	4.1E-154	0.607606	0.304	0.103	6.5E-150	Skin_Vascl	RALB
PNRC15	4.4E-154	0.600724	0.805	0.506	7E-150	Skin_Vascl	PNRC1
MYL67	5.6E-154	0.531716	0.963	0.803	8.9E-150	Skin_Vascl	MYL6

MGLL7	6.2E-154	0.636453	0.428	0.173	9.9E-150	Skin_Vascl	MGLL
DAAM13	6.6E-154	0.706641	0.41	0.165	1E-149	Skin_Vascl	DAAM1
GIPC21	3.9E-153	0.447198	0.132	0.025	6.3E-149	Skin_Vascl	GIPC2
NOP163	6.3E-153	0.490278	0.243	0.069	1E-148	Skin_Vascl	NOP16
RPS205	1.6E-152	0.51309	0.982	0.792	2.6E-148	Skin_Vascl	RPS20
FOSL12	1.7E-152	0.510093	0.27	0.08	2.7E-148	Skin_Vascl	FOSL1
PFDN54	4.9E-152	0.550521	0.95	0.723	7.8E-148	Skin_Vascl	PFDN5
NXPE31	6.1E-152	0.339185	0.119	0.02	9.7E-148	Skin_Vascl	NXPE3
SCARB11	8.3E-152	0.433585	0.174	0.04	1.3E-147	Skin_Vascl	SCARB1
HLA.B3	4.6E-151	0.350811	0.959	0.662	7.3E-147	Skin_Vascl	HLA.B
GRAMD1A	1.2E-150	0.448203	0.256	0.075	1.9E-146	Skin_Vascl	GRAMD1A
PGF4	2.7E-150	0.26172	0.143	0.027	4.3E-146	Skin_Vascl	PGF
PSMB82	9.9E-150	0.498058	0.46	0.187	1.6E-145	Skin_Vascl	PSMB8
HNRNPDL5	3.2E-149	0.542709	0.676	0.331	5E-145	Skin_Vascl	HNRNPDL
FERMT26	4.8E-149	0.542781	0.261	0.079	7.7E-145	Skin_Vascl	FERMT2
STX113	5E-149	0.497686	0.23	0.063	8E-145	Skin_Vascl	STX11
PRSS235	1.4E-148	0.461049	0.292	0.091	2.2E-144	Skin_Vascl	PRSS23
PLA2G166	2.1E-148	0.497408	0.344	0.122	3.4E-144	Skin_Vascl	PLA2G16
EHD26	5.2E-148	0.342206	0.184	0.043	8.3E-144	Skin_Vascl	EHD2
SMAD11	8.3E-148	0.572874	0.205	0.054	1.3E-143	Skin_Vascl	SMAD1
PIK3C2B1	1.8E-147	0.297346	0.112	0.018	2.9E-143	Skin_Vascl	PIK3C2B
GNAI25	2.7E-147	0.563766	0.502	0.221	4.3E-143	Skin_Vascl	GNAI2
EMP24	1.2E-146	0.532852	0.458	0.187	2E-142	Skin_Vascl	EMP2
JAG16	2.9E-146	0.389164	0.195	0.048	4.6E-142	Skin_Vascl	JAG1
THAP9.AS1	3.1E-146	0.550674	0.254	0.077	4.9E-142	Skin_Vascl	THAP9.AS1
RPS27A7	3.4E-146	0.516412	0.998	0.847	5.4E-142	Skin_Vascl	RPS27A
SLC41A1	1.4E-145	0.266924	0.123	0.022	2.3E-141	Skin_Vascl	SLC41A1
GAPDH7	1.4E-145	0.504091	0.94	0.761	2.3E-141	Skin_Vascl	GAPDH
RPS215	9.8E-145	0.578035	0.961	0.668	1.6E-140	Skin_Vascl	RPS21
SLCO4A1	1.6E-144	0.531503	0.31	0.104	2.6E-140	Skin_Vascl	SLCO4A1
SLC35G2	2.4E-144	0.371307	0.129	0.024	3.8E-140	Skin_Vascl	SLC35G2
DDX3X7	5.9E-144	0.728234	0.573	0.297	9.4E-140	Skin_Vascl	DDX3X
FCGRT5	6.5E-144	0.488625	0.573	0.273	1E-139	Skin_Vascl	FCGRT
RPS253	1.8E-143	0.453489	0.997	0.821	2.8E-139	Skin_Vascl	RPS25
AKAP123	3.9E-143	0.53573	0.247	0.072	6.2E-139	Skin_Vascl	AKAP12
INHBB3	3E-142	0.276708	0.113	0.019	4.7E-138	Skin_Vascl	INHBB
SHE1	1.8E-141	0.295011	0.116	0.02	2.9E-137	Skin_Vascl	SHE
RPL10A5	4.9E-141	0.449853	0.987	0.731	7.8E-137	Skin_Vascl	RPL10A
BAG32	5.1E-141	0.555542	0.375	0.145	8.1E-137	Skin_Vascl	BAG3
GLS8	4.6E-140	0.543091	0.324	0.114	7.3E-136	Skin_Vascl	GLS
C1orf1152	3.5E-139	0.48447	0.178	0.045	5.6E-135	Skin_Vascl	C1orf115
SP1004	6.1E-139	0.489758	0.361	0.136	9.7E-135	Skin_Vascl	SP100
CD553	1.5E-138	0.71772	0.537	0.262	2.3E-134	Skin_Vascl	CD55
HLA.G	2.6E-138	0.304489	0.123	0.023	4.1E-134	Skin_Vascl	HLA.G
RPS54	4.3E-138	0.420488	0.993	0.782	6.9E-134	Skin_Vascl	RPS5
GFOD21	6.9E-138	0.465658	0.208	0.057	1.1E-133	Skin_Vascl	GFOD2
NR2F25	9.9E-138	0.419348	0.269	0.084	1.6E-133	Skin_Vascl	NR2F2

IRF13	4.3E-136	0.546309	0.479	0.211	6.8E-132	Skin_Vascl	IRF1
CPXM22	4.7E-136	0.351894	0.148	0.032	7.5E-132	Skin_Vascl	CPXM2
RPL37A5	4.9E-136	0.463612	0.995	0.854	7.8E-132	Skin_Vascl	RPL37A
VCAM14	7.8E-136	0.689603	0.146	0.031	1.2E-131	Skin_Vascl	VCAM1
CCND18	1E-135	0.568914	0.321	0.116	1.6E-131	Skin_Vascl	CCND1
ZNF2672	1.4E-135	0.526413	0.243	0.075	2.2E-131	Skin_Vascl	ZNF267
YWHAG4	1.6E-135	0.702545	0.48	0.226	2.5E-131	Skin_Vascl	YWHAG
COMMD65	8.4E-135	0.556768	0.795	0.469	1.3E-130	Skin_Vascl	COMMD6
N4BP2L13	6E-134	0.365031	0.219	0.061	9.5E-130	Skin_Vascl	N4BP2L1
CYCS6	8.1E-134	0.421674	0.637	0.337	1.3E-129	Skin_Vascl	CYCS
FYN6	1.9E-133	0.275752	0.23	0.064	3E-129	Skin_Vascl	FYN
ATF34	4.3E-133	0.635479	0.519	0.24	6.9E-129	Skin_Vascl	ATF3
COL18A15	7E-133	0.345442	0.239	0.071	1.1E-128	Skin_Vascl	COL18A1
MORF4L14	2E-132	0.605413	0.754	0.453	3.2E-128	Skin_Vascl	MORF4L1
ZNF608	2.1E-132	0.310446	0.098	0.016	3.3E-128	Skin_Vascl	ZNF608
GLTSCR2	2.5E-131	0.354861	0.841	0.524	4E-127	Skin_Vascl	GLTSCR2
RGS52	4.7E-131	0.936847	0.132	0.027	7.5E-127	Skin_Vascl	RGS5
HIVEP23	6.6E-131	0.503345	0.271	0.089	1.1E-126	Skin_Vascl	HIVEP2
HNRNPA2E	2.1E-130	0.611248	0.788	0.487	3.4E-126	Skin_Vascl	HNRNPA2B1
ASAP14	2.6E-130	0.451005	0.278	0.092	4.2E-126	Skin_Vascl	ASAP1
BCL33	6.1E-130	0.451124	0.288	0.098	9.8E-126	Skin_Vascl	BCL3
CMTM86	1.4E-129	0.459206	0.221	0.067	2.2E-125	Skin_Vascl	CMTM8
EBPL3	2.6E-129	0.502372	0.341	0.132	4.2E-125	Skin_Vascl	EBPL
RBM8A4	4.6E-129	0.629172	0.614	0.338	7.3E-125	Skin_Vascl	RBM8A
LIMS14	1.5E-128	0.605581	0.391	0.165	2.3E-124	Skin_Vascl	LIMS1
MSC1	2.4E-128	0.293041	0.111	0.02	3.8E-124	Skin_Vascl	MSC
IER32	2.4E-128	0.271802	0.718	0.383	3.9E-124	Skin_Vascl	IER3
CYB5A7	3.1E-128	0.535677	0.414	0.179	5E-124	Skin_Vascl	CYB5A
MAGI12	3.3E-128	0.540045	0.218	0.066	5.2E-124	Skin_Vascl	MAGI1
HMGB110	1.2E-127	0.567945	0.853	0.571	1.9E-123	Skin_Vascl	HMGB1
FRMD4A4	1.3E-127	0.422555	0.184	0.049	2E-123	Skin_Vascl	FRMD4A
RBM172	2.6E-127	0.582337	0.41	0.177	4.2E-123	Skin_Vascl	RBM17
ATP8B11	2.8E-127	0.522206	0.222	0.068	4.5E-123	Skin_Vascl	ATP8B1
RASSF12	3.1E-127	0.432443	0.269	0.09	4.9E-123	Skin_Vascl	RASSF1
CDC42SE24	3.7E-127	0.464241	0.318	0.117	5.9E-123	Skin_Vascl	CDC42SE2
PPP2R2A2	6.9E-127	0.578007	0.327	0.126	1.1E-122	Skin_Vascl	PPP2R2A
LRRFIP15	9.5E-127	0.631707	0.649	0.368	1.5E-122	Skin_Vascl	LRRFIP1
ATP1B35	9.6E-127	0.532532	0.499	0.232	1.5E-122	Skin_Vascl	ATP1B3
OTUD42	2.2E-126	0.477139	0.25	0.082	3.5E-122	Skin_Vascl	OTUD4
PLXND13	2.8E-126	0.257865	0.136	0.029	4.5E-122	Skin_Vascl	PLXND1
GCH11	4.7E-126	0.424917	0.199	0.055	7.5E-122	Skin_Vascl	GCH1
RPS136	2.2E-125	0.454922	0.99	0.815	3.5E-121	Skin_Vascl	RPS13
AHI13	1.6E-124	0.571599	0.3	0.109	2.5E-120	Skin_Vascl	AHI1
SNX34	3.3E-124	0.612231	0.689	0.412	5.3E-120	Skin_Vascl	SNX3
RPS6KA37	3.9E-124	0.470322	0.271	0.093	6.2E-120	Skin_Vascl	RPS6KA3
CASP102	5.7E-124	0.337938	0.151	0.035	9E-120	Skin_Vascl	CASP10
MB21D21	1.1E-123	0.297574	0.12	0.024	1.8E-119	Skin_Vascl	MB21D2

RAPGEF31	2.2E-123	0.322026	0.141	0.032	3.6E-119	Skin_Vascl	RAPGEF3
SRGAP23	7.7E-123	0.446627	0.18	0.048	1.2E-118	Skin_Vascl	SRGAP2
PARVB4	1.5E-122	0.297858	0.167	0.041	2.3E-118	Skin_Vascl	PARVB
RPL36A3	1.5E-122	0.390088	0.858	0.505	2.3E-118	Skin_Vascl	RPL36A
RGS33	1.5E-122	0.407566	0.165	0.041	2.5E-118	Skin_Vascl	RGS3
IL6ST6	2.4E-122	0.596528	0.46	0.219	3.8E-118	Skin_Vascl	IL6ST
MAP3K111	3.1E-122	0.463626	0.26	0.088	4.9E-118	Skin_Vascl	MAP3K11
RPS4X2	4.5E-122	0.299595	0.997	0.767	7.2E-118	Skin_Vascl	RPS4X
BEST14	5.8E-122	0.296319	0.165	0.041	9.3E-118	Skin_Vascl	BEST1
PPP1R15A:	8.2E-122	0.458388	0.774	0.444	1.3E-117	Skin_Vascl	PPP1R15A
FAM60A4	1.5E-121	0.574484	0.283	0.103	2.4E-117	Skin_Vascl	FAM60A
GNA133	1.8E-121	0.385114	0.252	0.082	2.9E-117	Skin_Vascl	GNA13
TIPIN5	3.4E-121	0.485533	0.229	0.072	5.4E-117	Skin_Vascl	TIPIN
MORF4L24	3.7E-121	0.668676	0.554	0.297	6E-117	Skin_Vascl	MORF4L2
SNRPF4	6.5E-121	0.553702	0.489	0.234	1E-116	Skin_Vascl	SNRPF
BTF35	8.2E-121	0.485664	0.917	0.708	1.3E-116	Skin_Vascl	BTF3
ATOX16	4E-120	0.591559	0.529	0.27	6.3E-116	Skin_Vascl	ATOX1
EGR33	3.9E-119	0.320913	0.141	0.032	6.2E-115	Skin_Vascl	EGR3
EHD44	5.3E-119	0.465669	0.259	0.088	8.5E-115	Skin_Vascl	EHD4
RPL27A4	5.7E-119	0.373904	0.993	0.834	9.1E-115	Skin_Vascl	RPL27A
TMEM140:	8.6E-119	0.281776	0.106	0.02	1.4E-114	Skin_Vascl	TMEM140
PTPRG5	1.3E-118	0.277138	0.151	0.036	2E-114	Skin_Vascl	PTPRG
PIK3R31	1.6E-118	0.36261	0.157	0.039	2.5E-114	Skin_Vascl	PIK3R3
GUK14	1.9E-118	0.550822	0.805	0.553	3.1E-114	Skin_Vascl	GUK1
NFAT56	2E-118	0.555263	0.319	0.125	3.2E-114	Skin_Vascl	NFAT5
IFNGR15	2.1E-118	0.494085	0.334	0.132	3.4E-114	Skin_Vascl	IFNGR1
RPS15A4	2.2E-118	0.394668	0.999	0.847	3.6E-114	Skin_Vascl	RPS15A
ENTPD16	8.5E-118	0.315852	0.172	0.045	1.4E-113	Skin_Vascl	ENTPD1
ARL13B3	1E-117	0.373005	0.175	0.047	1.6E-113	Skin_Vascl	ARL13B
CPEB4	1.3E-117	0.700724	0.42	0.198	2.1E-113	Skin_Vascl	CPEB4
RABGGTB5	1.3E-116	0.511882	0.272	0.098	2E-112	Skin_Vascl	RABGGTB
PRRG1	1.5E-116	0.269925	0.096	0.017	2.4E-112	Skin_Vascl	PRRG1
HLA.F2	1.5E-116	0.290805	0.319	0.117	2.4E-112	Skin_Vascl	HLA.F
BCAR11	2E-116	0.481465	0.224	0.072	3.1E-112	Skin_Vascl	BCAR1
MEX3C	5.3E-116	0.349888	0.244	0.079	8.4E-112	Skin_Vascl	MEX3C
HSPD18	1.7E-115	0.5822	0.504	0.254	2.8E-111	Skin_Vascl	HSPD1
LDLR3	1.5E-114	0.511042	0.274	0.1	2.3E-110	Skin_Vascl	LDLR
RPL385	1.9E-114	0.466163	0.961	0.759	3E-110	Skin_Vascl	RPL38
GPBP15	3.4E-114	0.60922	0.578	0.317	5.3E-110	Skin_Vascl	GPBP1
DACH1	3.4E-114	0.305075	0.109	0.021	5.4E-110	Skin_Vascl	DACH1
IRF2BPL6	3.6E-114	0.426949	0.229	0.074	5.7E-110	Skin_Vascl	IRF2BPL
GOLIM44	4E-114	0.437863	0.245	0.081	6.3E-110	Skin_Vascl	GOLIM4
ANP32E5	1.2E-113	0.421207	0.269	0.094	1.9E-109	Skin_Vascl	ANP32E
CDK21	3E-113	0.321096	0.123	0.026	4.8E-109	Skin_Vascl	CDK2
MYO1E	3.4E-113	0.424112	0.191	0.057	5.4E-109	Skin_Vascl	MYO1E
CDC273	3.7E-113	0.506565	0.226	0.074	6E-109	Skin_Vascl	CDC27
CDC426	6.1E-113	0.745617	0.72	0.466	9.7E-109	Skin_Vascl	CDC42

TCF7L22	6.4E-113	0.524889	0.247	0.085	1E-108	Skin_Vascl	TCF7L2
SDE21	2.8E-112	0.459147	0.169	0.046	4.5E-108	Skin_Vascl	SDE2
SH3BP54	2.3E-111	0.373169	0.301	0.112	3.7E-107	Skin_Vascl	SH3BP5
ZBTB18	3.8E-111	0.314193	0.115	0.024	6.1E-107	Skin_Vascl	ZBTB18
VAMP32	4.5E-111	0.564009	0.321	0.132	7.2E-107	Skin_Vascl	VAMP3
MLKL1	5.1E-111	0.313804	0.105	0.021	8.1E-107	Skin_Vascl	MLKL
NOP101	1.1E-110	0.642321	0.627	0.365	1.7E-106	Skin_Vascl	NOP10
KCTD203	3.5E-110	0.395365	0.228	0.075	5.6E-106	Skin_Vascl	KCTD20
SFPQ8	3.5E-110	0.554287	0.533	0.282	5.6E-106	Skin_Vascl	SFPQ
CHD25	7.4E-110	0.598466	0.429	0.204	1.2E-105	Skin_Vascl	CHD2
CBX34	1.2E-109	0.487559	0.427	0.198	1.9E-105	Skin_Vascl	CBX3
STX61	1.8E-109	0.37514	0.146	0.036	2.8E-105	Skin_Vascl	STX6
PLSCR43	5.9E-109	0.343196	0.168	0.045	9.5E-105	Skin_Vascl	PLSCR4
ZBTB103	1.2E-108	0.453222	0.222	0.073	1.9E-104	Skin_Vascl	ZBTB10
RPS263	3.2E-108	0.460157	0.945	0.739	5.1E-104	Skin_Vascl	RPS26
PRDM12	5.1E-108	0.659838	0.388	0.167	8.1E-104	Skin_Vascl	PRDM1
BDKRB21	6.6E-108	0.281744	0.108	0.022	1E-103	Skin_Vascl	BDKRB2
ZBTB212	9.3E-108	0.389588	0.165	0.045	1.5E-103	Skin_Vascl	ZBTB21
FAM177A1	1.1E-107	0.538121	0.437	0.212	1.8E-103	Skin_Vascl	FAM177A1
SPSB12	1.8E-107	0.362328	0.191	0.057	2.8E-103	Skin_Vascl	SPSB1
RAB135	5E-107	0.659448	0.428	0.216	7.9E-103	Skin_Vascl	RAB13
QKI6	7E-107	0.51662	0.38	0.169	1.1E-102	Skin_Vascl	QKI
PTGS24	1.2E-106	0.576234	0.15	0.038	1.9E-102	Skin_Vascl	PTGS2
NOLC13	1.8E-106	0.415534	0.231	0.078	2.9E-102	Skin_Vascl	NOLC1
STX121	6.6E-106	0.538835	0.293	0.118	1.1E-101	Skin_Vascl	STX12
SLC25A251	1.7E-105	0.424821	0.193	0.059	2.7E-101	Skin_Vascl	SLC25A25
CNIH13	6.2E-105	0.46876	0.314	0.127	9.8E-101	Skin_Vascl	CNIH1
ARHGAP26	6.6E-105	0.40757	0.182	0.054	1.1E-100	Skin_Vascl	ARHGAP26
TNFRSF12A	1E-104	0.345494	0.32	0.124	1.6E-100	Skin_Vascl	TNFRSF12A
SLC25A373	1.5E-104	0.358368	0.385	0.172	2.3E-100	Skin_Vascl	SLC25A37
IFI64	8.9E-103	0.301542	0.367	0.151	1.41E-98	Skin_Vascl	IFI6
IFIT31	9.7E-103	0.272595	0.144	0.037	1.55E-98	Skin_Vascl	IFIT3
YES1	1.1E-102	0.492845	0.252	0.095	1.78E-98	Skin_Vascl	YES1
EIF4E3	1.2E-102	0.461024	0.473	0.24	1.85E-98	Skin_Vascl	EIF4E
CORO1C4	1.2E-102	0.503623	0.292	0.115	1.96E-98	Skin_Vascl	CORO1C
THBS14	2.4E-102	0.274395	0.233	0.078	3.87E-98	Skin_Vascl	THBS1
NPM13	5E-102	0.353127	0.841	0.528	7.94E-98	Skin_Vascl	NPM1
PIK3C2A1	1.3E-101	0.453197	0.225	0.078	2.05E-97	Skin_Vascl	PIK3C2A
TAX1BP33	2E-101	0.353537	0.242	0.084	3.17E-97	Skin_Vascl	TAX1BP3
ERH5	2.7E-101	0.528115	0.561	0.308	4.28E-97	Skin_Vascl	ERH
NCL9	2.7E-101	0.453213	0.521	0.267	4.33E-97	Skin_Vascl	NCL
SLC43A34	3.7E-101	0.253977	0.132	0.032	5.97E-97	Skin_Vascl	SLC43A3
EID16	4.1E-101	0.406999	0.633	0.353	6.48E-97	Skin_Vascl	EID1
FBL2	4.7E-101	0.327661	0.483	0.232	7.55E-97	Skin_Vascl	FBL
CRTAP6	1E-100	0.395364	0.289	0.112	1.6E-96	Skin_Vascl	CRTAP
PTPN144	1.2E-100	0.384876	0.181	0.056	1.94E-96	Skin_Vascl	PTPN14
CSGALNAC	1.3E-100	0.297582	0.155	0.042	2E-96	Skin_Vascl	CSGALNACT1

NOTCH41	1.5E-100	0.251266	0.096	0.019	2.42E-96	Skin_Vascl	NOTCH4
MRPL33	1.6E-100	0.617578	0.578	0.333	2.57E-96	Skin_Vascl	MRPL33
CCNL13	1.8E-100	0.541604	0.585	0.336	2.92E-96	Skin_Vascl	CCNL1
COX161	3.3E-100	0.429217	0.296	0.117	5.29E-96	Skin_Vascl	COX16
MPDZ4	5.3E-100	0.254798	0.123	0.029	8.49E-96	Skin_Vascl	MPDZ
HEY12	1E-99	0.568062	0.115	0.026	1.6E-95	Skin_Vascl	HEY1
MOB45	1.4E-99	0.402028	0.248	0.09	2.18E-95	Skin_Vascl	MOB4
RPL112	1.6E-99	0.305795	0.997	0.851	2.49E-95	Skin_Vascl	RPL11
LRIG34	2.3E-99	0.400582	0.164	0.048	3.69E-95	Skin_Vascl	LRIG3
RP11.841C	2.3E-99	0.31525	0.101	0.021	3.72E-95	Skin_Vascl	RP11.841O20.2
ZC3HAV14	2.5E-99	0.392153	0.256	0.095	4E-95	Skin_Vascl	ZC3HAV1
TMED29	3.1E-99	0.48438	0.603	0.342	5.02E-95	Skin_Vascl	TMED2
GSPT14	7E-99	0.528804	0.369	0.172	1.12E-94	Skin_Vascl	GSPT1
CAMSAP24	1.17E-98	0.430695	0.212	0.071	1.86E-94	Skin_Vascl	CAMSAP2
RPL274	2.66E-98	0.354402	0.961	0.747	4.24E-94	Skin_Vascl	RPL27
SLC39A13	3.21E-98	0.427251	0.273	0.106	5.13E-94	Skin_Vascl	SLC39A1
TPM4	4.18E-98	0.480514	0.7	0.418	6.67E-94	Skin_Vascl	TPM4
SNRPE4	4.19E-98	0.473647	0.505	0.263	6.69E-94	Skin_Vascl	SNRPE
APBB22	6.18E-98	0.284367	0.126	0.031	9.86E-94	Skin_Vascl	APBB2
PTGES36	7.22E-98	0.486887	0.654	0.389	1.15E-93	Skin_Vascl	PTGES3
RBX17	8.74E-98	0.510805	0.599	0.345	1.39E-93	Skin_Vascl	RBX1
TANK4	8.86E-98	0.612018	0.299	0.125	1.41E-93	Skin_Vascl	TANK
CX3CL12	1.04E-97	0.38722	0.104	0.022	1.66E-93	Skin_Vascl	CX3CL1
DENND5A3	1.22E-97	0.265977	0.149	0.04	1.95E-93	Skin_Vascl	DENND5A
SEC626	1.68E-97	0.503649	0.666	0.397	2.68E-93	Skin_Vascl	SEC62
CEBPG1	2.78E-97	0.45975	0.273	0.108	4.43E-93	Skin_Vascl	CEBPG
CD15111	3.25E-97	0.464219	0.445	0.217	5.19E-93	Skin_Vascl	CD151
CHMP4B4	4.18E-97	0.596323	0.553	0.302	6.67E-93	Skin_Vascl	CHMP4B
UBE2S7	5.03E-97	0.469357	0.31	0.129	8.02E-93	Skin_Vascl	UBE2S
ARPC5L1	5.43E-97	0.525555	0.49	0.262	8.67E-93	Skin_Vascl	ARPC5L
LAPTM4A5	5.97E-97	0.377564	0.789	0.515	9.52E-93	Skin_Vascl	LAPTM4A
TSPAN23	8.02E-97	0.276484	0.111	0.025	1.28E-92	Skin_Vascl	TSPAN2
RBM397	8.84E-97	0.560216	0.652	0.407	1.41E-92	Skin_Vascl	RBM39
SIK12	1.67E-96	0.354186	0.349	0.146	2.66E-92	Skin_Vascl	SIK1
PIP4K2A6	1.86E-96	0.331589	0.188	0.058	2.97E-92	Skin_Vascl	PIP4K2A
KIAA03551	2E-96	0.422156	0.226	0.08	3.19E-92	Skin_Vascl	KIAA0355
NFKB24	2.13E-96	0.372749	0.22	0.076	3.4E-92	Skin_Vascl	NFKB2
PELI11	3.51E-96	0.478971	0.412	0.194	5.6E-92	Skin_Vascl	PELI1
RHOU3	3.68E-96	0.325843	0.154	0.044	5.86E-92	Skin_Vascl	RHOU
MTPN6	3.79E-96	0.428146	0.338	0.147	6.05E-92	Skin_Vascl	MTPN
CDC267	3.84E-96	0.492106	0.294	0.124	6.13E-92	Skin_Vascl	CDC26
LEPROT5	6.37E-96	0.507697	0.48	0.253	1.02E-91	Skin_Vascl	LEPROT
RAN7	9.1E-96	0.454405	0.63	0.363	1.45E-91	Skin_Vascl	RAN
HSD3B72	9.9E-96	0.281043	0.132	0.034	1.58E-91	Skin_Vascl	HSD3B7
GADD45G4	1.65E-95	0.468139	0.199	0.065	2.63E-91	Skin_Vascl	GADD45G
LSM22	2.31E-95	0.366125	0.371	0.166	3.69E-91	Skin_Vascl	LSM2
CTNNB16	3.28E-95	0.679291	0.361	0.169	5.23E-91	Skin_Vascl	CTNNB1

CYB5B	3.3E-95	0.431258	0.268	0.105	5.26E-91	Skin_Vascl	CYB5B
SLC25A55	3.57E-95	0.433977	0.633	0.365	5.69E-91	Skin_Vascl	SLC25A5
LAMA55	3.87E-95	0.254426	0.116	0.027	6.17E-91	Skin_Vascl	LAMA5
HNRNPF3	6.69E-95	0.52505	0.539	0.304	1.07E-90	Skin_Vascl	HNRNPF
STRAP3	7.31E-95	0.507212	0.424	0.214	1.17E-90	Skin_Vascl	STRAP
JMJD1C4	9.02E-95	0.648717	0.442	0.232	1.44E-90	Skin_Vascl	JMJD1C
NPC24	1.19E-94	0.350123	0.698	0.436	1.9E-90	Skin_Vascl	NPC2
AC009501.	1.42E-94	0.320965	0.127	0.032	2.26E-90	Skin_Vascl	AC009501.4
KAT6A2	2.68E-94	0.371677	0.213	0.074	4.28E-90	Skin_Vascl	KAT6A
MBNL26	3.78E-94	0.484537	0.282	0.116	6.03E-90	Skin_Vascl	MBNL2
PPTC74	4.81E-94	0.345179	0.191	0.061	7.67E-90	Skin_Vascl	PPTC7
TRIM251	5.44E-94	0.387909	0.188	0.06	8.68E-90	Skin_Vascl	TRIM25
NXN1	5.74E-94	0.411013	0.207	0.071	9.16E-90	Skin_Vascl	NXN
SH2B33	8.8E-94	0.250529	0.12	0.029	1.4E-89	Skin_Vascl	SH2B3
PXDN2	1.99E-93	0.290908	0.14	0.038	3.17E-89	Skin_Vascl	PXDN
CARD83	2.13E-93	0.318459	0.172	0.052	3.4E-89	Skin_Vascl	CARD8
HEG12	2.96E-93	0.286982	0.23	0.08	4.73E-89	Skin_Vascl	HEG1
EIF4B6	4.54E-93	0.513699	0.529	0.296	7.24E-89	Skin_Vascl	EIF4B
LINC00998	5.07E-93	0.361529	0.197	0.066	8.08E-89	Skin_Vascl	LINC00998
BTG29	8.56E-93	0.290019	0.476	0.229	1.36E-88	Skin_Vascl	BTG2
PPFIBP12	1.22E-92	0.390363	0.262	0.103	1.95E-88	Skin_Vascl	PPFIBP1
METTL21A	1.34E-92	0.452704	0.208	0.072	2.14E-88	Skin_Vascl	METTL21A
TRMT1125	1.57E-92	0.495965	0.63	0.37	2.5E-88	Skin_Vascl	TRMT112
TRIM223	2.19E-92	0.268703	0.17	0.051	3.49E-88	Skin_Vascl	TRIM22
SELM11	2.32E-92	0.288432	0.649	0.342	3.7E-88	Skin_Vascl	SELM
NUCB13	2.38E-92	0.484575	0.409	0.203	3.79E-88	Skin_Vascl	NUCB1
RTN46	2.39E-92	0.4054	0.657	0.39	3.81E-88	Skin_Vascl	RTN4
ZNF222	7.1E-92	0.401658	0.267	0.106	1.13E-87	Skin_Vascl	ZNF22
VGLL45	2.48E-91	0.519754	0.304	0.133	3.95E-87	Skin_Vascl	VGLL4
ZFAND13	5.34E-91	0.473487	0.273	0.111	8.51E-87	Skin_Vascl	ZFAND1
ANKRD402	6.94E-91	0.338821	0.16	0.048	1.11E-86	Skin_Vascl	ANKRD40
RPS238	1.96E-90	0.362963	0.995	0.811	3.12E-86	Skin_Vascl	RPS23
SRSF28	2.19E-90	0.443618	0.576	0.328	3.49E-86	Skin_Vascl	SRSF2
SEC14L12	3.04E-90	0.441574	0.34	0.154	4.85E-86	Skin_Vascl	SEC14L1
PLS31	1.41E-89	0.448774	0.355	0.16	2.25E-85	Skin_Vascl	PLS3
SYPL18	1.51E-89	0.492767	0.485	0.26	2.4E-85	Skin_Vascl	SYPL1
COQ10B3	3.07E-89	0.545257	0.32	0.144	4.89E-85	Skin_Vascl	COQ10B
C5orf243	4.29E-89	0.466647	0.266	0.109	6.84E-85	Skin_Vascl	C5orf24
MAFK2	1.3E-88	0.332303	0.176	0.056	2.07E-84	Skin_Vascl	MAFK
AK41	2.79E-88	0.361769	0.172	0.056	4.46E-84	Skin_Vascl	AK4
NEDD85	2.93E-88	0.45152	0.668	0.419	4.68E-84	Skin_Vascl	NEDD8
DEDD	8.28E-88	0.344462	0.146	0.042	1.32E-83	Skin_Vascl	DEDD
SLC29A13	1.24E-87	0.314686	0.154	0.045	1.98E-83	Skin_Vascl	SLC29A1
RP11.295G	1.81E-87	0.444326	0.178	0.057	2.88E-83	Skin_Vascl	RP11.295G20.2
SBDS4	2.46E-87	0.379958	0.581	0.338	3.93E-83	Skin_Vascl	SBDS
BACE26	2.7E-87	0.498209	0.338	0.159	4.3E-83	Skin_Vascl	BACE2
TIMM103	3.14E-87	0.500317	0.332	0.153	5E-83	Skin_Vascl	TIMM10

PCBP23	3.45E-87	0.425925	0.671	0.417	5.51E-83	Skin_Vascl	PCBP2
EPB41L4A.	4.09E-87	0.400418	0.401	0.196	6.52E-83	Skin_Vascl	EPB41L4A.AS1
HNRNPH33	3.14E-86	0.428586	0.43	0.218	5.01E-82	Skin_Vascl	HNRNPH3
HOTAIRM1	4.78E-86	0.356515	0.299	0.125	7.63E-82	Skin_Vascl	HOTAIRM1
EIF13	5.07E-86	0.331862	0.988	0.908	8.09E-82	Skin_Vascl	EIF1
EIF3I1	6.27E-86	0.470701	0.453	0.241	9.99E-82	Skin_Vascl	EIF3I
PPM1D	6.37E-86	0.336312	0.155	0.047	1.02E-81	Skin_Vascl	PPM1D
AC016831.	7.32E-86	0.261081	0.087	0.018	1.17E-81	Skin_Vascl	AC016831.7
RGS25	7.65E-86	0.299463	0.25	0.095	1.22E-81	Skin_Vascl	RGS2
HPCAL11	7.81E-86	0.255708	0.214	0.076	1.25E-81	Skin_Vascl	HPCAL1
TMEM14A	1.78E-85	0.338784	0.224	0.082	2.84E-81	Skin_Vascl	TMEM14A
SUMO31	2.58E-85	0.481047	0.417	0.218	4.12E-81	Skin_Vascl	SUMO3
STX42	3.16E-85	0.365194	0.25	0.098	5.03E-81	Skin_Vascl	STX4
STARD3NL	5.48E-85	0.433235	0.222	0.084	8.75E-81	Skin_Vascl	STARD3NL
RAB213	6.83E-85	0.44777	0.348	0.163	1.09E-80	Skin_Vascl	RAB21
CYTH12	1.79E-84	0.40696	0.312	0.138	2.85E-80	Skin_Vascl	CYTH1
SEC11A6	1.82E-84	0.415944	0.542	0.311	2.9E-80	Skin_Vascl	SEC11A
CCNI8	2.01E-84	0.405868	0.759	0.514	3.2E-80	Skin_Vascl	CCNI
TAF4B2	2.3E-84	0.356328	0.12	0.031	3.67E-80	Skin_Vascl	TAF4B
RELB3	2.83E-84	0.32253	0.189	0.064	4.52E-80	Skin_Vascl	RELB
RAPGEF51	7.49E-84	0.31257	0.132	0.037	1.19E-79	Skin_Vascl	RAPGEF5
RPS6KA22	1.25E-83	0.273218	0.136	0.039	2E-79	Skin_Vascl	RPS6KA2
TBC1D91	1.43E-83	0.319904	0.144	0.042	2.29E-79	Skin_Vascl	TBC1D9
NFIA7	1.45E-83	0.394957	0.361	0.17	2.3E-79	Skin_Vascl	NFIA
ARMCX13	4.32E-83	0.334435	0.169	0.055	6.9E-79	Skin_Vascl	ARMCX1
VWA12	1.36E-82	0.432408	0.21	0.078	2.18E-78	Skin_Vascl	VWA1
C1orf545	1.54E-82	0.285505	0.153	0.046	2.46E-78	Skin_Vascl	C1orf54
CDKAL1	1.69E-82	0.304395	0.11	0.027	2.69E-78	Skin_Vascl	CDKAL1
FAU7	2.02E-82	0.282446	0.986	0.853	3.23E-78	Skin_Vascl	FAU
TNFSF9	3.36E-82	0.364979	0.086	0.018	5.35E-78	Skin_Vascl	TNFSF9
IMP34	3.89E-82	0.35513	0.378	0.184	6.21E-78	Skin_Vascl	IMP3
TBC1D4	6.79E-82	0.319778	0.159	0.051	1.08E-77	Skin_Vascl	TBC1D4
PARP142	8.97E-82	0.261585	0.214	0.078	1.43E-77	Skin_Vascl	PARP14
NAP1L14	1.14E-81	0.367479	0.66	0.397	1.81E-77	Skin_Vascl	NAP1L1
NFKB14	1.69E-81	0.33125	0.279	0.117	2.7E-77	Skin_Vascl	NFKB1
CTSH4	1.94E-81	0.478679	0.433	0.235	3.09E-77	Skin_Vascl	CTSH
TSPAN15	3.63E-81	0.310215	0.139	0.041	5.78E-77	Skin_Vascl	TSPAN15
IRF22	3.8E-81	0.361498	0.226	0.087	6.06E-77	Skin_Vascl	IRF2
HNRNPK4	9.3E-81	0.454635	0.677	0.443	1.48E-76	Skin_Vascl	HNRNPK
RSU13	1.07E-80	0.385515	0.28	0.12	1.7E-76	Skin_Vascl	RSU1
EIF3D1	3.33E-80	0.387317	0.502	0.281	5.31E-76	Skin_Vascl	EIF3D
TAPBP6	4.87E-80	0.301637	0.346	0.161	7.77E-76	Skin_Vascl	TAPBP
SNN2	6.07E-80	0.273546	0.14	0.041	9.69E-76	Skin_Vascl	SNN
TEAD2	8.11E-80	0.252403	0.104	0.026	1.29E-75	Skin_Vascl	TEAD2
JAK11	1.27E-79	0.408681	0.452	0.246	2.03E-75	Skin_Vascl	JAK1
COPS82	2.23E-79	0.384523	0.28	0.122	3.56E-75	Skin_Vascl	COPS8
BZW2	3.09E-79	0.305219	0.195	0.07	4.93E-75	Skin_Vascl	BZW2

HNRNPD5	3.47E-79	0.495043	0.432	0.237	5.54E-75	Skin_Vascl	HNRNPD
MICA1	6.19E-79	0.332113	0.169	0.056	9.87E-75	Skin_Vascl	MICA
GRPEL12	9.32E-79	0.44288	0.285	0.127	1.49E-74	Skin_Vascl	GRPEL1
SNRPD11	1.41E-78	0.382329	0.425	0.218	2.26E-74	Skin_Vascl	SNRPD1
TCEAL13	1.67E-78	0.291416	0.163	0.052	2.66E-74	Skin_Vascl	TCEAL1
WDR742	1.98E-78	0.349934	0.249	0.1	3.16E-74	Skin_Vascl	WDR74
UBXN12	2.3E-78	0.343908	0.626	0.388	3.67E-74	Skin_Vascl	UBXN1
TBC1D18	2.72E-78	0.28623	0.169	0.056	4.33E-74	Skin_Vascl	TBC1D1
CTA.29F11	4.97E-78	0.253879	0.193	0.068	7.92E-74	Skin_Vascl	CTA.29F11.1
NAB14	5.87E-78	0.342799	0.176	0.06	9.37E-74	Skin_Vascl	NAB1
SRP94	9.68E-78	0.481569	0.554	0.339	1.54E-73	Skin_Vascl	SRP9
KBTBD21	1.85E-77	0.295944	0.158	0.051	2.95E-73	Skin_Vascl	KBTBD2
FAM213A3	2.45E-77	0.51478	0.275	0.125	3.9E-73	Skin_Vascl	FAM213A
RPL2411	3.88E-77	0.320583	0.967	0.8	6.19E-73	Skin_Vascl	RPL24
ITGB1BP15	4.8E-77	0.312655	0.325	0.149	7.66E-73	Skin_Vascl	ITGB1BP1
SLC3A25	6.28E-77	0.33476	0.389	0.194	1E-72	Skin_Vascl	SLC3A2
PAN35	7.75E-77	0.410291	0.209	0.079	1.24E-72	Skin_Vascl	PAN3
OAF2	1.06E-76	0.260724	0.159	0.051	1.69E-72	Skin_Vascl	OAF
MYADM9	1.13E-76	0.362396	0.322	0.148	1.81E-72	Skin_Vascl	MYADM
EIF4A25	2.65E-76	0.443555	0.719	0.482	4.22E-72	Skin_Vascl	EIF4A2
PLIN23	4.36E-76	0.392818	0.281	0.124	6.95E-72	Skin_Vascl	PLIN2
RBM72	1.25E-75	0.436697	0.266	0.116	1.99E-71	Skin_Vascl	RBM7
ANKRD173	1.44E-75	0.425859	0.245	0.104	2.3E-71	Skin_Vascl	ANKRD17
SAP302	1.47E-75	0.304598	0.159	0.053	2.35E-71	Skin_Vascl	SAP30
PLOD21	2.55E-75	0.319795	0.13	0.038	4.07E-71	Skin_Vascl	PLOD2
TAF1D5	3.78E-75	0.436112	0.42	0.224	6.03E-71	Skin_Vascl	TAF1D
RANBP14	4.7E-75	0.35681	0.483	0.263	7.5E-71	Skin_Vascl	RANBP1
GNAQ7	7.47E-75	0.435504	0.33	0.161	1.19E-70	Skin_Vascl	GNAQ
ENAH7	8.53E-75	0.417853	0.264	0.116	1.36E-70	Skin_Vascl	ENAH
BCR	8.74E-75	0.416185	0.197	0.074	1.39E-70	Skin_Vascl	BCR
SLC25A64	1.16E-74	0.27056	0.859	0.563	1.86E-70	Skin_Vascl	SLC25A6
RPGR1	1.5E-74	0.331105	0.118	0.033	2.4E-70	Skin_Vascl	RPGR
PDLIM47	1.92E-74	0.391074	0.248	0.103	3.07E-70	Skin_Vascl	PDLIM4
FAM126A4	2.93E-74	0.284891	0.174	0.06	4.67E-70	Skin_Vascl	FAM126A
LARP62	3.14E-74	0.327898	0.208	0.079	5.01E-70	Skin_Vascl	LARP6
STAT5A1	3.27E-74	0.254191	0.138	0.042	5.21E-70	Skin_Vascl	STAT5A
FRYL2	3.88E-74	0.350834	0.22	0.087	6.19E-70	Skin_Vascl	FRYL
MARCKSL1	8.17E-74	0.365766	0.392	0.202	1.3E-69	Skin_Vascl	MARCKSL1
RSL1D13	1.04E-73	0.341294	0.456	0.245	1.66E-69	Skin_Vascl	RSL1D1
DEK8	1.2E-73	0.394651	0.46	0.256	1.91E-69	Skin_Vascl	DEK
MYCBP25	1.29E-73	0.347024	0.306	0.142	2.06E-69	Skin_Vascl	MYCBP2
SOX43	1.89E-73	0.44274	0.466	0.255	3.01E-69	Skin_Vascl	SOX4
NME12	2.74E-73	0.47292	0.329	0.162	4.37E-69	Skin_Vascl	NME1
PABPN17	3.16E-73	0.3955	0.311	0.148	5.04E-69	Skin_Vascl	PABPN1
RPL2214	3.85E-73	0.374229	0.932	0.69	6.14E-69	Skin_Vascl	RPL22
SETD54	6.71E-73	0.445701	0.262	0.116	1.07E-68	Skin_Vascl	SETD5
RAD23A1	1.01E-72	0.382901	0.391	0.203	1.62E-68	Skin_Vascl	RAD23A

DNAJB16	1.58E-72	0.419998	0.666	0.426	2.53E-68	Skin_Vascl	DNAJB1
RHOB4	2.08E-72	0.532921	0.381	0.198	3.32E-68	Skin_Vascl	RHOB
MYO1B4	2.43E-72	0.307881	0.191	0.07	3.87E-68	Skin_Vascl	MYO1B
TMEM165:	2.93E-72	0.485969	0.382	0.202	4.67E-68	Skin_Vascl	TMEM165
UBE2H3	3.72E-72	0.457673	0.3	0.144	5.93E-68	Skin_Vascl	UBE2H
PDGFA3	3.92E-72	0.267742	0.119	0.034	6.26E-68	Skin_Vascl	PDGFA
ALKBH22	4.89E-72	0.278926	0.142	0.045	7.79E-68	Skin_Vascl	ALKBH2
MATN24	5.66E-72	0.320113	0.134	0.041	9.03E-68	Skin_Vascl	MATN2
PFKFB34	8.6E-72	0.317219	0.231	0.094	1.37E-67	Skin_Vascl	PFKFB3
NUCKS18	1.8E-71	0.316022	0.498	0.28	2.87E-67	Skin_Vascl	NUCKS1
FILIP1L7	3.18E-71	0.272287	0.216	0.083	5.08E-67	Skin_Vascl	FILIP1L
RCN14	3.98E-71	0.404084	0.303	0.145	6.35E-67	Skin_Vascl	RCN1
PPP1R14B:	9.69E-71	0.419719	0.346	0.175	1.55E-66	Skin_Vascl	PPP1R14B
ARHGEF12	3.11E-70	0.438354	0.288	0.135	4.96E-66	Skin_Vascl	ARHGEF12
RAP1A3	3.61E-70	0.384769	0.47	0.272	5.75E-66	Skin_Vascl	RAP1A
ANAPC131	3.81E-70	0.433574	0.284	0.134	6.08E-66	Skin_Vascl	ANAPC13
WSB19	4.25E-70	0.384202	0.445	0.247	6.78E-66	Skin_Vascl	WSB1
HSPA86	5.43E-70	0.326029	0.744	0.507	8.66E-66	Skin_Vascl	HSPA8
SLIRP3	5.64E-70	0.481794	0.447	0.256	9E-66	Skin_Vascl	SLIRP
CD594	6.1E-70	0.324537	0.793	0.511	9.73E-66	Skin_Vascl	CD59
KLF104	2.42E-69	0.375295	0.3	0.14	3.86E-65	Skin_Vascl	KLF10
RBMX4	2.9E-69	0.288486	0.383	0.196	4.63E-65	Skin_Vascl	RBMX
WNK12	3.96E-69	0.361398	0.321	0.158	6.31E-65	Skin_Vascl	WNK1
ASXL13	4.05E-69	0.310044	0.199	0.077	6.45E-65	Skin_Vascl	ASXL1
MYBBP1A1	4.24E-69	0.25016	0.097	0.025	6.76E-65	Skin_Vascl	MYBBP1A
TACC18	9.2E-69	0.293248	0.341	0.164	1.47E-64	Skin_Vascl	TACC1
PPP4R22	1.47E-68	0.367234	0.271	0.125	2.34E-64	Skin_Vascl	PPP4R2
HMGN37	2.28E-68	0.323413	0.488	0.275	3.63E-64	Skin_Vascl	HMGN3
ARHGDIA5	2.82E-68	0.267708	0.382	0.194	4.5E-64	Skin_Vascl	ARHGDIA
RPL377	3.39E-68	0.292055	0.988	0.846	5.41E-64	Skin_Vascl	RPL37
NUFIP25	4.21E-68	0.412698	0.307	0.15	6.71E-64	Skin_Vascl	NUFIP2
RNPS12	5.38E-68	0.309822	0.38	0.196	8.57E-64	Skin_Vascl	RNPS1
BRD25	5.68E-68	0.438672	0.458	0.268	9.05E-64	Skin_Vascl	BRD2
RLIM	9.42E-68	0.345887	0.161	0.057	1.5E-63	Skin_Vascl	RLIM
FUS11	9.52E-68	0.327552	0.48	0.274	1.52E-63	Skin_Vascl	FUS
CTTNBP2N	2.38E-67	0.385641	0.245	0.107	3.8E-63	Skin_Vascl	CTTNBP2NL
PICALM1	3.54E-66	0.360845	0.439	0.245	5.64E-62	Skin_Vascl	PICALM
A4GALT3	4.02E-66	0.316503	0.209	0.085	6.41E-62	Skin_Vascl	A4GALT
TTC194	5.75E-66	0.394096	0.26	0.12	9.17E-62	Skin_Vascl	TTC19
RAB6A1	5.76E-66	0.419491	0.327	0.168	9.18E-62	Skin_Vascl	RAB6A
MCF2L2	6.64E-66	0.256038	0.1	0.027	1.06E-61	Skin_Vascl	MCF2L
EXOC61	7.7E-66	0.293046	0.121	0.037	1.23E-61	Skin_Vascl	EXOC6
EGR17	2.09E-65	0.641688	0.339	0.171	3.34E-61	Skin_Vascl	EGR1
CIRBP6	3.6E-65	0.284287	0.78	0.542	5.74E-61	Skin_Vascl	CIRBP
YY15	4.46E-65	0.418788	0.4	0.224	7.11E-61	Skin_Vascl	YY1
TRA2B4	5.13E-65	0.432073	0.483	0.286	8.19E-61	Skin_Vascl	TRA2B
RAB27A4	6.56E-65	0.288627	0.171	0.063	1.05E-60	Skin_Vascl	RAB27A

TSPAN91	6.79E-65	0.253547	0.106	0.03	1.08E-60	Skin_Vascl	TSPAN9
EIF2A3	1.43E-64	0.383628	0.303	0.151	2.28E-60	Skin_Vascl	EIF2A
NOB1	1.53E-64	0.269431	0.199	0.079	2.44E-60	Skin_Vascl	NOB1
SNAPC11	1.68E-64	0.361282	0.176	0.066	2.67E-60	Skin_Vascl	SNAPC1
GNS2	3.02E-64	0.339344	0.203	0.084	4.81E-60	Skin_Vascl	GNS
SIAH13	3.18E-64	0.408117	0.224	0.097	5.07E-60	Skin_Vascl	SIAH1
PSMA43	3.68E-64	0.371662	0.447	0.253	5.88E-60	Skin_Vascl	PSMA4
HNRNPH23	3.94E-64	0.336347	0.199	0.08	6.28E-60	Skin_Vascl	HNRNPH2
IKBIP3	7.6E-64	0.280086	0.163	0.059	1.21E-59	Skin_Vascl	IKBIP
WBP43	7.73E-64	0.395466	0.221	0.095	1.23E-59	Skin_Vascl	WBP4
PTP4A25	7.77E-64	0.401819	0.481	0.289	1.24E-59	Skin_Vascl	PTP4A2
IFRD13	8.1E-64	0.393174	0.397	0.217	1.29E-59	Skin_Vascl	IFRD1
HDGF3	8.19E-64	0.383543	0.282	0.136	1.31E-59	Skin_Vascl	HDGF
COX172	3.66E-63	0.536703	0.452	0.273	5.84E-59	Skin_Vascl	COX17
PKP46	3.99E-63	0.328941	0.188	0.076	6.36E-59	Skin_Vascl	PKP4
KLF113	4.55E-63	0.32085	0.178	0.069	7.25E-59	Skin_Vascl	KLF11
SYNJ22	5.48E-63	0.291161	0.143	0.049	8.75E-59	Skin_Vascl	SYNJ2
RHEB5	6.14E-63	0.38925	0.539	0.335	9.79E-59	Skin_Vascl	RHEB
SVIL7	6.24E-63	0.400039	0.255	0.118	9.95E-59	Skin_Vascl	SVIL
PPP1R15B3	7.47E-63	0.380452	0.305	0.15	1.19E-58	Skin_Vascl	PPP1R15B
RORA5	1.05E-62	0.288271	0.262	0.119	1.67E-58	Skin_Vascl	RORA
EIF5A8	1.05E-62	0.412704	0.462	0.276	1.67E-58	Skin_Vascl	EIF5A
SP32	1.98E-62	0.339906	0.215	0.091	3.16E-58	Skin_Vascl	SP3
RAB5A2	4.14E-62	0.453976	0.369	0.205	6.6E-58	Skin_Vascl	RAB5A
RAB5C1	4.22E-62	0.436453	0.431	0.253	6.73E-58	Skin_Vascl	RAB5C
ESD8	5.27E-62	0.368717	0.393	0.215	8.4E-58	Skin_Vascl	ESD
HNRNPM1	6.09E-62	0.283399	0.399	0.214	9.71E-58	Skin_Vascl	HNRNPM
SLC1A55	9.81E-62	0.376227	0.186	0.076	1.56E-57	Skin_Vascl	SLC1A5
ARL6IP13	9.82E-62	0.477458	0.504	0.315	1.57E-57	Skin_Vascl	ARL6IP1
DUT2	1.07E-61	0.290289	0.419	0.233	1.71E-57	Skin_Vascl	DUT
SELK6	1.09E-61	0.33314	0.681	0.466	1.74E-57	Skin_Vascl	SELK
SF16	1.57E-61	0.288932	0.414	0.231	2.51E-57	Skin_Vascl	SF1
ODF3B1	2.33E-61	0.253586	0.209	0.086	3.72E-57	Skin_Vascl	ODF3B
MEF2A5	6.29E-61	0.329954	0.235	0.104	1E-56	Skin_Vascl	MEF2A
FAM84B5	7.41E-61	0.381503	0.191	0.08	1.18E-56	Skin_Vascl	FAM84B
MAGOH2	1.05E-60	0.339778	0.336	0.175	1.68E-56	Skin_Vascl	MAGOH
SOD16	1.26E-60	0.298443	0.655	0.411	2.01E-56	Skin_Vascl	SOD1
PLEKHB23	1.59E-60	0.411441	0.292	0.147	2.54E-56	Skin_Vascl	PLEKHB2
ABCG1	1.7E-60	0.255309	0.184	0.072	2.71E-56	Skin_Vascl	ABCG1
COX7A2L1	2.33E-60	0.344991	0.521	0.318	3.71E-56	Skin_Vascl	COX7A2L
EIF2S33	4.24E-60	0.39412	0.305	0.154	6.77E-56	Skin_Vascl	EIF2S3
SMG16	5.88E-60	0.315815	0.255	0.119	9.38E-56	Skin_Vascl	SMG1
DNAJB64	1.29E-59	0.311526	0.641	0.416	2.06E-55	Skin_Vascl	DNAJB6
GNG122	1.82E-59	0.387947	0.356	0.192	2.91E-55	Skin_Vascl	GNG12
TRIM16	1.85E-59	0.496742	0.239	0.109	2.95E-55	Skin_Vascl	TRIM16
NMD33	2.69E-59	0.304216	0.186	0.075	4.28E-55	Skin_Vascl	NMD3
GBP11	4.96E-59	0.295103	0.163	0.061	7.91E-55	Skin_Vascl	GBP1

TNFAIP11	7.72E-59	0.297143	0.15	0.055	1.23E-54	Skin_Vascl	TNFAIP1
MRPS65	1.75E-58	0.328431	0.34	0.179	2.79E-54	Skin_Vascl	MRPS6
LAP33	2.25E-58	0.360245	0.256	0.124	3.59E-54	Skin_Vascl	LAP3
ERICH12	2.33E-58	0.283085	0.231	0.104	3.71E-54	Skin_Vascl	ERICH1
RRAGC3	2.44E-58	0.342153	0.226	0.101	3.89E-54	Skin_Vascl	RRAGC
PRKAR1A3	3.8E-58	0.404821	0.405	0.236	6.06E-54	Skin_Vascl	PRKAR1A
IPO76	8.73E-58	0.370706	0.225	0.102	1.39E-53	Skin_Vascl	IPO7
HMGN24	2.07E-57	0.32432	0.523	0.326	3.3E-53	Skin_Vascl	HMGN2
DNAJB96	2.16E-57	0.390449	0.372	0.205	3.44E-53	Skin_Vascl	DNAJB9
TNIP22	2.78E-57	0.282556	0.207	0.089	4.44E-53	Skin_Vascl	TNIP2
TOR3A2	3.48E-57	0.255689	0.144	0.052	5.55E-53	Skin_Vascl	TOR3A
CHTOP	4.01E-57	0.275503	0.225	0.101	6.4E-53	Skin_Vascl	CHTOP
RHOA4	6.45E-57	0.359752	0.673	0.471	1.03E-52	Skin_Vascl	RHOA
NUPR110	7.37E-57	0.253758	0.468	0.256	1.18E-52	Skin_Vascl	NUPR1
RFX22	9.04E-57	0.272449	0.135	0.047	1.44E-52	Skin_Vascl	RFX2
ITGAV4	9.64E-57	0.333586	0.212	0.094	1.54E-52	Skin_Vascl	ITGAV
PFDN11	1.31E-56	0.3517	0.243	0.114	2.09E-52	Skin_Vascl	PFDN1
LACTB2	1.34E-56	0.2778	0.198	0.084	2.13E-52	Skin_Vascl	LACTB
GRB101	1.47E-56	0.27648	0.126	0.043	2.34E-52	Skin_Vascl	GRB10
HNRNPU4	1.93E-56	0.355117	0.54	0.346	3.08E-52	Skin_Vascl	HNRNPU
TOB25	7.03E-56	0.270813	0.222	0.099	1.12E-51	Skin_Vascl	TOB2
ZNF5932	9.65E-56	0.365383	0.259	0.127	1.54E-51	Skin_Vascl	ZNF593
MIF3	1.77E-55	0.263384	0.716	0.478	2.83E-51	Skin_Vascl	MIF
CDC42EP4	2.06E-55	0.253553	0.148	0.055	3.29E-51	Skin_Vascl	CDC42EP4
WARS2	3.13E-55	0.316996	0.186	0.079	4.99E-51	Skin_Vascl	WARS
TOMM76	3.24E-55	0.251465	0.83	0.578	5.17E-51	Skin_Vascl	TOMM7
HSF1	7.43E-55	0.322435	0.239	0.113	1.18E-50	Skin_Vascl	HSF1
REV3L3	2.88E-54	0.305662	0.206	0.09	4.59E-50	Skin_Vascl	REV3L
SMIM122	4.65E-54	0.275218	0.225	0.104	7.42E-50	Skin_Vascl	SMIM12
SRI3	7.8E-54	0.288375	0.413	0.239	1.24E-49	Skin_Vascl	SRI
LYRM1	1.64E-53	0.414444	0.248	0.122	2.61E-49	Skin_Vascl	LYRM1
TSC22D24	2.69E-53	0.304019	0.229	0.105	4.29E-49	Skin_Vascl	TSC22D2
PPP1R105	4.62E-53	0.402351	0.287	0.151	7.38E-49	Skin_Vascl	PPP1R10
MRFAP14	5.39E-53	0.288174	0.442	0.264	8.6E-49	Skin_Vascl	MRFAP1
GABARAPL	5.44E-53	0.378019	0.308	0.165	8.67E-49	Skin_Vascl	GABARAPL1
RNF1310	9.29E-53	0.349168	0.306	0.165	1.48E-48	Skin_Vascl	RNF13
RASSF33	1.45E-52	0.361611	0.175	0.074	2.32E-48	Skin_Vascl	RASSF3
SAR1A5	1.53E-52	0.439914	0.386	0.23	2.44E-48	Skin_Vascl	SAR1A
BAZ1A4	1.53E-52	0.362149	0.412	0.242	2.44E-48	Skin_Vascl	BAZ1A
RBM65	1.56E-52	0.351221	0.23	0.109	2.49E-48	Skin_Vascl	RBM6
CHD99	2.21E-52	0.385511	0.306	0.165	3.52E-48	Skin_Vascl	CHD9
IMP42	2.38E-52	0.307819	0.263	0.132	3.79E-48	Skin_Vascl	IMP4
SEC61G7	3.66E-52	0.296998	0.631	0.411	5.84E-48	Skin_Vascl	SEC61G
MOB22	3.82E-52	0.307136	0.268	0.135	6.1E-48	Skin_Vascl	MOB2
FGFR1OP2	4.33E-52	0.299156	0.294	0.152	6.91E-48	Skin_Vascl	FGFR1OP2
ATF21	5.22E-51	0.315297	0.176	0.075	8.33E-47	Skin_Vascl	ATF2
KIAA0040	6.08E-51	0.266902	0.146	0.056	9.69E-47	Skin_Vascl	KIAA0040

MACF19	7.63E-51	0.327014	0.307	0.166	1.22E-46	Skin_Vascl	MACF1
ACBD33	9.15E-51	0.373658	0.241	0.121	1.46E-46	Skin_Vascl	ACBD3
CTTN3	1.74E-50	0.347465	0.357	0.205	2.77E-46	Skin_Vascl	CTTN
PQBP15	1.76E-50	0.270846	0.292	0.152	2.81E-46	Skin_Vascl	PQBP1
EGLN11	2.84E-50	0.299626	0.204	0.094	4.53E-46	Skin_Vascl	EGLN1
WAC3	6.41E-50	0.382069	0.36	0.21	1.02E-45	Skin_Vascl	WAC
PLCB13	1.99E-49	0.250148	0.112	0.039	3.17E-45	Skin_Vascl	PLCB1
RNF1684	2.8E-49	0.258151	0.21	0.096	4.46E-45	Skin_Vascl	RNF168
ADAR4	2.84E-49	0.315408	0.262	0.134	4.53E-45	Skin_Vascl	ADAR
C11orf583	3.57E-49	0.345422	0.546	0.365	5.69E-45	Skin_Vascl	C11orf58
DAPK32	5.22E-49	0.323188	0.19	0.084	8.33E-45	Skin_Vascl	DAPK3
IVNS1ABP9	5.84E-49	0.42204	0.277	0.148	9.31E-45	Skin_Vascl	IVNS1ABP
C19orf531	5.92E-49	0.292466	0.608	0.419	9.44E-45	Skin_Vascl	C19orf53
CYB5D21	6.83E-49	0.322159	0.163	0.068	1.09E-44	Skin_Vascl	CYB5D2
FIS13	9.51E-49	0.259139	0.527	0.335	1.52E-44	Skin_Vascl	FIS1
ARMCX35	1.07E-48	0.306571	0.274	0.144	1.71E-44	Skin_Vascl	ARMCX3
BRAF	1.08E-48	0.250938	0.134	0.051	1.72E-44	Skin_Vascl	BRAF
BRI38	1.25E-48	0.344138	0.526	0.344	2E-44	Skin_Vascl	BRI3
TSNAX	1.98E-48	0.303161	0.201	0.092	3.16E-44	Skin_Vascl	TSNAX
MAP1LC3A	2.48E-48	0.310179	0.361	0.208	3.96E-44	Skin_Vascl	MAP1LC3A
SELT6	2.71E-48	0.294281	0.382	0.225	4.32E-44	Skin_Vascl	SELT
UACA3	3.56E-48	0.353356	0.225	0.11	5.67E-44	Skin_Vascl	UACA
EIF4A35	3.75E-48	0.307685	0.346	0.197	5.99E-44	Skin_Vascl	EIF4A3
NONO3	4.16E-48	0.321372	0.332	0.189	6.63E-44	Skin_Vascl	NONO
ENSA1	6.66E-48	0.261556	0.393	0.233	1.06E-43	Skin_Vascl	ENSA
STRN33	6.9E-48	0.369814	0.197	0.091	1.1E-43	Skin_Vascl	STRN3
ELOVL53	8.13E-48	0.264032	0.223	0.106	1.3E-43	Skin_Vascl	ELOVL5
DNTTIP24	1.8E-47	0.272499	0.22	0.105	2.87E-43	Skin_Vascl	DNTTIP2
TMEM179I	2.1E-47	0.298089	0.342	0.196	3.35E-43	Skin_Vascl	TMEM179B
TMEM123	4.27E-47	0.263463	0.458	0.279	6.81E-43	Skin_Vascl	TMEM123
EPHX15	5.32E-47	0.261181	0.24	0.12	8.48E-43	Skin_Vascl	EPHX1
YWHAQ4	5.72E-47	0.281398	0.551	0.362	9.13E-43	Skin_Vascl	YWHAQ
HNRNPC5	6.39E-47	0.349795	0.619	0.427	1.02E-42	Skin_Vascl	HNRNPC
SPOP3	7.87E-47	0.324494	0.228	0.113	1.26E-42	Skin_Vascl	SPOP
DCTPP11	8.9E-47	0.280644	0.217	0.105	1.42E-42	Skin_Vascl	DCTPP1
SERTAD14	1.06E-46	0.274654	0.392	0.228	1.69E-42	Skin_Vascl	SERTAD1
SERTAD32	1.11E-46	0.366941	0.241	0.122	1.77E-42	Skin_Vascl	SERTAD3
TXN2	1.2E-46	0.310364	0.312	0.175	1.91E-42	Skin_Vascl	TXN2
TLE32	1.21E-46	0.263537	0.211	0.1	1.93E-42	Skin_Vascl	TLE3
FUBP12	1.23E-46	0.299879	0.206	0.098	1.95E-42	Skin_Vascl	FUBP1
CHMP32	1.32E-46	0.269591	0.304	0.166	2.1E-42	Skin_Vascl	CHMP3
CAMTA13	1.82E-46	0.336022	0.499	0.331	2.91E-42	Skin_Vascl	CAMTA1
GADD45GI	3.37E-46	0.255261	0.45	0.277	5.38E-42	Skin_Vascl	GADD45GIP1
BTG34	3.85E-46	0.278675	0.294	0.158	6.14E-42	Skin_Vascl	BTG3
LTBR5	4.08E-46	0.290277	0.19	0.087	6.51E-42	Skin_Vascl	LTBR
TERF2IP5	7.22E-46	0.255002	0.38	0.222	1.15E-41	Skin_Vascl	TERF2IP
RAB102	1.11E-45	0.3917	0.399	0.248	1.78E-41	Skin_Vascl	RAB10

PON22	1.6E-45	0.274687	0.163	0.07	2.56E-41	Skin_Vascl	PON2
LYRM41	9.74E-45	0.269244	0.226	0.111	1.55E-40	Skin_Vascl	LYRM4
TMEM230:	1.28E-44	0.272142	0.428	0.265	2.04E-40	Skin_Vascl	TMEM230
SRSF4	1.83E-44	0.3234	0.316	0.182	2.92E-40	Skin_Vascl	SRSF4
METAP22	2.49E-44	0.267947	0.323	0.18	3.98E-40	Skin_Vascl	METAP2
TES3	3.52E-44	0.297562	0.245	0.125	5.62E-40	Skin_Vascl	TES
ARID4B3	3.98E-44	0.291089	0.396	0.24	6.35E-40	Skin_Vascl	ARID4B
MAP47	6.4E-44	0.253154	0.247	0.127	1.02E-39	Skin_Vascl	MAP4
UHRF21	8.34E-44	0.309951	0.119	0.045	1.33E-39	Skin_Vascl	UHRF2
SMARCA24	1.24E-43	0.276084	0.222	0.112	1.98E-39	Skin_Vascl	SMARCA2
NFE2L14	1.28E-43	0.270701	0.191	0.09	2.03E-39	Skin_Vascl	NFE2L1
TBL1XR13	1.46E-43	0.315321	0.275	0.149	2.33E-39	Skin_Vascl	TBL1XR1
EIF1AX5	1.6E-43	0.310284	0.502	0.335	2.55E-39	Skin_Vascl	EIF1AX
BTBD73	1.71E-43	0.326182	0.199	0.097	2.72E-39	Skin_Vascl	BTBD7
KXD1	2.12E-43	0.291267	0.245	0.127	3.39E-39	Skin_Vascl	KXD1
ELF21	2.22E-43	0.3136	0.238	0.124	3.54E-39	Skin_Vascl	ELF2
LINC00116	2.31E-43	0.266609	0.226	0.114	3.69E-39	Skin_Vascl	LINC00116
MAPKAPK2	2.62E-43	0.285864	0.232	0.119	4.18E-39	Skin_Vascl	MAPKAPK2
PNPLA83	3.61E-43	0.322766	0.21	0.104	5.75E-39	Skin_Vascl	PNPLA8
SPEN3	4.35E-43	0.275844	0.21	0.103	6.94E-39	Skin_Vascl	SPEN
RQCD14	4.97E-43	0.300007	0.151	0.065	7.92E-39	Skin_Vascl	RQCD1
RNF54	8.55E-43	0.271834	0.264	0.141	1.36E-38	Skin_Vascl	RNF5
MCL16	1.05E-42	0.250694	0.558	0.378	1.67E-38	Skin_Vascl	MCL1
NUTF21	1.1E-42	0.27971	0.248	0.13	1.75E-38	Skin_Vascl	NUTF2
ADSS2	1.6E-42	0.289068	0.225	0.114	2.54E-38	Skin_Vascl	ADSS
ITGB17	2.87E-42	0.255341	0.536	0.354	4.57E-38	Skin_Vascl	ITGB1
BCL101	9.01E-42	0.369282	0.279	0.154	1.44E-37	Skin_Vascl	BCL10
CHORDC14	1.4E-41	0.285062	0.203	0.099	2.24E-37	Skin_Vascl	CHORDC1
SNRPG2	3.26E-41	0.302372	0.524	0.352	5.21E-37	Skin_Vascl	SNRPG
HNRNPAB7	5.69E-41	0.279547	0.293	0.165	9.08E-37	Skin_Vascl	HNRNPAB
EIF2S11	9.09E-41	0.292878	0.23	0.12	1.45E-36	Skin_Vascl	EIF2S1
PEA151	1.44E-40	0.302996	0.3	0.171	2.3E-36	Skin_Vascl	PEA15
STARD73	1.45E-40	0.253957	0.191	0.093	2.31E-36	Skin_Vascl	STARD7
PSMA36	2.03E-40	0.300106	0.322	0.192	3.24E-36	Skin_Vascl	PSMA3
DENND3	2.99E-40	0.260156	0.111	0.042	4.77E-36	Skin_Vascl	DENND3
EIF4G22	3.16E-40	0.266314	0.629	0.457	5.04E-36	Skin_Vascl	EIF4G2
MRPL323	4.05E-40	0.258328	0.251	0.135	6.46E-36	Skin_Vascl	MRPL32
GLUD14	5.16E-40	0.258229	0.288	0.161	8.23E-36	Skin_Vascl	GLUD1
ZNF4623	5.21E-40	0.271684	0.127	0.053	8.31E-36	Skin_Vascl	ZNF462
JMJD64	1.27E-39	0.252499	0.186	0.089	2.03E-35	Skin_Vascl	JMJD6
KHDRBS13	3.19E-39	0.259702	0.355	0.213	5.09E-35	Skin_Vascl	KHDRBS1
ARL6IP62	6.03E-39	0.250049	0.157	0.071	9.62E-35	Skin_Vascl	ARL6IP6
CELF14	9.05E-39	0.263174	0.232	0.123	1.44E-34	Skin_Vascl	CELF1
SHOC2	1.15E-38	0.335912	0.243	0.132	1.83E-34	Skin_Vascl	SHOC2
TOMM51	1.16E-38	0.35926	0.26	0.144	1.85E-34	Skin_Vascl	TOMM5
C1orf522	1.28E-38	0.28833	0.218	0.113	2.04E-34	Skin_Vascl	C1orf52
SYNGR23	1.34E-38	0.305261	0.369	0.232	2.14E-34	Skin_Vascl	SYNGR2

FBXO112	1.57E-38	0.281091	0.203	0.102	2.5E-34	Skin_Vascl	FBXO11
NTMT11	1.62E-38	0.257179	0.259	0.142	2.58E-34	Skin_Vascl	NTMT1
PSMA11	1.9E-38	0.287103	0.395	0.251	3.03E-34	Skin_Vascl	PSMA1
TAF132	2.92E-38	0.256462	0.121	0.049	4.66E-34	Skin_Vascl	TAF13
FLNB5	1.83E-37	0.255231	0.164	0.078	2.92E-33	Skin_Vascl	FLNB
SLC30A75	2.76E-37	0.320879	0.169	0.081	4.4E-33	Skin_Vascl	SLC30A7
EML42	3.19E-37	0.258368	0.224	0.118	5.09E-33	Skin_Vascl	EML4
SRSF14	4.2E-37	0.271144	0.269	0.152	6.7E-33	Skin_Vascl	SRSF1
YIPF53	9.44E-37	0.314877	0.217	0.116	1.51E-32	Skin_Vascl	YIPF5
RAI142	1E-36	0.255808	0.171	0.082	1.59E-32	Skin_Vascl	RAI14
NUDT45	1.02E-36	0.271411	0.266	0.151	1.62E-32	Skin_Vascl	NUDT4
CEBPZ2	1.56E-36	0.297582	0.224	0.119	2.5E-32	Skin_Vascl	CEBPZ
C1orf431	1.99E-36	0.266547	0.357	0.226	3.18E-32	Skin_Vascl	C1orf43
MED281	2.82E-36	0.27042	0.267	0.153	4.5E-32	Skin_Vascl	MED28
MCMBP	8.5E-36	0.251422	0.142	0.064	1.36E-31	Skin_Vascl	MCMBP
CLDND14	1.38E-35	0.2555	0.319	0.189	2.2E-31	Skin_Vascl	CLDND1
AK21	1.61E-35	0.317406	0.259	0.151	2.57E-31	Skin_Vascl	AK2
SEPT101	2.08E-35	0.29212	0.27	0.155	3.31E-31	Skin_Vascl	SEPT10
RNF1451	2.38E-35	0.269282	0.364	0.226	3.8E-31	Skin_Vascl	RNF145
GULP1	8.88E-35	0.318507	0.209	0.113	1.42E-30	Skin_Vascl	GULP1
RC3H1	1.04E-34	0.252368	0.176	0.087	1.65E-30	Skin_Vascl	RC3H1
DYNC1LI24	1.07E-34	0.29006	0.272	0.161	1.7E-30	Skin_Vascl	DYNC1LI2
LSM57	2.79E-34	0.262381	0.325	0.2	4.45E-30	Skin_Vascl	LSM5
TMEM170A	7.85E-34	0.251724	0.182	0.093	1.25E-29	Skin_Vascl	TMEM170A
BRD43	1.65E-33	0.254722	0.275	0.162	2.63E-29	Skin_Vascl	BRD4
LRRC11	2.45E-33	0.343016	0.161	0.08	3.91E-29	Skin_Vascl	LRRC1
FAM174A2	2.92E-33	0.261569	0.165	0.082	4.65E-29	Skin_Vascl	FAM174A
CDC42EP1	4.06E-33	0.277875	0.18	0.093	6.48E-29	Skin_Vascl	CDC42EP1
ATP5I3	4.23E-33	0.264212	0.534	0.37	6.75E-29	Skin_Vascl	ATP5I
PAK23	9.8E-33	0.25555	0.294	0.18	1.56E-28	Skin_Vascl	PAK2
KRAS1	1.44E-32	0.275916	0.302	0.186	2.29E-28	Skin_Vascl	KRAS
DHX153	2.31E-32	0.276622	0.204	0.111	3.69E-28	Skin_Vascl	DHX15
LRRC595	2.75E-31	0.26152	0.243	0.143	4.39E-27	Skin_Vascl	LRRC59
ATP13A33	3.06E-31	0.250583	0.179	0.093	4.87E-27	Skin_Vascl	ATP13A3
FOXC16	3.76E-30	0.259024	0.201	0.111	5.99E-26	Skin_Vascl	FOXC1
MKLN15	6.02E-30	0.251796	0.197	0.109	9.6E-26	Skin_Vascl	MKLN1
ETV63	1.09E-29	0.308697	0.196	0.109	1.73E-25	Skin_Vascl	ETV6
FBXL32	1.96E-29	0.28174	0.19	0.104	3.12E-25	Skin_Vascl	FBXL3
ABCE13	5.68E-29	0.263255	0.178	0.095	9.06E-25	Skin_Vascl	ABCE1
MRPL501	1.58E-28	0.26347	0.18	0.098	2.52E-24	Skin_Vascl	MRPL50
CHMP51	1.89E-28	0.27113	0.427	0.292	3.02E-24	Skin_Vascl	CHMP5
ZC3H151	2.86E-28	0.256543	0.344	0.228	4.56E-24	Skin_Vascl	ZC3H15
SNAP23	8.05E-28	0.263314	0.279	0.174	1.28E-23	Skin_Vascl	SNAP23
PDCD103	1.06E-27	0.283666	0.309	0.202	1.68E-23	Skin_Vascl	PDCD10
TIMM17A3	4.93E-27	0.255752	0.224	0.134	7.86E-23	Skin_Vascl	TIMM17A
MRPL171	1.02E-26	0.295197	0.174	0.097	1.62E-22	Skin_Vascl	MRPL17
PDCD47	2.19E-26	0.269678	0.286	0.183	3.5E-22	Skin_Vascl	PDCD4

FNDC3B5	3.65E-26	0.273661	0.243	0.149	5.83E-22	Skin_Vascl	FNDC3B
NDUFA4L2	3.71E-25	0.294984	0.145	0.075	5.91E-21	Skin_Vascl	NDUFA4L2
C1orf565	9.09E-25	0.2868	0.207	0.12	1.45E-20	Skin_Vascl	C1orf56
AAMDC2	8.95E-24	0.251954	0.241	0.152	1.43E-19	Skin_Vascl	AAMDC
MED211	5.89E-17	0.25583	0.214	0.144	9.39E-13	Skin_Vascl	MED21
IGFBP32	3.52E-14	0.303107	0.115	0.066	5.62E-10	Skin_Vascl	IGFBP3
CLDN52	0	3.679977	0.923	0.038	0	Skin_Eryth	CLDN5
TFPI5	0	3.370699	0.949	0.078	0	Skin_Eryth	TFPI
GNG115	0	3.175201	0.931	0.112	0	Skin_Eryth	GNG11
TFF34	0	2.820547	1	0.139	0	Skin_Eryth	TFF3
PROX12	0	2.526663	0.734	0.013	0	Skin_Eryth	PROX1
RAMP23	0	2.494738	0.828	0.073	0	Skin_Eryth	RAMP2
SNCG4	0	2.389603	0.81	0.117	0	Skin_Eryth	SNCG
SDPR4	0	2.250233	0.682	0.025	0	Skin_Eryth	SDPR
NTS1	0	1.883008	0.398	0.004	0	Skin_Eryth	NTS
CD2003	0	1.785566	0.398	0.019	0	Skin_Eryth	CD200
NRP23	0	1.578481	0.474	0.029	0	Skin_Eryth	NRP2
FXYD66	0	1.494206	0.511	0.028	0	Skin_Eryth	FXYD6
SCN3B	0	1.448267	0.456	0.008	0	Skin_Eryth	SCN3B
PARD6G2	0	1.295526	0.453	0.015	0	Skin_Eryth	PARD6G
TM4SF182	0	1.125981	0.394	0.017	0	Skin_Eryth	TM4SF18
FLT4	0	0.910666	0.299	0.003	0	Skin_Eryth	FLT4
GPM6A	0	0.841094	0.266	0.006	0	Skin_Eryth	GPM6A
BAALC2	0	0.827098	0.31	0.015	0	Skin_Eryth	BAALC
SLC24A1	0	0.819103	0.274	0.009	0	Skin_Eryth	SLC24A1
KBTBD11	0	0.704153	0.245	0.01	0	Skin_Eryth	KBTBD11
KLHL4	0	0.701666	0.186	0.001	0	Skin_Eryth	KLHL4
TIE12	0	0.688109	0.248	0.01	0	Skin_Eryth	TIE1
PIEZO2	0	0.652454	0.223	0.008	0	Skin_Eryth	PIEZO2
SEMA3A	0	0.641335	0.193	0.006	0	Skin_Eryth	SEMA3A
PKHD1L11	0	0.535488	0.175	0.004	0	Skin_Eryth	PKHD1L1
PTPRE5	2.3E-306	1.581096	0.558	0.052	3.6E-302	Skin_Eryth	PTPRE
MYCT12	1E-285	1.057644	0.365	0.024	1.7E-281	Skin_Eryth	MYCT1
ELK33	2.5E-280	1.540881	0.569	0.059	4.1E-276	Skin_Eryth	ELK3
NRN13	1.1E-277	1.1371	0.449	0.036	1.7E-273	Skin_Eryth	NRN1
PTX3	3.6E-276	0.522275	0.179	0.006	5.8E-272	Skin_Eryth	PTX3
DYSF	3.5E-274	0.507721	0.208	0.008	5.6E-270	Skin_Eryth	DYSF
PPFIBP13	5.2E-274	2.171942	0.715	0.104	8.3E-270	Skin_Eryth	PPFIBP1
DKK36	2.7E-270	1.262099	0.474	0.043	4.4E-266	Skin_Eryth	DKK3
EFEMP13	1.6E-269	1.751447	0.606	0.069	2.6E-265	Skin_Eryth	EFEMP1
KANK32	9.9E-265	0.962915	0.296	0.017	1.6E-260	Skin_Eryth	KANK3
EDNRB5	1.1E-263	0.984836	0.354	0.024	1.7E-259	Skin_Eryth	EDNRB
PALD11	1.6E-262	0.404593	0.153	0.005	2.5E-258	Skin_Eryth	PALD1
CTHRC12	7.9E-254	1.445598	0.558	0.061	1.3E-249	Skin_Eryth	CTHRC1
SEMA3D1	1.2E-253	1.004587	0.314	0.02	1.9E-249	Skin_Eryth	SEMA3D
PGM5.AS1	1.9E-250	0.368307	0.099	0.002	3E-246	Skin_Eryth	PGM5.AS1
APOLD14	6.9E-247	1.172829	0.383	0.03	1.1E-242	Skin_Eryth	APOLD1

STXBP62	1.1E-246	1.080225	0.391	0.032	1.8E-242	Skin_Eryth	STXBP6
SEMA6A2	2.4E-246	0.931799	0.365	0.028	3.8E-242	Skin_Eryth	SEMA6A
CHRDL12	1.6E-234	0.899331	0.35	0.026	2.5E-230	Skin_Eryth	CHRDL1
LMO23	5.1E-234	1.899348	0.631	0.089	8.1E-230	Skin_Eryth	LMO2
IGF14	5.6E-233	1.196661	0.412	0.036	8.9E-229	Skin_Eryth	IGF1
PLSCR44	4.3E-230	1.210525	0.46	0.046	6.8E-226	Skin_Eryth	PLSCR4
ANGPT24	1.5E-228	1.512824	0.35	0.027	2.4E-224	Skin_Eryth	ANGPT2
CNKSR31	7.2E-223	1.452725	0.493	0.056	1.1E-218	Skin_Eryth	CNKSR3
GATA22	1.7E-219	0.922128	0.321	0.024	2.7E-215	Skin_Eryth	GATA2
IGFBP76	2.2E-213	2.256563	0.993	0.263	3.4E-209	Skin_Eryth	IGFBP7
EGFL73	6.9E-212	1.481739	0.577	0.078	1.1E-207	Skin_Eryth	EGFL7
TSHZ27	2.3E-208	1.925457	0.661	0.109	3.6E-204	Skin_Eryth	TSHZ2
RASGRP32	1.9E-206	1.256887	0.434	0.047	3.1E-202	Skin_Eryth	RASGRP3
AKAP124	5E-206	1.934764	0.547	0.074	7.9E-202	Skin_Eryth	AKAP12
PGM53	2E-204	0.522787	0.212	0.011	3.2E-200	Skin_Eryth	PGM5
RGS165	2.3E-204	1.869204	0.609	0.087	3.7E-200	Skin_Eryth	RGS16
KCNN32	4.1E-204	0.574912	0.19	0.009	6.5E-200	Skin_Eryth	KCNN3
C16orf62	1.4E-203	1.229345	0.434	0.047	2.2E-199	Skin_Eryth	C16orf62
KIAA1456	1.8E-201	0.62322	0.193	0.01	2.8E-197	Skin_Eryth	KIAA1456
CALCRL3	2.1E-201	0.947805	0.365	0.033	3.3E-197	Skin_Eryth	CALCRL
NR2F26	1.9E-196	1.551493	0.588	0.086	3E-192	Skin_Eryth	NR2F2
NNMT4	6.5E-194	1.671182	0.675	0.109	1E-189	Skin_Eryth	NNMT
DTX1	6.5E-194	0.492326	0.135	0.005	1E-189	Skin_Eryth	DTX1
CAV18	1.2E-190	2.086475	0.876	0.197	1.9E-186	Skin_Eryth	CAV1
GIMAP83	2.7E-190	0.690175	0.226	0.014	4.3E-186	Skin_Eryth	GIMAP8
TBX12	4.6E-190	0.809033	0.288	0.022	7.4E-186	Skin_Eryth	TBX1
MSRB37	2.6E-186	0.944819	0.358	0.034	4.2E-182	Skin_Eryth	MSRB3
FABP55	1.4E-182	1.761891	0.704	0.134	2.3E-178	Skin_Eryth	FABP5
RAMP32	5.5E-181	0.993214	0.35	0.033	8.7E-177	Skin_Eryth	RAMP3
CRIP26	3.9E-180	2.047616	0.821	0.203	6.3E-176	Skin_Eryth	CRIP2
STON21	1.8E-179	0.646992	0.234	0.016	2.9E-175	Skin_Eryth	STON2
FLNC3	8.9E-179	0.555879	0.208	0.012	1.4E-174	Skin_Eryth	FLNC
TIMP37	1.8E-178	1.457092	0.807	0.165	2.9E-174	Skin_Eryth	TIMP3
ARHGAP29	2.6E-177	1.595307	0.635	0.113	4.2E-173	Skin_Eryth	ARHGAP29
KRT145	3.8E-176	0.681781	0.763	0.148	6E-172	Skin_Eryth	KRT14
S1PR12	1E-175	0.717918	0.259	0.019	1.6E-171	Skin_Eryth	S1PR1
CAV28	5.4E-175	1.686114	0.653	0.118	8.6E-171	Skin_Eryth	CAV2
CCND19	4E-174	1.766925	0.639	0.118	6.4E-170	Skin_Eryth	CCND1
CCL26	6.1E-169	1.825239	0.672	0.123	9.7E-165	Skin_Eryth	CCL2
DOCK53	1.5E-168	1.059606	0.314	0.03	2.3E-164	Skin_Eryth	DOCK5
EMCN3	4.1E-168	0.951324	0.365	0.038	6.5E-164	Skin_Eryth	EMCN
PDE2A2	4.8E-167	0.513704	0.193	0.011	7.6E-163	Skin_Eryth	PDE2A
UNC5B1	3.6E-164	0.454716	0.164	0.008	5.7E-160	Skin_Eryth	UNC5B
CD364	4.3E-164	0.594979	0.234	0.017	6.9E-160	Skin_Eryth	CD36
HYAL22	1.7E-163	1.285937	0.427	0.055	2.7E-159	Skin_Eryth	HYAL2
ST6GALNA	1.9E-162	0.397819	0.109	0.004	3.1E-158	Skin_Eryth	ST6GALNAC3
FAM89A1	1.4E-159	0.670683	0.245	0.019	2.3E-155	Skin_Eryth	FAM89A

TGFBR25	8.2E-156	1.486079	0.613	0.114	1.3E-151	Skin_Eryth	TGFBR2
LY965	4.1E-155	1.083917	0.478	0.068	6.5E-151	Skin_Eryth	LY96
RPS4Y19	1.5E-154	1.922777	0.88	0.272	2.4E-150	Skin_Eryth	RPS4Y1
LDB24	8.5E-154	0.990243	0.365	0.042	1.4E-149	Skin_Eryth	LDB2
ADCY42	1.1E-153	0.497961	0.204	0.014	1.8E-149	Skin_Eryth	ADCY4
AKR1C13	2.3E-152	1.01706	0.376	0.044	3.7E-148	Skin_Eryth	AKR1C1
MAF4	6.9E-151	1.374907	0.507	0.081	1.1E-146	Skin_Eryth	MAF
SOCS22	4.2E-150	1.091428	0.427	0.058	6.8E-146	Skin_Eryth	SOCS2
PCDH122	7.2E-150	0.319561	0.084	0.002	1.1E-145	Skin_Eryth	PCDH12
KDR2	3.1E-149	0.5271	0.175	0.011	4.9E-145	Skin_Eryth	KDR
FN13	5.8E-148	1.435433	0.566	0.097	9.3E-144	Skin_Eryth	FN1
CXCL24	2.1E-147	2.271041	0.712	0.162	3.4E-143	Skin_Eryth	CXCL2
TSPAN72	7.6E-147	0.783655	0.296	0.029	1.2E-142	Skin_Eryth	TSPAN7
ADIRF6	1.3E-145	2.131586	1	0.434	2.1E-141	Skin_Eryth	ADIRF
NUDT46	1.2E-143	1.600676	0.664	0.152	1.9E-139	Skin_Eryth	NUDT4
RCAN14	7.8E-143	1.300605	0.467	0.072	1.2E-138	Skin_Eryth	RCAN1
SGIP12	1.9E-141	0.396841	0.135	0.007	3E-137	Skin_Eryth	SGIP1
REEP11	2.1E-141	0.444557	0.168	0.01	3.4E-137	Skin_Eryth	REEP1
PTRF8	1.7E-140	1.372884	0.723	0.162	2.8E-136	Skin_Eryth	PTRF
SHC12	5.6E-139	1.10683	0.438	0.066	9E-135	Skin_Eryth	SHC1
C6orf1411	1.3E-138	0.822629	0.241	0.021	2E-134	Skin_Eryth	C6orf141
PEAR12	2.1E-136	0.378634	0.164	0.01	3.4E-132	Skin_Eryth	PEAR1
ACKR2	3.3E-135	0.391624	0.102	0.004	5.2E-131	Skin_Eryth	ACKR2
DMTN1	2.1E-132	0.609483	0.23	0.02	3.3E-128	Skin_Eryth	DMTN
CD912	3.2E-131	2.132339	0.971	0.452	5.1E-127	Skin_Eryth	CD9
GUCY1A33	6.3E-131	0.862401	0.296	0.033	1E-126	Skin_Eryth	GUCY1A3
KCTD125	3E-129	1.203114	0.515	0.092	4.9E-125	Skin_Eryth	KCTD12
BMP23	4.3E-129	0.939239	0.307	0.035	6.8E-125	Skin_Eryth	BMP2
HHEX3	6.9E-129	0.873707	0.285	0.03	1.1E-124	Skin_Eryth	HHEX
CNN34	2.7E-127	1.706953	0.766	0.223	4.2E-123	Skin_Eryth	CNN3
EFNA52	2E-126	0.794782	0.292	0.033	3.2E-122	Skin_Eryth	EFNA5
GPR1462	4.2E-126	0.586421	0.223	0.02	6.7E-122	Skin_Eryth	GPR146
CALD17	5.6E-126	1.29707	0.766	0.194	8.9E-122	Skin_Eryth	CALD1
LAYN2	4.5E-125	0.71111	0.266	0.028	7.2E-121	Skin_Eryth	LAYN
SNCA5	2.2E-124	0.817671	0.343	0.045	3.6E-120	Skin_Eryth	SNCA
RPL416	6.5E-124	1.728788	1	0.861	1E-119	Skin_Eryth	RPL41
TM4SF12	2.6E-123	1.533183	0.916	0.313	4.2E-119	Skin_Eryth	TM4SF1
ARL4A3	6.6E-122	1.549341	0.774	0.23	1E-117	Skin_Eryth	ARL4A
HIF3A2	3E-121	0.55948	0.197	0.016	4.8E-117	Skin_Eryth	HIF3A
ZNF5214	8.4E-119	0.633827	0.234	0.022	1.3E-114	Skin_Eryth	ZNF521
CYB5R36	8.9E-119	1.546786	0.745	0.22	1.4E-114	Skin_Eryth	CYB5R3
CLU5	1.2E-118	1.678352	0.73	0.199	2E-114	Skin_Eryth	CLU
SPTBN15	1.1E-117	1.375546	0.715	0.197	1.8E-113	Skin_Eryth	SPTBN1
THBD5	4.1E-117	0.765716	0.325	0.042	6.5E-113	Skin_Eryth	THBD
PTMS11	5.5E-117	1.580902	0.777	0.243	8.8E-113	Skin_Eryth	PTMS
MBOAT11	1.2E-115	0.585202	0.212	0.019	1.9E-111	Skin_Eryth	MBOAT1
EFNB23	2.9E-115	0.972192	0.35	0.05	4.6E-111	Skin_Eryth	EFNB2

CCDC1411	7.6E-115	0.462813	0.117	0.006	1.2E-110	Skin_Eryth	CCDC141
POLR2L5	1.2E-114	1.779718	0.953	0.472	2E-110	Skin_Eryth	POLR2L
RPS288	3.5E-114	1.355358	1	0.881	5.6E-110	Skin_Eryth	RPS28
SLC9A3R24	7.3E-114	1.421494	0.646	0.165	1.2E-109	Skin_Eryth	SLC9A3R2
VIM12	1.3E-113	1.525232	0.996	0.493	2.1E-109	Skin_Eryth	VIM
PHLDB26	1.4E-112	1.120226	0.354	0.052	2.2E-108	Skin_Eryth	PHLDB2
ETS17	6.4E-112	0.990483	0.442	0.076	1E-107	Skin_Eryth	ETS1
PDK43	1.7E-108	1.193559	0.303	0.04	2.8E-104	Skin_Eryth	PDK4
KRT54	1.9E-108	0.455347	0.453	0.078	3E-104	Skin_Eryth	KRT5
DLL42	2.1E-108	0.269924	0.117	0.006	3.4E-104	Skin_Eryth	DLL4
COL4A27	5.1E-107	0.846183	0.358	0.054	8.1E-103	Skin_Eryth	COL4A2
SHE2	8.7E-107	0.623614	0.215	0.021	1.4E-102	Skin_Eryth	SHE
PPP3CA8	1.7E-106	1.552295	0.602	0.154	2.7E-102	Skin_Eryth	PPP3CA
EFNA14	2.4E-106	1.199946	0.453	0.089	3.8E-102	Skin_Eryth	EFNA1
RPL347	3.8E-106	1.211381	1	0.919	6E-102	Skin_Eryth	RPL34
STOM6	7E-106	1.293645	0.613	0.152	1.1E-101	Skin_Eryth	STOM
RPL365	9.6E-106	1.268474	1	0.841	1.5E-101	Skin_Eryth	RPL36
SDCBP5	1.4E-105	1.579249	0.931	0.411	2.2E-101	Skin_Eryth	SDCBP
SNTB24	1.6E-105	1.021742	0.431	0.079	2.5E-101	Skin_Eryth	SNTB2
FZD63	1.2E-104	0.860828	0.285	0.037	1.9E-100	Skin_Eryth	FZD6
FSCN14	1.4E-104	0.886784	0.394	0.067	2.2E-100	Skin_Eryth	FSCN1
B3GNT72	3.8E-104	0.893129	0.23	0.025	6E-100	Skin_Eryth	B3GNT7
IFITM36	5.4E-104	1.279557	0.901	0.31	8.6E-100	Skin_Eryth	IFITM3
RP11.798M	4.7E-103	0.564769	0.208	0.021	7.5E-99	Skin_Eryth	RP11.798M19.6
LIMS15	1.4E-102	1.370611	0.62	0.167	2.17E-98	Skin_Eryth	LIMS1
SLC38A27	1.9E-102	1.612384	0.734	0.24	3.02E-98	Skin_Eryth	SLC38A2
TSPAN46	3.6E-101	1.117348	0.529	0.117	5.8E-97	Skin_Eryth	TSPAN4
WWTR13	6.8E-101	1.245132	0.54	0.129	1.08E-96	Skin_Eryth	WWTR1
SOX44	8.6E-101	2.077932	0.737	0.257	1.37E-96	Skin_Eryth	SOX4
PTMA7	3.4E-99	1.315884	1	0.819	5.44E-95	Skin_Eryth	PTMA
KLF46	3.6E-99	1.597609	0.704	0.225	5.79E-95	Skin_Eryth	KLF4
PDE1A1	5.6E-99	0.658376	0.288	0.038	8.92E-95	Skin_Eryth	PDE1A
OLFML2A6	5.6E-99	0.477753	0.219	0.023	8.93E-95	Skin_Eryth	OLFML2A
MID12	1.55E-98	0.655158	0.219	0.024	2.48E-94	Skin_Eryth	MID1
NT5E2	2.74E-98	0.527051	0.237	0.027	4.37E-94	Skin_Eryth	NT5E
UTRN6	1.3E-97	1.047959	0.515	0.115	2.07E-93	Skin_Eryth	UTRN
HSPB16	1.63E-97	1.28898	0.996	0.564	2.6E-93	Skin_Eryth	HSPB1
FOXP16	2.79E-97	1.539757	0.686	0.218	4.45E-93	Skin_Eryth	FOXP1
ABI3BP2	2.96E-97	0.74571	0.347	0.053	4.73E-93	Skin_Eryth	ABI3BP
PODXL5	3.46E-97	0.857618	0.321	0.05	5.51E-93	Skin_Eryth	PODXL
MPP7	2.79E-96	0.859298	0.318	0.049	4.44E-92	Skin_Eryth	MPP7
IGFBP46	1.2E-95	0.854372	0.573	0.129	1.92E-91	Skin_Eryth	IGFBP4
CEBPD4	1.87E-95	1.688867	0.843	0.336	2.99E-91	Skin_Eryth	CEBPD
VWF2	7.72E-95	0.691075	0.285	0.039	1.23E-90	Skin_Eryth	VWF
C2CD4B2	1.12E-94	0.757471	0.197	0.02	1.79E-90	Skin_Eryth	C2CD4B
ROBO3	1.62E-94	0.526322	0.179	0.017	2.59E-90	Skin_Eryth	ROBO3
GLIS3	9.2E-94	0.579956	0.193	0.02	1.47E-89	Skin_Eryth	GLIS3

TMEM255I	1.78E-93	0.513129	0.197	0.02	2.85E-89	Skin_Eryth	TMEM255B
CSGALNAC	3.9E-93	0.754123	0.296	0.043	6.22E-89	Skin_Eryth	CSGALNACT1
TCF46	1.2E-92	1.230015	0.631	0.173	1.92E-88	Skin_Eryth	TCF4
RP11.14N7	1.82E-92	0.673914	0.277	0.038	2.9E-88	Skin_Eryth	RP11.14N7.2
APP7	2.5E-92	1.449374	0.708	0.243	3.99E-88	Skin_Eryth	APP
RPS186	6.98E-92	1.096471	1	0.916	1.11E-87	Skin_Eryth	RPS18
LTC4S3	1.76E-91	0.67948	0.248	0.032	2.81E-87	Skin_Eryth	LTC4S
PGM2L12	1.21E-90	0.870203	0.292	0.044	1.93E-86	Skin_Eryth	PGM2L1
DOCK61	2.06E-90	0.556103	0.212	0.024	3.29E-86	Skin_Eryth	DOCK6
COX7A19	4.35E-90	1.355248	0.544	0.138	6.94E-86	Skin_Eryth	COX7A1
NBEAL16	8.64E-90	1.431068	0.839	0.319	1.38E-85	Skin_Eryth	NBEAL1
ATP5E4	1.32E-89	1.412873	0.982	0.684	2.1E-85	Skin_Eryth	ATP5E
NFATC12	1.49E-89	0.684373	0.285	0.041	2.37E-85	Skin_Eryth	NFATC1
TMOD12	4.36E-89	0.396139	0.153	0.013	6.95E-85	Skin_Eryth	TMOD1
SYNM3	6.62E-89	0.428707	0.186	0.019	1.06E-84	Skin_Eryth	SYNM
OAF3	7.87E-89	0.798364	0.321	0.052	1.26E-84	Skin_Eryth	OAF
RPL3915	1.4E-88	1.297914	1	0.676	2.23E-84	Skin_Eryth	RPL39
CPNE84	2.86E-88	0.845446	0.277	0.04	4.56E-84	Skin_Eryth	CPNE8
ZNF906	7.96E-88	0.999619	0.449	0.094	1.27E-83	Skin_Eryth	ZNF90
FCGRT6	2.16E-87	1.163993	0.766	0.277	3.45E-83	Skin_Eryth	FCGRT
HECW21	8.45E-87	0.351279	0.12	0.009	1.35E-82	Skin_Eryth	HECW2
PROCR4	1.61E-86	0.664211	0.237	0.03	2.56E-82	Skin_Eryth	PROCR
C10orf104	3.12E-86	1.188276	0.369	0.069	4.98E-82	Skin_Eryth	C10orf10
AL592183.	1.03E-85	0.517616	0.201	0.022	1.65E-81	Skin_Eryth	AL592183.1
SPHK13	1.17E-85	0.992897	0.35	0.064	1.86E-81	Skin_Eryth	SPHK1
NFIA8	1.53E-85	1.106942	0.599	0.172	2.45E-81	Skin_Eryth	NFIA
KIAA14622	2.33E-85	0.374972	0.135	0.011	3.72E-81	Skin_Eryth	KIAA1462
UBTD1	2.91E-85	0.66158	0.27	0.04	4.64E-81	Skin_Eryth	UBTD1
NUPR111	1.06E-84	1.251138	0.755	0.259	1.68E-80	Skin_Eryth	NUPR1
CDH52	7.05E-84	0.572282	0.212	0.025	1.13E-79	Skin_Eryth	CDH5
TMTC12	1.17E-83	0.494234	0.182	0.019	1.87E-79	Skin_Eryth	TMTC1
MARCKS7	1.34E-83	1.379862	0.748	0.283	2.14E-79	Skin_Eryth	MARCKS
MARCKSL1	4.87E-83	1.253248	0.642	0.204	7.77E-79	Skin_Eryth	MARCKSL1
SGCE5	1.05E-82	0.675736	0.266	0.039	1.68E-78	Skin_Eryth	SGCE
GYPC3	7.55E-82	1.034926	0.69	0.202	1.2E-77	Skin_Eryth	GYPC
BCL7C4	4.54E-81	0.997966	0.478	0.117	7.25E-77	Skin_Eryth	BCL7C
ARL6IP14	1.02E-80	1.439404	0.755	0.317	1.62E-76	Skin_Eryth	ARL6IP1
RBM37	1.41E-80	1.32039	0.872	0.439	2.25E-76	Skin_Eryth	RBM3
CNRIP17	1.74E-80	0.543824	0.263	0.038	2.78E-76	Skin_Eryth	CNRIP1
ITGA56	3.65E-80	0.545147	0.266	0.039	5.82E-76	Skin_Eryth	ITGA5
IPO111	3.84E-80	0.659273	0.23	0.031	6.12E-76	Skin_Eryth	IPO11
ZDHHC14	5.31E-80	0.55559	0.23	0.031	8.47E-76	Skin_Eryth	ZDHHC14
FKBP1A5	2.01E-79	1.35948	0.799	0.337	3.21E-75	Skin_Eryth	FKBP1A
ADD33	4.77E-79	1.101385	0.562	0.16	7.6E-75	Skin_Eryth	ADD3
ASGR1	5E-79	0.447191	0.226	0.03	7.97E-75	Skin_Eryth	ASGR1
C10orf128	1E-78	0.50037	0.186	0.021	1.6E-74	Skin_Eryth	C10orf128
THSD7A2	2.77E-78	0.380406	0.164	0.017	4.41E-74	Skin_Eryth	THSD7A

EHD27	8.97E-78	0.700175	0.281	0.045	1.43E-73	Skin_Eryth	EHD2
ZNF4671	2.39E-76	0.484941	0.201	0.025	3.81E-72	Skin_Eryth	ZNF467
SMAD12	4.01E-76	0.927188	0.307	0.056	6.4E-72	Skin_Eryth	SMAD1
AKR1C22	8.91E-76	0.559971	0.208	0.027	1.42E-71	Skin_Eryth	AKR1C2
NFIB13	1.56E-75	1.086558	0.62	0.19	2.49E-71	Skin_Eryth	NFIB
SOX73	1.9E-75	0.462959	0.182	0.021	3.03E-71	Skin_Eryth	SOX7
S100A107	4.6E-75	1.129681	1	0.675	7.34E-71	Skin_Eryth	S100A10
FGL25	4.74E-75	0.902551	0.438	0.098	7.55E-71	Skin_Eryth	FGL2
HLA.E5	7.18E-75	1.148947	0.953	0.609	1.15E-70	Skin_Eryth	HLA.E
C5orf244	7.06E-74	1.030006	0.442	0.111	1.13E-69	Skin_Eryth	C5orf24
RP11.390P	7.46E-74	0.352652	0.124	0.01	1.19E-69	Skin_Eryth	RP11.390P2.4
GGT55	8.4E-74	0.46454	0.197	0.024	1.34E-69	Skin_Eryth	GGT5
GADD45B4	1.86E-73	1.539229	0.818	0.376	2.96E-69	Skin_Eryth	GADD45B
COLEC122	3.37E-73	0.408268	0.215	0.029	5.38E-69	Skin_Eryth	COLEC12
PTPN145	3.95E-73	0.72094	0.307	0.057	6.29E-69	Skin_Eryth	PTPN14
MGST25	1.23E-72	1.336506	0.595	0.208	1.96E-68	Skin_Eryth	MGST2
AKR1C34	3E-72	0.823371	0.318	0.059	4.79E-68	Skin_Eryth	AKR1C3
YBX16	6.16E-71	0.976614	0.942	0.588	9.82E-67	Skin_Eryth	YBX1
HSPA1A9	6.44E-71	1.069243	0.792	0.325	1.03E-66	Skin_Eryth	HSPA1A
GIMAP63	1.16E-70	0.453139	0.219	0.03	1.84E-66	Skin_Eryth	GIMAP6
ADAMTS44	1.47E-70	0.49522	0.226	0.032	2.35E-66	Skin_Eryth	ADAMTS4
LINC00924	3.13E-70	0.391441	0.073	0.004	4.99E-66	Skin_Eryth	LINC00924
ACTN16	5.54E-70	1.257177	0.5	0.147	8.84E-66	Skin_Eryth	ACTN1
PRSS236	5.64E-70	0.906395	0.409	0.094	9E-66	Skin_Eryth	PRSS23
ANGPTL23	9.65E-70	0.483301	0.223	0.032	1.54E-65	Skin_Eryth	ANGPTL2
CLIC25	1.51E-69	0.640033	0.274	0.047	2.41E-65	Skin_Eryth	CLIC2
PLA2G167	1.92E-69	0.993985	0.467	0.125	3.06E-65	Skin_Eryth	PLA2G16
SYNPO3	3.61E-69	0.325355	0.15	0.016	5.77E-65	Skin_Eryth	SYNPO
ID36	1.14E-68	0.859244	0.588	0.172	1.82E-64	Skin_Eryth	ID3
TUBB9	6.05E-68	1.12513	0.763	0.31	9.65E-64	Skin_Eryth	TUBB
DPYSL28	1.93E-67	0.878076	0.416	0.098	3.08E-63	Skin_Eryth	DPYSL2
TTN3	3.01E-67	0.302279	0.117	0.01	4.81E-63	Skin_Eryth	TTN
APOL32	4.3E-67	0.456778	0.215	0.031	6.86E-63	Skin_Eryth	APOL3
SEPP14	1.93E-66	0.789438	0.712	0.265	3.08E-62	Skin_Eryth	SEPP1
SCG3	7.58E-66	0.59634	0.091	0.007	1.21E-61	Skin_Eryth	SCG3
LMNA13	1.52E-65	1.026312	0.832	0.365	2.42E-61	Skin_Eryth	LMNA
TMSB105	4.08E-65	0.96221	1	0.879	6.51E-61	Skin_Eryth	TMSB10
CXCL34	4.7E-65	0.870237	0.485	0.133	7.49E-61	Skin_Eryth	CXCL3
YWHAH7	6.87E-65	1.10423	0.599	0.207	1.1E-60	Skin_Eryth	YWHAH
AFAP1L11	8.55E-65	0.383299	0.161	0.019	1.36E-60	Skin_Eryth	AFAP1L1
DNAJB43	1.06E-64	0.898397	0.416	0.105	1.69E-60	Skin_Eryth	DNAJB4
STMN11	1.16E-64	0.927215	0.599	0.19	1.85E-60	Skin_Eryth	STMN1
GIMAP44	1.27E-64	0.509179	0.361	0.075	2.02E-60	Skin_Eryth	GIMAP4
MAP3K16	1.85E-64	0.820559	0.318	0.067	2.95E-60	Skin_Eryth	MAP3K1
MORF4L15	5.3E-64	1.097401	0.869	0.457	8.45E-60	Skin_Eryth	MORF4L1
CALM29	5.55E-64	1.001199	0.964	0.562	8.86E-60	Skin_Eryth	CALM2
ARID5B8	6.43E-64	1.206594	0.69	0.268	1.03E-59	Skin_Eryth	ARID5B

H3F3A18	9.34E-64	1.050428	0.923	0.478	1.49E-59	Skin_Eryth	H3F3A
PDLIM48	9.59E-63	0.951709	0.409	0.105	1.53E-58	Skin_Eryth	PDLIM4
S100A67	9.96E-63	0.884299	0.993	0.713	1.59E-58	Skin_Eryth	S100A6
TEK2	1.54E-62	0.300013	0.099	0.008	2.45E-58	Skin_Eryth	TEK
TPPP36	2.12E-62	0.98009	0.431	0.109	3.39E-58	Skin_Eryth	TPPP3
RPL326	2.72E-62	0.854895	1	0.872	4.34E-58	Skin_Eryth	RPL32
BCAR12	2.77E-62	0.743271	0.332	0.074	4.42E-58	Skin_Eryth	BCAR1
SHISA21	3.64E-62	0.258147	0.099	0.008	5.8E-58	Skin_Eryth	SHISA2
MEF2C8	5.79E-62	0.589484	0.361	0.08	9.23E-58	Skin_Eryth	MEF2C
ARL24	1.11E-61	0.99622	0.599	0.21	1.76E-57	Skin_Eryth	ARL2
ARL152	1.76E-61	0.497001	0.175	0.023	2.81E-57	Skin_Eryth	ARL15
SLC25A65	2.02E-61	0.989581	0.938	0.567	3.22E-57	Skin_Eryth	SLC25A6
VAMP54	2.25E-61	0.854076	0.547	0.168	3.59E-57	Skin_Eryth	VAMP5
MAN1A15	3.33E-61	0.63741	0.372	0.086	5.3E-57	Skin_Eryth	MAN1A1
DMKN3	4.31E-61	0.485594	0.507	0.156	6.87E-57	Skin_Eryth	DMKN
SLC2A36	4.91E-61	0.875856	0.478	0.129	7.83E-57	Skin_Eryth	SLC2A3
SERPINB93	7.8E-61	0.485484	0.307	0.062	1.24E-56	Skin_Eryth	SERPINB9
ID14	9.79E-61	0.722433	0.423	0.108	1.56E-56	Skin_Eryth	ID1
KALRN	1.04E-60	0.438786	0.164	0.021	1.66E-56	Skin_Eryth	KALRN
RPL35A5	1.09E-60	0.845986	1	0.863	1.73E-56	Skin_Eryth	RPL35A
HNRNPA1L	1.18E-60	0.723962	0.263	0.049	1.88E-56	Skin_Eryth	HNRNPA1L2
GPRC5B1	1.76E-60	0.554977	0.23	0.038	2.81E-56	Skin_Eryth	GPRC5B
NTAN15	1.93E-60	0.901087	0.438	0.123	3.08E-56	Skin_Eryth	NTAN1
RPL156	2.02E-60	0.771543	1	0.895	3.23E-56	Skin_Eryth	RPL15
RPS156	2.26E-60	0.831875	1	0.881	3.6E-56	Skin_Eryth	RPS15
RHOC7	2.76E-60	1.079058	0.617	0.223	4.4E-56	Skin_Eryth	RHOC
ANXA24	5.24E-60	1.033121	0.967	0.597	8.36E-56	Skin_Eryth	ANXA2
TMSB4X6	9.03E-60	0.793928	1	0.871	1.44E-55	Skin_Eryth	TMSB4X
SCARF13	1.81E-59	0.284654	0.12	0.012	2.89E-55	Skin_Eryth	SCARF1
ANKDD1A	1.85E-59	0.393002	0.15	0.018	2.96E-55	Skin_Eryth	ANKDD1A
GIMAP13	2.21E-59	0.429988	0.303	0.059	3.52E-55	Skin_Eryth	GIMAP1
C1orf215	6.58E-59	0.851062	0.391	0.101	1.05E-54	Skin_Eryth	C1orf21
EIF4A16	2.51E-58	1.033267	0.803	0.352	4E-54	Skin_Eryth	EIF4A1
SSH11	3.45E-58	0.533771	0.19	0.028	5.51E-54	Skin_Eryth	SSH1
ITGA91	5E-58	0.254915	0.117	0.012	7.98E-54	Skin_Eryth	ITGA9
ACKR34	7.16E-58	0.518815	0.19	0.028	1.14E-53	Skin_Eryth	ACKR3
HMGN38	1.06E-57	1.058256	0.679	0.278	1.69E-53	Skin_Eryth	HMGN3
NFAT57	1.09E-57	0.903853	0.438	0.127	1.74E-53	Skin_Eryth	NFAT5
NFATC25	2.68E-57	0.677339	0.245	0.045	4.27E-53	Skin_Eryth	NFATC2
KLF68	3.64E-57	1.213568	0.796	0.401	5.81E-53	Skin_Eryth	KLF6
FLI13	4.62E-57	0.351989	0.234	0.04	7.37E-53	Skin_Eryth	FLI1
CKS1B6	5.59E-57	0.843593	0.464	0.139	8.92E-53	Skin_Eryth	CKS1B
F81	5.88E-57	0.36529	0.128	0.014	9.38E-53	Skin_Eryth	F8
AGAP2.AS1	6.16E-57	0.311829	0.124	0.013	9.83E-53	Skin_Eryth	AGAP2.AS1
RPL86	7.72E-57	0.798528	1	0.849	1.23E-52	Skin_Eryth	RPL8
FAM43A2	1.48E-56	0.586082	0.215	0.036	2.35E-52	Skin_Eryth	FAM43A
RPS277	2.08E-56	0.862812	1	0.895	3.32E-52	Skin_Eryth	RPS27

NUCKS19	2.91E-56	0.997062	0.682	0.283	4.64E-52	Skin_Eryth	NUCKS1
TMEM204:	2.95E-56	0.409325	0.234	0.04	4.7E-52	Skin_Eryth	TMEM204
MYL68	6.44E-56	0.834311	0.982	0.805	1.03E-51	Skin_Eryth	MYL6
CAMK2D2	6.47E-56	0.62274	0.281	0.059	1.03E-51	Skin_Eryth	CAMK2D
SH2B34	7.32E-56	0.441229	0.193	0.03	1.17E-51	Skin_Eryth	SH2B3
RPL37A6	8.77E-56	0.779795	0.996	0.856	1.4E-51	Skin_Eryth	RPL37A
ACVRL13	1.14E-55	0.398718	0.186	0.027	1.82E-51	Skin_Eryth	ACVRL1
CRIM16	1.37E-55	0.669414	0.332	0.078	2.18E-51	Skin_Eryth	CRIM1
STARD91	1.55E-55	0.419684	0.168	0.023	2.47E-51	Skin_Eryth	STARD9
EEF1A115	1.82E-55	0.778768	1	0.915	2.9E-51	Skin_Eryth	EEF1A1
RP11.345J4	1.84E-55	0.526633	0.19	0.029	2.93E-51	Skin_Eryth	RP11.345J4.5
RPS147	2.19E-55	0.789576	1	0.882	3.49E-51	Skin_Eryth	RPS14
TMEM140:	2.34E-55	0.415589	0.157	0.021	3.73E-51	Skin_Eryth	TMEM140
IRF82	2.68E-55	0.672727	0.234	0.042	4.27E-51	Skin_Eryth	IRF8
LAMC15	4.09E-55	0.747837	0.328	0.077	6.52E-51	Skin_Eryth	LAMC1
CAMK14	4.68E-55	0.538993	0.208	0.035	7.46E-51	Skin_Eryth	CAMK1
LGALS19	6.5E-55	0.64379	0.927	0.41	1.04E-50	Skin_Eryth	LGALS1
STK32B	9.15E-55	0.328332	0.135	0.016	1.46E-50	Skin_Eryth	STK32B
TSPAN181	1.2E-54	0.346478	0.139	0.017	1.91E-50	Skin_Eryth	TSPAN18
MTUS14	1.48E-54	0.977192	0.478	0.157	2.36E-50	Skin_Eryth	MTUS1
PLXND14	1.81E-54	0.351102	0.193	0.03	2.89E-50	Skin_Eryth	PLXND1
RP3.512B1	2.06E-54	0.397161	0.12	0.013	3.29E-50	Skin_Eryth	RP3.512B11.3
DDX10	2.08E-54	0.716379	0.237	0.045	3.32E-50	Skin_Eryth	DDX10
CREB54	3.76E-54	0.590545	0.274	0.056	6E-50	Skin_Eryth	CREB5
LOX1	4.98E-54	0.411908	0.219	0.037	7.94E-50	Skin_Eryth	LOX
EPHB41	7.44E-54	0.503114	0.193	0.031	1.19E-49	Skin_Eryth	EPHB4
NID13	2.27E-53	0.398822	0.182	0.027	3.62E-49	Skin_Eryth	NID1
SYNE16	1.53E-52	0.60967	0.274	0.057	2.43E-48	Skin_Eryth	SYNE1
HOXD11	2.01E-52	0.294606	0.139	0.017	3.21E-48	Skin_Eryth	HOXD1
H2AFZ6	2.59E-52	1.054544	0.814	0.438	4.13E-48	Skin_Eryth	H2AFZ
U2AF16	3.67E-52	0.9017	0.642	0.255	5.86E-48	Skin_Eryth	U2AF1
FAM126A5	6.6E-52	0.667751	0.281	0.061	1.05E-47	Skin_Eryth	FAM126A
SMTN5	6.65E-52	0.504468	0.226	0.042	1.06E-47	Skin_Eryth	SMTN
MTRNR2L8	6.66E-52	0.491792	0.737	0.285	1.06E-47	Skin_Eryth	MTRNR2L8
RARRES25	1.06E-51	0.690757	0.354	0.088	1.7E-47	Skin_Eryth	RARRES2
TGFB32	2.61E-51	0.326754	0.139	0.018	4.16E-47	Skin_Eryth	TGFB3
SLC16A111	2.68E-51	0.298629	0.117	0.013	4.27E-47	Skin_Eryth	SLC16A11
SLC43A35	4.23E-51	0.515045	0.197	0.033	6.74E-47	Skin_Eryth	SLC43A3
PMP227	1.61E-50	0.546967	0.471	0.138	2.57E-46	Skin_Eryth	PMP22
EIF3E8	2.12E-50	0.94132	0.847	0.448	3.38E-46	Skin_Eryth	EIF3E
CNTNAP3B	2.45E-50	0.338145	0.124	0.015	3.91E-46	Skin_Eryth	CNTNAP3B
FOXO14	3.84E-50	0.560793	0.237	0.047	6.13E-46	Skin_Eryth	FOXO1
AK42	4.14E-50	0.562671	0.263	0.057	6.6E-46	Skin_Eryth	AK4
KLHL31	4.35E-50	0.369869	0.128	0.016	6.94E-46	Skin_Eryth	KLHL3
DPYSL35	1.15E-49	0.552457	0.263	0.055	1.84E-45	Skin_Eryth	DPYSL3
LAPTM54	1.17E-49	0.531493	0.562	0.172	1.87E-45	Skin_Eryth	LAPTM5
SET9	1.26E-49	1.078546	0.675	0.31	2.01E-45	Skin_Eryth	SET

MBNL110	1.71E-49	0.888336	0.504	0.17	2.72E-45	Skin_Eryth	MBNL1
PDLIM39	2.56E-49	0.535985	0.266	0.057	4.09E-45	Skin_Eryth	PDLIM3
DPEP21	5.49E-49	0.297773	0.153	0.022	8.75E-45	Skin_Eryth	DPEP2
HNRNPA15	7.16E-49	0.870187	0.905	0.545	1.14E-44	Skin_Eryth	HNRNPA1
RAP1B6	1.1E-48	0.990738	0.639	0.269	1.76E-44	Skin_Eryth	RAP1B
MPDZ5	1.53E-48	0.376871	0.182	0.03	2.43E-44	Skin_Eryth	MPDZ
EPB41L25	1.9E-48	0.644555	0.35	0.091	3.04E-44	Skin_Eryth	EPB41L2
ELK42	2.05E-48	0.619869	0.288	0.068	3.27E-44	Skin_Eryth	ELK4
SLC39A142	2.69E-48	0.596267	0.248	0.052	4.29E-44	Skin_Eryth	SLC39A14
ITGB1BP16	3E-48	0.789525	0.46	0.151	4.78E-44	Skin_Eryth	ITGB1BP1
SLC41A11	3.03E-48	0.420267	0.157	0.023	4.83E-44	Skin_Eryth	SLC41A1
CASK2	3.74E-48	0.693373	0.248	0.053	5.97E-44	Skin_Eryth	CASK
PPARG	4.72E-48	0.388204	0.142	0.02	7.53E-44	Skin_Eryth	PPARG
RPL76	5.94E-48	0.785656	0.996	0.797	9.47E-44	Skin_Eryth	RPL7
GUK15	6.22E-48	0.911471	0.861	0.557	9.93E-44	Skin_Eryth	GUK1
KMT2E6	6.24E-48	1.303889	0.628	0.299	9.95E-44	Skin_Eryth	KMT2E
RPS216	6.37E-48	0.825917	0.974	0.672	1.02E-43	Skin_Eryth	RPS21
HNRNPH17	6.84E-48	1.029487	0.653	0.297	1.09E-43	Skin_Eryth	HNRNPH1
RPS6KA23	6.98E-48	0.455941	0.212	0.04	1.11E-43	Skin_Eryth	RPS6KA2
TSHZ13	7.23E-48	0.51118	0.208	0.038	1.15E-43	Skin_Eryth	TSHZ1
SCN3A	8.53E-48	0.314147	0.091	0.009	1.36E-43	Skin_Eryth	SCN3A
YWHAE5	1.22E-47	1.029357	0.752	0.397	1.94E-43	Skin_Eryth	YWHAE
RASIP12	1.48E-47	0.393276	0.142	0.02	2.36E-43	Skin_Eryth	RASIP1
RPL18A5	2.8E-47	0.718658	0.996	0.823	4.47E-43	Skin_Eryth	RPL18A
SMAGP	3.08E-47	0.722384	0.347	0.095	4.91E-43	Skin_Eryth	SMAGP
GJA43	3.55E-47	0.485607	0.131	0.017	5.65E-43	Skin_Eryth	GJA4
YBX37	4.11E-47	1.024032	0.843	0.495	6.55E-43	Skin_Eryth	YBX3
ASAP15	4.83E-47	0.695528	0.35	0.095	7.71E-43	Skin_Eryth	ASAP1
TSPAN5	1.35E-46	0.733248	0.31	0.081	2.16E-42	Skin_Eryth	TSPAN5
DST8	4.14E-46	0.573675	0.511	0.184	6.6E-42	Skin_Eryth	DST
RPS196	4.29E-46	0.727688	1	0.875	6.85E-42	Skin_Eryth	RPS19
NBL16	4.47E-46	0.554123	0.398	0.11	7.12E-42	Skin_Eryth	NBL1
RUNX1T15	5.09E-46	0.436935	0.212	0.04	8.11E-42	Skin_Eryth	RUNX1T1
RPS86	5.38E-46	0.731545	1	0.849	8.58E-42	Skin_Eryth	RPS8
RPS297	8.66E-46	0.750081	0.985	0.79	1.38E-41	Skin_Eryth	RPS29
C4orf326	9.61E-46	0.743981	0.31	0.081	1.53E-41	Skin_Eryth	C4orf32
ROCK28	1.01E-45	0.732075	0.339	0.095	1.61E-41	Skin_Eryth	ROCK2
CORO1C5	1.14E-45	0.872406	0.383	0.117	1.81E-41	Skin_Eryth	CORO1C
NXN2	1.56E-45	0.640196	0.292	0.073	2.49E-41	Skin_Eryth	NXN
PCNP6	1.99E-45	0.882727	0.609	0.259	3.18E-41	Skin_Eryth	PCNP
NFKBIA6	2.2E-45	0.887967	0.898	0.516	3.51E-41	Skin_Eryth	NFKBIA
RPL107	2.47E-45	0.678425	1	0.877	3.94E-41	Skin_Eryth	RPL10
MDFIC3	2.64E-45	0.606295	0.299	0.076	4.21E-41	Skin_Eryth	MDFIC
CRTAP7	2.97E-45	0.670205	0.383	0.115	4.74E-41	Skin_Eryth	CRTAP
FCGR2A2	3E-45	0.376454	0.212	0.04	4.78E-41	Skin_Eryth	FCGR2A
RPL236	3.37E-45	0.830306	0.949	0.683	5.37E-41	Skin_Eryth	RPL23
PALM3	4.02E-45	0.374702	0.153	0.023	6.41E-41	Skin_Eryth	PALM

MATR36	4.1E-45	0.829141	0.387	0.115	6.54E-41	Skin_Eryth	MATR3
PTPRM2	5.53E-45	0.480276	0.168	0.028	8.82E-41	Skin_Eryth	PTPRM
ELMO14	5.93E-45	0.400183	0.186	0.033	9.45E-41	Skin_Eryth	ELMO1
NME1.NM	8.96E-45	0.270307	0.161	0.025	1.43E-40	Skin_Eryth	NME1.NME2
RAB11A1	1.07E-44	1.029464	0.752	0.383	1.71E-40	Skin_Eryth	RAB11A
SLC38A17	1.29E-44	0.629721	0.347	0.099	2.05E-40	Skin_Eryth	SLC38A1
S100A136	1.96E-44	0.771367	0.617	0.256	3.13E-40	Skin_Eryth	S100A13
PDGFA4	2.69E-44	0.40883	0.19	0.035	4.29E-40	Skin_Eryth	PDGFA
LHFP7	3.19E-44	0.533102	0.332	0.086	5.09E-40	Skin_Eryth	LHFP
CTNNB17	3.99E-44	1.067274	0.471	0.171	6.37E-40	Skin_Eryth	CTNNB1
IL6ST7	7.19E-44	0.973313	0.54	0.222	1.15E-39	Skin_Eryth	IL6ST
RPS15A5	8.72E-44	0.668477	1	0.849	1.39E-39	Skin_Eryth	RPS15A
EGLN32	9.54E-44	0.935681	0.325	0.09	1.52E-39	Skin_Eryth	EGLN3
CD15112	1.09E-43	0.760063	0.562	0.22	1.73E-39	Skin_Eryth	CD151
PDLIM16	1.12E-43	0.805512	0.558	0.224	1.78E-39	Skin_Eryth	PDLIM1
RPS27A8	1.19E-43	0.693674	1	0.849	1.89E-39	Skin_Eryth	RPS27A
CD595	1.54E-43	0.83346	0.894	0.515	2.46E-39	Skin_Eryth	CD59
SMYD2	1.95E-43	0.540767	0.255	0.06	3.11E-39	Skin_Eryth	SMYD2
WBP53	2.12E-43	0.864212	0.485	0.18	3.38E-39	Skin_Eryth	WBP5
RASSF91	2.88E-43	0.300441	0.099	0.011	4.6E-39	Skin_Eryth	RASSF9
SYT12	5.54E-43	0.306931	0.084	0.008	8.84E-39	Skin_Eryth	SYT1
TNXB3	5.77E-43	0.367538	0.299	0.073	9.21E-39	Skin_Eryth	TNXB
FRY5	6.79E-43	0.348341	0.139	0.02	1.08E-38	Skin_Eryth	FRY
STX114	1.07E-42	0.601397	0.27	0.065	1.71E-38	Skin_Eryth	STX11
RPL27A5	1.47E-42	0.65298	1	0.836	2.35E-38	Skin_Eryth	RPL27A
EXOC3L1	1.73E-42	0.254459	0.08	0.008	2.76E-38	Skin_Eryth	EXOC3L1
CMTM33	1.83E-42	0.508732	0.299	0.076	2.92E-38	Skin_Eryth	CMTM3
NGFRAP14	3.47E-42	0.854129	0.522	0.206	5.53E-38	Skin_Eryth	NGFRAP1
RPS239	5.04E-42	0.66669	1	0.813	8.04E-38	Skin_Eryth	RPS23
NDRG15	6.14E-42	0.72613	0.427	0.143	9.79E-38	Skin_Eryth	NDRG1
ITIH57	8.01E-42	0.416942	0.201	0.039	1.28E-37	Skin_Eryth	ITIH5
CIRBP7	1.01E-41	0.811246	0.85	0.545	1.61E-37	Skin_Eryth	CIRBP
TAX1BP34	1.31E-41	0.647335	0.318	0.086	2.08E-37	Skin_Eryth	TAX1BP3
CFL23	1.57E-41	0.747621	0.332	0.097	2.5E-37	Skin_Eryth	CFL2
ELMSAN16	1.58E-41	0.681992	0.296	0.078	2.52E-37	Skin_Eryth	ELMSAN1
RAC16	2.03E-41	0.884032	0.85	0.547	3.23E-37	Skin_Eryth	RAC1
RP11.138A	2.68E-41	0.670347	0.219	0.047	4.27E-37	Skin_Eryth	RP11.138A9.1
RPL386	4.58E-41	0.693701	0.989	0.762	7.3E-37	Skin_Eryth	RPL38
RPL13A4	6.93E-41	0.641858	1	0.881	1.1E-36	Skin_Eryth	RPL13A
MAP1B5	1.35E-40	0.427266	0.328	0.089	2.15E-36	Skin_Eryth	MAP1B
TGFB21	1.39E-40	0.351703	0.135	0.02	2.22E-36	Skin_Eryth	TGFB2
HLA.G1	1.41E-40	0.368569	0.15	0.024	2.26E-36	Skin_Eryth	HLA.G
TMEM123!	2.42E-40	0.769068	0.631	0.281	3.86E-36	Skin_Eryth	TMEM123
LINC00998	3E-40	0.574592	0.266	0.067	4.78E-36	Skin_Eryth	LINC00998
CD475	3.32E-40	0.779597	0.551	0.229	5.29E-36	Skin_Eryth	CD47
RPS217	3.86E-40	0.54593	1	0.8	6.15E-36	Skin_Eryth	RPS2
TOX2	4.08E-40	0.255209	0.088	0.009	6.51E-36	Skin_Eryth	TOX2

PTPN125	4.63E-40	0.675645	0.35	0.107	7.38E-36	Skin_Eryth	PTPN12
LIX1L4	6.24E-40	0.429833	0.215	0.046	9.95E-36	Skin_Eryth	LIX1L
NOTCH11	7.56E-40	0.434835	0.186	0.037	1.21E-35	Skin_Eryth	NOTCH1
MSN10	7.94E-40	0.671494	0.453	0.156	1.27E-35	Skin_Eryth	MSN
PPP1R21	9.84E-40	0.975257	0.58	0.271	1.57E-35	Skin_Eryth	PPP1R2
TOMM77	1.2E-39	0.78076	0.891	0.582	1.92E-35	Skin_Eryth	TOMM7
STX71	1.44E-39	0.669641	0.38	0.124	2.29E-35	Skin_Eryth	STX7
MEGF61	1.99E-39	0.266939	0.124	0.018	3.18E-35	Skin_Eryth	MEGF6
ITGAV5	3.32E-39	0.698655	0.321	0.095	5.29E-35	Skin_Eryth	ITGAV
C7orf55.LU	4.69E-39	0.415269	0.215	0.046	7.48E-35	Skin_Eryth	C7orf55.LUC7L2
RPL216	5.12E-39	0.681757	1	0.814	8.17E-35	Skin_Eryth	RPL21
RPL318	5.61E-39	0.606371	1	0.768	8.94E-35	Skin_Eryth	RPL31
SLC35F62	6.29E-39	0.542714	0.226	0.052	1E-34	Skin_Eryth	SLC35F6
C9orf38	6.49E-39	0.636887	0.332	0.098	1.03E-34	Skin_Eryth	C9orf3
ERICH13	6.53E-39	0.577206	0.347	0.105	1.04E-34	Skin_Eryth	ERICH1
HOXB71	8.84E-39	0.297319	0.142	0.023	1.41E-34	Skin_Eryth	HOXB7
ELN3	1.91E-38	0.402325	0.241	0.056	3.05E-34	Skin_Eryth	ELN
COX162	3.86E-38	0.76118	0.369	0.12	6.15E-34	Skin_Eryth	COX16
FAM198B4	4.51E-38	0.421058	0.179	0.035	7.2E-34	Skin_Eryth	FAM198B
ANKH4	4.75E-38	0.346143	0.182	0.036	7.57E-34	Skin_Eryth	ANKH
RPL319	4.86E-38	0.576236	1	0.843	7.75E-34	Skin_Eryth	RPL3
MYCN1	5.29E-38	0.344452	0.091	0.011	8.43E-34	Skin_Eryth	MYCN
CTSL7	8.03E-38	0.571702	0.343	0.104	1.28E-33	Skin_Eryth	CTSL
RPL266	9.68E-38	0.660823	1	0.835	1.54E-33	Skin_Eryth	RPL26
RNF1525	1.13E-37	0.502043	0.179	0.036	1.81E-33	Skin_Eryth	RNF152
PFDN55	1.58E-37	0.655405	0.956	0.727	2.52E-33	Skin_Eryth	PFDN5
TMEM246	3.1E-37	0.282639	0.113	0.016	4.95E-33	Skin_Eryth	TMEM246
DSP4	3.22E-37	0.601755	0.464	0.173	5.13E-33	Skin_Eryth	DSP
TIMP18	3.72E-37	0.469206	0.796	0.381	5.94E-33	Skin_Eryth	TIMP1
WSB110	4.39E-37	0.863137	0.566	0.25	6.99E-33	Skin_Eryth	WSB1
ALDH1A21	5.31E-37	0.399998	0.142	0.024	8.47E-33	Skin_Eryth	ALDH1A2
RPL356	5.48E-37	0.611361	0.996	0.836	8.73E-33	Skin_Eryth	RPL35
SRP95	8.04E-37	0.853712	0.653	0.341	1.28E-32	Skin_Eryth	SRP9
FNIP24	1.09E-36	0.710674	0.303	0.09	1.74E-32	Skin_Eryth	FNIP2
ARHGEF10	1.38E-36	0.561722	0.223	0.053	2.21E-32	Skin_Eryth	ARHGEF10
CDC427	1.66E-36	0.903746	0.788	0.47	2.65E-32	Skin_Eryth	CDC42
RP11.294J	1.67E-36	0.405525	0.157	0.029	2.67E-32	Skin_Eryth	RP11.294J22.6
RPS95	1.81E-36	0.588505	0.996	0.836	2.89E-32	Skin_Eryth	RPS9
IFITM27	1.99E-36	0.450569	0.712	0.304	3.18E-32	Skin_Eryth	IFITM2
SRSF66	2.08E-36	0.823869	0.398	0.144	3.31E-32	Skin_Eryth	SRSF6
CHST151	2.32E-36	0.366103	0.139	0.023	3.69E-32	Skin_Eryth	CHST15
PCBP24	2.77E-36	0.812379	0.748	0.42	4.41E-32	Skin_Eryth	PCBP2
SH2D3C2	3.05E-36	0.279826	0.109	0.015	4.87E-32	Skin_Eryth	SH2D3C
GIPC22	3.14E-36	0.527525	0.146	0.026	5.01E-32	Skin_Eryth	GIPC2
PRCP4	3.27E-36	0.721733	0.365	0.125	5.22E-32	Skin_Eryth	PRCP
RPS206	4.2E-36	0.613608	0.982	0.795	6.7E-32	Skin_Eryth	RPS20
HS3ST11	4.81E-36	0.550219	0.226	0.055	7.68E-32	Skin_Eryth	HS3ST1

RP11.138A	6.75E-36	0.59097	0.208	0.046	1.08E-31	Skin_Eryth	RP11.138A9.2
RPL36A4	9.43E-36	0.658326	0.898	0.51	1.5E-31	Skin_Eryth	RPL36A
RALGDS3	1.17E-35	0.583889	0.27	0.074	1.87E-31	Skin_Eryth	RALGDS
DYNLL16	1.19E-35	0.798477	0.843	0.561	1.9E-31	Skin_Eryth	DYNLL1
CDKN1A5	1.29E-35	0.652709	0.599	0.259	2.05E-31	Skin_Eryth	CDKN1A
HSD17B11	1.39E-35	0.616144	0.405	0.138	2.22E-31	Skin_Eryth	HSD17B11
ATP2B17	1.44E-35	0.653408	0.42	0.15	2.3E-31	Skin_Eryth	ATP2B1
PLA2G4C	1.63E-35	0.314592	0.095	0.012	2.59E-31	Skin_Eryth	PLA2G4C
FAM213A4	1.83E-35	0.672069	0.365	0.127	2.92E-31	Skin_Eryth	FAM213A
FAM63B2	1.95E-35	0.628256	0.325	0.103	3.11E-31	Skin_Eryth	FAM63B
PRKAA22	2.16E-35	0.491313	0.172	0.035	3.45E-31	Skin_Eryth	PRKAA2
CD3202	2.67E-35	0.51991	0.318	0.095	4.26E-31	Skin_Eryth	CD320
HSP90AB1	3.66E-35	0.798038	0.865	0.57	5.84E-31	Skin_Eryth	HSP90AB1
LINC.PINT2	3.79E-35	0.383649	0.197	0.043	6.04E-31	Skin_Eryth	LINC.PINT
RPL2215	3.96E-35	0.668733	0.967	0.693	6.31E-31	Skin_Eryth	RPL22
TBPL12	4.4E-35	0.579924	0.307	0.091	7.01E-31	Skin_Eryth	TBPL1
SPSB13	5.9E-35	0.486695	0.234	0.058	9.4E-31	Skin_Eryth	SPSB1
EBPL4	6.02E-35	0.62953	0.383	0.135	9.61E-31	Skin_Eryth	EBPL
GADD45A4	6.82E-35	0.735631	0.526	0.214	1.09E-30	Skin_Eryth	GADD45A
RPL125	8.9E-35	0.614172	1	0.853	1.42E-30	Skin_Eryth	RPL12
RPS254	8.97E-35	0.537855	1	0.824	1.43E-30	Skin_Eryth	RPS25
CDK174	1.1E-34	0.428742	0.186	0.04	1.76E-30	Skin_Eryth	CDK17
RAB3IL13	1.1E-34	0.317398	0.168	0.033	1.76E-30	Skin_Eryth	RAB3IL1
NFKBID5	1.15E-34	0.395948	0.201	0.044	1.83E-30	Skin_Eryth	NFKBID
GAPDH8	1.48E-34	0.609983	0.964	0.764	2.36E-30	Skin_Eryth	GAPDH
RP11.488L	1.58E-34	0.321429	0.12	0.019	2.52E-30	Skin_Eryth	RP11.488L18.10
SEPW19	1.77E-34	0.829363	0.679	0.358	2.83E-30	Skin_Eryth	SEPW1
AC010642.	2.06E-34	0.267964	0.179	0.036	3.28E-30	Skin_Eryth	AC010642.1
STOX21	2.94E-34	0.42595	0.139	0.025	4.69E-30	Skin_Eryth	STOX2
MIF4	3.03E-34	0.75725	0.807	0.481	4.84E-30	Skin_Eryth	MIF
NINJ15	3.07E-34	0.609021	0.405	0.145	4.9E-30	Skin_Eryth	NINJ1
RPL23A4	4.43E-34	0.542736	0.996	0.803	7.06E-30	Skin_Eryth	RPL23A
GNAI26	5.24E-34	0.790986	0.515	0.225	8.36E-30	Skin_Eryth	GNAI2
YES11	5.61E-34	0.603783	0.307	0.097	8.94E-30	Skin_Eryth	YES1
DNASE1L3	5.79E-34	0.255215	0.099	0.013	9.23E-30	Skin_Eryth	DNASE1L3
B2M6	6.6E-34	0.49648	1	0.929	1.05E-29	Skin_Eryth	B2M
VAMP33	7.01E-34	0.780322	0.372	0.135	1.12E-29	Skin_Eryth	VAMP3
G3BP15	1.04E-33	0.717565	0.423	0.161	1.65E-29	Skin_Eryth	G3BP1
MYH10	1.06E-33	0.431526	0.175	0.037	1.7E-29	Skin_Eryth	MYH10
SOCS37	1.11E-33	0.576743	0.445	0.164	1.78E-29	Skin_Eryth	SOCS3
KDM6B6	1.52E-33	0.656191	0.35	0.118	2.42E-29	Skin_Eryth	KDM6B
HNRNPA06	1.7E-33	0.670452	0.675	0.334	2.71E-29	Skin_Eryth	HNRNPA0
REL7	1.75E-33	0.809374	0.46	0.186	2.8E-29	Skin_Eryth	REL
SYPL19	1.85E-33	0.796455	0.562	0.263	2.95E-29	Skin_Eryth	SYPL1
CTD.2090I1	1.87E-33	0.333423	0.106	0.015	2.98E-29	Skin_Eryth	CTD.2090I13.1
RSL24D16	1.89E-33	0.694488	0.631	0.306	3.02E-29	Skin_Eryth	RSL24D1
HINT110	2.47E-33	0.689008	0.883	0.57	3.95E-29	Skin_Eryth	HINT1

RBMS36	2.92E-33	0.375922	0.215	0.052	4.65E-29	Skin_Eryth	RBMS3
YWHAG5	3.09E-33	0.944373	0.507	0.23	4.93E-29	Skin_Eryth	YWHAG
CD994	3.16E-33	0.749396	0.807	0.491	5.04E-29	Skin_Eryth	CD99
CAMKK22	3.39E-33	0.406719	0.197	0.046	5.41E-29	Skin_Eryth	CAMKK2
TP53TG1	3.67E-33	0.572801	0.292	0.089	5.86E-29	Skin_Eryth	TP53TG1
RPS116	4.18E-33	0.572211	0.982	0.777	6.66E-29	Skin_Eryth	RPS11
PXDC12	4.19E-33	0.648889	0.281	0.084	6.68E-29	Skin_Eryth	PXDC1
PLEKHG12	5.08E-33	0.27369	0.128	0.022	8.1E-29	Skin_Eryth	PLEKHG1
RPL275	5.74E-33	0.581417	0.982	0.75	9.15E-29	Skin_Eryth	RPL27
KLF26	5.78E-33	0.477198	0.496	0.191	9.22E-29	Skin_Eryth	KLF2
PROS15	5.95E-33	0.457422	0.193	0.043	9.48E-29	Skin_Eryth	PROS1
ICAM15	7.01E-33	0.712793	0.387	0.135	1.12E-28	Skin_Eryth	ICAM1
DAD19	8.32E-33	0.720986	0.719	0.398	1.33E-28	Skin_Eryth	DAD1
RAB314	8.47E-33	0.513413	0.307	0.094	1.35E-28	Skin_Eryth	RAB31
NAB15	9.93E-33	0.513023	0.234	0.062	1.58E-28	Skin_Eryth	NAB1
GLS9	1.1E-32	0.660918	0.347	0.117	1.75E-28	Skin_Eryth	GLS
POLE43	1.3E-32	0.586308	0.427	0.166	2.08E-28	Skin_Eryth	POLE4
COL18A16	1.75E-32	0.491256	0.263	0.073	2.79E-28	Skin_Eryth	COL18A1
TNFRSF43	1.85E-32	0.313671	0.15	0.028	2.96E-28	Skin_Eryth	TNFRSF4
ARID5A5	1.91E-32	0.504566	0.318	0.098	3.05E-28	Skin_Eryth	ARID5A
BMP2K2	2.57E-32	0.429971	0.186	0.043	4.1E-28	Skin_Eryth	BMP2K
SEMA4C3	2.89E-32	0.343378	0.139	0.025	4.61E-28	Skin_Eryth	SEMA4C
GOLIM45	3.52E-32	0.609124	0.281	0.084	5.62E-28	Skin_Eryth	GOLIM4
DAB24	3.62E-32	0.545327	0.307	0.094	5.78E-28	Skin_Eryth	DAB2
PDE7B1	3.67E-32	0.250584	0.08	0.01	5.85E-28	Skin_Eryth	PDE7B
ZBTB104	4.13E-32	0.586202	0.259	0.075	6.59E-28	Skin_Eryth	ZBTB10
RPS137	4.19E-32	0.547948	0.993	0.817	6.69E-28	Skin_Eryth	RPS13
RPS64	4.33E-32	0.389718	0.996	0.824	6.9E-28	Skin_Eryth	RPS6
ADAM101	5.54E-32	0.56114	0.259	0.076	8.83E-28	Skin_Eryth	ADAM10
SPRY23	7.61E-32	0.368427	0.146	0.028	1.21E-27	Skin_Eryth	SPRY2
AAED17	8.35E-32	0.42057	0.266	0.076	1.33E-27	Skin_Eryth	AAED1
TGFBR36	8.68E-32	0.393519	0.299	0.091	1.38E-27	Skin_Eryth	TGFBR3
S100A163	1.05E-31	0.649082	0.595	0.281	1.67E-27	Skin_Eryth	S100A16
NR4A27	1.13E-31	0.577321	0.467	0.185	1.81E-27	Skin_Eryth	NR4A2
CYR617	1.4E-31	0.410318	0.347	0.113	2.24E-27	Skin_Eryth	CYR61
LIPA2	1.45E-31	0.517106	0.259	0.073	2.31E-27	Skin_Eryth	LIPA
PLEKHA43	1.88E-31	0.338717	0.146	0.028	2.99E-27	Skin_Eryth	PLEKHA4
TNFAIP8L1	2.03E-31	0.409752	0.197	0.046	3.24E-27	Skin_Eryth	TNFAIP8L1
KRTCAP22	2.08E-31	0.752382	0.617	0.313	3.31E-27	Skin_Eryth	KRTCAP2
IRF14	2.59E-31	0.811747	0.496	0.215	4.13E-27	Skin_Eryth	IRF1
ANKRD403	3.2E-31	0.449548	0.201	0.049	5.11E-27	Skin_Eryth	ANKRD40
ID24	4.48E-31	0.47311	0.566	0.254	7.15E-27	Skin_Eryth	ID2
ZEB16	9.02E-31	0.42577	0.226	0.059	1.44E-26	Skin_Eryth	ZEB1
CLEC2B9	1.02E-30	0.568173	0.423	0.156	1.62E-26	Skin_Eryth	CLEC2B
SNX35	1.03E-30	0.722021	0.723	0.416	1.64E-26	Skin_Eryth	SNX3
CREM4	1.04E-30	0.562111	0.478	0.198	1.65E-26	Skin_Eryth	CREM
VAV3	1.07E-30	0.326276	0.131	0.024	1.71E-26	Skin_Eryth	VAV3

MITF1	1.22E-30	0.418759	0.182	0.042	1.95E-26	Skin_Eryth	MITF
USP313	1.7E-30	0.426905	0.168	0.037	2.71E-26	Skin_Eryth	USP31
CTTNBP22	1.75E-30	0.272467	0.088	0.012	2.79E-26	Skin_Eryth	CTTNBP2
ZFAS16	2.07E-30	0.586464	0.803	0.465	3.31E-26	Skin_Eryth	ZFAS1
COMMD66	2.2E-30	0.662951	0.803	0.473	3.5E-26	Skin_Eryth	COMMD6
SLC41A31	2.23E-30	0.477014	0.23	0.064	3.56E-26	Skin_Eryth	SLC41A3
BACE27	2.27E-30	0.71095	0.401	0.162	3.62E-26	Skin_Eryth	BACE2
ABLIM31	3.16E-30	0.341332	0.164	0.035	5.03E-26	Skin_Eryth	ABLIM3
TRMT1126	4.16E-30	0.665149	0.697	0.374	6.63E-26	Skin_Eryth	TRMT112
MARCH64	4.58E-30	0.78642	0.405	0.164	7.3E-26	Skin_Eryth	MARCH6
STAT39	4.96E-30	0.607931	0.456	0.19	7.91E-26	Skin_Eryth	STAT3
PEAK14	5.03E-30	0.37323	0.175	0.04	8.03E-26	Skin_Eryth	PEAK1
HMGB111	6.49E-30	0.685477	0.843	0.575	1.04E-25	Skin_Eryth	HMGB1
RP11.553L	6.79E-30	0.368189	0.204	0.052	1.08E-25	Skin_Eryth	RP11.553L6.5
PAFAH1B2	9.18E-30	0.615621	0.347	0.126	1.46E-25	Skin_Eryth	PAFAH1B2
PELI21	9.44E-30	0.336202	0.139	0.027	1.51E-25	Skin_Eryth	PELI2
GNG123	9.51E-30	0.64332	0.456	0.194	1.52E-25	Skin_Eryth	GNG12
SERPINE13	1.08E-29	0.413086	0.113	0.019	1.72E-25	Skin_Eryth	SERPINE1
TBC1D8	1.3E-29	0.331338	0.131	0.025	2.07E-25	Skin_Eryth	TBC1D8
RANBP15	1.6E-29	0.647432	0.566	0.266	2.55E-25	Skin_Eryth	RANBP1
QKI7	1.74E-29	0.684353	0.42	0.172	2.77E-25	Skin_Eryth	QKI
CSNK1A18	2.09E-29	0.68349	0.562	0.271	3.33E-25	Skin_Eryth	CSNK1A1
SULF22	2.14E-29	0.326964	0.153	0.032	3.41E-25	Skin_Eryth	SULF2
GGA12	2.16E-29	0.52974	0.336	0.119	3.45E-25	Skin_Eryth	GGA1
HSPA1B5	2.66E-29	0.527614	0.635	0.31	4.24E-25	Skin_Eryth	HSPA1B
MDK4	3.07E-29	0.64404	0.383	0.149	4.9E-25	Skin_Eryth	MDK
HYAL11	3.31E-29	0.254257	0.073	0.009	5.28E-25	Skin_Eryth	HYAL1
ANP32E6	4.28E-29	0.476733	0.299	0.097	6.82E-25	Skin_Eryth	ANP32E
ERF4	4.71E-29	0.550022	0.248	0.073	7.51E-25	Skin_Eryth	ERF
REEP3	4.86E-29	0.813843	0.438	0.195	7.76E-25	Skin_Eryth	REEP3
CDC42SE2!	6.09E-29	0.523935	0.339	0.12	9.71E-25	Skin_Eryth	CDC42SE2
SOD24	6.35E-29	0.455898	0.719	0.41	1.01E-24	Skin_Eryth	SOD2
RPS32	8.29E-29	0.288852	1	0.816	1.32E-24	Skin_Eryth	RPS3
EPB41L4A.	8.7E-29	0.627553	0.46	0.198	1.39E-24	Skin_Eryth	EPB41L4A.AS1
LRRC12	9.43E-29	0.589342	0.259	0.081	1.5E-24	Skin_Eryth	LRRC1
TSPYL24	1.01E-28	0.476164	0.281	0.088	1.61E-24	Skin_Eryth	TSPYL2
MORF4L25	1.08E-28	0.753246	0.584	0.301	1.72E-24	Skin_Eryth	MORF4L2
RPS55	1.16E-28	0.454986	1	0.786	1.86E-24	Skin_Eryth	RPS5
DAPK1	1.17E-28	0.399681	0.142	0.029	1.87E-24	Skin_Eryth	DAPK1
FCHSD21	1.51E-28	0.307848	0.142	0.029	2.4E-24	Skin_Eryth	FCHSD2
PPM1F2	2.13E-28	0.333346	0.146	0.031	3.39E-24	Skin_Eryth	PPM1F
TSPAN124	2.18E-28	0.424236	0.197	0.052	3.48E-24	Skin_Eryth	TSPAN12
ANKRD295	2.24E-28	0.309942	0.142	0.029	3.58E-24	Skin_Eryth	ANKRD29
ZFP36L17	2.61E-28	0.561851	0.788	0.469	4.17E-24	Skin_Eryth	ZFP36L1
SLC39A15	2.88E-28	0.532404	0.31	0.108	4.6E-24	Skin_Eryth	SLC39A1
CBX35	3.19E-28	0.558771	0.467	0.201	5.08E-24	Skin_Eryth	CBX3
SUMO32	3.34E-28	0.765931	0.474	0.221	5.33E-24	Skin_Eryth	SUMO3

ARRDC21	3.99E-28	0.358644	0.201	0.052	6.37E-24	Skin_Eryth	ARRDC2
CEP684	4.38E-28	0.433506	0.175	0.042	6.99E-24	Skin_Eryth	CEP68
RAB11FIP5	4.72E-28	0.523036	0.23	0.067	7.53E-24	Skin_Eryth	RAB11FIP5
MYLIP4	5.59E-28	0.506446	0.409	0.164	8.91E-24	Skin_Eryth	MYLIP
LAPTM4A6	6.44E-28	0.526478	0.828	0.518	1.03E-23	Skin_Eryth	LAPTM4A
RAPGEF52	7.27E-28	0.410198	0.164	0.038	1.16E-23	Skin_Eryth	RAPGEF5
BTG17	7.55E-28	0.547026	0.869	0.577	1.2E-23	Skin_Eryth	BTG1
CALHM25	7.71E-28	0.29367	0.157	0.034	1.23E-23	Skin_Eryth	CALHM2
NEDD86	8.16E-28	0.689587	0.704	0.423	1.3E-23	Skin_Eryth	NEDD8
JAG17	8.49E-28	0.345571	0.197	0.05	1.35E-23	Skin_Eryth	JAG1
GSTA43	8.69E-28	0.366847	0.175	0.042	1.39E-23	Skin_Eryth	GSTA4
EVA1A	9.86E-28	0.251923	0.084	0.012	1.57E-23	Skin_Eryth	EVA1A
HEY13	1.13E-27	0.506757	0.135	0.028	1.81E-23	Skin_Eryth	HEY1
ZFYVE216	1.33E-27	0.726206	0.365	0.149	2.11E-23	Skin_Eryth	ZFYVE21
MSMP	1.61E-27	0.27685	0.088	0.013	2.56E-23	Skin_Eryth	MSMP
EEF1D2	1.94E-27	0.351328	0.978	0.746	3.1E-23	Skin_Eryth	EEF1D
TLE11	2.28E-27	0.484959	0.23	0.068	3.63E-23	Skin_Eryth	TLE1
ERH6	2.63E-27	0.654483	0.606	0.311	4.2E-23	Skin_Eryth	ERH
SELT7	2.66E-27	0.644708	0.489	0.227	4.24E-23	Skin_Eryth	SELT
ELF15	2.78E-27	0.587283	0.42	0.178	4.44E-23	Skin_Eryth	ELF1
EID17	2.81E-27	0.609632	0.672	0.357	4.48E-23	Skin_Eryth	EID1
LTBP14	3.49E-27	0.340659	0.201	0.052	5.57E-23	Skin_Eryth	LTBP1
ITGA611	3.55E-27	0.561801	0.354	0.135	5.66E-23	Skin_Eryth	ITGA6
COX6C7	4.26E-27	0.645046	0.828	0.541	6.8E-23	Skin_Eryth	COX6C
IARS25	5.9E-27	0.579551	0.27	0.091	9.41E-23	Skin_Eryth	IARS2
SRSF38	6.06E-27	0.666292	0.737	0.433	9.67E-23	Skin_Eryth	SRSF3
RPS4X3	6.39E-27	0.366294	1	0.771	1.02E-22	Skin_Eryth	RPS4X
RPL113	7.79E-27	0.410786	1	0.854	1.24E-22	Skin_Eryth	RPL11
NR1D12	7.8E-27	0.366978	0.186	0.047	1.24E-22	Skin_Eryth	NR1D1
MYC4	9.45E-27	0.355205	0.365	0.129	1.51E-22	Skin_Eryth	MYC
RTN47	1.01E-26	0.6062	0.693	0.394	1.61E-22	Skin_Eryth	RTN4
SDC42	1.19E-26	0.628868	0.405	0.166	1.89E-22	Skin_Eryth	SDC4
EPS153	1.36E-26	0.538817	0.307	0.109	2.16E-22	Skin_Eryth	EPS15
KLF105	1.38E-26	0.575915	0.369	0.142	2.21E-22	Skin_Eryth	KLF10
INSIG14	1.53E-26	0.54036	0.453	0.207	2.44E-22	Skin_Eryth	INSIG1
SEPN11	2.25E-26	0.387049	0.172	0.043	3.58E-22	Skin_Eryth	SEPN1
SERBP15	2.54E-26	0.580228	0.653	0.354	4.05E-22	Skin_Eryth	SERBP1
EMP25	2.72E-26	0.533076	0.449	0.19	4.33E-22	Skin_Eryth	EMP2
HES12	3E-26	0.826112	0.478	0.22	4.79E-22	Skin_Eryth	HES1
FLOT12	3.5E-26	0.584134	0.361	0.142	5.58E-22	Skin_Eryth	FLOT1
SDC32	3.56E-26	0.331455	0.12	0.024	5.67E-22	Skin_Eryth	SDC3
PTPRK2	3.69E-26	0.479603	0.234	0.072	5.88E-22	Skin_Eryth	PTPRK
CNBP4	3.71E-26	0.586025	0.723	0.427	5.91E-22	Skin_Eryth	CNBP
MRPS66	3.9E-26	0.572948	0.423	0.181	6.22E-22	Skin_Eryth	MRPS6
TGIF16	6.43E-26	0.690742	0.438	0.196	1.03E-21	Skin_Eryth	TGIF1
RHEB6	8.47E-26	0.700355	0.606	0.338	1.35E-21	Skin_Eryth	RHEB
TNRC6B8	9.37E-26	0.627526	0.405	0.172	1.49E-21	Skin_Eryth	TNRC6B

C11orf584	1.22E-25	0.601131	0.661	0.368	1.95E-21	Skin_Eryth	C11orf58
PXN1	1.84E-25	0.426088	0.241	0.075	2.93E-21	Skin_Eryth	PXN
AHNAK10	1.94E-25	0.495774	0.693	0.385	3.09E-21	Skin_Eryth	AHNAK
SORBS14	1.95E-25	0.380111	0.15	0.035	3.11E-21	Skin_Eryth	SORBS1
SMARCA13	2.21E-25	0.409621	0.204	0.058	3.53E-21	Skin_Eryth	SMARCA1
AP1S25	2.29E-25	0.358781	0.347	0.125	3.65E-21	Skin_Eryth	AP1S2
EIF4E4	2.59E-25	0.561935	0.504	0.243	4.13E-21	Skin_Eryth	EIF4E
RALGAPA2	3.62E-25	0.475257	0.197	0.056	5.78E-21	Skin_Eryth	RALGAPA2
FHL15	3.78E-25	0.369948	0.354	0.129	6.03E-21	Skin_Eryth	FHL1
TCEAL45	3.82E-25	0.58535	0.38	0.159	6.09E-21	Skin_Eryth	TCEAL4
PXDN3	4.13E-25	0.385575	0.161	0.039	6.58E-21	Skin_Eryth	PXDN
ANKRD105	4.13E-25	0.514853	0.31	0.113	6.59E-21	Skin_Eryth	ANKRD10
TUBA1B10	4.57E-25	0.567539	0.664	0.354	7.29E-21	Skin_Eryth	TUBA1B
ZFP36L27	5.27E-25	0.368737	0.726	0.392	8.4E-21	Skin_Eryth	ZFP36L2
RIN23	6.33E-25	0.488344	0.234	0.073	1.01E-20	Skin_Eryth	RIN2
EPHX16	6.4E-25	0.543111	0.321	0.121	1.02E-20	Skin_Eryth	EPHX1
SRGAP24	6.57E-25	0.338127	0.186	0.05	1.05E-20	Skin_Eryth	SRGAP2
LAMB27	6.86E-25	0.513993	0.255	0.083	1.09E-20	Skin_Eryth	LAMB2
GLTSCR21	6.88E-25	0.352864	0.839	0.529	1.1E-20	Skin_Eryth	GLTSCR2
DBN13	7.28E-25	0.456783	0.19	0.052	1.16E-20	Skin_Eryth	DBN1
HSD3B73	7.69E-25	0.316717	0.15	0.035	1.23E-20	Skin_Eryth	HSD3B7
YPEL24	7.73E-25	0.60322	0.288	0.104	1.23E-20	Skin_Eryth	YPEL2
PAK24	8.33E-25	0.658824	0.409	0.181	1.33E-20	Skin_Eryth	PAK2
RHOB5	8.57E-25	0.661863	0.442	0.201	1.37E-20	Skin_Eryth	RHOB
ZDHHC173	8.77E-25	0.307427	0.15	0.035	1.4E-20	Skin_Eryth	ZDHHC17
CTSZ7	1.05E-24	0.374865	0.438	0.191	1.68E-20	Skin_Eryth	CTSZ
TMCO31	1.12E-24	0.412613	0.23	0.072	1.78E-20	Skin_Eryth	TMCO3
HIPK34	1.18E-24	0.484855	0.318	0.119	1.88E-20	Skin_Eryth	HIPK3
AAMDC3	1.19E-24	0.674168	0.361	0.153	1.9E-20	Skin_Eryth	AAMDC
ECE14	1.28E-24	0.38436	0.212	0.062	2.05E-20	Skin_Eryth	ECE1
CAMSAP25	1.39E-24	0.474316	0.234	0.073	2.22E-20	Skin_Eryth	CAMSAP2
NAP1L15	1.51E-24	0.583845	0.697	0.401	2.41E-20	Skin_Eryth	NAP1L1
KAT6A3	1.72E-24	0.441627	0.237	0.076	2.74E-20	Skin_Eryth	KAT6A
RASSF13	1.87E-24	0.47211	0.27	0.092	2.98E-20	Skin_Eryth	RASSF1
FMNL23	1.88E-24	0.307788	0.161	0.04	2.99E-20	Skin_Eryth	FMNL2
ATP5G22	1.93E-24	0.436649	0.858	0.572	3.08E-20	Skin_Eryth	ATP5G2
RPL193	2.25E-24	0.32764	1	0.849	3.59E-20	Skin_Eryth	RPL19
TPM41	2.74E-24	0.519018	0.741	0.422	4.37E-20	Skin_Eryth	TPM4
PEPD3	2.76E-24	0.492199	0.285	0.102	4.4E-20	Skin_Eryth	PEPD
TACC19	3.82E-24	0.530457	0.398	0.166	6.08E-20	Skin_Eryth	TACC1
WTAP5	3.89E-24	0.505947	0.54	0.281	6.2E-20	Skin_Eryth	WTAP
OST45	4.69E-24	0.62315	0.799	0.5	7.48E-20	Skin_Eryth	OST4
ESD9	4.99E-24	0.704421	0.456	0.217	7.96E-20	Skin_Eryth	ESD
TIMP22	5.13E-24	0.418562	0.661	0.35	8.18E-20	Skin_Eryth	TIMP2
WFS11	6.94E-24	0.392328	0.135	0.031	1.11E-19	Skin_Eryth	WFS1
BEST15	7.01E-24	0.25704	0.168	0.042	1.12E-19	Skin_Eryth	BEST1
ZC3HAV15	8.83E-24	0.42389	0.277	0.097	1.41E-19	Skin_Eryth	ZC3HAV1

SBDS5	9.78E-24	0.594966	0.602	0.341	1.56E-19	Skin_Eryth	SBDS
ARSJ	1.54E-23	0.313347	0.113	0.023	2.46E-19	Skin_Eryth	ARSJ
SKI4	1.67E-23	0.342241	0.204	0.06	2.66E-19	Skin_Eryth	SKI
MYL6B	1.7E-23	0.443793	0.318	0.121	2.71E-19	Skin_Eryth	MYL6B
CDC268	1.72E-23	0.555484	0.318	0.126	2.74E-19	Skin_Eryth	CDC26
SUMO16	2.4E-23	0.705097	0.609	0.347	3.83E-19	Skin_Eryth	SUMO1
FAM49A2	2.49E-23	0.303607	0.161	0.041	3.97E-19	Skin_Eryth	FAM49A
SLC7A13	2.64E-23	0.380084	0.179	0.05	4.21E-19	Skin_Eryth	SLC7A1
TMED210	2.7E-23	0.647838	0.613	0.345	4.31E-19	Skin_Eryth	TMED2
FOS10	3.9E-23	0.596053	0.752	0.425	6.22E-19	Skin_Eryth	FOS
SNRPF5	4.13E-23	0.507187	0.493	0.237	6.59E-19	Skin_Eryth	SNRPF
RAD23A2	4.23E-23	0.546319	0.445	0.206	6.74E-19	Skin_Eryth	RAD23A
S100A48	4.35E-23	0.364656	0.81	0.465	6.94E-19	Skin_Eryth	S100A4
FGFR14	4.59E-23	0.315711	0.263	0.087	7.32E-19	Skin_Eryth	FGFR1
EPAS14	4.76E-23	0.3822	0.263	0.09	7.59E-19	Skin_Eryth	EPAS1
CARD84	5.01E-23	0.339536	0.19	0.054	7.99E-19	Skin_Eryth	CARD8
CYB5B1	5.32E-23	0.528313	0.288	0.107	8.48E-19	Skin_Eryth	CYB5B
UBXN13	5.84E-23	0.502115	0.668	0.391	9.32E-19	Skin_Eryth	UBXN1
TMEM109	5.97E-23	0.410176	0.328	0.128	9.52E-19	Skin_Eryth	TMEM109
SPAG96	6.45E-23	0.65156	0.416	0.193	1.03E-18	Skin_Eryth	SPAG9
SFPQ9	7.73E-23	0.556238	0.547	0.285	1.23E-18	Skin_Eryth	SFPQ
GIT11	9.93E-23	0.384011	0.172	0.047	1.58E-18	Skin_Eryth	GIT1
TSPAN151	1.01E-22	0.366769	0.161	0.043	1.62E-18	Skin_Eryth	TSPAN15
CRK6	1.14E-22	0.514825	0.307	0.119	1.82E-18	Skin_Eryth	CRK
MPRIP.AS1	1.2E-22	0.265406	0.08	0.013	1.91E-18	Skin_Eryth	MPRIP.AS1
DUSP15	1.3E-22	0.57149	0.836	0.561	2.07E-18	Skin_Eryth	DUSP1
RPLP23	1.37E-22	0.289573	0.996	0.844	2.18E-18	Skin_Eryth	RPLP2
CEP85L1	1.46E-22	0.3953	0.248	0.083	2.33E-18	Skin_Eryth	CEP85L
RPL2412	1.6E-22	0.446385	0.985	0.802	2.55E-18	Skin_Eryth	RPL24
C1orf1982	1.78E-22	0.350841	0.197	0.059	2.83E-18	Skin_Eryth	C1orf198
BCAM5	2.11E-22	0.354885	0.234	0.076	3.37E-18	Skin_Eryth	BCAM
LMCD15	2.25E-22	0.349547	0.248	0.084	3.59E-18	Skin_Eryth	LMCD1
NCK15	2.26E-22	0.489183	0.288	0.108	3.61E-18	Skin_Eryth	NCK1
HTRA15	2.35E-22	0.338255	0.303	0.112	3.74E-18	Skin_Eryth	HTRA1
FRMD4B8	2.69E-22	0.556535	0.314	0.127	4.28E-18	Skin_Eryth	FRMD4B
RWDD12	2.87E-22	0.622331	0.558	0.308	4.57E-18	Skin_Eryth	RWDD1
NFIC9	3.03E-22	0.43088	0.391	0.171	4.83E-18	Skin_Eryth	NFIC
FKBP94	3.2E-22	0.333202	0.237	0.078	5.11E-18	Skin_Eryth	FKBP9
UBE2S8	3.49E-22	0.611236	0.325	0.131	5.57E-18	Skin_Eryth	UBE2S
ARHGEF12	3.62E-22	0.561356	0.332	0.137	5.78E-18	Skin_Eryth	ARHGEF12
CCDC85B5	4.22E-22	0.455176	0.54	0.272	6.73E-18	Skin_Eryth	CCDC85B
TOB26	4.92E-22	0.48212	0.274	0.1	7.84E-18	Skin_Eryth	TOB2
RBX18	4.97E-22	0.648126	0.609	0.349	7.92E-18	Skin_Eryth	RBX1
HNRNPAB8	5.44E-22	0.505476	0.38	0.166	8.67E-18	Skin_Eryth	HNRNPAB
ATP5I4	6.04E-22	0.686248	0.628	0.372	9.63E-18	Skin_Eryth	ATP5I
SRI4	6.7E-22	0.59987	0.478	0.242	1.07E-17	Skin_Eryth	SRI
TCEAL14	6.86E-22	0.334544	0.186	0.054	1.09E-17	Skin_Eryth	TCEAL1

ZFAND14	6.9E-22	0.563955	0.292	0.113	1.1E-17	Skin_Eryth	ZFAND1
SUMO27	7.23E-22	0.529363	0.843	0.601	1.15E-17	Skin_Eryth	SUMO2
ASXL14	9.02E-22	0.447293	0.234	0.079	1.44E-17	Skin_Eryth	ASXL1
SVIL8	1.11E-21	0.495071	0.303	0.12	1.77E-17	Skin_Eryth	SVIL
ROMO12	1.19E-21	0.692566	0.551	0.31	1.91E-17	Skin_Eryth	ROMO1
UCP22	1.23E-21	0.328904	0.376	0.154	1.96E-17	Skin_Eryth	UCP2
STARD74	1.47E-21	0.433629	0.259	0.094	2.34E-17	Skin_Eryth	STARD7
EEF25	1.49E-21	0.470219	0.891	0.607	2.38E-17	Skin_Eryth	EEF2
DDX3X8	1.55E-21	0.656414	0.54	0.301	2.47E-17	Skin_Eryth	DDX3X
HSPA23	1.59E-21	0.354867	0.215	0.069	2.53E-17	Skin_Eryth	HSPA2
MLXIP2	2.01E-21	0.447035	0.27	0.101	3.21E-17	Skin_Eryth	MLXIP
C17orf582	2.15E-21	0.324996	0.15	0.039	3.43E-17	Skin_Eryth	C17orf58
CBX67	2.44E-21	0.403935	0.245	0.084	3.9E-17	Skin_Eryth	CBX6
NTN11	2.52E-21	0.302791	0.102	0.02	4.03E-17	Skin_Eryth	NTN1
SEC11A7	3.46E-21	0.615906	0.555	0.315	5.51E-17	Skin_Eryth	SEC11A
RAB5C2	3.73E-21	0.56334	0.485	0.256	5.95E-17	Skin_Eryth	RAB5C
ATP1B36	4.08E-21	0.459355	0.485	0.236	6.51E-17	Skin_Eryth	ATP1B3
DDX216	4.27E-21	0.584195	0.423	0.199	6.81E-17	Skin_Eryth	DDX21
RPL10A6	4.64E-21	0.343069	0.989	0.734	7.4E-17	Skin_Eryth	RPL10A
MICAL32	5.12E-21	0.299104	0.124	0.029	8.16E-17	Skin_Eryth	MICAL3
FAU8	5.16E-21	0.366355	0.989	0.855	8.23E-17	Skin_Eryth	FAU
THUMPD1.	6.09E-21	0.494328	0.27	0.102	9.71E-17	Skin_Eryth	THUMPD1
REXO21	8.3E-21	0.631583	0.482	0.258	1.32E-16	Skin_Eryth	REXO2
SRP146	8.68E-21	0.544083	0.905	0.701	1.38E-16	Skin_Eryth	SRP14
HIF1A7	8.89E-21	0.65076	0.365	0.166	1.42E-16	Skin_Eryth	HIF1A
EIF1B4	9.12E-21	0.414133	0.496	0.25	1.45E-16	Skin_Eryth	EIF1B
FAM107B5	1.35E-20	0.528243	0.551	0.298	2.15E-16	Skin_Eryth	FAM107B
PIEZO1	1.7E-20	0.252566	0.215	0.069	2.72E-16	Skin_Eryth	PIEZO1
SEP152	1.72E-20	0.476883	0.558	0.305	2.74E-16	Skin_Eryth	SEP15
TNFRSF10I	1.72E-20	0.431305	0.157	0.043	2.75E-16	Skin_Eryth	TNFRSF10B
RNASEK3	1.73E-20	0.252758	0.135	0.033	2.77E-16	Skin_Eryth	RNASEK
PRKAR1A4	2.25E-20	0.633489	0.456	0.238	3.59E-16	Skin_Eryth	PRKAR1A
JUN8	2.28E-20	0.509856	0.781	0.487	3.64E-16	Skin_Eryth	JUN
AMD16	2.48E-20	0.619422	0.405	0.194	3.96E-16	Skin_Eryth	AMD1
CLIC18	3.79E-20	0.469621	0.77	0.475	6.05E-16	Skin_Eryth	CLIC1
ENY26	3.91E-20	0.599782	0.547	0.321	6.24E-16	Skin_Eryth	ENY2
ABCD42	3.98E-20	0.381645	0.201	0.065	6.35E-16	Skin_Eryth	ABCD4
NUMB1	4.58E-20	0.504523	0.292	0.119	7.3E-16	Skin_Eryth	NUMB
TBL1XR14	4.86E-20	0.544559	0.343	0.151	7.75E-16	Skin_Eryth	TBL1XR1
SNRPE5	5.12E-20	0.510238	0.507	0.266	8.16E-16	Skin_Eryth	SNRPE
CCDC504	5.15E-20	0.586018	0.321	0.137	8.21E-16	Skin_Eryth	CCDC50
KLF96	5.52E-20	0.380694	0.277	0.106	8.8E-16	Skin_Eryth	KLF9
NHSL12	5.56E-20	0.298111	0.146	0.039	8.86E-16	Skin_Eryth	NHSL1
SLC35G21	6.07E-20	0.281453	0.113	0.026	9.67E-16	Skin_Eryth	SLC35G2
MAP3K112	6.12E-20	0.411406	0.248	0.091	9.76E-16	Skin_Eryth	MAP3K11
STRN34	6.33E-20	0.588455	0.248	0.092	1.01E-15	Skin_Eryth	STRN3
RPL378	6.8E-20	0.415258	0.971	0.848	1.08E-15	Skin_Eryth	RPL37

SON9	7.71E-20	0.649337	0.657	0.41	1.23E-15	Skin_Eryth	SON
LEPROT6	7.84E-20	0.515768	0.485	0.257	1.25E-15	Skin_Eryth	LEPROT
ITPKB3	8.56E-20	0.336753	0.146	0.039	1.37E-15	Skin_Eryth	ITPKB
TIFA2	9.09E-20	0.363722	0.19	0.06	1.45E-15	Skin_Eryth	TIFA
HNRNPM2	9.83E-20	0.401281	0.449	0.216	1.57E-15	Skin_Eryth	HNRNPM
SEC627	1.04E-19	0.539885	0.664	0.401	1.66E-15	Skin_Eryth	SEC62
PRDX19	1.04E-19	0.546008	0.715	0.452	1.66E-15	Skin_Eryth	PRDX1
ARMCX14	1.14E-19	0.379092	0.182	0.056	1.81E-15	Skin_Eryth	ARMCX1
BRI39	1.25E-19	0.545867	0.588	0.346	1.99E-15	Skin_Eryth	BRI3
NCL10	1.5E-19	0.498584	0.515	0.27	2.39E-15	Skin_Eryth	NCL
CTNND17	1.53E-19	0.432657	0.259	0.099	2.44E-15	Skin_Eryth	CTNND1
TC2N4	1.57E-19	0.556439	0.339	0.154	2.5E-15	Skin_Eryth	TC2N
AP2M12	1.7E-19	0.48555	0.544	0.304	2.71E-15	Skin_Eryth	AP2M1
BID2	1.94E-19	0.33149	0.303	0.121	3.1E-15	Skin_Eryth	BID
DSEL2	1.96E-19	0.394324	0.135	0.035	3.12E-15	Skin_Eryth	DSEL
SMAD57	2.1E-19	0.498334	0.248	0.094	3.35E-15	Skin_Eryth	SMAD5
LINC00152	2.47E-19	0.357366	0.401	0.178	3.95E-15	Skin_Eryth	LINC00152
TCF7L15	2.8E-19	0.254774	0.124	0.03	4.47E-15	Skin_Eryth	TCF7L1
FUS12	3.09E-19	0.605288	0.511	0.277	4.94E-15	Skin_Eryth	FUS
CSRP25	3.1E-19	0.294	0.23	0.081	4.94E-15	Skin_Eryth	CSRP2
TMEM173	3.11E-19	0.339112	0.288	0.11	4.96E-15	Skin_Eryth	TMEM173
TMEM704	3.35E-19	0.590148	0.277	0.113	5.34E-15	Skin_Eryth	TMEM70
SH3BGRL8	3.36E-19	0.396705	0.493	0.244	5.36E-15	Skin_Eryth	SH3BGRL
SEPT116	3.4E-19	0.367211	0.241	0.086	5.43E-15	Skin_Eryth	SEPT11
PABPN18	3.82E-19	0.530618	0.336	0.151	6.09E-15	Skin_Eryth	PABPN1
PFDN12	4.39E-19	0.500049	0.285	0.116	6.99E-15	Skin_Eryth	PFDN1
PLCE11	4.63E-19	0.265479	0.102	0.022	7.38E-15	Skin_Eryth	PLCE1
ETS25	4.66E-19	0.43009	0.358	0.157	7.44E-15	Skin_Eryth	ETS2
ARRDC34	4.69E-19	0.71417	0.46	0.244	7.48E-15	Skin_Eryth	ARRDC3
PPP2R5E2	4.76E-19	0.48401	0.281	0.113	7.59E-15	Skin_Eryth	PPP2R5E
MYADM10	4.81E-19	0.550678	0.343	0.15	7.66E-15	Skin_Eryth	MYADM
HAPLN33	5.42E-19	0.255647	0.135	0.035	8.65E-15	Skin_Eryth	HAPLN3
RBMX5	6.37E-19	0.408905	0.416	0.198	1.02E-14	Skin_Eryth	RBMX
SRSF118	6.68E-19	0.530011	0.54	0.302	1.06E-14	Skin_Eryth	SRSF11
TCF7L23	6.81E-19	0.449659	0.237	0.088	1.09E-14	Skin_Eryth	TCF7L2
CKS25	7.92E-19	0.365903	0.336	0.143	1.26E-14	Skin_Eryth	CKS2
STARD31	8.07E-19	0.293448	0.19	0.062	1.29E-14	Skin_Eryth	STARD3
MAGI13	8.26E-19	0.432766	0.201	0.068	1.32E-14	Skin_Eryth	MAGI1
RNF19B	8.52E-19	0.542502	0.197	0.067	1.36E-14	Skin_Eryth	RNF19B
TIPIN6	8.56E-19	0.485326	0.212	0.074	1.37E-14	Skin_Eryth	TIPIN
SLC44A14	8.65E-19	0.327041	0.226	0.08	1.38E-14	Skin_Eryth	SLC44A1
CNIH14	1.07E-18	0.339413	0.31	0.129	1.7E-14	Skin_Eryth	CNIH1
LRRFIP16	1.09E-18	0.502898	0.624	0.373	1.74E-14	Skin_Eryth	LRRFIP1
GABARAPL	1.19E-18	0.623156	0.35	0.167	1.9E-14	Skin_Eryth	GABARAPL1
MRPL523	1.42E-18	0.546862	0.434	0.224	2.27E-14	Skin_Eryth	MRPL52
DUT3	1.93E-18	0.434632	0.456	0.236	3.08E-14	Skin_Eryth	DUT
PYURF4	1.93E-18	0.532654	0.464	0.247	3.08E-14	Skin_Eryth	PYURF

GNA134	1.98E-18	0.39952	0.234	0.085	3.16E-14	Skin_Eryth	GNA13
DCTPP12	2.17E-18	0.386789	0.266	0.106	3.46E-14	Skin_Eryth	DCTPP1
ARL6IP63	2.21E-18	0.385343	0.208	0.072	3.52E-14	Skin_Eryth	ARL6IP6
MRAS1	2.25E-18	0.328953	0.128	0.034	3.59E-14	Skin_Eryth	MRAS
PLXDC26	2.27E-18	0.261706	0.153	0.044	3.62E-14	Skin_Eryth	PLXDC2
HOXA7	2.56E-18	0.281835	0.095	0.02	4.08E-14	Skin_Eryth	HOXA7
CTSH5	2.59E-18	0.53795	0.449	0.238	4.14E-14	Skin_Eryth	CTSH
GCNT2	2.62E-18	0.288887	0.117	0.029	4.18E-14	Skin_Eryth	GCNT2
PLIN24	2.93E-18	0.605198	0.296	0.126	4.68E-14	Skin_Eryth	PLIN2
CHMP33	3E-18	0.46843	0.361	0.168	4.78E-14	Skin_Eryth	CHMP3
ATF47	3.43E-18	0.515176	0.569	0.337	5.46E-14	Skin_Eryth	ATF4
COX7C5	3.79E-18	0.488023	0.869	0.637	6.05E-14	Skin_Eryth	COX7C
NXT15	3.97E-18	0.4626	0.339	0.153	6.33E-14	Skin_Eryth	NXT1
NPM14	4.39E-18	0.364144	0.81	0.533	7E-14	Skin_Eryth	NPM1
TBCB3	5.16E-18	0.320771	0.427	0.202	8.23E-14	Skin_Eryth	TBCB
PSMA44	5.82E-18	0.484227	0.478	0.256	9.27E-14	Skin_Eryth	PSMA4
RNF1685	5.95E-18	0.432639	0.252	0.097	9.5E-14	Skin_Eryth	RNF168
CTTN4	6.42E-18	0.72575	0.401	0.207	1.02E-13	Skin_Eryth	CTTN
SPOP4	6.63E-18	0.400335	0.277	0.114	1.06E-13	Skin_Eryth	SPOP
NHP21	7.6E-18	0.434304	0.449	0.231	1.21E-13	Skin_Eryth	NHP2
DENND5A4	8E-18	0.277099	0.146	0.042	1.28E-13	Skin_Eryth	DENND5A
RP11.108M	8.49E-18	0.27121	0.109	0.026	1.35E-13	Skin_Eryth	RP11.108M9.4
LDHA7	8.5E-18	0.354854	0.715	0.44	1.36E-13	Skin_Eryth	LDHA
EDN13	9.07E-18	0.398169	0.139	0.039	1.45E-13	Skin_Eryth	EDN1
RNF55	1E-17	0.402141	0.321	0.143	1.6E-13	Skin_Eryth	RNF5
PHF143	1.04E-17	0.375625	0.296	0.125	1.66E-13	Skin_Eryth	PHF14
SEPHS22	1.09E-17	0.259687	0.193	0.064	1.74E-13	Skin_Eryth	SEPHS2
JAK12	1.13E-17	0.508468	0.464	0.249	1.8E-13	Skin_Eryth	JAK1
LSM73	1.24E-17	0.359594	0.529	0.285	1.97E-13	Skin_Eryth	LSM7
RNPS13	1.31E-17	0.435162	0.405	0.198	2.1E-13	Skin_Eryth	RNPS1
PNRC16	1.4E-17	0.363354	0.766	0.511	2.23E-13	Skin_Eryth	PNRC1
DNAJA16	1.46E-17	0.526131	0.602	0.361	2.33E-13	Skin_Eryth	DNAJA1
AK32	1.79E-17	0.389536	0.343	0.158	2.85E-13	Skin_Eryth	AK3
SLC25A56	1.84E-17	0.496777	0.617	0.369	2.93E-13	Skin_Eryth	SLC25A5
SOD17	1.9E-17	0.381527	0.704	0.415	3.03E-13	Skin_Eryth	SOD1
BCAP291	1.91E-17	0.430483	0.336	0.154	3.05E-13	Skin_Eryth	BCAP29
SRGAP32	1.98E-17	0.412902	0.128	0.035	3.16E-13	Skin_Eryth	SRGAP3
HSPA87	2.27E-17	0.561002	0.737	0.51	3.61E-13	Skin_Eryth	HSPA8
GADD45GI	2.49E-17	0.495645	0.504	0.28	3.97E-13	Skin_Eryth	GADD45GIP1
GNB2L11	2.7E-17	0.257085	0.956	0.753	4.31E-13	Skin_Eryth	GNB2L1
BTBD74	2.78E-17	0.487989	0.245	0.098	4.43E-13	Skin_Eryth	BTBD7
FAM127A4	3.01E-17	0.35684	0.237	0.091	4.8E-13	Skin_Eryth	FAM127A
ANXA510	3.05E-17	0.370444	0.58	0.317	4.87E-13	Skin_Eryth	ANXA5
PTGES37	3.14E-17	0.465987	0.639	0.393	5.01E-13	Skin_Eryth	PTGES3
RAN8	3.14E-17	0.482474	0.613	0.367	5.02E-13	Skin_Eryth	RAN
ZFHX36	3.35E-17	0.337801	0.234	0.09	5.35E-13	Skin_Eryth	ZFHX3
RBM398	3.58E-17	0.507159	0.639	0.411	5.7E-13	Skin_Eryth	RBM39

TMEM245:	3.7E-17	0.412574	0.197	0.07	5.91E-13	Skin_Eryth	TMEM245
HPCAL12	4.5E-17	0.319226	0.215	0.078	7.18E-13	Skin_Eryth	HPCAL1
SSBP24	4.62E-17	0.424304	0.201	0.071	7.37E-13	Skin_Eryth	SSBP2
PIM35	4.85E-17	0.506437	0.504	0.291	7.74E-13	Skin_Eryth	PIM3
LSM58	5.12E-17	0.509699	0.394	0.201	8.16E-13	Skin_Eryth	LSM5
RAB6A2	5.36E-17	0.482202	0.35	0.17	8.55E-13	Skin_Eryth	RAB6A
ITGB46	5.82E-17	0.390605	0.226	0.087	9.29E-13	Skin_Eryth	ITGB4
DYNC1H16	5.98E-17	0.428695	0.31	0.139	9.54E-13	Skin_Eryth	DYNC1H1
ATN12	6.18E-17	0.345522	0.161	0.05	9.86E-13	Skin_Eryth	ATN1
EIF1AX6	6.44E-17	0.459895	0.566	0.337	1.03E-12	Skin_Eryth	EIF1AX
BTF36	6.63E-17	0.377095	0.901	0.711	1.06E-12	Skin_Eryth	BTF3
SLC18B11	6.9E-17	0.323695	0.109	0.027	1.1E-12	Skin_Eryth	SLC18B1
TANC12	6.95E-17	0.360496	0.175	0.058	1.11E-12	Skin_Eryth	TANC1
HNRNPA3{	7.68E-17	0.505096	0.588	0.365	1.23E-12	Skin_Eryth	HNRNPA3
PPTC75	9.31E-17	0.344202	0.186	0.063	1.49E-12	Skin_Eryth	PPTC7
HP1BP34	9.39E-17	0.56719	0.423	0.224	1.5E-12	Skin_Eryth	HP1BP3
HNRNPDL6	1.05E-16	0.278931	0.613	0.336	1.68E-12	Skin_Eryth	HNRNPDL
HNRNPK5	1.07E-16	0.553332	0.661	0.446	1.71E-12	Skin_Eryth	HNRNPK
NPHP33	1.11E-16	0.310646	0.15	0.045	1.76E-12	Skin_Eryth	NPHP3
LYN1	1.24E-16	0.346194	0.288	0.122	1.98E-12	Skin_Eryth	LYN
EIF5A9	1.29E-16	0.486496	0.493	0.278	2.06E-12	Skin_Eryth	EIF5A
ARL5A4	1.29E-16	0.470151	0.387	0.196	2.06E-12	Skin_Eryth	ARL5A
C10orf112	1.3E-16	0.258718	0.161	0.051	2.07E-12	Skin_Eryth	C10orf11
GAS69	1.3E-16	0.282549	0.245	0.095	2.07E-12	Skin_Eryth	GAS6
NUDT36	1.34E-16	0.293094	0.255	0.103	2.13E-12	Skin_Eryth	NUDT3
HNRNPH24	1.34E-16	0.297426	0.219	0.081	2.14E-12	Skin_Eryth	HNRNPH2
LY6E3	1.35E-16	0.327107	0.602	0.331	2.15E-12	Skin_Eryth	LY6E
RPS264	1.36E-16	0.326882	0.942	0.742	2.16E-12	Skin_Eryth	RPS26
MPP12	1.78E-16	0.282415	0.142	0.042	2.85E-12	Skin_Eryth	MPP1
RHOA5	1.88E-16	0.458037	0.712	0.474	3.01E-12	Skin_Eryth	RHOA
LYRM42	1.97E-16	0.328691	0.27	0.113	3.13E-12	Skin_Eryth	LYRM4
NDUFA14	2.02E-16	0.432499	0.661	0.412	3.22E-12	Skin_Eryth	NDUFA1
MAP4K53	2.02E-16	0.321856	0.226	0.087	3.22E-12	Skin_Eryth	MAP4K5
BNIP26	2.04E-16	0.376465	0.288	0.124	3.25E-12	Skin_Eryth	BNIP2
UBE2E13	2.13E-16	0.460183	0.303	0.138	3.4E-12	Skin_Eryth	UBE2E1
SEC61G8	2.16E-16	0.429223	0.664	0.415	3.45E-12	Skin_Eryth	SEC61G
ITM2B7	2.27E-16	0.440011	0.92	0.706	3.61E-12	Skin_Eryth	ITM2B
PEA152	2.5E-16	0.447315	0.358	0.173	3.99E-12	Skin_Eryth	PEA15
CCDC1151	2.5E-16	0.327036	0.277	0.117	3.99E-12	Skin_Eryth	CCDC115
STRAP4	2.53E-16	0.471017	0.412	0.217	4.04E-12	Skin_Eryth	STRAP
APLP26	2.55E-16	0.520007	0.518	0.304	4.07E-12	Skin_Eryth	APLP2
PDE4B3	2.6E-16	0.279296	0.161	0.051	4.14E-12	Skin_Eryth	PDE4B
YAF23	2.67E-16	0.487941	0.241	0.097	4.26E-12	Skin_Eryth	YAF2
SF17	2.69E-16	0.414244	0.438	0.234	4.3E-12	Skin_Eryth	SF1
RAB8B4	2.81E-16	0.385712	0.245	0.097	4.48E-12	Skin_Eryth	RAB8B
MAML11	2.87E-16	0.311358	0.146	0.044	4.58E-12	Skin_Eryth	MAML1
VPS13C6	2.9E-16	0.276401	0.237	0.093	4.62E-12	Skin_Eryth	VPS13C

TMEM230:	2.99E-16	0.429351	0.482	0.267	4.77E-12	Skin_Eryth	TMEM230
INSIG21	3.36E-16	0.422703	0.19	0.068	5.36E-12	Skin_Eryth	INSIG2
LINC00969	3.77E-16	0.463046	0.263	0.111	6.01E-12	Skin_Eryth	LINC00969
KIAA03552	3.86E-16	0.387668	0.215	0.082	6.15E-12	Skin_Eryth	KIAA0355
FAM65A2	3.89E-16	0.307958	0.153	0.048	6.2E-12	Skin_Eryth	FAM65A
CRBN3	3.95E-16	0.32607	0.318	0.143	6.29E-12	Skin_Eryth	CRBN
AKIP1	4.01E-16	0.350733	0.168	0.056	6.4E-12	Skin_Eryth	AKIP1
CELF15	4.13E-16	0.469447	0.281	0.125	6.58E-12	Skin_Eryth	CELF1
DOCK91	4.33E-16	0.360626	0.245	0.098	6.9E-12	Skin_Eryth	DOCK9
HMGN25	4.38E-16	0.492124	0.551	0.328	6.98E-12	Skin_Eryth	HMGN2
NUTF22	4.8E-16	0.503911	0.292	0.132	7.66E-12	Skin_Eryth	NUTF2
FLNB6	4.81E-16	0.34332	0.208	0.079	7.67E-12	Skin_Eryth	FLNB
TNFAIP24	4.81E-16	0.410059	0.318	0.14	7.67E-12	Skin_Eryth	TNFAIP2
ITGB18	4.96E-16	0.475875	0.595	0.357	7.91E-12	Skin_Eryth	ITGB1
SPRED14	5.02E-16	0.255963	0.142	0.043	8.01E-12	Skin_Eryth	SPRED1
KHDRBS32	5.69E-16	0.308127	0.201	0.073	9.07E-12	Skin_Eryth	KHDRBS3
MGST313	6.21E-16	0.339775	0.606	0.335	9.91E-12	Skin_Eryth	MGST3
CYP1B12	6.83E-16	0.28878	0.161	0.051	1.09E-11	Skin_Eryth	CYP1B1
SBF22	6.87E-16	0.426692	0.182	0.065	1.1E-11	Skin_Eryth	SBF2
FERMT27	6.99E-16	0.324114	0.215	0.082	1.11E-11	Skin_Eryth	FERMT2
SORBS33	7.52E-16	0.313667	0.255	0.105	1.2E-11	Skin_Eryth	SORBS3
EIF59	8.79E-16	0.513167	0.577	0.36	1.4E-11	Skin_Eryth	EIF5
RBMS12	9.85E-16	0.415751	0.416	0.214	1.57E-11	Skin_Eryth	RBMS1
POLR2F3	1.12E-15	0.444997	0.453	0.252	1.79E-11	Skin_Eryth	POLR2F
BHLHE405	1.37E-15	0.347935	0.383	0.187	2.18E-11	Skin_Eryth	BHLHE40
SAR1A6	1.43E-15	0.599348	0.42	0.232	2.29E-11	Skin_Eryth	SAR1A
LUZP14	1.64E-15	0.536118	0.277	0.124	2.61E-11	Skin_Eryth	LUZP1
RTKN	1.72E-15	0.3073	0.099	0.025	2.74E-11	Skin_Eryth	RTKN
IRF2BPL7	1.86E-15	0.267418	0.204	0.076	2.97E-11	Skin_Eryth	IRF2BPL
TPM36	1.88E-15	0.441265	0.551	0.332	2.99E-11	Skin_Eryth	TPM3
C6orf482	1.92E-15	0.315174	0.566	0.312	3.06E-11	Skin_Eryth	C6orf48
HBEGF3	2.22E-15	0.324716	0.153	0.049	3.55E-11	Skin_Eryth	HBEGF
PQLC35	2.27E-15	0.327945	0.23	0.092	3.63E-11	Skin_Eryth	PQLC3
TMEM50B:	2.34E-15	0.42419	0.321	0.153	3.74E-11	Skin_Eryth	TMEM50B
PPP1R15A4	2.49E-15	0.354231	0.723	0.449	3.98E-11	Skin_Eryth	PPP1R15A
POLR2M	2.61E-15	0.270102	0.15	0.048	4.16E-11	Skin_Eryth	POLR2M
ELOVL54	2.65E-15	0.369978	0.255	0.108	4.22E-11	Skin_Eryth	ELOVL5
DDAH26	2.87E-15	0.438052	0.401	0.21	4.57E-11	Skin_Eryth	DDAH2
PMVK3	3.08E-15	0.352242	0.292	0.132	4.92E-11	Skin_Eryth	PMVK
TMEM256:	3.28E-15	0.429455	0.409	0.22	5.24E-11	Skin_Eryth	TMEM256
MED242	3.46E-15	0.654454	0.135	0.041	5.52E-11	Skin_Eryth	MED24
ZNF4624	3.63E-15	0.438351	0.157	0.054	5.79E-11	Skin_Eryth	ZNF462
HADHA3	3.64E-15	0.384709	0.387	0.2	5.8E-11	Skin_Eryth	HADHA
CHD71	4.24E-15	0.318552	0.146	0.047	6.77E-11	Skin_Eryth	CHD7
TROVE22	4.32E-15	0.385664	0.296	0.134	6.89E-11	Skin_Eryth	TROVE2
ST6GALNA	4.56E-15	0.275993	0.193	0.071	7.27E-11	Skin_Eryth	ST6GALNAC6
PEF11	4.64E-15	0.342822	0.234	0.095	7.41E-11	Skin_Eryth	PEF1

PCMTD2	4.69E-15	0.325882	0.179	0.064	7.48E-11	Skin_Eryth	PCMTD2
TMEM14C	4.74E-15	0.502551	0.449	0.255	7.56E-11	Skin_Eryth	TMEM14C
SMIM105	5.24E-15	0.277977	0.088	0.02	8.36E-11	Skin_Eryth	SMIM10
MKNK26	5.8E-15	0.441855	0.263	0.117	9.25E-11	Skin_Eryth	MKNK2
CREB11	5.83E-15	0.373663	0.281	0.126	9.3E-11	Skin_Eryth	CREB1
FEZ24	5.9E-15	0.435267	0.255	0.11	9.41E-11	Skin_Eryth	FEZ2
LACTB3	5.93E-15	0.420703	0.215	0.085	9.45E-11	Skin_Eryth	LACTB
YWHAQ5	5.98E-15	0.467026	0.588	0.365	9.54E-11	Skin_Eryth	YWHAQ
C19orf604	6.35E-15	0.392281	0.427	0.229	1.01E-10	Skin_Eryth	C19orf60
SLIRP4	6.43E-15	0.592641	0.449	0.258	1.03E-10	Skin_Eryth	SLIRP
CTD.2336C	6.52E-15	0.3128	0.168	0.059	1.04E-10	Skin_Eryth	CTD.2336O2.1
MRPL233	6.8E-15	0.335583	0.405	0.211	1.09E-10	Skin_Eryth	MRPL23
UBA23	6.87E-15	0.285288	0.27	0.116	1.1E-10	Skin_Eryth	UBA2
SERPINH1!	7.81E-15	0.278537	0.27	0.115	1.25E-10	Skin_Eryth	SERPINH1
CCDC82	8.12E-15	0.43864	0.219	0.087	1.29E-10	Skin_Eryth	CCDC82
BRK11	9E-15	0.39515	0.664	0.455	1.44E-10	Skin_Eryth	BRK1
LAMTOR42	9.24E-15	0.33753	0.617	0.389	1.47E-10	Skin_Eryth	LAMTOR4
MBNL27	1.05E-14	0.407501	0.266	0.119	1.67E-10	Skin_Eryth	MBNL2
PA2G42	1.12E-14	0.381215	0.453	0.244	1.79E-10	Skin_Eryth	PA2G4
CDC375	1.14E-14	0.453732	0.474	0.274	1.81E-10	Skin_Eryth	CDC37
CYLD5	1.15E-14	0.548249	0.274	0.125	1.83E-10	Skin_Eryth	CYLD
PGRMC16	1.17E-14	0.418953	0.336	0.166	1.87E-10	Skin_Eryth	PGRMC1
PARVA3	1.31E-14	0.388654	0.168	0.06	2.08E-10	Skin_Eryth	PARVA
EIF3K1	1.36E-14	0.265145	0.752	0.518	2.18E-10	Skin_Eryth	EIF3K
HOMER33	1.41E-14	0.306394	0.164	0.057	2.24E-10	Skin_Eryth	HOMER3
CREBRF2	1.45E-14	0.525487	0.296	0.14	2.32E-10	Skin_Eryth	CREBRF
FAM174B2	1.62E-14	0.373216	0.168	0.06	2.58E-10	Skin_Eryth	FAM174B
UBB3	1.64E-14	0.282849	0.912	0.73	2.62E-10	Skin_Eryth	UBB
MIR22HG5	1.73E-14	0.361322	0.215	0.085	2.75E-10	Skin_Eryth	MIR22HG
DAAM14	1.75E-14	0.480907	0.336	0.169	2.79E-10	Skin_Eryth	DAAM1
WBP44	2.55E-14	0.421737	0.23	0.097	4.06E-10	Skin_Eryth	WBP4
SNRPD12	2.57E-14	0.428513	0.412	0.221	4.1E-10	Skin_Eryth	SNRPD1
RPS6KA38	2.81E-14	0.386341	0.23	0.095	4.49E-10	Skin_Eryth	RPS6KA3
ZBTB205	2.91E-14	0.384509	0.288	0.134	4.63E-10	Skin_Eryth	ZBTB20
TTC195	2.91E-14	0.410377	0.266	0.122	4.65E-10	Skin_Eryth	TTC19
NR3C17	3.27E-14	0.354018	0.314	0.149	5.22E-10	Skin_Eryth	NR3C1
PFDN26	3.31E-14	0.414308	0.485	0.275	5.27E-10	Skin_Eryth	PFDN2
RPS2410	3.36E-14	0.311216	0.996	0.839	5.37E-10	Skin_Eryth	RPS24
BRD31	3.44E-14	0.328489	0.186	0.071	5.49E-10	Skin_Eryth	BRD3
GNB13	3.64E-14	0.449466	0.391	0.214	5.81E-10	Skin_Eryth	GNB1
HEBP1	3.66E-14	0.380788	0.361	0.189	5.83E-10	Skin_Eryth	HEBP1
RBM66	4.26E-14	0.401499	0.252	0.111	6.79E-10	Skin_Eryth	RBM6
ARGLU16	6.01E-14	0.417469	0.515	0.309	9.58E-10	Skin_Eryth	ARGLU1
LYRM5	6.74E-14	0.461033	0.285	0.136	1.07E-09	Skin_Eryth	LYRM5
PRPF6	7.05E-14	0.381213	0.27	0.123	1.13E-09	Skin_Eryth	PRPF6
CXXC53	7.45E-14	0.308368	0.318	0.153	1.19E-09	Skin_Eryth	CXXC5
WDR83OS:	7.52E-14	0.387169	0.569	0.368	1.2E-09	Skin_Eryth	WDR83OS

RPS126	7.72E-14	0.26877	0.993	0.861	1.23E-09	Skin_Eryth	RPS12
KTN14	7.86E-14	0.424918	0.606	0.397	1.25E-09	Skin_Eryth	KTN1
PLEKHA32	7.97E-14	0.424193	0.27	0.125	1.27E-09	Skin_Eryth	PLEKHA3
GLRX52	9E-14	0.343654	0.336	0.171	1.44E-09	Skin_Eryth	GLRX5
STX62	9.57E-14	0.25607	0.124	0.038	1.53E-09	Skin_Eryth	STX6
TSPAN141	9.67E-14	0.307592	0.27	0.123	1.54E-09	Skin_Eryth	TSPAN14
NDUFB25	1.08E-13	0.331484	0.617	0.375	1.72E-09	Skin_Eryth	NDUFB2
TMA73	1.11E-13	0.389321	0.872	0.66	1.77E-09	Skin_Eryth	TMA7
YY16	1.11E-13	0.477011	0.401	0.227	1.78E-09	Skin_Eryth	YY1
EIF3F	1.11E-13	0.31887	0.657	0.44	1.78E-09	Skin_Eryth	EIF3F
COX173	1.14E-13	0.591517	0.46	0.276	1.82E-09	Skin_Eryth	COX17
TBC1D41	1.15E-13	0.330907	0.15	0.052	1.84E-09	Skin_Eryth	TBC1D4
FADS34	1.21E-13	0.295255	0.131	0.041	1.93E-09	Skin_Eryth	FADS3
NDUFA41C	1.24E-13	0.296007	0.818	0.538	1.97E-09	Skin_Eryth	NDUFA4
TIMM132	1.3E-13	0.461337	0.427	0.247	2.07E-09	Skin_Eryth	TIMM13
ASH1L7	1.31E-13	0.341984	0.336	0.17	2.08E-09	Skin_Eryth	ASH1L
GPBP16	1.33E-13	0.418048	0.529	0.321	2.12E-09	Skin_Eryth	GPBP1
CAMTA14	1.42E-13	0.485758	0.526	0.334	2.27E-09	Skin_Eryth	CAMTA1
ANAPC163	1.51E-13	0.396476	0.58	0.371	2.41E-09	Skin_Eryth	ANAPC16
HMBOX1	1.61E-13	0.300731	0.164	0.06	2.56E-09	Skin_Eryth	HMBOX1
TMX14	1.73E-13	0.305372	0.241	0.105	2.76E-09	Skin_Eryth	TMX1
COX143	1.76E-13	0.32712	0.42	0.232	2.81E-09	Skin_Eryth	COX14
FDPS3	1.81E-13	0.479958	0.358	0.192	2.89E-09	Skin_Eryth	FDPS
PARK710	1.96E-13	0.334837	0.599	0.355	3.12E-09	Skin_Eryth	PARK7
MAP3K86	2.28E-13	0.355355	0.263	0.116	3.64E-09	Skin_Eryth	MAP3K8
LINC00657	2.56E-13	0.395766	0.376	0.203	4.09E-09	Skin_Eryth	LINC00657
RYBP3	2.67E-13	0.391266	0.296	0.146	4.26E-09	Skin_Eryth	RYBP
TCEAL82	2.69E-13	0.466811	0.325	0.167	4.29E-09	Skin_Eryth	TCEAL8
VEZT2	2.83E-13	0.422792	0.197	0.081	4.52E-09	Skin_Eryth	VEZT
KMT2A9	2.97E-13	0.391794	0.277	0.13	4.73E-09	Skin_Eryth	KMT2A
ENSA2	3.08E-13	0.310777	0.427	0.235	4.91E-09	Skin_Eryth	ENSA
FAM3C3	3.3E-13	0.468907	0.365	0.197	5.26E-09	Skin_Eryth	FAM3C
C16orf871	3.31E-13	0.356086	0.19	0.075	5.27E-09	Skin_Eryth	C16orf87
ATP5L3	3.43E-13	0.400687	0.832	0.66	5.48E-09	Skin_Eryth	ATP5L
MPST2	3.55E-13	0.458939	0.321	0.167	5.66E-09	Skin_Eryth	MPST
SLC16A12	3.58E-13	0.265484	0.135	0.044	5.71E-09	Skin_Eryth	SLC16A1
OAZ13	3.73E-13	0.301956	0.92	0.783	5.95E-09	Skin_Eryth	OAZ1
MZT2B4	3.81E-13	0.298573	0.701	0.461	6.08E-09	Skin_Eryth	MZT2B
GTF2I10	3.9E-13	0.272413	0.314	0.154	6.23E-09	Skin_Eryth	GTF2I
TFRC5	4.07E-13	0.257061	0.153	0.054	6.49E-09	Skin_Eryth	TFRC
USMG53	4.68E-13	0.434861	0.642	0.436	7.46E-09	Skin_Eryth	USMG5
LSM23	4.93E-13	0.302522	0.336	0.169	7.86E-09	Skin_Eryth	LSM2
NACA25	5.06E-13	0.35429	0.186	0.072	8.07E-09	Skin_Eryth	NACA2
VAPA6	5.17E-13	0.430448	0.609	0.4	8.24E-09	Skin_Eryth	VAPA
REV3L4	5.18E-13	0.284563	0.219	0.092	8.27E-09	Skin_Eryth	REV3L
FRMD63	5.33E-13	0.25905	0.212	0.086	8.51E-09	Skin_Eryth	FRMD6
CDKN1C2	5.54E-13	0.310628	0.325	0.161	8.84E-09	Skin_Eryth	CDKN1C

EIF5B2	5.84E-13	0.313067	0.478	0.277	9.31E-09	Skin_Eryth	EIF5B
NCKAP14	6.2E-13	0.391791	0.299	0.149	9.89E-09	Skin_Eryth	NCKAP1
POLR2I	6.37E-13	0.311322	0.453	0.264	1.02E-08	Skin_Eryth	POLR2I
AP3S13	6.97E-13	0.450838	0.478	0.292	1.11E-08	Skin_Eryth	AP3S1
SIK13	7.16E-13	0.251759	0.314	0.149	1.14E-08	Skin_Eryth	SIK1
SPG212	7.42E-13	0.35726	0.31	0.157	1.18E-08	Skin_Eryth	SPG21
HNRNPH34	7.78E-13	0.341611	0.409	0.221	1.24E-08	Skin_Eryth	HNRNPH3
DNAJC81	8.3E-13	0.325132	0.401	0.219	1.32E-08	Skin_Eryth	DNAJC8
FIS14	8.39E-13	0.375991	0.536	0.338	1.34E-08	Skin_Eryth	FIS1
SHFM12	8.41E-13	0.458066	0.642	0.431	1.34E-08	Skin_Eryth	SHFM1
NDUFS7	8.76E-13	0.403351	0.456	0.273	1.4E-08	Skin_Eryth	NDUFS7
COX203	9.76E-13	0.444458	0.321	0.166	1.56E-08	Skin_Eryth	COX20
ZMIZ13	9.93E-13	0.386799	0.193	0.079	1.58E-08	Skin_Eryth	ZMIZ1
SRSF29	1.06E-12	0.426031	0.533	0.332	1.69E-08	Skin_Eryth	SRSF2
TMEM205	1.29E-12	0.26949	0.38	0.205	2.05E-08	Skin_Eryth	TMEM205
VGLL46	1.32E-12	0.382059	0.277	0.136	2.1E-08	Skin_Eryth	VGLL4
EIF3L2	1.38E-12	0.25933	0.606	0.387	2.19E-08	Skin_Eryth	EIF3L
IL1R15	1.52E-12	0.261627	0.19	0.075	2.42E-08	Skin_Eryth	IL1R1
BRD26	1.61E-12	0.480878	0.442	0.271	2.57E-08	Skin_Eryth	BRD2
CYC13	1.71E-12	0.408753	0.401	0.231	2.73E-08	Skin_Eryth	CYC1
KDSR7	1.75E-12	0.314287	0.274	0.13	2.8E-08	Skin_Eryth	KDSR
EVA1C3	1.81E-12	0.302093	0.182	0.072	2.88E-08	Skin_Eryth	EVA1C
NKTR11	1.9E-12	0.355971	0.336	0.176	3.03E-08	Skin_Eryth	NKTR
MAP1LC3A	2.16E-12	0.462204	0.376	0.211	3.44E-08	Skin_Eryth	MAP1LC3A
CBY1	2.34E-12	0.294213	0.172	0.067	3.74E-08	Skin_Eryth	CBY1
INO80D2	2.58E-12	0.266376	0.164	0.062	4.12E-08	Skin_Eryth	INO80D
UNK	2.6E-12	0.304238	0.172	0.067	4.15E-08	Skin_Eryth	UNK
KRAS2	2.61E-12	0.448694	0.347	0.188	4.16E-08	Skin_Eryth	KRAS
FAM177A1	2.65E-12	0.415745	0.38	0.215	4.22E-08	Skin_Eryth	FAM177A1
ARL5B6	2.76E-12	0.349397	0.223	0.099	4.41E-08	Skin_Eryth	ARL5B
ABL23	2.89E-12	0.275012	0.212	0.09	4.61E-08	Skin_Eryth	ABL2
MED102	3.23E-12	0.315185	0.347	0.185	5.16E-08	Skin_Eryth	MED10
NR6A1	3.31E-12	0.27663	0.117	0.038	5.28E-08	Skin_Eryth	NR6A1
CCDC71L3	3.4E-12	0.428303	0.168	0.065	5.42E-08	Skin_Eryth	CCDC71L
EIF3D2	3.44E-12	0.333941	0.474	0.284	5.48E-08	Skin_Eryth	EIF3D
DEK9	3.69E-12	0.392738	0.442	0.259	5.89E-08	Skin_Eryth	DEK
THAP9.AS1	3.83E-12	0.27104	0.193	0.08	6.1E-08	Skin_Eryth	THAP9.AS1
WAC4	3.97E-12	0.49419	0.372	0.212	6.34E-08	Skin_Eryth	WAC
TRA2A5	4.01E-12	0.362298	0.318	0.164	6.4E-08	Skin_Eryth	TRA2A
NDUFC15	4.94E-12	0.450882	0.456	0.281	7.89E-08	Skin_Eryth	NDUFC1
HNRNPA2E	5.01E-12	0.339693	0.715	0.491	8E-08	Skin_Eryth	HNRNPA2B1
FAM133B7	5.25E-12	0.415122	0.358	0.198	8.37E-08	Skin_Eryth	FAM133B
RBM173	5.34E-12	0.298369	0.343	0.181	8.52E-08	Skin_Eryth	RBM17
PTP4A26	5.93E-12	0.369572	0.474	0.292	9.46E-08	Skin_Eryth	PTP4A2
CNIH42	6.08E-12	0.333956	0.277	0.137	9.69E-08	Skin_Eryth	CNIH4
ST132	6.58E-12	0.399804	0.602	0.406	1.05E-07	Skin_Eryth	ST13
IQCK1	6.64E-12	0.344317	0.135	0.048	1.06E-07	Skin_Eryth	IQCK

MACF110	6.83E-12	0.357306	0.318	0.168	1.09E-07	Skin_Eryth	MACF1
CALU5	6.9E-12	0.355645	0.328	0.175	1.1E-07	Skin_Eryth	CALU
DSTN7	7.11E-12	0.301242	0.803	0.563	1.13E-07	Skin_Eryth	DSTN
ZNF7063	7.32E-12	0.382337	0.54	0.345	1.17E-07	Skin_Eryth	ZNF706
SYNCRIP6	7.44E-12	0.37497	0.339	0.185	1.19E-07	Skin_Eryth	SYNCRIP
NEK73	7.62E-12	0.303565	0.19	0.079	1.22E-07	Skin_Eryth	NEK7
SQSTM11	7.7E-12	0.335313	0.686	0.48	1.23E-07	Skin_Eryth	SQSTM1
FLCN1	8.2E-12	0.275171	0.117	0.038	1.31E-07	Skin_Eryth	FLCN
EIF4B7	8.56E-12	0.334075	0.493	0.3	1.37E-07	Skin_Eryth	EIF4B
KCTD204	8.76E-12	0.375469	0.186	0.077	1.4E-07	Skin_Eryth	KCTD20
C12orf578	9.37E-12	0.26284	0.569	0.357	1.49E-07	Skin_Eryth	C12orf57
TMEM179I	9.62E-12	0.299339	0.361	0.198	1.53E-07	Skin_Eryth	TMEM179B
MRPS28	1.01E-11	0.263143	0.142	0.052	1.61E-07	Skin_Eryth	MRPS28
ST3GAL1	1.01E-11	0.265433	0.197	0.084	1.61E-07	Skin_Eryth	ST3GAL1
PQBP16	1.06E-11	0.311666	0.303	0.154	1.69E-07	Skin_Eryth	PQBP1
RAB324	1.11E-11	0.268996	0.208	0.089	1.77E-07	Skin_Eryth	RAB32
PKIG6	1.26E-11	0.261313	0.223	0.1	2.02E-07	Skin_Eryth	PKIG
MRPL421	1.32E-11	0.424705	0.263	0.13	2.1E-07	Skin_Eryth	MRPL42
CYB5A8	1.41E-11	0.280672	0.339	0.182	2.24E-07	Skin_Eryth	CYB5A
IPO77	1.42E-11	0.302133	0.226	0.104	2.27E-07	Skin_Eryth	IPO7
OAZ25	1.48E-11	0.343704	0.332	0.178	2.36E-07	Skin_Eryth	OAZ2
PDCL32	1.49E-11	0.251255	0.197	0.084	2.37E-07	Skin_Eryth	PDCL3
ATP5J9	1.54E-11	0.351568	0.653	0.436	2.46E-07	Skin_Eryth	ATP5J
PRPF38B5	1.55E-11	0.273643	0.358	0.193	2.47E-07	Skin_Eryth	PRPF38B
METTL91	1.56E-11	0.254131	0.391	0.222	2.48E-07	Skin_Eryth	METTL9
WNK13	1.57E-11	0.33741	0.307	0.16	2.5E-07	Skin_Eryth	WNK1
SNX22	1.59E-11	0.358026	0.288	0.148	2.54E-07	Skin_Eryth	SNX2
KPNA3	1.6E-11	0.338309	0.204	0.089	2.56E-07	Skin_Eryth	KPNA3
MSL2	1.61E-11	0.308272	0.113	0.037	2.57E-07	Skin_Eryth	MSL2
NOP102	1.84E-11	0.334031	0.573	0.369	2.94E-07	Skin_Eryth	NOP10
PPP2CB1	1.86E-11	0.405277	0.303	0.163	2.97E-07	Skin_Eryth	PPP2CB
BBC31	1.87E-11	0.28259	0.172	0.068	2.99E-07	Skin_Eryth	BBC3
MAPK14	1.88E-11	0.30614	0.153	0.059	3E-07	Skin_Eryth	MAPK14
MINOS12	1.95E-11	0.325489	0.493	0.307	3.11E-07	Skin_Eryth	MINOS1
CCNI9	1.97E-11	0.308267	0.723	0.518	3.15E-07	Skin_Eryth	CCNI
PPIA4	2.1E-11	0.322592	0.792	0.589	3.35E-07	Skin_Eryth	PPIA
DNAJB17	2.19E-11	0.36125	0.628	0.43	3.49E-07	Skin_Eryth	DNAJB1
TOMM221	2.33E-11	0.380683	0.372	0.213	3.71E-07	Skin_Eryth	TOMM22
TRIOBP2	2.65E-11	0.374066	0.23	0.108	4.23E-07	Skin_Eryth	TRIOBP
CCNL14	2.68E-11	0.27175	0.54	0.34	4.28E-07	Skin_Eryth	CCNL1
CGGBP11	2.71E-11	0.34992	0.292	0.151	4.33E-07	Skin_Eryth	CGGBP1
MSL11	2.74E-11	0.286292	0.215	0.098	4.37E-07	Skin_Eryth	MSL1
COX7A2L2	2.93E-11	0.364716	0.507	0.321	4.67E-07	Skin_Eryth	COX7A2L
CCT4	2.99E-11	0.317982	0.398	0.227	4.77E-07	Skin_Eryth	CCT4
RABGGTB6	3.06E-11	0.335716	0.219	0.101	4.88E-07	Skin_Eryth	RABGGTB
C14orf22	3.2E-11	0.401328	0.642	0.449	5.1E-07	Skin_Eryth	C14orf2
CDV34	3.21E-11	0.371314	0.325	0.174	5.12E-07	Skin_Eryth	CDV3

EIF2A4	3.27E-11	0.327289	0.296	0.153	5.21E-07	Skin_Eryth	EIF2A
SIRPA1	3.72E-11	0.315953	0.193	0.083	5.94E-07	Skin_Eryth	SIRPA
NAA102	3.75E-11	0.292515	0.303	0.16	5.98E-07	Skin_Eryth	NAA10
NUCB14	3.79E-11	0.465349	0.358	0.206	6.05E-07	Skin_Eryth	NUCB1
HNRNPD6	3.95E-11	0.388759	0.401	0.24	6.29E-07	Skin_Eryth	HNRNPD
PAN36	3.96E-11	0.336414	0.19	0.081	6.32E-07	Skin_Eryth	PAN3
MAF11	4.03E-11	0.282012	0.35	0.195	6.43E-07	Skin_Eryth	MAF1
RNF112	4.04E-11	0.405869	0.365	0.208	6.45E-07	Skin_Eryth	RNF11
NME13	4.1E-11	0.322894	0.31	0.165	6.53E-07	Skin_Eryth	NME1
PPP3R14	4.31E-11	0.272739	0.172	0.07	6.88E-07	Skin_Eryth	PPP3R1
KHDRBS14	4.32E-11	0.322889	0.38	0.215	6.89E-07	Skin_Eryth	KHDRBS1
BLCAP2	4.43E-11	0.314935	0.274	0.139	7.06E-07	Skin_Eryth	BLCAP
IFRD14	4.44E-11	0.481179	0.38	0.22	7.08E-07	Skin_Eryth	IFRD1
RPGR2	4.54E-11	0.282236	0.106	0.034	7.24E-07	Skin_Eryth	RPGR
NDUFS58	4.65E-11	0.346266	0.65	0.455	7.41E-07	Skin_Eryth	NDUFS5
ZNHIT11	4.72E-11	0.369021	0.464	0.295	7.53E-07	Skin_Eryth	ZNHIT1
MAFF1	5.22E-11	0.515533	0.401	0.238	8.33E-07	Skin_Eryth	MAFF
GOLGA71	5.53E-11	0.323478	0.325	0.175	8.82E-07	Skin_Eryth	GOLGA7
DYNLT32	5.54E-11	0.296526	0.259	0.127	8.84E-07	Skin_Eryth	DYNLT3
PRDX28	5.86E-11	0.271968	0.511	0.312	9.35E-07	Skin_Eryth	PRDX2
FGFR1OP2	6.17E-11	0.323378	0.296	0.154	9.84E-07	Skin_Eryth	FGFR1OP2
GLIPR25	6.3E-11	0.261001	0.197	0.085	1E-06	Skin_Eryth	GLIPR2
NUDC2	6.43E-11	0.390868	0.416	0.25	1.03E-06	Skin_Eryth	NUDC
MRPL324	6.63E-11	0.364962	0.27	0.137	1.06E-06	Skin_Eryth	MRPL32
NR4A15	7.28E-11	0.318326	0.434	0.257	1.16E-06	Skin_Eryth	NR4A1
MYH97	7.4E-11	0.290848	0.339	0.186	1.18E-06	Skin_Eryth	MYH9
KDELC22	7.86E-11	0.266868	0.109	0.036	1.25E-06	Skin_Eryth	KDELC2
SMDT13	8.12E-11	0.335198	0.558	0.373	1.29E-06	Skin_Eryth	SMDT1
YTHDF22	8.71E-11	0.373103	0.318	0.175	1.39E-06	Skin_Eryth	YTHDF2
UHRF22	8.82E-11	0.287338	0.128	0.046	1.41E-06	Skin_Eryth	UHRF2
MOB46	9.07E-11	0.277939	0.208	0.092	1.45E-06	Skin_Eryth	MOB4
RAB7A3	9.26E-11	0.332795	0.573	0.372	1.48E-06	Skin_Eryth	RAB7A
CCDC1241	9.32E-11	0.297892	0.299	0.159	1.49E-06	Skin_Eryth	CCDC124
DDIT32	9.39E-11	0.251122	0.299	0.154	1.5E-06	Skin_Eryth	DDIT3
MPG3	1E-10	0.379774	0.318	0.173	1.6E-06	Skin_Eryth	MPG
LINC00116	1.06E-10	0.35489	0.237	0.116	1.69E-06	Skin_Eryth	LINC00116
MYO5C2	1.06E-10	0.332158	0.131	0.049	1.7E-06	Skin_Eryth	MYO5C
FILIP1L8	1.1E-10	0.280561	0.197	0.085	1.75E-06	Skin_Eryth	FILIP1L
SDHD2	1.71E-10	0.522538	0.358	0.215	2.73E-06	Skin_Eryth	SDHD
RAB136	1.75E-10	0.426703	0.365	0.219	2.79E-06	Skin_Eryth	RAB13
POMP6	1.77E-10	0.351617	0.595	0.403	2.82E-06	Skin_Eryth	POMP
BMPR24	1.78E-10	0.328907	0.219	0.103	2.83E-06	Skin_Eryth	BMPR2
PRMT16	1.8E-10	0.263384	0.35	0.192	2.87E-06	Skin_Eryth	PRMT1
RALB1	1.85E-10	0.371081	0.219	0.106	2.95E-06	Skin_Eryth	RALB
SWAP702	1.86E-10	0.314044	0.274	0.14	2.97E-06	Skin_Eryth	SWAP70
CHTOP1	1.87E-10	0.286559	0.219	0.102	2.98E-06	Skin_Eryth	CHTOP
KLF114	1.95E-10	0.304157	0.168	0.071	3.12E-06	Skin_Eryth	KLF11

FRYL3	1.99E-10	0.332033	0.197	0.089	3.18E-06	Skin_Eryth	FRYL
RQCD15	2.03E-10	0.271696	0.161	0.066	3.24E-06	Skin_Eryth	RQCD1
POGZ3	2.12E-10	0.271357	0.197	0.089	3.38E-06	Skin_Eryth	POGZ
B3GNT52	2.12E-10	0.361445	0.201	0.092	3.39E-06	Skin_Eryth	B3GNT5
PSMB63	2.15E-10	0.304634	0.496	0.321	3.42E-06	Skin_Eryth	PSMB6
N4BP2L29	2.23E-10	0.285493	0.467	0.293	3.56E-06	Skin_Eryth	N4BP2L2
BANF12	2.3E-10	0.269919	0.398	0.233	3.67E-06	Skin_Eryth	BANF1
BCAT12	2.35E-10	0.296471	0.172	0.072	3.75E-06	Skin_Eryth	BCAT1
TSTA33	2.48E-10	0.493297	0.31	0.176	3.95E-06	Skin_Eryth	TSTA3
CHMP4B5	2.52E-10	0.452074	0.474	0.306	4.02E-06	Skin_Eryth	CHMP4B
GAS2L12	2.53E-10	0.286556	0.135	0.052	4.03E-06	Skin_Eryth	GAS2L1
PNISR7	2.8E-10	0.261409	0.54	0.347	4.46E-06	Skin_Eryth	PNISR
CHIC23	3E-10	0.354763	0.336	0.187	4.78E-06	Skin_Eryth	CHIC2
MVB12A	3.12E-10	0.290067	0.215	0.102	4.98E-06	Skin_Eryth	MVB12A
UBE2W3	3.12E-10	0.525362	0.237	0.121	4.98E-06	Skin_Eryth	UBE2W
SRSF94	3.16E-10	0.368786	0.526	0.354	5.04E-06	Skin_Eryth	SRSF9
GTF2H2	3.58E-10	0.34166	0.102	0.034	5.71E-06	Skin_Eryth	GTF2H2
TEAD21	3.73E-10	0.273523	0.088	0.027	5.95E-06	Skin_Eryth	TEAD2
ATRX7	4.27E-10	0.339263	0.358	0.207	6.81E-06	Skin_Eryth	ATRX
RAP1A4	4.65E-10	0.394157	0.431	0.275	7.41E-06	Skin_Eryth	RAP1A
RDX3	4.86E-10	0.397421	0.325	0.187	7.76E-06	Skin_Eryth	RDX
SEPT26	5E-10	0.406623	0.383	0.236	7.97E-06	Skin_Eryth	SEPT2
DUSP64	5.15E-10	0.297613	0.252	0.128	8.21E-06	Skin_Eryth	DUSP6
UGP21	5.16E-10	0.484854	0.456	0.3	8.23E-06	Skin_Eryth	UGP2
HNRNPC6	5.27E-10	0.434997	0.602	0.43	8.41E-06	Skin_Eryth	HNRNPC
PITPNB3	5.42E-10	0.354893	0.234	0.117	8.65E-06	Skin_Eryth	PITPNB
ATP5O2	5.47E-10	0.285575	0.62	0.43	8.73E-06	Skin_Eryth	ATP5O
PRDX52	6.35E-10	0.303464	0.701	0.491	1.01E-05	Skin_Eryth	PRDX5
C1orf1153	6.82E-10	0.262853	0.124	0.047	1.09E-05	Skin_Eryth	C1orf115
ZBTB7A4	6.82E-10	0.290433	0.245	0.123	1.09E-05	Skin_Eryth	ZBTB7A
GINM13	7.03E-10	0.25814	0.281	0.148	1.12E-05	Skin_Eryth	GINM1
PANK31	7.22E-10	0.355165	0.153	0.065	1.15E-05	Skin_Eryth	PANK3
MYEOV23	7.34E-10	0.310793	0.536	0.361	1.17E-05	Skin_Eryth	MYEOV2
NTPCR	7.61E-10	0.338951	0.182	0.084	1.21E-05	Skin_Eryth	NTPCR
NDUFB710	7.74E-10	0.287417	0.551	0.373	1.24E-05	Skin_Eryth	NDUFB7
RBM8A5	7.82E-10	0.328019	0.511	0.343	1.25E-05	Skin_Eryth	RBM8A
GSTO14	7.98E-10	0.259726	0.369	0.213	1.27E-05	Skin_Eryth	GSTO1
BPTF6	8.17E-10	0.362471	0.296	0.164	1.3E-05	Skin_Eryth	BPTF
CAPNS12	8.48E-10	0.413649	0.401	0.25	1.35E-05	Skin_Eryth	CAPNS1
MOCS21	9E-10	0.325758	0.208	0.101	1.44E-05	Skin_Eryth	MOCS2
PPIL31	9.02E-10	0.327772	0.157	0.066	1.44E-05	Skin_Eryth	PPIL3
SETD55	9.13E-10	0.296385	0.234	0.118	1.46E-05	Skin_Eryth	SETD5
RNASEH2C	9.15E-10	0.283765	0.328	0.186	1.46E-05	Skin_Eryth	RNASEH2C
FAM174A3	9.35E-10	0.279093	0.182	0.084	1.49E-05	Skin_Eryth	FAM174A
TNPO14	9.74E-10	0.340693	0.245	0.126	1.55E-05	Skin_Eryth	TNPO1
COMT2	1.02E-09	0.296543	0.321	0.183	1.63E-05	Skin_Eryth	COMT
TARDBP2	1.06E-09	0.339832	0.226	0.113	1.69E-05	Skin_Eryth	TARDBP

BCL7B1	1.26E-09	0.25264	0.212	0.101	2.01E-05	Skin_Eryth	BCL7B
PPP1R9A5	1.27E-09	0.36453	0.128	0.051	2.02E-05	Skin_Eryth	PPP1R9A
GABARAPL	1.3E-09	0.262874	0.682	0.481	2.08E-05	Skin_Eryth	GABARAPL2
THEM6	1.34E-09	0.266338	0.135	0.054	2.14E-05	Skin_Eryth	THEM6
AIDA3	1.43E-09	0.323385	0.212	0.104	2.28E-05	Skin_Eryth	AIDA
RAI143	1.44E-09	0.324845	0.182	0.083	2.3E-05	Skin_Eryth	RAI14
CTNNAL14	1.48E-09	0.255375	0.193	0.089	2.35E-05	Skin_Eryth	CTNNAL1
UBE2Z1	1.48E-09	0.373807	0.197	0.093	2.37E-05	Skin_Eryth	UBE2Z
MIIP	1.61E-09	0.270327	0.135	0.053	2.57E-05	Skin_Eryth	MIIP
CMPK15	1.61E-09	0.275545	0.467	0.301	2.57E-05	Skin_Eryth	CMPK1
DNAJB65	1.69E-09	0.278535	0.609	0.42	2.69E-05	Skin_Eryth	DNAJB6
NFE2L26	1.7E-09	0.367564	0.445	0.288	2.72E-05	Skin_Eryth	NFE2L2
TMEM165	1.75E-09	0.321113	0.35	0.205	2.79E-05	Skin_Eryth	TMEM165
GLUD15	1.77E-09	0.295123	0.296	0.163	2.82E-05	Skin_Eryth	GLUD1
PJA211	1.78E-09	0.347579	0.277	0.151	2.85E-05	Skin_Eryth	PJA2
MARCH3	1.86E-09	0.329841	0.131	0.052	2.97E-05	Skin_Eryth	MARCH3
FAM120A8	1.87E-09	0.329946	0.274	0.148	2.98E-05	Skin_Eryth	FAM120A
CCDC476	1.98E-09	0.34618	0.259	0.14	3.15E-05	Skin_Eryth	CCDC47
PSMA12	2.26E-09	0.314283	0.405	0.253	3.6E-05	Skin_Eryth	PSMA1
GBE13	2.35E-09	0.301543	0.139	0.056	3.75E-05	Skin_Eryth	GBE1
CASC46	2.39E-09	0.313748	0.237	0.122	3.82E-05	Skin_Eryth	CASC4
HIVEP24	2.46E-09	0.265589	0.197	0.092	3.93E-05	Skin_Eryth	HIVEP2
SRRM210	2.77E-09	0.296214	0.511	0.348	4.41E-05	Skin_Eryth	SRRM2
ARID4B4	2.85E-09	0.359173	0.391	0.242	4.54E-05	Skin_Eryth	ARID4B
SEC23A3	3.03E-09	0.323158	0.161	0.07	4.83E-05	Skin_Eryth	SEC23A
TMEM126I	3.06E-09	0.329044	0.237	0.123	4.89E-05	Skin_Eryth	TMEM126B
SERINC16	3.07E-09	0.385191	0.332	0.197	4.9E-05	Skin_Eryth	SERINC1
MAP48	3.66E-09	0.275874	0.248	0.129	5.84E-05	Skin_Eryth	MAP4
LBR2	3.82E-09	0.264701	0.153	0.065	6.09E-05	Skin_Eryth	LBR
UBE2D36	3.93E-09	0.324447	0.708	0.533	6.27E-05	Skin_Eryth	UBE2D3
CALM18	4.06E-09	0.363757	0.825	0.651	6.48E-05	Skin_Eryth	CALM1
GCH12	4.57E-09	0.339459	0.139	0.058	7.29E-05	Skin_Eryth	GCH1
TCEA14	5E-09	0.357807	0.383	0.24	7.97E-05	Skin_Eryth	TCEA1
ATF61	5.04E-09	0.25964	0.204	0.1	8.04E-05	Skin_Eryth	ATF6
COX6A12	5.07E-09	0.32907	0.719	0.531	8.09E-05	Skin_Eryth	COX6A1
ITGB3BP	5.23E-09	0.34142	0.153	0.067	8.34E-05	Skin_Eryth	ITGB3BP
MTCH12	5.69E-09	0.319187	0.387	0.243	9.07E-05	Skin_Eryth	MTCH1
ADSS3	5.88E-09	0.294999	0.226	0.116	9.39E-05	Skin_Eryth	ADSS
MRFAP15	6.04E-09	0.319708	0.416	0.266	9.63E-05	Skin_Eryth	MRFAP1
SDF43	6.11E-09	0.274685	0.347	0.209	9.75E-05	Skin_Eryth	SDF4
SEPHS1	6.15E-09	0.270968	0.135	0.056	9.81E-05	Skin_Eryth	SEPHS1
VKORC1L1	6.54E-09	0.278574	0.142	0.061	0.000104	Skin_Eryth	VKORC1L1
SKAP21	6.67E-09	0.342596	0.223	0.112	0.000106	Skin_Eryth	SKAP2
EIF2S34	6.94E-09	0.311748	0.281	0.157	0.000111	Skin_Eryth	EIF2S3
SLC40A11	7.14E-09	0.308074	0.168	0.077	0.000114	Skin_Eryth	SLC40A1
RBM73	7.17E-09	0.251681	0.23	0.118	0.000114	Skin_Eryth	RBM7
TXNRD2	7.67E-09	0.271195	0.109	0.041	0.000122	Skin_Eryth	TXNRD2

SMARCE11	8.26E-09	0.281041	0.252	0.135	0.000132	Skin_Eryth	SMARCE1
MRPL502	8.89E-09	0.266907	0.201	0.099	0.000142	Skin_Eryth	MRPL50
PSMB56	9.69E-09	0.361874	0.383	0.241	0.000155	Skin_Eryth	PSMB5
EFCAB143	9.73E-09	0.362458	0.215	0.112	0.000155	Skin_Eryth	EFCAB14
STRA133	1E-08	0.276775	0.328	0.195	0.00016	Skin_Eryth	STRA13
TRAPPC10:	1.02E-08	0.332595	0.139	0.059	0.000163	Skin_Eryth	TRAPPC10
RAB5A3	1.28E-08	0.384265	0.339	0.207	0.000203	Skin_Eryth	RAB5A
EIF1AY4	1.28E-08	0.278127	0.182	0.087	0.000204	Skin_Eryth	EIF1AY
CDC42BPA	1.35E-08	0.278376	0.157	0.07	0.000215	Skin_Eryth	CDC42BPA
CLDND15	1.36E-08	0.300071	0.328	0.191	0.000217	Skin_Eryth	CLDND1
LNPEP6	1.38E-08	0.254505	0.153	0.067	0.000221	Skin_Eryth	LNPEP
PLEKHB24	1.45E-08	0.368943	0.266	0.149	0.000231	Skin_Eryth	PLEKHB2
TAF1D6	1.45E-08	0.316612	0.369	0.227	0.000232	Skin_Eryth	TAF1D
TPD52L2	1.52E-08	0.266185	0.288	0.164	0.000242	Skin_Eryth	TPD52L2
MED282	1.6E-08	0.364846	0.274	0.155	0.000254	Skin_Eryth	MED28
NDUFB52	1.73E-08	0.333678	0.383	0.245	0.000276	Skin_Eryth	NDUFB5
ZNF223	1.87E-08	0.283487	0.212	0.108	0.000298	Skin_Eryth	ZNF22
TRIM475	1.92E-08	0.26582	0.139	0.059	0.000307	Skin_Eryth	TRIM47
PAQR31	1.93E-08	0.353094	0.102	0.038	0.000307	Skin_Eryth	PAQR3
MEF2A6	1.95E-08	0.291115	0.212	0.106	0.000311	Skin_Eryth	MEF2A
EI243	2.15E-08	0.29353	0.314	0.187	0.000343	Skin_Eryth	EI24
SNRNP40	2.18E-08	0.279065	0.182	0.089	0.000347	Skin_Eryth	SNRNP40
RRAGC4	2.2E-08	0.26773	0.204	0.103	0.000351	Skin_Eryth	RRAGC
YIPF54	2.44E-08	0.285909	0.223	0.118	0.000389	Skin_Eryth	YIPF5
PRDX36	2.52E-08	0.339585	0.274	0.156	0.000402	Skin_Eryth	PRDX3
SUMF24	2.66E-08	0.295174	0.219	0.115	0.000424	Skin_Eryth	SUMF2
UQCRQ6	2.72E-08	0.305809	0.682	0.499	0.000434	Skin_Eryth	UQCRQ
SMIM192	2.74E-08	0.37454	0.321	0.199	0.000436	Skin_Eryth	SMIM19
ST54	2.78E-08	0.277757	0.139	0.061	0.000444	Skin_Eryth	ST5
ARL13B4	3.19E-08	0.278289	0.12	0.049	0.00051	Skin_Eryth	ARL13B
MRPS18B	3.23E-08	0.272011	0.237	0.129	0.000516	Skin_Eryth	MRPS18B
UACA4	3.23E-08	0.282977	0.215	0.112	0.000516	Skin_Eryth	UACA
TMEM192:	3.32E-08	0.26685	0.135	0.058	0.00053	Skin_Eryth	TMEM192
TFG4	3.34E-08	0.415299	0.307	0.188	0.000532	Skin_Eryth	TFG
ZMYND11:	3.56E-08	0.300725	0.168	0.08	0.000568	Skin_Eryth	ZMYND11
VBP1	4.07E-08	0.266369	0.23	0.122	0.000649	Skin_Eryth	VBP1
EIF4G23	4.08E-08	0.292271	0.613	0.46	0.00065	Skin_Eryth	EIF4G2
UBE2E32	4.14E-08	0.310877	0.296	0.174	0.00066	Skin_Eryth	UBE2E3
SLC30A76	4.31E-08	0.307886	0.172	0.083	0.000687	Skin_Eryth	SLC30A7
INO80C	4.32E-08	0.273184	0.245	0.133	0.000688	Skin_Eryth	INO80C
VPS368	4.38E-08	0.274311	0.215	0.113	0.000699	Skin_Eryth	VPS36
JMJD1C5	5.17E-08	0.358156	0.369	0.235	0.000825	Skin_Eryth	JMJD1C
CISD13	5.2E-08	0.280374	0.248	0.137	0.000829	Skin_Eryth	CISD1
VEZF14	5.23E-08	0.258188	0.179	0.088	0.000834	Skin_Eryth	VEZF1
CLOCK1	5.62E-08	0.341122	0.153	0.07	0.000896	Skin_Eryth	CLOCK
WASF23	5.73E-08	0.303881	0.467	0.323	0.000913	Skin_Eryth	WASF2
NDUFA102	5.83E-08	0.258002	0.307	0.184	0.000929	Skin_Eryth	NDUFA10

ACAT12	6.08E-08	0.330482	0.241	0.134	0.000969	Skin_Eryth	ACAT1
DEGS13	6.4E-08	0.260933	0.234	0.126	0.001021	Skin_Eryth	DEGS1
GOPC3	6.75E-08	0.263066	0.197	0.101	0.001076	Skin_Eryth	GOPC
PIN4	7.87E-08	0.378381	0.321	0.2	0.001255	Skin_Eryth	PIN4
COX7B9	8.95E-08	0.314965	0.569	0.409	0.001427	Skin_Eryth	COX7B
FAM200B	9.13E-08	0.272676	0.27	0.158	0.001456	Skin_Eryth	FAM200B
ARID1B1	9.52E-08	0.307337	0.172	0.084	0.001519	Skin_Eryth	ARID1B
TRAPPC3	9.58E-08	0.260852	0.328	0.202	0.001528	Skin_Eryth	TRAPPC3
NFKBIZ5	9.7E-08	0.360729	0.376	0.245	0.001547	Skin_Eryth	NFKBIZ
NUFIP26	1.01E-07	0.365695	0.263	0.152	0.001606	Skin_Eryth	NUFIP2
PDCL1	1.07E-07	0.289947	0.172	0.085	0.001705	Skin_Eryth	PDCL
SCOC2	1.2E-07	0.285232	0.285	0.171	0.001908	Skin_Eryth	SCOC
PPP1R14B1	1.2E-07	0.31715	0.296	0.177	0.001918	Skin_Eryth	PPP1R14B
FAM204A1	1.2E-07	0.301318	0.281	0.164	0.00192	Skin_Eryth	FAM204A
SRRM17	1.21E-07	0.293231	0.474	0.327	0.001922	Skin_Eryth	SRRM1
SCARB210	1.25E-07	0.251042	0.27	0.157	0.001995	Skin_Eryth	SCARB2
UBE2H4	1.26E-07	0.421084	0.252	0.146	0.002017	Skin_Eryth	UBE2H
ATXN32	1.35E-07	0.267608	0.168	0.082	0.002152	Skin_Eryth	ATXN3
BIRC63	1.37E-07	0.25396	0.208	0.111	0.00218	Skin_Eryth	BIRC6
A4GALT4	1.48E-07	0.311259	0.175	0.087	0.002356	Skin_Eryth	A4GALT
SPEN4	1.53E-07	0.270492	0.201	0.104	0.002434	Skin_Eryth	SPEN
ALDH28	1.56E-07	0.267332	0.226	0.124	0.002485	Skin_Eryth	ALDH2
CAPZA26	1.68E-07	0.314211	0.42	0.279	0.002682	Skin_Eryth	CAPZA2
RNF1311	1.76E-07	0.282899	0.281	0.167	0.002808	Skin_Eryth	RNF13
KPNA43	1.97E-07	0.368963	0.255	0.147	0.003149	Skin_Eryth	KPNA4
GUSB2	2.48E-07	0.32389	0.237	0.137	0.003951	Skin_Eryth	GUSB
HDGF4	2.78E-07	0.399842	0.241	0.139	0.00444	Skin_Eryth	HDGF
PROSC	2.79E-07	0.255084	0.172	0.085	0.00445	Skin_Eryth	PROSC
EIF3A5	2.99E-07	0.307536	0.423	0.294	0.004767	Skin_Eryth	EIF3A
PSMD122	3.02E-07	0.344588	0.259	0.152	0.00482	Skin_Eryth	PSMD12
VAT12	3.06E-07	0.265747	0.245	0.138	0.004876	Skin_Eryth	VAT1
TRAM14	3.19E-07	0.293811	0.434	0.3	0.005094	Skin_Eryth	TRAM1
TM2D31	3.2E-07	0.27493	0.208	0.114	0.0051	Skin_Eryth	TM2D3
OTUD43	3.57E-07	0.287309	0.168	0.084	0.005697	Skin_Eryth	OTUD4
AKIRIN22	3.6E-07	0.278202	0.299	0.182	0.005745	Skin_Eryth	AKIRIN2
PCBD15	3.72E-07	0.299199	0.266	0.158	0.005933	Skin_Eryth	PCBD1
DPM34	4.24E-07	0.301365	0.292	0.181	0.006757	Skin_Eryth	DPM3
WARS3	5.31E-07	0.255848	0.161	0.08	0.008463	Skin_Eryth	WARS
RTF12	5.54E-07	0.31013	0.372	0.245	0.008833	Skin_Eryth	RTF1
ARHGAP26	5.55E-07	0.299802	0.124	0.056	0.008859	Skin_Eryth	ARHGAP26
STAG23	5.81E-07	0.263478	0.204	0.111	0.009269	Skin_Eryth	STAG2
MED132	6.12E-07	0.274409	0.226	0.128	0.009754	Skin_Eryth	MED13
PAIP11	6.29E-07	0.255091	0.255	0.152	0.010026	Skin_Eryth	PAIP1
SMIM41	6.57E-07	0.307546	0.157	0.078	0.010482	Skin_Eryth	SMIM4
DHX154	6.74E-07	0.295686	0.204	0.112	0.010742	Skin_Eryth	DHX15
NAA153	6.78E-07	0.268271	0.153	0.075	0.010821	Skin_Eryth	NAA15
SEPT102	7.09E-07	0.321172	0.263	0.157	0.011316	Skin_Eryth	SEPT10

ADNP2	7.11E-07	0.260553	0.179	0.094	0.011343	Skin_Eryth	ADNP
RAB144	7.3E-07	0.269175	0.299	0.188	0.011639	Skin_Eryth	RAB14
HNRNPU5	7.56E-07	0.338192	0.478	0.349	0.01205	Skin_Eryth	HNRNPU
SMG17	7.83E-07	0.313768	0.215	0.121	0.012496	Skin_Eryth	SMG1
CTTNBP2N	8.2E-07	0.297169	0.201	0.109	0.013075	Skin_Eryth	CTTNBP2NL
RHBDD2	8.47E-07	0.252439	0.215	0.12	0.013511	Skin_Eryth	RHBDD2
PSMC31	8.64E-07	0.282735	0.318	0.204	0.013776	Skin_Eryth	PSMC3
UPF21	8.72E-07	0.264753	0.252	0.147	0.013902	Skin_Eryth	UPF2
CSDE15	9.72E-07	0.252449	0.493	0.359	0.015497	Skin_Eryth	CSDE1
RSPRY1	1.06E-06	0.259104	0.139	0.067	0.016964	Skin_Eryth	RSPRY1
TAOK14	1.08E-06	0.268656	0.201	0.111	0.01724	Skin_Eryth	TAOK1
VPS284	1.09E-06	0.258962	0.482	0.349	0.017325	Skin_Eryth	VPS28
IMPAD15	1.41E-06	0.335756	0.172	0.092	0.02244	Skin_Eryth	IMPAD1
MRPL221	1.57E-06	0.282164	0.255	0.154	0.025066	Skin_Eryth	MRPL22
PRKRA2	1.67E-06	0.302213	0.219	0.126	0.026597	Skin_Eryth	PRKRA
AP1AR	1.71E-06	0.250425	0.109	0.049	0.027195	Skin_Eryth	AP1AR
TCEB14	1.97E-06	0.300311	0.493	0.359	0.031387	Skin_Eryth	TCEB1
TSPYL16	2.14E-06	0.309648	0.252	0.155	0.034123	Skin_Eryth	TSPYL1
DCAF72	2.27E-06	0.251147	0.172	0.091	0.036236	Skin_Eryth	DCAF7
ATF22	3.09E-06	0.257647	0.15	0.077	0.049279	Skin_Eryth	ATF2
GRPEL13	3.38E-06	0.289718	0.219	0.13	0.053947	Skin_Eryth	GRPEL1
PUM21	3.39E-06	0.280657	0.201	0.115	0.054021	Skin_Eryth	PUM2
WRNIP1	3.64E-06	0.257875	0.12	0.057	0.057983	Skin_Eryth	WRNIP1
UBE2E23	3.78E-06	0.265614	0.153	0.08	0.060316	Skin_Eryth	UBE2E2
PTBP11	4.14E-06	0.310545	0.245	0.149	0.065983	Skin_Eryth	PTBP1
FOSL25	4.3E-06	0.300854	0.339	0.22	0.06865	Skin_Eryth	FOSL2
MAFG2	4.31E-06	0.252705	0.215	0.124	0.068733	Skin_Eryth	MAFG
NRIP13	5.14E-06	0.256865	0.109	0.05	0.081994	Skin_Eryth	NRIP1
UBE2A3	5.68E-06	0.311002	0.318	0.213	0.090571	Skin_Eryth	UBE2A
COPS22	6.22E-06	0.293317	0.212	0.126	0.099282	Skin_Eryth	COPS2
ILK4	8.2E-06	0.255087	0.241	0.147	0.13084	Skin_Eryth	ILK
PRKD32	8.67E-06	0.279655	0.15	0.079	0.138248	Skin_Eryth	PRKD3
VDAC15	8.82E-06	0.294711	0.354	0.242	0.140686	Skin_Eryth	VDAC1
PHF232	9.06E-06	0.254551	0.175	0.097	0.144442	Skin_Eryth	PHF23
C1orf432	9.16E-06	0.283076	0.328	0.228	0.14605	Skin_Eryth	C1orf43
NT5C3B2	9.49E-06	0.251176	0.172	0.096	0.151344	Skin_Eryth	NT5C3B
ABCE14	1.2E-05	0.335401	0.172	0.097	0.191589	Skin_Eryth	ABCE1
MED4	1.21E-05	0.26344	0.281	0.184	0.192729	Skin_Eryth	MED4
PRPF40A6	1.22E-05	0.300626	0.285	0.188	0.194925	Skin_Eryth	PRPF40A
BAZ1A5	1.87E-05	0.330568	0.354	0.245	0.298318	Skin_Eryth	BAZ1A
RALGAPA1	3.45E-05	0.267391	0.139	0.075	0.549816	Skin_Eryth	RALGAPA1
GLOD41	4.34E-05	0.282099	0.197	0.12	0.692333	Skin_Eryth	GLOD4
LRRC596	4.42E-05	0.283159	0.226	0.144	0.704682	Skin_Eryth	LRRC59
DLG12	5.01E-05	0.278504	0.157	0.09	0.798949	Skin_Eryth	DLG1
MAP3K27	7.39E-05	0.257758	0.201	0.125	1	Skin_Eryth	MAP3K2
NDUFA64	0.000139	0.29244	0.431	0.322	1	Skin_Eryth	NDUFA6
ZNF7702	0.000141	0.315173	0.135	0.076	1	Skin_Eryth	ZNF770

RBBP43	0.000154	0.254643	0.226	0.15	1	Skin_Eryth	RBBP4
C2orf761	0.000582	0.285614	0.088	0.046	1	Skin_Eryth	C2orf76
FDX11	0.000637	0.253224	0.237	0.166	1	Skin_Eryth	FDX1
KIAA0907	0.000664	0.258355	0.139	0.084	1	Skin_Eryth	KIAA0907
PFDN4	0.000887	0.31842	0.241	0.168	1	Skin_Eryth	PFDN4
TSC22D25	0.000994	0.302836	0.168	0.107	1	Skin_Eryth	TSC22D2
MDM47	0.004866	0.30393	0.19	0.138	1	Skin_Eryth	MDM4
MLANA	0	5.448403	0.987	0.006	0	Skin_Mela	MLANA
PMEL	0	5.267686	0.936	0.009	0	Skin_Mela	PMEL
CAPN3	0	3.486348	0.846	0.036	0	Skin_Mela	CAPN3
KIT	0	2.770357	0.654	0.012	0	Skin_Mela	KIT
CRABP1	0	2.746577	0.551	0.005	0	Skin_Mela	CRABP1
BCAN	0	2.507221	0.59	0.004	0	Skin_Mela	BCAN
TRPM1	0	2.453487	0.615	0.008	0	Skin_Mela	TRPM1
CEP72	0	2.22819	0.474	0.01	0	Skin_Mela	CEP72
GPR143	0	2.070752	0.5	0.007	0	Skin_Mela	GPR143
MIA1	0	1.852856	0.449	0.003	0	Skin_Mela	MIA
CA14	0	1.261306	0.321	0.001	0	Skin_Mela	CA14
KCNJ13	0	0.664621	0.141	0.001	0	Skin_Mela	KCNJ13
IDI2.AS1	0	0.641177	0.141	0.001	0	Skin_Mela	IDI2.AS1
MITF2	6.9E-301	3.71925	0.859	0.042	1.1E-296	Skin_Mela	MITF
NSG12	4.9E-297	3.002369	0.808	0.037	7.8E-293	Skin_Mela	NSG1
SNCA6	2.3E-285	2.93307	0.872	0.045	3.6E-281	Skin_Mela	SNCA
MCOLN32	1.2E-279	2.256498	0.603	0.021	1.9E-275	Skin_Mela	MCOLN3
GPM6B2	3.3E-259	3.083946	0.808	0.042	5.2E-255	Skin_Mela	GPM6B
FRZB2	5.9E-244	2.263377	0.436	0.012	9.4E-240	Skin_Mela	FRZB
PLXNC12	4.7E-238	1.781281	0.474	0.015	7.5E-234	Skin_Mela	PLXNC1
RAB381	2E-226	2.017231	0.538	0.021	3.2E-222	Skin_Mela	RAB38
OCA2	5.8E-223	1.181924	0.256	0.005	9.2E-219	Skin_Mela	OCA2
QPCT3	3.2E-205	3.475262	0.885	0.066	5.2E-201	Skin_Mela	QPCT
GPR1552	7.8E-186	2.156699	0.564	0.028	1.2E-181	Skin_Mela	GPR155
MMP17	4.5E-156	0.897352	0.192	0.004	7.2E-152	Skin_Mela	MMP17
PRELP4	2.6E-143	2.351988	0.603	0.04	4.2E-139	Skin_Mela	PRELP
EDNRB6	6.4E-136	1.85289	0.462	0.025	1E-131	Skin_Mela	EDNRB
BIRC7	4.2E-131	0.907641	0.179	0.004	6.7E-127	Skin_Mela	BIRC7
CYB561A3	3.5E-126	2.887045	0.692	0.063	5.6E-122	Skin_Mela	CYB561A3
GPNMB4	4.5E-120	2.997193	0.936	0.126	7.1E-116	Skin_Mela	GPNMB
STXBP63	4.4E-117	1.900517	0.487	0.033	7E-113	Skin_Mela	STXBP6
MCHR1	1.7E-110	0.260234	0.051	0	2.7E-106	Skin_Mela	MCHR1
APOE4	1.1E-99	3.337442	0.962	0.166	1.73E-95	Skin_Mela	APOE
CDK22	9.28E-96	1.40158	0.41	0.028	1.48E-91	Skin_Mela	CDK2
PAG13	1.59E-95	2.025759	0.538	0.048	2.53E-91	Skin_Mela	PAG1
APOD2	6.41E-94	2.078853	0.91	0.141	1.02E-89	Skin_Mela	APOD
TPPP37	3.05E-89	2.918759	0.782	0.109	4.87E-85	Skin_Mela	TPPP3
TFAP2A3	1.84E-85	1.940507	0.526	0.052	2.93E-81	Skin_Mela	TFAP2A
SRPX2	1.52E-82	1.291502	0.41	0.031	2.42E-78	Skin_Mela	SRPX
LSAMP2	4.28E-82	1.230758	0.321	0.02	6.82E-78	Skin_Mela	LSAMP

OSTM1	2.78E-81	1.946561	0.526	0.054	4.43E-77	Skin_Mela	OSTM1
BMPR1B	9.46E-81	1.003498	0.167	0.006	1.51E-76	Skin_Mela	BMPR1B
SLC1A42	3.47E-80	1.523148	0.385	0.029	5.53E-76	Skin_Mela	SLC1A4
MYO5A2	1.15E-79	1.603782	0.41	0.033	1.83E-75	Skin_Mela	MYO5A
LZTS1	6.35E-78	0.51596	0.103	0.002	1.01E-73	Skin_Mela	LZTS1
IRF42	6.48E-78	0.945713	0.244	0.012	1.03E-73	Skin_Mela	IRF4
SERPINE22	6.56E-77	1.80541	0.551	0.06	1.05E-72	Skin_Mela	SERPINE2
CDH32	2.79E-76	1.305758	0.269	0.015	4.44E-72	Skin_Mela	CDH3
C4orf482	1.33E-75	2.658925	0.795	0.143	2.13E-71	Skin_Mela	C4orf48
TUBB2B2	2.77E-75	1.583993	0.308	0.02	4.42E-71	Skin_Mela	TUBB2B
PRODH	5.72E-66	0.559204	0.103	0.003	9.12E-62	Skin_Mela	PRODH
SCUBE2	2.3E-65	0.515246	0.141	0.005	3.67E-61	Skin_Mela	SCUBE2
RAB325	2.11E-64	2.003514	0.603	0.089	3.37E-60	Skin_Mela	RAB32
CHCHD6	2.29E-64	2.06166	0.538	0.071	3.65E-60	Skin_Mela	CHCHD6
PMP228	3.71E-64	2.063693	0.769	0.138	5.92E-60	Skin_Mela	PMP22
MFSD122	1.74E-63	2.358006	0.603	0.093	2.78E-59	Skin_Mela	MFSD12
L1CAM	1.83E-63	1.00175	0.179	0.008	2.91E-59	Skin_Mela	L1CAM
KCNAB21	1.3E-62	1.061708	0.321	0.025	2.07E-58	Skin_Mela	KCNAB2
CYGB5	1.88E-62	1.22762	0.321	0.025	3.01E-58	Skin_Mela	CYGB
PCDH11X	4.87E-61	0.270297	0.077	0.002	7.76E-57	Skin_Mela	PCDH11X
SLC45A2	3.45E-60	0.403834	0.064	0.001	5.5E-56	Skin_Mela	SLC45A2
DSTYK2	2.36E-59	1.539662	0.397	0.041	3.77E-55	Skin_Mela	DSTYK
SEPT42	2.4E-59	1.423838	0.308	0.025	3.83E-55	Skin_Mela	SEPT4
RAMP12	4.3E-59	1.190044	0.308	0.025	6.86E-55	Skin_Mela	RAMP1
CDC42EP34	7.14E-57	2.049691	0.59	0.095	1.14E-52	Skin_Mela	CDC42EP3
ZNF749	2.41E-53	0.4961	0.128	0.005	3.84E-49	Skin_Mela	ZNF749
ILVBL1	8.01E-53	1.52932	0.423	0.052	1.28E-48	Skin_Mela	ILVBL
DOCK54	1.67E-52	1.428182	0.321	0.03	2.66E-48	Skin_Mela	DOCK5
C9orf39	3.07E-50	1.995195	0.564	0.099	4.89E-46	Skin_Mela	C9orf3
PLOD33	3.39E-50	1.390852	0.359	0.039	5.4E-46	Skin_Mela	PLOD3
MT1F1	1.56E-48	1.209511	0.462	0.063	2.5E-44	Skin_Mela	MT1F
ASB11	1.72E-48	0.325324	0.051	0.001	2.75E-44	Skin_Mela	ASB11
CALD18	1.29E-46	1.572959	0.821	0.195	2.06E-42	Skin_Mela	CALD1
SDCBP6	1.37E-46	2.403965	0.936	0.412	2.19E-42	Skin_Mela	SDCBP
GNAI13	2.37E-46	1.462841	0.436	0.062	3.78E-42	Skin_Mela	GNAI1
NOV3	3.3E-46	1.441669	0.282	0.026	5.26E-42	Skin_Mela	NOV
IRS22	4.72E-46	1.757203	0.564	0.105	7.53E-42	Skin_Mela	IRS2
IFI27L25	1.2E-45	1.813539	0.654	0.14	1.92E-41	Skin_Mela	IFI27L2
PON23	1.62E-45	1.601275	0.462	0.072	2.58E-41	Skin_Mela	PON2
TPCN2	3.66E-44	0.85786	0.192	0.013	5.84E-40	Skin_Mela	TPCN2
GMPR4	3.8E-44	1.567342	0.359	0.045	6.07E-40	Skin_Mela	GMPR
EMP37	1.13E-43	2.219937	0.923	0.351	1.8E-39	Skin_Mela	EMP3
GNG116	3.64E-43	1.414199	0.603	0.114	5.81E-39	Skin_Mela	GNG11
SGK14	3.84E-43	1.834093	0.577	0.113	6.12E-39	Skin_Mela	SGK1
BSG1	1.12E-42	2.056852	0.923	0.388	1.79E-38	Skin_Mela	BSG
CD6314	1.21E-42	2.02212	1	0.625	1.93E-38	Skin_Mela	CD63
CAV19	1.6E-42	1.483242	0.821	0.198	2.55E-38	Skin_Mela	CAV1

FEZ12	2.59E-42	1.222909	0.321	0.036	4.13E-38	Skin_Mela	FEZ1
MOK	3.13E-42	1.408252	0.359	0.046	5E-38	Skin_Mela	MOK
IGFBP77	4.38E-42	1.401702	0.923	0.265	6.99E-38	Skin_Mela	IGFBP7
PIP4K2A7	2.14E-40	1.429615	0.41	0.06	3.41E-36	Skin_Mela	PIP4K2A
CD596	2.14E-40	2.328622	0.962	0.515	3.41E-36	Skin_Mela	CD59
VAMP55	3.65E-40	1.944459	0.679	0.169	5.83E-36	Skin_Mela	VAMP5
LGALS110	3.79E-40	1.983899	0.987	0.411	6.05E-36	Skin_Mela	LGALS1
SEMA3C4	1.42E-39	1.096418	0.308	0.035	2.27E-35	Skin_Mela	SEMA3C
PYURF5	6.11E-39	1.978338	0.769	0.247	9.74E-35	Skin_Mela	PYURF
ST3GAL62	1.43E-38	1.081529	0.282	0.031	2.28E-34	Skin_Mela	ST3GAL6
COMT3	4.04E-38	1.63774	0.679	0.182	6.45E-34	Skin_Mela	COMT
INPP4B3	5.78E-38	1.3166	0.385	0.057	9.21E-34	Skin_Mela	INPP4B
A1BG3	1.62E-37	1.215833	0.41	0.063	2.59E-33	Skin_Mela	A1BG
S100B2	5.51E-37	1.064178	0.269	0.028	8.79E-33	Skin_Mela	S100B
C5orf30	2.21E-36	0.713218	0.179	0.014	3.52E-32	Skin_Mela	C5orf30
TSPAN47	3.5E-36	1.535339	0.551	0.118	5.58E-32	Skin_Mela	TSPAN4
HMG20B	5.62E-36	1.93049	0.641	0.175	8.96E-32	Skin_Mela	HMG20B
GSTP17	1.24E-35	2.158482	0.923	0.557	1.98E-31	Skin_Mela	GSTP1
PIR1	9.56E-35	0.845297	0.231	0.023	1.52E-30	Skin_Mela	PIR
PRKCDBP6	3.05E-34	1.67782	0.564	0.129	4.87E-30	Skin_Mela	PRKCDBP
TBC1D162	3.81E-34	1.240537	0.308	0.041	6.07E-30	Skin_Mela	TBC1D16
MARCKSL1	4.37E-34	1.785809	0.692	0.205	6.97E-30	Skin_Mela	MARCKSL1
SLC16A10	5.37E-34	1.133159	0.231	0.024	8.57E-30	Skin_Mela	SLC16A10
ANXA25	7.05E-34	1.682224	0.987	0.598	1.12E-29	Skin_Mela	ANXA2
KRT146	8.95E-34	0.580159	0.641	0.149	1.43E-29	Skin_Mela	KRT14
STX72	9.12E-34	1.57494	0.538	0.124	1.45E-29	Skin_Mela	STX7
NAT8L	9.97E-34	0.315833	0.064	0.002	1.59E-29	Skin_Mela	NAT8L
LMNA14	2.68E-33	1.619342	0.91	0.366	4.28E-29	Skin_Mela	LMNA
VAT13	5.23E-33	1.530849	0.564	0.138	8.35E-29	Skin_Mela	VAT1
VIM13	5.92E-33	1.61735	0.974	0.494	9.44E-29	Skin_Mela	VIM
TUBB4A	6.55E-33	0.469585	0.09	0.004	1.04E-28	Skin_Mela	TUBB4A
SEPW110	2.06E-32	1.955099	0.846	0.359	3.28E-28	Skin_Mela	SEPW1
BACE28	2.51E-32	1.896675	0.59	0.162	4.01E-28	Skin_Mela	BACE2
ANKRD285	4.93E-32	1.696737	0.5	0.11	7.86E-28	Skin_Mela	ANKRD28
TSPO5	9.18E-32	1.668698	0.91	0.466	1.46E-27	Skin_Mela	TSPO
MYO10	2.56E-31	1.260044	0.359	0.06	4.08E-27	Skin_Mela	MYO10
EID18	4.7E-31	1.695741	0.846	0.358	7.49E-27	Skin_Mela	EID1
ASAH14	5.33E-31	1.93101	0.731	0.278	8.5E-27	Skin_Mela	ASAH1
GPR137B2	5.41E-31	1.120733	0.346	0.056	8.62E-27	Skin_Mela	GPR137B
GAS610	8.47E-31	1.514632	0.462	0.095	1.35E-26	Skin_Mela	GAS6
MLEC6	1.26E-30	1.597375	0.628	0.189	2.02E-26	Skin_Mela	MLEC
HSPB17	1.38E-30	1.297003	1	0.565	2.21E-26	Skin_Mela	HSPB1
LINC00511	1.58E-30	0.894513	0.231	0.026	2.52E-26	Skin_Mela	LINC00511
CTSA3	2.63E-30	1.460781	0.538	0.132	4.2E-26	Skin_Mela	CTSA
PFKM1	4.99E-30	0.976537	0.256	0.032	7.96E-26	Skin_Mela	PFKM
QDPR2	5.01E-29	1.528763	0.436	0.094	8E-25	Skin_Mela	QDPR
MC1R	9.85E-29	0.491402	0.103	0.006	1.57E-24	Skin_Mela	MC1R

RPL417	1.1E-28	1.425744	1	0.862	1.76E-24	Skin_Mela	RPL41
SPTSSA3	1.25E-28	1.666497	0.705	0.245	2E-24	Skin_Mela	SPTSSA
TMEM475	1.33E-28	1.117162	0.333	0.055	2.12E-24	Skin_Mela	TMEM47
TTYH31	1.81E-28	0.9771	0.244	0.031	2.89E-24	Skin_Mela	TTYH3
TAX1BP35	2.08E-28	1.410384	0.423	0.087	3.32E-24	Skin_Mela	TAX1BP3
AP1S26	2.59E-28	1.334533	0.526	0.125	4.13E-24	Skin_Mela	AP1S2
FABP56	2.59E-28	1.36636	0.538	0.135	4.13E-24	Skin_Mela	FABP5
APCDD12	3.47E-28	1.071949	0.282	0.04	5.53E-24	Skin_Mela	APCDD1
MAGED29	4.22E-28	1.471764	0.564	0.155	6.74E-24	Skin_Mela	MAGED2
RUNX34	6.28E-28	0.75801	0.256	0.033	1E-23	Skin_Mela	RUNX3
PLEKHA53	7.9E-28	1.542936	0.41	0.086	1.26E-23	Skin_Mela	PLEKHA5
CD913	1.03E-27	1.584246	0.91	0.453	1.64E-23	Skin_Mela	CD9
COPS83	1.68E-27	1.321495	0.5	0.124	2.67E-23	Skin_Mela	COPS8
GCNT21	2.53E-27	1.020001	0.231	0.029	4.04E-23	Skin_Mela	GCNT2
CISD14	2.75E-27	1.638324	0.513	0.137	4.38E-23	Skin_Mela	CISD1
PSAP4	2.84E-27	1.487057	0.872	0.435	4.52E-23	Skin_Mela	PSAP
PSEN2	5.18E-27	0.941818	0.205	0.023	8.26E-23	Skin_Mela	PSEN2
DST9	6.1E-27	1.240923	0.615	0.184	9.73E-23	Skin_Mela	DST
ABCB4	2.3E-26	0.465356	0.077	0.004	3.66E-22	Skin_Mela	ABCB4
MTHFD1L	2.76E-26	0.912663	0.192	0.021	4.4E-22	Skin_Mela	MTHFD1L
MFGE85	2.9E-26	1.246984	0.449	0.099	4.62E-22	Skin_Mela	MFGE8
SYNGR13	3.2E-26	1.530014	0.513	0.142	5.11E-22	Skin_Mela	SYNGR1
MT2A4	3.6E-26	0.498828	0.859	0.307	5.74E-22	Skin_Mela	MT2A
GYPC4	4.14E-26	1.291043	0.667	0.203	6.6E-22	Skin_Mela	GYPC
C6orf1	4.38E-26	1.258678	0.385	0.077	6.99E-22	Skin_Mela	C6orf1
KIF1A	4.7E-26	0.472783	0.103	0.006	7.49E-22	Skin_Mela	KIF1A
SLC25A49	4.93E-26	1.479931	0.462	0.115	7.86E-22	Skin_Mela	SLC25A4
C7orf55.LU	9.35E-26	0.869939	0.295	0.046	1.49E-21	Skin_Mela	C7orf55.LUC7L2
RPS4Y110	2.03E-25	1.340529	0.756	0.273	3.24E-21	Skin_Mela	RPS4Y1
CD995	2.12E-25	1.573781	0.885	0.492	3.38E-21	Skin_Mela	CD99
EPS154	2.87E-25	1.446863	0.449	0.11	4.58E-21	Skin_Mela	EPS15
NPC25	6.09E-25	1.402208	0.872	0.44	9.72E-21	Skin_Mela	NPC2
CD447	1.35E-24	1.256585	0.833	0.349	2.15E-20	Skin_Mela	CD44
CLEC11A2	1.85E-24	1.101309	0.385	0.08	2.95E-20	Skin_Mela	CLEC11A
LINC00998	1.95E-24	1.102451	0.346	0.068	3.12E-20	Skin_Mela	LINC00998
CTSF4	2.87E-24	1.600913	0.526	0.157	4.58E-20	Skin_Mela	CTSF
ITGB19	3.59E-24	1.602109	0.782	0.357	5.72E-20	Skin_Mela	ITGB1
H3F3A19	6.3E-24	1.2693	0.949	0.479	1.01E-19	Skin_Mela	H3F3A
HERC21	7.74E-24	1.231013	0.346	0.07	1.23E-19	Skin_Mela	HERC2
ENPP24	8.59E-24	0.803764	0.231	0.032	1.37E-19	Skin_Mela	ENPP2
CYSTM17	1.7E-23	1.375057	0.641	0.226	2.71E-19	Skin_Mela	CYSTM1
H2AFZ7	3.03E-23	1.306564	0.872	0.439	4.83E-19	Skin_Mela	H2AFZ
EPDR11	4.17E-23	0.80752	0.154	0.016	6.65E-19	Skin_Mela	EPDR1
AGPAT22	4.95E-23	1.569487	0.462	0.127	7.9E-19	Skin_Mela	AGPAT2
FGFBP22	5.55E-23	0.728784	0.256	0.038	8.86E-19	Skin_Mela	FGFBP2
RAB7A4	7.17E-23	1.617683	0.756	0.372	1.14E-18	Skin_Mela	RAB7A
CTSD4	1.85E-22	1.126663	0.731	0.293	2.96E-18	Skin_Mela	CTSD

YWHAH8	1.87E-22	1.644548	0.59	0.207	2.99E-18	Skin_Mela	YWHAH
FARP2	2.43E-22	0.852653	0.231	0.034	3.88E-18	Skin_Mela	FARP2
MGST314	3.13E-22	1.55298	0.744	0.335	4.99E-18	Skin_Mela	MGST3
ARL25	3.27E-22	1.420464	0.603	0.211	5.22E-18	Skin_Mela	ARL2
SLC3A26	3.52E-22	1.300986	0.59	0.198	5.61E-18	Skin_Mela	SLC3A2
SLC35F1	6.96E-22	0.42676	0.09	0.006	1.11E-17	Skin_Mela	SLC35F1
C3orf703	1.02E-21	0.460242	0.141	0.014	1.62E-17	Skin_Mela	C3orf70
LYST5	1.13E-21	1.560622	0.385	0.094	1.81E-17	Skin_Mela	LYST
MTRNR2L8	1.52E-21	1.294574	0.744	0.286	2.42E-17	Skin_Mela	MTRNR2L8
STMN12	1.64E-21	1.326081	0.59	0.191	2.61E-17	Skin_Mela	STMN1
DPYSL29	1.72E-21	1.160509	0.41	0.099	2.74E-17	Skin_Mela	DPYSL2
RP11.440I1	1.9E-21	0.261801	0.026	0.001	3.03E-17	Skin_Mela	RP11.440I14.2
RAB27A5	2.03E-21	1.210593	0.321	0.065	3.23E-17	Skin_Mela	RAB27A
TIMM133	2.1E-21	1.258326	0.641	0.247	3.35E-17	Skin_Mela	TIMM13
ATP6V1B24	4.13E-21	1.320747	0.372	0.088	6.59E-17	Skin_Mela	ATP6V1B2
ST6GALNA	4.73E-21	0.881451	0.282	0.05	7.54E-17	Skin_Mela	ST6GALNAC2
POLR2L6	8.34E-21	1.371939	0.833	0.473	1.33E-16	Skin_Mela	POLR2L
COL24A1	9.2E-21	0.286473	0.051	0.002	1.47E-16	Skin_Mela	COL24A1
TMEM14C	1.57E-20	1.255289	0.641	0.256	2.5E-16	Skin_Mela	TMEM14C
PTPRS3	1.89E-20	1.223193	0.308	0.062	3.01E-16	Skin_Mela	PTPRS
CPNE85	3.42E-20	0.899796	0.244	0.041	5.45E-16	Skin_Mela	CPNE8
TRPV21	4E-20	0.701423	0.179	0.023	6.37E-16	Skin_Mela	TRPV2
SCARB12	4.09E-20	1.148602	0.244	0.042	6.52E-16	Skin_Mela	SCARB1
CYP27A12	4.57E-20	0.889	0.269	0.05	7.28E-16	Skin_Mela	CYP27A1
DBI8	5.28E-20	1.220095	0.846	0.415	8.42E-16	Skin_Mela	DBI
SPARC7	6.11E-20	0.79703	0.551	0.162	9.74E-16	Skin_Mela	SPARC
C11orf964	7.79E-20	0.636787	0.423	0.104	1.24E-15	Skin_Mela	C11orf96
GSTA44	9.77E-20	0.958106	0.244	0.042	1.56E-15	Skin_Mela	GSTA4
TTYH22	1.04E-19	0.61039	0.128	0.013	1.66E-15	Skin_Mela	TTYH2
UBB4	1.05E-19	1.233541	0.974	0.73	1.67E-15	Skin_Mela	UBB
RENBP1	1.08E-19	0.668656	0.167	0.021	1.72E-15	Skin_Mela	RENBP
QPRT2	1.77E-19	0.804452	0.179	0.024	2.82E-15	Skin_Mela	QPRT
FADS12	2.25E-19	0.751499	0.192	0.028	3.59E-15	Skin_Mela	FADS1
DERA	2.66E-19	1.021462	0.372	0.095	4.25E-15	Skin_Mela	DERA
YWHAE6	3.37E-19	1.385938	0.744	0.398	5.37E-15	Skin_Mela	YWHAE
TTC39	3.38E-19	1.384953	0.603	0.234	5.39E-15	Skin_Mela	TTC3
FTL4	4.33E-19	0.370027	0.987	0.896	6.91E-15	Skin_Mela	FTL
RNF144A	4.36E-19	0.480535	0.128	0.013	6.96E-15	Skin_Mela	RNF144A
HSD17B14	4.89E-19	0.55465	0.154	0.019	7.8E-15	Skin_Mela	HSD17B14
ANXA511	5.13E-19	1.229327	0.731	0.318	8.18E-15	Skin_Mela	ANXA5
FMN11	5.69E-19	0.810362	0.192	0.028	9.07E-15	Skin_Mela	FMN1
LAMTOR51	7.32E-19	1.259592	0.744	0.373	1.17E-14	Skin_Mela	LAMTOR5
LINC00937	8.55E-19	0.557504	0.103	0.009	1.36E-14	Skin_Mela	LINC00937
LAPTM4A7	8.68E-19	1.02192	0.897	0.519	1.38E-14	Skin_Mela	LAPTM4A
ABCA51	1.35E-18	1.268935	0.385	0.104	2.16E-14	Skin_Mela	ABCA5
EIF4A17	1.88E-18	1.233088	0.769	0.353	2.99E-14	Skin_Mela	EIF4A1
CALM210	2.08E-18	1.044553	0.949	0.563	3.31E-14	Skin_Mela	CALM2

NR4A28	2.09E-18	1.159702	0.551	0.185	3.33E-14	Skin_Mela	NR4A2
PDLIM310	2.17E-18	1.256818	0.282	0.058	3.47E-14	Skin_Mela	PDLIM3
TMEM147:	2.26E-18	1.570639	0.59	0.244	3.61E-14	Skin_Mela	TMEM147
SPCS23	3.04E-18	1.093938	0.782	0.407	4.86E-14	Skin_Mela	SPCS2
C1orf1983	3.44E-18	1.131722	0.282	0.059	5.48E-14	Skin_Mela	C1orf198
CLCN71	3.49E-18	0.853271	0.231	0.041	5.56E-14	Skin_Mela	CLCN7
ARF51	3.53E-18	1.228951	0.577	0.222	5.63E-14	Skin_Mela	ARF5
PRDX110	6.61E-18	1.167568	0.833	0.452	1.05E-13	Skin_Mela	PRDX1
SEMA5A5	7.73E-18	0.81585	0.179	0.026	1.23E-13	Skin_Mela	SEMA5A
ZEB26	8.48E-18	1.285227	0.359	0.089	1.35E-13	Skin_Mela	ZEB2
ARHGDIA6	9.47E-18	1.176947	0.551	0.197	1.51E-13	Skin_Mela	ARHGDIA
PEBP110	9.9E-18	1.276055	0.795	0.456	1.58E-13	Skin_Mela	PEBP1
CRNDE5	1.36E-17	1.188947	0.474	0.155	2.16E-13	Skin_Mela	CRNDE
HMGB112	1.5E-17	0.975348	0.923	0.576	2.39E-13	Skin_Mela	HMGB1
TSC22D16	1.53E-17	1.197462	0.641	0.268	2.44E-13	Skin_Mela	TSC22D1
TMEM55A	1.98E-17	0.76436	0.231	0.041	3.16E-13	Skin_Mela	TMEM55A
CELF26	2.15E-17	1.017551	0.449	0.133	3.42E-13	Skin_Mela	CELF2
DGCR6	2.18E-17	0.817601	0.269	0.054	3.48E-13	Skin_Mela	DGCR6
FADD	2.25E-17	0.688896	0.205	0.034	3.59E-13	Skin_Mela	FADD
CAV29	2.82E-17	1.208209	0.41	0.12	4.49E-13	Skin_Mela	CAV2
TPP12	3.13E-17	1.279091	0.359	0.097	5E-13	Skin_Mela	TPP1
CST36	5.71E-17	0.540286	0.949	0.594	9.11E-13	Skin_Mela	CST3
S100A108	6.24E-17	0.963365	0.974	0.676	9.96E-13	Skin_Mela	S100A10
RAB2A1	8.97E-17	1.263717	0.718	0.392	1.43E-12	Skin_Mela	RAB2A
ARL6IP57	1.1E-16	1.24718	0.679	0.31	1.75E-12	Skin_Mela	ARL6IP5
FSTL4	1.27E-16	0.305002	0.077	0.006	2.02E-12	Skin_Mela	FSTL4
CLIC46	1.53E-16	1.17292	0.41	0.126	2.43E-12	Skin_Mela	CLIC4
SOX6	1.68E-16	0.782478	0.192	0.032	2.68E-12	Skin_Mela	SOX6
HEXA2	1.76E-16	1.221122	0.397	0.121	2.81E-12	Skin_Mela	HEXA
FBLN71	1.77E-16	0.514353	0.115	0.012	2.82E-12	Skin_Mela	FBLN7
ATOX17	2.11E-16	1.310752	0.603	0.274	3.37E-12	Skin_Mela	ATOX1
MAN2A2	2.26E-16	0.663081	0.179	0.028	3.61E-12	Skin_Mela	MAN2A2
KIAA14561	3.07E-16	0.537317	0.103	0.01	4.89E-12	Skin_Mela	KIAA1456
RASSF14	3.72E-16	1.106581	0.346	0.092	5.93E-12	Skin_Mela	RASSF1
DNAJC132	5.23E-16	0.726198	0.205	0.037	8.34E-12	Skin_Mela	DNAJC13
PITHD11	7.15E-16	1.124508	0.436	0.146	1.14E-11	Skin_Mela	PITHD1
LDHA8	1.35E-15	1.057891	0.808	0.44	2.15E-11	Skin_Mela	LDHA
VDAC16	1.76E-15	1.183428	0.564	0.242	2.8E-11	Skin_Mela	VDAC1
COX8A4	2.05E-15	1.116888	0.756	0.436	3.27E-11	Skin_Mela	COX8A
S100A137	2.22E-15	1.072181	0.603	0.257	3.54E-11	Skin_Mela	S100A13
RBX19	3.16E-15	1.173049	0.679	0.349	5.04E-11	Skin_Mela	RBX1
NBEAL17	3.23E-15	1.299806	0.705	0.32	5.15E-11	Skin_Mela	NBEAL1
TMTC2	4.68E-15	0.626868	0.167	0.026	7.47E-11	Skin_Mela	TMTC2
TMOD13	5.13E-15	0.475921	0.115	0.013	8.18E-11	Skin_Mela	TMOD1
COX5B4	7.99E-15	0.905182	0.859	0.566	1.27E-10	Skin_Mela	COX5B
ROPN1B	9.01E-15	0.929637	0.295	0.072	1.44E-10	Skin_Mela	ROPN1B
COX6A13	9.32E-15	1.05822	0.821	0.531	1.49E-10	Skin_Mela	COX6A1

AHCYL2	1.17E-14	0.502259	0.141	0.02	1.86E-10	Skin_Mela	AHCYL2
TUBB2A6	1.21E-14	1.137297	0.372	0.113	1.93E-10	Skin_Mela	TUBB2A
HPS4	1.46E-14	1.012895	0.231	0.049	2.33E-10	Skin_Mela	HPS4
AEBP14	1.81E-14	0.679148	0.321	0.08	2.88E-10	Skin_Mela	AEBP1
CPQ3	3.08E-14	0.997164	0.295	0.075	4.91E-10	Skin_Mela	CPQ
TMED211	4.47E-14	1.044432	0.679	0.346	7.13E-10	Skin_Mela	TMED2
LRRN4CL2	5.49E-14	0.532312	0.154	0.024	8.75E-10	Skin_Mela	LRRN4CL
NDUFB26	5.82E-14	0.969655	0.718	0.375	9.29E-10	Skin_Mela	NDUFB2
MIF5	5.84E-14	1.150674	0.769	0.482	9.32E-10	Skin_Mela	MIF
TMEM258!	6.29E-14	1.218793	0.731	0.453	1E-09	Skin_Mela	TMEM258
HSPA1A10	6.78E-14	0.904883	0.692	0.326	1.08E-09	Skin_Mela	HSPA1A
RTKN1	6.96E-14	0.614418	0.154	0.025	1.11E-09	Skin_Mela	RTKN
TMED106	7.54E-14	0.989322	0.667	0.321	1.2E-09	Skin_Mela	TMED10
NFATC26	7.69E-14	0.811035	0.218	0.045	1.23E-09	Skin_Mela	NFATC2
MT1M2	8.53E-14	0.401735	0.295	0.073	1.36E-09	Skin_Mela	MT1M
POLR2F4	9.55E-14	1.228861	0.551	0.253	1.52E-09	Skin_Mela	POLR2F
TMEM14A	1.09E-13	0.984444	0.308	0.085	1.73E-09	Skin_Mela	TMEM14A
PHPT110	1.2E-13	1.177872	0.641	0.325	1.92E-09	Skin_Mela	PHPT1
SPTBN16	1.36E-13	1.03678	0.5	0.198	2.18E-09	Skin_Mela	SPTBN1
LEF1	1.44E-13	0.722024	0.205	0.039	2.3E-09	Skin_Mela	LEF1
COL27A12	1.45E-13	0.615197	0.115	0.015	2.32E-09	Skin_Mela	COL27A1
RPLP19	1.72E-13	0.676758	1	0.915	2.74E-09	Skin_Mela	RPLP1
C5orf151	1.96E-13	0.916756	0.295	0.081	3.13E-09	Skin_Mela	C5orf15
DNAJB97	2.18E-13	1.108623	0.5	0.208	3.48E-09	Skin_Mela	DNAJB9
RHOC8	2.29E-13	1.246406	0.526	0.224	3.66E-09	Skin_Mela	RHOC
CFL12	2.55E-13	0.766717	0.962	0.709	4.06E-09	Skin_Mela	CFL1
SLC35B2	3.23E-13	0.92802	0.269	0.07	5.15E-09	Skin_Mela	SLC35B2
AC010642.	3.25E-13	0.563764	0.192	0.037	5.19E-09	Skin_Mela	AC010642.1
ARMC9	3.48E-13	0.586937	0.141	0.022	5.55E-09	Skin_Mela	ARMC9
COX7A23	3.83E-13	0.790724	0.885	0.571	6.11E-09	Skin_Mela	COX7A2
MARCKS8	4.35E-13	1.055198	0.603	0.284	6.94E-09	Skin_Mela	MARCKS
CD822	5.18E-13	0.829182	0.346	0.109	8.26E-09	Skin_Mela	CD82
CHADL1	5.27E-13	0.543643	0.115	0.015	8.41E-09	Skin_Mela	CHADL
MAP1B6	5.37E-13	0.913877	0.321	0.089	8.57E-09	Skin_Mela	MAP1B
RGS34	5.7E-13	0.790469	0.205	0.043	9.08E-09	Skin_Mela	RGS3
PARVA4	9.87E-13	0.811308	0.244	0.06	1.57E-08	Skin_Mela	PARVA
NR4A36	1E-12	1.014961	0.231	0.054	1.6E-08	Skin_Mela	NR4A3
CBR15	1.2E-12	1.034267	0.359	0.119	1.92E-08	Skin_Mela	CBR1
NINJ16	1.26E-12	1.035823	0.41	0.145	2.01E-08	Skin_Mela	NINJ1
ATP1B37	1.44E-12	1.148984	0.526	0.237	2.29E-08	Skin_Mela	ATP1B3
TIMP23	1.51E-12	0.979338	0.692	0.351	2.41E-08	Skin_Mela	TIMP2
BANF13	1.8E-12	1.037144	0.526	0.233	2.87E-08	Skin_Mela	BANF1
SGCE6	1.96E-12	0.828284	0.192	0.04	3.12E-08	Skin_Mela	SGCE
YBX17	1.98E-12	0.687051	0.872	0.589	3.16E-08	Skin_Mela	YBX1
IFT813	1.99E-12	0.736201	0.192	0.04	3.17E-08	Skin_Mela	IFT81
UQCRQ7	2.16E-12	1.000389	0.756	0.5	3.44E-08	Skin_Mela	UQCRQ
MORF4L16	2.24E-12	1.073703	0.744	0.459	3.58E-08	Skin_Mela	MORF4L1

TSC22D33	2.38E-12	0.987574	0.603	0.282	3.8E-08	Skin_Mela	TSC22D3
SKP16	2.51E-12	0.811173	0.859	0.571	4E-08	Skin_Mela	SKP1
PDLIM49	2.54E-12	1.065213	0.333	0.105	4.04E-08	Skin_Mela	PDLIM4
MTURN4	2.7E-12	0.852139	0.282	0.08	4.3E-08	Skin_Mela	MTURN
SNX36	3.02E-12	1.0403	0.731	0.416	4.81E-08	Skin_Mela	SNX3
IFFO1	3.28E-12	0.517619	0.128	0.02	5.23E-08	Skin_Mela	IFFO1
PIGT4	3.6E-12	0.98506	0.423	0.164	5.74E-08	Skin_Mela	PIGT
NLGN1	4.54E-12	0.477655	0.09	0.01	7.25E-08	Skin_Mela	NLGN1
CSPG42	5E-12	0.289479	0.064	0.006	7.98E-08	Skin_Mela	CSPG4
FASTK	5.04E-12	0.908447	0.333	0.108	8.04E-08	Skin_Mela	FASTK
ARL36	5.06E-12	0.942357	0.346	0.115	8.06E-08	Skin_Mela	ARL3
ELF16	5.72E-12	0.901988	0.449	0.178	9.12E-08	Skin_Mela	ELF1
HNRNPH25	6.38E-12	0.978787	0.282	0.082	1.02E-07	Skin_Mela	HNRNPH2
PTMA8	6.86E-12	0.66603	1	0.819	1.09E-07	Skin_Mela	PTMA
ZMAT3	6.95E-12	0.869002	0.244	0.063	1.11E-07	Skin_Mela	ZMAT3
RBM310	7.01E-12	1.002493	0.718	0.44	1.12E-07	Skin_Mela	RBM3
S100A18	7.13E-12	1.29593	0.333	0.104	1.14E-07	Skin_Mela	S100A1
FN14	7.45E-12	0.671052	0.333	0.099	1.19E-07	Skin_Mela	FN1
PAQR81	7.54E-12	0.420584	0.103	0.013	1.2E-07	Skin_Mela	PAQR8
PTPRM3	7.84E-12	0.679378	0.154	0.028	1.25E-07	Skin_Mela	PTPRM
BBC32	9.42E-12	0.854302	0.256	0.069	1.5E-07	Skin_Mela	BBC3
ETS18	1.01E-11	0.653332	0.282	0.077	1.61E-07	Skin_Mela	ETS1
SOD18	1.02E-11	0.940138	0.731	0.415	1.63E-07	Skin_Mela	SOD1
TUBA1B11	1.17E-11	1.064305	0.679	0.354	1.86E-07	Skin_Mela	TUBA1B
WDR83OS4	1.21E-11	0.936061	0.667	0.368	1.93E-07	Skin_Mela	WDR83OS
NDUFB711	1.33E-11	1.004154	0.654	0.373	2.12E-07	Skin_Mela	NDUFB7
CABLES1	1.41E-11	0.469864	0.128	0.021	2.26E-07	Skin_Mela	CABLES1
SLC27A34	1.46E-11	0.638048	0.154	0.028	2.33E-07	Skin_Mela	SLC27A3
CTD.2325P	1.52E-11	0.293724	0.051	0.004	2.42E-07	Skin_Mela	CTD.2325P2.4
TPI15	1.55E-11	0.862754	0.795	0.535	2.47E-07	Skin_Mela	TPI1
PCMTD21	1.62E-11	0.806795	0.244	0.065	2.58E-07	Skin_Mela	PCMTD2
SGCD2	1.62E-11	0.628065	0.128	0.021	2.58E-07	Skin_Mela	SGCD
CDC428	1.66E-11	0.963451	0.756	0.47	2.65E-07	Skin_Mela	CDC42
ACOT72	1.85E-11	0.662336	0.205	0.048	2.94E-07	Skin_Mela	ACOT7
SMIM106	1.89E-11	0.413759	0.128	0.021	3.01E-07	Skin_Mela	SMIM10
RETSAT	1.9E-11	0.697581	0.179	0.038	3.03E-07	Skin_Mela	RETSAT
NAP1L16	2.15E-11	0.955952	0.705	0.402	3.43E-07	Skin_Mela	NAP1L1
TUBA1A7	2.2E-11	0.7325	0.551	0.235	3.5E-07	Skin_Mela	TUBA1A
EDARADD	2.27E-11	0.266687	0.077	0.008	3.62E-07	Skin_Mela	EDARADD
SNX25	2.4E-11	0.571204	0.128	0.021	3.83E-07	Skin_Mela	SNX25
SDF44	2.59E-11	0.974483	0.474	0.209	4.13E-07	Skin_Mela	SDF4
CYTH33	2.6E-11	0.567161	0.154	0.029	4.14E-07	Skin_Mela	CYTH3
ENDOD15	2.69E-11	0.703359	0.167	0.034	4.29E-07	Skin_Mela	ENDOD1
LAMTOR43	3.01E-11	1.013506	0.654	0.389	4.8E-07	Skin_Mela	LAMTOR4
RAB5C3	3.01E-11	1.046581	0.526	0.256	4.81E-07	Skin_Mela	RAB5C
PDE4DIP2	3.16E-11	0.976629	0.244	0.067	5.03E-07	Skin_Mela	PDE4DIP
SNRPD23	3.17E-11	0.887633	0.744	0.433	5.06E-07	Skin_Mela	SNRPD2

CYP2U11	3.46E-11	0.423945	0.103	0.014	5.52E-07	Skin_Mela	CYP2U1
HAGHL	3.66E-11	0.646995	0.128	0.021	5.84E-07	Skin_Mela	HAGHL
NDUFA15	3.81E-11	1.0308	0.692	0.412	6.08E-07	Skin_Mela	NDUFA1
PRKD33	3.96E-11	0.796345	0.269	0.079	6.32E-07	Skin_Mela	PRKD3
NDUFB41	3.99E-11	1.013674	0.718	0.446	6.36E-07	Skin_Mela	NDUFB4
CPEB21	4.02E-11	0.936441	0.269	0.079	6.41E-07	Skin_Mela	CPEB2
FKBP1B	4.1E-11	0.557236	0.167	0.034	6.54E-07	Skin_Mela	FKBP1B
BEST16	4.26E-11	0.598652	0.192	0.043	6.8E-07	Skin_Mela	BEST1
S100A68	4.68E-11	0.647003	0.949	0.713	7.47E-07	Skin_Mela	S100A6
TM7SF3	4.94E-11	0.942419	0.308	0.102	7.88E-07	Skin_Mela	TM7SF3
ATP7A1	4.96E-11	0.660889	0.141	0.026	7.91E-07	Skin_Mela	ATP7A
TCEAL83	5.95E-11	1.031583	0.41	0.167	9.48E-07	Skin_Mela	TCEAL8
PCMT14	6.27E-11	0.834489	0.487	0.217	1E-06	Skin_Mela	PCMT1
ELMSAN17	6.5E-11	1.018992	0.269	0.079	1.04E-06	Skin_Mela	ELMSAN1
MSC2	6.88E-11	0.58384	0.128	0.022	1.1E-06	Skin_Mela	MSC
ATP6AP25	7.07E-11	0.977656	0.513	0.239	1.13E-06	Skin_Mela	ATP6AP2
NDUFS71	7.13E-11	1.101204	0.538	0.273	1.14E-06	Skin_Mela	NDUFS7
KLF69	7.42E-11	0.814361	0.718	0.402	1.18E-06	Skin_Mela	KLF6
NAV13	7.53E-11	0.554133	0.154	0.03	1.2E-06	Skin_Mela	NAV1
BPHL	7.63E-11	0.749291	0.154	0.03	1.22E-06	Skin_Mela	BPHL
PCDH72	7.73E-11	0.478303	0.128	0.022	1.23E-06	Skin_Mela	PCDH7
RPL37A7	7.91E-11	0.573189	1	0.856	1.26E-06	Skin_Mela	RPL37A
COX6B1	8.15E-11	0.76915	0.833	0.607	1.3E-06	Skin_Mela	COX6B1
MRPL414	9.07E-11	1.002548	0.577	0.31	1.45E-06	Skin_Mela	MRPL41
CTTN5	1.15E-10	0.979218	0.462	0.208	1.84E-06	Skin_Mela	CTTN
DMKN4	1.22E-10	0.289976	0.41	0.157	1.94E-06	Skin_Mela	DMKN
TMED92	1.32E-10	1.028844	0.551	0.292	2.11E-06	Skin_Mela	TMED9
SLC39A65	1.44E-10	1.04593	0.295	0.099	2.29E-06	Skin_Mela	SLC39A6
CORO1C6	1.5E-10	1.046334	0.333	0.118	2.4E-06	Skin_Mela	CORO1C
RABAC111	1.51E-10	0.794037	0.705	0.405	2.41E-06	Skin_Mela	RABAC1
SMPD11	1.67E-10	0.889436	0.282	0.089	2.67E-06	Skin_Mela	SMPD1
PAN37	1.68E-10	0.874369	0.269	0.081	2.68E-06	Skin_Mela	PAN3
ITM2C4	1.79E-10	0.836328	0.346	0.121	2.85E-06	Skin_Mela	ITM2C
ARSB1	1.79E-10	0.381912	0.09	0.012	2.86E-06	Skin_Mela	ARSB
SET10	1.8E-10	1.043281	0.59	0.311	2.87E-06	Skin_Mela	SET
LRPAP19	2.15E-10	0.80546	0.551	0.27	3.44E-06	Skin_Mela	LRPAP1
JOSD22	2.19E-10	0.808645	0.333	0.118	3.5E-06	Skin_Mela	JOSD2
GNG23	2.25E-10	0.600199	0.256	0.07	3.59E-06	Skin_Mela	GNG2
EVA1A1	2.61E-10	0.354793	0.09	0.012	4.16E-06	Skin_Mela	EVA1A
RP4.594I1(	2.65E-10	0.311693	0.077	0.009	4.23E-06	Skin_Mela	RP4.594I10.3
HRAS2	2.78E-10	0.811005	0.282	0.09	4.44E-06	Skin_Mela	HRAS
ASB92	2.89E-10	0.490928	0.115	0.019	4.6E-06	Skin_Mela	ASB9
CMPK16	2.99E-10	0.913399	0.564	0.301	4.77E-06	Skin_Mela	CMPK1
ANXA44	3.28E-10	1.071452	0.359	0.138	5.23E-06	Skin_Mela	ANXA4
ANKRD443	4.26E-10	0.673826	0.167	0.036	6.8E-06	Skin_Mela	ANKRD44
RPL3916	4.32E-10	0.593695	0.974	0.677	6.88E-06	Skin_Mela	RPL39
TMEM595	4.66E-10	0.675836	0.859	0.61	7.43E-06	Skin_Mela	TMEM59

NDUFA411	4.9E-10	0.844609	0.808	0.538	7.82E-06	Skin_Mela	NDUFA4
ST3GAL4	5.24E-10	0.913536	0.372	0.145	8.36E-06	Skin_Mela	ST3GAL4
HOXB72	5.37E-10	0.522449	0.128	0.023	8.56E-06	Skin_Mela	HOXB7
TOMM78	5.7E-10	0.656681	0.859	0.582	9.09E-06	Skin_Mela	TOMM7
MXI1	5.79E-10	1.044126	0.372	0.15	9.23E-06	Skin_Mela	MXI1
SCD2	6.27E-10	0.916217	0.256	0.08	1E-05	Skin_Mela	SCD
DCHS12	6.52E-10	0.312211	0.09	0.012	1.04E-05	Skin_Mela	DCHS1
SLC29A14	6.68E-10	0.668812	0.192	0.047	1.06E-05	Skin_Mela	SLC29A1
MT1E2	6.77E-10	0.311274	0.359	0.129	1.08E-05	Skin_Mela	MT1E
YWHAQ6	6.79E-10	1.066428	0.603	0.365	1.08E-05	Skin_Mela	YWHAQ
NPHP4	7.89E-10	0.279567	0.077	0.01	1.26E-05	Skin_Mela	NPHP4
AHNAK11	8.72E-10	0.873541	0.667	0.386	1.39E-05	Skin_Mela	AHNAK
GSTM41	1.01E-09	0.582196	0.167	0.038	1.6E-05	Skin_Mela	GSTM4
ARL6IP42	1.02E-09	0.839977	0.692	0.422	1.62E-05	Skin_Mela	ARL6IP4
ENAH8	1.05E-09	0.928269	0.321	0.119	1.68E-05	Skin_Mela	ENAH
SOX45	1.07E-09	0.873637	0.538	0.259	1.7E-05	Skin_Mela	SOX4
CIRBP8	1.08E-09	0.838623	0.795	0.546	1.72E-05	Skin_Mela	CIRBP
NR4A16	1.13E-09	1.039126	0.526	0.257	1.8E-05	Skin_Mela	NR4A1
CAMTA15	1.17E-09	0.944319	0.59	0.334	1.87E-05	Skin_Mela	CAMTA1
FXYD39	1.21E-09	0.708898	0.526	0.233	1.93E-05	Skin_Mela	FXYD3
C5orf245	1.39E-09	0.95112	0.308	0.112	2.21E-05	Skin_Mela	C5orf24
CNBP5	1.4E-09	0.669747	0.718	0.427	2.23E-05	Skin_Mela	CNBP
CYB5R37	1.41E-09	1.090707	0.462	0.222	2.25E-05	Skin_Mela	CYB5R3
RAC17	1.44E-09	0.698467	0.846	0.548	2.29E-05	Skin_Mela	RAC1
TIMM8B	1.52E-09	0.972997	0.462	0.226	2.42E-05	Skin_Mela	TIMM8B
SGIP13	1.59E-09	0.293946	0.064	0.007	2.53E-05	Skin_Mela	SGIP1
ST3GAL52	1.63E-09	0.509033	0.141	0.029	2.6E-05	Skin_Mela	ST3GAL5
DKK37	1.66E-09	0.88243	0.179	0.044	2.64E-05	Skin_Mela	DKK3
MRPL234	1.67E-09	0.817522	0.462	0.212	2.66E-05	Skin_Mela	MRPL23
SLC38A28	1.74E-09	0.755554	0.513	0.242	2.77E-05	Skin_Mela	SLC38A2
GAPDH9	1.87E-09	0.661028	0.936	0.764	2.97E-05	Skin_Mela	GAPDH
OST46	1.87E-09	0.800645	0.782	0.501	2.99E-05	Skin_Mela	OST4
ATP5I5	1.98E-09	1.068047	0.603	0.373	3.15E-05	Skin_Mela	ATP5I
COX7B10	2.01E-09	0.910479	0.641	0.409	3.2E-05	Skin_Mela	COX7B
SFXN32	2.01E-09	0.57964	0.179	0.044	3.21E-05	Skin_Mela	SFXN3
TRMT1127	2.19E-09	0.800494	0.641	0.374	3.5E-05	Skin_Mela	TRMT112
MAMSTR	2.25E-09	0.275058	0.051	0.005	3.59E-05	Skin_Mela	MAMSTR
PTTG13	2.6E-09	0.561125	0.269	0.084	4.14E-05	Skin_Mela	PTTG1
HES6	2.62E-09	0.517569	0.154	0.034	4.17E-05	Skin_Mela	HES6
CHPF6	2.62E-09	0.913535	0.256	0.083	4.18E-05	Skin_Mela	CHPF
CTNNAL15	2.78E-09	0.770012	0.269	0.09	4.44E-05	Skin_Mela	CTNNAL1
MATR37	2.81E-09	0.789154	0.321	0.116	4.48E-05	Skin_Mela	MATR3
SLC2A37	2.86E-09	0.445042	0.372	0.13	4.57E-05	Skin_Mela	SLC2A3
CALU6	2.95E-09	0.964554	0.397	0.176	4.71E-05	Skin_Mela	CALU
GRN5	3.01E-09	0.987878	0.551	0.312	4.8E-05	Skin_Mela	GRN
MTPN7	3.24E-09	0.893437	0.372	0.15	5.17E-05	Skin_Mela	MTPN
SLC2A13	3.29E-09	0.582835	0.115	0.021	5.24E-05	Skin_Mela	SLC2A13

SYDE2	3.31E-09	0.803058	0.103	0.017	5.28E-05	Skin_Mela	SYDE2
ARPP192	3.36E-09	0.683882	0.462	0.218	5.35E-05	Skin_Mela	ARPP19
OSBPL9	3.36E-09	1.049592	0.282	0.099	5.36E-05	Skin_Mela	OSBPL9
KIF3A	3.41E-09	0.747974	0.192	0.051	5.44E-05	Skin_Mela	KIF3A
RAB4B	3.57E-09	0.640012	0.179	0.044	5.69E-05	Skin_Mela	RAB4B
HNRNPA07	3.59E-09	0.772384	0.615	0.335	5.72E-05	Skin_Mela	HNRNPA0
GNAL2	3.68E-09	0.407111	0.103	0.017	5.86E-05	Skin_Mela	GNAL
YWHAZ4	3.82E-09	0.70796	0.795	0.51	6.09E-05	Skin_Mela	YWHAZ
ARMCX15	3.84E-09	0.803503	0.205	0.056	6.12E-05	Skin_Mela	ARMCX1
TRAPPC6A	4.01E-09	0.869349	0.359	0.143	6.4E-05	Skin_Mela	TRAPPC6A
PCNP7	4.04E-09	0.916466	0.5	0.26	6.45E-05	Skin_Mela	PCNP
AGAP2.AS1	4.05E-09	0.367202	0.09	0.013	6.46E-05	Skin_Mela	AGAP2.AS1
PHACTR13	4.2E-09	0.770132	0.154	0.035	6.7E-05	Skin_Mela	PHACTR1
TRIM23	4.88E-09	0.974395	0.295	0.109	7.79E-05	Skin_Mela	TRIM2
RAD23A3	5.37E-09	0.923747	0.436	0.206	8.57E-05	Skin_Mela	RAD23A
ACTB5	5.61E-09	0.446967	1	0.872	8.95E-05	Skin_Mela	ACTB
ATP5G311	5.81E-09	0.74118	0.628	0.362	9.27E-05	Skin_Mela	ATP5G3
IGSF83	6.07E-09	0.724691	0.205	0.057	9.68E-05	Skin_Mela	IGSF8
TMEM138	6.08E-09	0.716766	0.179	0.046	9.69E-05	Skin_Mela	TMEM138
FKBP1A6	7.38E-09	0.743721	0.603	0.338	0.000118	Skin_Mela	FKBP1A
SIGIRR	7.43E-09	0.75447	0.346	0.134	0.000118	Skin_Mela	SIGIRR
REXO22	7.74E-09	1.158224	0.474	0.259	0.000123	Skin_Mela	REXO2
CNIH15	7.9E-09	0.751508	0.333	0.13	0.000126	Skin_Mela	CNIH1
ATP6V0D1	1.02E-08	0.825851	0.423	0.203	0.000162	Skin_Mela	ATP6V0D1
WDR63	1.07E-08	0.492839	0.064	0.008	0.00017	Skin_Mela	WDR63
ZFYVE217	1.14E-08	1.043903	0.346	0.149	0.000182	Skin_Mela	ZFYVE21
FBXO71	1.15E-08	0.779213	0.372	0.158	0.000183	Skin_Mela	FBXO7
COX174	1.25E-08	1.139786	0.487	0.276	0.000199	Skin_Mela	COX17
KRT101	1.27E-08	0.312718	0.679	0.414	0.000202	Skin_Mela	KRT10
BCAP311	1.28E-08	0.702458	0.538	0.289	0.000205	Skin_Mela	BCAP31
TCF255	1.3E-08	1.08437	0.513	0.294	0.000207	Skin_Mela	TCF25
TBCB4	1.39E-08	0.81317	0.436	0.202	0.000222	Skin_Mela	TBCB
TGFB1I16	1.41E-08	0.69353	0.192	0.052	0.000225	Skin_Mela	TGFB1I1
CAPG4	1.48E-08	0.943098	0.385	0.167	0.000237	Skin_Mela	CAPG
CLU6	1.58E-08	0.992257	0.436	0.2	0.000252	Skin_Mela	CLU
TCEA21	1.61E-08	0.697847	0.269	0.093	0.000256	Skin_Mela	TCEA2
MZT11	1.63E-08	0.762524	0.231	0.073	0.00026	Skin_Mela	MZT1
WDR132	1.66E-08	0.818552	0.295	0.111	0.000265	Skin_Mela	WDR13
UBA24	1.68E-08	0.863319	0.308	0.116	0.000268	Skin_Mela	UBA2
RHOA6	1.7E-08	0.818275	0.705	0.474	0.000271	Skin_Mela	RHOA
SRP96	1.77E-08	0.93231	0.564	0.342	0.000282	Skin_Mela	SRP9
FADS35	1.79E-08	0.628715	0.167	0.042	0.000286	Skin_Mela	FADS3
C11orf585	1.79E-08	0.765357	0.615	0.368	0.000286	Skin_Mela	C11orf58
GADD45B5	1.86E-08	0.664928	0.667	0.377	0.000297	Skin_Mela	GADD45B
CCDC802	1.93E-08	0.547251	0.385	0.15	0.000308	Skin_Mela	CCDC80
BEX44	1.94E-08	1.016429	0.372	0.17	0.000309	Skin_Mela	BEX4
GLIPR26	1.98E-08	0.819217	0.256	0.085	0.000315	Skin_Mela	GLIPR2

LNP1	2.25E-08	0.424968	0.103	0.018	0.000359	Skin_Mela	LNP1
PEPD4	2.35E-08	0.670439	0.282	0.102	0.000376	Skin_Mela	PEPD
IFITM37	2.42E-08	0.316296	0.641	0.311	0.000386	Skin_Mela	IFITM3
DPP76	2.83E-08	0.851724	0.5	0.259	0.000451	Skin_Mela	DPP7
SPATS2L3	2.84E-08	0.981714	0.436	0.224	0.000453	Skin_Mela	SPATS2L
HMGN39	2.96E-08	0.948307	0.513	0.279	0.000473	Skin_Mela	HMGN3
TCEAL46	2.97E-08	0.716743	0.372	0.16	0.000473	Skin_Mela	TCEAL4
RHEB7	3.18E-08	0.857324	0.577	0.338	0.000507	Skin_Mela	RHEB
RP11.1391	3.4E-08	0.336924	0.064	0.008	0.000542	Skin_Mela	RP11.1391J7.1
FAR21	3.5E-08	0.518584	0.09	0.015	0.000558	Skin_Mela	FAR2
MBP3	3.52E-08	0.803293	0.359	0.154	0.000561	Skin_Mela	MBP
FAM177A1	3.55E-08	0.897365	0.436	0.216	0.000566	Skin_Mela	FAM177A1
COX6C8	3.56E-08	0.877439	0.731	0.542	0.000567	Skin_Mela	COX6C
CTNNB18	4.09E-08	1.253898	0.372	0.172	0.000652	Skin_Mela	CTNNB1
PSMD84	4.13E-08	0.846824	0.564	0.332	0.000659	Skin_Mela	PSMD8
PRSS237	4.22E-08	0.848903	0.269	0.095	0.000673	Skin_Mela	PRSS23
ARPC54	4.43E-08	0.881332	0.628	0.413	0.000706	Skin_Mela	ARPC5
GTF2F21	4.5E-08	0.902241	0.269	0.1	0.000717	Skin_Mela	GTF2F2
TAPBP7	4.54E-08	0.834725	0.372	0.164	0.000724	Skin_Mela	TAPBP
SLIRP5	4.65E-08	0.921033	0.474	0.259	0.000741	Skin_Mela	SLIRP
P2RX72	4.85E-08	0.30404	0.077	0.011	0.000773	Skin_Mela	P2RX7
CYCS7	5.01E-08	0.629611	0.577	0.342	0.0008	Skin_Mela	CYCS
ABL24	5.1E-08	0.911217	0.256	0.09	0.000813	Skin_Mela	ABL2
ODF2L2	5.16E-08	0.732844	0.295	0.113	0.000823	Skin_Mela	ODF2L
SLC4A2	5.23E-08	0.426287	0.141	0.033	0.000834	Skin_Mela	SLC4A2
SDC33	6.03E-08	0.57967	0.115	0.024	0.000961	Skin_Mela	SDC3
TMEM985	6.21E-08	0.69977	0.231	0.076	0.000991	Skin_Mela	TMEM98
FAM126A6	6.22E-08	0.577591	0.205	0.062	0.000991	Skin_Mela	FAM126A
GPAA16	6.89E-08	0.852093	0.333	0.143	0.001099	Skin_Mela	GPAA1
IFI272	8.35E-08	1.111609	0.423	0.199	0.001332	Skin_Mela	IFI27
TRAPPC11	8.6E-08	0.745717	0.462	0.237	0.001371	Skin_Mela	TRAPPC1
SLC43A36	9.26E-08	0.468024	0.141	0.033	0.001477	Skin_Mela	SLC43A3
ANKRD106	9.37E-08	0.641622	0.295	0.113	0.001495	Skin_Mela	ANKRD10
FAM89A2	9.61E-08	0.489566	0.103	0.02	0.001533	Skin_Mela	FAM89A
EIF3E9	9.63E-08	0.540218	0.731	0.449	0.001536	Skin_Mela	EIF3E
HES42	1.03E-07	0.616844	0.423	0.196	0.001638	Skin_Mela	HES4
ERH7	1.12E-07	0.889775	0.526	0.312	0.001794	Skin_Mela	ERH
DSTN8	1.19E-07	0.586986	0.808	0.563	0.001896	Skin_Mela	DSTN
HSPE17	1.22E-07	0.532661	0.628	0.373	0.001943	Skin_Mela	HSPE1
MEF2C9	1.27E-07	0.573391	0.244	0.081	0.002032	Skin_Mela	MEF2C
ATP10A	1.3E-07	0.364492	0.09	0.016	0.002073	Skin_Mela	ATP10A
PRMT22	1.32E-07	0.75085	0.359	0.158	0.002108	Skin_Mela	PRMT2
DLGAP1	1.5E-07	0.373738	0.077	0.012	0.002392	Skin_Mela	DLGAP1
CC2D2A2	1.64E-07	0.267309	0.09	0.016	0.002614	Skin_Mela	CC2D2A
RP11.345J4	1.69E-07	0.480807	0.128	0.029	0.0027	Skin_Mela	RP11.345J4.5
CRIP27	2.02E-07	0.686065	0.423	0.205	0.003224	Skin_Mela	CRIP2
EIF5A10	2.04E-07	1.013897	0.474	0.279	0.003254	Skin_Mela	EIF5A

ITPK11	2.21E-07	0.481837	0.128	0.03	0.003531	Skin_Mela	ITPK1
DAD110	2.42E-07	0.796205	0.603	0.399	0.003853	Skin_Mela	DAD1
UBL5	2.45E-07	0.573361	0.821	0.569	0.003915	Skin_Mela	UBL5
RCN22	2.46E-07	0.747323	0.346	0.155	0.00393	Skin_Mela	RCN2
ATP5E5	2.53E-07	0.671619	0.859	0.684	0.004039	Skin_Mela	ATP5E
CDH17	2.59E-07	0.701324	0.218	0.075	0.004125	Skin_Mela	CDH1
SNX301	2.67E-07	0.451998	0.115	0.025	0.004266	Skin_Mela	SNX30
USMG54	2.87E-07	0.852147	0.628	0.437	0.00458	Skin_Mela	USMG5
FRY6	2.89E-07	0.364013	0.103	0.021	0.004601	Skin_Mela	FRY
HSPA88	2.96E-07	0.599244	0.731	0.511	0.004718	Skin_Mela	HSPA8
HNRNPA2E	3.24E-07	0.553874	0.744	0.492	0.005166	Skin_Mela	HNRNPA2B1
PDIA35	3.43E-07	0.778777	0.538	0.323	0.005465	Skin_Mela	PDIA3
LGALS32	3.62E-07	0.254694	0.833	0.571	0.005777	Skin_Mela	LGALS3
FNTA	3.76E-07	0.681011	0.333	0.149	0.005997	Skin_Mela	FNTA
RP11.138A	4.12E-07	0.660719	0.167	0.047	0.00657	Skin_Mela	RP11.138A9.2
EMC72	4.16E-07	0.717171	0.397	0.199	0.006638	Skin_Mela	EMC7
SNHG72	4.22E-07	0.786082	0.449	0.223	0.006727	Skin_Mela	SNHG7
TBCA5	4.23E-07	0.770858	0.577	0.351	0.006744	Skin_Mela	TBCA
FAM96B2	4.26E-07	0.807861	0.462	0.259	0.006797	Skin_Mela	FAM96B
BCL2A12	4.37E-07	0.423296	0.141	0.035	0.006968	Skin_Mela	BCL2A1
ZBTB105	4.4E-07	0.828285	0.218	0.076	0.007025	Skin_Mela	ZBTB10
PSMA76	4.47E-07	0.720107	0.692	0.464	0.007128	Skin_Mela	PSMA7
SLC25A313	4.68E-07	0.662623	0.654	0.456	0.007458	Skin_Mela	SLC25A3
FCGRT7	4.7E-07	0.561476	0.513	0.278	0.007493	Skin_Mela	FCGRT
COPS62	4.91E-07	0.936532	0.397	0.211	0.007828	Skin_Mela	COPS6
MATN25	4.93E-07	0.582984	0.154	0.042	0.007856	Skin_Mela	MATN2
RNPS14	5.04E-07	0.592602	0.41	0.199	0.008031	Skin_Mela	RNPS1
UBA6.AS1	5.32E-07	0.268496	0.077	0.013	0.008486	Skin_Mela	UBA6.AS1
UBXN2A1	5.47E-07	0.479496	0.192	0.061	0.008723	Skin_Mela	UBXN2A
ANAPC132	5.76E-07	0.8827	0.308	0.136	0.009191	Skin_Mela	ANAPC13
CD15113	6E-07	0.808634	0.423	0.221	0.009565	Skin_Mela	CD151
KLHL424	6.07E-07	0.484114	0.141	0.037	0.009679	Skin_Mela	KLHL42
UBE2E33	6.12E-07	0.908691	0.359	0.174	0.009761	Skin_Mela	UBE2E3
SPRED15	6.23E-07	0.698568	0.154	0.043	0.009929	Skin_Mela	SPRED1
PPIA5	6.29E-07	0.621078	0.769	0.59	0.010029	Skin_Mela	PPIA
TMEM168	6.48E-07	0.492619	0.141	0.037	0.010332	Skin_Mela	TMEM168
APOBEC3C	6.52E-07	0.593245	0.179	0.054	0.010392	Skin_Mela	APOBEC3C
APP8	6.69E-07	0.812354	0.449	0.244	0.010667	Skin_Mela	APP
HIGD1A1	6.7E-07	0.871658	0.423	0.232	0.010681	Skin_Mela	HIGD1A
PFDN13	6.89E-07	0.700127	0.282	0.116	0.010996	Skin_Mela	PFDN1
CHCHD24	6.96E-07	0.614846	0.808	0.621	0.011101	Skin_Mela	CHCHD2
EMX23	7.04E-07	0.453879	0.128	0.031	0.011235	Skin_Mela	EMX2
AKAP125	7.25E-07	0.796265	0.218	0.075	0.011569	Skin_Mela	AKAP12
PSMB64	7.38E-07	0.684398	0.538	0.322	0.011767	Skin_Mela	PSMB6
SHFM13	7.46E-07	0.863863	0.628	0.431	0.011903	Skin_Mela	SHFM1
RAN9	7.47E-07	0.661376	0.59	0.367	0.011913	Skin_Mela	RAN
GAS73	7.83E-07	0.465077	0.167	0.048	0.012486	Skin_Mela	GAS7

ARMCX36	8E-07	0.722205	0.321	0.146	0.012755	Skin_Mela	ARMCX3
PTX31	8.13E-07	0.420271	0.051	0.006	0.012967	Skin_Mela	PTX3
SLC35A5	8.21E-07	0.517355	0.154	0.043	0.013091	Skin_Mela	SLC35A5
APOLD15	8.28E-07	0.360184	0.128	0.031	0.013208	Skin_Mela	APOLD1
SRP147	8.31E-07	0.579915	0.859	0.701	0.013249	Skin_Mela	SRP14
GSTO15	8.72E-07	0.750222	0.41	0.213	0.013907	Skin_Mela	GSTO1
PKIB2	8.75E-07	0.730117	0.179	0.057	0.013953	Skin_Mela	PKIB
PSMB31	8.84E-07	0.546343	0.564	0.345	0.014102	Skin_Mela	PSMB3
TMA74	9.18E-07	0.712661	0.795	0.661	0.014643	Skin_Mela	TMA7
CCDC1077	9.32E-07	0.644068	0.372	0.176	0.014867	Skin_Mela	CCDC107
IAH13	9.69E-07	0.816841	0.346	0.161	0.015459	Skin_Mela	IAH1
ATP6V0A1	9.73E-07	0.740589	0.179	0.057	0.01552	Skin_Mela	ATP6V0A1
TMX23	9.82E-07	0.642704	0.256	0.102	0.015665	Skin_Mela	TMX2
CBX68	9.91E-07	0.713637	0.231	0.084	0.015811	Skin_Mela	CBX6
SPAG71	1.01E-06	0.824164	0.359	0.176	0.016109	Skin_Mela	SPAG7
DNAJC6	1.01E-06	0.285892	0.038	0.004	0.016163	Skin_Mela	DNAJC6
ALDH7A13	1.02E-06	0.766814	0.192	0.065	0.016331	Skin_Mela	ALDH7A1
NAV22	1.08E-06	0.379436	0.09	0.018	0.017244	Skin_Mela	NAV2
PTMS12	1.13E-06	0.790736	0.449	0.245	0.018094	Skin_Mela	PTMS
FAM69B1	1.17E-06	0.292881	0.077	0.014	0.01862	Skin_Mela	FAM69B
VPS41	1.19E-06	0.59937	0.167	0.051	0.01891	Skin_Mela	VPS41
SERINC17	1.28E-06	0.734185	0.385	0.197	0.020437	Skin_Mela	SERINC1
HSP90AB1	1.31E-06	0.624629	0.756	0.57	0.020864	Skin_Mela	HSP90AB1
TCEB2	1.31E-06	0.391929	0.846	0.644	0.020866	Skin_Mela	TCEB2
PARK711	1.31E-06	0.782389	0.564	0.356	0.020888	Skin_Mela	PARK7
NUDT141	1.32E-06	0.68875	0.218	0.08	0.021082	Skin_Mela	NUDT14
ST8SIA11	1.34E-06	0.345422	0.077	0.014	0.021357	Skin_Mela	ST8SIA1
TSN	1.34E-06	0.778636	0.269	0.113	0.021387	Skin_Mela	TSN
DRAM21	1.37E-06	0.6858	0.295	0.128	0.021928	Skin_Mela	DRAM2
C3orf14	1.39E-06	0.750884	0.192	0.066	0.022102	Skin_Mela	C3orf14
PFDN56	1.4E-06	0.402471	0.923	0.727	0.02228	Skin_Mela	PFDN5
FKBP95	1.4E-06	0.7032	0.218	0.078	0.022367	Skin_Mela	FKBP9
AAED18	1.41E-06	0.488953	0.218	0.076	0.022512	Skin_Mela	AAED1
TUBB10	1.43E-06	0.707937	0.538	0.311	0.022767	Skin_Mela	TUBB
ILK5	1.43E-06	0.709574	0.321	0.148	0.022798	Skin_Mela	ILK
GADD45GI	1.44E-06	0.692995	0.487	0.28	0.022937	Skin_Mela	GADD45GIP1
DNASE21	1.45E-06	0.558626	0.205	0.071	0.0231	Skin_Mela	DNASE2
ZNF7036	1.5E-06	0.503537	0.154	0.044	0.023925	Skin_Mela	ZNF703
HEY14	1.62E-06	0.451815	0.115	0.028	0.025827	Skin_Mela	HEY1
ATP5G23	1.62E-06	0.43318	0.808	0.573	0.025834	Skin_Mela	ATP5G2
COL5A33	1.65E-06	0.282666	0.064	0.01	0.026254	Skin_Mela	COL5A3
M6PR3	1.73E-06	0.643874	0.295	0.129	0.027557	Skin_Mela	M6PR
AFF31	1.78E-06	0.370737	0.103	0.023	0.028324	Skin_Mela	AFF3
SERF26	1.82E-06	0.445599	0.974	0.823	0.029086	Skin_Mela	SERF2
ATP5J21	1.82E-06	0.702835	0.603	0.439	0.029086	Skin_Mela	ATP5J2
CUTA2	1.83E-06	0.632321	0.59	0.376	0.029139	Skin_Mela	CUTA
HSPA1B6	1.83E-06	0.517497	0.551	0.311	0.029198	Skin_Mela	HSPA1B

TRAK23	1.84E-06	0.628481	0.154	0.046	0.029335	Skin_Mela	TRAK2
DYNLL17	1.89E-06	0.470416	0.782	0.561	0.030167	Skin_Mela	DYNLL1
DIP2C	2.02E-06	0.386596	0.115	0.028	0.032245	Skin_Mela	DIP2C
CANX9	2.08E-06	0.63507	0.513	0.312	0.033188	Skin_Mela	CANX
CADM32	2.08E-06	0.60408	0.103	0.023	0.033233	Skin_Mela	CADM3
ANAPC11	2.11E-06	0.76515	0.564	0.377	0.033681	Skin_Mela	ANAPC11
DNAJC36	2.12E-06	0.869683	0.385	0.208	0.033842	Skin_Mela	DNAJC3
EMC102	2.16E-06	0.859568	0.321	0.153	0.034499	Skin_Mela	EMC10
RTN48	2.17E-06	0.605322	0.615	0.395	0.034589	Skin_Mela	RTN4
ALDOA1	2.22E-06	0.508737	0.782	0.642	0.035441	Skin_Mela	ALDOA
DCTN33	2.25E-06	0.751483	0.423	0.235	0.035812	Skin_Mela	DCTN3
FAM127C1	2.25E-06	0.555087	0.115	0.028	0.035918	Skin_Mela	FAM127C
FDFT12	2.29E-06	0.722327	0.397	0.212	0.036499	Skin_Mela	FDFT1
GNPTAB4	2.31E-06	0.63211	0.231	0.09	0.036878	Skin_Mela	GNPTAB
KRTCAP23	2.32E-06	0.845808	0.5	0.314	0.036973	Skin_Mela	KRTCAP2
ZNF4282	2.32E-06	0.700668	0.346	0.167	0.037073	Skin_Mela	ZNF428
SLC44A15	2.34E-06	0.769104	0.218	0.08	0.037394	Skin_Mela	SLC44A1
HNRNPH18	2.41E-06	0.728148	0.5	0.298	0.038487	Skin_Mela	HNRNPH1
GMFB2	2.44E-06	0.66844	0.192	0.067	0.038887	Skin_Mela	GMFB
RPS157	2.45E-06	0.388975	1	0.881	0.039131	Skin_Mela	RPS15
LRCH14	2.47E-06	0.616028	0.141	0.04	0.039352	Skin_Mela	LRCH1
PIN1	2.52E-06	0.68658	0.397	0.205	0.040179	Skin_Mela	PIN1
CNRIP18	2.55E-06	0.449428	0.141	0.039	0.040623	Skin_Mela	CNRIP1
EFNA15	2.67E-06	0.819373	0.231	0.09	0.042662	Skin_Mela	EFNA1
CHML1	2.71E-06	0.399432	0.103	0.023	0.043207	Skin_Mela	CHML
ARRDC4	2.77E-06	0.33125	0.128	0.034	0.044116	Skin_Mela	ARRDC4
MTCH2	2.77E-06	0.826091	0.295	0.135	0.044202	Skin_Mela	MTCH2
SLITRK42	2.95E-06	0.374509	0.103	0.023	0.046978	Skin_Mela	SLITRK4
SEMA6A3	3.11E-06	0.512638	0.115	0.029	0.049574	Skin_Mela	SEMA6A
NUCB15	3.14E-06	0.776874	0.385	0.206	0.050016	Skin_Mela	NUCB1
ZNF7064	3.19E-06	0.737358	0.538	0.345	0.050837	Skin_Mela	ZNF706
SUCLG12	3.23E-06	0.846042	0.346	0.177	0.051463	Skin_Mela	SUCLG1
B3GAT31	3.6E-06	0.704667	0.218	0.082	0.057496	Skin_Mela	B3GAT3
C3orf17	3.61E-06	0.563602	0.154	0.047	0.057609	Skin_Mela	C3orf17
HNRNPDL7	3.7E-06	0.479745	0.59	0.337	0.05904	Skin_Mela	HNRNPDL
ARSG1	3.7E-06	0.360064	0.103	0.024	0.059063	Skin_Mela	ARSG
NDUFC25	3.88E-06	0.691719	0.59	0.386	0.061868	Skin_Mela	NDUFC2
PALM4	3.92E-06	0.34868	0.103	0.024	0.062571	Skin_Mela	PALM
MYC5	4.09E-06	0.622298	0.308	0.13	0.065308	Skin_Mela	MYC
GRHL31	4.12E-06	0.374556	0.077	0.015	0.065649	Skin_Mela	GRHL3
WDFY1	4.13E-06	0.657841	0.167	0.054	0.065823	Skin_Mela	WDFY1
SRSF210	4.16E-06	0.779023	0.526	0.333	0.066302	Skin_Mela	SRSF2
ADAM102	4.17E-06	0.643944	0.205	0.076	0.06647	Skin_Mela	ADAM10
COX163	4.18E-06	0.594733	0.282	0.12	0.066613	Skin_Mela	COX16
PRDX37	4.25E-06	0.745542	0.321	0.157	0.06771	Skin_Mela	PRDX3
ETFB5	4.28E-06	0.668004	0.462	0.272	0.068208	Skin_Mela	ETFB
CRTAP8	4.4E-06	0.803495	0.269	0.115	0.070182	Skin_Mela	CRTAP

PRKAR1A5	4.68E-06	1.03916	0.41	0.239	0.074694	Skin_Mela	PRKAR1A
COX4I1	4.93E-06	0.288373	0.872	0.725	0.078611	Skin_Mela	COX4I1
AP2M13	5.08E-06	0.677474	0.5	0.305	0.08105	Skin_Mela	AP2M1
MRPL511	5.13E-06	0.69376	0.474	0.282	0.081759	Skin_Mela	MRPL51
ACOT94	5.22E-06	0.485206	0.179	0.061	0.083246	Skin_Mela	ACOT9
ARF11	5.28E-06	0.634294	0.641	0.466	0.084247	Skin_Mela	ARF1
NAPA	5.59E-06	0.7784	0.359	0.192	0.089199	Skin_Mela	NAPA
CLTA2	5.61E-06	0.684216	0.564	0.386	0.089441	Skin_Mela	CLTA
FIS15	5.68E-06	0.783947	0.513	0.338	0.090642	Skin_Mela	FIS1
RP11.138A	5.99E-06	0.56763	0.154	0.047	0.095477	Skin_Mela	RP11.138A9.1
SHISA4	6.1E-06	0.426729	0.115	0.03	0.097357	Skin_Mela	SHISA4
ZNF7046	6.21E-06	0.554203	0.192	0.069	0.099043	Skin_Mela	ZNF704
ARPC1B3	6.29E-06	0.460506	0.526	0.293	0.100265	Skin_Mela	ARPC1B
SRSF119	6.42E-06	0.702373	0.487	0.303	0.102423	Skin_Mela	SRSF11
CHN2	6.57E-06	0.386098	0.115	0.03	0.10484	Skin_Mela	CHN2
MAP3K32	6.88E-06	0.411077	0.128	0.036	0.109682	Skin_Mela	MAP3K3
SLC23A2	7.02E-06	0.281396	0.09	0.02	0.111999	Skin_Mela	SLC23A2
KHDRBS15	7.09E-06	0.655553	0.397	0.216	0.113145	Skin_Mela	KHDRBS1
TMEM117	7.26E-06	0.391858	0.09	0.02	0.115852	Skin_Mela	TMEM117
FOS11	7.38E-06	0.543341	0.705	0.425	0.117763	Skin_Mela	FOS
FZD3	7.73E-06	0.574238	0.141	0.043	0.123233	Skin_Mela	FZD3
SH3BGRL3	7.88E-06	0.336278	0.821	0.633	0.125643	Skin_Mela	SH3BGRL3
CNIH43	8.02E-06	0.715335	0.295	0.137	0.127914	Skin_Mela	CNIH4
ATP5B7	8.12E-06	0.842758	0.538	0.372	0.129528	Skin_Mela	ATP5B
DNPH11	8.21E-06	0.733551	0.308	0.147	0.130881	Skin_Mela	DNPH1
AK22	8.27E-06	0.74658	0.308	0.153	0.131867	Skin_Mela	AK2
SUMO17	8.3E-06	0.689245	0.526	0.347	0.132425	Skin_Mela	SUMO1
SEC61G9	8.61E-06	0.62814	0.615	0.415	0.137368	Skin_Mela	SEC61G
AXIN21	8.68E-06	0.384264	0.09	0.02	0.138506	Skin_Mela	AXIN2
HNRNPK6	9.08E-06	0.674668	0.603	0.447	0.144772	Skin_Mela	HNRNPK
PLA2G7	9.13E-06	0.347103	0.064	0.011	0.145661	Skin_Mela	PLA2G7
PSMB7	9.26E-06	0.78099	0.487	0.305	0.147759	Skin_Mela	PSMB7
ATP6V0B7	9.48E-06	0.5978	0.577	0.388	0.151155	Skin_Mela	ATP6V0B
FLNA10	9.68E-06	0.590683	0.321	0.15	0.154466	Skin_Mela	FLNA
WBP54	1.02E-05	0.827964	0.346	0.181	0.162529	Skin_Mela	WBP5
PLD35	1.02E-05	0.601953	0.333	0.165	0.163165	Skin_Mela	PLD3
RPS265	1.02E-05	0.289338	0.949	0.742	0.163232	Skin_Mela	RPS26
ATP5C14	1.04E-05	0.713572	0.462	0.282	0.165536	Skin_Mela	ATP5C1
RNF1811	1.07E-05	0.750767	0.474	0.303	0.170899	Skin_Mela	RNF181
SUPT4H11	1.08E-05	0.629445	0.423	0.243	0.172976	Skin_Mela	SUPT4H1
ZC3H12C1	1.15E-05	0.489045	0.115	0.031	0.18333	Skin_Mela	ZC3H12C
NDN5	1.16E-05	0.654314	0.179	0.063	0.185284	Skin_Mela	NDN
FYN7	1.18E-05	0.400766	0.192	0.067	0.187658	Skin_Mela	FYN
OS96	1.22E-05	0.691364	0.397	0.226	0.19432	Skin_Mela	OS9
TMEM230	1.22E-05	0.777077	0.449	0.267	0.19455	Skin_Mela	TMEM230
UPP11	1.23E-05	0.527743	0.282	0.124	0.196071	Skin_Mela	UPP1
PMM11	1.24E-05	0.605363	0.192	0.072	0.197664	Skin_Mela	PMM1

VAMP2	1.27E-05	0.552551	0.538	0.338	0.202683	Skin_Mela	VAMP2
AP3M21	1.3E-05	0.629258	0.141	0.044	0.207237	Skin_Mela	AP3M2
MMP141	1.33E-05	0.545784	0.167	0.056	0.211545	Skin_Mela	MMP14
ASXL15	1.34E-05	0.577153	0.205	0.079	0.214475	Skin_Mela	ASXL1
C21orf33	1.35E-05	0.49901	0.167	0.056	0.214532	Skin_Mela	C21orf33
HPCAL13	1.37E-05	0.570561	0.205	0.078	0.218549	Skin_Mela	HPCAL1
ADAMTS15	1.38E-05	0.344772	0.179	0.061	0.220517	Skin_Mela	ADAMTS1
TRIB2	1.39E-05	0.416802	0.141	0.043	0.221605	Skin_Mela	TRIB2
TONSL	1.39E-05	0.329603	0.064	0.012	0.222236	Skin_Mela	TONSL
NDUFA54	1.59E-05	0.72408	0.423	0.252	0.253334	Skin_Mela	NDUFA5
RBM8A6	1.65E-05	0.583606	0.526	0.343	0.262784	Skin_Mela	RBM8A
BAALC3	1.65E-05	0.314433	0.077	0.016	0.26284	Skin_Mela	BAALC
PRMT17	1.66E-05	0.530222	0.372	0.192	0.264369	Skin_Mela	PRMT1
SLC16A13	1.68E-05	0.542095	0.141	0.044	0.268716	Skin_Mela	SLC16A1
BRI310	1.77E-05	0.779611	0.526	0.347	0.281939	Skin_Mela	BRI3
BCAT2	1.82E-05	0.478987	0.141	0.044	0.290341	Skin_Mela	BCAT2
RNF243	1.84E-05	0.60711	0.179	0.065	0.294239	Skin_Mela	RNF24
NDUFB1	1.87E-05	0.802758	0.513	0.363	0.297589	Skin_Mela	NDUFB1
SEMA3D2	1.89E-05	0.29292	0.09	0.021	0.302188	Skin_Mela	SEMA3D
RP5.1085F	1.95E-05	0.273255	0.077	0.016	0.310644	Skin_Mela	RP5.1085F17.3
RASGEF1B4	1.98E-05	0.84686	0.179	0.066	0.315964	Skin_Mela	RASGEF1B
GAN1	2.02E-05	0.565804	0.167	0.058	0.321587	Skin_Mela	GAN
SSNA1	2.05E-05	0.766368	0.397	0.231	0.32706	Skin_Mela	SSNA1
GUCY1B33	2.08E-05	0.517692	0.09	0.021	0.331568	Skin_Mela	GUCY1B3
SON10	2.08E-05	0.59581	0.59	0.41	0.331929	Skin_Mela	SON
STRAP5	2.12E-05	0.693406	0.385	0.218	0.338749	Skin_Mela	STRAP
TMEM641	2.12E-05	0.405775	0.115	0.032	0.338758	Skin_Mela	TMEM64
POMP7	2.14E-05	0.70063	0.577	0.403	0.341093	Skin_Mela	POMP
SEPHS23	2.15E-05	0.461575	0.179	0.064	0.342887	Skin_Mela	SEPHS2
MAD2L1BP	2.18E-05	0.58397	0.154	0.052	0.34737	Skin_Mela	MAD2L1BP
MICA2	2.2E-05	0.411114	0.167	0.058	0.3503	Skin_Mela	MICA
RWDD13	2.2E-05	0.758327	0.487	0.309	0.351056	Skin_Mela	RWDD1
ITGB1BP17	2.27E-05	0.695735	0.308	0.152	0.361302	Skin_Mela	ITGB1BP1
DNAJC153	2.27E-05	0.5464	0.359	0.19	0.362254	Skin_Mela	DNAJC15
RNF1312	2.29E-05	0.730358	0.321	0.167	0.365571	Skin_Mela	RNF13
MRPS281	2.32E-05	0.607876	0.154	0.052	0.370041	Skin_Mela	MRPS28
FANCL	2.34E-05	0.486393	0.115	0.033	0.373648	Skin_Mela	FANCL
DHRS78	2.36E-05	0.743052	0.372	0.209	0.375859	Skin_Mela	DHRS7
GOLGA7B	2.49E-05	0.269891	0.051	0.008	0.397091	Skin_Mela	GOLGA7B
CALM19	2.52E-05	0.441334	0.795	0.651	0.401381	Skin_Mela	CALM1
MFAP22	2.54E-05	0.302507	0.103	0.026	0.405785	Skin_Mela	MFAP2
U2AF17	2.56E-05	0.642052	0.449	0.256	0.408299	Skin_Mela	U2AF1
NUCKS110	2.64E-05	0.683748	0.474	0.284	0.42104	Skin_Mela	NUCKS1
PSMB42	2.66E-05	0.728811	0.333	0.18	0.423821	Skin_Mela	PSMB4
DENND5B1	2.66E-05	0.332791	0.09	0.022	0.424022	Skin_Mela	DENND5B
FUS13	2.66E-05	0.70559	0.449	0.277	0.424174	Skin_Mela	FUS
CBR34	2.75E-05	0.653543	0.205	0.083	0.437941	Skin_Mela	CBR3

MAP3K17	2.76E-05	0.642651	0.179	0.068	0.440653	Skin_Mela	MAP3K1
EEA13	2.78E-05	0.642523	0.231	0.099	0.443006	Skin_Mela	EEA1
NHSL13	2.79E-05	0.539181	0.128	0.039	0.445254	Skin_Mela	NHSL1
MLLT10	2.85E-05	0.625464	0.192	0.075	0.454795	Skin_Mela	MLLT10
RPL27A6	2.96E-05	0.258703	0.987	0.836	0.472019	Skin_Mela	RPL27A
TRIQK1	2.96E-05	0.456902	0.128	0.039	0.472371	Skin_Mela	TRIQK
TRIM476	2.96E-05	0.547131	0.167	0.059	0.472696	Skin_Mela	TRIM47
NDUFA65	2.99E-05	0.74366	0.487	0.322	0.476999	Skin_Mela	NDUFA6
ZNF907	3.05E-05	0.596409	0.231	0.095	0.487214	Skin_Mela	ZNF90
MIR4458H	3.08E-05	0.314152	0.128	0.039	0.490459	Skin_Mela	MIR4458HG
LINC00969	3.08E-05	0.892655	0.244	0.112	0.491606	Skin_Mela	LINC00969
MAP49	3.1E-05	0.725463	0.269	0.129	0.494791	Skin_Mela	MAP4
MINOS13	3.11E-05	0.634675	0.487	0.308	0.495252	Skin_Mela	MINOS1
UROS2	3.21E-05	0.520958	0.231	0.1	0.512292	Skin_Mela	UROS
ZNF615	3.31E-05	0.326558	0.051	0.008	0.528325	Skin_Mela	ZNF615
CTH2	3.31E-05	0.444995	0.077	0.017	0.528615	Skin_Mela	CTH
SNRPE6	3.32E-05	0.746093	0.436	0.267	0.529586	Skin_Mela	SNRPE
TLE12	3.35E-05	0.745247	0.179	0.069	0.533668	Skin_Mela	TLE1
BAG41	3.7E-05	0.513968	0.141	0.046	0.59072	Skin_Mela	BAG4
RCBTB1	3.71E-05	0.315178	0.064	0.013	0.591076	Skin_Mela	RCBTB1
PRELID13	3.75E-05	0.626916	0.551	0.379	0.598273	Skin_Mela	PRELID1
NAA103	3.75E-05	0.728653	0.308	0.16	0.598284	Skin_Mela	NAA10
SLCO4A11	3.86E-05	0.553918	0.244	0.107	0.615791	Skin_Mela	SLCO4A1
ATP5L4	3.94E-05	0.414593	0.833	0.66	0.628318	Skin_Mela	ATP5L
CBFB2	4.02E-05	0.602417	0.218	0.093	0.640851	Skin_Mela	CBFB
MEGF9	4.04E-05	0.430163	0.115	0.034	0.644447	Skin_Mela	MEGF9
LIPA3	4.16E-05	0.499485	0.192	0.074	0.662821	Skin_Mela	LIPA
VPS72	4.18E-05	0.453099	0.192	0.076	0.667407	Skin_Mela	VPS72
TRPM43	4.2E-05	0.556875	0.167	0.061	0.669831	Skin_Mela	TRPM4
RPS289	4.35E-05	0.311405	1	0.881	0.693917	Skin_Mela	RPS28
DAG12	4.38E-05	0.627883	0.167	0.062	0.698263	Skin_Mela	DAG1
FSCN15	4.38E-05	0.514863	0.179	0.068	0.699326	Skin_Mela	FSCN1
CTBP24	4.51E-05	0.620612	0.231	0.103	0.719225	Skin_Mela	CTBP2
ZFX	4.66E-05	0.585569	0.167	0.062	0.743281	Skin_Mela	ZFX
DDX179	4.7E-05	0.659536	0.526	0.354	0.749456	Skin_Mela	DDX17
C1orf216	4.79E-05	0.310218	0.077	0.017	0.763664	Skin_Mela	C1orf216
MYO9B3	4.86E-05	0.689897	0.179	0.069	0.774971	Skin_Mela	MYO9B
CNPY23	4.86E-05	0.697437	0.359	0.206	0.775364	Skin_Mela	CNPY2
SCP2	4.94E-05	0.499946	0.538	0.36	0.787861	Skin_Mela	SCP2
ZBTB213	4.95E-05	0.522986	0.141	0.047	0.789095	Skin_Mela	ZBTB21
IL11RA2	5.07E-05	0.499031	0.154	0.053	0.808829	Skin_Mela	IL11RA
POLE44	5.12E-05	0.585191	0.321	0.166	0.815956	Skin_Mela	POLE4
SYNC1	5.18E-05	0.472454	0.141	0.047	0.826591	Skin_Mela	SYNC
BCL2L12	5.19E-05	0.483052	0.141	0.047	0.828107	Skin_Mela	BCL2L12
EIF4EBP15	5.3E-05	0.668634	0.295	0.151	0.844542	Skin_Mela	EIF4EBP1
SUMO28	5.36E-05	0.52426	0.744	0.602	0.854638	Skin_Mela	SUMO2
DCK2	5.38E-05	0.526688	0.167	0.06	0.858691	Skin_Mela	DCK

CADM4	5.47E-05	0.488095	0.09	0.023	0.872501	Skin_Mela	CADM4
PQLC36	5.52E-05	0.520501	0.218	0.092	0.879606	Skin_Mela	PQLC3
AAMDC4	5.58E-05	0.687427	0.295	0.154	0.889731	Skin_Mela	AAMDC
HINT111	5.61E-05	0.427261	0.769	0.57	0.895056	Skin_Mela	HINT1
C1orf433	5.64E-05	0.637567	0.385	0.228	0.899098	Skin_Mela	C1orf43
FKBP24	5.66E-05	0.340504	0.564	0.351	0.901955	Skin_Mela	FKBP2
NDUFA103	5.76E-05	0.66332	0.333	0.184	0.918588	Skin_Mela	NDUFA10
CDC123	5.79E-05	0.673179	0.231	0.103	0.923549	Skin_Mela	CDC123
SLC2A8	6.02E-05	0.584341	0.154	0.055	0.960276	Skin_Mela	SLC2A8
SEC22C1	6.04E-05	0.581936	0.192	0.079	0.962693	Skin_Mela	SEC22C
N4BP3	6.09E-05	0.405547	0.077	0.018	0.970717	Skin_Mela	N4BP3
NME14	6.1E-05	0.705155	0.308	0.165	0.972559	Skin_Mela	NME1
ITGAV6	6.14E-05	0.606148	0.218	0.096	0.978481	Skin_Mela	ITGAV
COX7A110	6.15E-05	0.738962	0.282	0.139	0.980864	Skin_Mela	COX7A1
LAMB28	6.23E-05	0.48356	0.205	0.083	0.993797	Skin_Mela	LAMB2
TMEM251	6.28E-05	0.646147	0.192	0.08	1	Skin_Mela	TMEM251
MPRIP.AS1	6.39E-05	0.475924	0.064	0.013	1	Skin_Mela	MPRIP.AS1
TAF9B	6.41E-05	0.440452	0.103	0.029	1	Skin_Mela	TAF9B
USP482	6.46E-05	0.630892	0.218	0.097	1	Skin_Mela	USP48
QKI8	6.49E-05	0.664835	0.321	0.172	1	Skin_Mela	QKI
UBE2E24	6.52E-05	0.736743	0.192	0.08	1	Skin_Mela	UBE2E2
MICU2	6.67E-05	0.551147	0.218	0.095	1	Skin_Mela	MICU2
CHPT16	6.92E-05	0.69193	0.359	0.207	1	Skin_Mela	CHPT1
AL592183.	7.02E-05	0.324753	0.09	0.023	1	Skin_Mela	AL592183.1
SEPT77	7.12E-05	0.432934	0.538	0.34	1	Skin_Mela	SEPT7
EFNB12	7.29E-05	0.530074	0.115	0.035	1	Skin_Mela	EFNB1
RTFDC1	7.38E-05	0.782565	0.346	0.198	1	Skin_Mela	RTFDC1
PHB6	7.49E-05	0.363688	0.436	0.241	1	Skin_Mela	PHB
IDH3B	7.58E-05	0.617011	0.269	0.132	1	Skin_Mela	IDH3B
PTGES38	7.6E-05	0.60174	0.564	0.393	1	Skin_Mela	PTGES3
APOA1BP1	8E-05	0.622363	0.269	0.133	1	Skin_Mela	APOA1BP
PLAG1	8.01E-05	0.475774	0.103	0.029	1	Skin_Mela	PLAG1
SYPL110	8.05E-05	0.67181	0.423	0.264	1	Skin_Mela	SYPL1
FAM127B3	8.09E-05	0.581958	0.192	0.08	1	Skin_Mela	FAM127B
ALG6	8.13E-05	0.369323	0.103	0.029	1	Skin_Mela	ALG6
COL4A28	8.18E-05	0.564968	0.154	0.055	1	Skin_Mela	COL4A2
RABL6	8.42E-05	0.527821	0.218	0.096	1	Skin_Mela	RABL6
HCG11	8.52E-05	0.387042	0.077	0.018	1	Skin_Mela	HCG11
RAB346	8.74E-05	0.456435	0.231	0.102	1	Skin_Mela	RAB34
BTG18	8.89E-05	0.319716	0.782	0.578	1	Skin_Mela	BTG1
AK13	8.95E-05	0.574827	0.282	0.143	1	Skin_Mela	AK1
FAM214B	9.57E-05	0.457032	0.115	0.036	1	Skin_Mela	FAM214B
RAB281	9.7E-05	0.509891	0.154	0.057	1	Skin_Mela	RAB28
VIMP6	9.74E-05	0.60153	0.462	0.292	1	Skin_Mela	VIMP
BRK12	9.85E-05	0.52791	0.603	0.455	1	Skin_Mela	BRK1
PBDC11	0.0001	0.599805	0.205	0.088	1	Skin_Mela	PBDC1
NDUFB109	0.0001	0.62339	0.551	0.385	1	Skin_Mela	NDUFB10

LAGE31	0.000101	0.584268	0.282	0.143	1	Skin_Mela	LAGE3
NDUFB92	0.000101	0.578764	0.526	0.346	1	Skin_Mela	NDUFB9
PDHB	0.000103	0.644736	0.244	0.117	1	Skin_Mela	PDHB
C1orf566	0.000103	0.522611	0.256	0.122	1	Skin_Mela	C1orf56
NDUFS65	0.000105	0.577309	0.474	0.324	1	Skin_Mela	NDUFS6
IPMK2	0.000106	0.477665	0.141	0.049	1	Skin_Mela	IPMK
DMXL1	0.000107	0.542634	0.154	0.057	1	Skin_Mela	DMXL1
RNH15	0.000108	0.696903	0.462	0.315	1	Skin_Mela	RNH1
BLOC1S11	0.000109	0.493808	0.513	0.342	1	Skin_Mela	BLOC1S1
CELSR21	0.000109	0.394847	0.064	0.014	1	Skin_Mela	CELSR2
BCL21	0.000111	0.538922	0.128	0.042	1	Skin_Mela	BCL2
DUT4	0.000112	0.690204	0.385	0.236	1	Skin_Mela	DUT
SFPQ10	0.000113	0.628602	0.449	0.286	1	Skin_Mela	SFPQ
NTAN16	0.000114	0.512018	0.256	0.124	1	Skin_Mela	NTAN1
ACYP12	0.000115	0.482527	0.167	0.064	1	Skin_Mela	ACYP1
SLC7A82	0.000116	0.62515	0.09	0.024	1	Skin_Mela	SLC7A8
TANGO2	0.000117	0.42795	0.09	0.024	1	Skin_Mela	TANGO2
BNIP27	0.00012	0.555499	0.256	0.125	1	Skin_Mela	BNIP2
GTF2IRD2E	0.00012	0.522414	0.09	0.024	1	Skin_Mela	GTF2IRD2B
LPL1	0.000121	0.322071	0.064	0.014	1	Skin_Mela	LPL
SLC12A72	0.000121	0.347293	0.09	0.024	1	Skin_Mela	SLC12A7
RPL126	0.000124	0.257815	1	0.853	1	Skin_Mela	RPL12
SNRPG3	0.000125	0.547801	0.526	0.355	1	Skin_Mela	SNRPG
TMEM192	0.000128	0.738708	0.154	0.058	1	Skin_Mela	TMEM192
SPG213	0.000129	0.59748	0.295	0.157	1	Skin_Mela	SPG21
FAM174A4	0.000131	0.750995	0.192	0.084	1	Skin_Mela	FAM174A
GALK12	0.000131	0.52231	0.167	0.065	1	Skin_Mela	GALK1
PFDN6	0.000131	0.731356	0.256	0.128	1	Skin_Mela	PFDN6
BROX1	0.000133	0.614456	0.231	0.109	1	Skin_Mela	BROX
TP53TG11	0.000136	0.577938	0.205	0.09	1	Skin_Mela	TP53TG1
C7orf503	0.000137	0.511846	0.269	0.135	1	Skin_Mela	C7orf50
PACS2	0.000137	0.483459	0.141	0.051	1	Skin_Mela	PACS2
NSMF	0.000137	0.353746	0.103	0.03	1	Skin_Mela	NSMF
C19orf605	0.000138	0.440783	0.397	0.23	1	Skin_Mela	C19orf60
C7orf55	0.000141	0.472376	0.231	0.106	1	Skin_Mela	C7orf55
PLP27	0.000142	0.438009	0.41	0.232	1	Skin_Mela	PLP2
RP3.395M	0.000143	0.253109	0.077	0.018	1	Skin_Mela	RP3.395M20.12
PUF602	0.000148	0.605983	0.295	0.157	1	Skin_Mela	PUF60
MARCH65	0.000152	0.912386	0.295	0.165	1	Skin_Mela	MARCH6
VPS285	0.000153	0.513793	0.513	0.349	1	Skin_Mela	VPS28
PHLPP1	0.000156	0.534422	0.128	0.044	1	Skin_Mela	PHLPP1
CERCAM3	0.000156	0.443764	0.154	0.058	1	Skin_Mela	CERCAM
RNF43	0.000157	0.410156	0.103	0.031	1	Skin_Mela	RNF43
APRT5	0.000159	0.41842	0.551	0.37	1	Skin_Mela	APRT
DYNLL2	0.000162	0.592598	0.244	0.117	1	Skin_Mela	DYNLL2
EIF3K2	0.000171	0.266642	0.692	0.518	1	Skin_Mela	EIF3K
MSRB27	0.000171	0.54261	0.256	0.13	1	Skin_Mela	MSRB2

RNF71	0.000171	0.691986	0.449	0.304	1	Skin_Mela	RNF7
GPC1	0.000172	0.510068	0.154	0.058	1	Skin_Mela	GPC1
OLFM14	0.000174	0.405317	0.09	0.025	1	Skin_Mela	OLFM1
NDUFA8	0.000174	0.572422	0.308	0.168	1	Skin_Mela	NDUFA8
PPFIA3	0.000174	0.441981	0.077	0.019	1	Skin_Mela	PPFIA3
BCAR13	0.000177	0.547598	0.179	0.075	1	Skin_Mela	BCAR1
NDUFAF42	0.000178	0.494648	0.192	0.082	1	Skin_Mela	NDUFAF4
DEGS14	0.000191	0.492381	0.256	0.127	1	Skin_Mela	DEGS1
TMEM50A	0.000192	0.583572	0.564	0.417	1	Skin_Mela	TMEM50A
SPSB14	0.000192	0.558113	0.154	0.059	1	Skin_Mela	SPSB1
GLOD42	0.000197	0.691091	0.244	0.12	1	Skin_Mela	GLOD4
HNRNPA39	0.000198	0.431111	0.538	0.365	1	Skin_Mela	HNRNPA3
ARHGEF40	0.000204	0.501869	0.115	0.038	1	Skin_Mela	ARHGEF40
ARID1B2	0.000209	0.55953	0.192	0.084	1	Skin_Mela	ARID1B
LONRF13	0.000213	0.622592	0.154	0.06	1	Skin_Mela	LONRF1
PPM1B	0.000218	0.711065	0.244	0.123	1	Skin_Mela	PPM1B
FIGN2	0.00022	0.370489	0.09	0.025	1	Skin_Mela	FIGN
FMR1	0.00022	0.472729	0.167	0.068	1	Skin_Mela	FMR1
MED212	0.00022	0.714324	0.269	0.145	1	Skin_Mela	MED21
NDUFS44	0.000224	0.612468	0.359	0.218	1	Skin_Mela	NDUFS4
TMEM220	0.000231	0.600102	0.141	0.053	1	Skin_Mela	TMEM220
SF3B51	0.000244	0.42817	0.526	0.363	1	Skin_Mela	SF3B5
SOCS61	0.000249	0.46972	0.128	0.046	1	Skin_Mela	SOCS6
EAPP3	0.000249	0.576731	0.321	0.184	1	Skin_Mela	EAPP
ATRAID6	0.000249	0.617336	0.436	0.291	1	Skin_Mela	ATRAID
KAT5	0.000258	0.67109	0.167	0.068	1	Skin_Mela	KAT5
THRAP34	0.000262	0.703391	0.308	0.176	1	Skin_Mela	THRAP3
RANBP16	0.000265	0.568932	0.423	0.267	1	Skin_Mela	RANBP1
MTDH8	0.000274	0.651139	0.462	0.309	1	Skin_Mela	MTDH
MOB47	0.000277	0.503992	0.205	0.093	1	Skin_Mela	MOB4
C11orf243	0.000278	0.427975	0.154	0.06	1	Skin_Mela	C11orf24
PSMD3	0.000279	0.61203	0.244	0.122	1	Skin_Mela	PSMD3
JKAMP1	0.00028	0.698025	0.218	0.107	1	Skin_Mela	JKAMP
COMMD72	0.000283	0.469639	0.256	0.129	1	Skin_Mela	COMMD7
GNG124	0.000284	0.564401	0.333	0.195	1	Skin_Mela	GNG12
LAMP12	0.000287	0.607282	0.436	0.298	1	Skin_Mela	LAMP1
CCT3	0.000287	0.559483	0.372	0.222	1	Skin_Mela	CCT3
PFDN27	0.000287	0.533243	0.436	0.275	1	Skin_Mela	PFDN2
BIK1	0.000287	0.53516	0.141	0.054	1	Skin_Mela	BIK
NOM11	0.000287	0.394387	0.115	0.039	1	Skin_Mela	NOM1
CASD11	0.000299	0.545388	0.103	0.032	1	Skin_Mela	CASD1
SRSF67	0.0003	0.655513	0.269	0.145	1	Skin_Mela	SRSF6
CISD3	0.0003	0.527708	0.218	0.103	1	Skin_Mela	CISD3
CYC14	0.000303	0.556031	0.372	0.231	1	Skin_Mela	CYC1
PKD23	0.000303	0.344216	0.09	0.026	1	Skin_Mela	PKD2
AP1S1	0.00031	0.468367	0.205	0.095	1	Skin_Mela	AP1S1
RBM67	0.00031	0.465559	0.231	0.111	1	Skin_Mela	RBM6

CNP	0.000312	0.554156	0.167	0.07	1	Skin_Mela	CNP
SEMA4C4	0.000313	0.283536	0.09	0.026	1	Skin_Mela	SEMA4C
KIAA1715	0.000327	0.439941	0.167	0.069	1	Skin_Mela	KIAA1715
DGUOK1	0.000336	0.501965	0.333	0.193	1	Skin_Mela	DGUOK
LAMP28	0.000338	0.662539	0.372	0.236	1	Skin_Mela	LAMP2
TMEM219	0.000345	0.578102	0.462	0.332	1	Skin_Mela	TMEM219
NDUFS32	0.000351	0.58961	0.321	0.189	1	Skin_Mela	NDUFS3
PPFIBP14	0.000353	0.908737	0.218	0.106	1	Skin_Mela	PPFIBP1
RSL24D17	0.000354	0.546359	0.462	0.307	1	Skin_Mela	RSL24D1
TNFRSF12A	0.000371	0.466271	0.256	0.127	1	Skin_Mela	TNFRSF12A
TGFBR13	0.000371	0.51181	0.154	0.062	1	Skin_Mela	TGFBR1
PLA2G151	0.000373	0.286262	0.077	0.021	1	Skin_Mela	PLA2G15
EXTL22	0.000384	0.593987	0.115	0.04	1	Skin_Mela	EXTL2
HIGD2A1	0.000384	0.551062	0.5	0.352	1	Skin_Mela	HIGD2A
WSB111	0.000386	0.694614	0.385	0.25	1	Skin_Mela	WSB1
BAMBI1	0.000394	0.296663	0.077	0.021	1	Skin_Mela	BAMBI
HNRNPM3	0.000396	0.44892	0.372	0.217	1	Skin_Mela	HNRNPM
HSPG24	0.000397	0.571165	0.231	0.114	1	Skin_Mela	HSPG2
EIF3I2	0.000399	0.587023	0.385	0.244	1	Skin_Mela	EIF3I
PTN3	0.000401	0.537049	0.167	0.07	1	Skin_Mela	PTN
SAP303	0.000402	0.46402	0.141	0.054	1	Skin_Mela	SAP30
SELM12	0.000406	0.278236	0.564	0.347	1	Skin_Mela	SELM
ATP6AP14	0.000408	0.740433	0.282	0.161	1	Skin_Mela	ATP6AP1
TMEM261	0.000415	0.42399	0.269	0.14	1	Skin_Mela	TMEM261
TAF4B3	0.000418	0.285104	0.103	0.033	1	Skin_Mela	TAF4B
MOB3B3	0.000418	0.270793	0.09	0.026	1	Skin_Mela	MOB3B
APMAP1	0.000419	0.502776	0.218	0.105	1	Skin_Mela	APMAP
TMX15	0.00042	0.566346	0.218	0.105	1	Skin_Mela	TMX1
ARMCX6	0.000424	0.509561	0.167	0.072	1	Skin_Mela	ARMCX6
C12orf579	0.000431	0.347334	0.538	0.358	1	Skin_Mela	C12orf57
C14orf23	0.000437	0.61746	0.564	0.449	1	Skin_Mela	C14orf2
TMEM126I	0.000441	0.492648	0.244	0.123	1	Skin_Mela	TMEM126B
MUM12	0.000446	0.535668	0.192	0.088	1	Skin_Mela	MUM1
CHD72	0.00045	0.367624	0.128	0.047	1	Skin_Mela	CHD7
CHST2	0.000453	0.348861	0.064	0.015	1	Skin_Mela	CHST2
RP9	0.000468	0.390774	0.141	0.054	1	Skin_Mela	RP9
ZKSCAN16	0.000477	0.541413	0.231	0.117	1	Skin_Mela	ZKSCAN1
AC009501.	0.000485	0.37475	0.103	0.033	1	Skin_Mela	AC009501.4
GPX83	0.000486	0.307069	0.115	0.04	1	Skin_Mela	GPX8
HSD17B7	0.000491	0.445295	0.103	0.033	1	Skin_Mela	HSD17B7
CREBL22	0.000495	0.492416	0.167	0.072	1	Skin_Mela	CREBL2
SEC11A8	0.000496	0.505801	0.462	0.315	1	Skin_Mela	SEC11A
HP1BP35	0.0005	0.646806	0.359	0.225	1	Skin_Mela	HP1BP3
SAR1A7	0.000502	0.671804	0.359	0.233	1	Skin_Mela	SAR1A
RP11.798M	0.000506	0.370947	0.077	0.021	1	Skin_Mela	RP11.798M19.6
TMEM5	0.000508	0.420838	0.154	0.063	1	Skin_Mela	TMEM5
TMEM140	0.000518	0.319935	0.077	0.021	1	Skin_Mela	TMEM140

KIAA09303	0.000527	0.359629	0.167	0.07	1	Skin_Mela	KIAA0930
DAAM15	0.000541	0.574938	0.295	0.169	1	Skin_Mela	DAAM1
TEX2642	0.000554	0.48651	0.308	0.175	1	Skin_Mela	TEX264
CAPZA27	0.000557	0.639434	0.41	0.28	1	Skin_Mela	CAPZA2
CES11	0.000569	0.435729	0.141	0.054	1	Skin_Mela	CES1
TSPAN35	0.000573	0.624741	0.295	0.172	1	Skin_Mela	TSPAN3
URM1	0.000574	0.480046	0.218	0.106	1	Skin_Mela	URM1
TMEM101	0.000575	0.483475	0.179	0.081	1	Skin_Mela	TMEM101
C6orf483	0.000583	0.265043	0.5	0.313	1	Skin_Mela	C6orf48
RPS27L4	0.000584	0.524855	0.603	0.446	1	Skin_Mela	RPS27L
FEZ25	0.000619	0.640698	0.218	0.11	1	Skin_Mela	FEZ2
IL6R2	0.000624	0.372458	0.115	0.04	1	Skin_Mela	IL6R
CTSL8	0.000644	0.391401	0.218	0.104	1	Skin_Mela	CTSL
DYNC1LI25	0.000647	0.618771	0.282	0.163	1	Skin_Mela	DYNC1LI2
SBDS6	0.000652	0.495082	0.487	0.342	1	Skin_Mela	SBDS
FAH	0.000655	0.259156	0.09	0.028	1	Skin_Mela	FAH
TMEM242	0.000659	0.47473	0.167	0.072	1	Skin_Mela	TMEM242
DYNC1H17	0.000659	0.613261	0.256	0.14	1	Skin_Mela	DYNC1H1
PTAR11	0.000664	0.544825	0.154	0.065	1	Skin_Mela	PTAR1
OTUD7B	0.000685	0.437808	0.141	0.056	1	Skin_Mela	OTUD7B
MYEOV24	0.000691	0.651373	0.5	0.362	1	Skin_Mela	MYEOV2
RRAGA3	0.000696	0.547168	0.244	0.129	1	Skin_Mela	RRAGA
ARL6IP64	0.000703	0.459693	0.167	0.072	1	Skin_Mela	ARL6IP6
DHRS4L2	0.000708	0.570412	0.179	0.081	1	Skin_Mela	DHRS4L2
C4orf461	0.000714	0.346278	0.09	0.028	1	Skin_Mela	C4orf46
SRSF39	0.000722	0.60633	0.577	0.434	1	Skin_Mela	SRSF3
NT5C5	0.000724	0.48756	0.244	0.125	1	Skin_Mela	NT5C
RUFY32	0.000733	0.737566	0.167	0.075	1	Skin_Mela	RUFY3
RPL387	0.000733	0.250446	0.936	0.762	1	Skin_Mela	RPL38
RABGGTB7	0.000738	0.563239	0.205	0.101	1	Skin_Mela	RABGGTB
PAFAH1B3	0.000742	0.45083	0.192	0.09	1	Skin_Mela	PAFAH1B3
ARL6IP15	0.000749	0.613161	0.449	0.318	1	Skin_Mela	ARL6IP1
LYRM9	0.00075	0.467906	0.128	0.049	1	Skin_Mela	LYRM9
YWHAB5	0.000759	0.4808	0.551	0.413	1	Skin_Mela	YWHAB
RHBDD21	0.000768	0.545288	0.231	0.12	1	Skin_Mela	RHBDD2
MPC16	0.000776	0.577188	0.333	0.21	1	Skin_Mela	MPC1
PPP1CA2	0.000784	0.475145	0.449	0.314	1	Skin_Mela	PPP1CA
IMPDH23	0.000785	0.581873	0.282	0.158	1	Skin_Mela	IMPDH2
DCAKD	0.00079	0.348816	0.077	0.022	1	Skin_Mela	DCAKD
TP53I132	0.000794	0.534754	0.167	0.073	1	Skin_Mela	TP53I13
CD1644	0.000812	0.536865	0.5	0.371	1	Skin_Mela	CD164
SELT8	0.000821	0.570188	0.359	0.227	1	Skin_Mela	SELT
ATP5O3	0.000823	0.440581	0.564	0.431	1	Skin_Mela	ATP5O
METTL92	0.000833	0.637311	0.346	0.222	1	Skin_Mela	METTL9
YIF1B	0.000837	0.520135	0.154	0.066	1	Skin_Mela	YIF1B
FAM212A	0.000849	0.250982	0.064	0.016	1	Skin_Mela	FAM212A
MRPL44	0.000859	0.53218	0.167	0.075	1	Skin_Mela	MRPL44

SLC25A40	0.000861	0.582571	0.09	0.029	1	Skin_Mela	SLC25A40
NAB22	0.000864	0.351399	0.09	0.028	1	Skin_Mela	NAB2
SLC25A366	0.000869	0.647698	0.231	0.123	1	Skin_Mela	SLC25A36
C11orf31	0.000875	0.498718	0.487	0.354	1	Skin_Mela	C11orf31
HNRNPLL2	0.000881	0.552144	0.154	0.066	1	Skin_Mela	HNRNPLL
CHMP4B6	0.000881	0.485253	0.462	0.307	1	Skin_Mela	CHMP4B
AHSA13	0.000887	0.667714	0.256	0.145	1	Skin_Mela	AHSA1
PGRMC17	0.000897	0.668634	0.282	0.167	1	Skin_Mela	PGRMC1
TFPT	0.000922	0.513722	0.179	0.084	1	Skin_Mela	TFPT
FERMT28	0.000933	0.452331	0.179	0.082	1	Skin_Mela	FERMT2
TRAPPC31	0.000934	0.660392	0.321	0.202	1	Skin_Mela	TRAPPC3
RTN2	0.000934	0.387086	0.103	0.036	1	Skin_Mela	RTN2
ZNF667.AS	0.000941	0.588666	0.205	0.103	1	Skin_Mela	ZNF667.AS1
PGAM16	0.000947	0.510137	0.359	0.229	1	Skin_Mela	PGAM1
LGALS3BP9	0.000949	0.586881	0.295	0.171	1	Skin_Mela	LGALS3BP
TSPAN152	0.000957	0.372692	0.115	0.043	1	Skin_Mela	TSPAN15
BLVRA1	0.000959	0.599858	0.218	0.113	1	Skin_Mela	BLVRA
G3BP16	0.00096	0.616687	0.282	0.162	1	Skin_Mela	G3BP1
DRAP14	0.000969	0.454162	0.487	0.335	1	Skin_Mela	DRAP1
LSM24	0.000999	0.600172	0.295	0.169	1	Skin_Mela	LSM2
COA31	0.001001	0.574139	0.295	0.178	1	Skin_Mela	COA3
RPL237	0.001003	0.251765	0.897	0.684	1	Skin_Mela	RPL23
BMP2K3	0.001005	0.463338	0.115	0.043	1	Skin_Mela	BMP2K
METTL21A	0.001014	0.439881	0.167	0.074	1	Skin_Mela	METTL21A
DTNBP1	0.001027	0.476315	0.154	0.066	1	Skin_Mela	DTNBP1
FNIP14	0.001046	0.585192	0.179	0.085	1	Skin_Mela	FNIP1
NDUFS59	0.001052	0.50805	0.577	0.455	1	Skin_Mela	NDUFS5
SNX73	0.001077	0.418694	0.154	0.067	1	Skin_Mela	SNX7
MRPL241	0.001101	0.486167	0.205	0.102	1	Skin_Mela	MRPL24
DMD5	0.001124	0.474795	0.128	0.051	1	Skin_Mela	DMD
RPP14	0.001128	0.409097	0.115	0.043	1	Skin_Mela	RPP14
UBL31	0.001134	0.558495	0.372	0.244	1	Skin_Mela	UBL3
TMEM128	0.001152	0.533805	0.154	0.068	1	Skin_Mela	TMEM128
DLC16	0.001157	0.350302	0.128	0.049	1	Skin_Mela	DLC1
ECH1	0.001168	0.570148	0.346	0.221	1	Skin_Mela	ECH1
ARMCX4	0.001172	0.273124	0.051	0.012	1	Skin_Mela	ARMCX4
TP53RK1	0.001175	0.435384	0.167	0.076	1	Skin_Mela	TP53RK
NDUFA111	0.001183	0.428387	0.564	0.438	1	Skin_Mela	NDUFA11
PA2G43	0.001199	0.470858	0.385	0.245	1	Skin_Mela	PA2G4
SLC25A57	0.001215	0.535609	0.513	0.369	1	Skin_Mela	SLC25A5
FAM76A1	0.001216	0.705596	0.141	0.059	1	Skin_Mela	FAM76A
DPM35	0.001223	0.591249	0.295	0.181	1	Skin_Mela	DPM3
SCCPDH	0.001231	0.43709	0.218	0.112	1	Skin_Mela	SCCPDH
PYCARD2	0.001237	0.366555	0.295	0.168	1	Skin_Mela	PYCARD
RP11.294J2	0.00124	0.275227	0.09	0.029	1	Skin_Mela	RP11.294J22.6
COL18A17	0.001258	0.347215	0.167	0.073	1	Skin_Mela	COL18A1
PBRM11	0.001259	0.560416	0.231	0.125	1	Skin_Mela	PBRM1

NDUFB6	0.001275	0.562287	0.346	0.223	1	Skin_Mela	NDUFB6
PRPF61	0.001284	0.491021	0.231	0.124	1	Skin_Mela	PRPF6
CDK19	0.001325	0.409874	0.128	0.052	1	Skin_Mela	CDK19
NDUFAB19	0.00135	0.478637	0.372	0.247	1	Skin_Mela	NDUFAB1
MRPS21	0.001351	0.584618	0.436	0.302	1	Skin_Mela	MRPS21
MDH11	0.001351	0.582063	0.308	0.191	1	Skin_Mela	MDH1
CERS21	0.001354	0.298176	0.179	0.083	1	Skin_Mela	CERS2
HIBADH2	0.001379	0.546877	0.167	0.077	1	Skin_Mela	HIBADH
RSU14	0.001434	0.478504	0.231	0.123	1	Skin_Mela	RSU1
FSTL14	0.001437	0.326724	0.282	0.155	1	Skin_Mela	FSTL1
ATP13A34	0.001442	0.447553	0.192	0.094	1	Skin_Mela	ATP13A3
OPTN1	0.001449	0.624598	0.308	0.194	1	Skin_Mela	OPTN
CHD910	0.001451	0.61964	0.282	0.167	1	Skin_Mela	CHD9
CBX36	0.00146	0.37183	0.333	0.202	1	Skin_Mela	CBX3
LINC00632	0.001478	0.254629	0.051	0.012	1	Skin_Mela	LINC00632
CD365	0.001501	0.396435	0.064	0.017	1	Skin_Mela	CD36
CIC1	0.001523	0.369262	0.115	0.044	1	Skin_Mela	CIC
LMCD16	0.001532	0.389443	0.179	0.085	1	Skin_Mela	LMCD1
SGPP11	0.001544	0.309841	0.077	0.024	1	Skin_Mela	SGPP1
SNRPD13	0.001548	0.381069	0.359	0.222	1	Skin_Mela	SNRPD1
ABHD22	0.001551	0.648538	0.218	0.116	1	Skin_Mela	ABHD2
SHF	0.001625	0.316681	0.051	0.012	1	Skin_Mela	SHF
PANX12	0.001634	0.297609	0.09	0.03	1	Skin_Mela	PANX1
MBNL111	0.001646	0.51523	0.295	0.171	1	Skin_Mela	MBNL1
ARAF	0.001647	0.362677	0.141	0.06	1	Skin_Mela	ARAF
ARHGAP24	0.001676	0.261554	0.103	0.037	1	Skin_Mela	ARHGAP24
PTEN5	0.001682	0.799779	0.231	0.128	1	Skin_Mela	PTEN
TECR6	0.00169	0.385777	0.474	0.331	1	Skin_Mela	TECR
PAPSS16	0.00173	0.646078	0.205	0.11	1	Skin_Mela	PAPSS1
NKD1	0.001774	0.308551	0.038	0.008	1	Skin_Mela	NKD1
SMPDL3A1	0.001776	0.578817	0.141	0.062	1	Skin_Mela	SMPDL3A
LURAP1	0.00179	0.419466	0.09	0.031	1	Skin_Mela	LURAP1
TTC7B	0.001802	0.30402	0.077	0.024	1	Skin_Mela	TTC7B
SAE1	0.001803	0.397611	0.167	0.077	1	Skin_Mela	SAE1
PNKD3	0.001827	0.536206	0.308	0.194	1	Skin_Mela	PNKD
MDH22	0.001831	0.573866	0.372	0.252	1	Skin_Mela	MDH2
PKM5	0.001832	0.398748	0.526	0.374	1	Skin_Mela	PKM
GSK3B	0.001863	0.516221	0.179	0.089	1	Skin_Mela	GSK3B
JAM32	0.001867	0.273604	0.077	0.024	1	Skin_Mela	JAM3
SLC35F63	0.001871	0.508379	0.128	0.053	1	Skin_Mela	SLC35F6
ATP6V0E2	0.001883	0.404365	0.167	0.077	1	Skin_Mela	ATP6V0E2
SCAMP41	0.001889	0.413033	0.141	0.061	1	Skin_Mela	SCAMP4
VPS293	0.001913	0.539993	0.397	0.282	1	Skin_Mela	VPS29
VKORC17	0.001915	0.478435	0.346	0.225	1	Skin_Mela	VKORC1
BAK1	0.001933	0.347932	0.103	0.038	1	Skin_Mela	BAK1
UBR42	0.00198	0.542335	0.179	0.088	1	Skin_Mela	UBR4
PSMA37	0.002023	0.58025	0.308	0.194	1	Skin_Mela	PSMA3

NME38	0.002025	0.435185	0.333	0.21	1	Skin_Mela	NME3
HUS1	0.002028	0.278225	0.115	0.045	1	Skin_Mela	HUS1
SIAH14	0.00203	0.53305	0.192	0.099	1	Skin_Mela	SIAH1
THAP51	0.002036	0.464377	0.167	0.078	1	Skin_Mela	THAP5
MEAF63	0.002056	0.472358	0.346	0.225	1	Skin_Mela	MEAF6
XRRA1	0.002064	0.484375	0.128	0.054	1	Skin_Mela	XRRA1
HPS3	0.002082	0.307749	0.09	0.031	1	Skin_Mela	HPS3
ID44	0.002095	0.405661	0.167	0.078	1	Skin_Mela	ID4
ESYT11	0.002132	0.397351	0.141	0.061	1	Skin_Mela	ESYT1
C8orf822	0.002157	0.450271	0.154	0.071	1	Skin_Mela	C8orf82
TUBG2	0.002187	0.272524	0.064	0.018	1	Skin_Mela	TUBG2
ASH2L	0.002188	0.343605	0.115	0.046	1	Skin_Mela	ASH2L
NDUFC16	0.002212	0.653509	0.385	0.282	1	Skin_Mela	NDUFC1
SAT23	0.002215	0.466979	0.321	0.205	1	Skin_Mela	SAT2
RAP2C3	0.002222	0.335152	0.128	0.053	1	Skin_Mela	RAP2C
ZFP36L18	0.002235	0.25129	0.641	0.469	1	Skin_Mela	ZFP36L1
NHP22	0.002241	0.425615	0.359	0.231	1	Skin_Mela	NHP2
FGF14.AS2	0.002263	0.279657	0.064	0.018	1	Skin_Mela	FGF14.AS2
SNX272	0.002335	0.518488	0.128	0.054	1	Skin_Mela	SNX27
HS1BP3	0.002361	0.2633	0.09	0.031	1	Skin_Mela	HS1BP3
AVPI12	0.002367	0.613163	0.231	0.128	1	Skin_Mela	AVPI1
DNAJB44	0.00242	0.473067	0.205	0.106	1	Skin_Mela	DNAJB4
MGME11	0.002431	0.411573	0.115	0.046	1	Skin_Mela	MGME1
ARL5A5	0.00245	0.53975	0.308	0.196	1	Skin_Mela	ARL5A
HMGN26	0.002483	0.387071	0.462	0.329	1	Skin_Mela	HMGN2
USP13	0.002494	0.268429	0.077	0.025	1	Skin_Mela	USP13
TTYH1	0.002528	0.589199	0.103	0.039	1	Skin_Mela	TTYH1
STAM	0.002529	0.42017	0.115	0.046	1	Skin_Mela	STAM
C19orf532	0.002533	0.472205	0.538	0.423	1	Skin_Mela	C19orf53
CRELD22	0.002534	0.517917	0.256	0.15	1	Skin_Mela	CRELD2
RNF1461	0.002543	0.425608	0.154	0.072	1	Skin_Mela	RNF146
POP52	0.002587	0.369175	0.192	0.098	1	Skin_Mela	POP5
EPS86	0.002606	0.39276	0.167	0.079	1	Skin_Mela	EPS8
RPP21	0.00262	0.252194	0.103	0.038	1	Skin_Mela	RPP21
RGS104	0.002629	0.313116	0.346	0.216	1	Skin_Mela	RGS10
ASAP21	0.002657	0.463517	0.167	0.08	1	Skin_Mela	ASAP2
XRN23	0.002687	0.600098	0.256	0.151	1	Skin_Mela	XRN2
GRWD11	0.002695	0.346186	0.115	0.046	1	Skin_Mela	GRWD1
C20orf273	0.002753	0.324762	0.154	0.07	1	Skin_Mela	C20orf27
AEBP23	0.002772	0.389354	0.141	0.063	1	Skin_Mela	AEBP2
HSBP12	0.002774	0.388467	0.564	0.432	1	Skin_Mela	HSBP1
ELMOD3	0.002786	0.332408	0.09	0.032	1	Skin_Mela	ELMOD3
SCRN2	0.002858	0.525946	0.141	0.064	1	Skin_Mela	SCRN2
KTN15	0.002873	0.488131	0.513	0.398	1	Skin_Mela	KTN1
REL8	0.00288	0.385057	0.308	0.187	1	Skin_Mela	REL
LIX1L5	0.002939	0.357913	0.115	0.047	1	Skin_Mela	LIX1L
RP11.14N7	0.00295	0.406725	0.103	0.039	1	Skin_Mela	RP11.14N7.2

CBR4	0.00295	0.449819	0.154	0.073	1	Skin_Mela	CBR4
SCAP	0.002958	0.452904	0.103	0.04	1	Skin_Mela	SCAP
MRPL524	0.002958	0.631791	0.333	0.225	1	Skin_Mela	MRPL52
ALG142	0.002983	0.556516	0.141	0.065	1	Skin_Mela	ALG14
SIRT2	0.003004	0.647871	0.192	0.101	1	Skin_Mela	SIRT2
C16orf522	0.003052	0.461166	0.115	0.047	1	Skin_Mela	C16orf52
CKS1B7	0.003062	0.709308	0.244	0.14	1	Skin_Mela	CKS1B
TMEM9B1	0.003067	0.663509	0.256	0.157	1	Skin_Mela	TMEM9B
ZSWIM7	0.003156	0.461603	0.192	0.101	1	Skin_Mela	ZSWIM7
COL6A33	0.003248	0.261547	0.192	0.092	1	Skin_Mela	COL6A3
AGA2	0.003253	0.456414	0.167	0.083	1	Skin_Mela	AGA
FAM127A5	0.003268	0.553607	0.179	0.092	1	Skin_Mela	FAM127A
BANP1	0.003273	0.498632	0.115	0.048	1	Skin_Mela	BANP
EDF12	0.003308	0.267421	0.756	0.625	1	Skin_Mela	EDF1
RP11.488L	0.003332	0.282545	0.064	0.019	1	Skin_Mela	RP11.488L18.10
XPO12	0.00346	0.589209	0.192	0.103	1	Skin_Mela	XPO1
ZNF330	0.003493	0.546426	0.192	0.103	1	Skin_Mela	ZNF330
AP2S12	0.003534	0.416915	0.462	0.342	1	Skin_Mela	AP2S1
LAMTOR21	0.00358	0.582145	0.333	0.234	1	Skin_Mela	LAMTOR2
ENOX2	0.003619	0.360195	0.064	0.019	1	Skin_Mela	ENOX2
SCARB211	0.003667	0.498808	0.256	0.157	1	Skin_Mela	SCARB2
NME1.NM	0.003669	0.265664	0.077	0.025	1	Skin_Mela	NME1.NME2
RGS123	0.003672	0.368197	0.103	0.04	1	Skin_Mela	RGS12
EIF4A26	0.003677	0.356507	0.603	0.486	1	Skin_Mela	EIF4A2
BAD1	0.003683	0.518416	0.295	0.19	1	Skin_Mela	BAD
MPV17	0.003691	0.475603	0.154	0.074	1	Skin_Mela	MPV17
LINC00657	0.003703	0.597412	0.308	0.204	1	Skin_Mela	LINC00657
AHNAK24	0.003728	0.560684	0.141	0.065	1	Skin_Mela	AHNAK2
VAMP84	0.003753	0.409974	0.538	0.409	1	Skin_Mela	VAMP8
TOLLIP	0.003789	0.469627	0.231	0.133	1	Skin_Mela	TOLLIP
NEDD87	0.003829	0.364862	0.551	0.423	1	Skin_Mela	NEDD8
NEK3	0.003829	0.279128	0.077	0.026	1	Skin_Mela	NEK3
TBC1D2B2	0.00387	0.281398	0.09	0.033	1	Skin_Mela	TBC1D2B
ENO14	0.003899	0.324088	0.538	0.412	1	Skin_Mela	ENO1
MANBAL2	0.003983	0.55065	0.179	0.094	1	Skin_Mela	MANBAL
TM2D13	0.004	0.673362	0.256	0.157	1	Skin_Mela	TM2D1
TMEM14B	0.004046	0.502414	0.359	0.253	1	Skin_Mela	TMEM14B
FAM3A	0.004047	0.549247	0.141	0.066	1	Skin_Mela	FAM3A
STX81	0.004067	0.355003	0.269	0.16	1	Skin_Mela	STX8
BNC21	0.004072	0.339454	0.064	0.02	1	Skin_Mela	BNC2
RAB40B	0.004086	0.519776	0.128	0.057	1	Skin_Mela	RAB40B
ZDHHC2	0.004109	0.492848	0.115	0.049	1	Skin_Mela	ZDHHC2
PSMB57	0.004145	0.436489	0.346	0.241	1	Skin_Mela	PSMB5
MYO1C6	0.004225	0.49292	0.244	0.146	1	Skin_Mela	MYO1C
TMEM256	0.00425	0.360872	0.333	0.22	1	Skin_Mela	TMEM256
ATP5A15	0.004326	0.388457	0.436	0.313	1	Skin_Mela	ATP5A1
PSMA13	0.004405	0.509503	0.359	0.254	1	Skin_Mela	PSMA1

MTCH13	0.004443	0.537375	0.346	0.243	1	Skin_Mela	MTCH1
CDK43	0.004448	0.518506	0.205	0.113	1	Skin_Mela	CDK4
NAE11	0.004459	0.367579	0.192	0.101	1	Skin_Mela	NAE1
UQCC2	0.00447	0.471225	0.256	0.155	1	Skin_Mela	UQCC2
GCSH6	0.004484	0.502184	0.218	0.126	1	Skin_Mela	GCSH
BMS12	0.004506	0.431614	0.141	0.066	1	Skin_Mela	BMS1
GLUD16	0.004548	0.412049	0.269	0.163	1	Skin_Mela	GLUD1
UBE2E14	0.004566	0.767658	0.231	0.139	1	Skin_Mela	UBE2E1
FBXO172	0.004588	0.330356	0.09	0.033	1	Skin_Mela	FBXO17
PEG3	0.004613	0.292124	0.09	0.034	1	Skin_Mela	PEG3
ZNF1063	0.004676	0.654812	0.231	0.139	1	Skin_Mela	ZNF106
DNAJC111	0.004676	0.295844	0.09	0.034	1	Skin_Mela	DNAJC11
CBX1	0.00468	0.453294	0.192	0.102	1	Skin_Mela	CBX1
RP11.108M	0.004694	0.289944	0.077	0.026	1	Skin_Mela	RP11.108M9.4
MCOLN11	0.00481	0.361933	0.115	0.049	1	Skin_Mela	MCOLN1
THRA2	0.004907	0.275342	0.103	0.041	1	Skin_Mela	THRA
GLO11	0.004951	0.748978	0.231	0.138	1	Skin_Mela	GLO1
SUB16	0.004982	0.28413	0.603	0.481	1	Skin_Mela	SUB1
FAM133B8	0.005004	0.759153	0.295	0.199	1	Skin_Mela	FAM133B
RBFA1	0.005033	0.440834	0.128	0.058	1	Skin_Mela	RBFA
PSMD9	0.005047	0.448008	0.205	0.113	1	Skin_Mela	PSMD9
RECQL3	0.005092	0.347376	0.128	0.057	1	Skin_Mela	RECQL
RAB2B	0.005146	0.557635	0.103	0.042	1	Skin_Mela	RAB2B
POLR2G	0.005188	0.368895	0.269	0.162	1	Skin_Mela	POLR2G
MAD2L2	0.005209	0.377828	0.192	0.103	1	Skin_Mela	MAD2L2
STX115	0.005246	0.423196	0.141	0.066	1	Skin_Mela	STX11
FEM1B1	0.005253	0.452963	0.192	0.103	1	Skin_Mela	FEM1B
ATAD3A1	0.005284	0.300561	0.09	0.034	1	Skin_Mela	ATAD3A
ANKRD52	0.00533	0.281686	0.051	0.014	1	Skin_Mela	ANKRD52
HNRNPR5	0.00535	0.563025	0.295	0.193	1	Skin_Mela	HNRNPR
TDRD32	0.00539	0.347436	0.103	0.042	1	Skin_Mela	TDRD3
EWSR1	0.005432	0.390401	0.295	0.189	1	Skin_Mela	EWSR1
ATP6V1G1	0.005476	0.387484	0.654	0.569	1	Skin_Mela	ATP6V1G1
ARPC26	0.005583	0.265712	0.679	0.575	1	Skin_Mela	ARPC2
ESD10	0.005613	0.585611	0.321	0.218	1	Skin_Mela	ESD
LINC00116	0.005669	0.568629	0.205	0.116	1	Skin_Mela	LINC00116
TUSC33	0.005779	0.365467	0.128	0.059	1	Skin_Mela	TUSC3
DCTN21	0.005789	0.449939	0.295	0.195	1	Skin_Mela	DCTN2
SPNS1	0.005802	0.261807	0.128	0.058	1	Skin_Mela	SPNS1
PFKL1	0.005806	0.468218	0.218	0.127	1	Skin_Mela	PFKL
KLC1	0.005834	0.479076	0.167	0.087	1	Skin_Mela	KLC1
CHAMP1	0.00585	0.310145	0.077	0.027	1	Skin_Mela	CHAMP1
IRF2BPL8	0.005855	0.485566	0.154	0.076	1	Skin_Mela	IRF2BPL
TMEM60	0.005862	0.504376	0.167	0.087	1	Skin_Mela	TMEM60
MGRN1	0.005901	0.321458	0.103	0.042	1	Skin_Mela	MGRN1
ZYG11B2	0.005911	0.426903	0.115	0.051	1	Skin_Mela	ZYG11B
ZNF7703	0.006061	0.327124	0.154	0.077	1	Skin_Mela	ZNF770

DDHD2	0.006071	0.304153	0.103	0.042	1	Skin_Mela	DDHD2
MBNL28	0.006071	0.590215	0.205	0.119	1	Skin_Mela	MBNL2
RAP1GAP2	0.006125	0.300754	0.09	0.035	1	Skin_Mela	RAP1GAP
MINK11	0.006128	0.324236	0.09	0.035	1	Skin_Mela	MINK1
TULP31	0.00614	0.42527	0.115	0.051	1	Skin_Mela	TULP3
DMTN2	0.006295	0.295838	0.064	0.021	1	Skin_Mela	DMTN
SH3BGRL9	0.006296	0.484925	0.359	0.245	1	Skin_Mela	SH3BGRL
NCBP2.AS2	0.006338	0.461962	0.205	0.115	1	Skin_Mela	NCBP2.AS2
SLC18B12	0.006391	0.345166	0.077	0.028	1	Skin_Mela	SLC18B1
SGCB4	0.006495	0.420027	0.141	0.068	1	Skin_Mela	SGCB
WWC31	0.00659	0.370344	0.103	0.043	1	Skin_Mela	WWC3
SRI5	0.006746	0.471523	0.346	0.242	1	Skin_Mela	SRI
PMF11	0.00687	0.489635	0.218	0.128	1	Skin_Mela	PMF1
POLR2K2	0.006904	0.422245	0.397	0.293	1	Skin_Mela	POLR2K
SEPT27	0.006908	0.527158	0.333	0.236	1	Skin_Mela	SEPT2
PACSIN2	0.006911	0.518556	0.179	0.098	1	Skin_Mela	PACSIN2
AIG13	0.006941	0.664373	0.192	0.109	1	Skin_Mela	AIG1
STX31	0.006944	0.468316	0.154	0.078	1	Skin_Mela	STX3
SSU72	0.007104	0.444236	0.423	0.322	1	Skin_Mela	SSU72
PIGA3	0.007415	0.417621	0.103	0.043	1	Skin_Mela	PIGA
NID14	0.007518	0.344408	0.077	0.028	1	Skin_Mela	NID1
MRFAP16	0.007529	0.483729	0.372	0.267	1	Skin_Mela	MRFAP1
PRPF38B6	0.00753	0.469448	0.295	0.193	1	Skin_Mela	PRPF38B
LPGAT13	0.007599	0.649785	0.167	0.09	1	Skin_Mela	LPGAT1
GTPBP6	0.007636	0.481528	0.154	0.078	1	Skin_Mela	GTPBP6
HIPK29	0.007698	0.433088	0.154	0.08	1	Skin_Mela	HIPK2
POT1	0.007712	0.344533	0.064	0.021	1	Skin_Mela	POT1
POGZ4	0.007765	0.476368	0.167	0.089	1	Skin_Mela	POGZ
DLG13	0.007884	0.564396	0.167	0.09	1	Skin_Mela	DLG1
SLC39A16	0.00797	0.470338	0.192	0.109	1	Skin_Mela	SLC39A1
KLF47	0.008041	0.405312	0.333	0.227	1	Skin_Mela	KLF4
MAGOH3	0.008113	0.58474	0.269	0.177	1	Skin_Mela	MAGOH
CYB5R11	0.008149	0.472773	0.205	0.119	1	Skin_Mela	CYB5R1
TMED1	0.008294	0.353983	0.154	0.079	1	Skin_Mela	TMED1
FSD1L	0.008295	0.395187	0.077	0.029	1	Skin_Mela	FSD1L
FGD5.AS13	0.008336	0.521818	0.192	0.11	1	Skin_Mela	FGD5.AS1
PPP2R2A3	0.008339	0.401191	0.218	0.129	1	Skin_Mela	PPP2R2A
HSPA93	0.008348	0.534768	0.256	0.165	1	Skin_Mela	HSPA9
TMEM179I	0.008358	0.466009	0.295	0.198	1	Skin_Mela	TMEM179B
COMMD1(	0.008415	0.514461	0.154	0.08	1	Skin_Mela	COMMD10
SNAPC5	0.008432	0.396802	0.141	0.07	1	Skin_Mela	SNAPC5
CTBP11	0.008446	0.458825	0.154	0.08	1	Skin_Mela	CTBP1
MCC3	0.008463	0.305532	0.09	0.036	1	Skin_Mela	MCC
SNRNP401	0.008489	0.44719	0.167	0.089	1	Skin_Mela	SNRNP40
HBS1L1	0.008504	0.455538	0.141	0.071	1	Skin_Mela	HBS1L
ANKRD39	0.008553	0.319057	0.115	0.052	1	Skin_Mela	ANKRD39
B3GNT25	0.008585	0.456614	0.141	0.069	1	Skin_Mela	B3GNT2

NARS3	0.008595	0.430445	0.231	0.142	1	Skin_Mela	NARS
SSH12	0.008643	0.325503	0.077	0.029	1	Skin_Mela	SSH1
RAB11FIP2	0.008676	0.267843	0.103	0.044	1	Skin_Mela	RAB11FIP2
BIN3	0.00869	0.298378	0.128	0.06	1	Skin_Mela	BIN3
CLDND16	0.008746	0.373146	0.295	0.191	1	Skin_Mela	CLDND1
EZR5	0.008759	0.409644	0.397	0.283	1	Skin_Mela	EZR
COL4A3BP	0.008815	0.47071	0.205	0.122	1	Skin_Mela	COL4A3BP
HNRNPH35	0.00886	0.490219	0.321	0.221	1	Skin_Mela	HNRNPH3
PTTG1IP	0.00888	0.447541	0.385	0.282	1	Skin_Mela	PTTG1IP
CORO73	0.008888	0.28431	0.09	0.036	1	Skin_Mela	CORO7
STOML21	0.008888	0.509289	0.269	0.177	1	Skin_Mela	STOML2
CBWD11	0.008991	0.328934	0.128	0.061	1	Skin_Mela	CBWD1
RHOB6	0.009053	0.294212	0.308	0.201	1	Skin_Mela	RHOB
GBA	0.009054	0.476117	0.103	0.045	1	Skin_Mela	GBA
CCDC43	0.009106	0.414304	0.103	0.045	1	Skin_Mela	CCDC43
NIT2	0.009179	0.359007	0.167	0.089	1	Skin_Mela	NIT2
RBBP72	0.009383	0.551364	0.244	0.156	1	Skin_Mela	RBBP7
THAP9.AS1	0.009477	0.380667	0.154	0.08	1	Skin_Mela	THAP9.AS1
SEC61B5	0.009524	0.279407	0.59	0.46	1	Skin_Mela	SEC61B
NDUFB53	0.009593	0.523897	0.333	0.245	1	Skin_Mela	NDUFB5
C14orf11	0.009608	0.458446	0.218	0.133	1	Skin_Mela	C14orf1
POMGNT1	0.009626	0.348804	0.103	0.045	1	Skin_Mela	POMGNT1
TMEM199	0.009687	0.258489	0.115	0.053	1	Skin_Mela	TMEM199
SSH2	0.009708	0.297011	0.154	0.079	1	Skin_Mela	SSH2
HNMT5	0.009767	0.370284	0.192	0.109	1	Skin_Mela	HNMT
TBC1D19	0.009778	0.255635	0.064	0.022	1	Skin_Mela	TBC1D19
AP3S14	0.009801	0.479943	0.385	0.292	1	Skin_Mela	AP3S1
RRP11	0.009805	0.407222	0.115	0.053	1	Skin_Mela	RRP1
FBXW21	0.009814	0.499626	0.167	0.091	1	Skin_Mela	FBXW2
PEX19	0.009879	0.343678	0.115	0.053	1	Skin_Mela	PEX19
CACYBP1	0.009955	0.353332	0.333	0.23	1	Skin_Mela	CACYBP
CDC376	0.009963	0.503647	0.372	0.275	1	Skin_Mela	CDC37
HBA21	0	10.42857	1	0.031	0	Skin_Lymp	HBA2
HBA11	0	10.05008	1	0.017	0	Skin_Lymp	HBA1
HBD1	0	5.85977	0.92	0.005	0	Skin_Lymp	HBD
AHSP	1.8E-165	1.802336	0.136	0.006	2.9E-161	Skin_Lymp	AHSP
SLC25A374	1.6E-155	3.895187	0.686	0.175	2.6E-151	Skin_Lymp	SLC25A37
SLC25A391	2.6E-103	3.601445	0.587	0.176	4.1E-99	Skin_Lymp	SLC25A39
SNCA7	7.65E-74	2.653204	0.269	0.045	1.22E-69	Skin_Lymp	SNCA
STRADB1	1.4E-59	2.584033	0.277	0.057	2.24E-55	Skin_Lymp	STRADB
GYPC5	9.6E-56	2.534282	0.508	0.203	1.53E-51	Skin_Lymp	GYPC
RP11.20D1	2.28E-55	0.858287	0.038	0.001	3.64E-51	Skin_Lymp	RP11.20D14.6
FBXO72	1.15E-42	2.718628	0.405	0.157	1.83E-38	Skin_Lymp	FBXO7
HBQ1	3.17E-34	1.002276	0.064	0.006	5.06E-30	Skin_Lymp	HBQ1
MYL41	5.71E-27	1.211565	0.072	0.009	9.11E-23	Skin_Lymp	MYL4
FKBP81	2.31E-22	2.435495	0.477	0.339	3.69E-18	Skin_Lymp	FKBP8
DMTN3	1.28E-19	1.340224	0.098	0.021	2.04E-15	Skin_Lymp	DMTN

FAM210B9	1.03E-17	2.29754	0.227	0.093	1.64E-13	Skin_Lymp	FAM210B
SERF27	1.1E-17	0.948663	0.739	0.823	1.75E-13	Skin_Lymp	SERF2
GMPR5	2.44E-13	1.502676	0.133	0.045	3.89E-09	Skin_Lymp	GMPR
PRDX53	4E-13	0.296119	0.174	0.493	6.37E-09	Skin_Lymp	PRDX5
PRDX64	6.3E-13	0.32011	0.114	0.375	1E-08	Skin_Lymp	PRDX6
SEC628	1.18E-12	0.260812	0.133	0.403	1.88E-08	Skin_Lymp	SEC62
UBB5	5.89E-11	1.845408	0.591	0.731	9.4E-07	Skin_Lymp	UBB
TRIM58	6.53E-11	0.697403	0.038	0.006	1.04E-06	Skin_Lymp	TRIM58
EMC3	2.46E-10	0.256987	0.064	0.252	3.92E-06	Skin_Lymp	EMC3
U2AF18	2.04E-08	0.327415	0.083	0.257	0.000325	Skin_Lymp	U2AF1
BNIP3L4	1.09E-07	0.487954	0.114	0.298	0.001736	Skin_Lymp	BNIP3L
C7orf73	1.61E-06	0.581313	0.125	0.298	0.025644	Skin_Lymp	C7orf73
PPM1A	1.72E-06	0.252746	0.064	0.194	0.027441	Skin_Lymp	PPM1A
NR4A17	5.88E-06	0.307864	0.11	0.258	0.093823	Skin_Lymp	NR4A1
SIK14	9.32E-06	0.256325	0.045	0.15	0.148599	Skin_Lymp	SIK1
EPB42	9.66E-06	0.498794	0.034	0.009	0.154093	Skin_Lymp	EPB42
AK14	1.01E-05	0.287485	0.042	0.143	0.160872	Skin_Lymp	AK1
C9orf782	2.08E-05	0.259412	0.064	0.175	0.331062	Skin_Lymp	C9orf78
RGS105	2.5E-05	0.407854	0.091	0.217	0.398701	Skin_Lymp	RGS10
ATP5E6	3.09E-05	0.371658	0.356	0.686	0.492362	Skin_Lymp	ATP5E
OAZ14	4.8E-05	0.901208	0.591	0.784	0.765032	Skin_Lymp	OAZ1
SBDS7	5.71E-05	0.641687	0.17	0.343	0.910267	Skin_Lymp	SBDS
C10orf107	6.15E-05	0.917492	0.049	0.018	0.981328	Skin_Lymp	C10orf107
RNF10	0.00011	0.635864	0.087	0.2	1	Skin_Lymp	RNF10
ASCC2	0.000122	0.366474	0.072	0.174	1	Skin_Lymp	ASCC2
NMB3	0.000347	0.272476	0.015	0.074	1	Skin_Lymp	NMB
UBA521	0.000768	0.454671	0.682	0.812	1	Skin_Lymp	UBA52
TESC5	0.001163	0.27998	0.045	0.114	1	Skin_Lymp	TESC
PIP4K2A8	0.00126	1.15631	0.102	0.061	1	Skin_Lymp	PIP4K2A
NUDT47	0.001358	0.497872	0.072	0.154	1	Skin_Lymp	NUDT4
DCAF121	0.001405	1.648702	0.155	0.11	1	Skin_Lymp	DCAF12
RAB40B1	0.001505	0.254192	0.011	0.058	1	Skin_Lymp	RAB40B
GJA44	0.001861	0.714042	0.042	0.017	1	Skin_Lymp	GJA4
MPP13	0.003456	0.935437	0.076	0.042	1	Skin_Lymp	MPP1
NFIX7	0.003923	0.52714	0.049	0.11	1	Skin_Lymp	NFIX
GLRX53	0.00473	0.669743	0.091	0.172	1	Skin_Lymp	GLRX5
TUBB2A7	0.005042	0.464452	0.053	0.114	1	Skin_Lymp	TUBB2A
MAP2K32	0.007552	0.667085	0.14	0.242	1	Skin_Lymp	MAP2K3
FCN1	0	4.504542	0.75	0.008	0	Blood_Mo	FCN1
TYROBP2	0	4.335557	0.978	0.106	0	Blood_Mo	TYROBP
AIF12	0	4.006534	0.875	0.099	0	Blood_Mo	AIF1
LST12	0	3.953617	0.794	0.083	0	Blood_Mo	LST1
TYMP4	0	3.58179	0.744	0.174	0	Blood_Mo	TYMP
HLA.DRB14	0	3.29666	0.926	0.204	0	Blood_Mo	HLA.DRB1
HLA.DRB53	0	3.290692	0.791	0.122	0	Blood_Mo	HLA.DRB5
LINC00936	0	3.264469	0.514	0.066	0	Blood_Mo	LINC00936
PYCARD3	0	3.229608	0.614	0.158	0	Blood_Mo	PYCARD

CD143	0	3.162277	0.527	0.098	0	Blood_Mo	CD14
LGALS22	0	3.079328	0.455	0.036	0	Blood_Mo	LGALS2
GPX12	0	3.06921	0.896	0.417	0	Blood_Mo	GPX1
CYBA4	0	3.054622	0.97	0.43	0	Blood_Mo	CYBA
CFP2	0	3.031899	0.428	0.019	0	Blood_Mo	CFP
FCER1G2	0	2.996796	0.707	0.098	0	Blood_Mo	FCER1G
S100A49	0	2.98022	0.996	0.454	0	Blood_Mo	S100A4
TMEM176I	0	2.961781	0.498	0.082	0	Blood_Mo	TMEM176B
TSPO6	0	2.903392	0.824	0.459	0	Blood_Mo	TSPO
LAMTOR44	0	2.606416	0.691	0.383	0	Blood_Mo	LAMTOR4
ATP5EP2	0	2.55038	0.391	0.048	0	Blood_Mo	ATP5EP2
SERPINA1	0	2.542634	0.368	0.056	0	Blood_Mo	SERPINA1
COTL14	0	2.533126	0.567	0.177	0	Blood_Mo	COTL1
IGSF62	0	2.517142	0.296	0.025	0	Blood_Mo	IGSF6
SPI12	0	2.474506	0.37	0.056	0	Blood_Mo	SPI1
MS4A6A2	0	2.469767	0.345	0.041	0	Blood_Mo	MS4A6A
IFI302	0	2.449877	0.313	0.013	0	Blood_Mo	IFI30
GMFG3	0	2.442965	0.621	0.201	0	Blood_Mo	GMFG
ARPC1B4	0	2.430702	0.631	0.286	0	Blood_Mo	ARPC1B
PSAP5	0	2.373551	0.765	0.428	0	Blood_Mo	PSAP
COX4I11	0	2.358183	0.94	0.72	0	Blood_Mo	COX4I1
FTL5	0	2.32985	1	0.893	0	Blood_Mo	FTL
GSTP18	0	2.177385	0.818	0.552	0	Blood_Mo	GSTP1
HLA.DMA4	0	2.155033	0.413	0.134	0	Blood_Mo	HLA.DMA
MYO1F2	0	2.129061	0.261	0.039	0	Blood_Mo	MYO1F
CSF3R1	0	2.105827	0.223	0.011	0	Blood_Mo	CSF3R
ITGB24	0	2.078135	0.37	0.104	0	Blood_Mo	ITGB2
LY862	0	2.064473	0.249	0.035	0	Blood_Mo	LY86
EIF3K3	0	2.00167	0.692	0.515	0	Blood_Mo	EIF3K
CST37	0	1.98948	0.943	0.586	0	Blood_Mo	CST3
EMP38	0	1.975236	0.65	0.345	0	Blood_Mo	EMP3
ATP5G24	0	1.969978	0.758	0.569	0	Blood_Mo	ATP5G2
HLA.DPB13	0	1.948827	0.638	0.21	0	Blood_Mo	HLA.DPB1
FGR1	0	1.93355	0.223	0.024	0	Blood_Mo	FGR
HLA.DQB14	0	1.915722	0.435	0.125	0	Blood_Mo	HLA.DQB1
GABARAP	0	1.915155	0.581	0.264	0	Blood_Mo	GABARAP
HLA.B4	0	1.91116	0.984	0.66	0	Blood_Mo	HLA.B
CD744	0	1.839627	0.923	0.351	0	Blood_Mo	CD74
OAZ15	0	1.807246	0.889	0.781	0	Blood_Mo	OAZ1
SH3BGRL3	0	1.804255	0.817	0.629	0	Blood_Mo	SH3BGRL3
RPL17	0	1.768688	0.781	0.52	0	Blood_Mo	RPL17
ATP5E7	0	1.676922	0.83	0.681	0	Blood_Mo	ATP5E
TMSB106	0	1.673425	0.999	0.876	0	Blood_Mo	TMSB10
HCK2	0	1.667524	0.162	0.018	0	Blood_Mo	HCK
RPS96	0	1.643966	0.996	0.833	0	Blood_Mo	RPS9
FPR11	0	1.60505	0.136	0.006	0	Blood_Mo	FPR1
HLA.C4	0	1.599562	0.925	0.681	0	Blood_Mo	HLA.C

SLC11A1	0	1.594945	0.141	0.014	0	Blood_Mo	SLC11A1
HLA.A3	0	1.585444	0.925	0.663	0	Blood_Mo	HLA.A
RPL36A5	0	1.575432	0.746	0.506	0	Blood_Mo	RPL36A
RPS218	0	1.561828	0.999	0.796	0	Blood_Mo	RPS2
PFN12	0	1.548409	0.856	0.58	0	Blood_Mo	PFN1
LGALS111	0	1.427807	0.81	0.402	0	Blood_Mo	LGALS1
TMSB4X7	0	1.400817	0.999	0.868	0	Blood_Mo	TMSB4X
RETN1	0	1.377183	0.1	0.005	0	Blood_Mo	RETN
HLA.DRA4	0	1.373424	0.724	0.242	0	Blood_Mo	HLA.DRA
SERF28	0	1.317405	0.881	0.822	0	Blood_Mo	SERF2
MYL69	0	1.295154	0.872	0.804	0	Blood_Mo	MYL6
LILRA2	0	1.258792	0.097	0.004	0	Blood_Mo	LILRA2
FAU9	0	1.208699	0.958	0.853	0	Blood_Mo	FAU
RPL13A5	0	1.205979	0.999	0.879	0	Blood_Mo	RPL13A
RPS148	0	1.146595	0.999	0.88	0	Blood_Mo	RPS14
CD300E	0	1.143095	0.086	0.003	0	Blood_Mo	CD300E
S100A69	0	1.043186	0.969	0.708	0	Blood_Mo	S100A6
RPL132	0	1.033227	0.998	0.917	0	Blood_Mo	RPL13
RPLP110	0	1.003766	0.998	0.914	0	Blood_Mo	RPLP1
ASGR2	0	0.955391	0.064	0.002	0	Blood_Mo	ASGR2
RPL194	0	0.943521	0.981	0.846	0	Blood_Mo	RPL19
LILRA5	0	0.86749	0.063	0.002	0	Blood_Mo	LILRA5
CD42	2.1E-303	1.72794	0.176	0.028	3.4E-299	Blood_Mo	CD4
PRAM1	1.3E-302	1.156868	0.085	0.007	2.1E-298	Blood_Mo	PRAM1
RPLP24	2.3E-302	0.852749	0.979	0.841	3.7E-298	Blood_Mo	RPLP2
PLCB21	3E-299	1.246025	0.107	0.011	4.7E-295	Blood_Mo	PLCB2
GPBAR1	8E-299	0.891451	0.064	0.004	1.3E-294	Blood_Mo	GPBAR1
ASGR11	5.3E-289	1.77118	0.169	0.027	8.5E-285	Blood_Mo	ASGR1
RPL127	4.9E-288	0.921713	0.97	0.85	7.9E-284	Blood_Mo	RPL12
CTSS3	8.8E-286	2.346561	0.475	0.21	1.4E-281	Blood_Mo	CTSS
PILRA2	1.6E-280	1.499147	0.137	0.019	2.6E-276	Blood_Mo	PILRA
LILRB22	1.3E-275	1.176962	0.097	0.01	2E-271	Blood_Mo	LILRB2
RPS74	8.3E-275	0.971102	0.927	0.814	1.3E-270	Blood_Mo	RPS7
CFL13	5.7E-270	1.441594	0.772	0.708	9.1E-266	Blood_Mo	CFL1
HLA.DQA1	9.1E-268	1.727685	0.342	0.102	1.5E-263	Blood_Mo	HLA.DQA1
HK3	5.1E-262	0.750904	0.052	0.003	8.2E-258	Blood_Mo	HK3
RPL267	6.2E-261	0.866124	0.957	0.833	9.9E-257	Blood_Mo	RPL26
KLF610	2.8E-260	1.996703	0.615	0.398	4.5E-256	Blood_Mo	KLF6
RPL114	2.9E-258	0.759289	0.979	0.851	4.7E-254	Blood_Mo	RPL11
UBA522	1.1E-257	0.961635	0.891	0.809	1.8E-253	Blood_Mo	UBA52
LSP13	2.7E-254	1.915797	0.482	0.226	4.4E-250	Blood_Mo	LSP1
TMEM176	9.8E-253	1.922799	0.244	0.061	1.6E-248	Blood_Mo	TMEM176A
LRRC251	3.6E-247	1.082735	0.085	0.008	5.7E-243	Blood_Mo	LRRC25
RPS164	3.8E-247	0.909171	0.926	0.834	6.1E-243	Blood_Mo	RPS16
HLA.DQA2	8.8E-247	1.721563	0.266	0.07	1.4E-242	Blood_Mo	HLA.DQA2
RPL184	9.5E-236	0.802185	0.949	0.877	1.5E-231	Blood_Mo	RPL18
FOS12	1.8E-232	1.0772	0.688	0.42	2.9E-228	Blood_Mo	FOS

EEF1D3	7.9E-230	1.105064	0.817	0.745	1.3E-225	Blood_Mo	EEF1D
PFDN57	2.9E-225	1.232337	0.785	0.726	4.7E-221	Blood_Mo	PFDN5
RPS33	2.9E-221	0.64649	0.967	0.813	4.6E-217	Blood_Mo	RPS3
VAMP85	8.7E-220	2.003906	0.579	0.406	1.4E-215	Blood_Mo	VAMP8
RPS65	5.3E-219	0.62012	0.97	0.822	8.4E-215	Blood_Mo	RPS6
CORO1A2	1E-218	1.418959	0.46	0.194	1.6E-214	Blood_Mo	CORO1A
RPL282	1.1E-216	0.709956	0.967	0.908	1.8E-212	Blood_Mo	RPL28
RPS197	5.9E-216	0.679353	0.979	0.873	9.4E-212	Blood_Mo	RPS19
MPEG12	4.2E-215	1.147844	0.104	0.014	6.6E-211	Blood_Mo	MPEG1
RPL87	1.3E-211	0.776978	0.939	0.847	2.1E-207	Blood_Mo	RPL8
HCST4	9.9E-196	1.534736	0.403	0.169	1.6E-191	Blood_Mo	HCST
LAPTM55	1.6E-195	1.64319	0.393	0.169	2.6E-191	Blood_Mo	LAPTM5
NCF22	1.5E-193	1.455747	0.126	0.022	2.4E-189	Blood_Mo	NCF2
RPS2810	1.6E-191	0.689937	0.96	0.88	2.5E-187	Blood_Mo	RPS28
CARD162	8.7E-191	1.93626	0.275	0.093	1.4E-186	Blood_Mo	CARD16
CLEC4E	3E-186	1.057027	0.074	0.008	4.7E-182	Blood_Mo	CLEC4E
FCGRT8	4.2E-186	2.086247	0.471	0.274	6.7E-182	Blood_Mo	FCGRT
RPS158	1.1E-184	0.654178	0.975	0.879	1.8E-180	Blood_Mo	RPS15
PSMA77	1.3E-184	2.018147	0.581	0.462	2.1E-180	Blood_Mo	PSMA7
NCF13	1.3E-181	1.468809	0.168	0.039	2E-177	Blood_Mo	NCF1
APOBR	1.8E-175	1.011933	0.072	0.008	2.9E-171	Blood_Mo	APOBR
CNPY31	8.6E-173	2.09756	0.316	0.13	1.4E-168	Blood_Mo	CNPY3
CRIP15	1.3E-169	1.852701	0.47	0.273	2.1E-165	Blood_Mo	CRIP1
RPS4X4	2.5E-168	0.564563	0.96	0.767	4E-164	Blood_Mo	RPS4X
CD300C	3.4E-168	0.596457	0.045	0.003	5.5E-164	Blood_Mo	CD300C
IER24	2E-167	1.333656	0.659	0.544	3.2E-163	Blood_Mo	IER2
SRGN5	3.4E-166	1.168652	0.451	0.219	5.5E-162	Blood_Mo	SRGN
CEBPD5	2E-162	1.513287	0.526	0.333	3.1E-158	Blood_Mo	CEBPD
RPS87	1.3E-156	0.653488	0.945	0.848	2.1E-152	Blood_Mo	RPS8
CTC.479C5	5.7E-152	0.917797	0.085	0.013	9.2E-148	Blood_Mo	CTC.479C5.12
HLA.DPA14	5E-150	1.068895	0.404	0.188	8E-146	Blood_Mo	HLA.DPA1
STXBP2	2.7E-148	2.2919	0.322	0.148	4.3E-144	Blood_Mo	STXBP2
C19orf38	1.6E-147	1.25746	0.096	0.017	2.5E-143	Blood_Mo	C19orf38
FOSB10	1.1E-146	1.687784	0.433	0.248	1.7E-142	Blood_Mo	FOSB
AOAH1	1.6E-146	1.098811	0.096	0.017	2.5E-142	Blood_Mo	AOAH
RPL3110	1.9E-141	0.589909	0.891	0.766	3.1E-137	Blood_Mo	RPL31
RPL77	3.9E-140	0.624389	0.911	0.795	6.3E-136	Blood_Mo	RPL7
SLC25A66	1.6E-138	1.293263	0.636	0.567	2.6E-134	Blood_Mo	SLC25A6
PARVG2	1.4E-135	1.303675	0.126	0.029	2.3E-131	Blood_Mo	PARVG
CD300LF	5.6E-135	0.771542	0.052	0.005	8.9E-131	Blood_Mo	CD300LF
OSCAR1	2.6E-134	0.778568	0.055	0.006	4.2E-130	Blood_Mo	OSCAR
EHBP1L13	2.4E-131	1.657728	0.173	0.052	3.9E-127	Blood_Mo	EHBP1L1
RNASET23	6.1E-130	1.911887	0.398	0.234	9.7E-126	Blood_Mo	RNASET2
RPL108	1.6E-129	0.468924	0.997	0.875	2.6E-125	Blood_Mo	RPL10
CPVL2	1.5E-128	1.606059	0.199	0.067	2.4E-124	Blood_Mo	CPVL
IGFLR1	2.7E-128	1.094793	0.105	0.022	4.3E-124	Blood_Mo	IGFLR1
RPL61	5.9E-126	0.614935	0.866	0.792	9.4E-122	Blood_Mo	RPL6

AP2S13	6.2E-126	2.034054	0.464	0.339	9.9E-122	Blood_Mo	AP2S1
LILRA1	1.4E-123	0.309903	0.021	0.001	2.2E-119	Blood_Mo	LILRA1
HLA.DMB3	9.7E-123	1.481867	0.194	0.065	1.5E-118	Blood_Mo	HLA.DMB
RPL18A6	1.7E-121	0.470384	0.968	0.82	2.7E-117	Blood_Mo	RPL18A
RPS266	2.5E-119	0.808077	0.78	0.742	4E-115	Blood_Mo	RPS26
CLEC4G	1.4E-118	0.388519	0.022	0.001	2.3E-114	Blood_Mo	CLEC4G
ATP5L21	3.6E-117	1.070848	0.093	0.019	5.7E-113	Blood_Mo	ATP5L2
C10orf542	1.3E-116	1.833115	0.324	0.168	2E-112	Blood_Mo	C10orf54
PSMB10	1.2E-115	1.658796	0.266	0.118	1.9E-111	Blood_Mo	PSMB10
CD300LB	1.5E-114	0.394878	0.024	0.001	2.3E-110	Blood_Mo	CD300LB
BLVRB2	2.4E-113	1.997729	0.423	0.285	3.8E-109	Blood_Mo	BLVRB
POU2F24	1.8E-112	1.496612	0.152	0.045	2.9E-108	Blood_Mo	POU2F2
BST26	3E-107	1.711749	0.33	0.179	4.8E-103	Blood_Mo	BST2
TNFRSF1B6	3.5E-107	1.436877	0.151	0.046	5.6E-103	Blood_Mo	TNFRSF1B
AGTRAP2	8.1E-107	1.86944	0.242	0.105	1.3E-102	Blood_Mo	AGTRAP
NLRP12	1.5E-104	0.44561	0.025	0.002	2.4E-100	Blood_Mo	NLRP12
STAB12	3.9E-104	0.956896	0.077	0.015	6.3E-100	Blood_Mo	STAB1
LILRB31	3.8E-103	0.654683	0.043	0.005	6.1E-99	Blood_Mo	LILRB3
NFE2	7.2E-101	0.866742	0.058	0.009	1.14E-96	Blood_Mo	NFE2
TREM1	1.6E-100	0.818494	0.058	0.009	2.51E-96	Blood_Mo	TREM1
TBXAS11	4.6E-100	1.218414	0.108	0.027	7.31E-96	Blood_Mo	TBXAS1
HIGD2A2	1.8E-99	1.881867	0.452	0.35	2.9E-95	Blood_Mo	HIGD2A
RASGRP41	5.1E-99	0.495089	0.032	0.003	8.07E-95	Blood_Mo	RASGRP4
RPL27A7	4.76E-97	0.472862	0.909	0.835	7.59E-93	Blood_Mo	RPL27A
CCL34	1.1E-96	0.870491	0.149	0.046	1.75E-92	Blood_Mo	CCL3
GAPDH10	5.87E-96	0.853962	0.725	0.765	9.37E-92	Blood_Mo	GAPDH
EIF4A18	1.25E-95	1.423207	0.471	0.351	2E-91	Blood_Mo	EIF4A1
VENTX1	3.16E-94	0.371626	0.022	0.001	5.03E-90	Blood_Mo	VENTX
ATP5D3	8.41E-93	1.649011	0.503	0.446	1.34E-88	Blood_Mo	ATP5D
SIRPB1	1.62E-92	0.58576	0.036	0.004	2.59E-88	Blood_Mo	SIRPB1
TNFSF13B4	5.06E-92	1.420262	0.132	0.041	8.07E-88	Blood_Mo	TNFSF13B
GRN6	5.68E-92	1.872321	0.418	0.31	9.06E-88	Blood_Mo	GRN
ATP6V0B8	2.31E-91	1.814333	0.464	0.386	3.68E-87	Blood_Mo	ATP6V0B
DUSP16	5.06E-91	0.908147	0.623	0.561	8.07E-87	Blood_Mo	DUSP1
CARD9	5.93E-90	0.702108	0.043	0.005	9.46E-86	Blood_Mo	CARD9
ATG16L21	1.63E-89	1.215992	0.108	0.029	2.59E-85	Blood_Mo	ATG16L2
RPS2411	1.97E-89	0.580378	0.874	0.839	3.14E-85	Blood_Mo	RPS24
MNDA2	5.19E-89	1.064224	0.102	0.027	8.27E-85	Blood_Mo	MNDA
UPK3A	8.98E-89	0.732124	0.047	0.007	1.43E-84	Blood_Mo	UPK3A
C20orf274	9.79E-89	1.50267	0.176	0.068	1.56E-84	Blood_Mo	C20orf27
NUP2141	1.51E-88	1.830764	0.204	0.087	2.4E-84	Blood_Mo	NUP214
ARHGAP43	1.29E-87	1.173958	0.114	0.032	2.06E-83	Blood_Mo	ARHGAP4
THEMIS23	3.01E-86	1.270521	0.126	0.039	4.8E-82	Blood_Mo	THEMIS2
CATSPER1	1.12E-85	0.878265	0.061	0.011	1.78E-81	Blood_Mo	CATSPER1
RPS117	1.85E-85	0.712314	0.782	0.778	2.95E-81	Blood_Mo	RPS11
LILRB41	1.43E-84	0.836906	0.066	0.013	2.27E-80	Blood_Mo	LILRB4
NBEAL18	2.65E-84	1.250874	0.44	0.318	4.23E-80	Blood_Mo	NBEAL1

CD332	2.15E-83	0.76728	0.06	0.011	3.42E-79	Blood_Mo	CD33
RPL7A10	2.2E-83	0.560047	0.861	0.813	3.51E-79	Blood_Mo	RPL7A
FAM26F1	1.69E-82	1.292597	0.133	0.044	2.7E-78	Blood_Mo	FAM26F
FMNL15	3.36E-82	1.365599	0.164	0.062	5.36E-78	Blood_Mo	FMNL1
MEFV	9.13E-80	0.320906	0.018	0.001	1.46E-75	Blood_Mo	MEFV
RP11.802E	1.26E-78	0.495877	0.033	0.004	2E-74	Blood_Mo	RP11.802E16.3
CD1D	5.41E-78	0.476095	0.036	0.004	8.63E-74	Blood_Mo	CD1D
PGLS2	5.43E-77	1.94037	0.366	0.261	8.66E-73	Blood_Mo	PGLS
RPL10A7	5.94E-77	0.466437	0.853	0.733	9.47E-73	Blood_Mo	RPL10A
RPL35A6	7.32E-76	0.453148	0.911	0.862	1.17E-71	Blood_Mo	RPL35A
ITGAX2	4.56E-75	0.775195	0.065	0.014	7.28E-71	Blood_Mo	ITGAX
TKT1	4.16E-74	1.881907	0.289	0.172	6.64E-70	Blood_Mo	TKT
PSME21	4.89E-72	1.780158	0.384	0.286	7.8E-68	Blood_Mo	PSME2
ARRB22	1.31E-71	1.466411	0.19	0.085	2.08E-67	Blood_Mo	ARRB2
C4orf483	2.66E-71	1.743117	0.255	0.141	4.25E-67	Blood_Mo	C4orf48
EIF14	3.95E-71	0.530418	0.864	0.91	6.3E-67	Blood_Mo	EIF1
SLC7A71	1.7E-70	1.152739	0.092	0.026	2.71E-66	Blood_Mo	SLC7A7
ZNF4672	2.26E-70	1.09529	0.088	0.024	3.61E-66	Blood_Mo	ZNF467
AMPD21	4.38E-70	1.136242	0.095	0.028	6.98E-66	Blood_Mo	AMPD2
LIMD21	1.12E-69	1.165843	0.305	0.172	1.79E-65	Blood_Mo	LIMD2
FOLR3	3.6E-69	0.65216	0.039	0.006	5.74E-65	Blood_Mo	FOLR3
RPL348	6.8E-69	0.326236	0.969	0.918	1.08E-64	Blood_Mo	RPL34
NME1.NM	2.88E-68	0.876553	0.087	0.024	4.6E-64	Blood_Mo	NME1.NME2
RPS187	1.48E-67	0.268273	0.986	0.915	2.37E-63	Blood_Mo	RPS18
NFAM1	2.24E-67	0.561175	0.037	0.005	3.57E-63	Blood_Mo	NFAM1
ACTB6	4.15E-67	0.332994	0.915	0.871	6.62E-63	Blood_Mo	ACTB
CD372	5.01E-67	0.977328	0.334	0.201	7.99E-63	Blood_Mo	CD37
RPS2310	1.95E-66	0.411765	0.922	0.812	3.1E-62	Blood_Mo	RPS23
NPC26	7.18E-66	1.419775	0.479	0.44	1.15E-61	Blood_Mo	NPC2
RPS102	7.57E-66	0.782704	0.631	0.608	1.21E-61	Blood_Mo	RPS10
IL1B2	1.28E-65	0.779608	0.12	0.041	2.05E-61	Blood_Mo	IL1B
MYO1G2	1.31E-65	1.099485	0.1	0.031	2.1E-61	Blood_Mo	MYO1G
RNASE2	8.8E-65	0.424686	0.03	0.004	1.4E-60	Blood_Mo	RNASE2
MARCO2	2.55E-64	0.786062	0.058	0.013	4.06E-60	Blood_Mo	MARCO
DOK24	2.74E-64	1.214931	0.15	0.061	4.37E-60	Blood_Mo	DOK2
RNASE62	1.54E-63	1.205159	0.113	0.039	2.46E-59	Blood_Mo	RNASE6
SF3B52	1.81E-63	1.7516	0.42	0.362	2.89E-59	Blood_Mo	SF3B5
CDA	7.53E-63	1.413545	0.132	0.05	1.2E-58	Blood_Mo	CDA
C1orf1622	3.91E-61	1.25746	0.189	0.089	6.23E-57	Blood_Mo	C1orf162
ARPC31	1.38E-60	1.094068	0.56	0.598	2.2E-56	Blood_Mo	ARPC3
C19orf533	6.78E-60	0.278526	0.167	0.428	1.08E-55	Blood_Mo	C19orf53
PRELID14	8.61E-60	1.716553	0.424	0.378	1.37E-55	Blood_Mo	PRELID1
TNNT11	1.22E-59	0.884782	0.061	0.015	1.94E-55	Blood_Mo	TNNT1
FXYD51	1.71E-59	1.037683	0.411	0.297	2.72E-55	Blood_Mo	FXYD5
ODF3B2	2.69E-59	1.516489	0.181	0.086	4.29E-55	Blood_Mo	ODF3B
ITGAL1	3.81E-59	0.746677	0.058	0.013	6.07E-55	Blood_Mo	ITGAL
UTF11	4.49E-59	0.592223	0.039	0.007	7.16E-55	Blood_Mo	UTF1

SYF21	7.19E-57	0.291524	0.117	0.332	1.15E-52	Blood_Mo	SYF2
KMT2E.AS1	8.9E-57	1.117844	0.102	0.035	1.42E-52	Blood_Mo	KMT2E.AS1
POLE45	2.75E-56	1.780585	0.263	0.164	4.39E-52	Blood_Mo	POLE4
SCIMP1	6.14E-56	0.64086	0.044	0.009	9.79E-52	Blood_Mo	SCIMP
BST11	1.6E-55	0.893164	0.066	0.017	2.56E-51	Blood_Mo	BST1
IRF51	2.16E-55	0.806328	0.056	0.013	3.45E-51	Blood_Mo	IRF5
CAPG5	3.27E-55	1.631182	0.265	0.165	5.22E-51	Blood_Mo	CAPG
RPLP02	4.73E-55	0.51172	0.767	0.799	7.54E-51	Blood_Mo	RPLP0
RAC18	4.73E-55	1.210077	0.519	0.549	7.55E-51	Blood_Mo	RAC1
LILRB11	1.31E-54	0.549292	0.039	0.007	2.09E-50	Blood_Mo	LILRB1
PSTPIP13	4.04E-54	0.888689	0.078	0.023	6.44E-50	Blood_Mo	PSTPIP1
CTD.2035E	5.41E-54	0.764348	0.063	0.016	8.63E-50	Blood_Mo	CTD.2035E11.3
SYK1	3.35E-53	1.060697	0.096	0.033	5.34E-49	Blood_Mo	SYK
CD300A1	3.44E-53	0.856736	0.071	0.02	5.49E-49	Blood_Mo	CD300A
C17orf49	3.22E-51	0.854334	0.081	0.026	5.14E-47	Blood_Mo	C17orf49
MRPL53	4.06E-51	0.948242	0.091	0.031	6.48E-47	Blood_Mo	MRPL53
SCAND1	4.46E-51	1.731473	0.392	0.343	7.11E-47	Blood_Mo	SCAND1
CEACAM4	5.95E-51	0.358436	0.02	0.002	9.5E-47	Blood_Mo	CEACAM4
CD483	1.12E-50	1.158231	0.222	0.122	1.79E-46	Blood_Mo	CD48
HCLS12	9.43E-50	1.224621	0.16	0.076	1.5E-45	Blood_Mo	HCLS1
ATP5L5	1.01E-49	1.021365	0.574	0.662	1.62E-45	Blood_Mo	ATP5L
ATP5F1	3.37E-49	0.277181	0.117	0.313	5.38E-45	Blood_Mo	ATP5F1
LAMTOR52	1.61E-47	0.414483	0.155	0.378	2.57E-43	Blood_Mo	LAMTOR5
HMHA11	2.7E-47	1.233227	0.144	0.066	4.3E-43	Blood_Mo	HMHA1
S100Z	4.87E-47	0.499049	0.02	0.002	7.76E-43	Blood_Mo	S100Z
ZDHHC1	2.21E-46	1.051413	0.08	0.027	3.52E-42	Blood_Mo	ZDHHC1
ARAP12	4.36E-46	1.153223	0.101	0.039	6.95E-42	Blood_Mo	ARAP1
ENY27	1.12E-45	0.349705	0.129	0.326	1.79E-41	Blood_Mo	ENY2
NDUFAB11	1.23E-44	0.303874	0.087	0.25	1.97E-40	Blood_Mo	NDUFAB1
WAS3	1.59E-44	0.941136	0.107	0.043	2.54E-40	Blood_Mo	WAS
ROMO13	1.97E-44	0.340923	0.123	0.315	3.15E-40	Blood_Mo	ROMO1
TPM37	5.26E-44	0.267543	0.139	0.337	8.39E-40	Blood_Mo	TPM3
BCAP312	6.08E-44	0.293241	0.113	0.293	9.7E-40	Blood_Mo	BCAP31
CSF1R2	9.09E-44	0.796356	0.066	0.02	1.45E-39	Blood_Mo	CSF1R
NRGN3	2.45E-43	0.771137	0.054	0.015	3.91E-39	Blood_Mo	NRGN
RELT2	5.01E-43	1.012656	0.083	0.03	8E-39	Blood_Mo	RELT
RPS138	5.22E-43	0.386355	0.851	0.817	8.32E-39	Blood_Mo	RPS13
RBM8A7	7.38E-43	0.368827	0.144	0.347	1.18E-38	Blood_Mo	RBM8A
CYTH43	7.39E-43	0.973246	0.082	0.029	1.18E-38	Blood_Mo	CYTH4
TXNL4A2	1.16E-42	0.275239	0.081	0.237	1.85E-38	Blood_Mo	TXNL4A
SEC11A9	1.77E-42	0.343606	0.13	0.32	2.83E-38	Blood_Mo	SEC11A
GNB2L12	1.92E-42	0.423116	0.761	0.753	3.06E-38	Blood_Mo	GNB2L1
FCGR1A1	3.48E-42	0.741715	0.06	0.018	5.55E-38	Blood_Mo	FCGR1A
NANS5	5.49E-42	0.325419	0.081	0.234	8.76E-38	Blood_Mo	NANS
MRFAP17	9.39E-42	0.342252	0.102	0.27	1.5E-37	Blood_Mo	MRFAP1
TRAPPC32	1.16E-41	0.283565	0.064	0.205	1.84E-37	Blood_Mo	TRAPPC3
NENF8	1.3E-41	0.337246	0.14	0.333	2.07E-37	Blood_Mo	NENF

PFDN28	4.13E-41	0.275934	0.109	0.279	6.59E-37	Blood_Mo	PFDN2
RASSF42	8.03E-41	1.111683	0.089	0.034	1.28E-36	Blood_Mo	RASSF4
JUND8	9.53E-41	1.236124	0.467	0.464	1.52E-36	Blood_Mo	JUND
NOP103	1.2E-40	0.360103	0.163	0.374	1.91E-36	Blood_Mo	NOP10
TUFM2	1.64E-40	0.294826	0.12	0.296	2.61E-36	Blood_Mo	TUFM
NDUFA131	1.7E-40	1.283065	0.411	0.387	2.72E-36	Blood_Mo	NDUFA13
DHRS4L21	2.73E-40	1.381784	0.154	0.08	4.36E-36	Blood_Mo	DHRS4L2
CLEC4A2	3.2E-40	0.791062	0.059	0.018	5.1E-36	Blood_Mo	CLEC4A
RGS191	2.32E-39	1.097137	0.135	0.065	3.7E-35	Blood_Mo	RGS19
GUK16	2.65E-39	0.275898	0.271	0.564	4.22E-35	Blood_Mo	GUK1
H2AFV3	2.99E-39	0.393127	0.12	0.295	4.76E-35	Blood_Mo	H2AFV
RBP72	3.28E-39	1.510499	0.158	0.085	5.24E-35	Blood_Mo	RBP7
RTF13	3.88E-39	0.365175	0.093	0.249	6.19E-35	Blood_Mo	RTF1
METTL93	4.1E-39	0.307199	0.08	0.225	6.53E-35	Blood_Mo	METTL9
TCF256	4.93E-39	0.352247	0.122	0.298	7.86E-35	Blood_Mo	TCF25
GPR162	1.03E-38	0.32982	0.019	0.003	1.64E-34	Blood_Mo	GPR162
TMED93	1.15E-38	0.338642	0.122	0.296	1.83E-34	Blood_Mo	TMED9
ATPIF12	2.38E-38	0.319016	0.184	0.399	3.79E-34	Blood_Mo	ATPIF1
TRMT1128	3.17E-38	0.294368	0.172	0.379	5.06E-34	Blood_Mo	TRMT112
PLAUR4	3.65E-38	1.23262	0.199	0.118	5.82E-34	Blood_Mo	PLAUR
LMAN22	7.65E-38	0.268107	0.102	0.259	1.22E-33	Blood_Mo	LMAN2
DOK31	9.54E-38	0.489783	0.034	0.007	1.52E-33	Blood_Mo	DOK3
CECR13	2.09E-37	1.019721	0.094	0.039	3.34E-33	Blood_Mo	CECR1
SNRPB6	2.15E-37	0.368796	0.149	0.338	3.43E-33	Blood_Mo	SNRPB
ERGIC32	3.41E-37	0.331975	0.136	0.315	5.44E-33	Blood_Mo	ERGIC3
SULT1A1	5.3E-37	1.068969	0.088	0.035	8.45E-33	Blood_Mo	SULT1A1
MSRB1	7.75E-37	1.327119	0.117	0.055	1.24E-32	Blood_Mo	MSRB1
ZGLP1	1.31E-36	0.553252	0.033	0.007	2.08E-32	Blood_Mo	ZGLP1
GPSM32	1.9E-36	1.022133	0.259	0.171	3.03E-32	Blood_Mo	GPSM3
TRPM2	2.84E-36	0.405267	0.023	0.004	4.53E-32	Blood_Mo	TRPM2
RENBP2	5.14E-36	0.822211	0.061	0.02	8.2E-32	Blood_Mo	RENBP
BUD31	5.63E-36	0.545909	0.132	0.308	8.98E-32	Blood_Mo	BUD31
CCNL15	7.71E-36	0.328499	0.155	0.344	1.23E-31	Blood_Mo	CCNL1
PSMB32	1.69E-35	1.639412	0.37	0.344	2.69E-31	Blood_Mo	PSMB3
SLC15A31	2.14E-35	0.793325	0.058	0.019	3.41E-31	Blood_Mo	SLC15A3
NLRP32	3E-35	0.473986	0.032	0.007	4.79E-31	Blood_Mo	NLRP3
COX175	3.95E-35	0.363521	0.118	0.28	6.29E-31	Blood_Mo	COX17
PADI4	6.46E-35	0.477282	0.031	0.007	1.03E-30	Blood_Mo	PADI4
MKKS3	7.45E-35	0.273279	0.049	0.163	1.19E-30	Blood_Mo	MKKS
PSMB95	3.18E-34	1.36942	0.243	0.168	5.06E-30	Blood_Mo	PSMB9
DPM36	8.46E-34	0.302951	0.063	0.184	1.35E-29	Blood_Mo	DPM3
CAMK15	1.03E-33	1.009898	0.084	0.034	1.64E-29	Blood_Mo	CAMK1
KCNAB22	4.18E-33	0.888059	0.067	0.024	6.67E-29	Blood_Mo	KCNAB2
AUP12	4.54E-33	0.291072	0.078	0.207	7.24E-29	Blood_Mo	AUP1
TRAPPC2L2	4.82E-33	0.422703	0.073	0.2	7.69E-29	Blood_Mo	TRAPPC2L
MRPL142	7.08E-33	0.424122	0.092	0.231	1.13E-28	Blood_Mo	MRPL14
NCF42	1.41E-32	0.946836	0.094	0.041	2.25E-28	Blood_Mo	NCF4

PSENEN1	1.43E-32	0.396895	0.086	0.22	2.27E-28	Blood_Mo	PSENEN
RP3.395M:	2.41E-32	0.70655	0.054	0.018	3.84E-28	Blood_Mo	RP3.395M20.12
CD682	3.47E-32	1.300202	0.189	0.118	5.53E-28	Blood_Mo	CD68
VKORC18	4.2E-32	0.324881	0.092	0.228	6.7E-28	Blood_Mo	VKORC1
ABI33	6.35E-32	0.787129	0.078	0.031	1.01E-27	Blood_Mo	ABI3
PSMB71	1.03E-31	0.501147	0.14	0.309	1.64E-27	Blood_Mo	PSMB7
SMIM72	1.3E-31	0.382288	0.066	0.184	2.07E-27	Blood_Mo	SMIM7
COMMD11	1.71E-31	0.341847	0.057	0.169	2.73E-27	Blood_Mo	COMMD1
EIF62	2.95E-31	0.509614	0.119	0.273	4.71E-27	Blood_Mo	EIF6
SYNGR24	3.15E-31	0.442347	0.099	0.238	5.03E-27	Blood_Mo	SYNGR2
SNRPD33	5.92E-31	0.41063	0.098	0.235	9.45E-27	Blood_Mo	SNRPD3
ANP32B	6.54E-31	0.337013	0.144	0.309	1.04E-26	Blood_Mo	ANP32B
MCL17	6.7E-31	1.396729	0.388	0.381	1.07E-26	Blood_Mo	MCL1
UQCR103	8.93E-31	0.406386	0.219	0.444	1.42E-26	Blood_Mo	UQCR10
DNAJC154	1.09E-30	0.269415	0.073	0.192	1.73E-26	Blood_Mo	DNAJC15
PHB23	1.12E-30	0.254576	0.077	0.199	1.79E-26	Blood_Mo	PHB2
EIF3F1	1.41E-30	0.339853	0.225	0.445	2.24E-26	Blood_Mo	EIF3F
HNRNPM4	1.54E-30	0.269677	0.09	0.22	2.46E-26	Blood_Mo	HNRNPM
RSBN1L2	2.38E-30	0.292053	0.064	0.177	3.79E-26	Blood_Mo	RSBN1L
EMC61	2.44E-30	0.496918	0.086	0.215	3.89E-26	Blood_Mo	EMC6
MRPS211	2.52E-30	0.424784	0.141	0.306	4.02E-26	Blood_Mo	MRPS21
PHPT111	4.35E-30	0.417368	0.156	0.329	6.95E-26	Blood_Mo	PHPT1
NDUFB101	6.04E-30	0.44193	0.191	0.39	9.63E-26	Blood_Mo	NDUFB10
TNFAIP8L2	6.24E-30	0.588476	0.045	0.014	9.95E-26	Blood_Mo	TNFAIP8L2
RPAIN	6.36E-30	0.300131	0.047	0.148	1.01E-25	Blood_Mo	RPAIN
LGALS91	7.76E-30	1.36595	0.178	0.112	1.24E-25	Blood_Mo	LGALS9
ASAH15	7.93E-30	0.486866	0.128	0.282	1.26E-25	Blood_Mo	ASAH1
SF18	8.54E-30	0.38558	0.101	0.238	1.36E-25	Blood_Mo	SF1
C20orf242	8.66E-30	1.398116	0.22	0.154	1.38E-25	Blood_Mo	C20orf24
YWHAB6	1.03E-29	0.476889	0.206	0.418	1.64E-25	Blood_Mo	YWHAB
IFNGR21	1.14E-29	0.300871	0.084	0.208	1.82E-25	Blood_Mo	IFNGR2
UBXN111	1.44E-29	1.174867	0.119	0.062	2.3E-25	Blood_Mo	UBXN11
MRPL211	1.81E-29	0.322761	0.066	0.178	2.88E-25	Blood_Mo	MRPL21
PIN11	1.95E-29	0.286628	0.085	0.208	3.11E-25	Blood_Mo	PIN1
RPL238	2.54E-29	0.653437	0.621	0.685	4.05E-25	Blood_Mo	RPL23
RGS26	2.76E-29	1.195431	0.162	0.096	4.41E-25	Blood_Mo	RGS2
SELPLG2	3.37E-29	0.848648	0.075	0.031	5.37E-25	Blood_Mo	SELPLG
TRA2B5	3.83E-29	0.404294	0.136	0.293	6.11E-25	Blood_Mo	TRA2B
C14orf24	4.01E-29	0.477727	0.225	0.454	6.4E-25	Blood_Mo	C14orf2
SAMHD13	4.73E-29	1.145295	0.127	0.068	7.54E-25	Blood_Mo	SAMHD1
FAM200B1	5.34E-29	0.295296	0.056	0.16	8.51E-25	Blood_Mo	FAM200B
PID13	5.71E-29	0.975965	0.084	0.037	9.11E-25	Blood_Mo	PID1
SCO22	6.14E-29	1.40206	0.153	0.09	9.79E-25	Blood_Mo	SCO2
UBA1	8.05E-29	0.253064	0.056	0.16	1.28E-24	Blood_Mo	UBA1
MGMT2	1.54E-28	0.317931	0.059	0.165	2.46E-24	Blood_Mo	MGMT
VAMP21	2.33E-28	0.296857	0.169	0.342	3.72E-24	Blood_Mo	VAMP2
CAPNS13	2.66E-28	0.460039	0.113	0.253	4.25E-24	Blood_Mo	CAPNS1

BRK13	4.96E-28	0.427514	0.235	0.46	7.92E-24	Blood_Mo	BRK1
ITGAM2	5.94E-28	0.670958	0.046	0.015	9.48E-24	Blood_Mo	ITGAM
RPS207	5.96E-28	0.385576	0.762	0.796	9.51E-24	Blood_Mo	RPS20
MDH23	6.29E-28	0.456472	0.116	0.256	1E-23	Blood_Mo	MDH2
RABGAP1L	9.27E-28	0.358237	0.059	0.162	1.48E-23	Blood_Mo	RABGAP1L
MRPS163	1.05E-27	0.338939	0.056	0.158	1.67E-23	Blood_Mo	MRPS16
POLR2F5	1.28E-27	0.488449	0.116	0.256	2.05E-23	Blood_Mo	POLR2F
RPL2413	1.58E-27	0.458381	0.727	0.804	2.52E-23	Blood_Mo	RPL24
SAT24	1.75E-27	1.643986	0.258	0.204	2.79E-23	Blood_Mo	SAT2
MZT2B5	2.9E-27	0.322333	0.243	0.466	4.62E-23	Blood_Mo	MZT2B
CRELD23	2.91E-27	0.267913	0.054	0.153	4.64E-23	Blood_Mo	CRELD2
FLNA11	2.93E-27	1.254242	0.213	0.148	4.67E-23	Blood_Mo	FLNA
SEC61A16	3.7E-27	0.296765	0.055	0.154	5.9E-23	Blood_Mo	SEC61A1
CLEC12A	4.85E-27	0.351204	0.023	0.005	7.73E-23	Blood_Mo	CLEC12A
DPEP22	5.29E-27	0.757108	0.057	0.021	8.44E-23	Blood_Mo	DPEP2
STAC31	6.79E-27	0.516318	0.035	0.01	1.08E-22	Blood_Mo	STAC3
BLOC1S12	7.65E-27	0.547916	0.17	0.346	1.22E-22	Blood_Mo	BLOC1S1
NDUFB12	8.64E-27	0.533414	0.182	0.367	1.38E-22	Blood_Mo	NDUFB1
TCIRG12	8.66E-27	1.23668	0.129	0.072	1.38E-22	Blood_Mo	TCIRG1
FES2	1.44E-26	0.956264	0.08	0.036	2.3E-22	Blood_Mo	FES
TMEM141	1.59E-26	0.415338	0.064	0.168	2.53E-22	Blood_Mo	TMEM141
TRMT12	1.88E-26	1.091338	0.105	0.054	3E-22	Blood_Mo	TRMT1
TMEM179I	2.01E-26	0.367463	0.085	0.201	3.21E-22	Blood_Mo	TMEM179B
MRPL512	2.71E-26	0.512131	0.136	0.286	4.32E-22	Blood_Mo	MRPL51
TIMP19	2.83E-26	0.837928	0.412	0.382	4.51E-22	Blood_Mo	TIMP1
ATOX18	2.98E-26	0.410961	0.132	0.277	4.75E-22	Blood_Mo	ATOX1
C11orf21	8.23E-26	0.486837	0.032	0.009	1.31E-21	Blood_Mo	C11orf21
TPGS11	9.48E-26	1.345026	0.158	0.099	1.51E-21	Blood_Mo	TPGS1
SLC24A4	1.04E-25	0.373286	0.02	0.004	1.66E-21	Blood_Mo	SLC24A4
CWC251	1.45E-25	0.256302	0.037	0.121	2.31E-21	Blood_Mo	CWC25
PPP4C	1.92E-25	0.476717	0.14	0.29	3.07E-21	Blood_Mo	PPP4C
MOB3A2	3E-25	1.083167	0.094	0.046	4.79E-21	Blood_Mo	MOB3A
RP11.820I1	3.35E-25	0.381384	0.024	0.005	5.35E-21	Blood_Mo	RP11.820I16.4
MAFG3	3.82E-25	0.256393	0.041	0.126	6.08E-21	Blood_Mo	MAFG
ATP6AP15	4.13E-25	0.390441	0.064	0.163	6.59E-21	Blood_Mo	ATP6AP1
LAT22	7.82E-25	0.798254	0.075	0.033	1.25E-20	Blood_Mo	LAT2
WDR611	9.43E-25	0.252046	0.045	0.131	1.5E-20	Blood_Mo	WDR61
C11orf311	1.15E-24	1.396264	0.36	0.354	1.83E-20	Blood_Mo	C11orf31
SRA13	1.32E-24	0.500282	0.078	0.185	2.11E-20	Blood_Mo	SRA1
CTA.384D8	1.45E-24	0.299163	0.018	0.004	2.31E-20	Blood_Mo	CTA.384D8.34
SUPT5H	4.27E-24	0.375563	0.044	0.129	6.8E-20	Blood_Mo	SUPT5H
PSMD71	5.55E-24	0.55074	0.098	0.215	8.85E-20	Blood_Mo	PSMD7
LRRFIP17	6.16E-24	0.514231	0.194	0.377	9.83E-20	Blood_Mo	LRRFIP1
CNPY24	7.17E-24	0.390966	0.094	0.208	1.14E-19	Blood_Mo	CNPY2
TIMM8B1	8.43E-24	0.561545	0.106	0.229	1.34E-19	Blood_Mo	TIMM8B
TOMM222	8.64E-24	0.443554	0.099	0.216	1.38E-19	Blood_Mo	TOMM22
CTB.55O6.	9.52E-24	0.385006	0.024	0.006	1.52E-19	Blood_Mo	CTB.55O6.12

EIF3G	1.23E-23	0.428491	0.215	0.404	1.97E-19	Blood_Mo	EIF3G
NDUFC26	1.36E-23	0.500482	0.202	0.391	2.17E-19	Blood_Mo	NDUFC2
PTAFR2	1.46E-23	0.663809	0.047	0.017	2.33E-19	Blood_Mo	PTAFR
RBM42	1.64E-23	0.370191	0.062	0.156	2.61E-19	Blood_Mo	RBM42
IDH3G	1.89E-23	0.395328	0.074	0.176	3.02E-19	Blood_Mo	IDH3G
MIEN1	2.63E-23	0.629824	0.145	0.292	4.2E-19	Blood_Mo	MIEN1
DNAJC73	3.64E-23	0.365282	0.066	0.162	5.81E-19	Blood_Mo	DNAJC7
HRH22	4.04E-23	0.475511	0.029	0.008	6.45E-19	Blood_Mo	HRH2
EIF3M1	5.14E-23	0.483826	0.129	0.263	8.2E-19	Blood_Mo	EIF3M
OAZ26	5.58E-23	0.412386	0.078	0.18	8.89E-19	Blood_Mo	OAZ2
CDK2AP23	6.27E-23	0.332702	0.071	0.168	9.99E-19	Blood_Mo	CDK2AP2
POLD4	7.09E-23	1.465959	0.265	0.22	1.13E-18	Blood_Mo	POLD4
NDUFS66	7.55E-23	0.582457	0.167	0.328	1.2E-18	Blood_Mo	NDUFS6
LSM61	8.76E-23	0.329145	0.056	0.145	1.4E-18	Blood_Mo	LSM6
SYNGR14	1.19E-22	0.416801	0.056	0.144	1.89E-18	Blood_Mo	SYNGR1
AP3D1	1.36E-22	0.297667	0.047	0.13	2.17E-18	Blood_Mo	AP3D1
VSTM1	1.5E-22	0.26298	0.014	0.002	2.39E-18	Blood_Mo	VSTM1
SLC2A6	1.54E-22	0.537892	0.036	0.012	2.45E-18	Blood_Mo	SLC2A6
PSMD4	1.76E-22	0.609573	0.114	0.236	2.81E-18	Blood_Mo	PSMD4
IMP43	2.15E-22	0.301225	0.052	0.137	3.43E-18	Blood_Mo	IMP4
EPN1	2.61E-22	0.305021	0.048	0.131	4.17E-18	Blood_Mo	EPN1
SF3B21	3E-22	0.507458	0.111	0.23	4.78E-18	Blood_Mo	SF3B2
TMEM208:	3.65E-22	0.495462	0.092	0.199	5.82E-18	Blood_Mo	TMEM208
SIGLEC91	3.82E-22	0.283575	0.019	0.004	6.09E-18	Blood_Mo	SIGLEC9
RAB11FIP1	4.87E-22	0.434973	0.095	0.204	7.77E-18	Blood_Mo	RAB11FIP1
PSMB16	6.42E-22	0.618128	0.213	0.405	1.02E-17	Blood_Mo	PSMB1
RP11.156E	6.63E-22	0.689642	0.05	0.019	1.06E-17	Blood_Mo	RP11.156E8.1
MRPL525	7.22E-22	0.505455	0.109	0.227	1.15E-17	Blood_Mo	MRPL52
PIH1D1	7.78E-22	0.356515	0.063	0.152	1.24E-17	Blood_Mo	PIH1D1
PREX13	1.33E-21	0.644413	0.053	0.021	2.12E-17	Blood_Mo	PREX1
LTB4R	2.18E-21	0.708359	0.052	0.021	3.48E-17	Blood_Mo	LTB4R
EMC103	2.39E-21	0.434863	0.065	0.155	3.81E-17	Blood_Mo	EMC10
AAMP1	2.55E-21	0.308089	0.056	0.141	4.07E-17	Blood_Mo	AAMP
LINC01004	3.1E-21	0.315371	0.021	0.005	4.95E-17	Blood_Mo	LINC01004
SERPINB61	3.47E-21	0.483924	0.111	0.227	5.53E-17	Blood_Mo	SERPINB6
NHP23	4.8E-21	0.467327	0.115	0.234	7.66E-17	Blood_Mo	NHP2
PSMA53	5.61E-21	0.3818	0.085	0.185	8.95E-17	Blood_Mo	PSMA5
BABAM1	7.14E-21	0.410112	0.061	0.147	1.14E-16	Blood_Mo	BABAM1
ARPC27	7.3E-21	0.383686	0.319	0.58	1.16E-16	Blood_Mo	ARPC2
RPL276	8.03E-21	0.416324	0.698	0.752	1.28E-16	Blood_Mo	RPL27
C16orf131	1.1E-20	0.516004	0.104	0.215	1.76E-16	Blood_Mo	C16orf13
SRRM211	1.23E-20	0.598027	0.186	0.352	1.96E-16	Blood_Mo	SRRM2
RPS6KA41	1.23E-20	0.781303	0.059	0.025	1.97E-16	Blood_Mo	RPS6KA4
MMP171	1.25E-20	0.295564	0.017	0.004	2E-16	Blood_Mo	MMP17
EMILIN23	1.32E-20	0.809656	0.066	0.03	2.1E-16	Blood_Mo	EMILIN2
MPHOSPH.	1.32E-20	0.601708	0.13	0.257	2.1E-16	Blood_Mo	MPHOSPH8
ERP291	1.35E-20	1.308527	0.357	0.355	2.15E-16	Blood_Mo	ERP29

NDUFS510	1.35E-20	0.478508	0.248	0.46	2.16E-16	Blood_Mo	NDUFS5
RRP12	1.72E-20	0.831146	0.059	0.026	2.74E-16	Blood_Mo	RRP12
MRPL28	2.31E-20	0.394587	0.064	0.15	3.69E-16	Blood_Mo	MRPL28
OS97	3.06E-20	0.483025	0.114	0.229	4.89E-16	Blood_Mo	OS9
COMT4	3.31E-20	1.59633	0.227	0.182	5.28E-16	Blood_Mo	COMT
SLC25A58	4.44E-20	0.443173	0.203	0.373	7.08E-16	Blood_Mo	SLC25A5
PSMB22	4.57E-20	0.543771	0.101	0.209	7.29E-16	Blood_Mo	PSMB2
MINOS14	4.88E-20	0.572626	0.163	0.311	7.79E-16	Blood_Mo	MINOS1
STUB11	5.04E-20	0.511913	0.131	0.256	8.04E-16	Blood_Mo	STUB1
LSM25	5.29E-20	0.379903	0.078	0.171	8.44E-16	Blood_Mo	LSM2
MIDN1	5.83E-20	0.283351	0.151	0.281	9.3E-16	Blood_Mo	MIDN
ZDHHC121	5.91E-20	0.384361	0.056	0.137	9.43E-16	Blood_Mo	ZDHHC12
AKR1A18	7.98E-20	0.514464	0.101	0.207	1.27E-15	Blood_Mo	AKR1A1
CACNA2D4	9.57E-20	0.715727	0.045	0.018	1.53E-15	Blood_Mo	CACNA2D4
IL10RA5	1.62E-19	0.792984	0.085	0.044	2.58E-15	Blood_Mo	IL10RA
SLC43A22	1.7E-19	0.856757	0.07	0.033	2.71E-15	Blood_Mo	SLC43A2
C6orf484	1.76E-19	0.327412	0.175	0.316	2.8E-15	Blood_Mo	C6orf48
COMMD4	2.34E-19	0.345727	0.047	0.121	3.72E-15	Blood_Mo	COMMD4
CYHR12	2.35E-19	0.27082	0.046	0.119	3.74E-15	Blood_Mo	CYHR1
PKM6	2.67E-19	0.456546	0.209	0.377	4.27E-15	Blood_Mo	PKM
PLP28	2.78E-19	0.36336	0.121	0.235	4.43E-15	Blood_Mo	PLP2
TSSC4	3.09E-19	0.33486	0.052	0.129	4.94E-15	Blood_Mo	TSSC4
PRKCSH1	3.3E-19	0.367149	0.067	0.152	5.26E-15	Blood_Mo	PRKCSH
LINC00937	3.33E-19	0.44799	0.028	0.008	5.31E-15	Blood_Mo	LINC00937
SNRNP703	4.53E-19	0.451697	0.089	0.186	7.23E-15	Blood_Mo	SNRNP70
FKBP82	4.81E-19	0.497665	0.187	0.343	7.67E-15	Blood_Mo	FKBP8
CELF16	5.47E-19	0.413695	0.051	0.127	8.72E-15	Blood_Mo	CELF1
TMEM256	5.84E-19	0.547327	0.112	0.223	9.32E-15	Blood_Mo	TMEM256
MRPL415	6.48E-19	0.680718	0.167	0.313	1.03E-14	Blood_Mo	MRPL41
GSTO16	7.7E-19	1.400976	0.251	0.213	1.23E-14	Blood_Mo	GSTO1
ATF6B	8.98E-19	0.261564	0.043	0.113	1.43E-14	Blood_Mo	ATF6B
APOA1BP2	9.64E-19	0.369444	0.057	0.134	1.54E-14	Blood_Mo	APOA1BP
IFFO11	1.05E-18	0.6445	0.047	0.019	1.67E-14	Blood_Mo	IFFO1
RASGRP22	1.42E-18	0.867705	0.107	0.062	2.27E-14	Blood_Mo	RASGRP2
C16orf741	1.5E-18	0.565954	0.05	0.021	2.4E-14	Blood_Mo	C16orf74
CLTA3	1.68E-18	0.662687	0.212	0.39	2.68E-14	Blood_Mo	CLTA
SDHAF2	1.79E-18	0.262254	0.045	0.115	2.86E-14	Blood_Mo	SDHAF2
MKNK27	1.95E-18	0.31687	0.047	0.119	3.11E-14	Blood_Mo	MKNK2
ATP5G312	2.13E-18	0.550264	0.201	0.366	3.39E-14	Blood_Mo	ATP5G3
STRA134	2.17E-18	0.563253	0.098	0.197	3.46E-14	Blood_Mo	STRA13
PLD36	2.41E-18	0.389885	0.079	0.167	3.85E-14	Blood_Mo	PLD3
OXA1L	2.46E-18	0.381043	0.07	0.153	3.93E-14	Blood_Mo	OXA1L
NAA104	2.89E-18	0.412405	0.075	0.162	4.6E-14	Blood_Mo	NAA10
TSPAN321	3.74E-18	0.498692	0.046	0.019	5.96E-14	Blood_Mo	TSPAN32
TMEM147	4.34E-18	0.574896	0.13	0.247	6.92E-14	Blood_Mo	TMEM147
COA41	4.41E-18	0.363921	0.048	0.119	7.03E-14	Blood_Mo	COA4
SLC16A3	4.62E-18	0.263807	0.075	0.16	7.37E-14	Blood_Mo	SLC16A3

POLR2E2	5.41E-18	0.479024	0.102	0.202	8.64E-14	Blood_Mo	POLR2E
CPNE13	5.65E-18	0.289293	0.056	0.13	9E-14	Blood_Mo	CPNE1
KRTCAP24	8.07E-18	0.4863	0.173	0.317	1.29E-13	Blood_Mo	KRTCAP2
PNKD4	1.05E-17	0.564046	0.098	0.196	1.68E-13	Blood_Mo	PNKD
ZEB2.AS1	1.25E-17	0.265451	0.013	0.003	1.99E-13	Blood_Mo	ZEB2.AS1
WDR334	1.28E-17	0.404201	0.071	0.154	2.04E-13	Blood_Mo	WDR33
ETV64	1.82E-17	0.291657	0.045	0.112	2.91E-13	Blood_Mo	ETV6
SDHB	1.99E-17	0.484271	0.079	0.166	3.17E-13	Blood_Mo	SDHB
SLC25A113	2.08E-17	0.25649	0.047	0.115	3.32E-13	Blood_Mo	SLC25A11
C5AR12	2.39E-17	0.61142	0.046	0.019	3.82E-13	Blood_Mo	C5AR1
CDK16	2.64E-17	0.27809	0.032	0.093	4.22E-13	Blood_Mo	CDK16
PNPLA2	2.7E-17	0.514212	0.085	0.174	4.3E-13	Blood_Mo	PNPLA2
PSMB43	2.86E-17	0.530694	0.09	0.182	4.56E-13	Blood_Mo	PSMB4
ETHE1	2.91E-17	0.571167	0.101	0.199	4.64E-13	Blood_Mo	ETHE1
MVB12A1	3.16E-17	0.255779	0.04	0.104	5.04E-13	Blood_Mo	MVB12A
RABAC112	5.37E-17	0.442957	0.233	0.409	8.57E-13	Blood_Mo	RABAC1
NDUFA112	5.42E-17	1.305416	0.389	0.439	8.65E-13	Blood_Mo	NDUFA11
NDUFS21	5.54E-17	0.393615	0.06	0.134	8.83E-13	Blood_Mo	NDUFS2
ANP32A	5.97E-17	0.432771	0.082	0.169	9.51E-13	Blood_Mo	ANP32A
UBALD21	6.12E-17	0.488438	0.085	0.173	9.76E-13	Blood_Mo	UBALD2
DNTTIP1	6.17E-17	0.250261	0.031	0.09	9.85E-13	Blood_Mo	DNTTIP1
ADAM83	6.9E-17	0.772797	0.06	0.028	1.1E-12	Blood_Mo	ADAM8
TBCB5	7.74E-17	0.418546	0.106	0.205	1.23E-12	Blood_Mo	TBCB
ARL4C5	7.82E-17	0.297498	0.099	0.191	1.25E-12	Blood_Mo	ARL4C
MPG4	1.35E-16	0.568277	0.087	0.175	2.15E-12	Blood_Mo	MPG
FUNDC21	1.47E-16	0.520593	0.098	0.19	2.34E-12	Blood_Mo	FUNDC2
NKAP2	1.6E-16	0.346722	0.038	0.099	2.54E-12	Blood_Mo	NKAP
HINT21	2E-16	0.598758	0.087	0.174	3.19E-12	Blood_Mo	HINT2
CTSD5	2.02E-16	1.129054	0.311	0.293	3.22E-12	Blood_Mo	CTSD
FIS16	2.04E-16	0.552617	0.191	0.342	3.25E-12	Blood_Mo	FIS1
NUMB2	2.16E-16	0.384836	0.052	0.121	3.45E-12	Blood_Mo	NUMB
TMEM261	2.3E-16	0.447059	0.066	0.142	3.66E-12	Blood_Mo	TMEM261
LAGE32	2.47E-16	0.405397	0.068	0.145	3.94E-12	Blood_Mo	LAGE3
ADRBK11	2.59E-16	1.166282	0.127	0.084	4.14E-12	Blood_Mo	ADRBK1
FIBP	2.81E-16	0.399472	0.081	0.165	4.49E-12	Blood_Mo	FIBP
ERCC14	8.1E-16	0.540072	0.077	0.157	1.29E-11	Blood_Mo	ERCC1
SSNA11	9.18E-16	0.691348	0.126	0.234	1.46E-11	Blood_Mo	SSNA1
SQRDL	9.89E-16	0.492317	0.064	0.137	1.58E-11	Blood_Mo	SQRDL
TCEA22	1.04E-15	0.256784	0.037	0.095	1.67E-11	Blood_Mo	TCEA2
DCTN34	1.17E-15	0.662737	0.129	0.237	1.86E-11	Blood_Mo	DCTN3
CD366	1.24E-15	0.474599	0.041	0.017	1.98E-11	Blood_Mo	CD36
IMPDH12	1.33E-15	0.99815	0.087	0.05	2.13E-11	Blood_Mo	IMPDH1
NUDT52	1.4E-15	0.333809	0.049	0.114	2.23E-11	Blood_Mo	NUDT5
KCNK66	1.53E-15	0.356132	0.042	0.103	2.45E-11	Blood_Mo	KCNK6
NAGK2	1.56E-15	1.298841	0.168	0.125	2.49E-11	Blood_Mo	NAGK
TOM1	1.63E-15	0.425904	0.064	0.136	2.59E-11	Blood_Mo	TOM1
NT5C6	1.64E-15	0.292756	0.058	0.127	2.61E-11	Blood_Mo	NT5C

MRPL45	1.7E-15	0.318314	0.054	0.121	2.71E-11	Blood_Mo	MRPL4
SPAG72	1.95E-15	0.533338	0.092	0.178	3.11E-11	Blood_Mo	SPAG7
ATF48	2.37E-15	0.70689	0.192	0.341	3.77E-11	Blood_Mo	ATF4
RP11.22N1	2.48E-15	0.480992	0.03	0.011	3.96E-11	Blood_Mo	RP11.22N19.2
PTRHD12	2.5E-15	0.712179	0.102	0.194	3.99E-11	Blood_Mo	PTRHD1
SND13	2.55E-15	0.31961	0.043	0.104	4.07E-11	Blood_Mo	SND1
ID15	2.95E-15	0.886303	0.155	0.108	4.71E-11	Blood_Mo	ID1
TBRG1	3.41E-15	0.447673	0.052	0.117	5.43E-11	Blood_Mo	TBRG1
ECH11	3.62E-15	0.580905	0.122	0.223	5.78E-11	Blood_Mo	ECH1
ATP6V0E1	4.96E-15	0.484956	0.31	0.55	7.91E-11	Blood_Mo	ATP6V0E1
FLOT13	5.01E-15	0.397241	0.071	0.145	7.99E-11	Blood_Mo	FLOT1
MRPL341	5.06E-15	0.622292	0.095	0.181	8.07E-11	Blood_Mo	MRPL34
DCXR5	5.55E-15	0.593436	0.122	0.223	8.86E-11	Blood_Mo	DCXR
TSR31	6.14E-15	0.325686	0.046	0.107	9.79E-11	Blood_Mo	TSR3
CYC15	6.85E-15	0.626966	0.128	0.233	1.09E-10	Blood_Mo	CYC1
SNRPD14	7.71E-15	0.590523	0.122	0.224	1.23E-10	Blood_Mo	SNRPD1
PLEKHO14	7.88E-15	1.050599	0.126	0.084	1.26E-10	Blood_Mo	PLEKHO1
PIK3R52	8.31E-15	0.379791	0.03	0.011	1.33E-10	Blood_Mo	PIK3R5
CMTM72	8.75E-15	0.969723	0.109	0.069	1.39E-10	Blood_Mo	CMTM7
NFKBIA7	9.47E-15	0.349226	0.507	0.517	1.51E-10	Blood_Mo	NFKBIA
AGPAT23	1.13E-14	0.48102	0.061	0.129	1.8E-10	Blood_Mo	AGPAT2
CCDC1242	1.25E-14	0.507818	0.082	0.161	2E-10	Blood_Mo	CCDC124
TMEM259	1.39E-14	0.460119	0.071	0.143	2.22E-10	Blood_Mo	TMEM259
NSMCE1	1.46E-14	0.384521	0.075	0.15	2.32E-10	Blood_Mo	NSMCE1
RPS219	1.48E-14	0.393167	0.626	0.674	2.35E-10	Blood_Mo	RPS21
BLVRA2	1.5E-14	1.347251	0.153	0.112	2.39E-10	Blood_Mo	BLVRA
PSMG4	2.08E-14	0.31134	0.042	0.099	3.31E-10	Blood_Mo	PSMG4
MTPN8	2.29E-14	0.534284	0.077	0.152	3.65E-10	Blood_Mo	MTPN
FERMT33	2.44E-14	0.818877	0.084	0.049	3.89E-10	Blood_Mo	FERMT3
CYBB1	2.92E-14	0.589862	0.046	0.021	4.66E-10	Blood_Mo	CYBB
RPS19BP1	3.42E-14	0.771195	0.16	0.283	5.45E-10	Blood_Mo	RPS19BP1
SNRPC	3.47E-14	0.609321	0.104	0.192	5.53E-10	Blood_Mo	SNRPC
ECSIT	3.49E-14	0.331842	0.036	0.09	5.56E-10	Blood_Mo	ECSIT
ARFGAP2	3.5E-14	0.302593	0.043	0.1	5.58E-10	Blood_Mo	ARFGAP2
ZYX4	3.54E-14	1.230779	0.156	0.116	5.64E-10	Blood_Mo	ZYX
AKNA2	3.97E-14	0.721657	0.067	0.036	6.33E-10	Blood_Mo	AKNA
PTK2B	4.32E-14	0.266995	0.063	0.129	6.89E-10	Blood_Mo	PTK2B
GLIPR27	4.35E-14	1.171077	0.125	0.085	6.94E-10	Blood_Mo	GLIPR2
RNASEK4	4.56E-14	0.741331	0.063	0.033	7.27E-10	Blood_Mo	RNASEK
TRAF7	6.12E-14	0.274773	0.03	0.079	9.76E-10	Blood_Mo	TRAF7
TSEN342	6.78E-14	0.492909	0.065	0.133	1.08E-09	Blood_Mo	TSEN34
URM11	6.78E-14	0.287557	0.049	0.107	1.08E-09	Blood_Mo	URM1
U2AF19	6.83E-14	1.3034	0.272	0.256	1.09E-09	Blood_Mo	U2AF1
LSM4	8.24E-14	0.615117	0.136	0.241	1.31E-09	Blood_Mo	LSM4
SUPT4H12	8.37E-14	0.756923	0.138	0.246	1.33E-09	Blood_Mo	SUPT4H1
SLC3A27	9.04E-14	0.5263	0.11	0.2	1.44E-09	Blood_Mo	SLC3A2
POP53	9.96E-14	0.302788	0.043	0.099	1.59E-09	Blood_Mo	POP5

MRPL273	1.21E-13	0.527012	0.074	0.145	1.93E-09	Blood_Mo	MRPL27
PTPN63	1.28E-13	0.904437	0.114	0.075	2.04E-09	Blood_Mo	PTPN6
CBR16	1.37E-13	0.435028	0.058	0.12	2.18E-09	Blood_Mo	CBR1
BEST17	1.55E-13	0.747379	0.074	0.042	2.48E-09	Blood_Mo	BEST1
MRPL16	2.09E-13	0.401452	0.049	0.107	3.34E-09	Blood_Mo	MRPL16
GLB11	2.44E-13	0.268923	0.027	0.073	3.9E-09	Blood_Mo	GLB1
USE11	2.55E-13	0.382501	0.054	0.114	4.07E-09	Blood_Mo	USE1
PRR13	3.16E-13	0.553846	0.181	0.309	5.05E-09	Blood_Mo	PRR13
PMVK4	3.81E-13	0.408642	0.067	0.134	6.08E-09	Blood_Mo	PMVK
COX5A2	3.92E-13	0.667686	0.242	0.416	6.25E-09	Blood_Mo	COX5A
PFKL2	3.97E-13	0.435643	0.064	0.128	6.34E-09	Blood_Mo	PFKL
BCKDK2	4.57E-13	0.493824	0.048	0.105	7.29E-09	Blood_Mo	BCKDK
GNB2	4.58E-13	0.758879	0.212	0.366	7.3E-09	Blood_Mo	GNB2
ZNF524	4.91E-13	0.381644	0.05	0.108	7.83E-09	Blood_Mo	ZNF524
MYCL	5.06E-13	0.298285	0.017	0.005	8.06E-09	Blood_Mo	MYCL
DDRGK13	5.12E-13	0.553869	0.081	0.154	8.17E-09	Blood_Mo	DDRGK1
TDG1	6.08E-13	0.282752	0.038	0.089	9.7E-09	Blood_Mo	TDG
MSRA1	6.41E-13	0.284755	0.036	0.085	1.02E-08	Blood_Mo	MSRA
JMJD65	6.67E-13	0.306518	0.04	0.092	1.06E-08	Blood_Mo	JMJD6
C21orf22	8.09E-13	0.294918	0.043	0.096	1.29E-08	Blood_Mo	C21orf2
MRPL242	8.33E-13	0.335641	0.048	0.104	1.33E-08	Blood_Mo	MRPL24
ADAP11	8.5E-13	0.580446	0.043	0.02	1.35E-08	Blood_Mo	ADAP1
FAM96B3	8.53E-13	0.728244	0.151	0.261	1.36E-08	Blood_Mo	FAM96B
ELOVL12	8.8E-13	0.340962	0.045	0.1	1.4E-08	Blood_Mo	ELOVL1
C19orf606	9.07E-13	1.332016	0.25	0.229	1.45E-08	Blood_Mo	C19orf60
LRPAP110	9.23E-13	0.635394	0.159	0.272	1.47E-08	Blood_Mo	LRPAP1
ALOX52	9.56E-13	0.548842	0.043	0.02	1.52E-08	Blood_Mo	ALOX5
PRDX54	9.8E-13	0.56158	0.287	0.496	1.56E-08	Blood_Mo	PRDX5
ITGB2.AS1	9.86E-13	0.450177	0.038	0.017	1.57E-08	Blood_Mo	ITGB2.AS1
PMF12	9.97E-13	0.458084	0.066	0.13	1.59E-08	Blood_Mo	PMF1
FAM127A6	1.12E-12	0.293727	0.041	0.093	1.79E-08	Blood_Mo	FAM127A
XXbac.B13	1.23E-12	0.371588	0.02	0.007	1.96E-08	Blood_Mo	XXbac.B135H6.15
IDH3B1	1.3E-12	0.494753	0.068	0.133	2.08E-08	Blood_Mo	IDH3B
ZFAND2B	1.33E-12	0.307731	0.047	0.101	2.12E-08	Blood_Mo	ZFAND2B
IL17RA1	1.34E-12	0.865338	0.063	0.034	2.13E-08	Blood_Mo	IL17RA
RNF1662	1.55E-12	0.763984	0.081	0.049	2.47E-08	Blood_Mo	RNF166
TMED11	1.66E-12	0.296901	0.033	0.08	2.64E-08	Blood_Mo	TMED1
NUP621	1.7E-12	0.26393	0.035	0.083	2.71E-08	Blood_Mo	NUP62
NLRP1	1.88E-12	0.824339	0.081	0.049	3E-08	Blood_Mo	NLRP1
CTDNEP12	2.07E-12	0.538145	0.071	0.137	3.3E-08	Blood_Mo	CTDNEP1
SSBP45	2.07E-12	0.295166	0.049	0.104	3.31E-08	Blood_Mo	SSBP4
CHMP2A	2.13E-12	0.719902	0.24	0.414	3.4E-08	Blood_Mo	CHMP2A
TMEM173	2.34E-12	0.256475	0.055	0.112	3.73E-08	Blood_Mo	TMEM173
KAT8	2.4E-12	0.331069	0.04	0.09	3.83E-08	Blood_Mo	KAT8
HNRNPL	2.61E-12	0.251098	0.04	0.091	4.16E-08	Blood_Mo	HNRNPL
PABPN19	2.61E-12	0.503431	0.082	0.153	4.17E-08	Blood_Mo	PABPN1
OTUB1	2.76E-12	0.506307	0.08	0.15	4.41E-08	Blood_Mo	OTUB1

MAD2L21	2.98E-12	0.306809	0.05	0.104	4.76E-08	Blood_Mo	MAD2L2
RASSF15	3.24E-12	0.279236	0.043	0.094	5.17E-08	Blood_Mo	RASSF1
DDX461	3.25E-12	0.709165	0.115	0.202	5.19E-08	Blood_Mo	DDX46
MPST3	3.29E-12	0.759626	0.093	0.169	5.25E-08	Blood_Mo	MPST
MXD3	4.56E-12	0.4792	0.031	0.013	7.27E-08	Blood_Mo	MXD3
NUBP2	4.65E-12	0.410832	0.059	0.118	7.42E-08	Blood_Mo	NUBP2
SMIM123	4.72E-12	0.462335	0.052	0.108	7.54E-08	Blood_Mo	SMIM12
MYEOV25	5.99E-12	0.742958	0.215	0.365	9.56E-08	Blood_Mo	MYEOV2
MLXIP3	5.99E-12	0.429974	0.049	0.103	9.56E-08	Blood_Mo	MLXIP
ATP6V0D1	6.29E-12	0.667929	0.118	0.206	1E-07	Blood_Mo	ATP6V0D1
PARK712	7.49E-12	0.545464	0.217	0.359	1.19E-07	Blood_Mo	PARK7
COX164	7.72E-12	0.370116	0.063	0.122	1.23E-07	Blood_Mo	COX16
WDR83OS!	7.87E-12	1.378118	0.337	0.369	1.26E-07	Blood_Mo	WDR83OS
BAX1	8.27E-12	1.249733	0.199	0.169	1.32E-07	Blood_Mo	BAX
ATP5O4	8.36E-12	0.677119	0.256	0.435	1.33E-07	Blood_Mo	ATP5O
RBCK11	8.97E-12	0.483343	0.084	0.153	1.43E-07	Blood_Mo	RBCK1
MEF2D	1.04E-11	0.320155	0.042	0.092	1.67E-07	Blood_Mo	MEF2D
DMAP1	1.05E-11	0.274245	0.029	0.072	1.68E-07	Blood_Mo	DMAP1
SSSCA11	1.23E-11	0.592609	0.073	0.137	1.96E-07	Blood_Mo	SSSCA1
GLT1D1	1.26E-11	0.274165	0.017	0.005	2.02E-07	Blood_Mo	GLT1D1
SERP16	1.36E-11	1.050973	0.436	0.549	2.17E-07	Blood_Mo	SERP1
CEBPA2	1.49E-11	0.650083	0.053	0.028	2.37E-07	Blood_Mo	CEBPA
PPIF1	1.75E-11	1.18222	0.138	0.103	2.8E-07	Blood_Mo	PPIF
REEP4	2.5E-11	0.251855	0.027	0.069	3.99E-07	Blood_Mo	REEP4
MPV171	2.64E-11	0.261868	0.032	0.075	4.22E-07	Blood_Mo	MPV17
TOLLIP1	2.93E-11	0.522715	0.072	0.135	4.67E-07	Blood_Mo	TOLLIP
NDUFB712	2.98E-11	0.712269	0.224	0.377	4.76E-07	Blood_Mo	NDUFB7
PLEKHJ11	3.03E-11	0.633048	0.096	0.17	4.83E-07	Blood_Mo	PLEKHJ1
RP11.85A1	3.21E-11	0.286548	0.018	0.006	5.12E-07	Blood_Mo	RP11.85A1.3
LENG1	3.45E-11	0.308856	0.036	0.081	5.51E-07	Blood_Mo	LENG1
RP11.108N	3.98E-11	0.597405	0.05	0.026	6.35E-07	Blood_Mo	RP11.108M9.4
PTPRE6	4.44E-11	0.863805	0.084	0.053	7.08E-07	Blood_Mo	PTPRE
SNX17	4.91E-11	0.604097	0.079	0.143	7.83E-07	Blood_Mo	SNX17
SNRNP252	5.19E-11	0.492241	0.048	0.098	8.27E-07	Blood_Mo	SNRNP25
ERICH14	5.65E-11	0.349207	0.054	0.107	9.01E-07	Blood_Mo	ERICH1
MRPL545	5.78E-11	0.728871	0.125	0.212	9.22E-07	Blood_Mo	MRPL54
C19orf25	6.17E-11	0.332674	0.041	0.087	9.83E-07	Blood_Mo	C19orf25
C19orf43	6.81E-11	1.151116	0.411	0.506	1.09E-06	Blood_Mo	C19orf43
REEP58	6.98E-11	0.60353	0.19	0.312	1.11E-06	Blood_Mo	REEP5
COA32	7.97E-11	0.770735	0.104	0.18	1.27E-06	Blood_Mo	COA3
APRT6	9.46E-11	1.082754	0.343	0.37	1.51E-06	Blood_Mo	APRT
SZRD1	1.05E-10	0.45943	0.045	0.092	1.67E-06	Blood_Mo	SZRD1
SLC31A23	1.11E-10	0.489307	0.058	0.112	1.78E-06	Blood_Mo	SLC31A2
TXN21	1.15E-10	0.763748	0.104	0.179	1.84E-06	Blood_Mo	TXN2
CLIC19	1.17E-10	0.924526	0.412	0.477	1.86E-06	Blood_Mo	CLIC1
RHOG3	1.2E-10	1.232093	0.211	0.186	1.91E-06	Blood_Mo	RHOG
ZNHIT12	1.53E-10	0.906843	0.179	0.298	2.44E-06	Blood_Mo	ZNHIT1

ARF52	1.6E-10	1.376131	0.237	0.222	2.55E-06	Blood_Mo	ARF5
PNKP3	1.89E-10	0.306507	0.037	0.081	3.01E-06	Blood_Mo	PNKP
MAP7D13	1.97E-10	0.38026	0.06	0.114	3.14E-06	Blood_Mo	MAP7D1
CUTA3	1.99E-10	0.608099	0.231	0.38	3.18E-06	Blood_Mo	CUTA
MRPL11	2.2E-10	0.640641	0.084	0.148	3.5E-06	Blood_Mo	MRPL11
UQCRFS12	2.43E-10	0.77433	0.165	0.273	3.88E-06	Blood_Mo	UQCRFS1
PTRH1	2.66E-10	0.302737	0.024	0.061	4.24E-06	Blood_Mo	PTRH1
NDUFA2	2.95E-10	0.827744	0.218	0.364	4.7E-06	Blood_Mo	NDUFA2
IFITM28	3.16E-10	0.798417	0.316	0.306	5.05E-06	Blood_Mo	IFITM2
NADK	3.25E-10	0.275417	0.03	0.069	5.19E-06	Blood_Mo	NADK
ZSWIM71	4.55E-10	0.462401	0.053	0.102	7.26E-06	Blood_Mo	ZSWIM7
RCSD12	4.79E-10	0.646556	0.082	0.052	7.63E-06	Blood_Mo	RCSD1
MRPL2010	5.97E-10	0.778073	0.167	0.273	9.52E-06	Blood_Mo	MRPL20
HPS11	6.06E-10	0.434177	0.049	0.096	9.67E-06	Blood_Mo	HPS1
UQCRC12	6.36E-10	0.700759	0.116	0.195	1.01E-05	Blood_Mo	UQCRC1
DUSP65	6.79E-10	0.499793	0.073	0.13	1.08E-05	Blood_Mo	DUSP6
GCA1	6.85E-10	0.267046	0.034	0.074	1.09E-05	Blood_Mo	GCA
DALRD3	7.28E-10	0.357626	0.044	0.088	1.16E-05	Blood_Mo	DALRD3
MARCH21	7.41E-10	0.270442	0.034	0.074	1.18E-05	Blood_Mo	MARCH2
ATF52	7.42E-10	0.342067	0.038	0.08	1.18E-05	Blood_Mo	ATF5
COX6A14	7.82E-10	0.538608	0.316	0.536	1.25E-05	Blood_Mo	COX6A1
KLF133	7.86E-10	0.306963	0.04	0.083	1.25E-05	Blood_Mo	KLF13
G0S22	8E-10	0.416176	0.159	0.122	1.28E-05	Blood_Mo	G0S2
UBR43	8.23E-10	0.409308	0.044	0.089	1.31E-05	Blood_Mo	UBR4
PEPD5	8.54E-10	0.367787	0.054	0.103	1.36E-05	Blood_Mo	PEPD
CUEDC2	8.72E-10	0.47584	0.068	0.123	1.39E-05	Blood_Mo	CUEDC2
PTPMT1	1.06E-09	0.416775	0.066	0.119	1.69E-05	Blood_Mo	PTPMT1
PPP1CA3	1.11E-09	0.734256	0.195	0.317	1.78E-05	Blood_Mo	PPP1CA
TALDO1	1.13E-09	1.10784	0.367	0.419	1.8E-05	Blood_Mo	TALDO1
EXOSC12	1.15E-09	0.325764	0.048	0.093	1.83E-05	Blood_Mo	EXOSC1
UROD	1.18E-09	0.511594	0.051	0.098	1.88E-05	Blood_Mo	UROD
NMRAL1	1.21E-09	0.400157	0.044	0.088	1.93E-05	Blood_Mo	NMRAL1
PSMG3	1.28E-09	0.50131	0.055	0.104	2.04E-05	Blood_Mo	PSMG3
TAGLN29	1.37E-09	0.36778	0.264	0.413	2.18E-05	Blood_Mo	TAGLN2
KIAA0319L	1.41E-09	0.383165	0.032	0.07	2.25E-05	Blood_Mo	KIAA0319L
PQBP17	1.5E-09	0.605849	0.091	0.155	2.39E-05	Blood_Mo	PQBP1
CTSZ8	1.55E-09	0.354237	0.12	0.194	2.48E-05	Blood_Mo	CTSZ
NXT16	1.61E-09	0.619349	0.091	0.155	2.56E-05	Blood_Mo	NXT1
EPC11	1.7E-09	0.505051	0.088	0.151	2.7E-05	Blood_Mo	EPC1
CYB561A3:	2.08E-09	0.252601	0.029	0.065	3.31E-05	Blood_Mo	CYB561A3
ERLIN2	2.08E-09	0.295881	0.027	0.062	3.32E-05	Blood_Mo	ERLIN2
USP4	2.16E-09	0.296559	0.031	0.068	3.45E-05	Blood_Mo	USP4
LINC00152	2.3E-09	0.332385	0.111	0.18	3.67E-05	Blood_Mo	LINC00152
IRF32	2.4E-09	0.399107	0.054	0.101	3.82E-05	Blood_Mo	IRF3
ASRGL1	2.4E-09	0.358758	0.04	0.081	3.83E-05	Blood_Mo	ASRGL1
SNF8	2.56E-09	0.836051	0.126	0.207	4.08E-05	Blood_Mo	SNF8
NBEAL21	2.63E-09	0.705029	0.057	0.034	4.2E-05	Blood_Mo	NBEAL2

MGAT14	2.89E-09	0.737418	0.132	0.216	4.61E-05	Blood_Mo	MGAT1
XPO6	3.04E-09	0.286282	0.027	0.063	4.84E-05	Blood_Mo	XPO6
FUOM2	3.55E-09	1.143079	0.107	0.078	5.67E-05	Blood_Mo	FUOM
DNASE22	3.68E-09	0.299686	0.034	0.072	5.87E-05	Blood_Mo	DNASE2
LRP14	3.81E-09	0.80733	0.114	0.083	6.08E-05	Blood_Mo	LRP1
PSME11	3.81E-09	1.048781	0.35	0.385	6.08E-05	Blood_Mo	PSME1
CTSC6	3.85E-09	0.332467	0.07	0.122	6.14E-05	Blood_Mo	CTSC
DUSP234	4.17E-09	1.404199	0.177	0.152	6.65E-05	Blood_Mo	DUSP23
IFT271	4.53E-09	0.469316	0.041	0.082	7.23E-05	Blood_Mo	IFT27
FAM96A2	4.59E-09	0.430743	0.054	0.1	7.32E-05	Blood_Mo	FAM96A
CBX69	4.75E-09	0.271991	0.044	0.085	7.57E-05	Blood_Mo	CBX6
WDR18	5.75E-09	0.275141	0.031	0.067	9.16E-05	Blood_Mo	WDR18
EIF4E21	6.12E-09	0.762527	0.102	0.17	9.77E-05	Blood_Mo	EIF4E2
DNPH12	6.39E-09	0.612075	0.088	0.148	0.000102	Blood_Mo	DNPH1
RIC8A	6.47E-09	0.309587	0.04	0.08	0.000103	Blood_Mo	RIC8A
TAPBPL1	6.47E-09	0.293224	0.031	0.066	0.000103	Blood_Mo	TAPBPL
ATP6V0A1	6.7E-09	0.353355	0.025	0.058	0.000107	Blood_Mo	ATP6V0A1
OXLD1	6.92E-09	0.501573	0.065	0.115	0.00011	Blood_Mo	OXLD1
NOTCH2NI	7.89E-09	1.001216	0.115	0.086	0.000126	Blood_Mo	NOTCH2NL
CDK5RAP3	9.91E-09	0.584737	0.075	0.13	0.000158	Blood_Mo	CDK5RAP3
JOSD23	1.02E-08	1.24083	0.145	0.118	0.000162	Blood_Mo	JOSD2
CLEC11A3	1.12E-08	0.252859	0.042	0.081	0.000179	Blood_Mo	CLEC11A
ARPC42	1.18E-08	0.76282	0.146	0.234	0.000189	Blood_Mo	ARPC4
HOTAIRM1	1.42E-08	0.5149	0.075	0.129	0.000226	Blood_Mo	HOTAIRM1
SUPT6H	1.59E-08	0.298184	0.03	0.064	0.000253	Blood_Mo	SUPT6H
SURF11	1.79E-08	0.736243	0.11	0.179	0.000286	Blood_Mo	SURF1
CKLF5	1.87E-08	1.122197	0.175	0.15	0.000298	Blood_Mo	CKLF
HLA.G2	1.91E-08	0.548752	0.044	0.024	0.000304	Blood_Mo	HLA.G
GRHPR1	1.96E-08	0.595584	0.084	0.141	0.000313	Blood_Mo	GRHPR
ARID5A6	1.97E-08	0.301616	0.056	0.1	0.000315	Blood_Mo	ARID5A
GADD45GI	1.99E-08	0.811639	0.177	0.282	0.000317	Blood_Mo	GADD45GIP1
SIGLEC7	2.01E-08	0.283244	0.021	0.009	0.00032	Blood_Mo	SIGLEC7
IFI65	2.09E-08	0.432225	0.096	0.156	0.000334	Blood_Mo	IFI6
HNMT6	2.11E-08	0.57246	0.063	0.11	0.000337	Blood_Mo	HNMT
BRI311	2.21E-08	0.690353	0.219	0.35	0.000353	Blood_Mo	BRI3
PPP1R183	2.67E-08	0.268345	0.07	0.119	0.000427	Blood_Mo	PPP1R18
SDF2L13	2.7E-08	0.656403	0.148	0.232	0.00043	Blood_Mo	SDF2L1
ARHGAP9	2.82E-08	0.698319	0.074	0.049	0.00045	Blood_Mo	ARHGAP9
C17orf891	2.92E-08	0.796423	0.12	0.193	0.000465	Blood_Mo	C17orf89
LTA4H	2.94E-08	0.491835	0.06	0.106	0.000468	Blood_Mo	LTA4H
B3GAT32	2.98E-08	0.357728	0.044	0.083	0.000475	Blood_Mo	B3GAT3
DOCK23	3.04E-08	0.527	0.038	0.02	0.000485	Blood_Mo	DOCK2
TNFAIP25	3.14E-08	1.040832	0.166	0.14	0.000501	Blood_Mo	TNFAIP2
MSRB28	3.19E-08	0.756795	0.078	0.131	0.000508	Blood_Mo	MSRB2
PDLIM75	3.31E-08	0.275042	0.031	0.065	0.000528	Blood_Mo	PDLIM7
SHMT22	3.92E-08	0.289574	0.033	0.068	0.000625	Blood_Mo	SHMT2
TAPBP8	4.15E-08	0.58221	0.102	0.166	0.000662	Blood_Mo	TAPBP

NUDT16	4.2E-08	1.159541	0.104	0.077	0.00067	Blood_Mo	NUDT16
RAB347	4.32E-08	0.526538	0.059	0.103	0.000689	Blood_Mo	RAB34
ARHGAP30	4.48E-08	0.759846	0.074	0.049	0.000715	Blood_Mo	ARHGAP30
LTBR6	4.5E-08	0.425565	0.049	0.09	0.000718	Blood_Mo	LTBR
GSTK11	4.89E-08	0.548178	0.194	0.3	0.000779	Blood_Mo	GSTK1
SLC38A29	5.17E-08	0.507665	0.157	0.244	0.000824	Blood_Mo	SLC38A2
CHMP4A1	5.23E-08	1.213386	0.185	0.165	0.000834	Blood_Mo	CHMP4A
AP1S27	5.53E-08	1.130331	0.151	0.125	0.000882	Blood_Mo	AP1S2
GIMAP72	6.47E-08	0.58629	0.155	0.121	0.001031	Blood_Mo	GIMAP7
SAC3D1	6.52E-08	0.259424	0.027	0.059	0.00104	Blood_Mo	SAC3D1
SYMPK	6.61E-08	0.331121	0.036	0.071	0.001055	Blood_Mo	SYMPK
VPS286	6.91E-08	0.860506	0.22	0.352	0.001103	Blood_Mo	VPS28
UQCRQ8	7.37E-08	0.604429	0.307	0.504	0.001176	Blood_Mo	UQCRQ
SCNM1	7.73E-08	0.955846	0.096	0.07	0.001233	Blood_Mo	SCNM1
PLD41	7.99E-08	0.255108	0.022	0.01	0.001274	Blood_Mo	PLD4
STAT21	8.1E-08	0.329671	0.037	0.072	0.001292	Blood_Mo	STAT2
RAB326	8.77E-08	1.03689	0.115	0.089	0.001399	Blood_Mo	RAB32
ANAPC15	8.91E-08	0.658526	0.075	0.125	0.00142	Blood_Mo	ANAPC15
ATPAF2	9.06E-08	0.250016	0.024	0.054	0.001444	Blood_Mo	ATPAF2
FGD3	9.64E-08	0.614671	0.051	0.031	0.001537	Blood_Mo	FGD3
MRPL122	1.01E-07	0.648496	0.076	0.127	0.001606	Blood_Mo	MRPL12
LYST6	1.01E-07	0.475496	0.053	0.095	0.001609	Blood_Mo	LYST
CYB561D2	1.02E-07	0.31096	0.037	0.072	0.001624	Blood_Mo	CYB561D2
PLEK3	1.02E-07	0.721385	0.079	0.054	0.001627	Blood_Mo	PLEK
TRPC4AP	1.03E-07	0.39863	0.037	0.072	0.001641	Blood_Mo	TRPC4AP
TRAPPC12	1.06E-07	1.272076	0.24	0.237	0.001683	Blood_Mo	TRAPPC1
ARL112	1.09E-07	0.263244	0.015	0.005	0.00174	Blood_Mo	ARL11
AP5B1	1.12E-07	0.465187	0.03	0.015	0.001788	Blood_Mo	AP5B1
STK17B5	1.15E-07	0.358265	0.062	0.106	0.001839	Blood_Mo	STK17B
ZFAS17	1.17E-07	0.432594	0.302	0.47	0.001862	Blood_Mo	ZFAS1
RGS106	1.23E-07	0.608728	0.141	0.218	0.001954	Blood_Mo	RGS10
FAM207A	1.28E-07	0.383053	0.042	0.078	0.002049	Blood_Mo	FAM207A
C7orf504	1.3E-07	0.663091	0.083	0.136	0.00208	Blood_Mo	C7orf50
DUSP222	1.33E-07	0.451862	0.051	0.091	0.002115	Blood_Mo	DUSP22
GNAI27	1.34E-07	1.250245	0.231	0.226	0.002137	Blood_Mo	GNAI2
CSF2RA2	1.4E-07	0.56361	0.047	0.028	0.002241	Blood_Mo	CSF2RA
PLEKHG2	1.54E-07	0.614318	0.047	0.028	0.002458	Blood_Mo	PLEKHG2
PRKAG21	1.56E-07	0.309323	0.033	0.065	0.002485	Blood_Mo	PRKAG2
COX191	1.57E-07	0.325552	0.044	0.08	0.002508	Blood_Mo	COX19
MS4A72	1.6E-07	0.56985	0.045	0.026	0.002555	Blood_Mo	MS4A7
P2RX11	1.78E-07	0.330427	0.017	0.007	0.002831	Blood_Mo	P2RX1
GRB23	1.97E-07	0.663103	0.11	0.174	0.003141	Blood_Mo	GRB2
ZDHHC241	1.98E-07	0.30393	0.029	0.059	0.003159	Blood_Mo	ZDHHC24
TRADD1	1.99E-07	0.453836	0.07	0.115	0.003176	Blood_Mo	TRADD
DBNL	2.13E-07	0.669091	0.115	0.181	0.003398	Blood_Mo	DBNL
FAM195A2	2.31E-07	0.714529	0.075	0.124	0.003684	Blood_Mo	FAM195A
MRPL235	2.39E-07	1.259174	0.219	0.212	0.003813	Blood_Mo	MRPL23

FCGR1B	2.87E-07	0.270457	0.017	0.007	0.004571	Blood_Mo	FCGR1B
ZNF3941	3.35E-07	0.458526	0.049	0.087	0.005351	Blood_Mo	ZNF394
MAP3K33	3.56E-07	0.686419	0.056	0.035	0.005675	Blood_Mo	MAP3K3
RIN3	4.07E-07	0.885015	0.102	0.078	0.006489	Blood_Mo	RIN3
UXT3	4.2E-07	0.783815	0.238	0.373	0.006699	Blood_Mo	UXT
YDJC	4.53E-07	0.267263	0.032	0.062	0.00723	Blood_Mo	YDJC
NFKBIL1	4.59E-07	0.304615	0.025	0.053	0.007321	Blood_Mo	NFKBIL1
NCKAP1L3	4.65E-07	0.522815	0.039	0.022	0.007409	Blood_Mo	NCKAP1L
PISD	4.66E-07	0.337123	0.029	0.058	0.007425	Blood_Mo	PISD
ARHGDIA7	4.72E-07	0.566241	0.129	0.199	0.007525	Blood_Mo	ARHGDIA
ANPEP3	4.81E-07	0.651883	0.053	0.034	0.007666	Blood_Mo	ANPEP
RP11.51J9.	4.82E-07	0.691225	0.073	0.05	0.007689	Blood_Mo	RP11.51J9.5
FAM173A1	5.19E-07	1.241396	0.158	0.137	0.008273	Blood_Mo	FAM173A
ATP5J22	5.24E-07	0.688394	0.274	0.443	0.008351	Blood_Mo	ATP5J2
TFEB1	5.28E-07	0.590315	0.047	0.028	0.008425	Blood_Mo	TFEB
TUBGCP21	5.74E-07	0.487858	0.052	0.09	0.009153	Blood_Mo	TUBGCP2
RNF1812	5.8E-07	0.892203	0.195	0.306	0.009254	Blood_Mo	RNF181
CTSA4	6.03E-07	0.657944	0.084	0.134	0.00962	Blood_Mo	CTSA
NFKBIE2	6.04E-07	0.411894	0.045	0.08	0.009637	Blood_Mo	NFKBIE
RAB27A6	7.15E-07	0.350226	0.035	0.066	0.011405	Blood_Mo	RAB27A
KLHL18	7.95E-07	0.293605	0.029	0.057	0.012681	Blood_Mo	KLHL18
NISCH	8.14E-07	0.350777	0.037	0.069	0.012975	Blood_Mo	NISCH
TLR22	8.73E-07	0.351543	0.025	0.013	0.013916	Blood_Mo	TLR2
GNPTG1	9.31E-07	0.451143	0.054	0.092	0.014842	Blood_Mo	GNPTG
RNPEPL12	9.44E-07	0.560996	0.068	0.111	0.015057	Blood_Mo	RNPEPL1
PPM1F3	9.59E-07	0.647509	0.049	0.031	0.015291	Blood_Mo	PPM1F
SNHG73	9.98E-07	0.515302	0.15	0.225	0.01592	Blood_Mo	SNHG7
WDR811	1.02E-06	0.404244	0.026	0.013	0.016304	Blood_Mo	WDR81
CTDSP12	1.06E-06	0.512161	0.061	0.101	0.016896	Blood_Mo	CTDSP1
NDUFS72	1.08E-06	1.31597	0.26	0.274	0.017172	Blood_Mo	NDUFS7
LAMTOR12	1.08E-06	1.332395	0.269	0.287	0.017253	Blood_Mo	LAMTOR1
KCNE31	1.13E-06	0.480282	0.026	0.013	0.017989	Blood_Mo	KCNE3
PET100	1.17E-06	0.765076	0.172	0.265	0.018625	Blood_Mo	PET100
COX144	1.19E-06	0.89059	0.153	0.235	0.018977	Blood_Mo	COX14
CRTAP9	1.38E-06	0.547774	0.072	0.116	0.021936	Blood_Mo	CRTAP
MAP1LC3B	1.55E-06	0.358979	0.04	0.072	0.024645	Blood_Mo	MAP1LC3B2
A1BG4	1.58E-06	0.828713	0.085	0.063	0.02517	Blood_Mo	A1BG
RP11.102N	1.73E-06	0.260135	0.016	0.007	0.027628	Blood_Mo	RP11.102N12.3
CSNK2B	1.76E-06	0.848922	0.206	0.319	0.028035	Blood_Mo	CSNK2B
ZMAT5	1.76E-06	0.366392	0.045	0.079	0.0281	Blood_Mo	ZMAT5
AC093673.	1.82E-06	0.378754	0.03	0.016	0.029038	Blood_Mo	AC093673.5
ABCA71	1.91E-06	0.495579	0.04	0.024	0.030438	Blood_Mo	ABCA7
COMMD5	1.99E-06	0.466897	0.046	0.08	0.031699	Blood_Mo	COMMD5
RABL61	1.99E-06	0.559402	0.059	0.097	0.031769	Blood_Mo	RABL6
DEF81	2.03E-06	0.549137	0.053	0.089	0.032441	Blood_Mo	DEF8
ICT1	2.1E-06	0.333353	0.033	0.062	0.033432	Blood_Mo	ICT1
UNC1192	2.11E-06	0.365505	0.042	0.073	0.033614	Blood_Mo	UNC119

HAUS7	2.36E-06	0.346449	0.027	0.014	0.037565	Blood_Mo	HAUS7
NDUFS33	2.43E-06	0.871997	0.125	0.19	0.038823	Blood_Mo	NDUFS3
RAB315	2.44E-06	0.9579	0.116	0.094	0.038924	Blood_Mo	RAB31
RILPL24	2.47E-06	1.021569	0.119	0.097	0.039336	Blood_Mo	RILPL2
RNF220	2.49E-06	0.256702	0.027	0.054	0.039662	Blood_Mo	RNF220
ZNF688	2.68E-06	0.363535	0.047	0.08	0.042747	Blood_Mo	ZNF688
S100A121	2.75E-06	0.446225	0.197	0.169	0.043823	Blood_Mo	S100A12
TGFB15	2.84E-06	0.881829	0.121	0.099	0.045223	Blood_Mo	TGFB1
SEMA6B1	2.85E-06	0.261867	0.018	0.008	0.045438	Blood_Mo	SEMA6B
DDX39A	3.04E-06	0.528264	0.078	0.122	0.048464	Blood_Mo	DDX39A
STYXL1	3.28E-06	0.332065	0.035	0.064	0.052357	Blood_Mo	STYXL1
CIITA2	3.33E-06	0.285188	0.021	0.01	0.05304	Blood_Mo	CIITA
RP11.1398	3.44E-06	0.433784	0.026	0.014	0.05481	Blood_Mo	RP11.1398P2.1
SNX201	3.46E-06	0.292623	0.024	0.012	0.055214	Blood_Mo	SNX20
SNHG155	3.47E-06	0.502539	0.071	0.112	0.055354	Blood_Mo	SNHG15
LY6E4	3.75E-06	0.53703	0.226	0.334	0.059854	Blood_Mo	LY6E
PHC22	3.92E-06	0.535266	0.039	0.069	0.062581	Blood_Mo	PHC2
EFTUD2	3.93E-06	0.27646	0.022	0.046	0.062633	Blood_Mo	EFTUD2
RFNG1	4.2E-06	0.253972	0.023	0.046	0.06692	Blood_Mo	RFNG
JTB6	4.21E-06	0.744425	0.243	0.374	0.067171	Blood_Mo	JTB
PGD	4.29E-06	0.720735	0.11	0.167	0.068461	Blood_Mo	PGD
CD1631	4.68E-06	0.41348	0.034	0.019	0.074664	Blood_Mo	CD163
MYO15B	4.79E-06	0.410496	0.03	0.016	0.076475	Blood_Mo	MYO15B
HAAO	5.03E-06	0.292059	0.018	0.009	0.080239	Blood_Mo	HAAO
FAM50A1	5.03E-06	0.737361	0.086	0.133	0.080247	Blood_Mo	FAM50A
HOMER34	5.03E-06	0.334269	0.031	0.058	0.080258	Blood_Mo	HOMER3
AIP2	5.21E-06	0.558285	0.085	0.13	0.083063	Blood_Mo	AIP
SLC39A103	5.85E-06	0.296279	0.032	0.058	0.093335	Blood_Mo	SLC39A10
POLR3K1	5.97E-06	0.550426	0.04	0.07	0.095192	Blood_Mo	POLR3K
ZNF385A4	6.14E-06	0.672463	0.056	0.037	0.097923	Blood_Mo	ZNF385A
EIF3B2	6.43E-06	0.664053	0.069	0.109	0.1025	Blood_Mo	EIF3B
SLC38A102	6.47E-06	0.56437	0.063	0.101	0.103138	Blood_Mo	SLC38A10
SLC39A4	6.63E-06	0.313251	0.035	0.062	0.105704	Blood_Mo	SLC39A4
KLF27	6.68E-06	0.250754	0.134	0.193	0.106607	Blood_Mo	KLF2
NUBP11	7.07E-06	0.320182	0.033	0.06	0.112776	Blood_Mo	NUBP1
PGP	7.81E-06	0.506784	0.078	0.12	0.124634	Blood_Mo	PGP
C6orf226	8.17E-06	0.280542	0.03	0.055	0.130304	Blood_Mo	C6orf226
LRCH4	9.6E-06	0.48785	0.066	0.104	0.153086	Blood_Mo	LRCH4
MED15	1.03E-05	0.531098	0.049	0.08	0.164514	Blood_Mo	MED15
RAB24	1.04E-05	0.892956	0.112	0.091	0.166658	Blood_Mo	RAB24
UBE2L6	1.05E-05	0.503063	0.082	0.125	0.167966	Blood_Mo	UBE2L6
PIEZO11	1.06E-05	0.357253	0.042	0.071	0.169377	Blood_Mo	PIEZO1
MIF4GD1	1.08E-05	0.29771	0.04	0.069	0.172516	Blood_Mo	MIF4GD
FCHO1	1.09E-05	0.299047	0.02	0.01	0.173958	Blood_Mo	FCHO1
RFXANK	1.16E-05	0.53541	0.063	0.099	0.184234	Blood_Mo	RFXANK
ADPGK1	1.23E-05	0.336615	0.039	0.067	0.195925	Blood_Mo	ADPGK
CASP12	1.26E-05	0.757987	0.076	0.056	0.201189	Blood_Mo	CASP1

EDF13	1.34E-05	0.549726	0.383	0.63	0.213653	Blood_Mo	EDF1
TMEM199	1.39E-05	0.330646	0.029	0.053	0.222466	Blood_Mo	TMEM199
SLC12A91	1.41E-05	0.635527	0.05	0.033	0.225153	Blood_Mo	SLC12A9
C17orf623	1.43E-05	0.404551	0.058	0.092	0.228324	Blood_Mo	C17orf62
RARA3	1.56E-05	0.2836	0.031	0.056	0.24832	Blood_Mo	RARA
C19orf241	1.71E-05	0.836516	0.128	0.19	0.272697	Blood_Mo	C19orf24
MYD88	1.71E-05	0.347884	0.043	0.072	0.273222	Blood_Mo	MYD88
C14orf801	1.78E-05	0.4861	0.037	0.023	0.283583	Blood_Mo	C14orf80
VMA211	1.96E-05	0.586287	0.07	0.108	0.313216	Blood_Mo	VMA21
NDUFS83	2.06E-05	0.807197	0.179	0.267	0.328585	Blood_Mo	NDUFS8
PLOD11	2.11E-05	0.314995	0.023	0.044	0.336824	Blood_Mo	PLOD1
OGFR	2.12E-05	0.481367	0.059	0.093	0.337956	Blood_Mo	OGFR
NFKB25	2.13E-05	0.42215	0.049	0.079	0.339629	Blood_Mo	NFKB2
ZNF780A	2.18E-05	0.277907	0.022	0.043	0.347049	Blood_Mo	ZNF780A
PLEKHM21	2.32E-05	0.363694	0.038	0.065	0.369443	Blood_Mo	PLEKHM2
VCAN2	2.46E-05	0.640576	0.115	0.093	0.391694	Blood_Mo	VCAN
CCM21	2.57E-05	0.44537	0.055	0.087	0.410185	Blood_Mo	CCM2
IQGAP15	2.8E-05	0.936265	0.175	0.261	0.446377	Blood_Mo	IQGAP1
C14orf159	2.91E-05	0.259587	0.027	0.049	0.464216	Blood_Mo	C14orf159
TXNRD21	3.15E-05	0.270221	0.022	0.042	0.502694	Blood_Mo	TXNRD2
ELP5	3.35E-05	0.507372	0.068	0.104	0.534552	Blood_Mo	ELP5
KIF22	3.36E-05	1.055264	0.105	0.086	0.5365	Blood_Mo	KIF22
DTYMK	3.43E-05	0.333265	0.029	0.051	0.547676	Blood_Mo	DTYMK
ENO15	3.72E-05	0.714677	0.273	0.416	0.592536	Blood_Mo	ENO1
DENND4B1	3.78E-05	0.470337	0.035	0.021	0.603457	Blood_Mo	DENND4B
PXN2	4.35E-05	0.417398	0.047	0.076	0.693599	Blood_Mo	PXN
CHCHD101	4.8E-05	0.709548	0.169	0.247	0.765408	Blood_Mo	CHCHD10
STAT61	4.92E-05	0.871827	0.092	0.073	0.785344	Blood_Mo	STAT6
OCEL1	5.82E-05	0.419793	0.04	0.066	0.928802	Blood_Mo	OCEL1
C21orf331	6.03E-05	0.303826	0.033	0.057	0.96228	Blood_Mo	C21orf33
TRABD2	6.34E-05	0.586396	0.08	0.119	1	Blood_Mo	TRABD
TPP13	6.46E-05	0.504303	0.064	0.098	1	Blood_Mo	TPP1
RRP7A	6.51E-05	0.714516	0.082	0.123	1	Blood_Mo	RRP7A
CD1C2	6.85E-05	0.340227	0.035	0.021	1	Blood_Mo	CD1C
HBEGF4	7.38E-05	0.30016	0.029	0.05	1	Blood_Mo	HBEGF
SIGLEC11	8.12E-05	0.254578	0.015	0.007	1	Blood_Mo	SIGLEC1
CPSF1	8.2E-05	0.265432	0.024	0.044	1	Blood_Mo	CPSF1
SLC19A1	8.59E-05	0.35122	0.018	0.009	1	Blood_Mo	SLC19A1
BRMS1	9.29E-05	0.569077	0.07	0.104	1	Blood_Mo	BRMS1
MAP3K113	9.81E-05	0.558987	0.06	0.092	1	Blood_Mo	MAP3K11
TGFBI7	0.000106	0.699643	0.081	0.063	1	Blood_Mo	TGFBI
PLXNB22	0.00012	0.740981	0.109	0.158	1	Blood_Mo	PLXNB2
CTSH6	0.000124	1.295981	0.228	0.239	1	Blood_Mo	CTSH
MTMR141	0.000125	0.447565	0.043	0.069	1	Blood_Mo	MTMR14
STRN4	0.000125	0.366218	0.029	0.05	1	Blood_Mo	STRN4
PRKACA	0.000137	0.426597	0.037	0.061	1	Blood_Mo	PRKACA
SIAH23	0.000144	0.575702	0.072	0.107	1	Blood_Mo	SIAH2

MNT	0.000145	0.362335	0.027	0.047	1	Blood_Mo	MNT
MAZ2	0.000146	0.536834	0.059	0.09	1	Blood_Mo	MAZ
MRPS122	0.000162	0.881397	0.098	0.141	1	Blood_Mo	MRPS12
ATXN2L	0.000176	0.400264	0.042	0.066	1	Blood_Mo	ATXN2L
NOL12	0.000179	0.409404	0.04	0.064	1	Blood_Mo	NOL12
CD3024	0.00018	0.367432	0.041	0.065	1	Blood_Mo	CD302
COQ7	0.000189	0.39846	0.044	0.069	1	Blood_Mo	COQ7
BID3	0.000189	1.018015	0.135	0.121	1	Blood_Mo	BID
SASH31	0.000221	0.364179	0.035	0.023	1	Blood_Mo	SASH3
GTPBP61	0.000224	0.443558	0.052	0.079	1	Blood_Mo	GTPBP6
FYN8	0.000256	0.266188	0.044	0.068	1	Blood_Mo	FYN
ATG2A1	0.000265	0.290405	0.026	0.045	1	Blood_Mo	ATG2A
ATP5G19	0.000277	0.805383	0.182	0.263	1	Blood_Mo	ATP5G1
PLA2G168	0.000285	0.723077	0.088	0.127	1	Blood_Mo	PLA2G16
IRF7	0.000299	0.52196	0.068	0.099	1	Blood_Mo	IRF7
PSMB65	0.000316	1.012977	0.218	0.324	1	Blood_Mo	PSMB6
ADAP22	0.000316	0.368034	0.026	0.016	1	Blood_Mo	ADAP2
NDUFB27	0.000323	0.993557	0.318	0.377	1	Blood_Mo	NDUFB2
G6PD	0.000324	0.360668	0.039	0.061	1	Blood_Mo	G6PD
NME39	0.000332	0.839523	0.148	0.211	1	Blood_Mo	NME3
PI4K2A	0.000359	0.344374	0.031	0.051	1	Blood_Mo	PI4K2A
SIPA11	0.000367	0.572163	0.061	0.09	1	Blood_Mo	SIPA1
LACTB4	0.000373	0.548885	0.058	0.086	1	Blood_Mo	LACTB
ARHGEF40	0.000391	0.259716	0.022	0.038	1	Blood_Mo	ARHGEF40
SIVA15	0.000404	0.858348	0.191	0.276	1	Blood_Mo	SIVA1
HDAC10	0.000416	0.42735	0.035	0.023	1	Blood_Mo	HDAC10
PLBD11	0.000426	0.520408	0.058	0.085	1	Blood_Mo	PLBD1
GRIPAP1	0.000429	0.280203	0.025	0.043	1	Blood_Mo	GRIPAP1
CIC2	0.000433	0.279133	0.026	0.044	1	Blood_Mo	CIC
HCAR32	0.000436	0.314311	0.025	0.015	1	Blood_Mo	HCAR3
TOR2A	0.000496	0.699519	0.059	0.044	1	Blood_Mo	TOR2A
STX10	0.000518	1.172432	0.137	0.126	1	Blood_Mo	STX10
ZRSR2	0.000522	0.274703	0.026	0.043	1	Blood_Mo	ZRSR2
PES1	0.000545	0.377319	0.03	0.048	1	Blood_Mo	PES1
UNC93B13	0.000549	0.799078	0.081	0.066	1	Blood_Mo	UNC93B1
RBBP63	0.000588	0.766126	0.12	0.168	1	Blood_Mo	RBBP6
ENGASE	0.000659	0.473026	0.032	0.021	1	Blood_Mo	ENGASE
TSC22D34	0.000737	0.82889	0.265	0.283	1	Blood_Mo	TSC22D3
UCP23	0.000753	0.423964	0.114	0.155	1	Blood_Mo	UCP2
CHPF22	0.00079	0.297062	0.023	0.039	1	Blood_Mo	CHPF2
TBC1D10B	0.000792	0.254771	0.02	0.036	1	Blood_Mo	TBC1D10B
IMPA21	0.000845	0.437394	0.04	0.062	1	Blood_Mo	IMPA2
BANF14	0.000868	0.761651	0.167	0.235	1	Blood_Mo	BANF1
ITGAE2	0.000871	0.804111	0.095	0.134	1	Blood_Mo	ITGAE
PSMB83	0.000883	1.107465	0.191	0.192	1	Blood_Mo	PSMB8
DOCK84	0.000903	0.298345	0.034	0.053	1	Blood_Mo	DOCK8
FYB3	0.000933	0.631083	0.088	0.073	1	Blood_Mo	FYB

TPCN12	0.001031	0.387647	0.035	0.054	1	Blood_Mo	TPCN1
CMTM34	0.001033	0.396476	0.053	0.077	1	Blood_Mo	CMTM3
COMMD91	0.001038	0.512604	0.057	0.083	1	Blood_Mo	COMMD9
MAST31	0.00104	0.326812	0.024	0.015	1	Blood_Mo	MAST3
MTHFR1	0.001083	0.281483	0.024	0.04	1	Blood_Mo	MTHFR
FOLR22	0.001188	0.37836	0.032	0.021	1	Blood_Mo	FOLR2
PIK3CD1	0.001214	0.340564	0.027	0.017	1	Blood_Mo	PIK3CD
CARS2	0.001217	0.911453	0.08	0.066	1	Blood_Mo	CARS2
SH3BP21	0.001246	0.464228	0.057	0.082	1	Blood_Mo	SH3BP2
PBX21	0.001299	0.325977	0.027	0.044	1	Blood_Mo	PBX2
DGKZ1	0.001322	0.790267	0.068	0.054	1	Blood_Mo	DGKZ
ZNF7871	0.001352	0.282561	0.019	0.033	1	Blood_Mo	ZNF787
IFI27L26	0.001401	0.989088	0.148	0.14	1	Blood_Mo	IFI27L2
BTK2	0.001439	0.337528	0.026	0.017	1	Blood_Mo	BTK
PHF21A	0.001443	0.251561	0.02	0.034	1	Blood_Mo	PHF21A
MVP2	0.001563	0.586633	0.065	0.092	1	Blood_Mo	MVP
ETFB6	0.001563	1.046043	0.192	0.274	1	Blood_Mo	ETFB
ENTPD17	0.001607	0.31194	0.03	0.047	1	Blood_Mo	ENTPD1
NUDT142	0.001709	0.664271	0.056	0.08	1	Blood_Mo	NUDT14
ATP5H	0.001713	1.027741	0.239	0.353	1	Blood_Mo	ATP5H
RPL388	0.001796	0.285905	0.616	0.766	1	Blood_Mo	RPL38
LYL12	0.001812	0.700833	0.085	0.071	1	Blood_Mo	LYL1
SLC27A35	0.001836	0.506544	0.039	0.028	1	Blood_Mo	SLC27A3
ALKBH7	0.001838	0.30377	0.233	0.318	1	Blood_Mo	ALKBH7
COX6B11	0.001911	0.746575	0.438	0.611	1	Blood_Mo	COX6B1
AP1M1	0.001936	0.34909	0.035	0.052	1	Blood_Mo	AP1M1
CIB13	0.002008	0.81215	0.244	0.354	1	Blood_Mo	CIB1
BIN21	0.002082	0.250644	0.056	0.079	1	Blood_Mo	BIN2
POLR2L7	0.00211	0.79872	0.376	0.476	1	Blood_Mo	POLR2L
LCP14	0.002224	0.385374	0.08	0.109	1	Blood_Mo	LCP1
CARD85	0.002344	0.329099	0.037	0.055	1	Blood_Mo	CARD8
SLC4A21	0.002353	0.275695	0.02	0.033	1	Blood_Mo	SLC4A2
SNX102	0.002413	0.351907	0.036	0.054	1	Blood_Mo	SNX10
HSD17B10	0.002466	1.149996	0.17	0.17	1	Blood_Mo	HSD17B10
ATG4B	0.002485	0.454624	0.047	0.068	1	Blood_Mo	ATG4B
SMDT14	0.002527	0.9076	0.257	0.376	1	Blood_Mo	SMDT1
INTS12	0.002584	0.48194	0.038	0.056	1	Blood_Mo	INTS1
MADD	0.002669	0.388464	0.023	0.037	1	Blood_Mo	MADD
MPP14	0.002686	0.312983	0.027	0.043	1	Blood_Mo	MPP1
TMEM219	0.002691	1.281949	0.281	0.333	1	Blood_Mo	TMEM219
LAMTOR2	0.002706	1.284651	0.217	0.234	1	Blood_Mo	LAMTOR2
AC079305.	0.002732	0.275124	0.019	0.012	1	Blood_Mo	AC079305.10
LENG81	0.002822	0.510788	0.057	0.08	1	Blood_Mo	LENG8
RNU12	0.002858	0.301666	0.027	0.018	1	Blood_Mo	RNU12
C9orf1421	0.00291	0.563834	0.109	0.147	1	Blood_Mo	C9orf142
CECR5	0.003008	0.311638	0.033	0.05	1	Blood_Mo	CECR5
DDT3	0.003048	1.291474	0.286	0.343	1	Blood_Mo	DDT

HAUS4	0.003771	0.257084	0.025	0.039	1	Blood_Mo	HAUS4
CHCHD5	0.003846	0.888871	0.115	0.157	1	Blood_Mo	CHCHD5
TMEM205	0.003852	1.194931	0.196	0.205	1	Blood_Mo	TMEM205
DMXL21	0.003979	0.256321	0.02	0.033	1	Blood_Mo	DMXL2
DHRS4	0.004093	0.353284	0.033	0.049	1	Blood_Mo	DHRS4
DGCR21	0.00416	0.4257	0.036	0.053	1	Blood_Mo	DGCR2
CYP27A13	0.004258	0.391991	0.034	0.05	1	Blood_Mo	CYP27A1
DENND6B1	0.004369	0.519374	0.042	0.032	1	Blood_Mo	DENND6B
GLIPR14	0.004516	0.89393	0.101	0.09	1	Blood_Mo	GLIPR1
CCDC159	0.004527	0.371211	0.037	0.053	1	Blood_Mo	CCDC159
CSK1	0.004545	0.758749	0.087	0.076	1	Blood_Mo	CSK
CCDC88B	0.004641	0.783226	0.076	0.064	1	Blood_Mo	CCDC88B
VPS16	0.004678	0.264066	0.024	0.037	1	Blood_Mo	VPS16
RNF1305	0.004699	1.123778	0.146	0.141	1	Blood_Mo	RNF130
CLEC10A2	0.004706	0.356129	0.04	0.03	1	Blood_Mo	CLEC10A
SELO	0.004757	0.288059	0.026	0.04	1	Blood_Mo	SELO
ACSS2	0.004811	0.309822	0.025	0.039	1	Blood_Mo	ACSS2
PEX16	0.005024	0.589202	0.068	0.093	1	Blood_Mo	PEX16
CCDC85B6	0.005773	1.117358	0.245	0.273	1	Blood_Mo	CCDC85B
PPCDC	0.005777	0.368586	0.03	0.045	1	Blood_Mo	PPCDC
ID25	0.006202	0.713759	0.242	0.255	1	Blood_Mo	ID2
GIMAP45	0.006209	0.28737	0.056	0.076	1	Blood_Mo	GIMAP4
FBL3	0.006458	0.803648	0.175	0.238	1	Blood_Mo	FBL
FKBP151	0.006613	0.299277	0.024	0.037	1	Blood_Mo	FKBP15
GLRX3	0.006978	1.07609	0.203	0.214	1	Blood_Mo	GLRX
MKL1	0.00705	0.275502	0.024	0.036	1	Blood_Mo	MKL1
CDK5	0.00745	0.259211	0.016	0.027	1	Blood_Mo	CDK5
MYO9B4	0.007657	0.822578	0.08	0.069	1	Blood_Mo	MYO9B
ZNF511	0.007828	0.667394	0.07	0.095	1	Blood_Mo	ZNF511
CCS3	0.007939	0.815292	0.084	0.112	1	Blood_Mo	CCS
ADAM154	0.008671	0.504501	0.056	0.076	1	Blood_Mo	ADAM15
TNK2	0.009125	0.529366	0.045	0.035	1	Blood_Mo	TNK2
IL6R3	0.009222	0.252763	0.027	0.041	1	Blood_Mo	IL6R
PPIB7	0.00952	0.69466	0.279	0.397	1	Blood_Mo	PPIB
B9D2	0.009821	0.3661	0.029	0.042	1	Blood_Mo	B9D2
DNASE1L3	0	3.305402	0.727	0.013	0	Blood_CD3	DNASE1L3
BATF33	0	2.299572	0.764	0.014	0	Blood_CD3	BATF3
IDO1	0	2.057607	0.6	0.005	0	Blood_CD3	IDO1
HLA.DOB1	0	2.04012	0.727	0.012	0	Blood_CD3	HLA.DOB
SERPINF2	0	1.88043	0.709	0.01	0	Blood_CD3	SERPINF2
WDFY41	0	1.675503	0.691	0.013	0	Blood_CD3	WDFY4
SLAMF82	0	1.366245	0.509	0.008	0	Blood_CD3	SLAMF8
FLT3	0	1.088568	0.418	0.006	0	Blood_CD3	FLT3
CRIP3	0	0.969863	0.364	0.004	0	Blood_CD3	CRIP3
IRF83	2.3E-288	3.286526	1	0.042	3.6E-284	Blood_CD3	IRF8
GCSAM	5E-287	1.026323	0.364	0.005	8.1E-283	Blood_CD3	GCSAM
HCK3	5.5E-272	1.584776	0.709	0.021	8.7E-268	Blood_CD3	HCK

LY863	4.6E-254	2.43134	0.927	0.039	7.4E-250	Blood_CD3	LY86
TLR101	8.2E-227	0.720453	0.273	0.004	1.3E-222	Blood_CD3	TLR10
MYCL1	1.8E-212	0.756405	0.309	0.005	2.9E-208	Blood_CD3	MYCL
CLNK1	1.2E-208	0.878455	0.309	0.005	2E-204	Blood_CD3	CLNK
PARVG3	5.3E-196	1.824483	0.727	0.03	8.5E-192	Blood_CD3	PARVG
MPEG13	2.1E-191	1.35611	0.509	0.015	3.3E-187	Blood_CD3	MPEG1
CPVL3	1.1E-185	4.765423	1	0.069	1.8E-181	Blood_CD3	CPVL
HLA.DMB4	4.2E-172	2.538928	1	0.067	6.6E-168	Blood_CD3	HLA.DMB
CFP3	1.3E-171	1.338276	0.655	0.027	2.1E-167	Blood_CD3	CFP
SPI13	2.5E-170	2.79057	0.945	0.062	3.9E-166	Blood_CD3	SPI1
HLA.DQA2	4.5E-170	3.864317	1	0.073	7.2E-166	Blood_CD3	HLA.DQA2
C1orf546	1.3E-164	2.132602	0.818	0.047	2.1E-160	Blood_CD3	C1orf54
LGALS23	5E-155	2.505071	0.782	0.044	7.9E-151	Blood_CD3	LGALS2
HLA.DQB2	3.8E-154	1.462677	0.6	0.026	6.1E-150	Blood_CD3	HLA.DQB2
UPK3A1	9.6E-148	0.772642	0.309	0.007	1.5E-143	Blood_CD3	UPK3A
MNDA3	1.8E-143	1.590136	0.6	0.028	2.8E-139	Blood_CD3	MNDA
C19orf381	1.5E-140	1.225904	0.473	0.018	2.4E-136	Blood_CD3	C19orf38
C20orf275	1.7E-136	2.577581	0.891	0.069	2.7E-132	Blood_CD3	C20orf27
CYP2E1	3.5E-132	0.702647	0.273	0.006	5.6E-128	Blood_CD3	CYP2E1
IGFLR11	1.3E-124	1.098913	0.509	0.023	2E-120	Blood_CD3	IGFLR1
C1orf1623	7.9E-124	2.575988	0.982	0.091	1.3E-119	Blood_CD3	C1orf162
HLA.DQA1	8.7E-124	4.41574	1	0.107	1.4E-119	Blood_CD3	HLA.DQA1
NME1.NM	2.1E-122	1.223897	0.527	0.025	3.3E-118	Blood_CD3	NME1.NME2
CD333	7.1E-114	0.812201	0.345	0.012	1.1E-109	Blood_CD3	CD33
IRF52	6.9E-109	1.079528	0.364	0.014	1.1E-104	Blood_CD3	IRF5
FGR2	3.2E-107	1.167163	0.527	0.028	5.2E-103	Blood_CD3	FGR
PPM1J	7.1E-106	1.251642	0.491	0.026	1.1E-101	Blood_CD3	PPM1J
P2RY61	8.2E-101	0.518848	0.182	0.004	1.31E-96	Blood_CD3	P2RY6
PPM1M	1.1E-100	1.278803	0.545	0.033	1.68E-96	Blood_CD3	PPM1M
HLA.DRB5	5.8E-100	4.214504	1	0.136	9.25E-96	Blood_CD3	HLA.DRB5
HLA.DQB1	1.3E-99	3.670261	1	0.131	2.13E-95	Blood_CD3	HLA.DQB1
HLA.DMA5	4.43E-91	2.957877	1	0.14	7.07E-87	Blood_CD3	HLA.DMA
PLEK4	9.33E-89	1.596469	0.673	0.054	1.49E-84	Blood_CD3	PLEK
RAB327	1.43E-86	2.002047	0.818	0.089	2.27E-82	Blood_CD3	RAB32
FCN11	4.37E-86	0.458505	0.436	0.024	6.97E-82	Blood_CD3	FCN1
ATP5EP21	5.39E-86	1.205672	0.673	0.055	8.59E-82	Blood_CD3	ATP5EP2
ITGB25	7.94E-86	1.997473	0.945	0.109	1.27E-81	Blood_CD3	ITGB2
TGFBI8	5.78E-85	1.436482	0.709	0.063	9.21E-81	Blood_CD3	TGFBI
PLCB22	2.12E-84	0.727603	0.309	0.013	3.39E-80	Blood_CD3	PLCB2
SUSD31	7.13E-84	1.148785	0.527	0.035	1.14E-79	Blood_CD3	SUSD3
MAP4K12	7.62E-84	0.719531	0.364	0.017	1.22E-79	Blood_CD3	MAP4K1
SNX15	4.96E-83	0.30581	0.127	0.002	7.92E-79	Blood_CD3	SNX15
PPT12	3.92E-82	2.378035	0.927	0.129	6.26E-78	Blood_CD3	PPT1
VAC14	4.66E-82	1.115526	0.418	0.024	7.44E-78	Blood_CD3	VAC14
MYO1F3	2.98E-81	1.081023	0.582	0.044	4.76E-77	Blood_CD3	MYO1F
SELPLG3	3.61E-81	1.065655	0.491	0.032	5.76E-77	Blood_CD3	SELPLG
NAAA2	2.54E-80	1.613267	0.8	0.088	4.05E-76	Blood_CD3	NAAA

AIF13	2E-79	1.620955	0.964	0.115	3.19E-75	Blood_CD3	AIF1
HLA.DPA15	2.67E-77	4.758183	1	0.192	4.25E-73	Blood_CD3	HLA.DPA1
PLCD1	8.38E-76	0.900161	0.418	0.026	1.34E-71	Blood_CD3	PLCD1
LST13	1.64E-74	1.442699	0.855	0.098	2.62E-70	Blood_CD3	LST1
CXCR32	1.39E-73	0.725295	0.364	0.02	2.21E-69	Blood_CD3	CXCR3
UCP24	3.54E-73	2.292543	0.982	0.154	5.64E-69	Blood_CD3	UCP2
NAGA1	2.59E-72	1.221434	0.527	0.043	4.12E-68	Blood_CD3	NAGA
COTL15	2.83E-72	2.92421	1	0.184	4.51E-68	Blood_CD3	COTL1
PYCARD4	5.05E-70	2.463044	0.982	0.167	8.06E-66	Blood_CD3	PYCARD
HLA.DPB14	3.58E-69	4.432263	1	0.219	5.7E-65	Blood_CD3	HLA.DPB1
HLA.DRB15	1.04E-67	4.004684	1	0.219	1.66E-63	Blood_CD3	HLA.DRB1
ICAM33	1.92E-66	1.754918	0.964	0.145	3.06E-62	Blood_CD3	ICAM3
ADA	4.29E-65	0.848151	0.473	0.036	6.84E-61	Blood_CD3	ADA
CD484	1.92E-64	1.716023	0.909	0.123	3.07E-60	Blood_CD3	CD48
LGALS92	2.05E-64	1.781401	0.818	0.113	3.26E-60	Blood_CD3	LGALS9
RGS192	2.44E-64	1.545742	0.636	0.066	3.89E-60	Blood_CD3	RGS19
AOAH2	2.55E-64	0.759109	0.327	0.018	4.07E-60	Blood_CD3	AOAH
AP1S28	3.29E-64	1.716197	0.873	0.125	5.24E-60	Blood_CD3	AP1S2
LSP14	8.85E-63	2.858637	1	0.231	1.41E-58	Blood_CD3	LSP1
HCLS13	2.83E-62	1.551967	0.691	0.078	4.51E-58	Blood_CD3	HCLS1
ITGB2.AS1	6.98E-62	0.724852	0.309	0.017	1.11E-57	Blood_CD3	ITGB2.AS1
REPIN1	9.54E-62	1.685442	0.618	0.069	1.52E-57	Blood_CD3	REPIN1
PSMB101	1.04E-61	1.444912	0.855	0.121	1.66E-57	Blood_CD3	PSMB10
GLIPR15	1.33E-61	1.603623	0.727	0.09	2.13E-57	Blood_CD3	GLIPR1
ITGB72	2.1E-61	0.953027	0.473	0.038	3.35E-57	Blood_CD3	ITGB7
SAMHD14	1.05E-60	1.283916	0.636	0.069	1.68E-56	Blood_CD3	SAMHD1
LIMD22	1.16E-60	2.037411	1	0.174	1.85E-56	Blood_CD3	LIMD2
RGS107	3.35E-60	2.590544	0.982	0.216	5.34E-56	Blood_CD3	RGS10
CD300C1	6.42E-60	0.385739	0.145	0.004	1.02E-55	Blood_CD3	CD300C
HLA.DRA5	7.16E-60	3.806162	1	0.252	1.14E-55	Blood_CD3	HLA.DRA
PSMB96	9.54E-60	2.128796	0.945	0.169	1.52E-55	Blood_CD3	PSMB9
FCER1G3	4.29E-59	0.882381	0.855	0.111	6.85E-55	Blood_CD3	FCER1G
C17orf491	7.22E-59	0.675119	0.382	0.027	1.15E-54	Blood_CD3	C17orf49
UNC93B14	2.36E-58	1.150994	0.6	0.066	3.77E-54	Blood_CD3	UNC93B1
ZNF4673	1.04E-57	0.880219	0.364	0.025	1.66E-53	Blood_CD3	ZNF467
BID4	3.17E-57	1.592955	0.8	0.121	5.06E-53	Blood_CD3	BID
AC093673.	9.35E-57	0.73731	0.291	0.016	1.49E-52	Blood_CD3	AC093673.5
MIF4GD2	2.79E-56	1.130166	0.6	0.068	4.46E-52	Blood_CD3	MIF4GD
TRPV22	4.84E-56	0.880002	0.345	0.023	7.73E-52	Blood_CD3	TRPV2
CAT3	5.11E-56	1.741601	0.764	0.115	8.14E-52	Blood_CD3	CAT
FAM105A3	3.5E-55	1.0144	0.436	0.037	5.58E-51	Blood_CD3	FAM105A
LILRA21	7.16E-55	0.383617	0.164	0.006	1.14E-50	Blood_CD3	LILRA2
TOR2A1	7.82E-55	1.030251	0.473	0.044	1.25E-50	Blood_CD3	TOR2A
APOL33	2.73E-54	0.76524	0.4	0.031	4.35E-50	Blood_CD3	APOL3
ADAM84	3.3E-53	0.716922	0.382	0.029	5.27E-49	Blood_CD3	ADAM8
SELL	7.48E-53	0.932608	0.6	0.064	1.19E-48	Blood_CD3	SELL
ABI34	1.89E-52	0.881261	0.4	0.032	3.01E-48	Blood_CD3	ABI3

FKBP1B1	5.2E-52	0.958103	0.4	0.034	8.29E-48	Blood_CD3	FKBP1B
UTF12	5.81E-52	0.467905	0.182	0.007	9.27E-48	Blood_CD3	UTF1
ARPC1B5	2.85E-51	2.685047	1	0.293	4.55E-47	Blood_CD3	ARPC1B
ASB21	2.86E-51	0.756147	0.2	0.009	4.56E-47	Blood_CD3	ASB2
WAS4	4.08E-51	0.963526	0.473	0.044	6.51E-47	Blood_CD3	WAS
RNASET24	1.69E-50	2.115179	1	0.237	2.7E-46	Blood_CD3	RNASET2
AIM2	2.9E-50	0.573142	0.2	0.009	4.63E-46	Blood_CD3	AIM2
TWF21	1.2E-49	1.516752	0.764	0.123	1.91E-45	Blood_CD3	TWF2
LCP15	2.01E-49	1.485735	0.727	0.108	3.2E-45	Blood_CD3	LCP1
TYMP5	5.32E-49	1.498306	0.927	0.185	8.49E-45	Blood_CD3	TYMP
CD745	1.29E-48	3.80625	1	0.363	2.06E-44	Blood_CD3	CD74
SCIMP2	2.83E-48	0.63669	0.2	0.009	4.51E-44	Blood_CD3	SCIMP
BTK3	8.2E-48	0.728412	0.273	0.017	1.31E-43	Blood_CD3	BTK
IL163	1.25E-47	0.895685	0.509	0.053	2E-43	Blood_CD3	IL16
FCGRT9	1.57E-47	2.126479	1	0.278	2.5E-43	Blood_CD3	FCGRT
CNPY32	2.11E-47	1.415618	0.782	0.133	3.36E-43	Blood_CD3	CNPY3
FES3	3.95E-47	0.805679	0.4	0.037	6.3E-43	Blood_CD3	FES
CORO1A3	3.97E-47	1.902679	0.964	0.199	6.34E-43	Blood_CD3	CORO1A
CDK2AP24	6.03E-47	1.764965	0.836	0.165	9.62E-43	Blood_CD3	CDK2AP2
FAM26F2	8.81E-47	0.937738	0.455	0.045	1.41E-42	Blood_CD3	FAM26F
PSMB84	8.93E-47	1.837824	0.891	0.191	1.42E-42	Blood_CD3	PSMB8
MTMR142	1.34E-46	1.052634	0.545	0.068	2.13E-42	Blood_CD3	MTMR14
ALDH29	2.06E-46	1.532823	0.727	0.124	3.28E-42	Blood_CD3	ALDH2
CSF2RA3	1.81E-45	0.9502	0.345	0.028	2.88E-41	Blood_CD3	CSF2RA
AKR7A21	5.03E-45	1.494152	0.782	0.148	8.02E-41	Blood_CD3	AKR7A2
CD373	3E-44	1.505383	0.982	0.203	4.78E-40	Blood_CD3	CD37
ID26	3.17E-44	2.379945	0.945	0.255	5.06E-40	Blood_CD3	ID2
SCNM11	3.66E-44	1.216232	0.545	0.07	5.83E-40	Blood_CD3	SCNM1
DBNL1	5.18E-44	1.686473	0.855	0.18	8.27E-40	Blood_CD3	DBNL
SNX37	1.21E-43	3.15654	0.982	0.416	1.92E-39	Blood_CD3	SNX3
PGLS3	1.8E-43	1.915337	0.964	0.263	2.86E-39	Blood_CD3	PGLS
FAM49B4	2.13E-43	1.521299	0.836	0.173	3.4E-39	Blood_CD3	FAM49B
MFNG1	3.08E-43	0.830621	0.436	0.044	4.91E-39	Blood_CD3	MFNG
PTPRE7	9.73E-43	0.981172	0.473	0.054	1.55E-38	Blood_CD3	PTPRE
OSCAR2	1.18E-42	0.510995	0.164	0.007	1.89E-38	Blood_CD3	OSCAR
MICAL12	2.16E-42	0.860834	0.364	0.033	3.44E-38	Blood_CD3	MICAL1
BASP15	2.17E-42	1.152585	0.6	0.084	3.46E-38	Blood_CD3	BASP1
F8A1	3.72E-42	0.765041	0.455	0.05	5.93E-38	Blood_CD3	F8A1
YIF1B1	5.25E-42	1.034848	0.509	0.065	8.37E-38	Blood_CD3	YIF1B
MYO1G3	1.08E-41	0.639551	0.364	0.032	1.72E-37	Blood_CD3	MYO1G
GRN7	2.05E-41	2.034317	0.982	0.312	3.27E-37	Blood_CD3	GRN
ROGDI2	3.82E-41	1.244603	0.473	0.059	6.09E-37	Blood_CD3	ROGDI
SNX221	4.04E-41	0.420389	0.164	0.007	6.44E-37	Blood_CD3	SNX22
GPSM33	4.46E-40	1.146042	0.909	0.173	7.12E-36	Blood_CD3	GPSM3
MVP3	1.09E-39	1.083046	0.6	0.091	1.74E-35	Blood_CD3	MVP
CNN21	3.04E-39	1.39432	0.836	0.175	4.86E-35	Blood_CD3	CNN2
TBC1D10C	3.64E-39	0.820306	0.564	0.073	5.8E-35	Blood_CD3	TBC1D10C

CORO74	4.93E-39	0.708478	0.364	0.035	7.86E-35	Blood_CD3	CORO7
RNH16	2.6E-38	1.940677	0.964	0.314	4.15E-34	Blood_CD3	RNH1
CST38	3.22E-38	3.312109	1	0.594	5.13E-34	Blood_CD3	CST3
COMMD92	3.79E-38	1.081893	0.545	0.082	6.04E-34	Blood_CD3	COMMD9
TAGLN210	5.93E-38	2.094166	1	0.409	9.46E-34	Blood_CD3	TAGLN2
UNC1193	7.41E-38	1.181478	0.509	0.072	1.18E-33	Blood_CD3	UNC119
FIBP1	4.12E-37	1.124585	0.782	0.163	6.57E-33	Blood_CD3	FIBP
SYK2	5.09E-37	0.832151	0.345	0.034	8.12E-33	Blood_CD3	SYK
SLC25A67	5.79E-37	2.33606	1	0.568	9.24E-33	Blood_CD3	SLC25A6
ATG3	6.84E-37	1.484551	0.764	0.166	1.09E-32	Blood_CD3	ATG3
NME46	8.2E-37	1.305017	0.709	0.143	1.31E-32	Blood_CD3	NME4
HMHA12	1.13E-36	0.862846	0.509	0.068	1.8E-32	Blood_CD3	HMHA1
OXA1L1	1.22E-36	1.49033	0.727	0.15	1.95E-32	Blood_CD3	OXA1L
CTC.479C5	1.75E-36	0.458313	0.218	0.014	2.79E-32	Blood_CD3	CTC.479C5.12
LAPTM56	2E-36	1.057781	0.891	0.173	3.2E-32	Blood_CD3	LAPTM5
C1orf1861	2.25E-36	0.456485	0.145	0.007	3.59E-32	Blood_CD3	C1orf186
UBL7	2.89E-36	1.207853	0.564	0.091	4.6E-32	Blood_CD3	UBL7
SNX202	2.9E-36	0.400494	0.2	0.012	4.62E-32	Blood_CD3	SNX20
SRGN6	4.83E-36	0.922116	0.982	0.224	7.71E-32	Blood_CD3	SRGN
FMNL16	4.94E-36	0.812116	0.491	0.064	7.88E-32	Blood_CD3	FMNL1
GSTP19	5.39E-36	2.219507	1	0.557	8.6E-32	Blood_CD3	GSTP1
FCHO11	6.27E-36	0.598348	0.182	0.01	1E-31	Blood_CD3	FCHO1
M6PR4	1.08E-35	1.118018	0.673	0.128	1.73E-31	Blood_CD3	M6PR
CYB5R38	1.1E-35	1.542636	0.873	0.222	1.75E-31	Blood_CD3	CYB5R3
GYPC6	2.18E-35	1.304264	0.909	0.203	3.47E-31	Blood_CD3	GYPC
ATP5A16	2.95E-35	1.776664	0.964	0.313	4.71E-31	Blood_CD3	ATP5A1
ACTB7	3.42E-35	2.026066	1	0.872	5.46E-31	Blood_CD3	ACTB
GPX13	7.23E-35	1.864526	1	0.427	1.15E-30	Blood_CD3	GPX1
GDI23	7.88E-35	1.56064	0.927	0.268	1.26E-30	Blood_CD3	GDI2
DCTPP13	9.05E-35	1.229912	0.6	0.106	1.44E-30	Blood_CD3	DCTPP1
EIF4A19	1.05E-34	2.042424	0.964	0.353	1.67E-30	Blood_CD3	EIF4A1
ZYX5	1.22E-34	1.247774	0.636	0.116	1.94E-30	Blood_CD3	ZYX
GMFG4	1.7E-34	1.18333	0.945	0.21	2.71E-30	Blood_CD3	GMFG
SNRPA	1.91E-34	0.909596	0.582	0.097	3.04E-30	Blood_CD3	SNRPA
SLC12A92	3.35E-34	0.760123	0.327	0.033	5.34E-30	Blood_CD3	SLC12A9
SPNS11	4.05E-34	0.848888	0.436	0.057	6.46E-30	Blood_CD3	SPNS1
DUSP28	4.97E-34	0.772239	0.709	0.126	7.93E-30	Blood_CD3	DUSP2
RAB4B1	5.81E-34	0.750661	0.382	0.044	9.27E-30	Blood_CD3	RAB4B
TRAPPC13	5.99E-34	1.568169	0.873	0.237	9.55E-30	Blood_CD3	TRAPPC1
SIGLEC71	8.57E-34	0.444313	0.164	0.009	1.37E-29	Blood_CD3	SIGLEC7
GSTM42	1.74E-33	0.698035	0.345	0.038	2.78E-29	Blood_CD3	GSTM4
SIGLEC101	1.92E-33	0.313334	0.127	0.006	3.06E-29	Blood_CD3	SIGLEC10
ZNF385A5	2.09E-33	0.729329	0.345	0.037	3.33E-29	Blood_CD3	ZNF385A
CFL14	2.8E-33	2.079822	1	0.71	4.47E-29	Blood_CD3	CFL1
PADI41	3.92E-33	0.443317	0.145	0.007	6.25E-29	Blood_CD3	PADI4
PSME12	4.26E-33	1.882541	0.982	0.384	6.79E-29	Blood_CD3	PSME1
H2AFY1	5.74E-33	1.734077	0.945	0.326	9.15E-29	Blood_CD3	H2AFY

RHOG4	6.72E-33	1.256643	0.8	0.186	1.07E-28	Blood_CD3	RHOG
MAP2K6	9.53E-33	0.369256	0.182	0.011	1.52E-28	Blood_CD3	MAP2K6
SLAMF71	1.15E-32	0.602107	0.236	0.018	1.83E-28	Blood_CD3	SLAMF7
LAMTOR13	1.62E-32	1.656742	0.927	0.286	2.59E-28	Blood_CD3	LAMTOR1
PSME22	1.69E-32	1.611828	0.945	0.288	2.69E-28	Blood_CD3	PSME2
PARP10	1.95E-32	0.71065	0.327	0.035	3.11E-28	Blood_CD3	PARP10
CYC16	2.11E-32	1.373459	0.855	0.231	3.36E-28	Blood_CD3	CYC1
BST27	2.54E-32	1.176605	0.818	0.182	4.05E-28	Blood_CD3	BST2
TAP13	3.83E-32	1.061736	0.564	0.096	6.11E-28	Blood_CD3	TAP1
PFN13	3.85E-32	2.000947	1	0.586	6.14E-28	Blood_CD3	PFN1
LAT23	3.91E-32	0.644595	0.327	0.034	6.24E-28	Blood_CD3	LAT2
ENSA3	4.34E-32	1.306752	0.873	0.235	6.92E-28	Blood_CD3	ENSA
MRPL236	6.06E-32	1.337049	0.836	0.212	9.67E-28	Blood_CD3	MRPL23
TPM38	7.73E-32	1.561914	0.964	0.332	1.23E-27	Blood_CD3	TPM3
SAMM50	1.82E-31	1.097084	0.491	0.079	2.91E-27	Blood_CD3	SAMM50
TBXAS12	1.84E-31	0.743211	0.291	0.029	2.94E-27	Blood_CD3	TBXAS1
ENO16	1.95E-31	1.785746	1	0.412	3.12E-27	Blood_CD3	ENO1
BEST18	3.86E-31	0.671111	0.364	0.043	6.16E-27	Blood_CD3	BEST1
AIP3	4.08E-31	1.008824	0.655	0.129	6.5E-27	Blood_CD3	AIP
C1QBP1	5.14E-31	1.602095	0.836	0.238	8.2E-27	Blood_CD3	C1QBP
CYBA5	6.01E-31	1.737146	1	0.441	9.59E-27	Blood_CD3	CYBA
GAPDH11	7.37E-31	1.665061	1	0.764	1.18E-26	Blood_CD3	GAPDH
IDO2	7.48E-31	0.299601	0.091	0.003	1.19E-26	Blood_CD3	IDO2
C10orf543	7.76E-31	1.168675	0.764	0.171	1.24E-26	Blood_CD3	C10orf54
ARF53	1.74E-30	1.525193	0.8	0.222	2.78E-26	Blood_CD3	ARF5
POLD41	1.86E-30	1.205891	0.855	0.22	2.97E-26	Blood_CD3	POLD4
SEMA4A2	5.51E-30	0.717887	0.327	0.038	8.79E-26	Blood_CD3	SEMA4A
PKM7	8.18E-30	1.598648	1	0.373	1.31E-25	Blood_CD3	PKM
PTGES2	8.2E-30	1.088121	0.564	0.107	1.31E-25	Blood_CD3	PTGES2
COMT5	1.18E-29	1.141337	0.745	0.183	1.88E-25	Blood_CD3	COMT
ARHGDIB1	1.19E-29	1.70392	1	0.448	1.9E-25	Blood_CD3	ARHGDIB
CPNE33	1.41E-29	1.388416	0.745	0.193	2.25E-25	Blood_CD3	CPNE3
MYD881	1.58E-29	1.096946	0.455	0.071	2.53E-25	Blood_CD3	MYD88
ARPC28	1.73E-29	1.80296	1	0.574	2.76E-25	Blood_CD3	ARPC2
TAPBPL2	2.01E-29	0.754489	0.436	0.065	3.21E-25	Blood_CD3	TAPBPL
EIF3L3	2.82E-29	1.695636	1	0.388	4.49E-25	Blood_CD3	EIF3L
SLC25A59	2.84E-29	1.652679	0.982	0.369	4.53E-25	Blood_CD3	SLC25A5
AP2S14	3.62E-29	1.568768	0.927	0.342	5.78E-25	Blood_CD3	AP2S1
B3GNTL1	4.03E-29	0.457944	0.182	0.013	6.42E-25	Blood_CD3	B3GNTL1
MSL31	4.72E-29	0.90088	0.436	0.066	7.52E-25	Blood_CD3	MSL3
CIITA3	4.8E-29	0.361375	0.164	0.01	7.65E-25	Blood_CD3	CIITA
ADPGK.AS1	5.1E-29	0.336593	0.127	0.006	8.14E-25	Blood_CD3	ADPGK.AS1
GABARAP1	5.27E-29	1.331334	0.964	0.271	8.41E-25	Blood_CD3	GABARAP
PSMA61	8.02E-29	0.740342	0.455	0.07	1.28E-24	Blood_CD3	PSMA6
BIK2	1.16E-28	0.895273	0.382	0.053	1.85E-24	Blood_CD3	BIK
NCF43	2.67E-28	0.691011	0.345	0.042	4.26E-24	Blood_CD3	NCF4
SPN2	9.29E-28	0.530433	0.255	0.024	1.48E-23	Blood_CD3	SPN

TSSC1	1.16E-27	0.729364	0.4	0.059	1.84E-23	Blood_CD3	TSSC1
CNPPD1	3.35E-27	1.039192	0.509	0.095	5.34E-23	Blood_CD3	CNPPD1
HNRNPM5	4.21E-27	1.187859	0.8	0.217	6.72E-23	Blood_CD3	HNRNPM
RPL171	6.39E-27	1.601387	1	0.525	1.02E-22	Blood_CD3	RPL17
IMPA22	6.59E-27	0.810916	0.4	0.061	1.05E-22	Blood_CD3	IMPA2
GNG72	7.94E-27	1.061987	0.4	0.061	1.27E-22	Blood_CD3	GNG7
H3F3C	8.37E-27	0.513041	0.255	0.026	1.33E-22	Blood_CD3	H3F3C
NDUFS22	1.09E-26	1.103583	0.6	0.132	1.74E-22	Blood_CD3	NDUFS2
SLC11A11	1.44E-26	0.373881	0.2	0.016	2.29E-22	Blood_CD3	SLC11A1
RPS6KA42	1.6E-26	0.540367	0.255	0.026	2.55E-22	Blood_CD3	RPS6KA4
OTUB11	2.03E-26	1.070591	0.636	0.148	3.23E-22	Blood_CD3	OTUB1
RPL43	2.16E-26	1.521814	1	0.7	3.44E-22	Blood_CD3	RPL4
CD72	2.36E-26	0.450024	0.145	0.009	3.76E-22	Blood_CD3	CD72
PRCP5	2.8E-26	1.094195	0.582	0.125	4.46E-22	Blood_CD3	PRCP
PLEKHO15	3.1E-26	0.860591	0.491	0.085	4.94E-22	Blood_CD3	PLEKHO1
FNBP15	5.21E-26	1.011289	0.6	0.127	8.3E-22	Blood_CD3	FNBP1
EIF3D3	5.43E-26	1.328408	0.855	0.285	8.65E-22	Blood_CD3	EIF3D
RPS6KA11	5.76E-26	0.844562	0.345	0.047	9.18E-22	Blood_CD3	RPS6KA1
FOS13	6.18E-26	1.409737	1	0.425	9.86E-22	Blood_CD3	FOS
ATP5F11	7.11E-26	1.293387	0.891	0.308	1.13E-21	Blood_CD3	ATP5F1
ATP5B8	9.93E-26	1.324955	0.964	0.371	1.58E-21	Blood_CD3	ATP5B
HLA.DOA3	1.02E-25	0.611941	0.2	0.017	1.63E-21	Blood_CD3	HLA.DOA
LYRM43	1.37E-25	1.078787	0.545	0.113	2.19E-21	Blood_CD3	LYRM4
APOBEC3G	1.53E-25	0.679817	0.345	0.046	2.45E-21	Blood_CD3	APOBEC3G
SNRNP253	2.16E-25	1.119088	0.491	0.097	3.44E-21	Blood_CD3	SNRNP25
SASH32	2.2E-25	0.470871	0.236	0.023	3.51E-21	Blood_CD3	SASH3
DUSP235	2.46E-25	1.08519	0.636	0.152	3.93E-21	Blood_CD3	DUSP23
EVL2	2.96E-25	0.729556	0.727	0.167	4.72E-21	Blood_CD3	EVL
MPST4	3.09E-25	1.131548	0.655	0.167	4.92E-21	Blood_CD3	MPST
HSD17B10	3.96E-25	1.099987	0.673	0.17	6.32E-21	Blood_CD3	HSD17B10
HAVCR22	4.13E-25	0.635095	0.218	0.02	6.58E-21	Blood_CD3	HAVCR2
CYTH44	4.18E-25	0.710862	0.273	0.03	6.67E-21	Blood_CD3	CYTH4
NCF14	6.22E-25	0.423728	0.327	0.041	9.91E-21	Blood_CD3	NCF1
HLA.C5	6.22E-25	1.548459	1	0.686	9.92E-21	Blood_CD3	HLA.C
TBCB6	8.6E-25	0.967757	0.764	0.202	1.37E-20	Blood_CD3	TBCB
ARPC32	1.17E-24	1.436337	1	0.597	1.87E-20	Blood_CD3	ARPC3
EXOSC5	1.56E-24	0.824853	0.345	0.049	2.49E-20	Blood_CD3	EXOSC5
MIIP1	2E-24	0.537809	0.364	0.054	3.18E-20	Blood_CD3	MIIP
HLA.B5	2.13E-24	1.599258	1	0.667	3.39E-20	Blood_CD3	HLA.B
TXN22	2.67E-24	1.153054	0.673	0.177	4.26E-20	Blood_CD3	TXN2
ELOVL13	2.76E-24	0.894217	0.491	0.098	4.4E-20	Blood_CD3	ELOVL1
CECR14	2.82E-24	0.574174	0.309	0.04	4.49E-20	Blood_CD3	CECR1
CLNS1A	2.86E-24	1.061674	0.636	0.155	4.56E-20	Blood_CD3	CLNS1A
CSK2	5.47E-24	0.955462	0.436	0.076	8.73E-20	Blood_CD3	CSK
RPLP03	6.52E-24	1.295092	1	0.798	1.04E-19	Blood_CD3	RPLP0
PSTPIP14	7.41E-24	0.425216	0.236	0.024	1.18E-19	Blood_CD3	PSTPIP1
UQCRC13	7.81E-24	1.359405	0.691	0.193	1.24E-19	Blood_CD3	UQCRC1

DDOST1	8.84E-24	1.115716	0.709	0.195	1.41E-19	Blood_CD3	DDOST
DEF6	9E-24	0.756927	0.4	0.064	1.44E-19	Blood_CD3	DEF6
TPMT	9.65E-24	0.925862	0.4	0.069	1.54E-19	Blood_CD3	TPMT
FARS2	9.76E-24	0.747758	0.309	0.042	1.56E-19	Blood_CD3	FARS2
PRMT18	1.47E-23	0.951299	0.727	0.192	2.34E-19	Blood_CD3	PRMT1
IDH23	1.69E-23	1.143514	0.691	0.194	2.7E-19	Blood_CD3	IDH2
FERMT34	1.75E-23	0.778487	0.345	0.049	2.78E-19	Blood_CD3	FERMT3
CTSZ9	2.01E-23	0.649232	0.745	0.192	3.21E-19	Blood_CD3	CTSZ
FGL26	2.03E-23	1.065497	0.509	0.099	3.24E-19	Blood_CD3	FGL2
IFT202	2.14E-23	1.11007	0.618	0.159	3.41E-19	Blood_CD3	IFT20
TMEM14A	2.65E-23	0.887033	0.455	0.085	4.23E-19	Blood_CD3	TMEM14A
HMGA11	2.89E-23	1.182181	0.855	0.285	4.6E-19	Blood_CD3	HMGA1
MCM51	3.06E-23	0.634727	0.273	0.033	4.88E-19	Blood_CD3	MCM5
RNF1663	3.19E-23	0.47524	0.345	0.049	5.09E-19	Blood_CD3	RNF166
THOC61	3.93E-23	0.816736	0.364	0.057	6.26E-19	Blood_CD3	THOC6
HLA.F3	4.16E-23	0.890634	0.564	0.12	6.64E-19	Blood_CD3	HLA.F
EIF3K4	4.36E-23	1.277273	1	0.518	6.95E-19	Blood_CD3	EIF3K
RPL88	5.26E-23	1.132597	1	0.849	8.39E-19	Blood_CD3	RPL8
BIN11	5.45E-23	0.787296	0.564	0.118	8.7E-19	Blood_CD3	BIN1
FKBP1A7	6.21E-23	1.322948	0.891	0.338	9.9E-19	Blood_CD3	FKBP1A
KLHL22	6.89E-23	0.487068	0.218	0.022	1.1E-18	Blood_CD3	KLHL22
CYB561A3	7.68E-23	0.908865	0.382	0.064	1.22E-18	Blood_CD3	CYB561A3
PTPN222	8.94E-23	0.329849	0.182	0.016	1.43E-18	Blood_CD3	PTPN22
TPI16	9.02E-23	1.393701	1	0.535	1.44E-18	Blood_CD3	TPI1
NPM31	1.11E-22	0.740034	0.455	0.085	1.76E-18	Blood_CD3	NPM3
GNB2L13	1.25E-22	1.265533	1	0.753	1.99E-18	Blood_CD3	GNB2L1
NBEAL19	1.88E-22	1.106811	0.927	0.32	2.99E-18	Blood_CD3	NBEAL1
TRABD3	2.06E-22	0.989727	0.527	0.118	3.29E-18	Blood_CD3	TRABD
SHKBP1	2.19E-22	0.772291	0.436	0.081	3.5E-18	Blood_CD3	SHKBP1
NCKAP1L4	2.43E-22	0.415008	0.218	0.022	3.87E-18	Blood_CD3	NCKAP1L
ADAP23	3.4E-22	0.390203	0.182	0.016	5.42E-18	Blood_CD3	ADAP2
ANPEP4	4.27E-22	0.534936	0.273	0.034	6.81E-18	Blood_CD3	ANPEP
HLA.A4	4.51E-22	1.401059	1	0.669	7.19E-18	Blood_CD3	HLA.A
CD381	4.55E-22	0.438165	0.145	0.011	7.26E-18	Blood_CD3	CD38
APH1A	5.23E-22	0.939117	0.655	0.176	8.35E-18	Blood_CD3	APH1A
PAFAH1B3	5.4E-22	0.838613	0.455	0.09	8.62E-18	Blood_CD3	PAFAH1B3
APEX11	8.79E-22	0.997357	0.636	0.165	1.4E-17	Blood_CD3	APEX1
ARHGAP44	9.23E-22	0.476535	0.273	0.034	1.47E-17	Blood_CD3	ARHGAP4
KCNK67	1.47E-21	0.942684	0.473	0.101	2.35E-17	Blood_CD3	KCNK6
ZNF296	1.63E-21	0.541063	0.2	0.02	2.6E-17	Blood_CD3	ZNF296
CD862	1.98E-21	0.446799	0.218	0.023	3.15E-17	Blood_CD3	CD86
FAM160A2	2.42E-21	0.544908	0.309	0.046	3.85E-17	Blood_CD3	FAM160A2
ATP5L22	2.72E-21	0.455893	0.2	0.02	4.34E-17	Blood_CD3	ATP5L2
PTPN64	3.38E-21	0.841457	0.418	0.076	5.4E-17	Blood_CD3	PTPN6
NMI1	3.46E-21	0.679269	0.382	0.067	5.52E-17	Blood_CD3	NMI
REC8	4.29E-21	0.629619	0.255	0.032	6.85E-17	Blood_CD3	REC8
HIGD2A3	4.51E-21	1.196546	0.891	0.352	7.2E-17	Blood_CD3	HIGD2A

VIM14	4.62E-21	1.224877	1	0.495	7.36E-17	Blood_CD3	VIM
TUBA1B12	4.73E-21	1.448876	0.927	0.354	7.55E-17	Blood_CD3	TUBA1B
ATP5G25	5.55E-21	1.188377	1	0.573	8.85E-17	Blood_CD3	ATP5G2
PABPC1	5.65E-21	1.176908	1	0.721	9.02E-17	Blood_CD3	PABPC1
THEMIS24	5.66E-21	0.765444	0.291	0.041	9.02E-17	Blood_CD3	THEMIS2
PSAP6	5.68E-21	1.012701	0.964	0.435	9.06E-17	Blood_CD3	PSAP
RPSA2	5.71E-21	1.247913	1	0.678	9.11E-17	Blood_CD3	RPSA
PARK713	7.25E-21	1.113629	0.927	0.356	1.16E-16	Blood_CD3	PARK7
NAP1L17	8.45E-21	1.224465	0.945	0.402	1.35E-16	Blood_CD3	NAP1L1
RHOF2	8.52E-21	0.667625	0.436	0.082	1.36E-16	Blood_CD3	RHOF
EIF3F2	9.99E-21	1.17632	0.982	0.44	1.59E-16	Blood_CD3	EIF3F
EEF1B25	1.25E-20	1.286492	1	0.597	2E-16	Blood_CD3	EEF1B2
ATPAF21	1.27E-20	0.711661	0.327	0.053	2.03E-16	Blood_CD3	ATPAF2
PCBP25	1.67E-20	1.30956	0.927	0.421	2.67E-16	Blood_CD3	PCBP2
NUDT161	1.77E-20	0.941629	0.4	0.078	2.82E-16	Blood_CD3	NUDT16
PHB24	1.87E-20	0.990635	0.673	0.196	2.99E-16	Blood_CD3	PHB2
S100B3	2.39E-20	1.146168	0.236	0.028	3.81E-16	Blood_CD3	S100B
TKT2	2.74E-20	0.949456	0.636	0.174	4.37E-16	Blood_CD3	TKT
PQBP18	3.14E-20	0.900755	0.6	0.154	5.01E-16	Blood_CD3	PQBP1
EVI2B5	3.79E-20	0.530604	0.382	0.064	6.05E-16	Blood_CD3	EVI2B
ANXA63	4.21E-20	0.708324	0.509	0.108	6.72E-16	Blood_CD3	ANXA6
NOL121	5.68E-20	0.583236	0.364	0.064	9.06E-16	Blood_CD3	NOL12
TSPAN334	7.48E-20	0.665199	0.291	0.044	1.19E-15	Blood_CD3	TSPAN33
PQLC2	8.56E-20	0.464603	0.182	0.018	1.37E-15	Blood_CD3	PQLC2
RAB33A	1.02E-19	0.411521	0.145	0.012	1.63E-15	Blood_CD3	RAB33A
TPP14	1.03E-19	0.915618	0.455	0.097	1.64E-15	Blood_CD3	TPP1
AGPAT1	1.07E-19	0.649371	0.364	0.066	1.71E-15	Blood_CD3	AGPAT1
SLC43A23	1.09E-19	0.525153	0.255	0.034	1.74E-15	Blood_CD3	SLC43A2
SEPHS24	1.51E-19	0.680657	0.364	0.064	2.41E-15	Blood_CD3	SEPHS2
EIF3H	1.77E-19	1.232676	0.945	0.455	2.82E-15	Blood_CD3	EIF3H
SPAG73	1.79E-19	1.010794	0.618	0.176	2.85E-15	Blood_CD3	SPAG7
CCT82	1.85E-19	0.884903	0.691	0.205	2.95E-15	Blood_CD3	CCT8
SNX103	2.19E-19	0.613009	0.327	0.053	3.49E-15	Blood_CD3	SNX10
RPS220	2.83E-19	1.270522	1	0.8	4.52E-15	Blood_CD3	RPS2
TNFSF13B5	2.94E-19	0.437531	0.291	0.042	4.69E-15	Blood_CD3	TNFSF13B
GTF3A1	2.94E-19	0.962722	0.764	0.25	4.69E-15	Blood_CD3	GTF3A
BEND5	3.65E-19	0.472248	0.182	0.019	5.82E-15	Blood_CD3	BEND5
ABHD14B	3.8E-19	0.801291	0.509	0.12	6.06E-15	Blood_CD3	ABHD14B
CSRP15	3.94E-19	0.770305	0.582	0.155	6.29E-15	Blood_CD3	CSRP1
APOBR1	4.25E-19	0.296087	0.127	0.01	6.78E-15	Blood_CD3	APOBR
LDHB	4.29E-19	0.848262	0.891	0.315	6.84E-15	Blood_CD3	LDHB
SPG214	4.89E-19	0.831885	0.582	0.157	7.8E-15	Blood_CD3	SPG21
MAPKAPK3	5.6E-19	0.63361	0.327	0.056	8.93E-15	Blood_CD3	MAPKAPK3
SERPINF13	5.85E-19	0.698611	0.582	0.139	9.33E-15	Blood_CD3	SERPINF1
HNRNPA16	6.85E-19	1.254635	1	0.546	1.09E-14	Blood_CD3	HNRNPA1
SLC25A314	7.33E-19	1.197489	0.927	0.456	1.17E-14	Blood_CD3	SLC25A3
SEPT63	7.39E-19	0.663631	0.455	0.095	1.18E-14	Blood_CD3	SEPT6

POLR2G1	7.77E-19	0.872597	0.6	0.161	1.24E-14	Blood_CD3	POLR2G
STARD75	8.12E-19	0.723305	0.436	0.094	1.29E-14	Blood_CD3	STARD7
PPA1	8.61E-19	1.152151	0.927	0.382	1.37E-14	Blood_CD3	PPA1
RPL62	9.57E-19	1.076101	1	0.793	1.53E-14	Blood_CD3	RPL6
GEMIN7	1.04E-18	0.606112	0.309	0.051	1.66E-14	Blood_CD3	GEMIN7
SNX171	1.09E-18	0.815578	0.545	0.141	1.74E-14	Blood_CD3	SNX17
NDUFS84	1.44E-18	1.068955	0.764	0.265	2.29E-14	Blood_CD3	NDUFS8
RPL56	1.47E-18	1.036543	1	0.728	2.34E-14	Blood_CD3	RPL5
RBM311	1.6E-18	1.216429	0.945	0.44	2.56E-14	Blood_CD3	RBM3
MYL610	1.62E-18	1.024723	1	0.806	2.58E-14	Blood_CD3	MYL6
TUBA1A8	1.66E-18	1.156015	0.727	0.235	2.65E-14	Blood_CD3	TUBA1A
PRELID15	2.03E-18	1.012661	0.927	0.379	3.24E-14	Blood_CD3	PRELID1
CSF3R2	2.37E-18	0.353635	0.164	0.016	3.78E-14	Blood_CD3	CSF3R
SERPINB1	2.93E-18	0.710432	0.836	0.282	4.68E-14	Blood_CD3	SERPINB1
VAMP86	4.02E-18	1.019858	0.964	0.409	6.42E-14	Blood_CD3	VAMP8
ARHGAP30	4.08E-18	0.503347	0.309	0.049	6.5E-14	Blood_CD3	ARHGAP30
PPP1CA4	4.28E-18	0.951405	0.836	0.314	6.82E-14	Blood_CD3	PPP1CA
COPE2	4.36E-18	1.085679	0.945	0.441	6.95E-14	Blood_CD3	COPE
CTSS4	4.85E-18	0.63284	0.727	0.216	7.73E-14	Blood_CD3	CTSS
MZT2A1	5.18E-18	1.134472	0.8	0.312	8.26E-14	Blood_CD3	MZT2A
PAK12	6.33E-18	0.746149	0.345	0.065	1.01E-13	Blood_CD3	PAK1
EIF4EBP16	7.62E-18	0.927145	0.545	0.151	1.21E-13	Blood_CD3	EIF4EBP1
LAP34	8.6E-18	0.948967	0.491	0.126	1.37E-13	Blood_CD3	LAP3
POLR2E3	8.72E-18	0.841483	0.655	0.2	1.39E-13	Blood_CD3	POLR2E
COX4I12	8.8E-18	0.927966	1	0.725	1.4E-13	Blood_CD3	COX4I1
C7orf505	9.04E-18	1.092093	0.509	0.135	1.44E-13	Blood_CD3	C7orf50
PLBD12	9.33E-18	0.825784	0.4	0.085	1.49E-13	Blood_CD3	PLBD1
FBXO27	9.5E-18	0.327925	0.127	0.01	1.52E-13	Blood_CD3	FBXO27
SYNGR25	9.6E-18	1.118417	0.691	0.234	1.53E-13	Blood_CD3	SYNGR2
ECH12	1.13E-17	1.211518	0.655	0.221	1.79E-13	Blood_CD3	ECH1
ATP5SL	1.19E-17	0.71359	0.327	0.059	1.9E-13	Blood_CD3	ATP5SL
MRPL531	1.47E-17	0.420015	0.236	0.032	2.34E-13	Blood_CD3	MRPL53
RPS66	1.64E-17	1.033554	1	0.825	2.61E-13	Blood_CD3	RPS6
CADM15	1.82E-17	0.796906	0.436	0.104	2.9E-13	Blood_CD3	CADM1
CRIP16	1.83E-17	0.651803	0.855	0.277	2.91E-13	Blood_CD3	CRIP1
C9orf1422	2.56E-17	0.504348	0.564	0.146	4.08E-13	Blood_CD3	C9orf142
PLP29	3.03E-17	0.788301	0.745	0.232	4.83E-13	Blood_CD3	PLP2
SPNS31	3.21E-17	0.594252	0.255	0.038	5.12E-13	Blood_CD3	SPNS3
DOCK24	3.79E-17	0.330465	0.182	0.02	6.04E-13	Blood_CD3	DOCK2
HPS12	3.89E-17	0.713708	0.418	0.095	6.2E-13	Blood_CD3	HPS1
CHKB	4.39E-17	0.461902	0.218	0.029	7E-13	Blood_CD3	CHKB
PSMB33	4.45E-17	0.902582	0.873	0.345	7.09E-13	Blood_CD3	PSMB3
RPS4X5	4.67E-17	0.96493	1	0.771	7.46E-13	Blood_CD3	RPS4X
RPS6KB22	4.81E-17	0.848568	0.455	0.112	7.67E-13	Blood_CD3	RPS6KB2
BRK14	5.11E-17	1.022838	0.927	0.455	8.16E-13	Blood_CD3	BRK1
PSMB17	5.13E-17	0.985354	0.891	0.4	8.19E-13	Blood_CD3	PSMB1
NSMCE11	5.36E-17	0.842231	0.545	0.148	8.54E-13	Blood_CD3	NSMCE1

ZRSR21	5.4E-17	0.513316	0.273	0.043	8.62E-13	Blood_CD3	ZRSR2
CDKN2C2	5.53E-17	0.522875	0.255	0.038	8.82E-13	Blood_CD3	CDKN2C
TAPBP9	5.64E-17	0.784729	0.582	0.164	8.99E-13	Blood_CD3	TAPBP
YDJC1	7.47E-17	0.663591	0.327	0.061	1.19E-12	Blood_CD3	YDJC
TUFM3	7.98E-17	0.996951	0.764	0.292	1.27E-12	Blood_CD3	TUFM
COX5A3	8.45E-17	1.201079	0.873	0.412	1.35E-12	Blood_CD3	COX5A
RPS75	8.45E-17	0.941874	1	0.816	1.35E-12	Blood_CD3	RPS7
NDUFS73	1.06E-16	1.030506	0.745	0.273	1.69E-12	Blood_CD3	NDUFS7
EIF3M2	1.07E-16	0.902227	0.745	0.26	1.71E-12	Blood_CD3	EIF3M
EEF1G	1.16E-16	0.539809	0.564	0.145	1.85E-12	Blood_CD3	EEF1G
RPL36A6	1.44E-16	0.950284	1	0.511	2.29E-12	Blood_CD3	RPL36A
APRT7	1.46E-16	0.742286	0.945	0.369	2.34E-12	Blood_CD3	APRT
FBL4	1.56E-16	0.90758	0.709	0.236	2.49E-12	Blood_CD3	FBL
MDH24	1.6E-16	1.220288	0.691	0.252	2.55E-12	Blood_CD3	MDH2
CXCR43	1.62E-16	0.380289	0.527	0.123	2.59E-12	Blood_CD3	CXCR4
ATP5D4	1.67E-16	1.081769	0.909	0.447	2.66E-12	Blood_CD3	ATP5D
RSL1D14	1.75E-16	0.972062	0.727	0.249	2.79E-12	Blood_CD3	RSL1D1
SRSF211	2.07E-16	0.865586	0.855	0.332	3.29E-12	Blood_CD3	SRSF2
CCDC121	2.22E-16	0.766705	0.564	0.163	3.55E-12	Blood_CD3	CCDC12
LAMTOR45	2.4E-16	0.989495	0.873	0.389	3.83E-12	Blood_CD3	LAMTOR4
PRR131	2.59E-16	0.940095	0.8	0.306	4.13E-12	Blood_CD3	PRR13
NIPSNAP3A	2.67E-16	0.819249	0.309	0.058	4.26E-12	Blood_CD3	NIPSNAP3A
ATP6V0B9	2.81E-16	1.069555	0.873	0.388	4.48E-12	Blood_CD3	ATP6V0B
ASNA1	2.83E-16	0.896718	0.564	0.169	4.52E-12	Blood_CD3	ASNA1
PSMD91	2.85E-16	0.771175	0.455	0.113	4.54E-12	Blood_CD3	PSMD9
DCTN22	3.55E-16	0.746341	0.618	0.195	5.66E-12	Blood_CD3	DCTN2
GRHPR2	3.87E-16	0.898505	0.509	0.139	6.17E-12	Blood_CD3	GRHPR
TMEM243	4.89E-16	0.773128	0.491	0.129	7.8E-12	Blood_CD3	TMEM243
ARRDC1	5.17E-16	0.875811	0.509	0.142	8.24E-12	Blood_CD3	ARRDC1
DCPS1	5.24E-16	0.554366	0.309	0.058	8.35E-12	Blood_CD3	DCPS
CALHM26	6.19E-16	0.454885	0.236	0.034	9.87E-12	Blood_CD3	CALHM2
C19orf607	6.77E-16	0.730024	0.691	0.229	1.08E-11	Blood_CD3	C19orf60
TNFAIP8L2	6.97E-16	0.353278	0.145	0.014	1.11E-11	Blood_CD3	TNFAIP8L2
FXYD52	7.12E-16	0.57105	0.891	0.299	1.14E-11	Blood_CD3	FXYD5
CLIC110	7.33E-16	1.089706	0.927	0.475	1.17E-11	Blood_CD3	CLIC1
PPIB8	7.43E-16	1.083306	0.891	0.394	1.19E-11	Blood_CD3	PPIB
RELT3	8.5E-16	0.441719	0.218	0.031	1.36E-11	Blood_CD3	RELT
ODF3B3	9.51E-16	0.480632	0.4	0.088	1.52E-11	Blood_CD3	ODF3B
ARL6IP43	1E-15	0.908286	0.945	0.421	1.6E-11	Blood_CD3	ARL6IP4
SCFD2	1.2E-15	0.321525	0.127	0.012	1.91E-11	Blood_CD3	SCFD2
ATG16L22	1.31E-15	0.579332	0.218	0.031	2.09E-11	Blood_CD3	ATG16L2
VASP5	1.4E-15	0.698419	0.491	0.13	2.23E-11	Blood_CD3	VASP
RNASE63	1.61E-15	0.639358	0.255	0.04	2.57E-11	Blood_CD3	RNASE6
CCDC88A2	1.83E-15	0.605017	0.418	0.096	2.92E-11	Blood_CD3	CCDC88A
RPL157	1.96E-15	0.790396	1	0.895	3.13E-11	Blood_CD3	RPL15
SNF81	2.49E-15	0.757325	0.618	0.205	3.97E-11	Blood_CD3	SNF8
OGFR1	2.65E-15	0.595928	0.4	0.092	4.23E-11	Blood_CD3	OGFR

GSTK12	2.92E-15	0.939303	0.764	0.297	4.65E-11	Blood_CD3	GSTK1
DNAJC41	3.19E-15	0.567326	0.582	0.18	5.09E-11	Blood_CD3	DNAJC4
ARPC43	3.46E-15	0.62196	0.691	0.232	5.53E-11	Blood_CD3	ARPC4
SNRPB7	3.47E-15	0.826582	0.836	0.334	5.53E-11	Blood_CD3	SNRPB
MARCKSL1	3.49E-15	0.740224	0.636	0.206	5.56E-11	Blood_CD3	MARCKSL1
TRIM224	3.51E-15	0.572557	0.291	0.053	5.6E-11	Blood_CD3	TRIM22
AP2M14	3.72E-15	0.815515	0.782	0.304	5.93E-11	Blood_CD3	AP2M1
HMGN1	3.78E-15	0.922919	0.927	0.453	6.02E-11	Blood_CD3	HMGN1
SEC133	3.86E-15	0.862371	0.473	0.135	6.16E-11	Blood_CD3	SEC13
LSM26	4.42E-15	0.852467	0.564	0.169	7.05E-11	Blood_CD3	LSM2
WBSCR221	4.51E-15	0.842077	0.582	0.186	7.19E-11	Blood_CD3	WBSCR22
LSM102	4.63E-15	0.609112	0.491	0.132	7.38E-11	Blood_CD3	LSM10
DECR12	4.72E-15	0.954574	0.527	0.163	7.53E-11	Blood_CD3	DECR1
NOP563	5.32E-15	0.687701	0.473	0.125	8.48E-11	Blood_CD3	NOP56
PPIA6	5.7E-15	0.961526	0.964	0.59	9.1E-11	Blood_CD3	PPIA
MRPL46	5.88E-15	0.648181	0.455	0.12	9.38E-11	Blood_CD3	MRPL4
RP11.1094	6.49E-15	0.515214	0.255	0.043	1.03E-10	Blood_CD3	RP11.1094M14.11
NIT21	7.53E-15	0.581456	0.382	0.089	1.2E-10	Blood_CD3	NIT2
LGMN1	7.54E-15	0.583098	0.473	0.128	1.2E-10	Blood_CD3	LGMN
HDAC3	7.59E-15	0.709633	0.364	0.082	1.21E-10	Blood_CD3	HDAC3
TMSB4X8	7.74E-15	0.847146	1	0.871	1.23E-10	Blood_CD3	TMSB4X
AUP13	7.8E-15	0.898655	0.6	0.204	1.24E-10	Blood_CD3	AUP1
YBX18	8.22E-15	0.827849	0.982	0.589	1.31E-10	Blood_CD3	YBX1
APBB1IP2	8.58E-15	0.434994	0.255	0.041	1.37E-10	Blood_CD3	APBB1IP
RPS3A4	8.67E-15	0.803289	1	0.831	1.38E-10	Blood_CD3	RPS3A
BTF37	8.68E-15	0.991653	1	0.712	1.38E-10	Blood_CD3	BTF3
ARF63	9.26E-15	1.011275	0.855	0.377	1.48E-10	Blood_CD3	ARF6
GNB21	9.36E-15	0.993022	0.818	0.362	1.49E-10	Blood_CD3	GNB2
IDH3B2	9.62E-15	0.8062	0.473	0.132	1.53E-10	Blood_CD3	IDH3B
VPS287	9.84E-15	0.87061	0.818	0.349	1.57E-10	Blood_CD3	VPS28
HDAC101	1.07E-14	0.322122	0.182	0.023	1.7E-10	Blood_CD3	HDAC10
RPS34	1.09E-14	0.833197	1	0.817	1.74E-10	Blood_CD3	RPS3
NACA26	1.09E-14	0.589894	0.345	0.073	1.74E-10	Blood_CD3	NACA2
ADORA2B1	1.36E-14	0.352853	0.127	0.012	2.17E-10	Blood_CD3	ADORA2B
GSTO17	1.36E-14	0.788085	0.636	0.213	2.17E-10	Blood_CD3	GSTO1
PPP1R184	1.55E-14	0.478778	0.473	0.118	2.46E-10	Blood_CD3	PPP1R18
FBXL15	1.59E-14	0.680927	0.436	0.114	2.53E-10	Blood_CD3	FBXL15
ATP5C15	1.83E-14	0.955299	0.709	0.282	2.92E-10	Blood_CD3	ATP5C1
MRPL55	1.91E-14	0.815443	0.618	0.212	3.04E-10	Blood_CD3	MRPL55
PSMA78	2.14E-14	0.881401	0.945	0.464	3.42E-10	Blood_CD3	PSMA7
GLB12	2.41E-14	0.738524	0.327	0.072	3.84E-10	Blood_CD3	GLB1
BCKDK3	2.41E-14	0.747253	0.4	0.103	3.84E-10	Blood_CD3	BCKDK
ELMO15	2.42E-14	0.430813	0.218	0.033	3.87E-10	Blood_CD3	ELMO1
APOA1BP3	2.58E-14	0.645481	0.473	0.132	4.12E-10	Blood_CD3	APOA1BP
QPRT3	2.66E-14	0.477086	0.182	0.024	4.24E-10	Blood_CD3	QPRT
NACA3	3E-14	0.812505	1	0.831	4.79E-10	Blood_CD3	NACA
PSMB66	3.04E-14	0.968584	0.764	0.322	4.84E-10	Blood_CD3	PSMB6

IMPDH13	3.18E-14	0.554275	0.273	0.051	5.07E-10	Blood_CD3	IMPDH1
RPS56	3.58E-14	0.830739	1	0.786	5.72E-10	Blood_CD3	RPS5
FUOM3	3.73E-14	0.621648	0.345	0.078	5.94E-10	Blood_CD3	FUOM
SRI6	4.12E-14	0.816273	0.673	0.242	6.57E-10	Blood_CD3	SRI
PNMA12	4.12E-14	0.58416	0.273	0.051	6.57E-10	Blood_CD3	PNMA1
TRADD2	4.22E-14	0.715195	0.436	0.114	6.73E-10	Blood_CD3	TRADD
SAAL1	4.64E-14	0.492234	0.2	0.03	7.4E-10	Blood_CD3	SAAL1
SERPINB62	4.74E-14	0.757957	0.636	0.224	7.55E-10	Blood_CD3	SERPINB6
NAA105	5.4E-14	0.63744	0.527	0.16	8.61E-10	Blood_CD3	NAA10
UBXN14	5.78E-14	0.848184	0.873	0.392	9.22E-10	Blood_CD3	UBXN1
ATP6V1F1	5.88E-14	0.81951	0.909	0.419	9.38E-10	Blood_CD3	ATP6V1F
UQCRC23	7.09E-14	0.757903	0.6	0.209	1.13E-09	Blood_CD3	UQCRC2
NDUFS45	7.09E-14	0.975651	0.6	0.218	1.13E-09	Blood_CD3	NDUFS4
ITPA	7.17E-14	0.741093	0.4	0.103	1.14E-09	Blood_CD3	ITPA
TMEM109	7.36E-14	0.496781	0.473	0.128	1.17E-09	Blood_CD3	TMEM109
RPL10A8	7.37E-14	0.83325	1	0.735	1.18E-09	Blood_CD3	RPL10A
SH3BGRL3	8.21E-14	0.799897	1	0.633	1.31E-09	Blood_CD3	SH3BGRL3
U2AF110	1.05E-13	0.729566	0.727	0.256	1.68E-09	Blood_CD3	U2AF1
ANXA512	1.14E-13	0.72638	0.818	0.318	1.83E-09	Blood_CD3	ANXA5
ARRB23	1.17E-13	0.503872	0.382	0.087	1.87E-09	Blood_CD3	ARRB2
RAMP13	1.29E-13	0.342842	0.182	0.025	2.05E-09	Blood_CD3	RAMP1
RRP7A1	1.41E-13	0.666572	0.436	0.122	2.25E-09	Blood_CD3	RRP7A
PDLIM76	1.49E-13	0.487495	0.309	0.064	2.38E-09	Blood_CD3	PDLIM7
SLC35C21	1.58E-13	0.67474	0.345	0.082	2.52E-09	Blood_CD3	SLC35C2
KCNAB23	1.9E-13	0.311148	0.182	0.025	3.03E-09	Blood_CD3	KCNAB2
PLD42	2.02E-13	0.278009	0.109	0.01	3.22E-09	Blood_CD3	PLD4
TOP1MT	2.03E-13	0.535341	0.218	0.036	3.24E-09	Blood_CD3	TOP1MT
NPM15	2.16E-13	0.863407	0.964	0.534	3.45E-09	Blood_CD3	NPM1
EFHD22	2.25E-13	0.742974	0.636	0.22	3.59E-09	Blood_CD3	EFHD2
UROD1	2.35E-13	0.590536	0.382	0.096	3.75E-09	Blood_CD3	UROD
MLF22	2.47E-13	0.789071	0.618	0.225	3.94E-09	Blood_CD3	MLF2
DHPS	2.52E-13	0.579645	0.455	0.129	4.01E-09	Blood_CD3	DHPS
ADAM283	2.53E-13	0.412898	0.182	0.026	4.04E-09	Blood_CD3	ADAM28
EIF63	2.62E-13	0.811454	0.691	0.269	4.19E-09	Blood_CD3	EIF6
COX7A2L3	2.69E-13	0.791213	0.782	0.321	4.3E-09	Blood_CD3	COX7A2L
RPL144	2.73E-13	0.752268	1	0.796	4.36E-09	Blood_CD3	RPL14
LSM41	2.75E-13	0.847378	0.655	0.239	4.38E-09	Blood_CD3	LSM4
RGS142	2.77E-13	0.44809	0.255	0.046	4.42E-09	Blood_CD3	RGS14
CCDC1122	2.8E-13	0.59445	0.291	0.06	4.46E-09	Blood_CD3	CCDC112
TMX16	3.33E-13	0.769876	0.4	0.105	5.31E-09	Blood_CD3	TMX1
THYN1	3.92E-13	0.654035	0.418	0.113	6.25E-09	Blood_CD3	THYN1
VOPP13	3.97E-13	0.641633	0.418	0.115	6.33E-09	Blood_CD3	VOPP1
SUPT4H13	4.06E-13	0.804576	0.636	0.243	6.47E-09	Blood_CD3	SUPT4H1
C19orf431	4.15E-13	0.882443	0.945	0.504	6.62E-09	Blood_CD3	C19orf43
IGBP1	4.65E-13	0.999832	0.582	0.217	7.42E-09	Blood_CD3	IGBP1
TMEM206	4.94E-13	0.612617	0.2	0.032	7.87E-09	Blood_CD3	TMEM206
NOTCH2NL	4.94E-13	0.524245	0.364	0.087	7.88E-09	Blood_CD3	NOTCH2NL

CAP11	5.03E-13	0.880088	0.873	0.412	8.02E-09	Blood_CD3	CAP1
METTL121	5.5E-13	0.465119	0.255	0.047	8.76E-09	Blood_CD3	METTL12
URM12	5.58E-13	0.653209	0.4	0.106	8.9E-09	Blood_CD3	URM1
C20orf243	5.72E-13	0.667054	0.509	0.155	9.12E-09	Blood_CD3	C20orf24
MPG5	6.62E-13	0.75439	0.527	0.173	1.06E-08	Blood_CD3	MPG
KCTD17	6.75E-13	0.365858	0.164	0.022	1.08E-08	Blood_CD3	KCTD17
FKBP83	6.88E-13	0.704151	0.782	0.339	1.1E-08	Blood_CD3	FKBP8
FAM118A1	8.07E-13	0.506231	0.327	0.073	1.29E-08	Blood_CD3	FAM118A
PREX14	8.24E-13	0.307732	0.164	0.022	1.31E-08	Blood_CD3	PREX1
AURKAIP1:	8.65E-13	0.507284	0.873	0.377	1.38E-08	Blood_CD3	AURKAIP1
EEF26	8.8E-13	0.875539	1	0.608	1.4E-08	Blood_CD3	EEF2
IDH3A	9.23E-13	0.578252	0.345	0.084	1.47E-08	Blood_CD3	IDH3A
VDAC17	1.06E-12	0.759827	0.636	0.242	1.69E-08	Blood_CD3	VDAC1
RILP1	1.07E-12	0.619237	0.273	0.057	1.71E-08	Blood_CD3	RILP
HSPA89	1.08E-12	0.797361	0.982	0.511	1.72E-08	Blood_CD3	HSPA8
PDHB1	1.08E-12	0.656236	0.418	0.117	1.72E-08	Blood_CD3	PDHB
RPL195	1.15E-12	0.691071	1	0.849	1.84E-08	Blood_CD3	RPL19
FLOT14	1.3E-12	0.628097	0.473	0.143	2.08E-08	Blood_CD3	FLOT1
RP4.737E2	1.31E-12	0.266901	0.109	0.011	2.09E-08	Blood_CD3	RP4.737E23.2
TMED94	1.41E-12	0.71116	0.727	0.292	2.25E-08	Blood_CD3	TMED9
SH3BGRL1(	1.44E-12	0.720461	0.673	0.245	2.29E-08	Blood_CD3	SH3BGRL
MTCH21	1.51E-12	0.666293	0.455	0.135	2.41E-08	Blood_CD3	MTCH2
EIF5AL1	1.63E-12	0.38196	0.218	0.038	2.6E-08	Blood_CD3	EIF5AL1
DNAJC155	1.64E-12	1.11923	0.545	0.19	2.62E-08	Blood_CD3	DNAJC15
R3HDM4	1.72E-12	0.565512	0.455	0.131	2.74E-08	Blood_CD3	R3HDM4
MRPS7	1.89E-12	0.6897	0.491	0.159	3.01E-08	Blood_CD3	MRPS7
FAM162A4	1.97E-12	0.779093	0.545	0.194	3.14E-08	Blood_CD3	FAM162A
NUP93	1.99E-12	0.496232	0.218	0.038	3.17E-08	Blood_CD3	NUP93
PDCD4.AS1	2.25E-12	0.362835	0.145	0.018	3.59E-08	Blood_CD3	PDCD4.AS1
PARP13	2.33E-12	0.544299	0.436	0.124	3.71E-08	Blood_CD3	PARP1
CD833	2.34E-12	0.370824	0.382	0.096	3.74E-08	Blood_CD3	CD83
OAZ27	2.36E-12	0.737133	0.527	0.178	3.77E-08	Blood_CD3	OAZ2
ETFA	2.48E-12	0.746816	0.455	0.144	3.95E-08	Blood_CD3	ETFA
RGCC10	2.79E-12	0.446083	0.655	0.214	4.46E-08	Blood_CD3	RGCC
HNRNPDL8	2.82E-12	0.704753	0.836	0.337	4.49E-08	Blood_CD3	HNRNPDL
PMPCB	3.03E-12	0.678482	0.436	0.131	4.83E-08	Blood_CD3	PMPCB
SLC25A392	3.03E-12	0.534251	0.527	0.177	4.83E-08	Blood_CD3	SLC25A39
NME2	3.55E-12	0.334524	0.182	0.028	5.66E-08	Blood_CD3	NME2
BAX2	3.63E-12	0.655444	0.527	0.169	5.78E-08	Blood_CD3	BAX
ERP292	3.84E-12	0.654199	0.818	0.355	6.13E-08	Blood_CD3	ERP29
DDRGK14	3.99E-12	0.783147	0.473	0.152	6.36E-08	Blood_CD3	DDRGK1
ECSIT1	4.04E-12	0.702345	0.345	0.088	6.45E-08	Blood_CD3	ECSIT
C21orf332	4.06E-12	0.414135	0.273	0.056	6.48E-08	Blood_CD3	C21orf33
NUDT17	4.19E-12	0.312831	0.091	0.008	6.69E-08	Blood_CD3	NUDT17
NDUFA104	4.23E-12	0.749054	0.527	0.184	6.75E-08	Blood_CD3	NDUFA10
LGALS112	4.28E-12	0.367548	0.982	0.411	6.83E-08	Blood_CD3	LGALS1
GALK13	4.31E-12	0.491997	0.291	0.065	6.87E-08	Blood_CD3	GALK1

MRPL31	4.56E-12	0.67241	0.436	0.132	7.28E-08	Blood_CD3	MRPL3
G6PC3	4.69E-12	0.487939	0.255	0.052	7.49E-08	Blood_CD3	G6PC3
TIMM134	4.97E-12	0.728122	0.636	0.247	7.92E-08	Blood_CD3	TIMM13
TRMT1129	5.36E-12	0.756533	0.818	0.374	8.55E-08	Blood_CD3	TRMT112
TRAPPC6A	5.43E-12	0.516391	0.473	0.143	8.66E-08	Blood_CD3	TRAPPC6A
HNRNPA08	5.57E-12	0.591442	0.818	0.335	8.89E-08	Blood_CD3	HNRNPA0
AAMP2	5.59E-12	0.555927	0.455	0.139	8.92E-08	Blood_CD3	AAMP
FLOT21	6.11E-12	0.599433	0.273	0.059	9.75E-08	Blood_CD3	FLOT2
ZDHHC242	6.92E-12	0.464662	0.273	0.059	1.1E-07	Blood_CD3	ZDHHC24
MRPL123	7.02E-12	0.686904	0.418	0.125	1.12E-07	Blood_CD3	MRPL12
MRPL161	7.02E-12	0.643276	0.382	0.105	1.12E-07	Blood_CD3	MRPL16
SDHC	7.51E-12	0.704708	0.582	0.214	1.2E-07	Blood_CD3	SDHC
RAC19	8.04E-12	0.742779	1	0.548	1.28E-07	Blood_CD3	RAC1
KLF611	8.05E-12	0.689833	0.873	0.402	1.28E-07	Blood_CD3	KLF6
TMEM219	8.36E-12	0.78836	0.745	0.332	1.33E-07	Blood_CD3	TMEM219
PFDN29	8.66E-12	0.693056	0.691	0.275	1.38E-07	Blood_CD3	PFDN2
CXCL162	8.69E-12	0.29579	0.473	0.143	1.39E-07	Blood_CD3	CXCL16
RAB348	8.72E-12	0.655751	0.382	0.102	1.39E-07	Blood_CD3	RAB34
LSM62	9.12E-12	0.718144	0.455	0.142	1.45E-07	Blood_CD3	LSM6
ST3GAL53	9.38E-12	0.340714	0.182	0.029	1.5E-07	Blood_CD3	ST3GAL5
NUDT221	9.53E-12	0.566032	0.382	0.107	1.52E-07	Blood_CD3	NUDT22
ECHDC15	9.88E-12	0.552521	0.4	0.117	1.58E-07	Blood_CD3	ECHDC1
RPL320	1.01E-11	0.706461	1	0.843	1.6E-07	Blood_CD3	RPL3
RPN25	1.01E-11	0.580277	0.655	0.246	1.61E-07	Blood_CD3	RPN2
PRPSAP2	1.02E-11	0.522297	0.291	0.066	1.63E-07	Blood_CD3	PRPSAP2
ERAL1	1.1E-11	0.459862	0.218	0.04	1.76E-07	Blood_CD3	ERAL1
ACTG11	1.14E-11	0.428038	1	0.825	1.81E-07	Blood_CD3	ACTG1
MTHFS	1.16E-11	0.33354	0.2	0.034	1.85E-07	Blood_CD3	MTHFS
SMIM124	1.17E-11	0.545008	0.382	0.106	1.87E-07	Blood_CD3	SMIM12
STOML22	1.31E-11	0.718591	0.509	0.177	2.09E-07	Blood_CD3	STOML2
AP1G2	1.37E-11	0.359738	0.236	0.046	2.19E-07	Blood_CD3	AP1G2
CLEC7A2	1.71E-11	0.374923	0.182	0.029	2.72E-07	Blood_CD3	CLEC7A
UQCRFS13	1.76E-11	0.892276	0.636	0.271	2.81E-07	Blood_CD3	UQCRFS1
NPC27	1.92E-11	0.53168	0.909	0.44	3.07E-07	Blood_CD3	NPC2
XRCC6	2E-11	0.841502	0.618	0.24	3.19E-07	Blood_CD3	XRCC6
RNF1351	2.07E-11	0.410651	0.2	0.035	3.31E-07	Blood_CD3	RNF135
S100A109	2.14E-11	0.685396	1	0.676	3.41E-07	Blood_CD3	S100A10
PFDN14	2.14E-11	0.561938	0.4	0.116	3.42E-07	Blood_CD3	PFDN1
C11orf312	2.38E-11	0.633137	0.8	0.354	3.8E-07	Blood_CD3	C11orf31
QARS1	2.39E-11	0.892171	0.491	0.176	3.81E-07	Blood_CD3	QARS
CTNNBL1	2.43E-11	0.508938	0.364	0.097	3.88E-07	Blood_CD3	CTNNBL1
PHACTR14	2.49E-11	0.381934	0.2	0.035	3.97E-07	Blood_CD3	PHACTR1
SSR24	2.57E-11	0.710277	0.873	0.429	4.1E-07	Blood_CD3	SSR2
ATP5G313	2.8E-11	0.708197	0.818	0.362	4.47E-07	Blood_CD3	ATP5G3
EEF1D4	2.82E-11	0.595792	1	0.747	4.49E-07	Blood_CD3	EEF1D
MDH12	2.82E-11	0.826465	0.527	0.19	4.5E-07	Blood_CD3	MDH1
CAPZB	2.92E-11	0.746789	0.891	0.462	4.66E-07	Blood_CD3	CAPZB

AKR1B12	2.97E-11	0.582538	0.4	0.111	4.74E-07	Blood_CD3	AKR1B1
ERGIC33	3.06E-11	0.719795	0.727	0.311	4.88E-07	Blood_CD3	ERGIC3
SEC11A10	3.27E-11	0.579019	0.764	0.315	5.21E-07	Blood_CD3	SEC11A
MRPL111	3.3E-11	0.534151	0.455	0.146	5.27E-07	Blood_CD3	MRPL11
IFI303	3.43E-11	0.301647	0.145	0.02	5.47E-07	Blood_CD3	IFI30
PSMC41	3.44E-11	0.466245	0.491	0.162	5.48E-07	Blood_CD3	PSMC4
OAZ16	3.61E-11	0.685409	1	0.783	5.76E-07	Blood_CD3	OAZ1
HM134	4.26E-11	0.702133	0.545	0.204	6.79E-07	Blood_CD3	HM13
DNPH13	4.27E-11	0.575172	0.455	0.147	6.8E-07	Blood_CD3	DNPH1
TPGS12	4.28E-11	0.643576	0.364	0.1	6.82E-07	Blood_CD3	TPGS1
IRF71	4.54E-11	0.60668	0.364	0.098	7.24E-07	Blood_CD3	IRF7
AKR1A19	4.65E-11	0.768575	0.545	0.204	7.42E-07	Blood_CD3	AKR1A1
RPL109	4.76E-11	0.664615	1	0.878	7.59E-07	Blood_CD3	RPL10
PCBD16	5.02E-11	0.623654	0.473	0.159	8E-07	Blood_CD3	PCBD1
ILK6	5.15E-11	0.594605	0.455	0.148	8.22E-07	Blood_CD3	ILK
CHMP4A2	6.18E-11	0.61257	0.491	0.166	9.86E-07	Blood_CD3	CHMP4A
CLIC26	6.24E-11	0.40137	0.236	0.047	9.95E-07	Blood_CD3	CLIC2
SMDT15	6.34E-11	0.62629	0.818	0.373	1.01E-06	Blood_CD3	SMDT1
COPZ14	6.64E-11	0.608043	0.564	0.217	1.06E-06	Blood_CD3	COPZ1
CBFA2T31	6.71E-11	0.341768	0.145	0.021	1.07E-06	Blood_CD3	CBFA2T3
ELOVL55	7.43E-11	0.50051	0.382	0.108	1.19E-06	Blood_CD3	ELOVL5
PEX161	7.58E-11	0.438563	0.345	0.093	1.21E-06	Blood_CD3	PEX16
SLC25A114	7.98E-11	0.653182	0.382	0.114	1.27E-06	Blood_CD3	SLC25A11
PON24	7.99E-11	0.599203	0.291	0.072	1.27E-06	Blood_CD3	PON2
ATXN101	8.56E-11	0.587932	0.436	0.141	1.37E-06	Blood_CD3	ATXN10
WDR771	9.2E-11	0.48118	0.255	0.057	1.47E-06	Blood_CD3	WDR77
RAB5C4	9.94E-11	0.746025	0.618	0.256	1.59E-06	Blood_CD3	RAB5C
LINC00667	9.98E-11	0.462933	0.291	0.07	1.59E-06	Blood_CD3	LINC00667
IMPDH24	1.01E-10	0.6557	0.473	0.158	1.6E-06	Blood_CD3	IMPDH2
COPS5	1.11E-10	0.592239	0.473	0.161	1.77E-06	Blood_CD3	COPS5
PLEKHJ12	1.11E-10	0.829543	0.473	0.168	1.78E-06	Blood_CD3	PLEKHJ1
DAPP11	1.17E-10	0.53428	0.327	0.085	1.87E-06	Blood_CD3	DAPP1
FAM173A2	1.33E-10	0.462517	0.436	0.138	2.12E-06	Blood_CD3	FAM173A
POLD21	1.35E-10	0.522753	0.4	0.122	2.16E-06	Blood_CD3	POLD2
CORO1B1	1.4E-10	0.424027	0.564	0.206	2.24E-06	Blood_CD3	CORO1B
LINC00936	1.43E-10	0.291915	0.309	0.075	2.29E-06	Blood_CD3	LINC00936
DUSP17	1.48E-10	0.678788	0.964	0.562	2.36E-06	Blood_CD3	DUSP1
RALY	1.49E-10	0.780598	0.618	0.258	2.38E-06	Blood_CD3	RALY
CCDC122	1.5E-10	0.304455	0.145	0.021	2.39E-06	Blood_CD3	CCDC122
RAP1A5	1.54E-10	0.801566	0.636	0.275	2.45E-06	Blood_CD3	RAP1A
RPL13A6	1.54E-10	0.69821	1	0.882	2.46E-06	Blood_CD3	RPL13A
NDUFA91	1.6E-10	0.57979	0.436	0.142	2.56E-06	Blood_CD3	NDUFA9
HMBS	1.83E-10	0.396339	0.182	0.032	2.93E-06	Blood_CD3	HMBS
MSN11	1.85E-10	0.457118	0.491	0.157	2.94E-06	Blood_CD3	MSN
DENND6B2	1.94E-10	0.343254	0.182	0.032	3.1E-06	Blood_CD3	DENND6B
NDUFS34	2.03E-10	0.638307	0.509	0.189	3.24E-06	Blood_CD3	NDUFS3
BAK11	2.13E-10	0.313329	0.2	0.038	3.39E-06	Blood_CD3	BAK1

NDUFC27	2.13E-10	0.689457	0.836	0.386	3.39E-06	Blood_CD3	NDUFC2
LDHA9	2.17E-10	0.648646	0.891	0.44	3.46E-06	Blood_CD3	LDHA
THBD6	2.32E-10	0.352591	0.218	0.043	3.7E-06	Blood_CD3	THBD
HAUS41	2.43E-10	0.44729	0.2	0.038	3.88E-06	Blood_CD3	HAUS4
NAGK3	2.49E-10	0.524619	0.4	0.126	3.97E-06	Blood_CD3	NAGK
TIMM17B1	2.5E-10	0.735144	0.382	0.119	3.99E-06	Blood_CD3	TIMM17B
RFXANK1	2.54E-10	0.591805	0.345	0.098	4.05E-06	Blood_CD3	RFXANK
NUTF23	2.62E-10	0.493677	0.418	0.132	4.18E-06	Blood_CD3	NUTF2
TMEM147	2.69E-10	0.681067	0.6	0.245	4.3E-06	Blood_CD3	TMEM147
RPL78	2.73E-10	0.706185	1	0.797	4.36E-06	Blood_CD3	RPL7
CD683	2.87E-10	0.481615	0.4	0.119	4.58E-06	Blood_CD3	CD68
C6orf485	2.93E-10	0.480861	0.764	0.313	4.67E-06	Blood_CD3	C6orf48
CCDC109B	3E-10	0.379962	0.364	0.099	4.79E-06	Blood_CD3	CCDC109B
DNAJC82	3.15E-10	0.512282	0.582	0.22	5.03E-06	Blood_CD3	DNAJC8
AC010642.	3.15E-10	0.30111	0.2	0.037	5.03E-06	Blood_CD3	AC010642.1
NDUFV15	3.19E-10	0.501925	0.491	0.173	5.09E-06	Blood_CD3	NDUFV1
HSH2D1	3.45E-10	0.441435	0.182	0.032	5.5E-06	Blood_CD3	HSH2D
RPL295	3.54E-10	0.640649	1	0.83	5.65E-06	Blood_CD3	RPL29
PSMC21	3.66E-10	0.549351	0.4	0.127	5.84E-06	Blood_CD3	PSMC2
EMP39	3.85E-10	0.49066	0.855	0.352	6.14E-06	Blood_CD3	EMP3
SRM2	3.87E-10	0.617496	0.473	0.164	6.18E-06	Blood_CD3	SRM
BLOC1S4	3.92E-10	0.522168	0.327	0.088	6.25E-06	Blood_CD3	BLOC1S4
NCBP2.AS2	3.93E-10	0.484886	0.382	0.115	6.26E-06	Blood_CD3	NCBP2.AS2
TBC1D7	4.01E-10	0.433417	0.218	0.046	6.4E-06	Blood_CD3	TBC1D7
VASH11	4.07E-10	0.314914	0.127	0.017	6.49E-06	Blood_CD3	VASH1
CMTM73	4.17E-10	0.298151	0.291	0.07	6.65E-06	Blood_CD3	CMTM7
CACYBP2	4.25E-10	0.722725	0.564	0.23	6.78E-06	Blood_CD3	CACYBP
BTN2A22	4.36E-10	0.296475	0.145	0.022	6.95E-06	Blood_CD3	BTN2A2
PDLIM23	4.41E-10	0.450282	0.509	0.179	7.04E-06	Blood_CD3	PDLIM2
RUSC11	4.67E-10	0.680303	0.255	0.061	7.44E-06	Blood_CD3	RUSC1
RFX5	4.8E-10	0.346144	0.164	0.027	7.65E-06	Blood_CD3	RFX5
DHRS4L22	4.87E-10	0.465947	0.309	0.081	7.77E-06	Blood_CD3	DHRS4L2
TMEM199	4.93E-10	0.44651	0.236	0.053	7.86E-06	Blood_CD3	TMEM199
CTSH7	5.55E-10	0.517371	0.6	0.238	8.85E-06	Blood_CD3	CTSH
INPP5D3	5.75E-10	0.359781	0.255	0.06	9.17E-06	Blood_CD3	INPP5D
NDUFA132	5.76E-10	0.515812	0.818	0.388	9.19E-06	Blood_CD3	NDUFA13
GRSF1	6.13E-10	0.613779	0.382	0.121	9.77E-06	Blood_CD3	GRSF1
RAB7A5	6.56E-10	0.709749	0.782	0.372	1.05E-05	Blood_CD3	RAB7A
CBL1	7.16E-10	0.522814	0.218	0.046	1.14E-05	Blood_CD3	CBL
RFT1	7.18E-10	0.394242	0.164	0.028	1.15E-05	Blood_CD3	RFT1
SNAPIN	7.21E-10	0.472166	0.309	0.083	1.15E-05	Blood_CD3	SNAPIN
AC010226.	7.25E-10	0.272884	0.127	0.018	1.16E-05	Blood_CD3	AC010226.4
ERCC15	7.5E-10	0.685657	0.436	0.155	1.2E-05	Blood_CD3	ERCC1
WDR83OS	7.53E-10	0.588931	0.782	0.368	1.2E-05	Blood_CD3	WDR83OS
CD403	7.71E-10	0.498209	0.291	0.075	1.23E-05	Blood_CD3	CD40
NAGPA	7.72E-10	0.339794	0.145	0.023	1.23E-05	Blood_CD3	NAGPA
TADA3	8.12E-10	0.540319	0.364	0.11	1.29E-05	Blood_CD3	TADA3

LAMTOR23	8.33E-10	0.761067	0.564	0.233	1.33E-05	Blood_CD3	LAMTOR2
CNDP2	8.81E-10	0.609318	0.364	0.112	1.41E-05	Blood_CD3	CNDP2
TMSB107	8.91E-10	0.627108	1	0.879	1.42E-05	Blood_CD3	TMSB10
DHRS79	9.44E-10	0.556403	0.545	0.209	1.51E-05	Blood_CD3	DHRS7
NT5C7	9.5E-10	0.468635	0.4	0.125	1.52E-05	Blood_CD3	NT5C
CCDC1152	9.57E-10	0.504675	0.382	0.117	1.53E-05	Blood_CD3	CCDC115
RAD23A4	1.02E-09	0.558001	0.545	0.206	1.62E-05	Blood_CD3	RAD23A
MED81	1.04E-09	0.856137	0.309	0.088	1.65E-05	Blood_CD3	MED8
KIAA01412	1.09E-09	0.427233	0.2	0.04	1.74E-05	Blood_CD3	KIAA0141
IFI352	1.1E-09	0.522339	0.291	0.075	1.75E-05	Blood_CD3	IFI35
SARNP	1.11E-09	0.492394	0.236	0.054	1.76E-05	Blood_CD3	SARNP
SMARCB11	1.17E-09	0.543241	0.4	0.129	1.87E-05	Blood_CD3	SMARCB1
SNRPD15	1.19E-09	0.652706	0.564	0.222	1.89E-05	Blood_CD3	SNRPD1
CPNE14	1.22E-09	0.566724	0.4	0.128	1.95E-05	Blood_CD3	CPNE1
MRPS343	1.23E-09	0.679972	0.509	0.197	1.96E-05	Blood_CD3	MRPS34
C12orf452	1.24E-09	0.644856	0.291	0.078	1.99E-05	Blood_CD3	C12orf45
RPL18A7	1.36E-09	0.59531	1	0.823	2.16E-05	Blood_CD3	RPL18A
MRPL18	1.37E-09	0.536852	0.582	0.237	2.19E-05	Blood_CD3	MRPL18
RPS103	1.52E-09	0.571766	1	0.608	2.43E-05	Blood_CD3	RPS10
SERBP16	1.52E-09	0.584021	0.764	0.355	2.43E-05	Blood_CD3	SERBP1
C1orf434	1.57E-09	0.701735	0.545	0.228	2.51E-05	Blood_CD3	C1orf43
PPP4C1	1.63E-09	0.727278	0.636	0.286	2.6E-05	Blood_CD3	PPP4C
TOMM223	1.64E-09	0.513443	0.545	0.213	2.61E-05	Blood_CD3	TOMM22
NHP24	1.69E-09	0.538401	0.582	0.231	2.69E-05	Blood_CD3	NHP2
IMP44	1.7E-09	0.638079	0.4	0.135	2.72E-05	Blood_CD3	IMP4
RBCK12	1.77E-09	0.527388	0.436	0.152	2.82E-05	Blood_CD3	RBCK1
NEDD88	1.84E-09	0.648451	0.836	0.423	2.93E-05	Blood_CD3	NEDD8
PSMA54	1.84E-09	0.510763	0.491	0.183	2.93E-05	Blood_CD3	PSMA5
BANK1	1.87E-09	0.262446	0.127	0.018	2.98E-05	Blood_CD3	BANK1
CKLF6	2.01E-09	0.739464	0.436	0.151	3.21E-05	Blood_CD3	CKLF
MOB3A3	2.13E-09	0.446944	0.218	0.047	3.4E-05	Blood_CD3	MOB3A
PSMD41	2.48E-09	0.463214	0.582	0.233	3.96E-05	Blood_CD3	PSMD4
EIF2A5	2.51E-09	0.547999	0.436	0.154	4.01E-05	Blood_CD3	EIF2A
SCAND11	2.54E-09	0.730367	0.709	0.344	4.05E-05	Blood_CD3	SCAND1
ZNHIT13	2.62E-09	0.555968	0.655	0.296	4.18E-05	Blood_CD3	ZNHIT1
ILF22	2.64E-09	0.59802	0.527	0.206	4.21E-05	Blood_CD3	ILF2
EIF3I3	2.69E-09	0.613401	0.582	0.244	4.29E-05	Blood_CD3	EIF3I
ECHS13	2.69E-09	0.685093	0.509	0.204	4.29E-05	Blood_CD3	ECHS1
NOA1	2.72E-09	0.372838	0.236	0.055	4.33E-05	Blood_CD3	NOA1
DCXR6	3.17E-09	0.710003	0.545	0.22	5.06E-05	Blood_CD3	DCXR
SYTL12	3.18E-09	0.423014	0.364	0.112	5.07E-05	Blood_CD3	SYTL1
PGAM17	3.4E-09	0.5493	0.564	0.229	5.42E-05	Blood_CD3	PGAM1
DDX56	3.52E-09	0.598872	0.927	0.602	5.61E-05	Blood_CD3	DDX5
RPS118	3.74E-09	0.570912	1	0.778	5.96E-05	Blood_CD3	RPS11
LTA4H1	3.82E-09	0.485033	0.345	0.105	6.09E-05	Blood_CD3	LTA4H
BZW21	3.86E-09	0.536279	0.273	0.072	6.15E-05	Blood_CD3	BZW2
PGK1	3.98E-09	0.535477	0.764	0.354	6.35E-05	Blood_CD3	PGK1

TSPAN153	4.03E-09	0.372943	0.2	0.043	6.43E-05	Blood_CD3	TSPAN15
MLX	4.05E-09	0.462215	0.327	0.098	6.46E-05	Blood_CD3	MLX
ARL5A6	4.23E-09	0.678146	0.491	0.196	6.74E-05	Blood_CD3	ARL5A
ACTR34	4.27E-09	0.765252	0.727	0.356	6.81E-05	Blood_CD3	ACTR3
KIAA09304	4.44E-09	0.362621	0.273	0.07	7.09E-05	Blood_CD3	KIAA0930
RPS88	4.48E-09	0.612989	1	0.85	7.14E-05	Blood_CD3	RPS8
SIPA12	4.58E-09	0.51445	0.309	0.089	7.3E-05	Blood_CD3	SIPA1
WDR612	4.58E-09	0.634283	0.382	0.129	7.31E-05	Blood_CD3	WDR61
RPH3AL	4.86E-09	0.255869	0.091	0.011	7.75E-05	Blood_CD3	RPH3AL
BLOC1S21	4.92E-09	0.387875	0.364	0.114	7.84E-05	Blood_CD3	BLOC1S2
TSPO7	5.32E-09	0.589432	0.855	0.466	8.48E-05	Blood_CD3	TSPO
TAX1BP36	5.65E-09	0.50496	0.309	0.087	9.01E-05	Blood_CD3	TAX1BP3
UROS3	6.23E-09	0.512854	0.327	0.1	9.94E-05	Blood_CD3	UROS
UCHL31	6.44E-09	0.530103	0.364	0.121	0.000103	Blood_CD3	UCHL3
RIN31	6.52E-09	0.445773	0.291	0.078	0.000104	Blood_CD3	RIN3
DRAP15	6.52E-09	0.518126	0.745	0.335	0.000104	Blood_CD3	DRAP1
KLRG11	6.74E-09	0.253753	0.145	0.024	0.000107	Blood_CD3	KLRG1
RAN10	6.76E-09	0.603982	0.764	0.367	0.000108	Blood_CD3	RAN
SRSF310	6.78E-09	0.62571	0.818	0.434	0.000108	Blood_CD3	SRSF3
CCT6A2	7.06E-09	0.607239	0.473	0.179	0.000113	Blood_CD3	CCT6A
TOMM401	7.24E-09	0.502598	0.309	0.09	0.000115	Blood_CD3	TOMM40
ARHGDIA8	7.33E-09	0.588328	0.509	0.197	0.000117	Blood_CD3	ARHGDIA
ADCY73	7.4E-09	0.338208	0.127	0.019	0.000118	Blood_CD3	ADCY7
CAPG6	7.43E-09	0.694608	0.455	0.167	0.000118	Blood_CD3	CAPG
ARF3	7.43E-09	0.495453	0.4	0.14	0.000118	Blood_CD3	ARF3
ZNF580	7.46E-09	0.533949	0.309	0.091	0.000119	Blood_CD3	ZNF580
SERPINA11	8.1E-09	0.303167	0.255	0.063	0.000129	Blood_CD3	SERPINA1
RPL128	8.3E-09	0.606402	1	0.853	0.000132	Blood_CD3	RPL12
STXBP21	8.37E-09	0.509785	0.418	0.152	0.000133	Blood_CD3	STXBP2
MFSD13	8.81E-09	0.577002	0.327	0.102	0.000141	Blood_CD3	MFSD1
MAGEF1	8.86E-09	0.459596	0.273	0.074	0.000141	Blood_CD3	MAGEF1
SH3D21	8.91E-09	0.275993	0.109	0.015	0.000142	Blood_CD3	SH3D21
SNHG74	9.22E-09	0.271656	0.582	0.223	0.000147	Blood_CD3	SNHG7
CHCHD3	9.55E-09	0.479319	0.309	0.092	0.000152	Blood_CD3	CHCHD3
CCT5	9.62E-09	0.647148	0.455	0.172	0.000153	Blood_CD3	CCT5
EID19	9.66E-09	0.567795	0.745	0.358	0.000154	Blood_CD3	EID1
HMGN310	1.04E-08	0.628408	0.618	0.279	0.000165	Blood_CD3	HMGN3
MSRA2	1.06E-08	0.576133	0.291	0.084	0.000169	Blood_CD3	MSRA
MAF12	1.08E-08	0.655969	0.491	0.195	0.000173	Blood_CD3	MAF1
PTDSS1	1.09E-08	0.508257	0.255	0.066	0.000173	Blood_CD3	PTDSS1
NUBPL	1.13E-08	0.35469	0.145	0.025	0.000181	Blood_CD3	NUBPL
MRPL243	1.35E-08	0.607627	0.327	0.102	0.000215	Blood_CD3	MRPL24
RGS35	1.38E-08	0.277147	0.2	0.043	0.000221	Blood_CD3	RGS3
STAT62	1.4E-08	0.37235	0.273	0.073	0.000224	Blood_CD3	STAT6
ARL26	1.49E-08	0.364957	0.545	0.211	0.000238	Blood_CD3	ARL2
EIF3G1	1.5E-08	0.574296	0.782	0.4	0.000239	Blood_CD3	EIF3G
TALDO11	1.55E-08	0.511027	0.818	0.417	0.000247	Blood_CD3	TALDO1

GDPD5	1.58E-08	0.290106	0.164	0.031	0.000252	Blood_CD3	GDPD5
ATP5O5	1.59E-08	0.661366	0.8	0.431	0.000253	Blood_CD3	ATP5O
NDUFB28	1.61E-08	0.408912	0.818	0.375	0.000257	Blood_CD3	NDUFB2
CTNNBIP11	1.66E-08	0.622343	0.436	0.171	0.000265	Blood_CD3	CTNNBIP1
DUT5	1.73E-08	0.526631	0.564	0.236	0.000275	Blood_CD3	DUT
COPS7A	1.75E-08	0.660382	0.255	0.07	0.000279	Blood_CD3	COPS7A
MRPS25	1.76E-08	0.449724	0.364	0.119	0.00028	Blood_CD3	MRPS25
TMEM160	1.79E-08	0.464313	0.509	0.205	0.000285	Blood_CD3	TMEM160
ACSS21	1.87E-08	0.451286	0.182	0.039	0.000298	Blood_CD3	ACSS2
TAF6	1.98E-08	0.308948	0.218	0.052	0.000315	Blood_CD3	TAF6
SH2D3C3	1.99E-08	0.301092	0.109	0.015	0.000318	Blood_CD3	SH2D3C
PDIA36	2.08E-08	0.55556	0.691	0.323	0.000331	Blood_CD3	PDIA3
CCNDBP1	2.09E-08	0.480755	0.527	0.213	0.000333	Blood_CD3	CCNDBP1
GLRX31	2.15E-08	0.572326	0.418	0.152	0.000343	Blood_CD3	GLRX3
HNRNPA2E	2.16E-08	0.660444	0.873	0.492	0.000345	Blood_CD3	HNRNPA2B1
PSMB72	2.17E-08	0.593385	0.655	0.305	0.000346	Blood_CD3	PSMB7
ANKRD651	2.19E-08	0.465494	0.164	0.032	0.000349	Blood_CD3	ANKRD65
RTFDC11	2.33E-08	0.519838	0.491	0.198	0.000371	Blood_CD3	RTFDC1
C17orf624	2.35E-08	0.404869	0.309	0.091	0.000374	Blood_CD3	C17orf62
PLB1	2.35E-08	0.279268	0.091	0.011	0.000375	Blood_CD3	PLB1
LYL13	2.39E-08	0.3532	0.273	0.072	0.000382	Blood_CD3	LYL1
SP110	2.49E-08	0.290428	0.255	0.065	0.000398	Blood_CD3	SP110
GHITM	2.51E-08	0.453184	0.691	0.334	0.0004	Blood_CD3	GHITM
MDP1	2.66E-08	0.302304	0.145	0.026	0.000425	Blood_CD3	MDP1
BRMS11	2.74E-08	0.516612	0.327	0.103	0.000438	Blood_CD3	BRMS1
NDUFAB11	2.96E-08	0.499453	0.564	0.246	0.000473	Blood_CD3	NDUFAB1
SMCO45	3.01E-08	0.581787	0.345	0.119	0.00048	Blood_CD3	SMCO4
CDKN2D1	3.02E-08	0.545926	0.291	0.083	0.000482	Blood_CD3	CDKN2D
PUF603	3.04E-08	0.612329	0.418	0.157	0.000484	Blood_CD3	PUF60
EVI2A5	3.04E-08	0.39863	0.182	0.038	0.000486	Blood_CD3	EVI2A
GAA1	3.12E-08	0.530913	0.273	0.079	0.000498	Blood_CD3	GAA
RFTN12	3.22E-08	0.25539	0.182	0.038	0.000514	Blood_CD3	RFTN1
RPL7A11	3.23E-08	0.549569	1	0.814	0.000515	Blood_CD3	RPL7A
SRSF95	3.29E-08	0.444166	0.745	0.354	0.000524	Blood_CD3	SRSF9
AHCY	3.48E-08	0.441992	0.345	0.115	0.000555	Blood_CD3	AHCY
CCT2	3.55E-08	0.52491	0.473	0.182	0.000566	Blood_CD3	CCT2
RPL96	3.65E-08	0.497023	1	0.75	0.000582	Blood_CD3	RPL9
SIGMAR1	3.84E-08	0.530565	0.2	0.046	0.000612	Blood_CD3	SIGMAR1
PHB7	4.05E-08	0.401061	0.6	0.241	0.000646	Blood_CD3	PHB
SNRPC1	4.25E-08	0.554682	0.473	0.19	0.000677	Blood_CD3	SNRPC
RPL185	4.46E-08	0.446448	1	0.878	0.000712	Blood_CD3	RPL18
ADSL	4.72E-08	0.482677	0.273	0.078	0.000753	Blood_CD3	ADSL
CLCN4	4.86E-08	0.51657	0.073	0.008	0.000775	Blood_CD3	CLCN4
RPL115	5.14E-08	0.475781	1	0.854	0.00082	Blood_CD3	RPL11
SF3B53	5.3E-08	0.586546	0.709	0.363	0.000846	Blood_CD3	SF3B5
LRCH41	5.45E-08	0.326511	0.327	0.103	0.000869	Blood_CD3	LRCH4
NXF11	5.64E-08	0.417504	0.273	0.078	0.0009	Blood_CD3	NXF1

CPQ4	5.67E-08	0.373422	0.273	0.075	0.000904	Blood_CD3	CPQ
PEF12	5.81E-08	0.432922	0.309	0.096	0.000927	Blood_CD3	PEF1
EPSTI12	5.9E-08	0.382005	0.2	0.047	0.000941	Blood_CD3	EPSTI1
RNF1261	6.13E-08	0.424468	0.4	0.142	0.000977	Blood_CD3	RNF126
SUCLG13	6.16E-08	0.645976	0.436	0.177	0.000982	Blood_CD3	SUCLG1
NUBP21	6.18E-08	0.427011	0.345	0.117	0.000986	Blood_CD3	NUBP2
OSBPL91	6.49E-08	0.587213	0.309	0.099	0.001035	Blood_CD3	OSBPL9
DCK3	6.51E-08	0.381541	0.236	0.06	0.001038	Blood_CD3	DCK
NME310	6.52E-08	0.443283	0.509	0.21	0.00104	Blood_CD3	NME3
PRMT23	6.88E-08	0.573166	0.418	0.158	0.001098	Blood_CD3	PRMT2
SEPT91	7.16E-08	0.473927	0.436	0.164	0.001142	Blood_CD3	SEPT9
SNHG156	7.49E-08	0.390598	0.345	0.111	0.001195	Blood_CD3	SNHG15
PSMA14	7.6E-08	0.721691	0.545	0.254	0.001212	Blood_CD3	PSMA1
SLC18B13	7.95E-08	0.268471	0.145	0.028	0.001268	Blood_CD3	SLC18B1
BANF15	8.14E-08	0.3192	0.564	0.233	0.001298	Blood_CD3	BANF1
ASPSCR1	8.22E-08	0.4379	0.255	0.072	0.00131	Blood_CD3	ASPSCR1
RPL23A5	8.23E-08	0.503538	1	0.804	0.001313	Blood_CD3	RPL23A
IAH14	8.3E-08	0.619886	0.418	0.161	0.001324	Blood_CD3	IAH1
PMF13	8.62E-08	0.470726	0.364	0.128	0.001375	Blood_CD3	PMF1
ZNF321	8.69E-08	0.431225	0.255	0.072	0.001386	Blood_CD3	ZNF32
PSMD31	9.53E-08	0.611457	0.345	0.122	0.00152	Blood_CD3	PSMD3
TRMT13	9.54E-08	0.301102	0.218	0.055	0.001521	Blood_CD3	TRMT1
ENTPD18	9.64E-08	0.333144	0.2	0.047	0.001537	Blood_CD3	ENTPD1
PES11	9.7E-08	0.264783	0.2	0.048	0.001547	Blood_CD3	PES1
SIRT6	9.78E-08	0.328732	0.164	0.034	0.00156	Blood_CD3	SIRT6
RPL133	9.93E-08	0.326371	1	0.918	0.001583	Blood_CD3	RPL13
ZNF480	1E-07	0.260462	0.182	0.04	0.001595	Blood_CD3	ZNF480
AATF	1.05E-07	0.450586	0.273	0.081	0.001672	Blood_CD3	AATF
EMC104	1.1E-07	0.483745	0.4	0.153	0.001751	Blood_CD3	EMC10
SAT25	1.13E-07	0.413371	0.491	0.205	0.001809	Blood_CD3	SAT2
SHISA52	1.17E-07	0.357821	0.327	0.104	0.001861	Blood_CD3	SHISA5
CCT31	1.19E-07	0.464231	0.527	0.222	0.001892	Blood_CD3	CCT3
METTL94	1.19E-07	0.518798	0.509	0.222	0.001904	Blood_CD3	METTL9
ZNF4283	1.2E-07	0.412224	0.436	0.167	0.001917	Blood_CD3	ZNF428
SH3BP11	1.21E-07	0.432409	0.218	0.055	0.001923	Blood_CD3	SH3BP1
TTC1	1.22E-07	0.688529	0.382	0.142	0.00194	Blood_CD3	TTC1
IKZF15	1.24E-07	0.302787	0.182	0.04	0.001984	Blood_CD3	IKZF1
ADRM1	1.29E-07	0.431227	0.582	0.263	0.002058	Blood_CD3	ADRM1
UBE2D21	1.29E-07	0.469985	0.691	0.345	0.002063	Blood_CD3	UBE2D2
PTRHD13	1.4E-07	0.965904	0.436	0.192	0.002233	Blood_CD3	PTRHD1
TSC22D4	1.42E-07	0.339341	0.491	0.199	0.002263	Blood_CD3	TSC22D4
RPL27A8	1.47E-07	0.482248	1	0.836	0.002351	Blood_CD3	RPL27A
SF3A2	1.56E-07	0.335202	0.236	0.063	0.002483	Blood_CD3	SF3A2
LMAN23	1.56E-07	0.531645	0.564	0.256	0.002493	Blood_CD3	LMAN2
GSS	1.6E-07	0.498091	0.255	0.075	0.002557	Blood_CD3	GSS
MRPL48	1.63E-07	0.339361	0.255	0.073	0.002605	Blood_CD3	MRPL48
COX8A5	1.64E-07	0.490662	0.836	0.436	0.002619	Blood_CD3	COX8A

IDH3G1	1.67E-07	0.434771	0.436	0.173	0.002668	Blood_CD3	IDH3G
TMEM106A	1.71E-07	0.276923	0.127	0.023	0.002735	Blood_CD3	TMEM106A
CDC1231	1.76E-07	0.686848	0.309	0.103	0.00281	Blood_CD3	CDC123
TFEB2	1.8E-07	0.366391	0.145	0.029	0.002875	Blood_CD3	TFEB
RNPS15	1.82E-07	0.393383	0.491	0.199	0.002902	Blood_CD3	RNPS1
GADD45GI	1.88E-07	0.476577	0.6	0.28	0.003	Blood_CD3	GADD45GIP1
SURF12	1.88E-07	0.49569	0.436	0.177	0.003	Blood_CD3	SURF1
ACADVL3	1.91E-07	0.539457	0.509	0.232	0.003053	Blood_CD3	ACADVL
PRPF31	1.94E-07	0.445944	0.327	0.11	0.003096	Blood_CD3	PRPF31
DDT4	1.98E-07	0.448064	0.691	0.341	0.003166	Blood_CD3	DDT
TMEM97	1.99E-07	0.37022	0.164	0.036	0.00318	Blood_CD3	TMEM97
PTP4A27	2.12E-07	0.449026	0.618	0.292	0.003387	Blood_CD3	PTP4A2
CUEDC21	2.15E-07	0.517848	0.345	0.122	0.003436	Blood_CD3	CUEDC2
RAB8A3	2.32E-07	0.59487	0.364	0.137	0.003699	Blood_CD3	RAB8A
PSEN21	2.49E-07	0.456921	0.127	0.024	0.003968	Blood_CD3	PSEN2
TNFRSF141	2.51E-07	0.29699	0.345	0.117	0.004004	Blood_CD3	TNFRSF14
RPS2311	2.53E-07	0.49614	1	0.814	0.004028	Blood_CD3	RPS23
LSM74	2.53E-07	0.438982	0.618	0.286	0.004032	Blood_CD3	LSM7
WDR181	2.53E-07	0.392766	0.236	0.066	0.00404	Blood_CD3	WDR18
WDR541	2.54E-07	0.313426	0.236	0.064	0.00405	Blood_CD3	WDR54
KTI12	2.57E-07	0.264979	0.127	0.023	0.004091	Blood_CD3	KTI12
C18orf32	2.64E-07	0.355153	0.218	0.057	0.004209	Blood_CD3	C18orf32
UBE2I4	2.72E-07	0.482575	0.564	0.264	0.004335	Blood_CD3	UBE2I
C15orf571	2.76E-07	0.486273	0.236	0.067	0.004398	Blood_CD3	C15orf57
GNAI28	2.83E-07	0.430018	0.527	0.226	0.00451	Blood_CD3	GNAI2
RPS97	2.87E-07	0.465293	1	0.837	0.00457	Blood_CD3	RPS9
SHMT23	2.96E-07	0.419603	0.236	0.067	0.004716	Blood_CD3	SHMT2
RANBP18	3.14E-07	0.639364	0.564	0.267	0.005013	Blood_CD3	RANBP1
H3F3B1	3.23E-07	0.354158	1	0.862	0.005153	Blood_CD3	H3F3B
RELB4	3.46E-07	0.355828	0.236	0.066	0.00552	Blood_CD3	RELB
UQCRH2	3.51E-07	0.498087	0.855	0.479	0.005595	Blood_CD3	UQCRH
DPP77	3.8E-07	0.253234	0.6	0.259	0.006055	Blood_CD3	DPP7
MKL11	3.86E-07	0.294479	0.164	0.036	0.006155	Blood_CD3	MKL1
PANK2	3.94E-07	0.458283	0.273	0.086	0.006287	Blood_CD3	PANK2
COX6A15	4.37E-07	0.502758	0.909	0.531	0.006976	Blood_CD3	COX6A1
KXD11	4.51E-07	0.619633	0.345	0.129	0.007196	Blood_CD3	KXD1
BCAT21	4.52E-07	0.441063	0.182	0.044	0.007205	Blood_CD3	BCAT2
ABTB1	4.53E-07	0.386902	0.236	0.066	0.007223	Blood_CD3	ABTB1
PARVB5	4.56E-07	0.391761	0.182	0.043	0.007271	Blood_CD3	PARVB
OLA1	4.69E-07	0.407638	0.364	0.135	0.007488	Blood_CD3	OLA1
PPCS1	4.78E-07	0.447475	0.455	0.196	0.00763	Blood_CD3	PPCS
ARAF1	4.82E-07	0.357795	0.218	0.06	0.007682	Blood_CD3	ARAF
CPSF3	4.84E-07	0.513683	0.2	0.052	0.007726	Blood_CD3	CPSF3
DAXX1	5.25E-07	0.465249	0.255	0.078	0.00838	Blood_CD3	DAXX
PRKAR1A6	5.35E-07	0.509362	0.527	0.239	0.008539	Blood_CD3	PRKAR1A
SPINT28	5.79E-07	0.431145	0.673	0.314	0.009237	Blood_CD3	SPINT2
ANP32B1	5.79E-07	0.474541	0.636	0.305	0.009239	Blood_CD3	ANP32B

NDUFV22	6.17E-07	0.474153	0.727	0.386	0.009848	Blood_CD3	NDUFV2
RPL3111	6.38E-07	0.360324	1	0.768	0.010173	Blood_CD3	RPL31
NDUFAF31	6.48E-07	0.563443	0.545	0.261	0.010328	Blood_CD3	NDUFAF3
RABGGTA	6.78E-07	0.404591	0.182	0.045	0.010806	Blood_CD3	RABGGTA
FAM96B4	6.93E-07	0.387053	0.564	0.259	0.011056	Blood_CD3	FAM96B
HPS31	7.23E-07	0.297391	0.145	0.031	0.011534	Blood_CD3	HPS3
ATP6V0D1	7.23E-07	0.568118	0.455	0.204	0.011537	Blood_CD3	ATP6V0D1
RBMX6	7.46E-07	0.410411	0.473	0.199	0.011894	Blood_CD3	RBMX
PIN12	7.51E-07	0.479562	0.473	0.205	0.011975	Blood_CD3	PIN1
CDC377	7.9E-07	0.423027	0.582	0.275	0.012605	Blood_CD3	CDC37
TMEM134:	7.91E-07	0.53662	0.418	0.175	0.012617	Blood_CD3	TMEM134
SSBP46	7.96E-07	0.26241	0.309	0.103	0.01269	Blood_CD3	SSBP4
HADHA4	8.05E-07	0.545516	0.455	0.201	0.012837	Blood_CD3	HADHA
MRPS111	8.49E-07	0.456799	0.309	0.11	0.013534	Blood_CD3	MRPS11
ZNF5933	8.93E-07	0.432939	0.345	0.129	0.014235	Blood_CD3	ZNF593
GALT	9.6E-07	0.285836	0.182	0.045	0.015319	Blood_CD3	GALT
MRPS231	9.76E-07	0.34296	0.291	0.098	0.015574	Blood_CD3	MRPS23
NT5DC13	9.78E-07	0.365595	0.255	0.08	0.015597	Blood_CD3	NT5DC1
PDCD21	1E-06	0.481908	0.364	0.141	0.01597	Blood_CD3	PDCD2
STARD32	1.01E-06	0.380096	0.218	0.062	0.016135	Blood_CD3	STARD3
TMEM101:	1.02E-06	0.385104	0.255	0.081	0.016258	Blood_CD3	TMEM101
ADI19	1.03E-06	0.434281	0.491	0.222	0.016399	Blood_CD3	ADI1
GTPBP62	1.05E-06	0.28719	0.255	0.078	0.016727	Blood_CD3	GTPBP6
RRP91	1.09E-06	0.268273	0.164	0.038	0.017391	Blood_CD3	RRP9
PRDX38	1.09E-06	0.580239	0.382	0.157	0.017407	Blood_CD3	PRDX3
UBE2E25	1.09E-06	0.442363	0.255	0.08	0.017436	Blood_CD3	UBE2E2
PHYKPL3	1.09E-06	0.34827	0.236	0.07	0.017461	Blood_CD3	PHYKPL
CCDC861	1.19E-06	0.301435	0.145	0.032	0.01899	Blood_CD3	CCDC86
FYN9	1.2E-06	0.312348	0.236	0.067	0.019213	Blood_CD3	FYN
FKBP3	1.21E-06	0.566216	0.4	0.168	0.019355	Blood_CD3	FKBP3
PSMD85	1.25E-06	0.306809	0.673	0.332	0.02	Blood_CD3	PSMD8
RPS165	1.33E-06	0.393286	1	0.836	0.021147	Blood_CD3	RPS16
SCO23	1.38E-06	0.468276	0.273	0.091	0.022077	Blood_CD3	SCO2
TMEM203:	1.4E-06	0.302864	0.255	0.08	0.022337	Blood_CD3	TMEM203
PTPMT11	1.41E-06	0.357814	0.327	0.118	0.022413	Blood_CD3	PTPMT1
C4orf484	1.41E-06	0.256958	0.382	0.144	0.022452	Blood_CD3	C4orf48
CCS4	1.42E-06	0.439758	0.309	0.111	0.022598	Blood_CD3	CCS
DCTN35	1.44E-06	0.562968	0.491	0.235	0.022941	Blood_CD3	DCTN3
MRPL433	1.51E-06	0.480274	0.436	0.192	0.024145	Blood_CD3	MRPL43
PTMA9	1.52E-06	0.426024	1	0.819	0.024212	Blood_CD3	PTMA
COASY	1.57E-06	0.369365	0.255	0.082	0.02501	Blood_CD3	COASY
POLDIP3	1.63E-06	0.364217	0.291	0.1	0.025969	Blood_CD3	POLDIP3
CPPED11	1.69E-06	0.329123	0.218	0.064	0.026996	Blood_CD3	CPPED1
NME6	1.7E-06	0.295447	0.145	0.032	0.027123	Blood_CD3	NME6
RPUSD3	1.72E-06	0.351478	0.218	0.063	0.027388	Blood_CD3	RPUSD3
THEM4	1.75E-06	0.284945	0.2	0.054	0.027831	Blood_CD3	THEM4
MRPL342	1.8E-06	0.462877	0.418	0.179	0.028699	Blood_CD3	MRPL34

BCAP313	1.81E-06	0.377598	0.6	0.289	0.028869	Blood_CD3	BCAP31
JTB7	1.84E-06	0.381545	0.745	0.371	0.029285	Blood_CD3	JTB
MSRB11	1.93E-06	0.456412	0.2	0.056	0.030784	Blood_CD3	MSRB1
NDUFB54	2.02E-06	0.623261	0.509	0.245	0.03225	Blood_CD3	NDUFB5
BABAM11	2.02E-06	0.375809	0.364	0.145	0.032255	Blood_CD3	BABAM1
AP1S11	2.14E-06	0.499751	0.273	0.095	0.034173	Blood_CD3	AP1S1
EDF14	2.26E-06	0.444085	0.964	0.625	0.035993	Blood_CD3	EDF1
PRDX29	2.34E-06	0.356608	0.655	0.312	0.037332	Blood_CD3	PRDX2
ATP5G110	2.34E-06	0.339178	0.564	0.261	0.037397	Blood_CD3	ATP5G1
PSMC5	2.35E-06	0.396977	0.545	0.26	0.037474	Blood_CD3	PSMC5
GSDMD2	2.6E-06	0.369577	0.291	0.1	0.041494	Blood_CD3	GSDMD
TRIAP1	2.66E-06	0.358666	0.273	0.093	0.042455	Blood_CD3	TRIAP1
STK16	2.73E-06	0.326792	0.236	0.075	0.043595	Blood_CD3	STK16
BLOC1S13	2.76E-06	0.408869	0.655	0.342	0.044041	Blood_CD3	BLOC1S1
CHCHD25	2.8E-06	0.468278	0.909	0.621	0.044585	Blood_CD3	CHCHD2
ARL4C6	2.81E-06	0.341005	0.455	0.189	0.044769	Blood_CD3	ARL4C
NUPL2	2.82E-06	0.3713	0.218	0.064	0.044916	Blood_CD3	NUPL2
ARPC55	2.85E-06	0.482937	0.764	0.413	0.045424	Blood_CD3	ARPC5
CISD31	2.9E-06	0.376497	0.291	0.103	0.046309	Blood_CD3	CISD3
OS98	2.93E-06	0.515376	0.473	0.226	0.046796	Blood_CD3	OS9
UBE2L3	2.94E-06	0.570629	0.6	0.315	0.046845	Blood_CD3	UBE2L3
TCIRG13	2.97E-06	0.348945	0.236	0.074	0.047337	Blood_CD3	TCIRG1
PLD37	3E-06	0.35915	0.4	0.165	0.047843	Blood_CD3	PLD3
AIFM1	3.02E-06	0.295592	0.164	0.041	0.048205	Blood_CD3	AIFM1
SUMO33	3.13E-06	0.492782	0.473	0.221	0.049973	Blood_CD3	SUMO3
SH3KBP14	3.22E-06	0.279451	0.273	0.088	0.051387	Blood_CD3	SH3KBP1
SSNA12	3.28E-06	0.375218	0.491	0.231	0.052392	Blood_CD3	SSNA1
GTF3C63	3.48E-06	0.728996	0.436	0.202	0.055571	Blood_CD3	GTF3C6
HSD17B11	3.51E-06	0.412384	0.364	0.138	0.055957	Blood_CD3	HSD17B11
NOC2L2	3.57E-06	0.47279	0.255	0.085	0.057013	Blood_CD3	NOC2L
EBPL5	3.64E-06	0.394838	0.345	0.135	0.058032	Blood_CD3	EBPL
CTD.3184A	3.65E-06	0.279766	0.145	0.033	0.058198	Blood_CD3	CTD.3184A7.4
LTV11	3.84E-06	0.334961	0.2	0.057	0.061286	Blood_CD3	LTV1
EIF5B3	3.85E-06	0.367815	0.564	0.278	0.061449	Blood_CD3	EIF5B
MFF	3.98E-06	0.416459	0.436	0.195	0.063489	Blood_CD3	MFF
CCDC1243	4E-06	0.391143	0.382	0.159	0.063825	Blood_CD3	CCDC124
PYCR2	4.02E-06	0.262143	0.236	0.074	0.064077	Blood_CD3	PYCR2
MRTO42	4.04E-06	0.369803	0.218	0.067	0.064453	Blood_CD3	MRTO4
DARS1	4.06E-06	0.393507	0.364	0.147	0.064721	Blood_CD3	DARS
ATP13A11	4.09E-06	0.31485	0.164	0.042	0.065299	Blood_CD3	ATP13A1
CECR51	4.53E-06	0.378408	0.182	0.05	0.072216	Blood_CD3	CECR5
PRKAG1	4.53E-06	0.493511	0.255	0.087	0.072229	Blood_CD3	PRKAG1
DEGS15	4.64E-06	0.457312	0.327	0.127	0.073991	Blood_CD3	DEGS1
SYF22	4.69E-06	0.488583	0.618	0.327	0.074816	Blood_CD3	SYF2
ATP1A112	4.7E-06	0.368676	0.545	0.256	0.074953	Blood_CD3	ATP1A1
EIF4E22	4.78E-06	0.530217	0.382	0.168	0.076172	Blood_CD3	EIF4E2
CCT7	4.78E-06	0.394488	0.455	0.201	0.076228	Blood_CD3	CCT7

GMPR21	5.11E-06	0.348002	0.255	0.086	0.081462	Blood_CD3	GMPR2
TSSC41	5.38E-06	0.387022	0.327	0.127	0.085818	Blood_CD3	TSSC4
OSTF11	5.45E-06	0.412026	0.509	0.242	0.086933	Blood_CD3	OSTF1
VAMP22	5.49E-06	0.344449	0.655	0.338	0.087595	Blood_CD3	VAMP2
STX32	5.73E-06	0.486667	0.236	0.078	0.09133	Blood_CD3	STX3
SNRNP704	5.73E-06	0.416165	0.418	0.184	0.09136	Blood_CD3	SNRNP70
MOAP11	5.82E-06	0.26618	0.236	0.076	0.09282	Blood_CD3	MOAP1
CLPP1	5.86E-06	0.379156	0.364	0.151	0.093395	Blood_CD3	CLPP
VPS294	5.9E-06	0.473305	0.545	0.282	0.094127	Blood_CD3	VPS29
TMEM261	5.96E-06	0.499598	0.345	0.14	0.095053	Blood_CD3	TMEM261
VDAC32	5.98E-06	0.461486	0.418	0.187	0.095361	Blood_CD3	VDAC3
MRPS123	6.33E-06	0.376245	0.345	0.14	0.100998	Blood_CD3	MRPS12
BRAT1	6.38E-06	0.331696	0.164	0.043	0.101703	Blood_CD3	BRAT1
MRPS15	6.54E-06	0.326161	0.455	0.21	0.104321	Blood_CD3	MRPS15
ABCB7	6.62E-06	0.285446	0.145	0.035	0.105537	Blood_CD3	ABCB7
ELP51	6.76E-06	0.315811	0.291	0.103	0.107755	Blood_CD3	ELP5
DAP31	7.19E-06	0.404438	0.309	0.119	0.114689	Blood_CD3	DAP3
NANS6	7.4E-06	0.496264	0.473	0.231	0.117991	Blood_CD3	NANS
STUB12	7.4E-06	0.378633	0.509	0.253	0.118072	Blood_CD3	STUB1
NDUFB101	7.48E-06	0.358195	0.727	0.385	0.119251	Blood_CD3	NDUFB10
XPA2	7.68E-06	0.375647	0.255	0.088	0.12247	Blood_CD3	XPA
ACOT95	8.1E-06	0.528584	0.2	0.061	0.129211	Blood_CD3	ACOT9
POMC	8.12E-06	0.305621	0.2	0.058	0.129557	Blood_CD3	POMC
UBA523	8.19E-06	0.250604	1	0.811	0.130623	Blood_CD3	UBA52
NSFL1C1	8.28E-06	0.50744	0.327	0.133	0.132058	Blood_CD3	NSFL1C
EGLN2	8.4E-06	0.268866	0.327	0.122	0.133993	Blood_CD3	EGLN2
SRSF5	8.54E-06	0.402079	0.782	0.488	0.136171	Blood_CD3	SRSF5
CBR17	8.85E-06	0.393098	0.309	0.119	0.141147	Blood_CD3	CBR1
FAM96A3	8.93E-06	0.454145	0.273	0.099	0.142354	Blood_CD3	FAM96A
NUDT53	9.08E-06	0.542136	0.291	0.112	0.144804	Blood_CD3	NUDT5
UBE2L61	9.14E-06	0.253727	0.327	0.124	0.145791	Blood_CD3	UBE2L6
NDUFA81	9.92E-06	0.496076	0.382	0.168	0.158217	Blood_CD3	NDUFA8
RBM421	1.01E-05	0.392417	0.364	0.154	0.160614	Blood_CD3	RBM42
STK11IP	1.02E-05	0.262635	0.109	0.022	0.162217	Blood_CD3	STK11IP
ALG32	1.02E-05	0.377387	0.273	0.1	0.163051	Blood_CD3	ALG3
SSRP11	1.02E-05	0.264304	0.291	0.107	0.163193	Blood_CD3	SSRP1
PPP1R8	1.03E-05	0.36789	0.2	0.061	0.164283	Blood_CD3	PPP1R8
DEK10	1.04E-05	0.366667	0.527	0.259	0.166494	Blood_CD3	DEK
CSNK2B1	1.05E-05	0.396124	0.6	0.317	0.168042	Blood_CD3	CSNK2B
PSMB44	1.1E-05	0.393101	0.4	0.18	0.175652	Blood_CD3	PSMB4
RHOC9	1.1E-05	0.342699	0.491	0.224	0.175978	Blood_CD3	RHOC
PA2G44	1.12E-05	0.39095	0.509	0.245	0.178811	Blood_CD3	PA2G4
TMEM230	1.15E-05	0.472395	0.527	0.267	0.184174	Blood_CD3	TMEM230
PSD41	1.18E-05	0.36198	0.145	0.036	0.187929	Blood_CD3	PSD4
HSPA52	1.18E-05	0.27183	0.691	0.372	0.188099	Blood_CD3	HSPA5
DBN14	1.18E-05	0.496332	0.182	0.052	0.188644	Blood_CD3	DBN1
PEBP111	1.24E-05	0.272153	0.909	0.456	0.197015	Blood_CD3	PEBP1

IFT521	1.26E-05	0.365842	0.2	0.062	0.200344	Blood_CD3	IFT52
H2AFV4	1.27E-05	0.581091	0.545	0.291	0.202387	Blood_CD3	H2AFV
CNBP6	1.3E-05	0.372118	0.764	0.427	0.20671	Blood_CD3	CNBP
MPPE1	1.31E-05	0.44337	0.218	0.071	0.209152	Blood_CD3	MPPE1
COMMD8	1.32E-05	0.376676	0.255	0.089	0.210212	Blood_CD3	COMMD8
NQO22	1.34E-05	0.368714	0.291	0.113	0.213364	Blood_CD3	NQO2
RGS27	1.37E-05	0.520761	0.273	0.097	0.218002	Blood_CD3	RGS2
FUCA16	1.39E-05	0.561708	0.327	0.14	0.221754	Blood_CD3	FUCA1
ATP6V1H1	1.4E-05	0.406741	0.291	0.113	0.223101	Blood_CD3	ATP6V1H
CHID15	1.42E-05	0.448914	0.382	0.171	0.226036	Blood_CD3	CHID1
PSENEN2	1.46E-05	0.482876	0.455	0.217	0.233095	Blood_CD3	PSENEN
PTPN185	1.51E-05	0.361073	0.255	0.091	0.240591	Blood_CD3	PTPN18
NDUFA113	1.51E-05	0.392323	0.745	0.438	0.241141	Blood_CD3	NDUFA11
GRINA	1.51E-05	0.318825	0.327	0.131	0.241545	Blood_CD3	GRINA
SEP153	1.55E-05	0.375516	0.582	0.306	0.246779	Blood_CD3	SEP15
SMS	1.58E-05	0.370175	0.509	0.243	0.252687	Blood_CD3	SMS
NSMCE4A3	1.64E-05	0.422084	0.236	0.082	0.26193	Blood_CD3	NSMCE4A
NOB11	1.67E-05	0.420729	0.236	0.081	0.265762	Blood_CD3	NOB1
SND14	1.68E-05	0.308802	0.273	0.102	0.267926	Blood_CD3	SND1
THOC71	1.68E-05	0.36915	0.418	0.196	0.268122	Blood_CD3	THOC7
CCDC1078	1.8E-05	0.30445	0.4	0.177	0.286705	Blood_CD3	CCDC107
SRP148	1.81E-05	0.433075	0.964	0.701	0.287967	Blood_CD3	SRP14
YWHAH9	1.82E-05	0.312474	0.455	0.208	0.289979	Blood_CD3	YWHAH
MAPK1IP1	1.89E-05	0.416228	0.4	0.185	0.300869	Blood_CD3	MAPK1IP1L
TBC1D22A	1.91E-05	0.357728	0.182	0.054	0.305208	Blood_CD3	TBC1D22A
TCTN32	1.92E-05	0.311947	0.218	0.073	0.306885	Blood_CD3	TCTN3
TMEM179I	1.93E-05	0.400557	0.418	0.198	0.308167	Blood_CD3	TMEM179B
MFSD3	2E-05	0.311716	0.145	0.038	0.319771	Blood_CD3	MFSD3
SIRT21	2.06E-05	0.29938	0.273	0.101	0.328849	Blood_CD3	SIRT2
MTDH9	2.25E-05	0.485813	0.582	0.309	0.359071	Blood_CD3	MTDH
FARSA	2.26E-05	0.264109	0.2	0.063	0.360497	Blood_CD3	FARSA
TCEAL32	2.34E-05	0.420518	0.255	0.093	0.373739	Blood_CD3	TCEAL3
ATP5S	2.45E-05	0.26388	0.236	0.082	0.390263	Blood_CD3	ATP5S
C16orf132	2.45E-05	0.462805	0.436	0.212	0.390829	Blood_CD3	C16orf13
SERTAD33	2.55E-05	0.459233	0.309	0.124	0.406041	Blood_CD3	SERTAD3
STK32C	2.59E-05	0.251332	0.164	0.046	0.412985	Blood_CD3	STK32C
B3GAT33	2.61E-05	0.255258	0.236	0.082	0.416368	Blood_CD3	B3GAT3
COPS84	2.63E-05	0.354989	0.309	0.124	0.419183	Blood_CD3	COPS8
RP13.270P	2.7E-05	0.297473	0.091	0.017	0.430855	Blood_CD3	RP13.270P17.3
CAMK2G2	2.73E-05	0.329073	0.182	0.054	0.435112	Blood_CD3	CAMK2G
MYL12A3	2.76E-05	0.301739	0.982	0.658	0.439652	Blood_CD3	MYL12A
SMARCE12	2.8E-05	0.252189	0.327	0.135	0.446039	Blood_CD3	SMARCE1
SFT2D14	2.81E-05	0.39467	0.418	0.204	0.448932	Blood_CD3	SFT2D1
KHDRBS16	2.82E-05	0.314367	0.455	0.216	0.449229	Blood_CD3	KHDRBS1
HSF11	2.85E-05	0.372388	0.291	0.115	0.454671	Blood_CD3	HSF1
ABCF2	2.88E-05	0.274188	0.164	0.046	0.459293	Blood_CD3	ABCF2
ATP5J23	2.9E-05	0.419334	0.745	0.439	0.463077	Blood_CD3	ATP5J2

HNRNPR6	3E-05	0.31054	0.418	0.193	0.478341	Blood_CD3	HNRNPR
C1orf217	3.02E-05	0.284777	0.273	0.101	0.48096	Blood_CD3	C1orf21
ELAC2	3.17E-05	0.257121	0.145	0.039	0.504873	Blood_CD3	ELAC2
NDUFS67	3.26E-05	0.288739	0.618	0.324	0.520272	Blood_CD3	NDUFS6
GTF2F1	3.38E-05	0.392607	0.291	0.115	0.539777	Blood_CD3	GTF2F1
STRA135	3.44E-05	0.254349	0.418	0.195	0.548477	Blood_CD3	STRA13
MRPL9	3.52E-05	0.471247	0.273	0.108	0.560789	Blood_CD3	MRPL9
TEX2643	3.56E-05	0.395152	0.382	0.175	0.567394	Blood_CD3	TEX264
PCGF1	3.57E-05	0.250441	0.127	0.031	0.568797	Blood_CD3	PCGF1
BUD311	3.62E-05	0.362032	0.564	0.304	0.57809	Blood_CD3	BUD31
INO80E	3.67E-05	0.32853	0.2	0.065	0.585514	Blood_CD3	INO80E
STX101	3.76E-05	0.298836	0.309	0.126	0.600077	Blood_CD3	STX10
TAF92	3.81E-05	0.298434	0.455	0.217	0.607947	Blood_CD3	TAF9
ACP53	3.86E-05	0.278203	0.182	0.055	0.614932	Blood_CD3	ACP5
MPLKIP2	3.86E-05	0.346247	0.364	0.164	0.614965	Blood_CD3	MPLKIP
HEXB7	3.86E-05	0.335931	0.364	0.162	0.615509	Blood_CD3	HEXB
UBE2A4	3.91E-05	0.45934	0.436	0.213	0.622831	Blood_CD3	UBE2A
ATP6V0E1	4.05E-05	0.377567	0.891	0.544	0.646716	Blood_CD3	ATP6V0E1
RPL268	4.09E-05	0.345193	1	0.835	0.651605	Blood_CD3	RPL26
PPP5C	4.18E-05	0.29003	0.145	0.039	0.666047	Blood_CD3	PPP5C
APOO	4.18E-05	0.3195	0.164	0.049	0.667347	Blood_CD3	APOO
VARS1	4.62E-05	0.34682	0.182	0.057	0.736976	Blood_CD3	VARS
CLASRP	4.63E-05	0.535695	0.182	0.057	0.73828	Blood_CD3	CLASRP
ADPGK2	4.92E-05	0.388853	0.2	0.067	0.784729	Blood_CD3	ADPGK
MGST26	4.96E-05	0.409623	0.418	0.209	0.791691	Blood_CD3	MGST2
EBNA1BP2	4.98E-05	0.658801	0.291	0.123	0.794139	Blood_CD3	EBNA1BP2
EMC41	5.36E-05	0.326714	0.491	0.256	0.854716	Blood_CD3	EMC4
ATP5L6	5.42E-05	0.343897	0.982	0.66	0.86409	Blood_CD3	ATP5L
SLC25A261	5.77E-05	0.315237	0.236	0.087	0.919962	Blood_CD3	SLC25A26
STRADB2	5.93E-05	0.258168	0.182	0.058	0.944998	Blood_CD3	STRADB
TECR7	6.06E-05	0.306635	0.618	0.331	0.965776	Blood_CD3	TECR
CPSF61	6.12E-05	0.289469	0.255	0.097	0.976742	Blood_CD3	CPSF6
POP54	6.13E-05	0.286431	0.255	0.098	0.977328	Blood_CD3	POP5
YWHAB7	6.23E-05	0.2575	0.709	0.413	0.993477	Blood_CD3	YWHAB
C14orf12	6.27E-05	0.30207	0.309	0.133	0.999983	Blood_CD3	C14orf1
ZDHHC122	6.29E-05	0.30256	0.309	0.135	1	Blood_CD3	ZDHHC12
MRPL212	6.76E-05	0.575712	0.364	0.175	1	Blood_CD3	MRPL21
HINT112	6.77E-05	0.371999	0.909	0.57	1	Blood_CD3	HINT1
GYG1	6.77E-05	0.467052	0.273	0.111	1	Blood_CD3	GYG1
AAED19	6.8E-05	0.414083	0.218	0.076	1	Blood_CD3	AAED1
RHOA7	6.81E-05	0.394169	0.764	0.474	1	Blood_CD3	RHOA
B4GALT31	6.97E-05	0.27047	0.236	0.089	1	Blood_CD3	B4GALT3
RUVBL1	7.04E-05	0.308476	0.182	0.058	1	Blood_CD3	RUVBL1
FTSJ2	7.08E-05	0.315122	0.164	0.05	1	Blood_CD3	FTSJ2
CCNB1IP11	7.17E-05	0.351169	0.255	0.1	1	Blood_CD3	CCNB1IP1
GUSB3	7.29E-05	0.561636	0.309	0.137	1	Blood_CD3	GUSB
ARFGAP21	7.31E-05	0.354064	0.255	0.099	1	Blood_CD3	ARFGAP2

NDUFA412	7.34E-05	0.284292	0.982	0.538	1	Blood_CD3	NDUFA4
PSMC32	7.37E-05	0.293651	0.418	0.204	1	Blood_CD3	PSMC3
ATIC	7.48E-05	0.381287	0.182	0.059	1	Blood_CD3	ATIC
UBE2V2	7.76E-05	0.339748	0.364	0.17	1	Blood_CD3	UBE2V2
DDX271	8.06E-05	0.283786	0.255	0.098	1	Blood_CD3	DDX27
RANGRF	8.29E-05	0.303183	0.255	0.093	1	Blood_CD3	RANGRF
ZMAT51	8.41E-05	0.279191	0.218	0.078	1	Blood_CD3	ZMAT5
SNW12	8.55E-05	0.334838	0.327	0.143	1	Blood_CD3	SNW1
NUBP12	8.65E-05	0.2778	0.182	0.059	1	Blood_CD3	NUBP1
FNTA1	8.76E-05	0.456354	0.327	0.149	1	Blood_CD3	FNTA
SNAPC51	8.93E-05	0.3978	0.2	0.07	1	Blood_CD3	SNAPC5
VPS25	9.56E-05	0.481155	0.273	0.115	1	Blood_CD3	VPS25
C14orf166	9.79E-05	0.329796	0.618	0.338	1	Blood_CD3	C14orf166
CLTA4	9.89E-05	0.275814	0.691	0.386	1	Blood_CD3	CLTA
ITGB1BP18	9.96E-05	0.250359	0.345	0.152	1	Blood_CD3	ITGB1BP1
NENF9	0.000101	0.291772	0.6	0.329	1	Blood_CD3	NENF
UBE2D4	0.000103	0.269162	0.145	0.042	1	Blood_CD3	UBE2D4
UTP61	0.000111	0.284452	0.182	0.06	1	Blood_CD3	UTP6
ZFAND15	0.000115	0.444268	0.273	0.114	1	Blood_CD3	ZFAND1
L3MBTL2	0.000119	0.346696	0.127	0.034	1	Blood_CD3	L3MBTL2
ENDOV	0.000122	0.310149	0.182	0.061	1	Blood_CD3	ENDOV
ANP32A1	0.000122	0.26362	0.364	0.167	1	Blood_CD3	ANP32A
WARS4	0.000123	0.27733	0.218	0.08	1	Blood_CD3	WARS
PPIE	0.000125	0.327697	0.255	0.101	1	Blood_CD3	PPIE
DEDD21	0.000126	0.311427	0.291	0.122	1	Blood_CD3	DEDD2
SYNGR15	0.000129	0.419681	0.309	0.142	1	Blood_CD3	SYNGR1
TSTA34	0.00013	0.325358	0.364	0.176	1	Blood_CD3	TSTA3
NSUN51	0.000133	0.289341	0.2	0.071	1	Blood_CD3	NSUN5
PAQR82	0.000135	0.295248	0.073	0.014	1	Blood_CD3	PAQR8
FPGS	0.000136	0.466728	0.145	0.043	1	Blood_CD3	FPGS
DNAJC74	0.000137	0.305369	0.345	0.16	1	Blood_CD3	DNAJC7
NUDCD2	0.000141	0.520652	0.291	0.13	1	Blood_CD3	NUDCD2
PRKX3	0.000146	0.27488	0.145	0.043	1	Blood_CD3	PRKX
MRPL2011	0.000148	0.458016	0.491	0.271	1	Blood_CD3	MRPL20
ATP5E8	0.000149	0.254335	0.945	0.684	1	Blood_CD3	ATP5E
RNASEH2C	0.00015	0.453699	0.382	0.187	1	Blood_CD3	RNASEH2C
C21orf59	0.000155	0.282395	0.273	0.114	1	Blood_CD3	C21orf59
BCL7B2	0.000156	0.276294	0.255	0.102	1	Blood_CD3	BCL7B
EMC73	0.00016	0.271658	0.4	0.199	1	Blood_CD3	EMC7
ZNF5111	0.000163	0.411197	0.236	0.094	1	Blood_CD3	ZNF511
PSMB58	0.000165	0.525957	0.436	0.241	1	Blood_CD3	PSMB5
ZNF5241	0.000172	0.450159	0.255	0.106	1	Blood_CD3	ZNF524
MRFAP18	0.000173	0.364341	0.491	0.267	1	Blood_CD3	MRFAP1
MRE11A2	0.000174	0.251227	0.127	0.035	1	Blood_CD3	MRE11A
DLD1	0.000174	0.570033	0.218	0.084	1	Blood_CD3	DLD
YRDC1	0.000175	0.306344	0.164	0.053	1	Blood_CD3	YRDC
MZT2B6	0.000179	0.368254	0.782	0.461	1	Blood_CD3	MZT2B

ASL1	0.000185	0.27053	0.164	0.053	1	Blood_CD3	ASL
WDR1	0.000185	0.415903	0.527	0.295	1	Blood_CD3	WDR1
RUFY11	0.000189	0.316577	0.164	0.053	1	Blood_CD3	RUFY1
CYHR13	0.000192	0.422398	0.273	0.117	1	Blood_CD3	CYHR1
COMMD12	0.000195	0.32818	0.345	0.166	1	Blood_CD3	COMMD1
GCA2	0.000195	0.360401	0.2	0.073	1	Blood_CD3	GCA
NME15	0.000211	0.377196	0.345	0.165	1	Blood_CD3	NME1
PXMP2	0.000214	0.296156	0.182	0.064	1	Blood_CD3	PXMP2
DAZAP1	0.000214	0.316364	0.291	0.129	1	Blood_CD3	DAZAP1
MRPS5	0.000222	0.27683	0.273	0.116	1	Blood_CD3	MRPS5
ISOC25	0.000228	0.440983	0.291	0.133	1	Blood_CD3	ISOC2
ZNF880	0.000233	0.250411	0.127	0.036	1	Blood_CD3	ZNF880
EFTUD21	0.000247	0.258315	0.145	0.045	1	Blood_CD3	EFTUD2
MAN1B11	0.000248	0.273438	0.2	0.074	1	Blood_CD3	MAN1B1
SCRN21	0.000248	0.317881	0.182	0.064	1	Blood_CD3	SCRN2
ANXA45	0.000249	0.279535	0.309	0.138	1	Blood_CD3	ANXA4
TMEM2084	0.000252	0.317658	0.382	0.197	1	Blood_CD3	TMEM208
MCUR11	0.000255	0.449154	0.327	0.158	1	Blood_CD3	MCUR1
CHPT17	0.000281	0.256688	0.418	0.207	1	Blood_CD3	CHPT1
FAM58A1	0.000294	0.285289	0.182	0.065	1	Blood_CD3	FAM58A
PDIA65	0.000298	0.280046	0.527	0.279	1	Blood_CD3	PDIA6
FAM50A2	0.000323	0.26265	0.291	0.132	1	Blood_CD3	FAM50A
AIG14	0.000324	0.356648	0.255	0.109	1	Blood_CD3	AIG1
ASAP16	0.000341	0.440778	0.236	0.095	1	Blood_CD3	ASAP1
RILPL25	0.000345	0.39304	0.236	0.097	1	Blood_CD3	RILPL2
SSB7	0.000346	0.298139	0.4	0.198	1	Blood_CD3	SSB
ATXN2L1	0.000358	0.32352	0.182	0.065	1	Blood_CD3	ATXN2L
TBCC2	0.00036	0.356382	0.236	0.096	1	Blood_CD3	TBCC
TRAPPC42	0.000368	0.272047	0.291	0.133	1	Blood_CD3	TRAPPC4
SNRPD34	0.000376	0.255937	0.436	0.232	1	Blood_CD3	SNRPD3
NKAP3	0.000402	0.274192	0.236	0.097	1	Blood_CD3	NKAP
TIMM17A4	0.000413	0.350601	0.291	0.136	1	Blood_CD3	TIMM17A
RNPEP	0.000416	0.56465	0.273	0.127	1	Blood_CD3	RNPEP
DNTTIP11	0.000417	0.341473	0.218	0.088	1	Blood_CD3	DNTTIP1
SCAMP22	0.000432	0.344544	0.364	0.189	1	Blood_CD3	SCAMP2
DEF82	0.000436	0.292333	0.218	0.088	1	Blood_CD3	DEF8
PRCC	0.00044	0.30775	0.2	0.077	1	Blood_CD3	PRCC
GLIPR28	0.000443	0.310138	0.218	0.085	1	Blood_CD3	GLIPR2
TNFAIP86	0.000449	0.280618	0.255	0.105	1	Blood_CD3	TNFAIP8
FBXO73	0.000455	0.407326	0.327	0.158	1	Blood_CD3	FBXO7
C19orf242	0.000459	0.394918	0.364	0.189	1	Blood_CD3	C19orf24
ATP5H1	0.000467	0.282997	0.582	0.35	1	Blood_CD3	ATP5H
KARS	0.000486	0.304392	0.327	0.159	1	Blood_CD3	KARS
SNX54	0.000495	0.289932	0.255	0.111	1	Blood_CD3	SNX5
NOL71	0.000504	0.282868	0.4	0.206	1	Blood_CD3	NOL7
PRKCSH2	0.000521	0.306885	0.309	0.15	1	Blood_CD3	PRKCSH
MYL12B1	0.000535	0.267144	0.909	0.607	1	Blood_CD3	MYL12B

MRPL281	0.000547	0.296796	0.309	0.148	1	Blood_CD3	MRPL28
COX165	0.000553	0.265545	0.273	0.12	1	Blood_CD3	COX16
BAZ1A6	0.000555	0.3033	0.455	0.245	1	Blood_CD3	BAZ1A
HEBP11	0.000569	0.318711	0.364	0.189	1	Blood_CD3	HEBP1
SDHB1	0.00058	0.341908	0.327	0.164	1	Blood_CD3	SDHB
TMEM216	0.000584	0.25032	0.127	0.039	1	Blood_CD3	TMEM216
TMEM42	0.000598	0.312076	0.164	0.058	1	Blood_CD3	TMEM42
UVRAG3	0.000607	0.47422	0.145	0.049	1	Blood_CD3	UVRAG
MRPL152	0.000619	0.421595	0.218	0.092	1	Blood_CD3	MRPL15
SUB17	0.000624	0.276876	0.782	0.48	1	Blood_CD3	SUB1
EIF2B1	0.000624	0.378619	0.182	0.069	1	Blood_CD3	EIF2B1
COMMD10	0.000671	0.287446	0.2	0.08	1	Blood_CD3	COMMD10
WDR335	0.000673	0.306601	0.309	0.152	1	Blood_CD3	WDR33
DCTD1	0.000682	0.395985	0.2	0.081	1	Blood_CD3	DCTD
ACSL51	0.000697	0.261911	0.109	0.031	1	Blood_CD3	ACSL5
PSMD72	0.000702	0.456913	0.382	0.212	1	Blood_CD3	PSMD7
GPAA17	0.000706	0.470609	0.291	0.143	1	Blood_CD3	GPAA1
MRPL37	0.000726	0.480317	0.218	0.093	1	Blood_CD3	MRPL37
CMTM35	0.000729	0.602189	0.2	0.076	1	Blood_CD3	CMTM3
MVB12A2	0.000775	0.264911	0.236	0.103	1	Blood_CD3	MVB12A
DDX39A1	0.000784	0.33462	0.273	0.121	1	Blood_CD3	DDX39A
HNRNPK7	0.000792	0.300783	0.691	0.447	1	Blood_CD3	HNRNPK
RPE	0.000794	0.337524	0.145	0.05	1	Blood_CD3	RPE
HPS41	0.000801	0.288012	0.145	0.049	1	Blood_CD3	HPS4
XRCC54	0.000861	0.301969	0.436	0.248	1	Blood_CD3	XRCC5
C8orf823	0.000877	0.262782	0.182	0.071	1	Blood_CD3	C8orf82
DDX541	0.0009	0.310263	0.164	0.06	1	Blood_CD3	DDX54
SAP18	0.000901	0.343297	0.836	0.539	1	Blood_CD3	SAP18
C12orf10	0.000907	0.325533	0.309	0.15	1	Blood_CD3	C12orf10
FDPS4	0.000921	0.284596	0.364	0.192	1	Blood_CD3	FDPS
PRPF38A1	0.000951	0.299649	0.255	0.117	1	Blood_CD3	PRPF38A
COQ41	0.001011	0.322223	0.218	0.095	1	Blood_CD3	COQ4
SLC39A41	0.001017	0.35569	0.164	0.061	1	Blood_CD3	SLC39A4
RPP25L	0.001021	0.350325	0.182	0.072	1	Blood_CD3	RPP25L
ICT11	0.001024	0.416765	0.164	0.061	1	Blood_CD3	ICT1
C19orf534	0.001026	0.268923	0.673	0.423	1	Blood_CD3	C19orf53
PTPN23	0.001028	0.362634	0.255	0.117	1	Blood_CD3	PTPN2
CCT41	0.001077	0.266783	0.418	0.227	1	Blood_CD3	CCT4
TXNRD22	0.001083	0.258316	0.127	0.041	1	Blood_CD3	TXNRD2
DLGAP41	0.00112	0.411435	0.255	0.119	1	Blood_CD3	DLGAP4
CLN61	0.001128	0.304126	0.127	0.042	1	Blood_CD3	CLN6
COMMD73	0.001133	0.251623	0.273	0.129	1	Blood_CD3	COMMD7
COX7C6	0.00115	0.2541	0.964	0.638	1	Blood_CD3	COX7C
CREG14	0.001224	0.278066	0.291	0.145	1	Blood_CD3	CREG1
CWC151	0.001257	0.25609	0.382	0.208	1	Blood_CD3	CWC15
CERS22	0.001283	0.340785	0.2	0.083	1	Blood_CD3	CERS2
SLC25A20	0.001312	0.580044	0.073	0.017	1	Blood_CD3	SLC25A20

YIF1A6	0.001368	0.312288	0.327	0.173	1	Blood_CD3	YIF1A
RPP30	0.001423	0.287891	0.2	0.085	1	Blood_CD3	RPP30
PHF111	0.00146	0.296043	0.218	0.095	1	Blood_CD3	PHF11
TXNL4A3	0.001462	0.417667	0.4	0.234	1	Blood_CD3	TXNL4A
AGGF11	0.001468	0.374728	0.182	0.075	1	Blood_CD3	AGGF1
NDUFB111	0.001513	0.285544	0.745	0.475	1	Blood_CD3	NDUFB11
MPV172	0.001522	0.320567	0.182	0.074	1	Blood_CD3	MPV17
CNOT8	0.001546	0.339034	0.273	0.133	1	Blood_CD3	CNOT8
PEX191	0.001582	0.36098	0.145	0.053	1	Blood_CD3	PEX19
HEXA3	0.001649	0.277919	0.255	0.121	1	Blood_CD3	HEXA
ACO23	0.001676	0.373264	0.218	0.099	1	Blood_CD3	ACO2
MAP3K87	0.001682	0.379253	0.255	0.117	1	Blood_CD3	MAP3K8
TRAPPC33	0.001703	0.331723	0.364	0.202	1	Blood_CD3	TRAPPC3
ADPRHL2	0.001804	0.331149	0.164	0.064	1	Blood_CD3	ADPRHL2
ETV65	0.001904	0.266477	0.236	0.111	1	Blood_CD3	ETV6
MRS2	0.001917	0.278004	0.127	0.044	1	Blood_CD3	MRS2
SLC22A181	0.002067	0.342007	0.164	0.064	1	Blood_CD3	SLC22A18
WDR133	0.002068	0.269898	0.236	0.111	1	Blood_CD3	WDR13
NPRL2	0.002082	0.30351	0.145	0.054	1	Blood_CD3	NPRL2
TMEM1415	0.002124	0.31217	0.309	0.165	1	Blood_CD3	TMEM141
DYNC1LI11	0.002136	0.337608	0.2	0.087	1	Blood_CD3	DYNC1LI1
SRA14	0.002196	0.572136	0.327	0.182	1	Blood_CD3	SRA1
DPM23	0.002201	0.283955	0.236	0.113	1	Blood_CD3	DPM2
NDUFB61	0.002203	0.387157	0.382	0.223	1	Blood_CD3	NDUFB6
HNRNPC7	0.002269	0.370434	0.636	0.43	1	Blood_CD3	HNRNPC
SNX23	0.002313	0.268986	0.291	0.149	1	Blood_CD3	SNX2
TMEM14C9	0.002396	0.440974	0.418	0.256	1	Blood_CD3	TMEM14C
PPA21	0.002427	0.252276	0.255	0.126	1	Blood_CD3	PPA2
STX18	0.002434	0.360072	0.182	0.078	1	Blood_CD3	STX18
STX73	0.002438	0.308935	0.255	0.125	1	Blood_CD3	STX7
FBXO61	0.002604	0.301892	0.127	0.045	1	Blood_CD3	FBXO6
LTBR7	0.002702	0.336969	0.2	0.089	1	Blood_CD3	LTBR
HSCB1	0.002863	0.279951	0.145	0.056	1	Blood_CD3	HSCB
PDAP11	0.002892	0.263114	0.382	0.219	1	Blood_CD3	PDAP1
DTNBP11	0.002915	0.27921	0.164	0.066	1	Blood_CD3	DTNBP1
SF3B4	0.003083	0.350736	0.218	0.103	1	Blood_CD3	SF3B4
MIS18BP14	0.003201	0.334613	0.218	0.103	1	Blood_CD3	MIS18BP1
ACP21	0.00323	0.306205	0.145	0.057	1	Blood_CD3	ACP2
CHTF8	0.003288	0.282366	0.182	0.079	1	Blood_CD3	CHTF8
RARA4	0.003298	0.283613	0.145	0.056	1	Blood_CD3	RARA
STIM1	0.003394	0.25294	0.145	0.057	1	Blood_CD3	STIM1
TIAL1	0.00345	0.353027	0.255	0.131	1	Blood_CD3	TIAL1
FAH1	0.003633	0.312646	0.091	0.028	1	Blood_CD3	FAH
SDR39U1	0.00366	0.253845	0.182	0.079	1	Blood_CD3	SDR39U1
FOXN21	0.003717	0.27036	0.109	0.037	1	Blood_CD3	FOXN2
LAGE33	0.003832	0.304205	0.273	0.143	1	Blood_CD3	LAGE3
ADRBK21	0.003957	0.265125	0.091	0.028	1	Blood_CD3	ADRBK2

TMEM9B2	0.004135	0.290735	0.291	0.157	1	Blood_CD3	TMEM9B
HSPBP1	0.004154	0.317028	0.145	0.058	1	Blood_CD3	HSPBP1
HSDL2	0.004195	0.27433	0.2	0.093	1	Blood_CD3	HSDL2
IFNGR22	0.004203	0.400682	0.364	0.205	1	Blood_CD3	IFNGR2
TRIM26	0.004212	0.274224	0.127	0.047	1	Blood_CD3	TRIM26
ACOT8	0.004402	0.309723	0.145	0.058	1	Blood_CD3	ACOT8
POLR3K2	0.004443	0.277385	0.164	0.069	1	Blood_CD3	POLR3K
RNASEH2B	0.004815	0.358613	0.218	0.105	1	Blood_CD3	RNASEH2B
IMMP2L1	0.00512	0.321995	0.127	0.049	1	Blood_CD3	IMMP2L
ACTN17	0.005289	0.315613	0.273	0.148	1	Blood_CD3	ACTN1
ANAPC151	0.00558	0.467684	0.236	0.124	1	Blood_CD3	ANAPC15
FLII	0.005601	0.27418	0.236	0.12	1	Blood_CD3	FLII
RCE1	0.005726	0.472806	0.127	0.05	1	Blood_CD3	RCE1
COMMD31	0.005924	0.288486	0.291	0.162	1	Blood_CD3	COMMD3
RHOT2	0.005976	0.359495	0.182	0.083	1	Blood_CD3	RHOT2
WDR41	0.006412	0.262364	0.145	0.061	1	Blood_CD3	WDR41
RSU15	0.006873	0.467365	0.236	0.123	1	Blood_CD3	RSU1
ZNF414	0.006885	0.303514	0.127	0.05	1	Blood_CD3	ZNF414
SCAMP42	0.007053	0.255378	0.145	0.062	1	Blood_CD3	SCAMP4
PRADC11	0.007355	0.279473	0.182	0.086	1	Blood_CD3	PRADC1
TXNDC121	0.00741	0.371815	0.255	0.14	1	Blood_CD3	TXNDC12
DNAJB121	0.007435	0.396433	0.218	0.114	1	Blood_CD3	DNAJB12
CHMP6	0.008152	0.308453	0.127	0.05	1	Blood_CD3	CHMP6
HSD17B12	0.009336	0.258384	0.218	0.114	1	Blood_CD3	HSD17B12
IL322	0	3.523413	0.914	0.169	0	Blood_Treg	IL32
LTB1	0	2.923398	0.934	0.158	0	Blood_Treg	LTB
CD3E2	0	2.534191	0.799	0.096	0	Blood_Treg	CD3E
CORO1A4	0	2.479988	0.863	0.184	0	Blood_Treg	CORO1A
FXYD53	0	2.397058	0.891	0.286	0	Blood_Treg	FXYD5
CD3D2	0	2.388369	0.78	0.11	0	Blood_Treg	CD3D
HLA.A5	0	2.354299	0.998	0.661	0	Blood_Treg	HLA.A
CD522	0	2.350893	0.926	0.212	0	Blood_Treg	CD52
ARHGDIB2	0	2.253456	0.938	0.437	0	Blood_Treg	ARHGDIB
LDHB1	0	2.24556	0.854	0.303	0	Blood_Treg	LDHB
RAC22	0	2.209722	0.697	0.144	0	Blood_Treg	RAC2
HLA.B6	0	2.192232	1	0.659	0	Blood_Treg	HLA.B
EVL3	0	2.190699	0.654	0.155	0	Blood_Treg	EVL
PFN14	0	2.185473	0.992	0.576	0	Blood_Treg	PFN1
LCK2	0	2.134404	0.56	0.073	0	Blood_Treg	LCK
GPSM34	0	2.054507	0.676	0.161	0	Blood_Treg	GPSM3
IL2RG3	0	2.035287	0.576	0.123	0	Blood_Treg	IL2RG
HLA.C6	0	1.985693	0.996	0.679	0	Blood_Treg	HLA.C
TRAF3IP31	0	1.977229	0.521	0.087	0	Blood_Treg	TRAF3IP3
LIMD23	0	1.960543	0.681	0.163	0	Blood_Treg	LIMD2
LAT3	0	1.951426	0.463	0.057	0	Blood_Treg	LAT
ARPC1B6	0	1.893609	0.706	0.283	0	Blood_Treg	ARPC1B
SH3BGRL3	0	1.891602	0.961	0.625	0	Blood_Treg	SH3BGRL3

S1PR41	0	1.888789	0.406	0.049	0	Blood_Tre|	S1PR4
CD22	0	1.877346	0.439	0.057	0	Blood_Tre|	CD2
UCP25	0	1.866353	0.474	0.147	0	Blood_Tre|	UCP2
CD272	0	1.854	0.476	0.077	0	Blood_Tre|	CD27
GSTK13	0	1.844499	0.63	0.289	0	Blood_Tre|	GSTK1
COTL16	0	1.815739	0.594	0.175	0	Blood_Tre|	COTL1
TSC22D35	0	1.807455	0.618	0.274	0	Blood_Tre|	TSC22D3
AES3	0	1.805471	0.714	0.388	0	Blood_Tre|	AES
ICAM34	0	1.780738	0.493	0.137	0	Blood_Tre|	ICAM3
TBC1D10C	0	1.773804	0.426	0.065	0	Blood_Tre|	TBC1D10C
GIMAP73	0	1.756519	0.533	0.112	0	Blood_Tre|	GIMAP7
RPSA3	0	1.736835	0.985	0.671	0	Blood_Tre|	RPSA
RPS221	0	1.735184	1	0.796	0	Blood_Tre|	RPS2
CD534	0	1.722033	0.48	0.115	0	Blood_Tre|	CD53
GLTSCR22	0	1.71358	0.893	0.521	0	Blood_Tre|	GLTSCR2
SEPT13	0	1.689392	0.406	0.092	0	Blood_Tre|	SEPT1
CD485	0	1.676546	0.501	0.115	0	Blood_Tre|	CD48
RPL44	0	1.674361	0.979	0.693	0	Blood_Tre|	RPL4
DOK25	0	1.672173	0.334	0.056	0	Blood_Tre|	DOK2
IL7R2	0	1.670312	0.347	0.055	0	Blood_Tre|	IL7R
RPS35	0	1.65584	1	0.812	0	Blood_Tre|	RPS3
CFL15	0	1.64435	0.949	0.704	0	Blood_Tre|	CFL1
LSP15	0	1.628356	0.591	0.223	0	Blood_Tre|	LSP1
PSME13	0	1.623316	0.685	0.377	0	Blood_Tre|	PSME1
CD374	0	1.598618	0.699	0.192	0	Blood_Tre|	CD37
EEF1D5	0	1.592896	0.979	0.741	0	Blood_Tre|	EEF1D
RPS67	0	1.589057	1	0.821	0	Blood_Tre|	RPS6
GMFG5	0	1.586945	0.616	0.201	0	Blood_Tre|	GMFG
RPS4X6	0	1.58389	1	0.766	0	Blood_Tre|	RPS4X
LAPTM57	0	1.562028	0.56	0.164	0	Blood_Tre|	LAPTM5
ISG204	0	1.542387	0.492	0.196	0	Blood_Tre|	ISG20
BIN22	0	1.531274	0.349	0.072	0	Blood_Tre|	BIN2
RPL63	0	1.522961	0.998	0.789	0	Blood_Tre|	RPL6
RPL327	0	1.516378	1	0.84	0	Blood_Tre|	RPL3
BIN12	0	1.508681	0.367	0.112	0	Blood_Tre|	BIN1
ITGB26	0	1.481002	0.415	0.102	0	Blood_Tre|	ITGB2
CXCR44	0	1.473156	0.403	0.117	0	Blood_Tre|	CXCR4
GNB2L14	0	1.473033	0.993	0.748	0	Blood_Tre|	GNB2L1
CD2471	0	1.472745	0.326	0.047	0	Blood_Tre|	CD247
SPOCK22	0	1.464691	0.282	0.038	0	Blood_Tre|	SPOCK2
DDX57	0	1.454812	0.852	0.596	0	Blood_Tre|	DDX5
FLT3LG	0	1.453592	0.305	0.051	0	Blood_Tre|	FLT3LG
RPL172	0	1.43506	0.856	0.518	0	Blood_Tre|	RPL17
IL10RA6	0	1.431195	0.252	0.04	0	Blood_Tre|	IL10RA
RPL13A7	0	1.416798	1	0.879	0	Blood_Tre|	RPL13A
IER25	0	1.412177	0.788	0.541	0	Blood_Tre|	IER2
B2M7	0	1.400257	1	0.927	0	Blood_Tre|	B2M

ACTB8	0	1.388878	0.996	0.869	0	Blood_Tre	ACTB
SLC25A68	0	1.350811	0.852	0.562	0	Blood_Tre	SLC25A6
JUNB4	0	1.349589	0.943	0.684	0	Blood_Tre	JUNB
RPL57	0	1.348608	0.988	0.722	0	Blood_Tre	RPL5
RPL1010	0	1.335285	1	0.875	0	Blood_Tre	RPL10
CD71	0	1.326112	0.424	0.094	0	Blood_Tre	CD7
RHOH1	0	1.306985	0.261	0.05	0	Blood_Tre	RHOH
RPL134	0	1.302731	1	0.917	0	Blood_Tre	RPL13
ARHGAP15	0	1.300834	0.264	0.058	0	Blood_Tre	ARHGAP15
RPL145	0	1.299685	0.99	0.792	0	Blood_Tre	RPL14
HNRNPA17	0	1.299248	0.831	0.539	0	Blood_Tre	HNRNPA1
CD62	0	1.282957	0.225	0.025	0	Blood_Tre	CD6
EEF27	0	1.274865	0.849	0.603	0	Blood_Tre	EEF2
RPLP04	0	1.249899	0.971	0.794	0	Blood_Tre	RPLP0
GIMAP46	0	1.249699	0.296	0.07	0	Blood_Tre	GIMAP4
SELL1	0	1.233929	0.28	0.059	0	Blood_Tre	SELL
HLA.E6	0	1.230485	0.851	0.604	0	Blood_Tre	HLA.E
EMP310	0	1.214682	0.709	0.343	0	Blood_Tre	EMP3
SUSD32	0	1.212714	0.21	0.031	0	Blood_Tre	SUSD3
CD3G2	0	1.208569	0.245	0.037	0	Blood_Tre	CD3G
MFNG2	0	1.205441	0.222	0.04	0	Blood_Tre	MFNG
RPL89	0	1.182926	0.997	0.846	0	Blood_Tre	RPL8
RPS76	0	1.179053	0.995	0.812	0	Blood_Tre	RPS7
RPL196	0	1.17414	0.998	0.846	0	Blood_Tre	RPL19
BTG19	0	1.157309	0.827	0.572	0	Blood_Tre	BTG1
S100A410	0	1.141947	0.926	0.455	0	Blood_Tre	S100A4
ITGB73	0	1.134349	0.195	0.034	0	Blood_Tre	ITGB7
RPL10A9	0	1.128626	0.992	0.729	0	Blood_Tre	RPL10A
RPL18A8	0	1.127186	0.999	0.819	0	Blood_Tre	RPL18A
RPL116	0	1.108683	1	0.851	0	Blood_Tre	RPL11
RPL296	0	1.070144	0.994	0.826	0	Blood_Tre	RPL29
RPL186	0	1.069655	0.995	0.876	0	Blood_Tre	RPL18
ZAP702	0	1.066685	0.185	0.026	0	Blood_Tre	ZAP70
RPL97	0	1.058282	0.985	0.745	0	Blood_Tre	RPL9
RPS104	0	1.057101	0.86	0.602	0	Blood_Tre	RPS10
RPS57	0	1.030851	0.982	0.782	0	Blood_Tre	RPS5
CD54	0	1.029699	0.156	0.015	0	Blood_Tre	CD5
RPL129	0	1.019919	0.993	0.85	0	Blood_Tre	RPL12
CYBA6	0	1.013326	0.801	0.433	0	Blood_Tre	CYBA
RPL158	0	1.007543	0.997	0.893	0	Blood_Tre	RPL15
FUT7	0	0.989909	0.127	0.008	0	Blood_Tre	FUT7
TNFRSF251	0	0.964249	0.149	0.019	0	Blood_Tre	TNFRSF25
TIGIT2	0	0.940406	0.104	0.01	0	Blood_Tre	TIGIT
RPS3A5	0	0.928724	0.989	0.828	0	Blood_Tre	RPS3A
RPL23A6	0	0.916525	0.995	0.799	0	Blood_Tre	RPL23A
NACA4	0	0.906576	0.95	0.828	0	Blood_Tre	NACA
RPL7A12	0	0.87135	0.983	0.81	0	Blood_Tre	RPL7A

TMSB4X9	0	0.858789	1	0.868	0	Blood_Tre	TMSB4X
RPS98	0	0.854123	0.989	0.833	0	Blood_Tre	RPS9
TMSB108	0	0.815222	0.993	0.876	0	Blood_Tre	TMSB10
RPS198	0	0.799093	0.996	0.873	0	Blood_Tre	RPS19
RPS166	0	0.789082	0.978	0.833	0	Blood_Tre	RPS16
RPL328	0	0.758031	0.997	0.869	0	Blood_Tre	RPL32
RPL283	0	0.742749	0.993	0.908	0	Blood_Tre	RPL28
FOXP3	0	0.507924	0.055	0.001	0	Blood_Tre	FOXP3
ACAP12	2E-307	1.187038	0.41	0.13	3.1E-303	Blood_Tre	ACAP1
IL2RA1	9.8E-304	0.611892	0.065	0.004	1.6E-299	Blood_Tre	IL2RA
TNFRSF44	1.1E-303	1.083156	0.164	0.025	1.7E-299	Blood_Tre	TNFRSF4
GAPDH12	3.5E-300	0.991029	0.907	0.761	5.6E-296	Blood_Tre	GAPDH
RPL3112	4.5E-298	0.767715	0.975	0.764	7.1E-294	Blood_Tre	RPL31
RPLP25	1.5E-296	0.702743	0.991	0.841	2.4E-292	Blood_Tre	RPLP2
CDC25B1	2.9E-296	1.180236	0.188	0.034	4.7E-292	Blood_Tre	CDC25B
RPS89	4.6E-294	0.762538	0.99	0.846	7.4E-290	Blood_Tre	RPS8
RPL217	1.3E-293	0.746656	0.997	0.81	2.1E-289	Blood_Tre	RPL21
SIRPG	1.6E-289	0.82177	0.124	0.015	2.6E-285	Blood_Tre	SIRPG
COX4I13	7.4E-288	0.917496	0.868	0.722	1.2E-283	Blood_Tre	COX4I1
RPL27A9	1.1E-284	0.726721	0.989	0.833	1.8E-280	Blood_Tre	RPL27A
NPM16	8.5E-278	1.116419	0.778	0.528	1.3E-273	Blood_Tre	NPM1
TRADD3	3.3E-271	1.587265	0.336	0.109	5.2E-267	Blood_Tre	TRADD
RPS255	5.2E-270	0.71216	0.977	0.821	8.2E-266	Blood_Tre	RPS25
UBA524	2.4E-265	0.693549	0.96	0.808	3.9E-261	Blood_Tre	UBA52
ICAM23	2.5E-264	1.212452	0.256	0.064	4E-260	Blood_Tre	ICAM2
EIF3K5	3.7E-264	1.216271	0.717	0.514	6E-260	Blood_Tre	EIF3K
PTGER2	8.9E-261	0.98199	0.139	0.021	1.4E-256	Blood_Tre	PTGER2
FYB4	6.8E-258	1.155369	0.266	0.069	1.1E-253	Blood_Tre	FYB
SKAP12	3.1E-257	1.232383	0.23	0.054	4.9E-253	Blood_Tre	SKAP1
RPS149	2.5E-256	0.659393	0.998	0.88	4E-252	Blood_Tre	RPS14
LEF11	8.5E-254	1.074162	0.185	0.036	1.4E-249	Blood_Tre	LEF1
MYO1G4	1E-252	0.961735	0.164	0.029	1.6E-248	Blood_Tre	MYO1G
EEF1A116	2E-250	0.658252	0.997	0.913	3.2E-246	Blood_Tre	EEF1A1
RPS188	8.6E-249	0.613657	0.998	0.914	1.4E-244	Blood_Tre	RPS18
GIMAP14	2.2E-246	1.081066	0.232	0.056	3.6E-242	Blood_Tre	GIMAP1
GYPC7	1.2E-245	1.203513	0.47	0.198	1.8E-241	Blood_Tre	GYPC
HSPA810	9.7E-243	1.218869	0.699	0.506	1.5E-238	Blood_Tre	HSPA8
HMHA13	1.3E-242	1.1451	0.248	0.064	2E-238	Blood_Tre	HMHA1
CD40LG2	1.2E-240	0.742883	0.11	0.014	1.9E-236	Blood_Tre	CD40LG
RPL79	5.4E-239	0.870888	0.935	0.794	8.6E-235	Blood_Tre	RPL7
NOSIP	5.4E-238	1.554711	0.537	0.291	8.7E-234	Blood_Tre	NOSIP
RPS15A6	1.4E-234	0.622811	0.993	0.847	2.2E-230	Blood_Tre	RPS15A
TRAT12	1.6E-234	0.85282	0.137	0.022	2.6E-230	Blood_Tre	TRAT1
CCR102	2.6E-233	0.927362	0.098	0.012	4.2E-229	Blood_Tre	CCR10
JUN9	2.2E-228	1.00279	0.743	0.482	3.5E-224	Blood_Tre	JUN
PTMA10	4.5E-227	0.63191	0.986	0.816	7.2E-223	Blood_Tre	PTMA
ARHGEF1	1.1E-223	1.167001	0.247	0.068	1.8E-219	Blood_Tre	ARHGEF1

WAS5	1.2E-223	0.9751	0.188	0.041	1.9E-219	Blood_Tre	WAS
MYL12A4	1E-220	0.868337	0.835	0.654	1.6E-216	Blood_Tre	MYL12A
RGS193	6.5E-220	1.142923	0.234	0.062	1E-215	Blood_Tre	RGS19
HCLS14	1.2E-218	1.121705	0.259	0.074	1.9E-214	Blood_Tre	HCLS1
EIF3F3	6.6E-215	1.261948	0.63	0.436	1.1E-210	Blood_Tre	EIF3F
RPS159	1.2E-214	0.572723	0.996	0.878	1.9E-210	Blood_Tre	RPS15
EIF15	1.2E-211	0.599724	0.976	0.908	1.9E-207	Blood_Tre	EIF1
TAGLN211	2.2E-211	1.255886	0.627	0.404	3.5E-207	Blood_Tre	TAGLN2
IL12RB1	1.9E-210	0.601404	0.084	0.01	3.1E-206	Blood_Tre	IL12RB1
CTLA41	1.1E-206	0.401017	0.042	0.002	1.7E-202	Blood_Tre	CTLA4
CNBP7	2.9E-204	1.296358	0.609	0.423	4.7E-200	Blood_Tre	CNBP
EEF1B26	6.5E-200	0.87172	0.784	0.593	1E-195	Blood_Tre	EEF1B2
BTF38	4.1E-198	0.835431	0.832	0.709	6.6E-194	Blood_Tre	BTF3
RCSD13	6.2E-198	1.003768	0.199	0.05	9.9E-194	Blood_Tre	RCSD1
LINC00861	1.6E-194	0.761041	0.123	0.021	2.5E-190	Blood_Tre	LINC00861
PPP1R185	1.7E-193	1.320641	0.309	0.114	2.7E-189	Blood_Tre	PPP1R18
ARHGDIA9	9.1E-193	1.411354	0.408	0.192	1.4E-188	Blood_Tre	ARHGDIA
HOXB2	2.2E-192	1.091696	0.176	0.042	3.6E-188	Blood_Tre	HOXB2
RPL269	3.5E-191	0.558205	0.983	0.832	5.6E-187	Blood_Tre	RPL26
ANXA64	1.1E-190	1.278557	0.294	0.104	1.7E-186	Blood_Tre	ANXA6
GTF3A2	1.1E-189	1.421712	0.46	0.246	1.7E-185	Blood_Tre	GTF3A
GZMM1	4.8E-188	0.830174	0.227	0.061	7.6E-184	Blood_Tre	GZMM
CMTM74	3.7E-186	1.146631	0.225	0.066	5.9E-182	Blood_Tre	CMTM7
DUSP18	5.5E-185	0.89495	0.761	0.558	8.8E-181	Blood_Tre	DUSP1
RPS127	1.5E-184	0.527224	0.995	0.858	2.4E-180	Blood_Tre	RPS12
FAU10	6.8E-184	0.545645	0.969	0.853	1.1E-179	Blood_Tre	FAU
RASAL31	2.3E-183	0.818001	0.138	0.027	3.7E-179	Blood_Tre	RASAL3
PSMB102	1.9E-182	1.187052	0.31	0.117	3.1E-178	Blood_Tre	PSMB10
ARPC29	5.6E-180	1.056591	0.695	0.572	8.9E-176	Blood_Tre	ARPC2
RPS267	8.8E-180	0.626556	0.9	0.739	1.4E-175	Blood_Tre	RPS26
CD692	6.2E-179	0.974671	0.231	0.068	1E-174	Blood_Tre	CD69
EIF3L4	5.1E-177	1.224723	0.568	0.384	8.1E-173	Blood_Tre	EIF3L
CCDC109B	2.4E-175	1.186459	0.271	0.095	3.8E-171	Blood_Tre	CCDC109B
GRK61	9.8E-175	1.187704	0.236	0.075	1.6E-170	Blood_Tre	GRK6
PRR132	8.1E-174	1.322047	0.499	0.302	1.3E-169	Blood_Tre	PRR13
APRT8	2.5E-173	1.204251	0.558	0.365	4E-169	Blood_Tre	APRT
SSR25	6.2E-173	1.201613	0.598	0.425	9.9E-169	Blood_Tre	SSR2
YBX19	4.7E-172	0.665079	0.768	0.585	7.5E-168	Blood_Tre	YBX1
DEF61	1.3E-171	1.081458	0.208	0.061	2E-167	Blood_Tre	DEF6
PLP210	2.1E-171	1.370244	0.434	0.227	3.4E-167	Blood_Tre	PLP2
P2RY8	9.4E-169	0.590512	0.086	0.012	1.5E-164	Blood_Tre	P2RY8
RASGRP23	1.6E-166	0.96686	0.207	0.06	2.6E-162	Blood_Tre	RASGRP2
CD996	3.8E-166	1.044018	0.654	0.489	6.1E-162	Blood_Tre	CD99
IFITM14	3.9E-166	1.051043	0.367	0.155	6.2E-162	Blood_Tre	IFITM1
LY6E5	1.1E-164	1.126339	0.539	0.327	1.7E-160	Blood_Tre	LY6E
PABPC11	7.5E-160	0.665481	0.822	0.719	1.2E-155	Blood_Tre	PABPC1
HCST5	4.9E-159	0.731318	0.399	0.168	7.8E-155	Blood_Tre	HCST

SELPLG4	1.8E-158	0.802703	0.135	0.03	2.9E-154	Blood_Tre	SELPLG
RNF1664	3.8E-158	0.989528	0.174	0.047	6E-154	Blood_Tre	RNF166
FERMT35	2.4E-157	0.940347	0.174	0.047	3.8E-153	Blood_Tre	FERMT3
C19orf432	5.5E-157	1.154594	0.615	0.501	8.8E-153	Blood_Tre	C19orf43
CD282	1.9E-156	0.525221	0.069	0.009	3E-152	Blood_Tre	CD28
CCND3	2.3E-155	1.165952	0.312	0.129	3.7E-151	Blood_Tre	CCND3
RPL36A7	3.8E-154	0.778631	0.714	0.506	6E-150	Blood_Tre	RPL36A
PIM23	3.3E-152	0.895899	0.155	0.039	5.3E-148	Blood_Tre	PIM2
ALOX5AP4	2.6E-151	0.998227	0.251	0.087	4.1E-147	Blood_Tre	ALOX5AP
OAZ17	3E-150	0.661716	0.847	0.782	4.7E-146	Blood_Tre	OAZ1
TMEM204	2.7E-148	0.844372	0.152	0.038	4.3E-144	Blood_Tre	TMEM204
PPP1CA5	8E-148	1.351402	0.48	0.31	1.3E-143	Blood_Tre	PPP1CA
MAP4K13	2E-146	0.648484	0.092	0.016	3.2E-142	Blood_Tre	MAP4K1
ANXA2R1	3.4E-145	0.800096	0.142	0.034	5.4E-141	Blood_Tre	ANXA2R
RGL41	1E-144	0.70632	0.107	0.021	1.6E-140	Blood_Tre	RGL4
LINC00426	3.9E-144	0.331369	0.034	0.002	6.2E-140	Blood_Tre	LINC00426
CNN22	1.2E-142	1.311912	0.35	0.171	2E-138	Blood_Tre	CNN2
EIF4A110	2.2E-141	0.914997	0.565	0.349	3.5E-137	Blood_Tre	EIF4A1
RPS278	5.7E-140	0.395644	0.999	0.892	9.2E-136	Blood_Tre	RPS27
IFITM29	1.4E-137	0.948447	0.515	0.301	2.3E-133	Blood_Tre	IFITM2
CXCR33	2.8E-137	0.781429	0.096	0.018	4.4E-133	Blood_Tre	CXCR3
RPL357	8.9E-137	0.474412	0.964	0.833	1.4E-132	Blood_Tre	RPL35
TXNIP5	4.7E-134	0.992443	0.562	0.373	7.5E-130	Blood_Tre	TXNIP
FAM159A	5.9E-134	0.580322	0.083	0.014	9.4E-130	Blood_Tre	FAM159A
VAMP23	8.2E-134	1.201595	0.498	0.334	1.3E-129	Blood_Tre	VAMP2
UBASH3A1	2.2E-132	0.434905	0.058	0.007	3.5E-128	Blood_Tre	UBASH3A
AQP33	7.3E-132	1.132288	0.369	0.189	1.2E-127	Blood_Tre	AQP3
RPS27A9	8.6E-132	0.473826	0.982	0.846	1.4E-127	Blood_Tre	RPS27A
TRAPPC14	4.9E-131	1.232501	0.406	0.233	7.9E-127	Blood_Tre	TRAPPC1
PSMB97	4.3E-130	1.110114	0.343	0.166	6.9E-126	Blood_Tre	PSMB9
IL164	8.7E-128	0.889124	0.17	0.051	1.4E-123	Blood_Tre	IL16
RNASET25	5.2E-126	1.175273	0.409	0.233	8.2E-122	Blood_Tre	RNASET2
RPS139	5.3E-121	0.449711	0.96	0.814	8.4E-117	Blood_Tre	RPS13
SUN21	1.5E-120	1.119073	0.277	0.122	2.4E-116	Blood_Tre	SUN2
SRGN7	9.7E-118	0.370723	0.438	0.219	1.5E-113	Blood_Tre	SRGN
JAKMIP1	2.1E-117	0.334292	0.033	0.003	3.3E-113	Blood_Tre	JAKMIP1
SNX203	6.3E-116	0.523038	0.068	0.011	1E-111	Blood_Tre	SNX20
HNRNPDL	1.5E-115	1.000128	0.5	0.333	2.4E-111	Blood_Tre	HNRNPDL
UBC1	3.1E-115	0.334995	0.94	0.84	4.9E-111	Blood_Tre	UBC
ATP5G26	1.8E-114	0.759616	0.67	0.571	2.8E-110	Blood_Tre	ATP5G2
MYO1F4	3E-113	0.774629	0.145	0.042	4.7E-109	Blood_Tre	MYO1F
ERP293	1.7E-112	1.061485	0.497	0.352	2.7E-108	Blood_Tre	ERP29
ARRB24	8.2E-112	0.941382	0.219	0.084	1.3E-107	Blood_Tre	ARRB2
SH2D2A2	1.7E-107	0.510765	0.072	0.013	2.8E-103	Blood_Tre	SH2D2A
EIF3G2	2.2E-107	1.017276	0.527	0.397	3.5E-103	Blood_Tre	EIF3G
RHOG5	2.3E-107	1.19962	0.336	0.183	3.7E-103	Blood_Tre	RHOG
ENO17	7.6E-107	1.034807	0.534	0.41	1.2E-102	Blood_Tre	ENO1

HNRNPA2E	1.4E-106	0.924628	0.6	0.49	2.2E-102	Blood_Tre	HNRNPA2B1
ATP5D5	1.6E-106	0.940017	0.558	0.445	2.5E-102	Blood_Tre	ATP5D
SASH33	6.8E-106	0.642036	0.095	0.021	1.1E-101	Blood_Tre	SASH3
UBXN15	8.9E-106	1.071192	0.511	0.389	1.4E-101	Blood_Tre	UBXN1
CLIC111	3.5E-105	0.899562	0.602	0.473	5.6E-101	Blood_Tre	CLIC1
SEPT92	5E-104	1.157321	0.31	0.161	8E-100	Blood_Tre	SEPT9
KLF28	7.3E-104	0.822641	0.36	0.188	1.2E-99	Blood_Tre	KLF2
PSME23	2.4E-102	1.123197	0.433	0.285	3.81E-98	Blood_Tre	PSME2
LY92	4.4E-102	0.552828	0.088	0.019	6.96E-98	Blood_Tre	LY9
RPL366	1.3E-100	0.36898	0.961	0.838	2.11E-96	Blood_Tre	RPL36
PYHIN11	1.6E-100	0.489564	0.069	0.012	2.57E-96	Blood_Tre	PYHIN1
SYTL13	4.1E-100	1.126286	0.243	0.11	6.54E-96	Blood_Tre	SYTL1
SH2D1A2	2.01E-98	0.476756	0.07	0.013	3.21E-94	Blood_Tre	SH2D1A
CAPZB1	2.9E-97	0.925056	0.556	0.46	4.63E-93	Blood_Tre	CAPZB
RPL277	3.67E-96	0.431179	0.875	0.748	5.85E-92	Blood_Tre	RPL27
RPS208	7.64E-96	0.407619	0.914	0.793	1.22E-91	Blood_Tre	RPS20
RARRES31	8.98E-96	0.686266	0.387	0.217	1.43E-91	Blood_Tre	RARRES3
GSDMD3	3.14E-95	1.02761	0.224	0.097	5E-91	Blood_Tre	GSDMD
RP11.51J9.	8.05E-95	0.824406	0.147	0.048	1.28E-90	Blood_Tre	RP11.51J9.5
EIF4A27	1.95E-94	0.901029	0.573	0.484	3.11E-90	Blood_Tre	EIF4A2
IL27RA1	2.63E-93	0.71677	0.124	0.037	4.2E-89	Blood_Tre	IL27RA
C9orf1423	3.77E-93	1.122032	0.282	0.143	6.01E-89	Blood_Tre	C9orf142
PSMA79	2.22E-92	0.939398	0.548	0.463	3.53E-88	Blood_Tre	PSMA7
CALM110	2.71E-91	0.677892	0.702	0.65	4.32E-87	Blood_Tre	CALM1
PBXIP13	3.35E-90	1.137147	0.239	0.112	5.34E-86	Blood_Tre	PBXIP1
C9orf783	7.01E-90	1.13245	0.308	0.171	1.12E-85	Blood_Tre	C9orf78
CIRBP9	9.52E-90	0.790811	0.627	0.544	1.52E-85	Blood_Tre	CIRBP
FOS14	1.57E-89	0.506726	0.605	0.421	2.51E-85	Blood_Tre	FOS
DENND1C	3.14E-89	0.432161	0.068	0.013	5.01E-85	Blood_Tre	DENND1C
TUBA4A3	4.2E-89	1.161917	0.305	0.166	6.71E-85	Blood_Tre	TUBA4A
EIF3H1	8.82E-89	0.987984	0.531	0.454	1.41E-84	Blood_Tre	EIF3H
GRAP22	1.1E-88	0.524657	0.072	0.015	1.75E-84	Blood_Tre	GRAP2
NINJ2	1.36E-87	0.721287	0.091	0.023	2.17E-83	Blood_Tre	NINJ2
CMTM36	1.83E-87	0.918851	0.184	0.074	2.92E-83	Blood_Tre	CMTM3
RHOF3	1.29E-86	0.958317	0.193	0.08	2.06E-82	Blood_Tre	RHOF
RNF1262	3.26E-86	1.126006	0.268	0.14	5.19E-82	Blood_Tre	RNF126
TTC39C2	2.8E-85	1.144592	0.217	0.099	4.47E-81	Blood_Tre	TTC39C
GBP51	1.23E-84	0.415848	0.049	0.008	1.96E-80	Blood_Tre	GBP5
PKM8	1.01E-83	0.946932	0.486	0.371	1.6E-79	Blood_Tre	PKM
GCSAM1	2.86E-80	0.309365	0.036	0.005	4.56E-76	Blood_Tre	GCSAM
GIMAP2	4.02E-79	0.641746	0.109	0.032	6.41E-75	Blood_Tre	GIMAP2
SIGIRR1	5.28E-79	1.023155	0.256	0.131	8.43E-75	Blood_Tre	SIGIRR
CUTA4	7.18E-79	0.936639	0.48	0.374	1.15E-74	Blood_Tre	CUTA
SLA21	7.25E-79	0.350727	0.046	0.007	1.16E-74	Blood_Tre	SLA2
CTC.479C5	9.46E-79	0.421606	0.064	0.013	1.51E-74	Blood_Tre	CTC.479C5.12
ARHGAP45	1.58E-78	0.608672	0.108	0.032	2.53E-74	Blood_Tre	ARHGAP4
PASK	1.14E-77	0.631883	0.09	0.024	1.81E-73	Blood_Tre	PASK

PTPN72	1.6E-77	0.596351	0.1	0.029	2.55E-73	Blood_Tre	PTPN7
ARL6IP44	7.82E-77	0.955856	0.502	0.42	1.25E-72	Blood_Tre	ARL6IP4
RPL3011	1.61E-76	0.309967	0.968	0.842	2.57E-72	Blood_Tre	RPL30
SHISA53	3.41E-76	1.010901	0.215	0.101	5.44E-72	Blood_Tre	SHISA5
RGS143	4.09E-76	0.779039	0.129	0.044	6.52E-72	Blood_Tre	RGS14
LTA	7.88E-76	0.295032	0.04	0.006	1.26E-71	Blood_Tre	LTA
STMN3	1.79E-75	0.756773	0.137	0.049	2.85E-71	Blood_Tre	STMN3
EIF3D4	8.46E-74	1.09879	0.394	0.282	1.35E-69	Blood_Tre	EIF3D
ARHGAP91	1.11E-73	0.746758	0.134	0.048	1.78E-69	Blood_Tre	ARHGAP9
CSK3	6.66E-72	0.862021	0.174	0.073	1.06E-67	Blood_Tre	CSK
GATA32	8.04E-72	1.045486	0.183	0.082	1.28E-67	Blood_Tre	GATA3
DUSP29	9.95E-72	0.808208	0.248	0.123	1.59E-67	Blood_Tre	DUSP2
SLA4	5.81E-71	0.596451	0.086	0.024	9.27E-67	Blood_Tre	SLA
F8A11	1.15E-69	0.728901	0.132	0.048	1.83E-65	Blood_Tre	F8A1
TESPA11	1.5E-69	0.404369	0.059	0.013	2.39E-65	Blood_Tre	TESPA1
ITGB2.AS1	1.81E-69	0.452541	0.067	0.016	2.88E-65	Blood_Tre	ITGB2.AS1
CRIP17	1.92E-68	0.71428	0.411	0.275	3.06E-64	Blood_Tre	CRIP1
ADAM85	1.99E-68	0.63309	0.093	0.028	3.18E-64	Blood_Tre	ADAM8
SRSF51	1.67E-67	0.813809	0.551	0.486	2.66E-63	Blood_Tre	SRSF5
ARPC33	2.1E-67	0.626977	0.654	0.595	3.35E-63	Blood_Tre	ARPC3
RPS2312	2.11E-67	0.27798	0.975	0.81	3.36E-63	Blood_Tre	RPS23
PTPRC4	3.06E-66	0.662125	0.221	0.105	4.88E-62	Blood_Tre	PTPRC
TBCB7	7.79E-66	1.040068	0.314	0.2	1.24E-61	Blood_Tre	TBCB
TAGAP4	1.55E-65	0.585338	0.093	0.028	2.48E-61	Blood_Tre	TAGAP
SH3BP12	1.67E-65	0.763207	0.137	0.053	2.66E-61	Blood_Tre	SH3BP1
MEI11	1.68E-65	0.666891	0.115	0.04	2.68E-61	Blood_Tre	MEI1
PFDN58	1.71E-65	0.412083	0.815	0.725	2.72E-61	Blood_Tre	PFDN5
GLRX4	1.06E-64	0.94778	0.329	0.211	1.69E-60	Blood_Tre	GLRX
CORO75	1.11E-64	0.660678	0.103	0.034	1.77E-60	Blood_Tre	CORO7
FMNL17	1.36E-64	0.724848	0.152	0.062	2.17E-60	Blood_Tre	FMNL1
TMC81	1.5E-64	0.586711	0.094	0.029	2.39E-60	Blood_Tre	TMC8
HNRNPA09	5.71E-64	0.930745	0.433	0.333	9.11E-60	Blood_Tre	HNRNPA0
BATF2	1.36E-63	0.741737	0.118	0.042	2.17E-59	Blood_Tre	BATF
TRAPPC6A	1.16E-62	0.959625	0.25	0.14	1.85E-58	Blood_Tre	TRAPPC6A
TPM39	1.28E-62	0.97244	0.422	0.33	2.04E-58	Blood_Tre	TPM3
CHCHD26	1.67E-62	0.598687	0.642	0.621	2.67E-58	Blood_Tre	CHCHD2
PSMB85	3.05E-62	1.006498	0.302	0.189	4.86E-58	Blood_Tre	PSMB8
RP3.395M1	1.27E-61	0.474374	0.068	0.017	2.03E-57	Blood_Tre	RP3.395M20.12
CORO1B2	3.84E-61	1.167886	0.308	0.204	6.12E-57	Blood_Tre	CORO1B
EDF15	9.54E-61	0.642787	0.622	0.625	1.52E-56	Blood_Tre	EDF1
PSTPIP15	1.28E-59	0.499014	0.079	0.023	2.05E-55	Blood_Tre	PSTPIP1
PIK3IP1	1.46E-59	0.988052	0.287	0.177	2.32E-55	Blood_Tre	PIK3IP1
OCIAD2	2.35E-59	0.936581	0.272	0.161	3.75E-55	Blood_Tre	OCIAD2
TMEM173	2.93E-59	0.881698	0.21	0.109	4.67E-55	Blood_Tre	TMEM173
UBE2L62	4.09E-59	0.971857	0.224	0.121	6.52E-55	Blood_Tre	UBE2L6
AIP4	1.9E-58	0.95477	0.231	0.127	3.03E-54	Blood_Tre	AIP
CCDC85B7	4.53E-58	0.936054	0.376	0.27	7.23E-54	Blood_Tre	CCDC85B

TNFRSF142	5.44E-58	0.962083	0.216	0.115	8.68E-54	Blood_Treg	TNFRSF14
TAP14	1.73E-57	0.934203	0.188	0.094	2.77E-53	Blood_Treg	TAP1
TWF22	5.9E-57	0.993721	0.221	0.121	9.41E-53	Blood_Treg	TWF2
SOD19	7.86E-57	0.770001	0.493	0.414	1.25E-52	Blood_Treg	SOD1
FAM65B	1.24E-56	0.583479	0.124	0.048	1.98E-52	Blood_Treg	FAM65B
ABHD14B1	1.45E-56	0.979165	0.217	0.118	2.31E-52	Blood_Treg	ABHD14B
PRAF2	1.85E-56	0.749309	0.128	0.052	2.96E-52	Blood_Treg	PRAF2
CHMP4A3	2.13E-56	1.00178	0.268	0.163	3.4E-52	Blood_Treg	CHMP4A
TNFAIP87	2.99E-56	0.807909	0.201	0.103	4.77E-52	Blood_Treg	TNFAIP8
FGD31	5.31E-56	0.561443	0.091	0.03	8.46E-52	Blood_Treg	FGD3
CASP13	1.34E-55	0.742696	0.132	0.055	2.14E-51	Blood_Treg	CASP1
CD962	1.71E-55	0.438299	0.071	0.02	2.72E-51	Blood_Treg	CD96
C10orf544	5.39E-55	0.89235	0.276	0.169	8.6E-51	Blood_Treg	C10orf54
ZFP36L28	1.5E-53	0.599059	0.504	0.39	2.39E-49	Blood_Treg	ZFP36L2
RAB371	6.53E-53	0.39585	0.055	0.013	1.04E-48	Blood_Treg	RAB37
RPL2414	2.87E-52	0.319196	0.878	0.801	4.57E-48	Blood_Treg	RPL24
ITM2B8	9.39E-52	0.374472	0.764	0.705	1.5E-47	Blood_Treg	ITM2B
HNRNPM6	9.44E-52	0.986724	0.312	0.215	1.51E-47	Blood_Treg	HNRNPM
CTD.2035E	5.76E-51	0.398433	0.061	0.016	9.19E-47	Blood_Treg	CTD.2035E11.3
LCP16	8.1E-51	0.759636	0.201	0.106	1.29E-46	Blood_Treg	LCP1
ICOS2	3.49E-50	0.25536	0.031	0.005	5.56E-46	Blood_Treg	ICOS
FXYD71	3.6E-50	0.38562	0.044	0.01	5.74E-46	Blood_Treg	FXYD7
HLA.F4	6.71E-50	0.788805	0.215	0.118	1.07E-45	Blood_Treg	HLA.F
NDUFB93	1.47E-49	0.8554	0.419	0.345	2.35E-45	Blood_Treg	NDUFB9
FLI14	6.32E-49	0.592908	0.102	0.039	1.01E-44	Blood_Treg	FLI1
CD43	1.08E-48	0.515264	0.087	0.03	1.73E-44	Blood_Treg	CD4
PRDX210	1.52E-48	0.883643	0.389	0.31	2.43E-44	Blood_Treg	PRDX2
BLOC1S41	4.64E-48	0.855094	0.17	0.086	7.39E-44	Blood_Treg	BLOC1S4
ITK2	4.17E-47	0.370926	0.051	0.013	6.64E-43	Blood_Treg	ITK
SLAMF13	1.11E-46	0.351143	0.049	0.012	1.77E-42	Blood_Treg	SLAMF1
DCK4	1.93E-46	0.723207	0.131	0.059	3.08E-42	Blood_Treg	DCK
MYL12B2	2.01E-46	0.571241	0.607	0.607	3.21E-42	Blood_Treg	MYL12B
IL2RB2	3.03E-46	0.342011	0.056	0.015	4.83E-42	Blood_Treg	IL2RB
BTN3A23	3.26E-46	0.660471	0.131	0.058	5.21E-42	Blood_Treg	BTN3A2
HIGD2A4	4.12E-46	0.856446	0.417	0.351	6.57E-42	Blood_Treg	HIGD2A
UBB6	1.14E-45	0.311627	0.768	0.729	1.81E-41	Blood_Treg	UBB
BTG210	1.34E-45	0.8785	0.329	0.231	2.14E-41	Blood_Treg	BTG2
KCNA31	1.76E-45	0.268512	0.031	0.006	2.81E-41	Blood_Treg	KCNA3
SLAMF6	4.54E-45	0.296612	0.037	0.008	7.25E-41	Blood_Treg	SLAMF6
ARHGAP3C	6.31E-45	0.566406	0.115	0.048	1.01E-40	Blood_Treg	ARHGAP30
PCED1B1	2.18E-44	0.344036	0.055	0.015	3.47E-40	Blood_Treg	PCED1B
SEPT64	3.28E-44	0.785446	0.176	0.093	5.23E-40	Blood_Treg	SEPT6
ECH13	4.08E-44	0.948616	0.305	0.219	6.51E-40	Blood_Treg	ECH1
LEPROTL12	1.35E-43	0.930061	0.258	0.168	2.15E-39	Blood_Treg	LEPROTL1
FKBP84	2.01E-43	0.846915	0.401	0.338	3.2E-39	Blood_Treg	FKBP8
ZNF775	4.22E-43	0.421141	0.059	0.018	6.73E-39	Blood_Treg	ZNF775
SHKBP11	7.38E-43	0.805	0.156	0.079	1.18E-38	Blood_Treg	SHKBP1

ARHGAP25	9.09E-43	0.455702	0.071	0.024	1.45E-38	Blood_Treg	ARHGAP25
XXbac.BPG	5.91E-42	0.426047	0.058	0.017	9.43E-38	Blood_Treg	XXbac.BPG299F13.17
C6orf486	4.66E-41	0.723692	0.398	0.311	7.43E-37	Blood_Treg	C6orf48
GRAP	1.99E-40	0.28836	0.044	0.011	3.17E-36	Blood_Treg	GRAP
RCAN33	2.33E-40	0.766611	0.131	0.063	3.71E-36	Blood_Treg	RCAN3
SF3B54	3.4E-40	0.821133	0.414	0.362	5.43E-36	Blood_Treg	SF3B5
CCR71	4.36E-40	0.421087	0.107	0.044	6.96E-36	Blood_Treg	CCR7
TRPV23	7.15E-40	0.418855	0.067	0.022	1.14E-35	Blood_Treg	TRPV2
GZMK2	8.08E-40	0.58033	0.05	0.014	1.29E-35	Blood_Treg	GZMK
RBM312	1.37E-39	0.727395	0.478	0.439	2.19E-35	Blood_Treg	RBM3
ATP6V0E2	3.47E-39	0.812081	0.147	0.076	5.54E-35	Blood_Treg	ATP6V0E2
PCBP26	4.95E-39	0.804309	0.454	0.42	7.89E-35	Blood_Treg	PCBP2
SPN3	1.86E-38	0.411658	0.068	0.023	2.96E-34	Blood_Treg	SPN
TMC6	3.44E-38	0.80674	0.178	0.101	5.49E-34	Blood_Treg	TMC6
HINT113	3.52E-38	0.477178	0.607	0.57	5.62E-34	Blood_Treg	HINT1
JTB8	3.99E-38	0.760682	0.419	0.37	6.36E-34	Blood_Treg	JTB
RNF113A	1.63E-37	0.710078	0.124	0.059	2.6E-33	Blood_Treg	RNF113A
CYTIP4	2.05E-37	0.642768	0.158	0.083	3.27E-33	Blood_Treg	CYTIP
CTD.3184A	2.28E-37	0.493746	0.083	0.032	3.63E-33	Blood_Treg	CTD.3184A7.4
SAMD31	6.8E-37	0.380142	0.063	0.021	1.08E-32	Blood_Treg	SAMD3
C19orf661	8.04E-37	0.726217	0.153	0.082	1.28E-32	Blood_Treg	C19orf66
CDKN2D2	9.1E-37	0.71625	0.154	0.082	1.45E-32	Blood_Treg	CDKN2D
TBCC3	9.75E-36	0.875306	0.167	0.095	1.56E-31	Blood_Treg	TBCC
ANP32B2	2.12E-35	0.893624	0.364	0.304	3.37E-31	Blood_Treg	ANP32B
KLF612	3.13E-35	0.624834	0.461	0.401	4.99E-31	Blood_Treg	KLF6
ATP5A17	7.29E-35	0.856509	0.369	0.312	1.16E-30	Blood_Treg	ATP5A1
NAP1L18	7.65E-35	0.735753	0.446	0.401	1.22E-30	Blood_Treg	NAP1L1
APBB1IP3	1.39E-34	0.490253	0.094	0.04	2.22E-30	Blood_Treg	APBB1IP
C1orf1624	2.66E-34	0.64611	0.164	0.09	4.24E-30	Blood_Treg	C1orf162
TSC22D41	3.88E-34	0.924237	0.27	0.197	6.19E-30	Blood_Treg	TSC22D4
TGFB16	4.67E-34	0.679479	0.171	0.097	7.45E-30	Blood_Treg	TGFB1
SNRPD24	1.42E-33	0.622498	0.476	0.433	2.26E-29	Blood_Treg	SNRPD2
GIMAP64	4.55E-33	0.463585	0.076	0.03	7.26E-29	Blood_Treg	GIMAP6
ZBP11	9.33E-33	0.294129	0.042	0.012	1.49E-28	Blood_Treg	ZBP1
EVI2B6	1.13E-32	0.560622	0.126	0.063	1.8E-28	Blood_Treg	EVI2B
CCM22	1.43E-32	0.759342	0.152	0.085	2.27E-28	Blood_Treg	CCM2
NECAP2	1.64E-32	0.89055	0.206	0.134	2.61E-28	Blood_Treg	NECAP2
LCP23	4.17E-32	0.420888	0.069	0.026	6.65E-28	Blood_Treg	LCP2
PARK714	7.29E-32	0.740922	0.402	0.355	1.16E-27	Blood_Treg	PARK7
POLD42	1.62E-31	0.809556	0.29	0.219	2.59E-27	Blood_Treg	POLD4
PTPN65	1.97E-31	0.612698	0.14	0.075	3.13E-27	Blood_Treg	PTPN6
WIPF16	4.82E-31	0.674736	0.159	0.091	7.7E-27	Blood_Treg	WIPF1
LINC00649	8.67E-31	0.424501	0.058	0.021	1.38E-26	Blood_Treg	LINC00649
ANAPC164	8.68E-31	0.767688	0.406	0.371	1.38E-26	Blood_Treg	ANAPC16
ABTB11	1.29E-30	0.660371	0.124	0.065	2.05E-26	Blood_Treg	ABTB1
RNU121	1.34E-30	0.385104	0.053	0.018	2.13E-26	Blood_Treg	RNU12
RPL36AL4	1.85E-30	0.374492	0.698	0.731	2.95E-26	Blood_Treg	RPL36AL

PRR52	2.54E-30	0.583501	0.094	0.043	4.05E-26	Blood_Tre|	PRR5
UBE2D22	3.24E-30	0.82579	0.382	0.344	5.17E-26	Blood_Tre|	UBE2D2
CCDC1079	1.51E-29	0.893837	0.243	0.175	2.4E-25	Blood_Tre|	CCDC107
PEX162	1.64E-29	0.740156	0.156	0.091	2.62E-25	Blood_Tre|	PEX16
GBP22	1.89E-29	0.880509	0.175	0.109	3.01E-25	Blood_Tre|	GBP2
ATP5EP22	3.18E-29	0.431058	0.11	0.054	5.08E-25	Blood_Tre|	ATP5EP2
KLRG12	4.09E-29	0.418925	0.062	0.024	6.53E-25	Blood_Tre|	KLRG1
POLR3GL1	4.52E-29	0.798724	0.196	0.127	7.21E-25	Blood_Tre|	POLR3GL
RNF167	6.35E-29	0.905989	0.212	0.146	1.01E-24	Blood_Tre|	RNF167
LDLRAP11	9.27E-29	0.625267	0.117	0.061	1.48E-24	Blood_Tre|	LDLRAP1
GALT1	1.25E-28	0.599543	0.093	0.044	1.99E-24	Blood_Tre|	GALT
GZMA2	2E-28	0.310789	0.089	0.039	3.19E-24	Blood_Tre|	GZMA
TNFAIP8L2	2.27E-28	0.280437	0.044	0.014	3.62E-24	Blood_Tre|	TNFAIP8L2
COPE3	5.54E-28	0.759867	0.439	0.441	8.83E-24	Blood_Tre|	COPE
NCF44	7.63E-28	0.529761	0.089	0.042	1.22E-23	Blood_Tre|	NCF4
SH3KBP15	5.02E-27	0.668346	0.148	0.087	8.01E-23	Blood_Tre|	SH3KBP1
OXA1L2	5.18E-27	0.867869	0.214	0.149	8.26E-23	Blood_Tre|	OXA1L
IKZF16	1.45E-26	0.469329	0.085	0.039	2.32E-22	Blood_Tre|	IKZF1
TUBA1A9	3.43E-26	0.700506	0.303	0.234	5.47E-22	Blood_Tre|	TUBA1A
ALDOA2	5.98E-26	0.358136	0.628	0.643	9.54E-22	Blood_Tre|	ALDOA
SLC9A3R1:	7.76E-26	0.906117	0.215	0.153	1.24E-21	Blood_Tre|	SLC9A3R1
TNFRSF18‹	1.55E-25	0.556385	0.095	0.047	2.47E-21	Blood_Tre|	TNFRSF18
AC010642.	2.64E-25	0.472698	0.079	0.036	4.2E-21	Blood_Tre|	AC010642.1
DPEP23	3.35E-25	0.355945	0.055	0.021	5.35E-21	Blood_Tre|	DPEP2
YWHAB8	4.1E-25	0.765046	0.42	0.413	6.54E-21	Blood_Tre|	YWHAB
C21orf333	4.14E-25	0.602036	0.105	0.055	6.61E-21	Blood_Tre|	C21orf33
LDHA10	4.23E-25	0.465443	0.48	0.44	6.74E-21	Blood_Tre|	LDHA
ADA1	4.93E-25	0.415893	0.078	0.035	7.86E-21	Blood_Tre|	ADA
TSPO8	5.88E-25	0.447668	0.477	0.466	9.38E-21	Blood_Tre|	TSPO
PGK11	6.51E-25	0.801297	0.379	0.354	1.04E-20	Blood_Tre|	PGK1
KCNAB24	7.59E-25	0.393843	0.06	0.024	1.21E-20	Blood_Tre|	KCNAB2
ILF3.AS11	8.75E-25	0.739319	0.166	0.105	1.4E-20	Blood_Tre|	ILF3.AS1
ORAI12	1.26E-24	0.733903	0.145	0.088	2.01E-20	Blood_Tre|	ORAI1
COMMD67	1.78E-24	0.501029	0.502	0.474	2.84E-20	Blood_Tre|	COMMD6
RGS108	1.79E-24	0.729693	0.276	0.215	2.86E-20	Blood_Tre|	RGS10
CISH	2.51E-24	0.533906	0.081	0.038	4E-20	Blood_Tre|	CISH
THEM41	3.64E-24	0.612982	0.102	0.053	5.81E-20	Blood_Tre|	THEM4
PSMC51	4.3E-24	0.803451	0.309	0.259	6.85E-20	Blood_Tre|	PSMC5
TRABD4	4.94E-24	0.794648	0.176	0.116	7.88E-20	Blood_Tre|	TRABD
TCF72	9.61E-24	0.45033	0.095	0.047	1.53E-19	Blood_Tre|	TCF7
APOL34	2.47E-23	0.407887	0.069	0.03	3.94E-19	Blood_Tre|	APOL3
PPIA7	2.9E-23	0.485232	0.56	0.591	4.63E-19	Blood_Tre|	PPIA
CTSA5	5.11E-23	0.920282	0.188	0.131	8.14E-19	Blood_Tre|	CTSA
FBL5	5.45E-23	0.72468	0.292	0.235	8.69E-19	Blood_Tre|	FBL
SNRPB8	5.91E-23	0.775201	0.362	0.333	9.43E-19	Blood_Tre|	SNRPB
LBH3	6.99E-23	0.582921	0.154	0.095	1.12E-18	Blood_Tre|	LBH
RP11.796E	7.53E-23	0.473537	0.079	0.037	1.2E-18	Blood_Tre|	RP11.796E2.4

RASA3	8.04E-23	0.394422	0.057	0.024	1.28E-18	Blood_Tre	RASA3
ARF54	1.07E-22	0.819011	0.272	0.222	1.71E-18	Blood_Tre	ARF5
EID110	1.21E-22	0.610972	0.397	0.357	1.93E-18	Blood_Tre	EID1
RBMX7	1.25E-22	0.813677	0.253	0.198	1.99E-18	Blood_Tre	RBMX
C17orf625	1.73E-22	0.698409	0.145	0.09	2.76E-18	Blood_Tre	C17orf62
CPA5	1.97E-22	0.382085	0.057	0.023	3.14E-18	Blood_Tre	CPA5
BUB33	2.51E-22	0.854019	0.229	0.174	4E-18	Blood_Tre	BUB3
FAM118A2	3.19E-22	0.653663	0.124	0.072	5.08E-18	Blood_Tre	FAM118A
MAD1L1	3.63E-22	0.623687	0.136	0.082	5.79E-18	Blood_Tre	MAD1L1
C17orf492	4.69E-22	0.350144	0.061	0.026	7.49E-18	Blood_Tre	C17orf49
RNF214	7.33E-22	0.656136	0.089	0.046	1.17E-17	Blood_Tre	RNF214
CCNI10	7.67E-22	0.54197	0.505	0.519	1.22E-17	Blood_Tre	CCNI
SSBP47	2.04E-21	0.663368	0.157	0.101	3.25E-17	Blood_Tre	SSBP4
C12orf753	2.05E-21	0.562025	0.198	0.136	3.26E-17	Blood_Tre	C12orf75
LINC00936	5.24E-21	0.496148	0.126	0.074	8.35E-17	Blood_Tre	LINC00936
PTGER43	6.72E-21	0.559446	0.094	0.05	1.07E-16	Blood_Tre	PTGER4
DDIT44	9.83E-21	0.674784	0.299	0.242	1.57E-16	Blood_Tre	DDIT4
CYTH45	4.38E-20	0.334576	0.065	0.03	6.99E-16	Blood_Tre	CYTH4
LPXN4	4.42E-20	0.487001	0.088	0.046	7.05E-16	Blood_Tre	LPXN
IKBKE	5.26E-20	0.321046	0.044	0.017	8.39E-16	Blood_Tre	IKBKE
TAZ	7.17E-20	0.451739	0.071	0.035	1.14E-15	Blood_Tre	TAZ
NAA106	2.3E-19	0.84578	0.209	0.159	3.67E-15	Blood_Tre	NAA10
DRAP16	6.23E-19	0.664024	0.361	0.335	9.93E-15	Blood_Tre	DRAP1
MTHFS1	7.01E-19	0.423754	0.068	0.033	1.12E-14	Blood_Tre	MTHFS
CCDC1671	1.46E-18	0.823517	0.188	0.139	2.32E-14	Blood_Tre	CCDC167
EGLN21	3.54E-18	0.667389	0.172	0.121	5.64E-14	Blood_Tre	EGLN2
ARL6IP58	6.81E-18	0.674207	0.341	0.31	1.09E-13	Blood_Tre	ARL6IP5
TPI17	8.1E-18	0.540511	0.496	0.536	1.29E-13	Blood_Tre	TPI1
CHST111	9.69E-18	0.385535	0.056	0.026	1.55E-13	Blood_Tre	CHST11
LAG32	1.06E-17	0.292707	0.033	0.012	1.68E-13	Blood_Tre	LAG3
TNFRSF1B7	1.17E-17	0.453943	0.087	0.048	1.87E-13	Blood_Tre	TNFRSF1B
SP1101	4.54E-17	0.51404	0.107	0.064	7.24E-13	Blood_Tre	SP110
CIB14	4.61E-17	0.649312	0.368	0.351	7.36E-13	Blood_Tre	CIB1
C19orf608	5.22E-17	0.697321	0.27	0.229	8.33E-13	Blood_Tre	C19orf60
EMB5	5.68E-17	0.372063	0.077	0.041	9.05E-13	Blood_Tre	EMB
VAMP87	6.41E-17	0.478898	0.42	0.409	1.02E-12	Blood_Tre	VAMP8
RNASEH2B	8.97E-17	0.693361	0.151	0.104	1.43E-12	Blood_Tre	RNASEH2B
SNRPA2	1.06E-16	0.672558	0.142	0.096	1.69E-12	Blood_Tre	SNRPA
ATP5F12	1.06E-16	0.775283	0.325	0.308	1.69E-12	Blood_Tre	ATP5F1
FYN10	1.14E-16	0.469862	0.11	0.066	1.83E-12	Blood_Tre	FYN
CD3203	1.74E-16	0.671923	0.141	0.095	2.78E-12	Blood_Tre	CD320
MAT2B	1.97E-16	0.720857	0.159	0.113	3.13E-12	Blood_Tre	MAT2B
CTB.31O20	2.2E-16	0.320574	0.047	0.02	3.5E-12	Blood_Tre	CTB.31O20.2
NAAA3	2.3E-16	0.662463	0.132	0.088	3.67E-12	Blood_Tre	NAAA
HAPLN34	2.42E-16	0.421956	0.067	0.034	3.87E-12	Blood_Tre	HAPLN3
PSMD92	2.46E-16	0.686826	0.158	0.112	3.93E-12	Blood_Tre	PSMD9
PRPS12	2.62E-16	0.549527	0.101	0.06	4.18E-12	Blood_Tre	PRPS1

PPM1M1	3.38E-16	0.391265	0.064	0.032	5.39E-12	Blood_Tre	PPM1M
APOBEC3G	3.84E-16	0.404606	0.082	0.045	6.13E-12	Blood_Tre	APOBEC3G
MRPS344	6.09E-16	0.783591	0.237	0.197	9.71E-12	Blood_Tre	MRPS34
RANGRF1	6.7E-16	0.509427	0.14	0.092	1.07E-11	Blood_Tre	RANGRF
ADSL1	6.92E-16	0.623513	0.12	0.077	1.1E-11	Blood_Tre	ADSL
CALM3	1.09E-15	0.502448	0.367	0.342	1.73E-11	Blood_Tre	CALM3
RPS6KA12	1.2E-15	0.508115	0.083	0.047	1.92E-11	Blood_Tre	RPS6KA1
VPS288	1.3E-15	0.762493	0.352	0.35	2.07E-11	Blood_Tre	VPS28
R3HDM41	1.44E-15	0.741516	0.175	0.13	2.3E-11	Blood_Tre	R3HDM4
SRP149	3.28E-15	0.273314	0.65	0.703	5.23E-11	Blood_Tre	SRP14
LAMTOR46	5.2E-15	0.553351	0.393	0.39	8.3E-11	Blood_Tre	LAMTOR4
IRF33	5.22E-15	0.686739	0.142	0.099	8.32E-11	Blood_Tre	IRF3
TPGS13	6.37E-15	0.570557	0.143	0.099	1.02E-10	Blood_Tre	TPGS1
AP2M15	1.01E-14	0.78613	0.316	0.304	1.6E-10	Blood_Tre	AP2M1
LINC00324	1.05E-14	0.25285	0.035	0.014	1.68E-10	Blood_Tre	LINC00324
PARVG4	1.17E-14	0.32118	0.06	0.03	1.86E-10	Blood_Tre	PARVG
NCF15	1.17E-14	0.251807	0.075	0.041	1.86E-10	Blood_Tre	NCF1
ZFYVE282	1.6E-14	0.334762	0.046	0.021	2.55E-10	Blood_Tre	ZFYVE28
UFC11	1.96E-14	0.774567	0.346	0.348	3.12E-10	Blood_Tre	UFC1
CCDC22	2.55E-14	0.390327	0.058	0.03	4.07E-10	Blood_Tre	CCDC22
MZT2A2	4.29E-14	0.809125	0.32	0.312	6.85E-10	Blood_Tre	MZT2A
CTSC7	4.56E-14	0.506411	0.166	0.12	7.27E-10	Blood_Tre	CTSC
MVP4	6.97E-14	0.609676	0.131	0.091	1.11E-09	Blood_Tre	MVP
ACTR1B	7.56E-14	0.658156	0.113	0.075	1.21E-09	Blood_Tre	ACTR1B
SNX172	7.71E-14	0.73172	0.181	0.141	1.23E-09	Blood_Tre	SNX17
GRAMD1A	7.74E-14	0.59676	0.117	0.077	1.23E-09	Blood_Tre	GRAMD1A
CPNE21	1.08E-13	0.378782	0.052	0.026	1.72E-09	Blood_Tre	CPNE2
NCR31	1.1E-13	0.276774	0.046	0.022	1.75E-09	Blood_Tre	NCR3
ISCU1	1.11E-13	0.699106	0.356	0.354	1.77E-09	Blood_Tre	ISCU
SLC2A38	1.28E-13	0.373637	0.179	0.129	2.03E-09	Blood_Tre	SLC2A3
URM13	1.32E-13	0.63075	0.146	0.105	2.1E-09	Blood_Tre	URM1
RAB4B2	1.36E-13	0.427728	0.076	0.044	2.17E-09	Blood_Tre	RAB4B
C9orf114	1.54E-13	0.373	0.058	0.03	2.45E-09	Blood_Tre	C9orf114
HMGN11	1.72E-13	0.608153	0.421	0.454	2.74E-09	Blood_Tre	HMGN1
SAMSN13	1.83E-13	0.329011	0.079	0.045	2.91E-09	Blood_Tre	SAMSN1
RP13.1032	1.85E-13	0.295091	0.044	0.02	2.95E-09	Blood_Tre	RP13.1032I1.7
U2AF111	1.92E-13	0.635405	0.286	0.255	3.07E-09	Blood_Tre	U2AF1
EIF3M3	2.53E-13	0.71578	0.284	0.259	4.03E-09	Blood_Tre	EIF3M
PARP101	2.56E-13	0.383092	0.063	0.034	4.08E-09	Blood_Tre	PARP10
CYFIP21	2.94E-13	0.330295	0.057	0.03	4.69E-09	Blood_Tre	CYFIP2
CDK2AP25	3.26E-13	0.759114	0.203	0.165	5.2E-09	Blood_Tre	CDK2AP2
HPCAL14	3.87E-13	0.561986	0.116	0.078	6.17E-09	Blood_Tre	HPCAL1
BST28	4.67E-13	0.433475	0.228	0.181	7.44E-09	Blood_Tre	BST2
GLIPR29	5.24E-13	0.553371	0.124	0.085	8.35E-09	Blood_Tre	GLIPR2
RAN11	8.41E-13	0.57786	0.37	0.368	1.34E-08	Blood_Tre	RAN
MZT2B7	9.2E-13	0.440589	0.443	0.462	1.47E-08	Blood_Tre	MZT2B
SCAMP3	1.25E-12	0.59295	0.111	0.075	1.99E-08	Blood_Tre	SCAMP3

IGBP11	1.78E-12	0.81521	0.244	0.217	2.84E-08	Blood_Tre	IGBP1
BAX3	2.44E-12	0.715085	0.205	0.169	3.9E-08	Blood_Tre	BAX
WDR542	2.45E-12	0.53433	0.098	0.064	3.91E-08	Blood_Tre	WDR54
SLC25A381	2.83E-12	0.465172	0.077	0.046	4.52E-08	Blood_Tre	SLC25A38
POLR2G2	3.21E-12	0.71808	0.198	0.161	5.12E-08	Blood_Tre	POLR2G
CPNE15	5.47E-12	0.689998	0.165	0.128	8.73E-08	Blood_Tre	CPNE1
C1QBP2	6.8E-12	0.682614	0.264	0.238	1.09E-07	Blood_Tre	C1QBP
DGKZ2	9E-12	0.424801	0.086	0.054	1.44E-07	Blood_Tre	DGKZ
NBEAL110	1.51E-11	0.280411	0.363	0.32	2.41E-07	Blood_Tre	NBEAL1
OXNAD11	1.79E-11	0.339205	0.064	0.036	2.85E-07	Blood_Tre	OXNAD1
C1orf228	2.04E-11	0.356242	0.068	0.04	3.26E-07	Blood_Tre	C1orf228
SCNM12	2.08E-11	0.495435	0.104	0.069	3.32E-07	Blood_Tre	SCNM1
TMUB1	2.44E-11	0.684559	0.146	0.11	3.9E-07	Blood_Tre	TMUB1
RSL1D15	3.04E-11	0.639816	0.272	0.248	4.85E-07	Blood_Tre	RSL1D1
RNPS16	3.22E-11	0.681541	0.228	0.198	5.13E-07	Blood_Tre	RNPS1
PHB25	3.68E-11	0.745254	0.224	0.195	5.86E-07	Blood_Tre	PHB2
ERP442	3.91E-11	0.256725	0.107	0.18	6.24E-07	Blood_Tre	ERP44
MAPKAPK5	5.78E-11	0.560295	0.108	0.074	9.22E-07	Blood_Tre	MAPKAPK5.AS1
NUDT16L1	6.31E-11	0.617488	0.137	0.102	1.01E-06	Blood_Tre	NUDT16L1
COX7A2L4	8.92E-11	0.642969	0.323	0.322	1.42E-06	Blood_Tre	COX7A2L
CTA.29F11	1.19E-10	0.555778	0.103	0.07	1.9E-06	Blood_Tre	CTA.29F11.1
HOXB.AS1	1.25E-10	0.292323	0.042	0.021	2E-06	Blood_Tre	HOXB.AS1
C7orf55.LU	1.32E-10	0.405274	0.074	0.046	2.11E-06	Blood_Tre	C7orf55.LUC7L2
ITM2A7	1.69E-10	0.369438	0.177	0.135	2.7E-06	Blood_Tre	ITM2A
XRCC61	1.85E-10	0.687991	0.26	0.24	2.95E-06	Blood_Tre	XRCC6
FDFT13	2.06E-10	0.259496	0.133	0.214	3.28E-06	Blood_Tre	FDFT1
LRCH42	2.14E-10	0.619511	0.136	0.102	3.41E-06	Blood_Tre	LRCH4
HDAC102	2.55E-10	0.288368	0.044	0.023	4.07E-06	Blood_Tre	HDAC10
SSNA13	3.06E-10	0.749039	0.25	0.231	4.88E-06	Blood_Tre	SSNA1
CCDC65	3.38E-10	0.298959	0.039	0.02	5.39E-06	Blood_Tre	CCDC65
STUB13	3.43E-10	0.717542	0.267	0.253	5.48E-06	Blood_Tre	STUB1
C5orf561	3.62E-10	0.471937	0.099	0.067	5.78E-06	Blood_Tre	C5orf56
THOC72	3.99E-10	0.265505	0.123	0.197	6.36E-06	Blood_Tre	THOC7
CALHM27	3.99E-10	0.342391	0.059	0.034	6.37E-06	Blood_Tre	CALHM2
EVI2A6	6.72E-10	0.294223	0.063	0.037	1.07E-05	Blood_Tre	EVI2A
XRCC55	6.89E-10	0.262861	0.16	0.25	1.1E-05	Blood_Tre	XRCC5
AP1G21	9.33E-10	0.451934	0.072	0.045	1.49E-05	Blood_Tre	AP1G2
MIF4GD3	1.19E-09	0.524233	0.098	0.068	1.89E-05	Blood_Tre	MIF4GD
H1FX2	1.33E-09	0.697237	0.279	0.26	2.13E-05	Blood_Tre	H1FX
DENND2D	1.36E-09	0.464816	0.086	0.057	2.17E-05	Blood_Tre	DENND2D
SH3YL14	1.45E-09	0.32345	0.133	0.212	2.32E-05	Blood_Tre	SH3YL1
C10orf128	1.58E-09	0.297643	0.04	0.021	2.52E-05	Blood_Tre	C10orf128
CKLF7	1.58E-09	0.575903	0.182	0.15	2.52E-05	Blood_Tre	CKLF
RPA2	1.68E-09	0.588005	0.128	0.096	2.68E-05	Blood_Tre	RPA2
ZRSR22	2.11E-09	0.406443	0.068	0.043	3.37E-05	Blood_Tre	ZRSR2
TRAF12	2.22E-09	0.253438	0.046	0.025	3.53E-05	Blood_Tre	TRAF1
DGKA	2.4E-09	0.43142	0.07	0.044	3.83E-05	Blood_Tre	DGKA

LSM103	2.41E-09	0.59559	0.163	0.131	3.85E-05	Blood_Tre	LSM10
MAPRE13	2.46E-09	0.253486	0.111	0.177	3.92E-05	Blood_Tre	MAPRE1
C14orf13	2.81E-09	0.254353	0.08	0.135	4.48E-05	Blood_Tre	C14orf1
SRSF41	3.63E-09	0.258971	0.117	0.186	5.8E-05	Blood_Tre	SRSF4
TIAL11	3.71E-09	0.251549	0.079	0.132	5.92E-05	Blood_Tre	TIAL1
MGAT4A3	3.75E-09	0.626094	0.134	0.103	5.98E-05	Blood_Tre	MGAT4A
NAP1L42	3.77E-09	0.263456	0.104	0.168	6.01E-05	Blood_Tre	NAP1L4
TMEM230	5.12E-09	0.696128	0.275	0.267	8.16E-05	Blood_Tre	TMEM230
KMT2E.AS	5.28E-09	0.332844	0.06	0.036	8.42E-05	Blood_Tre	KMT2E.AS1
RTFDC12	5.89E-09	0.306988	0.127	0.2	9.4E-05	Blood_Tre	RTFDC1
PPP6C	6.64E-09	0.306831	0.097	0.158	0.000106	Blood_Tre	PPP6C
TEX2644	7.51E-09	0.710364	0.199	0.175	0.00012	Blood_Tre	TEX264
MRPL237	7.63E-09	0.686033	0.232	0.211	0.000122	Blood_Tre	MRPL23
TACC3	7.98E-09	0.343105	0.059	0.036	0.000127	Blood_Tre	TACC3
TMEM9B3	9.04E-09	0.267166	0.099	0.158	0.000144	Blood_Tre	TMEM9B
SPIN2B	9.32E-09	0.274926	0.04	0.022	0.000149	Blood_Tre	SPIN2B
KARS1	1.27E-08	0.732843	0.184	0.159	0.000203	Blood_Tre	KARS
SUCLG14	1.33E-08	0.26759	0.114	0.179	0.000213	Blood_Tre	SUCLG1
TRABD2A	1.41E-08	0.288356	0.063	0.039	0.000224	Blood_Tre	TRABD2A
ARL5A7	1.49E-08	0.311802	0.127	0.198	0.000238	Blood_Tre	ARL5A
ZNF101	1.6E-08	0.31862	0.05	0.029	0.000255	Blood_Tre	ZNF101
BEX21	1.62E-08	0.584458	0.122	0.093	0.000258	Blood_Tre	BEX2
U2AF1L41	1.64E-08	0.446303	0.092	0.064	0.000262	Blood_Tre	U2AF1L4
PIN13	1.74E-08	0.678891	0.225	0.205	0.000277	Blood_Tre	PIN1
TSTD18	1.79E-08	0.562955	0.283	0.272	0.000285	Blood_Tre	TSTD1
CASP6	1.91E-08	0.435564	0.065	0.041	0.000305	Blood_Tre	CASP6
MTFP11	1.96E-08	0.355823	0.061	0.038	0.000313	Blood_Tre	MTFP1
SRSF78	2.09E-08	0.560728	0.336	0.344	0.000333	Blood_Tre	SRSF7
FKBP115	2.18E-08	0.411939	0.184	0.153	0.000347	Blood_Tre	FKBP11
LENG11	2.22E-08	0.568764	0.108	0.079	0.000354	Blood_Tre	LENG1
NSMCE12	2.26E-08	0.632015	0.175	0.148	0.000361	Blood_Tre	NSMCE1
NDUFA124	2.32E-08	0.269712	0.184	0.277	0.00037	Blood_Tre	NDUFA12
C1orf435	2.47E-08	0.294735	0.15	0.23	0.000394	Blood_Tre	C1orf43
GBP12	2.71E-08	0.443345	0.089	0.062	0.000432	Blood_Tre	GBP1
SMDT16	2.81E-08	0.55962	0.356	0.374	0.000448	Blood_Tre	SMDT1
ATP6V1E1	2.81E-08	0.278203	0.15	0.229	0.000448	Blood_Tre	ATP6V1E1
INO80E1	3.1E-08	0.508414	0.091	0.064	0.000494	Blood_Tre	INO80E
C6orf11	3.49E-08	0.495978	0.105	0.077	0.000556	Blood_Tre	C6orf1
MED103	3.55E-08	0.692751	0.206	0.185	0.000567	Blood_Tre	MED10
PYCR21	4.46E-08	0.511822	0.101	0.074	0.000711	Blood_Tre	PYCR2
CDC378	4.47E-08	0.707857	0.276	0.275	0.000714	Blood_Tre	CDC37
PI162	5.59E-08	0.285112	0.076	0.05	0.000891	Blood_Tre	PI16
PNRC17	5.89E-08	0.276962	0.486	0.512	0.00094	Blood_Tre	PNRC1
MIIP2	6.98E-08	0.478918	0.078	0.053	0.001114	Blood_Tre	MIIP
STK17A2	8.53E-08	0.537385	0.176	0.148	0.00136	Blood_Tre	STK17A
SUMO34	8.89E-08	0.311184	0.148	0.223	0.001418	Blood_Tre	SUMO3
PLGRKT	9.05E-08	0.53017	0.107	0.08	0.001444	Blood_Tre	PLGRKT

NCBP2	9.28E-08	0.264373	0.076	0.123	0.00148	Blood_Tre	NCBP2
NEDD89	1.2E-07	0.557762	0.385	0.424	0.001913	Blood_Tre	NEDD8
COX145	1.3E-07	0.267489	0.158	0.235	0.002076	Blood_Tre	COX14
EIF3I4	1.4E-07	0.72067	0.252	0.244	0.002231	Blood_Tre	EIF3I
IMP35	1.52E-07	0.675969	0.206	0.187	0.002418	Blood_Tre	IMP3
FDX12	1.53E-07	0.324067	0.109	0.168	0.002446	Blood_Tre	FDX1
UGP22	1.62E-07	0.337947	0.202	0.303	0.002585	Blood_Tre	UGP2
HIST2H2A(	1.98E-07	0.365484	0.06	0.039	0.003159	Blood_Tre	HIST2H2AC
CCDC1244	1.99E-07	0.258849	0.105	0.161	0.003177	Blood_Tre	CCDC124
RBM383	2E-07	0.311417	0.058	0.037	0.003182	Blood_Tre	RBM38
APBA3	2.04E-07	0.279238	0.038	0.021	0.00325	Blood_Tre	APBA3
ITGB1BP19	2.21E-07	0.614427	0.176	0.151	0.003521	Blood_Tre	ITGB1BP1
PLEKHJ13	2.23E-07	0.713506	0.188	0.168	0.003558	Blood_Tre	PLEKHJ1
OCIAD17	2.33E-07	0.297666	0.172	0.255	0.003711	Blood_Tre	OCIAD1
TMEM14B	2.48E-07	0.29715	0.172	0.255	0.003951	Blood_Tre	TMEM14B
DHPS1	2.5E-07	0.634411	0.154	0.129	0.003991	Blood_Tre	DHPS
DPP78	2.58E-07	0.607911	0.266	0.26	0.004111	Blood_Tre	DPP7
UBE2F	2.83E-07	0.27696	0.112	0.17	0.004515	Blood_Tre	UBE2F
SDF23	2.85E-07	0.278603	0.091	0.141	0.004544	Blood_Tre	SDF2
BCL22	3.3E-07	0.329712	0.063	0.041	0.005264	Blood_Tre	BCL2
STRAP6	3.44E-07	0.302683	0.148	0.219	0.00549	Blood_Tre	STRAP
SNRNP705	3.8E-07	0.299134	0.123	0.185	0.006063	Blood_Tre	SNRNP70
LINC.PINT3	3.85E-07	0.367426	0.065	0.043	0.006137	Blood_Tre	LINC.PINT
SPG215	4.72E-07	0.274109	0.104	0.159	0.00752	Blood_Tre	SPG21
TNF4	4.87E-07	0.255029	0.034	0.019	0.007764	Blood_Tre	TNF
IDH24	5.03E-07	0.28631	0.131	0.196	0.008029	Blood_Tre	IDH2
RPUSD31	5.45E-07	0.447938	0.087	0.063	0.008698	Blood_Tre	RPUSD3
ZYX6	6.29E-07	0.553024	0.141	0.116	0.010025	Blood_Tre	ZYX
EAPP4	6.33E-07	0.3006	0.124	0.186	0.010093	Blood_Tre	EAPP
PRR72	6.49E-07	0.426844	0.072	0.049	0.010358	Blood_Tre	PRR7
VDAC18	6.71E-07	0.371953	0.165	0.244	0.01071	Blood_Tre	VDAC1
GALM	6.9E-07	0.470333	0.073	0.051	0.01101	Blood_Tre	GALM
APBB1	7.28E-07	0.25532	0.047	0.029	0.011618	Blood_Tre	APBB1
PDCD22	8.99E-07	0.654026	0.162	0.14	0.014334	Blood_Tre	PDCD2
GLRX32	9.66E-07	0.256714	0.101	0.153	0.015411	Blood_Tre	GLRX3
[truncated: 505,119 more chars]
